# Exploring Evolutionary Medicine through Bibliometrics: Research Insights and Future Opportunities

**DOI:** 10.1093/emph/eoaf032

**Published:** 2025-11-11

**Authors:** Lukas Blumrich, Johnny Uelmen, Alexandre Archanjo Ferraro

**Affiliations:** Department of Pediatrics, Faculty of Medicine, University of São Paulo, Av. Dr. Enéas Carvalho de Aguiar, 647 - Cerqueira César, São Paulo - SP, 05403-000, Brazil; Department of Population Health Sciences, School of Medicine and Public Health University of Wisconsin-Madison, 610 Walnut Street, Madison, WI 53726, USA; Department of Pediatrics, Faculty of Medicine, University of São Paulo, Av. Dr. Enéas Carvalho de Aguiar, 647 - Cerqueira César, São Paulo - SP, 05403-000, Brazil

**Keywords:** evolutionary medicine, bibliometrics, interdisciplinary research, clinical translation, global health

## Abstract

**Background:**

Evolutionary medicine applies principles of evolutionary biology to elucidate the origins of human health and disease. Despite rapid growth since its emergence in the 1990s, the field lacks systematic bibliometric evaluation.

**Methods:**

We conducted the first comprehensive bibliometric analysis of evolutionary medicine using the Web of Science Core Collection. Two search strategies captured general literature (n = 885) and publications from *Evolution, Medicine, and Public Health* (*EMPH*, n = 358). We analyzed citation patterns, thematic clusters, and collaboration networks using Bibliometrix and VOSviewer.

**Results:**

The field exhibits steady growth, with high citation impact from review articles and a dominant presence of contributions from the USA, UK, and Germany. Six major keyword clusters were identified: drug resistance, infection, evolutionary mismatch, cancer, cognition, and mental health. However, topics such as clinical translation, One Health, Planetary Health, and race-related issues remain underrepresented. Moreover, standard database queries failed to capture most *EMPH* articles, highlighting a lack of field identification in metadata.

**Conclusions:**

This bibliometric overview reveals strengths and gaps in the evolutionary medicine literature. To enhance visibility, equity, and clinical relevance, future research should promote interdisciplinary integration, broader international collaboration, and more consistent field labeling in publications. These efforts are vital to advancing evolutionary perspectives in global biomedical and public health discourse.

## INTRODUCTION

Evolutionary medicine [1] is an interdisciplinary field that applies evolutionary biology to medicine and public health, aiming to address complex challenges in human health. While the integration of evolutionary ideas into health sciences dates back to the mid-20th century—exemplified by early works linking evolution to disease susceptibility [2–6]—the formal establishment of evolutionary medicine is commonly attributed to Nesse and Williams' landmark paper in 1991 [7].

Since then, the field has rapidly expanded [8], marked by the publication of foundational textbooks, the creation of research centers dedicated to evolutionary medicine, the establishment of the Society for Evolutionary Medicine and Public Health, and the launch of the journal *Evolution, Medicine, and Public Health* (*EMPH*). Interest in evolutionary medicine has grown internationally, attracting researchers from diverse disciplines such as biology, anthropology, epidemiology, genetics, genomics, and clinical medicine [8, 9].

Despite this expansion, no comprehensive analysis of the evolutionary medicine literature has been conducted. Consequently, the field's structural features, intellectual foundations, thematic evolution, and global collaboration networks remain poorly characterized. A systematic bibliometric analysis can illuminate these dimensions, helping to identify emerging research areas, underexplored topics, and key contributors. Such insights are critical not only for advancing the field conceptually but also for informing institutional strategy, research funding priorities, and interdisciplinary integration [10].

Bibliometrics [11], the application of quantitative methods to analyze academic publications, offers a powerful means of understanding the growth and influence of research within a given field. By examining collaboration networks, citation patterns, and keyword trends, bibliometric analyses can reveal important insights into a field’s intellectual landscape [10, 12]. In this study, we present the first bibliometric analysis of the evolutionary medicine literature, with the goal of identifying key trends, influential publications, opportunities for growth, and the overall structure of this dynamic and growing field.

## METHODS

### Data source

We retrieved bibliometric data from the Web of Science Core Collection (WoSCC), a comprehensive and widely used database for citation analysis and scholarly indexing. WoSCC was selected for its high data quality, structured citation tracking, and standardized metadata on authorship, affiliations, and country of origin. All data were extracted on May 7, 2025.

### Search strategy

Two search strategies were employed to capture the scope and specific contributions within the field of evolutionary medicine.

- **Search Strategy 1 (SS1)** targeted the general literature on the topic, using the terms *‘evolutionary medicine’ OR ‘Darwinian medicine’* across all searchable fields. No filters were applied for publication year, language, or document type, to ensure broad inclusion of relevant works.
- **Search Strategy 2 (SS2)** focused specifically on *EMPH*, the official journal of the International Society for Evolution, Medicine, and Public Health and the only journal dedicated exclusively to this field. All articles published by *EMPH* were included for separate analysis.

For both strategies, full bibliographic data were downloaded, including titles, abstracts, keywords, author affiliations, citations, and references. Additionally, all citing literature (i.e. publications citing articles retrieved in SS1) available within WoSCC was extracted to allow for comparative analyses. Full bibliographic data from SS1 and SS2 analyses are available in Supplementary File 1.

### Bibliometric analysis

Descriptive bibliometric indicators were analyzed using the *Bibliometrix* R package (version 4.0.0) [13], including author productivity, institutional output, country contributions, keyword frequencies, and journal impact. To examine collaboration patterns and intellectual structure, we conducted co-authorship, co-citation, and keyword co-occurrence analyses using *VOSviewer* (Leiden University, version 1.6.19). Network maps were generated to visualize international collaboration and thematic clusters.

As a comparator for keyword analyses, papers published in the last 5 years (2019–2024) in the WOSCC categories of Medicine General Internal + Medicine Research & Experimental and Medicine General Internal + Public, Environmental & Occupational Health were downloaded and author’s keywords plotted over time to identify trends and compare them to the Evolutionary Medicine literature.

To identify the core literature in Evolutionary Medicine, we analyzed the cited references within the articles identified by SS1. The top most cited articles were selected for further evaluation. Any articles or sources unrelated to evolutionary medicine, evolutionary biology, or health sciences were excluded from the analysis.

## RESULTS

### S‌S1 (general evolutionary medicine literature)

**Figure 1:**
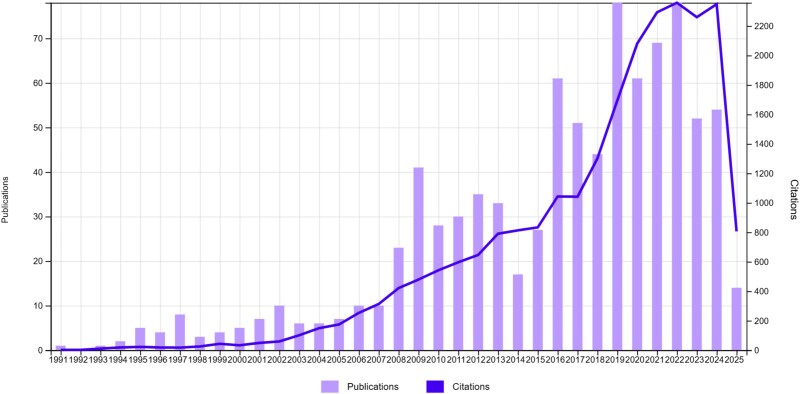
Publications and citations of SS1 by year.

SS1 identified a total of 885 publications on evolutionary medicine, spanning from 1991 to 2025 and accumulating 23 582 citations in total (Fig. 1). The citing literature for these articles encompassed 20 668 indexed publications in the Web of Science Core Collection. Using these publications and a cut-off of 25 citations, we identified 17 papers that compose the core literature of evolutionary medicine at the present date (Table 1).

**Table 1:** Core literature identified by SS1, defined by at least 25 citations.

Cited Reference	Citations
Nesse, R. M., & Williams, G. C. (2012). Why we get sick: The New science of Darwinian Medicine. Vintage.	117
Williams, G. C., & Nesse, R. M. (1991). The dawn of Darwinian medicine. The Quarterly review of biology, 66(1), 1–22.	114
Gluckman, P., Beedle, A., & Hanson, M. (2009). Principles of evolutionary medicine. Oxford University Press.	77
Williams, G.C. (1957) Pleiotropy, Natural Selection, and the Evolution of Senescence. Evolution, Volume 11, Issue 4, 1 December 1957, Pages 398–411,	52
Trevathan, W., Smith, E. O., & McKenna, J. J. (Eds.). (1999). Evolutionary Medicine. New York: Oxford University Press.	50
Nesse, R. M., & Stearns, S. C. (2008). The great opportunity: evolutionary applications to medicine and public health. Evolutionary Applications, 1(1), 28–48.	43
Nesse, R. M., Bergstrom, C. T., Ellison, P. T., Flier, J. S., Gluckman, P., Govindaraju, D. R.,... & Valle, D. (2010). Making evolutionary biology a basic science for medicine. Proceedings of the National Academy of Sciences, 107, 1800–1807.	42
Eaton, S. B., Konner, M., & Shostak, M. (1988). Stone agers in the fast lane: chronic degenerative diseases in evolutionary perspective. The American journal of medicine, 84(4), 739–749.	42
Neel, J. V. (1962). Diabetes mellitus: a ‘thrifty’ genotype rendered detrimental by ‘progress’?. American journal of human genetics, 14(4), 353.	35
Stearns, S. C. (2012). Evolutionary medicine: its scope, interest and potential. Proceedings of the Royal Society B: Biological Sciences, 279(1746), 4305–4321.	32
Ewald, P. W. (1994). Evolution of infectious disease. Oxford University Press.	32
Eaton, S. B., & Konner, M. (1985). Paleolithic nutrition: a consideration of its nature and current implications. New England Journal of Medicine, 312(5), 283–289.	30
Eaton SB, Strassman BI, Nesse RM, Neel JV, Ewald PW, Williams GC, *et al.* (2002) Evolutionary health promotion. Prev Med, 34 (2), pp. 109–118	30
Tinbergen, N. 1963. ‘On aims and methods of ethology.’ Zeitschrift für Tierpsychologie 20:410–433.	29
Ewald, P. W. (1994). Evolution of infectious disease. Oxford University Press, USA.	26
Theodosius Dobzhansky (1973) Nothing in Biology Makes Sense except in the Light of Evolution. The American Biology Teacher 1; 35 (3): 125–129.	26
Nesse, R. M. (2005) Maladaptation and Natural Selection. The Quarterly Review of Biology 80:1, 62–70	25

The documents retrieved through SS1 were classified into six major categories: original research articles (62.03%), review articles (20.56%), book chapters (9.83%), editorial material (5.65%), meeting abstracts (3.79%), and letters (3.27%) (Table 2).

**Table 2:** Document types^a^ for each search strategy.

**Document Types**			
**SS1**	**N (%)**	**SS2**	**N (%)**
Article	549 (62.0)	Article	213 (59.5)
Review Article	182 (20.6)	Review Article	47 (13.1)
Editorial Material	50 (5.6)	Editorial Material	73 (20.4)
Letter	29 (3.3)	Letter	6 (1.7)
Book Review	24 (2.7)	Book Review	13 (3.6)
Book Chapter	87 (9.8)	Other	6 (1.7)

a Documents may have more than one type in the database.

An analysis of author affiliations revealed that the most active countries in the field were the United States, Germany, and the United Kingdom. The leading contributing institutions were Duke University, the University of California, and Arizona State University. International collaboration networks, restricted to authors with at least five publications, are depicted in Figs. 3 and 4.

**Figure 2:**
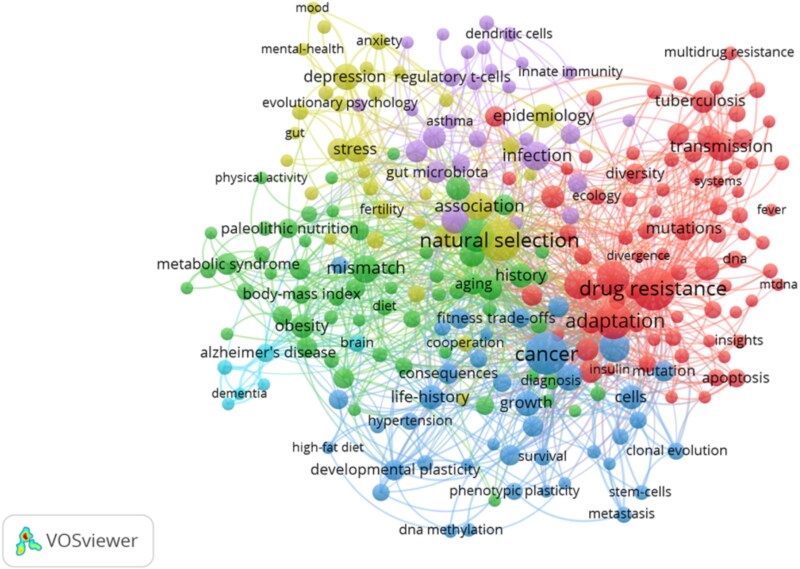
Keyword cluster analysis of SS1. Caption: All Keywords cluster analyses with VOSviewer. Clusters were labeled as drug resistance (red), hygiene and infection (purple), mental health (yellow), evolutionary mismatch (green), cognition (light blue) and cancer and developmental plasticity (dark blue).

**Figure 3:**
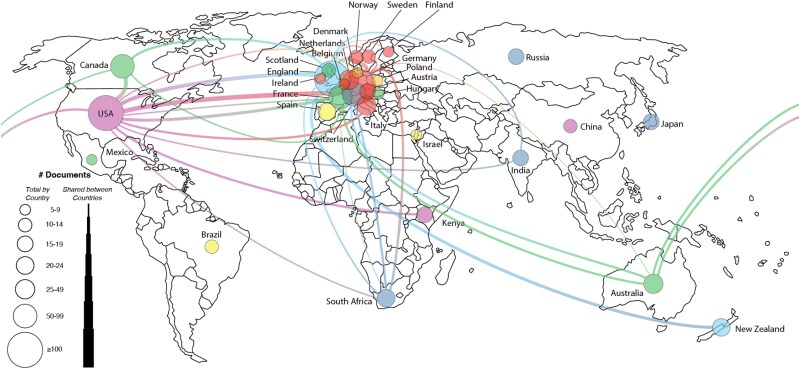
Map and network of publishing countries for SS1.

**Figure 4:**
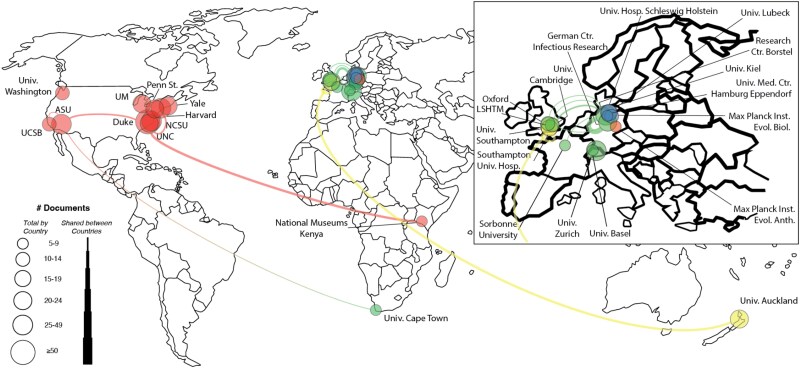
Network of institutional collaborations for SS1.

Citing articles for the SS1 dataset were primarily categorized by WOSCC under *Biochemistry & Molecular Biology* (10.02%), *Genetics & Heredity* (7.41%), and *Evolutionary Biology* (7.17%). The citing body of literature was predominantly produced by authors from the United States, China, and the United Kingdom (Table 3).

**Table 3:** Top 10 SS1 citing literature categories and countries.

Category	N (%)	Country	N (%)
Biochemistry Molecular Biology	2075 (10.0)	United States of America	8221 (39.7)
Genetics Heredity	1533 (7.4)	People’s Republic of China	2408 (11.6)
Evolutionary Biology	1485 (7.2)	England	2319 (11.2)
Multidisciplinary Sciences	1432 (6.9)	Germany	1740 (8.4)
Cell Biology	1367 (6.6)	Canada	1409 (6.8)
Biology	1256 (6.0)	France	1280 (6.2)
Neurosciences	1150 (5.6)	Australia	1168 (5.6)
Ecology	999 (4.8)	Italy	1126 (5.4)
Pharmacology Pharmacy	841 (4.1)	Spain	873 (4.2)
Microbiology	828 (4.0)	Netherlands	732 (3.5)

A co-occurrence analysis of author keywords and Keywords Plus revealed six distinct thematic clusters (Fig. 2). These clusters were interpreted as follows: [1] drug resistance (red), [2] hygiene and infection (purple), [3] evolutionary mismatch (green), [4] cancer and developmental plasticity (dark blue), [5] cognition (light blue), and [6] stress and mental health (yellow).

We also looked for relevant terms that characterize broader interdisciplinary foci in titles, abstracts and keywords. For example, only five articles mentioned “One Health,” one article referred to “Planetary Health,” and 11 articles included ‘DOHaD’ or related terms. References to ‘race,’ ‘racial,’ or associated concepts were found in only six articles.

The core literature analysis based on all the references of the papers identified by SS1 (n = 50 648) identified 17 highly cited sources with at least 25 citations each—representing only 0.033% of the total cited works. These included 13 peer-reviewed articles and four books, published between 1953 and 2012 (Table 1).

### S‌S2 (*EMPH* journal-specific analysis)

**Figure 5:**
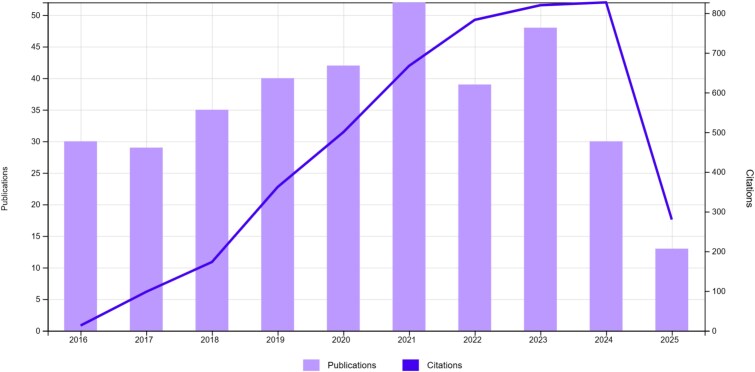
Publications and citations from SS2 by year.

SS2, which focused exclusively on publications from *Evolution, Medicine, and Public Health*, identified 358 articles published between 2016 and 2025. These works were cited by 4025 articles, accumulating 4515 total citations (Fig. 5). Forty-seven articles appeared in both SS1 and SS2 datasets. Document types for *EMPH* articles are detailed in Table 2.

**Figure 6:**
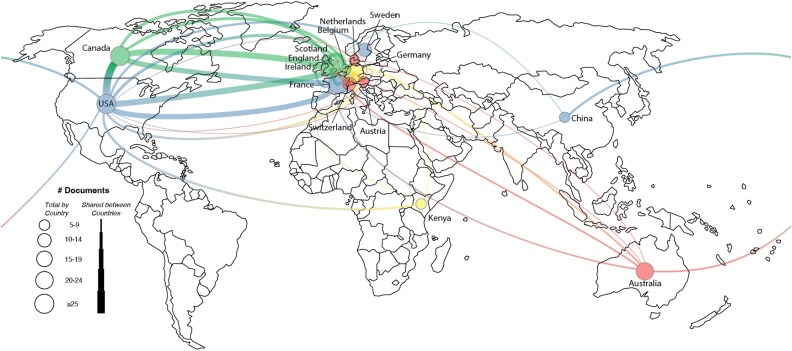
Map and network of publishing countries for SS2.

**Figure 7:**
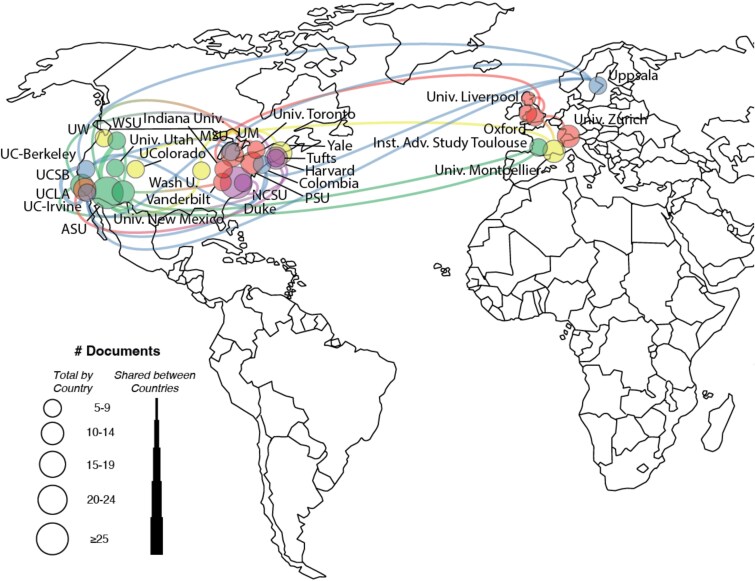
Network of institutional collaborations for SS2.

The most active contributing countries were the United States, the United Kingdom, and Canada. The most prolific institutions were the University of California, Duke University, and Arizona State University. Collaboration networks within the *EMPH* dataset are shown in Fig. 6 and Fig. 7.

Citing articles for *EMPH* publications were most frequently classified under *Biochemistry & Molecular Biology*, *Microbiology*, and *Evolutionary Biology* (Table 4). Comparative analysis of article types revealed that review articles received more citations on average (mean = 28.43; h-index = 21) than original research articles (mean = 10.94; h-index = 23). However, original research articles had a slightly higher proportion of citations in clinical medicine journals (36.9%) compared to review articles (33.0%). When adjusted by output, each original article contributed ~4.04 citations in clinical medicine journals, while review articles contributed 9.38.

**Table 4:** Top 10 SS2 citing literature categories and countries.

Category	N (%)	Country	N (%)
Biochemistry Molecular Biology	350 (8.4)	United States of America	1636 (39.4)
Microbiology	346 (8.3)	England	472 (11.4)
Evolutionary Biology	342 (8.2)	People’s Republic of China	438 (10.6)
Public Environmental Occupational Health	317 (7.6)	Germany	344 (8.3)
Biology	316 (7.6)	Canada	295 (7.1)
Multidisciplinary Sciences	309 (7.4)	Australia	260 (6.3)
Pharmacology Pharmacy	234 (5.6)	France	219 (5.3)
Genetics Heredity	207 (5.0)	Italy	196 (4.7)
Anthropology	205 (4.9)	India	170 (4.1)
Neurosciences	182 (4.4)	Spain	136 (3.3)

Keyword and abstract analyses showed that among *EMPH* articles

only three included the term “one health,” eight mentioned “DOHaD” or related terms

and none referenced “planetary health.” the terms “race,” “racism,” or similar constructs were identified in just five articles

### Keyword trends and integration opportunities

**Figure 8:**
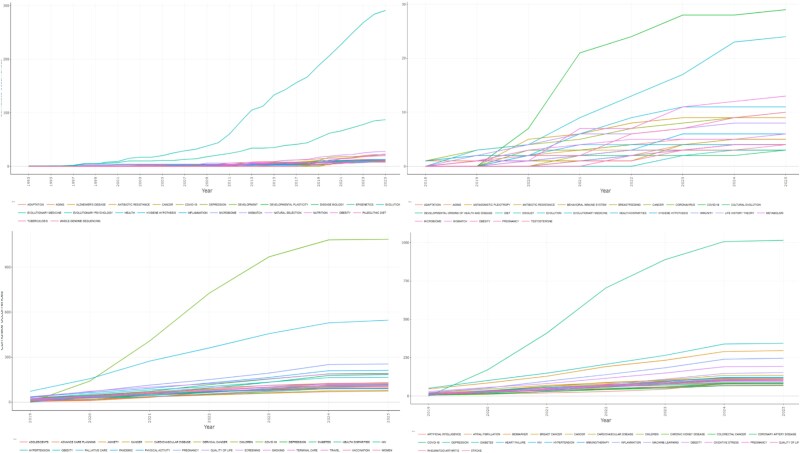
Authors’ keywords in the last 5 years (2019–2024) for SS1 (superior left panel), SS2 (superior right panel), internal medicine and public health (inferior left panel), and internal medicine and experimental research (inferior right panel).

Figure 8 presents keyword trends between 2019 and 2024 across SS1, SS2, and two comparator categories from Web of Science: Medicine General Internal plus Public Health (n = 12 553) and Medicine General Internal plus Experimental Research (n = 25 149). To provide a broad overview of scientific interest, we examined the 25 most frequent author keywords in each dataset. In SS1, the leading terms reflected core evolutionary concepts (‘evolutionary medicine,’ ‘evolution,’ and ‘natural selection’), whereas SS2 emphasized topical issues such as ‘COVID-19,’ ‘evolution,’ and ‘mismatch.’ In contrast, Internal Medicine plus Public Health publications were dominated by ‘COVID-19,’ ‘palliative care,’ and ‘pregnancy,’ while Internal Medicine plus Experimental Research highlighted ‘COVID-19,’ ‘diabetes,’ and ‘biomarker.’

## DISCUSSION

This study provides the first comprehensive bibliometric mapping of the field of Evolutionary Medicine, highlighting its growing international scope, thematic diversity, and institutional consolidation. With nearly 900 articles published between 1991 and 2025 and over 23 000 citations, the field exhibits steady, linear growth, supported in part by the dedicated journal *EMPH*. The creation of this flagship journal appears to have played a pivotal role in consolidating the field’s identity and scholarly visibility.

One of the central challenges in conducting bibliometric analysis of Evolutionary Medicine lies in the absence of standardized indexing for the field. As a highly interdisciplinary area without a dedicated category in major databases like Web of Science, Evolutionary Medicine remains difficult to isolate through conventional search strategies. Our approach prioritized sensitivity by using inclusive search terms; however, this analysis also underscores a broader issue: many articles within the field do not explicitly identify themselves as Evolutionary Medicine research. Strikingly, only 47 *EMPH* articles were retrieved through our main search strategy, despite the journal’s exclusive focus on evolution and health. This suggests that many authors may omit specific references to Evolutionary Medicine in titles, abstracts, or keywords—a practice that may inadvertently weaken the field’s academic visibility and hinder literature retrieval.

Strengthening the explicit identification of Evolutionary Medicine in publications could serve both scientific and strategic purposes. Particularly in countries or institutions without formal centers for Evolutionary Medicine, such identification may bolster efforts to legitimize the field, attract funding, and establish formal research agendas. In high-income countries with strong institutional output (e.g. the USA, Germany, and the UK), Evolutionary Medicine is already embedded within major universities. However, in low- and middle-income countries, barriers such as lack of dedicated funding streams and institutional recognition remain substantial. As our findings suggest, partnerships between established EvMed centers and peripheral institutions—particularly those in the Global South—could help address these disparities.

Our citing literature analysis suggests that while most of the literature is concentrated in the United States, United Kingdom and Canada (as is most of the research output in the biomedical sciences), developing countries have a growing interest in the field. This observation suggests a very fertile ground for international collaborations, and platforms like the International Society for Evolution, Medicine and Public Health’s *Directory* provide valuable tools for building such networks.

The thematic clusters identified through keyword co-occurrence analysis reflect both the intellectual breadth and fragmentation of the field. Six major clusters emerged: drug resistance, hygiene and infection, evolutionary mismatch, cancer and developmental plasticity, cognition, and stress/mental health. These align with foundational topics in Evolutionary Medicine and suggest both conceptual depth and topical diversification. However, the uneven distribution of these themes may indicate areas in need of further development. Strategic initiatives—such as special issues, thematic working groups, or targeted funding—could help stimulate growth in underrepresented domains.

Another notable feature of the field is its high proportion of review articles (over 20% in SS1), suggesting a continued emphasis on conceptual consolidation. Comparative analysis of *EMPH* articles revealed that review articles received more citations on average (mean = 28.43; h-index = 21) than original research articles (mean = 10.94; h-index = 23). Review articles also had a slightly higher proportion of citations in clinical medicine journals (36.9%) compared to original articles (33.0%). When adjusted for output, each original article contributed ~3.61 citations in clinical medicine journals, while each review article contributed 11.09. These findings indicate that review articles not only shape theoretical discourse but may also play a crucial role in guiding clinical audiences toward evolutionary perspectives. At the same time, expanding the production of original research—especially with applied or translational relevance—may strengthen the field’s impact in clinical and public health settings.

Despite these positive trends, our analysis revealed limited clinical penetration. Only a small number of articles across both search strategies referenced terms such as ‘clinic,’ ‘clinical,’ or ‘clinical medicine’ in titles or keywords. While the absence of these terms does not necessarily imply irrelevance, it does suggest missed opportunities for increasing visibility within the medical community. Including clinically relevant language in publication metadata may improve discoverability in clinical databases and journals and emphasize the translational potential of evolutionary insights.

Additionally, our findings show that citing literature for Evolutionary Medicine articles is still largely concentrated in molecular biology, microbiology, and evolutionary biology journals, rather than clinical or public health outlets. This trend likely reflects the field’s disciplinary origins and conceptual emphasis but may also point to a barrier in cross-disciplinary communication. Since citations tend to cluster within disciplines, the current pattern may represent a self-reinforcing cycle that limits the integration of evolutionary thinking into clinical domains. Proactive efforts to frame findings in ways that resonate with clinical concerns may help address this gap.

An intriguing observation is the scarcity of references to terms like ‘clinic,’ ‘clinical,’ or similar words in titles, abstracts, or keywords. SS1 found 14 articles containing clinic^*^ in the title or keywords (both authors and Keywords Plus), and five were found with SS2. This is notable because the absence of clinical applications is often suggested as a significant barrier to the integration of evolutionary medicine within the medical community. While discussions on clinical applications do not always require the use of these specific terms, including them can be instrumental in capturing attention from the medical literature and emphasizing their relevance.

Our comparative analysis of keyword trends further highlighted opportunities for integration between evolutionary medicine and clinical research. We selected Internal Medicine plus Public Health and Internal Medicine plus Experimental Research as comparators due to the interest in embedding evolutionary perspectives within clinical medicine. While the thematic focus of evolutionary medicine overlaps substantially with these areas, certain topics appear underexplored yet hold strong potential for broader engagement. For example, biomarker research—a major theme in experimental medicine—could be enriched by evolutionary perspectives on human evolutionary history and comparative biology, offering novel insights for both basic research and clinical practice. Similarly, non-communicable chronic diseases, including cancer, have long been central to evolutionary medicine, and greater efforts to publish outside of evolution-focused journals could substantially amplify the field’s influence [9, 14]. The same applies to mental health, given the historical role of psychiatry in shaping evolutionary approaches, particularly through the work of Randolph Nesse [15].

An unexpected finding was the limited use of integrative public health frameworks—such as One Health, Planetary Health, and DOHaD—in the Evolutionary Medicine literature. Given the conceptual overlap between these approaches and Evolutionary Medicine, their scarcity in titles, abstracts, and keywords is striking [16, 17]. Each emphasizes systemic, ecological, and developmental contexts in shaping human health—central concerns of Evolutionary Medicine. These absences represent important frontiers for future interdisciplinary collaboration, particularly as global health increasingly turns toward integrative models.

Another area where Evolutionary Medicine may offer critical contributions is in challenging racial essentialism in biomedical science [18–20]. Despite consensus in evolutionary biology and anthropology that race lacks a biological basis in humans, racial categories remain pervasive in clinical research and practice [18, 21]. Our analysis revealed that very few articles included terms such as ‘race,’ ‘racial,’ or ‘racism’ in their titles, abstracts, or keywords—suggesting that the field has yet to fully engage with this issue. As a discipline that bridges evolutionary theory and health, Evolutionary Medicine is uniquely positioned to clarify human biological diversity and contest misuses of race in medicine. Greater engagement with this topic could help address structural inequities and improve the scientific integrity of health research [22].

At the same time, analysis of citing literature journals suggests that readers of Evolutionary Medicine are more concentrated in molecular biology and biology compared to clinical medicine, indicated by the papers they cite. While this observation is not problematic and points to the interdisciplinarity of Evolutionary Medicine, the fact that most of the citing literature is not housed in clinical journals may suggest that direct clinical applications may at the time of being be limited, and mostly guide preclinical research.

The core literature identified through our search strategy represents the foundational works that shaped the discipline. Although there is an expected temporal gap between these seminal contributions and the current state of the field, they continue to provide valuable insights for both newcomers and established researchers interested in the intellectual evolution of evolutionary medicine. Notably, many of these works were authored by biologists or evolutionary anthropologists whose contributions often transcended what is now formally labeled as evolutionary medicine. As such, their work may not have been explicitly recognized—either by the authors themselves or by the medical community—as part of the field. This observation suggests that evolutionary medicine could greatly benefit from stronger engagement with anthropology, where many researchers may not yet fully appreciate the relevance of their work to health and medicine. Multidisciplinary meetings and cross-disciplinary initiatives thus represent important opportunities for fostering dialogue, recognition, and collaboration.

By contrast, areas such as palliative care remain largely absent from evolutionary medicine, despite their prominence in public health research, and could serve as promising entry points for expanding clinical visibility. Likewise, the field’s contributions to understanding health disparities remain substantial but underrepresented in mainstream medical literature [23, 24]. Increasing explicit engagement with these themes could help establish evolutionary medicine as a key contributor to contemporary clinical challenges.

Although ‘evolution’ and ‘evolutionary medicine’ emerged as the most frequent keywords in both SS1 and SS2, they appeared in only a minority of articles. Consistent use of these terms in titles, abstracts, and keywords would likely strengthen the field’s identity, improve discoverability, and expand its visibility across biomedical and public health audiences.

Given that these intersections exist but remain largely unrecognized by both the anthropological and medical communities, deliberate efforts to bridge perspectives should be actively encouraged. The *Clinical Briefs* section of *EMPH* [25], for instance, has proven valuable by providing concise and accessible evolutionary insights on medically relevant issues—resources that can be particularly useful when introducing evolutionary medicine to undergraduate students. Indeed, two of the authors of the present paper (L.B. and A.A.F.) received highly positive feedback from students regarding the clarity of this material when establishing the first undergraduate course on Evolutionary Medicine in Latin America at the University of São Paulo. One potential initiative that the community could foster is the use of correspondence pieces in both medical and anthropological journals to discuss evolutionary perspectives on novel and pertinent research articles.

Finally, issues of accessibility and equity remain pressing. While 58.08% of SS1 publications were open access, high publication costs continue to restrict participation from researchers in the Global South. In many countries, funding for publication fees is rare, and article processing charges can exceed the budgets of unfunded or early-career investigators. At the same time, countries with established scientific investment have faced disastrous cuts on research funds, compromising the already weak international collaboration networks (which often are sustained by grants from developed countries) and the advances in research infrastructure achieved in previous years. This economic barrier limits not only who gets to publish but also whose voices shape the trajectory of the field. Expanding access to publication support and advocating for equitable funding policies should be a priority for the global Evolutionary Medicine community.

## CONCLUSION

Evolutionary Medicine is a rapidly expanding field with a strong theoretical foundation and growing international reach. However, challenges remain in clinical integration, interdisciplinary visibility, and equitable access to publishing. Addressing these gaps through stronger international collaboration, explicit field identification, and greater clinical integration will be essential for the next stage of development. With sustained efforts, Evolutionary Medicine can play a transformative role in shaping future health research and policy.

## Supplementary Material

SS1_and_SS2_Results_eoaf032
