# Supplementary material for: Exploring Evolutionary Medicine through Bibliometrics: Research Insights and Future Opportunities
Source: Evol Med Public Health. 2025 Nov 11;13(1):399–410. doi: 10.1093/emph/eoaf032 (PMC12701569; doi:10.1093/emph/eoaf032)
Supplement: SS1_and_SS2_Results_eoaf032 [file ss1_and_ss2_results_eoaf032.pdf]

FN Clarivate Analytics Web of Science
VR 1.0
PT J
AU Everts, PA
   Mazzola, T
   Mautner, K
   Randelli, PS
   Podesta, L
AF Everts, Peter A. A.
   Mazzola, Timothy
   Mautner, Kenneth
   Randelli, Pietro S. S.
   Podesta, Luga
TI Modifying Orthobiological PRP Therapies Are Imperative for the
   Advancement of Treatment Outcomes in Musculoskeletal Pathologies
SO BIOMEDICINES
LA English
DT Review
DE evolutionary medicine; autologous orthobiologics; platelet-rich
plasma;
   platelets; angiogenesis; painkilling effects; immunomodulation;
dosing;
   bioformulations; platelet-leukocyte interactions
ID PLATELET-RICH PLASMA; REGENERATIVE MEDICINE; TISSUE-REPAIR; KNEE
   OSTEOARTHRITIS; ARACHIDONIC-ACID; FREE HEMOGLOBIN; FIBRIN MATRIX;
   LEUKOCYTE GEL; INFLAMMATION; PAIN
AB Autologous biological cellular preparations have materialized as a growing area of medical advancement in interventional (orthopedic) practices and surgical interventions to provide an optimal tissue healing environment, particularly in tissues where standard healing is disrupted and repair and ultimately restoration of function is at risk. These cellular therapies are often referred to as orthobiologics and are derived from patient's own tissues to prepare point of care platelet-rich plasma (PRP), bone marrow concentrate (BMC), and adipose tissue concentrate (ATC). Orthobiological preparations are biological materials comprised of a wide variety of cell populations, cytokines, growth factors, molecules, and signaling cells. They can modulate and influence many other resident cells after they have been administered in specific diseased microenvironments. Jointly, the various orthobiological cell preparations are proficient to counteract persistent inflammation, respond to catabolic reactions, and reinstate tissue homeostasis. Ultimately, precisely delivered orthobiologics with a proper dose and bioformulation will contribute to tissue repair. Progress has been made in understanding orthobiological technologies where the safety and relatively easy manipulation of orthobiological treatment tools has been demonstrated in clinical applications. Although more positive than negative patient outcome results have been registered in the literature, definitive and accepted standards to prepare specific cellular orthobiologics are still lacking. To promote significant and consistent clinical outcomes, we will present a review of methods for implementing dosing strategies, using bioformulations tailored to the pathoanatomic process of the tissue, and adopting variable preparation and injection volume policies. By optimizing the dose and specificity of orthobiologics, local cellular synergistic behavior will increase,

potentially leading to better pain killing effects, effective immunomodulation, control of inflammation, and (neo) angiogenesis, ultimately contributing to functionally restored body movement patterns.
C1 [Everts, Peter A. A.] Gulf Coast Biol, Educ & Res Div, Ft Myers, FL 33916 USA.
[Mazzola, Timothy] Breakthrough Regenerat Orthoped, Boulder, CO 80305 USA.
[Mautner, Kenneth] Emory Univ, Dept Phys Med & Rehabil, Atlanta, GA 30329 USA.
[Randelli, Pietro S. S.] Milan Univ, Inst Orthoped Gaetano Pini, I-20122 Milan, Italy.
[Podesta, Luga] Bluetail Med Grp, Naples, FL 34109 USA.
C3 Emory University; University of Milan; ASST Gaetano Pini CTO
RP Everts, PA (autor correspondente), Gulf Coast Biol, Educ & Res Div, Ft Myers, FL 33916 USA.
EM peter@gulfcoastbiologics.com
RI Everts, Peter/K-5001-2019
OI Everts, Peter A./0000-0003-3128-9380
CR Abbas A, 2022, J BONE JOINT SURG AM, V104, P559, DOI 10.2106/JBJS.20.02258
Amable PR, 2013, STEM CELL RES THER, V4, DOI 10.1186/scrt218
Andia I, 2021, THER ADV MUSCULOSKEL, V13, DOI 10.1177/1759720X211004336
Andia I, 2018, REGEN MED, V13, P717, DOI 10.2217/rme-2018-0042
Andia I, 2018, EXPERT OPIN BIOL TH, V18, P389, DOI 10.1080/14712598.2018.1424626
Andia I, 2013, NAT REV RHEUMATOL, V9, P721, DOI 10.1038/nrrheum.2013.141
Araya N, 2020, AM J SPORT MED, V48, P2004, DOI 10.1177/0363546520924011
Arora G, 2021, DERMATOL THER, V34, DOI 10.1111/dth.14343
Astudillo AM, 2012, BBA-MOL CELL BIOL L, V1821, P249, DOI 10.1016/j.bbalip.2011.11.006
Bansal H., RANDOMIZED CONTROLLE, DOI [10.21203/rs.3.rs-33058/v1, DOI 10.21203/RS.3.RS-33058/V1]
Bansal H, 2021, SCI REP-UK, V11, DOI 10.1038/s41598-021-83025-2
Belk JW, 2021, AM J SPORT MED, V49, P249, DOI 10.1177/0363546520909397
Bennell KL, 2021, JAMA-J AM MED ASSOC, V326, P2021, DOI 10.1001/jama.2021.19415
Berger DR, 2019, BONE JOINT RES, V8, P32, DOI 10.1302/2046-3758.81.BJR-2018-0164.R1
Bhan K, 2022, CUREUS J MED SCIENCE, V14, DOI 10.7759/cureus.26103
Bir SC, 2011, J VASC RES, V48, P195, DOI 10.1159/000318779
Blair P, 2009, BLOOD REV, V23, P177, DOI 10.1016/j.blre.2009.04.001
Bordoni B, 2020, CUREUS J MED SCIENCE, V12, DOI 10.7759/cureus.7092
Brill A, 2004, CARDIOVASC RES, V63, P226, DOI 10.1016/j.cardiores.2004.04.012
Cengiz IF, 2019, CURR REV MUSCULOSKE, V12, P406, DOI 10.1007/s12178-019-09573-3
Chabannes B, 2003, LAB INVEST, V83, P491, DOI 10.1097/01.LAB.0000062855.90029.D8
Chahla J, 2017, J BONE JOINT SURG AM, V99, P1769, DOI 10.2106/JBJS.16.01374

Chandrasekharan JA, 2015, J INFLAMM RES, V8, P181, DOI 10.2147/JIR.S90380
Cherian C., 2020, Biol. Orthop. J, V2, pe31, DOI [10.22374/boj.v2i1.11, DOI 10.22374/BOJ.V2I1.11]
Cianca JC, 2017, PHYS MED REH CLIN N, V28, P777, DOI 10.1016/j.pmr.2017.06.010
Clark SR, 2007, NAT MED, V13, P463, DOI 10.1038/nm1565
Clemetson KJ, 2000, BLOOD, V96, P4046
Collins T, 2021, EFORT OPEN REV, V6, P225, DOI [10.1302/2058-5241.6.200017, 10.1302/2058-5241.6.200011]
Das A, 2015, AM J PATHOL, V185, P2596, DOI 10.1016/j.ajpath.2015.06.001
Deal JB, 2017, ORTHOP J SPORTS MED, V5, DOI 10.1177/2325967117738238
Di Martino A, 2022, AM J SPORT MED, V50, P609, DOI 10.1177/03635465211064303
dos Santos RG, 2021, CYTOKINE, V144, DOI 10.1016/j.cyto.2021.155560
EDWARDS SW, 1995, TRENDS BIOCHEM SCI, V20, P362, DOI 10.1016/S0968-0004(00)89077-5
Escobar G, 2018, REGEN MED, V13, P427, DOI 10.2217/rme-2017-0122
Everts PA, 2008, EUR SURG RES, V40, P203, DOI 10.1159/000110862
Everts P.A., 2019, Wound HealingCurrent Perspectives, DOI 10.5772/intechopen.80502
Everts P.A., 2022, Clinical Guide to Musculoskeletal Medicine, P651, DOI [10.1007/978-3-030-92042-562, DOI 10.1007/978-3-030-92042-562]
Everts PAM, 2006, TRANSFUSION MED, V16, P363, DOI 10.1111/j.1365-3148.2006.00708.x
Everts P, 2020, INT J MOL SCI, V21, DOI 10.3390/ijms21207794
Everts PA, 2019, REGEN THER, V11, P56, DOI 10.1016/j.reth.2019.03.009
Everts Peter A M, 2006, J Extra Corpor Technol, V38, P174
Eymard F, 2021, KNEE SURG SPORT TR A, V29, P3195, DOI 10.1007/s00167-020-06102-5
Fadadu PP, 2019, REGION ANESTH PAIN M, V44, P652, DOI 10.1136/rapm-2018-100356
Fedorova NV, 2018, MEDIAT INFLAMM, V2018, DOI 10.1155/2018/1574928
Ferrante CJ, 2012, ADV WOUND CARE, V1, P10, DOI 10.1089/wound.2011.0307
Filardo G, 2018, KNEE SURG SPORT TR A, V26, P1984, DOI 10.1007/s00167-016-4261-4
Fitzpatrick J, 2017, ORTHOP J SPORTS MED, V5, DOI 10.1177/2325967116675272
Fitzpatrick J, 2017, AM J SPORT MED, V45, P226, DOI 10.1177/0363546516643716
Fu CJ, 2017, CLIN REHABIL, V31, P158, DOI 10.1177/0269215516634815
Ganor Y, 2003, J IMMUNOL, V170, P4362, DOI 10.4049/jimmunol.170.8.4362
Garcia A, 2011, WI S MAS SP, P1, DOI 10.1002/9780470940297
Gaudino SJ, 2019, FRONT IMMUNOL, V10, DOI 10.3389/fimmu.2019.00360
Gentile P, 2020, INT J MOL SCI, V21, DOI 10.3390/ijms21165702
Gentile P, 2020, INT J MOL SCI, V21, DOI 10.3390/ijms21020431
Giusti I, 2009, TRANSFUSION, V49, P771, DOI 10.1111/j.1537-2995.2008.02033.x
de Melo BAG, 2019, MOLECULES, V24, DOI 10.3390/molecules24152729
Gray MA, 2016, CURR BIOL, V26, P1955, DOI 10.1016/j.cub.2016.05.070
Grunspan DZ, 2018, EVOL MED PUBLIC HLTH, P13, DOI 10.1093/emph/eox025
Gunning WT, 2022, CELLS-BASEL, V11, DOI 10.3390/cells11050774

HAMBERG M, 1974, P NATL ACAD SCI USA, V71, P3400, DOI 10.1073/pnas.71.9.3400
Haunschild ED, 2020, ARTHROSCOPY, V36, P1765, DOI 10.1016/j.arthro.2020.01.038
Heijnen H, 2015, J THROMB HAEMOST, V13, P2141, DOI 10.1111/jth.13147
Helms CC, 2018, FRONT PHYSIOL, V9, DOI 10.3389/fphys.2018.00125
Hurley ET, 2019, AM J SPORT MED, V47, P753, DOI 10.1177/0363546517751397
Hutchinson ID, 2022, CURR REV MUSCULOSKE, V15, P456, DOI 10.1007/s12178-022-09778-z
Iberg CA, 2020, J IMMUNOL, V204, P733, DOI 10.4049/jimmunol.1901121
Italiani P, 2014, FRONT IMMUNOL, V5, DOI 10.3389/fimmu.2014.00514
Jain D, 2020, PAIN MED, V21, P2719, DOI 10.1093/pm/pnaa254
Jo CH, 2013, J ORAL IMPLANTOL, V39, P525, DOI 10.1563/AAID-JOI-D-10-00155
Johal H, 2019, SPORTS HEALTH, V11, P355, DOI 10.1177/1941738119834972
Jones HR, 2016, SEMIN IMMUNOL, V28, P137, DOI 10.1016/j.smim.2016.03.007
Kaiko GE, 2008, IMMUNOLOGY, V123, P326, DOI 10.1111/j.1365-2567.2007.02719.x
Kaminski R, 2018, BIOMED RES INT, V2018, DOI 10.1155/2018/9315815
Kapur R, 2015, J IMMUNOL, V194, P5579, DOI 10.4049/jimmunol.1500259
Korpershoek JV, 2020, ACTA ORTHOP, V91, P743, DOI 10.1080/17453674.2020.1795366
Koupenova M, 2018, CIRC RES, V122, P337, DOI 10.1161/CIRCRESAHA.117.310795
Kovtun A, 2016, EUR CELLS MATER, V32, P152, DOI 10.22203/eCM.v032a10
Kuffler DP, 2019, J PAIN RES, V12, P109, DOI 10.2147/JPR.S190065
Kuffler DP, 2015, MOL NEUROBIOL, V52, P990, DOI 10.1007/s12035-015-9251-x
Kumar A, 2020, BIOL ORTHOP J, V2, P12, DOI [10.22374/boj.v2i1.6, DOI 10.22374/BOJ.V2I1.6]
Lana Jose Fabio, 2019, J Clin Orthop Trauma, V10, pS179, DOI 10.1016/j.jcot.2019.01.011
Lansdown DA, 2017, OPER TECHN SPORT MED, V25, P7, DOI 10.1053/j.otsm.2016.12.002
Levy BD, 2001, NAT IMMUNOL, V2, P612, DOI 10.1038/89759
Lin KY, 2022, ORTHOP J SPORTS MED, V10, DOI 10.1177/23259671221084706
Lin MT, 2020, DIAGNOSTICS, V10, DOI 10.3390/diagnostics10040189
Lubkowska A, 2012, J Biol Regul Homeost Agents, V26, p3S
Lucock M.D., 2022, EXPLOR RES HYPOTHESI, V7, P108
Lutz C, 2022, INT ORTHOP, V46, P1381, DOI 10.1007/s00264-022-05389-y
Magalon J., 2016, BMJ OPEN SPORT EXERC, V2, pe000060, DOI [10.1136/bmjsem-2015-000060, DOI 10.1136/BMJSEM-2015-000060]
Magalon J, 2021, PLATELETS, V32, P200, DOI 10.1080/09537104.2020.1832653
Malanga G, 2014, PHYS MED REH CLIN N, V25, P881, DOI 10.1016/j.pmr.2014.06.007
Marathe Anuj, 2022, Arthrosc Sports Med Rehabil, V4, pe335, DOI 10.1016/j.asmr.2021.10.004
Mariani E, 2020, INT J MOL SCI, V21, DOI 10.3390/ijms21041328
Marques LF, 2015, PLATELETS, V26, P101, DOI 10.3109/09537104.2014.881991
Marx R E, 2001, Implant Dent, V10, P225

Mazzucco L, 2009, VOX SANG, V97, P110, DOI 10.1111/j.1423-0410.2009.01188.x
Miron RJ, 2020, BMC ORAL HEALTH, V20, DOI 10.1186/s12903-020-01299-w
Mohammadi S, 2017, TRANSFUS APHER SCI, V56, P226, DOI 10.1016/j.transci.2016.12.032
Moojen DJF, 2008, J ORTHOP RES, V26, P404, DOI 10.1002/jor.20519
Muchedzi TA, 2018, SURG-J R COLL SURG E, V16, P250, DOI 10.1016/j.surge.2017.08.004
Ogle ME, 2016, EXP BIOL MED, V241, P1084, DOI 10.1177/1535370216650293
Ozel I, 2022, CANCERS, V14, DOI 10.3390/cancers14030536
Page G, 2013, INT IMMUNOPHARMACOL, V17, P1176, DOI 10.1016/j.intimp.2013.06.004
Nguyen PA, 2018, J APPL ORAL SCI, V26, DOI 10.1590/1678-7757-2018-0077
Piao L, 2017, PLOS ONE, V12, DOI 10.1371/journal.pone.0187509
Podesta L, 2013, AM J SPORT MED, V41, P1689, DOI 10.1177/0363546513487979
Puzzitiello Richard N, 2020, Arthrosc Sports Med Rehabil, V2, pe161, DOI 10.1016/j.asmr.2020.01.002
Rendu F, 2001, PLATELETS, V12, P261, DOI 10.1080/09537100120068170
Repsold L, 2018, BIOMED RES INT, V2018, DOI 10.1155/2018/9405617
Richardson TP, 2001, NAT BIOTECHNOL, V19, P1029, DOI 10.1038/nbt1101-1029
Rodeo SA, 2012, AM J SPORT MED, V40, P1234, DOI 10.1177/0363546512442924
Rossi LA, 2021, ARTHROSCOPY, V37, P2745, DOI 10.1016/j.arthro.2021.03.079
Sadtler K, 2016, SCIENCE, V352, P366, DOI 10.1126/science.aad9272
Sakharkar N.S., 2022, INT J RECENT SURG ME, DOI [10.1055/s-0042-1751245, DOI 10.1055/S-0042-1751245]
Samadi P, 2019, AESTHET PLAST SURG, V43, P803, DOI 10.1007/s00266-018-1293-9
Schaer DJ, 2013, BLOOD, V121, P1276, DOI 10.1182/blood-2012-11-451229
Scheuerer B, 2000, BLOOD, V95, P1158, DOI 10.1182/blood.V95.4.1158.004k31_1158_1166
Senzel L, 2009, CURR OPIN HEMATOL, V16, P329, DOI 10.1097/MOH.0b013e32832e9dc6
Serhan CN, 2005, NAT IMMUNOL, V6, P1191, DOI 10.1038/ni1276
SERHAN CN, 1990, J CLIN INVEST, V85, P772, DOI 10.1172/JCI114503
Sommer C, 2004, MOL NEUROBIOL, V30, P117, DOI 10.1385/MN:30:2:117
Sprott H, 1998, RHEUMATOL INT, V18, P35, DOI 10.1007/s002960050051
Sugimoto MA, 2016, FRONT IMMUNOL, V7, DOI 10.3389/fimmu.2016.00160
Sweed T, 2021, KNEE SURG RELAT RES, V33, DOI 10.1186/s43019-021-00110-6
Tang YQ, 2002, INFECT IMMUN, V70, P6524, DOI 10.1128/IAI.70.12.6524-6533.2002
Tuakli-Wosornu YA, 2016, PM&R, V8, P1, DOI 10.1016/j.pmrj.2015.08.010
Uchiyama R, 2021, INT J MOL SCI, V22, DOI 10.3390/ijms22052336
Urits I, 2020, PAIN THER, V9, P145, DOI 10.1007/s40122-020-00157-5
Vasina EM, 2011, CELL DEATH DIS, V2, DOI 10.1038/cddis.2011.94
Verhaegen F, 2016, J SHOULDER ELB SURG, V25, P169, DOI 10.1016/j.jse.2015.10.009
Walsh TG, 2015, PLATELETS, V26, P199, DOI 10.3109/09537104.2014.909022
Weirather J, 2014, CIRC RES, V115, P55, DOI 10.1161/CIRCRESAHA.115.303895

   Wu WP, 2003, EUR J PHARMACOL, V478, P131, DOI
10.1016/j.ejphar.2003.08.047
   Wunderli SL, 2020, MATRIX BIOL, V89, P11, DOI
10.1016/j.matbio.2019.12.003
   Wynn TA, 2016, IMMUNITY, V44, P450, DOI 10.1016/j.immuni.2016.02.015
   Xing LY, 2004, CELL IMMUNOL, V231, P126, DOI
10.1016/j.cellimm.2004.12.008
   Xu J, 2020, AUST DENT J, V65, P131, DOI 10.1111/adj.12754
   Yoshida M, 2016, MLTJ-MUSCLE LIGAMENT, V6, P205, DOI
10.11138/mltj/2016.6.2.205
   Zheng CB, 2016, J TISSUE ENG REGEN M, V10, P428, DOI 10.1002/term.1756
   Zhou YQ, 2015, STEM CELL RES THER, V6, DOI 10.1186/s13287-015-0172-4
NR 141
TC 15
Z9 15
U1 0
U2 2
PU MDPI
PI BASEL
PA ST ALBAN-ANLAGE 66, CH-4052 BASEL, SWITZERLAND
EI 2227-9059
J9 BIOMEDICINES
JI Biomedicines
PD NOV
PY 2022
VL 10
IS 11
AR 2933
DI 10.3390/biomedicines10112933
PG 27
WC Biochemistry & Molecular Biology; Medicine, Research & Experimental;
   Pharmacology & Pharmacy
WE Science Citation Index Expanded (SCI-EXPANDED)
SC Biochemistry & Molecular Biology; Research & Experimental Medicine;
   Pharmacology & Pharmacy
GA 6U4TQ
UT WOS:000894361400001
PM 36428501
OA Green Published, gold
DA 2025-05-07
ER

PT J
AU Pepper, JW
AF Pepper, John W.
TI Drugs that target pathogen public goods are robust against evolved
drug
   resistance
SO EVOLUTIONARY APPLICATIONS
LA English
DT Article
DE biomedicine; cancer medicine; contemporary evolution; disease biology;
   evolutionary medicine; evolutionary theory; experimental evolution;
   microbial biology; natural selection

ID DIGITAL EVOLUTION; GROUP SELECTION; MECHANISMS; COVARIANCE
AB Pathogen drug resistance is a central problem in medicine and public health. It arises through somatic evolution, by mutation and selection among pathogen cells within a host. Here, we examine the hypothesis that evolution of drug resistance could be reduced by developing drugs that target the secreted metabolites produced by pathogen cells instead of directly targeting the cells themselves. Using an agent-based computational model of an evolving population of pathogen cells, we test this hypothesis and find support for it. We also use our model to explain this effect within the framework of standard evolutionary theory. We find that in our model, the drugs most robust against evolved drug resistance are those that target the most widely shared external products, or public goods, of pathogen cells. We also show that these drugs exert a weak selective pressure for resistance because they create only a weak correlation between drug resistance and cell fitness. The same principles apply to design of vaccines that are robust against vaccine escape. Because our theoretical results have crucial practical implications, they should be tested by empirical experiments.
C1 [Pepper, John W.] NCI, Canc Prevent Div, Bethesda, MD 20892 USA.
[Pepper, John W.] Santa Fe Inst, Santa Fe, NM 87501 USA.
C3 National Institutes of Health (NIH) - USA; NIH National Cancer Institute
(NCI); The Santa Fe Institute
RP Pepper, JW (autor correspondente), NCI, Canc Prevent Div, 6130 Execut Blvd,MSC 7315, Bethesda, MD 20892 USA.
EM pepperjw@mail.nih.gov
RI Pepper, John/W-5578-2019; Pepper, John/O-1662-2013
OI Pepper, John/0000-0001-9888-0437
FU NSF DDIG [1010669]; Direct For Biological Sciences; Division Of Integrative Organismal Systems [1010669] Funding Source: National Science Foundation
FX My doctoral student William W. Driscoll chose not to be listed as co-author on this paper, but was fully qualified to be. He wrote most of the software, carried out simulation experiments, collected results, and contributed substantially to writing. He was supported by NSF DDIG #1010669.
CR Adami C, 2004, ARTIF LIFE, V10, P117, DOI 10.1162/106454604773563540
André JB, 2005, ECOL LETT, V8, P800, DOI 10.1111/j.1461-0248.2005.00783.x
Driscoll WW, 2010, EVOLUTION, V64, P2682, DOI 10.1111/j.1558-5646.2010.01002.x
El'Garch F, 2007, ANTIMICROB AGENTS CH, V51, P1016, DOI 10.1128/AAC.00704-06
Fong I.W., 2003, Reemergence of Established Pathogens in the 21st Century
Hamilton W. D., 1975, Biosocial Anthropology., V133, P115
Lee HH, 2010, NATURE, V467, P82, DOI 10.1038/nature09354
Moscow Jeffrey, 2003, CANC MED, V6
O'Connor R, 2007, EXPERT OPIN DRUG MET, V3, P805, DOI 10.1517/17425255.3.6.805
Okasha S, 2004, BIOL PHILOS, V19, P167, DOI 10.1023/B:BIPH.0000024406.61340.6c

Pennock RT, 2007, J EXP THEOR ARTIF IN, V19, P29, DOI 10.1080/09528130601116113
Pepper JW, 2007, ARTIF LIFE, V13, P1, DOI 10.1162/artl.2007.13.1.1
Pepper JW, 2008, EVOLUTION, V62, P3185, DOI 10.1111/j.1558-5646.2008.00525.x
PRICE GR, 1972, ANN HUM GENET, V35, P485, DOI 10.1111/j.1469-1809.1957.tb01874.x
PRICE GR, 1970, NATURE, V227, P520, DOI 10.1038/227520a0
Stewart PS, 2003, J BACTERIOL, V185, P1485, DOI 10.1128/JB.185.5.1485-1491.2003
WADE MJ, 1985, AM NAT, V125, P61, DOI 10.1086/284328
West SA, 2007, J EVOLUTION BIOL, V20, P415, DOI 10.1111/j.1420-9101.2006.01258.x
NR 18
TC 36
Z9 44
U1 1
U2 27
PU WILEY-BLACKWELL
PI HOBOKEN
PA 111 RIVER ST, HOBOKEN 07030-5774, NJ USA
SN 1752-4571
J9 EVOL APPL
JI Evol. Appl.
PD NOV
PY 2012
VL 5
IS 7
BP 757
EP 761
DI 10.1111/j.1752-4571.2012.00254.x
PG 5
WC Evolutionary Biology
WE Science Citation Index Expanded (SCI-EXPANDED)
SC Evolutionary Biology
GA 030BW
UT WOS:000310543800009
PM 23144661
OA gold, Green Published
DA 2025-05-07
ER

PT J
AU Mitteroecker, P
Fischer, B
AF Mitteroecker, Philipp
Fischer, Barbara
TI Evolution of the human birth canal
SO AMERICAN JOURNAL OF OBSTETRICS AND GYNECOLOGY
LA English
DT Review
DE biocultural evolution; bipedality; cesarean delivery; childbirth;
evolutionary medicine; human evolution; pelvic floor disorders;
pelvis;

obstetrical dilemma; relaxin
ID PELVIC ORGAN PROLAPSE; SERUM RELAXIN CONCENTRATION; CLIFF-EDGE MODEL; HUMAN-BODY SIZE; SEXUAL-DIMORPHISM; PUBIC SYMPHYSIS; BONY PELVIS; NATURAL-SELECTION; FLOOR DYSFUNCTION; POCKET GOPHER
AB It seems puzzling why humans have evolved such a small and rigid birth canal that entails a relatively complex process of labor compared with the birth canal of our closest relatives, the great apes. This study reviewed insights into the evolution of the human birth canal from recent theoretical and empirical studies and discussed connections to obstetrics, gynecology, and orthopedics. Originating from the evolution of bipedality and the large human brain million years ago, the evolution of the human birth canal has been characterized by complex trade-off dynamics among multiple biological, environmental, and sociocultural factors. The long-held notion that a wider pelvis has not evolved because it would be disadvantageous for bipedal locomotion has not yet been empirically verified. However, recent clinical and biomechanical studies suggest that a larger birth canal would compromise pelvic floor stability and increase the risk of incontinence and pelvic organ prolapse. Several mammals have neonates that are equally large or even larger than human neonates compared to the size of the maternal birth canal. In these species, the pubic symphysis opens widely to allow successful delivery. Biomechanical and developmental constraints imposed by bipedality have hindered this evolutionary solution in humans and led to the comparatively rigid pelvic girdle in pregnant women. Mathematical models have shown why the evolutionary compromise to these antagonistic selective factors inevitably involves a certain rate of fetopelvic disproportion. In addition, these models predict that cesarean deliveries have disrupted the evolutionary equilibrium and led to new and ongoing evolutionary changes. Different forms of assisted birth have existed since the stone age and have become an integral part of human reproduction. Paradoxically, by buffering selection, they may also have hindered the evolution of a larger birth canal. Many of the biological, environmental, and sociocultural factors that have influenced the evolution of the human birth canal vary globally and are subject to ongoing transitions. These differences may have contributed to the global variation in the form of the birth canal and the difficulty of labor, and they likely continue to change human reproductive anatomy.
C1 [Mitteroecker, Philipp; Fischer, Barbara] Univ Vienna, Dept Evolutionary Biol, Unit Theoret Biol, Vienna, Austria.
C3 University of Vienna
RP Mitteroecker, P (autor correspondente), Univ Vienna, Dept Evolutionary Biol, Unit Theoret Biol, Vienna, Austria.
EM philipp.mitteroecker@univie.ac.at
RI Mitteroecker, Philipp/A-1591-2012
OI Mitteroecker, Philipp/0000-0002-5308-3837; Fischer, Barbara/0000-0002-4492-8906
FU Austrian Science Fund, Elise Richter Project [826-B]
FX This study received funding from the Austrian Science Fund, Elise Richter Project 826-B.
CR ABITBOL MM, 1988, AM J PHYS ANTHROPOL, V75, P53, DOI 10.1002/ajpa.1330750107
ALBERMAN E, 1991, J ROY SOC MED, V84, P257, DOI 10.1177/014107689108400505
Aldabe D, 2012, EUR SPINE J, V21, P1769, DOI 10.1007/s00586-012-2162-x

Allen J. A., 1877, Radical Review, Vi, P108
Arrowsmith S, 1996, Obstet Gynecol Surv, V51, P568, DOI 10.1097/00006254-199609000-00024
Ashton-Miller JA, 2009, ANNU REV BIOMED ENG, V11, P163, DOI 10.1146/annurev-bioeng-061008-124823
Awwad J, 2012, MENOPAUSE, V19, P1235, DOI 10.1097/gme.0b013e31826d2d94
Barabási AL, 2007, IEEE CONTR SYST MAG, V27, P33, DOI 10.1109/MCS.2007.384127
Barnawi N., 2013, J Res Nurs Midwif, V2, P114, DOI [10.14303/JRNM.2013.064, DOI 10.14303/JRNM.2013.064]
Becker I, 2010, J ANAT, V217, P475, DOI 10.1111/j.1469-7580.2010.01300.x
Bell G, 2017, ANNU REV ECOL EVOL S, V48, P605, DOI 10.1146/annurev-ecolsys-110316-023011
Bentham J, 2016, ELIFE, V5, DOI 10.7554/eLife.13410.001
Bergmann C., 1848, UEBER VERHALTNISSE W
Betrán AP, 2016, PLOS ONE, V11, DOI 10.1371/journal.pone.0148343
Betti L, 2018, P ROY SOC B-BIOL SCI, V285, DOI 10.1098/rspb.2018.1807
Bitti GT, 2014, RADIOGRAPHICS, V34, P429, DOI 10.1148/rg.342125050
Blencowe H, 2019, LANCET GLOB HEALTH, V7, pE849, DOI 10.1016/S2214-109X(18)30565-5
Bo K, 2004, SPORTS MED, V34, P451
Bo K, 2020, SPORTS MED, V50, P471, DOI 10.1007/s40279-019-01243-1
BRESLER JB, 1962, AM J PHYS ANTHROPOL, V20, P515, DOI 10.1002/ajpa.1330200412
Brown KM, 2013, INT UROGYNECOL J, V24, P431, DOI 10.1007/s00192-012-1876-y
CAMILLERI AP, 1981, EUR J OBSTET GYN R B, V12, P347, DOI 10.1016/0028-2243(81)90080-0
Catullo RA, 2019, CURR BIOL, V29, pR996, DOI 10.1016/j.cub.2019.08.028
Cosminsky Sheila., 1976, MED ANTHROPOL, P229
CRELIN ES, 1969, ANAT RECORD, V164, P349, DOI 10.1002/ar.1091640310
DALSTRA M, 1995, J BIOMECH, V28, P715, DOI 10.1016/0021-9290(94)00125-N
Davenport MH, 2019, BRIT J SPORT MED, V53, P99, DOI 10.1136/bjsports-2018-099821
Dawkins R., 1982, pi
de León MSP, 2008, P NATL ACAD SCI USA, V105, P13764, DOI 10.1073/pnas.0803917105
Derraik JGB, 2016, PLOS ONE, V11, DOI 10.1371/journal.pone.0154304
Dragoo JL, 2011, AM J SPORT MED, V39, P2175, DOI 10.1177/0363546511413378
Dragoo JL, 2011, INT J WOMENS HEALTH, V3, P19, DOI 10.2147/IJWH.S14188
Dunsworth H, 2015, ANNU REV ANTHROPOL, V44, P55, DOI 10.1146/annurev-anthro-102214-013918
Dunsworth HM, 2018, PERSPECT BIOL MED, V61, P249, DOI 10.1353/pbm.2018.0040
Dunsworth HM, 2012, P NATL ACAD SCI USA, V109, P15212, DOI 10.1073/pnas.1205282109
El Sayed RF, 2017, EUR RADIOL, V27, P2067, DOI 10.1007/s00330-016-4471-7
Facciolli A, 2009, ANN NY ACAD SCI, V1160, P221, DOI 10.1111/j.1749-6632.2008.03788.x

Feldman MW, 1996, TRENDS ECOL EVOL, V11, P453, DOI 10.1016/0169-5347(96)10052-5
Ferner K, 2017, J ANAT, V231, P798, DOI 10.1111/joa.12689
Fischer B., 2021, BMSAP, V33, P10
Fischer B, 2021, NAT ECOL EVOL, V5, P625, DOI 10.1038/s41559-021-01425-z
Fischer B, 2017, ANAT REC, V300, P698, DOI 10.1002/ar.23549
Fischer B, 2015, P NATL ACAD SCI USA, V112, P5655, DOI 10.1073/pnas.1420325112
Fremondiere P, 2016, P 6 M EUR SOC STUD H
Frémondière P, 2022, COMMUN BIOL, V5, DOI 10.1038/s42003-022-03321-z
GOLDSMITH LT, 1994, BIOL REPROD, V50, P258, DOI 10.1095/biolreprod50.2.258
Greenberg MB, 2006, AM J OBSTET GYNECOL, V195, P743, DOI 10.1016/j.ajog.2006.06.016
Greulich W W, 1944, Yale J Biol Med, V17, P91
Grossman R, 2017, P NATL ACAD SCI USA, V114, pE1304, DOI 10.1073/pnas.1620160114
Grunstra NDS, 2019, AM J HUM BIOL, V31, DOI 10.1002/ajhb.23227
Gruss LT, 2017, ANAT REC, V300, P739, DOI 10.1002/ar.23550
Gruss LT, 2015, PHILOS T R SOC B, V370, DOI 10.1098/rstb.2014.0063
Haeusler M, 2021, BIOL REV, V96, P2031, DOI 10.1111/brv.12744
Haeusler M, 2016, AM J PHYS ANTHROPOL, V159, P165
Handa VL, 2009, INT UROGYNECOL J, V20, P133, DOI 10.1007/s00192-008-0736-2
Handa VL, 2003, OBSTET GYNECOL, V102, P1283, DOI 10.1016/j.obstetgynecol.2003.08.022
HASS JD, 1980, HUM BIOL, V52, P459
Hazlett HC, 2017, NATURE, V542, P348, DOI 10.1038/nature21369
Hirata S, 2011, BIOL LETTERS, V7, P686, DOI 10.1098/rsbl.2011.0214
Hisaw FL, 1925, J EXP ZOOL, V42, P411, DOI 10.1002/jez.1400420403
Hisaw FL, 1924, AM NAT, V58, P93, DOI 10.1086/279962
HISAW FL, 1950, VITAM HORM, V8, P151, DOI 10.1016/S0083-6729(08)60670-6
Horikoshi M, 2016, NATURE, V538, P248, DOI 10.1038/nature19806
Hublin JJ, 2015, PHILOS T R SOC B, V370, DOI 10.1098/rstb.2014.0062
Huey RB, 2003, AM NAT, V161, P357, DOI 10.1086/346135
IGUCHI T, 1989, ANAT RECORD, V224, P490, DOI 10.1002/ar.1092240406
Islam RM, 2019, INT UROGYNECOL J, V30, P2001, DOI 10.1007/s00192-019-03992-z
Katzmarzyk PT, 1998, AM J PHYS ANTHROPOL, V106, P483, DOI 10.1002/(SICI)1096-8644(199808)106:4<483::AID-AJPA4>3.3.CO;2-K
Kibii JM, 2011, SCIENCE, V333, P1407, DOI 10.1126/science.1202521
Kirchengast S, 2003, AM J HUM BIOL, V15, P220, DOI 10.1002/ajhb.10139
Krebs V, 2009, CLIN ORTHOP RELAT R, V467, P868, DOI 10.1007/s11999-008-0317-1
Krogman W. M., 1951, Scientific American, V185, P54
Kruger JA, 2007, ULTRASOUND OBST GYN, V30, P81, DOI 10.1002/uog.4027
Kurki Helen K, 2013, Am J Hum Biol, V25, P795, DOI 10.1002/ajhb.22455
Kurki HK, 2013, AM J PHYS ANTHROPOL, V151, P88, DOI 10.1002/ajpa.22243
LANDE R, 1980, EVOLUTION, V34, P292, DOI [10.2307/2407393, 10.1111/j.1558-5646.1980.tb04817.x]
Laudicina NM, 2019, PLOS ONE, V14, DOI 10.1371/journal.pone.0221871

LAVELLE M, 1995, AM J PHYS ANTHROPOL, V98, P59, DOI 10.1002/ajpa.1330980106
Leutenegger W., 1985, P33
LEUTENEGGER W, 1978, NATURE, V272, P610, DOI 10.1038/272610a0
Li R, 2015, CLIN ANAT, V28, P813, DOI 10.1002/ca.22536
Li XS, 2008, LECT NOTES COMPUT SC, V5242, P750
Lien KC, 2004, OBSTET GYNECOL, V103, P31, DOI 10.1097/01.AOG.0000109207.22354.65
Logan ML, 2019, OECOLOGIA, V191, P817, DOI 10.1007/s00442-019-04541-7
Lovejoy CO, 2005, GAIT POSTURE, V21, P95, DOI 10.1016/j.gaitpost.2004.01.001
MacKay DF, 2010, PLOS MED, V7, DOI 10.1371/journal.pmed.1000289
MACLENNAN AH, 1986, LANCET, V2, P243
MEREDITH HV, 1970, HUM BIOL, V42, P217
Mikolajczyk RT, 2011, LANCET, V377, P1855, DOI 10.1016/S0140-6736(11)60364-4
Milsom I, 2019, CLIMACTERIC, V22, P217, DOI 10.1080/13697137.2018.1543263
Mitteroecker P, 2017, P NATL ACAD SCI USA, V114, P11669, DOI 10.1073/pnas.1712203114
Mitteroecker P, 2017, P NATL ACAD SCI USA, V114, pE1305, DOI 10.1073/pnas.1621176114
Mitteroecker P, 2016, P NATL ACAD SCI USA, V113, P14680, DOI 10.1073/pnas.1612410113
Moffett EA, 2017, ANAT REC, V300, P870, DOI 10.1002/ar.23572
MOLLOY WB, 1969, OBSTET GYNECOL, V34, P330
Montagu Ashely., 1989, GROWING YOUNG, VSecond
Morgan PM, 2013, HIP INT, V23, P212, DOI 10.5301/HIP.2013.10729
Muñoz MM, 2022, EVOLUTION, V76, P49, DOI 10.1111/evo.14388
Neuweiler G., BIOL BATS
Nissen HW, 1943, ANAT RECORD, V86, P567, DOI 10.1002/ar.1090860407
Nitti V W, 2001, Rev Urol, V3 Suppl 1, pS2
Owens K, 2002, EUR J OBSTET GYN R B, V105, P143
Parker EA, 2022, FRONT ENDOCRINOL, V13, DOI 10.3389/fendo.2022.827512
Patel RR, 2004, INT J EPIDEMIOL, V33, P107, DOI 10.1093/ije/dyg238
Pavlicev M, 2020, AM J OBSTET GYNECOL, V222, P3, DOI 10.1016/j.ajog.2019.06.043
Pedersen DR, 1997, J BIOMECH, V30, P959, DOI 10.1016/S0021-9290(97)00041-9
Pommerenke WT, 1933, ANAT RECORD, V57, P361, DOI 10.1002/ar.1090570407
Portmann A., 1969, Biologische Fragmente zu einer Lehre vom Menschen, P3
Reyes-Amaya N, 2017, ANAT REC, V300, P2150, DOI 10.1002/ar.23646
Rhodes AML, 2014, J CHILD ORTHOP, V8, P375, DOI 10.1007/s11832-014-0615-y
Rice F, 2010, EARLY HUM DEV, V86, P425, DOI 10.1016/j.earlhumdev.2010.05.021
Rich SA, 2008, Omertaa, J Appl Anthropol, P260
RIDLEY M, 1995, AM J PHYS ANTHROPOL, V97, P197, DOI 10.1002/ajpa.1330970209
Roff D. A., 1997, Evolutionary quantitative genetics
Ronchetti I, 2008, SPINE, V33, pE145, DOI 10.1097/BRS.0b013e3181657f03
Rosenberg K, 2002, BJOG-INT J OBSTET GY, V109, P1199, DOI 10.1016/S1470-0328(02)00410-X

Rosenberg Karen, 1995, Evolutionary Anthropology, V4, P161, DOI 10.1002/evan.1360040506
ROSENBERG KR, 1992, YEARB PHYS ANTHROPOL, V35, P89
Ruff C, 2002, ANNU REV ANTHROPOL, V31, P211, DOI 10.1146/annurev.anthro.31.040402.085407
Ruff CB, 1997, NATURE, V387, P173, DOI 10.1038/387173a0
RUFF CB, 1991, J HUM EVOL, V21, P81, DOI 10.1016/0047-2484(91)90001-C
RUFF CB, 1995, AM J PHYS ANTHROPOL, V98, P527, DOI 10.1002/ajpa.1330980412
Ruff Christopher B., 1994, Yearbook of Physical Anthropology, V37, P65
Ruth EB, 1937, ANAT RECORD, V67, P409, DOI 10.1002/ar.1090670404
SAUNDERS JBDM, 1953, J BONE JOINT SURG AM, V35-A, P543, DOI 10.2106/00004623-195335030-00003
Savell KRR, 2016, P NATL ACAD SCI USA, V113, P9492, DOI 10.1073/pnas.1603632113
Scheuer DL, 2014, Developmental juvenile osteology
SCHULTZ A H, 1969, P281
Schultz AH, 1949, AM J PHYS ANTHROP-NE, V7, P401, DOI 10.1002/ajpa.1330070307
SCHWABE C, 1990, COMP BIOCHEM PHYS B, V96, P15, DOI 10.1016/0305-0491(90)90335-Q
Simon H. A., 1969, The sciences of the artificial
Simpson SW, 2008, SCIENCE, V322, P1089, DOI 10.1126/science.1163592
Stansfield E, 2021, BMC BIOL, V19, DOI 10.1186/s12915-021-01150-w
Stansfield E, 2021, P NATL ACAD SCI USA, V118, DOI 10.1073/pnas.2022159118
Stein TA, 2009, AM J OBSTET GYNECOL, V200, DOI 10.1016/j.ajog.2008.10.040
Steinetz BG, 2008, AM J VET RES, V69, P59, DOI 10.2460/ajvr.69.1.59
Stone PK, 2016, AM J PHYS ANTHROPOL, V159, P150, DOI 10.1002/ajpa.22906
Sze EHM, 1999, OBSTET GYNECOL, V93, P229, DOI 10.1016/S0029-7844(98)00376-7
Tague RG, 2005, AM J PHYS ANTHROPOL, V127, P392, DOI 10.1002/ajpa.20226
Tague RG, 2003, J MAMMAL, V84, P1464, DOI 10.1644/BME-009
Tegerstedt G, 2005, INT UROGYNECOL J, V16, P497, DOI 10.1007/s00192-005-1326-1
Tilkens MJ, 2007, J HUM EVOL, V53, P286, DOI 10.1016/j.jhevol.2007.04.005
Todd TW, 1923, AM J ANAT, V31, P345, DOI 10.1002/aja.1000310403
Trevathan W.R., 2017, Human birth: An evolutionary perspective
UESUGI Y, 1992, ANAT REC, V234, P541, DOI 10.1002/ar.1092340409
Urban MC, 2013, NAT COMMUN, V4, DOI 10.1038/ncomms3085
Vergeldt TFM, 2015, INT UROGYNECOL J, V26, P1559, DOI 10.1007/s00192-015-2695-8
Vidal-Cordasco M, 2017, AM J PHYS ANTHROPOL, V164, P609, DOI 10.1002/ajpa.23301
Villar J, 2014, LANCET, V384, P857, DOI 10.1016/S0140-6736(14)60932-6
Vleeming A, 2008, EUR SPINE J, V17, P794, DOI 10.1007/s00586-008-0602-4
Vogel I, 1998, J PEDIATR ORTHOPED, V18, P535, DOI 10.1097/00004694-199807000-00026
Walker A., 1993, NARIOKOTOME HOMO ERE, P221

Walker GJA, 2011, INT UROGYNECOL J, V22, P127, DOI 10.1007/s00192-010-1215-0
Wall LL, 2006, LANCET, V368, P1201, DOI 10.1016/S0140-6736(06)69476-2
Wall LL, 1999, J WOMENS HEALTH, V8, P149, DOI 10.1089/jwh.1999.8.149
Wall-Scheffler CM, 2007, AM J PHYS ANTHROPOL, V133, P841, DOI 10.1002/ajpa.20603
Wall-Scheffler CM, 2017, ANAT REC, V300, P764, DOI 10.1002/ar.23553
Wall-Scheffler CM, 2013, J HUM EVOL, V64, P448, DOI 10.1016/j.jhevol.2013.01.014
Waring TM, 2021, P ROY SOC B-BIOL SCI, V288, DOI 10.1098/rspb.2021.0538
Warrener AG, 2015, PLOS ONE, V10, DOI 10.1371/journal.pone.0118903
Weaver TD, 2009, P NATL ACAD SCI USA, V106, P8151, DOI 10.1073/pnas.0812554106
Wells JCK, 2021, FRONT PHYSIOL, V12, DOI 10.3389/fphys.2021.696516
Wells JCK, 2018, FRONT PUBLIC HEALTH, V6, DOI 10.3389/fpubh.2018.00292
Wells JCK, 2015, PHILOS T R SOC B, V370, DOI 10.1098/rstb.2014.0067
Wells JCK, 2012, AM J PHYS ANTHROPOL, V149, P40, DOI 10.1002/ajpa.22160
Whitcome KK, 2017, ANAT REC, V300, P752, DOI 10.1002/ar.23551
Wilcox AJ, 2001, INT J EPIDEMIOL, V30, P1233, DOI 10.1093/ije/30.6.1233
Yasar L, 2019, INT UROGYNECOL J, V30, P271, DOI 10.1007/s00192-018-3663-x
Zaffarini E, 2019, P ROY SOC B-BIOL SCI, V286, DOI 10.1098/rspb.2018.2425
NR 169
TC 7
Z9 8
U1 4
U2 8
PU MOSBY-ELSEVIER
PI NEW YORK
PA 360 PARK AVENUE SOUTH, NEW YORK, NY 10010-1710 USA
SN 0002-9378
EI 1097-6868
J9 AM J OBSTET GYNECOL
JI Am. J. Obstet. Gynecol.
PD MAR
PY 2024
VL 230
IS 3
SU S
BP S841
EP S855
DI 10.1016/j.ajog.2022.09.010
EA MAR 2024
PG 15
WC Obstetrics & Gynecology
WE Science Citation Index Expanded (SCI-EXPANDED)
SC Obstetrics & Gynecology
GA A5G7E
UT WOS:001282818400014
PM 38462258

OA hybrid
DA 2025-05-07
ER

PT J
AU Vinogradov, AE
Anatskaya, OV
AF Vinogradov, Alexander E.
Anatskaya, Olga, V
TI Gradistics: An underappreciated dimension in evolutionary space
SO BIOSYSTEMS
LA English
DT Article
DE Complexity; Ecological niches; Regulation; Memory; Nervous system; Regulatory genes; Epigenetics; Regulatory adaptation; Whole-genome duplication; Evolutionary medicine
ID TRANSCRIPTION FACTORS; COMPLEXITY; MAMMALS; POPULATIONS; PLASTICITY; ENERGETICS; FRAMEWORK; REVEALS; BIOLOGY; NUMBER
AB The growth of complexity is an unsolved and underappreciated problem. We consider possible causes of this growth, hypotheses testing, molecular mechanisms, complexity measures, cases of simplification, and significance for biomedicine. We focus on a general ability of regulation, which is based on the growing information storage and processing capacities, as the main proxy of complexity. Natural selection is indifferent to complexity. However, complexification can be inferred from the same first principle, on which natural selection is founded. Natural selection depends on potentially unlimited reproduction under limited environmental conditions. Because of the demographic pressure, the simple ecological niches become fulfilled and diversified (due to species splitting and divergence). Diversification increases complexity of biocenoses. After the filling and diversification of simple niches, the more complex niches can arise. This is the 'atomic orbitals' (AO) model. Complexity has many shortcomings but it has an advantage. This advantage is ability to regulatory adaptation, including behavioral, formed in the evolution by means of genetic adaptation. Regulatory adaptation is much faster than genetic one because it is based on the information previously accumulated via genetic adaptation and learning. Regulatory adaptation further increases complexity of biocenoses. This is the 'regulatory advantage' (RA) model. The comparison of both models allows testable predictions. We focus on the animal evolution because of the appearance of higher regulatory level (nervous system), which is absent in other lineages, and relevance to humans (including biomedical aspects).
C1 [Vinogradov, Alexander E.; Anatskaya, Olga, V] Russian Acad Sci, Inst Cytol, St Petersburg 194064, Russia.
C3 Russian Academy of Sciences; St. Petersburg Scientific Centre of the Russian Academy of Sciences; Institute of Cytology RAS
RP Vinogradov, AE (autor correspondente), Russian Acad Sci, Inst Cytol, St Petersburg 194064, Russia.
EM aevin@incras.ru
RI Anatskaya, Olga/R-4042-2016; Vinogradov, Alexander/R-3923-2016
FU Institute of Cytology; Institute of Cytology
FX We thank the anonymous reviewers for valuable comments. The authors were
supported by the Institute of Cytology.

CR Abelson ES, 2019, OECOLOGIA, V191, P721, DOI 10.1007/s00442-019-04527-5
Abzhanov A, 2013, TRENDS GENET, V29, P712, DOI 10.1016/j.tig.2013.09.004
Anatskaya OV, 2022, INT J MOL SCI, V23, DOI 10.3390/ijms23073542
[Anonymous], 1970, Evolution by Gene Duplication
[Anonymous], 2016, RETROGRADE EVOLUTION
[Anonymous], 2002, The structure of evolutionary theory, DOI [10.4159/9780674417922, DOI 10.4159/9780674417922]
Bains William, 2016, Life-Basel, V6, P25, DOI 10.3390/life6030025
Baker EA, 2019, J DEV BIOL, V7, DOI 10.3390/jdb7040019
Bell G, 1997, BIOL J LINN SOC, V60, P345, DOI 10.1111/j.1095-8312.1997.tb01500.x
Benevento GL, 2019, P ROY SOC B-BIOL SCI, V286, DOI 10.1098/rspb.2019.0347
Bernhardt JR, 2020, PHILOS T R SOC B, V375, DOI 10.1098/rstb.2019.0454
Bizzarri M, 2020, ENTROPY-SWITZ, V22, DOI 10.3390/e22080885
Boddy AM, 2012, J EVOLUTION BIOL, V25, P981, DOI 10.1111/j.1420-9101.2012.02491.x
Brette R, 2021, ENEURO, V8, DOI 10.1523/ENEURO.0018-21.2021
Brocklehurst N, 2021, CURR BIOL, V31, P2955, DOI 10.1016/j.cub.2021.04.044
Chen CY, 2012, BIOINFORMATICS, V28, P3178, DOI 10.1093/bioinformatics/bts623
Chen L, 2014, MOL BIOL EVOL, V31, P1402, DOI 10.1093/molbev/msu083
Chen M, 2019, P NATL ACAD SCI USA, V116, P9931, DOI 10.1073/pnas.1820863116
Cheng F, 2018, NAT PLANTS, V4, P258, DOI 10.1038/s41477-018-0136-7
Claverie JM, 2001, SCIENCE, V291, P1255, DOI 10.1126/science.1058969
Coghlan Avril, 2005, P1
Corradetti B., 2021, WORLD J PEDIATR
Court F, 2017, ONCOTARGET, V8, P4110, DOI 10.18632/oncotarget.13746
Cowan N, 2019, PSYCHOL BULL, V145, DOI 10.1037/bul0000199
Davies PCW, 2011, PHYS BIOL, V8, DOI 10.1088/1478-3975/8/1/015001
Dicke U, 2016, PHILOS T R SOC B, V371, DOI 10.1098/rstb.2015.0180
Diogo R, 2015, BIOL REV, V90, P502, DOI 10.1111/brv.12121
Du K, 2020, NAT ECOL EVOL, V4, P841, DOI 10.1038/s41559-020-1166-x
Enriquez-Gasca R, 2020, VIRUSES-BASEL, V12, DOI 10.3390/v12101089
Erwin DH, 2020, DEVELOPMENT, V147, DOI 10.1242/dev.182899
Fernandes HBF, 2020, INTELLIGENCE, V80, DOI 10.1016/j.intell.2020.101456
Gao C, 2021, SCI REP-UK, V11, DOI 10.1038/s41598-021-82656-9
Gates AJ, 2016, SCI REP-UK, V6, DOI 10.1038/srep24456
Giuliani A, 2022, GENOMICS, V114, P215, DOI 10.1016/j.ygeno.2021.11.027
Gorban AN, 2022, PHYS LIFE REV, V40, P15, DOI 10.1016/j.plrev.2021.10.002
Gorban AN, 2021, PHYS LIFE REV, V37, P17, DOI 10.1016/j.plrev.2021.03.001
Grossnickle DM, 2019, TRENDS ECOL EVOL, V34, P936, DOI 10.1016/j.tree.2019.05.008
Hahn MW, 2002, EVOL DEV, V4, P73, DOI 10.1046/j.1525-142X.2002.01069.x
Hay WW, 2019, INT J EARTH SCI, V108, P587, DOI 10.1007/s00531-018-1670-2

Heim NA, 2017, P ROY SOC B-BIOL SCI, V284, DOI 10.1098/rspb.2017.1039
Henrich J, 2021, ANNU REV PSYCHOL, V72, P207, DOI 10.1146/annurev-psych-081920-042106
Huxley J., 1959, Publications Systematics Association, VNo. 3, P21
Interagency Ecological Program (IEP), 2021, EDI - LTER
Jeon AJ, 2020, BMC GENOMICS, V21, DOI 10.1186/s12864-020-07009-8
Jerison HJ, 2012, BIOLINGUISTICS, V6, P383
JERISON HJ, 1975, CURR ANTHROPOL, V16, P413
Kaas JH, 2019, PROG BRAIN RES, V250, P61, DOI 10.1016/bs.pbr.2019.03.017
Khyeam Sheamin, 2021, Adv Genet (Hoboken), V2, DOI 10.1002/ggn2.10042
Klamut J, 2020, ENTROPY-SWITZ, V22, DOI 10.3390/e22080866
Koonin EV, 2016, PHILOS T R SOC A, V374, DOI 10.1098/rsta.2015.0065
Kriegman S, 2018, SCI REP-UK, V8, DOI 10.1038/s41598-018-31868-7
Laumer CE, 2019, P ROY SOC B-BIOL SCI, V286, DOI 10.1098/rspb.2019.0831
Laumer CE, 2018, INTEGR COMP BIOL, V58, P623, DOI 10.1093/icb/icy075
Li X, 2021, ACCOUNTS CHEM RES, V54, P3734, DOI 10.1021/acs.accounts.1c00463
Li YN, 2021, MOL BIOL EVOL, V38, P4322, DOI 10.1093/molbev/msab170
Lineweaver CH, 2013, COMPLEXITY AND THE ARROW OF TIME, P3
Lineweaver CH, 2021, BIOESSAYS, V43, DOI 10.1002/bies.202000305
Liu YX, 2020, INTEGR COMP BIOL, V60, P991, DOI 10.1093/icb/icaa109
Ma'ayan A, 2017, J R SOC INTERFACE, V14, DOI 10.1098/rsif.2017.0391
Machnik M, 2020, CELLS-BASEL, V9, DOI 10.3390/cells9030653
Markov AV, 2016, BIOL DIRECT, V11, DOI 10.1186/s13062-016-0131-8
Marques DA, 2019, TRENDS ECOL EVOL, V34, P531, DOI 10.1016/j.tree.2019.02.008
McShea DW, 2019, EVOLUTION, V73, P1101, DOI 10.1111/evo.13735
McShea DW, 1996, EVOLUTION, V50, P477, DOI 10.1111/j.1558-5646.1996.tb03861.x
Moroz LL, 2021, FRONT CELL DEV BIOL, V9, DOI 10.3389/fcell.2021.726563
Navarrete A, 2011, NATURE, V480, P91, DOI 10.1038/nature10629
Neves K, 2020, NEUROSCI LETT, V735, DOI 10.1016/j.neulet.2020.135202
Newham E, 2022, BIOESSAYS, V44, DOI 10.1002/bies.202100060
Nguyen PD, 2021, CELL MOL LIFE SCI, V78, P5107, DOI 10.1007/s00018-021-03831-9
Niklas KJ, 2018, J EXP BOT, V69, P1437, DOI 10.1093/jxb/erx493
O'Malley MA, 2016, TRENDS ECOL EVOL, V31, P608, DOI 10.1016/j.tree.2016.04.004
Park WJ, 2020, CURR OPIN PHYSIOL, V16, P50, DOI 10.1016/j.cophys.2020.06.004
Perkins DR, 2022, J PHYSIOL-LONDON, V600, P583, DOI 10.1113/JP282282
Plattner H, 2022, J EUKARYOT MICROBIOL, V69, DOI 10.1111/jeu.12895
Poelmann RE, 2019, DEV DYNAM, V248, P634, DOI 10.1002/dvdy.45
Rebout N, 2021, ROY SOC OPEN SCI, V8, DOI 10.1098/rsos.200895
Richerson PJ, 2021, SCIENCE, V372, P806, DOI 10.1126/science.aba3776
Riede F, 2011, PHILOS T R SOC B, V366, P793, DOI 10.1098/rstb.2010.0266
Robertson FM, 2017, GENOME BIOL, V18, DOI 10.1186/s13059-017-1241-z
Roddy AB, 2021, MOL BIOL EVOL, V38, P3737, DOI 10.1093/molbev/msab142
Romanova DY, 2021, CELL TISSUE RES, V385, P623, DOI 10.1007/s00441-021-03459-y
Ruiz-Trillo I, 2020, DEVELOPMENT, V147, DOI 10.1242/dev.192575

Sames B, 2016, PALAEOGEOGR PALAEOCL, V441, P393, DOI 10.1016/j.palaeo.2015.10.045
Sasakura Y, 2018, WIRES DEV BIOL, V7, DOI 10.1002/wdev.304
Scanes CG, 2018, ANIMALS AND HUMAN SOCIETY, P103, DOI 10.1016/B978-0-12-805247-1.00006-X
Schad E, 2011, GENOME BIOL, V12, DOI 10.1186/gb-2011-12-12-r120
Schulkin J, 2019, TRENDS NEUROSCI, V42, P740, DOI 10.1016/j.tins.2019.07.010
Shabalina SA, 1997, P NATL ACAD SCI USA, V94, P13034, DOI 10.1073/pnas.94.24.13034
Shomar A, 2022, ISCIENCE, V25, DOI 10.1016/j.isci.2022.103924
Singh PP, 2020, NUCLEIC ACIDS RES, V48, pD724, DOI 10.1093/nar/gkz909
Smaers JB, 2021, SCI ADV, V7, DOI 10.1126/sciadv.abe2101
Spaans SK, 2015, EXTREMOPHILES, V19, P741, DOI 10.1007/s00792-015-0750-5
Suter DM, 2020, TRENDS CELL BIOL, V30, P491, DOI 10.1016/j.tcb.2020.03.003
Szklarczyk D, 2021, NUCLEIC ACIDS RES, V49, P10800, DOI 10.1093/nar/gkab835
Taft RJ, 2007, BIOESSAYS, V29, P288, DOI 10.1002/bies.20544
Torday JS, 2018, PROG BIOPHYS MOL BIO, V135, P1, DOI 10.1016/j.pbiomolbio.2017.12.003
Torres-Paz J, 2018, BIOL LETTERS, V14, DOI 10.1098/rsbl.2018.0101
Trigos AS, 2017, P NATL ACAD SCI USA, V114, P6406, DOI 10.1073/pnas.1617743114
Tsuboi M, 2018, NAT ECOL EVOL, V2, P1492, DOI 10.1038/s41559-018-0632-1
Turchin P., 2009, Secular Cycles
Turchin P, 2021, EVOL PSYCHOL-US, V19, DOI 10.1177/14747049211066600
Turchin Peter., 2016, Ultrasociety: how 10,000 years of war made humans the greatest cooperators on Earth
Venkatachalam AB, 2017, MOL GENET GENOMICS, V292, P699, DOI 10.1007/s00438-017-1313-5
Vinogradov AE, 2019, BIOSYSTEMS, V181, P82, DOI 10.1016/j.biosystems.2019.05.004
Vinogradov AE, 2019, GENOMICS, V111, P115, DOI 10.1016/j.ygeno.2018.02.015
Vinogradov AE, 2005, CHROMOSOMA, V113, P362, DOI 10.1007/s00412-004-0323-3
Vinogradov AE, 2007, NUCLEIC ACIDS RES, V35, P6350, DOI 10.1093/nar/gkm723
Vinogradov AE, 2022, INT J MOL SCI, V23, DOI 10.3390/ijms231911486
Vinogradov AE, 2021, INT J MOL SCI, V22, DOI 10.3390/ijms222111640
Vinogradov AE, 2020, FEBS J, V287, P4427, DOI 10.1111/febs.15257
Vinogradov AE, 2009, J MOL EVOL, V68, P192, DOI 10.1007/s00239-008-9185-2
WALSH A., 2016, Frontiers in Sociology, V1, P1, DOI DOI 10.3389/FSOC.2016.00008
Wang SQ, 2014, SCI REP-UK, V4, DOI 10.1038/srep06400
Westneat DF, 2019, TRENDS ECOL EVOL, V34, P555, DOI 10.1016/j.tree.2019.02.010
Williams G. C., 1966, P307
Woronoff G, 2020, P NATL ACAD SCI USA, V117, P10660, DOI 10.1073/pnas.1913767117

   Yeates LC, 2007, J EXP BIOL, V210, P1960, DOI 10.1242/jeb.02767
   Yruela I, 2017, GENOME BIOL EVOL, V9, P1248, DOI 10.1093/gbe/evx073
   Zaharias P, 2020, MOL PHYLOGENET EVOL, V142, DOI
10.1016/j.ympev.2019.106660
   Zdobnov EM, 2021, NUCLEIC ACIDS RES, V49, pD389, DOI
10.1093/nar/gkaa1009
   Zeitlinger J, 2020, CURR OPIN SYST BIOL, V23, P22, DOI
10.1016/j.coisb.2020.08.002
NR 121
TC 5
Z9 5
U1 0
U2 5
PU ELSEVIER SCI LTD
PI OXFORD
PA THE BOULEVARD, LANGFORD LANE, KIDLINGTON, OXFORD OX5 1GB, OXON,
ENGLAND
SN 0303-2647
EI 1872-8324
J9 BIOSYSTEMS
JI Biosystems
PD FEB
PY 2023
VL 224
AR 104844
DI 10.1016/j.biosystems.2023.104844
EA FEB 2023
PG 11
WC Biology; Mathematical & Computational Biology
WE Science Citation Index Expanded (SCI-EXPANDED)
SC Life Sciences & Biomedicine - Other Topics; Mathematical &
Computational
   Biology
GA 8V8BL
UT WOS:000930852300001
PM 36736879
DA 2025-05-07
ER

PT J
AU Nesse, RM
AF Nesse, Randolph M.
TI Evolutionary psychiatry: foundations, progress and challenges
SO WORLD PSYCHIATRY
LA English
DT Article
DE Evolutionary psychiatry; natural selection; vulnerability to diseases;
   evolutionary medicine; depression; anxiety; substance use disorders;
   eating disorders; schizophrenia
ID OBSESSIVE-COMPULSIVE DISORDER; GENERALIZED ANXIETY DISORDER;
TINBERGENS
   4 QUESTIONS; SICKNESS BEHAVIOR; PARTNER CHOICE; KIN SELECTION;
   COMPETITION HYPOTHESIS; BALANCING SELECTION; UNATTAINABLE GOALS;
SOCIAL

COMPETITION
AB Evolutionary biology provides a crucial foundation for medicine and behavioral science that has been missing from psychiatry. Its absence helps to explain slow progress; its advent promises major advances. Instead of offering a new kind of treatment, evolutionary psychiatry provides a scientific foundation useful for all kinds of treatment. It expands the search for causes from mechanistic explanations for disease in some individuals to evolutionary explanations for traits that make all members of a species vulnerable to disease. For instance, capacities for symptoms such as pain, cough, anxiety and low mood are universal because they are useful in certain situations. Failing to recognize the utility of anxiety and low mood is at the root of many problems in psychiatry. Determining if an emotion is normal and if it is useful requires understanding an individual's life situation. Conducting a review of social systems, parallel to the review of systems in the rest of medicine, can help achieve that understanding. Coping with substance abuse is advanced by acknowledging how substances available in modern environments hijack chemically mediated learning mechanisms. Understanding why eating spirals out of control in modern environments is aided by recognizing the motivations for caloric restriction and how it arouses famine protection mechanisms that induce binge eating. Finally, explaining the persistence of alleles that cause serious mental disorders requires evolutionary explanations of why some systems are intrinsically vulnerable to failure. The thrill of finding functions for apparent diseases is evolutionary psychiatry's greatest strength and weakness. Recognizing bad feelings as evolved adaptations corrects psychiatry's pervasive mistake of viewing all symptoms as if they were disease manifestations. However, viewing diseases such as panic disorder, melancholia and schizophrenia as if they are adaptations is an equally serious mistake in evolutionary psychiatry. Progress will come from framing and testing specific hypotheses about why natural selection left us vulnerable to mental disorders. The efforts of many people over many years will be needed before we will know if evolutionary biology can provide a new paradigm for understanding and treating mental disorders.
C1 [Nesse, Randolph M.] Univ Michigan, Dept Psychiat & Psychol, Ann Arbor, MI 48109 USA.
[Nesse, Randolph M.] Arizona State Univ, Ctr Evolut & Med, Tempe, AZ 85281 USA.
C3 University of Michigan System; University of Michigan; Arizona State University; Arizona State University-Tempe
RP Nesse, RM (autor correspondente), Univ Michigan, Dept Psychiat & Psychol, Ann Arbor, MI 48109 USA.; Nesse, RM (autor correspondente), Arizona State Univ, Ctr Evolut & Med, Tempe, AZ 85281 USA.
CR Abed R., 2022, Evolutionary psychiatry: Current perspectives on evolution and mental health, P169
Abed R. T., 2022, Evolutionary psychiatry: Evolutionary perspectives on mental health
Abed RT, 1998, BRIT J MED PSYCHOL, V71, P525, DOI 10.1111/j.2044-8341.1998.tb01007.x
Abed RT, 1998, BEHAV NEUROL, V11, P245
Abed RT., 2021, SAGE HDB EVOLUTIONAR, P24
Abrams M., 2021, NEW CBT CLIN EVOLUTI
Adriaens P.R., 2011, Maladapting Minds: Philosophy, Psychiatry, and Evolutionary Theory

Adriaens PR, 2010, HIST PSYCHIATR, V21, P131, DOI 10.1177/0957154X10370632
Agren J.A., 2021, The Gene's-eye View of Evolution
Ågren JA, 2022, J GENET, V101, DOI 10.1007/s12041-022-01383-w
AINSWORTH M D, 1962, Public Health Pap, V14, P97
Ainsworth M. D., 1978, Patterns of attachment: A psychological study of the strange situation, DOI DOI 10.4324/9780203758045
Akil H, 2010, SCIENCE, V327, P1580, DOI 10.1126/science.1188654
Akiskal HS, 2001, J AFFECT DISORDERS, V62, P17, DOI 10.1016/S0165-0327(00)00347-5
Al-Shawaf L., 2018, Encyclopedia of Evolutionary Psychological Science, P1, DOI [10.1007/978-3-319-16999-6_2158-1, DOI 10.1007/978-3-319-16999-6_2158-1]
Al-Shawaf L, 2016, EMOT REV, V8, P173, DOI 10.1177/1754073914565518
Alcock John., 2013, ANIMAL BEHAV EVOLUTI, V10th
Alcock John., 2001, The Triumph of Sociobiology
Allen NB, 2003, PSYCHOL BULL, V129, P887, DOI 10.1037/0033-2909.129.6.887
Allen NB, 2006, PROG NEURO-PSYCHOPH, V30, P815, DOI 10.1016/j.pnpbp.2006.01.007
AlShawaf L., OXFORD HDB EVOLUTION
AlShawaf L., 2019, AREO MAGAZINE 0820, V20
AlShawaf L., 2017, ENCY PERSONALITY IND, P1, DOI DOI 10.1007/978-3-319-28099-8_516-1
Andrews PW, 2009, PSYCHOL REV, V116, P620, DOI 10.1037/a0016242
Andrews PW., 2010, SA MIND, V20, P56
[Anonymous], 2011, Plasticity, Robustness, Development and Evolution
[Anonymous], 2005, Evolution: An Introduction
[Anonymous], EV PSYCH SPEC INT GR
[Anonymous], 2009, Evolution
Anttila V, 2018, SCIENCE, V360, P1313, DOI 10.1126/science.aap8757
Apicella CL, 2019, CURR BIOL, V29, pR447, DOI 10.1016/j.cub.2019.03.036
ARRINDELL WA, 1993, BEHAV RES THER, V31, P507, DOI 10.1016/0005-7967(93)90133-F
Aubert A, 1999, NEUROSCI BIOBEHAV R, V23, P1029, DOI 10.1016/S0149-7634(99)00034-2
Austin BURT., 2009, Genes in conflict: the biology of selfish genetic elements
Axelrod R., 1997, The complexity of cooperation: Agent-based models of competition and collaboration, V3, DOI DOI 10.1515/9781400822300
Axelrod R., 1984, The Evolution of Cooperation
Badcock C., 2019, DIAMETRIC MIND INSIG
Bailey NW, 2019, EVOLUTION, V73, P1549, DOI 10.1111/evo.13791
Barash D.P., 1977, Sociobiology and Behavior
Barclay P, 2007, P ROY SOC B-BIOL SCI, V274, P749, DOI 10.1098/rspb.2006.0209
Barkow J.H., 1992, ADAPTED MIND
Barlow DH., 2004, Anxiety and its disorders: The Nature and Treatment of Anxiety and Panic
Barr PB, 2020, TRANSL PSYCHIAT, V10, DOI 10.1038/s41398-020-00865-8
Baselmans BML, 2021, BIOL PSYCHIAT, V89, P11, DOI 10.1016/j.biopsych.2020.05.034

Bateson M, 2011, CAN J PSYCHIAT, V56, P707, DOI 10.1177/070674371105601202
Bateson P, 2013, TRENDS ECOL EVOL, V28, P712, DOI 10.1016/j.tree.2013.09.013
Beck AT, 2009, DEPRESSION: CAUSES AND TREATMENT, SECOND EDITION, P1
Beck AT., 1996, DEPRESSION EVOLUTION
Belsky J., 2005, ATTACHMENT BONDING N
Bender M., 2021, SCI AM, V325, P31
Bergman TJ, 2022, EVOL ANTHROPOL, V31, P12, DOI 10.1002/evan.21931
Bergstrom CarlT., 2012, Evolution
Bernhardt WM, 2006, KIDNEY INT, V69, P114, DOI 10.1038/sj.ki.5000062
Bolnick DI., 2017, MED SCI EDUC, V27, P137
Bondolfi G, 2015, EMOT REV, V7, P216, DOI 10.1177/1754073915575405
Boomsma K., 2022, DOMAINS MAJOR TRANSI
Border R., 2022, BIORXIV
BOWLBY J, 1956, BRIT J MED PSYCHOL, V29, P211, DOI 10.1111/j.2044-8341.1956.tb00915.x
BOWLBY J, 1953, J MENT SCI, V99, P265, DOI 10.1192/bjp.99.415.265
Bowlby J., 1982, ATTACHMENT LOSS VOL, V1
Bowles S, 2004, THEOR POPUL BIOL, V65, P17, DOI 10.1016/j.tpb.2003.07.001
BRETHERTON I, 1992, DEV PSYCHOL, V28, P759, DOI 10.1037/0012-1649.28.5.759
Brickman P., 1987, COMMITMENT CONFLICT
Brinkmann K., 2015, HDB BIOBEHAVIORAL AP, P333
Brosnan SF, 2017, EVOL PSYCHOL-SER, P93, DOI 10.1007/978-3-319-60576-0_4
BROWN GW, 1995, PSYCHOL MED, V25, P7, DOI 10.1017/S003329170002804X
Brown GW, 2002, J HEALTH SOC BEHAV, V43, P255, DOI 10.2307/3090203
Brüne M, 2006, PERSPECT BIOL MED, V49, P317, DOI 10.1353/pbm.2006.0037
Brüne M, 2012, WORLD PSYCHIATRY, V11, P55
Brüne M, 2002, PSYCHIATRY, V65, P48, DOI 10.1521/psyc.65.1.48.19759
Brune M., 2003, SOCIAL BRAIN EVOLUTI
Brune M., 2016, Textbook of evolutionary psychiatry and psychosomatic medicine, V2nd
Brune M., 2019, The Oxford handbook of evolutionary medicine
Brune M, 2008, Textbook of Evolutionary Psychiatry: The Origins of Psychopathology
Brüne M, 2022, J NERV MENT DIS, V210, P235, DOI 10.1097/NMD.0000000000001493
Brune Martin, 2014, BMC Psychiatry, V14, P1695, DOI 10.1186/s12888-014-0364-y
Buklijas T, 2013, CAMBRIDGE ENCYCLOPEDIA OF DARWIN AND EVOLUTIONARY THOUGHT, P505
Bulik CM, 2022, NAT NEUROSCI, V25, P543, DOI 10.1038/s41593-022-01071-z
Bulik CM, 2021, TRENDS ENDOCRIN MET, V32, P752, DOI 10.1016/j.tem.2021.07.010
Bulik-Sullivan B, 2015, NAT GENET, V47, P1236, DOI 10.1038/ng.3406
Buller D.J., 2005, Adapting minds: Evolutionary psychology and the persistent quest for human nature
Bumpus H.C., 1899, BIOL LECT, V6, P209
Buss D.M., 2005, HDB EVOLUTIONARY PSY
Buss D.M., 2003, EVOLUTION DESIRE STR

Buss D.M., 2015, The handbook of evolutionary psychology, Volume, V1
Buss David., 2015, Evolutionary psychology: The new science of the mind
Buss DM, 2020, EVOL BEHAV SCI, V14, P316, DOI 10.1037/ebs0000210
Buss DM, 2009, AM PSYCHOL, V64, P140, DOI 10.1037/a0013207
BUSS DM, 1993, PSYCHOL REV, V100, P204, DOI 10.1037/0033-295X.100.2.204
BUSS DM, 1988, J PERS SOC PSYCHOL, V54, P616, DOI 10.1037/0022-3514.54.4.616
Byars SG, 2014, P ROY SOC B-BIOL SCI, V281, DOI 10.1098/rspb.2014.0604
Cannon WB, 1914, AM J PHYSIOL, V33, P356, DOI 10.1152/ajplegacy.1914.33.2.356
Carver C.S., 2013, GOALS EMOTION HDB CO, P176, DOI DOI 10.1017/S1355617711000506
Carver CS, 2015, EMOT REV, V7, P301, DOI 10.1177/1754073915590616
CARVER CS, 1990, PSYCHOL REV, V97, P19, DOI 10.1037/0033-295X.97.1.19
CHAMBLESS DL, 1989, COGNITIVE THER RES, V13, P9, DOI 10.1007/BF01178486
Charlesworth D, 2006, PLOS GENET, V2, P379, DOI 10.1371/journal.pgen.0020064
Charlesworth D, 2017, P ROY SOC B-BIOL SCI, V284, DOI 10.1098/rspb.2016.2864
Chisholm JS, 2003, MACQUARIE MG COG SCI, P125
Chitty D., 1996, Do Lemmings Commit Suicide: Beautiful Hypotheses and Ugly Facts
Cohen MP., 2022, WILLIAMS EVOLUTIONAR
Conley BA, 2019, BIOL PHILOS, V35, DOI 10.1007/s10539-019-9731-x
COOK LM, 1986, SCIENCE, V231, P611, DOI 10.1126/science.231.4738.611
COSMIDES LM, 1981, J THEOR BIOL, V89, P83, DOI 10.1016/0022-5193(81)90181-8
Crawford C., 1998, Handbook of evolutionary psychology: Ideas, issues, and applications
Crespi B, 2019, EVOL MED PUBLIC HLTH, P121, DOI 10.1093/emph/eoz021
Crespi BJ, 2020, CLIN PSYCHOL REV, V78, DOI 10.1016/j.cpr.2020.101857
Crespi Bernard J., 2015, Evolution Medicine and Public Health, P216, DOI 10.1093/emph/eov021
Crespi BJ, 2011, EPIGENETICS HUM HLTH, P143, DOI 10.1007/978-3-642-17426-1_8
Cronin H., 1991, ANT PEACOCK ALTRUISM
Cross J.G., 1980, SOCIAL TRAPS
CROW TJ, 1995, BRIT J PSYCHIAT, V167, P12, DOI 10.1192/bjp.167.1.12
Crow TJ, 1997, SCHIZOPHR RES, V28, P127, DOI 10.1016/S0920-9964(97)00110-2
Cuijpers P, 2007, CLIN PSYCHOL REV, V27, P318, DOI 10.1016/j.cpr.2006.11.001
Daly M., 1983, Sex, evolution, and behavior, V2nd
Dantzer R, 2001, BRAIN BEHAV IMMUN, V15, P7, DOI 10.1006/brbi.2000.0613
Darwin C., 1872, P374
Davies N. B., 2012, An introduction to behavioral ecology, DOI DOI 10.4135/9781446211922.N13
Dawkins R., 2016, The selfish gene, V40th
De Block A, 2005, PHILOS PSYCHIATR PSY, V12, P315, DOI 10.1353/ppp.2006.0023

de Waal FBM, 2022, SCIENCE, V375, P1351, DOI 10.1126/science.abo2378
Deci EL, 2000, PSYCHOL INQ, V11, P227, DOI 10.1207/S15327965PLI1104_01
Del Giudice M., OXFORD HDB EVOLUTION
Del Giudice M., 2018, EVOLUTIONARY PSYCHOP, V1
Del Giudice M, 2023, NEUROSCI BIOBEHAV R, V144, DOI 10.1016/j.neubiorev.2022.104967
Del Giudice M, 2009, BEHAV BRAIN SCI, V32, P1, DOI 10.1017/S0140525X09000016
Dominy NJ, 2015, P NATL ACAD SCI USA, V112, P308, DOI 10.1073/pnas.1421566112
Dudley R, 2004, INTEGR COMP BIOL, V44, P315, DOI 10.1093/icb/44.4.315
Dugatkin L.A., 2020, Principles of animal behavior
Dugatkin LeeAnn., 2006, ALTRUISM EQUATION 7
Dunbar R., 2019, Alcohol and Humans: A Long and Social Affair, P163
Dunbar RIM, 2017, ADAPT HUM BEHAV PHYS, V3, P118, DOI 10.1007/s40750-016-0058-4
Dunbar R.I.M., 2022, Evolutionary Psychiatry, P202
Dunbar RIM., 2020, EVOLUTION WHAT EVERY, V2nd ed.
Durkee PK, 2022, PSYCHOL SCI, V33, P285, DOI 10.1177/09567976211031571
EBERHARD MJW, 1975, Q REV BIOL, V50, P1, DOI 10.1086/408298
Ellsworth PC, 2013, EMOT REV, V5, P125, DOI 10.1177/1754073912463617
Emmons R.A., 1996, The psychology of action, P313
Engel G L, 1972, Ciba Found Symp, V8, P57
Enthoven CA, 2019, EUR J EPIDEMIOL, V34, P777, DOI 10.1007/s10654-019-00512-7
Fabrega H., 2002, ORIGINS PSYCHOPATHOL
Fairbanks LA., 1977, ETHOLOGICAL PSYCHIAT
Faucher L., 2021, DEFINING MENTAL DISO
Fehr E, 1998, EUR ECON REV, V42, P845, DOI 10.1016/S0014-2921(97)00131-1
Feldman Barrett L., 2014, The psychological construction of emotion
Ferster C.B., 1974, PSYCHOL DEPRESSION C, P29
Fineberg N.A., 2021, The Neurobiology and Treatment of OCD: Accelerating Progress
FINLAYJONES R, 1981, PSYCHOL MED, V11, P803, DOI 10.1017/S0033291700041301
Frances A., 2013, Psychotherapy in Australia, V19, P14
Frank SA, 2013, J EVOLUTION BIOL, V26, P1151, DOI 10.1111/jeb.12131
Frank SA, 2012, J EVOLUTION BIOL, V25, P227, DOI 10.1111/j.1420-9101.2011.02431.x
Frank S.A., 2018, Control Theory Tutorial
Frank Steven A., 2006, P350
Frankenhuis WE, 2020, CURR OPIN BEHAV SCI, V36, P157, DOI 10.1016/j.cobeha.2020.10.012
Gadagkar R., 2022, WIRE SCI OCT
Gangestad S.W., 2007, The evolution of mind: Fundamental questions and controversies
Garland T, 2022, PHYSIOL BIOCHEM ZOOL, V95, P82, DOI 10.1086/717897
Gatward N, 2007, EUR EAT DISORD REV, V15, P1, DOI 10.1002/erv.718
Gaulin StephenJ. C., 2001, Psychology: An Evolutionary Approach
Gilbert P, 2001, AUST NZ J PSYCHIAT, V35, P17, DOI 10.1046/j.1440-1614.2001.00856.x
Gilbert P, 1998, PSYCHOL MED, V28, P585, DOI 10.1017/S0033291798006710

Gilbert P., 2000, Genes on the couch: Explorations in evolutionary psychotherapy
Gilbert P., 1992, DEPRESSION EVOLUTION
Gilbert P, 2020, FRONT PSYCHOL, V11, DOI 10.3389/fpsyg.2020.586161
Gilbert P, 2019, PSYCHOL PSYCHOTHER-T, V92, P164, DOI 10.1111/papt.12226
Gilbert P, 2015, EMOT REV, V7, P230, DOI 10.1177/1754073915576552
Gilbert P, 2014, WILEY BLACKWELL HANDBOOK OF SOCIAL ANXIETY DISORDER, P24
Giosan C., 2020, COGNITIVE EVOLUTIONA, P23
Giraud A, 2001, CURR OPIN MICROBIOL, V4, P582, DOI 10.1016/S1369-5274(00)00254-X
Glantz Kalman., 1989, Exiles from Eden: Psychotherapy from an Evolutionary Perspective
Gluckman P., 2006, MISMATCH WHY OUR WOR
Gluckman PeterD., 2009, Principles of evolutionary medicine
GOODMAN WK, 1989, ARCH GEN PSYCHIAT, V46, P1006
Gratten J, 2014, NAT NEUROSCI, V17, P782, DOI 10.1038/nn.3708
Greaves M, 2014, P ROY SOC B-BIOL SCI, V281, DOI 10.1098/rspb.2013.2955
Greenberg DM, 2018, P NATL ACAD SCI USA, V115, P12152, DOI 10.1073/pnas.1811032115
Griffin AS, 2004, NATURE, V430, P1024, DOI 10.1038/nature02744
Griffin AS, 2002, TRENDS ECOL EVOL, V17, P15, DOI 10.1016/S0169-5347(01)02355-2
Griffiths P.E., 1997, WHAT EMOTIONS REALLY
Grunspan DZ, 2019, EVOL MED PUBLIC HLTH, P82, DOI 10.1093/emph/eoz012
Guisinger S, 2003, PSYCHOL REV, V110, P745, DOI 10.1037/0033-295X.110.4.745
Gurven MD, 2020, EVOL HUM BEHAV, V41, P330, DOI 10.1016/j.evolhumbehav.2020.04.001
Gut E., 1989, PRODUCTIVE UNPRODUCT
Haase CM, 2013, DEV PSYCHOL, V49, P964, DOI 10.1037/a0029231
Hagen E. H., 2019, Handbook of Cognitive Archaeology, P471, DOI [10.4324/9780429488818-25, DOI 10.4324/9780429488818-25]
Hagen EH, 2013, FRONT PSYCHIATRY, V4, DOI 10.3389/fpsyt.2013.00142
Hagen EH, 2011, CAN J PSYCHIAT, V56, P716, DOI 10.1177/070674371105601203
Hagen EH, 2002, EVOL HUM BEHAV, V23, P323, DOI 10.1016/S1090-5138(01)00102-7
Hagen R, 2017, FRONT PSYCHOL, V8, DOI 10.3389/fpsyg.2017.00031
Haig D, 2022, BIOESSAYS, V44, DOI 10.1002/bies.202200023
Hamburg D., 1975, EMOTIONS THEIR PARAM, P235
HAMILTON WD, 1964, J THEOR BIOL, V7, P1, DOI [10.1016/0022-5193(64)90038-4, 10.1016/0022-5193(64)90039-6]
Hammerstein P., 2003, Genetic and Cultural Evolution of Cooperation
Hardy CL, 2006, PERS SOC PSYCHOL B, V32, P1402, DOI 10.1177/0146167206291006
HART BL, 1988, NEUROSCI BIOBEHAV R, V12, P123, DOI 10.1016/S0149-7634(88)80004-6
HARVEY PH, 1975, GENETICA, V45, P211, DOI 10.1007/BF01517197
Haselton M.G., 2006, HEARTS MINDS AFFECTI, P21
Haselton MG, 2009, SOC COGNITION, V27, P733, DOI 10.1521/soco.2009.27.5.733

Haselton MG, 2000, J PERS SOC PSYCHOL, V78, P81, DOI 10.1037/0022-3514.78.1.81
Hatoum AS, 2022, NEUROPSYCHOPHARMACOL, V47, P1739, DOI 10.1038/s41386-021-01209-w
Heckhausen J, 2022, MOTIV EMOTION, V46, P884, DOI 10.1007/s11031-022-09981-y
Hedrick PW, 2007, CURR BIOL, V17, pR230, DOI 10.1016/j.cub.2007.01.012
Hedrick PW, 2012, TRENDS ECOL EVOL, V27, P698, DOI 10.1016/j.tree.2012.08.012
Henrich J, 2021, ANNU REV PSYCHOL, V72, P207, DOI 10.1146/annurev-psych-081920-042106
Hidaka BH, 2015, BMC MED EDUC, V15, DOI 10.1186/s12909-015-0322-5
Hidaka BH, 2012, J AFFECT DISORDERS, V140, P205, DOI 10.1016/j.jad.2011.12.036
Higgins ET, 1997, J PERS SOC PSYCHOL, V72, P515, DOI 10.1037/0022-3514.72.3.515
Higgins ET, 1998, ADV EXP SOC PSYCHOL, V30, P1, DOI 10.1016/S0065-2601(08)60381-0
Hilbert A, 2014, PSYCHIAT RES, V220, P500, DOI 10.1016/j.psychres.2014.05.054
Hoff P, 2008, EUR ARCH PSY CLIN N, V258, P12, DOI 10.1007/s00406-008-2002-5
HOGSTEDT G, 1980, SCIENCE, V210, P1148, DOI 10.1126/science.210.4474.1148
Holland J., 2010, EE TIMES 0816, V16
Hollon SD, 2021, BEHAV RES THER, V143, DOI 10.1016/j.brat.2021.103849
Horwitz Allan V., 2007, The Loss of Sadness: How Psychiatry Transformed Normal Sorrow into Depressive Disorder
Hunt AD., 2022, EVOL MED PUBLIC HLTH
Hunt AD, 2022, EVOL HUM SCI, V4, DOI 10.1017/ehs.2022.23
Hurst LD, 2022, PLOS BIOL, V20, DOI 10.1371/journal.pbio.3001671
Hyman SE., 2022, OBSESSIVE COMPULSIVE
Insel TR, 2010, JAMA-J AM MED ASSOC, V303, P1970, DOI 10.1001/jama.2010.555
Jablonski NG, 2010, P NATL ACAD SCI USA, V107, P8962, DOI 10.1073/pnas.0914628107
Jaeger CB, 2020, EVOL HUM BEHAV, V41, P253, DOI 10.1016/j.evolhumbehav.2020.04.002
Jamie GA, 2020, TRENDS ECOL EVOL, V35, P795, DOI 10.1016/j.tree.2020.04.007
Jesus S, 2022, EUR PSYCHIAT, V65, pS735, DOI 10.1192/j.eurpsy.2022.1900
Johnson RW, 2002, VET IMMUNOL IMMUNOP, V87, P443, DOI 10.1016/S0165-2427(02)00069-7
Keller MC, 2007, AM J PSYCHIAT, V164, P1521, DOI 10.1176/appi.ajp.2007.06091564
Keller MC, 2006, J PERS SOC PSYCHOL, V91, P316, DOI 10.1037/0022-3514.91.2.316
Keller MC, 2006, BEHAV BRAIN SCI, V29, P385, DOI 10.1017/S0140525X06009095
Keltner D., 2006, Evolution and Social Psychology, P115
Kendler KS, 2009, PSYCHOL MED, V39, P1935, DOI 10.1017/S0033291709005753
KENDLER KS, 1992, ARCH GEN PSYCHIAT, V49, P716

Kennair LEO, 2017, EVOL PSYCHOL-SER, P73, DOI 10.1007/978-3-319-60576-0_3
Kennair LEO, 2003, CURR OPIN PSYCHIATR, V16, P691, DOI 10.1097/00001504-200311000-00015
Kennair LEO., 2018, ENCY EVOLUTIONARY PS, P1
Kennair LEO., 2007, J EVOL PSYCHOL, V5, P105
Kessler RC, 2022, JAMA NETW OPEN, V5, DOI 10.1001/jamanetworkopen.2022.17223
Kessler RC, 2013, ANNU REV PUBL HEALTH, V34, P119, DOI 10.1146/annurev-publhealth-031912-114409
Ketelaar T, 2015, EVOL PSYCHOL-SER, P51, DOI 10.1007/978-3-319-12697-5_5
KIRKWOOD TBL, 1991, PHILOS T R SOC B, V332, P15, DOI 10.1098/rstb.1991.0028
Kitano H, 2007, MOL SYST BIOL, V3, DOI 10.1038/msb4100179
KLERMAN GL, 1989, JAMA-J AM MED ASSOC, V261, P2229, DOI 10.1001/jama.261.15.2229
Klingenberg CP, 2019, FRONT ECOL EVOL, V7, DOI 10.3389/fevo.2019.00056
KLINGER E, 1975, PSYCHOL REV, V82, P1, DOI 10.1037/h0076171
Koch EM, 2021, FRONT GENET, V12, DOI 10.3389/fgene.2021.763363
Kohrt BA, 2020, CLIN PSYCHOL REV, V82, DOI 10.1016/j.cpr.2020.101920
Kokko H, 2006, ANNU REV ECOL EVOL S, V37, P43, DOI 10.1146/annurev.ecolsys.37.091305.110259
Kondrashov AS., 2017, Crumbling Genome: The Impact of Deleterious Mutations on Humans, DOI 10.1002/9781118952146
Konner M, 2021, HUM NATURE-INT BIOS, V32, P748, DOI 10.1007/s12110-021-09414-8
Konner Melvin., 1983, The Tangled Wing: Biological Constraints on the Human Spirit
Konner MJ., 1991, CHILDHOOD MULTICULTU, V1st ed.
Koppe K, 2017, J BEHAV THER EXP PSY, V54, P278, DOI 10.1016/j.jbtep.2016.10.003
Krebs D., 1987, Sociobiology and psychology: Ideas, issues, and applications, P81
Krebs J.R., 1997, Behavioural Ecology: An Evolutionary Approach, V4th
Kross E, 2021, TRENDS COGN SCI, V25, P55, DOI 10.1016/j.tics.2020.10.005
Kruger DJ, 2006, HUM NATURE-INT BIOS, V17, P74, DOI 10.1007/s12110-006-1021-z
Lalumière ML, 2001, EVOL HUM BEHAV, V22, P75, DOI 10.1016/S1090-5138(00)00064-7
Lancaster JB., 1992, Primatology Today, V1, P21
LANE LW, 1988, COMPR PSYCHIAT, V29, P598, DOI 10.1016/0010-440X(88)90080-6
Leary MR, 2000, ADV EXP SOC PSYCHOL, V32, P1, DOI 10.1016/S0065-2601(00)80003-9
Lehmann L, 2007, P NATL ACAD SCI USA, V104, P6736, DOI 10.1073/pnas.0700662104
Leonard HL, 2001, INT J NEUROPSYCHOPH, V4, P191, DOI 10.1017/S1461145701002371
Lewis AJ, 1934, J MENT SCI, V80, P1
Lewis DMG, 2020, CAMB HANDB PSYCHOL, P223
Lewis DMG, 2017, AM PSYCHOL, V72, P353, DOI 10.1037/a0040409

Little B. R., 2017, Personal Project Pursuit: Goals, Action, and Human Flourishing
Liu CX, 2019, FRONT GENET, V10, DOI 10.3389/fgene.2019.00389
Lopez-Leon S, 2021, NEUROSCI BIOBEHAV R, V124, P358, DOI 10.1016/j.neubiorev.2021.01.019
LOW BS, 1989, ETHOL SOCIOBIOL, V10, P457, DOI 10.1016/0162-3095(82)90041-3
LOW BS, 1989, J COMP PSYCHOL, V103, P311, DOI 10.1037/0735-7036.103.4.311
Lukaszewski AW, 2020, EUR J PERSONALITY, V34, P1151, DOI 10.1002/per.2292
Lynch M, 2016, NAT REV GENET, V17, P704, DOI 10.1038/nrg.2016.104
MACLEAN PD, 1985, PSYCHOL MED, V15, P219, DOI 10.1017/S0033291700023485
Maj M, 2020, WORLD PSYCHIATRY, V19, P269, DOI 10.1002/wps.20771
Maner JK, 2010, SOC ISS POLICY REV, V4, P111, DOI 10.1111/j.1751-2409.2010.01019.x
Mankf JE, 2023, NAT REV GENET, V24, P44, DOI 10.1038/s41576-022-00524-2
Mansell W, 2005, PSYCHOL PSYCHOTHER-T, V78, P141, DOI 10.1348/147608304X21400
Marks I. M., 1987, Fears, Phobias, and Rituals: Panic, Anxiety, and Their Disorders
MARKS IM, 1994, ETHOL SOCIOBIOL, V15, P247, DOI 10.1016/0162-3095(94)90002-7
Martin L.L., 1996, Striving and Feeling: Interactions Among Goals, Affect, and Self-regulation
Masel J, 2009, TRENDS GENET, V25, P395, DOI 10.1016/j.tig.2009.07.005
MAYR E, 1993, BIOL PHILOS, V8, P93, DOI 10.1007/BF00868508
MAYR E, 1961, SCIENCE, V134, P1501, DOI 10.1126/science.134.3489.1501
Mayr E., 1982, of the evolution of scientific thought and medical
McDade TW, 2016, EVOL MED PUBLIC HLTH, P1, DOI 10.1093/emph/eov033
McGrath J, 2008, EPIDEMIOL REV, V30, P67, DOI 10.1093/epirev/mxn001
McGrath JJ, 2006, BEHAV BRAIN SCI, V29, P417, DOI 10.1017/S0140525X06349096
McGuire M., 1998, DARWINIAN PSYCHIAT
MCGUIRE MT, 1979, PERSPECT BIOL MED, V23, P50
McGuire MT., 1976, MCLEAN HOSP J, V1, P21
McGuire MT., 1999, MALADAPTED MIND CLAS, P255
McNally RJ, 1999, LEA SER PER CLIN PSY, P3
MEALEY L, 1995, BEHAV BRAIN SCI, V18, P523, DOI 10.1017/S0140525X00039595
Meaney MJ, 2007, TRENDS MOL MED, V13, P269, DOI 10.1016/j.molmed.2007.05.003
Mineka S, 2002, BEHAV RES THER, V40, P173, DOI 10.1016/S0005-7967(01)00050-X
MINEKA S, 1980, ANIM LEARN BEHAV, V8, P653, DOI 10.3758/BF03197783
Molendijk ML, 2019, BEHAV BRAIN RES, V364, P1, DOI 10.1016/j.bbr.2019.02.005
Monroe SM, 2005, PSYCHOL REV, V112, P417, DOI 10.1037/0033-295X.112.2.417
Morgan TJH, 2016, CURR ANTHROPOL, V57, P370, DOI 10.1086/686531
Müller MJ, 2015, AM J CLIN NUTR, V102, P807, DOI 10.3945/ajcn.115.109173

Murphy JM, 2000, ARCH GEN PSYCHIAT, V57, P209, DOI 10.1001/archpsyc.57.3.209
Muscatell KA, 2009, J NERV MENT DIS, V197, P154, DOI 10.1097/NMD.0b013e318199f77b
MYERSON M, 1991, MED SCI SPORT EXER, V23, P15, DOI 10.1249/00005768-199101000-00004
Natterson-Horowitz B, 2020, INTEGRATING EVOLUTIONARY BIOLOGY INTO MEDICAL EDUCATION: FOR MATERNAL AND CHILD HEALTHCARE STUDENTS, CLINICIANS, AND SCIENTISTS, P187, DOI 10.1093/oso/9780198814153.003.0011
NattersonHorowitz B, 2019, Wildhood: the epic journey from adolescence to adulthood in humans and other animals
Nesse R M, 1990, Hum Nat, V1, P261, DOI 10.1007/BF02733986
Nesse R.M., 2009, UNDERSTANDING DEPRES, P17, DOI DOI 10.1093/MED/9780199533077.003.0003
Nesse R.M., 2011, Maladapting minds: Philosophy, psychiatry, and evolutionary theory, P167
Nesse R.M., 1994, WHY WE GET SICK NEW
Nesse Randolph M., 2007, Biology Theory, V2, P143, DOI 10.1162/biot.2007.2.2.143
Nesse RM, 2019, PHILOS T R SOC B, V374, DOI 10.1098/rstb.2019.0288
Nesse RM, 2019, DIALOGUES CLIN NEURO, V21, P167, DOI 10.31887/DCNS.2019.21.2/rmnesse
Nesse RM, 2017, BEHAV BRAIN SCI, V40, DOI 10.1017/S0140525X16001503
Nesse RM, 2013, TRENDS ECOL EVOL, V28, P681, DOI 10.1016/j.tree.2013.10.008
Nesse RM, 2012, BMC MED, V10, DOI 10.1186/1741-7015-10-5
Nesse RM, 2011, EVOL APPL, V4, P264, DOI 10.1111/j.1752-4571.2010.00181.x
Nesse Randolph M, 2002, World Psychiatry, V1, P7
Nesse RM, 2010, P NATL ACAD SCI USA, V107, P1800, DOI 10.1073/pnas.0906224106
Nesse RM, 2009, AM PSYCHOL, V64, P129, DOI 10.1037/a0013503
NESSE RM, 1990, J AM ACAD PSYCHOAN, V18, P260, DOI 10.1521/jaap.1.1990.18.2.260
Nesse RM, 2004, BEHAV BRAIN SCI, V27, P862, DOI 10.1017/S0140525X04300191
Nesse RM, 2005, Q REV BIOL, V80, P62, DOI 10.1086/431026
Nesse RM, 2005, EVOL HUM BEHAV, V26, P88, DOI 10.1016/j.evolhumbehav.2004.08.002
Nesse RM, 2000, ARCH GEN PSYCHIAT, V57, P14, DOI 10.1001/archpsyc.57.1.14
Nesse RM, 1997, SCIENCE, V278, P63, DOI 10.1126/science.278.5335.63
NESSE RM, 1987, ETHOL SOCIOBIOL, V8, pS73
Nesse RM, 1999, NEUROSCI BIOBEHAV R, V23, P895, DOI 10.1016/S0149-7634(99)00023-8
NESSE RM, 1984, COMPR PSYCHIAT, V25, P575, DOI 10.1016/0010-440X(84)90038-5
NESSE RM, 1994, ETHOL SOCIOBIOL, V15, P339, DOI 10.1016/0162-3095(94)90007-8
Nesse RM., 2008, RICHARD DAWKINS SCI, P203
Nesse RM., 2009, OXFORD COMPANION EMO, P158
Nesse RM., TACIT CREATIONISM EM
Nesse RM, 2022, KAPLAN SADOCKS COMPR, V10th ed
Nesse RM., 2019, PSYCHOL TODAY 0304, V4

Nesse RM., TASMANS PSYCHIAT
Nesse RM., 2017, COMPR TEXTB PSYCHIAT, V10th, P769
Nesse RM., 2022, GENETIC PROGRAMMING, P1
Nettle D, 2004, J AFFECT DISORDERS, V81, P91, DOI 10.1016/j.jad.2003.08.009
Nettle D, 2017, BEHAV BRAIN SCI, V40, DOI [10.1017/S0140525X16002041, 10.1017/S0140525X16000947]
Nettle D, 2013, P ROY SOC B-BIOL SCI, V280, DOI 10.1098/rspb.2013.1343
Nijhaut HF, 2019, WIRES SYST BIOL MED, V11, DOI 10.1002/wsbm.1440
NOE R, 1994, BEHAV ECOL SOCIOBIOL, V35, P1
Nunes A., 2022, PSYARXIV, V15
Ohman A., 1985, THEORECTICAL ISSUES, P123
Panksepp J, 2006, PROG NEURO-PSYCHOPH, V30, P774, DOI 10.1016/j.pnpbp.2006.01.004
Paradis James G., 1989, Evolution Ethics: T.H. Huxley's Evolution and Ethics with New Essays on its Victorian and Sociobiological Context
Pennisi E, 2012, SCIENCE, V336, P974, DOI 10.1126/science.336.6084.974
Pepper JW., 1999, DYNAMICS HUMAN PRIMA, P2
Perlman R, 2020, INTEGRATING EVOLUTIONARY BIOLOGY INTO MEDICAL EDUCATION: FOR MATERNAL AND CHILD HEALTHCARE STUDENTS, CLINICIANS, AND SCIENTISTS, P119, DOI 10.1093/oso/9780198814153.003.0007
Perlman RL, 2013, Evolution and Medicine, P1
Perlman Robert L, 2005, Med Health Care Philos, V8, P343, DOI 10.1007/s11019-005-2655-z
Perugi G, 2015, J AFFECT DISORDERS, V183, P119, DOI 10.1016/j.jad.2015.05.004
Petit-Demouliere B, 2005, PSYCHOPHARMACOLOGY, V177, P245, DOI 10.1007/s00213-004-2048-7
Petrie M, 2021, FRONT ECOL EVOL, V9, DOI 10.3389/fevo.2021.786868
Pfaff D, 2019, P NATL ACAD SCI USA, V116, P9704, DOI 10.1073/pnas.1903589116
Pfennig D. W., 2021, Phenotypic Plasticity & Evolution: Causes, Consequences, Controversies Internet, DOI 10.1201/9780429343001
Piel FB, 2010, NAT COMMUN, V1, DOI 10.1038/ncomms1104
Pinker S, 2010, P NATL ACAD SCI USA, V107, P8993, DOI 10.1073/pnas.0914630107
Plutchik R., 1970, FEELINGS EMOTIONS, P1, DOI DOI 10.1016/B978-0-12-063550-4.50007-3
Post RM, 1998, BIOL PSYCHIAT, V44, P193, DOI 10.1016/S0006-3223(98)00144-9
Power RA, 2015, NAT NEUROSCI, V18, P953, DOI 10.1038/nn.4040
PRICE J, 1994, BRIT J PSYCHIAT, V164, P309, DOI 10.1192/bjp.164.3.309
PRICE J, 1967, LANCET, V2, P243
Price J.S., 2013, New insights into anxiety disorders, P3, DOI DOI 10.5772/52902
Price JS., 1987, ETHOL SOCIOBIOL, V8
Queller DC, 2006, J EVOLUTION BIOL, V19, P1410, DOI 10.1111/j.1420-9101.2006.01167.x
Queller DC, 2002, CURR BIOL, V12, pR832, DOI 10.1016/S0960-9822(02)01344-1
Rantala MJ, 2018, BRAIN BEHAV IMMUN, V69, P603, DOI 10.1016/j.bbi.2017.10.012
Ridley M., 1996, The origins of virtue: Human instincts and the evolution of cooperation

Ripke S, 2014, NATURE, V511, P421, DOI 10.1038/nature13595
ROBINSON TE, 1993, BRAIN RES REV, V18, P247, DOI 10.1016/0165-0173(93)90013-P
Roff DA, 2007, J EVOLUTION BIOL, V20, P433, DOI 10.1111/j.1420-9101.2006.01255.x
Rook GAW, 2012, CLIN REV ALLERG IMMU, V42, P5, DOI 10.1007/s12016-011-8285-8
Rose H., 2000, Alas, poor Darwin: Arguments against evolutionary psychology
Rosenberg SM, 2012, BIOESSAYS, V34, P885, DOI 10.1002/bies.201200050
Rottenberg J., 2014, The depths: The evolutionary origins of the depression epidemic
Roulette CJ, 2014, EVOL HUM BEHAV, V35, P397, DOI 10.1016/j.evolhumbehav.2014.05.005
Ruan YS, 2020, MOL BIOL EVOL, V37, P1007, DOI 10.1093/molbev/msz283
Ruse M., 2023, UNDERSTANDING NATURA, V1st ed.
Rust NC, 2023, TRENDS NEUROSCI, V46, P3, DOI 10.1016/j.tins.2022.10.011
Santomauro DF, 2021, LANCET PSYCHIAT, V8, P320, DOI 10.1016/S2215-0366(21)00040-7
Scherer K.R., 2001, Appraisal Processes in Emotion: Theory, Methods, Research, DOI [10.1093/oso/9780195130072.003.0005, DOI 10.1093/OSO/9780195130072.003.0005]
Schulkin J., 2020, INTEGRATING EVOLUTIO, V1st ed.
Schulkin J, 2019, TRENDS NEUROSCI, V42, P740, DOI 10.1016/j.tins.2019.07.010
Segerstrale U., 2000, Defenders of the truth: The battle for science in the sociobiology debate and beyond
Shackelford TK, 1997, POLIT LIFE SCI, V16, P341, DOI 10.1017/S073093840002503X
Shackelford TK., 2017, EVOLUTION PSYCHOPATH
Shorter Edward., 1998, A History of Psychiatry: From the Era of the Asylum to the Age of Prozac
Shuster Stephen M., 2010, P434
Sieff DF, 2022, EVOLUTIONARY PSYCHIA, P244
Simons AM, 1997, OIKOS, V80, P401, DOI 10.2307/3546608
Singh T, 2022, NATURE, V604, P509, DOI 10.1038/s41586-022-04556-w
Slavin MalcolmOwen., 1992, The Adaptive Design of the Human Psyche: Psychoanalysis, Evolutionary Biology, and the Therapeutic Process
Slingerland E., 2021, Drunk: how we sipped, danced, and stumbled our way to civilization
Smelan OB, 2020, MOL PSYCHIATR, V25, P844, DOI 10.1038/s41380-018-0332-x
SMITH CA, 1985, J PERS SOC PSYCHOL, V48, P813, DOI 10.1037/0022-3514.48.4.813
Smith EO, 1999, EVOLUTIONARY MEDICI NE, P375
Smith J. M., 1964, Nature, V201, P1145, DOI 10.1038/2011145a0
SMITH JM, 1988, PHILOS T R SOC B, V319, P557, DOI 10.1098/rstb.1988.0065
Smith KM, 2020, EVOL HUM BEHAV, V41, P354, DOI 10.1016/j.evolhumbehav.2020.07.009
Smoller JW, 2019, MOL PSYCHIATR, V24, P409, DOI 10.1038/s41380-017-0010-4
Smuts B. B., 1985, Sex and friendship in baboons

SOLOMON RL, 1980, AM PSYCHOL, V35, P691, DOI 10.1037/0003-066X.35.8.691
St John-Smith P, 2013, HUM PSYCHOPHARM CLIN, V28, P394, DOI 10.1002/hup.2303
St JohnSmith P., 2022, EVOLUTIONARY PSYCHIA, P276
St JohnSmith P, 2022, EVOLUTIONARY PSYCHIA, P186
Stearns S.C., 1999, Evolution in health and disease
Stearns SC., 2016, Evolutionary Medicine
Stein DJ, 2022, WORLD PSYCHIATRY, V21, P393, DOI 10.1002/wps.20998
Stein DJ, 2019, NAT REV DIS PRIMERS, V5, DOI 10.1038/s41572-019-0102-3
Stein DJ, 2013, CURR OPIN PSYCHIATR, V26, P493, DOI 10.1097/YCO.0b013e3283642dfd
Stein DJ, 2006, PROG NEURO-PSYCHOPH, V30, P765, DOI 10.1016/j.pnpbp.2006.01.002
Stein DJ., 2011, PHILOS PSYCHOPHARMAC
Stein MB, 2008, LANCET, V371, P1115, DOI 10.1016/S0140-6736(08)60488-2
Sterling P., 2020, What is health? Allostasis and the evolution of human design
Stevens A., 2015, Evolutionary Psychiatry: A New Beginning
Stieglitz J, 2015, BRAIN BEHAV IMMUN, V49, P130, DOI 10.1016/j.bbi.2015.05.008
Stieglitz J, 2015, J GERONTOL B-PSYCHOL, V70, P948, DOI 10.1093/geronb/gbu080
Strassmann JE, 2011, ANNU REV MICROBIOL, V65, P349, DOI 10.1146/annurev.micro.112408.134109
Suddendorf Thomas., 2022, The Invention of Tomorrow. A Natural History of Foresight
Sullivan RJ, 2002, ADDICTION, V97, P389, DOI 10.1046/j.1360-0443.2002.00024.x
SURBEY MK, 1987, ETHOL SOCIOBIOL, V8, pS47
Swanepoel A., 2017, BJPsych Advances, V23, P410, DOI [DOI 10.1192/APT.BP.116.016659, 10.1192/apt.bp.116.0166594]
Symons D., 1979, The Evolution of Human Sexuality
Tinbergen N., 1963, Zeitschrift fuer Tierpsychologie, V20, P410
TOOBY J, 1990, ETHOL SOCIOBIOL, V11, P375, DOI 10.1016/0162-3095(90)90017-Z
Tooby J., 1996, Evolution of social behaviour patterns in primates and man, V88, P119, DOI DOI 10.1002/(SICI)1520-6300(1998)10:5<681::AID-
Trevathan WR., 2007, EVOLUTIONARY MED, V2nd ed.
Trillmichl F, 2018, BIOL LETTERS, V14, DOI 10.1098/rsbl.2017.0740
Trivers R., 2002, NATURAL SELECTION SO
Troisi A, 2001, FUNCT NEUROL, V16, P237
Troisi A, 2012, APPL EVOLUTIONARY PS, P276
Turkheimer E, 2014, ANNU REV PSYCHOL, V65, P515, DOI 10.1146/annurev-psych-113011-143752
van Dongen J, 2013, AM J MED GENET B, V162B, P122, DOI 10.1002/ajmg.b.32135
Varga S., 2016, Evolutionary Thinking in Medicine, P347
Vespignani A, 2010, NATURE, V464, P984, DOI 10.1038/464984a
Vogel EA, 2014, PSYCHOL POP MEDIA CU, V3, P206, DOI 10.1037/ppm0000047
VOLAND E, 1989, ETHOL SOCIOBIOL, V10, P223, DOI 10.1016/0162-3095(89)90001-0
Volkow ND, 2019, PHYSIOL REV, V99, P2115, DOI 10.1152/physrev.00014.2018

Volkow ND, 2018, AM J PSYCHIAT, V175, P729, DOI 10.1176/appi.ajp.2018.17101174
Wakefield JC., 2015, OXFORD HDB MOOD DISO, P83
Wakefield JC, 2007, WORLD PSYCHIATRY, V6, P149
Wakefield JC, 2017, EVOL PSYCHOL-SER, P37, DOI 10.1007/978-3-319-60576-0_2
Walsh B, 2018, EVOLUTION AND SELECTION OF QUANTITATIVE TRAITS, P1, DOI 10.1093/oso/9780198830870.001.0001
Wang JJ, 2016, P NATL ACAD SCI USA, V113, P6444, DOI 10.1073/pnas.1601465113
Watson HJ, 2019, NAT GENET, V51, P1207, DOI 10.1038/s41588-019-0439-2
Watson PJ, 2002, J AFFECT DISORDERS, V72, P1, DOI 10.1016/S0165-0327(01)00459-1
Weisfeld G., 1999, EVOLUTIONARY PRINCIP
Weiss KM, 2008, GENETICS, V179, P1741, DOI 10.1534/genetics.108.094128
Welling L.L. M., 2019, The Oxford Handbook of Evolutionary Psychology and Behavioral Endocrinology
Wenegrat B., 1984, Sociobiology and mental disorder
West SA, 2008, J EVOLUTION BIOL, V21, P374, DOI 10.1111/j.1420-9101.2007.01458.x
West SA, 2007, CURR BIOL, V17, pR661, DOI 10.1016/j.cub.2007.06.004
West-Eberhard Mary Jane, 2003, pi
WESTEBERHARD MJ, 1979, P AM PHILOS SOC, V123, P222
Westneat D. F., 2010, EVOLUTIONARY BEHAV E
Westneat DF, 2019, TRENDS ECOL EVOL, V34, P555, DOI 10.1016/j.tree.2019.02.010
Whiten A, 2012, PHILOS T R SOC B, V367, P2119, DOI 10.1098/rstb.2012.0114
Williams G. C., 1966, P307
WILLIAMS GC, 1957, EVOLUTION, V11, P398, DOI 10.1111/j.1558-5646.1957.tb02911.x
WILLIAMS GC, 1991, Q REV BIOL, V66, P1, DOI 10.1086/417048
Wilson E.O., 1975, P1
Winter J.C., 2000, TOBACCO USE NATIVE N
Wittchen HU, 2001, J CLIN PSYCHIAT, V62, P15
Wolf M, 2010, PHILOS T R SOC B, V365, P3959, DOI 10.1098/rstb.2010.0215
Wolpert Lewis, 2008, Philos Ethics Humanit Med, V3, P8, DOI 10.1186/1747-5341-3-8
Woolf CJ, 2011, PAIN, V152, pS2, DOI 10.1016/j.pain.2010.09.030
Woolfson A, 2019, NATURE, V566, P180, DOI 10.1038/d41586-019-00521-2
Wright R., 1994, MORAL ANIMAL EVOLUTI
Wrosch C, 2003, PERS SOC PSYCHOL B, V29, P1494, DOI 10.1177/0146167203256921
Wrosch C, 2007, PERS SOC PSYCHOL B, V33, P251, DOI 10.1177/0146167206294905
Zampieri F, 2009, Q REV BIOL, V84, P333, DOI 10.1086/648122
Zimmer C., 2011, EVOLUTION TRIUMPH ID
Zucker RA, 2014, NEBR SYM MOTIV, V61, P51, DOI 10.1007/978-1-4939-0653-6_4
NR 472
TC 38
Z9 39
U1 11

U2 40
PU WILEY
PI HOBOKEN
PA 111 RIVER ST, HOBOKEN 07030-5774, NJ USA
SN 1723-8617
EI 2051-5545
J9 WORLD PSYCHIATRY
JI World Psychiatry
PD JUN
PY 2023
VL 22
IS 2
BP 177
EP 202
DI 10.1002/wps.21072
PG 26
WC Psychiatry
WE Science Citation Index Expanded (SCI-EXPANDED); Social Science Citation Index (SSCI)
SC Psychiatry
GA G3PY9
UT WOS:000988329100003
PM 37159362
OA Green Submitted, Bronze, Green Published
DA 2025-05-07
ER

PT J
AU Gunady, EF
   Ware, KE
   Plumlee, SH
   Devos, N
   Corcoran, D
   Prinz, J
   Misetic, H
   Ciccarelli, FD
   Harrison, TM
   Thorne, JL
   Schopler, R
   Everitt, J
   Eward, WC
   Somarelli, JA
AF Gunady, Ella F.
   Ware, Kathryn E.
   Plumlee, Sarah Hoskinson
   Devos, Nicolas
   Corcoran, David
   Prinz, Joseph
   Misetic, Hrvoje
   Ciccarelli, Francesca D.
   Harrison, Tara M.
   Thorne, Jeffrey L.
   Schopler, Robert
   Everitt, Jeffrey, I

Eward, William C.
Somarelli, Jason A.
TI Exome sequencing of hepatocellular carcinoma in lemurs identifies potential cancer drivers A pilot study
SO EVOLUTION MEDICINE AND PUBLIC HEALTH
LA English
DT Article
DE liver cancer; mutation; prosimians; non-human primates; TP53; ARID1A; CTNNB1
ID MUTATIONAL PROCESSES; POOR-PROGNOSIS; OVEREXPRESSION; GENERATION; SIGNATURES; PATTERNS; GENES
AB Background and objectives Hepatocellular carcinoma occurs frequently in prosimians, but the cause of these liver cancers in this group is unknown. Characterizing the genetic changes associated with hepatocellular carcinoma in prosimians may point to possible causes, treatments and methods of prevention, aiding conservation efforts that are particularly crucial to the survival of endangered lemurs. Although genomic studies of cancer in non-human primates have been hampered by a lack of tools, recent studies have demonstrated the efficacy of using human exome capture reagents across primates. Methodology In this proof-of-principle study, we applied human exome capture reagents to tumor-normal pairs from five lemurs with hepatocellular carcinoma to characterize the mutational landscape of this disease in lemurs. Results Several genes implicated in human hepatocellular carcinoma, including ARID1A, TP53 and CTNNB1, were mutated in multiple lemurs, and analysis of cancer driver genes mutated in these samples identified enrichment of genes involved with TP53 degradation and regulation. In addition to these similarities with human hepatocellular carcinoma, we also noted unique features, including six genes that contain mutations in all five lemurs. Interestingly, these genes are infrequently mutated in human hepatocellular carcinoma, suggesting potential differences in the etiology and/or progression of this cancer in lemurs and humans. Conclusions and implications Collectively, this pilot study suggests that human exome capture reagents are a promising tool for genomic studies of cancer in lemurs and other non-human primates. Lay Summary Hepatocellular carcinoma occurs frequently in prosimians, but the cause of these liver cancers is unknown. In this proof-of-principle study, we applied human DNA sequencing tools to tumor-normal pairs from five lemurs with hepatocellular carcinoma and compared the lemur mutation profiles to those of human hepatocellular carcinomas.
C1 [Gunady, Ella F.; Ware, Kathryn E.; Somarelli, Jason A.] Duke Univ, Dept Med, Med Ctr, 905 Lasalle St,GSRBI Room 3044, Durham, NC 27710 USA.
[Plumlee, Sarah Hoskinson; Eward, William C.] Duke Univ, Dept Orthopaed, Med Ctr, Durham, NC 27710 USA.
[Devos, Nicolas; Corcoran, David; Prinz, Joseph] Duke Univ, Duke Ctr Genom & Computat Biol, Med Ctr, Durham, NC 27710 USA.
[Misetic, Hrvoje; Ciccarelli, Francesca D.] Francis Crick Inst, Canc Syst Biol Lab, London NW1 1AT, England.
[Misetic, Hrvoje; Ciccarelli, Francesca D.] Kings Coll London, Sch Canc & Pharmaceut Sci, London SE1 1UL, England.
[Harrison, Tara M.] North Carolina State Univ, Coll Vet Med, Dept Clin Sci, Raleigh, NC USA.
[Harrison, Tara M.] North Carolina State Univ, Coll Vet Med, Exot Species Canc Res Alliance, Raleigh, NC USA.

[Thorne, Jeffrey L.] North Carolina State Univ, Dept Biol Sci, Raleigh, NC USA.
[Thorne, Jeffrey L.] North Carolina State Univ, Dept Stat, Raleigh, NC USA.
[Schopler, Robert] Duke Lemur Ctr, Durham, NC 27705 USA.
[Everitt, Jeffrey, I] Duke Univ, Dept Pathol, Med Ctr, Durham, NC 27710 USA.
[Everitt, Jeffrey, I; Eward, William C.; Somarelli, Jason A.] Duke Canc Inst, Durham, NC 27710 USA.
C3 Duke University; Duke University; Duke University; Francis Crick Institute; University of London; King's College London; North Carolina State University; North Carolina State University; North Carolina State University; North Carolina State University; Duke University; Duke University
RP Somarelli, JA (autor correspondente), Duke Univ, Dept Med, Med Ctr, 905 Lasalle St,GSRBI Room 3044, Durham, NC 27710 USA.
EM jason.somarelli@duke.edu
RI Devos, Nicolas/E-7490-2015; Ciccarelli, Francesca/D-5380-2014
OI Gunady, Ella/0000-0002-9212-0127; Harrison, Tara/0000-0002-2072-5936; Somarelli, Jason/0000-0003-1510-9343; Everitt, Jeffrey/0000-0003-0273-6284; Ciccarelli, Francesca/0000-0002-9325-0900
FU Triangle Center for Evolutionary Medicine
FX This work was supported by the Triangle Center for Evolutionary Medicine (to T.M.H., J.L.T. and J.A.S.).
CR Ahn SM, 2014, HEPATOLOGY, V60, P1972, DOI 10.1002/hep.27198
Alexandrov LB, 2015, NAT GENET, V47, P1402, DOI 10.1038/ng.3441
Anandakrishnan R, 2019, PLOS COMPUT BIOL, V15, DOI 10.1371/journal.pcbi.1006881
[Anonymous], 2020, ALM 3 LEM N ATL RIGH
[Anonymous], LIHC
Ashburner M, 2000, NAT GENET, V25, P25, DOI 10.1038/75556
Braconi C, 2011, SEMIN ONCOL, V38, P752, DOI 10.1053/j.seminoncol.2011.08.001
Campbell CR, 2021, HEREDITY, V127, P233, DOI 10.1038/s41437-021-00446-5
Chen H, 2011, BMC BIOINFORMATICS, V12, DOI 10.1186/1471-2105-12-35
Chen H, 2019, FRONT IMMUNOL, V10, DOI 10.3389/fimmu.2019.01113
Chen SF, 2018, BIOINFORMATICS, V34, P884, DOI 10.1093/bioinformatics/bty560
Choi HS, 2010, BMC CANCER, V10, DOI 10.1186/1471-2407-10-122
Cibulskis K, 2013, NAT BIOTECHNOL, V31, P213, DOI 10.1038/nbt.2514
COSMIC Mutational Signatures, 2021, SBS SIGN
Croft D, 2014, NUCLEIC ACIDS RES, V42, pD472, DOI [10.1093/nar/gkz1031, 10.1093/nar/gkt1102]
Cullen JM, 2011, VET PATHOL, V48, P868, DOI 10.1177/0300985810388526
Fantini D, 2020, SCI REP-UK, V10, DOI 10.1038/s41598-020-75062-0
Fujimoto A, 2012, NAT GENET, V44, P760, DOI 10.1038/ng.2291
Goncearenco A, 2017, NUCLEIC ACIDS RES, V45, pW514, DOI 10.1093/nar/gkx367
Hämmerle M, 2013, HEPATOLOGY, V58, P1703, DOI 10.1002/hep.26537
Harding JJ, 2019, CLIN CANCER RES, V25, P2116, DOI 10.1158/1078-0432.CCR-18-2293

Ilboudo A, 2014, BMC CANCER, V14, DOI 10.1186/1471-2407-14-7
Jin X, 2012, PLOS ONE, V7, DOI 10.1371/journal.pone.0040637
Letouzé E, 2017, NAT COMMUN, V8, DOI 10.1038/s41467-017-01358-x
Lex A, 2014, IEEE T VIS COMPUT GR, V20, P1983, DOI 10.1109/TVCG.2014.2346248
Li H, 2010, BIOINFORMATICS, V26, P589, DOI 10.1093/bioinformatics/btp698
McAloose D, 2009, NAT REV CANCER, V9, P517, DOI 10.1038/nrc2665
McKenna A, 2010, GENOME RES, V20, P1297, DOI 10.1101/gr.107524.110
McLaren W, 2016, GENOME BIOL, V17, DOI 10.1186/s13059-016-0974-4
Mittermeier R, 2008, INT J PRIMATOL, V29, P1607, DOI 10.1007/s10764-008-9317-y
Mudunuri U, 2009, BIOINFORMATICS, V25, P555, DOI 10.1093/bioinformatics/btn654
Nulsen J, 2021, GENOME MED, V13, DOI 10.1186/s13073-021-00830-0
Pags H, 2017, BSGENOME BIOCONDUCTO
Picard, BROAD I
Rahbari R, 2016, NAT GENET, V48, P126, DOI 10.1038/ng.3469
Raudvere U, 2019, NUCLEIC ACIDS RES, V47, pW191, DOI 10.1093/nar/gkz369
Remick AK, 2009, VET PATHOL, V46, P746, DOI 10.1354/vp.08-VP-0154-R-FL
Repana D, 2019, GENOME BIOL, V20, DOI 10.1186/s13059-018-1612-0
Rosenthal R, 2016, GENOME BIOL, V17, DOI 10.1186/s13059-016-0893-4
Schulze K, 2015, NAT GENET, V47, P505, DOI 10.1038/ng.3252
Slenter DN, 2018, NUCLEIC ACIDS RES, V46, pD661, DOI 10.1093/nar/gkx1064
Somarelli JA, 2020, MOL BIOL EVOL, V37, P11, DOI 10.1093/molbev/msz254
Teufel A, 2007, WORLD J GASTROENTERO, V13, P2271, DOI 10.3748/wjg.v13.i16.2271
Tornesello ML, 2013, GENOMICS, V102, P74, DOI 10.1016/j.ygeno.2013.04.001
Vallender EJ, 2011, GENOME BIOL, V12, DOI 10.1186/gb-2011-12-9-r87
Yamashita T, 2009, J HEPATOL, V50, P100, DOI 10.1016/j.jhep.2008.07.036
Zadrozny LM, 2010, VET PATHOL, V47, P306, DOI 10.1177/0300985809359380
Zhang PP, 2014, J BIOL CHEM, V289, P24724, DOI 10.1074/jbc.M114.567818
Zhao YY, 2021, AGING-US, V13, P4696, DOI 10.18632/aging.202586
Zuguang G, 2017, COMPLEXHEATMAP BIOCO
NR 50
TC 0
Z9 0
U1 0
U2 0
PU OXFORD UNIV PRESS
PI OXFORD
PA GREAT CLARENDON ST, OXFORD OX2 6DP, ENGLAND
EI 2050-6201
J9 EVOL MED PUBLIC HLTH
JI Evol. Med. Public Health
PD JAN 5
PY 2022
VL 10
IS 1
BP 221

EP 230
DI 10.1093/emph/eoac016
EA MAY 2022
PG 10
WC Evolutionary Biology; Public, Environmental & Occupational Health
WE Science Citation Index Expanded (SCI-EXPANDED)
SC Evolutionary Biology; Public, Environmental & Occupational Health
GA 1C0XE
UT WOS:000792851500001
PM 35557512
OA Green Published, gold
DA 2025-05-07
ER

PT J
AU Gupta, MK
Vadde, R
AF Gupta, Manoj Kumar
Vadde, Ramakrishna
TI Divergent evolution and purifying selection of the Type 2 diabetes gene
sequences inDrosophila: a phylogenomic study
SO GENETICA
LA English
DT Article
DE Type 2 diabetes; Natural selection; Drosophila; Phylogenomics
ID POSITIVE SELECTION; ADAPTIVE EVOLUTION; NATURAL-SELECTION;
ANIMAL-MODELS; DROSOPHILA; INSULIN; PROTEIN; RESISTANCE; PRESSURES;
RECEPTORS
AB The recently developed phylogenomic approach provides a unique way to identify disease risk or protective allele in any organism. While risk alleles evolve mostly under purifying selection, protective alleles are evolving either under balancing or positive selection. Owing to insufficient information, authors employed the phylogenomic approach to detect the nature of selection acting on type 2 diabetes (T2D) genes inDrosophilagenus using various models of CODEML utility of PAML.The obtained result revealed that T2D gene sequences are evolving under purifying selection. However, only a few sites in membrane proteins encoded viaCG8051, ZnT35C,andkar,are significantly evolving under positive selection under specific scenarios, which might be because of positive or adaptive evolution in response to changing niche, diet or other factors. In the near future, this information will be highly useful in the field of evolutionary medicine and the drug discovery process.
C1 [Gupta, Manoj Kumar; Vadde, Ramakrishna] Yogi Vemana Univ, Dept Biotechnol & Bioinformat, Kadapa 516005, Andhra Pradesh, India.
C3 Yogi Vemana University
RP Vadde, R (autor correspondente), Yogi Vemana Univ, Dept Biotechnol & Bioinformat, Kadapa 516005, Andhra Pradesh, India.
EM vrkrishna70@gmail.com
RI Vadde, Ramakrishna/AAJ-5349-2020
OI Gupta, Manoj Kumar/0000-0003-1558-9471; Ramakrishna,
Vadde/0000-0002-5223-7208
CR Abraham Mark James, 2015, SoftwareX, V1-2, P19, DOI 10.1016/j.softx.2015.06.001

Ahmed-Braimah YH, 2017, G3-GENES GENOM GENET, V7, P3145, DOI 10.1534/g3.117.1136
Al-Daghri NM, 2017, BMC EVOL BIOL, V17, DOI 10.1186/s12862-017-0897-z
Alfa RW, 2016, DIS MODEL MECH, V9, P365, DOI 10.1242/dmm.023887
ALTSCHUL SF, 1990, J MOL BIOL, V215, P403, DOI 10.1006/jmbi.1990.9999
Alvarez-Rendón JP, 2018, BIOMED RES INT, V2018, DOI 10.1155/2018/1417528
[Anonymous], 2014, R LANG ENV STAT COMP
[Anonymous], 2006, COMPUTATIONAL MOL EV
Baker KD, 2007, CELL METAB, V6, P257, DOI 10.1016/j.cmet.2007.09.002
Balaresque PL, 2007, HUM MOL GENET, V16, pR134, DOI 10.1093/hmg/ddm242
Baynest HW, 2015, J DIABETES METAB, V6, DOI 10.4172/2155-6156.1000541
Berger O, 1997, BIOPHYS J, V72, P2002, DOI 10.1016/S0006-3495(97)78845-3
Blekhman R, 2008, CURR BIOL, V18, P883, DOI 10.1016/j.cub.2008.04.074
Clark AG, 2007, NATURE, V450, P203, DOI 10.1038/nature06341
Domazet-Loso T, 2003, GENOME RES, V13, P2213, DOI 10.1101/gr.1311003
Domazet-Loso T, 2008, MOL BIOL EVOL, V25, P2699, DOI 10.1093/molbev/msn214
Donde R, 2019, AMINO ACIDS, V51, P839, DOI 10.1007/s00726-019-02727-0
Emes RD, 2004, GENOME RES, V14, P591, DOI 10.1101/gr.1940604
Farfán M, 2009, BIOL DIRECT, V4, DOI 10.1186/1745-6150-4-23
Fischman BJ, 2011, P NATL ACAD SCI USA, V108, P10847, DOI 10.1073/pnas.1100301108
Gardiner A, 2009, BIOL LETTERS, V5, P244, DOI 10.1098/rsbl.2008.0723
Gouda G, 2020, J BIOMOL STRUCT DYN, V38, P1158, DOI 10.1080/07391102.2019.1597771
Graham P, 2017, CURR TOP DEV BIOL, V121, P397, DOI 10.1016/bs.ctdb.2016.07.011
Grönke S, 2010, PLOS GENET, V6, DOI 10.1371/journal.pgen.1000857
Grunspan DZ, 2018, EVOL MED PUBLIC HLTH, P13, DOI 10.1093/emph/eox025
Gupta MK, 2021, J BIOMOL STRUCT DYN, V39, P1447, DOI 10.1080/07391102.2020.1733666
Gupta MK, 2019, ZOOLOGY, V136, DOI 10.1016/j.zool.2019.125693
Gupta MK, 2019, J MOL GRAPH MODEL, V88, P209, DOI 10.1016/j.jmgm.2019.01.018
Gupta MK, 2020, J BIOMOL STRUCT DYN, V38, P137, DOI 10.1080/07391102.2019.1567391
Gupta MK, 2019, J CELL BIOCHEM, V120, P10323, DOI 10.1002/jcb.28316
Hill T, 2019, MOL BIOL EVOL, V36, P1405, DOI 10.1093/molbev/msz059
Hu C, 2009, PLOS ONE, V4, DOI 10.1371/journal.pone.0007643
Hu YH, 2011, BMC BIOINFORMATICS, V12, DOI 10.1186/1471-2105-12-357
Jennings BH, 2011, MATER TODAY, V14, P190, DOI 10.1016/S1369-7021(11)70113-4
King AJF, 2012, BRIT J PHARMACOL, V166, P877, DOI 10.1111/j.1476-5381.2012.01911.x
Klimentidis YC, 2011, HUM GENET, V129, P407, DOI 10.1007/s00439-010-0935-z
Koenker R., 2018, Package quantreg
Konig B, 2001, INT ENCY SOCIAL BEHA, P10392
Kosiol C, 2008, PLOS GENET, V4, DOI 10.1371/journal.pgen.1000144
Krieger MJB, 2002, SCIENCE, V295, P328, DOI 10.1126/science.1065247
LASKOWSKI RA, 1993, J APPL CRYSTALLOGR, V26, P283, DOI 10.1107/S0021889892009944

Lee GH, 2004, GENETICS, V167, P311, DOI 10.1534/genetics.167.1.311
Lefébure T, 2009, GENOME RES, V19, P1224, DOI 10.1101/gr.089250.108
Lemkul JA, 2015, GROMACS TUTORIAL KAL
Little BB, 2017, AM J PHYS ANTHROPOL, V162, P561, DOI 10.1002/ajpa.23139
Liu Y, 2013, GENOMICS, V101, P318, DOI 10.1016/j.ygeno.2013.03.001
Lynn DJ, 2005, GENETICS, V170, P1189, DOI 10.1534/genetics.104.039040
Maurer K.J., 2015, Laboratory Animal Medicine, P1497
Morgan CC, 2012, BMC EVOL BIOL, V12, DOI 10.1186/1471-2148-12-114
Murillo-Maldonado JM, 2017, MECH DEVELOP, V144, P150, DOI 10.1016/j.mod.2016.09.004
Musselman LP, 2011, DIS MODEL MECH, V4, P842, DOI 10.1242/dmm.007948
Nesse RM, 2008, EVOL APPL, V1, P28, DOI 10.1111/j.1752-4571.2007.00006.x
NGAI J, 1993, CELL, V72, P657, DOI 10.1016/0092-8674(93)90396-8
O'Halloran TV, 2013, J CLIN INVEST, V123, P4136, DOI 10.1172/JCI72325
Pound LD, 2013, DIABETES, V62, P1547, DOI 10.2337/db12-1067
Rees DA, 2005, DIABETIC MED, V22, P359, DOI 10.1111/j.1464-5491.2005.01499.x
Rutter GA, 2015, DIABETOLOGIA, V58, P31, DOI 10.1007/s00125-014-3405-7
Ségurel L, 2013, EUR J HUM GENET, V21, P1146, DOI 10.1038/ejhg.2012.295
Skyler JS, 2017, DIABETES, V66, P241, DOI 10.2337/db16-0806
Spires D, 2019, CURR TOP MEMBR, V83, P353, DOI 10.1016/bs.ctm.2019.01.001
Swanson WJ, 2003, MOL BIOL EVOL, V20, P18, DOI 10.1093/oxfordjournals.molbev.a004233
Szklarczyk D, 2017, NUCLEIC ACIDS RES, V45, pD362, DOI 10.1093/nar/gkw937
Teng LH, 2017, FRONT PLANT SCI, V8, DOI 10.3389/fpls.2017.01429
Thirlaway K, 2001, LIFESTYLE RESPONSES
Tiwari P, 2015, J DIABETES RES, V2015, DOI 10.1155/2015/340838
Turner PJ, 2005, XMGRACE VERS 5 1 19, V19
Vishnoi A, 2010, GENOME RES, V20, P1574, DOI 10.1101/gr.109595.110
Wagner A, 2007, GENETICS, V176, P2451, DOI 10.1534/genetics.107.074732
Walker B.R., 2013, DAVIDSONS PRINCIPLES
Wiederstein M, 2007, NUCLEIC ACIDS RES, V35, pW407, DOI 10.1093/nar/gkm290
Willett CS, 2000, MOL BIOL EVOL, V17, P553, DOI 10.1093/oxfordjournals.molbev.a026335
Wolf MG, 2010, J COMPUT CHEM, V31, P2169, DOI 10.1002/jcc.21507
Wong A, 2008, MOL BIOL EVOL, V25, P497, DOI 10.1093/molbev/msm270
Yang W, 2003, J MOL EVOL, V57, P212, DOI 10.1007/s00239-003-2467-9
Yang ZH, 2002, MOL BIOL EVOL, V19, P49, DOI 10.1093/oxfordjournals.molbev.a003981
Yang ZH, 2000, TRENDS ECOL EVOL, V15, P496, DOI 10.1016/S0169-5347(00)01994-7
Yang ZH, 2007, MOL BIOL EVOL, V24, P1586, DOI 10.1093/molbev/msm088
NR 77
TC 4
Z9 4
U1 3
U2 43
PU SPRINGER

PI DORDRECHT
PA VAN GODEWIJCKSTRAAT 30, 3311 GZ DORDRECHT, NETHERLANDS
SN 0016-6707
EI 1573-6857
J9 GENETICA
JI Genetica
PD DEC
PY 2020
VL 148
IS 5-6
BP 269
EP 282
DI 10.1007/s10709-020-00101-7
EA AUG 2020
PG 14
WC Genetics & Heredity
WE Science Citation Index Expanded (SCI-EXPANDED)
SC Genetics & Heredity
GA OP9EQ
UT WOS:000560284000001
PM 32804315
DA 2025-05-07
ER

PT J
AU Ehrhardt, B
Roeder, T
Krauss-Etschmann, S
AF Ehrhardt, Birte
Roeder, Thomas
Krauss-Etschmann, Susanne
TI Drosophila melanogaster as an Alternative Model to Higher
Organisms for In Vivo Lung Research
SO INTERNATIONAL JOURNAL OF MOLECULAR SCIENCES
LA English
DT Review
DE Drosophila melanogaster; COPD; asthma; lung research; alternative
model
ID CIGARETTE-SMOKE; FLY; EXPOSURE; ASTHMA; EXPRESSION; GENETICS;
DISEASES;
BIOLOGY; TARGET
AB COPD and asthma are lung diseases that cause considerable burden to more than 800 million people worldwide. As both lung diseases are so far incurable, it is mandatory to understand the mechanisms underlying disease development and progression for developing novel therapeutic approaches. Exposures to environmental cues such as cigarette smoke in earliest life are known to increase disease risks in the individual's own future. To explore the pathomechanisms leading to later airway disease, mammalian models are instrumental. However, such in vivo experiments are time-consuming and burdensome for the animals, which applies in particular to transgenerational studies. Along this line, the fruit fly Drosophila melanogaster comes with several advantages for research in this field. The short lifespan facilitates transgenerational studies. A high number of evolutionary conserved signaling pathways, together with a

large toolbox for tissue-specific gene modification, has the potential to identify novel target genes involved in disease development. A well-defined airway microbiome could help to untangle interactions between disease development and microbiome composition. In the following article, Drosophila melanogaster is therefore presented and discussed as an alternative in vivo model to investigate airway diseases that can complement and/or replace models in higher organisms.
C1 [Ehrhardt, Birte; Krauss-Etschmann, Susanne] German Ctr Lung Res DZL, Leibniz Lung Ctr, Airway Res Ctr North ARCN, Div Early Life Origins Chron Lung Dis,Res Ctr Bors, D-23845 Borstel, Germany.
[Roeder, Thomas] Christian Albrechts Univ Kiel, Inst Zool, Airway Res Ctr North ARCN, German Ctr Lung Res DZL,Div Mol Physiol, D-24118 Kiel, Germany.
[Krauss-Etschmann, Susanne] German Ctr Lung Res DZL, Res Ctr Borstel, Airway Res Ctr North ARCN, DZL Lab Expt Microbiome Res, D-23845 Borstel, Germany.
[Krauss-Etschmann, Susanne] Christian Albrechts Univ Kiel, Inst Expt Med, D-24105 Kiel, Germany.
C3 University of Kiel; Leibniz Association; Forschungszentrum Borstel; University of Kiel
RP Krauss-Etschmann, S (autor correspondente), German Ctr Lung Res DZL, Leibniz Lung Ctr, Airway Res Ctr North ARCN, Div Early Life Origins Chron Lung Dis,Res Ctr Bors, D-23845 Borstel, Germany.; Krauss-Etschmann, S (autor correspondente), German Ctr Lung Res DZL, Res Ctr Borstel, Airway Res Ctr North ARCN, DZL Lab Expt Microbiome Res, D-23845 Borstel, Germany.; Krauss-Etschmann, S (autor correspondente), Christian Albrechts Univ Kiel, Inst Expt Med, D-24105 Kiel, Germany.
EM skrauss-etschmann@fz-borstel.de
OI Ehrhardt, Birte/0000-0002-2332-9752
FU Leibniz Science Campus Evolutionary Medicine of the LUNG (EvoLUNG), Leibniz Association Germany; Leibniz Science Campus Evolutionary Medicine of the LUNG; Leibniz Association Germany
FX This research was supported by Leibniz Science Campus Evolutionary Medicine of the LUNG (EvoLUNG), Leibniz Association Germany.
CR Almansouri M., 2024, Curr. Trends Biotechnol. Pharm, V18, P1655, DOI [10.5530/ctbp.2024.1.13, DOI 10.5530/CTBP.2024.1.13]
Angstmann H, 2023, ENVIRON MICROBIOME, V18, DOI 10.1186/s40793-023-00506-9
[Anonymous], 2024, Alliance of Genome Resources Search across Species
Baldarelli RM, 2024, GENETICS, V227, DOI 10.1093/genetics/iyae031
Banzato R, 2024, INFLAMMATION, V47, P958, DOI 10.1007/s10753-023-01953-9
Basil MC, 2020, SEMIN CELL DEV BIOL, V100, P88, DOI 10.1016/j.semcdb.2019.11.006
Bergman P, 2017, CURR TOP DEV BIOL, V121, P29, DOI 10.1016/bs.ctdb.2016.07.002
Bossen J, 2019, MOL CANCER THER, V18, P1659, DOI 10.1158/1535-7163.MCT-19-0168
Brody JS, 2012, INT J CANCER, V131, P2754, DOI 10.1002/ijc.27829
Bui DS, 2022, LANCET RESP MED, V10, P478, DOI 10.1016/S2213-2600(21)00508-7
Bui DS, 2018, LANCET RESP MED, V6, P535, DOI 10.1016/S2213-2600(18)30100-0
Bult CJ, 2023, MAMM GENOME, V34, P531, DOI 10.1007/s00335-023-10015-2

Burggren W, 2017, BIOL OPEN, V6, P471, DOI 10.1242/bio.023994
Chen J, 2023, EUR RESPIR J, V62, DOI 10.1183/13993003.01374-2022
Cotterill S, 2024, INT J MOL SCI, V25, DOI 10.3390/ijms25010292
Dharmage SC, 2023, LANCET RESP MED, V11, P273, DOI 10.1016/S2213-2600(22)00364-2
Dionne MS, 2008, DIS MODEL MECH, V1, P43, DOI 10.1242/dmm.000307
Dong JJ, 2013, INT IMMUNOPHARMACOL, V17, P885, DOI 10.1016/j.intimp.2013.09.003
Duffy JB, 2002, GENESIS, V34, P1, DOI 10.1002/gene.10150
Ehrhardt B, 2024, SCI REP-UK, V14, DOI 10.1038/s41598-024-66752-0
El-Husseini ZW, 2020, LANCET RESP MED, V8, P1045, DOI 10.1016/S2213-2600(20)30363-5
El-Merhie N, 2021, SCI REP-UK, V11, DOI 10.1038/s41598-021-81607-8
Erzurum SC, 2016, ANN AM THORAC SOC, V13, pS35, DOI 10.1513/AnnalsATS.201506-385MG
Faramawy MM, 2009, J CRIT CARE, V24, DOI 10.1016/j.jcrc.2009.06.004
Ferrini M, 2017, PART FIBRE TOXICOL, V14, DOI 10.1186/s12989-017-0212-6
Fricker M, 2014, EXPERT OPIN DRUG DIS, V9, P629, DOI 10.1517/17460441.2014.909805
Global Initiative for Asthma (GINA), 2023, Global strategy for asthma management and prevention
Global Initiative for Chronic Obstructive Lung Disease, 2024, Global strategy for the diagnosis, management, and prevention of chronic obstructive pulmonary disease
Hales KG, 2015, GENETICS, V201, P815, DOI 10.1534/genetics.115.183392
Hammer B, 2021, INT J OBESITY, V45, P1623, DOI 10.1038/s41366-021-00798-2
Hammer B, 2018, CLIN EXP ALLERGY, V48, P1378, DOI 10.1111/cea.13283
Hetz SK, 2005, NATURE, V433, P516, DOI 10.1038/nature03106
Holgate ST, 2011, IMMUNOL REV, V242, P205, DOI 10.1111/j.1600-065X.2011.01030.x
Jennings BH, 2011, MATER TODAY, V14, P190, DOI 10.1016/S1369-7021(11)70113-4
Kallsen K, 2015, J ALLERGY CLIN IMMUN, V136, P1105, DOI 10.1016/j.jaci.2015.04.009
Li HJ, 2022, SCIENCE, V375, P991, DOI 10.1126/science.abk2432
Li Y, 2024, NAT COMMUN, V15, DOI 10.1038/s41467-024-46455-w
Neavin DR, 2018, INT J MOL SCI, V19, DOI 10.3390/ijms19123851
Nichols CD, 2012, JOVE-J VIS EXP, DOI 10.3791/3795
Pandey UB, 2011, PHARMACOL REV, V63, P411, DOI 10.1124/pr.110.003293
Pesola F, 2024, ADDICTION, V119, P1493, DOI 10.1111/add.16507
Piovesan A, 2019, BMC RES NOTES, V12, DOI 10.1186/s13104-019-4343-8
Prange R, 2018, AGING-US, V10, P2122, DOI 10.18632/aging.101536
Roeder T., 2024, Res. Sq, DOI [10.21203/rs.3.rs-3978430/v1, DOI 10.21203/RS.3.RS-3978430/V1]
Rosato E, 2006, NAT PROTOC, V1, P559, DOI 10.1038/nprot.2006.79
Russell RJ, 2024, EUR RESPIR J, V63, DOI 10.1183/13993003.01397-2023
Schottenfeld J, 2010, CURR OPIN CELL BIOL, V22, P633, DOI 10.1016/j.ceb.2010.07.016
Serban KA, 2018, METHODS MOL BIOL, V1809, P379, DOI 10.1007/978-1-4939-8570-8_25
Silverman EK, 2020, ANNU REV PHYSIOL, V82, P413, DOI 10.1146/annurev-physiol-021317-121224

Singh SP, 2017, J IMMUNOL, V198, P3815, DOI 10.4049/jimmunol.1700014
Sirocko KT, 2022, ENVIRON POLLUT, V309, DOI 10.1016/j.envpol.2022.119696
Spira A, 2004, P NATL ACAD SCI USA, V101, P10143, DOI 10.1073/pnas.0401422101
Svanes C, 2010, THORAX, V65, P14, DOI 10.1136/thx.2008.112136
Svanes C, 2023, J INTERN MED, V293, P531, DOI 10.1111/joim.13611
Takahashi N, 2024, ANTIOXIDANTS-BASEL, V13, DOI 10.3390/antiox13010030
Tomar A, 2024, NATURE, V630, P720, DOI 10.1038/s41586-024-07472-3
Tzou P, 2000, IMMUNITY, V13, P737, DOI 10.1016/S1074-7613(00)00072-8
Unachukwu U, 2017, FASEB J, V31, P2340, DOI 10.1096/fj.201601063R
Ünver R, 2016, TURK THORAC J, V17, P153, DOI [10.5152/TurkThoracJ.2016.003, 10.5578/ttj.30518]
van Hees H, 2011, AM J PHYSIOL-LUNG C, V301, pL110, DOI 10.1152/ajplung.00396.2010
Vitenberga Z, 2019, MEDICINA-LITHUANIA, V55, DOI 10.3390/medicina55080496
von Mutius E, 2018, LANCET RESP MED, V6, P482, DOI 10.1016/S2213-2600(18)30141-3
WHO, 2019, Global Health Estimates: Life expectancy and leading causes of death and disability
WHO, 2023, Lung cancer
Xiao R, 2016, ENVIRON MOL MUTAGEN, V57, P190, DOI 10.1002/em.21998
Yamamoto S, 2014, CELL, V159, P200, DOI 10.1016/j.cell.2014.09.002
Yatsenko AS, 2014, JOVE-J VIS EXP, DOI 10.3791/51681
NR 67
TC 0
Z9 0
U1 1
U2 2
PU MDPI
PI BASEL
PA ST ALBAN-ANLAGE 66, CH-4052 BASEL, SWITZERLAND
SN 1661-6596
EI 1422-0067
J9 INT J MOL SCI
JI Int. J. Mol. Sci.
PD OCT
PY 2024
VL 25
IS 19
AR 10324
DI 10.3390/ijms251910324
PG 14
WC Biochemistry & Molecular Biology; Chemistry, Multidisciplinary
WE Science Citation Index Expanded (SCI-EXPANDED)
SC Biochemistry & Molecular Biology; Chemistry
GA J0N2H
UT WOS:001334119200001
PM 39408654
OA gold
DA 2025-05-07
ER

PT J
AU Corbett, S
   Morin-Papunen, L
AF Corbett, Stephen
   Morin-Papunen, Laure
TI The Polycystic Ovary Syndrome and recent human evolution
SO MOLECULAR AND CELLULAR ENDOCRINOLOGY
LA English
DT Review
DE Polycystic Ovary Syndrome; Fertility selection; Fecundability;
   Reproductive ecology; Evolution; Thrifty genotype
ID EARLY-PREGNANCY LOSS; OLIGOMENORRHEA AND/OR HIRSUTISM; IMPAIRED
   GLUCOSE-TOLERANCE; SELF-REPORTED SYMPTOMS; BODY-MASS INDEX;
   TERM-FOLLOW-UP; INSULIN-RESISTANCE; WEIGHT-GAIN; LUTEINIZING-HORMONE;
   NATURAL-SELECTION
AB The Polycystic Ovary Syndrome (PCOS) is a complex endocrine disorder characterised both by reproductive and metabolic disturbance, and is the most common cause globally of ovarian infertility. It is also a familial polygenic condition, linked genetically to both Type 2 diabetes and the metabolic syndrome. The striking evolutionary paradox of this prominent genetically-based condition, which impairs fertility, is that not only should it have diminished in prevalence, but it should have done so rapidly - unless there has been some form of balancing selection. The emerging discipline of evolutionary medicine can provide important insights into the causes and patterns of occurrence of common diseases such as PCOS. In this paper we review the impacts of PCOS on infertility, fecundability and lifetime reproductive success and then critically appraise published hypotheses about the evolutionary origins of PCOS and related conditions. Crown Copyright (C) 2013 Published by Elsevier Ireland Ltd. All rights reserved.
C1 [Corbett, Stephen] Cumberland Hosp, Clin Support Div Western, Parramatta, Bc Nsw 2124, Australia.
   [Corbett, Stephen] Univ Sydney, Sch Publ Hlth, Sydney, NSW 2006, Australia.
   [Corbett, Stephen] Univ Sydney, Western Clin Sch, Sydney, NSW 2006, Australia.
   [Morin-Papunen, Laure] Dept Gynecol & Obstet, FIN-90029 Oulu, Finland.
C3 University of Sydney; University of Sydney
RP Corbett, S (autor correspondente), Cumberland Hosp, Clin Support Div Western, Gungurra Bldg 68,Locked Bag 7118, Parramatta, Bc Nsw 2124, Australia.
EM Stephen_Corbett@wsahs.nsw.gov.au; lmp@sun3.oulu.fi
OI Morin-Papunen, Laure/0000-0001-5987-7534
CR Abbott DH, 2002, J ENDOCRINOL, V174, DOI 10.1677/joe.0.1740001
   Abbott DH, 1998, TRENDS ENDOCRIN MET, V9, P62, DOI 10.1016/S1043-2760(98)00019-8
   Allen JS, 1996, CURR ANTHROPOL, V37, P831, DOI 10.1086/204566
   Asunción M, 2000, J CLIN ENDOCR METAB, V85, P2434, DOI 10.1210/jc.85.7.2434
   Azziz R, 2004, J CLIN ENDOCR METAB, V89, P453, DOI 10.1210/jc.2003-031122
   Azziz R, 2004, J CLIN ENDOCR METAB, V89, P2745, DOI 10.1210/jc.2003-032046
   Azziz R., 2010, FERTIL STERIL

Balen A, 2002, HUM REPROD, V17, P2219, DOI 10.1093/humrep/17.9.2219
Balen AH, 2007, BMJ-BRIT MED J, V335, P608, DOI 10.1136/bmj.39324.662049.80
BALEN AH, 1993, HUM REPROD, V8, P123, DOI 10.1093/humrep/8.suppl_2.123
Barnett DK, 2003, AM J HUM BIOL, V15, P296, DOI 10.1002/ajhb.10149
Bateson P, 2004, NATURE, V430, P419, DOI 10.1038/nature02725
Bellver J, 2004, AM J OBSTET GYNECOL, V190, P293, DOI 10.1016/j.ajog.2003.10.704
Black RE, 2010, LANCET, V375, P1969, DOI 10.1016/S0140-6736(10)60549-1
BODMER WF, 1965, GENETICS, V51, P411
Broekmans FJ, 2006, BJOG-INT J OBSTET GY, V113, P1210, DOI 10.1111/j.1471-0528.2006.01008.x
Bruni V, 2003, ANN NY ACAD SCI, V997, P307, DOI 10.1196/annals.1290.034
Byars SG, 2010, P NATL ACAD SCI USA, V107, P1787, DOI 10.1073/pnas.0906199106
Catalano PM, 1999, EUR J CLIN NUTR, V53, pS124, DOI 10.1038/sj.ejcn.1600753
Cavalli-Sforza L., 1971, GENETICS HUMAN POPUL, P184
Chen XL, 2008, EUR J OBSTET GYN R B, V139, P59, DOI 10.1016/j.ejogrb.2007.12.018
Chen ZJ, 2011, NAT GENET, V43, P55, DOI 10.1038/ng.732
Chiu CJ, 2010, DIABETES-METAB RES, V26, P323, DOI 10.1002/dmrr.1095
Chiu M, 2011, DIABETES CARE, V34, P1741, DOI 10.2337/dc10-2300
Colagiuri S, 2002, EUR J CLIN NUTR, V56, pS30, DOI 10.1038/sj.ejcn.1601351
COONEY GJ, 1994, BAILLIERE CLIN ENDOC, V8, P481, DOI 10.1016/S0950-351X(05)80283-6
COOPER HE, 1968, AM J OBSTET GYNECOL, V100, P371, DOI 10.1016/S0002-9378(15)33704-2
Corbett SJ, 2009, AM J HUM BIOL, V21, P587, DOI 10.1002/ajhb.20937
Cordain L, 2005, AM J CLIN NUTR, V81, P341, DOI 10.1093/ajcn.81.2.341
CROW JF, 1958, AM ANTHROPOL, V60, P1
DAHLGREN E, 1992, FERTIL STERIL, V57, P505
Darwin C, 2009, ON THE ORIGIN OF SPECIES, P1, DOI 10.1017/CBO9780511694295.004
de Lorgeril M, 2002, CARDIOVASC RES, V54, P503, DOI 10.1016/S0008-6363(01)00545-4
de Zegher F, 2009, J CLIN ENDOCR METAB, V94, P3682, DOI 10.1210/jc.2009-1608
Dei M, 2008, GYNECOL ENDOCRINOL, V24, P459, DOI 10.1080/09513590802246141
Dewailly D, 2006, J CLIN ENDOCR METAB, V91, P3922, DOI 10.1210/jc.2006-1054
Di Rienzo A, 2005, TRENDS GENET, V21, P596, DOI 10.1016/j.tig.2005.08.007
Diamanti-Kandarakis E, 1999, J CLIN ENDOCR METAB, V84, P4006, DOI 10.1210/jc.84.11.4006
Diamond J, 2003, NATURE, V423, P599, DOI 10.1038/423599a
Dokras A, 2006, OBSTET GYNECOL, V108, P61, DOI 10.1097/01.AOG.0000219768.08249.b6
Dunaif A, 1996, J CLIN ENDOCR METAB, V81, P942, DOI 10.1210/jc.81.3.942

DUNAIF A, 1987, J CLIN ENDOCR METAB, V65, P499, DOI 10.1210/jcem-65-3-499
Eggers S, 2007, ANTHROPOL ANZ, V65, P169, DOI 10.1127/anthranz/65/2007/169
Ehrmann DA, 2005, NEW ENGL J MED, V352, P1223, DOI 10.1056/NEJMra041536
Ellison PT, 2003, AM J HUM BIOL, V15, P342, DOI 10.1002/ajhb.10152
Escobar-Morreale HF, 2005, ENDOCR REV, V26, P251, DOI 10.1210/er.2004-0004
Fauser BCJM, 2004, HUM REPROD, V19, P41, DOI 10.1093/humrep/deh098
Fisher R., 1930, INHERITANCE HUMAN FE, P188
Fisher RA, 1930, FUNDAMENTAL THEOREM, P22
Flannery T., 1994, The Future Eaters: An Ecological History of the Australasian Lands and People
Fogel R. W., 2004, The Escape from Hunger and Premature Death, 1700-2100
Franks S, 1997, HUM REPROD, V12, P2641, DOI 10.1093/humrep/12.12.2641
Franks S, 2004, REV ENDOCR METAB DIS, V5, P69, DOI 10.1023/B:REMD.0000016125.05878.96
FRANKS S, 1995, NEW ENGL J MED, V333, P853, DOI 10.1056/NEJM199509283331307
Franks S, 2012, FERTIL STERIL, V97, P2, DOI 10.1016/j.fertnstert.2011.11.029
Gleicher N, 2006, REPROD BIOMED ONLINE, V12, P587, DOI 10.1016/S1472-6483(10)61184-4
Gluckman PD, 2005, TRENDS ECOL EVOL, V20, P527, DOI 10.1016/j.tree.2005.08.001
Glueck CJ, 1999, METABOLISM, V48, P1589, DOI 10.1016/S0026-0495(99)90250-0
Goodarzi MO, 2012, J MED GENET, V49, P90, DOI 10.1136/jmedgenet-2011-100427
Goodarzi MO, 2005, FERTIL STERIL, V84, P766, DOI 10.1016/j.fertnstert.2005.03.051
GOULD SJ, 1985, NAT HIST, V94, P12
Gray RH, 2000, AM J PUBLIC HEALTH, V90, P1452, DOI 10.2105/AJPH.90.9.1452
HAMILTONFAIRLEY D, 1992, BRIT J OBSTET GYNAEC, V99, P128, DOI 10.1111/j.1471-0528.1992.tb14470.x
Helgason A, 2007, NAT GENET, V39, P218, DOI 10.1038/ng1960
HENRY L, 1961, EUGEN QUART, V8, P81, DOI 10.1080/19485565.1961.9987465
Hickey M, 2009, J CLIN ENDOCR METAB, V94, P3714, DOI 10.1210/jc.2009-0544
Hodge AM, 1996, METABOLISM, V45, P627, DOI 10.1016/S0026-0495(96)90035-9
HOLLIDAY R, 1989, BIOESSAYS, V10, P125, DOI 10.1002/bies.950100408
HOLTE J, 1994, J CLIN ENDOCR METAB, V78, P1052, DOI 10.1210/jc.78.5.1052
HOMBURG R, 1988, BMJ-BRIT MED J, V297, P1024, DOI 10.1136/bmj.297.6655.1024
Hudecova M, 2009, HUM REPROD, V24, P1176, DOI 10.1093/humrep/den482
Jakubowicz DJ, 2004, J CLIN ENDOCR METAB, V89, P833, DOI 10.1210/jc.2003-030975
Jenike MR, 2001, BIOSOCIAL S, V13, P205
Kirchengast S., 2005, ACTA MED LITUANICA, V12, P22

Koivunen R, 2008, HUM REPROD, V23, P2134, DOI 10.1093/humrep/den136
Kumarapeli V, 2008, AM J EPIDEMIOL, V168, P321, DOI 10.1093/aje/kwn137
LAM DA, 1994, ANN NY ACAD SCI, V709, P9, DOI 10.1111/j.1749-6632.1994.tb30385.x
Larsen CS., 2000, CAMBRIDGE WORLD HIST, V1, P13
Lashen H, 2004, HUM REPROD, V19, P1644, DOI 10.1093/humrep/deh277
Lawson D.W., 2012, P R SOC B
Leonard WR, 2002, AM J HUM BIOL, V14, P547, DOI 10.1002/ajhb.10068
Lindholm Å, 2008, INT J GYNECOL OBSTET, V102, P39, DOI 10.1016/j.ijgo.2008.01.023
Lotka AJ, 1922, P NATL ACAD SCI USA, V8, P151, DOI 10.1073/pnas.8.6.151
Lotka AJ, 1922, P NATL ACAD SCI USA, V8, P147, DOI 10.1073/pnas.8.6.147
March WA, 2010, HUM REPROD, V25, P544, DOI 10.1093/humrep/dep399
McMichael A.J., 2001, Human frontiers, environments and disease: Past patterns, uncertain futures
Mellembakken JR, 2011, HUM REPROD, V26, P2499, DOI 10.1093/humrep/der214
Metwally M, 2008, FERTIL STERIL, V90, P714, DOI 10.1016/j.fertnstert.2007.07.1290
Moore SE, 2001, DIABETIC MED, V18, P646, DOI 10.1046/j.1464-5491.2001.00565.x
Moran C, 2010, GYNECOL OBSTET INVES, V69, P274, DOI 10.1159/000277640
Neel JV, 1999, NUTR REV, V57, pS2, DOI 10.1111/j.1753-4887.1999.tb01782.x
NEEL JV, 1962, AM J HUM GENET, V14, P353
Nesse RM, 2011, EVOL APPL, V4, P264, DOI 10.1111/j.1752-4571.2010.00181.x
Nikolaou D, 2004, HUM REPROD, V19, P2175, DOI 10.1093/humrep/deh419
ODEA K, 1988, DIABETES CARE, V11, P23, DOI 10.2337/diacare.11.1.23
Plomin R, 2009, NAT REV GENET, V10, P872, DOI 10.1038/nrg2670
Porte D, 2006, DIABETES, V55, pS155, DOI 10.2337/db06-S019
Prentice AM, 2005, P NUTR SOC, V64, P153, DOI 10.1079/PNS2005421
PRENTICE AM, 1994, P NUTR SOC, V53, P509, DOI 10.1079/PNS19940061
Pritchard JK, 2010, NAT REV GENET, V11, P665, DOI 10.1038/nrg2880
REGAN L, 1989, BMJ-BRIT MED J, V299, P541, DOI 10.1136/bmj.299.6698.541
Rich-Edwards JW, 2002, EPIDEMIOLOGY, V13, P184, DOI 10.1097/00001648-200203000-00013
ROBINSON S, 1992, CLIN ENDOCRINOL, V36, P537, DOI 10.1111/j.1365-2265.1992.tb02262.x
ROBINSON S, 1995, NATURE, V375, P640, DOI 10.1038/375640a0
Rodin DA, 1998, CLIN ENDOCRINOL, V49, P91, DOI 10.1046/j.1365-2265.1998.00492.x
Sadrzadeh S, 2003, HUM REPROD, V18, P2225, DOI 10.1093/humrep/deg409
SAGLE M, 1988, BMJ-BRIT MED J, V297, P1027, DOI 10.1136/bmj.297.6655.1027
Sam S, 2003, TRENDS ENDOCRIN MET, V14, P365, DOI 10.1016/j.tem.2003.08.002
Sanchón R, 2012, HUM REPROD, V27, P1209, DOI 10.1093/humrep/des028
Schmidhuber J., 2004, SCN News, P13
Shanley DP, 2000, EVOLUTION, V54, P740, DOI 10.1111/j.0014-3820.2000.tb00076.x

Shaw LMA, 2008, BJOG-INT J OBSTET GY, V115, P144, DOI 10.1111/j.1471-0528.2007.01453.x
Simoni M, 2008, HUM REPROD UPDATE, V14, P459, DOI 10.1093/humupd/dmn024
Speakman John R, 2006, Diab Vasc Dis Res, V3, P7, DOI 10.3132/dvdr.2006.010
Stearns S.C., 1992, pi
Stearns SC, 2010, P NATL ACAD SCI USA, V107, P1691, DOI 10.1073/pnas.0914475107
Strauss JF, 2012, J CLIN ENDOCR METAB, V97, P2286, DOI 10.1210/jc.2012-2109
SWINBURN BA, 1991, J CLIN INVEST, V88, P168, DOI 10.1172/JCI115274
Taponen S, 2003, J CLIN ENDOCR METAB, V88, P141, DOI 10.1210/jc.2002-020982
Taponen S, 2004, HUM REPROD, V19, P1083, DOI 10.1093/humrep/deh214
Tinbergen N., 1963, Zeitschrift fuer Tierpsychologie, V20, P410
TRAVIS J, 1988, TRENDS ECOL EVOL, V3, P227, DOI 10.1016/0169-5347(88)90163-2
Urbanek M, 2007, NAT CLIN PRACT ENDOC, V3, P103, DOI 10.1038/ncpendmet0400
Valeggia C, 2004, J BIOSOC SCI, V36, P573, DOI 10.1017/S0021932003006382
Valeggia C, 2009, AM J HUM BIOL, V21, P559, DOI 10.1002/ajhb.20907
van der Spuy ZM, 2004, BEST PRACT RES CL OB, V18, P755, DOI 10.1016/j.bpobgyn.2004.06.001
van der Steeg JW, 2008, HUM REPROD, V23, P324, DOI 10.1093/humrep/dem371
Villuendas G, 2002, J CLIN ENDOCR METAB, V87, P1134, DOI 10.1210/jc.87.3.1134
Vink J., 2009, J CLIN ENDOCR METAB, V91, P2100
Vitzthum VJ, 2009, YEARB PHYS ANTHROPOL, V52, P95, DOI 10.1002/ajpa.21195
Vitzthum VJ, 2001, FOUND HUM B, P179
Voight BF, 2006, PLOS BIOL, V4, P446, DOI 10.1371/journal.pbio.0040072
Wang JX, 2002, OBES RES, V10, P551, DOI 10.1038/oby.2002.74
Wells J., 2010, ADIPOSITY HUMAN EVOL
Welt CK, 2012, J CLIN ENDOCR METAB, V97, pE1342, DOI 10.1210/jc.2011-3478
Winter E, 2002, HUM REPROD, V17, P3220, DOI 10.1093/humrep/17.12.3220
Witchel SF, 1997, J CLIN ENDOCR METAB, V82, P2097, DOI 10.1210/jc.82.7.2097
Wrigley E.A., 1981, POPULATION HIST ENGL
Yildiz BO, 2012, HUM REPROD, V27, P3067, DOI 10.1093/humrep/des232
NR 139
TC 55
Z9 57
U1 1
U2 87
PU ELSEVIER IRELAND LTD
PI CLARE
PA ELSEVIER HOUSE, BROOKVALE PLAZA, EAST PARK SHANNON, CO, CLARE, 00000, IRELAND
SN 0303-7207
EI 1872-8057

J9 MOL CELL ENDOCRINOL
JI Mol. Cell. Endocrinol.
PD JUL 5
PY 2013
VL 373
IS 1-2
SI SI
BP 39
EP 50
DI 10.1016/j.mce.2013.01.001
PG 12
WC Cell Biology; Endocrinology & Metabolism
WE Science Citation Index Expanded (SCI-EXPANDED)
SC Cell Biology; Endocrinology & Metabolism
GA 161RP
UT WOS:000320211300006
PM 23352610
DA 2025-05-07
ER

PT J
AU Egeland, J
AF Egeland, Jonathan
TI Mismatch Resistance and the Problem of Evolutionary Novelty
SO BIOLOGICAL THEORY
LA English
DT Article; Early Access
DE Evolutionary mismatch; Evolutionary novelty; Mismatch resistance;
   Modularity; Scientific explanation; Variability selection
ID HUMAN-BEHAVIOR; CRITIQUE; INTELLIGENCE; MODULARITY; FITNESS; MEMORY;
   BRAIN
AB In evolutionary medicine and other related fields, the concept of evolutionary mismatch is used to explain phenomena whereby traits reduce in adaptive value and eventually become maladaptive as the environment changes. This article argues that there is a similar problem of persistent adaptivity-what has been called the problem of evolutionary novelty-and it introduces the concept of mismatch resistance in order to explain phenomena whereby traits retain their adaptive value in novel environments that are radically different from the organisms' environment of evolutionary adaptedness (EEA). The possible role of variability selection in the evolution of mismatch-resistant traits is discussed, and it is suggested that mismatch resistance provides a useful tool for making progress on certain issues related to evolutionary theory, such as the modularity debate, cases of adaptivity outside of organisms' ancestral environment, and the viability of naturalism as an overarching philosophical framework for understanding the natural world.
C1 [Egeland, Jonathan] Univ Agder, Kristiansand, Norway.
   [Egeland, Jonathan] Norwegian Univ Sci & Technol, Trondheim, Norway.
C3 University of Agder; Norwegian University of Science & Technology (NTNU)
RP Egeland, J (autor correspondente), Univ Agder, Kristiansand, Norway.; Egeland, J (autor correspondente), Norwegian Univ Sci & Technol, Trondheim, Norway.
EM jonathan.egeland@uia.no

RI Egeland, Jonathan/AHD-2017-2022
OI Egeland, Jonathan/0000-0001-5522-6777
FU University of Agder
FX No Statement Available
CR Barrett HC, 2012, THEOR PSYCHOL, V22, P683, DOI 10.1177/0959354312455469
Baumard N, 2023, EcoEvoRxiv, DOI [10.32942/X2MS51, DOI 10.32942/X2MS51]
Beilby J., 2002, NATURALISM DEFEATED, V1
Bitterman M.E., 2000, The evolution of cognition, P61
Bjorklund D. F., 2015, The Handbook of Evolutionary Psychology, P828, DOI [10.1002/9780470939376.CH29, DOI 10.1002/9780470939376.CH29]
Bojke L, 2021, HEALTH TECHNOL ASSES, V25, P1, DOI 10.3310/hta25370
Bolhuis JJ, 2001, TRENDS COGN SCI, V5, P426, DOI 10.1016/S1364-6613(00)01753-8
Bolhuis JJ, 2011, PLOS BIOL, V9, DOI 10.1371/journal.pbio.1001109
Bourrat P, 2024, BRIT J PHILOS SCI, V75, P921, DOI 10.1086/716543
Bowlby J., 1982, ATTACHMENT LOSS VOL, V1
Boyer P., 2005, The Handbook of Evolutionary Psychology, P96, DOI DOI 10.1002/9780470939376.CH3
Brady SP, 2019, AM NAT, V194, P495, DOI 10.1086/705020
Brown Gillian R., 2014, Journal of Bioeconomics, V16, P105, DOI 10.1007/s10818-013-9166-4
Burkart JM, 2017, BEHAV BRAIN SCI, V40, DOI 10.1017/S0140525X16000959
Burnham TerenceC., 2005, Analyse Kritik, V27, P113, DOI [DOI 10.1515/AUK-2005-0107, 10.1515/auk-2005-0107]
Buss DM, 2020, EVOL BEHAV SCI, V14, P316, DOI 10.1037/ebs0000210
Chiappe D, 2005, J GEN PSYCHOL, V132, P5, DOI 10.3200/GENP.132.1.5-40
Chiappe D, 2012, THEOR PSYCHOL, V22, P669, DOI 10.1177/0959354311398703
CHURCHLAND PS, 1987, J PHILOS, V84, P544, DOI 10.5840/jphil1987841026
Cofnas N, 2016, BIOL PHILOS, V31, P507, DOI 10.1007/s10539-016-9527-1
Cosmides L., 1992, The adapted mind, P163, DOI 10.1093/oso/9780195060232.003.0004
Dartnell L., 2019, Origins: how the earth shaped human history
Darwin C., 1871, DESCENT MAN, P387
Darwin C., 1859, On The Origin of Species by Means of Natural Selection, or the Preservation of Favoured Races in the Struggle for Life, P502, DOI [DOI 10.5962/BHL.TITLE.82303, 10.5962/bhl.title.82303]
Darwin Francis., 1887, The life and letters of Charles Darwin, including an autobiographical chapter
DAWKINS R, 1979, PROC R SOC SER B-BIO, V205, P489, DOI 10.1098/rspb.1979.0081
Dawkins R., 1996, BLIND WATCHMAKER WHY
Eaton SB, 2002, PREV MED, V34, P109, DOI 10.1006/pmed.2001.0876
EATON SB, 1988, AM J MED, V84, P739, DOI 10.1016/0002-9343(88)90113-1
Edward L., 2018, Encyclopedia of evolutionary psychological science, P1
Egeland J, 2024, New Ideas Psychol
Egeland J, 2023, INTEGR PSYCHOL BEHAV, V57, P390, DOI 10.1007/s12124-022-09736-x
Elimari N, 2020, FRONT PSYCHOL, V11, DOI 10.3389/fpsyg.2020.545632
Fawcett TW, 2013, BEHAV ECOL, V24, P2, DOI 10.1093/beheco/ars085

Fitelson B, 1998, PAC PHILOS QUART, V79, P115, DOI 10.1111/1468-0114.00053
Fodor J A., 1983, The modularity of mind, DOI DOI 10.7551/MITPRESS/4737.001.0001
Fodor JerryA., 2002, Naturalism Defeated?, P30
Foister TIF, 2023, EVOL ANTHROPOL, V32, P373, DOI 10.1002/evan.22005
Geary D., 2007, PSYCHOL PERSPECTIVES, P1
Geary D. C., 2005, ORIGIN MIND EVOLUTIO, DOI [10.1037/10871-000, DOI 10.1037/10871-000]
Geary DC, 2009, FOUNDATIONS IN EVOLUTIONARY COGNITIVE NEUROSCIENCE, P22, DOI 10.1017/CBO9780511626586.002
Geary DC, 2002, PSYCHOL BULL, V128, P667, DOI 10.1037//0033-2909.128.5.667
Gigerenzer G, 1996, PSYCHOL REV, V103, P592, DOI 10.1037/0033-295X.103.3.592
Gigerenzer G, 2011, ANNU REV PSYCHOL, V62, P451, DOI 10.1146/annurev-psych-120709-145346
Gluckman PeterD., 2009, Principles of evolutionary medicine
Godfrey-Smith P, 2002, EVOLUTION OF INTELLIGENCE, P223
GOULD SJ, 1979, PROC R SOC SER B-BIO, V205, P581, DOI 10.1098/rspb.1979.0086
Gregory T. Ryan, 2009, Evolution Education and Outreach, V2, P156
Griffiths PE, 2023, EVOL MED PUBLIC HLTH, V11, P277, DOI 10.1093/emph/eoad023
Hagen EH, 2006, THEOR POPUL BIOL, V69, P339, DOI 10.1016/j.tpb.2005.09.005
HAMILTON WD, 1982, SCIENCE, V218, P384, DOI 10.1126/science.7123238
HAMILTON WD, 1990, P NATL ACAD SCI USA, V87, P3566, DOI 10.1073/pnas.87.9.3566
Haselton M. G., 2015, HDB EVOLUTIONARY PSY, P724, DOI DOI 10.1002/9781119125563.EVPSYCH241
Haselton MG, 2006, PERS SOC PSYCHOL REV, V10, P47, DOI 10.1207/s15327957pspr1001_3
Hawks J, 2007, P NATL ACAD SCI USA, V104, P20753, DOI 10.1073/pnas.0707650104
Holyoak KJ, 2021, J COGNITIVE NEUROSCI, V33, P341, DOI 10.1162/jocn_a_01619
Kanazawa S., 2004, Managerial and Decision Economics, V25, P41, DOI [10.1002/mde.1130, DOI 10.1002/MDE.1130]
Kane MJ, 2002, PSYCHON B REV, V9, P637, DOI 10.3758/BF03196323
Kolar CS, 2001, TRENDS ECOL EVOL, V16, P199, DOI 10.1016/S0169-5347(01)02101-2
Kolodny O, 2015, CURR ZOOL, V61, P350, DOI 10.1093/czoolo/61.2.350
Laland KN, 2006, EVOL ANTHROPOL, V15, P95, DOI 10.1002/evan.20093
Lefebvre Louis, 2003, P39
Loulergue L, 2008, NATURE, V453, P383, DOI 10.1038/nature06950
Maslin MA, 2014, QUATERNARY SCI REV, V101, P1, DOI 10.1016/j.quascirev.2014.06.012
Mayr E., 1942, P1
Mercader J, 2021, NAT COMMUN, V12, DOI 10.1038/s41467-020-20176-2
Miller G, 2000, ANN NY ACAD SCI, V907, P62
Miller G, The nature of intelligence: Novartis Foundation symposium 233, V233, P260
Morris R, 2020, SYNTHESE, V197, P4021, DOI 10.1007/s11229-018-01915-x

Nesse RM, 1998, SCI AM, V279, P86, DOI 10.1038/scientificamerican1198-86
Pietraszewski D, 2022, PERSPECT PSYCHOL SCI, V17, P465, DOI 10.1177/1745691621997113
Plantinga A., 1993, Warrant and Proper Function, DOI [10.1093/0195078640.001.0001, DOI 10.1093/0195078640.001.0001]
Potts R, 1999, EVOL ANTHROPOL, V7, P81, DOI 10.1002/(SICI)1520-6505(1998)7:3<81::AID-EVAN3>3.0.CO;2-A
Potts R, 2013, QUATERNARY SCI REV, V73, P1, DOI 10.1016/j.quascirev.2013.04.003
Reader SM, 2011, PHILOS T R SOC B, V366, P1017, DOI 10.1098/rstb.2010.0342
Reichard SH, 1997, CONSERV BIOL, V11, P193, DOI 10.1046/j.1523-1739.1997.95473.x
Richerson Peter J., 2000, P329
Roy DS, 2022, NAT COMMUN, V13, DOI 10.1038/s41467-022-29384-4
Shackelford, 2018, ENCY PERSONALITY IND, P1, DOI DOI 10.1007/978-3-319-28099-81627-1
SMITH JM, 1973, NATURE, V246, P15, DOI 10.1038/246015a0
Sterelny K, 2012, JEAN NICOD LECT, P1
Sterelny K., 2003, THOUGHT HOSTILE WORL
Symons D., 1992, ADAPTED MIND, P137, DOI DOI 10.1093/OSO/9780195060232.003.0003
Todd PM., 2015, The handbook of evolutionary psychology, P776, DOI [10.1002/9780470939376.ch27, DOI 10.1002/9780470939376.CH27]
Tomasello M., 2022, The evolution of agency: Behavioral organization from lizards to humans, DOI [10.7551/mitpress/14238.001.0001, DOI 10.7551/MITPRESS/14238.001.0001]
TOOBY J, 1990, J PERS, V58, P17, DOI 10.1111/j.1467-6494.1990.tb00907.x
Tooby J., 1992, ADAPTED MIND, P19, DOI DOI 10.4324/9781410608994
Tooby J., 1995, Mapping the evolved functional organization of mind and brain, The cognitive neurosciences, (11851197)
Van Valen L., 1973, Evol. Theory, V1, P1
Villena D, 2023, INT STUD PHILOS SCI, V36, P317, DOI 10.1080/02698595.2023.2277999
Vrba E.S, 1988, Late Pliocene Climatic Events and Hominid Evolution
Wallace A.R., 1869, Lond. Q. Rev., P187
Wang ZJ, 2022, BEHAV SCI-BASEL, V12, DOI 10.3390/bs12070243
Whitman D. W., 2009, Phenotypic plasticity of insects: mechanisms and consequences, P1
WILLIAMS GC, 1991, Q REV BIOL, V66, P1, DOI 10.1086/417048
Williams GC., 1989, A sociobiological expansion of evolution and ethics, evolution and ethics, (179214)
NR 96
TC 2
Z9 2
U1 4
U2 6
PU SPRINGERNATURE
PI LONDON
PA CAMPUS, 4 CRINAN ST, LONDON, N1 9XW, ENGLAND
SN 1555-5542
EI 1555-5550

J9 BIOL THEORY
JI Biol. Theory
PD 2024 JUL 8
PY 2024
DI 10.1007/s13752-024-00468-2
EA JUL 2024
PG 13
WC Biology; History & Philosophy Of Science
WE Emerging Sources Citation Index (ESCI)
SC Life Sciences & Biomedicine - Other Topics; History & Philosophy of
   Science
GA XW1Z8
UT WOS:001264636100001
OA hybrid
DA 2025-05-07
ER

PT J
AU Ellison, PT
AF Ellison, PT
TI Evolutionary perspectives on the fetal origins hypothesis
SO AMERICAN JOURNAL OF HUMAN BIOLOGY
LA English
DT Article; Proceedings Paper
CT 29th Annual Meeting of the Human-Biology-Association
CY APR 14-15, 2004
CL Tampa, FL
SP Human Biol Assoc
ID HEART-DISEASE
AB The fetal origins hypothesis, or Barker hypothesis, is both stimulating and challenging for evolutionary human biologists. While evidence of a correlation between conditions around the time of birth and later health outcomes has been presented before, the more recent evidence of a connection between fetal growth and chronic disease risk later in life has attracted considerable attention among epidemiologists and human biologists. Several themes that are fundamental to human biology emerge from an engagement with the fetal origins hypothesis. Among them are the tension between concepts of pathology, constraint, and adaptation; the importance of a life history perspective that embraces the notion of trade-offs; the question of environmental predictability; and the mechanisms of energy mobilization and allocation. Bringing the insights of evolutionary biology to bear on the fetal origins hypothesis illustrates the value of the field now known as evolutionary medicine.
(C) 2004 Wiley-Liss, Inc.
C1 Harvard Univ, Dept Anthropol, Cambridge, MA 02138 USA.
C3 Harvard University
RP Harvard Univ, Dept Anthropol, 11 Divin Ave, Cambridge, MA 02138 USA.
EM AJHB@fas.harvard.edu
CR Barker D.J. P., 1994, MOTHERS BABIES DIS L
   BARKER DJP, 1995, BRIT MED J, V311, P171, DOI 10.1136/bmj.311.6998.171
   Ellison P., 2002, Human Growth and Development, P65
   Ellison P. T., 2001, FERTILE GROUND
   ELLISON PT, 1981, HUM BIOL, V53, P635

   ELLISON PT, 1990, AM ANTHROPOL, V92, P933, DOI
10.1525/aa.1990.92.4.02a00050
   Ellison PT, 2003, FERTIL STERIL, V80, P1279, DOI 10.1016/S0015-0282(03)02158-7
   FORSDAHL A, 1977, BRIT J PREV SOC MED, V31, P91
NR 8
TC 52
Z9 71
U1 0
U2 10
PU WILEY
PI HOBOKEN
PA 111 RIVER ST, HOBOKEN 07030-5774, NJ USA
SN 1042-0533
EI 1520-6300
J9 AM J HUM BIOL
JI Am. J. Hum. Biol.
PD JAN-FEB
PY 2005
VL 17
IS 1
BP 113
EP 118
DI 10.1002/ajhb.20097
PG 6
WC Anthropology; Biology
WE Science Citation Index Expanded (SCI-EXPANDED); Social Science Citation Index (SSCI); Conference Proceedings Citation Index - Science (CPCI-S)
SC Anthropology; Life Sciences & Biomedicine - Other Topics
GA 884SD
UT WOS:000226105500010
PM 15612045
DA 2025-05-07
ER

PT J
AU García-Diez, M
   Van Kerrebroeck, P
   Angulo, JC
AF Garcia-Diez, Marcos
   Van Kerrebroeck, Philip
   Angulo, Javier C.
TI Bipedalism and pelvic floor disorders, an evolutionary medical approach
SO CONTINENCE REPORTS
LA English
DT Article
DE Pelvic floor; Pelvic organs prolapse; Dystocia; Encephalization; Human evolution
ID MIDDLE PLEISTOCENE; LOS HUESOS; SHAPE; SIMA
AB Evolutionary medicine can help to better understand the basis of pelvic floor disorders. Some evidences of the anthropological literature are reviewed, with emphasis on the paleontological clues and phylogenetic

comparison of cephalo-pelvic relations in Homo and non-human primates, is undertaken to elucidate the origin of pelvic floor disturbances in humans. Labor difficulties inherent to bipedal gait and the encephalization process could have started several million years ago with Australopithecus and appear undeniable since Homo heidelbergensis, the precursor of Neanderthals. The mechanisms involved in modern human delivery with fetal rotation into the birth canal could be exclusive of Homo sapiens. Among pre-bipedal non-human primates only squirrel monkey can suffer severe dystocia and spontaneous pelvic organ prolapse. Better understanding of the evolutionary changes regarding the human pelvis and the pelvic floor could help us to better understand pelvic floor disorders and the interventions to avoid this highly prevalent ailment.
C1 [Garcia-Diez, Marcos] Univ Complutense, Dept Prehist Ancient Hitory & Archaeol, Madrid, Spain.
[Van Kerrebroeck, Philip] Univ Maastricht, Dept Urol, Maastricht, Netherlands.
[Angulo, Javier C.] Univ Europea Madrid, Clin Dept, Madrid, Spain.
C3 Complutense University of Madrid; Maastricht University; European University of Madrid
RP Angulo, JC (autor correspondente), Univ Europea Madrid, Clin Dept, Madrid, Spain.
EM javier.angulo@universidadeuropea.es
CR AKSEL S, 1983, LAB ANIM SCI, V33, P165
Arsuaga JL, 1999, NATURE, V399, P255, DOI 10.1038/20430
Benton ML, 2021, NAT REV GENET, V22, P269, DOI 10.1038/s41576-020-00305-9
Betti L, 2018, P ROY SOC B-BIOL SCI, V285, DOI 10.1098/rspb.2018.1807
Bonmatí A, 2010, P NATL ACAD SCI USA, V107, P18386, DOI 10.1073/pnas.1012131107
Carroll SP, 2014, SCIENCE, V346, P313, DOI 10.1126/science.1245993
Dixson BJW, 2022, ARCH SEX BEHAV, V51, P49, DOI 10.1007/s10508-021-01946-5
Fischer B, 2015, P NATL ACAD SCI USA, V112, P5655, DOI 10.1073/pnas.1420325112
Gómez-Robles A, 2024, NAT ECOL EVOL, V8, DOI 10.1038/s41559-023-02253-z
Gruss LT, 2015, PHILOS T R SOC B, V370, DOI 10.1098/rstb.2014.0063
Lewis JE, 2016, PHILOS T R SOC B, V371, DOI 10.1098/rstb.2015.0233
Arsuaga JL, 2015, P NATL ACAD SCI USA, V112, P11524, DOI 10.1073/pnas.1514828112
Merlo LMF, 2006, NAT REV CANCER, V6, P924, DOI 10.1038/nrc2013
da Cunha MGMCM, 2021, INT UROGYNECOL J, V32, P1331, DOI 10.1007/s00192-020-04638-1
Nesse RM, 2012, J MOL MED, V90, P509, DOI 10.1007/s00109-012-0889-9
Nesse RM, 2005, Q REV BIOL, V80, P62, DOI 10.1086/431026
Omenn GS, 2010, P NATL ACAD SCI USA, V107, P1702, DOI 10.1073/pnas.0906198106
Pavlicev M, 2020, AM J OBSTET GYNECOL, V222, P3, DOI 10.1016/j.ajog.2019.06.043
da Silva GP, 2021, LAB ANIM-UK, V55, P499, DOI 10.1177/00236772211032506
Potts R, 1998, YEARB PHYS ANTHROPOL, V41, P93
Trevathan W, 2015, PHILOS T R SOC B, V370, DOI 10.1098/rstb.2014.0065
van der Worp HB, 2010, PLOS MED, V7, DOI 10.1371/journal.pmed.1000245

   WASHBURN SL, 1960, SCI AM, V203, P63
   Weaver TD, 2009, P NATL ACAD SCI USA, V106, P8151, DOI
10.1073/pnas.0812554106
   Wells JCK, 2012, AM J PHYS ANTHROPOL, V149, P40, DOI
10.1002/ajpa.22160
NR 25
TC 0
Z9 0
U1 1
U2 1
PU ELSEVIER
PI AMSTERDAM
PA RADARWEG 29, 1043 NX AMSTERDAM, NETHERLANDS
EI 2772-9745
J9 CONTINENCE REP
JI Cont. Rep.
PD SEP
PY 2024
VL 11
AR 100058
DI 10.1016/j.contre.2024.100058
EA JUL 2024
PG 4
WC Urology & Nephrology
WE Emerging Sources Citation Index (ESCI)
SC Urology & Nephrology
GA L1B8X
UT WOS:001348155400001
OA gold
DA 2025-05-07
ER

PT J
AU Hofbauer, KG
   Huppertz, C
AF Hofbauer, KG
   Huppertz, C
TI Pharmacotherapy and evolution
SO TRENDS IN ECOLOGY & EVOLUTION
LA English
DT Review
ID ESSENTIAL-HYPERTENSION; RECOMBINANT LEPTIN; HUMAN OBESITY; THERAPY;
   DISEASE; WEIGHT; PHARMACOGENOMICS; ANGIOTENSIN; PERSPECTIVE
AB The therapeutic effects of a drug are the result of its action on a specific molecular target combined with the counterregulatory responses of the organism that aim to restore the affected parameters to previous values. The regulatory systems that control the steady state have evolved under natural selection, but because natural selection is a slow process and cannot foresee future developments, these systems are not always adjusted optimally to current conditions. Their activation can therefore reduce or prevent the desired effects of a drug and can even produce adverse effects. Recent examples from the treatment of obesity and arterial hypertension provide new insight to the contribution of such ancient counterregulatory responses to the therapeutic effects of modem

pharmacotherapy. This widens the scope of the rapidly growing field of evolutionary medicine, because it demonstrates that evolutionary principles apply not only to the pathogenesis of human diseases, but also to their treatment.
C1 Univ Basel, Biozentrum, Pharmazentrum, CH-4056 Basel, Switzerland.
Novartis Pharma AG, Pharma Res, CH-4002 Basel, Switzerland.
C3 University of Basel; Novartis
RP Univ Basel, Biozentrum, Pharmazentrum, Klingelbergstr 50-70, CH-4056 Basel, Switzerland.
EM karl.hofbauer@unibas.ch
CR Ahima RS, 2000, ANNU REV PHYSIOL, V62, P413, DOI 10.1146/annurev.physiol.62.1.413
Ahima RS, 1996, NATURE, V382, P250, DOI 10.1038/382250a0
Bond RA, 2001, TRENDS PHARMACOL SCI, V22, P273, DOI 10.1016/S0165-6147(00)01711-9
Bristow MR, 2000, CIRCULATION, V101, P558, DOI 10.1161/01.CIR.101.5.558
BRUNNER HR, 1980, AM J MED, V69, P739, DOI 10.1016/0002-9343(80)90444-1
Burnier M, 2001, CIRCULATION, V103, P904, DOI 10.1161/01.CIR.103.6.904
Carretero OA, 2000, CIRCULATION, V101, P446, DOI 10.1161/01.CIR.101.4.446
Carretero OA, 2000, CIRCULATION, V101, P329, DOI 10.1161/01.CIR.101.3.329
Chiesi M, 2001, TRENDS PHARMACOL SCI, V22, P247, DOI 10.1016/S0165-6147(00)01664-3
CODY RJ, 1983, HYPERTENSION, V5, P36
Considine RV, 1996, NEW ENGL J MED, V334, P292, DOI 10.1056/NEJM199602013340503
Eaton SB, 1999, EVOLUTION IN HEALTH AND DISEASE, P251
EATON SB, 1985, NEW ENGL J MED, V312, P283, DOI 10.1056/NEJM198501313120505
Farooqi IS, 1999, NEW ENGL J MED, V341, P879, DOI 10.1056/NEJM199909163411204
Fernández-Real JM, 1999, DIABETOLOGIA, V42, P1367, DOI 10.1007/s001250051451
Franceschi C, 2000, ANN NY ACAD SCI, V908, P244
Francis Gary S., 2001, American Journal of Medicine, V110, p37S
Friedman JM, 1998, NATURE, V395, P763, DOI 10.1038/27376
Gavras H, 2001, HYPERTENSION, V37, P342, DOI 10.1161/01.HYP.37.2.342
Guyton AC., 2000, MED PHYSL
Hardman JG., 2001, GOODMAN GILMANS PHAR, V10th ed
Heymsfield SB, 1999, JAMA-J AM MED ASSOC, V282, P1568, DOI 10.1001/jama.282.16.1568
JAMES GD, 1990, HYPERTENSION PATHOPH, P137
Kalow W, 2001, CLIN PHARMACOL THER, V70, P1, DOI 10.1067/mcp.2001.116714
Kaplan NM, 2000, DRUGS, V59, P21, DOI 10.2165/00003495-200059002-00003
Kopelman PG, 2000, NATURE, V404, P635, DOI 10.1038/35007508
Kurokawa K, 2001, NEPHRON, V89, P369, DOI 10.1159/000046105
LARAGH JH, 1990, HYPERTENSION PATHOPH
Lev-Ran A, 2001, DIABETES-METAB RES, V17, P347, DOI 10.1002/dmrr.230
Lippi G, 2000, QJM-MON J ASSOC PHYS, V93, P75, DOI 10.1093/qjmed/93.2.75

   Lowell BB, 2000, NATURE, V404, P652, DOI 10.1038/35007527
   Mantzoros CS, 1999, ANN INTERN MED, V130, P671, DOI 10.7326/0003-4819-130-8-199904200-00014
   McLeod HL, 2001, ANNU REV PHARMACOL, V41, P101, DOI 10.1146/annurev.pharmtox.41.1.101
   Montague CT, 1997, NATURE, V387, P903, DOI 10.1038/43185
   NEEL JV, 1962, AM J HUM GENET, V14, P353
   ODEA K, 1991, PHILOS T ROY SOC B, V334, P233, DOI 10.1098/rstb.1991.0112
   Reinhardt HW, 2000, NEWS PHYSIOL SCI, V15, P319
   Schwartz MW, 2000, NATURE, V404, P661, DOI 10.1038/35007534
   Spiegelman BM, 2001, CELL, V104, P531, DOI 10.1016/S0092-8674(01)00240-9
   Stearns SC, 1999, EVOLUTION IN HEALTH AND DISEASE, P3
   Stearns SC, 2001, Q REV BIOL, V76, P417, DOI 10.1086/420539
   Stini WA, 1998, COLLEGIUM ANTROPOL, V22, P411
   Strassmann BI, 1999, EVOLUTION IN HEALTH AND DISEASE, P91
   Stryer L., 1995, BIOCHEMISTRY-US, P603
   TARAZI RC, 1970, CIRCULATION, V41, P709, DOI 10.1161/01.CIR.41.4.709
   WENDORF M, 1991, DIABETES, V40, P161, DOI 10.2337/diabetes.40.2.161
NR 46
TC 8
Z9 9
U1 0
U2 5
PU ELSEVIER SCIENCE LONDON
PI LONDON
PA 84 THEOBALDS RD, LONDON WC1X 8RR, ENGLAND
SN 0169-5347
EI 1872-8383
J9 TRENDS ECOL EVOL
JI Trends Ecol. Evol.
PD JUL
PY 2002
VL 17
IS 7
BP 328
EP 334
AR PII S0169-5347(02)02515-6
DI 10.1016/S0169-5347(02)02515-6
PG 7
WC Ecology; Evolutionary Biology; Genetics & Heredity
WE Science Citation Index Expanded (SCI-EXPANDED)
SC Environmental Sciences & Ecology; Evolutionary Biology; Genetics &
   Heredity
GA 564UK
UT WOS:000176331800013
DA 2025-05-07
ER

PT J
AU Braak, H
   Del Tredici, K
AF Braak, Heiko

Del Tredici, Kelly
TI Evolutional Aspects of Alzheimer's Disease Pathogenesis
SO JOURNAL OF ALZHEIMERS DISEASE
LA English
DT Review
DE Alzheimer's disease; AT8 immunocytochemistry; cytoskeleton; dementia; evolutionary medicine; Gallyas silver method; neurofibrillary tangles; pretangles; tau protein; tauopathies
ID MILD COGNITIVE IMPAIRMENT; NEUROFIBRILLARY TANGLES; NATIONAL INSTITUTE;
NEURITIC PLAQUES; CEREBRAL-CORTEX; TAU; MYELINATION; PATHOLOGY; RECOMMENDATIONS; ACCUMULATION
AB Tau lesions (pretangles, neuropil threads, neurofibrillary tangles) that develop in a few types of nerve cells in the brain are essential to the pathogenesis of Alzheimer's disease (AD). The formation of non-argyrophilic pretangles marks the beginning of the pathological process and is of increasing interest because it is temporally closer to the prevailing conditions that induce the pathological process underlying AD in contrast to late-stage disease. Not all of the pretangle material, however, converts into argyrophilic neurofibrillary lesions. The propensity to develop tau lesions may be related to the exceptionally protracted myelination of late developing portions in the human brain.
C1 [Braak, Heiko; Del Tredici, Kelly] Univ Ulm, Ctr Biomed Res, Dept Neurol, D-89081 Ulm, Germany.
C3 Ulm University
RP Braak, H (autor correspondente), Univ Ulm, Ctr Biomed Res, Dept Neurol, Helmholtzstr 8-1, D-89081 Ulm, Germany.
EM heiko.braak@uni-ulm.de
RI Del Tredici, Kelly/AAT-1286-2020
FU German Research Council (Deutsche Forschungsgemeinschaft, DFG) [TR 1000/1-1]
FX This article was made possible by funding from the German Research Council (Deutsche Forschungsgemeinschaft, DFG) grant number TR 1000/1-1.
The authors also wish to express their appreciation to Mr. David Ewert (University of Ulm) for technical expertise and assistance (graphics).
CR Albert MS, 2011, ALZHEIMERS DEMENT, V7, P270, DOI 10.1016/j.jalz.2011.03.008
Alonso AD, 2008, CURR ALZHEIMER RES, V5, P375, DOI 10.2174/156720508785132307
Arnold SE, 1991, CEREB CORTEX, V1, P103, DOI 10.1093/cercor/1.1.103
BANCHER C, 1989, BRAIN RES, V477, P90, DOI 10.1016/0006-8993(89)91396-6
Braak H, 2004, NEUROBIOL AGING, V25, P19, DOI 10.1016/j.neurobiolaging.2003.04.001
Braak H, 1997, NEUROBIOL AGING, V18, P351, DOI 10.1016/S0197-4580(97)00056-0
BRAAK H, 1991, ACTA NEUROPATHOL, V82, P239, DOI 10.1007/BF00308809
Braak H., 1999, Cerebral Cortex: Neurodegenerative and Age-Related Changes in Structure and Function of Cerebral Cortex, P475
Braak H., 1994, NEURODEGENER DIS, P585
Braak H, 2006, ACTA NEUROPATHOL, V112, P389, DOI 10.1007/s00401-006-0127-z

Braak H, 2012, ALZHEIMERS DEMENT, V8, P227, DOI 10.1016/j.jalz.2012.01.011
Braak H, 2011, J NEUROPATH EXP NEUR, V70, P960, DOI 10.1097/NEN.0b013e318232a379
Braak H, 2011, ACTA NEUROPATHOL, V121, P171, DOI 10.1007/s00401-010-0789-4
Braak H, 1991, BRAIN PATHOL, V1, P213, DOI 10.1111/j.1750-3639.1991.tb00661.x
Bulic B, 2010, NEUROPHARMACOLOGY, V59, P276, DOI 10.1016/j.neuropharm.2010.01.016
Clavaguera F, 2009, NAT CELL BIOL, V11, P909, DOI 10.1038/ncb1901
CORK LC, 1988, J NEUROPATH EXP NEUR, V47, P629, DOI 10.1097/00005072-198811000-00006
Dolan D, 2010, BRAIN, V133, P2225, DOI 10.1093/brain/awq141
Elobeid A, 2012, ACTA NEUROPATHOL, V123, P97, DOI 10.1007/s00401-011-0906-z
Esiri M.M., 1997, GREENFIELDS NEUROPAT, V2, P153
Fewster PH, 1991, Dementia, V2, P121, DOI DOI 10.1159/000107187
Fodero-Tavoletti MT, 2011, BRAIN, V134, P1089, DOI 10.1093/brain/awr038
Frost B, 2009, J BIOL CHEM, V284, P12845, DOI 10.1074/jbc.M808759200
GERMAN DC, 1987, NEUROSCIENCE, V21, P305, DOI 10.1016/0306-4522(87)90123-0
Goedert M, 1999, PHILOS T ROY SOC B, V354, P1101, DOI 10.1098/rstb.1999.0466
Goedert M., 1997, MOL GENETIC BASIS NE, P613
Grinberg LT, 2009, NEUROPATH APPL NEURO, V35, P406, DOI [10.1111/j.1365-2990.2008.00997.x, 10.1111/j.1365-2990.2009.00997.x]
Grudzien A, 2007, NEUROBIOL AGING, V28, P327, DOI 10.1016/j.neurobiolaging.2006.02.007
Guo JL, 2011, J BIOL CHEM, V286, P15317, DOI 10.1074/jbc.M110.209296
Hyman BT, 1997, J NEUROPATH EXP NEUR, V56, P1095, DOI 10.1097/00005072-199710000-00002
Iqbal K, 2009, ACTA NEUROPATHOL, V118, P53, DOI 10.1007/s00401-009-0486-3
Jack CR, 2010, LANCET NEUROL, V9, P119, DOI 10.1016/S1474-4422(09)70299-6
Jensen JR, 2011, J ALZHEIMERS DIS, V26, P147, DOI 10.3233/JAD-2011-0003
Korczyn AD, 2008, ALZHEIMERS DEMENT, V4, P176, DOI 10.1016/j.jalz.2007.11.008
Mandelkow E, 2007, BRAIN PATHOL, V17, P83, DOI 10.1111/j.1750-3639.2007.00053.x
Markesbery WR, 2006, ARCH NEUROL-CHICAGO, V63, P38, DOI 10.1001/archneur.63.1.38
Mesulam M, 2004, ANN NEUROL, V55, P815, DOI 10.1002/ana.20100
Molnar FJ, 2009, J AM GERIATR SOC, V57, P536, DOI 10.1111/j.1532-5415.2008.02122.x
Montine TJ, 2012, ACTA NEUROPATHOL, V123, P1, DOI 10.1007/s00401-011-0910-3
Nelson PT, 2007, J NEUROPATH EXP NEUR, V66, P1136, DOI 10.1097/nen.0b013e31815c5efb
Nelson PT, 2011, ACTA NEUROPATHOL, V121, P571, DOI 10.1007/s00401-011-0826-y

NELSON PT, 1994, NEUROSCI LETT, V170, P187, DOI 10.1016/0304-3940(94)90270-4
Pimplikar SW, 2009, INT J BIOCHEM CELL B, V41, P1261, DOI 10.1016/j.biocel.2008.12.015
Sabbagh MN, 2010, CURR ALZHEIMER RES, V7, P280, DOI 10.2174/156720510791162340
Sara SJ, 2009, NAT REV NEUROSCI, V10, P211, DOI 10.1038/nrn2573
Schönheit B, 2004, NEUROBIOL AGING, V25, P697, DOI 10.1016/j.neurobioalging.2003.09.009
Schultz C, 2000, J NEUROPATH EXP NEUR, V59, P39, DOI 10.1093/jnen/59.1.39
TROJANOWSKI JQ, 1995, NEUROBIOL AGING, V16, P335, DOI 10.1016/0197-4580(94)00176-2
Ullén F, 2009, NEURON GLIA BIOL, V5, P28, DOI 10.1017/S1740925X09990330
VANDERKNAAP MS, 1991, DEV MED CHILD NEUROL, V33, P849
Wollen KA, 2010, ALTERN MED REV, V15, P223
NR 51
TC 40
Z9 42
U1 0
U2 35
PU IOS PRESS
PI AMSTERDAM
PA NIEUWE HEMWEG 6B, 1013 BG AMSTERDAM, NETHERLANDS
SN 1387-2877
EI 1875-8908
J9 J ALZHEIMERS DIS
JI J. Alzheimers Dis.
PY 2013
VL 33
SU 1
SI SI
BP S155
EP S161
DI 10.3233/JAD-2012-129029
PG 7
WC Neurosciences
WE Science Citation Index Expanded (SCI-EXPANDED)
SC Neurosciences & Neurology
GA 083FH
UT WOS:000314447600015
PM 22699850
DA 2025-05-07
ER

PT J
AU Meers, MB
Demers, NE
Hewett, A
Sorrelle, D
AF Meers, Mason B.
Demers, Nora Egan
Hewett, Audra

Sorrelle, Dakota
TI Phylogeny can inform animal model development for both inherited and induced conditions: Duchenne Muscular Dystrophy (DMD) and Fetal Alcohol Spectrum Disorders (FASD)
SO IDEAS IN ECOLOGY AND EVOLUTION
LA English
DT Article
DE phylogency; model animals; evolutionary medicine; Duchenne Muscular Dystrophy (DMD); Fetal Alcohol Spectrum Disorders (FASD); Fetal Alcohol Syndrome (FAS)
ID CRANIAL NEURAL CREST; ETHANOL EXPOSURE; SKELETAL-MUSCLE; CELL-DEATH; MOUSE; GENE; MDX; MYOPATHY; ZEBRAFISH; HUMANS
AB The use of animal models in research on human and veterinary diseases and disorders is retracting, though it is likely to remain critical for decades. In light of increasing regulation and expectations of judicious use of animal subjects, we examine the idea that the use of animal models can be guided by phylogenetic relationships and modern evolutionary and cladistic analyses. Given that inherited disorders, and indeed, even the developmental and physiological responses to non inherited conditions, are subject to evolutionary forces, it follows that the observed differences in model organisms are the products of evolutionary divergence. Understanding that divergence has the potential to elucidate which taxa are most likely to exhibit any given symptom or manifest a reaction in a broadly predictable fashion. We examine two case studies, one the inherited disorder Duchenne Muscular Dystrophy, and the other an entirely environmentally induced problem, Fetal Alcohol Spectrum Disorder, or Fetal Alcohol Syndrome. Both case studies reveal symptoms are largely congruent with phylogeny, suggesting relatively conservative evolution of developmental pathways. It follows that it is possible to characterize the manifestation of symptoms or dysmorphologies to broad phylogenetic groups. These data can then be used to inform research into possible treatments based on molecular genetic techniques sourced from unaffected taxa or even provide an evolutionary rationale for maximizing ethical decisions in the use and development of animal models in biomedical research. We argue that the technique should become standard practice in the development of animal models.
C1 [Meers, Mason B.; Hewett, Audra; Sorrelle, Dakota] Univ Tampa, Coll Nat & Hlth Sci, Dept Biol, 401 W Kennedy Blvd, Tampa, FL 33606 USA.
[Demers, Nora Egan] Florida Gulf Coast Univ, Coll Arts & Sci, Dept Biol Sci, 10501 FGCU Blvd, Ft Myers, FL 33965 USA.
[Hewett, Audra] Next Generat Chiropract, 8390 Champ Gate Blvd,Suite 315, Champions Gate, FL 33896 USA.
C3 University of Tampa; State University System of Florida; Florida Gulf Coast University
RP Meers, MB (autor correspondente), Univ Tampa, Coll Nat & Hlth Sci, Dept Biol, 401 W Kennedy Blvd, Tampa, FL 33606 USA.
EM mmeers@ut.edu; ndemers@fgcu.edu; drahewett292@gmail.com; dakota.sorrelle@spartans.ut.edu
CR ABEL EL, 1995, NEUROTOXICOL TERATOL, V17, P445, DOI 10.1016/0892-0362(95)98055-6
ABEL EL, 1995, NEUROTOXICOL TERATOL, V17, P437, DOI 10.1016/0892-0362(95)00005-C

Ahlgren SC, 2002, P NATL ACAD SCI USA, V99, P10476, DOI 10.1073/pnas.162356199
ALLEN ER, 1979, VIRCHOWS ARCH B, V31, P243, DOI 10.1007/BF02889941
ANDERSON JE, 1987, ANAT RECORD, V219, P243, DOI 10.1002/ar.1092190305
[Anonymous], 1991, COMP METHOD EVOLUTIO
[Anonymous], 2019, Mesquite: a modular system for evolutionary analysis. Ver 3.61 software
ARAHATA K, 1993, P JPN ACAD B-PHYS, V69, P259, DOI 10.2183/pjab.69.259
Astley SJ, 2000, ALCOHOL ALCOHOLISM, V35, P509, DOI 10.1093/alcalc/35.5.509
Astley SJ, 2000, ALCOHOL ALCOHOLISM, V35, P400, DOI 10.1093/alcalc/35.4.400
Astley SJ, 1999, TERATOLOGY, V59, P163, DOI 10.1002/(SICI)1096-9926(199903)59:3<163::AID-TERA8>3.0.CO;2-8
Astley SJ, 2011, J POPUL THER CLIN PH, V18, pE231
BARNARD EA, 1982, J PHYSIOL-LONDON, V331, P333, DOI 10.1113/jphysiol.1982.sp014375
Bassett D, 2004, CLIN EXP PHARMACOL P, V31, P537, DOI 10.1111/j.1440-1681.2004.04030.x
Becker HC, 1996, PHARMACOL BIOCHEM BE, V55, P501, DOI 10.1016/S0091-3057(96)00255-9
Becker SRB, 1998, ALCOHOL ALCOHOLISM, V33, P457, DOI 10.1093/alcalc/33.5.457
Bilotta J, 2004, NEUROTOXICOL TERATOL, V26, P737, DOI 10.1016/j.ntt.2004.06.011
BRIDGES LR, 1986, J NEUROL SCI, V72, P147, DOI 10.1016/0022-510X(86)90003-1
BROCKDORFF N, 1987, NATURE, V328, P166, DOI 10.1038/328166a0
BULFIELD G, 1984, P NATL ACAD SCI-BIOL, V81, P1189, DOI 10.1073/pnas.81.4.1189
CARPENTER JL, 1989, AM J PATHOL, V135, P909
CARTWRIGHT MM, 1995, ALCOHOL CLIN EXP RES, V19, P378, DOI 10.1111/j.1530-0277.1995.tb01519.x
Carvan MJ, 2004, NEUROTOXICOL TERATOL, V26, P757, DOI 10.1016/j.ntt.2004.06.016
Chiari Y, 2012, BMC BIOL, V10, DOI 10.1186/1741-7007-10-65
Collins CA, 2003, INT J EXP PATHOL, V84, P165, DOI 10.1046/j.1365-2613.2003.00354.x
COOPER BJ, 1988, J HERED, V79, P405, DOI 10.1093/oxfordjournals.jhered.a110543
COULTON GR, 1988, NEUROPATH APPL NEURO, V14, P53, DOI 10.1111/j.1365-2990.1988.tb00866.x
Darwin C., 1871, P423
DOMINGUEZSTEGLICH M, 1990, GENOMICS, V8, P536, DOI 10.1016/0888-7543(90)90041-R
DRISCOLL CD, 1990, NEUROTOXICOL TERATOL, V12, P231, DOI 10.1016/0892-0362(90)90094-S
Eaton SL, 2017, MAMM GENOME, V28, P324, DOI 10.1007/s00335-017-9687-6
Emerson SB, 1998, Q REV BIOL, V73, P141, DOI 10.1086/420182
Emery AEH, 1998, BRIT MED J, V317, P991, DOI 10.1136/bmj.317.7164.991
FARRIS JS, 1970, SYST ZOOL, V19, P83, DOI 10.2307/2412028
Frost H. M., 1976, 1 WORKSHOP BONE MORP, P219
Gibson MAS, 2000, NEUROTOXICOL TERATOL, V22, P183, DOI 10.1016/S0892-0362(99)00074-4

Gieseler K, 1999, NEUROGENETICS, V2, P87, DOI 10.1007/s100480050057
Gil-Mohapel J, 2010, BRAIN RES REV, V64, P283, DOI 10.1016/j.brainresrev.2010.04.011
Goloboff PA, 2018, CLADISTICS, V34, P407, DOI 10.1111/cla.12205
Guerri C, 2001, NEUROTOXICOLOGY, V22, P593, DOI 10.1016/S0161-813X(01)00037-7
HARPER JA, 1964, POULTRY SCI, V43, P1326, DOI 10.3382/ps.0430577
HARPER JA, 1967, J HERED, V58, P189, DOI 10.1093/oxfordjournals.jhered.a107581
Harris SJ, 2000, J ANAT, V197, P477, DOI 10.1046/j.1469-7580.2000.19730477.x
Hegen M, 2008, ANN RHEUM DIS, V67, P1505, DOI 10.1136/ard.2007.076430
HEGREBERG GA, 1975, J HERED, V66, P63, DOI 10.1093/oxfordjournals.jhered.a108583
HEGREBERG GA, 1974, ARCH PATHOL, V97, P225
HEGREBERG GA, 1976, AM J PATHOL, V85, P233
Herrtage E., 1966, J BRIT VET ASS, V105, P334
HOFFMAN EP, 1987, CELL, V51, P919, DOI 10.1016/0092-8674(87)90579-4
HOMBURGER F, 1966, ANN NY ACAD SCI, V138, P14, DOI 10.1111/j.1749-6632.1966.tb41151.x
Hoogerwaard EM, 1999, LANCET, V353, P2116, DOI 10.1016/S0140-6736(98)10028-4
Johnson GJ, 1999, THROMB HAEMOSTASIS, V81, P835
JOHNSTON MC, 1966, ANAT RECORD, V156, P143, DOI 10.1002/ar.1091560204
KARPATI G, 1988, MUSCLE NERVE, V11, P795, DOI 10.1002/mus.880110802
KLUGE AG, 1989, SYST ZOOL, V38, P7, DOI 10.2307/2992432
Knight Andrew, 2008, Philos Ethics Humanit Med, V3, P16, DOI 10.1186/1747-5341-3-16
KORNEGAY JN, 1990, ADV EXP MED BIOL, V280, P267
KORNEGAY JN, 1988, MUSCLE NERVE, V11, P1056, DOI 10.1002/mus.880111008
Kubotsu SL, 2003, ALCOHOL CLIN EXP RES, V27, P712, DOI [10.1111/j.1530-0277.2003.tb04409.x, 10.1097/01.ALC.0000062650.09584.2A]
LAUDER GV, 1990, ANNU REV ECOL SYST, V21, P317
Lebel C, 2011, NEUROPSYCHOL REV, V21, P102, DOI 10.1007/s11065-011-9163-0
Lloyd TE, 2010, ANN NY ACAD SCI, V1184, pE1, DOI 10.1111/j.1749-6632.2010.05432.x
Lockwood CA, 2004, P NATL ACAD SCI USA, V101, P4356, DOI 10.1073/pnas.0306235101
Loucks E, 2004, NEUROTOXICOL TERATOL, V26, P745, DOI 10.1016/j.ntt.2004.06.017
Maddison WP, 1997, SYST BIOL, V46, P523, DOI 10.2307/2413694
May PA, 2009, DEV DISABIL RES REV, V15, P176, DOI 10.1002/ddrr.68
McClure KD, 2011, DIS MODEL MECH, V4, P335, DOI 10.1242/dmm.006411
MCGAVIN MD, 1969, PATHOL VET, V6, P513, DOI 10.1177/030098586900600604
Medina AE, 2006, J NEUROSCI, V26, P1057, DOI 10.1523/JNEUROSCI.4177-05.2006
Medina AE, 2005, J NEUROPHYSIOL, V93, P1317, DOI 10.1152/jn.00714.2004
Medine AE, 2005, DEV BRAIN RES, V157, P107, DOI 10.1016/j.devbrainres.2005.03.012
Meyer L., 1986, HDB BEHAV TERATOLOGY, P101
Miller MW, 2007, CEREB CORTEX, V17, P2961, DOI 10.1093/cercor/bhm024
Nakamura A, 2011, J BIOMED BIOTECHNOL, DOI 10.1155/2011/184393
Nestler EJ, 2010, NAT NEUROSCI, V13, P1161, DOI 10.1038/nn.2647

O'Leary-Moore SK, 2011, NEUROPSYCHOL REV, V21, P167, DOI 10.1007/s11065-011-9164-z
OSHEA KS, 1979, J ANAT, V128, P65
Oxendine SL, 2006, AQUAT TOXICOL, V80, P396, DOI 10.1016/j.aquatox.2006.10.007
Ozawa E, 1998, MUSCLE NERVE, V21, P421, DOI 10.1002/(SICI)1097-4598(199804)21:4<421::AID-MUS1>3.3.CO;2-W
Parnell SE, 2007, EXP PHYSIOL, V92, P933, DOI 10.1113/expphysiol.2007.038091
Parviainen R, 2020, BONE, V137, DOI 10.1016/j.bone.2020.115462
Peterson KJ, 2008, PHILOS T R SOC B, V363, P1435, DOI 10.1098/rstb.2007.2233
Pound P, 2018, J TRANSL MED, V16, DOI 10.1186/s12967-018-1678-1
Price SA, 2005, BIOL REV, V80, P445, DOI 10.1017/S1464793105006743
Ramadoss J, 2007, ALCOHOL CLIN EXP RES, V31, P1738, DOI 10.1111/j.1530-0277.2007.00477.x
Ramadoss J, 2007, ALCOHOL CLIN EXP RES, V31, P1252, DOI 10.1111/j.1530-0277.2007.00422.x
Ramadoss J, 2006, ALCOHOL, V38, P185, DOI 10.1016/j.alcohol.2006.06.004
Rand M.S., 2008, Sourcebook of models for biomedical research, P9, DOI [DOI 10.1007/978-1-59745-285-4_2, 10.1007/978-1-59745-285-42]
Reeves RH, 2001, TRENDS GENET, V17, P83, DOI 10.1016/S0168-9525(00)02172-7
Reimers MJ, 2004, NEUROTOXICOL TERATOL, V26, P769, DOI 10.1016/j.ntt.2004.06.012
Richtsmeier JT, 2000, DEV DYNAM, V217, P137, DOI 10.1002/(SICI)1097-0177(200002)217:2<137::AID-DVDY1>3.0.CO;2-N
Rifas L, 1997, P NATL ACAD SCI USA, V94, P7549, DOI 10.1073/pnas.94.14.7549
ROBERDS SL, 1993, J BIOL CHEM, V268, P11496
Sansom RS, 2018, BIOL LETTERS, V14, DOI 10.1098/rsbl.2018.0263
SCHMITZ JA, 1975, CAN J COMP MED, V39, P389
Shaffer HB, 1996, AM ZOOL, V36, P24
SHAFFER HB, 1986, SYST ZOOL, V35, P124, DOI 10.2307/2413296
Streissguth A., 1986, ALCOHOL BRAIN DEV, P3
STREISSGUTH AP, 1980, SCIENCE, V209, P353, DOI 10.1126/science.6992275
Strömland K, 2002, ALCOHOL ALCOHOLISM, V37, P2, DOI 10.1093/alcalc/37.1.2
Sulik KK, 2005, EXP BIOL MED, V230, P366, DOI 10.1177/15353702-0323006-04
SULIK KK, 1981, SCIENCE, V214, P936, DOI 10.1126/science.6795717
Taghli-Lamallem O, 2008, AGING CELL, V7, P237, DOI 10.1111/j.1474-9726.2008.00367.x
Tsai KL, 2007, MAMM GENOME, V18, P444, DOI 10.1007/s00335-007-9037-1
Uddin M, 2004, P NATL ACAD SCI USA, V101, P2957, DOI 10.1073/pnas.0308725100
VALENTINE BA, 1992, AM J MED GENET, V42, P352, DOI 10.1002/ajmg.1320420320
Valenzuela CF, 2012, TRENDS NEUROSCI, V35, P284, DOI 10.1016/j.tins.2012.01.006
Wall RJ, 2008, THERIOGENOLOGY, V69, P2, DOI 10.1016/j.theriogenology.2007.09.030
Walmsley GL, 2010, PLOS ONE, V5, DOI 10.1371/journal.pone.0008647

Watchko JF, 2002, J APPL PHYSIOL, V93, P407, DOI 10.1152/japplphysiol.01242.2001
WENTINK GH, 1972, VET PATHOL, V9, P328, DOI 10.1177/030098587200900504
Wiley E.O., 1991, SPECIAL PUBL U KENSA, V19
Wilkinson M, 1995, CLADISTICS, V11, P297, DOI 10.1016/0748-3007(95)90017-9
Willmann R, 2009, NEUROMUSCULAR DISORD, V19, P241, DOI 10.1016/j.nmd.2008.11.015
WITMER LM, 1995, FUNCTIONAL MORPHOLOGY IN VERTEBRATE PALEONTOLOGY, P19
Wright AM, 2014, PLOS ONE, V9, DOI 10.1371/journal.pone.0109210
Yang YL, 2012, CEREB CORTEX, V22, P1170, DOI 10.1093/cercor/bhr193
NR 117
TC 0
Z9 0
U1 2
U2 3
PU QUEENS UNIV
PI KINGSTON
PA C/O LONNIE AARSSEN, ED, DEPT BIOLOGY, KINGSTON, ON K7L 3N6, CANADA
SN 1918-3178
J9 IDEAS ECOL EVOL
JI Ideas Ecol. Evol.
PY 2021
VL 14
IS 1
BP 1
EP 17
DI 10.24908/iee.2021.14.1.n
PG 17
WC Evolutionary Biology
WE Emerging Sources Citation Index (ESCI)
SC Evolutionary Biology
GA UX6AW
UT WOS:000700926800001
OA gold
DA 2025-05-07
ER

PT S
AU Braak, H
Del Tredici, K
AF Braak, Heiko
Del Tredici, Kelly
BE Perry, G
Zhu, X
Smith, MA
Sorensen, A
Avila, J
TI Evolutional Aspects of Alzheimer's Disease Pathogenesis
SO ALZHEIMER'S DISEASE: ADVANCES FOR A NEW CENTURY
SE Advances in Alzheimers Disease
LA English
DT Article; Book Chapter
DE Alzheimer's disease; AT8 immunocytochemistry; cytoskeleton; dementia;

evolutionary medicine; Gallyas silver method; neurofibrillary tangles;
pretangles; tau protein; tauopathies
ID MILD COGNITIVE IMPAIRMENT; NEUROFIBRILLARY TANGLES; NATIONAL
INSTITUTE;
NEURITIC PLAQUES; CEREBRAL-CORTEX; TAU; MYELINATION; PATHOLOGY;
RECOMMENDATIONS; ACCUMULATION
AB Tau lesions (pretangles, neuropil threads, neurofibrillary tangles) that develop in a few types of nerve cells in the brain are essential to the pathogenesis of Alzheimer's disease (AD). The formation of non-argyrophilic pretangles marks the beginning of the pathological process and is of increasing interest because it is temporally closer to the prevailing conditions that induce the pathological process underlying AD in contrast to late-stage disease. Not all of the pretangle material, however, converts into argyrophilic neurofibrillary lesions. The propensity to develop tau lesions may be related to the exceptionally protracted myelination of late developing portions in the human brain.
C1 [Braak, Heiko; Del Tredici, Kelly] Univ Ulm, Dept Neurol, Ctr Biomed Res, D-89081 Ulm, Germany.
C3 Ulm University
RP Braak, H (autor correspondente), Univ Ulm, Dept Neurol, Ctr Biomed Res, Helmholtzstr 8-1, D-89081 Ulm, Germany.
EM heiko.braak@uni-ulm.de
RI Del Tredici, Kelly/AAT-1286-2020
CR Albert MS, 2011, ALZHEIMERS DEMENT, V7, P270, DOI 10.1016/j.jalz.2011.03.008
Alonso AD, 2008, CURR ALZHEIMER RES, V5, P375, DOI 10.2174/156720508785132307
Arnold SE, 1991, CEREB CORTEX, V1, P103, DOI 10.1093/cercor/1.1.103
BANCHER C, 1989, BRAIN RES, V477, P90, DOI 10.1016/0006-8993(89)91396-6
Braak H, 2004, NEUROBIOL AGING, V25, P19, DOI 10.1016/j.neurobiolaging.2003.04.001
Braak H, 1997, NEUROBIOL AGING, V18, P351, DOI 10.1016/S0197-4580(97)00056-0
BRAAK H, 1991, ACTA NEUROPATHOL, V82, P239, DOI 10.1007/BF00308809
Braak H., 1999, Cerebral Cortex: Neurodegenerative and Age-Related Changes in Structure and Function of Cerebral Cortex, P475
Braak H., 1994, NEURODEGENER DIS, P585
Braak H, 2006, ACTA NEUROPATHOL, V112, P389, DOI 10.1007/s00401-006-0127-z
Braak H, 2012, ALZHEIMERS DEMENT, V8, P227, DOI 10.1016/j.jalz.2012.01.011
Braak H, 2011, J NEUROPATH EXP NEUR, V70, P960, DOI 10.1097/NEN.0b013e318232a379
Braak H, 2011, ACTA NEUROPATHOL, V121, P171, DOI 10.1007/s00401-010-0789-4
Braak H, 1991, BRAIN PATHOL, V1, P213, DOI 10.1111/j.1750-3639.1991.tb00661.x
Bulic B, 2010, NEUROPHARMACOLOGY, V59, P276, DOI 10.1016/j.neuropharm.2010.01.016
Clavaguera F, 2009, NAT CELL BIOL, V11, P909, DOI 10.1038/ncb1901
CORK LC, 1988, J NEUROPATH EXP NEUR, V47, P629, DOI 10.1097/00005072-198811000-00006
Dolan D, 2010, BRAIN, V133, P2225, DOI 10.1093/brain/awq141

Elobeid A, 2012, ACTA NEUROPATHOL, V123, P97, DOI 10.1007/s00401-011-0906-z
Esiri M.M., 1997, GREENFIELDS NEUROPAT, V2, P153
Fewster PH, 1991, Dementia, V2, P121, DOI DOI 10.1159/000107187
Fodero-Tavoletti MT, 2011, BRAIN, V134, P1089, DOI 10.1093/brain/awr038
Frost B, 2009, J BIOL CHEM, V284, P12845, DOI 10.1074/jbc.M808759200
GERMAN DC, 1987, NEUROSCIENCE, V21, P305, DOI 10.1016/0306-4522(87)90123-0
Goedert M, 1999, PHILOS T ROY SOC B, V354, P1101, DOI 10.1098/rstb.1999.0466
Goedert M., 1997, MOL GENETIC BASIS NE, P613
Grinberg LT, 2009, NEUROPATH APPL NEURO, V35, P406, DOI [10.1111/j.1365-2990.2008.00997.x, 10.1111/j.1365-2990.2009.00997.x]
Grudzien A, 2007, NEUROBIOL AGING, V28, P327, DOI 10.1016/j.neurobiolaging.2006.02.007
Guo JL, 2011, J BIOL CHEM, V286, P15317, DOI 10.1074/jbc.M110.209296
Hyman BT, 1997, J NEUROPATH EXP NEUR, V56, P1095, DOI 10.1097/00005072-199710000-00002
Iqbal K, 2009, ACTA NEUROPATHOL, V118, P53, DOI 10.1007/s00401-009-0486-3
Jack CR, 2010, LANCET NEUROL, V9, P119, DOI 10.1016/S1474-4422(09)70299-6
Jensen JR, 2011, J ALZHEIMERS DIS, V26, P147, DOI 10.3233/JAD-2011-0003
Korczyn AD, 2008, ALZHEIMERS DEMENT, V4, P176, DOI 10.1016/j.jalz.2007.11.008
Mandelkow E, 2007, BRAIN PATHOL, V17, P83, DOI 10.1111/j.1750-3639.2007.00053.x
Markesbery WR, 2006, ARCH NEUROL-CHICAGO, V63, P38, DOI 10.1001/archneur.63.1.38
Mesulam M, 2004, ANN NEUROL, V55, P815, DOI 10.1002/ana.20100
Molnar FJ, 2009, J AM GERIATR SOC, V57, P536, DOI 10.1111/j.1532-5415.2008.02122.x
Montine TJ, 2012, ACTA NEUROPATHOL, V123, P1, DOI 10.1007/s00401-011-0910-3
Nelson PT, 2007, J NEUROPATH EXP NEUR, V66, P1136, DOI 10.1097/nen.0b013e31815c5efb
Nelson PT, 2011, ACTA NEUROPATHOL, V121, P571, DOI 10.1007/s00401-011-0826-y
NELSON PT, 1994, NEUROSCI LETT, V170, P187, DOI 10.1016/0304-3940(94)90270-4
Pimplikar SW, 2009, INT J BIOCHEM CELL B, V41, P1261, DOI 10.1016/j.biocel.2008.12.015
Sabbagh MN, 2010, CURR ALZHEIMER RES, V7, P280, DOI 10.2174/156720510791162340
Sara SJ, 2009, NAT REV NEUROSCI, V10, P211, DOI 10.1038/nrn2573
Schönheit B, 2004, NEUROBIOL AGING, V25, P697, DOI 10.1016/j.neurobioalging.2003.09.009
Schultz C, 2000, J NEUROPATH EXP NEUR, V59, P39, DOI 10.1093/jnen/59.1.39
TROJANOWSKI JQ, 1995, NEUROBIOL AGING, V16, P335, DOI 10.1016/0197-4580(94)00176-2

   Ullén F, 2009, NEURON GLIA BIOL, V5, P28, DOI
10.1017/S1740925X09990330
   VANDERKNAAP MS, 1991, DEV MED CHILD NEUROL, V33, P849
   Wollen KA, 2010, ALTERN MED REV, V15, P223
NR 51
TC 1
Z9 1
U1 0
U2 9
PU IOS PRESS
PI AMSTERDAM
PA NIEUWE HEMWEG 6B, 1013 BG AMSTERDAM, NETHERLANDS
SN 2210-5727
BN 978-1-61499-154-0; 978-1-61499-153-3
J9 ADV ALZH DIS
PY 2013
VL 3
BP 155
EP 161
DI 10.3233/978-1-61499-154-0-155
PG 7
WC Clinical Neurology; Psychiatry
WE Book Citation Index– Science (BKCI-S)
SC Neurosciences & Neurology; Psychiatry
GA BGV89
UT WOS:000324325100015
DA 2025-05-07
ER

PT J
AU Frankenhuis, WE
   Nettle, D
   Dall, SRX
AF Frankenhuis, Willem E.
   Nettle, Daniel
   Dall, Sasha R. X.
TI A case for environmental statistics of early-life effects
SO PHILOSOPHICAL TRANSACTIONS OF THE ROYAL SOCIETY B-BIOLOGICAL SCIENCES
LA English
DT Article
DE evolution; development; environmental statistics; early-life effects;
   sensitive periods
ID DEVELOPMENTAL PLASTICITY; BAYESIAN MODELS; EVOLUTION; INFORMATION;
CUES;
   HISTORY; COLOR; VARIABILITY; AGE; CONSEQUENCES
AB There is enduring debate over the question of which early-life effects
are adaptive and which ones are not. Mathematical modelling shows that
early-life effects can be adaptive in environments that have particular
statistical properties, such as reliable cues to current conditions and
high autocorrelation of environmental states. However, few empirical
studies have measured these properties, leading to an impasse. Progress,
therefore, depends on research that quantifies cue reliability and
autocorrelation of environmental parameters in real environments. These
statistics may be different for social and non-social aspects of the

environment. In this paper, we summarize evolutionary models of early-life effects. Then, we discuss empirical data on environmental statistics from a range of disciplines. We highlight cases where data on environmental statistics have been used to test competing explanations of early-life effects. We conclude by providing guidelines for new data collection and reflections on future directions.
This article is part of the theme issue 'Developing differences: early-life effects and evolutionary medicine'.
C1 [Frankenhuis, Willem E.] Radboud Univ Nijmegen, Behav Sci Inst, NL-6500 HE Nijmegen, Netherlands.
[Nettle, Daniel] Newcastle Univ, Ctr Behav & Evolut, Newcastle Upon Tyne NE1 7RU, Tyne & Wear, England.
[Nettle, Daniel] Newcastle Univ, Inst Neurosci, Newcastle Upon Tyne NE1 7RU, Tyne & Wear, England.
[Dall, Sasha R. X.] Univ Exeter, Ctr Ecol & Conservat, Penryn TR10 9FE, England.
C3 Radboud University Nijmegen; Newcastle University - UK; Newcastle University - UK; University of Exeter
RP Frankenhuis, WE (autor correspondente), Radboud Univ Nijmegen, Behav Sci Inst, NL-6500 HE Nijmegen, Netherlands.
EM wfrankenhuis@gmail.com
RI Nettle, Daniel/B-2259-2008; Dall, Sasha/B-2642-2010
OI Frankenhuis, Willem/0000-0002-4628-1712; Nettle, Daniel/0000-0001-9089-2599
FU Netherlands Organization for Scientific Research [016.155.195]; James S.
McDonnell Foundation [220020502]; Jacobs Foundation [2017 1261 02]; European Research Council [AdG 666669 COMSTAR]
FX This research was supported by grants from the Netherlands Organization
for Scientific Research (016.155.195), the James S. McDonnell Foundation
(220020502), and the Jacobs Foundation (2017 1261 02) to W.E.F., and by
a grant from the European Research Council (AdG 666669 COMSTAR) to D.N.
CR Aben J, 2018, METHODS ECOL EVOL, V9, P624, DOI 10.1111/2041-210X.12902
Adolph KE, 2012, PSYCHOL INQ, V23, P244, DOI 10.1080/1047840X.2012.705133
[Anonymous], BIORXIV
Baig U, 2011, J OBES, V2011, DOI 10.1155/2011/861049
Bateson P, 2004, NATURE, V430, P419, DOI 10.1038/nature02725
Bateson P, 2014, J PHYSIOL-LONDON, V592, P2357, DOI 10.1113/jphysiol.2014.271460
Beecher MD, 2005, TRENDS ECOL EVOL, V20, P143, DOI 10.1016/j.tree.2005.01.004
BELSKY J, 1991, CHILD DEV, V62, P647, DOI 10.2307/1131166
Berghänel A, 2016, P ROY SOC B-BIOL SCI, V283, DOI 10.1098/rspb.2016.1304
Boettiger C, 2018, ECOL LETT, V21, P1255, DOI 10.1111/ele.13085
Botero CA, 2015, P NATL ACAD SCI USA, V112, P184, DOI 10.1073/pnas.1408589111
Briga M, 2017, P ROY SOC B-BIOL SCI, V284, DOI 10.1098/rspb.2016.2287

Brumbach BH, 2009, HUM NATURE-INT BIOS, V20, P25, DOI 10.1007/s12110-009-9059-3
Burgess SC, 2014, OIKOS, V123, P769, DOI 10.1111/oik.01235
Burgess SC, 2011, J EXP BIOL, V214, P2329, DOI 10.1242/jeb.054718
Burggren WW, 2015, PHYSIOL BIOCHEM ZOOL, V88, P91, DOI 10.1086/679906
Burton T, 2014, P ROY SOC B-BIOL SCI, V281, DOI 10.1098/rspb.2014.0311
Byrne R.W., 1988, MACHIAVELLIAN INTELL
CHENEY DL, 1977, BEHAV ECOL SOCIOBIOL, V2, P303, DOI 10.1007/BF00299742
Chevin LM, 2015, EVOLUTION, V69, P2767, DOI 10.1111/evo.12755
Clark G., 2014, SON ALSO RISES SURNA
Clark G, 2015, ECON J, V125, P61, DOI 10.1111/ecoj.12165
COHEN D, 1991, THEOR POPUL BIOL, V39, P63, DOI 10.1016/0040-5809(91)90041-D
Costantini D, 2014, BIOL LETTERS, V10, DOI 10.1098/rsbl.2013.1010
Dall SRX, 2015, TRENDS ECOL EVOL, V30, P327, DOI 10.1016/j.tree.2015.04.002
Del Giudice M, 2014, P ROY SOC B-BIOL SCI, V281, DOI 10.1098/rspb.2013.2222
DeWitt TJ, 1998, TRENDS ECOL EVOL, V13, P77, DOI 10.1016/S0169-5347(97)01274-3
Douhard M, 2016, P ROY SOC B-BIOL SCI, V283, DOI 10.1098/rspb.2016.1760
Dunlap AS, 2016, CURR OPIN BEHAV SCI, V12, P73, DOI 10.1016/j.cobeha.2016.09.010
Ellis BJ, 2011, DEV PSYCHOPATHOL, V23, P7, DOI 10.1017/S0954579410000611
English S, 2016, AM NAT, V187, P620, DOI 10.1086/685644
Estes L, 2018, NAT ECOL EVOL, V2, P819, DOI 10.1038/s41559-018-0524-4
Fawcett TW, 2015, FRONT ZOOL, V12, DOI 10.1186/1742-9994-12-S1-S3
Fawcett TW, 2010, P ROY SOC B-BIOL SCI, V277, P1427, DOI 10.1098/rspb.2009.2088
Fischer B, 2014, AM NAT, V183, P108, DOI 10.1086/674008
Fischer B, 2011, OIKOS, V120, P258, DOI 10.1111/j.1600-0706.2010.18642.x
FOTHERINGHAM AS, 1993, INT J GEOGR INF SYST, V7, P3, DOI 10.1080/02693799308901936
Frankenhuis WE, 2019, BEHAV PROCESS, V161, P94, DOI 10.1016/j.beproc.2018.01.008
Frankenhuis WE, 2013, DEVELOPMENTAL SCI, V16, P584, DOI 10.1111/desc.12053
Frankenhuis WE, 2011, P ROY SOC B-BIOL SCI, V278, P3558, DOI 10.1098/rspb.2011.0055
Gabriel W, 2005, AM NAT, V166, P339, DOI 10.1086/432558
Gluckman PD, 2007, AM J HUM BIOL, V19, P1, DOI 10.1002/ajhb.20590
Godoy I, 2016, BEHAV ECOL SOCIOBIOL, V70, P1601, DOI 10.1007/s00265-016-2168-1
Godoy I, 2016, ANIM BEHAV, V116, P139, DOI 10.1016/j.anbehav.2016.03.031
Halley JM, 2004, FLUCT NOISE LETT, V4, pR1, DOI 10.1142/S0219477504001884
Halley JM, 1996, TRENDS ECOL EVOL, V11, P33, DOI 10.1016/0169-5347(96)81067-6

Hanson MA, 2014, PHYSIOL REV, V94, P1027, DOI 10.1152/physrev.00029.2013
Hart B., 1995, Meaningful differences in the everyday experience of young American children
Hayward Adam D., 2013, Evolution Medicine and Public Health, P106, DOI 10.1093/emph/eot007
Hayward AD, 2013, P NATL ACAD SCI USA, V110, P13886, DOI 10.1073/pnas.1301817110
Higginson AD, 2018, P ROY SOC B-BIOL SCI, V285, DOI 10.1098/rspb.2017.2411
Houston A. I., 1999, Models of Adaptive Behaviour: An Approach Based on State
Inchausti P, 2002, EVOL ECOL RES, V4, P1033
Inchausti P, 2001, SCIENCE, V293, P655, DOI 10.1126/science.293.5530.655
Jetz W, 2011, CURR BIOL, V21, P72, DOI 10.1016/j.cub.2010.11.075
Jones JH, 2005, AM J HUM BIOL, V17, P22, DOI 10.1002/ajhb.20099
Kacelnik A, 2012, STRUNGMANN FORUM REP, P21
Kernaléguen L, 2016, OECOLOGIA, V180, P657, DOI 10.1007/s00442-015-3407-2
Kokko H, 1999, P ROY SOC B-BIOL SCI, V266, P571, DOI 10.1098/rspb.1999.0674
Kuijper B, 2014, PLOS COMPUT BIOL, V10, DOI 10.1371/journal.pcbi.1003550
Lea AJ, 2017, EVOL MED PUBLIC HLTH, P162, DOI 10.1093/emph/eox019
Lieberman D, 2007, NATURE, V445, P727, DOI 10.1038/nature05510
MANGEL M, 1990, J THEOR BIOL, V146, P317, DOI 10.1016/S0022-5193(05)80742-8
Mangel M, 1988, DYNAMIC MODELING BEH
Marshall DJ, 2007, OIKOS, V116, P1957, DOI 10.1111/j.2007.0030-1299.16203.x
Marshall DJ, 2015, ECOL LETT, V18, P174, DOI 10.1111/ele.12402
Marshall HH, 2017, ECOL EVOL, V7, P1712, DOI 10.1002/ece3.2747
MCNAMARA J, 1980, J THEOR BIOL, V85, P673, DOI 10.1016/0022-5193(80)90265-9
McNamara JM, 2006, OIKOS, V112, P243, DOI 10.1111/j.0030-1299.2006.14228.x
MCNAMARA JM, 1987, J THEOR BIOL, V125, P385, DOI 10.1016/S0022-5193(87)80209-6
McNamara JM, 2016, ECOL LETT, V19, P1267, DOI 10.1111/ele.12663
McNamara JM, 2009, TRENDS ECOL EVOL, V24, P670, DOI 10.1016/j.tree.2009.05.011
MCPEEK MA, 1992, AM NAT, V140, P1010, DOI 10.1086/285453
Monaghan P, 2008, PHILOS T R SOC B, V363, P1635, DOI 10.1098/rstb.2007.0011
Mulder MB, 2009, SCIENCE, V326, P682, DOI 10.1126/science.1178336
Nettle D, 2017, PEERJ, V5, DOI 10.7717/peerj.3528
Nettle D, 2015, P ROY SOC B-BIOL SCI, V282, P23, DOI 10.1098/rspb.2015.1005
Nettle D, 2013, P ROY SOC B-BIOL SCI, V280, DOI 10.1098/rspb.2013.1343
Panchanathan K, 2016, P ROY SOC B-BIOL SCI, V283, DOI 10.1098/rspb.2015.2439
Pelletier JD, 1997, J CLIMATE, V10, P1331, DOI 10.1175/1520-0442(1997)010<1331:AAMOTN>2.0.CO;2

PROMISLOW DEL, 1990, J ZOOL, V220, P417, DOI 10.1111/j.1469-7998.1990.tb04316.x
Quinlan RJ, 2007, P ROY SOC B-BIOL SCI, V274, P121, DOI 10.1098/rspb.2006.3690
Richerson P., 2005, NOT GENES ALONE CULT
Richerson PJ, 2019, BEHAV PROCESS, V161, P54, DOI 10.1016/j.beproc.2018.02.002
Rickard IJ, 2014, PERSPECT PSYCHOL SCI, V9, P3, DOI 10.1177/1745691613513467
Ripa J, 1996, P ROY SOC B-BIOL SCI, V263, P1751, DOI 10.1098/rspb.1996.0256
Ritchie ME, 1999, NATURE, V400, P557, DOI 10.1038/23010
Ruokolainen L, 2009, TRENDS ECOL EVOL, V24, P555, DOI 10.1016/j.tree.2009.04.009
Rutz C, 2013, METHODS ECOL EVOL, V4, P114, DOI 10.1111/2041-210x.12003
Singh S, 2010, IEEE T AUTON MENT DE, V2, P70, DOI 10.1109/TAMD.2010.2051031
Smith LB, 2018, TRENDS COGN SCI, V22, P325, DOI 10.1016/j.tics.2018.02.004
Smith LB, 2017, FRONT PSYCHOL, V8, DOI 10.3389/fpsyg.2017.02124
Smith LB, 2015, J COGN DEV, V16, P407, DOI 10.1080/15248372.2014.933430
Stamps JA, 2017, ANIM BEHAV, V126, P53, DOI 10.1016/j.anbehav.2017.01.013
Stamps JA, 2016, TRENDS ECOL EVOL, V31, P260, DOI 10.1016/j.tree.2016.01.012
Stamps JA, 2014, AM NAT, V184, P647, DOI 10.1086/678116
STEELE JH, 1985, NATURE, V313, P355, DOI 10.1038/313355a0
Takesian AE, 2013, PROG BRAIN RES, V207, P3, DOI 10.1016/B978-0-444-63327-9.00001-1
Travis JMJ, 2001, ECOL RES, V16, P157, DOI 10.1046/j.1440-1703.2001.00381.x
Trimmer Pete C., 2015, Evolution Medicine and Public Health, P123, DOI 10.1093/emph/eov009
Trimmer PC, 2011, ANIM COGN, V14, P465, DOI 10.1007/s10071-011-0387-4
Uller T, 2013, J EVOLUTION BIOL, V26, P2161, DOI 10.1111/jeb.12212
Utz M, 2014, J THEOR BIOL, V340, P60, DOI 10.1016/j.jtbi.2013.08.038
Vasseur DA, 2004, ECOLOGY, V85, P1146, DOI 10.1890/02-3122
Watanabe YY, 2013, P NATL ACAD SCI USA, V110, P2199, DOI 10.1073/pnas.1216244110
Weisleder A, 2013, PSYCHOL SCI, V24, P2143, DOI 10.1177/0956797613488145
Wells JCK., 2017, The Arc of Life: Evolution and Health Across the Life Course, P21
Wells JCK, 2007, TRENDS ENDOCRIN MET, V18, P331, DOI 10.1016/j.tem.2007.07.006
Whitehead H, 2009, EVOL HUM BEHAV, V30, P261, DOI 10.1016/j.evolhumbehav.2009.02.003
Yoda K, 2011, PLOS ONE, V6, DOI 10.1371/journal.pone.0019602
Zeanah CH, 2011, MONOGR SOC RES CHILD, V76, P147, DOI 10.1111/j.1540-5834.2011.00631.x
Zollman KJS, 2013, P ROY SOC B-BIOL SCI, V280, DOI 10.1098/rspb.2012.1878
NR 112

TC 55
Z9 58
U1 0
U2 21
PU ROYAL SOC
PI LONDON
PA 6-9 CARLTON HOUSE TERRACE, LONDON SW1Y 5AG, ENGLAND
SN 0962-8436
EI 1471-2970
J9 PHILOS T R SOC B
JI Philos. Trans. R. Soc. B-Biol. Sci.
PD APR 15
PY 2019
VL 374
IS 1770
AR 20180110
DI 10.1098/rstb.2018.0110
PG 11
WC Biology
WE Science Citation Index Expanded (SCI-EXPANDED); Social Science Citation Index (SSCI)
SC Life Sciences & Biomedicine - Other Topics
GA HN8ZI
UT WOS:000460487000002
PM 30966883
OA hybrid, Green Published, Green Submitted, Green Accepted
DA 2025-05-07
ER

PT J
AU Zhang, B
   Gems, D
AF Zhang, Bruce
   Gems, David
TI Gross ways to live long: Parasitic worms as an anti-inflammaging
   therapy?
SO ELIFE
LA English
DT Review
ID PREVIOUS SCHISTOSOME INFECTION; HELMINTH INFECTION; ANTHELMINTIC
   TREATMENT; SYSTEMIC INFLAMMATION; RHEUMATOID-ARTHRITIS; FILARIAL
   INFECTION; METABOLIC SYNDROME; CONTROLLED TRIAL; GUT MICROBIOTA;
   ASSOCIATION
AB Evolutionary medicine argues that disease can arise because modern conditions do not match those in which we evolved. For example, a decline in exposure to commensal microbes and gastrointestinal helminths in developed countries has been linked to increased prevalence of allergic and autoimmune inflammatory disorders (the hygiene hypothesis). Accordingly, probiotic therapies that restore 'old friend' microbes and helminths have been explored as Darwinian treatments for these disorders. A further possibility is that loss of old friend commensals also increases the sterile, aging-associated inflammation known as inflammaging, which contributes to a range of age-related diseases, including cardiovascular disease, dementia, and cancer. Interestingly,

Crowe et al., 2020 recently reported that treatment with a secreted glycoprotein from a parasitic nematode can protect against murine aging by induction of anti-inflammatory mechanisms. Here, we explore the hypothesis that restorative helminth therapy would have anti-inflammaging effects. Could worm infections provide broad-spectrum protection against age-related disease?
C1 [Zhang, Bruce; Gems, David] UCL, Inst Hlth Ageing, London, England.
[Zhang, Bruce; Gems, David] UCL, Res Dept Genet Evolut & Environm, London, England.
C3 University of London; University College London; University of London; University College London
RP Gems, D (autor correspondente), UCL, Inst Hlth Ageing, London, England.
EM david.gems@ucl.ac.uk
RI Zhang, Bruce/HDM-2569-2022
OI Zhang, Bruce/0000-0003-0956-6142; Gems, David/0000-0002-6653-4676
FU Wellcome Trust [098565/Z/12/Z, 215574/Z/19/Z]; Wellcome Trust [215574/Z/19/Z] Funding Source: Wellcome Trust
FX Wellcome Trust 098565/Z/12/Z David Gemsr Wellcome Trust 215574/Z/19/Z David Gems
CR Akbar AN, 2020, SCIENCE, V369, P256, DOI 10.1126/science.abb0762
Aprahamian TR, 2015, INT J PARASITOL, V45, P203, DOI 10.1016/j.ijpara.2014.12.006
Aravindhan V, 2012, AM J TROP MED HYG, V86, P828, DOI 10.4269/ajtmh.2012.11-0773
Aravindhan V, 2010, PLOS NEGLECT TROP D, V4, DOI 10.1371/journal.pntd.0000707
Austad S.N., 2016, Advances in Geroscience eds, P1, DOI 10.1007/978-3-319-23246-1_1
Blagosklonny MV, 2002, NATURE, V416, P373, DOI 10.1038/416373a
Bourke CD, 2013, J INFECT DIS, V208, P159, DOI 10.1093/infdis/jis524
Buford TW, 2017, MICROBIOME, V5, DOI 10.1186/s40168-017-0296-0
Calabrese V, 2018, FREE RADICAL BIO MED, V115, P80, DOI 10.1016/j.freeradbiomed.2017.10.379
Callejas BE, 2019, INT J CANCER, V145, P3126, DOI 10.1002/ijc.32626
Charabati M, 2020, MULT SCLER J, V26, P1448, DOI 10.1177/1352458519899040
Chen YH, 2013, J CLIN ENDOCR METAB, V98, pE283, DOI 10.1210/jc.2012-2517
Cheng A.M., 2015, Journal of Evolutionary Medicine, V3, P1, DOI DOI 10.4303/JEM/235910
Correale J, 2007, ANN NEUROL, V61, P97, DOI 10.1002/ana.21067
Correale J, 2011, J NEUROIMMUNOL, V233, P6, DOI 10.1016/j.jneuroim.2011.01.002
Croese J, 2015, J ALLERGY CLIN IMMUN, V135, P508, DOI 10.1016/j.jaci.2014.07.022
Crowe J, 2020, PLOS PATHOG, V16, DOI 10.1371/journal.ppat.1008391
de Candia P, 2019, FRONT ENDOCRINOL, V10, DOI 10.3389/fendo.2019.00451
Dixit A, 2017, INT J MOL SCI, V18, DOI 10.3390/ijms18102141
Doenhoff MJ, 2002, PARASITOLOGY, V125, P415, DOI 10.1017/S0031182002002275
Elliott DE, 2017, PARASITE IMMUNOL, V39, DOI 10.1111/pim.12407
Ferrucci L, 2018, NAT REV CARDIOL, V15, P505, DOI 10.1038/s41569-018-0064-2

Franceschi C, 2000, ANN NY ACAD SCI, V908, P244
Franceschi C, 2014, J GERONTOL A-BIOL, V69, pS4, DOI 10.1093/gerona/glu057
Fransen F, 2017, FRONT IMMUNOL, V8, DOI 10.3389/fimmu.2017.01385
Gaze S, 2012, PLOS PATHOG, V8, DOI 10.1371/journal.ppat.1002520
George PJ, 2014, PLOS NEGLECT TROP D, V8, DOI 10.1371/journal.pntd.0003289
Hays R, 2015, DIABETES RES CLIN PR, V107, P355, DOI 10.1016/j.diabres.2015.01.012
Huang X, 2018, MEDICINE, V97, DOI 10.1097/MD.0000000000012087
Kamada N, 2013, NAT REV IMMUNOL, V13, P321, DOI 10.1038/nri3430
Langdon K, 2019, INT IMMUNOPHARMACOL, V66, P366, DOI 10.1016/j.intimp.2018.11.034
Léon-Cabrera S, 2014, INT J BIOL SCI, V10, P948, DOI 10.7150/ijbs.9033
Leonardi GC, 2018, IMMUN AGEING, V15, DOI 10.1186/s12979-017-0112-5
Maizels RM, 2020, ALLERGY, V75, P524, DOI 10.1111/all.13944
Marsland BJ, 2008, EUR J IMMUNOL, V38, P479, DOI 10.1002/eji.200737827
McInnes IB, 2003, J IMMUNOL, V171, P2127, DOI 10.4049/jimmunol.171.4.2127
Mendonça SCL, 2006, ACTA TROP, V99, P102, DOI 10.1016/j.actatropica.2006.06.006
Morimoto M, 2016, J VET MED SCI, V78, P1855, DOI 10.1292/jvms.16-0183
Moyat M, 2019, CLIN TRANSL IMMUNOL, V8, DOI 10.1002/cti2.1089
Mpairwe H, 2014, PEDIAT ALLERG IMM-UK, V25, P481, DOI 10.1111/pai.12251
Mpairwe H, 2011, PEDIAT ALLERG IMM-UK, V22, P305, DOI 10.1111/j.1399-3038.2010.01122.x
Mraz M, 2014, J ENDOCRINOL, V222, pR113, DOI 10.1530/JOE-14-0283
Musella A, 2018, FRONT AGING NEUROSCI, V10, DOI 10.3389/fnagi.2018.00238
Narasimhan PB, 2016, INFECT IMMUN, V84, P2463, DOI 10.1128/IAI.00174-16
Nusse YM, 2018, NATURE, V559, P109, DOI 10.1038/s41586-018-0257-1
Osbourn M, 2017, IMMUNITY, V47, P739, DOI 10.1016/j.immuni.2017.09.015
Pace F, 2018, PHARMACOL RES, V132, P33, DOI 10.1016/j.phrs.2018.04.008
Panda AK, 2013, ARTHRITIS RHEUM-US, V65, P1402, DOI 10.1002/art.37883
Pandey P, 2015, OXF MED CASE REP, P251, DOI 10.1093/omcr/omv027
Pastille E, 2017, PLOS PATHOG, V13, DOI 10.1371/journal.ppat.1006649
Pineda MA, 2012, ARTHRITIS RHEUM-US, V64, P3168, DOI 10.1002/art.34581
Rajamanickam A, 2020, PLOS NEGLECT TROP D, V14, DOI 10.1371/journal.pntd.0008101
Rajamanickam A, 2019, CLIN INFECT DIS, V69, P697, DOI 10.1093/cid/ciy935
Rea IM, 2018, FRONT IMMUNOL, V9, DOI 10.3389/fimmu.2018.00586
Rook GAW, 2004, SPRINGER SEMIN IMMUN, V25, P237, DOI 10.1007/s00281-003-0148-9
Rzepecka J, 2015, J AUTOIMMUN, V60, P59, DOI 10.1016/j.jaut.2015.04.005
Sanai SA, 2016, MULT SCLER J, V22, P717, DOI 10.1177/1352458516634871
Shen SW, 2015, PARASITE IMMUNOL, V37, P333, DOI 10.1111/pim.12187
Smallwood TB, 2017, FRONT IMMUNOL, V8, DOI 10.3389/fimmu.2017.00453
Sonnenberg GF, 2011, NAT IMMUNOL, V12, P383, DOI 10.1038/ni.2025
STRACHAN DP, 1989, BRIT MED J, V299, P1259, DOI 10.1136/bmj.299.6710.1259

Tahapary DL, 2017, CLIN INFECT DIS, V65, P764, DOI 10.1093/cid/cix416
Tchkonia T, 2010, AGING CELL, V9, P667, DOI 10.1111/j.1474-9726.2010.00608.x
Teixeira AL, 2012, CURR GERIATR REP, V1, P111, DOI 10.1007/s13670-012-0010-9
Thevaranjan N, 2017, CELL HOST MICROBE, V21, P455, DOI 10.1016/j.chom.2017.03.002
Tsai CY, 2019, INT J MOL SCI, V20, DOI 10.3390/ijms20163878
TURTON JA, 1976, LANCET, V2, P686
Velasquez-Manoff M., 2012, An epidemic of absence: A new way of understanding allergies and autoimmune diseases
Vennervald BJ, 2009, PARASITE IMMUNOL, V31, P686, DOI 10.1111/j.1365-3024.2009.01163.x
Wammes LJ, 2016, P NATL ACAD SCI USA, V113, P12526, DOI 10.1073/pnas.1604570113
Wolfs IMJ, 2014, FASEB J, V28, P288, DOI 10.1096/fj.13-235911
Wu D, 2011, SCIENCE, V332, P243, DOI 10.1126/science.1201475
Xi HK, 2016, J EXP MED, V213, P189, DOI 10.1084/jem.20150894
Xia SJ, 2016, J IMMUNOL RES, V2016, DOI 10.1155/2016/8426874
Zhang Hui, 2012, Zhongguo Xue Xi Chong Bing Fang Zhi Za Zhi, V24, P654
Zhuang Yong, 2014, Inflammation & Allergy Drug Targets, V13, P153
Zuo L, 2019, INT J MOL SCI, V20, DOI 10.3390/ijms20184472
NR 77
TC 18
Z9 18
U1 1
U2 21
PU eLIFE SCIENCES PUBL LTD
PI CAMBRIDGE
PA SHERATON HOUSE, CASTLE PARK, CAMBRIDGE, CB3 0AX, ENGLAND
SN 2050-084X
J9 ELIFE
JI eLife
PD FEB 2
PY 2021
VL 10
AR e65180
DI 10.7554/eLife.65180
PG 12
WC Biology
WE Science Citation Index Expanded (SCI-EXPANDED)
SC Life Sciences & Biomedicine - Other Topics
GA QH1VB
UT WOS:000618064800001
PM 33526169
OA Green Submitted, Green Published, gold
DA 2025-05-07
ER

PT J
AU Benton, ML
Abraham, A
LaBella, AL
Abbot, P

Rokas, A
Capra, JA
AF Benton, Mary Lauren
Abraham, Abin
LaBella, Abigail L.
Abbot, Patrick
Rokas, Antonis
Capra, John A.
TI The influence of evolutionary history on human health and disease
SO NATURE REVIEWS GENETICS
LA English
DT Review
ID DELETERIOUS MUTATION LOAD; N-GLYCOLYLNEURAMINIC ACID; GENOME-WIDE ASSOCIATION; POLYGENIC RISK SCORES; BODY-MASS INDEX; HLA-C GENES; NATURAL-SELECTION; TRADE-OFFS; NEANDERTHAL ANCESTRY; ALTITUDE ADAPTATION
AB Nearly all genetic variants that influence disease risk have human-specific origins; however, the systems they influence have ancient roots that often trace back to evolutionary events long before the origin of humans. Here, we review how advances in our understanding of the genetic architectures of diseases, recent human evolution and deep evolutionary history can help explain how and why humans in modern environments become ill. Human populations exhibit differences in the prevalence of many common and rare genetic diseases. These differences are largely the result of the diverse environmental, cultural, demographic and genetic histories of modern human populations. Synthesizing our growing knowledge of evolutionary history with genetic medicine, while accounting for environmental and social factors, will help to achieve the promise of personalized genomics and realize the potential hidden in an individual's DNA sequence to guide clinical decisions. In short, precision medicine is fundamentally evolutionary medicine, and integration of evolutionary perspectives into the clinic will support the realization of its full potential.
C1 [Benton, Mary Lauren; Rokas, Antonis; Capra, John A.] Vanderbilt Univ, Sch Med, Dept Biomed Informat, Nashville, TN 37212 USA.
[Benton, Mary Lauren] Baylor Univ, Dept Comp Sci, Waco, TX 76798 USA.
[Abraham, Abin; Rokas, Antonis] Vanderbilt Univ, Vanderbilt Genet Inst, 221 Kirkland Hall, Nashville, TN 37235 USA.
[Abraham, Abin] Vanderbilt Univ, Vanderbilt Univ Med Ctr, 221 Kirkland Hall, Nashville, TN 37235 USA.
[LaBella, Abigail L.; Abbot, Patrick; Rokas, Antonis; Capra, John A.] Vanderbilt Univ, Dept Biol Sci, 221 Kirkland Hall, Nashville, TN 37235 USA.
[Capra, John A.] Univ Calif San Francisco, Bakar Computat Hlth Sci Inst, San Francisco, CA 94143 USA.
[Capra, John A.] Univ Calif San Francisco, Dept Epidemiol & Biostat, San Francisco, CA 94143 USA.
C3 Vanderbilt University; Baylor University; Vanderbilt University; Vanderbilt University; Vanderbilt University; University of California System; University of California San Francisco; University of California System; University of California San Francisco
RP Capra, JA (autor correspondente), Vanderbilt Univ, Sch Med, Dept Biomed Informat, Nashville, TN 37212 USA.; Capra, JA (autor

correspondente), Vanderbilt Univ, Dept Biol Sci, 221 Kirkland Hall, Nashville, TN 37235 USA.; Capra, JA (autor correspondente), Univ Calif San Francisco, Bakar Computat Hlth Sci Inst, San Francisco, CA 94143 USA.; Capra, JA (autor correspondente), Univ Calif San Francisco, Dept Epidemiol & Biostat, San Francisco, CA 94143 USA.
EM tony.capra@ucsf.edu
RI Benton, Mary Lauren/GQZ-3501-2022; Rokas, Antonis/A-9775-2008
OI Rokas, Antonis/0000-0002-7248-6551; Benton, Mary Lauren/0000-0002-5485-1041; Abraham, Abin/0000-0002-9951-2879
FU National Institutes of Health (NIH) [T32LM012412, R35GM127087]; Burroughs Wellcome Fund Preterm Birth Initiative; March of Dimes Prematurity Research Center Ohio Collaborative; American Heart Association; National Institute of General Medical Sciences [R35GM127087] Funding Source: NIH RePORTER
FX The authors thank L. Muglia and members of the Capra, Rokas and Abbot laboratories for helpful discussions. They also thank the National Institutes of Health (NIH) (T32LM012412 to M.L.B. and R35GM127087 to J.A.C.), the Burroughs Wellcome Fund Preterm Birth Initiative (A.R. and J.A.C.), the March of Dimes Prematurity Research Center Ohio Collaborative (P.A., A.R. and J.A.C.) and the American Heart Association (A.A.) for support.
CR Abbot P, 2017, ELIFE, V6, DOI 10.7554/eLife.30994
Abbot P, 2017, CURR BIOL, V27, pR127, DOI 10.1016/j.cub.2016.10.046
Ackermann M, 2007, AGING CELL, V6, P235, DOI 10.1111/j.1474-9726.2007.00281.x
Aktipis CA, 2013, EVOL APPL, V6, P144, DOI 10.1111/eva.12034
Al-Nbaheen M, 2013, STEM CELL REV REP, V9, P32, DOI 10.1007/s12015-012-9365-8
Albuquerque TAF, 2018, BIOL REV, V93, P1715, DOI 10.1111/brv.12415
Alföldi J, 2013, GENOME RES, V23, P1063, DOI 10.1101/gr.157503.113
Andersson DI, 2020, FEMS MICROBIOL REV, V44, P171, DOI 10.1093/femsre/fuaa001
Antonacci F, 2014, NAT GENET, V46, P1293, DOI 10.1038/ng.3120
Rodríguez JA, 2017, NAT ECOL EVOL, V1, DOI 10.1038/s41559-016-0055
ARNOLD AJ, 1982, PALEOBIOLOGY, V8, P113, DOI 10.1017/S0094837300004462
Arora G, 2009, MED HYPOTHESES, V73, P453, DOI 10.1016/j.mehy.2009.03.035
Asgari S, 2020, NATURE, V582, P234, DOI 10.1038/s41586-020-2302-0
Aungst H, 2020, INTEGRATING EVOLUTIONARY BIOLOGY INTO MEDICAL EDUCATION: FOR MATERNAL AND CHILD HEALTHCARE STUDENTS, CLINICIANS, AND SCIENTISTS, P91, DOI 10.1093/oso/9780198814153.003.0006
Azevedo L, 2015, HUM GENOMICS, V9, DOI 10.1186/s40246-015-0043-1
Barber MF, 2017, GENOME BIOL EVOL, V9, P1757, DOI 10.1093/gbe/evx120
Barreiro LB, 2010, NAT REV GENET, V11, P17, DOI 10.1038/nrg2698
Bergmann A, 2010, J CELL MOL MED, V14, P1857, DOI 10.1111/j.1582-4934.2009.00820.x
Bigham AW, 2014, GENE DEV, V28, P2189, DOI 10.1101/gad.250167.114
Bolker JA, 2019, BRAIN BEHAV EVOLUT, V93, P82, DOI 10.1159/000500317
Bowton E, 2014, SCI TRANSL MED, V6, DOI 10.1126/scitranslmed.3008604
Brady SP, 2019, AM NAT, V194, P495, DOI 10.1086/705020
Bush WS, 2016, NAT REV GENET, V17, P129, DOI 10.1038/nrg.2015.36
Byars SG, 2017, PLOS GENET, V13, DOI 10.1371/journal.pgen.1006328

Bycroft C, 2018, NATURE, V562, P203, DOI 10.1038/s41586-018-0579-z
Calvert C, 2014, NEW ENGL J MED, V371, P1075, DOI [10.1056/NEJMra1213566, 10.1056/NEJMc1408436]
Carlson CS, 2013, PLOS BIOL, V11, DOI 10.1371/journal.pbio.1001661
Carroll SP, 2014, SCIENCE, V346, P313, DOI 10.1126/science.1245993
Caulin AF, 2011, TRENDS ECOL EVOL, V26, P175, DOI 10.1016/j.tree.2011.01.002
Chen CY, 2008, MOL PHYLOGENET EVOL, V47, P637, DOI 10.1016/j.ympev.2007.12.026
Chen L, 2020, CELL, V180, P677, DOI 10.1016/j.cell.2020.01.012
Chou HH, 1998, P NATL ACAD SCI USA, V95, P11751, DOI 10.1073/pnas.95.20.11751
Chuong EB, 2016, SCIENCE, V351, P1083, DOI 10.1126/science.aad5497
Clark AG, 2003, COLD SPRING HARB SYM, V68, P471
Cook JP, 2016, EUR J HUM GENET, V24, P1175, DOI 10.1038/ejhg.2016.17
Coppola L, 2019, J TRANSL MED, V17, DOI 10.1186/s12967-019-1922-3
Corbett S, 2018, NAT REV GENET, V19, P419, DOI 10.1038/s41576-018-0012-3
Crawford NG, 2017, SCIENCE, V358, P887, DOI 10.1126/science.aan8433
Crespi B, 2005, TRENDS ECOL EVOL, V20, P545, DOI 10.1016/j.tree.2005.07.007
Crespi Bernard J., 2015, Evolution Medicine and Public Health, P216, DOI 10.1093/emph/eov021
Dannemann M, 2017, AM J HUM GENET, V101, P578, DOI 10.1016/j.ajhg.2017.09.010
Dannemann M, 2016, AM J HUM GENET, V98, P22, DOI 10.1016/j.ajhg.2015.11.015
de Groot NG, 2010, P NATL ACAD SCI USA, V107, P15175, DOI 10.1073/pnas.1009136107
Delser PM, 2013, HUM GENET, V132, P563, DOI 10.1007/s00439-013-1268-5
Deng LQ, 2014, CELL, V159, P1290, DOI 10.1016/j.cell.2014.10.057
Dennis MY, 2016, CURR OPIN GENET DEV, V41, P44, DOI 10.1016/j.gde.2016.08.001
Dennis MY, 2012, CELL, V149, DOI 10.1016/j.cell.2012.03.033
Deschamps M, 2016, AM J HUM GENET, V98, P5, DOI 10.1016/j.ajhg.2015.11.014
Dewey FE, 2016, SCIENCE, V354, DOI 10.1126/science.aaf6814
Di Rienzo A, 2005, TRENDS GENET, V21, P596, DOI 10.1016/j.tig.2005.08.007
Do R, 2015, NAT GENET, V47, P126, DOI 10.1038/ng.3186
Doan RN, 2016, CELL, V167, P341, DOI 10.1016/j.cell.2016.08.071
Domazet-Loso T, 2010, BMC BIOL, V8, DOI 10.1186/1741-7007-8-66
Duncan L, 2019, NAT COMMUN, V10, DOI 10.1038/s41467-019-11112-0
Dunne DW, 2005, NAT REV IMMUNOL, V5, P420, DOI 10.1038/nri1601
Ellison Peter T., 2014, Evolution Medicine and Public Health, P93, DOI 10.1093/emph/eou015
Enard D, 2018, CELL, V175, P360, DOI 10.1016/j.cell.2018.08.034
Enriquez-Navas PM, 2016, SCI TRANSL MED, V8, DOI 10.1126/scitranslmed.aad7842
Enriquez-Navas PM, 2015, CANCER RES, V75, P4675, DOI 10.1158/0008-5472.CAN-15-1337
Erlebacher A, 2013, ANNU REV IMMUNOL, V31, P387, DOI 10.1146/annurev-immunol-032712-100003

Fairlamb AH, 2016, NAT MICROBIOL, V1, DOI [10.1038/NMICROBIOL.2016.92, 10.1038/nmicrobiol.2016.92]
Fan SH, 2016, SCIENCE, V354, P54, DOI 10.1126/science.aaf5098
Fay JC, 2013, APPL TRANSL GENOM, V2, P42, DOI 10.1016/j.atg.2013.08.001
Fernández-Rhodes L, 2017, HUM GENET, V136, P771, DOI 10.1007/s00439-017-1787-6
Fiddes IT, 2018, CELL, V173, P1356, DOI 10.1016/j.cell.2018.03.051
Flajnik MF, 2010, NAT REV GENET, V11, P47, DOI 10.1038/nrg2703
Flatt T, 2018, BMC BIOL, V16, DOI 10.1186/s12915-018-0562-z
Florio M, 2015, SCIENCE, V347, P1465, DOI 10.1126/science.aaa1975
Frank SA, 2011, P NATL ACAD SCI USA, V108, P10886, DOI 10.1073/pnas.1100921108
Fu QM, 2013, CURR BIOL, V23, P553, DOI 10.1016/j.cub.2013.02.044
Fu WQ, 2013, ANNU REV GENOM HUM G, V14, P467, DOI 10.1146/annurev-genom-091212-153509
Fumagalli M, 2015, SCIENCE, V349, P1343, DOI 10.1126/science.aab2319
Futuyma DJ, 2010, EVOLUTION, V64, P1865, DOI 10.1111/j.1558-5646.2010.00960.x
Gatenby RA, 2020, NAT REV CLIN ONCOL, V17, P675, DOI 10.1038/s41571-020-0411-1
Gerlinger M, 2014, ANNU REV GENET, V48, P215, DOI 10.1146/annurev-genet-120213-092314
Gerstung M, 2020, NATURE, V578, P122, DOI 10.1038/s41586-019-1907-7
Gibson G, 2009, NAT REV GENET, V10, P134, DOI 10.1038/nrg2502
Gittelman RM, 2016, CURR BIOL, V26, P3375, DOI 10.1016/j.cub.2016.10.041
Gluckman P, 2006, DEVELOPMENTAL ORIGINS OF HEALTH AND DISEASE, P1, DOI 10.2277/ 0521847435
Gluckman PD, 2006, MOL CELL ENDOCRINOL, V254, P26, DOI 10.1016/j.mce.2006.04.005
Gravel S, 2016, GENETICS, V203, P451, DOI 10.1534/genetics.115.184630
Guernsey MW, 2017, ELIFE, V6, DOI 10.7554/eLife.27450
Guerrier S, 2009, CELL, V138, P990, DOI 10.1016/j.cell.2009.06.047
Hammer A, 2011, J REPROD IMMUNOL, V90, P21, DOI 10.1016/j.jri.2011.05.001
Hammer MF, 2011, P NATL ACAD SCI USA, V108, P15123, DOI 10.1073/pnas.1109300108
Harris K, 2016, GENETICS, V203, P881, DOI 10.1534/genetics.116.186890
He SH, 2017, CELL, V169, P1000, DOI 10.1016/j.cell.2017.05.015
Hibino T, 2006, DEV BIOL, V300, P349, DOI 10.1016/j.ydbio.2006.08.065
Hiby SE, 2004, J EXP MED, V200, P957, DOI 10.1084/jem.20041214
Hiby SE, 2010, J CLIN INVEST, V120, P4102, DOI 10.1172/JCI43998
Hindorff LA, 2018, NAT REV GENET, V19, P175, DOI 10.1038/nrg.2017.89
Hsieh P, 2016, GENOME RES, V26, P291, DOI 10.1101/gr.196634.115
Huerta-Sánchez E, 2014, NATURE, V512, P194, DOI 10.1038/nature13408
Huo DZ, 2017, JAMA ONCOL, V3, P1654, DOI 10.1001/jamaoncol.2017.0595
Irie A, 1998, J BIOL CHEM, V273, P15866, DOI 10.1074/jbc.273.25.15866
Jacqueline C, 2017, EVOL APPL, V10, P215, DOI 10.1111/eva.12444
Jones JDG, 2006, NATURE, V444, P323, DOI 10.1038/nature05286
Juric I, 2016, PLOS GENET, V12, DOI 10.1371/journal.pgen.1006340
Karczewski KJ, 2020, NATURE, V581, P434, DOI 10.1038/s41586-020-2308-7
Katz PS, 2016, CURR BIOL, V26, pR649, DOI 10.1016/j.cub.2016.05.071
Keinan A, 2012, SCIENCE, V336, P740, DOI 10.1126/science.1217283

Kelly Derek E, 2017, Curr Opin Syst Biol, V1, P102, DOI 10.1016/j.coisb.2016.12.018
Kessler MD, 2016, NAT COMMUN, V7, DOI 10.1038/ncomms12521
Khera AV, 2018, NAT GENET, V50, P1219, DOI 10.1038/s41588-018-0183-z
Kiepiela P, 2004, NATURE, V432, P769, DOI 10.1038/nature03113
Kim MS, 2018, GENOME BIOL, V19, DOI 10.1186/s13059-018-1561-7
Kinzler KW, 1997, NATURE, V386, P761, DOI 10.1038/386761a0
Kwiatkowski DP, 2005, AM J HUM GENET, V77, P171, DOI 10.1086/432519
LaBella AL, 2020, NAT COMMUN, V11, DOI 10.1038/s41467-020-17258-6
Lachance J, 2018, CANCER RES, V78, P2432, DOI 10.1158/0008-5472.CAN-17-1550
Lachance J, 2012, CELL, V150, P457, DOI 10.1016/j.cell.2012.07.009
Lachance J, 2010, BMC MED GENOMICS, V3, DOI 10.1186/1755-8794-3-57
Li RW, 2020, NAT REV GENET, V21, P493, DOI 10.1038/s41576-020-0224-1
Li YR, 2014, GENOME MED, V6, DOI 10.1186/s13073-014-0091-5
Lynch M, 2016, GENETICS, V202, P869, DOI 10.1534/genetics.115.180471
Mägi R, 2017, HUM MOL GENET, V26, P3639, DOI 10.1093/hmg/ddx280
Mak IWY, 2014, AM J TRANSL RES, V6, P114
Manrai AK, 2016, NEW ENGL J MED, V375, P655, DOI 10.1056/NEJMsa1507092
Manus MB, 2018, EVOL MED PUBLIC HLTH, P190, DOI 10.1093/emph/eoy023
Marciniak S, 2017, NAT REV GENET, V18, DOI 10.1038/nrg.2017.65
Marinic M, 2020, PLOS GENET, V16, DOI 10.1371/journal.pgen.1008666
Marques-Bonet T, 2009, COLD SH Q B, V74, P355, DOI 10.1101/sqb.2009.74.011
Márquez-Luna C, 2017, GENET EPIDEMIOL, V41, P811, DOI 10.1002/gepi.22083
Martin AR, 2019, NAT GENET, V51, P584, DOI 10.1038/s41588-019-0379-x
Martin AR, 2017, AM J HUM GENET, V100, P635, DOI 10.1016/j.ajhg.2017.03.004
Martin MP, 2007, NAT GENET, V39, P733, DOI 10.1038/ng2035
Martin MJ, 2005, P NATL ACAD SCI USA, V102, P12819, DOI 10.1073/pnas.0503819102
Mathieson I, 2015, NATURE, V528, P499, DOI 10.1038/nature16152
McArthur E, 2020, BIORXIV, DOI [10.1101/2020.06.08.140087, DOI 10.1101/2020.06.08.140087]
McCarty CA, 2011, BMC MED GENOMICS, V4, DOI 10.1186/1755-8794-4-13
McCoy RC, 2017, CELL, V168, P916, DOI 10.1016/j.cell.2017.01.038
McLaren PJ, 2015, P NATL ACAD SCI USA, V112, P14658, DOI 10.1073/pnas.1514867112
Meadows JRS, 2017, NAT REV GENET, V18, P624, DOI 10.1038/nrg.2017.51
Median AG, 2012, TRENDS GENET, V28, P487, DOI 10.1016/j.tig.2012.06.008
Michor F, 2004, NAT REV CANCER, V4, P197, DOI 10.1038/nrc1295
Minster RL, 2016, NAT GENET, V48, P1049, DOI 10.1038/ng.3620
Moffett A, 2016, REPROD BIOMED ONLINE, V33, P763, DOI 10.1016/j.rbmo.2016.08.019
Moon JM, 2019, BIOESSAYS, V41, DOI 10.1002/bies.201900072
Moorjani P, 2016, P NATL ACAD SCI USA, V113, P10607, DOI 10.1073/pnas.1600374113
Mostafavi H, 2020, ELIFE, V9, DOI 10.7554/eLife.48376
Muehlenbachs A, 2008, P NATL ACAD SCI USA, V105, P14488, DOI 10.1073/pnas.0803657105
Muñoz S, 2017, CHROMOSOMA, V126, P1, DOI 10.1007/s00412-016-0573-x
Nédélec Y, 2016, CELL, V167, P657, DOI 10.1016/j.cell.2016.09.025

Nguyen DH, 2006, P NATL ACAD SCI USA, V103, P7765, DOI 10.1073/pnas.0510484103
Nielsen R, 2017, NATURE, V541, P302, DOI 10.1038/nature21347
O'Bleness M, 2012, NAT REV GENET, V13, P853, DOI 10.1038/nrg3336
Orlando L, 2015, NAT REV GENET, V16, P395, DOI 10.1038/nrg3935
Parham P, 1996, SCIENCE, V272, P67, DOI 10.1126/science.272.5258.67
Patterson N, 2006, NATURE, V441, P1103, DOI 10.1038/nature04789
Petrovski S, 2016, GENOME BIOL, V17, DOI 10.1186/s13059-016-1016-y
Polimanti R, 2017, PLOS GENET, V13, DOI 10.1371/journal.pgen.1006618
Popejoy AB, 2016, NATURE, V538, P161, DOI 10.1038/538161a
Prüfer K, 2014, NATURE, V505, P43, DOI 10.1038/nature12886
Quach H, 2017, J EXP MED, V214, P877, DOI 10.1084/jem.20161942
Quach H, 2016, CELL, V167, P643, DOI 10.1016/j.cell.2016.09.024
Quansah E, 2018, GENOMICS, V110, P1, DOI 10.1016/j.ygeno.2017.07.009
Quintana-Murci L, 2019, CELL, V177, P184, DOI 10.1016/j.cell.2019.02.033
Racimo F, 2017, MOL BIOL EVOL, V34, P296, DOI 10.1093/molbev/msw216
Raj T, 2013, AM J HUM GENET, V92, P517, DOI 10.1016/j.ajhg.2013.03.001
Ramamoorthy A, 2019, CTS-CLIN TRANSL SCI, V12, P3, DOI 10.1111/cts.12593
Redman CWG, 2010, AM J REPROD IMMUNOL, V63, P534, DOI 10.1111/j.1600-0897.2010.00831.x
Rees JS, 2020, TRENDS GENET, V36, P415, DOI 10.1016/j.tig.2020.03.006
Ricklefs RE, 2002, TRENDS ECOL EVOL, V17, P462, DOI 10.1016/S0169-5347(02)02578-8
Rinker DC, 2020, NAT ECOL EVOL, V4, P1332, DOI 10.1038/s41559-020-1261-z
Robertson SA, 2019, NEW ENGL J MED, V380, P1080, DOI 10.1056/NEJMcibr1817501
Robinson DP, 2012, HORM BEHAV, V62, P263, DOI 10.1016/j.yhbeh.2012.02.023
Rokas A, 2008, ANNU REV GENET, V42, P235, DOI 10.1146/annurev.genet.42.110807.091513
Sáez-Cirión A, 2013, IMMUNOL REV, V254, P281, DOI 10.1111/imr.12076
Sankararaman S, 2016, CURR BIOL, V26, P1241, DOI 10.1016/j.cub.2016.03.037
Sankararaman S, 2014, NATURE, V507, P354, DOI 10.1038/nature12961
Schmidt A, 2015, J REPROD IMMUNOL, V108, P65, DOI 10.1016/j.jri.2015.03.001
Sella G, 2019, ANNU REV GENOM HUM G, V20, P461, DOI 10.1146/annurev-genom-083115-022316
Seluanov A, 2018, NAT REV CANCER, V18, P433, DOI 10.1038/s41568-018-0004-9
Sharp PM, 2010, PHILOS T R SOC B, V365, P2487, DOI 10.1098/rstb.2010.0031
Shendure J, 2017, NATURE, V550, P345, DOI 10.1038/nature24286
Shi HWB, 2020, AM J HUM GENET, V106, P805, DOI 10.1016/j.ajhg.2020.04.012
Shubin N, 2009, NATURE, V457, P818, DOI 10.1038/nature07891
Shubin Neil., 2008, Your inner fish: A journey into the 3.5-billion-year history of the human body
Sikela JM, 2018, HUM GENET, V137, P1, DOI 10.1007/s00439-017-1865-9
Simons YB, 2016, CURR OPIN GENET DEV, V41, P150, DOI 10.1016/j.gde.2016.09.006

Simons YB, 2014, NAT GENET, V46, P220, DOI 10.1038/ng.2896
Simonti CN, 2016, SCIENCE, V351, P737, DOI 10.1126/science.aad2149
Sirugo G, 2019, CELL, V177, P26, DOI [10.1016/j.cell.2019.04.032, 10.1016/j.cell.2019.02.048]
Skoglund P, 2018, ANNU REV GENOM HUM G, V19, P381, DOI 10.1146/annurev-genom-083117-021749
Skov L, 2020, NATURE, V582, P78, DOI 10.1038/s41586-020-2225-9
Smallwood TB, 2017, FRONT IMMUNOL, V8, DOI 10.3389/fimmu.2017.00453
Sobotková K, 2019, TRENDS PARASITOL, V35, P501, DOI 10.1016/j.pt.2019.04.009
Soto PC, 2013, J MOL MED, V91, P261, DOI 10.1007/s00109-012-0951-7
Srinivasan S, 2016, BIOL PSYCHIAT, V80, P284, DOI 10.1016/j.biopsych.2015.10.009
Stearns S. C., 2016, THIS FOUNDATIONAL TX
STEARNS SC, 1989, FUNCT ECOL, V3, P259, DOI 10.2307/2389364
Sulak M, 2016, ELIFE, V5, DOI [10.7554/eLife.11994, 10.7554/eLife.24307]
Suzuki IK, 2018, CELL, V173, P1370, DOI 10.1016/j.cell.2018.03.067
Than NG, 2018, FRONT IMMUNOL, V9, DOI 10.3389/fimmu.2018.01661
Thomas F, 2013, EVOL APPL, V6, P1, DOI 10.1111/eva.12021
Timpson NJ, 2018, NAT REV GENET, V19, P110, DOI 10.1038/nrg.2017.101
Tollis M, 2019, MOL BIOL EVOL, V36, P1746, DOI 10.1093/molbev/msz099
Torkamani A, 2018, NAT REV GENET, V19, P581, DOI 10.1038/s41576-018-0018-x
Turanov AA, 2018, NAT BIOTECHNOL, V36, P1164, DOI 10.1038/nbt.4297
Uricchio LH, 2016, GENOME RES, V26, P863, DOI 10.1101/gr.202440.115
van der Worp HB, 2010, PLOS MED, V7, DOI 10.1371/journal.pmed.1000245
van Manen D, 2011, PLOS ONE, V6, DOI 10.1371/journal.pone.0022208
Varki A, 2001, YEARB PHYS ANTHROPOL, V44, P54, DOI 10.1002/ajpa.10018
Varki A, 2010, P NATL ACAD SCI USA, V107, P8939, DOI 10.1073/pnas.0914634107
Varki A, 2009, P NATL ACAD SCI USA, V106, P14739, DOI 10.1073/pnas.0908196106
Varki NM, 2015, PHILOS T R SOC B, V370, DOI 10.1098/rstb.2014.0225
Vazquez JM, 2018, CELL REP, V24, P1765, DOI 10.1016/j.celrep.2018.07.042
Vernot B, 2014, SCIENCE, V343, P1017, DOI 10.1126/science.1245938
Vogelstein B, 2013, SCIENCE, V339, P1546, DOI 10.1126/science.1235122
Xu K, 2015, MOL BIOL EVOL, V32, P1148, DOI 10.1093/molbev/msv031
Yang J, 2015, NAT GENET, V47, P1114, DOI 10.1038/ng.3390
Young MJ, 2016, CURR OPIN GENET DEV, V38, P52, DOI 10.1016/j.gde.2016.03.005
Zeberg H, 2020, NATURE, V587, P610, DOI 10.1038/s41586-020-2818-3
Zeberg H, 2020, MOL BIOL EVOL, V37, P2655, DOI 10.1093/molbev/msaa119
Zera AJ, 2001, ANNU REV ECOL SYST, V32, P95, DOI 10.1146/annurev.ecolsys.32.081501.114006
NR 215
TC 132
Z9 140
U1 6
U2 100
PU NATURE PORTFOLIO
PI BERLIN
PA HEIDELBERGER PLATZ 3, BERLIN, 14197, GERMANY

SN 1471-0056
EI 1471-0064
J9 NAT REV GENET
JI Nat. Rev. Genet.
PD MAY
PY 2021
VL 22
IS 5
BP 269
EP 283
DI 10.1038/s41576-020-00305-9
EA JAN 2021
PG 15
WC Genetics & Heredity
WE Science Citation Index Expanded (SCI-EXPANDED)
SC Genetics & Heredity
GA RQ1FD
UT WOS:000605548000001
PM 33408383
OA Bronze, Green Published
DA 2025-05-07
ER

PT J
AU Baxter, FA
Drake, AJ
AF Baxter, Faye A.
Drake, Amanda J.
TI Non-genetic inheritance via the male germline in mammals
SO PHILOSOPHICAL TRANSACTIONS OF THE ROYAL SOCIETY B-BIOLOGICAL SCIENCES
LA English
DT Article
DE transgenerational epigenetic inheritance; intergenerational epigenetic inheritance; DNA methylation; histones; sRNA; developmental origins of health and disease
ID HIGH-FAT DIET; TRANSGENERATIONAL EPIGENETIC INHERITANCE; DOUBLE-STRANDED-RNA; INTERGENERATIONAL TRANSMISSION; ENDOCRINE DISRUPTORS; DNA METHYLATION; GENE-EXPRESSION; BIRTH-WEIGHT; TET PROTEINS; EPIGENOME
AB Numerous studies in humans and in animal models have demonstrated that exposure to adverse environmental conditions in early life results in long-term structural and functional changes in an organism, increasing the risk of cardiometabolic, neurobehavioural and reproductive disorders in later life. Such effects are not limited to the first generation offspring but may be transmitted to a second or a number of subsequent generations, through non-genomic mechanisms. While the transmission of 'programmed' effects through the maternal line could occur as a consequence of multiple influences, for example, altered maternal physiology, the inheritance of effects through the male line is more difficult to explain and there is much interest in a potential role for transgenerational epigenetic inheritance. In this review, we will discuss the mechanisms by which induced effects may be transmitted through the paternal lineage, with a particular focus on the role of epigenetic inheritance.

This article is part of the theme issue 'Developing differences: early-life effects and evolutionary medicine'.
C1 [Baxter, Faye A.; Drake, Amanda J.] Royal Hosp Sick Children, 9 Sci Rd, Edinburgh EH9 1LF, Midlothian, Scotland.
[Drake, Amanda J.] Univ Edinburgh, Queens Med Res Inst, Univ British Heart Fdn, Ctr Cardiovasc Sci, 47 Little France Crescent, Edinburgh EH16 4TJ, Midlothian, Scotland.
C3 University of Edinburgh
RP Drake, AJ (autor correspondente), Royal Hosp Sick Children, 9 Sci Rd, Edinburgh EH9 1LF, Midlothian, Scotland.; Drake, AJ (autor correspondente), Univ Edinburgh, Queens Med Res Inst, Univ British Heart Fdn, Ctr Cardiovasc Sci, 47 Little France Crescent, Edinburgh EH16 4TJ, Midlothian, Scotland.
EM mandy.drake@ed.ac.uk
OI Baxter, Faye/0000-0002-7937-1909; drake, amanda/0000-0001-8633-333X
FU Medical Research Council [MR/K018310/1]; British Heart Foundation; MRC [MR/K018310/1] Funding Source: UKRI
FX Work in A.J.D.'s laboratory is supported by the Medical Research Council
(MR/K018310/1) and the British Heart Foundation.
CR Alcazar RM, 2008, GENETICS, V180, P1275, DOI 10.1534/genetics.108.089433
Anway MD, 2005, SCIENCE, V308, P1466, DOI 10.1126/science.1108190
Ashe A, 2012, CELL, V150, P88, DOI 10.1016/j.cell.2012.06.018
Avril I, 2002, J ENDOCRINOL, V174, DOI 10.1677/joe.0.1740215
BAIRD D, 1985, BRIT J OBSTET GYNAEC, V92, P115, DOI 10.1111/j.1471-0528.1985.tb01062.x
Barbosa TD, 2016, MOL METAB, V5, P184, DOI 10.1016/j.molmet.2015.12.002
BARKER DJP, 1989, BMJ-BRIT MED J, V298, P564, DOI 10.1136/bmj.298.6673.564
Ben Maamar M, 2018, ENVIRON EPIGENETICS, V4, DOI 10.1093/eep/dvy010
Blewitt ME, 2006, PLOS GENET, V2, P399, DOI 10.1371/journal.pgen.0020049
Bohacek J, 2015, MOL PSYCHIATR, V20, P621, DOI 10.1038/mp.2014.80
Braun K, 2014, J NEUROENDOCRINOL, V26, P697, DOI 10.1111/jne.12174
Brieño-Enríquez MA, 2015, PLOS ONE, V10, DOI 10.1371/journal.pone.0124296
Bromfield JJ, 2014, P NATL ACAD SCI USA, V111, P2200, DOI 10.1073/pnas.1305609111
Carone BR, 2010, CELL, V143, P1084, DOI 10.1016/j.cell.2010.12.008
Cartier J, 2018, GENOME BIOL, V19, DOI 10.1186/s13059-018-1422-4
Chambers TJG, 2016, SCI REP-UK, V6, DOI 10.1038/srep31857
Chen Q, 2016, SCIENCE, V351, P397, DOI 10.1126/science.aad7977
Conine CC, 2018, DEV CELL, V46, P470, DOI 10.1016/j.devcel.2018.06.024
Daxinger L, 2012, NAT REV GENET, V13, P153, DOI 10.1038/nrg3188
Devanapally S, 2015, P NATL ACAD SCI USA, V112, P2133, DOI 10.1073/pnas.1423333112
Dias BG, 2014, NAT NEUROSCI, V17, P89, DOI 10.1038/nn.3594
Drake AJ, 2005, AM J PHYSIOL-REG I, V288, pR34, DOI 10.1152/ajpregu.00106.2004
Drake AJ, 2004, J ENDOCRINOL, V180, P1, DOI 10.1677/joe.0.1800001
Drake AJ, 2011, EPIGENETICS-US, V6, P1334, DOI 10.4161/epi.6.11.17942
Dunn GA, 2011, ENDOCRINOLOGY, V152, P2228, DOI 10.1210/en.2010-1461

EMANUEL I, 1992, BRIT J OBSTET GYNAEC, V99, P67, DOI 10.1111/j.1471-0528.1992.tb14396.x
Erkek S, 2013, NAT STRUCT MOL BIOL, V20, P868, DOI 10.1038/nsmb.2599
Francis DD, 1999, ANN NY ACAD SCI, V896, P66, DOI 10.1111/j.1749-6632.1999.tb08106.x
Franklin TB, 2010, BIOL PSYCHIAT, V68, P408, DOI 10.1016/j.biopsych.2010.05.036
Gapp K, 2014, NAT COMMUN, V5, DOI 10.1038/ncomms6466
Gapp K, 2014, NAT NEUROSCI, V17, P667, DOI 10.1038/nn.3695
Godfrey Keith M, 2016, Nestle Nutr Inst Workshop Ser, V85, P71, DOI 10.1159/000439488
Grandjean V, 2015, SCI REP-UK, V5, DOI 10.1038/srep18193
Grishok A, 2000, SCIENCE, V287, P2494, DOI 10.1126/science.287.5462.2494
Guo F, 2017, CELL RES, V27, P967, DOI 10.1038/cr.2017.82
Hauser MT, 2011, BBA-GENE REGUL MECH, V1809, P459, DOI 10.1016/j.bbagrm.2011.03.007
Heard E, 2014, CELL, V157, P95, DOI 10.1016/j.cell.2014.02.045
Hitchins MP, 2011, CANCER CELL, V20, P200, DOI 10.1016/j.ccr.2011.07.003
Hoffman DJ, 2017, NUTR REV, V75, P951, DOI 10.1093/nutrit/nux053
Iqbal K, 2015, GENOME BIOL, V16, DOI 10.1186/s13059-015-0619-z
Isbel L, 2015, INT J EPIDEMIOL, V44, P1109, DOI 10.1093/ije/dyv024
Ito S, 2011, SCIENCE, V333, P1300, DOI 10.1126/science.1210597
Ito S, 2010, NATURE, V466, P1129, DOI 10.1038/nature09303
Jimenez-Chillaron JC, 2009, DIABETES, V58, P460, DOI 10.2337/db08-0490
Kaati G, 2002, EUR J HUM GENET, V10, P682, DOI 10.1038/sj.ejhg.5200859
Kaati G, 2007, EUR J HUM GENET, V15, P784, DOI 10.1038/sj.ejhg.5201832
Kawashima T, 2014, NAT REV GENET, V15, P613, DOI 10.1038/nrg3685
Kiani J, 2013, PLOS GENET, V9, DOI 10.1371/journal.pgen.1003498
Korgan AC, 2018, PSYCHONEUROENDOCRINO, V91, P20, DOI 10.1016/j.psyneuen.2018.02.010
Korgan AC, 2016, ENEURO, V3, DOI 10.1523/ENEURO.0231-16.2016
Lane M, 2014, PLOS ONE, V9, DOI 10.1371/journal.pone.0100832
Lappalainen T, 2017, NAT REV GENET, V18, P441, DOI 10.1038/nrg.2017.32
Li L, 2018, NAT CELL BIOL, V20, P847, DOI 10.1038/s41556-018-0123-2
Lister R, 2009, NATURE, V462, P315, DOI 10.1038/nature08514
Manikkam M, 2012, PLOS ONE, V7, DOI 10.1371/journal.pone.0046249
Mashoodh R, 2012, P NATL ACAD SCI USA, V109, P17232, DOI 10.1073/pnas.1121083109
Minkina O, 2018, TRENDS GENET, V34, P54, DOI 10.1016/j.tig.2017.09.012
Nadeau JH, 2015, GENOME BIOL, V16, DOI 10.1186/s13059-015-0709-y
Ng SF, 2010, NATURE, V467, P963, DOI 10.1038/nature09491
Palmer Nicole O, 2012, Spermatogenesis, V2, P253
Radford EJ, 2018, NAT REV ENDOCRINOL, V14, P345, DOI 10.1038/s41574-018-0005-5
Radford EJ, 2014, SCIENCE, V345, P785, DOI 10.1126/science.1255903
Radford EJ, 2012, PLOS GENET, V8, P41, DOI 10.1371/journal.pgen.1002605
Rakyan VK, 2003, P NATL ACAD SCI USA, V100, P2538, DOI 10.1073/pnas.0436776100
Rando OJ, 2012, CELL, V151, P702, DOI 10.1016/j.cell.2012.10.020
Rechavi O, 2011, CELL, V147, P1248, DOI 10.1016/j.cell.2011.10.042

Rodgers AB, 2013, J NEUROSCI, V33, P9003, DOI 10.1523/JNEUROSCI.0914-13.2013
Rose CM, 2014, EPIGENET CHROMATIN, V7, DOI 10.1186/1756-8935-7-19
Rose CM, 2013, BIOCHEM SOC T, V41, P809, DOI 10.1042/BST20120356
Schneider S, 2008, REPROD TOXICOL, V25, P352, DOI 10.1016/j.reprotox.2008.04.001
Seisenberger S, 2013, PHILOS T R SOC B, V368, DOI 10.1098/rstb.2011.0330
Sharma U, 2016, SCIENCE, V351, P391, DOI 10.1126/science.aad6780
Shea JM, 2015, DEV CELL, V35, P750, DOI 10.1016/j.devcel.2015.11.024
Siklenka K, 2015, SCIENCE, V350, DOI 10.1126/science.aab2006
Smallwood SA, 2012, TRENDS GENET, V28, P33, DOI 10.1016/j.tig.2011.09.004
Szabó PE, 2015, GENOME BIOL, V16, DOI 10.1186/s13059-015-0714-1
Szyf M, 2015, TRENDS MOL MED, V21, P134, DOI 10.1016/j.molmed.2014.12.004
Terashima M, 2015, EPIGENETICS-US, V10, P861, DOI 10.1080/15592294.2015.1075691
Vassoler FM, 2013, NAT NEUROSCI, V16, P42, DOI 10.1038/nn.3280
Vojtech L, 2014, NUCLEIC ACIDS RES, V42, P7290, DOI 10.1093/nar/gku347
Waterland RA, 2003, MOL CELL BIOL, V23, P5293, DOI 10.1128/MCB.23.15.5293-5300.2003
Waterland RA, 2007, FASEB J, V21, P3380, DOI 10.1096/fj.07-8229com
Waterland RA, 2006, GENESIS, V44, P401, DOI 10.1002/dvg.20230
Xu QH, 2018, TRENDS CELL BIOL, V28, P237, DOI 10.1016/j.tcb.2017.10.008
Yuan SQ, 2016, DEVELOPMENT, V143, P635, DOI 10.1242/dev.131755
Zeybel M, 2012, NAT MED, V18, P1592, DOI 10.1038/nm1012-1592c
Zhang YF, 2018, NAT CELL BIOL, V20, P535, DOI 10.1038/s41556-018-0087-2
NR 87
TC 29
Z9 32
U1 0
U2 27
PU ROYAL SOC
PI LONDON
PA 6-9 CARLTON HOUSE TERRACE, LONDON SW1Y 5AG, ENGLAND
SN 0962-8436
EI 1471-2970
J9 PHILOS T R SOC B
JI Philos. Trans. R. Soc. B-Biol. Sci.
PD APR 15
PY 2019
VL 374
IS 1770
AR 20180118
DI 10.1098/rstb.2018.0118
PG 7
WC Biology
WE Science Citation Index Expanded (SCI-EXPANDED)
SC Life Sciences & Biomedicine - Other Topics
GA HN8ZI
UT WOS:000460487000009

PM 30966887
OA hybrid, Green Published
DA 2025-05-07
ER

PT J
AU Pretelli, I
   Crittenden, AN
   Dounias, E
   Friant, S
   Koster, J
   Kramer, KL
   Mangola, SM
   Saez, AM
   Lew-Levy, S
AF Pretelli, Ilaria
   Crittenden, Alyssa N.
   Dounias, Edmond
   Friant, Sagan
   Koster, Jeremy
   Kramer, Karen L.
   Mangola, Shani M.
   Saez, Almudena Mari
   Lew-Levy, Sheina
TI Child and adolescent foraging: New directions in evolutionary research
SO EVOLUTIONARY ANTHROPOLOGY
LA English
DT Article
DE childhood evolution; children; community resilience; foraging; immune
   development; inclusive fitness
ID LIFE-HISTORY; TIME ALLOCATION; OKAVANGO DELTA; HADZA; CONSUMPTION;
   DEPENDENCY; MENOPAUSE; COMMUNITY; KNOWLEDGE; PROVINCE
AB Young children and adolescents in subsistence societies forage for a
wide range of resources. They often target child-specific foods, they can
be very successful foragers, and they share their produce widely within
and outside of their nuclear family. At the same time, while foraging,
they face risky situations and are exposed to diseases that can influence
their immune development. However, children's foraging has largely been
explained in light of their future (adult) behavior. Here, we reinterpret
findings from human behavioral ecology, evolutionary medicine and
cultural evolution to center foraging children's contributions to life
history evolution, community resilience and immune development. We
highlight the need to foreground immediate alongside delayed benefits and
costs of foraging, including inclusive fitness benefits, when discussing
children's food production from an evolutionary perspective. We conclude
by recommending that researchers carefully consider children's social and
ecological context, develop cross-cultural perspectives, and incorporate
children's foraging into Indigenous sovereignty discourse.
C1 [Pretelli, Ilaria] Toulouse Sch Econ, Inst Adv Study Toulouse,
Toulouse, France.
   [Pretelli, Ilaria] Univ Toulouse Capitole, Toulouse, France.
   [Pretelli, Ilaria; Koster, Jeremy] Max Planck Inst Evolutionary
Anthropol, Dept Human Behav Ecol & Culture, Leipzig, Germany.

[Crittenden, Alyssa N.] Univ Nevada, Dept Anthropol, Las Vegas, NV USA.
[Dounias, Edmond] Univ Montpellier, CEFE, CNRS, EPHE,IRD, Montpellier, France.
[Friant, Sagan] Penn State Univ, Dept Anthropol, University Pk, PA USA.
[Kramer, Karen L.] Univ Utah, Dept Anthropol, Salt Lake City, UT USA.
[Mangola, Shani M.] Law Sch Tanzania, Dar Es Salaam, Tanzania.
[Mangola, Shani M.] Olanakwe Community Fund, Mangola, Tanzania.
[Saez, Almudena Mari] French Natl Inst Sustainable Dev, TransVHIMI Unit, IRD, Montpellier, France.
[Lew-Levy, Sheina] Univ Durham, Dept Psychol, Durham, England.
C3 Universite de Toulouse; Universite Toulouse 1 Capitole; Max Planck Society; Nevada System of Higher Education (NSHE); University of Nevada Las Vegas; Universite PSL; Ecole Pratique des Hautes Etudes (EPHE); Institut Agro; Montpellier SupAgro; CIRAD; Centre National de la Recherche Scientifique (CNRS); Institut de Recherche pour le Developpement (IRD); Universite Paul-Valery; Universite de Montpellier; Pennsylvania Commonwealth System of Higher Education (PCSHE); Pennsylvania State University; Penn State Behrend; Pennsylvania State University - University Park; Utah System of Higher Education; University of Utah; Universite de Montpellier; Institut de Recherche pour le Developpement (IRD); Durham University
RP Pretelli, I (autor correspondente), Toulouse Sch Econ, Inst Adv Study Toulouse, Toulouse, France.; Pretelli, I (autor correspondente), Univ Toulouse Capitole, Toulouse, France.
EM ilaria.pretelli@iast.fr; alyssa.crittenden@unlv.edu; edmond.dounias@ird.fr; sagan.friant@psu.edu; jeremy_koster@eva.mpg.de; karen.kramer@anthro.utah.edu; shani.mangola@olanakwe.org; almudena.marisaez@ird.fr; sheina.lew-levy@durham.ac.uk
RI LewLevy, Sheina/KHD-8618-2024; Saez, Almudena/AAU-9835-2021; Pretelli, Ilaria/HPH-1132-2023; Kramer, Karen/AAM-3022-2020
OI Friant, Sagan/0000-0003-1664-5180; Mari Saez, Almudena/0000-0002-5429-8485; Pretelli, Ilaria/0000-0002-8244-5737; Lew-Levy, Sheina/0000-0002-1250-6418
FU French National Research Agency (ANR) [ANR-17-EURE-0010]
FX French National Research Agency (ANR),Grant/Award Number: ANR-17-EURE-0010
CR ALVARD MS, 1993, HUM ECOL, V21, P355, DOI 10.1007/BF00891140
Andersen KG, 2012, PHILOS T R SOC B, V367, P868, DOI 10.1098/rstb.2011.0299
Baize S, 2014, NEW ENGL J MED, V371, P1418, DOI 10.1056/NEJMoa1404505
Bird DW, 2002, HUM NATURE-INT BIOS, V13, P269, DOI 10.1007/s12110-002-1010-9
Bird DW., 2005, Hunter-gatherer childhoods : evolutionary, developmental cultural perspectivesJournal Article, P129
Bjorklund DF, 2000, CHILD DEV, V71, P1687, DOI 10.1111/1467-8624.00258
Blurton Jones N.G., 1997, Uniting psychology and biology: Integrative perspectives on human development, P279, DOI DOI 10.1037/10242-029
Blurton JonesN., 1989, COMP SOCIOECOLOGY, P367
Bock J, 2004, HUM NATURE-INT BIOS, V15, P63, DOI 10.1007/s12110-004-1004-x

Bock J, 2002, HUM NATURE-INT BIOS, V13, P161, DOI 10.1007/s12110-002-1007-4
Bonwitt J, 2017, PLOS NEGLECT TROP D, V11, DOI 10.1371/journal.pntd.0005699
Bonwitt J, 2016, ECOHEALTH, V13, P234, DOI 10.1007/s10393-016-1098-8
Boyette A. H., 2019, Hunter Gatherer Research, V3, P475, DOI [10.3828/hgr.2017.23, DOI 10.3828/HGR.2017.23]
Boyette AH, 2016, CHILD DEV, V87, P759, DOI 10.1111/cdev.12496
Budowle R, 2019, J AGRIC FOOD SYST CO, V9, P145, DOI [10.5304/jafscd.2019.09B.018, 10.5304/jafscd.2019.09b.018]
Carlson CJ, 2022, NATURE, V607, P555, DOI 10.1038/s41586-022-04788-w
Cidro J., 2015, CANADIAN J URBAN RES, V24, P24
Cinelli C, 2024, SOCIOL METHOD RES, V53, P1071, DOI 10.1177/00491241221099552
Cote Charlotte., 2016, HUMANITIES-BASEL, V5, P57, DOI DOI 10.3390/H5030057
Crittenden A.N., 2016, Childhood: Origins, evolution, and implications, P155
Crittenden AN, 2008, HUM NATURE-INT BIOS, V19, P249, DOI 10.1007/s12110-008-9043-3
Crittenden AN, 2015, PLOS ONE, V10, DOI 10.1371/journal.pone.0131996
Crittenden AN, 2013, EVOL HUM BEHAV, V34, P299, DOI 10.1016/j.evolhumbehav.2013.04.004
Dounias E., 2017, ANTHROPOCHILDREN, V7, DOI [10.25518/2034-8517.2799, DOI 10.25518/2034-8517.2799]
Dounias E., 1993, MIMEOGRAPH, P2
Dounias E., 2014, FORAGING TO FORAGING
Dounias E, 2016, ECOL SOC, V21, DOI 10.5751/ES-07946-210123
Douno M, 2021, PLOS NEGLECT TROP D, V15, DOI 10.1371/journal.pntd.0009212
DRAPER P, 1988, ETHNOLOGY, V27, P339, DOI 10.2307/3773398
Duda R., 2023, ARXIV, DOI [10.31235/osf.io/sv89a, DOI 10.31235/OSF.IO/SV89A]
Duda Romain, 2017, African Study Monographs, V38, P97
Fernández-Llamazares A, 2021, J ETHNOBIOL, V41, P144, DOI 10.2993/0278-0771-41.2.144
Fouts HN, 2016, BEHAVIOUR, V153, P663, DOI 10.1163/1568539X-00003362
Frankenhuis WE, 2022, DEV PSYCHOPATHOL, V34, P473, DOI 10.1017/S0954579421001401
Friant S, 2022, ONE HEALTH OUTLOOK, V4, DOI 10.1186/s42522-022-00060-3
Friant S, 2015, PLOS NEGLECT TROP D, V9, DOI 10.1371/journal.pntd.0003792
Froehle AW, 2019, AM J HUM BIOL, V31, DOI 10.1002/ajhb.23209
Gallois S, 2017, J ETHNOBIOL, V37, P60, DOI 10.2993/0278-0771-37.1.60
Gibb R, 2020, NATURE, V584, P398, DOI 10.1038/s41586-020-2562-8
Gibson MA, 2015, EVOL ANTHROPOL, V24, P3, DOI 10.1002/evan.21432
Gompper M.E., 2014, Free-Ranging Dogs and Wildlife Conservation, P265, DOI [10.7717/peerj.9906, DOI 10.7717/PEERJ.9906]
GOODMAN MJ, 1985, ANN HUM BIOL, V12, P169, DOI 10.1080/03014468500007661
Gopnik A, 2020, PHILOS T R SOC B, V375, DOI 10.1098/rstb.2019.0502
Gurven M, 2006, J HUM EVOL, V51, P454, DOI 10.1016/j.jhevol.2006.05.003
Gurven Michael, 2006, Soc Biol, V53, P81

HAMILTON WD, 1966, J THEOR BIOL, V12, P12, DOI 10.1016/0022-5193(66)90184-6
Haun BK, 2019, PLOS NEGLECT TROP D, V13, DOI 10.1371/journal.pntd.0007614
HAWKES K, 1995, CURR ANTHROPOL, V36, P688, DOI 10.1086/204420
Hawkes K, 1997, CURR ANTHROPOL, V38, P551, DOI 10.1086/204646
Hawkes K, 1998, P NATL ACAD SCI USA, V95, P1336, DOI 10.1073/pnas.95.3.1336
Hewlett BS, 2011, PHILOS T R SOC B, V366, P1168, DOI 10.1098/rstb.2010.0373
HEYMANN DL, 1980, J INFECT DIS, V142, P372, DOI 10.1093/infdis/142.3.372
Hoover E, 2017, AM INDIAN CULT RES J, V41, P31, DOI 10.17953/aicrj.41.3.hoover
Ichikawa Mitsuo, 1998, African Study Monographs Supplementary Issue, V25, P105
Jang H, 2022, P ROY SOC B-BIOL SCI, V289, DOI 10.1098/rspb.2022.1407
Jankhöfer CH, 2019, T ROY SOC TROP MED H, V113, pS111
Kandza V., 2020, SELECTION FOOD SPECI
Kaplan H, 2000, EVOL ANTHROPOL, V9, P156, DOI 10.1002/1520-6505(2000)9:4<156::AID-EVAN5>3.0.CO;2-7
Kaplan H, 1996, YEARB PHYS ANTHROPOL, V39, P91
KAWABE T, 1983, Journal of Human Ergology, V12, P65
Kline MA, 2013, HUM NATURE-INT BIOS, V24, P351, DOI 10.1007/s12110-013-9180-1
Koster J, 2020, SCI ADV, V6, DOI 10.1126/sciadv.aax9070
Koster JM, 2013, HUM ECOL, V41, P221, DOI 10.1007/s10745-012-9549-5
Kramer K., 2009, SUBSTITUTE PARENTS B, P77
Kramer K. L., 2005, MAYA CHILDREN, P123
Kramer KL, 2021, HUM NATURE-INT BIOS, V32, P87, DOI 10.1007/s12110-021-09392-x
Kramer KL, 2018, PHYSIOL BEHAV, V193, P117, DOI 10.1016/j.physbeh.2018.02.054
Kramer KL, 2014, TRENDS ECOL EVOL, V29, P600, DOI 10.1016/j.tree.2014.09.001
Kramer KL, 2011, HUM NATURE-INT BIOS, V22, P303, DOI 10.1007/s12110-011-9122-8
Kramer KL, 2010, EVOL ANTHROPOL, V19, P136, DOI 10.1002/evan.20265
Lee R. B., 1979, KUNG SAN MEN WOM
Lew-Levy S., LEARNING WALK FOREST
Lew-Levy S, 2022, SCI REP-UK, V12, DOI 10.1038/s41598-022-12217-1
Lew-Levy S, 2017, HUM NATURE-INT BIOS, V28, P367, DOI 10.1007/s12110-017-9302-2
Marshall AJ, 2009, AM J PHYS ANTHROPOL, V140, P603, DOI 10.1002/ajpa.21082
Milstein MS, 2020, ECOHEALTH, V17, P424, DOI 10.1007/s10393-021-01513-3
Nolen LD, 2015, AM J TROP MED HYG, V93, P410, DOI 10.4269/ajtmh.15-0168
Page AE, 2021, PHILOS T R SOC B, V376, DOI 10.1098/rstb.2020.0026
Patel R, 2009, J PEASANT STUD, V36, P663, DOI 10.1080/03066150903143079
Pollom TR, 2020, HUM NATURE-INT BIOS, V31, P123, DOI 10.1007/s12110-020-09364-7

   Porcher V., 2023, PROFICIENT ADULTS DI
   Pretelli I, 2022, SCI ADV, V8, DOI 10.1126/sciadv.abn9889
   Reiches MW, 2009, AM J HUM BIOL, V21, P421, DOI 10.1002/ajhb.20906
   Reyes-García V, 2019, FOOD SECUR, V11, P109, DOI 10.1007/s12571-018-0882-4
   Saéz AM, 2015, EMBO MOL MED, V7, P17, DOI 10.15252/emmm.201404792
   Scheidel A, 2023, SCI ADV, V9, DOI 10.1126/sciadv.ade9557
   Seang S, 2022, LANCET, V400, P658, DOI 10.1016/S0140-6736(22)01487-8
   Shoemaker T, 2012, EMERG INFECT DIS, V18, P1480, DOI 10.3201/eid1809.111536
   SMITH EA, 1985, ETHOL SOCIOBIOL, V6, P27, DOI 10.1016/0162-3095(85)90039-1
   Terashima H., 2007, ANIMAL SYMBOLISM KEY, P147
   Tucker B., 2005, HUNTER GATHERER CHIL, P147
   van der Wal JEM, 2022, FRONT CONSERV SCI, V2, DOI 10.3389/fcosc.2021.727479
   van Vliet N, 2015, ORYX, V49, P165, DOI 10.1017/S0030605313000549
   Villmoare B., 2023, EVOLUTIONARY ORIGINS, DOI [10.21203/rs.3.rs-2515272/v1, DOI 10.21203/RS.3.RS-2515272/V1]
   Walker R, 2002, J HUM EVOL, V42, P639, DOI 10.1006/jhev.2001.0541
   WEISNER TS, 1977, CURR ANTHROPOL, V18, P169, DOI 10.1086/201883
NR 97
TC 2
Z9 2
U1 1
U2 4
PU WILEY
PI HOBOKEN
PA 111 RIVER ST, HOBOKEN 07030-5774, NJ USA
SN 1060-1538
EI 1520-6505
J9 EVOL ANTHROPOL
JI Evol. Anthropol.
PD APR
PY 2024
VL 33
IS 2
DI 10.1002/evan.22020
EA JAN 2024
PG 11
WC Anthropology
WE Social Science Citation Index (SSCI)
SC Anthropology
GA ND3K5
UT WOS:001140993200001
PM 38214699
DA 2025-05-07
ER

PT J
AU Balbin, MM
   Hull, D
   Guest, C
   Nichols, L

Dunn, R
Thakur, S
AF Balbin, Michelle M.
Hull, Dawn
Guest, Chloe
Nichols, Lauren
Dunn, Robert
Thakur, Siddhartha
TI Antimicrobial resistance and virulence factors profile of Salmonella spp. and Escherichia coli isolated from different environments exposed to anthropogenic activity
SO JOURNAL OF GLOBAL ANTIMICROBIAL RESISTANCE
LA English
DT Article
DE Anthropogenic areas; Whole-genome sequencing; FosA7 gene; pHN7A8 (F33:A-:B-plasmid)
AB Objective: The study aimed to identify the antimicrobial resistance (AMR) determinants and virulence factors in Salmonella spp. and Escherichia coli recovered from different anthropogenic areas in North Carolina.

Methods: Soil samples were collected from different anthropogenic areas, urban and natural. The minimum inhibitory concentration (MIC) was determined by using the broth microdilution method. Whole-genome sequencing (WGS) and analysis were done to identify the AMR determinants and virulence factors.

Results: A higher prevalence of Salmonella spp. and E. coli was detected in the urban environment. The Salmonella spp. isolates showed resistance to sulfisoxazole and streptomycin, whereas E. coli was resistant to sulfisoxazole, cefoxitin and ampicillin. Salmonella serotypes Schwarzengrund and Mississippi were identified based on WGS analysis. Aminoglycoside resistance genes and IncEIB and IncFIC(FII) plasmids were detected among Salmonella spp. In general, E. coli was predominated by isolates from phylogroups B1, B2 and D. The multidrug transporter mdfA gene was detected in most of the E. coli from both the urban (100%) and natural (84.5%) environments. The FosA7 gene was detected in an isolate from a residential yard. The pCoo and pB171 plasmids were detected in an urban environment; col(156) and pHN7A8 plasmids were detected in natural environments.

Conclusions: The detection of AMR determinants and virulence factors in these bacteria is significant in understanding the occurrence and even the development of AMR. The presence of these determinants in different anthropogenic areas suggests the need to conduct longitudinal studies for comparing the profile of pathogens across different environments. (C) 2020 The Author(s). Published by Elsevier Ltd on behalf of International Society for Antimicrobial Chemotherapy.
C1 [Balbin, Michelle M.; Hull, Dawn; Thakur, Siddhartha] North Carolina State Univ, Coll Vet Med, Dept Populat Hlth & Pathobiol, Raleigh, NC USA.
[Guest, Chloe; Thakur, Siddhartha] Univ Surrey, Fac Hlth & Med Sci, Guildford GU2 7XH, Surrey, England.
[Nichols, Lauren; Dunn, Robert] North Carolina State Univ, Coll Agriculutre & Life Sci, Dept Appl Ecol, Raleigh, NC USA.
[Balbin, Michelle M.] North Carolina State Univ, Comparat Med Inst, Raleigh, NC USA.
C3 North Carolina State University; University of Surrey; North Carolina

State University; North Carolina State University
RP Thakur, S (autor correspondente), 1060 William Moore Dr, Raleigh, NC 27606 USA.
EM sthakur@ncsu.edu
RI Dunn, Robert/B-1360-2013
OI Thakur, Siddhartha/0000-0003-3787-9939; Dunn, Robert/0000-0002-6030-4837; Nichols, Lauren/0000-0002-5599-8547; , Dawn/0000-0002-2065-7400
FU Duke Triangle Center for Evolutionary Medicine (TriCEM) [1U18FD00678801]
FX This project was supported by the Duke Triangle Center for Evolutionary Medicine (TriCEM). The whole-genome sequencing was completed by the FDAGenomeTrakr program funded grant 1U18FD00678801.
CR Akiyama T, 2012, J ANTIMICROB CHEMOTH, V67, P101, DOI 10.1093/jac/dkr414
Aminov RI, 2010, FRONT MICROBIOL, V1, DOI 10.3389/fmicb.2010.00134
Bengtsson-Palme J, 2018, FEMS MICROBIOL REV, V42, P68, DOI 10.1093/femsre/fux053
Berendonk TU, 2015, NAT REV MICROBIOL, V13, P310, DOI 10.1038/nrmicro3439
Fratamico PM, 2009, FOOD ANAL METHOD, V2, P169, DOI 10.1007/s12161-008-9046-z
Gutiérrez-Rodríguez E, 2012, J APPL MICROBIOL, V112, P109, DOI 10.1111/j.1365-2672.2011.05184.x
Gyles C, 2014, VET PATHOL, V51, P328, DOI 10.1177/0300985813511131
Huijbers PMC, 2015, ENVIRON SCI TECHNOL, V49, P11993, DOI 10.1021/acs.est.5b02566
Ibn Mafiz A, 2018, INT J ANTIMICROB AG, V52, P241, DOI 10.1016/j.ijantimicag.2018.05.016
Jennings E, 2017, CELL HOST MICROBE, V22, P217, DOI 10.1016/j.chom.2017.07.009
Johnson TJ, 2009, MICROBIOL MOL BIOL R, V73, P750, DOI 10.1128/MMBR.00015-09
Larsson DGJ, 2018, ENVIRON INT, V117, P132, DOI 10.1016/j.envint.2018.04.041
Lou LX, 2019, FRONT CELL INFECT MI, V9, DOI 10.3389/fcimb.2019.00270
Martinez JL, 2009, ENVIRON POLLUT, V157, P2893, DOI 10.1016/j.envpol.2009.05.051
o A Resistance R., 2014, Review on Antimicrobial Resistance
Pornsukarom S, 2018, BMC GENOMICS, V19, DOI 10.1186/s12864-018-5137-4
Pornsukarom S, 2016, PLOS ONE, V11, DOI 10.1371/journal.pone.0164621
Rehman MA, 2017, ANTIMICROB AGENTS CH, V61, DOI [10.1128/AAC.00410-17, 10.1128/aac.00410-17]
US Food and Drug Administration, 2016, NAT ANT RES MON SYST
van Elsas JD, 2011, ISME J, V5, P173, DOI 10.1038/ismej.2010.80
Yang J., 2020, VIRULENCE FACTORS PA
NR 21
TC 19
Z9 21
U1 1
U2 12
PU ELSEVIER SCI LTD
PI OXFORD

PA THE BOULEVARD, LANGFORD LANE, KIDLINGTON, OXFORD OX5 1GB, OXON, ENGLAND
SN 2213-7165
EI 2213-7173
J9 J GLOB ANTIMICROB RE
JI J. Glob. Antimicrob. Resist.
PD SEP
PY 2020
VL 22
BP 578
EP 583
DI 10.1016/j.jgar.2020.05.016
PG 6
WC Infectious Diseases; Pharmacology & Pharmacy
WE Science Citation Index Expanded (SCI-EXPANDED)
SC Infectious Diseases; Pharmacology & Pharmacy
GA NS4CB
UT WOS:000572210500025
PM 32534045
OA gold
DA 2025-05-07
ER

PT J
AU Fernández-Aragón, C
   Fernández-Arias, C
AF Fernandez-Aragon, Cristina
   Fernandez-Arias, Cristina
TI The evolutionary theory in medical education: from Darwin to educational
   technology
SO TECNOLOGIA CIENCIA Y EDUCACION
LA English
DT Article
DE scientific competence; challenge-based learning; global competence; <<
   One Health >> approach; gamification; educational escape room;
   evolutionary medicine; active learning methodologies
ID SCIENCE
AB The theory of evolution is a fundamental principle in the life sciences. However, more than a century and a half after the publication of Charles Darwin's On the Origin of Species, medicine has yet to integrate these evolutionary principles into daily practice or medical training. This work proposes an educational experience aimed at promoting evolutionary literacy among Medical students while fostering essential 21st century skills, such as scientific, global, and digital competencies. The proposal is based on a thorough analysis of students' alternative conceptions and is enriched with innovative elements offered by educational technologies. It addresses relevant medical issues -such as antibiotic resistance, the emergence of zoonotic diseases, and the evolution of human populations in response to pathogens- from a global perspective like that of << One Health >>. The initial diagnosis reveals gaps in knowledge and acceptance of certain aspects of evolutionary theory. After implementing the educational proposal, an improvement in this knowledge and an increase in interest and motivation regarding the

addressed topics were observed. This highlights the potential of active methodologies and educational technology as drivers of learning, an aspect that deserves more attention within higher education scenarios.
C1 [Fernandez-Aragon, Cristina] Univ Distancia Madrid, UDIMA, Madrid, Spain.
[Fernandez-Arias, Cristina] Univ Complutense Madrid, Madrid, Spain.
C3 Madrid Open University, UDIMA; Complutense University of Madrid
RP Fernández-Aragón, C (autor correspondente), Univ Distancia Madrid, UDIMA, Madrid, Spain.
EM mariacristina.fernandez.a@udima.es; crifer25@ucm.es
RI Yuksel, Asiye/F-5938-2018
OI Yuksel, Asiye/0000-0003-0749-3576
CR Alba N. de, 2017, Ensenanza Universitaria. Como mejorarla, P37
[Anonymous], 2021, Breast cancer: World Health Organization Internet
[Anonymous], 2021, Ley Organica 8/2021, de 4 de junio, de proteccion integral a la infancia y la adolescencia frente a la violencia
[Anonymous], 2018, PREPARING OUR YOUTH
Beniermann A., 2021, Evolution Education Question- naire on Acceptance and Knowledge (EEQ)- Standardised and Ready-to-Use Protocols to Measure Acceptance of Evolution and Knowledge about Evolution in an International Context, DOI [10.5281/ze nodo.4554742, DOI 10.5281/ZENODO.4554742, 10.5281/zenodo.4554742]
Beniermann A., 2019, Evolution-von Akzeptanz und Zweifeln: Empirische Studien uber Einstelungen zu Evolution und Bewusstsein, DOI [10.1007/978-3-658-24105-6, DOI 10.1007/978-3-658-24105-6]
Benton ML, 2021, NAT REV GENET, V22, P269, DOI 10.1038/s41576-020-00305-9
Bilbao-Quintana N., 2022, Educ. Knowl. Soc. (EKS), V23, P1, DOI [10.14201/eks27126, DOI 10.14201/EKS27126]
Bland J Martin, 2004, BMC Med Educ, V4, P31, DOI 10.1186/1472-6920-4-31
Caal P., 2011, Didctica de la bio- loga y la geologa
Casado-Ledesma L, 2023, READ WRIT, V36, P965, DOI 10.1007/s11145-021-10191-0
Chen J, 2022, FRONT PSYCHOL, V13, DOI 10.3389/fpsyg.2022.994700
Cook DA, 2016, MED EDUC, V50, P997, DOI 10.1111/medu.13074
Danaei G, 2011, LANCET, V377, P568, DOI 10.1016/S0140-6736(10)62036-3
De La Cruz Velazco Pedro Hernán, 2022, Horizontes Rev. Inv. Cs. Edu., V6, P1409, DOI 10.33996/revistahorizontes.v6i25.422
Delgado-Quiones E. G., 2022, Revista de Medicina del Instituto Mexicano del Seguro Social, V60
Domenech Girbau M., 2009, Ensenanza de las Ciencias: Revista de Investigacion y Experiencias Didacticas, V1, P148
Friedrichsen P. J, 2018, EVOLUTION ED GLOBE, P41, DOI DOI 10.1007/978-3-319-90939-4_3
Gallagher SE, 2023, TEACH HIGH EDUC, V28, P1135, DOI 10.1080/13562517.2020.1863354
Galli L.G., 2020, EVOL EDUC OUTREACH, V13, P1, DOI DOI 10.1186/s12052-020-00120-0
Gefaell J, 2020, PLOS ONE, V15, DOI 10.1371/journal.pone.0238345
Glaze AL, 2015, SCI EDUC, V99, P500, DOI 10.1002/sce.21158
Graves Joseph L Jr, 2016, Evol Med Public Health, V2016, P358
Gregory T. Ryan, 2009, Evolution Education and Outreach, V2, P156
Grunspan DZ, 2019, EVOL MED PUBLIC HLTH, P82, DOI 10.1093/emph/eoz012

Harms U., 2019, Evolution Education Re-considered, P271, DOI [DOI 10.1007/978-3-030-14698-6, 10.1007/978-3-030-14698-6]
Heddy BC, 2013, SCI EDUC, V97, P723, DOI 10.1002/sce.21072
Herrada Valverde R. I., 2018, Campo Abierto: Revista de Educacion, V37, P157
Kampourakis K, 2014, UNDERSTANDING EVOLUTION, P1, DOI 10.1017/CBO9781139542357
Kampourakis Kostas, 2022, Evolution (N Y), V15, P21, DOI 10.1186/s12052-022-00180-4
Khoshnoodifar Mehrnoosh, 2023, J Adv Med Educ Prof, V11, P230, DOI 10.30476/JAMP.2023.98953.1817
Kuschmierz P., 2021, EVOL EDUC OUTREACH, V14, P17, DOI DOI 10.1186/s12052-021-00158-8
Kuschmierz P, 2020, INT J SCI EDUC, V42, P2601, DOI 10.1080/09500693.2020.1822561
Li XS, 2023, SCI EDUC-NETHERLANDS, V32, P123, DOI 10.1007/s11191-021-00288-x
Mardones Cofre H., 2017, Ensenanza de las Ciencias: Revista de Investigacion y Experiencias Didacticas, num. extra, P2491
Miller JD, 2006, SCIENCE, V313, P765, DOI 10.1126/science.1126746
Mindell D.P., 2006, The evolving world: Evolution in everyday life
Murray CJL, 2022, LANCET, V399, P629, DOI 10.1016/S0140-6736(21)02724-0
Natterson-Horowitz B, 2023, Front Sci, V1, DOI 10.3389/fsci.2023.997136
Nesse RM, 2010, P NATL ACAD SCI USA, V107, P1800, DOI 10.1073/pnas.0906224106
Perez Vazquez E., 2019, Investigacion e innovacion en la ensenanza superior, P660
R Core Team, 2022, R: A language and environment for statistical computing
Ratinho E, 2023, HELIYON, V9, DOI 10.1016/j.heliyon.2023.e19033
Robledo P, 2015, RIE-REV INVESTIG EDU, V33, P369, DOI 10.6018/rie.33.2.201381
Rubio Pulido M., 2017, Diseno universal para el aprendizaje, porque todos somos todos
Xu MS, 2023, FRONT PUBLIC HEALTH, V11, DOI 10.3389/fpubh.2023.1113682
Yousuf A., 2011, Evolution: Education and Outreach, V4, P580, DOI [10.1007/s12052-011-0376-8, DOI 10.1007/S12052-011-0376-8]
NR 47
TC 0
Z9 0
U1 0
U2 0
PU CENTRO ESTUDIOS FINANCIEROS
PI MADRID
PA P A GENERAL MARTANEZ CAMPOS, MADRID, 5, SPAIN
SN 2444-250X
EI 2444-2887
J9 TECNOL CIENC ED-ESP
JI Tecnol. Cienc. Educ.
PD JAN-APR
PY 2025
IS 30

BP 67
EP 90
DI 10.51302/tce.2025.22213
PG 24
WC Education & Educational Research
WE Emerging Sources Citation Index (ESCI)
SC Education & Educational Research
GA T7E9U
UT WOS:001406604500003
OA gold
DA 2025-05-07
ER

PT J
AU Pimenoff, VN
de Oliveira, CM
Bravo, IG
AF Pimenoff, Ville N.
Mendes de Oliveira, Cristina
Bravo, Ignacio G.
TI Transmission between Archaic and Modern Human Ancestors during the Evolution of the Oncogenic Human Papillomavirus 16
SO MOLECULAR BIOLOGY AND EVOLUTION
LA English
DT Article
DE divergence; evolutionary medicine; Hominin evolution; host-switch;
human
papillomavirus; infection and cancer; sexually transmitted infection; variant; virus-host coevolution
ID MULTIPLE SEQUENCE ALIGNMENT; WHOLE-GENOME ASSOCIATION; ASIAN-AMERICAN VARIANTS; CERVICAL-CANCER; TYPE-18 VARIANTS; HPV-16 VARIANTS; NEANDERTHAL; HISTORY; WOMEN; SELECTION
AB Every human suffers through life a number of papillomaviruses (PVs) infections, most of them asymptomatic. A notable exception are persistent infections by Human papillomavirus 16 (HPV16), the most oncogenic infectious agent for humans and responsible for most infection-driven anogenital cancers. Oncogenic potential is not homogeneous among HPV16 lineages, and genetic variation within HPV16 exhibits some geographic structure. However, an in-depth analysis of the HPV16 evolutionary history was still wanting. We have analyzed extant HPV16 diversity and compared the evolutionary and phylogeographical patterns of humans and of HPV16. We show that codivergence with modern humans explains at most 30% of the present viral geographical distribution. The most explanatory scenario suggests that ancestral HPV16 already infected ancestral human populations and that viral lineages co-diverged with the hosts in parallel with the split between archaic Neanderthal-Denisovans and ancestral modern human populations, generating the ancestral HPV16A and HPV16BCD viral lineages, respectively. We propose that after out-of-Africa migration of modern human ancestors, sexual transmission between human populations introduced HPV16A into modern human ancestor populations. We hypothesize that differential coevolution of HPV16 lineages with different but closely related ancestral human populations and subsequent host-switch events in parallel with introgression of archaic alleles into the genomes of modern human ancestors may be largely

responsible for the present-day differential prevalence and association with cancers for HPV16 variants.
C1 [Pimenoff, Ville N.; Mendes de Oliveira, Cristina; Bravo, Ignacio G.] Catalan Inst Oncol, Infect & Canc Lab, Canc Epidemiol Res Programme, Barcelona, Spain.
[Pimenoff, Ville N.] Bellvitge Inst Biomed Res IDIBELL, Unit Biomarkers & Susceptibil, Barcelona, Spain.
[Mendes de Oliveira, Cristina] Univ Sao Paulo, Inst Trop Med, Virol Lab, Sao Paulo, Brazil.
[Bravo, Ignacio G.] Natl Ctr Sci Res CNRS, UMR CNRS 5290, IRD 224, MIVEGEC,UM, Montpellier, France.
C3 Institut Catala d'Oncologia; Institut d'Investigacio Biomedica de Bellvitge (IDIBELL); Universidade de Sao Paulo; Universite de Montpellier; Centre National de la Recherche Scientifique (CNRS); Institut de Recherche pour le Developpement (IRD)
RP Pimenoff, VN (autor correspondente), Catalan Inst Oncol, Infect & Canc Lab, Canc Epidemiol Res Programme, Barcelona, Spain.; Pimenoff, VN (autor correspondente), Bellvitge Inst Biomed Res IDIBELL, Unit Biomarkers & Susceptibil, Barcelona, Spain.
EM ville.pimenoff@gmail.com
RI Oliveira, Cristina/F-7193-2012; Bravo, Ignacio/GQB-3780-2022; Bravo, Ignacio G./A-2483-2009; Pimenoff, Ville Nikolai/H-9865-2019
OI Bravo, Ignacio G./0000-0003-3389-3389; Pimenoff, Ville Nikolai/0000-0002-0813-7031
FU European Society of Clinical Microbiology and Infectious Diseases (ESCMID); Aaltonen Foundation (VNP); Otto A. Malmi Foundation (VNP); Sao Paulo Research Foundation (FAPESP) [2011/24035-2, 2012/23290-1]; Europen Research Council [647916]; Fundacao de Amparo a Pesquisa do Estado de Sao Paulo (FAPESP) [11/24035-2, 12/23290-1] Funding Source: FAPESP
FX This research was financially supported by The European Society of Clinical Microbiology and Infectious Diseases (ESCMID Research Grant 2015 VNP), Aaltonen Foundation (VNP), Otto A. Malmi Foundation (VNP), The Sao Paulo Research Foundation (FAPESP grant #2011/24035-2 and #2012/23290-1, CMO) and the Europen Research Council (Contract #647916, IGB). No agency had any role in the interpretation of the results or in the preparation of the article. Alexander Herbig is warmly thanked for the help in the analysis of the metagenomic data of the European Copper Age glacier mummy. Xavier Bosch, Samuel Alizon and Yannis Michalakis are thanked for helpful comments and review of an initial version of the article.
CR Abi-Rached L, 2011, SCIENCE, V334, P89, DOI 10.1126/science.1209202
Altshuler DM, 2015, NATURE, V526, P68, DOI 10.1038/nature15393
Antonsson A, 2003, J CLIN MICROBIOL, V41, P2509, DOI 10.1128/JCM.41.6.2509-2514.2003
Ashford RW, 2000, J MED MICROBIOL, V49, P771, DOI 10.1099/0022-1317-49-9-771
Baele G, 2013, MOL BIOL EVOL, V30, P239, DOI 10.1093/molbev/mss243
Baele G, 2012, MOL BIOL EVOL, V29, P2157, DOI 10.1093/molbev/mss084

Berger SA, 2011, BIOINFORMATICS, V27, P2068, DOI 10.1093/bioinformatics/btr320
Bernard Hans-Ulrich, 1994, Trends in Microbiology, V2, P140, DOI 10.1016/0966-842X(94)90602-5
Berumen J, 2001, JNCI-J NATL CANCER I, V93, P1325, DOI 10.1093/jnci/93.17.1325
Bouvard V., 2009, Lancet Oncology, V10, P321, DOI [DOI 10.1016/S1470-2045(09)70096-8, 10.1016/S1470-2045(09)70096-8, DOI 10.1016/S1470-2045, 10.1016/s1470-2045, 10.1016/S1470-2045]
Bravo Ignacio G., 2015, Evolution Medicine and Public Health, P32, DOI 10.1093/emph/eov003
Browning SR, 2007, AM J HUM GENET, V81, P1084, DOI 10.1086/521987
Bruni L, 2010, J INFECT DIS, V202, P1789, DOI 10.1086/657321
Buck LT, 2014, CURR BIOL, V24, pR214, DOI 10.1016/j.cub.2013.12.048
Burk RD, 2003, CANCER RES, V63, P7215
Bzhalava D, 2015, VIROLOGY, V476, P341, DOI 10.1016/j.virol.2014.12.028
Cann HM, 2002, SCIENCE, V296, P261
Castresana J, 2000, MOL BIOL EVOL, V17, P540, DOI 10.1093/oxfordjournals.molbev.a026334
Chesson HW, 2014, SEX TRANSM DIS, V41, P660, DOI 10.1097/OLQ.0000000000000193
Cornet I, 2013, BRIT J CANCER, V108, P240, DOI 10.1038/bjc.2012.508
Cornet I, 2012, J VIROL, V86, P6855, DOI 10.1128/JVI.00483-12
Dannemann M, 2016, AM J HUM GENET, V98, P22, DOI 10.1016/j.ajhg.2015.11.015
Darriba D, 2012, NAT METHODS, V9, P772, DOI 10.1038/nmeth.2109
Doorbar J, 2012, VACCINE, P1
Drummond AJ, 2005, MOL BIOL EVOL, V22, P1185, DOI [10.1093/molbev/msi103, 10.1093/molbev/mss075]
Edgar RC, 2004, NUCLEIC ACIDS RES, V32, P1792, DOI 10.1093/nar/gkh340
Einstein MH, 2009, LANCET INFECT DIS, V9, P347, DOI 10.1016/S1473-3099(09)70108-2
Freitas LB, 2014, PLOS ONE, V9, DOI 10.1371/journal.pone.0100746
Fu QM, 2014, NATURE, V514, P445, DOI 10.1038/nature13810
García-Vallvé S, 2005, TRENDS MICROBIOL, V13, P514, DOI 10.1016/j.tim.2005.09.003
Gottschling M, 2007, MOL BIOL EVOL, V24, P1242, DOI 10.1093/molbev/msm039
Gottschling M, 2011, MOL BIOL EVOL, V28, P2101, DOI 10.1093/molbev/msr030
Green RE, 2010, SCIENCE, V328, P710, DOI 10.1126/science.1188021
HO L, 1993, J VIROL, V67, P6413, DOI 10.1128/JVI.67.11.6413-6423.1993
Holmes EC, 2008, ANNU REV MICROBIOL, V62, P307, DOI 10.1146/annurev.micro.62.081307.162912
Hurles ME, 2002, GENETICS, V160, P289
Johnson KP, 2003, SYST BIOL, V52, P37, DOI 10.1080/10635150390132704
Kapusinszky B, 2015, J VIROL, V89, P8152, DOI 10.1128/JVI.00671-15
Katoh Kazutaka, 2013, Mol Biol Evol, V30, P772, DOI 10.1093/molbev/mst010
Kidd JM, 2007, PLOS GENET, V3, DOI 10.1371/journal.pgen.0030063
Pond SLK, 2006, MOL BIOL EVOL, V23, P1891, DOI 10.1093/molbev/msl051
Kuhlwilm M, 2016, NATURE, V530, P429, DOI 10.1038/nature16544

Kumar S, 2002, P NATL ACAD SCI USA, V99, P803, DOI 10.1073/pnas.022629899
Lartillot N, 2004, MOL BIOL EVOL, V21, P1095, DOI 10.1093/molbev/msh112
Lazaridis I, 2014, NATURE, V513, P409, DOI 10.1038/nature13673
Librado P, 2009, BIOINFORMATICS, V25, P1451, DOI 10.1093/bioinformatics/btp187
Lippold Sebastian, 2014, Investig Genet, V5, P13, DOI 10.1186/2041-2223-5-13
Liu W, 2015, NATURE, V526, P696, DOI 10.1038/nature15696
Lopera EA, 2014, INFECT GENET EVOL, V28, P339, DOI 10.1016/j.meegid.2014.10.014
López-Bueno A, 2016, J VIROL, V90, P8768, DOI 10.1128/JVI.01369-16
Ma YF, 2014, J VIROL, V88, P4786, DOI 10.1128/JVI.00093-14
Maixner F, 2014, PLOS ONE, V9, DOI 10.1371/journal.pone.0099994
Mendez FL, 2016, AM J HUM GENET, V98, P728, DOI 10.1016/j.ajhg.2016.02.023
Mendoza L, 2013, INT J GYNECOL OBSTET, V122, P44, DOI 10.1016/j.ijgo.2013.01.020
Meyer M, 2012, SCIENCE, V338, P222, DOI 10.1126/science.1224344
Molden T, 2016, PAPILLOMAVIRUS RES, V2, P153, DOI 10.1016/j.pvr.2016.05.002
Moscicki AB, 2012, VACCINE, V30, pF24, DOI 10.1016/j.vaccine.2012.05.089
Picconi MA, 2003, J MED VIROL, V69, P546, DOI 10.1002/jmv.10343
Pond SK, 2005, MOL BIOL EVOL, V22, P2375, DOI 10.1093/molbev/msi232
Pond SLK, 2005, MOL BIOL EVOL, V22, P1208, DOI 10.1093/molbev/msi105
Prüfer K, 2014, NATURE, V505, P43, DOI 10.1038/nature12886
Purcell S, 2007, AM J HUM GENET, V81, P559, DOI 10.1086/519795
Pybus OG, 2000, GENETICS, V155, P1429
Qin PF, 2015, MOL BIOL EVOL, V32, P2665, DOI 10.1093/molbev/msv141
Qmichou Z, 2013, BMC INFECT DIS, V13, DOI 10.1186/1471-2334-13-378
Rector A, 2007, GENOME BIOL, V8, DOI 10.1186/gb-2007-8-4-r57
Reed DL, 2004, PLOS BIOL, V2, P1972, DOI 10.1371/journal.pbio.0020340
Reich D, 2010, NATURE, V468, P1053, DOI 10.1038/nature09710
ROBINSON DF, 1981, MATH BIOSCI, V53, P131, DOI 10.1016/0025-5564(81)90043-2
Sankararaman S, 2014, NATURE, V507, P354, DOI 10.1038/nature12961
Sawyer S, 2015, P NATL ACAD SCI USA, V112, P15696, DOI 10.1073/pnas.1519905112
Scally A, 2012, NAT REV GENET, V13, P745, DOI 10.1038/nrg3295
Schiffman M, 2010, CANCER RES, V70, P3159, DOI 10.1158/0008-5472.CAN-09-4179
Shah SD, 2010, MOL BIOL EVOL, V27, P1301, DOI 10.1093/molbev/msq015
Sharp PM, 2002, CELL, V108, P305, DOI 10.1016/S0092-8674(02)00639-6
Shimodaira H, 1999, MOL BIOL EVOL, V16, P1114, DOI 10.1093/oxfordjournals.molbev.a026201
Soria-Carrasco V, 2007, BIOINFORMATICS, V23, P2954, DOI 10.1093/bioinformatics/btm466
Stamatakis A, 2014, BIOINFORMATICS, V30, P1312, DOI 10.1093/bioinformatics/btu033
Stringer CB, 2015, P NATL ACAD SCI USA, V112, P15542, DOI 10.1073/pnas.1522477112
Tan SE, 2013, SEX HEALTH, V10, P18, DOI 10.1071/SH11179

   Vernot B, 2016, SCIENCE, V352, P235, DOI 10.1126/science.aad9416
   Vernot B, 2015, AM J HUM GENET, V96, P448, DOI
10.1016/j.ajhg.2015.01.006
   Vernot B, 2014, SCIENCE, V343, P1017, DOI 10.1126/science.1245938
   Villa LL, 2000, J GEN VIROL, V81, P2959, DOI 10.1099/0022-1317-81-12-2959
   Wertheim JO, 2014, MOL BIOL EVOL, V31, P2356, DOI
10.1093/molbev/msu185
   Wertheim JO, 2011, MOL BIOL EVOL, V28, P3355, DOI
10.1093/molbev/msr170
   WRIGHT S, 1951, ANN EUGENIC, V15, P323
   Xi LF, 2006, J NATL CANCER I, V98, P1045, DOI 10.1093/jnci/djj297
   Zuna RE, 2011, INFECT AGENTS CANCER, V6, DOI 10.1186/1750-9378-6-19
NR 89
TC 64
Z9 68
U1 2
U2 31
PU OXFORD UNIV PRESS
PI OXFORD
PA GREAT CLARENDON ST, OXFORD OX2 6DP, ENGLAND
SN 0737-4038
EI 1537-1719
J9 MOL BIOL EVOL
JI Mol. Biol. Evol.
PD JAN
PY 2017
VL 34
IS 1
BP 4
EP 19
DI 10.1093/molbev/msw214
PG 16
WC Biochemistry & Molecular Biology; Evolutionary Biology; Genetics &
   Heredity
WE Science Citation Index Expanded (SCI-EXPANDED)
SC Biochemistry & Molecular Biology; Evolutionary Biology; Genetics &
   Heredity
GA EO2FG
UT WOS:000396511400001
PM 28025273
OA Green Published, hybrid
DA 2025-05-07
ER

PT J
AU Tan, CY
   Surana, N
AF Tan, Chin Yee
   Surana, Neil
TI An immunomodulatory human commensal coordinately regulates inflammatory
   and metabolic disease
SO JOURNAL OF IMMUNOLOGY

LA English
DT Meeting Abstract
CT Immunology Meeting
CY MAY 06-10, 2022
CL Portland, OR
FU National University of Singapore; Triangle Center for Evolutionary
Medicine
FX Supported by a Development Award from the National University of
Singapore and a Graduate Student Award from the Triangle Center for
Evolutionary Medicine
NR 0
TC 0
Z9 0
U1 0
U2 2
PU AMER ASSOC IMMUNOLOGISTS
PI BETHESDA
PA 9650 ROCKVILLE PIKE, BETHESDA, MD 20814 USA
SN 0022-1767
EI 1550-6606
J9 J IMMUNOL
JI J. Immunol.
PD MAY 1
PY 2022
VL 208
IS 1
SU S
DI 10.4049/jimmunol.208.Supp.113.02
PG 1
WC Immunology
WE Science Citation Index Expanded (SCI-EXPANDED); Conference Proceedings
Citation Index - Science (CPCI-S)
SC Immunology
GA 6R8FC
UT WOS:000892531601151
DA 2025-05-07
ER

PT J
AU Reese, AT
Kartzinel, TR
Petrone, BL
Turnbaugh, PJ
Pringle, RM
David, LA
AF Reese, Aspen T.
Kartzinel, Tyler R.
Petrone, Brianna L.
Turnbaugh, Peter J.
Pringle, Robert M.
David, Lawrence A.
TI Using DNA Metabarcoding To Evaluate the Plant Component of Human
Diets:
a Proof of Concept

SO MSYSTEMS
LA English
DT Article
DE human diet; DNA metabarcoding; trnL(UAA)-P6; diet log
ID DIVERSITY; PATTERNS
AB Dietary intake is difficult to measure reliably in humans because approaches typically rely on self-reporting, which can be incomplete and biased. In field studies of animals, DNA sequencing-based approaches such as metabarcoding have been developed to characterize diets, but such approaches have not previously been widely applied to humans. Here, we present data derived from sequencing of a chloroplast DNA marker (the P6 loop of the trnL [UAA] intron) in stool samples collected from 11 individuals consuming both controlled and freely selected diets. The DNA metabarcoding strategy resulted in successful PCR amplification in about 50% of samples, which increased to a 70% success rate in samples from individuals eating a controlled plant-rich diet. Detection of plant taxa among sequenced samples yielded a recall of 0.86 and a precision of 0.55 compared to a written diet record during controlled feeding of plant-based foods. The majority of sequenced plant DNA matched common human food plants, including grains, vegetables, fruits, and herbs prepared both cooked and uncooked. Moreover, DNA metabarcoding data were sufficient to distinguish between baseline and treatment diet arms of the study. Still, the relatively high PCR failure rate and an inability to distinguish some dietary plants at the sequence level using the trnL-P6 marker suggest that future methodological refinements are necessary. Overall, our results suggest that DNA metabarcoding provides a promising new method for tracking human plant intake and that similar approaches could be used to characterize the animal and fungal components of our omnivorous diets.

IMPORTANCE Current methods for capturing human dietary patterns typically rely on individual recall and as such are subject to the limitations of human memory. DNA sequencing-based approaches, frequently used for profiling nonhuman diets, do not suffer from the same limitations. Here, we used metabarcoding to broadly characterize the plant portion of human diets for the first time. The majority of sequences corresponded to known human foods, including all but one foodstuff included in an experimental plant-rich diet. Metabarcoding could distinguish between experimental diets and matched individual diet records from controlled settings with high accuracy. Because this method is independent of survey language and timing, it could also be applied to geographically and culturally disparate human populations, as well as in retrospective studies involving banked human stool.
C1 [Reese, Aspen T.] Harvard Univ, Cambridge, MA 02138 USA.
[Kartzinel, Tyler R.] Brown Univ, Dept Ecol & Evolutionary Biol, Providence, RI 02912 USA.
[Petrone, Brianna L.] Duke Univ, Med Scientist Training Program, Durham, NC USA.
[Petrone, Brianna L.; David, Lawrence A.] Duke Univ, Dept Mol Genet & Microbiol, Durham, NC 27708 USA.
[Turnbaugh, Peter J.] Univ Calif San Francisco, Dept Microbiol & Immunol, San Francisco, CA 94143 USA.
[Pringle, Robert M.] Princeton Univ, Dept Ecol & Evolutionary Biol, Princeton, NJ 08544 USA.

[David, Lawrence A.] Duke Univ, Ctr Genom & Computat Biol, Durham, NC 27708 USA.
C3 Harvard University; Brown University; Duke University; Duke University;
University of California System; University of California San Francisco;
Princeton University; Duke University
RP David, LA (autor correspondente), Duke Univ, Dept Mol Genet & Microbiol, Durham, NC 27708 USA.; Pringle, RM (autor correspondente), Princeton Univ, Dept Ecol & Evolutionary Biol, Princeton, NJ 08544 USA.; David, LA (autor correspondente), Duke Univ, Ctr Genom & Computat Biol, Durham, NC 27708 USA.
EM rpringle@princeton.edu; lawrence.david@duke.edu
RI Turnbaugh, Peter/JTV-0906-2023; David, Lawrence/HCJ-0062-2022; David, Lawrence/B-1044-2010
OI Turnbaugh, Peter/0000-0002-0888-2875; David, Lawrence/0000-0002-3570-4767; Pringle, Robert/0000-0001-7487-5393; Petrone, Brianna/0000-0003-4778-7595
FU Triangle Center for Evolutionary Medicine; Children's Health and Discovery Initiative
FX L.A.D. acknowledges support from the Triangle Center for Evolutionary Medicine and the Children's Health and Discovery Initiative.
CR [Anonymous], 2019, VEGAN COMMUNITY ECOL
Archer E, 2015, MAYO CLIN PROC, V90, P911, DOI 10.1016/j.mayocp.2015.04.009
Archer E, 2013, PLOS ONE, V8, DOI 10.1371/journal.pone.0076632
Boyer F, 2016, MOL ECOL RESOUR, V16, P176, DOI 10.1111/1755-0998.12428
Budischak SA, 2018, FRONT IMMUNOL, V9, DOI 10.3389/fimmu.2018.02453
Caporaso JG, 2011, P NATL ACAD SCI USA, V108, P4516, DOI 10.1073/pnas.1000080107
Carmody RN, 2009, J HUM EVOL, V57, P379, DOI 10.1016/j.jhevol.2009.02.011
Coissac E, 2012, MOL ECOL, V21, P1834, DOI 10.1111/j.1365-294X.2012.05550.x
Craine JM, 2016, PLOS ONE, V11, DOI 10.1371/journal.pone.0161511
Craine JM, 2015, SCI REP-UK, V5, DOI 10.1038/srep16738
CUMMINGS JH, 1976, AM J CLIN NUTR, V29, P1468, DOI 10.1093/ajcn/29.12.1468
CUMMINGS JH, 1976, GUT, V17, P210, DOI 10.1136/gut.17.3.210
CUNNINGHAM KM, 1991, GUT, V32, P483, DOI 10.1136/gut.32.5.483
David LA, 2014, NATURE, V505, P559, DOI 10.1038/nature12820
De Barba M, 2014, MOL ECOL RESOUR, V14, P306, DOI 10.1111/1755-0998.12188
Deagle BE, 2019, MOL ECOL, V28, P391, DOI 10.1111/mec.14734
Elbrecht V, 2018, PEERJ, V6, DOI 10.7717/peerj.4644
García-Robledo C, 2013, PLOS ONE, V8, DOI 10.1371/journal.pone.0052967
Gebremedhin B, 2016, PLOS ONE, V11, DOI 10.1371/journal.pone.0159133
Kartzinel TR, 2015, P NATL ACAD SCI USA, V112, P8019, DOI 10.1073/pnas.1503283112
Louarn S, 2007, GENET RESOUR CROP EV, V54, P1717, DOI 10.1007/s10722-006-9181-6
Macko SA, 1999, PHILOS T ROY SOC B, V354, P65, DOI 10.1098/rstb.1999.0360
Maixner F, 2018, CURR BIOL, V28, P2348, DOI 10.1016/j.cub.2018.05.067

Pompanon F, 2012, MOL ECOL, V21, P1931, DOI 10.1111/j.1365-294X.2011.05403.x
Pringle RM, 2019, NATURE, V570, P58, DOI 10.1038/s41586-019-1264-6
Proches S, 2008, BIOSCIENCE, V58, P151, DOI 10.1641/B580209
R Core Team, 2017, R LANG ENV STAT COMP
Ross-Ibarra J, 2007, P NATL ACAD SCI USA, V104, P8641, DOI 10.1073/pnas.0700643104
Schnell IB, 2015, MOL ECOL RESOUR, V15, P1289, DOI 10.1111/1755-0998.12402
Schnorr SL, 2014, NAT COMMUN, V5, DOI 10.1038/ncomms4654
Soininen EM, 2009, FRONT ZOOL, V6, DOI 10.1186/1742-9994-6-16
Song SJ, 2016, MSYSTEMS, V1, DOI 10.1128/mSystems.00021-16
Subar AF, 2015, J NUTR, V145, P2639, DOI 10.3945/jn.115.219634
Sugimoto M, 2016, NUTR RES, V36, P818, DOI 10.1016/j.nutres.2016.04.011
Taberlet P, 2007, NUCLEIC ACIDS RES, V35, DOI 10.1093/nar/gkl938
Taberlet P, 2012, MOL ECOL, V21, P2045, DOI 10.1111/j.1365-294X.2012.05470.x
Termote C, 2011, GENET RESOUR CROP EV, V58, P585, DOI 10.1007/s10722-010-9602-4
Thompson FE, 2017, NUTRITION IN THE PREVENTION AND TREATMENT OF DISEASE, 4TH EDITION, P5, DOI 10.1016/B978-0-12-802928-2.00001-1
Tonguç M, 2004, EUPHYTICA, V137, P193, DOI 10.1023/B:EUPH.0000041577.84388.43
Warinner C, 2014, SCI REP-UK, V4, DOI 10.1038/srep07104
Willerslev E, 2014, NATURE, V506, P47, DOI 10.1038/nature12921
Wu GD, 2011, SCIENCE, V334, P105, DOI 10.1126/science.1208344
NR 42
TC 24
Z9 25
U1 2
U2 19
PU AMER SOC MICROBIOLOGY
PI WASHINGTON
PA 1752 N ST NW, WASHINGTON, DC 20036-2904 USA
SN 2379-5077
J9 MSYSTEMS
JI mSystems
PD SEP-OCT
PY 2019
VL 4
IS 5
AR e00458-19
DI 10.1128/mSystems.00458-19
PG 10
WC Microbiology
WE Science Citation Index Expanded (SCI-EXPANDED)
SC Microbiology
GA JS7OM
UT WOS:000500493300017
PM 31594830
OA Green Published, gold
DA 2025-05-07
ER

PT J
AU Paietta, EN
   Kraberger, S
   Custer, JM
   Vargas, KL
   Van Doorslaer, K
   Yoder, AD
   Varsani, A
AF Paietta, Elise N.
   Kraberger, Simona
   Custer, Joy M.
   Vargas, Karla L.
   Van Doorslaer, Koenraad
   Yoder, Anne D.
   Varsani, Arvind
TI Identification of diverse papillomaviruses in captive black-and-white
   ruffed lemurs (Varecia variegata)
SO ARCHIVES OF VIROLOGY
LA English
DT Article
ID CAPSID PROTEIN; SELECTION
AB Papillomaviruses (PVs) are host-species-specific and tissue-specific viruses that infect a diverse array of vertebrate hosts, including humans and non-human primates, with varying pathogenic outcomes. Although primate PVs have been studied extensively, no complete genome sequences of PVs from lemurs have been determined to date. Saliva samples from three critically endangered, captive black-and-white ruffed lemurs (Varecia variegata variegata) at the Duke Lemur Center (USA) were analyzed, using high-throughput sequencing, for the presence of oral papillomaviruses. We identified three PVs from two individuals, one of which had a coinfection with two different PVs. Two of the three PVs share 99.6% nucleotide sequence identity, and we have named these isolates "Varecia variegata papillomavirus 1" (VavPV1). The third PV shares similar to 63% nucleotide sequence identity with VavPV1, and thus, we have named it "Varecia variegata papillomavirus 2" (VavPV2). Based on their E1 + E2 + L1 protein sequence phylogeny, the VavPVs form a distinct clade. This clade likely represents a novel genus, with VavPV1 and VavPV2 belonging to two distinct species. Our findings represent the first complete genome sequences of PVs found in lemuriform primates, with their presence suggesting the potential existence of diverse PVs across the over 100 species of lemurs.
C1 [Paietta, Elise N.; Yoder, Anne D.] Duke Univ, Dept Biol, Durham, NC 27708 USA.
   [Kraberger, Simona; Custer, Joy M.; Vargas, Karla L.; Varsani, Arvind] Arizona State Univ, Biodesign Ctr Fundamental & Appl Microbiom, Ctr Evolut & Med, Tempe, AZ 85287 USA.
   [Kraberger, Simona; Custer, Joy M.; Vargas, Karla L.; Varsani, Arvind] Arizona State Univ, Sch Life Sci, Tempe, AZ 85287 USA.
   [Van Doorslaer, Koenraad] Univ Arizona, Inst BIO5, UA Canc Ctr, Canc Biol Grad Interdisciplinary Program,Genet Gra, Tucson, AZ 85724 USA.
   [Varsani, Arvind] Univ Cape Town, Dept Integrat Biomed Sci, Struct Biol Res Unit, ZA-7925 Cape Town, South Africa.
C3 Duke University; Arizona State University; Arizona State

University-Tempe; Arizona State University; Arizona State
University-Tempe; University of Arizona; University of Cape Town
RP Yoder, AD (autor correspondente), Duke Univ, Dept Biol, Durham, NC 27708 USA.; Varsani, A (autor correspondente), Arizona State Univ, Biodesign Ctr Fundamental & Appl Microbiom, Ctr Evolut & Med, Tempe, AZ 85287 USA.; Varsani, A (autor correspondente), Arizona State Univ, Sch Life Sci, Tempe, AZ 85287 USA.; Varsani, A (autor correspondente), Univ Cape Town, Dept Integrat Biomed Sci, Struct Biol Res Unit, ZA-7925 Cape Town, South Africa.
EM anne.yoder@duke.edu; arvind.varsani@asu.edu
RI Varsani, Arvind/JOZ-5299-2023
OI Varsani, Arvind/0000-0003-4111-2415; Custer, Joy/0000-0001-9963-1133
FU TriCEM (Triangle Center for Evolutionary Medicine), Duke Biology; Sigma
Xi
FX The work described here was supported by TriCEM (Triangle Center for Evolutionary Medicine), Duke Biology, and Sigma Xi grants awarded to ENP.
CR ALTSCHUL SF, 1990, J MOL BIOL, V215, P403, DOI 10.1006/jmbi.1990.9999
Antonsson A, 2002, J VIROL, V76, P12537, DOI 10.1128/JVI.76.24.12537-12542.2002
Barrett MA, 2013, BIOL CONSERV, V157, P409, DOI 10.1016/j.biocon.2012.09.003
Bergin IL, 2013, VET PATHOL, V50, P200, DOI 10.1177/0300985812439725
Bergvall M, 2013, VIROLOGY, V445, P35, DOI 10.1016/j.virol.2013.07.020
Bernard HU, 2010, VIROLOGY, V401, P70, DOI 10.1016/j.virol.2010.02.002
Bolger AM, 2014, BIOINFORMATICS, V30, P2114, DOI 10.1093/bioinformatics/btu170
Buchfink B, 2015, NAT METHODS, V12, P59, DOI 10.1038/nmeth.3176
Buck CB, 2013, VIROLOGY, V445, P169, DOI 10.1016/j.virol.2013.05.038
Bushnell B, 2014, No. LBNL-7065E
Capella-Gutiérrez S, 2009, BIOINFORMATICS, V25, P1972, DOI 10.1093/bioinformatics/btp348
Chen ZG, 2019, FRONT MICROBIOL, V10, DOI 10.3389/fmicb.2019.02093
Chen ZG, 2009, VIROLOGY, V393, P304, DOI 10.1016/j.virol.2009.07.012
D'arc M, 2020, VIRUS EVOL, V6, DOI 10.1093/ve/veaa036
Darriba D, 2011, BIOINFORMATICS, V27, P1164, DOI 10.1093/bioinformatics/btr088
Silvestre RVD, 2016, GENOME ANNOUNCEMENTS, V4, DOI 10.1128/genomeA.00725-16
Hoffmann M, 2019, PATHOGENS, V8, DOI 10.3390/pathogens8010013
Joh J, 2009, J GEN VIROL, V90, P987, DOI 10.1099/vir.0.006544-0
Katoh K, 2013, MOL BIOL EVOL, V30, P772, DOI 10.1093/molbev/mst010
Letunic I, 2021, NUCLEIC ACIDS RES, V49, pW293, DOI 10.1093/nar/gkab301
Li DH, 2015, BIOINFORMATICS, V31, P1674, DOI 10.1093/bioinformatics/btv033
Long T, 2022, PLOS PATHOG, V18, DOI 10.1371/journal.ppat.1010444
McBride AA, 2013, VIROLOGY, V445, P57, DOI 10.1016/j.virol.2013.06.006
Middleton K, 2003, J VIROL, V77, P10186, DOI 10.1128/JVI.77.19.10186-10201.2003
Minh BQ, 2020, MOL BIOL EVOL, V37, P1530, DOI 10.1093/molbev/msaa015
Muhire BM, 2014, PLOS ONE, V9, DOI 10.1371/journal.pone.0108277
Rector A, 2013, VIROLOGY, V445, P213, DOI 10.1016/j.virol.2013.05.007

   Roman A, 2013, VIROLOGY, V445, P138, DOI 10.1016/j.virol.2013.04.013
   Tisza MJ, 2021, VIRUS EVOL, V7, DOI 10.1093/ve/veaa100
   Van Doorslaer K, 2018, J GEN VIROL, V99, P989, DOI
10.1099/jgv.0.001105
   Van Doorslaer K, 2017, NUCLEIC ACIDS RES, V45, pD499, DOI
10.1093/nar/gkw879
   Vande Pol SB, 2013, VIROLOGY, V445, P115, DOI
10.1016/j.virol.2013.04.026
   Wang JW, 2013, VIROLOGY, V445, P175, DOI 10.1016/j.virol.2013.04.017
   Willemsen A, 2019, PHILOS T R SOC B, V374, DOI 10.1098/rstb.2018.0303
NR 34
TC 3
Z9 3
U1 0
U2 3
PU SPRINGER WIEN
PI Vienna
PA Prinz-Eugen-Strasse 8-10, A-1040 Vienna, AUSTRIA
SN 0304-8608
EI 1432-8798
J9 ARCH VIROL
JI Arch. Virol.
PD JAN
PY 2023
VL 168
IS 1
AR 13
DI 10.1007/s00705-022-05679-1
PG 6
WC Virology
WE Science Citation Index Expanded (SCI-EXPANDED)
SC Virology
GA 7L0LM
UT WOS:000905667200005
PM 36576610
DA 2025-05-07
ER

PT J
AU Matthews, J
   Rajakumar, B
   Carreon, CK
   Morton, SU
AF Matthews, Jadyn
   Rajakumar, Brammy
   Carreon, Chrystalle Katte
   Morton, Sarah U.
TI Placental-Heart Axis: An Evolutionary Perspective
SO INTERNATIONAL JOURNAL OF MOLECULAR SCIENCES
LA English
DT Review
DE placenta; evolutionary biology; congenital heart disease
ID FETAL-GROWTH; SOCIAL DETERMINANTS; FETUSES; HEALTH; ENVIRONMENT;
   MEDICINE; INFANTS; IMPROVE; DISEASE; SIZE

AB To maintain its development, the growing fetus is directly dependent on the placenta, an organ that acts as both a modulator and mediator. As an essential component of pregnancy that is derived from both maternal and fetal tissues, the placenta facilitates the passage of all oxygen and nutrients from the expecting parent to their fetuses. Further, the placenta conveys multiple impacts of the maternal environment to the growing fetus. The timing of placental development parallels that of the fetal cardiovascular system, and placental anomalies are implicated as a potential cause of congenital heart disease. For example, congenital heart disease is more common in pregnancies complicated by maternal preeclampsia, a condition characterized by placental dysfunction. Given the placenta's intermediary links to the maternal environment and fetal health outcomes, it is an emerging focus of evolutionary medicine, which seeks to understand how interactions between humans and the environment affect our biology and give rise to disease. The present review provides an overview of the evolutionary and developmental courses of the placenta as well as their implications on infant health.
C1 [Matthews, Jadyn; Rajakumar, Brammy; Morton, Sarah U.] Boston Childrens Hosp, Dept Pediat, Div Newborn Med, Boston, MA 02115 USA.
[Carreon, Chrystalle Katte] Boston Childrens Hosp, Dept Pathol, Boston, MA 02115 USA.
[Carreon, Chrystalle Katte] Harvard Med Sch, Dept Pathol, Boston, MA 02115 USA.
[Morton, Sarah U.] Harvard Med Sch, Dept Pediat, Boston, MA 02115 USA.
C3 Harvard University; Harvard University Medical Affiliates; Boston Children's Hospital; Harvard University; Harvard University Medical Affiliates; Boston Children's Hospital; Harvard University; Harvard Medical School; Harvard University; Harvard Medical School
RP Morton, SU (autor correspondente), Boston Childrens Hosp, Dept Pediat, Div Newborn Med, Boston, MA 02115 USA.; Morton, SU (autor correspondente), Harvard Med Sch, Dept Pediat, Boston, MA 02115 USA.
EM jadynmatthews@college.harvard.edu; brajakumar@hms.harvard.edu; katte.carreon@childrens.harvard.edu; sarah.morton@childrens.harvard.edu
OI Morton, Sarah/0000-0002-7816-2646; Carreon, Chrystalle Katte/0000-0003-2152-7998
FU National Institutes of Health; American Heart Association Career Development Award (SUM); [K08HL157653]
FX This work was supported by an American Heart Association Career Development Award (SUM), and the National Institutes of Health (K08HL157653, SUM).
CR [Anonymous], 2023, Diabetes Prevalence Expected to Double Globally by 2050
[Anonymous], 2022, Air pollution and your health
Basile AJ, 2021, EVOL MED PUBLIC HLTH, V9, P157, DOI 10.1093/emph/eoab010
Benagiano G, 2023, INT J MOL SCI, V24, DOI 10.3390/ijms242015420
Bhatt J, 2018, Your Health|AHA News
Braveman P, 2014, PUBLIC HEALTH REP, V129, P19, DOI 10.1177/00333549141291S206
Bronson SL, 2016, NEUROPSYCHOPHARMACOL, V41, P207, DOI 10.1038/npp.2015.231
Burton GJ, 2015, AM J OBSTET GYNECOL, V213, DOI 10.1016/j.ajog.2015.07.050

Butler SC, 2019, CONGENIT HEART DIS, V14, P236, DOI 10.1111/chd.12686
Caruso M, 2012, INT J MOL CELL MED, V1, P64
Challa HJ., 2024, STATPEARLS
Cherubini M, 2021, MICROMACHINES-BASEL, V12, DOI 10.3390/mi12080884
Clouchoux C, 2013, CEREB CORTEX, V23, P2932, DOI 10.1093/cercor/bhs281
Co-Vu J, 2017, ECHOCARDIOGR-J CARD, V34, P1822, DOI 10.1111/echo.13722
Cole LA, 2010, REPROD BIOL ENDOCRIN, V8, DOI 10.1186/1477-7827-8-102
Courtney JA, 2018, FRONT PHYSIOL, V9, DOI 10.3389/fphys.2018.01045
Demianczyk A., 2024, Pediatrics, DOI [10.1007/s40746-024-00303-7, DOI 10.1007/S40746-024-00303-7]
Dorey F., 2018, How do We Affect Our Evolution?-The Australian Museum
Ernst L.M., 2019, COLOR ATLAS HUMAN FE, DOI [10.1007/978-3-030-11425-1, DOI 10.1007/978-3-030-11425-1]
Falcón M, 2002, ARCH ENVIRON HEALTH, V57, P598, DOI 10.1080/00039890209602094
Garcia-Martin I, 2021, INT J MOL SCI, V22, DOI 10.3390/ijms22147458
Gluckman PD, 2011, EVOL APPL, V4, P249, DOI 10.1111/j.1752-4571.2010.00164.x
Herrick EJ, 2024, StatPearls
Jiang XQ, 2016, J THORAC DIS, V8, pE31, DOI 10.3978/j.issn.2072-1439.2015.11.50
Josowitz Rebecca, 2023, Neoreviews, V24, pe569, DOI 10.1542/neo.24-9-e569
Khong TY, 2016, ARCH PATHOL LAB MED, V140, P698, DOI 10.5858/arpa.2015-0225-CC
Lee FT, 2021, TRANSL PEDIATR, V10, P2197, DOI 10.21037/tp-20-226
Lee JG, 2024, NAT COMMUN, V15, DOI 10.1038/s41467-024-45647-8
Leon RL, 2022, PEDIATR RES, V91, P787, DOI 10.1038/s41390-021-01521-7
Lieberman M.D., 2013, The Story of the Human Body, P1
Limperopoulos C, 2010, CIRCULATION, V121, P26, DOI 10.1161/CIRCULATIONAHA.109.865568
Marable CA, 2022, FRONT NEUROSCI-SWITZ, V16, DOI 10.3389/fnins.2022.1017953
Masoller N, 2016, FETAL DIAGN THER, V39, P269, DOI 10.1159/000439527
Matthiesen NB, 2016, CIRCULATION, V134, P1546, DOI 10.1161/CIRCULATIONAHA.116.021793
Miremberg H, 2019, PRENATAL DIAG, V39, P962, DOI 10.1002/pd.5515
Morton SU, 2022, NAT REV CARDIOL, V19, P26, DOI 10.1038/s41569-021-00587-4
Natterson-Horowitz B, 2023, Front Sci, V1, DOI 10.3389/fsci.2023.997136
Naugler CT, 2008, CAN FAM PHYSICIAN, V54, P1265
Nesse R., 1999, Life Sci. Res. Life Sci. Res. Publ. China, V3, P1
Olmos-Ortiz A, 2021, INT J MOL SCI, V22, DOI 10.3390/ijms22158087
Olshaker H, 2018, AM J NEURORADIOL, V39, P1164, DOI 10.3174/ajnr.A5628
Ortinau C, 2013, J PEDIATR-US, V163, P1507, DOI 10.1016/j.jpeds.2013.06.045
Ortinau C, 2012, J THORAC CARDIOV SUR, V143, P1264, DOI 10.1016/j.jtcvs.2011.10.039
Ortinau CM, 2018, NEUROIMAGE-CLIN, V20, P913, DOI 10.1016/j.nicl.2018.09.029
Ortinau CM, 2019, CEREB CORTEX, V29, P3605, DOI 10.1093/cercor/bhy235
Park SG, 2021, CELLS DEV, V165, DOI 10.1016/j.cdev.2021.203663
Perry GH, 2021, ELIFE, V10, DOI 10.7554/eLife.69398

Peyvandi S, 2023, J AM COLL CARDIOL, V81, P253, DOI 10.1016/j.jacc.2022.10.029
Pike NA, 2024, J CARDIOVASC DEV DIS, V11, DOI 10.3390/jcdd11080236
Radford BN, 2023, NAT COMMUN, V14, DOI 10.1038/s41467-023-36740-5
Rajagopalan V, 2018, PRENATAL DIAG, V38, P1042, DOI 10.1002/pd.5374
Rajashekhar G, 2003, J SOC GYNECOL INVEST, V10, P352, DOI 10.1016/S1071-5576(03)00121-7
Ramirez Alcantara J., 2024, StatPearls
Redline RW, 2021, MODERN PATHOL, V34, P1074, DOI 10.1038/s41379-021-00747-4
Roberts RM, 2016, REPRODUCTION, V152, pR179, DOI 10.1530/REP-16-0325
Roseboom TJ, 2011, PLACENTA, V32, P395, DOI 10.1016/j.placenta.2011.03.001
Rosenfeld CS, 2021, J NEUROSCI RES, V99, P271, DOI 10.1002/jnr.24603
Roy A, 2022, BIOMED RES INT, V2022, DOI 10.1155/2022/6751456
Rühli FJ, 2013, BMC MED, V11, DOI 10.1186/1741-7015-11-115
Rutherford JN, 2009, AM J HUM BIOL, V21, P745, DOI 10.1002/ajhb.20923
Sandovici I, 2012, REPROD BIOMED ONLINE, V25, P68, DOI 10.1016/j.rbmo.2012.03.017
Schlatterer SD, 2019, SCI REP-UK, V9, DOI 10.1038/s41598-019-40894-y
Scott RL, 2022, P NATL ACAD SCI USA, V119, DOI 10.1073/pnas.2210633119
Sferruzzi-Perri AN, 2023, EXP PHYSIOL, V108, P371, DOI 10.1113/EP090442
Sood E, 2024, CIRCULATION, V149, pe997, DOI 10.1161/CIR.0000000000001211
Stanek J, 2023, J PERINAT MED, V51, P704, DOI 10.1515/jpm-2022-0478
Sun LQ, 2015, CIRCULATION, V131, P1313, DOI 10.1161/CIRCULATIONAHA.114.013051
Verheugt CL, 2010, EUR HEART J, V31, P1220, DOI 10.1093/eurheartj/ehq032
Wang Y., 2010, Vascular Biology of the Placenta, DOI [10.4199/C00016ED1V01Y201008ISP009, DOI 10.4199/C00016ED1V01Y201008ISP009]
Ware J, 2020, CARDIOL YOUNG, V30, P1609, DOI 10.1017/S1047951120003534
Wildman DE, 2011, PLACENTA, V32, pS142, DOI 10.1016/j.placenta.2011.01.005
Williams CY, 2023, FRONT ENDOCRINOL, V14, DOI 10.3389/fendo.2023.1100724
Wilsterman K, 2023, P NATL ACAD SCI USA, V120, DOI 10.1073/pnas.2218049120
Wong P, 2018, PAED CHILD HEALT-CAN, V23, P92, DOI 10.1093/pch/pxx146
Yasuhara J, 2021, TRANSL PEDIATR, V10, P2366, DOI 10.21037/tp-21-297
Zhang FF, 2020, BMJ-BRIT MED J, V369, DOI 10.1136/bmj.m2511
NR 76
TC 1
Z9 1
U1 5
U2 5
PU MDPI
PI BASEL
PA ST ALBAN-ANLAGE 66, CH-4052 BASEL, SWITZERLAND
SN 1661-6596
EI 1422-0067
J9 INT J MOL SCI

JI Int. J. Mol. Sci.
PD OCT
PY 2024
VL 25
IS 20
AR 11212
DI 10.3390/ijms252011212
PG 12
WC Biochemistry & Molecular Biology; Chemistry, Multidisciplinary
WE Science Citation Index Expanded (SCI-EXPANDED)
SC Biochemistry & Molecular Biology; Chemistry
GA K1U7M
UT WOS:001341807400001
PM 39456993
OA gold
DA 2025-05-07
ER

PT J
AU Painter, DT
   Daniels, BC
   Jost, J
AF Painter, Deryc T.
   Daniels, Bryan C.
   Jost, Juergen
TI Network Analysis for the Digital Humanities: Principles, Problems, Extensions
SO ISIS
LA English
DT Article
ID CO-AUTHORSHIP NETWORKS; CENTRALITY
AB Traditional historical scholarship struggles to keep up with the rapid pace of modern scientific publication trends. Even focusing on a particular scientific field, the rate of new publications far outpaces even the most studious historian's research capacity. This essay summarizes an approach to this problem that uses computational techniques of network analysis. As a complement to close analysis of particular documents, network analysis can give a large-scale perspective on the history of science, identifying relational patterns across a vast number of documents that might otherwise require an entire career to digest. To demonstrate the power of the approach, the essay applies network theory to a corpus of publications in evolutionary medicine. Four distinct networks, including those focused on authors, keywords, and citations, quickly unearth a range of relevant historical information. The essay illustrates how interpretable historical conclusions are drawn from a variety of quantitative metrics. The aim is to provide an overview of network techniques for historians looking to add robust network analysis to their research repertoire.
C1 [Painter, Deryc T.] Arizona State Univ, Laubichler Lab Computat HPS, Tempe, AZ 85287 USA.
   [Painter, Deryc T.; Daniels, Bryan C.] Arizona State Univ, ASU SFI Ctr Bio Social Complex Syst, Tempe, AZ 85281 USA.
   [Jost, Juergen] Max Planck Inst Math Sci, Inselstr 22, D-04103 Leipzig, Germany.

C3 Arizona State University; Arizona State University-Tempe; Arizona State
University; Arizona State University-Tempe; Max Planck Society
RP Painter, DT (autor correspondente), Arizona State Univ, Laubichler Lab Computat HPS, Tempe, AZ 85287 USA.; Painter, DT (autor correspondente), Arizona State Univ, ASU SFI Ctr Bio Social Complex Syst, Tempe, AZ 85281 USA.
EM deryc.painter@asu.edu; bryan.daniels.1@asu.edu; jjost@mis.mpg.de
RI Daniels, Bryan/G-3615-2012; Painter, Deryc/P-8605-2019
OI Daniels, Bryan/0000-0002-5870-1059; Painter, Deryc T./0000-0001-8326-5407
CR Ahuja G, 2012, ORGAN SCI, V23, P434, DOI 10.1287/orsc.1110.0695
Albert R, 2002, REV MOD PHYS, V74, P47, DOI 10.1103/RevModPhys.74.47
Amaral LAN, 2000, P NATL ACAD SCI USA, V97, P11149, DOI 10.1073/pnas.200327197
[Anonymous], 1999, EVOLUTIONARY MED
Atkins S., 1992, Literary & Linguistic Computing, V7, P1, DOI 10.1093/llc/7.1.1
Baker Paul, 2006, BAKER BROWN CORPUS G
Banerjee A, 2008, LINEAR ALGEBRA APPL, V428, P3015, DOI 10.1016/j.laa.2008.01.029
Banerjee A, 2009, DISCRETE APPL MATH, V157, P2425, DOI 10.1016/j.dam.2008.06.033
Barabási AL, 2003, SCI AM, V288, P60, DOI 10.1038/scientificamerican0503-60
Barabási AL, 2002, PHYSICA A, V311, P590, DOI 10.1016/S0378-4371(02)00736-7
Barthélemy M, 2004, EUR PHYS J B, V38, P163, DOI 10.1140/epjb/e2004-00111-4
Borgatti SP, 2005, SOC NETWORKS, V27, P55, DOI 10.1016/j.socnet.2004.11.008
Boyack KW, 2010, J AM SOC INF SCI TEC, V61, P2389, DOI 10.1002/asi.21419
Carley Kathleen M., 2018, ENCY SOCIAL NETWORK, P1
Chin A., 2012, Proceedings of the 2012 32nd International Conference on Distributed Computing Systems Workshops (ICDCS Workshops), P79, DOI 10.1109/ICDCSW.2012.56
Couto T, 2006, OPENING INFORMATION HORIZONS, P75
Crawford W., 2015, Doing corpus linguistics
Cugmas Marjan, 2017, ARXIV171100770
Donner RV, 2011, INT J BIFURCAT CHAOS, V21, P1019, DOI 10.1142/S0218127411029021
Forman R, 2003, DISCRETE COMPUT GEOM, V29, P323, DOI 10.1007/s00454-002-0743-x
Fortunato S, 2010, PHYS REP, V486, P75, DOI 10.1016/j.physrep.2009.11.002
Glanzel Wolfgang, 2005, MEASURING EVALUATING, P695
Gluckman PeterD., 2009, Principles of evolutionary medicine
GRANOVETTER MS, 1973, AM J SOCIOL, V78, P1360, DOI 10.1086/225469
Gu Nini, 2016, CHICAGO POLICY REV O
Jost J., 2005, Dynamical Systems: Examples of Complex Behaviour
Jost J., 2017, RIEMANNIAN GEOMETRY, DOI [10.1007/978-3-319-61860-9, DOI 10.1007/978-3-319-61860-9]
KESSLER MM, 1963, AM DOC, V14, P10, DOI 10.1002/asi.5090140103

Kumar S, 2015, ASLIB J INFORM MANAG, V67, P55, DOI 10.1108/AJIM-09-2014-0116
Li EY, 2013, RES POLICY, V42, P1515, DOI 10.1016/j.respol.2013.06.012
Li K, 2018, SCIENTOMETRICS, V115, P1, DOI 10.1007/s11192-017-2622-5
Liu RL, 2017, SCIENTOMETRICS, V110, P915, DOI 10.1007/s11192-016-2196-7
Marwan N, 2009, PHYS LETT A, V373, P4246, DOI 10.1016/j.physleta.2009.09.042
Moreno JL, 1938, SOCIOMETRY, V1, P342, DOI 10.2307/2785588
Nesse, 2012, WHY WE GET SICK NEW
Nesse R.M., 2018, EvMed Network
Newman M. E. J., SOCIAL NETWORKS, P39
Newman MEJ, 2004, P NATL ACAD SCI USA, V101, P5200, DOI 10.1073/pnas.0307545100
Newman MEJ, 2001, P NATL ACAD SCI USA, V98, P404, DOI 10.1073/pnas.021544898
Painter Deryc T., THEORY BIOSCIENCES
Pontecorvi Matteo, 2011, J MATH SOCIOL, V25, P163
Portenoy J., 2017, Frontiers in Research Metrics and Analytics, V2, P8, DOI 10.3389/frma.2017.00008
Reppen Randi, 2010, ROUTLEDGE HDB CORPUS, P59
Saucan E, 2018, MATCH-COMMUN MATH CO, V80, P605
Scott Mike., 2008, WordSmith Tools Version 5, P122
Sedighi M, 2016, LIBR REV, V65, P52, DOI 10.1108/LR-07-2015-0075
Shannon P, 2003, GENOME RES, V13, P2498, DOI 10.1101/gr.1239303
Sharma S., 2015, iConference
Skolarus TA, 2017, IMPLEMENT SCI, V12, DOI 10.1186/s13012-017-0628-2
Sreejith RP, 2017, CHAOS SOLITON FRACT, V101, P50, DOI 10.1016/j.chaos.2017.05.021
TRAVERS J, 1969, SOCIOMETRY, V32, P425, DOI 10.2307/2786545
Van Eck Nees Jan, 2009, VOSVIEWER MANUAL
Veling A, 1999, IJCAI-99: PROCEEDINGS OF THE SIXTEENTH INTERNATIONAL JOINT CONFERENCE ON ARTIFICIAL INTELLIGENCE, VOLS 1 & 2, P694
Watts DJ, 1998, NATURE, V393, P440, DOI 10.1038/30918
WILLIAMS GC, 1991, Q REV BIOL, V66, P1, DOI 10.1086/417048
Yang Y, 2008, PHYSICA A, V387, P1381, DOI 10.1016/j.physa.2007.10.055
Yi S, 2012, SCIENTOMETRICS, V90, P1015, DOI 10.1007/s11192-011-0560-1
Zhao Dangzhi, 2018, J ASSOC INF SCI TECH, V85, P348
NR 58
TC 13
Z9 15
U1 3
U2 31
PU UNIV CHICAGO PRESS
PI CHICAGO
PA 1427 E 60TH ST, CHICAGO, IL 60637-2954 USA
SN 0021-1753
EI 1545-6994
J9 ISIS
JI Isis
PD SEP 1
PY 2019
VL 110
IS 3

BP 538
EP 554
DI 10.1086/705532
PG 17
WC History & Philosophy Of Science
WE Science Citation Index Expanded (SCI-EXPANDED); Social Science Citation Index (SSCI); Arts & Humanities Citation Index (A&HCI)
SC History & Philosophy of Science
GA IU7VX
UT WOS:000483792500008
OA Bronze
DA 2025-05-07
ER

PT J
AU Suhail, Y
   Afzal, J
   Kshitiz
AF Suhail, Yasir
   Afzal, Junaid
   Kshitiz
TI Evolved Resistance to Placental Invasion Secondarily Confers Increased Survival in Melanoma Patients
SO JOURNAL OF CLINICAL MEDICINE
LA English
DT Article
DE melanoma metastasis; stromal invasion; cancer dissemination; cancer associated fibroblasts; invasibility; placentation; cancer microenvironment
ID CANCER; EXPRESSION; FIBROBLASTS; METASTASIS; BIOLOGY; OBESITY; STROMA; MODEL
AB Mammals exhibit large differences in rates of cancer malignancy, even though the tumor formation rates may be similar. In placental mammals, rates of malignancy correlate with the extent of placental invasion. Our Evolved Levels of Invasibility (ELI) framework links these two phenomena identifying genes that potentially confer resistance in stromal fibroblasts to limit invasion, from trophoblasts in the endometrium, and from disseminating melanoma in the skin. Herein, using patient data from The Cancer Genome Atlas (TCGA), we report that these anti-invasive genes may be crucial in melanoma progression in human patients, and that their loss is correlated with increased cancer spread and lowered survival. Our results suggest that, surprisingly, these anti-invasive genes, which have lower expression in humans compared to species with non-invasive placentation, may potentially prevent stromal invasion, while a further reduction in their levels increases the malignancy and lethality of melanoma. Our work links evolution, comparative biology, and cancer progression across tissues, indicating new avenues for using evolutionary medicine to prognosticate and treat human cancers.
C1 [Suhail, Yasir; Kshitiz] Univ Connecticut, Dept Biomed Engn, Storrs, CT 06269 USA.
   [Suhail, Yasir; Kshitiz] Canc Syst Biol CaSB Yale, Yale West Campus, West Haven, CT 06477 USA.
   [Suhail, Yasir; Kshitiz] Univ Connecticut Hlth, Ctr Cell Anal & Modeling, Farmington, CT 06032 USA.

[Afzal, Junaid] Univ Calif San Francisco, Dept Cardiol, San Francisco, CA 94143 USA.
C3 University of Connecticut; Yale University; University of Connecticut; University of California System; University of California San Francisco
RP Kshitiz (autor correspondente), Univ Connecticut, Dept Biomed Engn, Storrs, CT 06269 USA.; Kshitiz (autor correspondente), Canc Syst Biol CaSB Yale, Yale West Campus, West Haven, CT 06477 USA.; Kshitiz (autor correspondente), Univ Connecticut Hlth, Ctr Cell Anal & Modeling, Farmington, CT 06032 USA.
EM yasir.suhail@uconn.edu; Junaid.Afzal@ucsf.edu; kshitiz@uchc.edu
RI Afzal, Junaid/L-3643-2019; Suhail, Yasir/M-7280-2018; , Kshitiz/B-9143-2013
OI Afzal, Junaid/0000-0002-2877-234X; Suhail, Yasir/0000-0002-1296-9738; , Kshitiz/0000-0003-4241-9427
FU University of Connecticut; National Cancer Institute [1U54CA209992-02]
FX This research was funded by the startup funds from the University of Connecticut, and partly by the National Cancer Institute-funded U54 Center 1U54CA209992-02 subcontracted at UConn.
CR ANDERSEN PK, 1982, ANN STAT, V10, P1100, DOI 10.1214/aos/1176345976
Anderson AM, 2017, SCI REP-UK, V7, DOI 10.1038/srep39873
Boddy AM, 2020, EVOL MED PUBLIC HLTH, P187, DOI 10.1093/emph/eoaa015
D'Souza Alaric W., 2014, Evolution Medicine and Public Health, P136, DOI 10.1093/emph/eou022
Filer Andrew, 2007, Discov Med, V7, P20
Hillers LE, 2018, NEOPLASIA, V20, P1161, DOI 10.1016/j.neo.2018.09.004
Houthuijzen JM, 2018, CANCER METAST REV, V37, P577, DOI 10.1007/s10555-018-9768-3
Hu GM, 2018, J CANCER, V9, P3736, DOI 10.7150/jca.28054
Hutchenreuther J, 2018, J CELL COMMUN SIGNAL, V12, P113, DOI 10.1007/s12079-017-0419-1
Kalluri R, 2016, NAT REV CANCER, V16, P582, DOI 10.1038/nrc.2016.73
Kodigepalli KM, 2018, CELL CYCLE, V17, P2564, DOI 10.1080/15384101.2018.1550955
Korotkevich G., 2021, PREPRINT, DOI [DOI 10.1101/060012, :10.1101/060012]
Krtolica A, 2003, Adv Gerontol, V11, P109
Kshitiz, 2019, NAT ECOL EVOL, V3, P1743, DOI 10.1038/s41559-019-1046-4
Kshitiz, 2020, KSHITIZ CIS REGULATO, DOI [10.1101/2020.09.04.283366, DOI 10.1101/2020.09.04.283366]
LAING RW, 1992, J PATHOL, V166, P420
Li JJ, 2019, CANCER MED-US, V8, P2288, DOI 10.1002/cam4.2063
Li XQ, 2012, CURR PHARM DESIGN, V18, P2404, DOI 10.2174/138161281120922404
Lonsdale J, 2013, NAT GENET, V45, P580, DOI 10.1038/ng.2653
Ma X, 2020, HUMAN VULNERABILITY, DOI [10.1101/2020.08.03.234617, DOI 10.1101/2020.08.03.234617]
Pereira BA, 2019, TRENDS CANCER, V5, P724, DOI 10.1016/j.trecan.2019.09.010
Pothuraju R, 2018, J EXP CLIN CANC RES, V37, DOI 10.1186/s13046-018-0963-4
PRIESTER WA, 1971, JNCI-J NATL CANCER I, V47, P1333

Quail DF, 2019, NAT REV ENDOCRINOL, V15, P139, DOI 10.1038/s41574-018-0126-x
R Core Team, 2020, R: a language and environment for statistical computing. R Foundation for Statistical Computing v.4.3.0
Ren XW, 2018, GENOME BIOL, V19, DOI 10.1186/s13059-018-1593-z
Ruhland MK, 2016, NAT COMMUN, V7, DOI 10.1038/ncomms11762
Sahai E, 2020, NAT REV CANCER, V20, P174, DOI 10.1038/s41568-019-0238-1
Seluanov A, 2018, NAT REV CANCER, V18, P433, DOI 10.1038/s41568-018-0004-9
Shao CJ, 2018, CELL DEATH DIS, V9, DOI 10.1038/s41419-018-0906-1
Shelley AJ, 2019, J CUTAN MED SURG, V23, P229, DOI 10.1177/1203475418800946
Siletz A, 2013, CANCER MICROENVIRON, V6, P91, DOI 10.1007/s12307-012-0121-z
Subramanian A, 2005, P NATL ACAD SCI USA, V102, P15545, DOI 10.1073/pnas.0506580102
Suhail Y, 2019, CELL SYST, V9, P109, DOI 10.1016/j.cels.2019.07.003
Suvà ML, 2019, MOL CELL, V75, P7, DOI 10.1016/j.molcel.2019.05.003
Tang ZF, 2017, NUCLEIC ACIDS RES, V45, pW98, DOI 10.1093/nar/gkx247
Therneau T. M., 2000, STAT BIOL HEALTH
Therneau TM, 1999, LIFETIME DATA ANAL, V5, P99, DOI 10.1023/A:1009691327335
Wagner GP, 2020, EVOL MED PUBLIC HLTH, P211, DOI 10.1093/emph/eoaa024
Wang BS, 2020, TRENDS CANCER, V6, P838, DOI 10.1016/j.trecan.2020.05.004
Weinstein JN, 2013, NAT GENET, V45, P1113, DOI 10.1038/ng.2764
Wu JL, 2017, J CANCER, V8, P852, DOI 10.7150/jca.17845
NR 42
TC 9
Z9 9
U1 0
U2 5
PU MDPI
PI BASEL
PA ST ALBAN-ANLAGE 66, CH-4052 BASEL, SWITZERLAND
EI 2077-0383
J9 J CLIN MED
JI J. Clin. Med.
PD FEB
PY 2021
VL 10
IS 4
AR 595
DI 10.3390/jcm10040595
PG 10
WC Medicine, General & Internal
WE Science Citation Index Expanded (SCI-EXPANDED)
SC General & Internal Medicine
GA QP7YH
UT WOS:000624047800001
PM 33562461
OA gold, Green Published
DA 2025-05-07

ER

PT J
AU Rodriguez-Morales, R
AF Rodriguez-Morales, Roberto
TI Sensing in the dark: Constructive evolution of the lateral line system in blind populations of Astyanax mexicanus
SO ECOLOGY AND EVOLUTION
LA English
DT Review
DE adaptation; cavefish; evolution; hair cells; lateral line; sensory systems
ID HAIR CELL REGENERATION; CAVE FISH; SCHOOLING BEHAVIOR; MATE-CHOICE; MATING PREFERENCES; GENE-EXPRESSION; EYE REGRESSION; SLEEP LOSS; ZEBRAFISH; NEUROMASTS
AB Cave-adapted animals evolve a suite of regressive and constructive traits that allow survival in the dark. Most studies aiming at understanding cave animal evolution have focused on the genetics and environmental underpinnings of regressive traits, with special emphasis on vision loss. Possibly as a result of vision loss, other non-visual sensory systems have expanded and compensated in cave species. For instance, in many cave-dwelling fish species, including the blind cavefish of the Mexican tetra, Astyanax mexicanus, a major non-visual mechanosensory system called the lateral line, compensated for vision loss through morphological expansions. While substantial work has shed light on constructive adaptation of this system, there are still many open questions regarding its developmental origin, synaptic plasticity, and overall adaptive value. This review provides a snapshot of the current state of knowledge of lateral line adaption in A. mexicanus, with an emphasis on anatomy, synaptic plasticity, and behavior. Multiple open avenues for future research in this system, and how these can be leveraged as tools for both evolutionary biology and evolutionary medicine, are discussed.
C1 [Rodriguez-Morales, Roberto] Univ Puerto Rico, Sch Med, Dept Anat & Neurobiol, San Juan, PR 00925 USA.
C3 University of Puerto Rico; University of Puerto Rico Medical Sciences Campus
RP Rodriguez-Morales, R (autor correspondente), Univ Puerto Rico, Sch Med, Dept Anat & Neurobiol, San Juan, PR 00925 USA.
EM roberto.rodriguez19@upr.edu
FU Common Fund [U54 MD007600]
FX Common Fund, Grant/Award Number: U54 MD007600.
CR ABDELLATIF H, 1990, NATURWISSENSCHAFTEN, V77, P237, DOI 10.1007/BF01138492
Alunni A, 2007, J COMP NEUROL, V505, P221, DOI 10.1002/cne.21488
Andersson M, 1996, TRENDS ECOL EVOL, V11, P53, DOI 10.1016/0169-5347(96)81042-1
ARNOLD GP, 1974, BIOL REV, V49, P515, DOI 10.1111/j.1469-185X.1974.tb01173.x
Asaoka R, 2014, ICHTHYOL RES, V61, P49, DOI 10.1007/s10228-013-0373-8
Aspbury AS, 2002, BEHAV ECOL SOCIOBIOL, V51, P238, DOI 10.1007/S00265-001-0443-1
Baek S, 2022, DEV CELL, V57, P799, DOI 10.1016/j.devcel.2022.03.001

Baker CF, 1999, J COMP PHYSIOL A, V184, P519, DOI 10.1007/s003590050351
Behra M, 2012, BMC DEV BIOL, V12, DOI 10.1186/1471-213X-12-6
Berning D, 2023, FRONT ECOL EVOL, V11, DOI 10.3389/fevo.2023.1177532
BLAXTER JHS, 1987, BIOL REV, V62, P471, DOI 10.1111/j.1469-185X.1987.tb01638.x
BLECKMANN H, 1991, J COMP PHYSIOL A, V168, P749
Blin M, 2018, DEV BIOL, V441, P242, DOI 10.1016/j.ydbio.2018.04.019
Bradic M, 2012, BMC EVOL BIOL, V12, DOI 10.1186/1471-2148-12-9
Bradley J. G., 2018, The population ecology and behavior of the cave salamander, Eurycea lucifuga (Rafinesque, 1822) (Paper 3041) [Electronic theses and dissertations], DOI [10.18297/etd/3041, DOI 10.18297/ETD/3041]
Bright M, 2010, OCEANOGR MAR BIOL, V48, P213
Butler JM, 2016, J EXP BIOL, V219, P2781, DOI 10.1242/jeb.133801
Butler JM, 2015, J EXP BIOL, V218, P3284, DOI 10.1242/jeb.125948
CAHN PH, 1968, B MAR SCI, V18, P660
Chin JSR, 2018, DEV BIOL, V441, P319, DOI 10.1016/j.ydbio.2018.05.009
Chitnis AB, 2012, DEV NEUROBIOL, V72, P234, DOI 10.1002/dneu.20962
Chiu LL, 2008, JARO-J ASSOC RES OTO, V9, P178, DOI 10.1007/s10162-008-0118-y
Coffin AB, 2013, JARO-J ASSOC RES OTO, V14, P645, DOI 10.1007/s10162-013-0404-1
Coombs S., 1988, P553
Coombs S., 2014, The Lateral Line System
Coombs S, 2020, J EXP BIOL, V223, DOI 10.1242/jeb.223008
Culver D.C., 1982, P1
Culver DC, 2019, BIO HABIT, P1, DOI 10.1093/oso/9780198820765.001.0001
Dambly-Chaudière C, 2003, BIOL CELL, V95, P579, DOI 10.1016/j.biolcel.2003.10.005
Denton E.J., 1989, P229
Duboué ER, 2012, BRAIN BEHAV EVOLUT, V80, P233, DOI 10.1159/000341403
Duboué ER, 2011, CURR BIOL, V21, P671, DOI 10.1016/j.cub.2011.03.020
Dufton M, 2012, PLOS ONE, V7, DOI 10.1371/journal.pone.0050308
Elipot Y, 2013, CURR BIOL, V23, P1, DOI 10.1016/j.cub.2012.10.044
Espinasa L., 2017, SUBTERR BIOL, V21, P1, DOI [DOI 10.3897/subtbiol.21.11046, 10.3897/subtbiol.21.11046]
Espinasa L, 2022, SUBTERR BIOL, V43, P169, DOI 10.3897/subtbiol.43.79318
Faucher K, 2010, ANIM BEHAV, V79, P679, DOI 10.1016/j.anbehav.2009.12.020
FLOCK A, 1973, J PHYSIOL-LONDON, V235, P591, DOI 10.1113/jphysiol.1973.sp010406
Ghysen A, 2007, GENE DEV, V21, P2118, DOI 10.1101/gad.1568407
Gillespie PG, 2009, CELL, V139, P33, DOI 10.1016/j.cell.2009.09.010
Greenwood AK, 2013, CURR BIOL, V23, P1884, DOI 10.1016/j.cub.2013.07.058
Gross JB, 2016, SYMMETRY-BASEL, V8, DOI 10.3390/sym8110118
Gross JB, 2012, BMC EVOL BIOL, V12, DOI 10.1186/1471-2148-12-105
Gross JB, 2009, PLOS GENET, V5, DOI 10.1371/journal.pgen.1000326
Haas P, 2006, DEV CELL, V10, P673, DOI 10.1016/j.devcel.2006.02.019
Head JR, 2013, DEV DYNAM, V242, P832, DOI 10.1002/dvdy.23973
Herman A, 2018, MOL ECOL, V27, P4397, DOI 10.1111/mec.14877
Hernández PP, 2006, HEARING RES, V213, P1, DOI 10.1016/j.heares.2005.10.015

HUDSPETH AJ, 1982, J NEUROSCI, V2, P1
Hyacinthe C, 2019, NAT COMMUN, V10, DOI 10.1038/s41467-019-12078-9
Iwasaki M, 2020, DEV DYNAM, V249, P1440, DOI 10.1002/dvdy.225
Iwashita M, 2021, ELIFE, V10, DOI [10.7554/eLife.72463, 10.7554/eLife.72463.sa1, 10.7554/eLife.72463.sa2]
Jaggard J, 2017, J EXP BIOL, V220, P284, DOI 10.1242/jeb.145128
Jaggard JB, 2020, SCI ADV, V6, DOI 10.1126/sciadv.aba3126
Jaggard JB, 2019, JOVE-J VIS EXP, DOI 10.3791/59198
Jaggard JB, 2018, ELIFE, V7, DOI 10.7554/eLife.32637
Janssen J, 2000, J COMP PHYSIOL A, V186, P957, DOI 10.1007/s003590000148
Jeffery WR, 1998, AM ZOOL, V38, P685
Jiang LJ, 2014, P NATL ACAD SCI USA, V111, pE1383, DOI 10.1073/pnas.1402898111
Johnson GD, 2009, BIOL LETTERS, V5, P235, DOI 10.1098/rsbl.2008.0722
KIMMEL CB, 1995, DEV DYNAM, V203, P253, DOI 10.1002/aja.1002030302
Klaassen H, 2018, DEV BIOL, V441, P313, DOI 10.1016/j.ydbio.2018.03.014
Klein A, 2015, INTEGR ZOOL, V10, P111, DOI 10.1111/1749-4877.12101
Kowalko JE, 2013, CURR BIOL, V23, P1874, DOI 10.1016/j.cub.2013.07.056
Kozol RA, 2023, ELIFE, V12, DOI 10.7554/eLife.80777
Krishnan J, 2017, PHILOS T R SOC B, V372, DOI 10.1098/rstb.2015.0487
Larson DJ, 2014, J EXP BIOL, V217, P2193, DOI 10.1242/jeb.101931
LEKANDER BERTIL, 1949, ACTA ZOOL [STOCKHOLM], V30, P1
Lenzi D, 2001, BIOESSAYS, V23, P831, DOI 10.1002/bies.1118
Lloyd E, 2018, DEV BIOL, V441, P328, DOI 10.1016/j.ydbio.2018.04.027
Loomis C, 2019, FRONT NEUROANAT, V13, DOI 10.3389/fnana.2019.00088
Lukasz D, 2022, ELIFE, V11, DOI 10.7554/eLife.77775
Lunsford ET, 2022, ELIFE, V11, DOI 10.7554/eLife.77387
Lunsford ET, 2019, J NEUROPHYSIOL, V122, P2438, DOI 10.1152/jn.00594.2019
Lush ME, 2014, DEV DYNAM, V243, P1187, DOI [10.1002/DVDY.24167, 10.1002/dvdy.24167]
Marshall NJ, 1996, J FISH BIOL, V49, P239, DOI 10.1111/j.1095-8649.1996.tb06079.x
Matthews G, 2010, NAT REV NEUROSCI, V11, P812, DOI 10.1038/nrn2924
McGaugh SE, 2020, J EXP ZOOL PART B, V334, P497, DOI 10.1002/jez.b.22949
McGaugh SE, 2014, NAT COMMUN, V5, DOI 10.1038/ncomms6307
McKee A, 2020, P ROY SOC B-BIOL SCI, V287, DOI 10.1098/rspb.2020.0568
Mekdara PJ, 2018, J EXP BIOL, V221, DOI 10.1242/jeb.175166
Menuet A, 2007, DEVELOPMENT, V134, P845, DOI 10.1242/dev.02780
Middlemiss KL, 2017, J COMP PHYSIOL A, V203, P807, DOI 10.1007/s00359-017-1192-6
Mitchell R., 1977, MEXICAN EYELESS CHAR
MONTGOMERY JC, 1987, SCIENCE, V235, P195, DOI 10.1126/science.235.4785.195
Montgomery JC, 1997, NATURE, V389, P960, DOI 10.1038/40135
Moser T, 2006, CELL TISSUE RES, V326, P347, DOI 10.1007/s00441-006-0276-3
Nelson G., 1972, Cephalic sensory canals, pitlines, and the classification of esocoid fishes, with notes on galaxiids and other teleosts
Nogare DED, 2020, ELIFE, V9, DOI 10.7554/eLife.58251

O'Quin KE, 2013, PLOS ONE, V8, DOI 10.1371/journal.pone.0057281
Ornelas-Garcia Claudia Patricia, 2008, BMC Evol Biol, V8, P340, DOI 10.1186/1471-2148-8-340
Owens KN, 2009, HEARING RES, V253, P32, DOI 10.1016/j.heares.2009.03.001
Parzefall J., 1983, Memoires de Biospeologie, V10, P171
Parzefall Jakob, 2002, Memoires de Biospeologie, V28, P157
Patch A, 2022, PLOS ONE, V17, DOI 10.1371/journal.pone.0265894
Patton P, 2010, J COMP PHYSIOL A, V196, P853, DOI 10.1007/s00359-010-0567-8
Pérez-Rodríguez R, 2021, PEERJ, V9, DOI 10.7717/peerj.10784
Pichler P, 2020, CURR BIOL, V30, P150, DOI 10.1016/j.cub.2019.11.020
Pichon F, 2004, EVOL DEV, V6, P187, DOI 10.1111/j.1525-142X.2004.04024.x
Piotrowski T, 2014, DEV BIOL, V389, P68, DOI 10.1016/j.ydbio.2014.02.016
Plath M, 2004, BEHAV ECOL SOCIOBIOL, V55, P596, DOI 10.1007/s00265-003-0750-9
POPPER A N, 1970, Animal Behaviour, V18, P552, DOI 10.1016/0003-3472(70)90052-7
Poulson TL, 2001, ENVIRON BIOL FISH, V62, P345, DOI 10.1023/A:1011893916855
Powers AK, 2020, J EXP ZOOL PART B, V334, P450, DOI 10.1002/jez.b.22932
Powers AK, 2018, DEV BIOL, V441, P252, DOI 10.1016/j.ydbio.2018.04.001
Ptacek MB, 1997, EVOLUTION, V51, P1217, DOI 10.1111/j.1558-5646.1997.tb03969.x
Pumphrey H. C., 1988, European Journal of Physics, V9, P225, DOI 10.1088/0143-0807/9/3/011
PUZDROWSKI RL, 1989, BRAIN BEHAV EVOLUT, V34, P110, DOI 10.1159/000116496
Riddle MR, 2021, BMC ECOL EVOL, V21, DOI 10.1186/s12862-021-01823-8
Riddle MR, 2018, NATURE, V555, P647, DOI 10.1038/nature26136
Rodriguez-Morales R, 2022, BMC ECOL EVOL, V22, DOI 10.1186/s12862-022-02069-8
Rosenthal GG, 1998, P NATL ACAD SCI USA, V95, P4431, DOI 10.1073/pnas.95.8.4431
RYAN MJ, 1992, AM NAT, V139, pS4, DOI 10.1086/285303
RYAN MJ, 1987, SCIENCE, V236, P595, DOI 10.1126/science.236.4801.595
Sarrazin AF, 2010, J NEUROSCI, V30, P8234, DOI 10.1523/JNEUROSCI.5137-09.2010
SCHLUPP I, 1994, SCIENCE, V263, P373, DOI 10.1126/science.8278809
Schmid C, 2020, FRONT CELL NEUROSCI, V14, DOI 10.3389/fncel.2020.00037
Schmitz A, 2008, J MORPHOL, V269, P751, DOI 10.1002/jmor.10621
Sharma S, 2009, J COMP PHYSIOL A, V195, P225, DOI 10.1007/s00359-008-0400-9
Soares D, 2020, J CAVE KARST STUD, V82, P198, DOI 10.4311/2019LSC0115
SONG JK, 1991, BRAIN BEHAV EVOLUT, V37, P10, DOI 10.1159/000114343
Stahl BA, 2019, DEV DYNAM, V248, P679, DOI 10.1002/dvdy.32
Strecker U, 2012, MOL PHYLOGENET EVOL, V62, P62, DOI 10.1016/j.ympev.2011.09.005
TAPPE DONALD T., 1941, JOUR MAMMAL, V22, P117, DOI 10.2307/1374908
TEYKE T, 1990, BRAIN BEHAV EVOLUT, V35, P23, DOI 10.1159/000115853
Thomas ED, 2019, ELIFE, V8, DOI 10.7554/eLife.43736

Thomas ED, 2015, WIRES DEV BIOL, V4, P1, DOI 10.1002/wdev.160
Tidswell B., 2023, bioRxiv, DOI [10.1101/2023.07.25.550510, DOI 10.1101/2023.07.25.550510]
Valdimarsson SK, 2001, ANIM BEHAV, V61, P1143, DOI 10.1006/anbe.2001.1710
Valera G, 2021, CURR BIOL, V31, P1463, DOI 10.1016/j.cub.2021.01.045
Van Trump WJ, 2013, INTEGR COMP BIOL, V53, P799, DOI 10.1093/icb/ict064
Van Trump WJ, 2010, HEARING RES, V261, P42, DOI 10.1016/j.heares.2010.01.001
Warren WC, 2021, NAT COMMUN, V12, DOI 10.1038/s41467-021-21733-z
Webb JF, 2003, DEV DYNAM, V228, P370, DOI 10.1002/dvdy.10385
WEBB JF, 1989, BRAIN BEHAV EVOLUT, V33, P34, DOI 10.1159/000115896
Witte K, 1998, BEHAV ECOL, V9, P534, DOI 10.1093/beheco/9.5.534
Xiong SL, 2018, DEV BIOL, V441, P297, DOI 10.1016/j.ydbio.2018.06.003
Yamamoto Y, 2004, NATURE, V431, P844, DOI 10.1038/nature02864
Yamamoto Y, 2000, SCIENCE, V289, P631, DOI 10.1126/science.289.5479.631
Yamamoto Y, 2009, DEV BIOL, V330, P200, DOI 10.1016/j.ydbio.2009.03.003
Yoshizawa M, 2014, J EXP BIOL, V217, P886, DOI 10.1242/jeb.094599
Yoshizawa M, 2012, BMC BIOL, V10, DOI 10.1186/1741-7007-10-108
Yoshizawa Masato, 2011, Communicative & Integrative Biology, V4, P89, DOI 10.4161/cib.4.1.14118
Yoshizawa M, 2010, CURR BIOL, V20, P1631, DOI 10.1016/j.cub.2010.07.017
NR 144
TC 1
Z9 1
U1 2
U2 6
PU WILEY
PI HOBOKEN
PA 111 RIVER ST, HOBOKEN 07030-5774, NJ USA
SN 2045-7758
J9 ECOL EVOL
JI Ecol. Evol.
PD APR
PY 2024
VL 14
IS 4
AR e11286
DI 10.1002/ece3.11286
PG 15
WC Ecology; Evolutionary Biology
WE Science Citation Index Expanded (SCI-EXPANDED)
SC Environmental Sciences & Ecology; Evolutionary Biology
GA OG7P8
UT WOS:001206187500001
PM 38654714
OA Green Published, gold
DA 2025-05-07
ER

PT J
AU Jaschke, N
   Wang, A
   Hofbauer, LC
   Rauner, M
   Rachner, TD
AF Jaschke, Nikolai
   Wang, Andrew
   Hofbauer, Lorenz C.
   Rauner, Martina
   Rachner, Tilman D.
TI Late-onset hypogonadism: Clinical evidence, biological aspects and evolutionary considerations
SO AGEING RESEARCH REVIEWS
LA English
DT Review
DE Testosterone; Late-onset hypogonadism; TRT; Evolution; Hormones; Endocrinology
AB The growing life expectancy in modern societies has raised scientific interest in identifying medical interventions to alleviate age-associated pathologies such as vascular calcification, cognitive decline, sarcopenia, osteoporosis and sexual dysfunction. Although no such single treatment has thus far been established in humans, some clinicians and patients have set their hopes on testosterone replacement therapy (TRT) as a potential ?fountain of youth? for aging men. While TRT has proven effective in ameliorating distinct symptoms of late-onset hypogonadism (LOH), its safety remains to be demonstrated. Besides humans, multiple other species exhibit agerelated reductions in circulating testosterone levels, raising the question whether such changes are an inherent, pathological feature of growing organismal age or rather reflect an adaptive response. In this manuscript, we apply key principles of evolutionary medicine to testosterone biology and LOH to provide a novel perspective on these two fields. Additionally, we discuss insightful data derived from the animal kingdom to illustrate the plasticity of individual testosterone trajectories across the lifespan, outline cost-benefitconsiderations of TRT in LOH and highlight potential caveats of such therapies.
C1 [Jaschke, Nikolai; Hofbauer, Lorenz C.; Rauner, Martina; Rachner, Tilman D.] Tech Univ Dresden, Dept Med 3, Dresden, Germany.
   [Jaschke, Nikolai; Hofbauer, Lorenz C.; Rauner, Martina; Rachner, Tilman D.] Tech Univ Dresden, Ctr Hlth Aging, Fetscherstr 74, D-01037 Dresden, Germany.
   [Wang, Andrew] Yale Univ, Sch Med, Dept Med Rheumatol Allergy & Immunol, New Haven, CT USA.
   [Wang, Andrew] Yale Univ, Sch Med, Dept Immunobiol, New Haven, CT USA.
C3 Technische Universitat Dresden; Technische Universitat Dresden; Yale University; Yale University
RP Jaschke, N (autor correspondente), Tech Univ Dresden, Ctr Hlth Aging, Fetscherstr 74, D-01037 Dresden, Germany.; Jaschke, N (autor correspondente), Tech Univ Dresden, Dept Med 3, Div Endocrinol & Metab Bone Dis, Fetscherstr 74, D-01037 Dresden, Germany.
EM nikolaipirmin.jaschke@uniklinikum-dresden.de
RI Rauner, Martina/A-1665-2015; Hofbauer, Lorenz C./G-2490-2010
OI Hofbauer, Lorenz C./0000-0002-8691-8423; Rauner,

Martina/0000-0002-4067-6799
FU Deutsche Forschungsgemeinschaft (DFG) [HO 1875/24-1, HO 1875/261, RA 2151/4-1, RA1923/121]; German Academic Scholarship Foundation; Mildred Scheel Nachwuchszentrum (MSNZ); NIH [K08AI128745]; National Institute of
Allergy and Infectious Diseases [K08AI128745] Funding Source: NIH RePORTER
FX The authors would like to thank Tiziana Klawitter for her excellent help
with the figures. This work was funded by theDeutsche Forschungsgemeinschaft (DFG) to LCH (HO 1875/24-1 and HO 1875/261), TDR
(RA 2151/4-1) and MR (RA1923/121). Further funding was provided by the German Academic Scholarship Foundation and Mildred Scheel Nachwuchszentrum (MSNZ) to NJ. AW was supported by the NIH Clinical Investigator Award (K08AI128745) .
CR Adelborg K, 2019, AM J CARDIOL, V123, P1757, DOI 10.1016/j.amjcard.2019.02.042
Aguiar-Oliveira MH, 2019, ENDOCR REV, V40, P575, DOI 10.1210/er.2018-00216
Almanza A, 2019, FEBS J, V286, P241, DOI 10.1111/febs.14608
Andersson CR, 2017, SCAND J CLIN LAB INV, V77, P582, DOI 10.1080/00365513.2017.1339231
Araujo AB, 2007, ARCH INTERN MED, V167, P1252, DOI 10.1001/archinte.167.12.1252
Arnold FJ, 2019, NEUROTHERAPEUTICS, V16, P928, DOI 10.1007/s13311-019-00790-9
Atsuta N, 2006, BRAIN, V129, P1446, DOI 10.1093/brain/awl096
Austad SN, 2016, CELL METAB, V23, P1022, DOI 10.1016/j.cmet.2016.05.019
Badders NM, 2018, NAT MED, V24, P427, DOI 10.1038/nm.4500
Balchin D, 2016, SCIENCE, V353, DOI 10.1126/science.aac4354
Banno H, 2009, ANN NEUROL, V65, P140, DOI 10.1002/ana.21540
Basualto-Alarcón C, 2013, MED SCI SPORT EXER, V45, P1712, DOI 10.1249/MSS.0b013e31828cf5f3
Beehner JC, 2009, HORM BEHAV, V56, P472, DOI 10.1016/j.yhbeh.2009.08.005
Beitel LK, 2013, FRONT NEUROL, V4, DOI 10.3389/fneur.2013.00053
Bhasin S, 2018, J CLIN ENDOCR METAB, V103, P1715, DOI 10.1210/jc.2018-00229
Brooks RC, 2017, ANN NY ACAD SCI, V1389, P92, DOI 10.1111/nyas.13302
Brouillette J, 2005, CARDIOVASC RES, V65, P148, DOI 10.1016/j.cardiores.2004.09.012
Cakir I, 2019, TRENDS ENDOCRIN MET, V30, P163, DOI 10.1016/j.tem.2019.01.002
Calof OM, 2005, J GERONTOL A-BIOL, V60, P1451, DOI 10.1093/gerona/60.11.1451
Cangemi R, 2010, AGING CELL, V9, P236, DOI 10.1111/j.1474-9726.2010.00553.x
Cates KA, 2019, GEN COMP ENDOCR, V279, P164, DOI 10.1016/j.ygcen.2019.03.013
Chester RC, 2018, CLIN CARDIOL, V41, P247, DOI 10.1002/clc.22891
CHRISTEFF N, 1988, J STEROID BIOCHEM, V29, P435, DOI 10.1016/0022-4731(88)90254-3

Corona G, 2011, EUR J ENDOCRINOL, V165, P687, DOI 10.1530/EJE-11-0447
de Groot PCM, 2011, HUM REPROD UPDATE, V17, P495, DOI 10.1093/humupd/dmr001
DLUZEN D, 1994, J NEUROCHEM, V62, P94
DRORI D, 1976, EXP GERONTOL, V11, P25, DOI 10.1016/0531-5565(76)90007-3
Ebrahimi F, 2018, J CLIN ENDOCR METAB, V103, P3466, DOI 10.1210/jc.2018-00739
Ellison PT, 2002, HUM REPROD, V17, P3251, DOI 10.1093/humrep/17.12.3251
Ellison PT, 1996, HUM BIOL, V68, P955
Eskelinen EL, 2019, INT J BIOCHEM CELL B, V111, P1, DOI 10.1016/j.biocel.2019.03.010
FANJUL LF, 1981, HORM METAB RES, V13, P356, DOI 10.1055/s-2007-1019265
Feldman HA, 2002, J CLIN ENDOCR METAB, V87, P589, DOI 10.1210/jc.87.2.589
Fiers T, 2014, STEROIDS, V86, P5, DOI 10.1016/j.steroids.2014.04.013
Finegold JA, 2013, INT J CARDIOL, V168, P934, DOI 10.1016/j.ijcard.2012.10.046
Finkelstein JS, 2013, NEW ENGL J MED, V369, P2457, DOI 10.1056/NEJMc1313169
Finsterer J, 2010, J NEUROL SCI, V298, P1, DOI 10.1016/j.jns.2010.08.025
Furman D, 2014, P NATL ACAD SCI USA, V111, P869, DOI 10.1073/pnas.1321060111
Gagliano-Jucá T, 2019, NAT REV CARDIOL, V16, P555, DOI 10.1038/s41569-019-0211-4
Gesquiere LR, 2011, SCIENCE, V333, P357, DOI 10.1126/science.1207120
Gillies GE, 2014, FRONT NEUROENDOCRIN, V35, P370, DOI 10.1016/j.yfrne.2014.02.002
GRIGGS RC, 1989, J APPL PHYSIOL, V66, P498, DOI 10.1152/jappl.1989.66.1.498
Grosen A, 2019, J CROHNS COLITIS, V13, P564, DOI 10.1093/ecco-jcc/jjy198
Hackney A.C., 2018, J BIOCH PHYSL, V1
Hammes SR, 2019, J CLIN INVEST, V129, P1818, DOI 10.1172/JCI125755
Haring R, 2013, ANDROLOGY-US, V1, P17, DOI 10.1111/j.2047-2927.2012.00002.x
Haring R, 2013, CLIN ENDOCRINOL, V78, P629, DOI 10.1111/cen.12013
Harrison DE, 2009, NATURE, V460, P392, DOI 10.1038/nature08221
Hashizume A, 2017, J NEUROL NEUROSUR PS, V88, P1026, DOI 10.1136/jnnp-2017-316015
Hipp MS, 2019, NAT REV MOL CELL BIO, V20, P421, DOI 10.1038/s41580-019-0101-y
Hunt KE, 2018, CONSERV PHYSIOL, V6, DOI 10.1093/conphys/coy049
Johnson SL, 2015, AGEING RES REV, V19, P22, DOI 10.1016/j.arr.2014.10.007
Kaminetsky J, 2015, SEX MED-UK, V3, P269, DOI 10.1002/sm2.80
Kaminetsky JC, 2019, J UROLOGY, V201, P587, DOI 10.1016/j.juro.2018.09.057
Kanik KS, 2000, J CLIN ENDOCR METAB, V85, P1461, DOI 10.1210/jc.85.4.1461
Katsuno M, 2002, NEURON, V35, P843, DOI 10.1016/S0896-6273(02)00834-6

Katsuno M, 2010, LANCET NEUROL, V9, P875, DOI 10.1016/S1474-4422(10)70182-4
Kelly DM, 2013, J ENDOCRINOL, V217, pR25, DOI 10.1530/JOE-12-0455
Khera M, 2016, MAYO CLIN PROC, V91, P908, DOI 10.1016/j.mayocp.2016.04.022
KIRKWOOD TBL, 1982, HUM GENET, V60, P101, DOI 10.1007/BF00569695
Lopes RF, 2009, OSTEOPOROSIS INT, V20, P1175, DOI 10.1007/s00198-008-0796-7
Lotti F, 2013, ANDROLOGY-US, V1, P229, DOI 10.1111/j.2047-2927.2012.00031.x
Lotti F, 2014, ASIAN J ANDROL, V16, P295, DOI 10.4103/1008-682X.122341
Luo S, 2019, BMJ-BRIT MED J, V364, DOI 10.1136/bmj.l476
MACHIDA T, 1981, HORM BEHAV, V15, P238, DOI 10.1016/0018-506X(81)90013-1
Mangiamele LA, 2016, P NATL ACAD SCI USA, V113, P5664, DOI 10.1073/pnas.1603329113
Marjoribanks J, 2017, COCHRANE DB SYST REV, DOI 10.1002/14651858.CD004143.pub5
Mathers CD, 2009, BRIT MED BULL, V92, P7, DOI 10.1093/bmb/ldp028
Min KJ, 2012, CURR BIOL, V22, pR792, DOI 10.1016/j.cub.2012.06.036
Morelli A, 2014, MOL CELL ENDOCRINOL, V382, P107, DOI 10.1016/j.mce.2013.09.017
Morgentaler A, 2009, EUR UROL, V55, P310, DOI 10.1016/j.eururo.2008.09.024
Muehlenbein MP, 2005, AM J HUM BIOL, V17, P527, DOI 10.1002/ajhb.20419
Nedelsky NB, 2010, NEURON, V67, P936, DOI 10.1016/j.neuron.2010.08.034
Ni W, 2015, PLOS ONE, V10, DOI 10.1371/journal.pone.0122279
Nieschlag E, 2020, ANDROLOGY-US, V8, P1506, DOI 10.1111/andr.12719
Nieschlag E, 2004, HUM REPROD UPDATE, V10, P409, DOI 10.1093/humupd/dmh035
Nieschlag E, 2019, EUR J ENDOCRINOL, V180, pR201, DOI 10.1530/EJE-19-0071
Nieschlag E, 2015, REV ENDOCR METAB DIS, V16, P199, DOI 10.1007/s11154-015-9320-5
Nieschlag E, 2015, BEST PRACT RES CL EN, V29, P77, DOI 10.1016/j.beem.2014.09.008
O'Brien KA, 2018, J EXP BIOL, V221, DOI 10.1242/jeb.177683
Okin D, 2012, CURR BIOL, V22, pR733, DOI 10.1016/j.cub.2012.07.029
Okun MS, 2006, ARCH NEUROL-CHICAGO, V63, P729, DOI 10.1001/archneur.63.5.729
Osibogun O, 2020, TRENDS CARDIOVAS MED, V30, P399, DOI 10.1016/j.tcm.2019.08.010
Parua S, 1998, ANDROLOGIA, V30, P105
Perry JRB, 2010, HUM MOL GENET, V19, P535, DOI 10.1093/hmg/ddp522
Plymate S R, 1988, Steroids, V52, P339, DOI 10.1016/0039-128X(88)90136-5
PLYMATE SR, 1988, J CLIN ENDOCR METAB, V67, P460, DOI 10.1210/jcem-67-3-460
Pye SR, 2014, J CLIN ENDOCR METAB, V99, P1357, DOI 10.1210/jc.2013-2052
Ramachandran S, 2018, ANDROLOGY-US, V6, P846, DOI 10.1111/andr.12520
RIGAUDIERE N, 1976, J REPROD FERTIL, V48, P291, DOI 10.1530/jrf.0.0480291

Rosenbohm A, 2018, J NEUROL, V265, P1026, DOI 10.1007/s00415-018-8790-2
Saad F, 2020, AGING MALE, V23, P81, DOI 10.1080/13685538.2019.1575354
Sharma R, 2016, CHEST, V150, P563, DOI 10.1016/j.chest.2016.05.007
Sharma R, 2015, EUR HEART J, V36, P2706, DOI 10.1093/eurheartj/ehv346
Shores MM, 2014, J CLIN ENDOCR METAB, V99, P2061, DOI 10.1210/jc.2013-3576
Snyder PJ, 2016, NEW ENGL J MED, V374, P611, DOI 10.1056/NEJMoa1506119
Snyder PJ, 2018, ENDOCR REV, V39, P369, DOI 10.1210/er.2017-00234
Srinivas-Shankar U, 2006, NAT CLIN PRACT UROL, V3, P653, DOI 10.1038/ncpuro0650
Stearns S., 2008, FDN EVOLUTIONARY PSY, P47
Stearns S., 2016, EVOLUTIONARY MED, V306
Stearns S.C., 1992, pi
Stearns SC, 2012, P ROY SOC B-BIOL SCI, V279, P4305, DOI 10.1098/rspb.2012.1326
Storer TW, 2017, J CLIN ENDOCR METAB, V102, P583, DOI 10.1210/jc.2016-2771
Tang YJ, 2007, J ENDOCRINOL INVEST, V30, P451, DOI 10.1007/BF03346327
Thompson IM, 2003, NEW ENGL J MED, V349, P215, DOI 10.1056/NEJMoa030660
Tyshkovskiy A, 2019, CELL METAB, V30, P573, DOI 10.1016/j.cmet.2019.06.018
Vigen R, 2013, JAMA-J AM MED ASSOC, V310, P1829, DOI 10.1001/jama.2013.280386
Wallace IR, 2013, CLIN ENDOCRINOL, V78, P321, DOI 10.1111/cen.12086
Wang A, 2019, SCIENCE, V363, P140, DOI 10.1126/science.aar3932
Watts EL, 2018, EUR UROL, V74, P585, DOI 10.1016/j.eururo.2018.07.024
Weir HJ, 2017, CELL METAB, V26, P884, DOI 10.1016/j.cmet.2017.09.024
West PM, 2002, SCIENCE, V297, P1339, DOI 10.1126/science.1073257
Wu FCW, 2010, NEW ENGL J MED, V363, P123, DOI 10.1056/NEJMoa0911101
Xu L, 2013, BMC MED, V11, DOI 10.1186/1741-7015-11-108
Yeap BB, 2009, J CLIN ENDOCR METAB, V94, P2353, DOI 10.1210/jc.2008-2416
YU YL, 1994, J NEURAL TRANSM-PARK, V8, P215, DOI 10.1007/BF02260942
Yuan R, 2009, AGING CELL, V8, P277, DOI 10.1111/j.1474-9726.2009.00478.x
Yusuf S, 2020, LANCET, V395, P795, DOI 10.1016/S0140-6736(19)32008-2
Zitzmann M, 2006, J CLIN ENDOCR METAB, V91, P4335, DOI 10.1210/jc.2006-0401
Zitzmann M, 2009, NAT REV ENDOCRINOL, V5, P673, DOI 10.1038/nrendo.2009.212
NR 120
TC 9
Z9 12
U1 1
U2 9
PU ELSEVIER IRELAND LTD
PI CLARE
PA ELSEVIER HOUSE, BROOKVALE PLAZA, EAST PARK SHANNON, CO, CLARE, 00000, IRELAND
SN 1568-1637
EI 1872-9649
J9 AGEING RES REV

JI Ageing Res. Rev.
PD MAY
PY 2021
VL 67
AR 101301
DI 10.1016/j.arr.2021.101301
EA FEB 2021
PG 8
WC Cell Biology; Geriatrics & Gerontology
WE Science Citation Index Expanded (SCI-EXPANDED)
SC Cell Biology; Geriatrics & Gerontology
GA RF5FN
UT WOS:000634864200001
PM 33610812
OA Green Accepted
DA 2025-05-07
ER

PT J
AU Abondio, P
   Cilli, E
   Luiselli, D
AF Abondio, Paolo
   Cilli, Elisabetta
   Luiselli, Donata
TI Inferring Signatures of Positive Selection in Whole-Genome Sequencing
   Data: An Overview of Haplotype-Based Methods
SO GENES
LA English
DT Review
DE positive selection; whole-genome sequencing; haplotype-based methods;
   haplotype; linkage; selective sweep; haplotype homozygosity; haplotype
   composition; haplotype frequency; statistics
ID LINKAGE DISEQUILIBRIUM; RECOMBINATION RATES; POPULATION-STRUCTURE;
   GENETIC HITCHHIKING; R PACKAGE; SWEEPS; SCANS; ADAPTATION; PROGRAM;
   HUMANS
AB Signatures of positive selection in the genome are a characteristic
mark of adaptation that can reveal an ongoing, recent, or ancient
response to environmental change throughout the evolution of a
population. New sources of food, climate conditions, and exposure to
pathogens are only some of the possible sources of selective pressure,
and the rise of advantageous genetic variants is a crucial determinant of
survival and reproduction. In this context, the ability to detect these
signatures of selection may pinpoint genetic variants that are
responsible for a significant change in gene regulation, gene expression,
or protein synthesis, structure, and function. This review focuses on
statistical methods that take advantage of linkage disequilibrium and
haplotype determination to reveal signatures of positive selection in
whole-genome sequencing data, showing that they emerge from different
descriptions of the same underlying event. Moreover, considerations are
provided around the application of these statistics to different species,
their suitability for ancient DNA, and the usefulness of discovering
variants under selection for biomedicine and public health in an
evolutionary medicine framework.

C1 [Abondio, Paolo; Cilli, Elisabetta; Luiselli, Donata] Univ Bologna, Dept Cultural Heritage, Via Ariani 1, I-48121 Ravenna, Italy.
[Abondio, Paolo] Univ Bologna, Lab Mol Anthropol, Via Selmi 3, I-40126 Bologna, Italy.
[Abondio, Paolo] Univ Bologna, Dept Biol Geol & Environm Sci, Ctr Genome Biol, Via Selmi 3, I-40126 Bologna, Italy.
[Luiselli, Donata] Fano Marine Ctr, Interinst Ctr Res Marine Biodivers Resources & Bi, Viale Adriat 1-N, I-61032 Fano, Italy.
C3 University of Bologna; University of Bologna; University of Bologna
RP Abondio, P (autor correspondente), Univ Bologna, Dept Cultural Heritage, Via Ariani 1, I-48121 Ravenna, Italy.; Abondio, P (autor correspondente), Univ Bologna, Lab Mol Anthropol, Via Selmi 3, I-40126 Bologna, Italy.; Abondio, P (autor correspondente), Univ Bologna, Dept Biol Geol & Environm Sci, Ctr Genome Biol, Via Selmi 3, I-40126 Bologna, Italy.
EM paolo.abondio2@unibo.it; elisabetta.cilli@unibo.it; donata.luiselli@unibo.it
RI ; CILLI, ELISABETTA/AAV-7363-2021; Abondio, Paolo/AFI-0344-2022
OI Luiselli, Donata/0000-0003-2105-2478; CILLI, ELISABETTA/0000-0003-0407-267X; Abondio, Paolo/0000-0002-9626-8495
FU Ministero dell'Universita e della Ricerca, PRIN 2017 [20177PJ9XF_004]
FX This research was funded by Ministero dell'Universita e della Ricerca, PRIN 2017 grant number 20177PJ9XF_004 to D.L.
CR Abecasis GR, 2005, HUM HERED, V59, P118, DOI 10.1159/000085226
[Anonymous], REHH SEARCHING FOOTP
[Anonymous], EFFICIENT PROGRAM CA
[Anonymous], LASSI PLUS PROGRAM C
Ausmees K, 2022, G3-GENES GENOM GENET, V12, DOI 10.1093/g3journal/jkac089
Bao Q, 2022, FRONT GENET, V13, DOI 10.3389/fgene.2022.798076
Barton NH, 2000, PHILOS T R SOC B, V355, P1553, DOI 10.1098/rstb.2000.0716
Benton ML, 2021, NAT REV GENET, V22, P269, DOI 10.1038/s41576-020-00305-9
Briggs AW, 2010, NUCLEIC ACIDS RES, V38, DOI 10.1093/nar/gkp1163
Dabney J, 2013, CSH PERSPECT BIOL, V5, DOI [10.1101/cshperspect.a012567, DOI 10.1101/cshperspect.a012567]
DeGiorgio M, 2022, PLOS GENET, V18, DOI 10.1371/journal.pgen.1010134
Fagny M, 2014, MOL BIOL EVOL, V31, P1850, DOI 10.1093/molbev/msu118
Feleke SM, 2021, NAT MICROBIOL, V6, P1289, DOI 10.1038/s41564-021-00962-4
Ferrer-Admetlla A, 2014, MOL BIOL EVOL, V31, P1275, DOI 10.1093/molbev/msu077
Garud NR, 2021, PLOS GENET, V17, DOI 10.1371/journal.pgen.1009373
Garud NR, 2015, PLOS GENET, V11, DOI 10.1371/journal.pgen.1005004
Gautier M, 2017, MOL ECOL RESOUR, V17, P78, DOI 10.1111/1755-0998.12634
Gautier M, 2012, BIOINFORMATICS, V28, P1176, DOI 10.1093/bioinformatics/bts115
Geibel J, 2022, BMC GENOMICS, V23, DOI 10.1186/s12864-022-08418-7
Gnecchi-Ruscone GA, 2018, GENOME BIOL EVOL, V10, P2919, DOI 10.1093/gbe/evy233
Gokcumen O, 2011, GENOME BIOL, V12, DOI 10.1186/gb-2011-12-5-r52
Günther T, 2019, PLOS GENET, V15, DOI 10.1371/journal.pgen.1008302

Guo Y, 2022, FRONT GENET, V13, DOI 10.3389/fgene.2022.812753
Haasl RJ, 2014, GENOME BIOL EVOL, V6, P1843, DOI 10.1093/gbe/evu134
Haasl RJ, 2013, MOL BIOL EVOL, V30, P285, DOI 10.1093/molbev/mss247
Hanchard NA, 2006, AM J HUM GENET, V78, P153, DOI 10.1086/499252
Harris AM, 2020, GENETICS, V215, P143, DOI 10.1534/genetics.120.303137
Harris AM, 2018, GENETICS, V210, P1429, DOI 10.1534/genetics.118.301502
Henderson IR, 2021, ANNU REV GENET, V55, P23, DOI 10.1146/annurev-genet-021721-033821
HENDERSON SA, 1970, ANNU REV GENET, V4, P295, DOI 10.1146/annurev.ge.04.120170.001455
Duarte INH, 2022, PLOS ONE, V17, DOI 10.1371/journal.pone.0264279
Höllinger I, 2019, PLOS GENET, V15, DOI 10.1371/journal.pgen.1008035
Irving-Pease EK, 2021, FRONT GENET, V12, DOI 10.3389/fgene.2021.703541
Jeffreys AJ, 2005, NAT GENET, V37, P601, DOI 10.1038/ng1565
Jensen-Seaman MI, 2004, GENOME RES, V14, P528, DOI 10.1101/gr.1970304
Jeong C, 2014, CURR OPIN GENET DEV, V29, P1, DOI 10.1016/j.gde.2014.06.011
Kassegne K, 2020, FRONT IMMUNOL, V11, DOI 10.3389/fimmu.2020.552698
Kauer MO, 2003, GENETICS, V165, P1137
Kim Y, 2000, GENETICS, V155, P1415
Kimura R, 2007, PLOS ONE, V2, DOI 10.1371/journal.pone.0000286
Klassmann A, 2022, PLOS ONE, V17, DOI 10.1371/journal.pone.0262024
Koch E, 2013, PLOS ONE, V8, DOI 10.1371/journal.pone.0080754
Kuhn A, 2014, P NATL ACAD SCI USA, V111, P8131, DOI 10.1073/pnas.1401532111
Kuhn JMM, 2018, PLOS ONE, V13, DOI 10.1371/journal.pone.0195491
Landini A, 2021, EVOL APPL, V14, P297, DOI 10.1111/eva.13090
Lange JD, 2016, MOL ECOL, V25, P3081, DOI 10.1111/mec.13671
Lerat E, 2019, MOL ECOL, V28, P1506, DOI 10.1111/mec.14963
Lin YL, 2019, GENOME BIOL EVOL, V11, P1136, DOI 10.1093/gbe/evz058
Liu DY, 2021, BMC GENOMICS, V22, DOI 10.1186/s12864-021-08042-x
Liu YH, 2021, MOL BIOL EVOL, V38, P4884, DOI 10.1093/molbev/msab214
Lucas ER, 2019, GENOME RES, V29, P1250, DOI 10.1101/gr.245795.118
Maclean CA, 2015, MOL BIOL EVOL, V32, P3027, DOI 10.1093/molbev/msv172
McPeek MS, 1999, AM J HUM GENET, V65, P858, DOI 10.1086/302537
Mueller JC, 2004, BRIEF BIOINFORM, V5, P355, DOI 10.1093/bib/5.4.355
Myers S, 2005, SCIENCE, V310, P321, DOI 10.1126/science.1117196
Myers TA, 2020, FRONT GENET, V11, DOI 10.3389/fgene.2020.00157
Nesse RM, 2010, P NATL ACAD SCI USA, V107, P1800, DOI 10.1073/pnas.0906224106
Nesse RM, 2008, LANCET, pS21
Nolte W, 2019, PLOS ONE, V14, DOI 10.1371/journal.pone.0215913
Novembre J, 2012, PHILOS T R SOC B, V367, P878, DOI 10.1098/rstb.2011.0305
Ojeda-Granados C, 2022, MOL BIOL EVOL, V39, DOI 10.1093/molbev/msab290
Park S, 2019, BMC PLANT BIOL, V19, DOI 10.1186/s12870-018-1621-x
Pfaffelhuber P, 2008, GENETICS, V179, P527, DOI 10.1534/genetics.107.081497
Pollard T.D., 2008, CELL BIOL, V2nd
Pritchard JK, 2001, AM J HUM GENET, V69, P1, DOI 10.1086/321275
Pritchard JK, 2010, CURR BIOL, V20, pR208, DOI 10.1016/j.cub.2009.11.055

Ranathunge C, 2021, bioRxiv, DOI [10.1101/2021.08.01.454655, 10.1101/2021.08.01.454655, DOI 10.1101/2021.08.01.454655]
Rees JS, 2020, TRENDS GENET, V36, P415, DOI 10.1016/j.tig.2020.03.006
Reich DE, 2001, NATURE, V411, P199, DOI 10.1038/35075590
Rishishwar L, 2018, GENE, V675, P69, DOI 10.1016/j.gene.2018.06.077
Sabatti C, 2002, GENETICS, V160, P1707
Sabeti PC, 2007, NATURE, V449, P913, DOI 10.1038/nature06250
Sabeti PC, 2002, NATURE, V419, P832, DOI 10.1038/nature01140
Saitou M, 2020, J MOL EVOL, V88, P104, DOI 10.1007/s00239-019-09911-6
Santos WB, 2021, BMC GENOMICS, V22, DOI 10.1186/s12864-021-08053-8
Sazzini M, 2020, BMC BIOL, V18, DOI 10.1186/s12915-020-00778-4
Schlamp F, 2016, MOL ECOL, V25, P342, DOI 10.1111/mec.13485
Selscan, PROGR CALC EHH BAS S
Seo D, 2022, SCI REP-UK, V12, DOI 10.1038/s41598-022-09425-0
Skoglund P, 2014, P NATL ACAD SCI USA, V111, P2229, DOI 10.1073/pnas.1318934111
Smith J.M., 1974, Res., V23, P35, DOI [10.1017/S0016672300014634, DOI 10.1017/S0016672300014634]
Stapley J, 2017, PHILOS T R SOC B, V372, DOI 10.1098/rstb.2016.0455
Stephan W, 2019, GENETICS, V211, P5, DOI 10.1534/genetics.118.301319
Stephan W, 2016, MOL ECOL, V25, P79, DOI 10.1111/mec.13288
Sudmant PH, 2015, SCIENCE, V349, DOI 10.1126/science.aab3761
Szpak M, 2019, FEBS LETT, V593, P1431, DOI 10.1002/1873-3468.13447
Szpiech ZA, 2021, EVOL LETT, V5, P408, DOI 10.1002/evl3.232
Szpiech ZA, 2014, MOL BIOL EVOL, V31, P2824, DOI 10.1093/molbev/msu211
Tang K, 2007, PLOS BIOL, V5, P1587, DOI 10.1371/journal.pbio.0050171
Voight BF, 2006, PLOS BIOL, V4, P446, DOI 10.1371/journal.pbio.0040072
Wall JD, 2003, NAT REV GENET, V4, P587, DOI 10.1038/nrg1123
Wang Y, 2009, P NATL ACAD SCI USA, V106, P6215, DOI 10.1073/pnas.0900418106
Wells JCK, 2017, LANCET, V390, P500, DOI 10.1016/S0140-6736(17)30572-X
Werren EA, 2021, HUM GENET, V140, P241, DOI 10.1007/s00439-020-02206-7
Yeaman S, 2022, GENETICS, V220, DOI 10.1093/genetics/iyab134
Zhang C, 2006, BIOINFORMATICS, V22, P2122, DOI 10.1093/bioinformatics/btl365
Zhao JT, 2022, HORTIC RES-ENGLAND, V9, DOI 10.1093/hr/uhab009
Zorc M, 2022, GENET SEL EVOL, V54, DOI 10.1186/s12711-022-00718-6
NR 98
TC 14
Z9 15
U1 4
U2 32
PU MDPI
PI BASEL
PA ST ALBAN-ANLAGE 66, CH-4052 BASEL, SWITZERLAND
EI 2073-4425
J9 GENES-BASEL
JI Genes
PD MAY
PY 2022
VL 13
IS 5
AR 926
DI 10.3390/genes13050926

PG 16
WC Genetics & Heredity
WE Science Citation Index Expanded (SCI-EXPANDED)
SC Genetics & Heredity
GA 1R2KN
UT WOS:000803204300001
PM 35627311
OA Green Published, gold
DA 2025-05-07
ER

PT J
AU Power, ML
Snead, C
Reed, EG
Schulkin, J
AF Power, Michael L.
Snead, Carrie
Reed, Eda G.
Schulkin, Jay
TI Integrating evolution into medical education for women's health care practitioners
SO EVOLUTION MEDICINE AND PUBLIC HEALTH
LA English
DT Article
DE lactation; microbiome; medical training; obstetrician-gynecologists
ID VAGINAL MICROBIOME; BACTERIAL VAGINOSIS; AIR-POLLUTION; INFECTIOUS MASTITIS; LACTATION; BIOLOGY; BIRTH; RISK; MILK
AB Evolution is a fundamental principle in biology; however, it has been neglected in medical education. We argue that an evolutionary perspective is especially important for women's health care providers, as selection will act strongly on reproductive parameters, and the biological costs of female reproduction are generally more resource expensive than for men (e.g. due to gestation and lactation) with greater effects on health and wellbeing. An evolutionary perspective is needed to understand antibiotic resistance, disease and health risks associated with mismatches between our evolved adaptations and current conditions, the importance of the microbiome and the maternal role in how infants acquire and develop their early-life microbiome (vaginal birth, lactation), and the importance of breastmilk as a biochemical signal from mothers to their babies. We present data that obstetrician-gynecologists' views regarding the inclusion of evolution within their training is generally positive, but many barriers are perceived. Requiring coursework in evolutionary biology with an emphasis on evolutionary medicine prior to enrollment in medical school may be a solution.
C1 [Power, Michael L.; Reed, Eda G.] Smithsonian Natl Zool Pk, POB 37012,MRC 5503, Washington, DC 20013 USA.
[Power, Michael L.; Reed, Eda G.] Conservat Biol Inst, POB 37012,MRC 5503, Washington, DC 20013 USA.
[Power, Michael L.; Snead, Carrie] Amer Coll Obstetricians & Gynecologists, 409 12th St SW, Washington, DC 20024 USA.
[Reed, Eda G.] Johns Hopkins Univ, Baltimore, MD 21205 USA.
[Schulkin, Jay] Univ Washington, Sch Med, Obstet & Gynecol, Seattle, WA 98195 USA.

C3 Smithsonian Institution; Smithsonian National Zoological Park &
Conservation Biology Institute; Johns Hopkins University; University
of
Washington; University of Washington Seattle
RP Power, ML (autor correspondente), Smithsonian Natl Zool Pk, POB
37012,MRC 5503, Washington, DC 20013 USA.; Power, ML (autor
correspondente), Conservat Biol Inst, POB 37012,MRC 5503, Washington, DC
20013 USA.
EM powerm@si.edu
FU Health Resources and Services Administration (HRSA) of the US
Department
of Health and Human Services (HHS) [UA6MC31609]
FX This project is supported by the Health Resources and Services
Administration (HRSA) of the US Department of Health and Human
Services
(HHS) under cooperative agreement UA6MC31609: PregnancyRelated Care
Research Network. This information or content and conclusions are
those
of the author and should not be construed as the official position or
policy of, nor should any endorsements be inferred by HRSA, HHS, or
the
U.S. Government.
CR Abbott A, 2006, MED EDUC, V40, P471, DOI 10.1111/j.1365-
2929.2006.02428.x
American Public Health Association, 2017, Advancing a "One Health"
approach to promote health at the human - animal -environment interface
Amir LH, 2016, INT BREASTFEED J, V11, DOI 10.1186/s13006-016-0078-5
AMSEL R, 1983, AM J MED, V74, P14, DOI 10.1016/0002-9343(83)91112-9
[Anonymous], 2017, Obstet Gynecol, V130, pe274, DOI
10.1097/AOG.0000000000002402
Arroyo R, 2010, CLIN INFECT DIS, V50, P1551, DOI 10.1086/652763
Arthur A., 2018, Journal of Evolutionary Medicine, V6, P1
Barnes ME, 2016, CBE-LIFE SCI EDUC, V15, DOI 10.1187/cbe.15-11-0243
Barnes ME, 2017, CBE LIFE SCI ED, V1
Bibbins-Domingo K, 2017, JAMA-J AM MED ASSOC, V317, P1661, DOI
10.1001/jama.2017.3439
Bouchard DS, 2015, PLOS ONE, V10, DOI 10.1371/journal.pone.0144831
Brotman RM, 2011, J CLIN INVEST, V121, P4610, DOI 10.1172/JCI57172
Cabrera-Rubio R, 2012, AM J CLIN NUTR, V96, P544, DOI
10.3945/ajcn.112.037382
Espeche MC, 2012, ANAEROBE, V18, P103, DOI
10.1016/j.anaerobe.2012.01.002
Centers for Disease Control and Prevention (CDC), 2019, ANTIBIOTIC
RESISTANC
COE CL, 1994, AM J PRIMATOL, V32, P51, DOI 10.1002/ajp.1350320106
Committee on Obstetrics Practice American College of Obstetricians and
Gynecology, 2018, OBSTET GYNECOL, V132, pe259
Crosley EJ, 2013, PLACENTA, V34, P127, DOI
10.1016/j.placenta.2012.12.001
DOBZHANSKY T, 1973, AM BIOL TEACH, V35, P125, DOI 10.2307/4444260
Dominguez-Bello MG, 2016, NAT MED, V22, P250, DOI 10.1038/nm.4039
Elliot MG, 2015, PHILOS T R SOC B, V370, DOI 10.1098/rstb.2014.0069
Fernández L, 2016, CLIN INFECT DIS, V62, P568, DOI 10.1093/cid/civ974
Garcia M, 2017, AM J PRIMATOL, V79, DOI 10.1002/ajp.22614

Gluckman P, 2016, PRINCIPLES OF EVOLUTIONARY MEDICINE, 2ND EDITION, P1, DOI 10.1093/acprof:oso/9780199663927.001.0001
Gluckman PD, 2020, INTEGRATING EVOLUTIONARY BIOLOGY INTO MEDICAL EDUCATION: FOR MATERNAL AND CHILD HEALTHCARE STUDENTS, CLINICIANS, AND SCIENTISTS, P13, DOI 10.1093/oso/9780198814153.003.0002
Gluckman PeterD., 2009, Principles of evolutionary medicine
Graves Joseph L Jr, 2016, Evol Med Public Health, V2016, P358
GREENE WA, 1991, J DAIRY SCI, V74, P2976, DOI 10.3168/jds.S0022-0302(91)78483-X
HAIG D, 1993, Q REV BIOL, V68, P495, DOI 10.1086/418300
Harris Eugene E, 2005, BMC Med Educ, V5, P16, DOI 10.1186/1472-6920-5-16
Hidaka BH, 2015, BMC MED EDUC, V15, DOI 10.1186/s12909-015-0322-5
KENNELL J, 1991, JAMA-J AM MED ASSOC, V265, P2197, DOI 10.1001/jama.265.17.2197
Kenyon C, 2013, AM J OBSTET GYNECOL, V209, P505, DOI 10.1016/j.ajog.2013.05.006
Klostermann K, 2008, J DAIRY RES, V75, P365, DOI 10.1017/S0022029908003373
Labov JB, 2011, EVOL ED OUTREACH, V4, P561, DOI [10.1007/s12052-011-0367-9, DOI 10.1007/S12052-011-0367-9.]
Lamont RF, 2011, BJOG-INT J OBSTET GY, V118, P533, DOI 10.1111/j.1471-0528.2010.02840.x
Ma B, 2012, ANNU REV MICROBIOL, V66, P371, DOI 10.1146/annurev-micro-092611-150157
Mannucci PM, 2015, INTERN EMERG MED, V10, P657, DOI 10.1007/s11739-015-1276-7
Mayr E., 1982, of the evolution of scientific thought and medical
McDonald B, 2019, SCIENCE, V365, P984, DOI 10.1126/science.aay0618
Mendola P, 2019, INT J ENV RES PUB HE, V16, DOI 10.3390/ijerph16183365
Montoya-Williams D, 2018, NEONATOLOGY, V114, P103, DOI 10.1159/000487102
Muletz-Wolz CR, 2019, AM J PRIMATOL, V81, DOI 10.1002/ajp.22994
Nasioudis D, 2017, BJOG-INT J OBSTET GY, V124, P61, DOI 10.1111/1471-0528.14209
Nesse R.M., 1994, WHY WE GET SICK NEW
Nesse RM, 2003, BIOSCIENCE, V53, P585, DOI 10.1641/0006-3568(2003)053[0585:EBITMS]2.0.CO;2
NUGENT RP, 1991, J CLIN MICROBIOL, V29, P297, DOI 10.1128/JCM.29.2.297-301.1991
Paz S, 2016, LANCET, V387, P745, DOI 10.1016/S0140-6736(16)00256-7
Pennisi E, 2011, SCIENCE, V334, P1486, DOI 10.1126/science.334.6062.1486
Perlman RL, 2013, Evolution and Medicine, P1
Pimenoff VN, 2018, GENES-BASEL, V9, DOI 10.3390/genes9070317
Power ML, 2013, APPL TRANSL GENOM, V2, P55, DOI 10.1016/j.atg.2013.06.001
Power MichaelL., 2016, Milk: the biology of lactation
Rajagopalan S, 2018, J AM COLL CARDIOL, V72, P2054, DOI 10.1016/j.jacc.2018.07.099
Ravel J, 2011, P NATL ACAD SCI USA, V108, P4680, DOI 10.1073/pnas.1002611107
Rosenberg K, 2002, BJOG-INT J OBSTET GY, V109, P1199, DOI 10.1016/S1470-0328(02)00410-X

Schulkin J., 2020, INTEGRATING EVOLUTIO, V1st ed.
Soleimani N. A., 2010, African Journal of Microbiology Research, V4, P2169
Stearns S. C., 2015, Evolutionary Medicine
Stearns S.C., 2008, Evolution in health and disease
Stuebe AM, 2020, INTEGRATING EVOLUTIONARY BIOLOGY INTO MEDICAL EDUCATION: FOR MATERNAL AND CHILD HEALTHCARE STUDENTS, CLINICIANS, AND SCIENTISTS, P67, DOI 10.1093/oso/9780198814153.003.0005
Thomas F, 2016, TRENDS CANCER, V2, P409, DOI 10.1016/j.trecan.2016.06.009
Tinbergen N., 1963, Zeitschrift fuer Tierpsychologie, V20, P410
Trevathan WR, 2020, INTEGRATING EVOLUTIONARY BIOLOGY INTO MEDICAL EDUCATION: FOR MATERNAL AND CHILD HEALTHCARE STUDENTS, CLINICIANS, AND SCIENTISTS, P47, DOI 10.1093/oso/9780198814153.003.0004
Trevathan WR, 1999, EVOLUTIONARY MEDICI NE, P183
Van Boeckel TP, 2019, SCIENCE, V365, P1266, DOI 10.1126/science.aaw1944
Wells JCK, 2020, INTEGRATING EVOLUTIONARY BIOLOGY INTO MEDICAL EDUCATION: FOR MATERNAL AND CHILD HEALTHCARE STUDENTS, CLINICIANS, AND SCIENTISTS, P27, DOI 10.1093/oso/9780198814153.003.0003
Wells JCK, 2017, LANCET, V390, P500, DOI 10.1016/S0140-6736(17)30572-X
Wilson DS, 2005, PLOS BIOL, V3, P2058, DOI 10.1371/journal.pbio.0030364
Yildirim S, 2014, ISME J, V8, P2431, DOI 10.1038/ismej.2014.90
Zhang LQ, 2019, NAT SUSTAIN, V2, P1011, DOI 10.1038/s41893-019-0387-y
NR 71
TC 4
Z9 6
U1 1
U2 9
PU OXFORD UNIV PRESS
PI OXFORD
PA GREAT CLARENDON ST, OXFORD OX2 6DP, ENGLAND
EI 2050-6201
J9 EVOL MED PUBLIC HLTH
JI Evol. Med. Public Health
PD APR 6
PY 2020
IS 1
BP 60
EP 67
DI 10.1093/emph/eoaa009
PG 8
WC Evolutionary Biology; Public, Environmental & Occupational Health
WE Science Citation Index Expanded (SCI-EXPANDED)
SC Evolutionary Biology; Public, Environmental & Occupational Health
GA LP5FQ
UT WOS:000534343700001
PM 32382419
OA Green Published, gold, Green Submitted
DA 2025-05-07
ER

PT J

AU Lonfat, BMK
   Kaufmann, IM
   Rühli, F
AF Lonfat, Bettina M. Kreissl
   Kaufmann, Ina Maria
   Ruehli, Frank
TI A Code of Ethics for Evidence-Based Research With Ancient Human Remains
SO ANATOMICAL RECORD-ADVANCES IN INTEGRATIVE ANATOMY AND EVOLUTIONARY BIOLOGY
LA English
DT Article
DE medical ethics; research ethics; human remains; personal rights
ID DECLARATION; HELSINKI; DEATH
AB As clinical research constantly advances and the concept of evolution becomes a strong and influential part of basic medical research, the absence of a discourse that deals with the use of ancient human remains in evidence-based research is becoming unbearable. While topics such as exhibition and excavation of human remains are established ethical fields of discourse, when faced with instrumentalization of ancient human remains for research (i.e., ancient DNA extractions for disease marker analyses) the answers from traditional ethics or even more practical fields of bio-ethics or more specific biomedical ethics are rare to non-existent. The Centre for Evolutionary Medicine at the University of Zurich solved their needs for discursive action through the writing of a self-given code of ethics which was written in dialogue with the researchers at the Institute and was published online in Sept. 2011: . The philosophico-ethical basis for this a code of conduct and ethics and the methods are published in this article. Anat Rec, 298:1175-1181, 2015. (c) 2015 Wiley Periodicals, Inc.
C1 [Lonfat, Bettina M. Kreissl; Kaufmann, Ina Maria; Ruehli, Frank] Univ Zurich, Inst Anat, Ctr Evolutionary Med, CH-8057 Zurich, Switzerland.
C3 University of Zurich
RP Rühli, F (autor correspondente), Univ Zurich, Fac Med, Inst Evolutionary Med, Bldg 42,Floor G,Room 70,Winterthurerstr 190, CH-8057 Zurich, Switzerland.
EM frank.ruehli@iem.uzh.ch
RI Ruhli, Frank/H-8789-2017
OI Ruhli, Frank/0000-0001-7937-001X
FU Maxi Foundation
FX Grant sponsor: Maxi Foundation.
CR Cantor NL, 2010, AFTER WE DIE
   Centre for Evolutionary Medicine University of Zurich ed, COD ETH NEW PRINC ET
   Christie B, 2000, BRIT MED J, V321, P913, DOI 10.1136/bmj.321.7266.913
   Collier CDA, 2003, SOCIOL QUART, V44, P727, DOI 10.1111/j.1533-8525.2003.tb00533.x
   Dawson A.J., 1994, J APPL PHILOS, V11, P145, DOI DOI 10.1111/J.1468-5930.1994.TB00104.X
   FORD RC, 1994, J BUS ETHICS, V13, P205, DOI 10.1007/BF02074820
   FREY R.G., 2003, COMPANION APPL ETHIC
   Goodyear MDE, 2007, BRIT MED J, V335, P624, DOI 10.1136/bmj.39339.610000.BE
   Green ErnesteneL., 1984, ETHICS VALUES ARCHAE

   Habermas Jurgen., 1994, JUSTIFICATION APPL
   Kaestle FA, 2002, YEARB PHYS ANTHROPOL, V45, P92, DOI
10.1002/ajpa.10179
   Kaufmann IM, 2010, J MED ETHICS, V36, P608, DOI
10.1136/jme.2010.036608
   Kuffer A., 2007, Unter dem Schutz der Himmelsgottin: Agyptische Sarge,
Mumien und Masken in der Schweiz
   LEVENBOOK BB, 1984, ETHICS, V94, P407, DOI 10.1086/292557
   Museums ICO, PREAMBLE COD ETH ICO
   Nations GAOTU, 1966, INT COV CIV POL RIGH
   Rawls John., 2000, LECT HIST MORAL PHIL
   Rühli FJ, 2007, JAMA-J AM MED ASSOC, V298, P2618, DOI
10.1001/jama.298.22.2618-b
   Seiler R, 2011, 2 BOLZ MUMM C MUMM I
   Swiss Academy of Medical Sciences, 2008, GUID US HUM BOD PART
   Wanek J, 2011, SKELETAL RADIOL, V40, P595, DOI 10.1007/s00256-010-
1035-9
   Wilkinson T M, 2002, J Appl Philos, V19, P31, DOI 10.1111/1468-
5930.00202
NR 22
TC 19
Z9 23
U1 2
U2 18
PU WILEY-BLACKWELL
PI HOBOKEN
PA 111 RIVER ST, HOBOKEN 07030-5774, NJ USA
SN 1932-8486
EI 1932-8494
J9 ANAT REC
JI Anat. Rec.
PD JUN
PY 2015
VL 298
IS 6
BP 1175
EP 1181
DI 10.1002/ar.23126
PG 7
WC Anatomy & Morphology
WE Science Citation Index Expanded (SCI-EXPANDED); Social Science
Citation Index (SSCI); Arts & Humanities Citation Index (A&HCI)
SC Anatomy & Morphology
GA CI8EX
UT WOS:000355003600026
PM 25998650
OA Bronze
DA 2025-05-07
ER

PT J
AU Rossier, BC
   Bochud, M
   Devuyst, O

AF Rossier, Bernard C.
   Bochud, Murielle
   Devuyst, Olivier
TI The Hypertension Pandemic: An Evolutionary Perspective
SO PHYSIOLOGY
LA English
DT Review
ID SALT-SENSITIVE HYPERTENSION; GENOME-WIDE ASSOCIATION; BLOOD-PRESSURE;
   NORADRENERGIC MECHANISMS; POTASSIUM-DEPLETION; CARDIAC-HYPERTROPHY;
   ZYMOGEN ACTIVATION; NATURAL-SELECTION; SODIUM-EXCRETION; SEXUAL-
ACTIVITY
AB Hypertension affects over 1.2 billion individuals worldwide and has become the most critical and expensive public health problem. Hypertension is a multifactorial disease involving environmental and genetic factors together with risk-conferring behaviors. The cause of the disease is identified in similar to 10% of the cases (secondary hypertension), but in 90% of the cases no etiology is found (primary or essential hypertension). For this reason, a better understanding of the mechanisms controlling blood pressure in normal and hypertensive patients is the aim of very active experimental and clinical research. In this article, we review the importance of the renin-angiotensin-aldosterone system (RAAS) for the control of blood pressure, focusing on the evolution of the system and its critical importance for adaptation of vertebrates to a terrestrial and dry environment. The evolution of blood pressure control during the evolution of primates, hominins, and humans is discussed, together with the role of common genetic factors and the possible causes of the current hypertension pandemic in the light of evolutionary medicine.
C1 [Rossier, Bernard C.] Univ Lausanne, Dept Pharmacol & Toxicol, Lausanne, Switzerland.
   [Bochud, Murielle] Univ Lausanne Hosp, Inst Social & Prevent Med, Lausanne, Switzerland.
   [Devuyst, Olivier] Univ Zurich, Inst Physiol, Zurich, Switzerland.
C3 University of Lausanne; University of Lausanne; Centre Hospitalier
   Universitaire Vaudois (CHUV); University of Zurich
RP Rossier, BC (autor correspondente), Univ Lausanne, Dept Pharmacol & Toxicol, Lausanne, Switzerland.
EM Bernard.Rossier@unil.ch
RI Bochud, Murielle/A-3981-2010
OI Devuyst, Olivier/0000-0003-3744-4767
FU European Community's Seventh Framework Programme [305608]; Swiss
   National Centre of Competence in Research Kidney Control of Homeostasis
   (NCCR Kidney.CH) program; Swiss National Science Foundation
   [310030146490]; Rare Disease Initiative Zurich (radiz); TransCure; Swiss
   National Science Foundation for a Special Program in University Medicine
   multicentric project [33CM30-124087/1, 30CM30_140331]
FX O.D. is supported by grants from the European Community's Seventh
   Framework Programme (305608 EURenOmics), the Swiss National Centre of
   Competence in Research Kidney Control of Homeostasis (NCCR Kidney.CH)
   program, the Swiss National Science Foundation (310030146490), and the
   Rare Disease Initiative Zurich (radiz), a clinical research priority

program of the University of Zurich, Switzerland. M.B. is supported by the Swiss National Centre of Competence in Research Kidney Control of Homeostasis (NCCR Kidney.CH) and TransCure (www.nccrtranscure.ch) programs. She received support from Swiss National Science Foundation for a Special Program in University Medicine multicentric project (33CM30-124087/1; 30CM30_140331).
CR Abegglen LM, 2015, JAMA-J AM MED ASSOC, V314, P1850, DOI 10.1001/jama.2015.13134
Aburto NJ, 2013, BMJ-BRIT MED J, V346, DOI 10.1136/bmj.f1378
Alwan H, 2015, J HYPERTENS, V33, P2061, DOI 10.1097/HJH.0000000000000681
Ares GR, 2011, AM J PHYSIOL-RENAL, V301, pF1143, DOI 10.1152/ajprenal.00396.2011
Arnold AC, 2013, CURR HYPERTENS REP, V15, P304, DOI 10.1007/s11906-013-0362-3
August P, 2013, ADV CHRONIC KIDNEY D, V20, P396, DOI 10.1053/j.ackd.2013.07.002
Babbitt CC, 2011, P ROY SOC B-BIOL SCI, V278, P961, DOI 10.1098/rspb.2010.2433
Bankir L, 2010, AM J PHYSIOL-RENAL, V299, pF917, DOI 10.1152/ajprenal.00413.2010
Binia A, 2015, J HYPERTENS, V33, P1509, DOI 10.1097/HJH.0000000000000611
Bremner JD, 1996, SYNAPSE, V23, P39, DOI 10.1002/(SICI)1098-2396(199605)23:1<39::AID-SYN5>3.0.CO;2-I
Bremner JD, 1996, SYNAPSE, V23, P28, DOI 10.1002/(SICI)1098-2396(199605)23:1<28::AID-SYN4>3.3.CO;2-4
Castro H, 2013, SEMIN NEPHROL, V33, P277, DOI 10.1016/j.semnephrol.2013.04.008
Chen L, 2008, PLOS MED, V5, DOI 10.1371/journal.pmed.0050052
Claas SA, 2016, CURR CARDIOL REP, V18, DOI 10.1007/s11886-016-0728-7
Coffman TM, 2011, NAT MED, V17, P1402, DOI 10.1038/nm.2541
Cogswell ME, 2016, NEW ENGL J MED, V375, P580, DOI 10.1056/NEJMsb1607161
Coruzzi P, 2001, J CLIN ENDOCR METAB, V86, P2857, DOI 10.1210/jc.86.6.2857
Dong NZ, 2013, J BIOL CHEM, V288, P7867, DOI 10.1074/jbc.M112.411512
Dries DL, 2005, CIRCULATION, V112, P2403, DOI 10.1161/CIRCULATIONAHA.105.568881
Ehret GB, 2011, NATURE, V478, P103, DOI 10.1038/nature10405
Ehret GB, 2010, CURR HYPERTENS REP, V12, P17, DOI 10.1007/s11906-009-0086-6
Ellison David H, 2015, Trans Am Clin Climatol Assoc, V126, P46
Ettehad D, 2016, LANCET, V387, P957, DOI 10.1016/S0140-6736(15)01225-8
Ewald PW, 2013, EVOL APPL, V6, P70, DOI 10.1111/eva.12023
Ewald PW, 2012, J MOL MED, V90, P535, DOI 10.1007/s00109-012-0891-2
EWALD PW, 1995, J PARASITOL, V81, P659, DOI 10.2307/3283951
Feigin VL, 2016, LANCET NEUROL, V15, P913, DOI 10.1016/S1474-4422(16)30073-4
Forouzanfar MH, 2015, LANCET, V386, P2287, DOI 10.1016/S0140-6736(15)00128-2
Fournier D, 2012, J MOL MED, V90, P495, DOI 10.1007/s00109-012-0894-z
Fumagalli M, 2011, PLOS GENET, V7, DOI 10.1371/journal.pgen.1002355

Ghirotto S, 2016, J AM SOC NEPHROL, V27, P2983, DOI 10.1681/ASN.2015070830
Graham LA, 2014, HYPERTENSION, V63, P551, DOI 10.1161/HYPERTENSIONAHA.113.01423
Guessous I, 2014, CURR HYPERTENS REP, V16, DOI 10.1007/s11906-014-0468-2
Hall JE, 1996, KIDNEY INT, pS35
Hedayati SS, 2012, CLIN J AM SOC NEPHRO, V7, P315, DOI 10.2215/CJN.02060311
Hottenga JJ, 2005, TWIN RES HUM GENET, V8, P499, DOI 10.1375/twin.8.5.499
JACOB F, 1977, SCIENCE, V196, P1161, DOI 10.1126/science.860134
Jeunemaitre X, 1997, AM J HUM GENET, V60, P1448, DOI 10.1086/515452
JEUNEMAITRE X, 1992, CELL, V71, P169, DOI 10.1016/0092-8674(92)90275-H
Ji WZ, 2008, NAT GENET, V40, P592, DOI 10.1038/ng.118
Kaminski P, 2013, NEUROENDOCRINOL LETT, V34, P585
Kario K, 2013, NAT REV NEPHROL, V9, P726, DOI 10.1038/nrneph.2013.224
Kato N, 2015, NAT GENET, V47, P1282, DOI 10.1038/ng.3405
Kim Darae, 2016, Clin Hypertens, V22, P17, DOI 10.1186/s40885-016-0052-y
Kosuge K, 2007, MOL PHARMACOL, V72, P826, DOI 10.1124/mol.107.034504
KRISHNA GG, 1989, NEW ENGL J MED, V320, P1177, DOI 10.1056/NEJM198905043201804
Kuhn M, 2016, PHYSIOL REV, V96, P751, DOI 10.1152/physrev.00022.2015
Langley SR, 2013, CARDIOVASC RES, V97, P653, DOI 10.1093/cvr/cvs329
Lek M, 2016, NATURE, V536, P285, DOI 10.1038/nature19057
Lewington S, 2002, LANCET, V360, P1903, DOI 10.1016/S0140-6736(02)11911-8
Lifton RP, 2001, CELL, V104, P545, DOI 10.1016/S0092-8674(01)00241-0
Lifton RP, 2002, COLD SPRING HARB SYM, V67, P445, DOI 10.1101/sqb.2002.67.445
Luca F, 2010, ANNU REV NUTR, V30, P291, DOI 10.1146/annurev-nutr-080508-141048
Manolio TA, 2009, NATURE, V461, P747, DOI 10.1038/nature08494
Meneton P, 2005, PHYSIOL REV, V85, P679, DOI 10.1152/physrev.00056.2003
Mente A, 2016, LANCET, V388, P465, DOI 10.1016/S0140-6736(16)30467-6
Montani JP, 2002, INT J OBESITY, V26, pS28, DOI 10.1038/sj.ijo.0802125
Nakajima T, 2004, AM J HUM GENET, V74, P898, DOI 10.1086/420793
NEEL JV, 1962, AM J HUM GENET, V14, P353
NEMEC ED, 1976, AM HEART J, V92, P274, DOI 10.1016/S0002-8703(76)80106-8
OLIVER WJ, 1975, CIRCULATION, V52, P146, DOI 10.1161/01.CIR.52.1.146
Padmanabhan S, 2015, CIRC RES, V116, P937, DOI 10.1161/CIRCRESAHA.116.303647
Padmanabhan S, 2010, PLOS GENET, V6, DOI 10.1371/journal.pgen.1001177
Pechère-Bertschi A, 2004, AM J HYPERTENS, V17, P994, DOI 10.1016/j.amjhyper.2004.08.009
Pilic L, 2016, NUTR REV, V74, P645, DOI 10.1093/nutrit/nuw028
Prentice AM, 2008, INT J OBESITY, V32, P1607, DOI 10.1038/ijo.2008.147
Rahimi K, 2015, CIRC RES, V116, P925, DOI 10.1161/CIRCRESAHA.116.304723
Rame JE, 2007, HYPERTENSION, V49, P857, DOI 10.1161/01.HYP.0000258566.95867.9e

Rampoldi L, 2011, KIDNEY INT, V80, P338, DOI 10.1038/ki.2011.134
Roger VL, 2012, CIRCULATION, V125, pE2, DOI [10.1161/CIR.0b013e31823ac046, 10.1161/CIR.0b013e3182456d46]
Rossier BC, 2016, NEPHRON, V134, P5, DOI 10.1159/000444307
Rossier BC, 2015, PHYSIOL REV, V95, P297, DOI 10.1152/physrev.00011.2014
Rossier BC, 2013, FEBS LETT, V587, P1929, DOI 10.1016/j.febslet.2013.05.013
Sacks FM, 1999, CLIN CARDIOL, V22, P6, DOI 10.1002/clc.4960221503
Sacks FM, 2001, NEW ENGL J MED, V344, P3, DOI 10.1056/NEJM200101043440101
Schiffman JD, 2015, PHILOS T R SOC B, V370, DOI 10.1098/rstb.2014.0231
Schulte K, 2015, NEPHROL DIAL TRANSPL, V30, P713, DOI 10.1093/ndt/gfu275
Speakman JR, 2008, INT J OBESITY, V32, P1611, DOI 10.1038/ijo.2008.161
Studer RA, 2013, BIOCHEM J, V449, P581, DOI 10.1042/BJ20121221
Sudmant PH, 2015, NATURE, V526, P75, DOI 10.1038/nature15394
Taylor RS, 2011, COCHRANE DB SYST REV, DOI 10.1002/14651858.CD009217
te Riet L, 2015, CIRC RES, V116, P960, DOI 10.1161/CIRCRESAHA.116.303587
Terker AS, 2015, CELL METAB, V21, P39, DOI 10.1016/j.cmet.2014.12.006
Theilig F, 2015, AM J PHYSIOL-RENAL, V308, pF1047, DOI 10.1152/ajprenal.00164.2014
Thomas O, 2016, J HUM HYPERTENS, V30, P386, DOI 10.1038/jhh.2015.95
Thompson EE, 2004, AM J HUM GENET, V75, P1059, DOI 10.1086/426406
Trudu M, 2013, NAT MED, V19, P1655, DOI 10.1038/nm.3384
TRUSWELL AS, 1972, AM HEART J, V84, P5, DOI 10.1016/0002-8703(72)90299-2
Turner BL, 2013, NUTR REV, V71, P501, DOI 10.1111/nure.12039
Valente C, 2015, BMC GENET, V16, DOI 10.1186/s12863-015-0212-1
Wang W, 2012, HYPERTENSION, V60, P1352, DOI 10.1161/HYPERTENSIONAHA.112.201244
Wang W, 2012, KIDNEY INT, V82, P26, DOI 10.1038/ki.2012.41
Wang W, 2008, CIRC RES, V103, P502, DOI 10.1161/CIRCRESAHA.108.177352
Wasser WG, 2012, J NEPHROL, V25, P603, DOI 10.5301/jn.5000179
Watkins WS, 2010, J HYPERTENS, V28, P65, DOI 10.1097/HJH.0b013e328332031a
Whelton SP, 2002, ANN INTERN MED, V136, P493, DOI 10.7326/0003-4819-136-7-200204020-00006
WIINBERG N, 1995, AM J HYPERTENS, V8, P978, DOI 10.1016/0895-7061(95)00216-2
Wu QY, 2009, KIDNEY INT, V75, P142, DOI 10.1038/ki.2008.418
Xue-rui T, 2008, BLOOD PRESS MONIT, V13, P211, DOI 10.1097/MBP.0b013e3283057a71
Yang QH, 2011, ARCH INTERN MED, V171, P1183, DOI 10.1001/archinternmed.2011.257
Ye KX, 2011, ADV NUTR, V2, P486, DOI 10.3945/an.111.001024
NR 101
TC 124
Z9 131
U1 0
U2 45
PU AMER PHYSIOLOGICAL SOC
PI BETHESDA

PA 9650 ROCKVILLE PIKE, BETHESDA, MD 20814 USA
SN 1548-9213
EI 1548-9221
J9 PHYSIOLOGY
JI Physiology
PD MAR
PY 2017
VL 32
IS 2
BP 112
EP 125
DI 10.1152/physiol.00026.2016
PG 14
WC Physiology
WE Science Citation Index Expanded (SCI-EXPANDED)
SC Physiology
GA EN8EF
UT WOS:000396233000005
PM 28202622
DA 2025-05-07
ER

PT J
AU Mansley, MK
Ivy, JR
Bailey, MA
AF Mansley, Morag K.
Ivy, Jessica R.
Bailey, Matthew A.
TI ISN Forefronts Symposium 2015: The Evolution of Hypertension-Old Genes,
New Concepts
SO KIDNEY INTERNATIONAL REPORTS
LA English
DT Article
DE blood pressure; evolution; hypertension; inflammation; kidney; pressure
natriuresis; salt
ID URINARY SODIUM-EXCRETION; BLOOD-PRESSURE; RENAL DENERVATION; SALT;
ALDOSTERONE; KIDNEY; ASSOCIATION; VARIANTS; HSD11B2; CELLS
AB Hypertension is known as the "silent killer," driving the global public health burden of cardiovascular and renal disease. Blood pressure homeostasis is intimately associated with sodium balance and the distribution of sodium between fluid compartments and within tissues. On a population level, most societies consume 10 times more salt that the 0.5 g required by physiological need. This high salt intake is strongly linked to hypertension and to the World Health Organization targeting a similar to 30% relative reduction in mean population salt intake to arrest the global mortality due to cardiovascular disease. But how does a habitually high-salt diet cause blood pressure to rise? In this focused review, we discuss 2 "evolutionary medicine" concepts, presented at the ISN Forefront Meeting "Immunomodulation of Cardio-renal Function." We first examine how ancestral variants in genes that conferred a selection advantage during early human development are now maladaptive. We then

discuss the conservation of "renal" sodium transport processes across multiple organ systems, including the brain. These systems influence sodium appetite and can exert an often-overlooked effect on long-term blood pressure control.
C1 [Mansley, Morag K.; Ivy, Jessica R.; Bailey, Matthew A.] Univ Edinburgh, British Heart Fdn Ctr Cardiovasc Sci, Edinburgh, Midlothian, Scotland.
C3 University of Edinburgh
RP Bailey, MA (autor correspondente), Univ Edinburgh, Queens Med Res Inst, BHF Ctr Cardiovasc Sci, Edinburgh EH16 6NT, Midlothian, Scotland.
EM matthew.bailey@ed.ac.uk
RI Bailey, Matthew/AAH-3395-2020; Mansley, Morag/AAQ-2837-2020; Ivy, Jessica/AAY-6150-2021
OI Bailey, Matthew/0000-0003-4244-5668; Ivy, Jessica/0000-0003-1004-4376; Mansley, Morag/0000-0002-8082-0273
FU British Heart Foundation; Kidney Research UK
FX The authors thank The British Heart Foundation and Kidney Research UK for research funding.
CR [Anonymous], 2012, Guideline: Sodium intake for adults and children
Bailey MA, 2008, J AM SOC NEPHROL, V19, P47, DOI 10.1681/ASN.2007040401
Bailey MA, 2011, HYPERTENSION, V57, P515, DOI 10.1161/HYPERTENSIONAHA.110.163782
Bailey MA, 2009, HYPERTENSION, V54, P890, DOI 10.1161/HYPERTENSIONAHA.109.134973
Bankir L, 2008, HYPERTENSION, V51, P891, DOI 10.1161/HYPERTENSIONAHA.107.105510
Bhatt DL, 2014, NEW ENGL J MED, V370, P1393, DOI 10.1056/NEJMoa1402670
Bie P., 2016, ACTA PHYSL
Birukov A, 2016, AM J CLIN NUTR, V104, P49, DOI 10.3945/ajcn.116.132951
Chandrashekar J, 2010, NATURE, V464, P297, DOI 10.1038/nature08783
Chapman K, 2013, PHYSIOL REV, V93, P1139, DOI 10.1152/physrev.00020.2012
Colin-Ramirez E, 2015, J CARDIOVASC NURS, V30, P58, DOI 10.1097/JCN.0000000000000124
de Souza JT, 2012, APPETITE, V58, P418, DOI 10.1016/j.appet.2011.09.021
Di Rienzo A, 2005, TRENDS GENET, V21, P596, DOI 10.1016/j.tig.2005.08.007
Evans LC, 2016, CIRCULATION, V133, P1360, DOI 10.1161/CIRCULATIONAHA.115.019341
Geerling JC, 2006, J NEUROSCI, V26, P411, DOI 10.1523/JNEUROSCI.3115-05.2006
Geerling JC, 2008, EXP PHYSIOL, V93, P177, DOI 10.1113/expphysiol.2007.039891
Genovese G, 2010, SCIENCE, V329, P841, DOI 10.1126/science.1193032
Ghirotto S., J Am Soc Nephrol
Gomez-Sanchez EP, 2005, AM J PHYSIOL-ENDOC M, V288, pE342, DOI 10.1152/ajpendo.00355.2004
GOMEZSANCHEZ EP, 1992, AM J PHYSIOL, V263, pE1125
GOMEZSANCHEZ EP, 1986, ENDOCRINOLOGY, V118, P819, DOI 10.1210/endo-118-2-819

GUYTON AC, 1972, ANNU REV PHYSIOL, V34, P13, DOI 10.1146/annurev.ph.34.030172.000305
Hall JE, 2016, CIRCULATION, V133, P894, DOI 10.1161/CIRCULATIONAHA.115.018526
Hunter RW, 2015, CURR OPIN PHARMACOL, V21, P105, DOI 10.1016/j.coph.2015.01.005
Ingram MC, 1996, CLIN EXP PHARMACOL P, V23, P375, DOI 10.1111/j.1440-1681.1996.tb02744.x
Ivy JR, 2014, J PHYSIOL-LONDON, V592, P3955, DOI 10.1113/jphysiol.2014.271676
JEUNEMAITRE X, 1992, CELL, V71, P169, DOI 10.1016/0092-8674(92)90275-H
Kario K, CIRC J
Kato N, 2015, NAT GENET, V47, P1282, DOI 10.1038/ng.3405
Kearney PM, 2005, LANCET, V365, P217, DOI 10.1016/S0140-6736(05)70151-3
Köttgen A, 2010, NAT GENET, V42, P376, DOI 10.1038/ng.568
Köttgen A, 2009, NAT GENET, V41, P712, DOI 10.1038/ng.377
Krum H, 2014, LANCET, V383, P622, DOI 10.1016/S0140-6736(13)62192-3
Krum H, 2009, LANCET, V373, P1275, DOI 10.1016/S0140-6736(09)60566-3
Mansley MK, 2015, AM J PHYSIOL-RENAL, V308, pF450, DOI 10.1152/ajprenal.00548.2014
Mente A, 2016, LANCET, V388, P465, DOI 10.1016/S0140-6736(16)30467-6
Morris RC, 2016, CIRCULATION, V133, P881, DOI 10.1161/CIRCULATIONAHA.115.017923
Mozaffarian D, 2015, CIRCULATION, V131, pE29, DOI 10.1161/CIR.0000000000000152
Mullins LJ, 2006, PHYSIOL REV, V86, P709, DOI 10.1152/physrev.00016.2005
Mune T, 2013, HYPERTENS RES, V36, P614, DOI 10.1038/hr.2013.13
Nakajima T, 2004, AM J HUM GENET, V74, P898, DOI 10.1086/420793
NEEL JV, 1962, AM J HUM GENET, V14, P353
Padmanabhan S, 2010, PLOS GENET, V6, DOI 10.1371/journal.pgen.1001177
Rossier BC, 2015, PHYSIOL REV, V95, P297, DOI 10.1152/physrev.00011.2014
STAMLER J, 1989, HYPERTENSION, V14, P570, DOI 10.1161/01.HYP.14.5.570
Terker AS, 2014, HYPERTENSION, V64, P178, DOI 10.1161/HYPERTENSIONAHA.114.03335
Trudu M, 2013, NAT MED, V19, P1655, DOI 10.1038/nm.3384
Van Huysse JW, 2012, HYPERTENSION, V60, P691, DOI 10.1161/HYPERTENSIONAHA.112.193045
Wang HW, 2016, NEUROSCIENCE, V314, P90, DOI 10.1016/j.neuroscience.2015.11.055
Warnock DG, 2014, NAT REV NEPHROL, V10, P146, DOI 10.1038/nrneph.2013.275
Weder AB, 2007, HYPERTENSION, V49, P260, DOI 10.1161/01.HYP.0000255165.84684.9d
World Health Organization, 2013, GLOB BRIEF HYP
Wyrwoll C, 2015, PSYCHONEUROENDOCRINO, V59, P59, DOI 10.1016/j.psyneuen.2015.05.003
Young JH, 2005, PLOS GENET, V1, P730, DOI 10.1371/journal.pgen.0010082
NR 54
TC 7
Z9 6
U1 0

U2 4
PU ELSEVIER SCIENCE INC
PI NEW YORK
PA STE 800, 230 PARK AVE, NEW YORK, NY 10169 USA
SN 2468-0249
J9 KIDNEY INT REP
JI Kidney Int. Rep.
PD SEP
PY 2016
VL 1
IS 3
BP 197
EP 203
DI 10.1016/j.ekir.2016.08.003
PG 7
WC Urology & Nephrology
WE Emerging Sources Citation Index (ESCI)
SC Urology & Nephrology
GA FB1ZO
UT WOS:000405943000012
PM 27722209
OA gold, Green Published
DA 2025-05-07
ER

PT J
AU van Dijk, G
   van Heijningen, S
   Reijne, AC
   Nyakas, C
   van der Zee, EA
   Eisel, ULM
AF van Dijk, Gertjan
   van Heijningen, Steffen
   Reijne, Aaffien C.
   Nyakas, Csaba
   van der Zee, Eddy A.
   Eisel, Ulrich L. M.
TI Integrative neurobiology of metabolic diseases, neuroinflammation, and
   neurodegeneration
SO FRONTIERS IN NEUROSCIENCE
LA English
DT Review
DE neuroinflammation; obesity; metabolic syndrome; type-2 diabetes
   mellitus; TNF; blood-brain barrier; aging; Alzheimer's disease
ID GROWTH-FACTOR-I; HIGH-FAT-DIET; NECROSIS-FACTOR-ALPHA;
   CENTRAL-NERVOUS-SYSTEM; AMYLOID-BETA-PROTEIN; BODY-MASS INDEX;
   TRIGLYCERIDE-RICH LIPOPROTEINS; MILD COGNITIVE IMPAIRMENT; BRAIN-
BARRIER
   TRANSPORT; OBESE ZUCKER RATS
AB Alzheimer's disease (AD) is a complex, multifactorial disease with a
number of leading mechanisms, including neuroinflammation, processing of
amyloid precursor protein (APP) to amyloid peptide, tau protein
hyperphosphorylation, relocalization, and deposition. These mechanisms

are propagated by obesity, the metabolic syndrome and type-2 diabetes mellitus. Stress, sedentariness, dietary overconsumption of saturated fat and refined sugars, and circadian derangements/disturbed sleep contribute to obesity and related metabolic diseases, but also accelerate age-related damage and senescence that all feed the risk of developing AD too. The complex and interacting mechanisms are not yet completely understood and will require further analysis. Instead of investigating AD as a mono- or oligocausal disease we should address the disease by understanding the multiple underlying mechanisms and how these interact. Future research therefore might concentrate on integrating these by "systems biology" approaches, but also to regard them from an evolutionary medicine point of view. The current review addresses several of these interacting mechanisms in animal models and compares them with clinical data giving an overview about our current knowledge and puts them into an integrated framework.
C1 [van Dijk, Gertjan; van Heijningen, Steffen; Reijne, Aaffien C.] Univ Groningen, Groningen Inst Evolutionary Life Sci, Dept Behav Neurosci, NL-9747 AG Groningen, Netherlands.
[Reijne, Aaffien C.] Univ Groningen, Univ Med Ctr Groningen, Syst Biol Ctr Energy Metab & Ageing, NL-9747 AG Groningen, Netherlands.
[Nyakas, Csaba; van der Zee, Eddy A.; Eisel, Ulrich L. M.] Univ Groningen, Groningen Inst Evolutionary Life Sci, Dept Mol Neurobiol, NL-9747 AG Groningen, Netherlands.
[Eisel, Ulrich L. M.] Univ Groningen, Univ Med Ctr Groningen, Univ Ctr Psychiat, NL-9747 AG Groningen, Netherlands.
C3 University of Groningen; University of Groningen; University of Groningen; University of Groningen
RP van Dijk, G (autor correspondente), Univ Groningen, Groningen Inst Evolutionary Life Sci, Dept Behav Neurosci, Nijenborgh 7, NL-9747 AG Groningen, Netherlands.
EM gertjan.van.dijk@rug.nl
RI van der Zee, Eddy/AAZ-2120-2020
OI Eisel, Ulrich/0000-0003-4178-0384; van Dijk, Gertjan/0000-0002-6565-4019
CR Åberg ND, 2010, ENDOCR DEV, V17, P63, DOI 10.1159/000262529
Åberg ND, 2000, ENDOCRINOLOGY, V141, P3879, DOI 10.1210/en.141.10.3879
Aguiar AS, 2011, MECH AGEING DEV, V132, P560, DOI 10.1016/j.mad.2011.09.005
AHMAD I, 1989, MOL CELL ENDOCRINOL, V65, P103, DOI 10.1016/0303-7207(89)90170-6
Akassoglou K, 1997, J IMMUNOL, V158, P438
Anstey KJ, 2014, INT J EPIDEMIOL, V43, P1874, DOI 10.1093/ije/dyu170
ARAUJO DM, 1989, BRAIN RES, V498, P257, DOI 10.1016/0006-8993(89)91104-9
Aronne LJ, 1997, OBES RES, V5, P354, DOI 10.1002/j.1550-8528.1997.tb00564.x
Avena NM, 2007, EXP CLIN PSYCHOPHARM, V15, P481, DOI 10.1037/1064-1297.15.5.481
Bagyinszky E, 2014, CLIN INTERV AGING, V9, P535, DOI 10.2147/CIA.S51571
Balasa B, 2000, J IMMUNOL, V165, P7330, DOI 10.4049/jimmunol.165.12.7330
Banks WA, 2005, CURR PHARM DESIGN, V11, P973, DOI 10.2174/1381612053381684

Banks WA, 1996, PEPTIDES, V17, P305, DOI 10.1016/0196-9781(96)00025-3
Banks WA, 2004, DIABETES, V53, P1253, DOI 10.2337/diabetes.53.5.1253
Banks WA, 2010, P NATL ACAD SCI USA, V107, P22272, DOI 10.1073/pnas.1016369107
Barberger-Gateau P, 2011, CURR ALZHEIMER RES, V8, P479, DOI 10.2174/156720511796391926
Barcia AM, 2005, CLIN INFECT DIS, V41, pS498, DOI 10.1086/432005
Bartke A, 2008, CLIN INTERV AGING, V3, P659, DOI 10.2147/CIA.S3697
BASKIN DG, 1985, LIFE SCI, V36, P627, DOI 10.1016/0024-3205(85)90166-3
Bazan NG, 2007, CURR OPIN CLIN NUTR, V10, P136, DOI 10.1097/MCO.0b013e32802b7030
Belayev L, 2009, STROKE, V40, P3121, DOI 10.1161/STROKEAHA.109.555979
Belkhelfa M, 2014, J INTERF CYTOK RES, V34, P839, DOI 10.1089/jir.2013.0085
Benthem L, 2000, AM J PHYSIOL-ENDOC M, V279, pE1286, DOI 10.1152/ajpendo.2000.279.6.E1286
Bentivoglio M, 2006, CHRONOBIOL INT, V23, P437, DOI 10.1080/07420520500545797
Berridge KC, 2009, PHYSIOL BEHAV, V97, P537, DOI 10.1016/j.physbeh.2009.02.044
Berthoud HR, 2011, CURR OPIN NEUROBIOL, V21, P888, DOI 10.1016/j.conb.2011.09.004
Bertram L, 2007, NAT GENET, V39, P17, DOI 10.1038/ng1934
Bhattacharyya S, 2007, BLOOD, V109, P4313, DOI 10.1182/blood-2006-10-048215
Biessels GJ, 2006, LANCET NEUROL, V5, P64, DOI 10.1016/S1474-4422(05)70284-2
Bitar MS, 2000, SURGERY, V127, P687, DOI 10.1067/msy.2000.105869
BJORNTORP P, 1991, DIABETES CARE, V14, P1132, DOI 10.2337/diacare.14.12.1132
Blagosklonny MV, 2013, AGING-US, V5, P227, DOI 10.18632/aging.100551
Borovikova LV, 2000, NATURE, V405, P458, DOI 10.1038/35013070
BRAAK H, 1991, ACTA NEUROPATHOL, V82, P239, DOI 10.1007/BF00308809
Brands AMA, 2005, DIABETES CARE, V28, P726, DOI 10.2337/diacare.28.3.726
Brennan FM, 2008, EUR J IMMUNOL, V38, P1106, DOI 10.1002/eji.200737821
Brookmeyer R, 1998, AM J PUBLIC HEALTH, V88, P1337, DOI 10.2105/AJPH.88.9.1337
Brookmeyer R, 2007, ALZHEIMERS DEMENT, V3, P186, DOI 10.1016/j.jalz.2007.04.381
Bruun JM, 2003, EUR J ENDOCRINOL, V148, P535, DOI 10.1530/eje.0.1480535
Burns A, 2009, BMJ-BRIT MED J, V338, DOI 10.1136/bmj.b158
Cao GQ, 2007, CURR ALZHEIMER RES, V4, P179, DOI 10.2174/156720507780362173
Cao L, 2011, CELL METAB, V14, P324, DOI 10.1016/j.cmet.2011.06.020
Capuron L, 2011, PHARMACOL THERAPEUT, V130, P226, DOI 10.1016/j.pharmthera.2011.01.014
Castrillo A, 2004, ANNU REV CELL DEV BI, V20, P455, DOI 10.1146/annurev.cellbio.20.012103.134432
Cederholm T, 2013, ADV NUTR, V4, P672, DOI 10.3945/an.113.004556
Chakravarthy MV, 2004, J APPL PHYSIOL, V96, P3, DOI 10.1152/japplphysiol.00757.2003

Cohen S, 2012, P NATL ACAD SCI USA, V109, P5995, DOI 10.1073/pnas.1118355109
Considine RV, 1996, NEW ENGL J MED, V334, P292, DOI 10.1056/NEJM199602013340503
Cordido F, 2010, MEDIAT INFLAMM, V2010, DOI 10.1155/2010/434562
Cotman CW, 2005, NEUROCHEM RES, V30, P877, DOI 10.1007/s11064-005-6960-y
Craft S, 2005, NEUROBIOL AGING, V26, pS65, DOI 10.1016/j.neurobiolaging.2005.08.021
CRAIG BW, 1989, MECH AGEING DEV, V49, P159, DOI 10.1016/0047-6374(89)90099-7
CRAS P, 1991, P NATL ACAD SCI USA, V88, P7552, DOI 10.1073/pnas.88.17.7552
Cutuli D, 2014, FRONT AGING NEUROSCI, V6, DOI 10.3389/fnagi.2014.00220
Dallman MF, 2010, TRENDS ENDOCRIN MET, V21, P159, DOI 10.1016/j.tem.2009.10.004
DALLMAN MF, 1993, FRONT NEUROENDOCRIN, V14, P303, DOI 10.1006/frne.1993.1010
Dandona P, 2004, TRENDS IMMUNOL, V25, P4, DOI 10.1016/j.it.2003.10.013
Davidson TL, 2012, PHYSIOL BEHAV, V107, P26, DOI 10.1016/j.physbeh.2012.05.015
Davis JF, 2010, TRENDS ENDOCRIN MET, V21, P68, DOI 10.1016/j.tem.2009.08.004
Davis LM, 2008, J NEUROSCI RES, V86, P1812, DOI 10.1002/jnr.21628
de Jonge L, 2010, OBESITY, V18, P414, DOI 10.1038/oby.2009.408
De Souza CT, 2005, ENDOCRINOLOGY, V146, P4192, DOI 10.1210/en.2004-1520
DEKOSKY ST, 1990, ANN NEUROL, V27, P457, DOI 10.1002/ana.410270502
DErcole AJ, 1996, ENDOCRIN METAB CLIN, V25, P573, DOI 10.1016/S0889-8529(05)70341-8
Dhingra S, 2009, CARDIOVASC RES, V82, P59, DOI 10.1093/cvr/cvp040
Dong HX, 2009, J ALZHEIMERS DIS, V18, P459, DOI 10.3233/JAD-2009-1152
DOURNAUD P, 1995, NEUROBIOL AGING, V16, P817, DOI 10.1016/0197-4580(95)00086-T
DOYLE P, 1993, AM J PHYSIOL, V264, pE29, DOI 10.1152/ajpendo.1993.264.1.E29
DUBEY AK, 1988, J CLIN ENDOCR METAB, V67, P1064, DOI 10.1210/jcem-67-5-1064
Dumery L, 2001, PATHOL BIOL, V49, P72, DOI 10.1016/S0369-8114(00)00009-2
Dunaiski V, 2002, GROWTH HORM IGF RES, V12, P381, DOI 10.1016/S1096-6374(02)00080-1
Eikelenboom P, 2006, J NEURAL TRANSM, V113, P1685, DOI 10.1007/s00702-006-0575-6
Eikelenboom P, 2010, NEURODEGENER DIS, V7, P38, DOI 10.1159/000283480
Emmerzaal TL, 2015, J ALZHEIMERS DIS, V43, P739, DOI 10.3233/JAD-141086
Enhamre-Brolin E, 2013, BEHAV BRAIN RES, V238, P273, DOI 10.1016/j.bbr.2012.10.036
Eskelinen MH, 2008, INT J GERIATR PSYCH, V23, P741, DOI 10.1002/gps.1969
Esteve E, 2005, CLIN NUTR, V24, P16, DOI 10.1016/j.clnu.2004.08.004
Fain JN, 2006, VITAM HORM, V74, P443, DOI 10.1016/S0083-6729(06)74018-3

Familian A, 2006, GLIA, V53, P233, DOI 10.1002/glia.20268
Farooqi IS, 2007, SCIENCE, V317, P1355, DOI 10.1126/science.1144599
FEINGOLD KR, 1990, AM J PHYSIOL, V259, pE177, DOI 10.1152/ajpendo.1990.259.2.E177
Fischer R, 2011, PLOS ONE, V6, DOI 10.1371/journal.pone.0027621
Fitzpatrick S, 2013, NEUROPSYCHOL REV, V23, P138, DOI 10.1007/s11065-013-9224-7
Folli F, 1996, MOL NEUROBIOL, V13, P155, DOI 10.1007/BF02740639
Freeman LR, 2014, NUTR NEUROSCI, V17, P241, DOI 10.1179/1476830513Y.0000000092
Freeman LR, 2013, FREE RADICAL BIO MED, V56, P226, DOI 10.1016/j.freeradbiomed.2012.08.577
Fuente-Martín E, 2012, J CLIN INVEST, V122, P3900, DOI 10.1172/JCI64102
Gao Q, 2008, FEBS LETT, V582, P132, DOI 10.1016/j.febslet.2007.11.063
GARCIAARAGON J, 1992, DEVELOPMENT, V114, P869
Gasior M, 2006, BEHAV PHARMACOL, V17, P431, DOI 10.1097/00008877-200609000-00009
Gavillet M, 2008, GLIA, V56, P975, DOI 10.1002/glia.20671
Ghanim H, 2012, J CLIN ENDOCR METAB, V97, pE1197, DOI 10.1210/jc.2011-3284
Giordano CR, 2014, SCI REP-UK, V4, DOI 10.1038/srep04983
Godfrey RJ, 2003, SPORTS MED, V33, P599, DOI 10.2165/00007256-200333080-00005
Goossens GH, 2012, CURR OPIN CLIN NUTR, V15, P539, DOI 10.1097/MCO.0b013e328358fa87
Grant RW, 2015, OBESITY, V23, P512, DOI 10.1002/oby.21003
Grassi G, 2005, HYPERTENSION, V46, P321, DOI 10.1161/01.HYP.0000174243.39897.6c
Grillo CA, 2003, NEUROSCIENCE, V121, P133, DOI 10.1016/S0306-4522(03)00343-9
Gupta S, 2005, CELL DEATH DIFFER, V12, P177, DOI 10.1038/sj.cdd.4401557
Gustafson B, 2010, J ATHEROSCLER THROMB, V17, P332, DOI 10.5551/jat.3939
Haan MN, 2006, NAT CLIN PRACT NEURO, V2, P159, DOI 10.1038/ncpneuro0124
Hanci M, 1994, J Neurosurg Sci, V38, P43
Hanzel CE, 2014, NEUROBIOL AGING, V35, P2249, DOI 10.1016/j.neurobiolaging.2014.03.026
Harris HW, 2000, J ENDOTOXIN RES, V6, P421, DOI 10.1179/096805100101532351
Hattiangady B, 2005, EXP NEUROL, V195, P353, DOI 10.1016/j.expneurol.2005.05.014
He P, 2007, J CELL BIOL, V178, P829, DOI 10.1083/jcb.200705042
HO KKY, 1993, HORM RES, V40, P80, DOI 10.1159/000183771
Ho L, 2004, FASEB J, V18, P902, DOI 10.1096/fj.03-0978fje
Hoebel BG, 2009, J ADDICT MED, V3, P33, DOI 10.1097/ADM.0b013e31819aa621
Hojlund K, 2006, DIABETOLOGIA, V49, P1283, DOI 10.1007/s00125-006-0240-5
HOTAMISLIGIL GS, 1995, J CLIN INVEST, V95, P2409, DOI 10.1172/JCI117936

Hut RA, 2011, BEHAV BRAIN RES, V221, P466, DOI 10.1016/j.bbr.2010.11.039
Inoue J, 2000, EXP CELL RES, V254, P14, DOI 10.1006/excr.1999.4733
Jaeger LB, 2009, BRAIN BEHAV IMMUN, V23, P507, DOI 10.1016/j.bbi.2009.01.017
James MJ, 2000, AM J CLIN NUTR, V71, p343S, DOI 10.1093/ajcn/71.1.343s
Jang IS, 2003, BRIT J NUTR, V89, P429, DOI 10.1079/BJN2002801
Janson J, 2004, DIABETES, V53, P474, DOI 10.2337/diabetes.53.2.474
Jayaraman A, 2014, J NEUROINFLAMM, V11, DOI 10.1186/s12974-014-0162-y
Jiang Q, 2008, NEURON, V58, P681, DOI 10.1016/j.neuron.2008.04.010
Jiao JJ, 2014, AM J CLIN NUTR, V100, P1422, DOI 10.3945/ajcn.114.095315
Julio-Amilpas A, 2015, J CEREBR BLOOD F MET, V35, P851, DOI 10.1038/jcbfm.2015.1
Kaiyala KJ, 2000, DIABETES, V49, P1525, DOI 10.2337/diabetes.49.9.1525
Kalmijn S, 1997, AM J EPIDEMIOL, V145, P33, DOI 10.1093/oxfordjournals.aje.a009029
Kanaley JA, 2008, CURR OPIN CLIN NUTR, V11, P50, DOI 10.1097/MCO.0b013e3282f2b0ad
KENNEDY GC, 1953, PROC R SOC SER B-BIO, V140, P578, DOI 10.1098/rspb.1953.0009
Kim B, 2009, ENDOCRINOLOGY, V150, P5294, DOI 10.1210/en.2009-0695
Kivipelto M, 2001, BMJ-BRIT MED J, V322, P1447, DOI 10.1136/bmj.322.7300.1447
Kmiec Z, 2001, ADV ANAT EMBRYOL CEL, V161, P1
Koekkoek Paula S, 2014, Handb Clin Neurol, V126, P145, DOI 10.1016/B978-0-444-53480-4.00011-4
Kohman RA, 2012, BRAIN BEHAV IMMUN, V26, P803, DOI 10.1016/j.bbi.2011.10.006
Kontermann RE, 2008, J IMMUNOTHER, V31, P225, DOI 10.1097/CJI.0b013e31816a88f9
Kooistra M, 2013, J NEUROL SCI, V332, P69, DOI 10.1016/j.jns.2013.06.019
Koolhaas JM, 2011, NEUROSCI BIOBEHAV R, V35, P1291, DOI 10.1016/j.neubiorev.2011.02.003
Kranzler JH, 1998, J CLIN ENDOCR METAB, V83, P1953, DOI 10.1210/jc.83.6.1953
Krstic D, 2013, NAT REV NEUROL, V9, P25, DOI 10.1038/nrneurol.2012.236
Kudlow PA, 2013, SLEEP MED, V14, P943, DOI 10.1016/j.sleep.2013.04.017
Labrousse VF, 2012, PLOS ONE, V7, DOI 10.1371/journal.pone.0036861
Laitinen MH, 2006, DEMENT GERIATR COGN, V22, P99, DOI 10.1159/000093478
Landry GJ, 2014, FRONT AGING NEUROSCI, V6, DOI 10.3389/fnagi.2014.00325
Leise TL, 2013, AGE, V35, P2137, DOI 10.1007/s11357-012-9502-y
Levin B E, 1993, Obes Res, V1, P281
Li RC, 2011, NEUROSCIENCE, V196, P237, DOI 10.1016/j.neuroscience.2011.08.029
Lionetti L, 2009, NUTR METAB CARDIOVAS, V19, P146, DOI 10.1016/j.numecd.2008.10.010
Liu CC, 2013, NAT REV NEUROL, V9, P106, DOI 10.1038/nrneurol.2012.263
Liu Y, 2009, J NEUROCHEM, V111, P242, DOI 10.1111/j.1471-4159.2009.06320.x

LOBIE PE, 1993, DEV BRAIN RES, V74, P225, DOI 10.1016/0165-3806(93)90008-X
Lu T, 2004, NATURE, V429, P883, DOI 10.1038/nature02661
LUITEN PGM, 1985, BRAIN RES, V329, P374, DOI 10.1016/0006-8993(85)90554-2
Lumeng CN, 2011, J CLIN INVEST, V121, P2111, DOI 10.1172/JCI57132
Magistretti PJ, 1999, PHILOS T R SOC B, V354, P1155, DOI 10.1098/rstb.1999.0471
Makimura H, 2008, J CLIN ENDOCR METAB, V93, P4254, DOI 10.1210/jc.2008-1333
Makki Kassem, 2013, ISRN Inflamm, V2013, P139239, DOI 10.1155/2013/139239
Malone JI, 2008, PEDIATR DIABETES, V9, P531, DOI 10.1111/j.1399-5448.2008.00431.x
Marcell TJ, 1999, METABOLISM, V48, P665, DOI 10.1016/S0026-0495(99)90069-0
Marchetti L, 2004, J BIOL CHEM, V279, P32869, DOI 10.1074/jbc.M311766200
Marino JS, 2011, TRENDS ENDOCRIN MET, V22, P275, DOI 10.1016/j.tem.2011.03.001
Marosi K, 2012, NEUROSCIENCE, V226, P21, DOI 10.1016/j.neuroscience.2012.09.001
Martin ASG, 2006, AM J CLIN NUTR, V83, p461S, DOI 10.1093/ajcn/83.2.461s
Mattson MP, 2006, NAT REV NEUROSCI, V7, P278, DOI 10.1038/nrn1886
McAllister EJ, 2009, CRIT REV FOOD SCI, V49, P868, DOI 10.1080/10408390903372599
Mccoy MK, 2008, J NEUROINFLAMM, V5, DOI 10.1186/1742-2094-5-45
McEwen BS, 2003, HORM BEHAV, V43, P2, DOI 10.1016/S0018-506X(02)00024-7
McEwen BS, 2004, EUR J PHARMACOL, V490, P13, DOI 10.1016/j.ejphar.2004.02.041
Moller DE, 2000, TRENDS ENDOCRIN MET, V11, P212, DOI 10.1016/S1043-2760(00)00272-1
Morris MC, 2004, NEUROLOGY, V62, P1573, DOI 10.1212/01.WNL.0000123250.82849.B6
Mulder CK, 2013, FRONT MOL NEUROSCI, V6, DOI 10.3389/fnmol.2013.00008
Nathan C, 2002, NATURE, V420, P846, DOI 10.1038/nature01320
Naudé PJW, 2011, FEBS J, V278, P888, DOI 10.1111/j.1742-4658.2011.08017.x
Neumann H, 2009, BRAIN, V132, P288, DOI 10.1093/brain/awn109
Norman GJ, 2010, MOL PSYCHIATR, V15, P404, DOI 10.1038/mp.2009.91
O' Neill C, 2013, EXP GERONTOL, V48, P647, DOI 10.1016/j.exger.2013.02.025
Ogawa S, 2005, CELL, V122, P707, DOI 10.1016/j.cell.2005.06.029
Ohta H, 2005, ATHEROSCLEROSIS, V180, P11, DOI 10.1016/j.atherosclerosis.2004.11.016
Orr SK, 2013, PROSTAG LEUKOTR ESS, V88, P97, DOI 10.1016/j.plefa.2012.05.008
OWEN OE, 1967, J CLIN INVEST, V46, P1589, DOI 10.1172/JCI105650
Pan WH, 2011, CURR PHARM DESIGN, V17, P3729, DOI 10.2174/138161211798220918
Park SE, 2011, J NEUROINFLAMM, V8, DOI 10.1186/1742-2094-8-12

Pascualy M, 2000, BIOL PSYCHIAT, V48, P247, DOI 10.1016/S0006-3223(00)00879-9
Patel AB, 2014, P NATL ACAD SCI USA, V111, P5385, DOI 10.1073/pnas.1403576111
Pavlov VA, 2012, NAT REV ENDOCRINOL, V8, P743, DOI 10.1038/nrendo.2012.189
Permana PA, 2006, BIOCHEM BIOPH RES CO, V341, P507, DOI 10.1016/j.bbrc.2006.01.012
Petersen AMW, 2005, J APPL PHYSIOL, V98, P1154, DOI 10.1152/japplphysiol.00164.2004
Picardi PK, 2010, FEBS LETT, V584, P3179, DOI 10.1016/j.febslet.2010.05.064
Popa C, 2007, J LIPID RES, V48, P751, DOI 10.1194/jlr.R600021-JLR200
Posey KA, 2009, AM J PHYSIOL-ENDOC M, V296, pE1003, DOI 10.1152/ajpendo.90377.2008
Prasad MR, 1998, NEUROCHEM RES, V23, P81, DOI 10.1023/A:1022457605436
Pritzlaff CJ, 2000, J APPL PHYSIOL, V89, P937, DOI 10.1152/jappl.2000.89.3.937
Profenno LA, 2010, BIOL PSYCHIAT, V67, P505, DOI 10.1016/j.biopsych.2009.02.013
Puglielli L, 2003, NAT NEUROSCI, V6, P345, DOI 10.1038/nn0403-345
Querfurth HW, 2010, NEW ENGL J MED, V362, P329, DOI 10.1056/NEJMra0909142
Rajpathak SN, 2009, DIABETES-METAB RES, V25, P3, DOI 10.1002/dmrr.919
Rath PC, 1999, J CLIN IMMUNOL, V19, P350, DOI 10.1023/A:1020546615229
Reader BF, 2015, NEUROSCIENCE, V289, P429, DOI 10.1016/j.neuroscience.2015.01.001
Roenneberg T, 2012, CURR BIOL, V22, P939, DOI 10.1016/j.cub.2012.03.038
Rosenbaum M, 2014, J ENDOCRINOL, V223, pT83, DOI 10.1530/JOE-14-0358
Ruiz-Torres A, 2002, GERONTOLOGY, V48, P401, DOI 10.1159/000065507
Sadki A, 2007, NEUROBIOL AGING, V28, P296, DOI 10.1016/j.neurobiolaging.2005.12.007
Salles J, 2012, J NUTR BIOCHEM, V23, P1685, DOI 10.1016/j.jnutbio.2011.12.001
SantaCruz K, 2005, SCIENCE, V309, P476, DOI 10.1126/science.1113694
Scheepens A, 2001, NEUROSCIENCE, V104, P677, DOI 10.1016/S0306-4522(01)00109-9
Schroeter ML, 2009, NEUROIMAGE, V47, P1196, DOI 10.1016/j.neuroimage.2009.05.037
Schwartz MW, 2000, NATURE, V404, P661, DOI 10.1038/35007534
Seaquist ER, 2001, DIABETES, V50, P2203, DOI 10.2337/diabetes.50.10.2203
Sébert SP, 2005, INT J OBESITY, V29, P324, DOI 10.1038/sj.ijo.0802823
Shytle RD, 2004, J NEUROCHEM, V89, P337, DOI 10.1046/j.1471-4159.2004.02347.x
Signore AP, 2008, J NEUROCHEM, V106, P1977, DOI 10.1111/j.1471-4159.2008.05457.x
Sims-Robinson C, 2010, NAT REV NEUROL, V6, P551, DOI 10.1038/nrneurol.2010.130
Singh KP, 1998, BURNS, V24, P733, DOI 10.1016/S0305-4179(98)00113-2
Skrapari I, 2007, OBESITY, V15, P1685, DOI 10.1038/oby.2007.201
Song EF, 2014, J AM HEART ASSOC, V3, DOI 10.1161/JAHA.114.000837
Soscia SJ, 2010, PLOS ONE, V5, DOI 10.1371/journal.pone.0009505

Speakman John R, 2006, Diab Vasc Dis Res, V3, P7, DOI 10.3132/dvdr.2006.010
Srivareerat M, 2009, BIOL PSYCHIAT, V65, P918, DOI 10.1016/j.biopsych.2008.08.021
Staels B, 2006, NAT MED, V12, P54, DOI 10.1038/nm0106-54
Steen E, 2005, J ALZHEIMERS DIS, V7, P63, DOI 10.3233/jad-2005-7107
Steyn FJ, 2013, J ENDOCRINOL, V218, P233, DOI 10.1530/JOE-13-0084
Stranahan AM, 2008, HIPPOCAMPUS, V18, P1085, DOI 10.1002/hipo.20470
STRITTMATTER WJ, 1993, P NATL ACAD SCI USA, V90, P1977, DOI 10.1073/pnas.90.5.1977
Suh YH, 2002, PHARMACOL REV, V54, P469, DOI 10.1124/pr.54.3.469
Suzuki H, 1998, MECH AGEING DEV, V101, P119, DOI 10.1016/S0047-6374(97)00169-3
Takach O, 2015, NEUROBIOL AGING, V36, P1378, DOI 10.1016/j.neurobiolaging.2014.11.018
Takada Y, 2004, J BIOL CHEM, V279, P39541, DOI 10.1074/jbc.M403449200
Takeuchi O, 2010, CELL, V140, P805, DOI 10.1016/j.cell.2010.01.022
Tanzi RE, 1999, J CLIN INVEST, V104, P1175, DOI 10.1172/JCI8593
Teerds KJ, 2011, HUM REPROD UPDATE, V17, P667, DOI 10.1093/humupd/dmr017
Thaler JP, 2012, J CLIN INVEST, V122, P153, DOI 10.1172/JCI59660
Thaler JP, 2010, ENDOCRINOLOGY, V151, P4109, DOI 10.1210/en.2010-0336
Timmermans S, 2014, J NEUROIMMUNE PHARM, V9, P209, DOI 10.1007/s11481-013-9502-4
Tolppanen AM, 2014, J ALZHEIMERS DIS, V38, P201, DOI 10.3233/JAD-130698
Tracey KJ, 2007, J CLIN INVEST, V117, P289, DOI 10.1172/JCI30555
Trejo JI, 2007, MOL PSYCHIATR, V12, P1118, DOI 10.1038/sj.mp.4002076
Trejo JL, 2004, J MOL MED, V82, P156, DOI 10.1007/s00109-003-0499-7
Trejo JL, 2002, REV NEUROSCIENCE, V13, P365
Tsigos C, 1999, METABOLISM, V48, P1332, DOI 10.1016/S0026-0495(99)90277-9
Tulipano G, 2007, PITUITARY, V10, P267, DOI 10.1007/s11102-007-0054-6
Unger RH, 2010, TRENDS ENDOCRIN MET, V21, P345, DOI 10.1016/j.tem.2010.01.009
Urayama A, 2008, ENDOCRINOLOGY, V149, P3592, DOI 10.1210/en.2008-0008
Vahl N, 1997, AM J PHYSIOL-ENDOC M, V272, pE1108, DOI 10.1152/ajpendo.1997.272.6.E1108
Vainik U, 2013, NEUROSCI BIOBEHAV R, V37, P279, DOI 10.1016/j.neubiorev.2012.11.008
Van der Zee EA, 1999, BRAIN RES, V816, P572, DOI 10.1016/S0006-8993(98)01239-6
Vaz M, 1997, CIRCULATION, V96, P3423
Veldhuis JD, 1997, J CLIN ENDOCR METAB, V82, P3414, DOI 10.1210/jc.82.10.3414
Vonck K, 2014, NEUROSCI BIOBEHAV R, V45, P63, DOI 10.1016/j.neubiorev.2014.05.005
Vykoukal D, 2011, J VASC SURG, V54, P819, DOI 10.1016/j.jvs.2011.01.003
Wajant H, 2003, CELL DEATH DIFFER, V10, P45, DOI 10.1038/sj.cdd.4401189
Wang BH, 2006, PFLUG ARCH EUR J PHY, V452, P418, DOI 10.1007/s00424-006-0055-8
Wang H, 2003, NATURE, V421, P384, DOI 10.1038/nature01339

Wang YY, 2011, AM J PHYSIOL-ENDOC M, V300, pE155, DOI 10.1152/ajpendo.00301.2010
Wang Y, 2007, CLIN CHEM, V53, P34, DOI [10.1007/s11461-007-0005-4, 10.1373/clinchem.2006.075614]
Weinert D, 2000, CHRONOBIOL INT, V17, P261, DOI 10.1081/CBI-100101048
Wideman L, 2002, SPORTS MED, V32, P987, DOI 10.2165/00007256-200232150-00003
Winer DA, 2011, NAT MED, V17, P610, DOI 10.1038/nm.2353
Winkler T, 2000, AMINO ACIDS, V19, P363, DOI 10.1007/s007260070067
Winocur G, 2005, BEHAV NEUROSCI, V119, P1389, DOI 10.1037/0735-7044.119.5.1389
Winston BW, 1999, CLIN INVEST MED, V22, P236
WISCHIK CM, 1988, P NATL ACAD SCI USA, V85, P4506, DOI 10.1073/pnas.85.12.4506
Wisor JP, 2011, SLEEP, V34, P261, DOI 10.1093/sleep/34.3.261
Wohleb ES, 2015, FRONT NEUROSCI-SWITZ, V8, DOI 10.3389/fnins.2014.00447
Wohleb ES, 2014, J NEUROSCI, V34, P2583, DOI 10.1523/JNEUROSCI.3723-13.2014
Woo J, 2011, MED CLIN N AM, V95, P477, DOI 10.1016/j.mcna.2011.02.009
Wood IS, 2009, P NUTR SOC, V68, P370, DOI 10.1017/S0029665109990206
Woods JA, 2012, AGING DIS, V3, P130
Woods SC, 2003, J NUTR, V133, P1081, DOI 10.1093/jn/133.4.1081
Xiong XQ, 2012, HYPERTENSION, V60, P1280, DOI 10.1161/HYPERTENSIONAHA.112.198002
Xu WL, 2011, NEUROLOGY, V76, P1568, DOI 10.1212/WNL.0b013e3182190d09
YAMAMOTO H, 1991, ACTA ENDOCRINOL-COP, V124, P497, DOI 10.1530/acta.0.1240497
Yamane K, 2003, J IMMUNOL, V171, P3855, DOI 10.4049/jimmunol.171.7.3855
Yamauchi T, 2001, NAT MED, V7, P941, DOI 10.1038/90984
Yamauchi Toshimasa, 2003, Current Drug Targets - Immune Endocrine and Metabolic Disorders, V3, P243, DOI 10.2174/1568008033340090
Yan H, 2011, J ENDOCRINOL, V211, P27, DOI 10.1530/JOE-11-0200
Ye J, 2009, INT J OBESITY, V33, P54, DOI 10.1038/ijo.2008.229
Yehuda S, 2012, MED HYPOTHESES, V79, P456, DOI 10.1016/j.mehy.2012.06.021
Yi CX, 2012, PHYSIOL BEHAV, V106, P485, DOI 10.1016/j.physbeh.2012.03.021
Zeltser LM, 2012, NAT NEUROSCI, V15, P1336, DOI 10.1038/nn.3219
Zendedel A, 2015, J NEUROIMMUNOL, V278, P200, DOI 10.1016/j.jneuroim.2014.11.007
Zettlitz KA, 2010, MABS-AUSTIN, V2, P639, DOI 10.4161/mabs.2.6.13583
Zhang H, 2014, PLOS ONE, V9, DOI 10.1371/journal.pone.0108518
ZHANG YY, 1994, NATURE, V372, P425, DOI 10.1038/372425a0
Zhao E, 2014, SPORTS HEALTH, V6, P63, DOI 10.1177/1941738113477832
NR 279
TC 57
Z9 61
U1 0
U2 26
PU FRONTIERS MEDIA SA
PI LAUSANNE
PA AVENUE DU TRIBUNAL FEDERAL 34, LAUSANNE, CH-1015, SWITZERLAND

EI 1662-453X
J9 FRONT NEUROSCI-SWITZ
JI Front. Neurosci.
PD MAY 18
PY 2015
VL 9
AR 173
DI 10.3389/fnins.2015.00173
PG 19
WC Neurosciences
WE Science Citation Index Expanded (SCI-EXPANDED)
SC Neurosciences & Neurology
GA CS3NO
UT WOS:000361981400001
PM 26041981
OA Green Published, gold
DA 2025-05-07
ER

PT S
AU Swynghedauw, B
   Delcayre, C
   Samuel, JL
   Mebazaa, A
   Cohen-Solal, A
AF Swynghedauw, Bernard
   Delcayre, Claude
   Samuel, Jane-Lyse
   Mebazaa, Alexandre
   Cohen-Solal, Alain
BE Beyar, R
   Landesberg, A
TI Molecular mechanisms in evolutionary cardiology failure
SO ANALYSIS OF CARDIAC DEVELOPMENT: FROM EMBRYO TO OLD AGE
SE Annals of the New York Academy of Sciences
LA English
DT Article; Proceedings Paper
CT 6th Larry and Horti Fairberg Workshop on Analysis of Cardiac
   Development, From Embryo to Old Age
CY MAR 30-APR 01, 2009
CL Tech-Israel Inst Technol, Haifa, ISRAEL
SP Rambam Med Ctr
HO Tech-Israel Inst Technol
DE heart failure; cardiac hypertrophy; microRNA; fibrosis; fetal
   hypothesis; wall stress hypothesis; Laplace's law; aldosterone;
   evolution; Darwinian or evolutionary medicine
ID GENE-EXPRESSION; CARDIAC-HYPERTROPHY; HEART-FAILURE; PRESSURE-
OVERLOAD;
   PHENOTYPIC PLASTICITY; ALDOSTERONE; RAT; CARDIOMYOPATHY;
TRANSCRIPTOME;
   MICROARRAY
AB Integration of the relevant evolutionary paradigm in cardiology has
not yet been fully achieved: In the past, heart failure (HF) was mainly
ascribed to infections, and the origins of cardiac hypertrophy (CH) were

regarded as mechanical. Recent changes in lifestyle have both reduced the incidence of infections and increased lifespan, and H F is now seen as a complex disease one that is still caused by mechanical disorder, but also associated with ischemia and senescence. The long-held view that CH serves to restore myocardial economy back to normal is still valid. The adaptive process is characterized by a quantitative and a qualitative fetal gene reprogramming, which is now being confirmed by recent advances in microRNA research. It underscores the fact CH is the physiologic reaction of the heart to a pathologic stimulus. The goal for therapy is economic, not inotropic. Another major issue is myocardial fibrosis, a major determinant of diastolic function and arrhythmias. Recent changes in lifestyle have crucially modified the context in which HF occurs.
C1 [Swynghedauw, Bernard; Cohen-Solal, Alain] Hop Lariboisiere, INSERM, U942, Dept Cardiol, F-75475 Paris 10, France.
[Mebazaa, Alexandre] Hop Lariboisiere, Dept Anesthesiol, F-75475 Paris, France.
C3 Institut National de la Sante et de la Recherche Medicale (Inserm); Assistance Publique Hopitaux Paris (APHP); Universite Paris Cite; Hopital Universitaire Lariboisiere-Fernand-Widal - APHP; Universite Paris Cite; Assistance Publique Hopitaux Paris (APHP); Hopital Universitaire Lariboisiere-Fernand-Widal - APHP
RP Swynghedauw, B (autor correspondente), Hop Lariboisiere, INSERM, U942, Dept Cardiol, F-75475 Paris 10, France.
EM Bernard.Swynghedauw@inserm.fr
RI Deniau, Benjamin/AAU-7314-2021
OI Samuel, Jane-Lise/0000-0002-7346-2404; Mebazaa, Alexandre/0000-0001-8715-7753
CR Akazawa H, 2003, CIRC RES, V92, P1079, DOI 10.1161/01.RES.0000072977.86706.23
Alpert N.R., 1992, HEART CARDIOVASCULAR, P111
[Anonymous], TIMES BOOKS
Assayag P, 1998, CARDIOVASC RES, V38, P169, DOI 10.1016/S0008-6363(97)00283-6
Barrans JD, 2002, AM J PATHOL, V160, P2035, DOI 10.1016/S0002-9440(10)61153-4
Boheler KR, 2003, P NATL ACAD SCI USA, V100, P2754, DOI 10.1073/pnas.0436564100
Brancaccio M, 2003, NAT MED, V9, P68, DOI 10.1038/nm805
Buckberg G D, 2001, Semin Thorac Cardiovasc Surg, V13, P358
Causton HC, 2001, MOL BIOL CELL, V12, P323, DOI 10.1091/mbc.12.2.323
Depre C, 1998, NAT MED, V4, P1269, DOI 10.1038/3253
DeWitt T.J., 2004, Phenotypic Plasticity: Functional and Conceptual Approaches
Doughty Paul, 2004, P126
Esposito G, 2002, CIRCULATION, V105, P85, DOI 10.1161/hc0102.101365
Gao Z, 2006, J MOL CELL CARDIOL, V40, P76, DOI 10.1016/j.yjmcc.2005.08.002
Gerstein MB, 2007, GENOME RES, V17, P669, DOI 10.1101/gr.6339607
Giampietri C, 2008, J HYPERTENS, V26, P1008, DOI 10.1097/HJH.0b013e3282f6a179
Gibson G., 2002, PRIMER GENOME SCI
IHLVAHL R, 1995, J MOL CELL CARDIOL, V27, P437, DOI 10.1016/S0022-2828(08)80040-5

Kittleson MM, 2005, PHYSIOL GENOMICS, V21, P299, DOI 10.1152/physiolgenomics.00255.2004
Kodde IF, 2007, COMP BIOCHEM PHYS A, V146, P26, DOI 10.1016/j.cbpa.2006.09.014
LaFramboise WA, 2005, J MOL CELL CARDIOL, V38, P103, DOI 10.1016/j.yjmcc.2004.09.011
Léger J, 2006, ARCH MAL COEUR VAISS, V99, P231
LOMPRE AM, 1979, NATURE, V282, P105, DOI 10.1038/282105a0
Lynch M, 2007, P NATL ACAD SCI USA, V104, P8597, DOI 10.1073/pnas.0702207104
Meeson AP, 2001, CIRC RES, V88, P713, DOI 10.1161/hh0701.089753
Molkentin JD, 1998, CELL, V93, P215, DOI 10.1016/S0092-8674(00)81573-1
Nanni L, 2006, J MOL CELL CARDIOL, V41, P934, DOI 10.1016/j.yjmcc.2006.08.009
Price TD, 2003, P ROY SOC B-BIOL SCI, V270, P1433, DOI 10.1098/rspb.2003.2372
ROBERT V, 1995, HYPERTENSION, V26, P971, DOI 10.1161/01.HYP.26.6.971
Ruiz P, 2006, J MOL CELL CARDIOL, V40, P13, DOI 10.1016/j.yjmcc.2005.09.010
Samuel JL, 2008, CARDIOVASC RES, V79, P218, DOI 10.1093/cvr/cvn061
Samuel JL, 2008, J HYPERTENS, V26, P857, DOI 10.1097/HJH.0b013e3282fbf619
Sanoudou D, 2005, PHYSIOL GENOMICS, V21, P131, DOI 10.1152/physiolgenomics.00259.2004
Schaub MC, 2006, J MOL CELL CARDIOL, V41, P183, DOI 10.1016/j.yjmcc.2006.04.005
Silvestre JS, 1999, CIRCULATION, V99, P2694, DOI 10.1161/01.CIR.99.20.2694
Silvestre JS, 1998, J BIOL CHEM, V273, P4883, DOI 10.1074/jbc.273.9.4883
Sotiriou C, 2009, NEW ENGL J MED, V360, P790, DOI 10.1056/NEJMra0801289
Steenman M, 2005, CLIN CHEM LAB MED, V43, P696, DOI 10.1515/CCLM.2005.118
Swynghedauw B, 1999, PHYSIOL REV, V79, P215, DOI 10.1152/physrev.1999.79.1.215
Swynghedauw B, 2000, CURR OPIN CARDIOL, V15, P247, DOI 10.1097/00001573-200007000-00007
SWYNGHEDAUW B, 1986, PHYSIOL REV, V66, P710, DOI 10.1152/physrev.1986.66.3.710
Swynghedauw B, 2006, J EXP BIOL, V209, P2320, DOI 10.1242/jeb.02084
Thum T, 2007, CIRCULATION, V116, P258, DOI 10.1161/CIRCULATIONAHA.107.687947
Thum T, 2008, NATURE, V456, P980, DOI 10.1038/nature07511
NR 44
TC 21
Z9 24
U1 0
U2 6
PU BLACKWELL PUBLISHING
PI OXFORD
PA 9600 GARSINGTON RD, OXFORD OX4 2DQ, OXEN, ENGLAND
SN 0077-8923
BN 978-1-57331-747-4

J9 ANN NY ACAD SCI
JI Ann.NY Acad.Sci.
PY 2010
VL 1188
BP 58
EP 67
DI 10.1111/j.1749-6632.2009.05084.x
PG 10
WC Multidisciplinary Sciences
WE Conference Proceedings Citation Index - Science (CPCI-S); Science Citation Index Expanded (SCI-EXPANDED)
SC Science & Technology - Other Topics
GA BOV10
UT WOS:000277731600009
PM 20201887
DA 2025-05-07
ER

PT J
AU do Amaral-Silva, L
Gargaglioni, LH
Steiner, AA
Oliveira, MT
Bícego, KC
AF do Amaral-Silva, Lara
Gargaglioni, Luciane H.
Steiner, Alexandre A.
Oliveira, Marcos T.
Bicego, Kenia Cardoso
TI Regulated hypothermia in response to endotoxin in birds
SO JOURNAL OF PHYSIOLOGY-LONDON
LA English
DT Article
DE anapyrexia; birds; hypothermia; inflammation; metabolic rate; peripheral
vasodilatation; tachypnea
ID LPS-INDUCED HYPOTHERMIA; SKELETAL-MUSCLE; BACTERIAL LIPOPOLYSACCHARIDE;
THERMOREGULATORY RESPONSES; GALLUS-DOMESTICUS; BAROMETRIC METHOD;
TRADE-OFFS; FEVER; TEMPERATURE; THERMOGENESIS
AB Key points
The costs associated with immune and thermal responses may exceed the benefits to the host during severe inflammation. In this case, regulated hypothermia instead of fever can occur in rodents as a beneficial strategy to conserve energy for vital functions with consequent tissue protection and hypoxia prevention.
We tested the hypothesis that this phenomenon is not exclusive to mammals, but extends to the other endothermic group, birds.
A decrease in metabolic rate without any failure in mitochondrial respiration, nor oxygen delivery, is the main evidence supporting the regulated nature of endotoxin-induced hypothermia in chicks.
Thermolytic mechanisms such as tachypnea and cutaneous vasodilatation can also be recruited to facilitate body temperature decrease under lipopolysaccharide treatment, especially in the cold.

Our findings bring a new perspective for evolutionary medicine studies on energy trade-off in host defence because regulated hypothermia may be a phenomenon spread among vertebrates facing a severe immune challenge.
A switch from fever to regulated hypothermia can occur in mammals under circumstances of reduced physiological fitness (e.g. sepsis) to direct energy to defend vital systems. Birds in which the cost to resist a pathogen is additive to the highest metabolic rate and body temperature (T-b) among vertebrates may also benefit from regulated hypothermia during systemic inflammation. Here, we show that the decrease in T-b observed during an immune challenge in birds is a regulated hypothermia, and not a result of metabolic failure. We investigated O-2 consumption (thermogenesis index), ventilation (respiratory heat loss), skin temperature (sensible heat loss) and muscle mitochondrial respiration (thermogenic tissue) during T-b fall in chicken chicks challenged with endotoxin [lipopolysaccharide (LPS)]. Chicks injected with LPS were also tested regarding the capacity to raise O-2 consumption to meet an increased demand driven by 2,4-dinitrophenol. LPS decreased T-b and the metabolic rate of chicks without affecting muscle uncoupled, coupled and non-coupled mitochondrial respiration. LPS-challenged chicks were indeed capable of increasing metabolic rate in response to 2,4-dinitrophenol, indicating no O-2 delivery limitation. Additionally, chicks did not attempt to prevent T-b from falling during hypothermia but, instead, activated cutaneous and respiratory thermolytic mechanisms, providing an additional cooling force. These data provide the first evidence of the regulated nature of the hypothermic response to endotoxin in birds. Therefore, it changes the current understanding of bird's thermoregulation during severe inflammation, indicating that regulated hypothermia is either a convergent trait for endotherms or a conserved response among vertebrates, which adds a new perspective for evolutionary medicine research.
C1 [do Amaral-Silva, Lara; Gargaglioni, Luciane H.; Bicego, Kenia Cardoso] Sao Paulo State Univ, Dept Anim Morphol & Physiol, 321 McIver St,312 Eberhart Bldg,Greensboro 27412, Jaboticabal, SP, Brazil.
[Oliveira, Marcos T.] Sao Paulo State Univ, Dept Technol, Jaboticabal, SP, Brazil.
[Steiner, Alexandre A.] Univ Sao Paulo, Inst Biomed Sci, Dept Immunol, Sao Paulo, SP, Brazil.
C3 Universidade Estadual Paulista; Universidade Estadual Paulista; Universidade de Sao Paulo; Institute Biomed Science, University Sao Paulo
RP do Amaral-Silva, L (autor correspondente), Sao Paulo State Univ, Dept Anim Morphol & Physiol, 321 McIver St,312 Eberhart Bldg,Greensboro 27412, Jaboticabal, SP, Brazil.
EM lara.amaral-silva@unesp.br
RI Gargaglioni, Luciane/F-9922-2012; Steiner, Alexandre/G-1288-2012; Oliveira, Marcos Tulio/B-3249-2014; Bicego, Kenia/G-6184-2012
OI Gargaglioni, Luciane/0000-0003-4097-5286; Steiner, Alexandre/0000-0002-0802-4619; Oliveira, Marcos Tulio/0000-0003-4779-5755; Bicego, Kenia/0000-0002-1180-1132; Silva, Lara/0000-0001-6273-3240
FU Sao Paulo Research Foundation (FAPESP) [2017/12 627-9, 2014/0 2253-6, 2018/0 3418-0]; FAPESP [2017/00864-6]; Formas [2017-00864] Funding Source: Formas; Fundacao de Amparo a Pesquisa do Estado de Sao Paulo (FAPESP) [17/00864-6] Funding Source: FAPESP

FX This study was funded by Sao Paulo Research Foundation (FAPESP; 2017/12
627-9 to KCB; 2014/0 2253-6 to MTO; 2018/0 3418-0 to AAS). LAS was the
recipient of a FAPESP PhD fellowship (2017/00864-6). This study was part
of the activities developed by LAS during her PhD at the Animal Science
Graduate Program, FCAV-UNESP, Jaboticabal, SP, Brazil.
CR Adelman JS, 2010, J EXP BIOL, V213, P4099, DOI 10.1242/jeb.049528
Al-Saffar H, 2013, BRAIN BEHAV IMMUN, V28, P188, DOI 10.1016/j.bbi.2012.11.015
Almeida MC, 2006, EUR J NEUROSCI, V23, P3359, DOI 10.1111/j.1460-9568.2006.04854.x
Amaral-Silva L, 2020, ENVIRON TOXICOL CHEM, V39, P2208, DOI 10.1002/etc.4832
ARAD Z, 1982, COMP BIOCHEM PHYS A, V72, P179, DOI 10.1016/0300-9629(82)90029-9
Bicego KC, 2007, COMP BIOCHEM PHYS A, V147, P616, DOI 10.1016/j.cbpa.2006.06.032
Bícego KC, 2017, J EXP BIOL, V220, P4634, DOI 10.1242/jeb.171702
Bicudo JEPW, 2002, J EXP BIOL, V205, P2267
Blatteis CM, 2003, J THERM BIOL, V28, P1, DOI 10.1016/S0306-4565(02)00034-7
Brealey David, 2002, Lancet (North American Edition), V360, P219, DOI 10.1016/S0140-6736(02)09459-X
Burness G, 2010, J EXP BIOL, V213, P1386, DOI 10.1242/jeb.027011
Butler PJ, 2004, RESP PHYSIOL NEUROBI, V141, P297, DOI 10.1016/j.resp.2004.01.010
Cannon B, 2004, PHYSIOL REV, V84, P277, DOI 10.1152/physrev.00015.2003
Clarke A, 2010, BIOL REV, V85, P703, DOI 10.1111/j.1469-185X.2010.00122.x
Cornelius EA, 2017, PHYSIOL BIOCHEM ZOOL, V90, P190, DOI 10.1086/689913
Corrigan JJ, 2014, J PHYSIOL-LONDON, V592, P3901, DOI 10.1113/jphysiol.2014.277277
Dantonio V, 2016, AM J PHYSIOL-REG I, V310, pR896, DOI 10.1152/ajpregu.00453.2015
do Amaral-Silva L, 2017, COMP BIOCHEM PHYS A, V207, P93, DOI 10.1016/j.cbpa.2017.03.002
DRORBAUGH JE, 1955, PEDIATRICS, V16, P81
DUCHAMP C, 1993, AM J PHYSIOL, V265, pR1076, DOI 10.1152/ajpregu.1993.265.5.R1076
Evans JK, 2017, HORM BEHAV, V88, P112, DOI 10.1016/j.yhbeh.2016.12.003
Evans SS, 2015, NAT REV IMMUNOL, V15, P335, DOI 10.1038/nri3843
Fonseca MT, 2016, J APPL PHYSIOL, V120, P1394, DOI 10.1152/japplphysiol.00004.2016
Ganeshan K, 2019, CELL, V177, P399, DOI 10.1016/j.cell.2019.01.050
Garami A, 2018, HAND CLINIC, V157, P565, DOI 10.1016/B978-0-444-64074-1.00034-3
GLEESON M, 1986, COMP BIOCHEM PHYS A, V83, P283, DOI 10.1016/0300-9629(86)90575-X
Gray DA, 2013, J COMP PHYSIOL B, V183, P297, DOI 10.1007/s00360-012-0718-z

HARRIS RA, 1971, ARCH BIOCHEM BIOPHYS, V142, P435, DOI 10.1016/0003-9861(71)90507-8
HILLMAN PE, 1982, AM J PHYSIOL, V242, pR582, DOI 10.1152/ajpregu.1982.242.5.R582
Hohtola Esa, 2012, P155
Iups T., 2001, Jap. J. Physiol., V51, P280
King MO, 2013, J EXP BIOL, V216, P2097, DOI 10.1242/jeb.079574
Kluger MJ, 1996, INFECT DIS CLIN N AM, V10, P1, DOI 10.1016/S0891-5520(05)70282-8
Koteja P, 1996, FUNCT ECOL, V10, P675, DOI 10.2307/2390179
Krall CM, 2010, AM J PHYSIOL-REG I, V298, pR1512, DOI 10.1152/ajpregu.00158.2010
Lee SY, 2008, COMP BIOCHEM PHYS C, V148, P324, DOI 10.1016/j.cbpc.2008.05.009
Legendre LJ, 2020, PHILOS T R SOC B, V375, DOI 10.1098/rstb.2019.0136
Liu E, 2012, AM J PHYSIOL-REG I, V302, pR1372, DOI 10.1152/ajpregu.00023.2012
Lochmiller RL, 2000, OIKOS, V88, P87, DOI 10.1034/j.1600-0706.2000.880110.x
Logan ML, 2019, IDEAS ECOL EVOL, V12, P1, DOI 10.24908/iee.2019.12.1.n
McCafferty DJ, 2013, IBIS, V155, P4, DOI 10.1111/ibi.12010
McKechnie AE, 2002, CONDOR, V104, P705, DOI 10.1650/0010-5422(2002)104[0705:AFHRAR]2.0.CO;2
McKechnie AE, 2016, J EXP BIOL, V219, P2145, DOI 10.1242/jeb.138776
Meir JU, 2019, ELIFE, V8, DOI 10.7554/eLife.44986
Merchant M, 2008, VET IMMUNOL IMMUNOP, V125, P176, DOI 10.1016/j.vetimm.2008.04.014
Mortola JP, 2011, COMPR PHYSIOL, V1, P1679, DOI 10.1002/cphy.c100008
Mortola JP, 2009, COMP BIOCHEM PHYS A, V153, P359, DOI 10.1016/j.cbpa.2009.02.041
Mortola JP, 1998, CAN J PHYSIOL PHARM, V76, P937, DOI 10.1139/cjpp-76-10-11-937
Niewold TA, 2007, POULTRY SCI, V86, P605, DOI 10.1093/ps/86.4.605
Nikami H, 2008, LIFE SCI, V82, P1191, DOI 10.1016/j.lfs.2008.04.003
Nomoto S, 2003, JPN J PHYSIOL, V53, P253, DOI 10.2170/jjphysiol.53.253
Nord A, 2020, J EXP BIOL, V223, DOI 10.1242/jeb.219287
Owen-Ashley NT, 2006, HORM BEHAV, V49, P15, DOI 10.1016/j.yhbeh.2005.04.009
Pesta D, 2012, METHODS MOL BIOL, V810, P25, DOI 10.1007/978-1-61779-382-0_3
PITTMAN QJ, 1976, AM J PHYSIOL, V230, P1284, DOI 10.1152/ajplegacy.1976.230.5.1284
Protti A, 2014, PLOS ONE, V9, DOI 10.1371/journal.pone.0096205
Rakus K, 2017, DEV COMP IMMUNOL, V66, P84, DOI 10.1016/j.dci.2016.06.027
Romanovsky AA, 2005, FRONT BIOSCI-LANDMRK, V10, P2193, DOI 10.2741/1690
Romanovsky AA, 2002, J APPL PHYSIOL, V92, P2667, DOI 10.1152/japplphysiol.01173.2001
Romanovsky AA, 1996, AM J PHYSIOL-REG I, V270, pR693, DOI 10.1152/ajpregu.1996.270.4.R693
Rowland LA, 2015, BIOL REV, V90, P1279, DOI 10.1111/brv.12157
Ruf T, 2015, BIOL REV, V90, P891, DOI 10.1111/brv.12137
Ruhs EC, 2019, PHYSIOL BIOCHEM ZOOL, V92, P106, DOI 10.1086/701389

Schrott LM, 2004, BRAIN BEHAV IMMUN, V18, P515, DOI 10.1016/j.bbi.2003.12.006
Scott GR, 2008, J EXP BIOL, V211, P1326, DOI 10.1242/jeb.015958
Shahir MH, 2012, BRIT POULTRY SCI, V53, P538, DOI 10.1080/00071668.2012.719604
Shearer, 2010, JOVE-J VIS EXP, P1
Sirsat SKG, 2016, J EXP BIOL, V219, P1214, DOI 10.1242/jeb.132282
Sköld-Chiriac S, 2015, J EXP BIOL, V218, P2961, DOI 10.1242/jeb.122150
Steiner AA, 2019, TRENDS ENDOCRIN MET, V30, P875, DOI 10.1016/j.tem.2019.08.012
Steiner AA, 2009, AM J PHYSIOL-REG I, V297, pR485, DOI 10.1152/ajpregu.91026.2008
Stier A, 2014, J COMP PHYSIOL B, V184, P1021, DOI 10.1007/s00360-014-0856-6
Straub, 2015, ORIGIN CHRONIC INFLA, P151
Tattersall GJ, 2003, J EXP BIOL, V206, P33, DOI 10.1242/jeb.00057
Tattersall GJ, 2009, J PHYSIOL-LONDON, V587, P5259, DOI 10.1113/jphysiol.2009.175828
Tattersall GJ, 2009, SCIENCE, V325, P468, DOI 10.1126/science.1175553
Tazawa H, 2004, AVIAN POULT BIOL REV, V15, P166, DOI 10.3184/147020604783638056
Tickle PG, 2018, SCI REP-UK, V8, DOI 10.1038/s41598-018-22604-2
Topp H, 2008, PEDIATR RES, V64, P246, DOI 10.1203/PDR.0b013e31817cfca6
Toro-Velasquez PA, 2014, COMP BIOCHEM PHYS A, V176, P13, DOI 10.1016/j.cbpa.2014.06.008
Toyomizu M, 2002, FEBS LETT, V529, P313, DOI 10.1016/S0014-5793(02)03395-1
Trumbeckaite S, 2001, EUR J BIOCHEM, V268, P1422, DOI 10.1046/j.1432-1327.2001.02012.x
Tsang M, 2006, PHYSIOL BEHAV, V89, P235, DOI 10.1016/j.physbeh.2006.06.005
Tzschentke B, 1999, ORNIS FENNICA, V76, P189
Walter I, 2009, J EXP BIOL, V212, P2328, DOI 10.1242/jeb.029009
WOLFENSON D, 1981, PFLUG ARCH EUR J PHY, V390, P86, DOI 10.1007/BF00582717
WOOD SC, 1991, ANNU REV PHYSIOL, V53, P71, DOI 10.1146/annurev.ph.53.030191.000443
NR 87
TC 11
Z9 11
U1 1
U2 7
PU WILEY
PI HOBOKEN
PA 111 RIVER ST, HOBOKEN 07030-5774, NJ USA
SN 0022-3751
EI 1469-7793
J9 J PHYSIOL-LONDON
JI J. Physiol.-London
PD JUN
PY 2021
VL 599
IS 11
BP 2969

EP 2986
DI 10.1113/JP281385
EA MAY 2021
PG 18
WC Neurosciences; Physiology
WE Science Citation Index Expanded (SCI-EXPANDED)
SC Neurosciences & Neurology; Physiology
GA SK5ZK
UT WOS:000646294700001
PM 33823064
OA Bronze
DA 2025-05-07
ER

PT J
AU Paietta, EN
Kraberger, S
Custer, JM
Vargas, KL
Ehmke, E
Yoder, AD
Varsani, A
AF Paietta, Elise N.
Kraberger, Simona
Custer, Joy M.
Vargas, Karla L.
Ehmke, Erin
Yoder, Anne D.
Varsani, Arvind
TI Metagenome-assembled microvirus and cressdnavirus genomes from fecal samples of house mice (Mus musculus)
SO MICROBIOLOGY RESOURCE ANNOUNCEMENTS
LA English
DT Article
DE microvirus; cressdnavirus; Mus musculus
AB House mice, Mus musculus, are highly adapted to anthropogenic spaces. Fecal samples were collected from house mice entering primate enclosure areas at the Duke Lemur Center (Durham, NC, USA). We identified 14 cressdnavirus and 59 microvirus genomes in these mouse feces.
C1 [Paietta, Elise N.; Yoder, Anne D.] Duke Univ, Dept Biol, Durham, NC USA.
[Kraberger, Simona; Custer, Joy M.; Vargas, Karla L.; Varsani, Arvind] Arizona State Univ, Biodesign Ctr Fundamental & Appl Microbi, Ctr Evolut & Med, Sch Life Sci, Tempe, AZ 85306 USA.
[Ehmke, Erin] Duke Lemur Ctr, Durham, NC USA.
[Varsani, Arvind] Univ Cape Town, Dept Integrat Biomed Sci, Struct Biol Res Unit, Cape Town, South Africa.
C3 Duke University; Arizona State University; Arizona State University-Tempe; University of Cape Town
RP Varsani, A (autor correspondente), Arizona State Univ, Biodesign Ctr Fundamental & Appl Microbi, Ctr Evolut & Med, Sch Life Sci, Tempe, AZ 85306 USA.; Varsani, A (autor correspondente), Univ Cape Town, Dept Integrat Biomed Sci, Struct Biol Res Unit, Cape Town, South Africa.
EM arvind.varsani@asu.edu

RI Ehmke, Erin/KWU-9139-2024; Varsani, Arvind/JOZ-5299-2023
OI Varsani, Arvind/0000-0003-4111-2415
FU DU | Triangle Comparative and Evolutionary Medicine Center, Duke
University (TriCEM); Sigma Xia (SigmaXiSociety)
FX DU | Triangle Comparative and Evolutionary Medicine Center, Duke
University (TriCEM) Elise N. Paietta Anne D. YoderSigma Xia
(SigmaXiSociety) Elise N. Paietta
CR Bolger AM, 2014, BIOINFORMATICS, V30, P2114, DOI
10.1093/bioinformatics/btu170
Buchfink B, 2015, NAT METHODS, V12, P59, DOI 10.1038/nmeth.3176
Bushnell B, 2015, BBMAP SHORT READ ALI
Cao Y, 2014, BIOINFORMATICS, V30, P1674, DOI
10.1093/bioinformatics/btu104
Chalkowski K, 2018, TRENDS PARASITOL, V34, P655, DOI
10.1016/j.pt.2018.05.008
Cherwa JE., 2011, ELS, DOI [10.1002/047001590X, DOI
10.1002/047001590X]
Díez-Villaseñor C, 2019, NAT COMMUN, V10, DOI 10.1038/s41467-018-
08167-w
Fiallo-Olivé E, 2021, J GEN VIROL, V102, DOI 10.1099/jgv.0.001696
Kieft K, 2020, MICROBIOME, V8, DOI 10.1186/s40168-020-00867-0
Krupovic M, 2020, J VIROL, V94, DOI 10.1128/JVI.00582-20
MUHIRE BM, 2014, PLOS ONE, V9, DOI DOI 10.1371/JOURNAL.PONE.0108277
Pankovics P, 2021, INFECT GENET EVOL, V93, DOI
10.1016/j.meegid.2021.104942
Phan TG, 2011, PLOS PATHOG, V7, DOI 10.1371/journal.ppat.1002218
Pitt, 2012, INVASIVE RODENTS US
Pittermannova P, 2021, PATHOGENS, V10, DOI 10.3390/pathogens10060777
Tisza MJ, 2021, VIRUS EVOL, V7, DOI 10.1093/ve/veaa100
Williams SH, 2018, MBIO, V9, DOI [10.1128/mBio.01354-17,
10.1128/mbio.01354-17]
NR 17
TC 0
Z9 0
U1 1
U2 5
PU AMER SOC MICROBIOLOGY
PI WASHINGTON
PA 1752 N ST NW, WASHINGTON, DC 20036-2904 USA
SN 2576-098X
J9 MICROBIOL RESOUR ANN
JI Microbiol. Resour. Ann.
PD AUG 13
PY 2024
VL 13
IS 8
DI 10.1128/mra.00331-24
EA JUL 2024
PG 4
WC Microbiology
WE Emerging Sources Citation Index (ESCI)
SC Microbiology
GA C6B7D
UT WOS:001265166100003

PM 38975773
OA Green Published, gold
DA 2025-05-07
ER

PT J
AU Kou, HH
   Parker, W
AF Kou, Henry H.
   Parker, William
TI Intestinal worms eating neuropsychiatric disorders? Apparently so
SO BRAIN RESEARCH
LA English
DT Article
DE Allergy; Autoimmunity; Biota; Biome; Helminth; Inflammation
ID GUT MICROBIOME; OLD FRIENDS; INFLAMMATION; HYGIENE; RECONSTITUTION;
   DEPRESSION; NUTRITION; HELMINTHS; PARASITES; EXERCISE
AB A number of factors in Western society, including inflammatory diets, sedentary lifestyles, vitamin D deficiency and chronic psychological stress, are known to induce inflammation and to be associated with neuropsychiatric disorders. One factor that is emerging as a potential inflammation inducing factor is biota depletion, or loss of biodiversity from the ecosystem of the human body as a result of industrialization. Originally known as the "hygiene hypothesis", biota alteration theory describes the effects of biota alteration on the human immune system. Work on this topic has pinpointed depletion of helminths as a key loss to the body's ecosystem in Western society, and suggests that some exposure to helminths, ubiquitous prior to the modern era, may be necessary for normal immune system development. Sociomedical studies of humans "self-treating" with helminths as well as limited studies in animal models strongly suggest that helminth therapy may be a productive approach toward treating a range of neuropsychiatric disorders, including chronic fatigue, migraine headaches, depression and anxiety disorders. However, helminth therapy faces some daunting hurdles, including the lack of a financial incentive for development, despite a tremendous potential market for the organisms. It is argued that benevolent donation for early trials as well as changes in regulatory policy to accommodate helminth therapy may be important for the field to develop. It is hoped that future success with some high-profile trials can propel the field, now dominated more by self-treatment than by clinical trials, forward into the main stream of medicine. (C) 2018 Elsevier B.V. All rights reserved.
C1 [Kou, Henry H.; Parker, William] Duke Univ, Med Ctr, Dept Surg, Durham, NC 27710 USA.
C3 Duke University
RP Parker, W (autor correspondente), Duke Univ, Med Ctr, Dept Surg, Durham, NC 27710 USA.
EM Henry.Kou@Duke.edu; William.Parker@Duke.edu
OI Parker, William/0000-0003-3644-9152
FU Triangle Center for Evolutionary Medicine (TriCEM); inflammation in
   Western society
FX This work was supported by the Triangle Center for Evolutionary Medicine
   (TriCEM), and by Immunity's Forge, a non-profit organization supporting

research on inflammation in Western society.

CR [Anonymous], 2016, CLIN RES REGUL AFF, DOI DOI 10.1080/10601333.2016.1210159
Becker KG, 2007, MED HYPOTHESES, V69, P731, DOI 10.1016/j.mehy.2007.02.019
Bilbo SD, 2011, MED HYPOTHESES, V77, P494, DOI 10.1016/j.mehy.2011.06.019
Blackley C.R., 1873, EXPT RESEARCHES CAUS
Carabotti M, 2015, ANN GASTROENTEROL, V28, P203
Cheng A.M., 2015, J EVOL MED, V3
Correale J, 2007, ANN NEUROL, V61, P97, DOI 10.1002/ana.21067
Dantzer R, 2008, NAT REV NEUROSCI, V9, P46, DOI 10.1038/nrn2297
Flowers S, 2013, NATURE, V493, P163, DOI 10.1038/493163c
Gleeson M, 2004, J SPORT SCI, V22, P115, DOI 10.1080/02640410310001640590
Holick MF, 2007, NEW ENGL J MED, V357, P266, DOI 10.1056/NEJMra070553
Kau AL, 2011, NATURE, V474, P327, DOI 10.1038/nature10213
Krishnadas R, 2012, J NEUROL NEUROSUR PS, V83, P495, DOI 10.1136/jnnp-2011-301779
Lakhan SE, 2010, NUTR METAB, V7, DOI 10.1186/1743-7075-7-79
Liu J, 2017, J HELMINTHOL, V91, P267, DOI 10.1017/S0022149X16000316
Lukes J, 2015, PLOS PATHOG, V11, DOI 10.1371/journal.ppat.1005039
Lukes J, 2014, TRENDS PARASITOL, V30, P377, DOI 10.1016/j.pt.2014.06.005
Moller P, 1996, CHEM-BIOL INTERACT, V102, P17, DOI 10.1016/0009-2797(96)03729-5
Muegge BD, 2011, SCIENCE, V332, P970, DOI 10.1126/science.1198719
National Bureau of Statistics of China, 2016, NON TRADITIONAL REF
Newman A., 1999, NY TIMES
Parker William, 2013, Evolution Medicine and Public Health, P89, DOI 10.1093/emph/eot008
Parker W, 2012, CURR MED RES OPIN, V28, P1193, DOI 10.1185/03007995.2012.695731
Raison CL, 2010, ARCH GEN PSYCHIAT, V67, P1211, DOI 10.1001/archgenpsychiatry.2010.161
Rook GAW, 2014, CLIN EXP IMMUNOL, V177, P1, DOI 10.1111/cei.12269
Rook G. A. W., 2009, HYGIENE HYPOTHESIS D
Rook GAW, 2014, ADV EXP MED BIOL, V817, P319, DOI 10.1007/978-1-4939-0897-4_15
Rook GAW, 2009, IMMUNOLOGY, V126, P3, DOI 10.1111/j.1365-2567.2008.03007.x
Sears B, 2015, J AM COLL NUTR, V34, P14, DOI 10.1080/07315724.2015.1080105
Sears B, 2011, J OBES, V2011, DOI 10.1155/2011/431985
Smyth K, 2017, J CLIN MED, V6, DOI 10.3390/jcm6100098
STRACHAN DP, 1989, BRIT MED J, V299, P1259, DOI 10.1136/bmj.299.6710.1259
Summers RW, 2005, GUT, V54, P87, DOI 10.1136/gut.2004.041749
TURTON JA, 1976, LANCET, V2, P686
Weinstock JV, 2012, NATURE, V491, P183, DOI 10.1038/491183a
Williamson LL, 2016, BRAIN BEHAV IMMUN, V51, P14, DOI 10.1016/j.bbi.2015.07.006
Yatsunenko T, 2012, NATURE, V486, P222, DOI 10.1038/nature11053

Zaccone P, 2006, PARASITE IMMUNOL, V28, P515, DOI 10.1111/j.1365-3024.2006.00879.x
NR 38
TC 4
Z9 5
U1 1
U2 49
PU ELSEVIER
PI AMSTERDAM
PA RADARWEG 29, 1043 NX AMSTERDAM, NETHERLANDS
SN 0006-8993
EI 1872-6240
J9 BRAIN RES
JI Brain Res.
PD AUG 15
PY 2018
VL 1693
SI SI
BP 218
EP 221
DI 10.1016/j.brainres.2018.01.023
PN B
PG 4
WC Neurosciences
WE Science Citation Index Expanded (SCI-EXPANDED)
SC Neurosciences & Neurology
GA GL1ZQ
UT WOS:000436913200019
PM 29402395
DA 2025-05-07
ER

PT J
AU Swynghedauw, B
AF Swynghedauw, Bernard
TI Medical consequences of global warming
SO PRESSE MEDICALE
LA French
DT Review
ID CLIMATE-CHANGE; AMBIENT-TEMPERATURE; OZONE DEPLETION; BLOOD-PRESSURE; SKIN-CANCER; HEAT WAVES; EL-NINO; MORTALITY; HEALTH; IMPACT
AB Introduction > The global warming of the planet and its anthropogenic origin ore no longer debatable. Nevertheless, from a medical point of view, while the epidemiological consequences of the warming are rather well-known, the biological consequences ore still poorly documented. This is a good example of evolutionary (or darwinian) medicine.
Methods > The research strategy of this systematic review is based on both PubMed during the period of 2000-2007 and several reviews articles for the period > 2000.
Results > From a medical point of view, there ore four types of consequences.
1-The simple elevation of the average external temperature is accompanied by on increased global mortality and morbidity, the mortality/external temperature is a J curve, with the worm branch more

pronounced than the cold one. A recent study on 50 different cities had confirmed that global, and more specifically cardiovascular mortalities were enhanced at the two extreme of the temperatures.

2-The acute heatwaves, such as that which happened in France in August 2003, hove been studied in detail by several groups. The mortality which was observed during the recent heatwaves was not compensated by harvesting, strongly suggesting that we were dealing with heat stroke, and that such an increased mortality was more reflecting the limits of our adaptational capacities than aggravation of a previously altered health status.

3-Climate changes have modified the repartition and virulence of pathogenic agents (dengue, malaria...) and above all their vectors. Such modifications were exponential and ore likely to reflect the biological properties of parasites.

4-Indirect consequences of global worming include variations in the hydraulic cycle, the new form of tropical hurricanes and many different changes affecting both biodiversity and ecosystems. They will likely result in on increased level of poverty.

Discussion > These finding gave rise to several basic biological questions, rarely evoked, and that concern the limits of the adaptational capacities of human genome. our genome has indeed been shaped in the post by a rather cold environment which has acutely been modified. The immediate physiological regulation includes sweating and skin vasodilatation. The latter may strongly enhance the cardiac output which explains the heat-induced cardiac decompensation. Long term regulation depends upon the numerous mechanisms of uncoupling of the mitochondrial respiration. For the moment, the thermolytic mechanisms and their regulation were rather poorly documented.

C1 Hop Lariboisiere, INSERM, U689, Ctr Rech Cardiovasc, F-75475 Paris 10, France.
C3 Universite Paris Cite; Institut National de la Sante et de la Recherche
Medicale (Inserm); Assistance Publique Hopitaux Paris (APHP); Hopital Universitaire Lariboisiere-Fernand-Widal - APHP
RP Swynghedauw, B (autor correspondente), Hop Lariboisiere, INSERM, U689, Ctr Rech Cardiovasc, 41 Bd Chapelle, F-75475 Paris 10, France.
EM Bernard.Swynghedauw@larib.inserm.fr
CR Agar N, 2005, MUTAT RES-FUND MOL M, V571, P121, DOI 10.1016/j.mrfmmm.2004.11.016
ALGORE, 2007, URGENCE PLANETE TERR
[Anonymous], 2007, Nature, V445, P567
[Anonymous], 1999, EVOLUTIONARY MED
[Anonymous], TRAITE CARDIOLOGIE
Bach JF, 2002, NEW ENGL J MED, V347, P911, DOI 10.1056/NEJMra020100
Basu R, 2002, EPIDEMIOL REV, V24, P190, DOI 10.1093/epirev/mxf007
Belmin J, 2003, PRESSE MED, V32, P1591
BERGER P, 1994, RYTHMES CHAOS
Booth S, 2005, ENVIRON HEALTH PERSP, V113, P521, DOI 10.1289/ehp.7603
Bouchama A, 2002, NEW ENGL J MED, V346, P1978, DOI 10.1056/NEJMra011089
Braga ALF, 2001, EPIDEMIOLOGY, V12, P662
Checkley W, 2000, LANCET, V355, P442, DOI 10.1016/S0140-6736(00)82010-3
Claessens YE, 2006, CRIT CARE, V10, DOI 10.1186/cc5092

Cordain L, 1997, WORLD REV NUTR DIET, V81, P49
Dhainaut JF, 2004, CRIT CARE, V8, P1, DOI 10.1186/cc2404
Díaz S, 2006, PLOS BIOL, V4, P1300, DOI 10.1371/journal.pbio.0040277
EATON SB, 1985, NEW ENGL J MED, V312, P283, DOI 10.1056/NEJM198501313120505
Ebi KL, 2005, ENVIRON HEALTH PERSP, V113, P335, DOI 10.1289/ehp.7380
ELBERT T, 1994, PHYSIOL REV, V74, P1, DOI 10.1152/physrev.1994.74.1.1
Flannery T.F., 2006, WEATHER MAKERS MAN I
Gordon CJ, 2003, ENVIRON RES, V92, P1, DOI 10.1016/S0013-9351(02)00008-7
GUENARD H, 1996, PHYSL ANIMALE
Haines A, 2006, LANCET, V367, P2101, DOI [10.1016/S0140-6736(06)68933-2, 10.1016/j.puhe.2006.01.002]
Hales S, 2003, LANCET, V362, P1775, DOI 10.1016/S0140-6736(03)14939-2
Hansen J, 2006, P NATL ACAD SCI USA, V103, P14288, DOI 10.1073/pnas.0606291103
HEMON D, 2003, RAPPORT ETAPE 1 3 ES, P1
Hopp MJ, 2003, CLIM RES, V25, P85, DOI 10.3354/cr025085
*INT FED RED CROSS, WORLD DIS REP
JOUZEL J, 2007, CLIMAT JEU DANGEREUX
Keatinge WR, 1997, LANCET, V349, P1341, DOI 10.1016/S0140-6736(96)12338-2
Kenney WL, 2003, J APPL PHYSIOL, V95, P2598, DOI 10.1152/japplphysiol.00202.2003
Kerr RA, 2006, SCIENCE, V312, P1854, DOI 10.1126/science.312.5782.1854
Lavallart Benoit, 2004, Rev Prat, V54, P1298
Le Tertre A, 2006, EPIDEMIOLOGY, V17, P75, DOI 10.1097/01.ede.0000187650.36636.1f
Lowell BB, 2000, NATURE, V404, P652, DOI 10.1038/35007527
MADRONICH S, 1993, NATURE, V366, P23, DOI 10.1038/366023a0
Martens WJM, 1998, ENVIRON HEALTH PERSP, V106, P241, DOI 10.2307/3433924
McMichael AJ, 2006, LANCET, V367, P859, DOI 10.1016/S0140-6736(06)68079-3
Medina-Ramón M, 2007, OCCUP ENVIRON MED, V64, P827, DOI 10.1136/oem.2007.033175
Medina-Ramón M, 2006, ENVIRON HEALTH PERSP, V114, P1331, DOI 10.1289/ehp.9074
Meehl GA, 2004, SCIENCE, V305, P994, DOI 10.1126/science.1098704
Modesti PA, 2006, HYPERTENSION, V47, P155, DOI 10.1161/01.HYP.0000199192.17126.d4
Momiyama M, 1972, Int J Biometeorol, V16, P329, DOI 10.1007/BF01553618
MORISON WL, 1989, PHOTOCHEM PHOTOBIOL, V50, P515, DOI 10.1111/j.1751-1097.1989.tb05557.x
NELSON N, 1947, AM J PHYSIOL, V151, P626, DOI 10.1152/ajplegacy.1947.151.2.626
Nyberg J, 2007, NATURE, V447, P698, DOI 10.1038/nature05895
O'Neill MS, 2005, INT J BIOMETEOROL, V50, P121, DOI 10.1007/s00484-005-0269-z
Pandolf KB, 1997, EXP AGING RES, V23, P69, DOI 10.1080/03610739708254027
Pascal M, 2006, INT J BIOMETEOROL, V50, P144, DOI 10.1007/s00484-005-0003-x

Pascual M, 2000, SCIENCE, V289, P1766, DOI 10.1126/science.289.5485.1766
Patz JA, 2006, P NATL ACAD SCI USA, V103, P5635, DOI 10.1073/pnas.0601493103
Patz JA, 1996, JAMA-J AM MED ASSOC, V275, P217, DOI 10.1001/jama.275.3.217
Patz JA, 2005, NATURE, V438, P310, DOI 10.1038/nature04188
Roos P. B., 2015, International Journal of Climate Change: Impacts and Responses, V7, P13
Sakurada S, 1998, J APPL PHYSIOL, V84, P207, DOI 10.1152/jappl.1998.84.1.207
Sega R, 1998, J HYPERTENS, V16, P1585, DOI 10.1097/00004872-199816110-00004
Sheridan S.C., 1998, World Resource Review, V10, P375
Sheth T, 1999, J AM COLL CARDIOL, V33, P1916, DOI 10.1016/S0735-1097(99)00137-0
Silva JE, 2006, PHYSIOL REV, V86, P435, DOI 10.1152/physrev.00009.2005
Slaper H, 1996, NATURE, V384, P256, DOI 10.1038/384256a0
Stearns S.C., 1999, Evolution in health and disease
Stott PA, 2004, NATURE, V432, P610, DOI 10.1038/nature03089
Swynghedauw B, 2004, ACTA CHIR BELG, V104, P132, DOI 10.1080/00015458.2004.11679525
Swynghedauw Bernard, 2008, P197, DOI 10.1007/978-3-540-78993-2_12
Tanser FC, 2003, LANCET, V362, P1792, DOI 10.1016/S0140-6736(03)14898-2
Tibbetts J, 2007, ENVIRON HEALTH PERSP, V115, pA196, DOI 10.1289/ehp.115-a196
URBACH F, 1991, ENVIRON HEALTH PERSP, V96, P175, DOI 10.2307/3431228
Valleron AJ, 2004, CR BIOL, V327, P1125, DOI 10.1016/j.crvi.2004.09.009
Weiss RA, 2004, NAT MED, V10, pS70, DOI 10.1038/nm1150
Zhang XB, 2007, NATURE, V448, P461, DOI 10.1038/nature06025
[No title captured]
NR 72
TC 8
Z9 10
U1 1
U2 43
PU MASSON EDITEUR
PI MOULINEAUX CEDEX 9
PA 21 STREET CAMILLE DESMOULINS, ISSY, 92789 MOULINEAUX CEDEX 9, FRANCE
SN 0755-4982
EI 2213-0276
J9 PRESSE MED
JI Presse Med.
PD APR
PY 2009
VL 38
IS 4
BP 551
EP 561
DI 10.1016/j.lpm.2008.02.022
PG 11
WC Medicine, General & Internal

WE Science Citation Index Expanded (SCI-EXPANDED)
SC General & Internal Medicine
GA 440AX
UT WOS:000265671300006
PM 19059749
DA 2025-05-07
ER

PT J
AU Jatzlauk, G
Bartel, S
Heine, H
Schloter, M
Krauss-Etschmann, S
AF Jatzlauk, G.
Bartel, S.
Heine, H.
Schloter, M.
Krauss-Etschmann, S.
TI Influences of environmental bacteria and their metabolites on allergies,
asthma, and host microbiota
SO ALLERGY
LA English
DT Review
DE asthma; environment and hygiene hypothesis; prevention
ID PLACEBO-CONTROLLED TRIAL; RANDOMIZED CONTROLLED-TRIALS; DAY-CARE ATTENDANCE; REGULATORY T-CELLS; CHILDHOOD ASTHMA; ATOPIC-DERMATITIS; CESAREAN-SECTION; GUT MICROBIOTA; EARLY-LIFE; DOUBLE-BLIND
AB The prevalence of allergic diseases and asthma has dramatically increased over the last decades, resulting in a high burden for patients and healthcare systems. Thus, there is an unmet need to develop preventative strategies for these diseases. Epidemiological studies show that reduced exposure to environmental bacteria in early life (eg, birth by cesarean section, being formula-fed, growing up in an urban environment or with less contact to various persons) is associated with an increased risk to develop allergies and asthma later in life. Conversely, a reduced risk for asthma is consistently found in children growing up on traditional farms, thereby being exposed to a wide spectrum of microbes. However, clinical studies with bacteria to prevent allergic diseases are still rare and to some extent contradicting. A detailed mechanistic understanding of how environmental microbes influence the development of the human microbiome and the immune system is important to enable the development of novel preventative approaches that are based on the early modulation of the host microbiota and immunity. In this mini-review, we summarize current knowledge and experimental evidence for the potential of bacteria and their metabolites to be used for the prevention of asthma and allergic diseases.
C1 [Jatzlauk, G.; Bartel, S.; Krauss-Etschmann, S.] Leibniz Ctr Med & Biosci, Res Ctr Borstel, Div Early Life Origins Chron Lung Dis Prior Area, Borstel, Germany.
[Jatzlauk, G.; Bartel, S.; Heine, H.; Krauss-Etschmann, S.] German Ctr Lung Res DZL, ARCN, Borstel, Germany.

[Heine, H.] Leibniz Ctr Med & Biosci, Res Ctr Borstel, Div Innate Immun Prior Area Asthma & Allergy, Borstel, Germany.
[Schloter, M.] Helmholtz Zentrum Munchen, Res Unit Environm Genom, Oberschleissheim, Germany.
[Krauss-Etschmann, S.] Christian Albrechts Univ Kiel, Inst Expt Med, Kiel, Germany.
C3 Leibniz Association; Forschungszentrum Borstel; Leibniz Association; Forschungszentrum Borstel; Helmholtz Association; Helmholtz-Center Munich - German Research Center for Environmental Health; University of Kiel
RP Krauss-Etschmann, S (autor correspondente), Leibniz Ctr Med & Biosci, Res Ctr Borstel, Borstel, Germany.
EM skrauss-etschmann@fz-borstel.de
RI Schloter, Michael/H-9151-2013; Heine, Holger/H-4552-2012; Bartel, Sabine/P-9463-2019; Krauss-Etschmann, Susanne/H-5198-2018
OI Krauss-Etschmann, Susanne/0000-0001-5945-5702; Bartel, Sabine/0000-0002-9163-795X
FU Leibniz competition SAW; Leibniz Campus Evolutionary Medicine of the Lung
FX Funded by the Leibniz competition SAW 2015, "The lung microbiota at the interface between airway epithelium and environment" (SKE) and the Leibniz Campus Evolutionary Medicine of the Lung (SKE, SB).
CR Abrahamsson TR, 2014, CLIN EXP ALLERGY, V44, P842, DOI 10.1111/cea.12253
Abrahamsson TR, 2012, J ALLERGY CLIN IMMUN, V129, P434, DOI 10.1016/j.jaci.2011.10.025
Accordini S, 2013, INT ARCH ALLERGY IMM, V160, P93, DOI 10.1159/000338998
Arcos-Burgos M, 2002, CLIN GENET, V61, P233, DOI 10.1034/j.1399-0004.2002.610401.x
Arnold IC, 2011, J CLIN INVEST, V121, P3088, DOI 10.1172/JCI45041
Arpaia N, 2013, NATURE, V504, P451, DOI 10.1038/nature12726
Arrieta MC, 2015, SCI TRANSL MED, V7, DOI 10.1126/scitranslmed.aab2271
Asher MI., 2014, The global asthma report 2014
Azad MB, 2013, CAN MED ASSOC J, V185, P385, DOI 10.1503/cmaj.121189
Ball TM, 2000, NEW ENGL J MED, V343, P538, DOI 10.1056/NEJM200008243430803
Bantz Selene K, 2014, J Clin Cell Immunol, V5
Bermudez-Brito M, 2013, PLOS ONE, V8, DOI 10.1371/journal.pone.0059370
Betran AP, 2016, BJOG-INT J OBSTET GY, V123, P667, DOI 10.1111/1471-0528.13526
Bisgaard H, 2011, J ALLERGY CLIN IMMUN, V128, P646, DOI 10.1016/j.jaci.2011.04.060
Bode L, 2015, EARLY HUM DEV, V91, P619, DOI 10.1016/j.earlhumdev.2015.09.001
Brown SDM, 2006, GENOME DYN, V2, P33, DOI 10.1159/000095091
Cahenzli J, 2013, CELL HOST MICROBE, V14, P559, DOI 10.1016/j.chom.2013.10.004
Chen Y, 2008, J INFECT DIS, V198, P553, DOI 10.1086/590158
Chu DM, 2017, NAT MED, V23, P314, DOI 10.1038/nm.4272
Cuello-Garcia CA, 2015, J ALLERGY CLIN IMMUN, V136, P952, DOI [10.1016/j.jaci.2015.04.031, 10.1016/j.jaci.2015.07.021]

Debarry J, 2007, J ALLERGY CLIN IMMUN, V119, P1514, DOI 10.1016/j.jaci.2007.03.023
DeKruyff RH, 2014, IMMUNOL REV, V260, P235, DOI 10.1111/imr.12187
Dickson RP, 2013, EXPERT REV RESP MED, V7, P245, DOI [10.1586/ers.13.24, 10.1586/ERS.13.24]
Dominguez-Bello MG, 2016, NAT MED, V22, P250, DOI 10.1038/nm.4039
Dominguez-Bello MG, 2010, P NATL ACAD SCI USA, V107, P11971, DOI 10.1073/pnas.1002601107
Ege MJ, 2011, NEW ENGL J MED, V364, P701, DOI 10.1056/NEJMoa1007302
Fong FLY, 2016, SCI REP-UK, V6, DOI 10.1038/srep22845
Fujimura KE, 2016, NAT MED, V22, P1187, DOI 10.1038/nm.4176
Gerhold K, 2002, J ALLERGY CLIN IMMUN, V110, P110, DOI 10.1067/mai.2002.125831
Gibson J, 2013, EUROPEAN LUNG WHITE BOOK: RESPIRATORY HEALTH AND DISEASE IN EUROPE, P1
GlobalData, 2014, ASTHM EP FOR 2023
Gollwitzer ES, 2014, NAT MED, V20, P642, DOI 10.1038/nm.3568
Haahtela T, 2015, CLIN EXP ALLERGY, V45, P891, DOI 10.1111/cea.12527
Haby MM, 2000, PEDIATR PULM, V30, P377, DOI 10.1002/1099-0496(200011)30:5<377::AID-PPUL3>3.3.CO;2-V
Hasegawa K, 2017, PEDIATR INT, V59, P473, DOI 10.1111/ped.13168
Herbst T, 2011, AM J RESP CRIT CARE, V184, P198, DOI 10.1164/rccm.201010-1574OC
Hunt JRF, 2005, CLIN EXP ALLERGY, V35, P685, DOI 10.1111/j.1365-2222.2005.02239.x
Illi S, 2012, J ALLERGY CLIN IMMUN, V129, P1470, DOI 10.1016/j.jaci.2012.03.013
Kalliomäki M, 2003, LANCET, V361, P1869, DOI 10.1016/S0140-6736(03)13490-3
Kalliomäki M, 2001, LANCET, V357, P1076, DOI 10.1016/S0140-6736(00)04259-8
Kanauchi O, 2005, CURR PHARM DESIGN, V11, P1047, DOI 10.2174/1381612053381675
Kauth M, 2016, PEDIAT ALLERG IMM-UK, V27, P340, DOI 10.1111/pai.12559
Kepert I, 2017, J ALLERGY CLIN IMMUN, V139, P1525, DOI 10.1016/j.jaci.2016.09.003
Khanna R, 2011, IMMUNOL CELL BIOL, V89, P330, DOI 10.1038/icb.2010.173
Kim SO, 2014, ANN ALLERG ASTHMA IM, V113, P217, DOI 10.1016/j.anai.2014.05.021
Kitagaki K, 2006, CLIN EXP IMMUNOL, V143, P249, DOI 10.1111/j.1365-2249.2005.03003.x
Kopp MV, 2008, PEDIATRICS, V121, pE850, DOI 10.1542/peds.2007-1492
Krämer U, 1999, LANCET, V353, P450, DOI 10.1016/S0140-6736(98)06329-6
Kukkonen K, 2007, J ALLERGY CLIN IMMUN, V119, P192, DOI 10.1016/j.jaci.2006.09.009
Laursen MF, 2015, BMC MICROBIOL, V15, DOI 10.1186/s12866-015-0477-6
Lewis ZT, 2017, NESTLE NUTR WORKS SE, V88, P149, DOI 10.1159/000455399
Li YN, 2012, ANN ALLERG ASTHMA IM, V109, P201, DOI 10.1016/j.anai.2012.06.017
Lim SK, 2017, SCI REP-UK, V7, DOI 10.1038/srep40040
Lodge CJ, 2015, ACTA PAEDIATR, V104, P38, DOI 10.1111/apa.13132
Malago JJ, 2005, CLIN EXP IMMUNOL, V141, P62, DOI 10.1111/j.1365-2249.2005.02810.x
Mazmanian SK, 2005, CELL, V122, P107, DOI 10.1016/j.cell.2005.05.007

Mechoud MA, 2012, INT IMMUNOPHARMACOL, V14, P446, DOI 10.1016/j.intimp.2012.08.020
Ober C, 2000, AM J HUM GENET, V67, P1154, DOI 10.1016/S0002-9297(07)62946-2
Olszak T, 2012, SCIENCE, V336, P489, DOI 10.1126/science.1219328
Osborn D A., 2007, Cochrane Database Syst Rev
Pawankar R, 2014, WORLD ALLERGY ORGAN, V7, DOI 10.1186/1939-4551-7-12
Pistiner M, 2008, J ALLERGY CLIN IMMUN, V122, P274, DOI 10.1016/j.jaci.2008.05.007
Riedler J, 2001, LANCET, V358, P1129, DOI 10.1016/S0140-6736(01)06252-3
Riedler J, 2000, CLIN EXP ALLERGY, V30, P194, DOI 10.1046/j.1365-2222.2000.00799.x
Russell SL, 2012, EMBO REP, V13, P440, DOI 10.1038/embor.2012.32
Rutayisire E, 2016, BMC GASTROENTEROL, V16, DOI 10.1186/s12876-016-0498-0
Schuijs MJ, 2015, SCIENCE, V349, P1106, DOI 10.1126/science.aac6623
Shim JU, 2016, J ALLERGY CLIN IMMUN, V137, P426, DOI 10.1016/j.jaci.2015.07.010
Shin JH, 2016, FOOD NUTR RES, V60, DOI 10.3402/fnr.v60.32550
Stein K, 2017, J ALLERGY CLIN IMMUN, V139, P667, DOI 10.1016/j.jaci.2016.06.018
Stein MM, 2016, NEW ENGL J MED, V375, P411, DOI 10.1056/NEJMoa1508749
Steinmeyer S, 2015, CURR ALLERGY ASTHM R, V15, DOI 10.1007/s11882-015-0524-2
Strachan DP, 1996, ARCH DIS CHILD, V74, P422, DOI 10.1136/adc.74.5.422
Taylor AL, 2007, J ALLERGY CLIN IMMUN, V119, P184, DOI 10.1016/j.jaci.2006.08.036
Thavagnanam S, 2008, CLIN EXP ALLERGY, V38, P629, DOI 10.1111/j.1365-2222.2007.02780.x
To T, 2013, BMC PUBLIC HEALTH, V13, DOI 10.1186/1471-2458-13-254
Tollanes MC, 2008, J PEDIATR-US, V153, P112, DOI 10.1016/j.jpeds.2008.01.029
Torow N, 2017, MUCOSAL IMMUNOL, V10, P5, DOI 10.1038/mi.2016.81
Townshend J, 2007, BRIT MED J, V335, P198, DOI 10.1136/bmj.39234.651412.AE
Victora CG, 2016, LANCET, V387, P475, DOI 10.1016/S0140-6736(15)01024-7
Von Ehrenstein OS, 2000, CLIN EXP ALLERGY, V30, P187, DOI 10.1046/j.1365-2222.2000.00801.x
von Mutius E, 2007, IMMUNOBIOLOGY, V212, P433, DOI 10.1016/j.imbio.2007.03.002
Waterston RH, 2002, NATURE, V420, P520, DOI 10.1038/nature01262
Wickens K, 2008, J ALLERGY CLIN IMMUN, V122, P788, DOI 10.1016/j.jaci.2008.07.011
Xu JF, 2001, AM J HUM GENET, V68, P1437, DOI 10.1086/320589
Zhou S, 2017, FEMS MICROBIOL LETT, V364, DOI 10.1093/femsle/fnw288
Zuany-Amorim C, 2002, NAT MED, V8, P625, DOI 10.1038/nm0602-625
Zuccotti G, 2015, ALLERGY, V70, P1356, DOI 10.1111/all.12700
NR 88
TC 50
Z9 58
U1 1
U2 54

PU WILEY
PI HOBOKEN
PA 111 RIVER ST, HOBOKEN 07030-5774, NJ USA
SN 0105-4538
EI 1398-9995
J9 ALLERGY
JI Allergy
PD DEC
PY 2017
VL 72
IS 12
BP 1859
EP 1867
DI 10.1111/all.13220
PG 9
WC Allergy; Immunology
WE Science Citation Index Expanded (SCI-EXPANDED)
SC Allergy; Immunology
GA FN3TG
UT WOS:000415921800003
PM 28600901
OA Bronze
DA 2025-05-07
ER

PT J
AU Cheyney, M
   Davis-Floyd, R
AF Cheyney, Melissa
   Davis-Floyd, Robbie
TI Birth and the Big Bad Wolf: Biocultural Evolution and Human Childbirth,
   Part 2
SO INTERNATIONAL JOURNAL OF CHILDBIRTH
LA English
DT Article
DE childbirth; midwifery; obstetrics; subsistence strategies;
   human-technology coevolution
ID SUPINE POSITION; MATERNITY CARE; UNITED-STATES; OUTCOMES; LABOR
AB In Part 2 of this two-part article, we further employ the lens of evolutionary medicine to explore similarities in premodern biocultural features of birth, arguing that these were an outgrowth of our common evolutionary heritage as bipedal primates. These practices grew out of the empiricism of millennia of trial and error and supported humans to give birth in closer alignment with our evolved biology. We argue that many common obstetric procedures today work against this evolved biology. In seeking to manage birth, we sometimes generate an obstetric paradox wherein we (over)intervene in human childbirth to try to keep it safe, yet thereby cause harm. We describe premodern birthing patterns in three sections: (a) eating and drinking at will and unrestrained movement in labor with upright pushing; (b) obligate midwifery and continuous labor support; and (c) the low-intervention birth/long-term breastfeeding/co-sleeping adaptive complex, and discuss how these are still relevant today. We conclude with a set of suggestions for improving the global

technocratic treatment of birth and with a futuristic epilogue about a 7th, cyborgian pig that asks: What will become of birth as humans continue to coevolve with our technologies?
C1 [Cheyney, Melissa] Oregon State Univ, Dept Anthropol, Corvallis, OR 97331 USA.
[Cheyney, Melissa] Midwives Alliance North Amer, Div Res, Montvale, NJ USA.
[Davis-Floyd, Robbie] Univ Texas Austin, Dept Anthropol, Austin, TX 78712 USA.
[Davis-Floyd, Robbie] Soc Appl Anthropol, Oklahoma City, OK USA.
[Davis-Floyd, Robbie] Council Anthropol & Reprod, Austin, TX USA.
C3 Oregon State University; University of Texas System; University of Texas
Austin
RP Davis-Floyd, R (autor correspondente), Univ Texas Austin, Austin, TX 78712 USA.
EM davis-floyd@austin.utexas.edu
OI Davis-Floyd, Robbie/0000-0003-1963-6423
CR Alliman J, 2019, BIRTH-ISS PERINAT C, V46, P234, DOI 10.1111/birt.12433
Althabe F, 2006, BIRTH-ISS PERINAT C, V33, P270, DOI 10.1111/j.1523-536X.2006.00118.x
Anim-Somuah M, 2011, COCHRANE DB SYST REV, DOI 10.1002/14651858.CD000331.pub3
[Anonymous], BIRTH SETTINGS AM OU, DOI DOI 10.17226/25636
[Anonymous], 2000, FEELING SAFE ENOUGH
Berta M, 2019, BMC PREGNANCY CHILDB, V19, DOI 10.1186/s12884-019-2620-0
Bodner-Adler B, 2003, WIEN KLIN WOCHENSCHR, V115, P720, DOI 10.1007/BF03040889
Buckley SJ, 2015, HORMONAL PHYSL CHILD
Cheyney M., 2019, SQUARING CIRCLE NORM, P204
Cheyney M, 2014, J MIDWIFERY WOM HEAL, V59, P17, DOI 10.1111/jmwh.12172
Dahlberg U, 2013, MIDWIFERY, V29, P407, DOI 10.1016/j.midw.2012.09.006
Das RR, 2014, INT J PEDIAT, V2014, DOI 10.1155/2014/468538
Davis Dana-Ain, 2019, Med Anthropol, V38, P560, DOI 10.1080/01459740.2018.1549389
Davis Dana-Ain., 2019, REPROD INJUSTICE RAC, DOI DOI 10.18574/NYU/9781479812271.001.0001
Davis-Floyd R., BIRTHING TECHNO SAPI
Davis-Floyd R., 2018, WAYS KNOWING BIRTH M, P221
Davis-Floyd R., 2018, Ways of knowing about birth: Mothers, midwives, medicine, and birth activism
Davis-Floyd Robbie., 2003, Birth as an American Rite of Passage
Davis-Floyd RobbieE., 2019, Birth in Eight Cultures
de Jonge A, 2009, MIDWIFERY, V25, P439, DOI 10.1016/j.midw.2007.07.013
DeLoache JudyS., 2000, A World of Babies: Imagined Childcare Guides for Seven Societies
Downe S, 2007, J ADV NURS, V57, P127, DOI 10.1111/j.1365-2648.2006.04079.x
Dunham B, 2016, HUM NATURE-INT BIOS, V27, P471, DOI 10.1007/s12110-016-9266-7

Gettlert LT, 2011, AM J PHYS ANTHROPOL, V144, P454, DOI 10.1002/ajpa.21426
Gupta Janesh K, 2017, Cochrane Database Syst Rev, V5, pCD002006, DOI 10.1002/14651858.CD002006.pub4
Haraway D., 1991, Simians, Cyborgs and Women: The Reinvention of Nature, DOI DOI 10.2307/3178066
Hauck FR, 2011, PEDIATRICS, V128, P103, DOI 10.1542/peds.2010-3000
Hodnett ED, 2013, COCHRANE DB SYST REV, P1, DOI [10.1002/14651858.CD003766.pub4, 10.1002/14651858.CD003766.pub6, 10.1002/14651858.CD003766.pub2, 10.1002/14651858.CD003766.pub3, 10.1002/14651858.CD003766.pub5]
Hrdy S.B., 2017, Hunter-gatherer childhoods, P65, DOI [DOI 10.4324/9780203789445-4, 10.4324/9780203789445-4, DOI 10.4324/9780203789445-4/COMES-CHILDMAN-SARAH-BLAFFER-HRDY, 10.4324/9780203789445-4/comes-childman-sarah-blaffer-hrdy]
Hrdy SB, 2016, HORM BEHAV, V77, P272, DOI 10.1016/j.yhbeh.2015.10.016
Hrdy SarahBlaffer., 1999, Mother Nature: Maternal Instincts and How They Shape the Human Species
Huber U., 2006, MIDRS Midwifery Journal Digest, V16, P445
Jolly A., 1999, LUCYS LEGACY SEX INT, V1st
Jones L, 2012, COCHRANE DB SYST REV, DOI 10.1002/14651858.CD009234.pub2
Lawrence A, 2013, COCHRANE DB SYST REV
Ludington-Hoe S., 1991, BIENN M SOC RES CHIL
Ludington-Hoe S M, 1991, J Perinatol, V11, P19
McKenna J., 2001, SUDDEN INFANT DEATH, P258
McKenna J., 2003, MANNER BORN BIRTH RI, P63
Mckenna J., 2020, SAFE INFANT SLEEP EX
McKenna James J, 2005, Paediatr Respir Rev, V6, P134, DOI 10.1016/j.prrv.2005.03.006
McKenna JJ, 2016, ACTA PAEDIATR, V105, P17, DOI 10.1111/apa.13161
Miller S, 2016, LANCET, V388, P2176, DOI 10.1016/S0140-6736(16)31472-6
Mobbs EJ, 2016, ACTA PAEDIATR, V105, P24, DOI 10.1111/apa.13034
Montague A., 1971, Touching: The human significance of the skin
Moore ER, 2016, COCHRANE DB SYST REV, DOI 10.1002/14651858.CD003519.pub4
Odent Michel., 2007, Birth and Breastfeeding: Rediscovering the Needs of Women during Pregnancy and Childbirth
Olsen O, 2012, COCHRANE DB SYST REV, DOI 10.1002/14651858.CD000352.pub2
Priddis H, 2012, WOMEN BIRTH, V25, P100, DOI 10.1016/j.wombi.2011.05.001
Renfrew MJ, 2012, COCHRANE DB SYST REV, DOI [10.1002/14651858.CD001141.pub5, 10.1002/14651858.CD001141.pub4]
Reynolds Peter C., 1991, Stealing Fire: The Atomic Bomb as Symbolic Body
Rosenberg Karen, 1995, Evolutionary Anthropology, V4, P161, DOI 10.1002/evan.1360040506
Rosenberg KR, 2018, EVOL MED PUBLIC HLTH, P67, DOI 10.1093/emph/eoy006
Sandall J, 2013, COCHRANE DB SYST REV, DOI [10.1002/14651858.CD004667.pub5, 10.1002/14651858.CD004667.pub3]
Singata M, 2013, COCHRANE DB SYST REV, DOI 10.1002/14651858.CD003930.pub3

   Small M.F., 2001, Kids : How biology and culture shape the way we raise our children
   Small M.F., 1999, Our babies, ourselves: How biology and culture shape the way we parent
   Sng BL, 2014, COCHRANE DB SYST REV, DOI 10.1002/14651858.CD007238.pub2
   Sosa G, 2012, MIDWIFERY, V28, P451, DOI 10.1016/j.midw.2011.07.001
   Stapleton SR, 2013, J MIDWIFERY WOM HEAL, V58, P3, DOI 10.1111/jmwh.12003
   Sutton J., 1996, UNDERSTANDING TEACHI
   Trevathan W., 2003, CHILDRENS ENV, V11, P88
   Trevathan W.R., 2017, Human birth: An evolutionary perspective
   Trevathan W, 2015, PHILOS T R SOC B, V370, DOI 10.1098/rstb.2014.0065
   Trevathan WR, 1999, EVOLUTIONARY MEDICI NE, P183
   Wang ZH, 2012, J MED HYPOTHESES IDE, V6, P28, DOI 10.1016/j.jmhi.2012.03.013
   World Health Organization, 2020, WHO REC INF FEED
   Zhang HY, 2017, MIDWIFERY, V50, P117, DOI 10.1016/j.midw.2017.03.022
   Zielinski R, 2015, INT J WOMENS HEALTH, V7, P361, DOI 10.2147/IJWH.S55561
NR 69
TC 4
Z9 4
U1 0
U2 20
PU SPRINGER PUBLISHING CO
PI NEW YORK
PA 11 WEST 42ND STREET, NEW YORK, NY 10036 USA
SN 2156-5287
EI 2156-5295
J9 INT J CHILDBIRTH
JI Int. J. Childbirth
PY 2020
VL 10
IS 2
BP 66
EP 78
DI 10.1891/IJCBIRTH-D-19-00029
PG 13
WC Nursing
WE Emerging Sources Citation Index (ESCI)
SC Nursing
GA OH4YH
UT WOS:000582582400001
DA 2025-05-07
ER

PT J
AU Alcock, J
   Masters, A
AF Alcock, Joe
   Masters, Alix
TI Cytokine storms, evolution and COVID-19
SO EVOLUTION MEDICINE AND PUBLIC HEALTH
LA English

DT Review
DE SARS-CoV-2; Covid-19; cytokine storm; corticosteroids; immunity
ID RESPIRATORY-DISTRESS-SYNDROME; SEPTIC SHOCK; SEVERE SEPSIS;
HEALTHY-CHILDREN; DENDRITIC CELLS; INTERFERON; MORTALITY; DISEASE;
AGE;
CORTICOSTEROIDS
AB Since the identification of severe illness caused by the novel coronavirus SARS-CoV-2, the role of the host immune system in causing disease has attracted widespread attention, along with intense interest in medical interventions that target the host immune response. A wide variety of agents have been proposed to treat a cytokine storm in coronavirus disease 2019 (COVID-19), but so far, only one class of medications, corticosteroids, has proved useful. In recent decades, experimental therapies for cytokine storms have been tried and mostly failed to help patients with severe sepsis and other infections. We summarize this history in order to frame expectations for novel interventions in COVID-19 and to bring an evolutionary medicine perspective to the concept of cytokine storms and their treatment.

Lay Summary: Many treatments for COVID-19 are aimed at calming a cytokine storm, a dangerous immune overreaction to the infection. Treating cytokine storms has been tried for decades in sepsis and other viral illnesses, but these treatments most often do not work. We explain why cytokine storms should be rare, and what special evolutionary circumstances can cause them to occur.
C1 [Alcock, Joe] Univ New Mexico, Dept Emergency Med, MSC11 6025 1, Albuquerque, NM 87131 USA.
[Masters, Alix] Thomas Jefferson Univ, Sidney Kimmel Med Coll, Philadelphia, PA 19107 USA.
C3 University of New Mexico; Thomas Jefferson University
RP Alcock, J (autor correspondente), Univ New Mexico, Dept Emergency Med, MSC11 6025 1, Albuquerque, NM 87131 USA.
EM joalcock@salud.unm.edu
OI Alcock, Joe/0000-0003-0807-164X
CR Agrawal A, 2013, GERONTOLOGY, V59, P421, DOI 10.1159/000350536
Alcock J, 2018, J CLIN MED, V7, DOI 10.3390/jcm7090247
Beigel JH, 2020, NEW ENGL J MED, V383, P1813, DOI [10.1056/NEJMoa2007764, 10.1056/NEJMc2022236]
BERNARD GR, 1987, NEW ENGL J MED, V317, P1565, DOI 10.1056/NEJM198712173172504
Boor PPC, 2017, TRANSL RES, V188, P67, DOI 10.1016/j.trsl.2016.11.006
Brenner EJ, 2020, GASTROENTEROLOGY, V159, P481, DOI 10.1053/j.gastro.2020.05.032
Brun-Buisson C, 2011, AM J RESP CRIT CARE, V183, P1200, DOI 10.1164/rccm.201101-0135OC
Channappanavar R, 2016, CELL HOST MICROBE, V19, P181, DOI 10.1016/j.chom.2016.01.007
Chen NS, 2020, LANCET, V395, P507, DOI 10.1016/S0140-6736(20)30211-7
Colavita F, 2019, VIRUSES-BASEL, V11, DOI 10.3390/v11040373
Crespi B, 2020, EVOL MED PUBLIC HLTH, P314, DOI 10.1093/emph/eoaa036
Cruz AR, 2012, PLOS NEGLECT TROP D, V6, DOI 10.1371/journal.pntd.0001717
DAMAS P, 1992, ANN SURG, V215, P356, DOI 10.1097/00000658-199204000-00009

DASILVA AMT, 1993, NEW ENGL J MED, V328, P1457, DOI 10.1056/NEJM199305203282005
Davies NG, 2020, NAT MED, V26, P1205, DOI 10.1038/s41591-020-0962-9
Decker ML, 2017, SCI REP-UK, V7, DOI 10.1038/s41598-017-17865-2
Dellinger RP, 2018, JAMA-J AM MED ASSOC, V320, P1455, DOI 10.1001/jama.2018.14618
Draulans Dirk., 2020, SCIENCE, DOI [10.1126/science.abc7042, DOI 10.1126/SCIENCE.ABC7042]
Fajnzylber J, 2020, NAT COMMUN, V11, DOI 10.1038/s41467-020-19057-5
Feldmann M, 2020, LANCET, V395, P1407, DOI 10.1016/S0140-6736(20)30858-8
Feldstein LR, 2020, NEW ENGL J MED, V383, P334, DOI 10.1056/NEJMoa2021680
Fisher CJ, 1996, NEW ENGL J MED, V334, P1697, DOI 10.1056/NEJM199606273342603
Geretti AM, 2021, CLIN INFECT DIS, V73, pE2095, DOI 10.1093/cid/ciaa1605
Godfred-Cato S, 2020, MMWR-MORBID MORTAL W, V69, P1074, DOI 10.15585/mmwr.mm6932e2
Griffin GE, 1998, J ANTIMICROB CHEMOTH, V41, P25, DOI 10.1093/jac/41.suppl_1.25
Guerra-Silveira F, 2013, PLOS ONE, V8, DOI 10.1371/journal.pone.0062390
Gupta RS, 2011, PEDIATRICS, V128, pE9, DOI 10.1542/peds.2011-0204
Hobbs CV, 2020, FRONT PEDIATR, V8, DOI 10.3389/fped.2020.593455
Hoffmann F, 2005, EUR CYTOKINE NETW, V16, P283
Horby P, 2021, NEW ENGL J MED, V384, P693, DOI [10.1056/NEJMoa2022926, 10.1056/NEJMoa2021436]
Hui DS, 2018, ANTIVIR RES, V150, P202, DOI 10.1016/j.antiviral.2018.01.002
Koella JC, 2007, Evolution in health and disease, P153
Konno Y, 2020, CELL REP, V32, DOI 10.1016/j.celrep.2020.108185
Kruger DJ, 2006, HUM NATURE-INT BIOS, V17, P74, DOI 10.1007/s12110-006-1021-z
La Gruta NL, 2007, IMMUNOL CELL BIOL, V85, P85, DOI 10.1038/sj.icb.7100026
LeGrand EK, 2012, Q REV BIOL, V87, P3, DOI 10.1086/663946
Li CJ, 2011, J VIROL, V85, P10955, DOI 10.1128/JVI.05792-11
Little TJ, 2010, PLOS PATHOG, V6, DOI 10.1371/journal.ppat.1001006
Liu BW, 2020, J AUTOIMMUN, V111, DOI 10.1016/j.jaut.2020.102452
Lochmiller RL, 2000, OIKOS, V88, P87, DOI 10.1034/j.1600-0706.2000.880110.x
Lowe D., 2020, PIPELINE
Lu XX, 2020, NEW ENGL J MED, V382, P1663, DOI 10.1056/NEJMc2005073
Marshall JC, 2019, JAMA-J AM MED ASSOC, V321, P903, DOI 10.1001/jama.2018.20882
McDade TW, 2003, YEARB PHYS ANTHROPOL, V46, P100, DOI 10.1002/ajpa.10398
Meduri GU, 2007, CHEST, V131, P954, DOI 10.1378/chest.06-2100
Mehta P, 2020, LANCET, V395, P1033, DOI 10.1016/S0140-6736(20)30628-0
Monk PD, 2021, LANCET RESP MED, V9, P196, DOI 10.1016/S2213-2600(20)30511-7
Mossman KL, 2005, VIRAL IMMUNOL, V18, P267, DOI 10.1089/vim.2005.18.267

Muehlenbein MP, 2005, AM J HUM BIOL, V17, P527, DOI 10.1002/ajhb.20419
Nakra NA, 2020, CHILDREN-BASEL, V7, DOI 10.3390/children7070069
Nesse RM, 2019, PHILOS T R SOC B, V374, DOI 10.1098/rstb.2019.0288
Nesse RM, 2001, ANN NY ACAD SCI, V935, P75
Nickbakhsh S, 2020, J INFECT DIS, V222, P17, DOI 10.1093/infdis/jiaa185
Opal SM, 2013, JAMA-J AM MED ASSOC, V309, P1154, DOI 10.1001/jama.2013.2194
Parr JB, 2021, JAMA INTERN MED, V181, P12, DOI 10.1001/jamainternmed.2020.6557
Peiris JSM, 2010, CURR OPIN IMMUNOL, V22, P475, DOI 10.1016/j.coi.2010.06.003
Reinhart K, 2001, CRIT CARE MED, V29, P765, DOI 10.1097/00003246-200104000-00015
Remy KE, 2020, LANCET RESP MED, V8, P946, DOI 10.1016/S2213-2600(20)30217-4
Ritchie AI, 2020, LANCET, V395, P1111, DOI 10.1016/S0140-6736(20)30691-7
Roche HL, ROCHE PROVIDES UPDAT
Roghmann MC, 2001, AM J MED SCI, V322, P68, DOI 10.1097/00000441-200108000-00003
Russell B, 2020, ECANCERMEDICALSCIENC, V14, P1, DOI 10.3332/ecancer.2020.1022
Russo R., 2020, GENETIC ANAL NOVEL S, DOI 10.1101/2020.04.23.057190
Rutledge GA, 2020, PLOS ONE, V15, DOI 10.1371/journal.pone.0240132
Rygård SL, 2018, INTENS CARE MED, V44, P1003, DOI 10.1007/s00134-018-5197-6
Schneider DS, 2008, NAT REV IMMUNOL, V8, P889, DOI 10.1038/nri2432
Schoggins JW, 2011, CURR OPIN VIROL, V1, P519, DOI 10.1016/j.coviro.2011.10.008
Schultze A, 2020, LANCET RESP MED, V8, P1106, DOI 10.1016/S2213-2600(20)30415-X
Senda T, 2019, MUCOSAL IMMUNOL, V12, P378, DOI 10.1038/s41385-018-0110-8
Siordia JA Jr, 2020, J CLIN VIROL, V127, DOI 10.1016/j.jcv.2020.104357
Strand V, 2015, ARTHRITIS RES THER, V17, DOI 10.1186/s13075-015-0880-2
Taneja V, 2018, FRONT IMMUNOL, V9, DOI 10.3389/fimmu.2018.01931
Taylor SL, 2018, TRENDS MICROBIOL, V26, P92, DOI 10.1016/j.tim.2017.09.011
Teijaro JR, 2011, CELL, V146, P980, DOI 10.1016/j.cell.2011.08.015
The Editors, EVOL MED REV
Tisoncik JR, 2012, MICROBIOL MOL BIOL R, V76, P16, DOI 10.1128/MMBR.05015-11
Tsagarakis NJ, 2018, MEDICINE, V97, DOI [10.1097/MD.0000000000010903, 10.1097/md.0000000000010903]
Turke PW, 1997, IMMUNOL TODAY, V18, P407, DOI 10.1016/S0167-5699(97)81056-0
VANVALEN L, 1977, AM NAT, V111, P809, DOI 10.1086/283213
Venkatesh B, 2018, NEW ENGL J MED, V378, P797, DOI 10.1056/NEJMoa1705835
Villar J, 2016, TRIALS, V17, DOI 10.1186/s13063-016-1456-4
Von Haam E, 1942, J IMMUNOL, V43, P109
Wilson JG, 2020, JCI INSIGHT, V5, DOI 10.1172/jci.insight.140289
Zhang ZH, 2015, SCI REP-UK, V5, DOI 10.1038/srep17654

   Zhou P, 2016, P NATL ACAD SCI USA, V113, P2696, DOI
10.1073/pnas.1518240113
   Zhou Q, 2020, FRONT IMMUNOL, V11, DOI 10.3389/fimmu.2020.01061
   Zhu XJ, 2020, VIRUS RES, V285, DOI 10.1016/j.virusres.2020.198005
   Zuk M, 2009, PLOS PATHOG, V5, DOI 10.1371/journal.ppat.1000267
NR 88
TC 11
Z9 12
U1 2
U2 36
PU OXFORD UNIV PRESS
PI OXFORD
PA GREAT CLARENDON ST, OXFORD OX2 6DP, ENGLAND
EI 2050-6201
J9 EVOL MED PUBLIC HLTH
JI Evol. Med. Public Health
PY 2021
VL 9
IS 1
BP 83
EP 92
DI 10.1093/emph/eoab005
EA FEB 2021
PG 10
WC Evolutionary Biology; Public, Environmental & Occupational Health
WE Science Citation Index Expanded (SCI-EXPANDED)
SC Evolutionary Biology; Public, Environmental & Occupational Health
GA QR3OF
UT WOS:000625121100001
PM 34552755
OA gold, Green Submitted, Green Published
DA 2025-05-07
ER

PT J
AU Roberts, CA
AF Roberts, Charlotte A.
TI Old World tuberculosis: Evidence from human remains with a review of
   current research and future prospects
SO TUBERCULOSIS
LA English
DT Article
DE Europe; Diagnosis; Limitations; aDNA analysis; Diet; Mobility; Vitamin
   D; Agriculture; Urbanism
ID MYCOBACTERIUM-TUBERCULOSIS; DIET
AB The evidence for TB in archaeological human remains for the Old World
is reviewed in published and some unpublished sources. The evidence of
Pott's disease was considered specific for TB, with other bone changes,
such as rib lesions, as non-specific. Limitations of the data are
discussed. Most evidence for TB comes from skeletons from the northern
hemisphere, particularly in Europe in the late Medieval period (12th-16th
centuries AD), but there is early evidence in the Near/Middle East and
Egypt. Many parts of Africa, Asia and Australasia have very little or no
evidence. aDNA analysis has provided data on species and strains of the

Mycobacterium tuberculosis complex organisms affecting people in the past. The extant data suggest the first epidemiological transition (Neolithic agriculture and permanent settlements) led to an increase in TB, with later increases in urban environments of the late Medieval period. A number of causative factors were at play. Future research, particularly using biomolecular analysis, has the potential to further contribute to our understanding of the origin and evolution of TB, thus merging the disciplines of palaeopathology and evolutionary medicine. (C) 2015 Elsevier Ltd. All rights reserved.
C1 Univ Durham, Dept Archaeol, Durham DH1 3LE, England.
C3 Durham University
RP Roberts, CA (autor correspondente), Univ Durham, Dept Archaeol, S Rd, Durham DH1 3LE, England.
EM c.a.roberts@durham.ac.uk
RI Roberts, Charlotte Ann/H-2622-2019
OI Roberts, Charlotte Ann/0000-0002-2259-807X
FU NERC [NE/E018564/1, NE/E015697/1] Funding Source: UKRI
CR Baker MA, 2012, J INFECT DIS, V205, P1043, DOI 10.1093/infdis/jis026
Bouwman AS, 2012, P NATL ACAD SCI USA, V109, P18511, DOI 10.1073/pnas.1209444109
Buikstra JE., 2012, The Global History of Paleopathology
Di Angelantonio E, 2019, LANCET GLOB HEALTH, V7, pE1332, DOI 10.1016/S2214-109X(19)30318-3
Fletcher HA, 2003, AM J PHYS ANTHROPOL, V120, P144, DOI 10.1002/ajpa.10114
Groenheit R, 2012, PLOS ONE, V7, DOI 10.1371/journal.pone.0046848
Hershkovitz I, 2008, PLOS ONE, V3, DOI 10.1371/journal.pone.0003426
Hewison M, 2012, P NUTR SOC, V71, P50, DOI 10.1017/S0029665111001650
Hu P, 2011, MOL BIOL REP, V38, P489, DOI 10.1007/s11033-010-0132-0
Khan R, 2010, MED HYPOTHESES, V75, P363, DOI 10.1016/j.mehy.2010.03.033
Kohler K., 2012, International Journal of Osteoarchaeology
MacLachlan JH, 2012, EMERG INFECT DIS, V18, P1879, DOI 10.3201/eid1811.120456
Morse D., 1967, DIS ANTIQUITY, P249
Müldner G, 2011, J ARCHAEOL SCI, V38, P280, DOI 10.1016/j.jas.2010.09.003
Nicklisch N, 2012, AM J PHYS ANTHROPOL, V149, P391, DOI 10.1002/ajpa.22137
Ortner DonaldJ., 2003, IDENTIFICATION PATHO
Oxlade O, 2012, PLOS ONE, V7, DOI 10.1371/journal.pone.0047533
Public Health England, 2013, TUB UK 2013 REP
Ricks PM, 2011, PLOS ONE, V6, DOI 10.1371/journal.pone.0027405
Roberts C., 2003, HLTH DIS BRITAIN PRE
Roberts CA., 2003, BIOARCHAEOLOGY TUBER
Roberts C, 2012, BLACKW COMPANION ANT, P434
Smith F.B., 1988, RETREAT TUBERCULOSIS
Stone AC, 2009, YEARB PHYS ANTHROPOL, V52, P66, DOI 10.1002/ajpa.21185
Sumpter C, 2013, TROP MED INT HEALTH, V18, P101, DOI 10.1111/tmi.12013
Thye T, 2012, NAT GENET, V44, P257, DOI 10.1038/ng.1080
Wilbur AK, 2008, CURR ANTHROPOL, V49, P963, DOI 10.1086/592434
Wilbur AK, 2009, J ARCHAEOL SCI, V36, P1990, DOI 10.1016/j.jas.2009.05.020
WOOD JW, 1992, CURR ANTHROPOL, V33, P343, DOI 10.1086/204084

Zink AR, 2007, INT J OSTEOARCHAEOL, V17, P380, DOI 10.1002/oa.909
NR 30
TC 20
Z9 23
U1 2
U2 32
PU CHURCHILL LIVINGSTONE
PI EDINBURGH
PA JOURNAL PRODUCTION DEPT, ROBERT STEVENSON HOUSE, 1-3 BAXTERS PLACE,
LEITH WALK, EDINBURGH EH1 3AF, MIDLOTHIAN, SCOTLAND
SN 1472-9792
EI 1873-281X
J9 TUBERCULOSIS
JI Tuberculosis
PD JUN
PY 2015
VL 95
SU 1
BP S117
EP S121
DI 10.1016/j.tube.2015.02.018
PG 5
WC Immunology; Microbiology; Respiratory System
WE Science Citation Index Expanded (SCI-EXPANDED); Social Science
Citation Index (SSCI)
SC Immunology; Microbiology; Respiratory System
GA CM8ER
UT WOS:000357931300021
PM 25802030
OA Green Accepted
DA 2025-05-07
ER

PT J
AU Rossier, BC
AF Rossier, Bernard C.
TI Osmoregulation during Long-Term Fasting in Lungfish and Elephant Seal:
Old and New Lessons for the Nephrologist
SO NEPHRON
LA English
DT Review
DE Arginine vasopressin; Aldosterone; Kidney function; Osmoregulation;
Renin angiotensin system; Salt balance; Water balance
ID EPITHELIAL SODIUM-CHANNEL; AFRICAN LUNGFISH; CONCENTRATING ABILITY;
ALDOSTERONE; EXPRESSION; TRANSPORT; EVOLUTION; FISH
AB Vertebrates control the osmolality of their extra- and intracellular
compartments despite large variations in salt and water intake.
Aldosterone-dependent sodium reabsorption and vasopressin-dependent water
transport in the distal nephron and collecting duct play a critical role
in the final control of sodium and water balance. Long-term fasting (no
eating, no drinking) represents an osmotic challenge for survival.
Evolution has found very different solutions to meet this challenge. To
illustrate this point, I will discuss osmoregulation of a mammal
(elephant seal pup) and of a fish (lungfish) that are able to survive

long-term fasting for months or even years. Homer W. Smith taught us how informative comparative anatomy and physiology of the kidney could help physiologists and nephrologists to better understand how the kidney works. In recent years, comparative genomics, transcriptomics and proteomics across the tree of life have led to the emergence of a new discipline, evolutionary medicine. In the near future, physiologists and nephrologists will benefit from this new field of investigation, thanks to its potential for the identification of novel drug targets and therapies. (C) 2016 S. Karger AG, Basel
C1 [Rossier, Bernard C.] Univ Lausanne, Dept Pharmacol & Toxicol, Fac Biol & Med, Rue Bugnon 27, CH-1005 Lausanne, Switzerland.
C3 University of Lausanne
RP Rossier, BC (autor correspondente), Univ Lausanne, Dept Pharmacol & Toxicol, Fac Biol & Med, Rue Bugnon 27, CH-1005 Lausanne, Switzerland.
EM Bernard.Rossier@unil.ch
CR Abegglen LM, 2015, JAMA-J AM MED ASSOC, V314, P1850, DOI 10.1001/jama.2015.13134
Al-Awqati Q, 2006, KIDNEY INT, V70, P2, DOI 10.1038/sj.ki.5001687
BANKIR L, 1985, AM J PHYSIOL, V249, pR643, DOI 10.1152/ajpregu.1985.249.6.R643
BEUCHAT CA, 1990, AM J PHYSIOL, V258, pR298, DOI 10.1152/ajpregu.1990.258.2.R298
de Seigneux S, 2007, AM J PHYSIOL-RENAL, V293, pF87, DOI 10.1152/ajprenal.00431.2006
Fournier D, 2012, J MOL MED, V90, P495, DOI 10.1007/s00109-012-0894-z
Hembree DI, 2010, PROG MOL SUBCELL BIO, V49, P245, DOI 10.1007/978-3-642-02421-4_12
JACOB F, 1977, SCIENCE, V196, P1161, DOI 10.1126/science.860134
King HM, 2011, P NATL ACAD SCI USA, V108, P21146, DOI 10.1073/pnas.1118669109
Konno N, 2010, ENDOCRINOLOGY, V151, P1089, DOI 10.1210/en.2009-1070
Konno N, 2009, J EXP BIOL, V212, P2183, DOI 10.1242/jeb.029322
Kwon TH, 2002, AM J PHYSIOL-RENAL, V283, pF1403, DOI 10.1152/ajprenal.00059.2002
Masilamani S, 1999, J CLIN INVEST, V104, pR19, DOI 10.1172/JCI7840
Ortiz RM, 2001, J EXP BIOL, V204, P1831
REIF MC, 1986, J CLIN INVEST, V77, P1291, DOI 10.1172/JCI112433
Rossier BC, 2015, PHYSIOL REV, V95, P297, DOI 10.1152/physrev.00011.2014
Schiffman JD, 2015, PHILOS T R SOC B, V370, DOI 10.1098/rstb.2014.0231
Smith H.W., 1953, From Fish to Philosopher; the Story of Our Internal Environment
Smith HW, 1930, J BIOL CHEM, V88, P97
Uchiyama M, 2015, COMP BIOCHEM PHYS A, V183, P1, DOI 10.1016/j.cbpa.2014.12.028
Uchiyama M, 2012, P ROY SOC B-BIOL SCI, V279, P4795, DOI 10.1098/rspb.2012.1945
Zhu M, 2002, NATURE, V418, P767, DOI 10.1038/nature00871
NR 22
TC 9
Z9 9
U1 0
U2 24
PU KARGER

PI BASEL
PA ALLSCHWILERSTRASSE 10, CH-4009 BASEL, SWITZERLAND
SN 1660-8151
EI 2235-3186
J9 NEPHRON
JI Nephron
PY 2016
VL 134
IS 1
BP 5
EP 9
DI 10.1159/000444307
PG 5
WC Urology & Nephrology
WE Science Citation Index Expanded (SCI-EXPANDED)
SC Urology & Nephrology
GA DW1RT
UT WOS:000383421000002
PM 26901864
OA Bronze
DA 2025-05-07
ER

PT J
AU Douhard, M
   Plard, F
   Gaillard, JM
   Capron, G
   Delorme, D
   Klein, F
   Duncan, P
   Loe, LE
   Bonenfant, C
AF Douhard, Mathieu
   Plard, Floriane
   Gaillard, Jean-Michel
   Capron, Gilles
   Delorme, Daniel
   Klein, Francois
   Duncan, Patrick
   Loe, Leif Egil
   Bonenfant, Christophe
TI Fitness consequences of environmental conditions at different life
   stages in a long-lived vertebrate
SO PROCEEDINGS OF THE ROYAL SOCIETY B-BIOLOGICAL SCIENCES
LA English
DT Article
DE phenotypic plasticity; development; adaptation; silver spoon effect;
   long-term cohort effects
ID PREDICTIVE ADAPTIVE RESPONSE; ROE DEER; PHENOTYPIC PLASTICITY;
   BODY-MASS; DEVELOPMENTAL PLASTICITY; REPRODUCTIVE SUCCESS;
   POPULATION-DYNAMICS; SURVIVAL; GROWTH; DENSITY
AB The predictive adaptive response (PAR) hypothesis proposes that
animals adjust their physiology and developmental trajectory during early

life in anticipation of their future environments. Accordingly, when environmental conditions in early life match environmental conditions during adulthood, individual fitness should be greater. Here, we test this hypothesis in a long-lived mammal, the roe deer, using data from two contrasting populations, intensively monitored for more than 35 years. In the highly productive site, the fitness of female roe deer increased with the quality of environment during adulthood and, contrary to predictions of PAR, individuals born in good conditions always outperformed those born under poor conditions. In the resource-limited site, the fitness of female roe deer born in poor years was better than those born in good conditions in poor years when the animals were adult, but not in good years. Although consistent with predictions of PAR, we showed that this pattern is likely to be a consequence of increased viability selection during the juvenile stage for animals born in poor years. While PARs are often advanced in evolutionary medicine, our findings suggest that detailed biological processes should be investigated before drawing conclusions about the existence of this phenomenon.
C1 [Douhard, Mathieu; Plard, Floriane; Gaillard, Jean-Michel; Bonenfant, Christophe] Univ Lyon, F-69000 Lyon, France.
[Douhard, Mathieu; Plard, Floriane; Gaillard, Jean-Michel; Bonenfant, Christophe] Univ Lyon 1, F-69622 Villeurbanne, France.
[Douhard, Mathieu; Plard, Floriane; Gaillard, Jean-Michel; Bonenfant, Christophe] Univ Lyon 1, CNRS, UMR 5558, Lab Biometrie & Biol Evolut, F-69622 Villeurbanne, France.
[Douhard, Mathieu; Loe, Leif Egil] Norwegian Univ Life Sci, Dept Ecol & Nat Resource Management, N-1432 As, Norway.
[Capron, Gilles; Delorme, Daniel; Klein, Francois] CNERA Cervides Sangliers, Off Natl Chasse & Faune Sauvage, F-55000 Bar Le Duc, France.
[Duncan, Patrick] Univ Rochelle, CNRS, UMR 7372, Ctr Etudes Biol Chize, F-79360 Beauvoir Sur Niort, France.
C3 Universite Claude Bernard Lyon 1; Centre National de la Recherche Scientifique (CNRS); CNRS - Institute of Ecology & Environment (INEE); Universite Claude Bernard Lyon 1; VetAgro Sup; Norwegian University of Life Sciences; Centre National de la Recherche Scientifique (CNRS); CNRS
- Institute of Ecology & Environment (INEE)
RP Douhard, M (autor correspondente), Univ Lyon, F-69000 Lyon, France.
EM mathieu.douhard@gmail.com
RI Bonenfant, Christophe/I-8645-2019; Loe, Leif Egil/M-5798-2016
OI Loe, Leif Egil/0000-0003-4804-2253; Bonenfant, Christophe/0000-0002-9924-419X
FU French Ministry of Higher Education and Research; CNRS; ONCFS
FX M. Douhard and F. Plard were supported by PhD scholarships from the French Ministry of Higher Education and Research. C. Bonenfant and J.-M.
Gaillard are supported by research grants from CNRS and ONCFS.
CR Agresti A, 2002, CATEGORICAL DATA ANA, DOI DOI 10.1002/0471249688
ALBON SD, 1987, J ANIM ECOL, V56, P69, DOI 10.2307/4800
Andersen R, 2000, J ANIM ECOL, V69, P672, DOI 10.1046/j.1365-2656.2000.00425.x
Bateson P, 2004, NATURE, V430, P419, DOI 10.1038/nature02725
Beckerman AP, 2002, TRENDS ECOL EVOL, V17, P263, DOI 10.1016/S0169-5347(02)02469-2
Bunnell F.L., 1987, P145

Caswell Hal, 2001, pi
Choquet R, 2011, METHODS ECOL EVOL, V2, P383, DOI 10.1111/j.2041-210X.2011.00088.x
Choquet R, 2009, ENVIRON ECOL STAT SE, V3, P845, DOI 10.1007/978-0-387-78151-8_39
Christensen R.H., 2013, ordinal-regression models for ordinal data R package; version 2013.9-30
Cleal JK, 2007, P NATL ACAD SCI USA, V104, P9529, DOI 10.1073/pnas.0610373104
Clutton-Brock T, 2010, TRENDS ECOL EVOL, V25, P562, DOI 10.1016/j.tree.2010.08.002
Dantzer B, 2013, SCIENCE, V340, P1215, DOI 10.1126/science.1235765
Douhard M, 2013, ECOLOGY, V94, P1805, DOI 10.1890/13-0034.1
Festa-Bianchet M, 2004, BEHAV ECOL, V15, P305, DOI 10.1093/beheco/arh014
Fisher R., 1930, GENETICAL THEORY NAT
Gaillard J.-M., 2004, Animal Biodiversity and Conservation, V27, P47
Gaillard JM, 2000, ANNU REV ECOL SYST, V31, P367, DOI 10.1146/annurev.ecolsys.31.1.367
GAILLARD JM, 1992, CAN J ZOOL, V70, P1541, DOI 10.1139/z92-212
GAILLARD JM, 1993, OECOLOGIA, V94, P57, DOI 10.1007/BF00317301
Gaillard JM, 1997, OECOLOGIA, V112, P502, DOI 10.1007/s004420050338
Gaillard JM, 1998, ECOLOGY, V79, P2878, DOI 10.2307/176523
Gaillard JM, 2000, P ROY SOC B-BIOL SCI, V267, P471, DOI 10.1098/rspb.2000.1024
Gluckman PD, 2006, EUR J ENDOCRINOL, V155, pS47, DOI 10.1530/eje.1.02233
Gluckman PD, 2005, TRENDS ECOL EVOL, V20, P527, DOI 10.1016/j.tree.2005.08.001
Gluckman PD, 2005, P ROY SOC B-BIOL SCI, V272, P671, DOI 10.1098/rspb.2004.3001
Gluckman PD, 2004, SCIENCE, V305, P1733, DOI 10.1126/science.1095292
Grafen A., 1988, reproductive Success, P454, DOI DOI 10.1098/RSTB.2007.0011
HALES CN, 1992, DIABETOLOGIA, V35, P595, DOI 10.1093/ije/dyt133
Hamel S, 2009, J ANIM ECOL, V78, P143, DOI 10.1111/j.1365-2656.2008.01459.x
Hamel S, 2009, ECOLOGY, V90, P1981, DOI 10.1890/08-0596.1
Hayward Adam D., 2013, Evolution Medicine and Public Health, P106, DOI 10.1093/emph/eot007
Hayward AD, 2013, P NATL ACAD SCI USA, V110, P13886, DOI 10.1073/pnas.1301817110
Hertwig O., 1900, BIOL PROBLEM TODAY P
Hewison AJM, 2002, J ZOOL, V257, P303, DOI 10.1017/S0952836902000900
Jonsson KI, 1997, OIKOS, V78, P57, DOI 10.2307/3545800
Kuzawa CW, 2005, AM J HUM BIOL, V17, P5, DOI 10.1002/ajhb.20091
LEBRETON JD, 1992, ECOL MONOGR, V62, P67, DOI 10.2307/2937171
LEE TM, 1988, AM J PHYSIOL, V255, pR831, DOI 10.1152/ajpregu.1988.255.5.R831
Lindström J, 1999, TRENDS ECOL EVOL, V14, P343, DOI 10.1016/S0169-5347(99)01639-0
Linnell JDC, 1998, EUROPEAN ROE DEER: THE BIOLOGY OF SUCCESS, P11
Loison A, 1999, ECOLOGY, V80, P2539, DOI 10.2307/177239
LUCAS A, 1991, CIBA F SYMP, V156, P38

Lummaa V, 2002, TRENDS ECOL EVOL, V17, P141, DOI 10.1016/S0169-5347(01)02414-4
Madsen T, 2000, J ANIM ECOL, V69, P952, DOI 10.1046/j.1365-2656.2000.00477.x
Metcalfe NB, 2001, TRENDS ECOL EVOL, V16, P254, DOI 10.1016/S0169-5347(01)02124-3
Monaghan P, 2008, PHILOS T R SOC B, V363, P1635, DOI 10.1098/rstb.2007.0011
Nettle D, 2013, P ROY SOC B-BIOL SCI, V280, DOI 10.1098/rspb.2013.1343
Nussey DH, 2007, J EVOLUTION BIOL, V20, P831, DOI 10.1111/j.1420-9101.2007.01300.x
Pettorelli N, 2006, OIKOS, V112, P565, DOI 10.1111/j.0030-1299.2006.14447.x
Pettorelli N, 2003, ECOLOGY, V84, P3307, DOI 10.1890/02-0602
Pettorelli N, 2003, OECOLOGIA, V137, P363, DOI 10.1007/s00442-003-1364-7
Pigliucci M, 2006, J EXP BIOL, V209, P2362, DOI 10.1242/jeb.02070
Rickard IJ, 2007, TRENDS ENDOCRIN MET, V18, P94, DOI 10.1016/j.tem.2007.02.004
Sadleir R.M.F.S., 1987, P123
Stearns SC, 2012, P ROY SOC B-BIOL SCI, V279, P4305, DOI 10.1098/rspb.2012.1326
Toïgo C, 2006, ECOGRAPHY, V29, P301, DOI 10.1111/j.2006.0906-7590.04394.x
van den Heuvel J, 2013, AM NAT, V181, pE28, DOI 10.1086/668818
Vanpé C, 2009, J MAMMAL, V90, P661, DOI 10.1644/08-MAMM-A-137R.1
Warner DA, 2008, J ANIM ECOL, V77, P1242, DOI 10.1111/j.1365-2656.2008.01442.x
Wells JCK, 2007, TRENDS ENDOCRIN MET, V18, P331, DOI 10.1016/j.tem.2007.07.006
Wells JCK, 2006, TRENDS ECOL EVOL, V21, P424, DOI 10.1016/j.tree.2006.05.006
WESTEBERHARD MJ, 1989, ANNU REV ECOL SYST, V20, P249, DOI 10.1146/annurev.es.20.110189.001341
WILLIAMS GC, 1991, Q REV BIOL, V66, P1, DOI 10.1086/417048
NR 64
TC 79
Z9 84
U1 0
U2 89
PU ROYAL SOC
PI LONDON
PA 6-9 CARLTON HOUSE TERRACE, LONDON SW1Y 5AG, ENGLAND
SN 0962-8452
EI 1471-2954
J9 P ROY SOC B-BIOL SCI
JI Proc. R. Soc. B-Biol. Sci.
PD JUN 22
PY 2014
VL 281
IS 1785
AR 20140276
DI 10.1098/rspb.2014.0276
PG 8

WC Biology; Ecology; Evolutionary Biology
WE Science Citation Index Expanded (SCI-EXPANDED)
SC Life Sciences & Biomedicine - Other Topics; Environmental Sciences & Ecology; Evolutionary Biology
GA AH0LT
UT WOS:000335812100015
PM 24789898
OA Bronze, Green Published
DA 2025-05-07
ER

PT J
AU Eggleson, KK
AF Eggleson, Kathleen K.
TI STEM CELL-BASED THERAPIES promises, obstacles, discordance, and the agora
SO PERSPECTIVES IN BIOLOGY AND MEDICINE
LA English
DT Article
ID SPINAL-CORD-INJURY; INSULIN-PRODUCING CELLS; TYPE-1 DIABETES-MELLITUS; EVOLUTIONARY HEALTH PROMOTION; BONE-MARROW-CELLS; IN-VIVO; MYOCARDIAL-INFARCTION; ISLET TRANSPLANTATION; SECRETING CELLS; IPS CELLS
AB Stem cell research has entered the public consciousness through the media. Proponents and opponents of all such research, or of human embryonic stem cell research specifically, engage in heated exchanges in the modern public forum where stakeholders negotiate, the agora. One common claim that emerges from the fray is that a particular type of stem cell research should be pursued as the most promising path toward the reduction of suffering and untimely death for all of humanity. Upon evaluation, experimental data regarding the potential role of stem cells in regenerative therapies for three conditions spinal cord injury, type 1 diabetes, and cardiovascular disease tell distinct, complex, and inconclusive stories. Further analyses in this article incorporate realistic considerations of a broad range of relevant factors: limited funding for biomedical research, media motives, the discordance hypothesis of evolutionary medicine, the relationship between religion and science, medical care in developing nations, and culture wars over abortion. Holistic investigation inspired by the current agora conversation supports the need to drastically change interactions regarding stem cell research so that its potential to benefit humanity may be more fully realized.
C1 Univ Notre Dame, Ctr Nano Sci & Technol, Notre Dame, IN 46556 USA.
C3 University of Notre Dame
RP Eggleson, KK (autor correspondente), Univ Notre Dame, Ctr Nano Sci & Technol, 275 Fitzpatrick Hall, Notre Dame, IN 46556 USA.
EM keggleso@nd.edu
CR Adeyi O, 2007, DIR DEV, P1, DOI 10.1596/978-0-8213-7044-5
Almad A, 2011, NEUROTHERAPEUTICS, V8, P262, DOI 10.1007/s13311-011-0033-5
Alsaleh FM, 2012, J CLIN PHARM THER, V37, P140, DOI 10.1111/j.1365-2710.2011.01283.x
Amariglio N, 2009, PLOS MED, V6, P221, DOI 10.1371/journal.pmed.1000029

[Anonymous], 2011, National Prevention Strategy
[Anonymous], 2010, National Health and Nutrition Examination Survey
[Anonymous], 2011, Health, United States, 2010: With special feature on death and dying
Arboleda D, 2011, CELL MOL NEUROBIOL, V31, P1113, DOI 10.1007/s10571-011-9712-3
Aviv V, 2009, J BIOL CHEM, V284, P33509, DOI 10.1074/jbc.M109.017608
Azzi J, 2010, EXPERT REV CLIN IMMU, V6, P111, DOI 10.1586/ECI.09.67
Balsam LB, 2004, NATURE, V428, P668, DOI 10.1038/nature02460
Bergmann O, 2009, SCIENCE, V324, P98, DOI 10.1126/science.1164680
Berman DM, 2010, DIABETES, V59, P2558, DOI 10.2337/db10-0136
Blakemore C., 2007, PUBLIC UNDERSTANDING
BRAUNWALD, 1997, J MOD, V337, P1360
British-North American Committee (BNAC), 2004, GUID BEN RESP OPP BN, VBN-47
Bromberg JS, 2007, AM J TRANSPLANT, V7, P2217, DOI 10.1111/j.1600-6143.2007.01957.x
Bryson C., 2004, FLUORIDE DECEPTION
Burt Richard K., 2002, Autoimmunity Reviews, V1, P133, DOI 10.1016/S1568-9972(02)00033-2
Campbell PM, 2007, AM J TRANSPLANT, V7, P2311, DOI 10.1111/j.1600-6143.2007.01923.x
Chandra V, 2009, STEM CELLS, V27, P1941, DOI 10.1002/stem.117
Chong AS, 2006, SCIENCE, V311, P1774, DOI 10.1126/science.1123510
Christoforou N, 2010, PLOS ONE, V5, DOI 10.1371/journal.pone.0011536
Cooper CB, 2011, BIOSCIENCE, V61, P231, DOI 10.1525/bio.2011.61.3.8
Couri CEB, 2009, JAMA-J AM MED ASSOC, V301, P1573, DOI 10.1001/jama.2009.470
Couzin-Frankel J, 2010, SCIENCE, V327, P772, DOI 10.1126/science.327.5967.772
Crowe MJ, 1997, NAT MED, V3, P73, DOI 10.1038/nm0197-73
Dean SK, 2006, TRANSPLANTATION, V82, P1175, DOI 10.1097/01.tp.0000239518.23354.64
Diabet Control Complications Trial Res Grp, 1998, ANN INTERN MED, V128, P517, DOI 10.7326/0003-4819-128-7-199804010-00001
Dieguez-Acuña F, 2010, INT J BIOCHEM CELL B, V42, P1651, DOI 10.1016/j.biocel.2009.12.001
Dimomeletis I, 2010, EXP HEMATOL, V38, P1105, DOI 10.1016/j.exphem.2010.06.013
DOBZHANSKY T, 1964, AM ZOOL, V4, P443
Doull J., 2006, Fluoride in drinking water: a scientific review of EPA's standards, Committee on Fluoride in Drinking Water, Board on Environmental Studies and Toxicology, Division on Earth and Life Sciences, National Research Council of the National Academies
Dressel R, 2010, FASEB J, V24, P2164, DOI 10.1096/fj.09-134957
Eaton SB, 2002, PREV MED, V34, P109, DOI 10.1006/pmed.2001.0876
Eaton SB, 2002, PREV MED, V34, P119, DOI 10.1006/pmed.2001.0966
Faustman DL, 2010, INT J BIOCHEM CELL B, V42, P1576, DOI 10.1016/j.biocel.2010.06.012
Fawcett JW, 2007, SPINAL CORD, V45, P190, DOI 10.1038/sj.sc.3102007
Fiorina P, 2009, J IMMUNOL, V183, P993, DOI 10.4049/jimmunol.0900803
Fitch MT, 2008, EXP NEUROL, V209, P294, DOI 10.1016/j.expneurol.2007.05.014

Ross LF, 2007, JAMA-J AM MED ASSOC, V298, P285, DOI 10.1001/jama.298.3.285-a
Gibbons M, 1999, NATURE, V402, pC81, DOI 10.1038/35011576
Greenwood HL, 2006, PLOS MED, V3, P1496, DOI 10.1371/journal.pmed.0030381
Guest JD, 2005, EXP NEUROL, V192, P384, DOI 10.1016/j.expneurol.2004.11.033
Hess D, 2003, NAT BIOTECHNOL, V21, P763, DOI 10.1038/nbt841
Hill CE, 2001, EXP NEUROL, V171, P153, DOI 10.1006/exnr.2001.7734
Hirschhorn Joel N, 2003, Pediatr Diabetes, V4, P87, DOI 10.1034/j.1399-5448.2001.00013.x
Hofstetter CP, 2005, NAT NEUROSCI, V8, P346, DOI 10.1038/nn1405
ISSCR, 2008, PAT HDB STEM CELL TH
Jurewicz M, 2010, DIABETES, V59, P3139, DOI 10.2337/db10-0542
Kakulas B A, 1999, J Spinal Cord Med, V22, P119
Kang HM, 2009, STEM CELLS, V27, P1999, DOI 10.1002/stem.127
Keirstead HS, 1997, J NEUROPATH EXP NEUR, V56, P1191, DOI 10.1097/00005072-199711000-00003
Kennedy Donald, 2006, Science, V311, P335
Kilkenny C, 2009, PLOS ONE, V4, DOI 10.1371/journal.pone.0007824
Kim K, 2010, NATURE, V467, P285, DOI 10.1038/nature09342
King M, 2008, ANN NY ACAD SCI, V1150, P46, DOI 10.1196/annals.1447.009
Kiuru M, 2009, CELL STEM CELL, V4, P289, DOI 10.1016/j.stem.2009.03.010
Knowles P., 2009, PSYCCRITIQUES, V54
Kodama S, 2003, SCIENCE, V302, P1223, DOI 10.1126/science.1088949
Konner M, 2001, WESTERN J MED, V174, P360, DOI 10.1136/ewjm.174.5.360
Krishna KA, 2007, REGEN MED, V2, P171, DOI 10.2217/17460751.2.2.171
Kroon E, 2008, NAT BIOTECHNOL, V26, P443, DOI 10.1038/nbt1393
Kwon BK, 2010, SPINE, V35, pS263, DOI 10.1097/BRS.0b013e3181f3286d
Laflamme MA, 2011, NATURE, V473, P326, DOI 10.1038/nature10147
Lechner A, 2004, DIABETES, V53, P616, DOI 10.2337/diabetes.53.3.616
Lee RH, 2006, P NATL ACAD SCI USA, V103, P17438, DOI 10.1073/pnas.0608249103
Lee Y. K., 2011, THERAPY, V8, P69
Li HY, 2010, J PHARMACOL EXP THER, V335, P817, DOI 10.1124/jpet.110.169284
Lonyai A, 2008, HORM METAB RES, V40, P137, DOI 10.1055/s-2007-1022560
Madec AM, 2009, DIABETOLOGIA, V52, P1391, DOI 10.1007/s00125-009-1374-z
Madonna R, 2011, J BIOTECHNOL, V154, P291, DOI 10.1016/j.jbiotec.2011.05.014
Maehr R, 2011, CLIN PHARMACOL THER, V89, P750, DOI 10.1038/clpt.2011.1
Manohar R, 2009, MOL THER, V17, P936, DOI 10.1038/mt.2009.93
Marbán E, 2007, CIRC RES, V100, P445, DOI 10.1161/01.RES.0000260271.33215.9b
Mathews SC, 2010, AM J MED QUAL, V25, P429, DOI 10.1177/1062860610369171
Mathiasen AB, 2009, FUTUR CARDIOL, V5, P605, DOI 10.2217/FCA.09.42
Milanetti F, 2010, PEDIATR CLIN N AM, V57, P239, DOI 10.1016/j.pcl.2009.12.003
Miura K, 2009, NAT BIOTECHNOL, V27, P743, DOI 10.1038/nbt.1554
Mooney C., 2011, MX JONES MAY

Moretto-Zita M, 2010, P NATL ACAD SCI USA, V107, P13312, DOI 10.1073/pnas.1005847107
Mozid AM, 2011, BRIT MED BULL, V98, P143, DOI 10.1093/bmb/ldr014
Müller-Ehmsen J, 2006, J MOL CELL CARDIOL, V41, P876, DOI 10.1016/j.yjmcc.2006.07.023
Murry CE, 2004, NATURE, V428, P664, DOI 10.1038/nature02446
Nadal-Ginard B, 2003, CIRC RES, V92, P139, DOI 10.1161/01.RES.0000053618.86362.DF
Nagaya M, 2009, J ENDOCRINOL, V201, P37, DOI 10.1677/JOE-08-0482
National Institutes of Health (NIH), 2011, NIH REPORT RES PORTF
National Institutes of Health (NIH), 2011, REP GRANTS APPL AW S
Naujok O, 2009, DIABETES-METAB RES, V25, P464, DOI 10.1002/dmrr.965
Neshati Z, 2010, J PHYSIOL BIOCHEM, V66, P181, DOI 10.1007/s13105-010-0013-y
Nesse RM, 1998, SCI AM, V279, P86, DOI 10.1038/scientificamerican1198-86
Nishio J, 2006, SCIENCE, V311, P1775, DOI 10.1126/science.1124004
Nygren JM, 2004, NAT MED, V10, P494, DOI 10.1038/nm1040
Ngoc PK, 2011, HUM CELL, V24, P86, DOI 10.1007/s13577-011-0018-z
Pinzon A, 2008, EXP NEUROL, V213, P129, DOI 10.1016/j.expneurol.2008.05.018
Pittenger MF, 2004, CIRC RES, V95, P9, DOI 10.1161/01.RES.0000135902.99383.6f
Regaldo A, 2011, TECHNOL REV JUL
Roger VL, 2011, CIRCULATION, V123, pE18, DOI 10.1161/CIR.0b013e3182009701
Rosengren AH, 2009, ISLETS, V1, P10, DOI 10.4161/isl.1.1.8529
Rowland JW, 2008, NEUROSURG FOCUS, V25, DOI 10.3171/FOC.2008.25.11.E2
Saketkhou BB, 1997, ANN INTERN MED, V127, P126, DOI 10.7326/0003-4819-127-2-199707150-00005
Schäfer MS, 2009, SCI COMMUN, V30, P475, DOI 10.1177/1075547008326943
Schuldiner M, 2003, STEM CELLS, V21, P257, DOI 10.1634/stemcells.21-3-257
Segers VFM, 2008, NATURE, V451, P937, DOI 10.1038/nature06800
Shao SY, 2011, J ENDOCRINOL, V208, P245, DOI 10.1530/JOE-10-0378
Shapiro AMJ, 2006, NEW ENGL J MED, V355, P1318, DOI 10.1056/NEJMoa061267
Sharp KG, 2012, EXP NEUROL, V233, P625, DOI 10.1016/j.expneurol.2010.12.020
Silver J, 2004, NAT REV NEUROSCI, V5, P146, DOI 10.1038/nrn1326
Snarski E, 2011, BONE MARROW TRANSPL, V46, P562, DOI 10.1038/bmt.2010.147
Sordi V, 2010, STEM CELLS, V28, P140, DOI 10.1002/stem.259
Stauffer K., 2010, Anatomy physiology for psychotherapists : Connecting body and soul, V1st
Steward O, 2006, EXP NEUROL, V198, P483, DOI 10.1016/j.expneurol.2005.12.034
Steward O, 2008, EXP NEUROL, V209, P446, DOI 10.1016/j.expneurol.2007.12.010
Taneera J, 2006, DIABETES, V55, P290, DOI 10.2337/diabetes.55.02.06.db05-1212
Thomas KE, 2011, BRIT MED BULL, V98, P127, DOI 10.1093/bmb/ldr013
Thonhoff JR, 2009, CURR STEM CELL RES T, V4, P178, DOI 10.2174/157488809789057392

Thuret S, 2006, NAT REV NEUROSCI, V7, P628, DOI 10.1038/nrn1955
Tsuji O, 2010, P NATL ACAD SCI USA, V107, P12704, DOI 10.1073/pnas.0910106107
Tzukerman M, 2003, P NATL ACAD SCI USA, V100, P13507, DOI 10.1073/pnas.2235551100
Urbanek K, 2005, CIRC RES, V97, P663, DOI 10.1161/01.RES.0000183733.53101.11
van der Bogt KEA, 2008, CIRCULATION, V118, pS121, DOI 10.1161/CIRCULATIONAHA.107.759480
Vardanyan M, 2010, CURR OPIN ORGAN TRAN, V15, P124, DOI 10.1097/MOT.0b013e32833553f8
Voltarelli JC, 2007, JAMA-J AM MED ASSOC, V297, P1568, DOI 10.1001/jama.297.14.1568
Voltarelli JC, 2008, ANN NY ACAD SCI, V1150, P220, DOI 10.1196/annals.1447.048
Wagner RT, 2010, TRANSL RES, V156, P169, DOI 10.1016/j.trsl.2010.06.005
Wainwright SP, 2006, SOC SCI MED, V63, P2052, DOI 10.1016/j.socscimed.2006.05.003
Wen YJ, 2011, EXPERT OPIN BIOL TH, V11, P41, DOI 10.1517/14712598.2011.540235
Yanovski SZ, 2011, NEW ENGL J MED, V364, P987, DOI 10.1056/NEJMp1009229
Yechoor V, 2009, ENDOCRINOLOGY, V150, P4863, DOI 10.1210/en.2009-0527
Yechoor V, 2009, DEV CELL, V16, P358, DOI 10.1016/j.devcel.2009.01.012
Yeghiazarians Y, 2009, MOL THER, V17, P1250, DOI 10.1038/mt.2009.85
Zalzman M, 2005, DIABETES, V54, P2568, DOI 10.2337/diabetes.54.9.2568
Zhong BN, 2011, MOL THER, V19, P1667, DOI 10.1038/mt.2011.51
NR 133
TC 10
Z9 13
U1 0
U2 31
PU JOHNS HOPKINS UNIV PRESS
PI BALTIMORE
PA JOURNALS PUBLISHING DIVISION, 2715 NORTH CHARLES ST, BALTIMORE, MD 21218-4363 USA
SN 0031-5982
EI 1529-8795
J9 PERSPECT BIOL MED
JI Perspect. Biol. Med.
PD WIN
PY 2012
VL 55
IS 1
BP 1
EP 25
PG 25
WC History & Philosophy Of Science; Medicine, Research & Experimental
WE Science Citation Index Expanded (SCI-EXPANDED)
SC History & Philosophy of Science; Research & Experimental Medicine
GA 903EB
UT WOS:000301097800001
PM 22643713

DA 2025-05-07
ER

PT B
AU van der Steen, WJ
AF van der Steen, Wim J.
BE Reydon, TAC
   Hemerik, L
TI Infectious Biology: Curse or Blessing? Reflections on Biology in Other
   Disciplines, with a Case Study of Migraine
SO CURRENT THEMES IN THEORETICAL BIOLOGY: A DUTCH PERSPECTIVE
LA English
DT Article; Book Chapter
DE aura; dietary deficiency; endothelium; evolutionary psychiatry;
   evolutionary psychology; evolutionary medicine; fatty acid; functional
   explanation; level of organization; migraine; platelet; serotonin;
   vasopressin
ID POLYUNSATURATED FATTY-ACIDS; EICOSAPENTAENOIC ACID; EVOLUTIONARY
   PSYCHOLOGY; DOCOSAHEXAENOIC ACID; FISH-OIL; VASOPRESSIN; ENDOTHELIN-1;
   MECHANISMS; MAGNESIUM; OMEGA-3-FATTY-ACIDS
AB Biology has come to play important roles in many other disciplines.
Some applications of evolutionary thought outside biology are
disappointing, but promising approaches are feasible in medicine. Biology
has a rich store of valuable knowledge extending to evolution, which is
often disregarded in medicine. For example, the role of omega-3 fatty
acids in the genesis of disease deserves much more attention. Biomedical
research should pay more attention to higher levels of organization and
to functional explanation. A case study of migraine illustrates all this.
Biology is an infectious discipline in that parts of it are incorporated
in many disciplines. But in the process of incorporation it is often
distorted by transformations and omissions. Nobody would deny that
applications of biology to medicine, for example, often amount to a
blessing. At the same time, some distortions of biology may tend to
transform blessings into a curse.
RP van der Steen, WJ (autor correspondente), Maluslaan 3, NL-1185 KZ
Amstelveen, Netherlands.
EM wjvds@wanadoo.nl
CR Almallah YZ, 2000, J CLIN IMMUNOL, V20, P68, DOI
10.1023/A:1006698728816
   APPENZELLER O, 1991, MED CLIN N AM, V75, P763, DOI 10.1016/S0025-
7125(16)30448-5
   Bäcker M, 2001, CEPHALALGIA, V21, P611, DOI 10.1046/j.1468-
2982.2001.00219.x
   Banks William A., 1997, P3
   Barkow J.H., 1992, ADAPTED MIND
   Bjorklund D.F., 2002, ORIGINS HUMAN NATURE
   Boska MD, 2002, NEUROLOGY, V58, P1227, DOI 10.1212/WNL.58.8.1227
   Brandon R, 1990, Adaptation and environment
   Burian RM, 1996, STUD HIST PHILOS SCI, V27, P1, DOI 10.1016/0039-
3681(95)00034-8
   Buschmann J, 1996, HEADACHE, V36, P586, DOI 10.1046/j.1526-
4610.1996.3610586.x
   Buss D., 1998, Evolutionary psychology, V3rd
   BUSS DM, 1995, PSYCHOL INQ, V6, P1, DOI 10.1207/s15327965pli0601_1

Cady R K, 1999, Clin Cornerstone, V1, P21, DOI 10.1016/S1098-3597(99)90037-6
Chikanza IC, 1998, BRIT J RHEUMATOL, V37, P131
Clandinin M. Thomas, 1997, P15
Constantinescu CS, 2002, HEADACHE, V42, P227, DOI 10.1046/j.1526-4610.2002.02057.x
COSMIDES L, 1994, COGNITION, V50, P41, DOI 10.1016/0010-0277(94)90020-5
Covelli V, 1998, IMMUNOPHARM IMMUNOT, V20, P199, DOI 10.3109/08923979809038539
DALY M, 1988, SCIENCE, V242, P519, DOI 10.1126/science.3175672
Daly M., 1988, HOMICIDE
Daly Martin., 1998, TRUTH CINDERELLA DAR
DAVIDOFF R.A., 2002, Migraine: Manifestations, Pathogenesis and Management
Davies PS, 1996, MIND MACH, V6, P559
de Jong HL, 1998, PHILOS PSYCHOL, V11, P183, DOI 10.1080/09515089808573255
de Tommaso M, 2002, INT J PSYCHOPHYSIOL, V44, P239, DOI 10.1016/S0167-8760(02)00009-0
Diamond S, 2002, CNS DRUGS, V16, P385, DOI 10.2165/00023210-200216060-00003
Dreier JP, 2002, BRAIN, V125, P102, DOI 10.1093/brain/awf007
EATON SB, 1985, NEW ENGL J MED, V312, P283, DOI 10.1056/NEJM198501313120505
Empl M, 1999, CEPHALALGIA, V19, P713, DOI 10.1046/j.1468-2982.1999.019008713.x
Engler MB, 2000, BRIT J PHARMACOL, V131, P1793, DOI 10.1038/sj.bjp.0703754
Erasmus U., 1993, Fats That Heal, Fats That Kill: The Complete Guide to Fats, Oils, Cholesterol and Human Health
Ewald P W, 1994, Evolution of Infectious Diseases
Flammer J, 2001, PROG RETIN EYE RES, V20, P319, DOI 10.1016/S1350-9462(00)00028-8
Frank S.A., 2002, Immunology and Evolution of Infectious Disease
Giffin NJ, 2002, CURR OPIN NEUROL, V15, P303, DOI 10.1097/00019052-200206000-00013
Greenblatt Charles., 2003, Emerging Pathogens: The Archaeology, Ecology, and Evolution of Infectious Disease
Griffiths PE, 1996, BRIT J PHILOS SCI, V47, P511, DOI 10.1093/bjps/47.4.511
Grimble RF, 2001, P NUTR SOC, V60, P389, DOI 10.1079/PNS2001102
Grimm H, 2002, BRIT J NUTR, V87, pS59, DOI 10.1079/BJN2001457
Guidetti V, 1998, CEPHALALGIA, V18, P455, DOI 10.1046/j.1468-2982.1998.1807455.x
Gupta VK, 1997, CEPHALALGIA, V17, P561, DOI 10.1046/j.1468-2982.1997.1705561.x
Hamazaki T, 1996, J CLIN INVEST, V97, P1129, DOI 10.1172/JCI118507
Hamazaki T, 2002, NUTR NEUROSCI, V5, P37, DOI 10.1080/10284150290007119
HANINGTON E, 1981, LANCET, V2, P720
Harel Z, 2002, J ADOLESCENT HEALTH, V31, P154, DOI 10.1016/S1054-139X(02)00349-X

Hasselblatt M, 1999, NEUROREPORT, V10, P423, DOI 10.1097/00001756-199902050-00039
Healy D., 2002, CREATION PSYCHOPHARM
Hellstrom HR, 1999, MED HYPOTHESES, V53, P194, DOI 10.1054/mehy.1998.0745
Holman Ralph T., 1997, P139
Horrobin David F., 1997, P245
Horrobin DF, 2002, PROSTAG LEUKOTR ESS, V66, P83, DOI 10.1054/plef.2001.0338
Jamin SP, 1999, RES COMMUN MOL PATH, V105, P271
Johnson S, 2001, MED HYPOTHESES, V56, P163, DOI 10.1054/mehy.2000.1133
Katz R, 2001, J MOL NEUROSCI, V16, P333, DOI 10.1385/JMN:16:2-3:333
KEYS A, 1970, CIRCULATION, V41, pI1
Laland K.N., 2002, SENSE NONSENSE EVOLU
Lloyd EA, 1999, BIOL PHILOS, V14, P211, DOI 10.1023/A:1006638501739
MARTELLETTI P, 1989, HEADACHE, V29, P664, DOI 10.1111/j.1526-4610.1989.hed2910664.x
Mauskop A, 2002, HEADACHE, V42, P242, DOI 10.1046/j.1526-4610.2002.02075.x
Mayer K, 2002, AM J PHYSIOL-HEART C, V283, pH811, DOI 10.1152/ajpheart.00235.2002
Mezei Z, 2000, PLATELETS, V11, P222
Moberg G. P, 2000, BIOL ANIMAL STRESS B, DOI DOI 10.1079/9780851993591.0000
Nesse R.M., 1994, WHY WE GET SICK
NESSE RM, 1998, SCI AM NOV, P58
Peet M, 2001, SCHIZOPHR RES, V49, P243, DOI 10.1016/S0920-9964(00)00083-9
Peet M, 2002, J PSYCHIAT RES, V36, P7, DOI 10.1016/S0022-3956(01)00048-6
Pradalier A, 2001, CEPHALALGIA, V21, P818, DOI 10.1046/j.1468-2982.2001.218240.x
Puri BK, 2002, NEUROREPORT, V13, P123, DOI 10.1097/00001756-200201210-00029
Richardson RC, 1996, PHILOS SCI, V63, P122, DOI 10.1086/289899
ROSE S, 2000, ALAS POOR DARWIN ARG, P106
SACKS O, 1985, MIGRAINE REVISED EXP
Scott LV, 1998, LIFE SCI, V62, P1985, DOI 10.1016/S0024-3205(98)00027-7
Serhan CN, 2001, J CLIN INVEST, V107, P1481, DOI 10.1172/JCI13375
Sethi S, 2002, BLOOD, V100, P1340, DOI 10.1182/blood-2002-01-0316
Sheftell FD, 2002, HEADACHE, V42, P58, DOI 10.1046/j.1526-4610.2002.02015.x
Shimokawa H, 2001, WORLD REV NUTR DIET, V88, P100
Silberstein SD, 2001, HEADACHE, V41, pS11
Simopoulos AP, 2001, J NUTR, V131, p3065S, DOI 10.1093/jn/131.11.3065S
SMITH RS, 1992, MED HYPOTHESES, V39, P168, DOI 10.1016/0306-9877(92)90181-B
Stevens A., 1996, EVOLUTIONARY PSYCHIA
Stoll A., 2001, OMEGA 3 CONNECTION
SWANSON DR, 1986, PERSPECT BIOL MED, V30, P7
Swanson DR, 1999, LIBR TRENDS, V48, P48
SWANSON DR, 1987, J AM SOC INFORM SCI, V38, P228, DOI 10.1002/(SICI)1097-4571(198707)38:4<228::AID-ASI2>3.0.CO;2-G

Swanson DR, 1997, ARTIF INTELL, V91, P183, DOI 10.1016/S0004-3702(97)00008-8
SWANSON DR, 1990, B MED LIBR ASSOC, V78, P29
SWANSON DR, 1988, PERSPECT BIOL MED, V31, P526
SWANSON DR, 2001, B AM SOC INFORM FEB, P12
SWANSON DR, 1991, SIGIR91 P 14 ANN INT
Terada S, 2001, MICROBIOL IMMUNOL, V45, P473, DOI 10.1111/j.1348-0421.2001.tb02647.x
Van der Steen, 2003, BOUNDARIES BIOMEDICI
van der Steen W.J., 2001, Methods and morals in the life sciences
van der Steen W. J., 2000, EVOLUTION NATURAL HI
van der Steen WJ, 2003, ACTA BIOTHEOR, V51, P151, DOI 10.1023/A:1025119821313
van der Steen WJ, 2001, ACTA BIOTHEOR, V49, P125, DOI 10.1023/A:1010240129648
van der Steen WJ, 1993, PRACTICAL PHILOS LIF
VANDERSTEEN WJ, 2003, DRUGS VERSUS DIETS D
Voulgari PV, 2000, ANN RHEUM DIS, V59, P206, DOI 10.1136/ard.59.3.206
Wilson D., 2002, DARWINS CATHEDRAL EV
Yamada N, 1998, J NUTR SCI VITAMINOL, V44, P279, DOI 10.3177/jnsv.44.279
NR 100
TC 1
Z9 1
U1 0
U2 1
PU SPRINGER
PI DORDRECHT
PA PO BOX 17, 3300 AA DORDRECHT, NETHERLANDS
BN 978-1-4020-2904-2
PY 2005
BP 71
EP 94
D2 10.1007/1-4020-2904-7
PG 24
WC Mathematical & Computational Biology; History & Philosophy Of Science
WE Book Citation Index– Science (BKCI-S)
SC Mathematical & Computational Biology; History & Philosophy of Science
GA BKP64
UT WOS:000268884300004
DA 2025-05-07
ER

PT J
AU Sadhir, S
McGrosky, A
Swanson, ZS
Tomechko, K
Pontzer, H
AF Sadhir, Srishti
McGrosky, Amanda
Swanson, Zane S.
Tomechko, Keri
Pontzer, Herman

TI Maternal Metabolic Limits In Endurance Athletes During Pregnancy
SO MEDICINE & SCIENCE IN SPORTS & EXERCISE
LA English
DT Meeting Abstract
CT Annual Meeting of the American-College-of-Sports-Medicine (ACSM)
CY MAY 28-31, 2024
CL Boston, MA
SP Amer Coll Sports Med
C1 [Sadhir, Srishti; McGrosky, Amanda; Tomechko, Keri; Pontzer, Herman] Duke Univ, Durham, NC USA.
[Swanson, Zane S.] Ctr Strateg & Int Studies, Washington, DC USA.
C3 Duke University; Centre for Strategic & International Studies
EM srishtis394@gmail.com
RI McGrosky, Amanda/ABM-9253-2022; Sadhir, Srishti/JDX-0199-2023
FU National Science Foundation [2316583]; Leakey Foundation; Triangle Center for Evolutionary Medicine
FX This study was funded by The National Science Foundation (BA-DDRIG #2316583), The Leakey Foundation, and the Triangle Center for Evolutionary Medicine.
NR 0
TC 0
Z9 0
U1 0
U2 0
PU LIPPINCOTT WILLIAMS & WILKINS
PI PHILADELPHIA
PA TWO COMMERCE SQ, 2001 MARKET ST, PHILADELPHIA, PA 19103 USA
SN 0195-9131
EI 1530-0315
J9 MED SCI SPORT EXER
JI Med. Sci. Sports Exerc.
PD OCT
PY 2024
VL 56
IS 10
SU S
MA 109
BP 16
EP 16
PG 1
WC Sport Sciences
WE Science Citation Index Expanded (SCI-EXPANDED); Conference Proceedings Citation Index - Science (CPCI-S)
SC Sport Sciences
GA G2P9I
UT WOS:001315123200033
DA 2025-05-07
ER

PT J
AU Halawani, O
Dunn, RR
Grunden, AM
Smith, AA

AF Halawani, Omar
   Dunn, Robert R.
   Grunden, Amy M.
   Smith, Adrian A.
TI Bacterial exposure leads to variable mortality but not a measurable
   increase in surface antimicrobials across ant species
SO PEERJ
LA English
DT Article
DE Entomopathogen; Metapleural gland; Social immunity; Bacterial
exposure;
   Social insects; Sociobiology; Antimicrobial
ID METAPLEURAL GLAND; GROUP-SIZE; FIRE ANT; DEFENSES
AB Social insects have co-existed with microbial species for millions of years and have evolved a diversity of collective defenses, including the use of antimicrobials. While many studies have revealed strategies that ants use against microbial entomopathogens, and several have shown ant-produced compounds inhibit environmental bacterial growth, few studies have tested whether exposure to environmental bacteria represents a health threat to ants. We compare four ant species' responses to exposure to Escherichia coli and Staphylococcus epidermidis bacteria in order to broaden our understanding of microbial health-threats to ants and their ability to defend against them. In a first experiment, we measure worker mortality of Solenopsis invicta, Brachymyrmex chinensis, Aphaenogaster rudis, and Dorymyrmex bureni in response to exposure to E. coli and S. epidermidis. We found that exposure to E. coli was lethal for S. invicta and D. bureni, while all other effects of exposure were not different from experimental controls. In a second experiment, we compared the antimicrobial ability of surface extracts from bacteria-exposed and non-exposed S. invicta and B. chinensis worker ants, to see if exposure to E. coli or S. epidermidis led to an increase in antimicrobial compounds. We found no difference in the inhibitory effects from either treatment group in either species. Our results demonstrate the susceptibility to bacteria is varied across ant species. This variation may correlate with an ant species' use of surface antimicrobials, as we found significant mortality effects in species which also were producing antimicrobials. Further exploration of a wide range of both bacteria and ant species is likely to reveal unique and nuanced antimicrobial strategies and deepen our understanding of how ant societies respond to microbial health threats.
C1 [Halawani, Omar; Dunn, Robert R.] North Carolina State Univ, Dept Appl Ecol, Raleigh, NC 27695 USA.
   [Grunden, Amy M.] North Carolina State Univ, Dept Plant & Microbial Biol, Raleigh, NC USA.
   [Smith, Adrian A.] North Carolina State Univ, Dept Biol Sci, Raleigh, NC 27695 USA.
   [Smith, Adrian A.] North Carolina Museum Nat Sci, Res & Collect, Raleigh, NC 27695 USA.
C3 North Carolina State University; North Carolina State University; North
   Carolina State University
RP Halawani, O (autor correspondente), North Carolina State Univ, Dept Appl Ecol, Raleigh, NC 27695 USA.; Smith, AA (autor correspondente), North Carolina State Univ, Dept Biol Sci, Raleigh, NC 27695 USA.; Smith,

AA (autor correspondente), North Carolina Museum Nat Sci, Res & Collect, Raleigh, NC 27695 USA.
EM oghalawani@gmail.com; aasmith7@ncsu.edu
RI Dunn, Robert/B-1360-2013
OI Grunden, Amy/0000-0002-8025-753X; Dunn, Robert/0000-0002-6030-4837
FU Triangle Center for Evolutionary Medicine (TriCEM)
FX Funding for this study was provided by a Triangle Center for Evolutionary Medicine (TriCEM) grant awarded to Adrian Smith. There was no additional external funding received for this study. The funders had no role in study design, data collection and analysis, decision to publish, or preparation of the manuscript.
CR BLUM MS, 1958, SCIENCE, V128, P306, DOI 10.1126/science.128.3319.306-a
Bos N, 2019, INSECTS, V10, DOI 10.3390/insects10090271
Braun K, 2018, TOXICOL IN VITRO, V52, P214, DOI 10.1016/j.tiv.2018.06.019
Calvo B, 2016, R J, V8, P248
Cremer S, 2007, CURR BIOL, V17, pR693, DOI 10.1016/j.cub.2007.06.008
de Carvalho DB, 2019, TOXINS, V11, DOI 10.3390/toxins11070420
García S, 2010, INFORM SCIENCES, V180, P2044, DOI 10.1016/j.ins.2009.12.010
Graystock P, 2011, BEHAV ECOL SOCIOBIOL, V65, P2319, DOI 10.1007/s00265-011-1242-y
Hoggard SJ, 2013, ANN ENTOMOL SOC AM, V106, P53, DOI 10.1603/AN11187
Hoggard SJ, 2011, PLOS ONE, V6, DOI 10.1371/journal.pone.0021763
Hughes WOH, 2002, P ROY SOC B-BIOL SCI, V269, P1811, DOI 10.1098/rspb.2002.2113
Ishak HD, 2011, MICROB ECOL, V61, P821, DOI 10.1007/s00248-010-9793-4
JOUVENAZ DP, 1972, ANTIMICROB AGENTS CH, V2, P291, DOI 10.1128/AAC.2.4.291
Kautz S., 2013, PSYCHE-J ENTOMOL, V2013, P936341, DOI DOI 10.1155/2013/936341
Kay AD, 2014, P ROY SOC B-BIOL SCI, V281, DOI 10.1098/rspb.2013.2374
Konrad M, 2018, P NATL ACAD SCI USA, V115, P2782, DOI 10.1073/pnas.1713501115
Konrad M, 2012, PLOS BIOL, V10, DOI 10.1371/journal.pbio.1001300
Levy S.B., 1992, The antibiotic paradox
LOFGREN CS, 1975, ANNU REV ENTOMOL, V20, P1, DOI 10.1146/annurev.en.20.010175.000245
Lucas JM, 2019, P ROY SOC B-BIOL SCI, V286, DOI 10.1098/rspb.2019.1026
MacGowen J., 2014, ANTS FORMICIDAE SE U
MACKINTOSH JA, 1995, CAN J MICROBIOL, V41, P136, DOI 10.1139/m95-018
Meunier J, 2015, PHILOS T R SOC B, V370, DOI 10.1098/rstb.2014.0102
Penick CA, 2018, ROY SOC OPEN SCI, V5, DOI 10.1098/rsos.171332
Pull CD, 2018, ELIFE, V7, DOI [10.7554/eLife.32073, 10.7554/elife.32073]
R Core Team, 2017, R LANG ENV STAT COMP
Ramalho MO, 2017, BMC EVOL BIOL, V17, DOI 10.1186/s12862-017-0945-8
Reber A, 2011, J EVOLUTION BIOL, V24, P954, DOI 10.1111/j.1420-9101.2011.02230.x
Schlüns H, 2009, MYRMECOL NEWS, V12, P237
Schmid-Hempel P., 1998, Parasites in social insects.
Sengupta S, 2013, FRONT MICROBIOL, V4, DOI 10.3389/fmicb.2013.00047

   Stow A, 2007, BIOL LETTERS, V3, P422, DOI 10.1098/rsbl.2007.0178
   Stucki D, 2019, PEERJ, V7, DOI 10.7717/peerj.6428
   Tragust S, 2013, CURR BIOL, V23, P76, DOI 10.1016/j.cub.2012.11.034
   Traniello JFA, 2002, P NATL ACAD SCI USA, V99, P6838, DOI
10.1073/pnas.102176599
   Turnbull C, 2011, BIOL LETTERS, V7, P249, DOI 10.1098/rsbl.2010.0719
   Wenseleers T, 1998, P ROY SOC B-BIOL SCI, V265, P1447, DOI
10.1098/rspb.1998.0456
   Yek SH, 2012, P ROY SOC B-BIOL SCI, V279, P4215, DOI
10.1098/rspb.2012.1458
   Yek SH, 2011, BIOL REV, V86, P774, DOI 10.1111/j.1469-
185X.2010.00170.x
NR 39
TC 0
Z9 0
U1 0
U2 11
PU PEERJ INC
PI LONDON
PA 341-345 OLD ST, THIRD FLR, LONDON, EC1V 9LL, ENGLAND
SN 2167-8359
J9 PEERJ
JI PeerJ
PD DEC 3
PY 2020
VL 8
AR e10412
DI 10.7717/peerj.10412
PG 15
WC Multidisciplinary Sciences
WE Science Citation Index Expanded (SCI-EXPANDED)
SC Science & Technology - Other Topics
GA OZ9KX
UT WOS:000595237000003
PM 33344078
OA gold, Green Published
DA 2025-05-07
ER

PT J
AU Vaziri, F
   Kohl, TA
   Ghajavand, H
   Kamakoli, MK
   Merker, M
   Hadifar, S
   Khanipour, S
   Fateh, A
   Masoumi, M
   Sladat, SD
   Niemann, S
AF Vaziri, Farzam
   Kohl, Thomas A.
   Ghajavand, Hasan

Kamakoli, Mansour Kargarpour
   Merker, Matthias
   Hadifar, Shima
   Khanipour, Sharareh
   Fateh, Abolfazi
   Masoumi, Morteza
   Sladat, Seyed Davar
   Niemann, Stefan
TI Genetic Diversity of Multi- and Extensively Drug-Resistant
   Mycobacterium tuberculosis Isolates in the Capital of Iran,
   Revealed by Whole-Genome Sequencing
SO JOURNAL OF CLINICAL MICROBIOLOGY
LA English
DT Article
DE Mycobacterium tuberculosis; Tehran; extensive drug resistance;
multidrug
   resistance; whole-genome sequencing
ID MUTATIONS; TRANSMISSION; GYRB
AB The emergence and spread of multidrug resistant (MDR) Mycobacterium tuberculosis complex (MTBC) strains is a critical global health problem. Between 2014 and 2018, 606 MTBC strains were isolated from 13,892 suspected pulmonary tuberculosis (TB) patients in Tehran, Iran, including 16 (2.6%) MDR-TB cases. A combination of phenotypic and genotypic methods (whole-genome sequencing) was employed for the identification of additional drug resistances and strain-to-strain genetic distances as a marker for recent transmission events. MDR and extensively drug-resistant (XDR) TB cases were almost exclusively infected by lineage 2/Beijing strains (14/16, P < 0.001). We further showed that recent transmission and/or recent introduction of lineage 2/Beijing strains contribute to high XDR-TB rates among all MDR-TB cases and should be considered an emerging threat for TB control in Tehran. In addition, the extensive pre-existing drug resistance profiles of MDR/XDR strains will further challenge TB diagnostics in the region.
C1 [Vaziri, Farzam; Ghajavand, Hasan; Kamakoli, Mansour Kargarpour; Hadifar, Shima; Khanipour, Sharareh; Fateh, Abolfazi; Masoumi, Morteza; Sladat, Seyed Davar] Pasteur Inst Iran, Dept Mycobacteriol & Pulm Res, Tehran, Iran.
   [Vaziri, Farzam; Ghajavand, Hasan; Kamakoli, Mansour Kargarpour; Hadifar, Shima; Fateh, Abolfazi; Sladat, Seyed Davar] Pasteur Inst Iran, MRC, Tehran, Iran.
   [Kohl, Thomas A.; Merker, Matthias; Niemann, Stefan] Res Ctr Borstel, Div Mol & Expt Mycobacteriol, Borstel, Germany.
   [Kohl, Thomas A.; Merker, Matthias; Niemann, Stefan] German Ctr Infect Res DZIF, Partner Site Hamburg Lubeck Borstel, Borstel, Germany.
C3 Pasteur Network; Pasteur Institute of Iran; Pasteur Network; Pasteur
   Institute of Iran; Leibniz Association; Forschungszentrum Borstel;
   German Center for Infection Research
RP Niemann, S (autor correspondente), Res Ctr Borstel, Div Mol & Expt Mycobacteriol, Borstel, Germany.; Niemann, S (autor correspondente), German Ctr Infect Res DZIF, Partner Site Hamburg Lubeck Borstel, Borstel, Germany.
EM sniemann@fz.borstel.de
RI Niemann, Stefan/C-9327-2011; Fateh, Abolfazl/K-9507-2017; Vaziri,
   Farzam/K-9535-2017; Siadat, Seyed/A-2204-2012; Kohl, Thomas

Andreas/AAB-9782-2020; Hadifar, Shima/K-5752-2019; Kargarpour Kamakoli,
Mansour/W-7206-2018; Merker, Matthias/P-7399-2015
OI Siadat, Seyed Davar/0000-0002-6892-5603; Kohl, Thomas
Andreas/0000-0002-1126-6803; Hadifar, Shima/0000-0003-4702-634X;
Kargarpour Kamakoli, Mansour/0000-0002-8588-6407; Vaziri,
Farzam/0000-0001-7788-2548; Merker, Matthias/0000-0003-1386-2331;
Niemann, Stefan/0000-0002-6604-0684
FU German Center for Infection Research (DZIF); Leibniz Science Campus
Evolutionary Medicine of the Lung (EvoLUNG)
FX This work was partially funded by the German Center for Infection
Research (DZIF) and the Leibniz Science Campus Evolutionary Medicine of
the Lung (EvoLUNG).
CR Ali A, 2015, PLOS ONE, V10, DOI 10.1371/journal.pone.0117771
[Anonymous], GLOB TUB CONTR SURV
Baym M, 2015, PLOS ONE, V10, DOI 10.1371/journal.pone.0128036
Caminero JA, 2010, LANCET INFECT DIS, V10, P621, DOI 10.1016/S1473-3099(10)70139-0
Casali N, 2014, NAT GENET, V46, P279, DOI 10.1038/ng.2878
Chien JY, 2016, ANTIMICROB AGENTS CH, V60, P2090, DOI 10.1128/AAC.01049-15
Cole ST, 1998, NATURE, V393, P537, DOI 10.1038/31159
Coll F, 2014, NAT COMMUN, V5, DOI 10.1038/ncomms5812
Comas I, 2012, NAT GENET, V44, P106, DOI 10.1038/ng.1038
Dheda K, 2016, LANCET, V387, P1211, DOI 10.1016/S0140-6736(15)00151-8
Drobniewski F, 2007, CLIN MICROBIOL INFEC, V13, P1144, DOI 10.1111/j.1469-0691.2007.01813.x
Eldholm V, 2015, NAT COMMUN, V6, DOI 10.1038/ncomms8119
Georghiou SB, 2012, PLOS ONE, V7, DOI 10.1371/journal.pone.0033275
Gygli SM, 2017, FEMS MICROBIOL REV, V41, P354, DOI 10.1093/femsre/fux011
Khanipour S, 2016, J GLOB ANTIMICROB RE, V5, P7, DOI 10.1016/j.jgar.2016.01.007
Kumar Satish, 2014, Genomics & Informatics, V12, P276, DOI 10.5808/GI.2014.12.4.276
Li J, 2014, EMERG MICROBES INFEC, V3, DOI 10.1038/emi.2014.21
Li QJ, 2016, ANTIMICROB AGENTS CH, V60, P2807, DOI 10.1128/AAC.02358-15
Nguyen L, 2016, ARCH TOXICOL, V90, P1585, DOI 10.1007/s00204-016-1727-6
Merker M, 2015, NAT GENET, V47, P242, DOI 10.1038/ng.3195
Mokrousov I, 2017, MOL PHYLOGENET EVOL, V116, P202, DOI 10.1016/j.ympev.2017.09.002
Mokrousov I, 2016, LANCET INFECT DIS, V16, P1326, DOI 10.1016/S1473-3099(16)30460-1
Nosova EY, 2013, J MED MICROBIOL, V62, P108, DOI 10.1099/jmm.0.046821-0
Palomino JC, 2002, ANTIMICROB AGENTS CH, V46, P2720, DOI 10.1128/AAC.46.8.2720-2722.2002
Papaventsis D, 2017, CLIN MICROBIOL INFEC, V23, P61, DOI 10.1016/j.cmi.2016.09.008
Roetzer A, 2013, PLOS MED, V10, DOI 10.1371/journal.pmed.1001387

   Satta G, 2018, CLIN MICROBIOL INFEC, V24, P604, DOI
10.1016/j.cmi.2017.10.030
   Stucki D, 2015, J INFECT DIS, V211, P1306, DOI 10.1093/infdis/jiu601
   Tarashi S, 2017, TUBERCULOSIS, V107, P31, DOI
10.1016/j.tube.2017.03.005
   Walker TM, 2013, LANCET INFECT DIS, V13, P137, DOI 10.1016/S1473-
3099(12)70277-3
   Wang F, 2007, J EXP MED, V204, P73, DOI 10.1084/jem.20062100
   Zaunbrecher MA, 2009, P NATL ACAD SCI USA, V106, P20004, DOI
10.1073/pnas.0907925106
NR 32
TC 29
Z9 29
U1 0
U2 13
PU AMER SOC MICROBIOLOGY
PI WASHINGTON
PA 1752 N ST NW, WASHINGTON, DC 20036-2904 USA
SN 0095-1137
EI 1098-660X
J9 J CLIN MICROBIOL
JI J. Clin. Microbiol.
PD JAN
PY 2019
VL 57
IS 1
AR e01477
DI 10.1128/JCM.01477-18
PG 7
WC Microbiology
WE Science Citation Index Expanded (SCI-EXPANDED)
SC Microbiology
GA HG5LI
UT WOS:000455018000019
PM 30404943
OA Green Published
DA 2025-05-07
ER

PT J
AU Kohl, TA
   Utpatel, C
   Nieman, S
   Moser, I
AF Kohl, Thomas A.
   Utpatel, Christian
   Nieman, Stefan
   Moser, Irmgard
TI Mycobacterium bovis Persistence in Two Different Captive Wild
   Animal Populations in Germany: a Longitudinal Molecular
Epidemiological
   Study Revealing Pathogen Transmission by Whole-Genome Sequencing
SO JOURNAL OF CLINICAL MICROBIOLOGY
LA English

DT Article
DE tuberculosis; Mycobacterium bovis; transmission; spoligotyping;
MIRU-VNTR-typing; whole-genome sequencing; Mycobacterium tuberculosis
complex; molecular epidemiology; veterinary microbiology
ID TUBERCULOSIS COMPLEX BACTERIA; VARIABLE-NUMBER; MIRU-VNTRPLUS;
IDENTIFICATION; INFECTION; SYSTEM; TOOL
AB Bovine tuberculosis (bTB) caused by Mycobacterium bovis is a transmissible disease notifiable to the World Organization for Animal Health and to the European Union, with ongoing efforts of surveillance and eradication in every EU member state. In Germany, a country which has been declared officially free from bovine tuberculosis since 1997 by the EU, M. bovis infections still occur sporadically in cattle and other mammals, including humans. Here, the transmission routes of a bTB outbreak in a wildlife park in Germany affecting different cervid species, bison, lynx, and pot-bellied pigs were followed by employing whole-genome sequencing (WGS) combined with spoligotyping and mycobacterial interspersed repetitive-unit-variablenumber tandem-repeat (MIRU-VNTR) typing. One single M. bovis strain persisted from 2002 to 2015, and transmission between the park and a distantly located captive cervid farm was verified. The spoligotyping patterns remained identical, while MIRU-VNTR typing of 24 loci of the standardized panel and locus 2163a as an additional locus revealed one change at locus 2165 in a strain from a fallow deer and one at locus 2461 in isolates from red deer over the whole time period. WGS analysis confirmed the close relatedness of the isolates, with a maximum of 12 single nucleotide polymorphisms (SNPs) detected between any two sequenced isolates. In conclusion, our data confirm a longitudinal outbreak of M. bovis in a German wildlife park and provide the first insights into the dynamics of different genotyping markers in M. bovis.
C1 [Kohl, Thomas A.; Utpatel, Christian; Nieman, Stefan] Res Ctr Borstel, Prior Area Infect, Mol & Expt Mycobacteriol, Borstel, Germany.
[Kohl, Thomas A.; Utpatel, Christian; Nieman, Stefan] German Ctr Infect Res, TTU TB, Borstel, Germany.
[Moser, Irmgard] Friedrich Loeffler Inst, Fed Inst Anim Hlth, Inst Mol Pathogenesis, Jena, Germany.
C3 Leibniz Association; Forschungszentrum Borstel; German Center for
Infection Research; Friedrich Loeffler Institute; Eberhard Karls
University of Tubingen; Eberhard Karls University Hospital
RP Moser, I (autor correspondente), Friedrich Loeffler Inst, Fed Inst Anim Hlth, Inst Mol Pathogenesis, Jena, Germany.
EM 0003000B00A2.gast@fli.de
RI Niemann, Stefan/C-9327-2011; Kohl, Thomas Andreas/AAB-9782-2020
OI Moser, Irmgard/0000-0003-2796-6967; Kohl, Thomas
Andreas/0000-0002-1126-6803; Niemann, Stefan/0000-0002-6604-0684
FU German Center for Infection Research (DZIF); Leibniz Science Campus
Evolutionary Medicine of the Lung (EvoLUNG)
FX The work was partially funded by the German Center for Infection
Research (DZIF) and the Leibniz Science Campus Evolutionary Medicine
of
the Lung (EvoLUNG).
CR Allix-Béguec C, 2008, J CLIN MICROBIOL, V46, P2692, DOI
10.1128/JCM.00540-08
Andrievskaia O, 2018, VET MICROBIOL, V214, P44, DOI
10.1016/j.vetmic.2017.12.005

[Anonymous], 2016, GLOB TUB REP
Barasona JA, 2017, TRANSBOUND EMERG DIS, V64, P1148, DOI 10.1111/tbed.12480
Baym M, 2015, PLOS ONE, V10, DOI 10.1371/journal.pone.0128036
Berg S, 2009, PLOS ONE, V4, DOI 10.1371/journal.pone.0005068
Biek R, 2015, TRENDS ECOL EVOL, V30, P306, DOI 10.1016/j.tree.2015.03.009
Biek R, 2012, PLOS PATHOG, V8, DOI 10.1371/journal.ppat.1003008
Bjorn-Mortensen K, 2016, SCI REP-UK, V6, DOI 10.1038/srep33180
Bjorn-Mortensen K, 2017, EUR RESPIR J, V49, DOI 10.1183/13993003.01851-2016
Brooks-Pollock E, 2014, NATURE, V511, P228, DOI 10.1038/nature13529
Brudey K, 2006, BMC MICROBIOL, V6, DOI 10.1186/1471-2180-6-23
Çavusoglu C, 2017, MIKROBIYOL BUL, V51, P165, DOI 10.5578/mb.53963
Cousins DV, 1998, INT J TUBERC LUNG D, V2, P471
Crispell J, 2017, BMC GENOMICS, V18, DOI 10.1186/s12864-017-3569-x
Deutsches Institut fur Normung e. V, 2011, DIN TASCH, P338
Dye C, 2010, SCIENCE, V328, P856, DOI 10.1126/science.1185449
Eder J, 2006, INFEKTIOLOGIE AKTUEL, P101
European Commission Health & Consumer Protection Directorate-General Veterinary and International Affairs Unit G5-Veterinary Programmes, SANCO100672013 EUR C
European Food Safety Authority, 2017, EFSA J, V15, DOI [10.2903/j.efsa.2017.5077, 10.2903/j.efsa.2017.4971]
Eyre DW, 2013, NEW ENGL J MED, V369, P1195, DOI 10.1056/NEJMoa1216064
Filliol I, 2002, EMERG INFECT DIS, V8, P1347, DOI 10.3201/eid0811.020125
Fink M, 2015, EMERG INFECT DIS, V21, P464, DOI 10.3201/eid2103.141119
Friedrich-Loeffler-Institut Federal Research Institute for Animal Health, 2016, AMTL METH
Frothingham R, 1998, MICROBIOL-UK, V144, P1189, DOI 10.1099/00221287-144-5-1189
Harrison EM, 2013, EMBO MOL MED, V5, P509, DOI 10.1002/emmm.201202413
He ZL, 2016, NUCLEIC ACIDS RES, V44, pW236, DOI 10.1093/nar/gkw370
Hlokwe TM, 2013, TRANSBOUND EMERG DIS, V60, P111, DOI 10.1111/tbed.12096
Kamerbeek J, 1997, J CLIN MICROBIOL, V35, P907, DOI 10.1128/JCM.35.4.907-914.1997
Kao RR, 2016, REV SCI TECH OIE, V35, P241, DOI 10.20506/rst.35.1.2430
Malm S, 2017, CONGO EMERG INFECT D, V23, P423
Merker M, 2017, ADV EXP MED BIOL, V1019, P43, DOI 10.1007/978-3-319-64371-7_3
Meyn A, 1961, MONATSH TIERHEILKUND, V14, P71
Moser I, 2008, VET MICROBIOL, V127, P399, DOI 10.1016/j.vetmic.2007.08.028
Müller B, 2013, EMERG INFECT DIS, V19, P899, DOI 10.3201/eid1906.120543
Navarro Y, 2015, VET MICROBIOL, V175, P99, DOI 10.1016/j.vetmic.2014.11.004
Niemann S, 2016, MICROBIOL SPECTR, V4, DOI 10.1128/microbiolspec.TBTB2-0022-2016
Oelemann MC, 2007, J CLIN MICROBIOL, V45, P691, DOI 10.1128/JCM.01393-06

OREILLY LM, 1995, TUBERCLE LUNG DIS, V76, P1, DOI 10.1016/0962-8479(95)90591-X
Palmer Mitchell V, 2012, Vet Med Int, V2012, P684720, DOI 10.1155/2012/684720
Price MN, 2010, PLOS ONE, V5, DOI 10.1371/journal.pone.0009490
Probst C, 2011, EPIDEMIOL INFECT, V139, P105, DOI 10.1017/S0950268810000786
RODRIGUEZ JG, 1995, MICROBIOL-SGM, V141, P2131, DOI 10.1099/13500872-141-9-2131
Roetzer A, 2013, PLOS MED, V10, DOI 10.1371/journal.pmed.1001387
Ruettger A, 2012, J CLIN MICROBIOL, V50, P2492, DOI 10.1128/JCM.00442-12
Schmidbauer SM, 2007, VET REC, V161, P304, DOI 10.1136/vr.161.9.304
Skuce RA, 2002, MICROBIOL-SGM, V148, P519, DOI 10.1099/00221287-148-2-519
Skuce RA, 2001, TUBERCULOSIS, V81, P169, DOI 10.1054/tube.2000.0270
Supply P, 2006, J CLIN MICROBIOL, V44, P4498, DOI 10.1128/JCM.01392-06
Trewby H, 2016, EPIDEMICS-NETH, V14, P26, DOI 10.1016/j.epidem.2015.08.003
VANEMBDEN JDA, 1993, J CLIN MICROBIOL, V31, P406, DOI 10.1128/JCM.31.2.406-409.1993
Walker TM, 2013, LANCET INFECT DIS, V13, P137, DOI 10.1016/S1473-3099(12)70277-3
Weniger T, 2010, NUCLEIC ACIDS RES, V38, pW326, DOI 10.1093/nar/gkq351
NR 53
TC 11
Z9 12
U1 5
U2 28
PU AMER SOC MICROBIOLOGY
PI WASHINGTON
PA 1752 N ST NW, WASHINGTON, DC 20036-2904 USA
SN 0095-1137
EI 1098-660X
J9 J CLIN MICROBIOL
JI J. Clin. Microbiol.
PD SEP
PY 2018
VL 56
IS 9
AR e00302-18
DI 10.1128/JCM.00302-18
PG 9
WC Microbiology
WE Science Citation Index Expanded (SCI-EXPANDED)
SC Microbiology
GA GR6OB
UT WOS:000442773300010
PM 29950330
OA Bronze, Green Published
DA 2025-05-07
ER

PT J

AU Amoroso, CR
   Nunn, CL
AF Amoroso, Caroline R.
   Nunn, Charles L.
TI Epidemiological transitions in human evolution and the richness of
   viruses, helminths, and protozoa
SO EVOLUTION MEDICINE AND PUBLIC HEALTH
LA English
DT Article
DE phylogenetic comparative analysis; disease; host-pathogen interactions;
   parasite diversity; species richness; human uniqueness
ID MAMMAL PARASITE DATABASE; SPECIES RICHNESS; INFECTIOUS-DISEASE;
   HOST-SPECIFICITY; ONLINE RESOURCE; AVOIDANCE; DISGUST; RANGE;
   TRANSMISSION; PHYLOGENY
AB Background and objectives: In absolute terms, humans are extremely highly parasitized compared to other primates. This may reflect that humans are outliers in traits correlated with parasite richness: population density, geographic range area, and study effort. The high degree of parasitism could also reflect amplified disease risk associated with agriculture and urbanization. Alternatively, controlling for other variables, cultural and psychological adaptations could have reduced parasitism in humans over evolutionary time.

Methodology: We predicted the number of parasites that would infect a nonhuman primate with human phenotypic characteristics and phylogenetic position, and then compared observed parasitism of humans in eight geopolitical countries to the predicted distributions. The analyses incorporated study effort, phylogeny, and drivers of parasitism in 33 primate species.

Results: Analyses of individual countries were not supportive of either hypothesis. When analyzed collectively, however, human populations showed consistently lower than expected richness of protozoa and helminths, but higher richness of viruses. Thus, human evolutionary innovations and new parasite exposures may have impacted groups of parasites in different ways, with support for both hypotheses in the overall analysis.

Conclusions and implications: The high level of parasitism observed in humans only applies to viruses, and was not extreme in any of our tests of individual countries. In contrast, we find consistent reductions in protozoa and helminths across countries, suggesting reduced parasitism by these groups during human evolution. We propose that hygienic and technological advances might have extinguished fecal-orally or indirectly transmitted parasites like helminths, whereas higher human densities and host-shifting potential of viruses have supported increased virus richness.

Lay Summary: Vastly more parasite species infect humans than any other primate host. Controlling for factors that influence parasite richness, such as the intensity of study effort and body mass, we find that humans may have more viruses, but fewer helminths and protozoa, than expected based on evolutionary analyses of parasitism in other primates.
C1 [Amoroso, Caroline R.; Nunn, Charles L.] Duke Univ, Dept Evolutionary Anthropol, 130 Sci Dr, Durham, NC 27707 USA.

[Amoroso, Caroline R.] Univ Virginia, Dept Biol, 485 McCormick Rd, Charlottesville, VA 22904 USA.

[Nunn, Charles L.] Duke Univ, Duke Global Hlth Inst, 310 Trent Dr, Durham, NC 27710 USA.
C3 Duke University; University of Virginia; Duke University
RP Amoroso, CR (autor correspondente), Univ Virginia, Dept Biol, 485 McCormick Rd, Charlottesville, VA 22904 USA.
EM cra2z@virginia.edu
RI Amoroso, Caroline/AAV-7410-2021
OI Amoroso, Caroline/0000-0001-5456-7975
FU NSF [BCS-1355902]; Graduate Research Fellowship from the Triangle Center
for Evolutionary Medicine
FX This project was funded by the NSF (BCS-1355902) and a Graduate Research
Fellowship from the Triangle Center for Evolutionary Medicine that supported CRA.
CR Arnold C, 2010, EVOL ANTHROPOL, V19, P114, DOI 10.1002/evan.20251
Barrett R, 1998, ANNU REV ANTHROPOL, V27, P247, DOI 10.1146/annurev.anthro.27.1.247
Bilbo SD, 2011, MED HYPOTHESES, V77, P494, DOI 10.1016/j.mehy.2011.06.019
Billing J, 1998, Q REV BIOL, V73, P3, DOI 10.1086/420058
CHAO A, 1987, BIOMETRICS, V43, P783, DOI 10.2307/2531532
Cleaveland S, 2001, PHILOS T R SOC B, V356, P991, DOI 10.1098/rstb.2001.0889
Cooper N, 2012, ECOL LETT, V15, P1370, DOI 10.1111/j.1461-0248.2012.01858.x
Curtis V, 2001, PERSPECT BIOL MED, V44, P17, DOI 10.1353/pbm.2001.0001
Curtis V, 2018, PHILOS T R SOC B, V373, DOI 10.1098/rstb.2017.0208
Curtis V, 2011, PHILOS T R SOC B, V366, P389, DOI 10.1098/rstb.2010.0117
Curtis VA, 2007, J EPIDEMIOL COMMUN H, V61, P660, DOI 10.1136/jech.2007.062380
Curtis VA, 2014, TRENDS IMMUNOL, V35, P457, DOI 10.1016/j.it.2014.08.006
Davies TJ, 2008, P ROY SOC B-BIOL SCI, V275, P1695, DOI 10.1098/rspb.2008.0284
Dunn RR, 2017, TRENDS PARASITOL, V33, P4, DOI 10.1016/j.pt.2016.09.007
Dunn RR, 2009, P ROY SOC B-BIOL SCI, V276, P3037, DOI 10.1098/rspb.2009.0413
Freckleton RP, 2002, AM NAT, V160, P712, DOI 10.1086/343873
GREGORY RD, 1990, FUNCT ECOL, V4, P645, DOI 10.2307/2389732
Kamiya T, 2014, BIOL REV, V89, P123, DOI 10.1111/brv.12046
Kavaliers M, 2004, HORM BEHAV, V46, P272, DOI 10.1016/j.yhbeh.2004.03.005
Longdon B, 2011, PLOS PATHOG, V7, DOI 10.1371/journal.ppat.1002260
Macpherson CNL, 2005, INT J PARASITOL, V35, P1319, DOI 10.1016/j.ijpara.2005.06.004
McElreath R, 2020, R PACKAGE VERSION 1
McElreath R, 2016, TEXT STAT SCI, pXI
McMichael AJ, 2001, ECOSYST HEALTH, V7, P107, DOI 10.1046/j.1526-0992.2001.007002107.x
Nunn C.L., 2014, Modern Phylogenetic Comparative Methods and Their Application in Evolutionary Biology, P481
Nunn CL, 2005, EVOL ANTHROPOL, V14, P1, DOI 10.1002/evan.20041

Nunn CL, 2003, AM NAT, V162, P597, DOI 10.1086/378721
Parrish CR, 2008, MICROBIOL MOL BIOL R, V72, P457, DOI 10.1128/MMBR.00004-08
Pedersen AB, 2005, INT J PARASITOL, V35, P647, DOI 10.1016/j.ijpara.2005.01.005
Poulin R, 1998, J PARASITOL, V84, P485, DOI 10.2307/3284710
POULIN R, 1995, ECOL MONOGR, V65, P283, DOI 10.2307/2937061
ROZIN P, 1987, PSYCHOL REV, V94, P23, DOI 10.1037/0033-295X.94.1.23
Stan Development Team, 2020, RStan: the R interface to Stan
Stephens PR, 2017, ECOLOGY, V98, P1476, DOI 10.1002/ecy.1799
Tatem AJ, 2006, ADV PARASIT, V62, P293, DOI 10.1016/S0065-308X(05)62009-X
Teitelbaum CS, 2020, ECOGRAPHY, V43, P1316, DOI 10.1111/ecog.05143
Townsend AK, 2020, P ROY SOC B-BIOL SCI, V287, DOI 10.1098/rspb.2020.1039
Vavrek MJ, 2011, PALAEONTOL ELECTRON, V14
Vining AQ, 2016, EVOL MED PUBLIC HLTH, P312, DOI 10.1093/emph/eow026
Walpole SC, 2012, BMC PUBLIC HEALTH, V12, DOI 10.1186/1471-2458-12-439
Werner CS, 2020, P ROY SOC B-BIOL SCI, V287, DOI 10.1098/rspb.2020.0397
WHITE LJT, 1994, J ANIM ECOL, V63, P499, DOI 10.2307/5217
Wolfe ND, 2007, NATURE, V447, P279, DOI 10.1038/nature05775
Wolfe ND, 2005, EMERG INFECT DIS, V11, P1822, DOI 10.3201/eid1112.040789
Woolhouse M, 2012, PHILOS T R SOC B, V367, P2864, DOI 10.1098/rstb.2011.0354
Woolhouse MEJ, 2005, TRENDS ECOL EVOL, V20, P238, DOI 10.1016/j.tree.2005.02.009
Woolhouse MEJ, 2005, EMERG INFECT DIS, V11, P1842, DOI 10.3201/eid1112.050997
Wrangham RW, 1999, CURR ANTHROPOL, V40, P567, DOI 10.1086/300083
NR 48
TC 5
Z9 5
U1 0
U2 2
PU OXFORD UNIV PRESS
PI OXFORD
PA GREAT CLARENDON ST, OXFORD OX2 6DP, ENGLAND
EI 2050-6201
J9 EVOL MED PUBLIC HLTH
JI Evol. Med. Public Health
PY 2021
VL 9
IS 1
BP 139
EP 147
DI 10.1093/emph/eoab009
EA FEB 2021
PG 9
WC Evolutionary Biology; Public, Environmental & Occupational Health
WE Science Citation Index Expanded (SCI-EXPANDED); Social Science Citation Index (SSCI)
SC Evolutionary Biology; Public, Environmental & Occupational Health

GA RG7MY
UT WOS:000635719400001
PM 33738104
OA gold, Green Published
DA 2025-05-07
ER

PT J
AU Paietta, EN
Kraberger, S
Lund, MC
Vargas, KL
Custer, JM
Ehmke, E
Yoder, AD
Varsani, A
AF Paietta, Elise N.
Kraberger, Simona
Lund, Michael C.
Vargas, Karla L.
Custer, Joy M.
Ehmke, Erin
Yoder, Anne D.
Varsani, Arvind
TI Diverse Circular DNA Viral Communities in Blood, Oral, and Fecal Samples
of Captive Lemurs
SO VIRUSES-BASEL
LA English
DT Article
DE lemurs; Anelloviridae; Cressdnaviricota; Microviridae; Inoviridae;
Caudoviricetes
ID STOOL SAMPLES; VIRUSES; VIROME; GIARDIA; CATTA; METAGENOMICS;
BIOGEOGRAPHY; PERFORMANCE; POPULATION; PLANTS
AB Few studies have addressed viral diversity in lemurs despite their unique evolutionary history on the island of Madagascar and high risk of extinction. Further, while a large number of studies on animal viromes focus on fecal samples, understanding viral diversity across multiple sample types and seasons can reveal complex viral community structures within and across species. Groups of captive lemurs at the Duke Lemur Center (Durham, NC, USA), a conservation and research center, provide an opportunity to build foundational knowledge on lemur-associated viromes. We sampled individuals from seven lemur species, i.e., collared lemur (Eulemur collaris), crowned lemur (Eulemur coronatus), blue-eyed black lemur (Eulemur flavifrons), ring-tailed lemur (Lemur catta), Coquerel's sifaka (Propithecus coquereli), black-and-white ruffed lemur (Varecia variegata variegata), and red ruffed lemur (Varecia rubra), across two lemur families (Lemuridae, Indriidae). Fecal, blood, and saliva samples were collected from Coquerel's sifaka and black-and-white ruffed lemur individuals across two sampling seasons to diversify virome biogeography and temporal sampling. Using viral metagenomic workflows, the complete genomes of anelloviruses (n = 4), cressdnaviruses (n = 47), caudoviruses (n = 15), inoviruses (n = 34), and microviruses (n = 537) were determined from lemur blood, feces, and saliva. Many virus genomes, especially

bacteriophages, identified in this study were present across multiple lemur species. Overall, the work presented here uses a viral metagenomics approach to investigate viral communities inhabiting the blood, oral cavity, and feces of healthy captive lemurs.
C1 [Paietta, Elise N.; Yoder, Anne D.] Duke Univ, Dept Biol, Durham, NC 27708 USA.
[Kraberger, Simona; Lund, Michael C.; Vargas, Karla L.; Custer, Joy M.; Varsani, Arvind] Arizona State Univ, Biodesign Ctr Fundamental & Appl Microbi, Ctr Evolut & Med, Tempe, AZ 85287 USA.
[Kraberger, Simona; Lund, Michael C.; Vargas, Karla L.; Custer, Joy M.; Varsani, Arvind] Arizona State Univ, Sch Life Sci, Tempe, AZ 85287 USA.
[Ehmke, Erin] Duke Univ, Duke Lemur Ctr, Durham, NC 27708 USA.
[Varsani, Arvind] Univ Cape Town, Dept Integrat Biomed Sci, Struct Biol Res Unit, ZA-7925 Cape Town, South Africa.
C3 Duke University; Arizona State University; Arizona State University-Tempe; Arizona State University; Arizona State University-Tempe; Duke University; University of Cape Town
RP Paietta, EN (autor correspondente), Duke Univ, Dept Biol, Durham, NC 27708 USA.; Varsani, A (autor correspondente), Arizona State Univ, Biodesign Ctr Fundamental & Appl Microbi, Ctr Evolut & Med, Tempe, AZ 85287 USA.; Varsani, A (autor correspondente), Arizona State Univ, Sch Life Sci, Tempe, AZ 85287 USA.; Varsani, A (autor correspondente), Univ Cape Town, Dept Integrat Biomed Sci, Struct Biol Res Unit, ZA-7925 Cape Town, South Africa.
EM elise.paietta@duke.edu; arvind.varsani@asu.edu
RI Varsani, Arvind/JOZ-5299-2023; Ehmke, Erin/KWU-9139-2024
OI Kraberger, Simona/0000-0002-7037-9242; Custer, Joy/0000-0001-9963-1133;
Varsani, Arvind/0000-0003-4111-2415
FU TriCEM (Triangle Center for Evolutionary Medicine); Duke Biology; Duke Lemur Center; Sigma Xi
FX The research described here was supported by the TriCEM (Triangle Center
for Evolutionary Medicine), Duke Biology, the Duke Lemur Center, and Sigma Xi grants awarded to Elise N. Paietta.
CR Abe N, 2002, VET PARASITOL, V106, P203, DOI 10.1016/S0304-4017(02)00050-X
dos Reis LDA, 2022, J PLANT PATHOL, V104, P1129, DOI 10.1007/s42161-022-01151-w
Amatya R, 2017, GENOME ANNOUNCEMENTS, V5, DOI [10.1128/genomea.00698-17, 10.1128/genomeA.00698-17]
Amoroso CR, 2017, BEHAVIOUR, V154, P1239, DOI [10.1163/1568539X-00003466, 10.1163/1568539x-00003466]
Anindita PD, 2019, SCI REP-UK, V9, DOI 10.1038/s41598-019-41358-z
Bigarré L, 2005, J GEN VIROL, V86, P631, DOI 10.1099/vir.0.80573-0
Blinkova O, 2010, J GEN VIROL, V91, P74, DOI 10.1099/vir.0.015446-0
Bolatti EM, 2020, VIRUSES-BASEL, V12, DOI 10.3390/v12040422
Bolger AM, 2014, BIOINFORMATICS, V30, P2114, DOI 10.1093/bioinformatics/btu170
Breitbart M, 2017, J GEN VIROL, V98, P1997, DOI 10.1099/jgv.0.000871
Briddon R.W., 2009, Encyclopedia of Life Sciences, P1
Britt A, 2000, FOLIA PRIMATOL, V71, P133, DOI 10.1159/000021741
Buchfink B, 2015, NAT METHODS, V12, P59, DOI 10.1038/nmeth.3176

Buck C. B., 2024, bioRxiv, DOI [10.1101/2024.03.25.586562, DOI 10.1101/2024.03.25.586562]
Bushnell B, 2015, BBMAP SHORT READ ALI
Butkovic A, 2023, VIRUS EVOL, V9, DOI 10.1093/ve/vead035
Capella-Gutiérrez S, 2009, BIOINFORMATICS, V25, P1972, DOI 10.1093/bioinformatics/btp348
Chabi-Jesus C, 2020, ARCH VIROL, V165, P1225, DOI 10.1007/s00705-020-04569-8
Chrzastek K, 2021, MICROORGANISMS, V9, DOI 10.3390/microorganisms9122602
Collins CL, 2022, MICROBIOL RESOUR ANN, V11, DOI 10.1128/mra.00681-22
Custer JM, 2022, VIROLOGY, V565, P38, DOI 10.1016/j.virol.2021.10.004
D'arc M, 2018, RETROVIROLOGY, V15, DOI 10.1186/s12977-018-0402-9
da Silva JPH, 2023, ARCH VIROL, V168, DOI 10.1007/s00705-023-05852-0
Darriba D, 2011, BIOINFORMATICS, V27, P1164, DOI 10.1093/bioinformatics/btr088
Díez-Villaseñor C, 2019, NAT COMMUN, V10, DOI 10.1038/s41467-018-08167-w
Dixon BR, 2021, RES VET SCI, V135, P283, DOI 10.1016/j.rvsc.2020.09.034
Doore SM, 2016, VIROLOGY, V491, P45, DOI 10.1016/j.virol.2016.01.020
dos Santos RN, 2020, BRAZ J MICROBIOL, V51, P1941, DOI 10.1007/s42770-020-00350-w
Duarte MA, 2019, VIRUSES-BASEL, V11, DOI 10.3390/v11090803
Esposito AM, 2022, MICROORGANISMS, V10, DOI 10.3390/microorganisms10091815
Feng B, 2023, VIRUS RES, V323, DOI 10.1016/j.virusres.2022.199004
Feng YY, 2011, CLIN MICROBIOL REV, V24, P110, DOI 10.1128/CMR.00033-10
Fiallo-Olivé E, 2021, J GEN VIROL, V102, DOI 10.1099/jgv.0.001696
FJ W., 1991, Primatology Today
Fomsgaard AS, 2020, J ZOO AQUAR RES, V8, P253, DOI 10.19227/jzar.v8i4.509
Fontenele RS, 2020, ARCH VIROL, V165, P2891, DOI 10.1007/s00705-020-04801-5
Fontenele RS, 2019, VIRUSES-BASEL, V11, DOI 10.3390/v11080710
Fu LM, 2012, BIOINFORMATICS, V28, P3150, DOI 10.1093/bioinformatics/bts565
GANZHORN JU, 1987, ETHOLOGY, V74, P146
Gogarten JF, 2021, P NATL ACAD SCI USA, V118, DOI 10.1073/pnas.2013535118
GORBALENYA AE, 1990, FEBS LETT, V262, P145, DOI 10.1016/0014-5793(90)80175-I
Greene LK, 2023, ZOO BIOL, V42, P209, DOI 10.1002/zoo.21742
Gronenborn B, 2018, SCI REP-UK, V8, DOI 10.1038/s41598-018-23739-y
Guindon S, 2010, SYST BIOL, V59, P307, DOI 10.1093/sysbio/syq010
HAMLEN HJ, 1994, LAB ANIM SCI, V44, P235
Hao FM, 2021, VIROLOGY, V553, P111, DOI 10.1016/j.virol.2020.11.007
Harding C, 2023, VIROLOGY, V580, P98, DOI 10.1016/j.virol.2023.02.004
Hatcher EL, 2017, NUCLEIC ACIDS RES, V45, pD482, DOI 10.1093/nar/gkw1065
Herrera JP, 2017, INT J PRIMATOL, V38, P692, DOI 10.1007/s10764-017-9974-9
Herrera JP, 2016, J ANIM ECOL, V85, P982, DOI 10.1111/1365-2656.12532
Hess SC, 2024, ARCH VIROL, V169, DOI 10.1007/s00705-023-05937-w

Hrazdilová K, 2016, VIROLOGY, V487, P207, DOI 10.1016/j.virol.2015.10.016
Ilyina TS, 2015, MOL GENET MICROBIOL+, V30, P1, DOI 10.3103/S0891416815010036
ILYINA TV, 1992, NUCLEIC ACIDS RES, V20, P3279, DOI 10.1093/nar/20.13.3279
IUCN, The IUCN Red List of Threatened Species
Jimoh AO, 2023, MICROBIOL RESOUR ANN, V12, DOI 10.1128/mra.01143-22
Kaczorowska J, 2022, J VIROL, V96, DOI 10.1128/jvi.00109-22
Kalishman J, 1996, LAB ANIM SCI, V46, P116
Kapusinszky B, 2017, J VIROL, V91, DOI 10.1128/JVI.00952-17
Kapusinszky B, 2015, J VIROL, V89, P8152, DOI 10.1128/JVI.00671-15
Katoh K, 2013, MOL BIOL EVOL, V30, P772, DOI 10.1093/molbev/mst010
Kazlauskas D, 2018, VIRUSES-BASEL, V10, DOI 10.3390/v10040187
Keeler EL, 2023, CELL HOST MICROBE, V31, P58, DOI 10.1016/j.chom.2022.11.002
Kieft K, 2020, MICROBIOME, V8, DOI 10.1186/s40168-020-00867-0
Kinsella CM, 2022, VIRUS EVOL, V8, DOI 10.1093/ve/veac087
Kinsella CM, 2020, NAT COMMUN, V11, DOI 10.1038/s41467-020-18474-w
Kirchberger PC, 2022, MBIO, V13, DOI 10.1128/mbio.00588-22
Knezevic P, 2021, J GEN VIROL, V102, DOI 10.1099/jgv.0.001614
Kraberger S, 2019, VIRUSES-BASEL, V11, DOI 10.3390/v11060532
Kraberger S, 2019, INFECT GENET EVOL, V71, P179, DOI 10.1016/j.meegid.2019.03.024
Kraberger S, 2017, GENOME ANNOUNCEMENTS, V5, DOI [10.1128/genomeA.00445-17, 10.1128/genomea.00445-17]
Krupovic M, 2022, ARCH VIROL, V167, P2907, DOI 10.1007/s00705-022-05557-w
Krupovic M, 2021, ARCH VIROL, V166, P3245, DOI 10.1007/s00705-021-05224-6
Letunic I, 2021, NUCLEIC ACIDS RES, V49, pW293, DOI 10.1093/nar/gkab301
Levy H, 2020, VIRUSES-BASEL, V12, DOI 10.3390/v12091029
Li DH, 2015, BIOINFORMATICS, V31, P1674, DOI 10.1093/bioinformatics/btv033
Li H, 2019, VIROL J, V16, DOI 10.1186/s12985-019-1211-z
Li LL, 2015, J GEN VIROL, V96, P2721, DOI 10.1099/vir.0.000199
Li LL, 2010, J VIROL, V84, P6955, DOI 10.1128/JVI.00501-10
Li PF, 2020, SCI ADV, V6, DOI 10.1126/sciadv.aay9634
Liang GX, 2021, NAT REV MICROBIOL, V19, P514, DOI 10.1038/s41579-021-00536-5
Lima DA, 2017, J GEN VIROL, V98, P690, DOI 10.1099/jgv.0.000711
Liu AJ, 2023, TRANSBOUND EMERG DIS, V2023, DOI 10.1155/2023/4214812
Lund MC, 2023, VIROLOGY, V585, P42, DOI 10.1016/j.virol.2023.05.006
Male MF, 2016, INFECT GENET EVOL, V39, P279, DOI 10.1016/j.meegid.2016.02.009
Medvedeva S, 2023, NAT MICROBIOL, V8, P2170, DOI 10.1038/s41564-023-01485-w
Minh BQ, 2020, MOL BIOL EVOL, V37, P1530, DOI 10.1093/molbev/msaa015
Minot S, 2011, GENOME RES, V21, P1616, DOI 10.1101/gr.122705.111
Montazeri R, 2016, EUPHYTICA, V210, P31, DOI 10.1007/s10681-016-1693-3
Moraru C, 2020, VIRUSES-BASEL, V12, DOI 10.3390/v12111268
Muhire BM, 2014, PLOS ONE, V9, DOI 10.1371/journal.pone.0108277
Nash TE, 2011, J VIROL, V85, P1182, DOI 10.1128/JVI.02143-10

Nazik H, 2017, MICROBIOL-SGM, V163, P1568, DOI 10.1099/mic.0.000539
Negrey JD, 2022, J ANIM ECOL, V91, P1999, DOI 10.1111/1365-2656.13799
Ng TFF, 2015, VIRUS EVOL, V1, DOI 10.1093/ve/vev017
Nishimura Y, 2017, BIOINFORMATICS, V33, P2379, DOI 10.1093/bioinformatics/btx157
Okamoto H, 2009, CURR TOP MICROBIOL, V331, P35
Olivo D, 2024, MICROORGANISMS, V12, DOI 10.3390/microorganisms12010196
Orton JP, 2020, VIRUSES-BASEL, V12, DOI 10.3390/v12020143
Paietta EN, 2024, VIRUSES-BASEL, V16, DOI 10.3390/v16010037
Paietta EN, 2023, VIRUSES-BASEL, V15, DOI 10.3390/v15091821
Paietta EN, 2023, ARCH VIROL, V168, DOI 10.1007/s00705-022-05679-1
Pearson VM, 2016, PEERJ, V4, DOI 10.7717/peerj.2585
Penner JC, 2016, MICROBIOL-SGM, V162, P1583, DOI 10.1099/mic.0.000344
Phan TG, 2011, PLOS PATHOG, V7, DOI 10.1371/journal.ppat.1002218
Rascovan N, 2016, ANNU REV MICROBIOL, V70, P125, DOI 10.1146/annurev-micro-102215-095431
Rosario K, 2012, J GEN VIROL, V93, P2668, DOI 10.1099/vir.0.045948-0
Roux S, 2023, PLOS BIOL, V21, DOI 10.1371/journal.pbio.3002083
Sawaswong V, 2019, VIRUSES-BASEL, V11, DOI 10.3390/v11100971
Setz E., 1992, P 14 C INT PRIM SOC
Shan TL, 2022, MICROBIOME, V10, DOI 10.1186/s40168-022-01246-7
Shannon P, 2003, GENOME RES, V13, P2498, DOI 10.1101/gr.1239303
Shi Y, 2021, FRONT VET SCI, V8, DOI 10.3389/fvets.2021.695088
Smith K, 2021, VIROLOGY, V559, P156, DOI 10.1016/j.virol.2021.04.004
Stöver BC, 2010, BMC BIOINFORMATICS, V11, DOI 10.1186/1471-2105-11-7
Taylor LJ, 2022, CURR OPIN VIROL, V55, DOI 10.1016/j.coviro.2022.101248
Thomas JE, 2021, J GEN VIROL, V102, DOI 10.1099/jgv.0.001544
Tisza MJ, 2021, VIRUS EVOL, V7, DOI 10.1093/ve/veaa100
Tisza MJ, 2020, ELIFE, V9, DOI 10.7554/eLife.51971
Townsend EM, 2021, FRONT CELL INFECT MI, V11, DOI 10.3389/fcimb.2021.643214
Trubl G, 2023, bioRxiv, DOI [10.1101/2023.06.13.544858, 10.1101/2023.06.13.544858, DOI 10.1101/2023.06.13.544858]
Uchiyama A, 2007, J VIROL, V81, P8587, DOI 10.1128/JVI.00301-07
Varsani A, 2023, ARCH VIROL, V168, DOI 10.1007/s00705-023-05903-6
Varsani A, 2021, ARCH VIROL, V166, P2943, DOI 10.1007/s00705-021-05192-x
Varsani A, 2021, ARCH VIROL, V166, P2911, DOI 10.1007/s00705-021-05183-y
Victoria JG, 2009, J VIROL, V83, P4642, DOI 10.1128/JVI.02301-08
Vieira AC, 2022, FRONT MICROBIOL, V13, DOI 10.3389/fmicb.2022.874319
Villanova F, 2022, VIRUSES-BASEL, V14, DOI 10.3390/v14071472
Villers LM, 2008, AM J PRIMATOL, V70, P175, DOI 10.1002/ajp.20482
Wang H, 2019, VIRUS EVOL, V5, DOI 10.1093/ve/vez013
Witt AA, 2022, VIRUS GENES, V58, P214, DOI 10.1007/s11262-022-01897-6
Xiao WM, 2021, FRONT GENET, V12, DOI 10.3389/fgene.2021.627128
Xiao YQ, 2020, ARCH VIROL, V165, P2847, DOI 10.1007/s00705-020-04808-y
Yang SX, 2022, ENVIRON MICROBIOME, V17, DOI 10.1186/s40793-022-00453-x
Yang SX, 2021, J MED VIROL, V93, P1786, DOI 10.1002/jmv.26524
Yinda CK, 2019, MSPHERE, V4, DOI 10.1128/mSphere.00585-18
Yoder AD, 2007, CURR BIOL, V17, pR866, DOI 10.1016/j.cub.2007.07.050
Yu X, 2010, P NATL ACAD SCI USA, V107, P8387, DOI 10.1073/pnas.0913535107

   Zallot R, 2019, BIOCHEMISTRY-US, V58, P4169, DOI
10.1021/acs.biochem.9b00735
   Zhang T, 2006, PLOS BIOL, V4, P108, DOI 10.1371/journal.pbio.0040003
   Zhang W, 2017, MICROBIOME, V5, DOI 10.1186/s40168-017-0308-0
   Zhang XP, 2020, PLOS ONE, V15, DOI 10.1371/journal.pone.0228673
   Zhang YJ, 2023, MICROORGANISMS, V11, DOI
10.3390/microorganisms11102454
   Zhao GY, 2019, VIROLOGY, V527, P77, DOI 10.1016/j.virol.2018.10.001
NR 144
TC 1
Z9 1
U1 9
U2 9
PU MDPI
PI BASEL
PA ST ALBAN-ANLAGE 66, CH-4052 BASEL, SWITZERLAND
EI 1999-4915
J9 VIRUSES-BASEL
JI Viruses-Basel
PD JUL
PY 2024
VL 16
IS 7
AR 1099
DI 10.3390/v16071099
PG 34
WC Virology
WE Science Citation Index Expanded (SCI-EXPANDED)
SC Virology
GA ZX6T5
UT WOS:001278631200001
PM 39066262
OA Green Published, gold
DA 2025-05-07
ER

PT J
AU Gröschel, MI
   Walker, TM
   van der Werf, TS
   Lange, C
   Niemann, S
   Merker, M
AF Groeschel, Matthias I.
   Walker, Timothy M.
   van der Werf, Tjip S.
   Lange, Christoph
   Niemann, Stefan
   Merker, Matthias
TI Pathogen-based precision medicine for drug-resistant tuberculosis
SO PLOS PATHOGENS
LA English
DT Article
ID BREAKPOINTS; MUTATIONS; DIAGNOSIS

C1 [Groeschel, Matthias I.; Niemann, Stefan; Merker, Matthias] Res Ctr Borstel, Mol & Expt Mycobacteriol, Borstel, Germany.
[Groeschel, Matthias I.; van der Werf, Tjip S.] Univ Groningen, Dept Pulm Dis & TB, Univ Med Ctr Groningen, Groningen, Netherlands.
[Walker, Timothy M.] Univ Oxford, John Radcliffe Hosp, Nuffield Dept Med, Oxford, England.
[Lange, Christoph] Res Ctr Borstel, Clin Infect Dis, Borstel, Germany.
[Lange, Christoph] Univ Lubeck, Int Hlth Infect Dis, Lubeck, Germany.
[Lange, Christoph] Karolinska Inst, Dept Med, Stockholm, Sweden.
[Lange, Christoph; Niemann, Stefan] German Ctr Infect Res DZIF, TB Unit, Borstel, Germany.
C3 Leibniz Association; Forschungszentrum Borstel; University of Groningen;
University of Oxford; Leibniz Association; Forschungszentrum Borstel;
University of Lubeck; Karolinska Institutet; German Center for Infection
Research
RP Gröschel, MI (autor correspondente), Res Ctr Borstel, Mol & Expt Mycobacteriol, Borstel, Germany.; Gröschel, MI (autor correspondente), Univ Groningen, Dept Pulm Dis & TB, Univ Med Ctr Groningen, Groningen, Netherlands.
EM mgroeschel@fz-borstel.de
RI Groeschel, Matthias/AAI-5984-2021; Niemann, Stefan/C-9327-2011; Walker,
Timothy/AAD-5991-2019; van der Werf, Tjip/J-1053-2014; Lange,
Christoph/J-6289-2012; Merker, Matthias/P-7399-2015
OI Niemann, Stefan/0000-0002-6604-0684; Lange,
Christoph/0000-0002-9691-4741; van der Werf, Tjip S/0000-0002-4824-1642;
Merker, Matthias/0000-0003-1386-2331; Walker,
Timothy/0000-0003-0421-9264
FU Leibniz Science Campus Evolutionary Medicine of the Lung (EvoLung)
FX TMW is an NIHR Academic Clinical Lecturer.MM received partial funding
through the Leibniz Science Campus Evolutionary Medicine of the Lung
(EvoLung). The funders had no role in study design, data collection and
analysis, decision to publish, or preparation of the manuscript.
CR Ahuja SD, 2012, PLOS MED, V9, DOI 10.1371/journal.pmed.1001300
Allix-Béguec C, 2018, NEW ENGL J MED, V379, P1403, DOI 10.1056/NEJMoa1800474
Ängeby K, 2012, B WORLD HEALTH ORGAN, V90, P693, DOI 10.2471/BLT.11.096644
Cabibbe AM, 2018, EUR RESPIR J, V51, DOI 10.1183/13993003.00387-2018
Coll F, 2018, NAT GENET, V50, P307, DOI 10.1038/s41588-017-0029-0
Desjardins CA, 2016, NAT GENET, V48, P544, DOI 10.1038/ng.3548
Dheda K, 2017, LANCET RESP MED, V5, P291, DOI [10.1016/S2213-2600(17)30079-6, 10.1016/s2213-2600(17)30079-6]
Doyle RM, 2018, J CLIN MICROBIOL, V56, DOI [10.1128/jcm.00666-18, 10.1128/JCM.00666-18]
Farhat MR, 2017, CLIN INFECT DIS, V65, P1364, DOI 10.1093/cid/cix556
Günther G, 2016, NEW ENGL J MED, V375, P1103, DOI 10.1056/NEJMc1603274
Heyckendorf J, 2018, ANTIMICROB AGENTS CH, V62, DOI 10.1128/AAC.01550-17
Holt KE, 2018, NAT GENET, V50, P849, DOI 10.1038/s41588-018-0117-9

   Hu Yi, 2016, Int J Mycobacteriol, V5 Suppl 1, pS34, DOI
10.1016/j.ijmyco.2016.11.007
   Huyen MNT, 2013, ANTIMICROB AGENTS CH, V57, P3620, DOI
10.1128/AAC.00077-13
   Ling DI, 2008, EUR RESPIR J, V32, P1165, DOI 10.1183/09031936.00061808
   Miotto P, 2017, EUR RESPIR J, V50, DOI 10.1183/13993003.01354-2017
   National Research Council, 2011, PREC MED BUILD KNOWL
   Nimmo C, 2017, INT J INFECT DIS, V62, P44, DOI
10.1016/j.ijid.2017.07.007
   Pankhurst LJ, 2016, LANCET RESP MED, V4, P49, DOI 10.1016/S2213-
2600(15)00466-X
   Pasipanodya JG, 2018, CLIN INFECT DIS, V67, P717, DOI
10.1093/cid/ciy184
   Reed MB, 2009, J CLIN MICROBIOL, V47, P1119, DOI 10.1128/JCM.02142-08
   Rigouts L, 2016, J ANTIMICROB CHEMOTH, V71, P314, DOI
10.1093/jac/dkv360
   Schleusener V, 2017, SCI REP-UK, V7, DOI 10.1038/srep46327
   Schön T, 2009, J ANTIMICROB CHEMOTH, V64, P786, DOI 10.1093/jac/dkp262
   Suliman S, 2018, AM J RESP CRIT CARE, V197, P1198, DOI
10.1164/rccm.201711-2340OC
   World Health Organization, 2018, TUB COUNTR PROF
   Yadon AN, 2017, NAT COMMUN, V8, DOI 10.1038/s41467-017-00721-2
NR 27
TC 44
Z9 45
U1 0
U2 1
PU PUBLIC LIBRARY SCIENCE
PI SAN FRANCISCO
PA 1160 BATTERY STREET, STE 100, SAN FRANCISCO, CA 94111 USA
SN 1553-7366
EI 1553-7374
J9 PLOS PATHOG
JI PLoS Pathog.
PD OCT
PY 2018
VL 14
IS 10
AR e1007297
DI 10.1371/journal.ppat.1007297
PG 7
WC Microbiology; Parasitology; Virology
WE Science Citation Index Expanded (SCI-EXPANDED)
SC Microbiology; Parasitology; Virology
GA GY9PA
UT WOS:000448978700012
PM 30335850
OA Green Published, gold, Green Submitted
DA 2025-05-07
ER

PT J
AU Amoroso, CR
   Hanna, EK

LaBar, KS
Borg, JS
Sinnott-Armstrong, W
Zucker, NL
AF Amoroso, Caroline R.
Hanna, Eleanor K.
LaBar, Kevin S.
Borg, Jana Schaich
Sinnott-Armstrong, Walter
Zucker, Nancy L.
TI Disgust Theory Through the Lens of Psychiatric Medicine
SO CLINICAL PSYCHOLOGICAL SCIENCE
LA English
DT Review
DE disgust; emotion; evolutionary psychology; psychiatry; psychopathology
ID BLOOD-INJECTION-INJURY; POSTTRAUMATIC-STRESS-DISORDER;
INDIVIDUAL-DIFFERENCES; MENTAL POLLUTION; SENSITIVITY; SYMPTOMS;
DOMAINS; PTSD; ANXIETY; EMOTION
AB The elicitors of disgust are heterogeneous, which makes attributing one function to disgust challenging. Theorists have proposed that disgust solves multiple adaptive problems and comprises multiple functional domains. However, theories conflict with regard to what the domains are and how they should be delineated. In this article, we examine clinical evidence of aberrant disgust symptoms in the contamination subtype of obsessive-compulsive disorder, blood-injury-injection phobia, and posttraumatic stress disorder to adjudicate between two prevailing theories of disgust. We argue that the pattern of disgust sensitivities in these psychiatric disorders sheds new light on the domain structure of disgust. Specifically, the supported domain structure of disgust is likely similar to an adaptationist model of disgust, with more subdivisions of the domain of pathogen disgust. We discuss the implications of this approach for the prevention and treatment of psychiatric disorders relevant to disgust.
C1 [Amoroso, Caroline R.] Duke Univ, Dept Evolutionary Anthropol, Durham, NC 27708 USA.
[Hanna, Eleanor K.; LaBar, Kevin S.; Sinnott-Armstrong, Walter] Duke Univ, Ctr Cognit Neurosci, Box 90999, Durham, NC 27708 USA.
[Hanna, Eleanor K.; LaBar, Kevin S.; Zucker, Nancy L.] Duke Univ, Dept Psychol & Neurosci, Durham, NC 27708 USA.
[LaBar, Kevin S.; Zucker, Nancy L.] Duke Univ, Sch Med, Psychiat & Behav Sci, Durham, NC 27708 USA.
[Borg, Jana Schaich] Duke Univ, Social Sci Res Inst, Durham, NC 27708 USA.
[Sinnott-Armstrong, Walter] Duke Univ, Dept Philosophy, Durham, NC 27708 USA.
[Sinnott-Armstrong, Walter] Duke Univ, Kenan Inst Eth, Durham, NC 27708 USA.
C3 Duke University; Duke University; Duke University; Duke University; Duke
University; Duke University; Duke University
RP LaBar, KS (autor correspondente), Duke Univ, Ctr Cognit Neurosci, Box 90999, Durham, NC 27708 USA.
EM klabar@duke.edu
RI Amoroso, Caroline/AAV-7410-2021

OI Schaich Borg, Jana/0000-0002-0066-761X; Amoroso,
Caroline/0000-0001-5456-7975
FU Triangle Center for Evolutionary Medicine; Kenan Institute for Ethics;
Duke Institute for Brain Sciences
FX This project was funded by the Triangle Center for Evolutionary
Medicine, the Kenan Institute for Ethics, and a Research Incubator
Award
from the Duke Institute for Brain Sciences.
CR American Psychiatric Association (APA), 2013, Diagnostic and
statistical manual of mental disorders, V5th, DOI
[10.1176/appi.books.9780890425596.744053, DOI
10.1176/APPI.BOOKS.9780890425596.744053, DOI
10.1176/APPI.BOOKS.9780890425596]
[Anonymous], 2008, Handbook of emotions
[Anonymous], 1997, Psychology and Developing Societies, DOI [DOI
10.1177/097133369700900105, 10.1177/097133369700900105]
[Anonymous], 1968, Diagnostic and statistical manual of mental
disorders, V2nd
Arocho J.M., 2015, Moral disgust as a transdiagnostic feature in
obsessive-compulsive disorder and posttraumatic stress disorder (Doctoral
dissertation, Fordham University)
Athey AJ, 2015, J OBSESS-COMPULS REL, V4, P20, DOI
10.1016/j.jocrd.2014.11.001
Badour CL, 2016, PSYCHOL TRAUMA-US, V8, P267, DOI 10.1037/tra0000079
Badour CL, 2013, COGNITIVE THER RES, V37, P697, DOI 10.1007/s10608-
013-9529-0
Berle D, 2012, J BEHAV THER EXP PSY, V43, P656, DOI
10.1016/j.jbtep.2011.09.002
Bianchi KN, 2012, J ANXIETY DISORD, V26, P753, DOI
10.1016/j.janxdis.2012.06.004
Bomyea J, 2012, CLIN PSYCHOL REV, V32, P630, DOI
10.1016/j.cpr.2012.06.008
Bomyea J, 2012, COGNITIVE THER RES, V36, P190, DOI 10.1007/s10608-010-
9331-1
Brake CA, 2017, J ANXIETY DISORD, V47, P1, DOI
10.1016/j.janxdis.2017.01.003
Capestany BH, 2014, SOC NEUROSCI-UK, V9, P265, DOI
10.1080/17470919.2014.892531
Chapman HA, 2014, EMOTION, V14, P341, DOI 10.1037/a0035120
Cisler JM, 2010, BEHAV RES THER, V48, P74, DOI
10.1016/j.brat.2009.09.003
CONNOLLY J, 1976, BEHAV THER, V7, P8, DOI 10.1016/S0005-7894(76)80214-
6
Curtis V, 2018, PHILOS T R SOC B, V373, DOI 10.1098/rstb.2017.0208
Curtis V, 2011, PHILOS T R SOC B, V366, P3478, DOI
10.1098/rstb.2011.0165
Curtis V, 2011, PHILOS T R SOC B, V366, P389, DOI
10.1098/rstb.2010.0117
de Jong PJ, 1998, PERS INDIV DIFFER, V24, P153, DOI 10.1016/S0191-
8869(97)00178-5
Dewey D, 2014, PSYCHOL REP, V115, P1, DOI 10.2466/16.02.PR0.115c11z7
EKMAN P, 1992, COGNITION EMOTION, V6, P169, DOI
10.1080/02699939208411068

EKMAN P, 1987, J PERS SOC PSYCHOL, V53, P712, DOI 10.1037/0022-3514.53.4.712
Ekman P., 2003, Unmasking the face: A guide to recognizing emotions from facial expressions
Engelhard IM, 2011, J ANXIETY DISORD, V25, P58, DOI 10.1016/j.janxdis.2010.08.003
Fairbrother N, 2005, BEHAV RES THER, V43, P121, DOI 10.1016/j.brat.2003.12.005
Fairbrother N, 2004, BEHAV RES THER, V42, P173, DOI 10.1016/S0005-7967(03)00108-6
Feldner MT, 2010, INT J COGN THER, V3, P11, DOI 10.1521/ijct.2010.3.1.11
FOY DW, 1984, J CONSULT CLIN PSYCH, V52, P79, DOI 10.1037/0022-006X.52.1.79
GARCIA J, 1955, SCIENCE, V122, P157
Gould SJ, 1997, P NATL ACAD SCI USA, V94, P10750, DOI 10.1073/pnas.94.20.10750
HAIDT J, 1994, PERS INDIV DIFFER, V16, P701, DOI 10.1016/0191-8869(94)90212-7
Harris AA, 2019, INT J EAT DISORDER, V52, P543, DOI 10.1002/eat.23047
Harris LT, 2007, SOC COGN AFFECT NEUR, V2, P45, DOI 10.1093/scan/nsl037
Harrison NA, 2010, J NEUROSCI, V30, P12878, DOI 10.1523/JNEUROSCI.1725-10.2010
Hathaway LM, 2010, ANXIETY STRESS COPIN, V23, P119, DOI 10.1080/10615800902818771
Insel T, 2010, AM J PSYCHIAT, V167, P748, DOI 10.1176/appi.ajp.2010.09091379
Kelly D., 2011, Yuck! The nature and moral significance of disgust, DOI [10.7551/mitpress/8303.001.0001, DOI 10.7551/MITPRESS/8303.001.0001]
Krzanowska E, 2017, ARCH PSYCHIATR PSYCH, V19, P7, DOI 10.12740/APP/76150
Kupfer TR, 2018, EMOTION, V18, P959, DOI 10.1037/emo0000395
Lieberman D., 2018, OBJECTION DISGUST MO
McKay D, 2006, J BEHAV THER EXP PSY, V37, P53, DOI 10.1016/j.jbtep.2005.09.005
McKay D., 2009, Disgust and Its Disorders, P211
McKeever VM, 2003, REV GEN PSYCHOL, V7, P237, DOI 10.1037/1089-2680.7.3.237
MEEHL PE, 1962, AM PSYCHOL, V17, P827, DOI 10.1037/h0041029
Melli G, 2015, INT J COGN THER, V8, P193, DOI 10.1521/ijct_2015_8_01
Moretz MW, 2008, J ANXIETY DISORD, V22, P707, DOI 10.1016/j.janxdis.2007.07.004
Nesse R M, 1990, Hum Nat, V1, P261, DOI 10.1007/BF02733986
Nesse R.M., 1994, WHY WE GET SICK NEW
Nesse RM, 2001, WESTERN J MED, V174, P358, DOI 10.1136/ewjm.174.5.358
Nussbaum MarthaC., 2010, From Disgust to Humanity: Sexual Orientation and Constitutional Law
O'Donnell ML, 2010, PSYCHIAT RES, V176, P257, DOI 10.1016/j.psychres.2009.05.012
Ojserkis R, 2014, BEHAV MODIF, V38, P801, DOI 10.1177/0145445514543465
Olatunji B.O., 2009, DISGUST ITS DISORDER
Olatunji BO, 2006, J BEHAV THER EXP PSY, V37, P16, DOI 10.1016/j.jbtep.2005.09.002

Olatunji BO, 2006, J ANXIETY DISORD, V20, P23, DOI 10.1016/j.janxdis.2004.11.009
Olatunji BO, 2005, J SOC CLIN PSYCHOL, V24, P932, DOI 10.1521/jscp.2005.24.7.932
Olatunji BO, 2005, BEHAV RES THER, V43, P1069, DOI 10.1016/j.brat.2004.08.002
Olatunji BO, 2005, PERS INDIV DIFFER, V38, P891, DOI 10.1016/j.paid.2004.06.012
Olatunji BO, 2004, BEHAV RES THER, V42, P93, DOI 10.1016/S0005-7967(03)00102-5
Olatunji BO, 2008, J RES PERS, V42, P1243, DOI 10.1016/j.jrp.2008.03.009
Olatunji BO, 2007, PSYCHOL ASSESSMENT, V19, P281, DOI 10.1037/1040-3590.19.3.281
Olatunji BO, 2007, J PSYCHOPATHOL BEHAV, V29, P115, DOI 10.1007/s10862-006-9027-8
Olatunji BO, 2007, BRIT J PSYCHIAT, V190, P457, DOI 10.1192/bjp.bp.106.032631
Olatunji BO, 2007, BEHAV RES THER, V45, P263, DOI 10.1016/j.brat.2006.03.004
Olatunji BO, 2017, PERSPECT PSYCHOL SCI, V12, P613, DOI 10.1177/1745691616688879
Olatunji BO, 2016, J ANXIETY DISORD, V44, P63, DOI 10.1016/j.janxdis.2016.10.010
Olatunji BO, 2015, PSYCHOL ASSESSMENT, V27, P102, DOI 10.1037/pas0000039
Olatunji BO, 2014, BEHAV THER, V45, P469, DOI 10.1016/j.beth.2014.02.006
Olatunji BO, 2010, BEHAV THER, V41, P475, DOI 10.1016/j.beth.2009.11.005
Olatunji BO, 2009, J ANXIETY DISORD, V23, P1091, DOI 10.1016/j.janxdis.2009.07.012
OST LG, 1992, J ABNORM PSYCHOL, V101, P68, DOI 10.1037/0021-843X.101.1.68
Page AC, 2003, J ANXIETY DISORD, V17, P45, DOI 10.1016/S0887-6185(02)00169-X
PAGE AC, 1994, CLIN PSYCHOL REV, V14, P443, DOI 10.1016/0272-7358(94)90036-1
Phillips ML, 1998, BRIT J PSYCHIAT, V172, P373, DOI 10.1192/bjp.172.5.373
Poli A, 2019, BEHAV THER, V50, P380, DOI 10.1016/j.beth.2018.07.006
Power MJ, 2013, BEHAV COGN PSYCHOTH, V41, P162, DOI 10.1017/S1352465812000148
Rottman J., 2018, MORAL PSYCHOL DISGUS, P27
ROZIN P, 1987, PSYCHOL REV, V94, P23, DOI 10.1037/0033-295X.94.1.23
Rozin P, 2013, TRENDS COGN SCI, V17, P367, DOI 10.1016/j.tics.2013.06.001
Sawchuk CN, 2000, BEHAV RES THER, V38, P753, DOI 10.1016/S0005-7967(99)00093-5
Sawchuk CN, 2002, BEHAV RES THER, V40, P1031, DOI 10.1016/S0005-7967(01)00093-6
Schnall S, 2008, PERS SOC PSYCHOL B, V34, P1096, DOI 10.1177/0146167208317771
Shin LM, 1999, AM J PSYCHIAT, V156, P575

Simon E, 2017, PSYCHOPHYSIOLOGY, V54, P1512, DOI 10.1111/psyp.12900
Tolin DF, 1997, BEHAV RES THER, V35, P949, DOI 10.1016/S0005-7967(97)00048-X
Tolin DF, 2006, J BEHAV THER EXP PSY, V37, P30, DOI 10.1016/j.jbtep.2005.09.003
Tolin DF, 2004, J BEHAV THER EXP PSY, V35, P193, DOI 10.1016/j.jbtep.2004.04.009
Tooby J, 1985, EMERGING SYNTHESES S, P1, DOI DOI 10.1109/LMWC.2010.2104138
Tooby J., 2008, HDB EMOTIONS, Vthird, P114, DOI DOI 10.2307/2076468
Tybur JM, 2013, PSYCHOL REV, V120, P65, DOI 10.1037/a0030778
Tybur JM, 2009, J PERS SOC PSYCHOL, V97, P103, DOI 10.1037/a0015474
van Delft I, 2016, J TRAUMA STRESS, V29, P581, DOI 10.1002/jts.22118
van Overveld WJM, 2006, PERS INDIV DIFFER, V41, P1241, DOI 10.1016/j.paid.2006.04.021
Viar MA, 2010, J ANXIETY DISORD, V24, P941, DOI 10.1016/j.janxdis.2010.06.021
VRANA SR, 1993, PSYCHOPHYSIOLOGY, V30, P279, DOI 10.1111/j.1469-8986.1993.tb03354.x
Weidmann A, 2009, ANXIETY STRESS COPIN, V22, P549, DOI 10.1080/10615800802541986
Woody SR, 2002, J ANXIETY DISORD, V16, P543, DOI 10.1016/S0887-6185(02)00173-1
NR 100
TC 11
Z9 13
U1 2
U2 17
PU SAGE PUBLICATIONS INC
PI THOUSAND OAKS
PA 2455 TELLER RD, THOUSAND OAKS, CA 91320 USA
SN 2167-7026
EI 2167-7034
J9 CLIN PSYCHOL SCI
JI Clin. Psychol. Sci.
PD JAN
PY 2020
VL 8
IS 1
BP 3
EP 24
AR 2167702619863769
DI 10.1177/2167702619863769
EA NOV 2019
PG 22
WC Psychology, Clinical; Psychiatry; Psychology
WE Science Citation Index Expanded (SCI-EXPANDED); Social Science Citation Index (SSCI)
SC Psychology; Psychiatry
GA KD1PM
UT WOS:000496068900001
DA 2025-05-07
ER

PT J
AU Hopperstad, KA
   Reiskind, MH
   Labadie, PE
   Reiskind, MOB
AF Hopperstad, Kristen A.
   Reiskind, Michael H.
   Labadie, Paul E.
   Reiskind, Martha O. Burford
TI Patterns of genetic divergence among populations of Aedes
   aegypti L. (Diptera: Culicidae) in the southeastern USA
SO PARASITES & VECTORS
LA English
DT Article
DE Aedes aegypti; Genetic structure; Competitive exclusion; Satyrization
ID HABITAT SEGREGATION; ALBOPICTUS DIPTERA; SOUTH FLORIDA; SOFTWARE;
   PHYLOGEOGRAPHY; SATYRIZATION; COEXISTENCE; COMPETITION; MOSQUITO;
   VECTORS
AB Background: The yellow fever mosquito, Aedes aegypti is a public health concern in the USA, especially in the wake of emergent diseases such as Zika and chikungunya. Aedes aegypti populations dwindled after the invasion of Aedes albopictus in the 1980s and many populations were extirpated. However, in some areas Ae. aegypti persisted in small populations and there are reports of recent resurgences of Ae. aegypti in Florida, Louisiana, Nevada and California. We assessed the population genetic structure of Ae. aegypti in Florida and Georgia, which has concomitant consequences related to mosquito dispersal, pesticide resistance and vectorial capacity.

   Methods: We collected Ae. aegypti across Florida and in Georgia using ovitraps. We hatched the eggs and reared them to adults, and after sacrifice we extracted their DNA. We then probed each individual for variation in 6 microsatellite markers, which we used to address population genetic characteristics.

   Results: We collected Ae. aegypti and genotyped seven Florida populations and one Georgia population using microsatellite markers. We found evidence of isolation by distance model of gene flow supported by driving distance among cities within Florida and two theoretic genetic clusters.

   Conclusions: Significant genetic structure between some populations with substantial gene flow between geographically distant cities suggests regional genetic structuring of Ae. aegypti in Florida. This study provides information on the genetic exchange between populations of Ae. aegypti in the southeastern USA and suggests potential routes of spread of this species.
C1 [Hopperstad, Kristen A.; Reiskind, Michael H.; Labadie, Paul E.] North Carolina State Univ, Dept Entomol & Plant Pathol, Raleigh, NC 27695 USA.
   [Reiskind, Martha O. Burford] North Carolina State Univ, Dept Biol Sci, Raleigh, NC 27695 USA.
C3 North Carolina State University; North Carolina State University
RP Reiskind, MOB (autor correspondente), North Carolina State Univ, Dept Biol Sci, Raleigh, NC 27695 USA.
EM mbreiski@ncsu.edu
RI Reiskind, Michael/I-5614-2012; (Hopperstad) Breaux, Kristen/U-4728-2018

OI (Hopperstad) Breaux, Kristen/0000-0002-3622-153X; Burford Reiskind, Martha/0000-0001-6826-9215
FU National Science Foundation Graduate Research Fellowship [DGE-1252376];
Triangle Center for Evolutionary Medicine
FX This material is based upon work supported by the National Science Foundation Graduate Research Fellowship under Grant No. (DGE-1252376) and the Triangle Center for Evolutionary Medicine.
CR Bargielowski I, 2014, EVOL ECOL, V28, P193, DOI 10.1007/s10682-013-9669-4
Bargielowski IE, 2013, P NATL ACAD SCI USA, V110, P2888, DOI 10.1073/pnas.1219599110
Beerli P, 2006, BIOINFORMATICS, V22, P341, DOI 10.1093/bioinformatics/bti803
Beerli P, 2001, P NATL ACAD SCI USA, V98, P4563, DOI 10.1073/pnas.081068098
Beerli P, 2010, GENETICS, V185, P313, DOI 10.1534/genetics.109.112532
BLAND JM, 1995, BRIT MED J, V310, P170, DOI 10.1136/bmj.310.6973.170
Bradburd GS, 2018, GENETICS, V210, P33, DOI 10.1534/genetics.118.301333
Braks MAH, 2003, J MED ENTOMOL, V40, P785, DOI 10.1603/0022-2585-40.6.785
Brown JE, 2011, P ROY SOC B-BIOL SCI, V278, P2446, DOI 10.1098/rspb.2010.2469
Brown JE, 2013, J MED ENTOMOL, V50, P294, DOI 10.1603/ME12173
Calvez E, 2016, PLOS NEGLECT TROP D, V10, DOI 10.1371/journal.pntd.0004374
CDC, 2018, DIV VECT BORN DIS
CHAKRABORTY R, 1992, HUM GENET, V88, P267, DOI 10.1007/BF00197257
da Silva AG, 2012, EVOL APPL, V5, P664, DOI 10.1111/j.1752-4571.2012.00244.x
Damal K, 2013, AM J TROP MED HYG, V89, P482, DOI 10.4269/ajtmh.13-0102
Earl DA, 2012, CONSERV GENET RESOUR, V4, P359, DOI 10.1007/s12686-011-9548-7
Evanno G, 2005, MOL ECOL, V14, P2611, DOI 10.1111/j.1365-294X.2005.02553.x
Gloria-Soria A, 2016, MED VET ENTOMOL, V30, P235, DOI 10.1111/mve.12153
Gloria-Soria A, 2016, MOL ECOL, V25, P5377, DOI 10.1111/mec.13866
Gloria-Soria A, 2014, PLOS NEGLECT TROP D, V8, DOI 10.1371/journal.pntd.0003029
Hemme RR, 2010, PLOS NEGLECT TROP D, V4, DOI 10.1371/journal.pntd.0000634
Hopperstad KA, 2016, J MED ENTOMOL, V53, P836, DOI 10.1093/jme/tjw050
Hotez PJ, 2016, PLOS NEGLECT TROP D, V10, DOI 10.1371/journal.pntd.0003895
Huber K, 2004, ACTA TROP, V90, P23, DOI 10.1016/j.actatropica.2003.09.012
Hubisz MJ, 2009, MOL ECOL RESOUR, V9, P1322, DOI 10.1111/j.1755-0998.2009.02591.x
Jombart T, 2008, BIOINFORMATICS, V24, P1403, DOI 10.1093/bioinformatics/btn129
Juliano SA, 2002, OECOLOGIA, V130, P458, DOI 10.1007/s004420100811

Juliano SA, 2010, ISR J ECOL EVOL, V56, P325, DOI 10.1560/IJEE.55.3-4.325
Juliano SA, 2009, ANNU REV ENTOMOL, V54, P37, DOI 10.1146/annurev.ento.54.110807.090611
Kalinowski ST, 2005, MOL ECOL NOTES, V5, P187, DOI 10.1111/j.1471-8286.2004.00845.x
Kamvar ZN, 2014, PEERJ, V2, DOI 10.7717/peerj.281
Kaplan L, 2010, BIOL INVASIONS, V12, P3277, DOI 10.1007/s10530-010-9721-z
KASS RE, 1995, J AM STAT ASSOC, V90, P773, DOI 10.1080/01621459.1995.10476572
Kopelman NM, 2015, MOL ECOL RESOUR, V15, P1179, DOI 10.1111/1755-0998.12387
Lounibos LP, 2018, BIOL INVASIONS, V20, P1913, DOI 10.1007/s10530-018-1674-7
Lounibos LP, 2016, J MED ENTOMOL, V53, P1385, DOI 10.1093/jme/tjw122
Lounibos LP, 2002, ANNU REV ENTOMOL, V47, P233, DOI 10.1146/annurev.ento.47.091201.145206
Lovin DD, 2009, BMC GENOMICS, V10, DOI 10.1186/1471-2164-10-590
Merrill SA, 2005, AM J TROP MED HYG, V72, P304, DOI 10.4269/ajtmh.2005.72.304
MOORE CG, 1988, J AM MOSQUITO CONTR, V4, P356
MORLAN HB, 1965, AM J TROP MED HYG, V14, P892, DOI 10.4269/ajtmh.1965.14.892
OMEARA GF, 1995, J MED ENTOMOL, V32, P554, DOI 10.1093/jmedent/32.4.554
Padmanabha H, 2012, PLOS NEGLECT TROP D, V6, DOI 10.1371/journal.pntd.0001799
Peakall R, 2012, BIOINFORMATICS, V28, P2537, DOI 10.1093/bioinformatics/bts460
Pritchard JK, 2000, GENETICS, V155, P945
Rasheed SB, 2013, MED VET ENTOMOL, V27, P430, DOI 10.1111/mve.12001
Reiskind MH, 2013, MED VET ENTOMOL, V27, P421, DOI 10.1111/mve.12000
Reiskind MH, 2009, MED VET ENTOMOL, V23, P62, DOI 10.1111/j.1365-2915.2008.00782.x
Reiskind MOB, 2016, MOL ECOL RESOUR, V16, P1303, DOI 10.1111/1755-0998.12527
Reiskind MOB, 2018, MOL ECOL, V27, P3641, DOI 10.1111/mec.14821
REITER P, 1995, AM J TROP MED HYG, V52, P177, DOI 10.4269/ajtmh.1995.52.177
Rey JR, 2006, J MED ENTOMOL, V43, P1134, DOI 10.1603/0022-2585(2006)43[1134:HSOMAV]2.0.CO;2
Rousset F, 2008, MOL ECOL RESOUR, V8, P103, DOI 10.1111/j.1471-8286.2007.01931.x
Slotman MA, 2007, MOL ECOL NOTES, V7, P168, DOI 10.1111/j.1471-8286.2006.01533.x
Tripet F, 2011, AM J TROP MED HYG, V85, P265, DOI 10.4269/ajtmh.2011.10-0677
Van Oosterhout C, 2004, MOL ECOL NOTES, V4, P535, DOI 10.1111/j.1471-8286.2004.00684.x
NR 56
TC 7
Z9 8
U1 1

U2 7
PU BMC
PI LONDON
PA CAMPUS, 4 CRINAN ST, LONDON N1 9XW, ENGLAND
SN 1756-3305
J9 PARASITE VECTOR
JI Parasites Vectors
PD OCT 30
PY 2019
VL 12
IS 1
AR 511
DI 10.1186/s13071-019-3769-0
PG 9
WC Parasitology; Tropical Medicine
WE Science Citation Index Expanded (SCI-EXPANDED)
SC Parasitology; Tropical Medicine
GA JT1TA
UT WOS:000500779100007
PM 31666113
OA Green Published, gold
DA 2025-05-07
ER

PT J
AU Estes, S
   Phillips, PC
   Denver, DR
AF Estes, Suzanne
   Phillips, Patrick C.
   Denver, Dee R.
TI FITNESS RECOVERY AND COMPENSATORY EVOLUTION IN NATURAL MUTANT LINES OF
   C. ELEGANS
SO EVOLUTION
LA English
DT Article
DE Adaptation; Caenorhabditis; epistasis; experimental evolution;
mutation
   accumulation; reversion
ID LIFE-HISTORY TRAITS; CAENORHABDITIS-ELEGANS; LINKAGE DISEQUILIBRIUM;
   MUTATION ACCUMULATION; POPULATION EXTINCTION; ADAPTATION; GENETICS;
   COST; PLEIOTROPY; EPISTASIS
AB Deleterious mutation accumulation plays a central role in evolutionary
genetics, conservation biology, human health, and evolutionary medicine
(e.g., methods of viral attenuation for live vaccines). It is therefore
important to understand whether and how quickly populations with
accumulated deleterious mutational loads can recover fitness through
adaptive evolution. We used laboratory experimental evolution with four
long-term mutation-accumulation (MA) lines of Caenorhabditis elegans
nematodes to study the dynamics of such fitness evolution. We previously
showed that when homozygous mutant populations are evolved in large
population sizes, they can rapidly achieve wild-type fitness through the
accumulation of new beneficial or compensatory epistatic mutations. Here,
we expand this approach to demonstrate that when replicate lineages are

initiated from the same mutant genotype, phenotypic evolution is only sometimes repeatable. MA genotypes that recovered ancestral fitness in the previous experiment did not always do so here. Further, the pattern of adaptive evolution in independently evolved replicates was contingent upon the MA genotype and varied among fitness-related traits. Our findings suggest that new beneficial mutations can drive rapid fitness evolution, but that the adaptive process is rendered somewhat unpredictable by its susceptibility to chance events and sensitivity to the evolutionary history of the starting population.
C1 [Estes, Suzanne] Portland State Univ, Dept Biol, Portland, OR 97201 USA.
[Phillips, Patrick C.] Univ Oregon, Dept Biol, Eugene, OR 97403 USA.
[Phillips, Patrick C.] Univ Oregon, Ctr Ecol & Evolut, Eugene, OR 97403 USA.
[Denver, Dee R.] Oregon State Univ, Dept Zool, Corvallis, OR 97331 USA.
[Denver, Dee R.] Oregon State Univ, Ctr Genome Res & Biocomp, Corvallis, OR 97331 USA.
C3 Portland State University; University of Oregon; University of Oregon; Oregon State University; Oregon State University
RP Estes, S (autor correspondente), Portland State Univ, Dept Biol, Portland, OR 97201 USA.
EM estess@pdx.edu
OI Phillips, Patrick/0000-0001-7271-342X
FU NSF [DEB-062521, DEB-0743871, DEB-0236180, DEB-0641066]; PSU Faculty
FX We thank L. Albergotti, J. Anderson, K. Hicks, S. Martha, C. Palmer, and T. Tague for laboratory assistance, and B. Ajie, C. Baer, J. Bull, H. Teotonio, and H. Wichman for helpful discussions. Also, our sincere thanks to A. Agrawal, A. Cutter, and an anonymous reviewer for their insightful comments on the manuscript. This work was supported by NSF grants DEB-062521 to SE and DEB-0743871 to SE and DRD, PSU Faculty Enhancement Grant to SE, and NSF grants DEB-0236180 and DEB-0641066 to PCP.
CR Ajie BC, 2005, GENETICS, V170, P655, DOI 10.1534/genetics.104.040014
[Anonymous], 1999, BIOSTAT ANAL
Barrière A, 2007, GENETICS, V176, P999, DOI 10.1534/genetics.106.067223
Burch CL, 2000, NATURE, V406, P625, DOI 10.1038/35020564
Charlesworth B, 1994, EVOLUTION AGE STRUCT
Cooper TF, 2007, EVOLUTION, V61, P1495, DOI 10.1111/j.1558-5646.2007.00109.x
Cowperthwaite MC, 2008, PLOS COMPUT BIOL, V4, DOI 10.1371/journal.pcbi.1000110
Cutter AD, 2006, GENETICS, V172, P171, DOI 10.1534/genetics.105.048207
Denver DR, 2010, GENOME RES, V20, P1663, DOI 10.1101/gr.108191.110
Denver DR, 2009, P NATL ACAD SCI USA, V106, P16310, DOI 10.1073/pnas.0904895106
Denver DR, 2003, MOL BIOL EVOL, V20, P393, DOI 10.1093/molbev/msg044
Denver DR, 2004, NATURE, V430, P679, DOI 10.1038/nature02697
Denver DR, 2000, SCIENCE, V289, P2342, DOI 10.1126/science.289.5488.2342
Dolgin ES, 2007, EVOLUTION, V61, P1339, DOI 10.1111/j.1558-5646.2007.00118.x

Elena SF, 2003, NAT REV GENET, V4, P457, DOI 10.1038/nrg1088
Estes S, 2005, GENETICS, V170, P645, DOI 10.1534/genetics.104.040022
Estes S, 2003, EVOLUTION, V57, P1022, DOI 10.1111/j.0014-3820.2003.tb00313.x
Estes S, 2006, EVOLUTION, V60, P2655, DOI 10.1111/j.0014-3820.2006.tb01897.x
Fisher R.A., 1930, The Genetical Theory of Natural Selection-A Complete Variorum Edition
Gavrilets S, 1997, TRENDS ECOL EVOL, V12, P307, DOI 10.1016/S0169-5347(97)01098-7
Haag ES, 2007, GENETICA, V129, P45, DOI 10.1007/s10709-006-0032-3
Jones AG, 2007, EVOLUTION, V61, P727, DOI 10.1111/j.1558-5646.2007.00071.x
Keightley PD, 2000, GENETICS, V156, P143
Keightley PD, 2000, SCIENCE, V290, P331, DOI 10.1126/science.290.5490.331
Keightley PD, 2006, NATURE, V443, P89, DOI 10.1038/nature05049
Kimura M, 1985, J GENET, V64, P7, DOI [10.1007/BF02923549, DOI 10.1007/BF02923549]
LANDE R, 1994, EVOLUTION, V48, P1460, DOI 10.1111/j.1558-5646.1994.tb02188.x
Lenormand T, 2009, TRENDS ECOL EVOL, V24, P157, DOI 10.1016/j.tree.2008.09.014
Lynch M, 1999, EVOLUTION, V53, P645, DOI 10.1111/j.1558-5646.1999.tb05361.x
LYNCH M, 1995, AM NAT, V146, P489, DOI 10.1086/285812
Lynch M., 2007, ORIGINS GENOME ARCHI
Martin G, 2006, EVOLUTION, V60, P893
Moore FBG, 2000, P ROY SOC B-BIOL SCI, V267, P515, DOI 10.1098/rspb.2000.1030
Morran LT, 2009, NATURE, V462, P350, DOI 10.1038/nature08496
Orr HA, 2008, AM NAT, V172, P160, DOI 10.1086/589460
Orr HA, 2005, EVOLUTION, V59, P216
Orr HA, 2000, EVOLUTION, V54, P13, DOI 10.1111/j.0014-3820.2000.tb00002.x
Orr HA, 1999, GENET RES, V74, P207, DOI 10.1017/S0016672399004164
Orr HA, 1998, EVOLUTION, V52, P935, DOI 10.1111/j.1558-5646.1998.tb01823.x
Phillips PC, 2008, NAT REV GENET, V9, P855, DOI 10.1038/nrg2452
Phillips PC, 1996, GENET RES, V67, P271, DOI 10.1017/S0016672300033759
Phillips PC., 2000, EPISTASIS EVOLUTIONA, P20
Poon A, 2005, GENETICS, V170, P1323, DOI 10.1534/genetics.104.037259
Poon A, 2000, EVOLUTION, V54, P1467, DOI 10.1111/j.0014-3820.2000.tb00693.x
Rockman MV, 2009, PLOS GENET, V5, DOI 10.1371/journal.pgen.1000419
Rozen DE, 2008, PLOS ONE, V3, DOI 10.1371/journal.pone.0001715
Sanjuán R, 2005, GENETICS, V170, P1001, DOI 10.1534/genetics.105.040741
Silander OK, 2007, PLOS BIOL, V5, P922, DOI 10.1371/journal.pbio.0050094
Stern DL, 2009, SCIENCE, V323, P746, DOI 10.1126/science.1158997
Teotónio H, 2002, J EVOLUTION BIOL, V15, P608, DOI 10.1046/j.1420-9101.2002.00424.x

   Teotónio H, 2001, EVOLUTION, V55, P653, DOI 10.1554/0014-
3820(2001)055[0653:PRE]2.0.CO;2
   TRAVISANO M, 1995, SCIENCE, V267, P87, DOI 10.1126/science.7809610
   VASI F, 1994, AM NAT, V144, P432, DOI 10.1086/285685
   Vassilieva LL, 2000, EVOLUTION, V54, P1234, DOI 10.1111/j.0014-
3820.2000.tb00557.x
   WAGNER GP, 1990, EVOLUTION, V44, P715, DOI 10.1111/j.1558-
5646.1990.tb05950.x
   Wagner GP, 2008, NATURE, V452, P470, DOI 10.1038/nature06756
   Weinreich DM, 2006, SCIENCE, V312, P111, DOI 10.1126/science.1123539
   Welch JJ, 2003, EVOLUTION, V57, P1723, DOI 10.1554/02-673
   Whitlock MC, 2003, ANN ZOOL FENN, V40, P169
   Whitlock MC, 2000, EVOLUTION, V54, P1855, DOI 10.1111/j.0014-
3820.2000.tb01232.x
NR 60
TC 20
Z9 28
U1 1
U2 35
PU WILEY
PI HOBOKEN
PA 111 RIVER ST, HOBOKEN 07030-5774, NJ USA
SN 0014-3820
EI 1558-5646
J9 EVOLUTION
JI Evolution
PD AUG
PY 2011
VL 65
IS 8
BP 2335
EP 2344
DI 10.1111/j.1558-5646.2011.01276.x
PG 10
WC Ecology; Evolutionary Biology; Genetics & Heredity
WE Science Citation Index Expanded (SCI-EXPANDED)
SC Environmental Sciences & Ecology; Evolutionary Biology; Genetics &
   Heredity
GA 798DL
UT WOS:000293182700016
PM 21790579
DA 2025-05-07
ER

PT J
AU Leonti, M
   Staub, PO
   Cabras, S
   Castellanos, ME
   Casu, L
AF Leonti, Marco
   Staub, Peter O.
   Cabras, Stefano
   Eugenia Castellanos, Maria

Casu, Laura
TI From cumulative cultural transmission to evidence-based medicine: evolution of medicinal plant knowledge in Southern Italy
SO FRONTIERS IN PHARMACOLOGY
LA English
DT Article
DE traditional medical knowledge; cultural transmission and evolution; causal effect; evidence-based; herbal medicine; historical ethnopharmacology; De Materia Medica; globalization
ID TRADITIONAL PHYTOTHERAPY; FOLK MEDICINE; NATIONAL-PARK; CAMPANIA; ETHNOPHARMACOLOGY; SARDINIA; PERSPECTIVES; MECHANISMS; DISTRICT;
PLACEBO
AB In Mediterranean cultures written records of medicinal plant use have a long tradition. This written record contributed to building a consensus about what was perceived to be an efficacious pharmacopeia. Passed down through millennia, these scripts have transmitted knowledge about plant uses, with high fidelity, to scholars and laypersons alike. Herbal medicine's importance and the long-standing written record call for a better understanding of the mechanisms influencing the transmission of contemporary medicinal plant knowledge. Here we contextualize herbal medicine within evolutionary medicine and cultural evolution. Cumulative knowledge transmission is approached by estimating the causal effect of two seminal scripts about materia rnedica written by Dioscorides and Galen, two classical Greco-Roman physicians, on today's medicinal plant use in the Southern Italian regions of Campania, Sardinia, and Sicily. Plant-use combinations are treated as transmissible cultural traits (or "memes"), which in analogy to the biological evolution of genetic traits, are subjected to mutation and selection. Our results suggest that until today ancient scripts have exerted a strong influence on the use of herbal medicine. We conclude that the repeated empirical testing and scientific study of health care claims is guiding and shaping the selection of efficacious treatments and evidence-based herbal medicine.
C1 [Leonti, Marco; Staub, Peter O.] Univ Cagliari, Dept Biomed Sci, Cagliari, Italy.
[Cabras, Stefano] Univ Cagliari, Dept Math & Informat, Cagliari, Italy.
[Cabras, Stefano] Univ Carlos III Madrid, Dept Stat, E-28903 Getafe, Spain.
[Eugenia Castellanos, Maria] Rey Juan Carlos Univ, Dept Informat & Stat, Mostoles, Spain.
[Casu, Laura] Univ Cagliari, Dept Life & Environm Sci, Cagliari, Italy.
C3 University of Cagliari; University of Cagliari; Universidad Carlos III de Madrid; Universidad Rey Juan Carlos; University of Cagliari
RP Leonti, M (autor correspondente), Univ Cagliari, Dept Biomed Sci, Via Osped 72, Cagliari, Italy.
EM marcoleonti@netscape.net
RI Castellanos, María/E-9851-2016; Cabras, Stefano/AAA-2477-2019
OI Cabras, Stefano/0000-0001-6690-8378; Casu, Laura/0000-0001-6480-8680
FU People Programme (Marie Curie Actions) of the European Union's 7th Framework programme FP7 under REA [PITN-GA-2013-606895-MedPlant]
FX The research leading to these results has received funding from the People Programme (Marie Curie Actions) of the European Union's 7th Framework programme FP7/2007/2013 under REA grant agreement no

PITN-GA-2013-606895-MedPlant.
CR Amico F. P., 1997, Fitoterapia, V68, P143
[Anonymous], POPOLAMENTO VEGETALE
[Anonymous], QUADERNI BOT AMBIENT
[Anonymous], 1995, ACTA TECHNOL LEGIS M
ANTONONE R, 1988, J ETHNOPHARMACOL, V22, P295, DOI 10.1016/0378-8741(88)90240-1
Arber A., 1953, BOTANY
Arcidiacono S., 1999, Quad. Bot. Ambient. Appl, V10, P105
Arcidiacono S., 2007, Quad. Bot. Ambient. Appl, V18, P103
Atzei A.D., 1991, Boll Soc Sarda Di Scienze Nat, V28, P137
Atzei AD, 1994, Le piante nelle terapie tradizionali. Sardegna sud-occidentale
Ballero M., 1993, Fitoterapia, V64, P141
Ballero M, 2001, FITOTERAPIA, V72, P788, DOI 10.1016/S0367-326X(01)00334-3
BALLERO M, 1991, Fitoterapia, V62, P524
Ballero M, 1998, FITOTERAPIA, V69, P52
Ballero M., 1994, Annali Di Botanica, V52, P489
Ballero M., 1997, ACTA PHYTOTERAPEUTIC, V2, P23
Ballero M., 1982, REND SEM FS U CAGLIA, V52, P115
Ballero M., 1998, Atti Della Soc. Toscana Sci. Nat. Mem. Ser. B, V105, P83
Barbagallo C., 2004, B ACCAD GIOENIA SCI, V37, P83
BARBAGALLO C, 1979, FITOTERAPIA, V50, P56
Barbrook AC, 1998, NATURE, V394, P839, DOI 10.1038/29667
Brown A., 2014, ETHNOBOT RES APPL, V12, P571
Bruni A, 1997, J ETHNOPHARMACOL, V57, P97, DOI 10.1016/S0378-8741(97)00055-X
BYE R, 1995, RECENT ADV PHYTOCHEM, V29, P65
Camarda I., 1990, BOLL SOC SARDA SCI N, V27, P147
Cardoso GC, 2011, EVOLUTION, V65, P295, DOI 10.1111/j.1558-5646.2010.01102.x
Catanzaro F., 1970, FITOTERAPIA, V2, P66
CAVALLI-SFORZA L L, 1982, Science (Washington D C), V218, P19, DOI 10.1126/science.7123211
Cavalli-Sforza LL, 1981, MONOGRAPHS POPULATIO
Chipman HA, 2010, ANN APPL STAT, V4, P266, DOI 10.1214/09-AOAS285
Cosmacini G., 2009, ARTE LUNGA STORIA ME
Dal Cero M, 2014, J ETHNOPHARMACOL, V151, P253, DOI 10.1016/j.jep.2013.10.035
Dawkins R., 2016, The selfish gene, V40th
De Natale A, 2007, J ETHNOPHARMACOL, V109, P295, DOI 10.1016/j.jep.2006.07.038
DEFEO V, 1993, J ETHNOPHARMACOL, V39, P39, DOI 10.1016/0378-8741(93)90049-B
DEFEO V, 1992, J ETHNOPHARMACOL, V36, P113, DOI 10.1016/0378-8741(92)90010-O
DEFEO V, 1991, FITOTERAPIA, V63, P337
Di Novella R, 2013, J ETHNOPHARMACOL, V145, P328, DOI 10.1016/j.jep.2012.10.065
Di Sanzo P, 2013, J ETHNOBIOL ETHNOMED, V9, DOI 10.1186/1746-4269-9-19
Diamond JM, 2005, Guns, Germs & Steel: The Fate of Human Societies

Dobson Annette J., 2008, An Introduction to Generalized Linear Models, DOI DOI 10.2307/1269239
Etkin NinaL., 2006, Edible Medicines: An Ethnopharmacology of Food
Etkin NL, 2001, J ETHNOPHARMACOL, V76, P177, DOI 10.1016/S0378-8741(01)00232-X
ETKIN NL, 1988, ANNU REV ANTHROPOL, V17, P23, DOI 10.1146/annurev.an.17.100188.000323
Galenus C., 1561, CLAUDII GALENI SIMPL
GALT AH, 1978, ECON BOT, V32, P20, DOI 10.1007/BF02906726
Guarino C., 2008, Ethnobotany Research and Applications, V6, P255
GUGLIELMINO CR, 1995, P NATL ACAD SCI USA, V92, P7585, DOI 10.1073/pnas.92.16.7585
Hallpike C., 1988, PRINCIPLES SOCIAL EV
Hastie T., 2009, ELEMENTS STAT LEARNI, DOI [DOI 10.1007/978-0-387-84858-7, 10.1007/978-0-387-84858-7]
Heinrich M.B.J., 2004, FUNDAMENTALS PHARMAC, V1st
Heinrich M, 2006, J ETHNOPHARMACOL, V107, P157, DOI 10.1016/j.jep.2006.05.035
Henrich J, 1998, EVOL HUM BEHAV, V19, P215, DOI 10.1016/S1090-5138(98)00018-X
HEWLETT BS, 1986, AM ANTHROPOL, V88, P922
Hill JL, 2011, J COMPUT GRAPH STAT, V20, P217, DOI 10.1198/jcgs.2010.08162
Ilardi V., 1992, Quad. Bot. Ambient. Appl, V3, P41
Kronfeldner ME, 2007, BIOL PHILOS, V22, P493, DOI 10.1007/s10539-006-9037-7
Laland KN, 2006, TRENDS ECOL EVOL, V21, P542, DOI 10.1016/j.tree.2006.06.005
Lehmann L, 2010, J EVOLUTION BIOL, V23, P2356, DOI 10.1111/j.1420-9101.2010.02096.x
LENTINI F, 1996, ATT CONV GENZ SPEC A, P117
Lentini F., 1990, Quad. Bot. Ambient. Appl, V1, P103
Lentini F., 1997, ACTA PHYTOTERAPEUTIC, V4, P88
Lentini F., 1991, ATTI ACCAD SCI LETT, V11, P67
Lentini F, 1987, ATTI ACC SC LETT AR, P1
Lentini F., 1996, NATURA, P33
Lentini F., 1987, URBAN, V60, P151
Leonti M, 2013, FRONT PHARMACOL, V4, DOI 10.3389/fphar.2013.00092
Leonti M, 2011, J ETHNOPHARMACOL, V134, P542, DOI 10.1016/j.jep.2011.01.017
Leonti M, 2010, J ETHNOPHARMACOL, V130, P379, DOI 10.1016/j.jep.2010.05.021
Leonti M, 2009, J ETHNOPHARMACOL, V121, P55, DOI 10.1016/j.jep.2008.10.027
Leslie Charles., 1976, Asian Medical Systems, P1
Leto C, 2013, J ETHNOPHARMACOL, V146, P90, DOI 10.1016/j.jep.2012.11.042
Lewis HM, 2012, PHILOS T R SOC B, V367, P2171, DOI 10.1098/rstb.2012.0119
Loi M. C., 2005, Journal of Herbs, Spices & Medicinal Plants, V11, P67, DOI 10.1300/J044v11n03_07
Loi MC, 2004, FITOTERAPIA, V75, P277, DOI 10.1016/j.fitote.2004.01.008
Mann R.D., 1984, Modern drug use: An enquiry on historical principles
Matthioli A., 1967, DISCORSI MPA MATTHIO, P1568

Mesoudi A, 2004, EVOLUTION, V58, P1
Mesoudi A, 2013, STRUNGMANN FORUM REP, P193
Moerman DE, 2002, ANN INTERN MED, V136, P471, DOI 10.7326/0003-4819-136-6-200203190-00011
Motti R, 2009, HUM ECOL, V37, P775, DOI 10.1007/s10745-009-9254-1
Napoli M., 2002, Boll. Accad. Gioenia Sci. Nat, V35, P361
Nunn CL, 2010, PHILOS T R SOC B, V365, P3807, DOI 10.1098/rstb.2010.0009
O'Brien MJ, 2010, PHILOS T R SOC B, V365, P3797, DOI 10.1098/rstb.2010.0012
Pagel M, 2004, NATURE, V428, P275, DOI 10.1038/428275a
Palmer L.R., 1977, LA LINGUA LATINA
Palmese MT, 2001, FITOTERAPIA, V72, P619, DOI 10.1016/S0367-326X(01)00288-X
Pennisi E, 2010, SCIENCE, V328, P165, DOI 10.1126/science.328.5975.165
Pollio A, 2008, J ETHNOPHARMACOL, V116, P469, DOI 10.1016/j.jep.2007.12.013
Porretta A, 2003, INFECTION, V31, P16, DOI 10.1007/s15010-003-8229-1
Preuss J., 1971, BIBLISCH TALMUDISCHE
Raimondo F.M., 1990, Nat. Sicil, V14, P77
Reader's Digest, 1980, SEGR VIRT PIANT MED
Rendell L, 2010, SCIENCE, V328, P208, DOI 10.1126/science.1184719
Reyes-García V, 2009, EVOL HUM BEHAV, V30, P274, DOI 10.1016/j.evolhumbehav.2009.02.001
Riddle J.M., 1985, DIOSCORIDEPHARM ME
Rief W, 2011, CLIN PHARMACOL THER, V90, P722, DOI 10.1038/clpt.2011.204
Saitta A., 1967, CIVILTA PASSATO MOND, VII
Savo V, 2011, J ETHNOPHARMACOL, V135, P376, DOI 10.1016/j.jep.2011.03.027
Scherrer AM, 2005, J ETHNOPHARMACOL, V97, P129, DOI 10.1016/j.jep.2004.11.002
Signorini MA, 2009, J ETHNOBIOL ETHNOMED, V5, DOI 10.1186/1746-4269-5-6
Singer Charles., 1927, J HELLENIC STUD, V47, P1, DOI DOI 10.2307/625251
Soldati GT, 2015, PLOS ONE, V10, DOI 10.1371/journal.pone.0126389
Strimling P, 2009, P NATL ACAD SCI USA, V106, P13870, DOI 10.1073/pnas.0903180106
Tanaka M. M., 2009, PLOS ONE, V4, pe5192, DOI [10.1371/journal.pone.0005192, DOI 10.1371/JOURNAL.PONE.0005192]
Tennie C, 2009, PHILOS T R SOC B, V364, P2405, DOI 10.1098/rstb.2009.0052
Tiemersma EW, 2004, EMERG INFECT DIS, V10, P1627, DOI 10.3201/eid1009.040069
Tomasello M., 1999, The cultural origins of human cognition
Totelin Laurence M V, 2009, Stud Anc Med, V34, P1
URDANG G, 1951, Bull World Health Organ, V4, P577
NR 110
TC 20
Z9 21
U1 1
U2 35
PU FRONTIERS MEDIA SA

PI LAUSANNE
PA AVENUE DU TRIBUNAL FEDERAL 34, LAUSANNE, CH-1015, SWITZERLAND
SN 1663-9812
J9 FRONT PHARMACOL
JI Front. Pharmacol.
PD SEP 30
PY 2015
VL 6
AR 207
DI 10.3389/fphar.2015.00207
PG 15
WC Pharmacology & Pharmacy
WE Science Citation Index Expanded (SCI-EXPANDED); Social Science Citation Index (SSCI)
SC Pharmacology & Pharmacy
GA CX3TC
UT WOS:000365621200001
PM 26483686
OA Green Published, gold
DA 2025-05-07
ER

PT J
AU Tesicky, M
   Vinkler, M
AF Tesicky, Martin
   Vinkler, Michal
TI Trans-Species Polymorphism in Immune Genes: General Pattern or
   MHC-Restricted Phenomenon?
SO JOURNAL OF IMMUNOLOGY RESEARCH
LA English
DT Review
ID MAJOR HISTOCOMPATIBILITY COMPLEX; CLASS-I GENES; SELF-INCOMPATIBILITY
   LOCUS; TERM BALANCING SELECTION; HOST-DEFENSE PEPTIDES; MATING-TYPE
   LOCUS; NATURAL-SELECTION; HINGE REGION; ANTIMICROBIAL PEPTIDES;
   RECOGNITION RECEPTORS
AB Immunity exhibits extraordinarily high levels of variation. Evolution of the immune system in response to host-pathogen interactions in particular ecological contexts appears to be frequently associated with diversifying selection increasing the genetic variability. Many studies have documented that immunologically relevant polymorphism observed today may be tens of millions years old and may predate the emergence of present species. This pattern can be explained by the concept of trans-species polymorphism (TSP) predicting the maintenance and sharing of favourable functionally important alleles of immune-related genes between species due to ongoing balancing selection. Despite the generality of this concept explaining the long-lasting adaptive variation inherited from ancestors, current research in TSP has vastly focused only on major histocompatibility complex (MHC). In this review we summarise the evidence available on TSP in human and animal immune genes to reveal that TSP is not a MHC-specific evolutionary pattern. Further research should clearly pay more attention to the investigation of TSP in innate immune genes and especially pattern recognition receptors which are promising candidates for this type of evolution. More effort should also be made to

distinguish TSP from convergent evolution and adaptive introgression. Identification of balanced TSP variants may represent an accurate approach in evolutionary medicine to recognise disease-resistance alleles.
C1 [Tesicky, Martin; Vinkler, Michal] Charles Univ Prague, Fac Sci, Dept Zool, CR-12844 Prague, Czech Republic.
C3 Charles University Prague
RP Vinkler, M (autor correspondente), Charles Univ Prague, Fac Sci, Dept Zool, Vinicna 7, CR-12844 Prague, Czech Republic.
EM michal.vinkler@natur.cuni.cz
RI Tesicky, Martin/HTR-4769-2023; Tesicky, Martin/L-3370-2017; Vinkler, Michal/G-2072-2011
OI Tesicky, Martin/0000-0001-8097-5331; Vinkler, Michal/0000-0003-3572-9494
FU Czech Science Foundation [P506/15-11782S]; Grant Agency of the Charles University [540214]; Institutional Research Support [SVV-260 208/2015]
FX The authors are grateful to Lenka Kubickova and Karel Kodejs and three anonymous reviewers for their helpful comments on the paper and Dagmar Vinklerova for language corrections. This study was supported by the Czech Science Foundation (Grant no. P506/15-11782S), by the Grant Agency of the Charles University (Grant no. 540214), and through the Institutional Research Support (SVV-260 208/2015).
CR Acevedo-Whitehouse K, 2006, TRENDS ECOL EVOL, V21, P433, DOI 10.1016/j.tree.2006.05.010
Aguilar A, 2007, J MOL EVOL, V65, P34, DOI 10.1007/s00239-006-0222-8
Alcaide M, 2011, MOL BIOL EVOL, V28, P1703, DOI 10.1093/molbev/msq351
ARDEN B, 1982, P NATL ACAD SCI-BIOL, V79, P2342, DOI 10.1073/pnas.79.7.2342
Ballingall KT, 2010, PLOS ONE, V5, DOI 10.1371/journal.pone.0011402
Bernatchez L, 2003, J EVOLUTION BIOL, V16, P363, DOI 10.1046/j.1420-9101.2003.00531.x
Bos DH, 2006, MOL BIOL EVOL, V23, P137, DOI 10.1093/molbev/msj016
Bowdish DME, 2005, CURR PROTEIN PEPT SC, V6, P35, DOI 10.2174/1389203053027494
Braida L, 2004, AIDS, V18, P1598, DOI 10.1097/01.aids.0000131363.82951.fb
Buhler S, 2011, PLOS ONE, V6, DOI 10.1371/journal.pone.0014643
Cagliani R, 2010, HUM GENET, V128, P577, DOI 10.1007/s00439-010-0884-6
Cooke GS, 2001, NAT REV GENET, V2, P967, DOI 10.1038/35103577
Cuperus T, 2013, DEV COMP IMMUNOL, V41, P352, DOI 10.1016/j.dci.2013.04.019
de Craen AJM, 2005, GENES IMMUN, V6, P167, DOI 10.1038/sj.gene.6364162
DWYER KG, 1991, PLANT MOL BIOL, V16, P481, DOI 10.1007/BF00024000
Edwards SV, 1998, TRENDS ECOL EVOL, V13, P305, DOI 10.1016/S0169-5347(98)01416-5
Esteves PJ, 2005, IMMUNOGENETICS, V57, P874, DOI 10.1007/s00251-005-0022-0
FEHLING HJ, 1994, SCIENCE, V265, P1234, DOI 10.1126/science.8066463
Ferguson W, 2008, MOL BIOL EVOL, V25, P1609, DOI 10.1093/molbev/msn106
Ferguson W, 2012, MOL BIOL EVOL, V29, P1093, DOI 10.1093/molbev/msr247
Ferrer-Admetlla A, 2008, J IMMUNOL, V181, P1315, DOI 10.4049/jimmunol.181.2.1315

Ferwerda B, 2007, P NATL ACAD SCI USA, V104, P16645, DOI 10.1073/pnas.0704828104
Fornuskova A, 2014, ECOL EVOL, V4, P2931, DOI 10.1002/ece3.1137
Ganz T, 2003, NAT REV IMMUNOL, V3, P710, DOI 10.1038/nri1180
Glaberman S, 2008, IMMUNOGENETICS, V60, P371, DOI 10.1007/s00251-008-0298-y
Go Y, 2002, IMMUNOGENETICS, V54, P403, DOI 10.1007/s00251-002-0480-6
GRANT PR, 1992, SCIENCE, V256, P193, DOI 10.1126/science.256.5054.193
Grossen C, 2014, PLOS GENET, V10, DOI 10.1371/journal.pgen.1004438
Hedrick PW, 2013, MOL ECOL, V22, P4606, DOI 10.1111/mec.12415
Hedrick PW, 2002, EVOLUTION, V56, P1902, DOI 10.1111/j.0014-3820.2002.tb00116.x
Heimpel GE, 2008, ANNU REV ENTOMOL, V53, P209, DOI 10.1146/annurev.ento.53.103106.093441
Hellgren O, 2011, MOL ECOL RESOUR, V11, P686, DOI 10.1111/j.1755-0998.2011.02995.x
Hollox EJ, 2008, BMC EVOL BIOL, V8, DOI 10.1186/1471-2148-8-113
HORNG WJ, 1980, MOL IMMUNOL, V17, P111, DOI 10.1016/0161-5890(80)90130-3
Hovanessian AG, 2007, BIOCHIMIE, V89, P779, DOI 10.1016/j.biochi.2007.02.003
Hughes AL, 1998, ANNU REV GENET, V32, P415, DOI 10.1146/annurev.genet.32.1.415
IOERGER TR, 1990, P NATL ACAD SCI USA, V87, P9732, DOI 10.1073/pnas.87.24.9732
Janova E, 2009, IMMUNOGENETICS, V61, P513, DOI 10.1007/s00251-009-0380-0
Jaratlerdsiri W, 2014, PLOS ONE, V9, DOI 10.1371/journal.pone.0087534
Jaratlerdsiri W, 2014, IMMUNOGENETICS, V66, P53, DOI 10.1007/s00251-013-0746-1
Jeffery KJM, 2000, MICROBES INFECT, V2, P1335, DOI 10.1016/S1286-4579(00)01287-9
Johnson WE, 2009, IMMUNOGENETICS, V61, P163, DOI 10.1007/s00251-009-0358-y
Kamath PL, 2011, BMC EVOL BIOL, V11, DOI 10.1186/1471-2148-11-128
KAWAMURA S, 1990, J MOL BIOL, V215, P201, DOI 10.1016/S0022-2836(05)80336-5
Kermarrec N, 1999, IMMUNOGENETICS, V49, P517, DOI 10.1007/s002510050529
Key FM, 2014, CURR OPIN GENET DEV, V29, P45, DOI 10.1016/j.gde.2014.08.001
King RC., 2006, A Dictionary of Genetics, V7th
Kiryu I, 2005, FISH SHELLFISH IMMUN, V18, P243, DOI 10.1016/j.fsi.2004.07.007
KLEIN J, 1987, HUM IMMUNOL, V19, P155, DOI 10.1016/0198-8859(87)90066-8
Klein J, 1998, ANNU REV ECOL SYST, V29, P1, DOI 10.1146/annurev.ecolsys.29.1.1
Klein J., 1980, Immunology, V80, P239
Klein J, 2007, ANNU REV GENET, V41, P281, DOI 10.1146/annurev.genet.41.110306.130137
Kriener K, 2000, IMMUNOGENETICS, V51, P169, DOI 10.1007/s002510050028
Kriener K, 2001, HUM IMMUNOL, V62, P1, DOI 10.1016/S0198-8859(00)00233-0

Kundu S, 2007, BIOL J LINN SOC, V91, P493, DOI 10.1111/j.1095-8312.2007.00814.x
KUPFERMANN H, 1992, HUM IMMUNOL, V34, P267, DOI 10.1016/0198-8859(92)90026-J
Lechner S, 2014, MOL BIOL EVOL, V31, P272, DOI 10.1093/molbev/mst207
Lee MS, 2007, ANNU REV BIOCHEM, V76, P447, DOI 10.1146/annurev.biochem.76.060605.122847
Leffler EM, 2013, SCIENCE, V339, P1578, DOI 10.1126/science.1234070
Li L, 2011, J MOL EVOL, V72, P474, DOI 10.1007/s00239-011-9446-3
Loisel DA, 2006, P NATL ACAD SCI USA, V103, P16331, DOI 10.1073/pnas.0607662103
Lukens L, 1996, GENETICS, V144, P1471
MALE CJ, 1979, INFECT IMMUN, V26, P254, DOI 10.1128/IAI.26.1.254-261.1979
Mansour SC, 2014, TRENDS IMMUNOL, V35, P443, DOI 10.1016/j.it.2014.07.004
MARTINKO JM, 1993, IMMUNOGENETICS, V37, P274
Mashimo T, 2002, P NATL ACAD SCI USA, V99, P11311, DOI 10.1073/pnas.172195399
MAYER WE, 1988, EMBO J, V7, P2765, DOI 10.1002/j.1460-2075.1988.tb03131.x
Meyer D, 2001, ANN HUM GENET, V65, P1, DOI 10.1046/j.1469-1809.2001.6510001.x
Milinski M, 2006, ANNU REV ECOL EVOL S, V37, P159, DOI 10.1146/annurev.ecolsys.37.091305.110242
Miura F, 2010, P NATL ACAD SCI USA, V107, P21599, DOI 10.1073/pnas.1012881107
Mogensen TH, 2009, CLIN MICROBIOL REV, V22, P240, DOI 10.1128/CMR.00046-08
Muirhead CA, 2002, GENETICS, V161, P633
Nadachowska-Brzyska K, 2012, MOL ECOL, V21, P887, DOI 10.1111/j.1365-294X.2011.05347.x
Nagl S, 1998, P NATL ACAD SCI USA, V95, P14238, DOI 10.1073/pnas.95.24.14238
Neefjes J, 2011, NAT REV IMMUNOL, V11, P823, DOI 10.1038/nri3084
Newman RM, 2006, P NATL ACAD SCI USA, V103, P19134, DOI 10.1073/pnas.0605838103
Nonaka M, 2000, IMMUNOGENETICS, V51, P186, DOI 10.1007/s002510050030
Noro M, 2014, IMMUNOGENETICS, V66, P325, DOI 10.1007/s00251-014-0767-4
Orrù V, 2013, CELL, V155, P242, DOI 10.1016/j.cell.2013.08.041
Otting N, 2002, IMMUNOGENETICS, V54, P230, DOI 10.1007/s00251-002-0461-9
Ottová E, 2005, FISH SHELLFISH IMMUN, V18, P199, DOI 10.1016/j.fsi.2004.07.004
Penn DJ, 1999, AM NAT, V153, P145, DOI 10.1086/303166
Perelygin AA, 2002, P NATL ACAD SCI USA, V99, P9322, DOI 10.1073/pnas.142287799
Pinheiro A, 2011, IMMUNOGENETICS, V63, P397, DOI 10.1007/s00251-011-0533-9
Piontkivska H, 2003, MOL BIOL EVOL, V20, P601, DOI 10.1093/molbev/msg064
Reche PA, 2003, J MOL BIOL, V331, P623, DOI 10.1016/S0022-2836(03)00750-2

Richman AD, 1996, SCIENCE, V273, P1212, DOI 10.1126/science.273.5279.1212
Samonte IE, 2007, MOL BIOL EVOL, V24, P2069, DOI 10.1093/molbev/msm138
Sanchez-Mazas A, 2014, J IMMUNOL RES, V2014, DOI 10.1155/2014/971818
Sato A, 2011, MOL BIOL EVOL, V28, P1943, DOI 10.1093/molbev/msr015
SchmidHempel P, 2011, EVOLUTIONARY PARASITOLOGY: THE INTEGRATED STUDY OF INFECTIONS, IMMUNOLOGY, ECOLOGY, AND GENETICS, P1
Ségurel L, 2012, P NATL ACAD SCI USA, V109, P18493, DOI 10.1073/pnas.1210603109
Simková A, 2013, PLOS ONE, V8, DOI 10.1371/journal.pone.0065883
Sommer Simone, 2005, Frontiers in Zoology, V2, P1, DOI 10.1186/1742-9994-2-16
Sorci G, 1997, TRENDS ECOL EVOL, V12, P196, DOI 10.1016/S0169-5347(97)01056-2
Spurgin LG, 2010, P ROY SOC B-BIOL SCI, V277, P979, DOI 10.1098/rspb.2009.2084
Stremlau M, 2004, NATURE, V427, P848, DOI 10.1038/nature02343
Su C, 1999, P NATL ACAD SCI USA, V96, P9710, DOI 10.1073/pnas.96.17.9710
Sumiyama K, 1998, ANTHROPOL SCI, V106, P31, DOI 10.1537/ase.106.31
Sumiyama K, 2002, MOL BIOL EVOL, V19, P1093, DOI 10.1093/oxfordjournals.molbev.a004167
Takahashi K, 2000, J HERED, V91, P198, DOI 10.1093/jhered/91.3.198
TAKAHATA N, 1990, P NATL ACAD SCI USA, V87, P2419, DOI 10.1073/pnas.87.7.2419
Takeuchi O, 2010, CELL, V140, P805, DOI 10.1016/j.cell.2010.01.022
Tennessen JA, 2008, MOL BIOL EVOL, V25, P2669, DOI 10.1093/molbev/msn208
Trowsdale J, 2011, IMMUNOL LETT, V137, P1, DOI 10.1016/j.imlet.2011.01.002
Tsukamoto K, 2012, MOL BIOL EVOL, V29, P3071, DOI 10.1093/molbev/mss113
van Diepen LTA, 2013, MOL BIOL EVOL, V30, P2286, DOI 10.1093/molbev/mst126
van Oosterhout C, 2009, P ROY SOC B-BIOL SCI, V276, P657, DOI 10.1098/rspb.2008.1299
Vincek V, 1997, P ROY SOC B-BIOL SCI, V264, P111, DOI 10.1098/rspb.1997.0017
Vinkler M, 2015, GENETICA, V143, P101, DOI 10.1007/s10709-015-9819-4
Vinkler M, 2014, GENET SEL EVOL, V46, DOI 10.1186/s12711-014-0072-6
Vyas JM, 2008, NAT REV IMMUNOL, V8, P607, DOI 10.1038/nri2368
Wallny HJ, 2006, P NATL ACAD SCI USA, V103, P1434, DOI 10.1073/pnas.0507386103
Wang DQ, 2010, FEBS LETT, V584, P3331, DOI 10.1016/j.febslet.2010.05.065
Wang GS, 2009, NUCLEIC ACIDS RES, V37, pD933, DOI 10.1093/nar/gkn823
Wegner KM, 2012, MOL ECOL, V21, P779, DOI 10.1111/j.1365-294X.2011.05401.x
Wegner KM, 2004, MICROBES INFECT, V6, P1110, DOI 10.1016/j.micinf.2004.05.025
Woolhouse MEJ, 2002, NAT GENET, V32, P569, DOI 10.1038/ng1202-569
Xu SX, 2009, J MOL EVOL, V69, P260, DOI 10.1007/s00239-009-9272-z
Yeager M, 1999, IMMUNOL REV, V167, P45, DOI 10.1111/j.1600-065X.1999.tb01381.x

Zhao MA, 2013, BMC EVOL BIOL, V13, DOI 10.1186/1471-2148-13-113
NR 121
TC 55
Z9 58
U1 0
U2 35
PU HINDAWI LTD
PI LONDON
PA ADAM HOUSE, 3RD FLR, 1 FITZROY SQ, LONDON, W1T 5HF, ENGLAND
SN 2314-8861
EI 2314-7156
J9 J IMMUNOL RES
JI J Immunol. Res.
PY 2015
VL 2015
AR 838035
DI 10.1155/2015/838035
PG 10
WC Immunology
WE Science Citation Index Expanded (SCI-EXPANDED)
SC Immunology
GA CK1FG
UT WOS:000355951500001
PM 26090501
OA Green Published, gold, Green Submitted
DA 2025-05-07
ER

PT J
AU Nunn, R
AF Nunn, Robin
TI Many-models medicine: diversity as the best medicine
SO JOURNAL OF EVALUATION IN CLINICAL PRACTICE
LA English
DT Article
DE agent-based simulation; evidence-based medicine; expertise; medical epistemology; models of medicine; unification of science
ID SYSTEMS; SCIENCE
AB Many medicines have been proposed to cure various ills of biomedicine including evidence-based medicine, evolutionary medicine, narrative medicine, and complexity medicine, among others. To the extent that all models are idealizations or abstractions, all of these model medicines are imperfect in some respects. In the absence of a single unified model, if indeed unification is possible or even desirable, and despite the relative advantages of one model or another, in practice many models and methods are necessary in medicine. In this article, I consider the value of such diversity in models and methods. I briefly describe several models. Then I discuss simulations of agents who use diverse models. Advocates of models such as those discussed here typically claim that we should use their preferred model because it is the best. Evidence-based medicine, for instance, has been promoted as the single best model of medicine while other models have been cast as lesser models or in opposition to it and each other. But isolated models and methods may never be as good as groups of models and methods. Debates about various

individual models may result in better outcomes, but explicitly choosing to use many models is likely to produce even better outcomes.
C1 Univ Toronto, IHPST, Victoria Coll, Toronto, ON M5S 1K7, Canada.
C3 University of Toronto; Victoria University Toronto
RP Nunn, R (autor correspondente), Univ Toronto, IHPST, Victoria Coll, 91 Charles St W, Toronto, ON M5S 1K7, Canada.
EM r.nunn@utoronto.ca
CR [Anonymous], 2007, DIFFERENCE POWER DIV, DOI DOI 10.1126/science.1121066
Castillo O., 2008, DEP TECHNICAL REPORT, V119
Charon R, 2008, LANCET, V371, P296, DOI 10.1016/S0140-6736(08)60156-7
ENGEL GL, 1977, SCIENCE, V196, P129, DOI 10.1126/science.847460
GOULD SJ, 1979, PROC R SOC SER B-BIO, V205, P581, DOI 10.1098/rspb.1979.0086
Greenhalgh T, 1999, BMJ-BRIT MED J, V318, P323, DOI 10.1136/bmj.318.7179.323
Greenhalgh T., 1998, Narrative Based Medicine: dialogue and discourse in clinical practice
HARDIN G, 1968, SCIENCE, V162, P1243, DOI 10.1126/science.162.3859.1243
Hong L, 2004, P NATL ACAD SCI USA, V101, P16385, DOI 10.1073/pnas.0403723101
Jadad A.R., 2007, Randomised Controlled Trials: Questions, Answers, and Musings, V2nd
KLEINMAN A, 1978, SOC SCI MED-MED ANTH, V12, P85, DOI 10.1016/0160-7987(78)90014-5
Krause S, 2011, ANIM BEHAV, V81, P941, DOI 10.1016/j.anbehav.2010.12.018
Liker A, 2009, P NATL ACAD SCI USA, V106, P7893, DOI 10.1073/pnas.0900042106
Mayo-Wilson C, 2011, PHILOS SCI, V78, P653, DOI 10.1086/661777
MERTON RK, 1984, J HIST BEHAV SCI, V20, P319, DOI 10.1002/1520-6696(198410)20:4<319::AID-JHBS2300200402>3.0.CO;2-3
Miles A, 2009, J EVAL CLIN PRACT, V15, P409, DOI 10.1111/j.1365-2753.2009.01204.x
Moretto E. N., 2010, COCHRANE DB SYST REV
Morrison M, 2011, STUD HIST PHILOS SCI, V42, P342, DOI 10.1016/j.shpsa.2010.11.042
Norman CD, 2011, AM J EVAL, V32, P70, DOI 10.1177/1098214010379038
Nunn R.J., 2007, Emergence: Complexity and Organization, V9, P93
Nunn R, 2011, J EVAL CLIN PRACT, V17, P920, DOI 10.1111/j.1365-2753.2011.01727.x
Queen's University News, 2012, QUEENS U NEWS
Rauhut H, 2011, J MATH PSYCHOL, V55, P191, DOI 10.1016/j.jmp.2010.10.002
Rueger A, 2005, BRIT J PHILOS SCI, V56, P579, DOI 10.1093/bjps/axi128
Silva SA, 2011, J EVAL CLIN PRACT, V17, P585, DOI 10.1111/j.1365-2753.2010.01551.x
Simons AM, 2004, TRENDS ECOL EVOL, V19, P453, DOI 10.1016/j.tree.2004.07.001
Stokols D, 2008, AM J PREV MED, V35, pS77, DOI 10.1016/j.amepre.2008.05.002
Sumpter DJT, 2008, CURR BIOL, V18, P1773, DOI 10.1016/j.cub.2008.09.064

   Teller P, 2001, ERKENNTNIS, V55, P393, DOI 10.1023/A:1013349314515
   Ward AJW, 2011, P NATL ACAD SCI USA, V108, P2312, DOI
10.1073/pnas.1007102108
   Zollman KJS, 2010, ERKENNTNIS, V72, P17, DOI 10.1007/s10670-009-9194-6
NR 31
TC 5
Z9 6
U1 0
U2 12
PU WILEY
PI HOBOKEN
PA 111 RIVER ST, HOBOKEN 07030-5774, NJ USA
SN 1356-1294
EI 1365-2753
J9 J EVAL CLIN PRACT
JI J. Eval. Clin. Pract.
PD OCT
PY 2012
VL 18
IS 5
BP 974
EP 978
DI 10.1111/j.1365-2753.2012.01903.x
PG 5
WC Health Care Sciences & Services; Medical Informatics; Medicine,
General
   & Internal
WE Science Citation Index Expanded (SCI-EXPANDED); Social Science
Citation Index (SSCI)
SC Health Care Sciences & Services; Medical Informatics; General &
Internal
   Medicine
GA 010BQ
UT WOS:000309069800008
PM 22994994
DA 2025-05-07
ER

PT J
AU Greenspoon, PB
   Mideo, N
AF Greenspoon, Philip B.
   Mideo, Nicole
TI Evolutionary rescue of a parasite population by mutation rate
evolution
SO THEORETICAL POPULATION BIOLOGY
LA English
DT Article
DE Mutation rate; Parasites; Evolutionary rescue; Drug resistance;
Modifier
   model
ID EXTINCTION; RESISTANCE; HYPERMUTATION; PLASTICITY; DYNAMICS
AB The risk of antibiotic resistance evolution in parasites is a major
problem for public health. Identifying factors which promote antibiotic

resistance evolution is thus a priority in evolutionary medicine. The rate at which new mutations enter the parasite population is one important predictor; however, mutation rate is not necessarily a fixed quantity, as is often assumed, but can itself evolve. Here we explore the possible impacts of mutation rate evolution on the fate of a disease circulating in a host population, which is being treated with drugs, the use of which varies over time. Using an evolutionary rescue framework, we find that mutation rate evolution provides a dramatic increase in the probability that a parasite population survives treatment in only a limited region, while providing little or no advantage in other regions. Both epidemiological features, such as the virulence of infection, and population genetic parameters, such as recombination rate, play important roles in determining the probability of evolutionary rescue and whether mutation rate evolution enhances the probability of evolutionary rescue or not. While efforts to curtail mutation rate evolution in parasites may be worthwhile under some circumstances, our results suggest that this need not always be the case. (C) 2017 Elsevier Inc. All rights reserved.
C1 [Greenspoon, Philip B.; Mideo, Nicole] Univ Toronto, Dept Ecol & Evolutionary Biol, Toronto, ON M5S 3B2, Canada.
C3 University of Toronto
RP Greenspoon, PB (autor correspondente), Univ Toronto, Dept Ecol & Evolutionary Biol, Toronto, ON M5S 3B2, Canada.
EM philip.greenspoon@utoronto.ca
RI Greenspoon, Philip/I-9180-2019; Mideo, Nicole/F-4520-2010
OI Mideo, Nicole/0000-0002-8719-3620; Greenspoon, Philip/0000-0001-6284-7248
FU Natural Sciences and Engineering Research Council of Canada [436171]; Canada Foundation for Innovation under Compute Canada; Government of Ontario, Ontario Research Fund - Research Excellence; University of Toronto
FX We thank Aneil Agrawal and Leithen M'Gonigle for comments on this manuscript, and the Natural Sciences and Engineering Research Council of Canada for funding (N.M., Discovery Grant 436171). Computations were performed on the GPC supercomputer at the SciNet HPC Consortium (Loken et al., 2010). SciNet is funded by the Canada Foundation for Innovation under the auspices of Compute Canada, the Government of Ontario, Ontario Research Fund - Research Excellence, and the University of Toronto.
CR Alexander HK, 2014, EVOL APPL, V7, P1161, DOI 10.1111/eva.12221
Andersson DI, 2014, NAT REV MICROBIOL, V12, P465, DOI 10.1038/nrmicro3270
Ashander J., 2016, P ROY SOC LOND B BIO, V283, P1
Auerbach A, 2015, J CYST FIBROS, V14, P347, DOI 10.1016/j.jcf.2014.09.011
Baym M, 2016, SCIENCE, V351, DOI 10.1126/science.aad3292
Bergstrom CT, 2004, P NATL ACAD SCI USA, V101, P13285, DOI 10.1073/pnas.0402298101
Blanquart F., 2017, P ROY SOC LOND B BIO, V284, P1
Blázquez J, 2003, CLIN INFECT DIS, V37, P1201, DOI 10.1086/378810
Carja O., 2016, BIORXIV, P1
Carlson SM, 2014, TRENDS ECOL EVOL, V29, P521, DOI 10.1016/j.tree.2014.06.005

Chait R, 2007, NATURE, V446, P668, DOI 10.1038/nature05685
Chevin L.-M., 2010, PLOS BIOL, P1
Chevin LM, 2013, PHILOS T R SOC B, V368, DOI 10.1098/rstb.2012.0089
Chevin LM, 2010, EVOLUTION, V64, P1143, DOI 10.1111/j.1558-5646.2009.00875.x
Cirz RT, 2005, PLOS BIOL, V3, P1024, DOI 10.1371/journal.pbio.0030176
Davies J, 2010, MICROBIOL MOL BIOL R, V74, P417, DOI 10.1128/MMBR.00016-10
Day T, 2016, PLOS COMPUT BIOL, V12, DOI 10.1371/journal.pcbi.1004689
Day T, 2012, EVOLUTION, V66, P1582, DOI 10.1111/j.1558-5646.2011.01533.x
Denamur E, 2006, MOL MICROBIOL, V60, P820, DOI 10.1111/j.1365-2958.2006.05150.x
Eyre-Walker A, 2007, NAT REV GENET, V8, P610, DOI 10.1038/nrg2146
Gerrish PJ, 2003, LANCET INFECT DIS, V3, P28, DOI 10.1016/S1473-3099(03)00485-7
Giraud A, 2002, ANTIMICROB AGENTS CH, V46, P863, DOI 10.1128/AAC.46.3.863-865.2002
Greenspoon P.B., 2013, Proc. R. Soc. Lond. B, V280, P1
Hao YQ, 2015, ECOL LETT, V18, P892, DOI 10.1111/ele.12465
Hegreness M, 2008, P NATL ACAD SCI USA, V105, P13977, DOI 10.1073/pnas.0805965105
Imamovic L, 2013, SCI TRANSL MED, V5, DOI 10.1126/scitranslmed.3006609
Iranzo J, 2011, P NATL ACAD SCI USA, V108, P16008, DOI 10.1073/pnas.1110489108
ISHII K, 1989, GENETICS, V121, P163
Kohanski MA, 2010, MOL CELL, V37, P311, DOI 10.1016/j.molcel.2010.01.003
Lachapelle J, 2012, EVOLUTION, V66, P3508, DOI 10.1111/j.1558-5646.2012.01697.x
LEIGH EG, 1973, GENETICS, V73, P1
LEIGH EG, 1970, AM NAT, V104, P301, DOI 10.1086/282663
LIBERMAN U, 1986, THEOR POPUL BIOL, V30, P125, DOI 10.1016/0040-5809(86)90028-6
Lindsey HA, 2013, NATURE, V494, P463, DOI 10.1038/nature11879
Loken Chris, 2010, Journal of Physics: Conference Series, V256, DOI 10.1088/1742-6596/256/1/012026
M'Gonigle LK, 2009, THEOR POPUL BIOL, V75, P301, DOI 10.1016/j.tpb.2009.03.003
Orr HA, 2008, AM NAT, V172, P160, DOI 10.1086/589460
Orr HA, 2014, PLOS GENET, V10, DOI 10.1371/journal.pgen.1004551
Pal C, 2007, NATURE, V450, P1079, DOI 10.1038/nature06350
Pena-Miller R, 2013, PLOS BIOL, V11, DOI 10.1371/journal.pbio.1001540
Perales C, 2009, PLOS PATHOG, V5, DOI 10.1371/journal.ppat.1000658
Perron GG, 2010, AM NAT, V176, P303, DOI 10.1086/655217
Ram Y, 2012, EVOLUTION, V66, P2315, DOI 10.1111/j.1558-5646.2012.01576.x
Reed TE, 2010, P ROY SOC B-BIOL SCI, V277, P3391, DOI 10.1098/rspb.2010.0771
Sniegowski PD, 1997, NATURE, V387, P703, DOI 10.1038/42701
Taddei F, 1997, NATURE, V387, P700, DOI 10.1038/42696
Tanaka MM, 2003, GENETICS, V164, P843
Travis JMJ, 2002, P ROY SOC B-BIOL SCI, V269, P591, DOI 10.1098/rspb.2001.1902

   Uecker H, 2016, GENETICS, V202, P721, DOI 10.1534/genetics.115.180299
   Uecker H, 2014, AM NAT, V183, pE17, DOI 10.1086/673914
   Wilson BA, 2017, GENETICS, V205, P1573, DOI
10.1534/genetics.116.191478
   Wu Y, 2014, J THEOR BIOL, V340, P222, DOI 10.1016/j.jtbi.2013.09.026
   zur Wiesch PA, 2014, PLOS PATHOG, V10, DOI
10.1371/journal.ppat.1004225
NR 53
TC 1
Z9 3
U1 0
U2 31
PU ACADEMIC PRESS INC ELSEVIER SCIENCE
PI SAN DIEGO
PA 525 B ST, STE 1900, SAN DIEGO, CA 92101-4495 USA
SN 0040-5809
EI 1096-0325
J9 THEOR POPUL BIOL
JI Theor. Popul. Biol.
PD OCT
PY 2017
VL 117
BP 64
EP 75
DI 10.1016/j.tpb.2017.08.004
PG 12
WC Ecology; Evolutionary Biology; Genetics & Heredity; Mathematical &
   Computational Biology
WE Science Citation Index Expanded (SCI-EXPANDED)
SC Environmental Sciences & Ecology; Evolutionary Biology; Genetics &
   Heredity; Mathematical & Computational Biology
GA FK0NM
UT WOS:000413176500005
PM 28866008
DA 2025-05-07
ER

PT J
AU Koshiyama, H
   Ogawa, Y
   Tanaka, K
   Tanaka, I
AF Koshiyama, Hiroyuki
   Ogawa, Yoshihiro
   Tanaka, Kiyoshi
   Tanaka, Issei
TI Integrated network systems and evolutionary developmental
endocrinology
SO MEDICAL HYPOTHESES
LA English
DT Article
ID DIABETES-MELLITUS; BIOLOGY; OBESITY; BONE; GENE; LINK
AB Endocrine system has been considered to be a linear one, but the 'real
world endocrine system' is a complex system, which is difficult to

investigate using conventional strategies, such as single nucleotide polymorphism, genome-wide analysis, or gene targeting in animals. Here we propose a new strategy to comprehend the endocrine system as a complex network system. We introduced several novel concepts, such as complex system, network analysis, systems biology and evolutionary medicine, into the comprehension of endocrine system as a whole complex network system. This system is considered to be a scale-free network with key molecules such as acetyl CoA, NAD or ATP as 'hubs'. This system is robust against simple mutations, but various complex diseases may attack hubs. The system is also 'fractals', since there exist similar network systems among cells, proteins, and transcription factors in the lower levels, and there are similar ones among disease and social network in the higher levels.

We propose to call this model 'Integrated Network Systems and Evolutionary DEvelopmental ENdocrinology (INS-EDEN)'. This novel framework will facilitate us to develop a new approach for understanding and treatment of various complex diseases related to endocrinology, and identify a unified theory of complex diseases. (C) 2009 Elsevier Ltd. All rights reserved.
C1 [Koshiyama, Hiroyuki] Kitano Hosp, Tazuke Kofukai Fdn, Ctr Diabet & Endocrinol, Med Res Inst, Osaka 5308480, Japan.
[Koshiyama, Hiroyuki; Tanaka, Kiyoshi] Kyoto Univ, Grad Sch Med, Dept Diabet & Clin Nutr, Kyoto, Japan.
[Ogawa, Yoshihiro] Tokyo Med & Dent Univ, Med Res Inst, Dept Mol Med & Metab, Tokyo, Japan.
[Ogawa, Yoshihiro] Tokyo Med & Dent Univ, Med Res Inst, Ctr Excellence Program Frontier Res Mol Destruct, Tokyo, Japan.
[Tanaka, Kiyoshi] Kyoto Womens Univ, Dept Food & Nutr, Kyoto, Japan.
[Tanaka, Issei] Hirakata Kosai Hosp, Hirakata, Osaka, Japan.
C3 Kitano Hospital; Kyoto University; Institute of Science Tokyo; Tokyo Medical & Dental University (TMDU); Institute of Science Tokyo; Tokyo Medical & Dental University (TMDU); Kyoto Womens University
RP Koshiyama, H (autor correspondente), Kitano Hosp, Tazuke Kofukai Fdn, Ctr Diabet & Endocrinol, Med Res Inst, Osaka 5308480, Japan.
EM h-koshiyama@kitano-hp.or.jp
RI Koshiyama, Hiroyuki/H-3877-2011
CR [Anonymous], Kyoto encyclopedia of genes and genomes
[Anonymous], 2007, Principles of Development
Bainbridge WilliamSims., 2007, Nanoconvergence: The Unity of Nanoscience, Biotechnology, Information Technology, and Cognitive Science
Barabási AL, 2004, NAT REV GENET, V5, P101, DOI 10.1038/nrg1272
Barabási AL, 2007, NEW ENGL J MED, V357, P404, DOI 10.1056/NEJMe078114
Burnett JR, 2002, ANN CLIN BIOCHEM, V39, P203, DOI 10.1258/0004563021902134
DEFRONZO RA, 2008, BANTING MEDAL LECT
DEFRONZO RA, 2007, EXPLORING NEW FRONTI
Ellison P.T., 2009, ENDOCRINOLOGY SOCIAL
Gardner TS, 2003, SCIENCE, V301, P102, DOI 10.1126/science.1081900
Gibson G., 2009, It Takes a Genome: How a Clash Between Our Genes and Modern Life Is Making Us Sick
Gilbert S., 2000, Developmental Biology
Gilchrist M, 2006, NATURE, V441, P173, DOI 10.1038/nature04768
GOULD SJ, 1993, LIFES GRANDEUR SPEED
HALES CN, 1992, DIABETOLOGIA, V35, P595, DOI 10.1093/ije/dyt133

Hamerman D, 2005, QJM-INT J MED, V98, P467, DOI 10.1093/qjmed/hci077
Ikeda H, 2007, BIOCHEM BIOPH RES CO, V364, P457, DOI 10.1016/j.bbrc.2007.10.058
Jones S, 2008, LANCET, pS1
Kafri R, 2009, CELL, V136, P389, DOI 10.1016/j.cell.2009.01.027
Kahn R, 2005, DIABETES CARE, V28, P2289, DOI 10.2337/diacare.28.9.2289
Kirschner MW., 2005, The plausibility of life: Resolving Darwins dilemma
Kitzbichler MG, 2009, PLOS COMPUT BIOL, V5, DOI 10.1371/journal.pcbi.1000314
Koshiyama H, 2007, DIABETIC MED, V24, P571, DOI 10.1111/j.1464-5491.2007.02174.x
Koshiyama H, 2006, MED HYPOTHESES, V66, P960, DOI 10.1016/j.mehy.2005.11.024
Koshiyama H, 2006, MED HYPOTHESES, V66, P473, DOI 10.1016/j.mehy.2005.10.015
Koshiyama H, 2006, MED HYPOTHESES, V67, P307, DOI 10.1016/j.mehy.2006.02.033
Koshiyama Hiroyuki, 2003, Nihon Naika Gakkai Zasshi, V92, P629
Koshiyama H, 2008, CLIN MED INSIGHTS-EN, V1, P1
Kronenberg H., 2008, WILLIAMS TXB ENDOCRI
LANE N, 2005, POWER SUICIDE SEX MI
Lane N., 2003, OXYGEN MOL MADE WORL
Macosko EZ, 2009, NATURE, V458, P1171, DOI 10.1038/nature07886
Matsuzawa Yuji, 2005, Semin Vasc Med, V5, P34, DOI 10.1055/s-2005-871744
NEEL JV, 1962, AM J HUM GENET, V14, P353
Newman M, 2006, STRUCTURE DYNAMICS N
Pimanda JE, 2006, MOL CELL BIOL, V26, P2615, DOI 10.1128/MCB.26.7.2615-2625.2006
Shoelson SE, 2009, NAT MED, V15, P373, DOI 10.1038/nm0409-373
Shubin Neil., 2009, YOUR INNER FISH
Strogatz S., 2003, SYNC, V1st
Sugden A, 2009, SCIENCE, V323, P727, DOI 10.1126/science.323.5915.727
Trevathan WR., 2008, Evolutionary Medicine and Health: New Perspectives
Wilson E.O., 1998, Consilience: The Unity of Knowledge, V1st ed.
NR 42
TC 4
Z9 4
U1 0
U2 4
PU ELSEVIER
PI AMSTERDAM
PA RADARWEG 29, 1043 NX AMSTERDAM, NETHERLANDS
SN 0306-9877
EI 1532-2777
J9 MED HYPOTHESES
JI Med. Hypotheses
PD JAN
PY 2010
VL 74
IS 1
BP 132

EP 138
DI 10.1016/j.mehy.2009.07.028
PG 7
WC Medicine, Research & Experimental
WE Science Citation Index Expanded (SCI-EXPANDED)
SC Research & Experimental Medicine
GA 547VU
UT WOS:000273918500039
PM 19674847
DA 2025-05-07
ER

PT J
AU Morozova, I
   Flegontov, P
   Mikheyev, AS
   Bruskin, S
   Asgharian, H
   Ponomarenko, P
   Klyuchnikov, V
   ArunKumar, G
   Prokhortchouk, E
   Gankin, Y
   Rogaev, E
   Nikolsky, Y
   Baranova, A
   Elhaik, E
   Tatarinova, TV
AF Morozova, Irina
   Flegontov, Pavel
   Mikheyev, Alexander S.
   Bruskin, Sergey
   Asgharian, Hosseinali
   Ponomarenko, Petr
   Klyuchnikov, Vladimir
   ArunKumar, GaneshPrasad
   Prokhortchouk, Egor
   Gankin, Yuriy
   Rogaev, Evgeny
   Nikolsky, Yuri
   Baranova, Ancha
   Elhaik, Eran
   Tatarinova, Tatiana V.
TI Toward high-resolution population genomics using archaeological samples
SO DNA RESEARCH
LA English
DT Review
DE ancient DNA; bioinformatics; epigenetics; population genetics;
   next-generation sequencing
ID ANCIENT DNA REVEALS; MODERN HUMANS; GENETIC-CHARACTERIZATION;
   NEANDERTHAL ANCESTRY; MISCODING LESIONS; METHYLATION MAPS; HUMAN
   DISPERSALS; INFLUENZA-VIRUS; LOS HUESOS; SEQUENCE

AB The term 'ancient DNA' (aDNA) is coming of age, with over 1,200 hits in the PubMed database, beginning in the early 1980s with the studies of 'molecular paleontology'. Rooted in cloning and limited sequencing of DNA from ancient remains during the pre-PCR era, the field has made incredible progress since the introduction of PCR and next-generation sequencing. Over the last decade, aDNA analysis ushered in a new era in genomics and became the method of choice for reconstructing the history of organisms, their biogeography, and migration routes, with applications in evolutionary biology, population genetics, archaeogenetics, paleoepidemiology, and many other areas. This change was brought by development of new strategies for coping with the challenges in studying aDNA due to damage and fragmentation, scarce samples, significant historical gaps, and limited applicability of population genetics methods. In this review, we describe the state-of-the-art achievements in aDNA studies, with particular focus on human evolution and demographic history. We present the current experimental and theoretical procedures for handling and analysing highly degraded aDNA. We also review the challenges in the rapidly growing field of ancient epigenomics. Advancement of aDNA tools and methods signifies a new era in population genetics and evolutionary medicine research.
C1 [Morozova, Irina] Univ Zurich, Inst Evolutionary Med, Zurich, Switzerland.
[Flegontov, Pavel] Univ Ostrava, Fac Sci, Dept Biol & Ecol, Ostrava, Czech Republic.
[Flegontov, Pavel; Tatarinova, Tatiana V.] Russian Acad Sci, AA Kharkevich Inst Informat Transmiss Problems, Bioinformat Ctr, Moscow, Russia.
[Mikheyev, Alexander S.] Sci & Technol Grad Univ, Okinawa Inst, Ecol & Evolut Unit, Okinawa, Japan.
[Bruskin, Sergey; Rogaev, Evgeny; Nikolsky, Yuri] RAS, Vavilov Inst Gen Genet, Moscow, Russia.
[Asgharian, Hosseinali] Univ Southern Calif, Dept Computat & Mol Biol, Los Angeles, CA USA.
[Ponomarenko, Petr; Tatarinova, Tatiana V.] Childrens Hosp Los Angeles, Ctr Personalized Med, Los Angeles, CA USA.
[Ponomarenko, Petr; Tatarinova, Tatiana V.] Univ Southern Calif, Spatial Sci Inst, Los Angeles, CA USA.
[Klyuchnikov, Vladimir] Donskaya Archeol, Rostov Na Donu, Russia.
[ArunKumar, GaneshPrasad] SASTRA Univ, Sch Chem & Biotechnol, Tanjore, India.
[Prokhortchouk, Egor] RAS, Biotechnol Res Ctr, Moscow, Russia.
[Prokhortchouk, Egor] Lomonosov Moscow State Univ, Dept Biol, Moscow, Russia.
[Gankin, Yuriy] EPAM Syst, Newtown, PA USA.
[Rogaev, Evgeny] Univ Massachusetts, Sch Med, Worcester, MA USA.
[Nikolsky, Yuri] F1 Gen, San Diego, CA USA.
[Nikolsky, Yuri; Baranova, Ancha] George Mason Univ, Sch Syst Biol, Fairfax, VA 22030 USA.
[Baranova, Ancha] Med Genet Res Ctr, Moscow, Russia.
[Baranova, Ancha] Atlas Biomed Grp, Moscow, Russia.
[Elhaik, Eran] Univ Sheffield, Dept Anim & Plant Sci, Sheffield, S Yorkshire, England.
C3 University of Zurich; University of Ostrava; Russian Academy of

   Sciences; Kharkevich Institute for Information Transmission Problems
of
   the RAS; Russian Academy of Sciences; Vavilov Institute of General
   Genetics; University of Southern California; Children's Hospital Los
   Angeles; University of Southern California; Shanmugha Arts, Science,
   Technology & Research Academy (SASTRA); Research Center of
Biotechnology
   RAS; Russian Academy of Sciences; Lomonosov Moscow State University;
   University of Massachusetts System; University of Massachusetts
   Worcester; George Mason University; Research Centre for Medical
   Genetics; University of Sheffield
RP Morozova, I (autor correspondente), Univ Zurich, Inst Evolutionary
Med, Zurich, Switzerland.; Tatarinova, TV (autor correspondente), Russian
Acad Sci, AA Kharkevich Inst Informat Transmiss Problems, Bioinformat
Ctr, Moscow, Russia.; Tatarinova, TV (autor correspondente), Childrens
Hosp Los Angeles, Ctr Personalized Med, Los Angeles, CA USA.; Tatarinova,
TV (autor correspondente), Univ Southern Calif, Spatial Sci Inst, Los
Angeles, CA USA.
EM irina.morozova@iem.uzh.ch; tatiana.tatarinova@usc.edu
RI Tatarinova, Tatiana/AAH-1915-2020; Bruskin, Sergey/J-9598-2018; G,
   ARUNKUMAR/C-3063-2017; Rogaev, Evgeny/AAN-7662-2020; Asgharian,
   Hosseinali/AAZ-3379-2020; Elhaik, Eran/H-6838-2019; Flegontov,
   Pavel/G-9748-2014; Prokhortchouk, Egor/I-9108-2014; Baranova,
   Ancha/B-4608-2012; Mikheyev, Alexander/E-7999-2013; Tatarinova,
   Tatiana/K-3445-2016
OI Baranova, Ancha/0000-0001-6810-5982; Mikheyev,
   Alexander/0000-0003-4369-1019; Tatarinova, Tatiana/0000-0003-1787-
1112;
   Morozova, Irina/0000-0002-3063-4966; ArunKumar,
   GaneshPrasad/0000-0002-3108-0491; prokhortchouk,
   egor/0000-0001-8234-9975; Rogaev, Evgeny/0000-0002-0351-8783; Elhaik,
   Eran/0000-0003-4795-1084; Flegontov, Pavel/0000-0001-9759-4981
FU Swiss Maxi Foundation; NIH [GM098741, MH091561]; Russian Science
   Foundation [14-14-01202, 14-50-00029]; Moravian Silesian region
   [MSK2013-DT1, MSK2013-DT2, MSK2014-DT1]; Institution Development
Program
   of the University of Ostrava; NSF Division of Environmental Biology
   [1456634]; Royal Society International Exchanges Award [IE140020]; MRC
   Confidence in Concept Scheme Award-University of Sheffield
   [MC_PC_14115]; Russian Science Foundation [14-14-01202] Funding
Source:
   Russian Science Foundation; MRC [MC_PC_14115] Funding Source: UKRI;
   Direct For Biological Sciences; Division Of Environmental Biology
   [1456634] Funding Source: National Science Foundation
FX I.M. was supported by Swiss Maxi Foundation grant. H.A. was supported
by
   NIH grants GM098741 and MH091561. E.P. was supported by Russian
Science
   Foundation grant 14-14-01202. P.F. was supported by the Moravian
   Silesian region projects MSK2013-DT1, MSK2013-DT2, and MSK2014-DT1 and
   by the Institution Development Program of the University of Ostrava.
   T.V.T., E.E., and P.P. were supported by NSF Division of Environmental
   Biology Award 1456634. E.E. was supported by The Royal Society
   International Exchanges Award (IE140020) and MRC Confidence in Concept

Scheme Award 2014-University of Sheffield (Ref: MC_PC_14115). E.R. was supported by Russian Science Foundation grant 14-50-00029.
CR Abu-Remaileh M, 2015, CANCER RES, V75, P2120, DOI 10.1158/0008-5472.CAN-14-3295
Adcock GJ, 2001, P NATL ACAD SCI USA, V98, P537, DOI 10.1073/pnas.98.2.537
Adler CJ, 2013, NAT GENET, V45, P450, DOI 10.1038/ng.2536
Alexander DH, 2009, GENOME RES, V19, P1655, DOI 10.1101/gr.094052.109
ALLARD MW, 1995, SCIENCE, V268, P1192, DOI 10.1126/science.7761840
Allentoft ME, 2015, NATURE, V522, P167, DOI 10.1038/nature14507
Andrews S., 2015, Babraham Bioinformatics-FastQC a quality control tool for high throughput sequence data, V532, P1
[Anonymous], 2016, NATURE
[Anonymous], PLOS GENET
Anthony DW, 2007, HORSE, THE WHEEL, AND LANGUAGE: HOW BRONZE-AGE RIDERS FROM THE EURASIAN STEPPES SHAPED THE MODERN WORLD, P1
Arsuaga JL, 2014, SCIENCE, V344, P1358, DOI 10.1126/science.1253958
ArunKumar G, 2015, J HUM GENET, V60, P493, DOI 10.1038/jhg.2015.51
Austin JJ, 1997, P ROY SOC B-BIOL SCI, V264, P467, DOI 10.1098/rspb.1997.0067
Austin JJ, 1997, TRENDS ECOL EVOL, V12, P303, DOI 10.1016/S0169-5347(97)01102-6
Barnes I, 2002, SCIENCE, V295, P2267, DOI 10.1126/science.1067814
Berman BP, 2012, NAT GENET, V44, P40, DOI 10.1038/ng.969
Bos KI, 2014, NATURE, V514, P494, DOI 10.1038/nature13591
Briggs AW, 2007, P NATL ACAD SCI USA, V104, P14616, DOI 10.1073/pnas.0704665104
Briggs AW, 2010, NUCLEIC ACIDS RES, V38, DOI 10.1093/nar/gkp1163
Briggs AW, 2009, SCIENCE, V325, P318, DOI 10.1126/science.1174462
Brogaard K, 2012, NATURE, V486, P496, DOI 10.1038/nature11142
Brosch R, 2002, P NATL ACAD SCI USA, V99, P3684, DOI 10.1073/pnas.052548299
Brotherton P, 2013, NAT COMMUN, V4, DOI 10.1038/ncomms2656
Burbano HA, 2012, PLOS ONE, V7, DOI 10.1371/journal.pone.0032877
Burbano HA, 2010, SCIENCE, V328, P723, DOI 10.1126/science.1188046
Cale CM, 2015, NATURE, V526, P611, DOI 10.1038/526611a
CANO RJ, 1993, BIOTECHNIQUES, V15, P432
CANO RJ, 1993, NATURE, V363, P536, DOI 10.1038/363536a0
Cerqueira CCS, 2012, AM J HUM BIOL, V24, P705, DOI 10.1002/ajhb.22263
Chopra P, 2014, BMC GENOMICS, V15, DOI 10.1186/1471-2164-15-131
Churchhouse C, 2013, GENET EPIDEMIOL, V37, P1, DOI 10.1002/gepi.21692
Cooper A, 2000, SCIENCE, V289, P1139
Crawford GE, 2006, GENOME RES, V16, P123, DOI 10.1101/gr.4074106
Dabney J, 2013, P NATL ACAD SCI USA, V110, P15758, DOI 10.1073/pnas.1314445110
Damgaard PB, 2015, SCI REP-UK, V5, DOI 10.1038/srep11184
Darling MI, 2014, MEM I OSWALDO CRUZ, V109, P131
DePristo MA, 2011, NAT GENET, V43, P491, DOI 10.1038/ng.806
Elhaik E, 2014, NAT COMMUN, V5, DOI 10.1038/ncomms4513
Elhaik E, 2013, GENOME BIOL EVOL, V5, P1021, DOI 10.1093/gbe/evt066
Elhaik E, 2012, PLOS ONE, V7, DOI 10.1371/journal.pone.0049837
Eriksson A, 2012, P NATL ACAD SCI USA, V109, P13956, DOI 10.1073/pnas.1200567109
Falush D, 2003, GENETICS, V164, P1567

Flegontov P., 2016, SCI REP, V6
Fu QM, 2016, NATURE, V534, P200, DOI 10.1038/nature17993
Fu Q, 2015, NATURE, V524, P216, DOI 10.1038/nature14558
Gansauge MT, 2014, GENOME RES, V24, P1543, DOI 10.1101/gr.174201.114
Gao SZ, 2015, AM J PHYS ANTHROPOL, V157, P71, DOI 10.1002/ajpa.22690
Gilbert MTP, 2008, SCIENCE, V320, P1787, DOI 10.1126/science.1159750
Gilbert MTP, 2007, SCIENCE, V317, P1927, DOI 10.1126/science.1146971
Gilbert MTP, 2003, AM J HUM GENET, V72, P32, DOI 10.1086/345378
Gilbert MTP, 2003, AM J HUM GENET, V72, P48, DOI 10.1086/345379
Ginolhac A, 2011, BIOINFORMATICS, V27, P2153, DOI 10.1093/bioinformatics/btr347
Gokhman D, 2014, SCIENCE, V344, P523, DOI 10.1126/science.1250368
GOLENBERG EM, 1991, PHILOS T ROY SOC B, V333, P419, DOI 10.1098/rstb.1991.0092
GOLENBERG EM, 1990, NATURE, V344, P656, DOI 10.1038/344656a0
Graur D, 2001, MOL BIOL EVOL, V18, P1143, DOI 10.1093/oxfordjournals.molbev.a003887
Green RE, 2008, CELL, V134, P416, DOI 10.1016/j.cell.2008.06.021
Green RE, 2006, NATURE, V444, P330, DOI 10.1038/nature05336
Green RE, 2010, SCIENCE, V328, P710, DOI 10.1126/science.1188021
Green RE, 2009, EMBO J, V28, P2494, DOI 10.1038/emboj.2009.222
Gutiérrez G, 1998, MOL BIOL EVOL, V15, P926, DOI 10.1093/oxfordjournals.molbev.a025998
Haak W, 2015, NATURE, V522, P207, DOI 10.1038/nature14317
Haak W, 2010, PLOS BIOL, V8, DOI 10.1371/journal.pbio.1000536
HAGELBERG E, 1991, P ROY SOC B-BIOL SCI, V244, P45, DOI 10.1098/rspb.1991.0049
Haile J, 2009, P NATL ACAD SCI USA, V106, P22352, DOI 10.1073/pnas.0912510106
HANDT O, 1994, EXPERIENTIA, V50, P524, DOI 10.1007/BF01921720
HANDT O, 1994, SCIENCE, V264, P1775, DOI 10.1126/science.8209259
Hansen AJ, 2001, MOL BIOL EVOL, V18, P262, DOI 10.1093/oxfordjournals.molbev.a003800
Hansen KD, 2011, NAT GENET, V43, P768, DOI 10.1038/ng.865
HEDGES SB, 1995, SCIENCE, V268, P1191, DOI 10.1126/science.7761839
Hendrich B, 1998, MOL CELL BIOL, V18, P6538, DOI 10.1128/MCB.18.11.6538
HENIKOFF S, 1995, SCIENCE, V268, P1192, DOI 10.1126/science.7761841
HIGUCHI R, 1984, NATURE, V312, P282, DOI 10.1038/312282a0
Hofreiter M, 2001, NUCLEIC ACIDS RES, V29, P4793, DOI 10.1093/nar/29.23.4793
Jaenicke-Després V, 2003, SCIENCE, V302, P1206, DOI 10.1126/science.1089056
Jeong M, 2014, NAT GENET, V46, P17, DOI 10.1038/ng.2836
Jones M., 2002, The molecule hunt: archaeology and the search for ancient DNA
Jónsson H, 2013, BIOINFORMATICS, V29, P1682, DOI 10.1093/bioinformatics/btt193
Kader F, 2015, FORENSIC SCI INT, V249, P255, DOI 10.1016/j.forsciint.2015.01.037
Kao WC, 2009, GENOME RES, V19, P1884, DOI 10.1101/gr.095299.109
Karmin M, 2015, GENOME RES, V25, P459, DOI 10.1101/gr.186684.114
Keller A, 2012, NAT COMMUN, V3, DOI 10.1038/ncomms1701

Kerpedjiev P, 2014, BMC BIOINFORMATICS, V15, DOI 10.1186/1471-2105-15-100
Kharlamova A, 2016, QUATERN INT, V406, P86, DOI 10.1016/j.quaint.2014.09.073
Knapp M, 2015, INVESTIG GENET, V6, DOI 10.1186/s13323-015-0020-4
Kolman CJ, 2000, AM J PHYS ANTHROPOL, V111, P5
Kozlov K, 2015, BMC GENOMICS, V16, DOI 10.1186/1471-2164-16-S8-S9
Krause J, 2007, CURR BIOL, V17, P1908, DOI 10.1016/j.cub.2007.10.008
Krause J, 2007, NATURE, V449, P902, DOI 10.1038/nature06193
Krause J, 2010, NATURE, V464, P894, DOI 10.1038/nature08976
Krause J, 2010, CURR BIOL, V20, P231, DOI 10.1016/j.cub.2009.11.068
Kuhlwilm M, 2016, NATURE, V530, P429, DOI 10.1038/nature16544
Kushniarevich A, 2015, PLOS ONE, V10, DOI 10.1371/journal.pone.0135820
Lacan M, 2011, P NATL ACAD SCI USA, V108, P9788, DOI 10.1073/pnas.1100723108
Lai CSL, 2001, NATURE, V413, P519, DOI 10.1038/35097076
Lalueza-Fox C, 2007, SCIENCE, V318, P1453, DOI 10.1126/science.1147417
Lalueza-Fox C, 2009, BIOL LETTERS, V5, P809, DOI 10.1098/rsbl.2009.0532
Lalueza-Fox C, 2008, BMC EVOL BIOL, V8, DOI 10.1186/1471-2148-8-342
Lazaridis I, 2014, NATURE, V513, P409, DOI 10.1038/nature13673
Leslie S, 2015, NATURE, V519, P309, DOI 10.1038/nature14230
Li H, 2009, BIOINFORMATICS, V25, P1754, DOI [10.1093/bioinformatics/btp698, 10.1093/bioinformatics/btp324, 10.1093/bioinformatics/btp352]
Li Yi, 1995, Acta Scientiarum Naturalium Universitatis Pekinensis, V31, P148
Lia VV, 2007, P ROY SOC B-BIOL SCI, V274, P545, DOI 10.1098/rspb.2006.3747
Limburg PA, 2002, P ROY SOC B-BIOL SCI, V269, P281, DOI 10.1098/rspb.2001.1868
LINDAHL T, 1977, J BIOL CHEM, V252, P3286
Lindgreen Stinus, 2014, BMC Res Notes, V7, P698, DOI 10.1186/1756-0500-7-698
Lindgreen Stinus, 2012, BMC Res Notes, V5, P337, DOI 10.1186/1756-0500-5-337
Liu W, 2015, NATURE, V526, P696, DOI 10.1038/nature15696
Llamas B, 2012, PLOS ONE, V7, DOI 10.1371/journal.pone.0030226
Llorente MG, 2015, SCIENCE, V350, P820, DOI 10.1126/science.aad2879
Lohse K, 2014, GENETICS, V196, P1241, DOI 10.1534/genetics.114.162396
Arsuaga JL, 2015, P NATL ACAD SCI USA, V112, P11524, DOI 10.1073/pnas.1514828112
Lynch M, 2014, GENETICS, V198, P269, DOI 10.1534/genetics.114.166843
Malaspinas AS, 2014, CURR BIOL, V24, pR1035, DOI 10.1016/j.cub.2014.09.078
Marean CW, 2015, EVOL ANTHROPOL, V24, P62, DOI 10.1002/evan.21443
Martin M., 2015, EMBNET J, V17
Mathieson I, 2015, NATURE, V528, P499, DOI 10.1038/nature16152
Matisoo-Smith E, 2015, J HUM EVOL, V79, P93, DOI 10.1016/j.jhevol.2014.10.017
McKenna A, 2010, GENOME RES, V20, P1297, DOI 10.1101/gr.107524.110
Menotti F., 2013, The Oxford Handbook of Wetland Archaeology
Meyer M, 2016, NATURE, V531, P504, DOI 10.1038/nature17405
Meyer M, 2014, NATURE, V505, P403, DOI 10.1038/nature12788

Meyer M, 2012, SCIENCE, V338, P222, DOI 10.1126/science.1224344
Mikheyev AS, 2009, MOL ECOL, V18, P2937, DOI 10.1111/j.1365-294X.2009.04213.x
Mikheyev AS, 2015, NAT COMMUN, V6, DOI 10.1038/ncomms8991
Millar CD, 2008, TRENDS ECOL EVOL, V23, P386, DOI 10.1016/j.tree.2008.04.002
Miller W, 2008, NATURE, V456, P387, DOI 10.1038/nature07446
Moorjani P, 2016, P NATL ACAD SCI USA, V113, P5652, DOI 10.1073/pnas.1514696113
Nakamura R, 2014, DEVELOPMENT, V141, P2568, DOI 10.1242/dev.108548
Nerlich A.G., 2009, INTERDISCIP PERSPECT, V2009
Nicholls H, 2005, PLOS BIOL, V3, P192, DOI 10.1371/journal.pbio.0030056
Noonan JP, 2006, SCIENCE, V314, P1113, DOI 10.1126/science.1131412
Novembre J, 2008, NATURE, V456, P98, DOI 10.1038/nature07331
Olalde I, 2014, SCI REP-UK, V4, DOI 10.1038/srep04666
Olalde I, 2014, NATURE, V507, P225, DOI 10.1038/nature12960
Orlando L, 2013, NATURE, V499, P74, DOI 10.1038/nature12323
Oskam CL, 2010, P ROY SOC B-BIOL SCI, V277, P1991, DOI 10.1098/rspb.2009.2019
Pääbo S, 2004, ANNU REV GENET, V38, P645, DOI 10.1146/annurev.genet.37.110801.143214
PAABO S, 1988, NUCLEIC ACIDS RES, V16, P9775, DOI 10.1093/nar/16.20.9775
Parks M, 2015, PHILOS T R SOC B, V370, DOI 10.1098/rstb.2013.0381
Patterson N, 2012, GENETICS, V192, P1065, DOI 10.1534/genetics.112.145037
Pedersen JS, 2014, GENOME RES, V24, P454, DOI 10.1101/gr.163592.113
Pickrell JK, 2014, TRENDS GENET, V30, P377, DOI 10.1016/j.tig.2014.07.007
Poinar H, 2003, CURR BIOL, V13, P1150, DOI 10.1016/S0960-9822(03)00450-0
Poinar HN, 2006, SCIENCE, V311, P392, DOI 10.1126/science.1123360
Prüfer K, 2014, NATURE, V505, P43, DOI 10.1038/nature12886
Prüfer K, 2010, GENOME BIOL, V11, DOI 10.1186/gb-2010-11-5-r47
Qin PF, 2015, MOL BIOL EVOL, V32, P2665, DOI 10.1093/molbev/msv141
Racimo F, 2015, NAT REV GENET, V16, P359, DOI 10.1038/nrg3936
Raghavan M, 2015, SCIENCE, V349, DOI 10.1126/science.aab3884
Raghavan M, 2014, SCIENCE, V345, P1020, DOI 10.1126/science.1255832
Raghavan M, 2014, NATURE, V505, P87, DOI 10.1038/nature12736
Rasmussen M, 2011, SCIENCE, V334, P94, DOI 10.1126/science.1211177
Rasmussen M, 2010, NATURE, V463, P757, DOI 10.1038/nature08835
Rasmussen S, 2015, CELL, V163, P571, DOI 10.1016/j.cell.2015.10.009
Reich D, 2011, AM J HUM GENET, V89, P516, DOI 10.1016/j.ajhg.2011.09.005
Reich D, 2010, NATURE, V468, P1053, DOI 10.1038/nature09710
Reid AH, 1999, P NATL ACAD SCI USA, V96, P1651, DOI 10.1073/pnas.96.4.1651
Renaud G., 2015, aLib: a sequencing pipeline for ancient and modern DNA
Renaud G, 2015, GENOME BIOL, V16, DOI 10.1186/s13059-015-0776-0
Renaud G, 2014, NUCLEIC ACIDS RES, V42, DOI 10.1093/nar/gku699
Renaud G, 2013, BIOINFORMATICS, V29, P1208, DOI 10.1093/bioinformatics/btt117

Rivollat M, 2015, PLOS ONE, V10, DOI 10.1371/journal.pone.0125521
Sánchez-Quinto F, 2015, PHILOS T R SOC B, V370, DOI 10.1098/rstb.2013.0374
Sankararaman S, 2008, AM J HUM GENET, V82, P290, DOI 10.1016/j.ajhg.2007.09.022
Sankararaman S, 2016, CURR BIOL, V26, P1241, DOI 10.1016/j.cub.2016.03.037
Sankararaman S, 2014, NATURE, V507, P354, DOI 10.1038/nature12961
Sarkissian CD, 2013, PLOS GENET, V9, DOI 10.1371/journal.pgen.1003296
Sawyer S, 2015, P NATL ACAD SCI USA, V112, P15696, DOI 10.1073/pnas.1519905112
Schubert M, 2014, NAT PROTOC, V9, P1056, DOI 10.1038/nprot.2014.063
Schubert M, 2012, BMC GENOMICS, V13, DOI 10.1186/1471-2164-13-178
Seguin-Orlando A, 2014, SCIENCE, V346, P1113, DOI 10.1126/science.aaa0114
Shapiro B, 2004, SCIENCE, V306, P1561, DOI 10.1126/science.1101074
Skoglund P, 2014, SCIENCE, V344, P747, DOI 10.1126/science.1253448
Skoglund P, 2014, P NATL ACAD SCI USA, V111, P2229, DOI 10.1073/pnas.1318934111
Skoglund P, 2013, J ARCHAEOL SCI, V40, P4477, DOI 10.1016/j.jas.2013.07.004
Skoglund P, 2012, SCIENCE, V336, P466, DOI 10.1126/science.1216304
Skotte L, 2013, GENETICS, V195, P693, DOI 10.1534/genetics.113.154138
Smith O, 2014, SCI REP-UK, V4, DOI 10.1038/srep05559
Sohn KA, 2012, GENETICS, V191, P1295, DOI 10.1534/genetics.112.140228
St John J., 2015, SeqPrep
Stone AC, 1996, AM J PHYS ANTHROPOL, V99, P231
Sudmant PH, 2015, SCIENCE, V349, DOI 10.1126/science.aab3761
Tang H, 2006, AM J HUM GENET, V79, P1, DOI 10.1086/504302
Tatarinova Tatiana, 2013, Quant Biol, V1, P261
Taubenberger JK, 2007, ANTIVIR THER, V12, P581
Taubenberger JK, 2007, JAMA-J AM MED ASSOC, V297, P2025, DOI 10.1001/jama.297.18.2025
Taubenberger JK, 1997, SCIENCE, V275, P1793, DOI 10.1126/science.275.5307.1793
Tin MMY, 2015, MOL ECOL RESOUR, V15, P329, DOI 10.1111/1755-0998.12314
Troukhan M, 2009, OMICS, V13, P139, DOI 10.1089/omi.2008.0034
Tumpey TM, 2004, P NATL ACAD SCI USA, V101, P3166, DOI 10.1073/pnas.0308391100
Tumpey TM, 2005, SCIENCE, V310, P77, DOI 10.1126/science.1119392
Veeramah KR, 2014, NAT REV GENET, V15, P149, DOI 10.1038/nrg3625
Vernot B, 2015, AM J HUM GENET, V96, P448, DOI 10.1016/j.ajhg.2015.01.006
Vernot B, 2014, CURR BIOL, V24, pR845, DOI 10.1016/j.cub.2014.07.079
Vernot B, 2014, SCIENCE, V343, P1017, DOI 10.1126/science.1245938
Vreeland RH, 2000, NATURE, V407, P897, DOI 10.1038/35038060
Wall JD, 2013, GENETICS, V194, P199, DOI 10.1534/genetics.112.148213
Willerslev E, 2014, NATURE, V506, P47, DOI 10.1038/nature12921
WOODWARD SR, 1994, SCIENCE, V266, P1229
Xie W, 2013, CELL, V153, P1134, DOI 10.1016/j.cell.2013.04.022
Yang WY, 2014, G3-GENES GENOM GENET, V4, P2505, DOI 10.1534/g3.114.014274
Yang WY, 2012, NAT GENET, V44, P725, DOI 10.1038/ng.2285
Yunusbayev B, 2015, PLOS GENET, V11, DOI 10.1371/journal.pgen.1005068

Zakany J, 2007, CURR OPIN GENET DEV, V17, P359, DOI 10.1016/j.gde.2007.05.011
   Zeng J, 2012, AM J HUM GENET, V91, P455, DOI 10.1016/j.ajhg.2012.07.024
   Zhao YB, 2010, J HUM GENET, V55, P215, DOI 10.1038/jhg.2010.8
   ZISCHLER H, 1995, SCIENCE, V268, P1192, DOI 10.1126/science.7605504
NR 208
TC 17
Z9 19
U1 2
U2 75
PU OXFORD UNIV PRESS
PI OXFORD
PA GREAT CLARENDON ST, OXFORD OX2 6DP, ENGLAND
SN 1340-2838
EI 1756-1663
J9 DNA RES
JI DNA Res.
PD AUG
PY 2016
VL 23
IS 4
BP 295
EP 310
DI 10.1093/dnares/dsw029
PG 16
WC Genetics & Heredity
WE Science Citation Index Expanded (SCI-EXPANDED)
SC Genetics & Heredity
GA DY0EB
UT WOS:000384768700001
PM 27436340
OA gold, Green Accepted, Green Published
DA 2025-05-07
ER

PT J
AU Zhang, JF
   Tarbet, EB
   Toro, H
   Tang, DCC
AF Zhang, Jianfeng
   Tarbet, E. Bart
   Toro, Haroldo
   Tang, De-chu C.
TI Adenovirus-vectored drug-vaccine duo as a potential driver for
   conferring mass protection against infectious diseases
SO EXPERT REVIEW OF VACCINES
LA English
DT Review
DE adenovirus; drug resistance; drug-vaccine duo; influenza therapy; mass
   immunization; nasal vaccine; safety; vaccine
ID IMMUNODEFICIENCY-VIRUS VACCINE; AVIAN INFLUENZA-VIRUS; DENDRITIC CELLS;

GENETIC IMMUNIZATION; IMMUNE-RESPONSES; YERSINIA-PESTIS; HIV-1 VACCINE;
DOUBLE-BLIND; T-CELLS; MUCOSAL

AB The disease-fighting power of vaccines has been a public health bonanza credited with the worldwide reduction of mortality and morbidity. The goal to further amplify its power by boosting vaccine coverage requires the development of a new generation of rapid-response vaccines that can be mass produced at low costs and mass administered by nonmedical personnel. The new vaccines also have to be endowed with a higher safety margin than that of conventional vaccines. The nonreplicating adenovirus-vectored vaccine holds promise in boosting vaccine coverage because the vector can be rapidly manufactured in serum-free suspension cells in response to a surge in demand, and noninvasively administered by nasal spray into human subjects in compliance with evolutionary medicine. In contrast to parenteral injection, noninvasive mucosal vaccination minimizes systemic inflammation. Moreover, preexisting adenovirus immunity does not interfere appreciably with the potency of an adenovirus-vectored nasal vaccine. Nasal administration of adenovirus vectors encoding pathogen antigens is not only fear-free and painless, but also confers rapid and sustained protection against mucosal pathogens as a drug-vaccine duo since adenovirus particles alone without transgene expression can induce an anti-influenza state in the airway. In addition to human vaccination, animals can also be mass immunized by this class of vectored vaccines.

C1 [Zhang, Jianfeng; Tang, De-chu C.] Vaxin Inc, Birmingham, AL 35203 USA.
[Tarbet, E. Bart] Utah State Univ, Inst Antiviral Res, Logan, UT 84322 USA.
[Toro, Haroldo] Auburn Univ, Dept Pathobiol, Auburn, AL 36849 USA.
C3 Utah System of Higher Education; Utah State University; Auburn University System; Auburn University
RP Tang, DCC (autor correspondente), Vaxin Inc, 1500 1st Ave N, Birmingham, AL 35203 USA.
EM dechutang@gmail.com
OI Tarbet, Bart/0000-0002-4255-9094

FU NIH [2R44AI068285-02, 1UC1AI067198-01, 1UC1AI067205-1]
FX The authors at Vaxin Inc. and Auburn University are shareholders of Vaxin Inc. and inventors on patents pertaining to adenovirus-vectored vaccines and/or DVD. They have also received grant support from the NIH (2R44AI068285-02, 1UC1AI067198-01 and 1UC1AI067205-1). The authors have no other relevant affiliations or financial involvement with any organization or entity with a financial interest in or financial conflict with the subject matter or materials discussed in the manuscript apart from those disclosed.

CR Alcock R, 2010, SCI TRANSL MED, V2, DOI 10.1126/scitranslmed.3000490
Appledorn DM, 2011, CLIN VACCINE IMMUNOL, V18, P150, DOI 10.1128/CVI.00341-10
Avakian AP, 2007, EXPERT REV VACCINES, V6, P457, DOI 10.1586/14760584.6.3.457
Barouch DH, 2011, VACCINE, V29, P5203, DOI 10.1016/j.vaccine.2011.05.025

Benlahrech A, 2009, P NATL ACAD SCI USA, V106, P19940, DOI 10.1073/pnas.0907898106
Boyer JL, 2005, HUM GENE THER, V16, P157, DOI 10.1089/hum.2005.16.157
Boyer JL, 2010, HUM GENE THER, V21, P891, DOI 10.1089/hum.2009.148
Brandes M, 2005, SCIENCE, V309, P264, DOI 10.1126/science.1110267
Brandtzaeg P, 2011, AM J RESP CRIT CARE, V183, P1595, DOI 10.1164/rccm.201011-1783OC
Buchbinder SP, 2008, LANCET, V372, P1881, DOI 10.1016/S0140-6736(08)61591-3
Carlson BC, 2000, AM J PATHOL, V156, P2057, DOI 10.1016/S0002-9440(10)65077-8
Carter NJ, 2011, DRUGS, V71, P1591, DOI 10.2165/11206860-000000000-00000
Chen EC, 2011, PLOS PATHOG, V7, DOI 10.1371/journal.ppat.1002155
Chiuchiolo MJ, 2006, J INFECT DIS, V194, P1249, DOI 10.1086/507644
Couch RB, 2004, NEW ENGL J MED, V350, P860, DOI 10.1056/NEJMp048006
Croyle MA, 2001, GENE THER, V8, P1281, DOI 10.1038/sj.gt.3301527
Croyle MA, 2008, PLOS ONE, V3, DOI 10.1371/journal.pone.0003548
del Rio ML, 2010, IMMUNOL REV, V234, P268, DOI 10.1111/j.0105-2896.2009.00874.x
Evans RK, 2004, J PHARM SCI-US, V93, P2458, DOI 10.1002/jps.20157
Fallaux FJ, 1998, HUM GENE THER, V9, P1909, DOI 10.1089/hum.1998.9.13-1909
Feng T, 2010, J IMMUNOL, V185, P5915, DOI 10.4049/jimmunol.1001233
Finch CE, 2004, SCIENCE, V305, P1736, DOI 10.1126/science.1092556
FOX JL, 2006, MICROBE, V1, P554
Gabitzsch ES, 2009, VACCINE, V27, P6394, DOI 10.1016/j.vaccine.2009.06.028
Gallichan WS, 1996, J EXP MED, V184, P1879, DOI 10.1084/jem.184.5.1879
Gallichan WS, 1998, J INFECT DIS, V177, P1155, DOI 10.1086/515286
Gao WT, 2006, J VIROL, V80, P1959, DOI 10.1128/JVI.80.4.1959-1964.2006
Geisbert TW, 2011, J VIROL, V85, P4222, DOI 10.1128/JVI.02407-10
Geisbert TW, 2010, J VIROL, V84, P10386, DOI 10.1128/JVI.00594-10
Glenn GM, 2000, NAT MED, V6, P1403, DOI 10.1038/82225
Gregor MF, 2011, ANNU REV IMMUNOL, V29, P415, DOI 10.1146/annurev-immunol-031210-101322
HAJAHMAD Y, 1986, J VIROL, V57, P267, DOI 10.1128/JVI.57.1.267-274.1986
Hartman ZC, 2008, VIRUS RES, V132, P1, DOI 10.1016/j.virusres.2007.10.005
Henderson H, 2009, VACCINE, V27, P7194, DOI 10.1016/j.vaccine.2009.09.030
Higashimoto Yuji, 2006, Inflammation & Allergy Drug Targets, V5, P229, DOI 10.2174/187152806779010927
Hoelscher MA, 2006, LANCET, V367, P475, DOI 10.1016/S0140-6736(06)68076-8
Howell MR, 1998, AM J PREV MED, V14, P168, DOI 10.1016/S0749-3797(97)00064-0
Jae SY, 2010, CLIN AUTON RES, V20, P229, DOI 10.1007/s10286-010-0069-3
Jones S, 2009, VACCINE, V27, P2506, DOI 10.1016/j.vaccine.2009.02.061
Joseph A, 2006, VACCINE, V24, P3990, DOI 10.1016/j.vaccine.2005.12.017
Kaplan DH, 2010, TRENDS IMMUNOL, V31, P446, DOI 10.1016/j.it.2010.08.006

Karron RA, 2009, VACCINE, V27, P4953, DOI 10.1016/j.vaccine.2009.05.099
Kaufman DR, 2010, J VIROL, V84, P5986, DOI 10.1128/JVI.02563-09
Kutzler MA, 2008, NAT REV GENET, V9, P776, DOI 10.1038/nrg2432
Lanza GA, 2011, J INTERN MED, V269, P118, DOI 10.1111/j.1365-2796.2010.02285.x
Lemiale F, 2003, J VIROL, V77, P10078, DOI 10.1128/JVI.77.18.10078-10087.2003
Lewis DJM, 2009, PLOS ONE, V4, DOI 10.1371/journal.pone.0006999
Li C, 2002, MOL THER, V5, P745, DOI 10.1006/mthe.2002.0605
Lichtenstein DL, 2004, CANCER GENE THER, V11, P819, DOI 10.1038/sj.cgt.7700765
Littel-van den Hurk SV, 2010, EXPERT REV VACCINES, V9, P503, DOI [10.1586/erv.10.42, 10.1586/ERV.10.42]
Lobanova LM, 2010, VACCINE, V28, P7613, DOI 10.1016/j.vaccine.2010.09.055
LUBECK MD, 1994, AIDS RES HUM RETROV, V10, P1443, DOI 10.1089/aid.1994.10.1443
McConnell MJ, 2007, MOL THER, V15, P203, DOI 10.1038/sj.mt.6300034
McElrath MJ, 2008, LANCET, V372, P1894, DOI 10.1016/S0140-6736(08)61592-5
MCWILLIAM AS, 1995, IMMUNOL CELL BIOL, V73, P405, DOI 10.1038/icb.1995.63
Minor P, 2009, VACCINE, V27, P2649, DOI 10.1016/j.vaccine.2009.02.071
Molenaar R, 2009, J IMMUNOL, V183, P6395, DOI 10.4049/jimmunol.0900311
Norton EB, 2010, J VIROL, V84, P2983, DOI 10.1128/JVI.01805-09
Nwanegbo E, 2004, CLIN DIAGN LAB IMMUN, V11, P351, DOI 10.1128/CDLI.11.2.351-357.2004
O'Callaghan DS, 2010, J THORAC ONCOL, V5, P2024, DOI 10.1097/JTO.0b013e3181f387e4
OCKENHOUSE C, 2010, C GEN BAS VACC VIENN
Osada T, 2009, CANCER GENE THER, V16, P673, DOI 10.1038/cgt.2009.17
Parks RJ, 1996, P NATL ACAD SCI USA, V93, P13565, DOI 10.1073/pnas.93.24.13565
Patel A, 2010, PLOS ONE, V5, DOI 10.1371/journal.pone.0015301
Peng ZH, 2005, HUM GENE THER, V16, P1016, DOI 10.1089/hum.2005.16.1016
Poland GA, 2005, VACCINE, V23, P2078, DOI 10.1016/j.vaccine.2005.01.012
Poland GA, 2009, CLIN INFECT DIS, V48, P1254, DOI 10.1086/598989
Pratt WD, 2010, CLIN VACCINE IMMUNOL, V17, P572, DOI 10.1128/CVI.00467-09
Raper SE, 2003, MOL GENET METAB, V80, P148, DOI 10.1016/j.ymgme.2003.08.016
Roy S, 2011, J GENE MED, V13, P17, DOI 10.1002/jgm.1530
Safronetz D, 2009, J VIROL, V83, P7285, DOI 10.1128/JVI.00373-09
SALOMON ME, 1987, AM J DIS CHILD, V141, P796, DOI 10.1001/archpedi.1987.04460070098034
San Martín C, 2003, CURR TOP MICROBIOL, V272, P57
Saurer L, 2007, J IMMUNOL, V179, P3504, DOI 10.4049/jimmunol.179.6.3504
Seto J, 2010, VIROLOGY, V404, P180, DOI 10.1016/j.virol.2010.05.010
Sever JL, 2004, PHARMACOEPIDEM DR S, V13, P825, DOI 10.1002/pds.936
Sharma A, 2009, VIROLOGY, V386, P44, DOI 10.1016/j.virol.2009.01.008

Shi ZK, 2001, J VIROL, V75, P11474, DOI 10.1128/JVI.75.23.11474-11482.2001
Singh S, 2010, VIROLOGY, V405, P62, DOI 10.1016/j.virol.2010.05.002
Soloff AC, 2010, CELL RES, V20, P872, DOI 10.1038/cr.2010.94
Song KM, 2010, P NATL ACAD SCI USA, V107, P22213, DOI 10.1073/pnas.1015536108
Stone D, 2007, MOL THER, V15, P2146, DOI 10.1038/sj.mt.6300319
Sullivan SP, 2010, NAT MED, V16, P915, DOI 10.1038/nm.2182
Takahashi E, 2010, MICROBES INFECT, V12, P778, DOI 10.1016/j.micinf.2010.04.013
TANG DC, 1992, NATURE, V356, P152, DOI 10.1038/356152a0
Tang DC, 1997, NATURE, V388, P729, DOI 10.1038/41917
Tang DCC, 2008, EXPERT REV VACCINES, V7, P399, DOI 10.1586/14760584.7.4.399
Tang DCC, 2009, EXPERT REV VACCINES, V8, P469, DOI [10.1586/erv.09.1, 10.1586/ERV.09.1]
Tang J, 2004, GENE THER, V11, P1408, DOI 10.1038/sj.gt.3302316
Thiele AT, 2006, J VIROL, V80, P1826, DOI 10.1128/JVI.80.4.1826-1836.2006
Toovey Stephen, 2008, Travel Med Infect Dis, V6, P114, DOI 10.1016/j.tmaid.2008.03.003
Toro H, 2009, POULTRY SCI, V88, P867, DOI 10.3382/ps.2008-00333
Toro H, 2010, AVIAN DIS, V54, P224, DOI 10.1637/8773-033109-Reg.1
Toro H, 2008, VACCINE, V26, P2640, DOI 10.1016/j.vaccine.2008.02.056
Toro H, 2007, VACCINE, V25, P2886, DOI 10.1016/j.vaccine.2006.09.047
Toro H, 2011, AVIAN DIS, V55, P43, DOI 10.1637/9516-090210-Reg.1
Tuvim MJ, 2009, PLOS ONE, V4, DOI 10.1371/journal.pone.0004176
ULMER JB, 1993, SCIENCE, V259, P1745, DOI 10.1126/science.8456302
van Ginkel FW, 2009, DEV COMP IMMUNOL, V33, P28, DOI 10.1016/j.dci.2008.07.018
Van Kampen KR, 2005, VACCINE, V23, P1029, DOI 10.1016/j.vaccine.2004.07.043
Wang J, 2004, J IMMUNOL, V173, P6357, DOI 10.4049/jimmunol.173.10.6357
Wang LS, 2009, J VIROL, V83, P7166, DOI 10.1128/JVI.00374-09
Wei CJ, 2010, SCIENCE, V329, P1060, DOI 10.1126/science.1192517
Wesley RD, 2004, VACCINE, V22, P3427, DOI 10.1016/j.vaccine.2004.02.040
Witte ME, 2010, MITOCHONDRION, V10, P411, DOI 10.1016/j.mito.2010.05.014
Worgall S, 2007, J VIROL, V81, P13801, DOI 10.1128/JVI.01246-07
Xiang ZQ, 1996, VIROLOGY, V219, P220, DOI 10.1006/viro.1996.0239
Xing Z, 2009, PLOS ONE, V4, DOI 10.1371/journal.pone.0005856
Xu Q, 2009, GENE THER, V16, P367, DOI 10.1038/gt.2008.181
Yamaguchi T, 2010, P NATL ACAD SCI USA, V107, P17286, DOI 10.1073/pnas.1009823107
YANG YP, 1994, P NATL ACAD SCI USA, V91, P4407, DOI 10.1073/pnas.91.10.4407
Zhang JF, 2006, INFECT IMMUN, V74, P3607, DOI 10.1128/IAI.01836-05
Zhang JF, 2011, PLOS ONE, V6, DOI 10.1371/journal.pone.0022605
Zhang SY, 2007, SCIENCE, V317, P1522, DOI 10.1126/science.1139522
ZHANG Z, 2011, HUM GENE TH IN PRESS
Zhu JD, 1999, HUM GENE THER, V10, P113, DOI 10.1089/10430349950019246
Zhu JA, 2008, MOL THER, V16, P1300, DOI 10.1038/mt.2008.88
NR 117

TC 14
Z9 16
U1 0
U2 12
PU TAYLOR & FRANCIS LTD
PI ABINGDON
PA 2-4 PARK SQUARE, MILTON PARK, ABINGDON OR14 4RN, OXON, ENGLAND
SN 1476-0584
EI 1744-8395
J9 EXPERT REV VACCINES
JI Expert Rev. Vaccines
PD NOV
PY 2011
VL 10
IS 11
BP 1539
EP 1552
DI 10.1586/ERV.11.141
PG 14
WC Immunology
WE Science Citation Index Expanded (SCI-EXPANDED)
SC Immunology
GA 858MS
UT WOS:000297803200014
PM 22043954
DA 2025-05-07
ER

PT J
AU McDade, TW
   Worthman, CM
AF McDade, TW
   Worthman, CM
TI Evolutionary process and the ecology of human immune function
SO AMERICAN JOURNAL OF HUMAN BIOLOGY
LA English
DT Article
ID AGE-RELATED-CHANGES; CELLULAR IMMUNOSUPPRESSION; THYMUS INTERACTIONS;
   SYSTEM; INFECTION; SUBPOPULATIONS; COMPETITION; HOMEOSTASIS;
VIRULENCE;
   CHILDREN
AB Evolutionary principles inform central design features of human immune defenses and provide key insights into this complicated host defense system. This article explores the selection pressures and adaptive responses that have elaborated the immune system over the course of evolution and discusses their implications for understanding contemporary immune development and function. Special attention is given to the challenges posed by diverse, rapidly evolving pathogens and the mammalian response to these challenges. The process of lymphocyte diversity generation and subsequent clonal selection is quintessentially Darwinian: pathogens provide selection pressure that drives differential replication of host immune cell lines, resulting in changes in genetic frequencies within an individual's population of lymphocytes. The immune system also incorporates nongenetic transgenerational processes in the transfer of

antibodies from mother to offspring through the placenta and breast milk. The consequences of these observations for human development, health, and the ecology of immune function are considered throughout the life cycle. Specifically, evolutionary processes provide insight into autoimmunity, thymic function, lymphocyte development, infectious disease risk, and lactation. While much work in evolutionary medicine focuses on the discordance between evolved biology and rapidly changing cultural environments, with respect to the immune system, evolutionary processes may be most revealing when applied within individuals. (C) 1999 Wiley-Liss, Inc.
C1 Emory Univ, Dept Anthropol, Atlanta, GA 30322 USA.
C3 Emory University
RP Emory Univ, Dept Anthropol, Atlanta, GA 30322 USA.
EM tmcdade@emory.edu
OI Worthman, Carol M/0000-0002-5397-2298
CR ANDERSON R M, 1991
ARMELAGOS G, IN PRESS EVOLUTIONAR
Billington WD., 1992, IMMUNOLOGICAL OBSTET, P290
BULL JJ, 1994, EVOLUTION, V48, P1423, DOI 10.1111/j.1558-5646.1994.tb02185.x
Buss L.W., 1987, The evolution of individuality
CARDARELLI NF, 1989, THYMUS HLTH SENESCEN
CHANGEAUX JP, 1985, NEURONAL MAN BIOL MI
Cotman CW., 1987, NEUROIMMUNE ENDOCRIN
DENNY TN, 1990, PEDIATR RES, V27, pA155
DUPASQUIER L, 1992, APMIS, V100, P383
EDELMAN GM, 1987, IMMUNOL REV, V100, P11, DOI 10.1111/j.1600-065X.1987.tb00526.x
EDELMAN GM, 1994, GENETICS, V138, P975
Ewald P W, 1994, Evolution of Infectious Diseases
FABRIS N, 1992, ANN NY ACAD SCI, V663, P335, DOI 10.1111/j.1749-6632.1992.tb38677.x
FABRIS N, 1988, ANN NY ACAD SCI, V521, P72, DOI 10.1111/j.1749-6632.1988.tb35266.x
FABRIS N, 1989, HORM RES, V31, P85, DOI 10.1159/000181093
FEACHEM RG, 1984, B WORLD HEALTH ORGAN, V62, P271
FLINN MV, 1996, AM J PHYS ANTHR S, V22, P105
FRANK MM, 1995, SAMTERS IMMUNOLOGIC, V2
GALA RR, 1991, P SOC EXP BIOL MED, V198, P513
Gearhart Patricia J., 1993, P865
GOYA RG, 1992, EXP CLIN IMMUNOGENET, V9, P188
GREGORY RL, 1994, LAB MED, V25, P724, DOI 10.1093/labmed/25.11.724
HADDEN JW, 1992, ANN NY ACAD SCI, V673, P231, DOI 10.1111/j.1749-6632.1992.tb27458.x
HANNET I, 1992, IMMUNOL TODAY, V13, P215, DOI 10.1016/0167-5699(92)90157-3
HANSON LA, 1989, IMMUNOLOGIC DISORDER, P116
HART BL, 1990, NEUROSCI BIOBEHAV R, V14, P273, DOI 10.1016/S0149-7634(05)80038-7
HICKS MJ, 1983, AM J CLIN PATHOL, V80, P159, DOI 10.1093/ajcp/80.2.159
HICKS MJ, 1983, ARCH PATHOL LAB MED, V107, P518
HOFF C, 1990, AM J HUM BIOL, V2, P419, DOI 10.1002/ajhb.1310020409
HOFF C, 1991, HUM BIOL, V63, P129

HOSHOWER LM, 1994, AM J PHYS ANTHROPOL, V94, P421, DOI 10.1002/ajpa.1330940310
JAMES SP, 1993, DIGEST DIS, V11, P146, DOI 10.1159/000171407
KAY MMB, 1979, J INVEST DERMATOL, V73, P29, DOI 10.1111/1523-1747.ep12532756
KELLER MA, 1992, IMMUNOLOGICAL OBSTET, P315
KELLEY KW, 1987, ANN NY ACAD SCI, V496, P91, DOI 10.1111/j.1749-6632.1987.tb35750.x
KLUGER MJ, 1986, YALE J BIOL MED, V59, P89
Kuby J., 1994, IMMUNOLOGY, V2nd
Levin Bruce R., 1994, Trends in Microbiology, V2, P76, DOI 10.1016/0966-842X(94)90538-X
LEWIS DB, 1992, FETAL NEONATAL PHYSL, P1404
MARCHALONIS JJ, 1994, ANN NY ACAD SCI, V712, P1
McDade TW, 1998, J DEV BEHAV PEDIATR, V19, P286, DOI 10.1097/00004703-199808000-00008
McDade TW, 1998, AM J HUM BIOL, V10, P131
MCDADE TW, 1996, AM J PHYS ANTHR S, V22, P162
McLean AR, 1997, P NATL ACAD SCI USA, V94, P5792, DOI 10.1073/pnas.94.11.5792
Mittler JE, 1996, J ACQ IMMUN DEF SYND, V12, P233, DOI 10.1097/00042560-199607000-00003
MULLERHERMELINK HK, 1982, ADV EXP MED BIOL, V149, P303
NAYAK N, 1987, J INFECT DIS, V155, P724, DOI 10.1093/infdis/155.4.724
OGRA PL, 1990, INFECT DIS, P68
Paul W. E., 2012, Fundamental Immunology
PCIKERING LK, 1986, HUMAN LACTATION, V2, P499
PIRENNE H, 1992, PEDIATR RES, V32, P81, DOI 10.1203/00006450-199207000-00016
PROFET M, 1991, Q REV BIOL, V66, P23, DOI 10.1086/417049
Rakic Pasko, 1995, P127
REINISCH CL, 1989, IMMUNOL TODAY, V10, P278, DOI 10.1016/0167-5699(89)90142-4
ROITT I.M., 1993, IMMUNOLOGY, V3rd
Rowe G. W., 1994, THEORETICAL MODELS B
SAXON A, 1989, IMMUNOLOGIC DISORDER, P40
Scammon R.E., 1930, MEASUREMENT MAN, P171
SCHUURS AHWM, 1990, J STEROID BIOCHEM, V35, P157, DOI 10.1016/0022-4731(90)90270-3
SHELLDUNCAN B, 1993, AM J HUM BIOL, V5, P225, DOI 10.1002/ajhb.1310050212
ShellDuncan B, 1997, AM J HUM BIOL, V9, P381, DOI 10.1002/(SICI)1520-6300(1997)9:3<381::AID-AJHB10>3.0.CO;2-I
SHKHINEK EK, 1985, NEUROHUMORAL MAINTEN, P98
SPRENT J, 1993, CURR OPIN IMMUNOL, V5, P433, DOI 10.1016/0952-7915(93)90065-Z
Sprent Jonathan, 1993, P75
STEINMANN GG, 1985, SCAND J IMMUNOL, V22, P563, DOI 10.1111/j.1365-3083.1985.tb01916.x
TONEGAWA S, 1983, NATURE, V302, P575, DOI 10.1038/302575a0
TOSI P, 1982, CLIN EXP IMMUNOL, V47, P497
TRAVIS J, 1993, SCIENCE, V261, P164, DOI 10.1126/science.8327890
Ulijaszek S, 1997, COLLEGIUM ANTROPOL, V21, P3
Weisz-Carrington P., 1987, HORMONES IMMUNITY, P172

WILLIAMS GC, 1991, Q REV BIOL, V66, P1, DOI 10.1086/417048
WILSON CB, 1990, INFECT DIS, P17
ZINKERNAGEL RM, 1985, BRIT MED BULL, V41, P92, DOI 10.1093/oxfordjournals.bmb.a072033
[No title captured]
NR 75
TC 36
Z9 50
U1 0
U2 19
PU WILEY
PI HOBOKEN
PA 111 RIVER ST, HOBOKEN 07030-5774, NJ USA
SN 1042-0533
EI 1520-6300
J9 AM J HUM BIOL
JI Am. J. Hum. Biol.
PD NOV-DEC
PY 1999
VL 11
IS 6
BP 705
EP 717
DI 10.1002/(SICI)1520-6300(199911/12)11:6<705::AID-AJHB1>3.0.CO;2-G
PG 13
WC Anthropology; Biology
WE Science Citation Index Expanded (SCI-EXPANDED); Social Science Citation Index (SSCI)
SC Anthropology; Life Sciences & Biomedicine - Other Topics
GA 251UZ
UT WOS:000083465900001
PM 11533987
DA 2025-05-07
ER

PT J
AU Serebrova, VN
Trifonova, EA
Stepanov, VA
AF Serebrova, V. N.
Trifonova, E. A.
Stepanov, V. A.
TI Natural Selection as a Driver for the Genetic Component of Preeclampsia
SO MOLECULAR BIOLOGY
LA English
DT Article
DE preeclampsia; differentially expressed genes; regulatory single nucleotide polymorphisms; human populations; genetic diversity; natural selection
ID C/EBP-BETA; EVOLUTIONARY ORIGINS; OXIDATIVE STRESS; PLACENTAL TISSUE; DOWN-REGULATION; EXPRESSION; ASSOCIATION; LEPTIN; DIFFERENTIATION; PREGNANCY

AB Preeclampsia (PE) is a severe hypertensive pathology and affects 2-8% of pregnancies worldwide. Its etiopathogenesis is poorly understood, and prognostic biomarkers and effective treatments are unavailable for this pregnancy complication, determining the high rates of maternal and perinatal morbidity and mortality. Racial and ethnic differences in PE incidence are of interest to study in terms of evolutionary medicine because such variability can be considered as a side effect of adaptive changes that have occurred in the genetic structure of modern populations since the dispersal of Homo sapiens from Africa. Genetic diversity at 10 regulatory single nucleotide polymorphisms (rSNPs) associated with PE was studied in North Eurasian populations and world populations of the 1000 Genomes Project. The role of natural selection in the formation of this genetic diversity was assessed at the microevolutionary level. High interpopulation diversity was observed with the greatest contribution being made by allele frequencies of NDRG1 rs3802252 (F-ST = 0.157). Signatures of natural selection were detected for rs10423795 of LHB, rs2167270 of LEP, rs2227262 and rs3802252 of NDRG1, rs56153523 and rs8109071 of SYDE1, and rs72959687 of INHA. The results are consistent with two evolutionary hypotheses of PE, namely, those of ancestral susceptibility and genetic conflicts.
C1 [Serebrova, V. N.; Trifonova, E. A.; Stepanov, V. A.] Russian Acad Sci, Tomsk Natl Med Res Ctr, Inst Med Genet, Tomsk 634050, Russia.
C3 Russian Academy of Sciences; Tomsk National Research Medical Center; Research Institute of Medical Genetics - Tomsk
RP Serebrova, VN (autor correspondente), Russian Acad Sci, Tomsk Natl Med Res Ctr, Inst Med Genet, Tomsk 634050, Russia.
EM vika.serebrova@medgenetics.ru
RI Stepanov, Vadim/G-4320-2013; Serebrova, Victoria/A-8176-2014; Trifonova,
Ekaterina/A-8975-2014
OI Trifonova, Ekaterina/0000-0003-1311-7403
FU Russian Foundation for Basic Research [18-29-13045]
FX This work was supported by the Russian Foundation for Basic Research (project no. 18-29-13045).
CR Abrams ET, 2011, AM ANTHROPOL, V113, P417, DOI 10.1111/j.1548-1433.2011.01351.x
Ahmed SIY, 2017, MED HYPOTHESES, V104, P128, DOI 10.1016/j.mehy.2017.05.007
Auton A., 2015, NATURE, V526, P68
Belomestnov S.R., 2016, KLINICHESKIE REKOMEN
Boyle AP, 2012, GENOME RES, V22, P1790, DOI 10.1101/gr.137323.112
Brown EA, 2013, TRENDS GENET, V29, P585, DOI 10.1016/j.tig.2013.03.001
Brown IJ, 2009, INT J EPIDEMIOL, V38, P791, DOI 10.1093/ije/dyp139
Burton GJ, 2019, BMJ-BRIT MED J, V366, DOI 10.1136/bmj.l2381
Cassano S, 2014, METABOLISM, V63, P1272, DOI 10.1016/j.metabol.2014.06.010
Chaline J, 2003, J REPROD IMMUNOL, V59, P137, DOI 10.1016/S0165-0378(03)00043-3
Chen B, 2006, J BIOL CHEM, V281, P2764, DOI 10.1074/jbc.M507330200
Cole LA, 2008, J REPROD MED, V53, P549
Crosley EJ, 2013, PLACENTA, V34, P127, DOI 10.1016/j.placenta.2012.12.001
Di Rienzo A, 2005, TRENDS GENET, V21, P596, DOI 10.1016/j.tig.2005.08.007

Ellen TP, 2008, CARCINOGENESIS, V29, P2, DOI 10.1093/carcin/bgm200
Elliot MG, 2016, J REPROD IMMUNOL, V114, P75, DOI 10.1016/j.jri.2016.02.003
Elliot MG, 2015, PHILOS T R SOC B, V370, DOI 10.1098/rstb.2014.0069
Enquobahrie DA, 2008, AM J OBSTET GYNECOL, V199, DOI 10.1016/j.ajog.2008.04.020
Fu YX, 1997, GENETICS, V147, P915
Gathiram P, 2016, CARDIOVASC J AFR, V27, P71, DOI 10.5830/CVJA-2016-009
Gong J, 2012, PAEDIATR PERINAT EP, V26, P45, DOI 10.1111/j.1365-3016.2011.01222.x
Gormley M, 2017, AM J OBSTET GYNECOL, V217, DOI 10.1016/j.ajog.2017.03.017
HAIG D, 1993, Q REV BIOL, V68, P495, DOI 10.1086/418300
Handa VL, 2008, OBSTET GYNECOL, V111, P914, DOI 10.1097/AOG.0b013e318169ce03
Haram K, 2014, J PREGNANCY, V2014, DOI 10.1155/2014/910751
He P, 2014, ENDOCRINOLOGY, V155, P299, DOI 10.1210/en.2013-1350
Hedrick P.W., 2000, GENETICS POPULATIONS
Huggins CJ, 2013, MOL CELL BIOL, V33, P3242, DOI 10.1128/MCB.01674-12
Kaartokallio T, 2016, SCI REP-UK, V6, DOI 10.1038/srep29085
Kalinderis M, 2015, ARCH GYNECOL OBSTET, V292, P343, DOI 10.1007/s00404-015-3659-4
Kim ES, 2014, ONCOGENE, V33, P3583, DOI 10.1038/onc.2013.319
Kumar P, 2013, MOL CELL BIOL, V33, P1782, DOI 10.1128/MCB.01228-12
Lapaire O, 2012, FETAL DIAGN THER, V31, P147, DOI 10.1159/000337325
Liu N, 2016, NUCLEIC ACIDS RES, V44, P8693, DOI 10.1093/nar/gkw549
Lo HF, 2017, J PATHOL, V241, P324, DOI 10.1002/path.4835
Louwen F, 2012, ONCOTARGET, V3, P759
Lu W, 2020, MOL MED REP, V21, P1208, DOI 10.3892/mmr.2019.10901
Matarese G, 2005, J IMMUNOL, V174, P3137, DOI 10.4049/jimmunol.174.6.3137
Meng T, 2012, OMICS, V16, P301, DOI 10.1089/omi.2011.0066
Nakimuli A, 2014, AM J OBSTET GYNECOL, V210, P510, DOI 10.1016/j.ajog.2013.10.879
Nishizawa H, 2011, REPROD BIOL ENDOCRIN, V9, DOI 10.1186/1477-7827-9-107
Paiwattananupant K, 2008, HYPERTENS PREGNANCY, V27, P337, DOI 10.1080/10641950802020545
Phipps EA, 2019, NAT REV NEPHROL, V15, P275, DOI 10.1038/s41581-019-0119-6
Pijnenborg R, 2008, HYPERTENS PREGNANCY, V27, P183, DOI 10.1080/10641950701826711
Radde J, 2004, INT J GYNECOL PATHOL, V23, P373, DOI 10.1097/01.pgp.0000139645.32997.e1
Raghavan M, 2015, SCIENCE, V349, DOI 10.1126/science.aab3884
Ramathal C, 2011, J BIOL CHEM, V286, P19860, DOI 10.1074/jbc.M110.191759
Reimer T, 2002, MOL HUM REPROD, V8, P674, DOI 10.1093/molehr/8.7.674
Robillard PY, 2017, J REPROD IMMUNOL, V123, P72, DOI 10.1016/j.jri.2017.09.006
Robillard PY, 2002, AM J REPROD IMMUNOL, V47, P104, DOI 10.1034/j.1600-0897.2002.1o043.x

[Сереброва В.Н. Serebrova V.N.], 2019, [Медицинская генетика, Medical Genetics, Meditsinskaya genetika], V18, P35, DOI 10.25557/2073-7998.2019.01.35-38
[Сереброва В.Н. Serebrova V.N.], 2018, [Медицинская генетика, Meditsinskaya genetika], V17, P32, DOI 10.25557/2073-7998.2018.01.32-36
Serebrova VN, 2016, MOL BIOL+, V50, P768, DOI 10.1134/S0026893316050162
Serebrova V.N., 2018, RES RESULTS BIOMED, V4, P38, DOI [10.18413/2313-8955-2018-4-3-0-4, DOI 10.18413/2313-8955-2018-4-3-0-4]
Serebrova V.N., 2019, MEZHD K 7 SEZD VAV O, P673
Shimono A, 1999, MECH DEVELOP, V83, P39, DOI 10.1016/S0925-4773(99)00025-8
Sitras V, 2009, PLACENTA, V30, P424, DOI 10.1016/j.placenta.2009.01.012
Szklarczyk D, 2017, NUCLEIC ACIDS RES, V45, pD362, DOI 10.1093/nar/gkw937
TAJIMA F, 1989, GENETICS, V123, P585
Tannetta D, 2013, CURR HYPERTENS REP, V15, P590, DOI 10.1007/s11906-013-0395-7
Taylor BD, 2015, HYPERTENSION, V65, P594, DOI 10.1161/HYPERTENSIONAHA.114.03979
Than NG, 2014, PLACENTA, V35, P855, DOI 10.1016/j.placenta.2014.07.015
Tinel M, 2003, J HEPATOL, V39, P171, DOI 10.1016/S0168-8278(03)00238-1
Trifonova EA, 2020, RUSS J GENET+, V56, P513, DOI 10.1134/S1022795420050130
Trifonova EA, 2014, ACTA NATURAE, V6, P71, DOI 10.32607/20758251-2014-6-2-71-83
Vaiman D, 2016, PLOS ONE, V11, DOI 10.1371/journal.pone.0165849
Vaiman D, 2013, PLOS ONE, V8, DOI 10.1371/journal.pone.0065498
Várkonyi T, 2011, PLACENTA, V32, pS21, DOI 10.1016/j.placenta.2010.04.014
Wang W, 2012, MOL ENDOCRINOL, V26, P2016, DOI 10.1210/me.2012-1169
WATTERSON GA, 1975, THEOR POPUL BIOL, V7, P256, DOI 10.1016/0040-5809(75)90020-9
Weedon-Fekjær MS, 2014, PLACENTA, V35, P709, DOI 10.1016/j.placenta.2014.07.002
Wu KJ, 1999, SCIENCE, V283, P676, DOI 10.1126/science.283.5402.676
Wu YF, 2016, EXP THER MED, V12, P201, DOI 10.3892/etm.2016.3261
Xiao J, 2014, J HUM HYPERTENS, V28, P694, DOI 10.1038/jhh.2013.148
Zadora J, 2017, CIRCULATION, V136, P1824, DOI 10.1161/CIRCULATIONAHA.117.028110
Zhang GX, 2016, MED SCI MONITOR, V22, P2202, DOI 10.12659/MSM.896552
Zhang M, 2016, P NATL ACAD SCI USA, V113, pE7069, DOI 10.1073/pnas.1607849113
Zhang YC, 2018, J MOL ENDOCRINOL, V60, P133, DOI 10.1530/JME-17-0180
Zhou L, 2016, INFLAMM RES, V65, P717, DOI 10.1007/s00011-016-0953-y
Zhuang B, 2015, PLACENTA, V36, P839, DOI 10.1016/j.placenta.2015.06.016
NR 80
TC 0
Z9 0
U1 0
U2 0
PU PLEIADES PUBLISHING INC

PI NEW YORK
PA PLEIADES HOUSE, 7 W 54 ST, NEW YORK, NY, UNITED STATES
SN 0026-8933
EI 1608-3245
J9 MOL BIOL+
JI Mol. Biol.
PD MAY
PY 2021
VL 55
IS 3
BP 381
EP 397
DI 10.1134/S0026893321020308
PG 17
WC Biochemistry & Molecular Biology
WE Science Citation Index Expanded (SCI-EXPANDED)
SC Biochemistry & Molecular Biology
GA XP5UD
UT WOS:000730929300008
OA Bronze
DA 2025-05-07
ER

PT J
AU Antolin, MF
   Jenkins, KP
   Bergstrom, CT
   Crespi, BJ
   De, S
   Hancock, A
   Hanley, KA
   Meagher, TR
   Moreno-Estrada, A
   Nesse, RM
   Omenn, GS
   Stearns, SC
AF Antolin, Michael F.
   Jenkins, Kristin P.
   Bergstrom, Carl T.
   Crespi, Bernard J.
   De, Subhajyoti
   Hancock, Angela
   Hanley, Kathryn A.
   Meagher, Thomas R.
   Moreno-Estrada, Andres
   Nesse, Randolph M.
   Omenn, Gilbert S.
   Stearns, Stephen C.
TI EVOLUTION AND MEDICINE IN UNDERGRADUATE EDUCATION: A PRESCRIPTION FOR
   ALL BIOLOGY STUDENTS
SO EVOLUTION
LA English
DT Review
DE Biomedical research; evolution of disease; host-pathogen interactions;

human genetics; medical practice; mismatch hypothesis; premedical curriculum; public health; science education
ID DEGENERATIVE DISEASES; ANTIBIOTIC-RESISTANCE; POPULATION-GENETICS; GLOBAL DISTRIBUTION; POSITIVE SELECTION; ASSOCIATION; VARIANT; CANCER; VIRUS; PIGMENTATION
AB The interface between evolutionary biology and the biomedical sciences promises to advance understanding of the origins of genetic and infectious diseases in humans, potentially leading to improved medical diagnostics, therapies, and public health practices. The biomedical sciences also provide unparalleled examples for evolutionary biologists to explore. However, gaps persist between evolution and medicine, for historical reasons and because they are often perceived as having disparate goals. Evolutionary biologists have a role in building a bridge between the disciplines by presenting evolutionary biology in the context of human health and medical practice to undergraduates, including premedical and preprofessional students. We suggest that students will find medical examples of evolution engaging. By making the connections between evolution and medicine clear at the undergraduate level, the stage is set for future health providers and biomedical scientists to work productively in this synthetic area. Here, we frame key evolutionary concepts in terms of human health, so that biomedical examples may be more easily incorporated into evolution courses or more specialized courses on evolutionary medicine. Our goal is to aid in building the scientific foundation in evolutionary biology for all students, and to encourage evolutionary biologists to join in the integration of evolution and medicine.
C1 [Antolin, Michael F.] Colorado State Univ, Dept Biol, Ft Collins, CO 80523 USA.
[Bergstrom, Carl T.] Univ Washington, Dept Biol, Seattle, WA 98195 USA.
[Crespi, Bernard J.] Simon Fraser Univ, Dept Biol Sci, Burnaby, BC V5A 1S6, Canada.
[De, Subhajyoti] Harvard Univ, Sch Publ Hlth, Dana Farber Canc Inst, Dept Biostat & Computat Biol, Boston, MA 02115 USA.
[Hancock, Angela] Univ Chicago, Dept Ecol & Evolut, Chicago, IL 60637 USA.
[Hanley, Kathryn A.] New Mexico State Univ, Dept Biol, Las Cruces, NM 88003 USA.
[Meagher, Thomas R.] Univ St Andrews, Sch Biol, St Andrews KY15 4RP, Fife, Scotland.
[Moreno-Estrada, Andres] Stanford Univ, Sch Med, Dept Genet, Stanford, CA 94305 USA.
[Nesse, Randolph M.] Univ Michigan, Dept Psychiat, Ann Arbor, MI 48109 USA.
[Omenn, Gilbert S.] Univ Michigan, Sch Publ Hlth, Dept Internal Med, Ctr Computat Med & Bioinformat, Ann Arbor, MI 48109 USA.
[Stearns, Stephen C.] Yale Univ, Dept Ecol & Evolutionary Biol, New Haven, CT 06520 USA.
[Omenn, Gilbert S.] Univ Michigan, Sch Publ Hlth, Ctr Computat Med & Bioinformat, Dept Human Genet, Ann Arbor, MI 48109 USA.
[Jenkins, Kristin P.] Natl Evolutionary Synth Ctr, Durham, NC 27705 USA.
C3 Colorado State University System; Colorado State University Fort Collins; University of Washington; University of Washington Seattle;

Simon Fraser University; Harvard University; Harvard University Medical
Affiliates; Dana-Farber Cancer Institute; Harvard T.H. Chan School of Public Health; University of Chicago; New Mexico State University; University of St Andrews; Stanford University; University of Michigan System; University of Michigan; University of Michigan System; University of Michigan; Yale University; University of Michigan System;
University of Michigan; The National Evolutionary Synthesis Center
RP Antolin, MF (autor correspondente), Colorado State Univ, Dept Biol, Ft Collins, CO 80523 USA.
EM michael.antolin@colostate.edu
RI ANTOLIN, MICHAEL/AAT-5971-2021; Bergstrom, Carl/H-2299-2011; Stearns, Stephen/F-4099-2012; Moreno-Estrada, Andres/R-3950-2017
OI Bergstrom, Carl/0000-0002-2070-385X; Stearns, Stephen/0000-0002-6621-4373; Antolin, Michael Franc/0000-0001-6498-617X;
Nesse, Randolph/0000-0003-1768-0949; Jenkins, Kristin/0000-0002-7795-4270; Moreno-Estrada, Andres/0000-0001-8329-8292;
Omenn, Gilbert S./0000-0002-8976-6074
FU Education and Outreach Committee of the Society for the Study of Evolution; National Evolutionary Synthesis Center
FX Financial support for this symposium was provided by the Education and Outreach Committee of the Society for the Study of Evolution, by the National Evolutionary Synthesis Center, and by the co-authors home institutions. We thank the local organizers of the 2011 Evolution meetings, particularly R. Broughton, I. Schlupp, and L. Weider (special
thanks for the use of the bicycle!).
CR Acuña-Alonzo V, 2010, HUM MOL GENET, V19, P2877, DOI 10.1093/hmg/ddq173
Aitken William, 1885, Glasgow Med J, V24, P98
Alcock J., 2011, Evol Educ Outreach, P574, DOI [DOI 10.1007/S12052-011-0362-1, https://doi.org/10.1007/s12052-011-0362-1]
ALLISON AC, 1954, BRIT MED J, V1, P290, DOI 10.1136/bmj.1.4857.290
Ambrose Susan A, 2010, How Learning Works: Seven Research-Based Principles for Smart Teaching
Anderson W, 2004, OSIRIS, V19, P39, DOI 10.1086/649393
Andersson DI, 2010, NAT REV MICROBIOL, V8, P260, DOI 10.1038/nrmicro2319
[Anonymous], EVOL ED OUTREACH
[Anonymous], 2006, Population Genetics and Microevolutionary Theory
[Anonymous], 2009, SCI FDN FUT PHYS
[Anonymous], 1998, TEACH EV NAT SCI
[Anonymous], B CARNEGIE FDN ADV T
[Anonymous], WE GET SICK NEW SCI
Antolin M. F., 2011, CONCHIS OMN IN PRESS
Antolin MichaelF., 2009, Evolution, P281
Antolini M., 2011, EVOL EDUCA OUTREACH, V4, P613, DOI [10.1007/s12052-011-0372-z, DOI 10.1007/S12052-011-0372-Z]
Antonovics J, 2007, PLOS BIOL, V5, P137, DOI 10.1371/journal.pbio.0050030
Auton A, 2009, GENOME RES, V19, P795, DOI 10.1101/gr.088898.108

Bach JF, 2002, NEW ENGL J MED, V347, P911, DOI 10.1056/NEJMra020100
Bateson P, 2004, NATURE, V430, P419, DOI 10.1038/nature02725
Bean DC, 2005, J ANTIMICROB CHEMOTH, V56, P962, DOI 10.1093/jac/dki332
Beardsley P. M., 2011, EVOL EDUC OUTREACH, V4, P603, DOI [10.1007/s12052-011-0361-2, DOI 10.1007/S12052-011-0361-2]
Bergstrom C. T., 2007, EVOLUTION HLTH DIS, P122
Bergstrom CT., 2011, EVOLUTION
Bransford J. D., 2000, PEOPLE LEARN BRAIN M, DOI [10.17226/9853, DOI 10.17226/9853]
Braude S, 1999, BEHAV ECOL, V10, P345, DOI 10.1093/beheco/10.3.345
Bribiescas R. G., 2007, Evolution in Health and Disease, P77, DOI DOI 10.1093/ACPROF:OSO/9780199207466.003.0007
Buklijas T., 2011, EVOLUTION ED OUTREAC, V4, P595, DOI [10.1007/s12052-011-0374-x, DOI 10.1007/S12052-011-0374-X), DOI 10.1007/S12052-011-0374-X]
Burch CL, 2003, J EVOLUTION BIOL, V16, P1223, DOI 10.1046/j.1420-9101.2003.00632.x
Burnet M., 1972, The Natural History of Infectious Disease
Bynum W F, 1983, Gesnerus, V40, P43
Carey JV, 2010, J CLIN NURS, V19, P2377, DOI 10.1111/j.1365-2702.2010.03258.x
Casas-Selves Matias, 2011, Evolution (N Y), V4, P624
Cavalli-Sforza LL, 2003, NAT GENET, V33, P266, DOI 10.1038/ng1113
Childs B, 2005, ANNU REV GENOM HUM G, V6, P313, DOI 10.1146/annurev.genom.6.080604.162345
College Board, 2011, ADV AP
Coller BS, 1997, J CLIN INVEST, V99, P1467, DOI 10.1172/JCI119307
Cordain L, 2005, AM J CLIN NUTR, V81, P341, DOI 10.1093/ajcn.81.2.341
Correale J, 2007, ANN NEUROL, V61, P97, DOI 10.1002/ana.21067
Costa V, 2010, J NUTR BIOCHEM, V21, P457, DOI 10.1016/j.jnutbio.2009.10.012
Crespi B, 2008, BEHAV BRAIN SCI, V31, P241, DOI 10.1017/S0140525X08004214
Crespi BJ, 2011, EPIGENETICS HUM HLTH, P143, DOI 10.1007/978-3-642-17426-1_8
Darwin C., 1871, P423
Darwin Charles., 1875, VARIATION ANIMALS PL
Darwin E ., 1801, ZOONOMIA ORGANIC LAW
De S, 2011, TRENDS GENET, V27, P217, DOI 10.1016/j.tig.2011.03.002
Diamond J, 2003, NATURE, V423, P599, DOI 10.1038/423599a
Downie J., 2004, BIOSCIENCE ED E J, V4, P1
EATON SB, 1988, AM J MED, V84, P739, DOI 10.1016/0002-9343(88)90113-1
Eckert LO, 1998, OBSTET GYNECOL, V92, P757, DOI 10.1016/S0029-7844(98)00264-6
Enattah NS, 2002, NAT GENET, V30, P233, DOI 10.1038/ng826
FDA, 2011, VACC BLOOD BIOL
Fumagalli M, 2010, PLOS GENET, V6, DOI 10.1371/journal.pgen.1000849
Galvani AP, 2003, TRENDS ECOL EVOL, V18, P132, DOI 10.1016/S0169-5347(02)00050-2
Genereux D.P., 2005, Evolutionary Science, P145
Gluckman PeterD., 2009, Principles of evolutionary medicine
Greaves M, 2002, LANCET ONCOL, V3, P244, DOI 10.1016/S1470-2045(02)00716-7
Gregg C, 2010, SCIENCE, V329, P643, DOI 10.1126/science.1190830

Grice EA, 2011, NAT REV MICROBIOL, V9, P244, DOI 10.1038/nrmicro2537
Grossman SR, 2010, SCIENCE, V327, P883, DOI 10.1126/science.1183863
Haig D, 2004, ANNU REV GENET, V38, P553, DOI 10.1146/annurev.genet.37.110801.142741
Haldane J. B. S., 1949, 8 INT C GEN STOCKH S
HALDANE JBS, 1964, PERSPECT BIOL MED, V7, P343
Hanahan D, 2011, CELL, V144, P646, DOI 10.1016/j.cell.2011.02.013
Hancock AM, 2011, PLOS GENET, V7, DOI 10.1371/journal.pgen.1001375
Hancock AM, 2010, P NATL ACAD SCI USA, V107, P8924, DOI 10.1073/pnas.0914625107
Hanley Kathryn A, 2011, Evolution (N Y), V4, P635
Harper R. M. J., 1975, EVOLUTION ILLNESS
Harris Eugene E, 2005, BMC Med Educ, V5, P16, DOI 10.1186/1472-6920-5-16
HARVEY JJ, 1964, NATURE, V204, P1104, DOI 10.1038/2041104b0
Hillis DM, 2007, EVOLUTION, V61, P1261, DOI 10.1111/j.1558-5646.2007.00126.x
Huxley T H, 1881, Br Med J, V2, P273
Itan Y, 2009, PLOS COMPUT BIOL, V5, DOI 10.1371/journal.pcbi.1000491
Jablonski NG, 2000, J HUM EVOL, V39, P57, DOI 10.1006/jhev.2000.0403
Janzen V, 2006, NATURE, V443, P421, DOI 10.1038/nature05159
Jenkins K. P., 2011, EVOL ED OUTREACH, V4, P556, DOI DOI 10.1007/S12052-011-0375-9
Jiang YQ, 2010, BIOMARK MED, V4, P523, DOI 10.2217/BMM.10.66
JOHNSON RL, 1987, AM BIOL TEACH, V49, P93, DOI 10.2307/4448445
Katzmarzyk PT, 1998, AM J PHYS ANTHROPOL, V106, P483, DOI 10.1002/(SICI)1096-8644(199808)106:4<483::AID-AJPA4>3.3.CO;2-K
Kew OM, 2005, ANNU REV MICROBIOL, V59, P587, DOI 10.1146/annurev.micro.58.030603.123625
KIRSTEN WH, 1970, BIBL HAEMATOL, V1969, P246
Knight JC, 2009, QJM-INT J MED, V102, P757, DOI 10.1093/qjmed/hcp115
Krizhanovsky V, 2009, NATURE, V460, P1085, DOI 10.1038/4601085a
Lamason RL, 2005, SCIENCE, V310, P1782, DOI 10.1126/science.1116238
Lederberg J, 2000, SCIENCE, V288, P287, DOI 10.1126/science.288.5464.287
Levin BR, 2000, GENETICS, V154, P985
Levin BR, 1997, CLIN INFECT DIS, V24, pS9, DOI 10.1093/clinids/24.Supplement_1.S9
MAYR E, 1961, SCIENCE, V134, P1501, DOI 10.1126/science.134.3489.1501
Méthot PO, 2011, THEOR MED BIOETH, V32, P75, DOI 10.1007/s11017-010-9167-4
Meyer UA, 1999, EVOLUTION IN HEALTH AND DISEASE, P41
Monath T CM., 2008, Vaccines, V5th, P959
Morton DJ, 1926, SCIENCE, V64, P394, DOI 10.1126/science.64.1660.394
Murgia C, 2006, CELL, V126, P477, DOI 10.1016/j.cell.2006.05.051
Nelson DR, 2004, PHARMACOGENETICS, V14, P1, DOI 10.1097/00008571-200401000-00001
Nesse RM, 2008, EVOL APPL, V1, P28, DOI 10.1111/j.1752-4571.2007.00006.x
Nesse RM, 2011, EVOL APPL, V4, P264, DOI 10.1111/j.1752-4571.2010.00181.x
Nesse RM, 2010, P NATL ACAD SCI USA, V107, P1800, DOI 10.1073/pnas.0906224106

Nesse RM, 2003, BIOSCIENCE, V53, P585, DOI 10.1641/0006-3568(2003)053[0585:EBITMS]2.0.CO;2
Nielsen R, 2009, GENOME RES, V19, P838, DOI 10.1101/gr.088336.108
NSF/AAAS, 2011, VIS CHANG UND BIOL E
Omenn Gilbert S, 2011, Evolution (N Y), V4, P567
Omenn GS, 2010, P NATL ACAD SCI USA, V107, P1702, DOI 10.1073/pnas.0906198106
Osada Y, 2010, J BIOMED BIOTECHNOL, DOI 10.1155/2010/743758
Park KJ, 2011, J GEN VIROL, V92, P36, DOI 10.1099/vir.0.024992-0
Pickrell JK, 2009, GENOME RES, V19, P826, DOI 10.1101/gr.087577.108
Piel FB, 2010, NAT COMMUN, V1, DOI 10.1038/ncomms1104
Plotkin S. A., 2001, HIST VACCINE DEV
Poolman EM, 2007, J R SOC INTERFACE, V4, P91, DOI 10.1098/rsif.2006.0154
Porter R., 1998, GREATEST BENEFIT MAN
Reddy A, 2009, PARASITOL RES, V104, P217, DOI 10.1007/s00436-008-1297-5
Reich DE, 2001, TRENDS GENET, V17, P502, DOI 10.1016/S0168-9525(01)02410-6
Reid G, 2001, AM J CLIN NUTR, V73, p437S, DOI 10.1093/ajcn/73.2.437s
Rieder E, 2001, Dev Biol (Basel), V105, P111
Rimoin AW, 2010, P NATL ACAD SCI USA, V107, P16262, DOI 10.1073/pnas.1005769107
Roberts D.F., 1978, CLIMATE HUMAN VARIAB, VSecond
Rook GAW, 2009, PROG INFLAMM RES SER, P1, DOI 10.1007/978-3-7643-8903-1_1
Rosenberg NA, 2010, NAT REV GENET, V11, P356, DOI 10.1038/nrg2760
Schrag SJ, 1996, NATURE, V381, P120, DOI 10.1038/381120b0
Seppala H, 1997, NEW ENGL J MED, V337, P441, DOI 10.1056/NEJM199708143370701
SIEGEL RL, 2020, CA Cancer J Clin, V70, P7, DOI [DOI 10.3322/CAAC.20107, 10.3322/caac.21590, DOI 10.3322/CAAC.21590]
SIMOONS FJ, 1969, AM J DIG DIS, V14, P819, DOI 10.1007/BF02233204
Stearns S.C., 2008, Evolution in health and disease
Stearns S. C., 2001, MECH LIFE HIST EVOLU, P365
Stearns SC, 2010, P NATL ACAD SCI USA, V107, P1691, DOI 10.1073/pnas.0914475107
Stewart EJ, 2005, PLOS BIOL, V3, P295, DOI 10.1371/journal.pbio.0030045
STRACHAN DP, 1989, BRIT MED J, V299, P1259, DOI 10.1136/bmj.299.6710.1259
Strassmann BI, 1999, EVOLUTION IN HEALTH AND DISEASE, P91
Sulem P, 2007, NAT GENET, V39, P1443, DOI 10.1038/ng.2007.13
Summers RW, 2005, GUT, V54, P87, DOI 10.1136/gut.2004.041749
Swynghedauw B, 2004, ACTA CHIR BELG, V104, P132, DOI 10.1080/00015458.2004.11679525
Takahashi M, 2008, J INFECT DIS, V197, pS41, DOI 10.1086/522132
Tanaka MM, 2011, EVOLUTION, V65, P1376, DOI 10.1111/j.1558-5646.2011.01223.x
Tinbergen N., 1963, Zeitschrift fuer Tierpsychologie, V20, P410
Tishkoff SA, 2007, NAT GENET, V39, P31, DOI 10.1038/ng1946
Tosh PK, 2008, MAYO CLIN PROC, V83, P77, DOI 10.4065/83.1.77
Trevathan WR., 2008, Evolutionary Medicine and Health: New Perspectives

   Vasilakis N, 2011, NAT REV MICROBIOL, V9, P532, DOI
10.1038/nrmicro2595
   Vignuzzi M, 2008, NAT MED, V14, P154, DOI 10.1038/nm1726
   Villarreal-Molina MT, 2008, DIABETES, V57, P509, DOI 10.2337/db07-0484
   Wallace DC, 2005, ANNU REV GENET, V39, P359, DOI
10.1146/annurev.genet.39.110304.095751
   Weismann August., 1882, UEBER DAUER LEBENS
   Williams G. C., 1966, P307
   WILLIAMS GC, 1957, EVOLUTION, V11, P398, DOI 10.1111/j.1558-
5646.1957.tb02911.x
   WILLIAMS GC, 1991, Q REV BIOL, V66, P1, DOI 10.1086/417048
   Wolfe ND, 2007, NATURE, V447, P279, DOI 10.1038/nature05775
   Zampieri F, 2009, Q REV BIOL, V84, P333, DOI 10.1086/648122
NR 146
TC 23
Z9 28
U1 1
U2 120
PU OXFORD UNIV PRESS
PI OXFORD
PA GREAT CLARENDON ST, OXFORD OX2 6DP, ENGLAND
SN 0014-3820
EI 1558-5646
J9 EVOLUTION
JI Evolution
PD JUN
PY 2012
VL 66
IS 6
BP 1991
EP 2006
DI 10.1111/j.1558-5646.2011.01552.x
PG 16
WC Ecology; Evolutionary Biology; Genetics & Heredity
WE Science Citation Index Expanded (SCI-EXPANDED); Social Science
Citation Index (SSCI)
SC Environmental Sciences & Ecology; Evolutionary Biology; Genetics &
   Heredity
GA 951YR
UT WOS:000304757200026
PM 22671563
OA Green Published, Bronze, Green Submitted
DA 2025-05-07
ER

PT J
AU Boudesseul, J
   Vieira, L
   Bègue, L
AF Boudesseul, Jordane
   Vieira, Luc
   Begue, Laurent
TI An Evolutionary Approach to Binge Drinking Impression Formation: A
   Cross-Cultural Comparison Between France and Peru

SO EVOLUTIONARY PSYCHOLOGY
LA English
DT Article
DE evolution; health; alcohol; prevention; mating
ID ALCOHOL-CONSUMPTION; TEMPORAL CONTEXT; RISK; METAANALYSIS; PREVENTION; SELECTION; BEHAVIOR; SIGNALS; ADULTS
AB Evolutionary medicine proposes studying alcohol use and abuse through the lens of modern evolutionary theory. This study (uw/) follows this approach and uses an evolutionary framework to predict how young adults (18-35 years old) form impression of a binge drinker. We predicted that displaying sexual dysfunctions (short-term risk) in a binge drinking video would negatively influence attitudes and expectations of a target when compared to cognitive (short-term risk) or long-term deficits. In the following studies, we use a Zahavian framework to understand and influence impression formation of a male binge drinker among women (intersexual selection) and men (intrasexual competition) participants in a subsequent task. Via a randomized experimental online study in France (N = 177, M = 23.39 [4.91], 43.50% men) and a preregistered conceptual replication study in Peru (N = 176, M = 25.61 [4.76], 53.41% men), women exposed to a binge drinking video-describing sexual impotence after a binge drinking episode-tended to downgrade attractiveness evaluation of the binge drinker. However, male participants were not impacted by the different types of signals displayed in the videos. These results show that evolutionary theory could help us understand impression formation in binge drinking context and call for gender-specific health messages.
C1 [Boudesseul, Jordane] Univ Lima, Fac Psicol, IDIC, Ave Javier Prado Este 4600, Lima 33, Peru.
[Vieira, Luc] Univ Strasbourg, Lab Psychol Cognit, Strasbourg, France.
[Begue, Laurent] Univ Grenoble Alpes, Lab Interuniv Psychol, St Martin Dheres, Rhone Alpes, France.
C3 Universidad de Lima; Universites de Strasbourg Etablissements Associes;
Universite de Strasbourg; Communaute Universite Grenoble Alpes;
Universite Grenoble Alpes (UGA)
RP Boudesseul, J (autor correspondente), Univ Lima, Fac Psicol, IDIC, Ave Javier Prado Este 4600, Lima 33, Peru.
EM jmj.boudesseul@gmail.com
OI Boudesseul, Jordane/0000-0001-7920-4812
FU Universite Grenoble Alpes
FX The author(s) disclosed receipt of the following financial support for the research, authorship, and/or publication of this article: This research was supported by grants from the Universite Grenoble Alpes and
the Instituto de Investigacion Cientifica.
CR Ajzen I., 1985, ACTION CONTROL COGNI, P11, DOI [10.1007/978-3-642-69746-32, DOI 10.1007/978-3-642-69746-3_2, DOI 10.1007/978-3-642-69746-32]
[Anonymous], 2015, EASY EMG E BOOK GUID
Babor T., 2011, ALCOHOL NO ORDINARY
Bernstein MH, 2016, ALCOHOL ALCOHOLISM, V51, P106, DOI 10.1093/alcalc/agv091
Brauer M, 2005, ANN PSYCHOL, V105, P273
Bush K, 1998, ARCH INTERN MED, V158, P1789, DOI 10.1001/archinte.158.16.1789

Chung T, 2018, ALCOHOL RES-CURR REV, V39, P5
Corrao G, 2004, PREV MED, V38, P613, DOI 10.1016/j.ypmed.2003.11.027
Courtney KE, 2009, PSYCHOL BULL, V135, P142, DOI 10.1037/a0014414
D'Amico EJ, 2002, ADDICTION, V97, P563, DOI 10.1046/j.1360-0443.2002.00115.x
Durkin K., 1999, College Student Journal, V33, P450
Freimuth VS, 1997, HEALTH EDUC BEHAV, V24, P555, DOI 10.1177/109019819702400504
Gallopel-Morvan K., 2016, FRANCES EVIN LAW CON
Geary DC, 2017, Q REV BIOL, V92, P361, DOI 10.1086/694934
Gerend MA, 2008, J EXP SOC PSYCHOL, V44, P1167, DOI 10.1016/j.jesp.2008.02.007
Gibson C, 2004, J CRIM JUST, V32, P411, DOI 10.1016/j.jcrimjus.2004.06.003
GRAFEN A, 1990, J THEOR BIOL, V144, P517, DOI 10.1016/S0022-5193(05)80088-8
Guérin S, 2013, EUR J PUBLIC HEALTH, V23, P588, DOI 10.1093/eurpub/ckt015
Higgins ET, 2008, HANDBOOK OF MOTIVATION AND COGNITION ACROSS CULTURES, P161, DOI 10.1016/B978-0-12-373694-9.00008-8
Hill EM, 2002, ADDICTION, V97, P401, DOI 10.1046/j.1360-0443.2002.00020.x
Hutter RRC, 2015, BRIT J HEALTH PSYCH, V20, P498, DOI 10.1111/bjhp.12111
Jessor R., 1991, ADOLESCENCE PROBLEM
Johnston KL, 2003, PSYCHOL HEALTH, V18, P63, DOI 10.1080/0887044021000037835
Kahneman D., 2013, Handbook of the Fundamentals of Financial Decision Making: Part I, P269, DOI [10.1142/9789814417358_0016, DOI 10.1142/9789814417358_0016]
Kanny D, 2018, AM J PREV MED, V54, P486, DOI 10.1016/j.amepre.2017.12.021
Koop P., 2015, Le cout social des drogues en France
Kurman J., 2011, ONLINE READINGS PSYC, V5, P3, DOI 10.9707/2307-0919.1109
Lockwood P, 2005, PERS SOC PSYCHOL B, V31, P379, DOI 10.1177/0146167204271598
Loyau A, 2005, ETHOLOGY, V111, P810, DOI 10.1111/j.1439-0310.2005.01091.x
Mateos C, 1997, ANIM BEHAV, V53, P471, DOI 10.1006/anbe.1996.0297
McClelland GH, 2014, HANDBOOK OF RESEARCH METHODS IN SOCIAL AND PERSONALITY PSYCHOLOGY, SECOND EDITION, P608
National Institute on Alcohol Abuse and Alcoholism, 2004, NIAAA Council approves definition of binge drinking
Nguyen-Thanh V., 2019, B EPIDEMIOLOGIQUE HE, V5-6, P87
Norman P, 2007, ADDICT BEHAV, V32, P1753, DOI 10.1016/j.addbeh.2006.12.009
Oei TPS, 2004, ADDICT BEHAV, V29, P159, DOI 10.1016/S0306-4603(03)00076-5
Penaranda L., 2014, ESTUDIO PREVENCION C
Spach M, 2016, SANTE PUBLIQUE, V28, P461, DOI 10.3917/spub.164.0461
Sylwester K, 2011, SEX ROLES, V64, P695, DOI 10.1007/s11199-010-9790-6
Tannenbaum MB, 2015, PSYCHOL BULL, V141, P1178, DOI 10.1037/a0039729

Tymula A, 2012, P NATL ACAD SCI USA, V109, P17135, DOI 10.1073/pnas.1207144109
Van 't Veer AE, 2016, J EXP SOC PSYCHOL, V67, P2, DOI 10.1016/j.jesp.2016.03.004
Vincke E, 2017, EVOL PSYCHOL-US, V15, DOI 10.1177/1474704917707073
Vincke E, 2016, EVOL PSYCHOL-US, V14, DOI 10.1177/1474704916631615
White BX, 2018, HEALTH PSYCHOL REV, V12, P147, DOI 10.1080/17437199.2018.1448292
Yom-Tov E, 2018, NPJ DIGIT MED, V1, DOI 10.1038/s41746-018-0031-7
ZAHAVI A, 1975, J THEOR BIOL, V53, P205, DOI 10.1016/0022-5193(75)90111-3
Zahavi A., 1997, The Handicap Principle: A Missing Piece of Darwins Puzzle, DOI [DOI 10.1017/CBO9781107415324.004, 10.1017/CBO9781107415324.004]
Zwaan RA, 2018, BEHAV BRAIN SCI, V41, DOI 10.1017/S0140525X17001972
NR 48
TC 0
Z9 0
U1 1
U2 2
PU SAGE PUBLICATIONS INC
PI THOUSAND OAKS
PA 2455 TELLER RD, THOUSAND OAKS, CA 91320 USA
SN 1474-7049
J9 EVOL PSYCHOL-US
JI Evol. Psychol.
PD JAN
PY 2020
VL 18
IS 1
AR 1474704919897602
DI 10.1177/1474704919897602
PG 10
WC Psychology, Experimental
WE Social Science Citation Index (SSCI)
SC Psychology
GA KR2TS
UT WOS:000517473200001
PM 32101034
OA Green Submitted, Green Published, gold
DA 2025-05-07
ER

PT J
AU Tissot, S
Guimard, L
Meliani, J
Boutry, J
Dujon, AM
Capp, JP
Tökölyi, J
Biro, PA
Beckmann, C
Fontenille, L

Do Khoa, N
Hamede, R
Roche, B
Ujvari, B
Nedelcu, AM
Thomas, F
AF Tissot, Sophie
Guimard, Lena
Meliani, Jordan
Boutry, Justine
Dujon, Antoine M.
Capp, Jean-Pascal
Tokolyi, Jacint
Biro, Peter A.
Beckmann, Christa
Fontenille, Laura
Do Khoa, Nam
Hamede, Rodrigo
Roche, Benjamin
Ujvari, Beata
Nedelcu, Aurora M.
Thomas, Frederic
TI The impact of food availability on tumorigenesis is evolutionarily conserved
SO SCIENTIFIC REPORTS
LA English
DT Article
ID CALORIC RESTRICTION; CHLAMYDOMONAS-REINHARDTII; STRESS RESISTANCE; DIETARY PATTERNS; LIFE-STYLE; TRADE-OFFS; CANCER; SURVIVAL; HYDRA; SIZE
AB The inability to control cell proliferation results in the formation of tumors in many multicellular lineages. Nonetheless, little is known about the extent of conservation of the biological traits and ecological factors that promote or inhibit tumorigenesis across the metazoan tree. Particularly, changes in food availability have been linked to increased cancer incidence in humans, as an outcome of evolutionary mismatch. Here, we apply evolutionary oncology principles to test whether food availability, regardless of the multicellular lineage considered, has an impact on tumorigenesis. We used two phylogenetically unrelated model systems, the cnidarian Hydra oligactis and the fish Danio rerio, to investigate the impact of resource availability on tumor occurrence and progression. Individuals from healthy and tumor-prone lines were placed on four diets that differed in feeding frequency and quantity. For both models, frequent overfeeding favored tumor emergence, while lean diets appeared more protective. In terms of tumor progression, high food availability promoted it, whereas low resources controlled it, but without having a curative effect. We discuss our results in light of current ideas about the possible conservation of basic processes governing cancer in metazoans (including ancestral life history trade-offs at the cell level) and in the framework of evolutionary medicine.
C1 [Tissot, Sophie; Guimard, Lena; Meliani, Jordan; Boutry, Justine; Roche, Benjamin; Thomas, Frederic] Univ Montpellier, CREEC MIVEGEC, CNRS, IRD, Montpellier, France.

[Dujon, Antoine M.; Biro, Peter A.; Beckmann, Christa; Ujvari, Beata] Deakin Univ, Sch Life & Environm Sci, Waurn Ponds, Vic, Australia.
[Capp, Jean-Pascal] Univ Toulouse, Toulouse Biotechnol Inst, INSA, CNRS,INRAE, Toulouse, France.
[Tokolyi, Jacint] Univ Debrecen, Dept Evolutionary Zool, MTA DE Momentum Ecol Evolut & Dev Biol Res Grp, H-4032 Debrecen, Hungary.
[Beckmann, Christa] Western Sydney Univ, Sch Sci, Hawkesbury Campus,Locked Bag 1797, Richmond, NSW 2753, Australia.
[Beckmann, Christa] Western Sydney Univ, Hawkesbury Inst Environm, Penrith, NSW, Australia.
[Fontenille, Laura; Do Khoa, Nam] AZELEAD, 377 Rue Prof Blayac, F-34080 Montpellier, France.
[Hamede, Rodrigo] Univ Tasmania, Sch Nat Sci, Hobart, Tas, Australia.
[Roche, Benjamin] Univ Nacl Autonoma Mexico, Fac Med Vet & Zootecnia, Dept Etol Fauna Silvestre & Anim Lab, Mexico City, Mexico.
[Nedelcu, Aurora M.] Univ New Brunswick, Dept Biol, Fredericton, NB, Canada.
C3 Universite de Montpellier; Institut de Recherche pour le Developpement (IRD); Centre National de la Recherche Scientifique (CNRS); Deakin University; Centre National de la Recherche Scientifique (CNRS); Universite Federale Toulouse Midi-Pyrenees (ComUE); Universite de Toulouse; Institut National des Sciences Appliquees de Toulouse; INRAE;
University of Debrecen; Western Sydney University; Western Sydney University; University of Tasmania; Universidad Nacional Autonoma de Mexico; University of New Brunswick
RP Tissot, S (autor correspondente), Univ Montpellier, CREEC MIVEGEC, CNRS, IRD, Montpellier, France.
EM sophie.tissot@ird.fr
RI Roche, Benjamin/AFL-0683-2022; Tökölyi, Jácint/HTL-9021-2023; Boutry, Justine/JPK-2624-2023; Biro, Peter/D-4327-2009; Ujvari, Beata/L-7541-2019; Beckmann, Christa/JVY-7142-2024; Dujon, Antoine/Q-7075-2019; Ujvari, Beata/K-8722-2012; Beckmann, Christa/G-2754-2012
OI Ujvari, Beata/0000-0003-2391-2988; Beckmann, Christa/0000-0002-7904-7228
FU We are grateful to Marina Mione for providing us with the pictures of zebrafish.
FX We are grateful to Marina Mione for providing us with the pictures of zebrafish.
CR Aktipis CA, 2013, NAT REV CANCER, V13, P883, DOI 10.1038/nrc3606
Aktipis CA, 2013, EVOL APPL, V6, P144, DOI 10.1111/eva.12034
Alidadi M, 2021, SEMIN CANCER BIOL, V73, P30, DOI 10.1016/j.semcancer.2020.09.010
Anderson RM, 2010, TRENDS ENDOCRIN MET, V21, P134, DOI 10.1016/j.tem.2009.11.005
Biro PA, 2022, EVOL MED PUBLIC HLTH, V10, P170, DOI 10.1093/emph/eoac012
Biro PA, 2020, TRENDS CANCER, V6, P829, DOI 10.1016/j.trecan.2020.06.001
Bolar Kartikeya, 2019, CRAN
Boroughs LK, 2015, NAT CELL BIOL, V17, P351, DOI 10.1038/ncb3124
Boutry J., Spontaneously occurring germline tumors in different wild-derived strains of hydra

Boutry J, 2023, SCI REP-UK, V13, DOI 10.1038/s41598-023-34656-0
Boutry J, 2022, ISCIENCE, V25, DOI 10.1016/j.isci.2022.105034
Boutry J, 2022, SCI TOTAL ENVIRON, V803, DOI 10.1016/j.scitotenv.2021.149923
Brown KF, 2018, BRIT J CANCER, V118, P1130, DOI 10.1038/s41416-018-0029-6
Brownell K.D., 2004, Food Fight: The Inside Story of the Food Industry, America's Obesity Crisis, and What We Can Do About It
Brunstrom JM, 2018, PHYSIOL BEHAV, V193, P261, DOI 10.1016/j.physbeh.2018.02.038
Brunstrom JM, 2018, APPETITE, V120, P589, DOI 10.1016/j.appet.2017.10.015
Buono R, 2018, TRENDS ENDOCRIN MET, V29, P271, DOI 10.1016/j.tem.2018.01.008
Buschemeyer WC, 2010, PROSTATE, V70, P1037, DOI 10.1002/pros.21136
Cameron-Pack ME, 2022, BIOL LETTERS, V18, DOI 10.1098/rsbl.2022.0059
Carrera-Bastos P, 2011, RES REP CLIN CARDIOL, V2, P15, DOI 10.2147/RRCC.S16919
Castro-Espin C, 2022, NUTRIENTS, V14, DOI 10.3390/nu14020348
Chang CW, 2005, PLANT PHYSIOL, V138, P319, DOI 10.1104/pp.105.059550
Chen H, 2015, NAT COMMUN, V6, DOI 10.1038/ncomms7367
Christensen Rune Haubo Bojesen, 2024, CRAN
Davies JP, 1996, EMBO J, V15, P2150, DOI 10.1002/j.1460-2075.1996.tb00568.x
Deserti MI, 2017, ANIM BIOL, V67, P287, DOI 10.1163/15707563-00002537
Domazet-Loso T, 2014, NAT COMMUN, V5, DOI 10.1038/ncomms5222
Drewnowski A, 2005, AM J CLIN NUTR, V82, p265S, DOI 10.1093/ajcn/82.1.265S
ERASO P, 1985, FEBS LETT, V191, P51, DOI 10.1016/0014-5793(85)80991-1
Finkel SE, 2006, NAT REV MICROBIOL, V4, P113, DOI 10.1038/nrmicro1340
Foster KR, 2004, NATURE, V431, P693, DOI 10.1038/nature02894
Giraudeau M, 2018, NAT ECOL EVOL, V2, P1065, DOI 10.1038/s41559-018-0558-7
Glade M J, 1999, Nutrition, V15, P523
Gluckman P., 2006, MISMATCH WHY OUR WOR
Gluckman P. D., 2016, Principles of Evolutionary Medicine, V2nd, DOI [10.1093/acprof:oso/9780199663927.001.0001, DOI 10.1093/ACPROF:OSO/9780199663927.001.0001]
Grigura V, 2018, BIOL OPEN, V7, DOI 10.1242/bio.030726
Grosso G, 2017, NUTR REV, V75, P405, DOI 10.1093/nutrit/nux012
GURKEWITZ S, 1980, INT J INVER REP DEV, V2, P199
Hecker B., 1976, Coelenterate Ecology and Behavior, P175, DOI [10.1007/978-1-4757-9724-4_19, DOI 10.1007/978-1-4757-9724-4_19]
Heidemann C, 2008, CIRCULATION, V118, P230, DOI 10.1161/CIRCULATIONAHA.108.771881
Herman CP, 2015, PHYSIOL BEHAV, V144, P129, DOI 10.1016/j.physbeh.2015.03.025
Hursting SD, 2013, CANCER METAB, V1, DOI 10.1186/2049-3002-1-10
KAGAWA Y, 1978, PREV MED, V7, P205, DOI 10.1016/0091-7435(78)90246-3
Lee C, 2012, SCI TRANSL MED, V4, DOI 10.1126/scitranslmed.3003293
Lv MM, 2014, PLOS ONE, V9, DOI 10.1371/journal.pone.0115147
maximeherve, Maxime HERVE - R et statistiques
Mayne ST, 2016, NAT REV CLIN ONCOL, V13, P504, DOI 10.1038/nrclinonc.2016.24

Mortzfeld BM, 2019, NAT COMMUN, V10, DOI 10.1038/s41467-019-11136-6
Moseley JL, 2006, EUKARYOT CELL, V5, P26, DOI 10.1128/EC.5.1.26-44.2006
Murphy R, 2020, METABOLITES, V10, DOI 10.3390/metabo10040123
Nedelcu AM, 2006, MOL BIOL EVOL, V23, P1460, DOI 10.1093/molbev/msl016
Norin T, 2017, BIOL LETTERS, V13, DOI 10.1098/rsbl.2017.0298
OTTO JJ, 1977, J CELL SCI, V28, P117
Papadimitriou N, 2021, NAT COMMUN, V12, DOI 10.1038/s41467-021-24861-8
Parkin DM, 2011, BRIT J CANCER, V105, pS77, DOI 10.1038/bjc.2011.489
Pedersen Thomas Lin, 2024, CRAN
Pesavento PA, 2018, NAT REV CANCER, V18, P646, DOI 10.1038/s41568-018-0045-0
Pomatto-Watson LCD, 2021, NAT COMMUN, V12, DOI 10.1038/s41467-021-26431-4
R Core Team, 2020, R: a language and environment for statistical computing. R Foundation for Statistical Computing v.4.3.0
Raffaghello L, 2008, P NATL ACAD SCI USA, V105, P8215, DOI 10.1073/pnas.0708100105
Rathje K, 2020, PLOS PATHOG, V16, DOI 10.1371/journal.ppat.1008375
Saggere RMS, 2022, P ROY SOC B-BIOL SCI, V289, DOI 10.1098/rspb.2021.2669
Santoriello C, 2010, PLOS ONE, V5, DOI 10.1371/journal.pone.0015170
Simone BA, 2013, FUTURE ONCOL, V9, P959, DOI [10.2217/fon.13.31, 10.2217/FON.13.31]
Singh JE, 2020, BMJ OPEN, V10, DOI 10.1136/bmjopen-2019-034730
STEARNS SC, 1989, FUNCT ECOL, V3, P259, DOI 10.2307/2389364
Strassmann JE, 2011, P NATL ACAD SCI USA, V108, P10855, DOI 10.1073/pnas.1102451108
Therneau Terry M, 2024, CRAN
Váchová L, 2005, J CELL BIOL, V169, P711, DOI 10.1083/jcb.200410064
Veettil SK, 2021, JAMA NETW OPEN, V4, DOI 10.1001/jamanetworkopen.2020.37341
Vidoni C, 2021, J CANCER PREV, V26, P224, DOI 10.15430/JCP.2021.26.4.224
West SA, 2007, ANNU REV ECOL EVOL S, V38, P53, DOI 10.1146/annurev.ecolsys.38.091206.095740
WILSON RB, 1988, MOL CELL BIOL, V8, P505, DOI 10.1128/MCB.8.1.505
Zuur Alain F., 2009, P1
NR 74
TC 6
Z9 6
U1 3
U2 8
PU NATURE PORTFOLIO
PI BERLIN
PA HEIDELBERGER PLATZ 3, BERLIN, 14197, GERMANY
SN 2045-2322
J9 SCI REP-UK
JI Sci Rep
PD NOV 14
PY 2023
VL 13
IS 1
AR 19825

DI 10.1038/s41598-023-46896-1
PG 13
WC Multidisciplinary Sciences
WE Science Citation Index Expanded (SCI-EXPANDED)
SC Science & Technology - Other Topics
GA Y4DU0
UT WOS:001104793000027
PM 37963956
OA Green Submitted, Green Published, gold
DA 2025-05-07
ER

PT J
AU Wells, JCK
Nesse, RM
Sear, R
Johnstone, RA
Stearns, SC
AF Wells, Jonathan C. K.
Nesse, Randolph M.
Sear, Rebecca
Johnstone, Rufus A.
Stearns, Stephen C.
TI Evolutionary public health: introducing the concept
SO LANCET
LA English
DT Article
ID HUMAN BEHAVIORAL ECOLOGY; CHILD UNDERNUTRITION; BIRTH-WEIGHT; ADULT HEALTH; REPRODUCTION; MORTALITY; RISK; MALNUTRITION; ASSOCIATION; FERTILITY
AB The emerging discipline of evolutionary medicine is breaking new ground in understanding why people become ill. However, the value of evolutionary analyses of human physiology and behaviour is only beginning to be recognised in the field of public health. Core principles come from life history theory, which analyses the allocation of finite amounts of energy between four competing functions-maintenance, growth, reproduction, and defence. A central tenet of evolutionary theory is that organisms are selected to allocate energy and time to maximise reproductive success, rather than health or longevity. Ecological interactions that influence mortality risk, nutrient availability, and pathogen burden shape energy allocation strategies throughout the life course, thereby affecting diverse health outcomes. Public health interventions could improve their own effectiveness by incorporating an evolutionary perspective. In particular, evolutionary approaches offer new opportunities to address the complex challenges of global health, in which populations are differentially exposed to the metabolic consequences of poverty, high fertility, infectious diseases, and rapid changes in nutrition and lifestyle. The effect of specific interventions is predicted to depend on broader factors shaping life expectancy. Among the important tools in this approach are mathematical models, which can explore probable benefits and limitations of interventions in silico, before their implementation in human populations.
C1 [Wells, Jonathan C. K.] UCL Great Ormond St Inst Child Hlth, Childhood Nutr Res Ctr, London WC1N 1EH, England.

[Nesse, Randolph M.] Arizona State Univ, Ctr Evolut & Med, Phoenix, AZ USA.
[Sear, Rebecca] London Sch Hyg & Trop Med, London, England.
[Johnstone, Rufus A.] Univ Cambridge, Dept Zool, Cambridge, England.
[Stearns, Stephen C.] Yale Univ, Dept Ecol & Evolutionary Biol, New Haven, CT USA.
C3 University of London; University College London; Arizona State University; Arizona State University-Downtown Phoenix; University of London; London School of Hygiene & Tropical Medicine; University of Cambridge; Yale University
RP Wells, JCK (autor correspondente), UCL Great Ormond St Inst Child Hlth, Childhood Nutr Res Ctr, London WC1N 1EH, England.
EM jonathan.wells@ucl.ac.uk
RI Stearns, Stephen/F-4099-2012; Wells, Jonathan/A-4604-2009; Sear, Rebecca/K-9616-2019
OI Sear, Rebecca/0000-0002-4315-0223
FU Leverhulme Trust
FX Preparation of this manuscript was facilitated by support of the Arizona
State University Centre for Evolution and Medicine provided for an authors' meeting. The model of growth was produced through a grant to RAJ and JCKW from the Leverhulme Trust.
CR Alvergne A, 2014, P ROY SOC B-BIOL SCI, V281, DOI 10.1098/rspb.2014.1733
[Anonymous], 1859, ORIGINS SPECIES MEAN
[Anonymous], DEATH HOPE AND SEX
[Anonymous], LIFE COURSE APPROACH
[Anonymous], 2011, Global status report on noncommunicable diseases 2010
[Anonymous], DIPLOMATS DIABETES D
[Anonymous], HUM NAT
[Anonymous], MATERN CHILD NUTR
[Anonymous], 2014, EVOL MED PUBLIC HLTH, DOI DOI 10.1093/EMPH/EOU005
[Anonymous], MOD MED
[Anonymous], PRINCIPLES PUBLIC HL
Ardia DR, 2011, FUNCT ECOL, V25, P61, DOI 10.1111/j.1365-2435.2010.01759.x
Arias CA, 2009, NEW ENGL J MED, V360, P439, DOI 10.1056/NEJMp0804651
Barbujani G, 1997, P NATL ACAD SCI USA, V94, P4516, DOI 10.1073/pnas.94.9.4516
Barker D.J. P., 1992, FETAL INFANT ORIGINS
Bartz S, 2014, J CLIN ENDOCR METAB, V99, P2128, DOI 10.1210/jc.2013-4018
Benhariz M, 1997, CLIN NUTR, V16, P251, DOI 10.1016/S0261-5614(97)80037-4
Black RE, 2008, LANCET, V371, P243, DOI 10.1016/S0140-6736(13)60937-X
Caudell MA, 2012, HUM BIOL, V84, P101, DOI 10.3378/027.084.0201
Ceesay SM, 1997, BMJ-BRIT MED J, V315, P786, DOI 10.1136/bmj.315.7111.786
Cohen JC, 2004, SCIENCE, V305, P869, DOI 10.1126/science.1099870
Colleran H, 2014, P ROY SOC B-BIOL SCI, V281, DOI 10.1098/rspb.2013.2732
Collins FS, 2001, CANCER, V91, P221, DOI 10.1002/1097-0142(20010101)91:1+<221::AID-CNCR8>3.0.CO;2-9

Crimmins EM, 2006, P NATL ACAD SCI USA, V103, P498, DOI 10.1073/pnas.0501470103
Darmasseelane K, 2014, PLOS ONE, V9, DOI 10.1371/journal.pone.0087896
Dennett Daniel., 1995, Darwin?s Dangerous Idea - Evolution and the Meanings of Life
Desborough JP, 2000, BRIT J ANAESTH, V85, P109, DOI 10.1093/bja/85.1.109
DOBZHANSKY T, 1973, AM BIOL TEACH, V35, P125, DOI 10.2307/4444260
Dobzhansky Th., 1983, Human Culture, a Moment in Evolution
Doyal L., 1979, POLITICAL EC HLTH
FINCH CE, 1995, Q REV BIOL, V70, P1, DOI 10.1086/418864
Frazer KA, 2009, NAT REV GENET, V10, P241, DOI 10.1038/nrg2554
Gale EAM, 2013, LANCET, V381, P1956, DOI 10.1016/S0140-6736(12)62207-7
Gandon S, 2001, NATURE, V414, P751, DOI 10.1038/414751a
Gibson MA, 2006, PLOS MED, V3, P476, DOI 10.1371/journal.pmed.0030087
Gluckman PD, 2008, NEW ENGL J MED, V359, P61, DOI 10.1056/NEJMra0708473
Godfrey KM, 2010, TRENDS ENDOCRIN MET, V21, P199, DOI 10.1016/j.tem.2009.12.008
GOLDGAR DE, 1994, J NATL CANCER I, V86, P1600, DOI 10.1093/jnci/86.21.1600
Guyatt HL, 2004, CLIN MICROBIOL REV, V17, P760, DOI 10.1128/CMR.17.4.760-769.2004
HAIG D, 1993, Q REV BIOL, V68, P495, DOI 10.1086/418300
HAMILTON WD, 1964, J THEOR BIOL, V7, P1, DOI [10.1016/0022-5193(64)90038-4, 10.1016/0022-5193(64)90039-6]
Hillhouse JJ, 2002, J BEHAV MED, V25, P395, DOI 10.1023/A:1015870516460
Hirschhorn JN, 2009, NEW ENGL J MED, V360, P1699, DOI 10.1056/NEJMp0808934
Hrdy S.B., 2009, Mothers and others: The evolutionary origins of mutual understanding
Jablonka E., 2005, Evolution in four dimensions: Genetic, epigenetic, behavioral, and symbolic variation in the history of life
Jasienska G, 2017, LANCET, V390, P510, DOI [10.1016/S0140-6736(17)30573-1, 10.1016/s0140-6736(17)30573-1]
Jones KE, 2008, NATURE, V451, P990, DOI 10.1038/nature06536
Jorgensen C, 2007, SCIENCE, V318, P1247, DOI 10.1126/science.1148089
Keinan A, 2012, SCIENCE, V336, P740, DOI 10.1126/science.1217283
Kleinridders A, 2014, DIABETES, V63, P2232, DOI 10.2337/db14-0568
Krieger N, 2004, EPIDEMIOL REV, V26, P92, DOI 10.1093/epirev/mxh009
Laland KN, 2006, EVOL ANTHROPOL, V15, P95, DOI 10.1002/evan.20093
LASKER GW, 1969, SCIENCE, V166, P1480, DOI 10.1126/science.166.3912.1480
Lawson DW, 2008, INT J EPIDEMIOL, V37, P1408, DOI 10.1093/ije/dyn116
Lawson DW, 2012, P ROY SOC B-BIOL SCI, V279, P4755, DOI 10.1098/rspb.2012.1635
Le Bourg E, 2007, AGEING RES REV, V6, P141, DOI 10.1016/j.arr.2007.04.002
Lee G, 2012, INT J EPIDEMIOL, V41, P531, DOI 10.1093/ije/dyr190
Li J., 2006, Drug Discovery Today: Disease Mechanisms, V3, P387, DOI DOI 10.1016/J.DDMEC.2006.09.003
Lochmiller RL, 2000, OIKOS, V88, P87, DOI 10.1034/j.1600-0706.2000.880110.x

Lustig RH, 2006, NAT CLIN PRACT ENDOC, V2, P447, DOI 10.1038/ncpendmet0220
Marmot M, 2005, LANCET, V365, P1099, DOI [10.1016/S0140-6736(05)74234-3, 10.1016/S0140-6736(12)61228-8]
McGregor G, 2015, MOL ENDOCRINOL, V29, P3, DOI 10.1210/me.2014-1328
MOORE T, 1991, TRENDS GENET, V7, P45, DOI 10.1016/0168-9525(91)90230-N
Murk W, 2011, PEDIATRICS, V127, P1125, DOI 10.1542/peds.2010-2092
Nesse R.M., 1994, WHY WE GET SICK NEW
Nesse RM, 2010, P NATL ACAD SCI USA, V107, P1800, DOI 10.1073/pnas.0906224106
Nettle D, 2013, BEHAV ECOL, V24, P1031, DOI 10.1093/beheco/ars222
.Office for National Statistics, 2014, STAT B IN HLTH LIF E
Omenn GS, 2010, P NATL ACAD SCI USA, V107, P1702, DOI 10.1073/pnas.0906198106
Ono C, 2013, PHARMACOGENOMICS, V14, P1745, DOI 10.2217/pgs.13.171
Pampel FC, 2010, ANNU REV SOCIOL, V36, P349, DOI 10.1146/annurev.soc.012809.102529
Panter-Brick C, 2001, BRIT J NUTR, V85, P125, DOI 10.1079/BJN2000225
Penn DJ, 2007, P NATL ACAD SCI USA, V104, P553, DOI 10.1073/pnas.0609301103
Pepper GV, 2014, HUM NATURE-INT BIOS, V25, P378, DOI 10.1007/s12110-014-9204-5
Roff Derek A., 1992
Rook G, 2017, LANCET, V390, P521, DOI 10.1016/S0140-6736(17)30566-4
Schneider JE, 2004, PHYSIOL BEHAV, V81, P289, DOI 10.1016/j.physbeh.2004.02.007
SEAQUIST ER, 1989, NEW ENGL J MED, V320, P1161, DOI 10.1056/NEJM198905043201801
Sermon K, 2004, LANCET, V363, P1633, DOI 10.1016/S0140-6736(04)16209-0
SMITH CC, 1974, AM NAT, V108, P499, DOI 10.1086/282929
Stearns SC, 2000, P NATL ACAD SCI USA, V97, P3309, DOI 10.1073/pnas.060289597
Stearns SC., 2016, Evolutionary Medicine
Stearns SC, 2010, NAT REV GENET, V11, P611, DOI 10.1038/nrg2831
Thavagnanam S, 2008, CLIN EXP ALLERGY, V38, P629, DOI 10.1111/j.1365-2222.2007.02780.x
Trasande L, 2013, INT J OBESITY, V37, P16, DOI 10.1038/ijo.2012.132
TRIVERS RL, 1974, AM ZOOL, V14, P249
VAN NOORDWIJK AJ, 1986, AM NAT, V128, P137, DOI 10.1086/284547
Victora CG, 2008, LANCET, V371, P340, DOI 10.1016/S0140-6736(07)61692-4
WADE N, 1974, SCIENCE, V186, P1093, DOI 10.1126/science.186.4169.1093
Weatherall DJ, 2010, BLOOD, V115, P4331, DOI 10.1182/blood-2010-01-251348
Wells JCK, 2016, METABOLIC GHETTO: AN EVOLUTIONARY PERSPECTIVE ON NUTRITION, POWER RELATIONS AND CHRONIC DISEASE
Wells JCK, 2003, Q REV BIOL, V78, P169, DOI 10.1086/374952
Wells JCK, 2007, YEARB PHYS ANTHROPOL, V50, P191, DOI 10.1002/ajpa.20735
Wells JCK, 2016, EVOL MED PUBLIC HLTH, P133, DOI 10.1093/emph/eow011
Wells JCK, 2012, CURR ANTHROPOL, V53, pS466, DOI 10.1086/667606
Whitehead RD, 2014, HEALTH PSYCHOL, V33, P99, DOI 10.1037/a0032322
Wilson E.O., 1998, Consilience: The Unity of Knowledge, V1st ed.
Winterhalder B, 2000, EVOL ANTHROPOL, V9, P51

   Wise RA, 1996, ANNU REV NEUROSCI, V19, P319, DOI
10.1146/annurev.ne.19.030196.001535
NR 99
TC 135
Z9 141
U1 1
U2 63
PU ELSEVIER SCIENCE INC
PI NEW YORK
PA STE 800, 230 PARK AVE, NEW YORK, NY 10169 USA
SN 0140-6736
EI 1474-547X
J9 LANCET
JI Lancet
PD JUL 29
PY 2017
VL 390
IS 10093
BP 500
EP 509
DI 10.1016/S0140-6736(17)30572-X
PG 10
WC Medicine, General & Internal
WE Science Citation Index Expanded (SCI-EXPANDED)
SC General & Internal Medicine
GA FB9MO
UT WOS:000406463400031
PM 28792412
OA Green Submitted, Green Accepted
DA 2025-05-07
ER

PT J
AU Echaubard, P
   Sripa, B
   Mallory, FF
   Wilcox, BA
AF Echaubard, Pierre
   Sripa, Banchob
   Mallory, Frank F.
   Wilcox, Bruce A.
TI The role of evolutionary biology in research and control of liver
flukes
   in Southeast Asia
SO INFECTION GENETICS AND EVOLUTION
LA English
DT Review
DE Opisthorchis; Epidemiology; Transmission; Infectious disease;
   Host-parasite; Evolution
ID OPISTHORCHIS-VIVERRINI INFECTION; BITHYNIA-SIAMENSIS-GONIOMPHALOS; 1ST
   INTERMEDIATE HOSTS; KHON-KAEN PROVINCE; SCHISTOSOMA-MANSONI; HELMINTH
   INFECTION; SENSU-LATO; GENE FLOW; HEPATOBILIARY ABNORMALITIES;
   CLINICAL-MANIFESTATION

AB Stimulated largely by the availability of new technology, biomedical research at the molecular-level and chemical-based control approaches arguably dominate the field of infectious diseases. Along with this, the proximate view of disease etiology predominates to the exclusion of the ultimate, evolutionary biology-based, causation perspective. Yet, historically and up to today, research in evolutionary biology has provided much of the foundation for understanding the mechanisms underlying disease transmission dynamics, virulence, and the design of effective integrated control strategies.

Here we review the state of knowledge regarding the biology of Asian liver Fluke-host relationship, parasitology, phylodynamics, drug-based interventions and liver Fluke-related cancer etiology from an evolutionary biology perspective. We consider how evolutionary principles, mechanisms and research methods could help refine our understanding of clinical disease associated with infection by Liver Flukes as well as their transmission dynamics.

We identify a series of questions for an evolutionary biology research agenda for the liver Fluke that should contribute to an increased understanding of liver Fluke-associated diseases. Finally, we describe an integrative evolutionary medicine approach to liver Fluke prevention and control highlighting the need to better contextualize interventions within a broader human health and sustainable development framework. (C) 2016 Elsevier B.V. All rights reserved.
C1 [Echaubard, Pierre; Sripa, Banchob] Khon Kaen Univ, Fac Med, WHO Collaborating Ctr Res & Control Opisthorchias, Trop Dis Res Lab,Dept Expt Pathol, Khon Kaen 40002, Thailand.

[Echaubard, Pierre; Mallory, Frank F.] Laurentian Univ, Dept Biol, Sudbury, ON P3E 2C6, Canada.

[Echaubard, Pierre; Wilcox, Bruce A.] Mahidol Univ, Fac Publ Hlth, Global Hlth Asia, Bangkok, Thailand.

[Sripa, Banchob] Khon Kaen Univ, Dept Parasitol, Liver Fluke & Cholangiocarcinoma Res Ctr, Khon Kaen 40002, Thailand.

[Wilcox, Bruce A.] Tufts Univ, Cummings Sch Vet Med, Medford, MA 02155 USA.

C3 Khon Kaen University; Laurentian University; Mahidol University; Khon Kaen University; Tufts University

RP Echaubard, P (autor correspondente), Khon Kaen Univ, Fac Med, WHO Collaborating Ctr Res & Control Opisthorchias, Trop Dis Res Lab,Dept Expt Pathol, Khon Kaen 40002, Thailand.; Echaubard, P (autor correspondente), Laurentian Univ, Dept Biol, Sudbury, ON P3E 2C6, Canada.; Echaubard, P (autor correspondente), Mahidol Univ, Fac Publ Hlth, Global Hlth Asia, Bangkok, Thailand.

EM pn_echaubard@laurentian.ca

RI Echaubard, Pierre/H-3500-2019

OI Sripa, Banchob/0000-0001-8899-5919
FU Higher Education Research Promotion and National Research University Project of Thailand; Office of the Higher Education Commission, through the Health Cluster (SHeP-GMS); Khon Kaen University, from the Thailand Research Fund (TRF) [RTA 5680006]; National Institute of Allergy and Infectious Diseases (NIAID), NIH [P50AI098639]; Department of Biology, Laurentian University, Sudbury, Ontario, Canada

FX We would like to thank John F. Smith, Carsten Richter, Radhakrishnan Muthukumar, Kanin Salao and Isabelle Jala and two anonymous reviewers

for comments and suggestions during the preparation of the manuscript,
as well as the organizers and participants of the Molecular
Epidemiology
and Evolutionary Genetic of Infectious Diseases (MEEGID) conference
held
in Bangkok, Thailand in December 2014 for fruitful discussions. We
gratefully acknowledge support from the Higher Education Research
Promotion and National Research University Project of Thailand, Office
of the Higher Education Commission, through the Health Cluster
(SHeP-GMS), Khon Kaen University, from the Thailand Research Fund
(TRF)
grant number RTA 5680006 and from the National Institute of Allergy
and
Infectious Diseases (NIAID), NIH, grant number P50AI098639. PE is a
post-doctoral research scholar of Khon Kaen University. BS is a TRF
Senior Research Scholar. Other financial support was provided by the
Department of Biology, Laurentian University, Sudbury, Ontario,
Canada.
The content is solely the responsibility of the authors and does not
necessarily represent the official views of the TRF, NIAID or the NIH.
CR Aktipis CA, 2013, EVOL APPL, V6, P144, DOI 10.1111/eva.12034
Allen JE, 2011, PLOS PATHOG, V7, DOI 10.1371/journal.ppat.1002003
Alonso D, 2006, AM J TROP MED HYG, V74, P342, DOI
10.4269/ajtmh.2006.74.342
ANDERSON RM, 1982, PARASITOLOGY, V85, P411, DOI
10.1017/S0031182000055360
Andrews RH, 2008, TRENDS PARASITOL, V24, P497, DOI
10.1016/j.pt.2008.08.011
[Anonymous], 2007, J EXP MED, V204, P237, DOI 10.1084/jem.2042fta
[Anonymous], 1994, IARC Monogr Eval Carcinog Risks Hum, V61, P1
[Anonymous], 1976, Plagues and Peoples
ANTIA R, 1994, AM NAT, V144, P457, DOI 10.1086/285686
Antonovics J, 2007, PLOS BIOL, V5, P137, DOI
10.1371/journal.pbio.0050030
Artis D, 2008, MUCOSAL IMMUNOL, V1, P252, DOI 10.1038/mi.2008.21
Auld SKJR, 2015, HEREDITY, V114, P125, DOI 10.1038/hdy.2014.84
Aunpromma S, 2012, PARASITOL INT, V61, P60, DOI
10.1016/j.parint.2011.08.004
Bircher J, 2014, J PUBLIC HEALTH POL, V35, P363, DOI
10.1057/jphp.2014.19
Blasco-Costa I, 2013, PARASITOLOGY, V140, P1316, DOI
10.1017/S0031182013000784
Bonesso-Sabadini PIP, 2002, MEM I OSWALDO CRUZ, V97, P381, DOI
10.1590/S0074-02762002000300019
Bourke CD, 2013, J INFECT DIS, V208, P159, DOI 10.1093/infdis/jis524
Brinkworth JessicaF., 2013, Primates, Pathogens, and Evolution
BULL JJ, 1994, EVOLUTION, V48, P1423, DOI 10.1111/j.1558-
5646.1994.tb02185.x
Castrillo A, 2004, ANNU REV CELL DEV BI, V20, P455, DOI
10.1146/annurev.cellbio.20.012103.134432
CERAMI A, 1994, PARASITOL TODAY, V10, P404, DOI 10.1016/0169-
4758(94)90234-8
Cho I, 2012, NAT REV GENET, V13, P260, DOI 10.1038/nrg3182

Davies CM, 2002, PARASITOLOGY, V124, P31, DOI 10.1017/S0031182001008873
Dieckmann U., 2002, Pursuit Virulence Manage
Doenhoff MJ, 2002, T ROY SOC TROP MED H, V96, P465, DOI 10.1016/S0035-9203(02)90405-0
Dybdahl MF, 2008, AM NAT, V172, P772, DOI 10.1086/592866
Echaubard P, 2014, EVOL APPL, V7, P723, DOI 10.1111/eva.12169
Elkins DB, 1996, AM J TROP MED HYG, V55, P295, DOI 10.4269/ajtmh.1996.55.295
Ewald P W, 1994, Evolution of Infectious Diseases
Ewald P.W., 1988, OXFORD SURVEYS EVOLU
EWALD PW, 1983, ANNU REV ECOL SYST, V14, P465, DOI 10.1146/annurev.es.14.110183.002341
Fallon PG, 1997, EXP PARASITOL, V86, P29, DOI 10.1006/expr.1997.4149
Fellous S, 2009, TRENDS PARASITOL, V25, P62, DOI 10.1016/j.pt.2008.11.010
Frank SA, 1996, Q REV BIOL, V71, P37, DOI 10.1086/419267
Fumagalli M, 2009, J EXP MED, V206, P1395, DOI 10.1084/jem.20082779
Galvani AP, 2003, TRENDS ECOL EVOL, V18, P132, DOI 10.1016/S0169-5347(02)00050-2
Gandon S, 1996, P ROY SOC B-BIOL SCI, V263, P1003, DOI 10.1098/rspb.1996.0148
Gandon S, 2002, J EVOLUTION BIOL, V15, P451, DOI 10.1046/j.1420-9101.2002.00402.x
Gandon S, 2001, NATURE, V414, P751, DOI 10.1038/414751a
Gandon S, 2009, AM NAT, V173, P212, DOI 10.1086/593706
Glaser R, 2005, NAT REV IMMUNOL, V5, P243, DOI 10.1038/nri1571
Grenfell BT, 2004, SCIENCE, V303, P327, DOI 10.1126/science.1090727
Grundy-Warr C, 2012, PARASITOL INT, V61, P65, DOI 10.1016/j.parint.2011.06.015
Gryseels B, 2001, TROP MED INT HEALTH, V6, P864, DOI 10.1046/j.1365-3156.2001.00811.x
HARINASUTA C, 1984, ARZNEIMITTEL-FORSCH, V34-2, P1164
Harling Melanie, 2009, J Occup Med Toxicol, V4, P4, DOI 10.1186/1745-6673-4-4
HASWELLELKINS MR, 1991, PARASITOLOGY, V103, P97, DOI 10.1017/S0031182000059333
Horwitz P, 2005, INT J PARASITOL, V35, P725, DOI 10.1016/j.ijpara.2005.03.002
Hussaarts L, 2011, J ALLERGY CLIN IMMUN, V128, P733, DOI 10.1016/j.jaci.2011.05.012
IARC, 2011, INF LIV LUK OP VIV O
Institute of Medicine, 2006, END WAR MET CHANG AG
IOM (Institue of Medicine), 2001, NEW HOR HLTH INT APP
Ismail M, 1999, AM J TROP MED HYG, V60, P932, DOI 10.4269/ajtmh.1999.60.932
Jittimanee J, 2012, ACTA TROP, V122, P189, DOI 10.1016/j.actatropica.2012.01.010
Jongsuksuntigul P, 2003, ACTA TROP, V88, P229, DOI 10.1016/j.actatropica.2003.01.002
Kaewkes S, 2003, ACTA TROP, V88, P177, DOI 10.1016/j.actatropica.2003.05.001
Kaewkes S, 2012, PARASITOL INT, V61, P46, DOI 10.1016/j.parint.2011.08.015

Kaewpitoon N, 2008, WORLD J GASTROENTERO, V14, P666, DOI 10.3748/wjg.14.666
Kamsa-Ard S, 2013, ASIAN PAC J CANCER P, V14, P7011, DOI 10.7314/APJCP.2013.14.11.7011
Karvonen A, 2004, PARASITOLOGY, V129, P87, DOI 10.1017/S0031182004005281
Keiser J, 2013, J HELMINTHOL, V87, P252, DOI 10.1017/S0022149X12000387
Keiser J, 2005, EMERG INFECT DIS, V11, P1507, DOI 10.3201/eid1110.050614
Keiser J, 2009, CLIN MICROBIOL REV, V22, P466, DOI 10.1128/CMR.00012-09
Kiatsopit N, 2014, PARASITE VECTOR, V7, DOI 10.1186/s13071-014-0551-1
Kiatsopit N, 2013, INFECT GENET EVOL, V14, P313, DOI 10.1016/j.meegid.2012.12.026
Koelle K, 2006, SCIENCE, V314, P1898, DOI 10.1126/science.1132745
KRIVENKO V V, 1981, Gigiena i Sanitariya, P62
KRIVENKO V V, 1979, Gigiena i Sanitariya, P80
Laine AL, 2014, P ROY SOC B-BIOL SCI, V281, DOI 10.1098/rspb.2014.0522
Lambrechts L, 2005, MALARIA J, V4, DOI 10.1186/1475-2875-4-3
Laoprom N, 2010, INFECT GENET EVOL, V10, P146, DOI 10.1016/j.meegid.2009.11.005
Laoprom N, 2009, J PARASITOL, V95, P1307, DOI 10.1645/GE-2116.1
Lederberg J, 2000, SCIENCE, V288, P287, DOI 10.1126/science.288.5464.287
Lenormand T, 2002, TRENDS ECOL EVOL, V17, P183, DOI 10.1016/S0169-5347(02)02497-7
Levin BR, 1996, EMERG INFECT DIS, V2, P93, DOI 10.3201/eid0202.960203
Li T, 2010, TRENDS PARASITOL, V26, P264, DOI 10.1016/j.pt.2010.02.007
Liang You-Sheng, 2011, Zhongguo Xue Xi Chong Bing Fang Zhi Za Zhi, V23, P605
Little TJ, 2010, PLOS PATHOG, V6, DOI 10.1371/journal.ppat.1001006
Lively CM, 2000, NATURE, V405, P679, DOI 10.1038/35015069
Lochhead P, 2015, MODERN PATHOL, V28, P14, DOI 10.1038/modpathol.2014.81
Ludwig-Portugall I, 2012, FRONT IMMUNOL, V3, DOI 10.3389/fimmu.2012.00008
Lun ZR, 2005, LANCET INFECT DIS, V5, P31, DOI 10.1016/S1473-3099(04)01252-6
Lustigman S, 2012, PLOS NEGLECT TROP D, V6, DOI 10.1371/journal.pntd.0001445
MacNeish R.S., 1992, ORIGINS AGR SETTLED
Magen E, 2005, MED HYPOTHESES, V64, P904, DOI 10.1016/j.mehy.2004.09.028
Magen E, 2013, INT J PARASITOL, V43, P819, DOI 10.1016/j.ijpara.2013.04.008
Mairiang E, 2003, ACTA TROP, V88, P221, DOI 10.1016/j.actatropica.2003.03.001
Mairiang E, 2012, PARASITOL INT, V61, P208, DOI 10.1016/j.parint.2011.07.009
Maizels RM, 2012, CURR OPIN IMMUNOL, V24, P459, DOI 10.1016/j.coi.2012.06.003
Manel S, 2005, TRENDS ECOL EVOL, V20, P136, DOI 10.1016/S0169-5347(03)00008-9

Manel S, 2013, TRENDS ECOL EVOL, V28, P614, DOI 10.1016/j.tree.2013.05.012
MAY RM, 1994, J THEOR BIOL, V170, P95, DOI 10.1006/jtbi.1994.1171
Mayr E., 1982, of the evolution of scientific thought and medical
McSorley HJ, 2012, CLIN MICROBIOL REV, V25, P585, DOI 10.1128/CMR.05040-11
Medley G. F., 1994, Parasitic and infectious diseases: epidemiology and ecology., P141
Medzhitov R, 2012, SCIENCE, V335, P936, DOI 10.1126/science.1214935
Michalakis Yannis, 2009, P19
Michalakis Y, 2009, NATURE, V462, P298, DOI 10.1038/462298a
Mishra PK, 2014, MUCOSAL IMMUNOL, V7, P753, DOI 10.1038/mi.2014.23
Mitta G, 2012, DEV COMP IMMUNOL, V37, P1, DOI 10.1016/j.dci.2011.09.002
Miwa Masanao, 2014, World J Gastrointest Pathophysiol, V5, P570, DOI 10.4291/wjgp.v5.i4.570
Montresor A., 1998, Guidelines for the evaluation of soil-transmitted helminthiasis and schistosomiasis at community level-A guide for managers of control programmes
Montresor A, 2008, PLOS NEGLECT TROP D, V2, DOI 10.1371/journal.pntd.0000278
Moolasart J, 2012, SE ASIAN J TROP MED, V43, P795
Moreno-Smith M, 2010, FUTURE ONCOL, V6, P1863, DOI 10.2217/FON.10.142
Nair SS, 2011, HEPATOLOGY, V54, P1388, DOI 10.1002/hep.24518
Naylor RL, 2000, NATURE, V405, P1017, DOI 10.1038/35016500
Nesse RM, 2008, EVOL APPL, V1, P28, DOI 10.1111/j.1752-4571.2007.00006.x
Nesse RM, 2013, TRENDS ECOL EVOL, V28, P681, DOI 10.1016/j.tree.2013.10.008
Palumbi SR, 2001, SCIENCE, V293, P1786, DOI 10.1126/science.293.5536.1786
Panuwet P, 2012, ENVIRON SCI POLICY, V17, P72, DOI 10.1016/j.envsci.2011.12.005
Parker GA, 2003, NATURE, V425, P480, DOI 10.1038/nature02012
Paterson S, 2007, P ROY SOC B-BIOL SCI, V274, P1467, DOI 10.1098/rspb.2006.0433
Petney T, 2012, PARASITOL INT, V61, P38, DOI 10.1016/j.parint.2011.07.019
Petney TN, 2013, INT J PARASITOL, V43, P1031, DOI 10.1016/j.ijpara.2013.07.007
Pinlaor S, 2004, CARCINOGENESIS, V25, P1535, DOI 10.1093/carcin/bgh157
Pinlaor S, 2008, AM J TROP MED HYG, V78, P564, DOI 10.4269/ajtmh.2008.78.564
Porco TC, 2005, J THEOR BIOL, V233, P91, DOI 10.1016/j.jtbi.2004.09.009
Poulin R., 2011, Evolutionary Ecology of Parasites, DOI DOI 10.1515/9781400840809
Poulin R., 1998, EVOL ECOL PARASITES
Prayong P, 2014, ASIAN PAC J CANCER P, V15, P5443, DOI 10.7314/APJCP.2014.15.13.5443
Prugnolle F, 2005, EVOLUTION, V59, P296, DOI 10.1111/j.0014-3820.2005.tb00990.x
Rainboth W. J., 1991, Cyprinid Fishes: Systematics, Biology and Exploitation, P156

Read AF, 2001, SCIENCE, V292, P1099, DOI 10.1126/science.1059410
Read AF, 1999, EVOLUTION IN HEALTH AND DISEASE, P205
Read AF, 2011, P NATL ACAD SCI USA, V108, P10871, DOI 10.1073/pnas.1100299108
Reiche EMV, 2004, LANCET ONCOL, V5, P617, DOI 10.1016/S1470-2045(04)01597-9
RENAUD F, 1991, J THEOR BIOL, V152, P319, DOI 10.1016/S0022-5193(05)80197-3
Restif O, 2009, INFECT GENET EVOL, V9, P108, DOI 10.1016/j.meegid.2008.09.007
Rigg J, 2006, WORLD DEV, V34, P180, DOI 10.1016/j.worlddev.2005.07.015
Rim HJ, 2003, PARASITOL RES, V91, P267, DOI 10.1007/s00436-003-0963-x
Roff Derek A., 1992
Saengsawang P, 2013, ASIAN PAC J CANCER P, V14, P2963, DOI 10.7314/APJCP.2013.14.5.2963
Saijuntha W, 2009, PARASITOLOGY, V136, P1057, DOI 10.1017/S0031182009006441
Saijuntha W, 2007, INT J PARASITOL, V37, P695, DOI 10.1016/j.ijpara.2006.12.008
San José B, 2000, ALCOHOL ALCOHOLISM, V35, P307, DOI 10.1093/alcalc/35.3.307
Sapolsky R.M., 2004, Why zebras don't get ulcers: The acclaimed guide to stress, stressrelated diseases, and coping
Sayasone S, 2009, T ROY SOC TROP MED H, V103, P247, DOI 10.1016/j.trstmh.2008.10.011
SchmidHempel P, 2011, EVOLUTIONARY PARASITOLOGY: THE INTEGRATED STUDY OF INFECTIONS, IMMUNOLOGY, ECOLOGY, AND GENETICS, P1
Seeman TE, 1996, PSYCHOSOM MED, V58, P459, DOI 10.1097/00006842-199609000-00008
Seto EYW, 2011, AM J TROP MED HYG, V85, P74, DOI 10.4269/ajtmh.2011.10-0542
Shor DB, 2013, CLIN REV ALLERG IMMU, V45, P211, DOI 10.1007/s12016-012-8352-9
SITHITHAWORN P, 1991, PARASITOLOGY, V102, P277, DOI 10.1017/S0031182000062594
Sithithaworn P, 2014, J HEPATO-BIL-PAN SCI, V21, P301, DOI 10.1002/jhbp.62
Sithithaworn P, 2012, PARASITOL INT, V61, P10, DOI 10.1016/j.parint.2011.08.014
Sithithaworn P, 2012, PARASITOL INT, V61, P32, DOI 10.1016/j.parint.2011.07.020
Skorping A, 1998, ECOL LETT, V1, P10, DOI 10.1046/j.1461-0248.1998.0007d.x
SLATKIN M, 1987, SCIENCE, V236, P787, DOI 10.1126/science.3576198
Smith HV, 1998, PARASITOLOGY, V117, pS113, DOI 10.1017/S0031182099004898
Smout MJ, 2011, MOL BIOSYST, V7, P1367, DOI 10.1039/c0mb00295j
Soares MP, 2014, TRENDS IMMUNOL, V35, P483, DOI 10.1016/j.it.2014.08.001
Soukhathammavong PA, 2015, ACTA TROP, V141, P303, DOI 10.1016/j.actatropica.2014.09.014
Sriamporn S, 2004, TROP MED INT HEALTH, V9, P588, DOI 10.1111/j.1365-3156.2004.01234.x

Sripa B, 2000, PARASITE IMMUNOL, V22, P139, DOI 10.1046/j.1365-3024.2000.00286.x
Sripa B, 2003, ACTA TROP, V88, P209, DOI 10.1016/j.actatropica.2003.08.002
Sripa B, 2015, ACTA TROP, V141, P361, DOI 10.1016/j.actatropica.2014.07.017
Sripa B, 2012, TRENDS PARASITOL, V28, P395, DOI 10.1016/j.pt.2012.07.006
Sripa B, 2011, ACTA TROP, V120, pS158, DOI 10.1016/j.actatropica.2010.07.006
Sripa B, 2010, ADV PARASIT, V72, P305, DOI 10.1016/S0065-308X(10)72011-X
Sripa B, 2009, HEPATOLOGY, V50, P1273, DOI 10.1002/hep.23134
Stearns S.C., 1992, pi
Sung KC, 2011, J CLIN ENDOCR METAB, V96, P1093, DOI 10.1210/jc.2010-2190
TAMM ME, 1993, BRIT J MED PSYCHOL, V66, P213, DOI 10.1111/j.2044-8341.1993.tb01745.x
Tawatsin A., 2015, Med Res Arch, V3, DOI DOI 10.18103/MRA.V0I3.176
Taylor MD, 2012, TRENDS IMMUNOL, V33, P181, DOI 10.1016/j.it.2012.01.001
Tesana S, 2012, PARASITOL INT, V61, P52, DOI 10.1016/j.parint.2011.08.003
Thaenkham U, 2011, J PARASITOL, V97, P1152, DOI 10.1645/GE-2764.1
Thaenkham U, 2010, PARASITOL INT, V59, P242, DOI 10.1016/j.parint.2010.02.009
Thompson JN, 2005, INTERSPEC INTERACT
Thrall PH, 2002, PLANT PATHOL, V51, P169, DOI 10.1046/j.1365-3059.2002.00683.x
Tinbergen N., 1963, Zeitschrift fuer Tierpsychologie, V20, P410
Townsend AR, 2003, FRONT ECOL ENVIRON, V1, P240, DOI 10.2307/3868011
TULJAPURKAR S, 1991, SCIENCE, V254, P591, DOI 10.1126/science.254.5031.591
van den Biggelaar AHJ, 2002, J INFECT DIS, V186, P1474, DOI 10.1086/344352
VATANASAPT V, 1990, LANCET, V335, P116, DOI 10.1016/0140-6736(90)90591-R
Waller P. J., 1985, Resistance in nematodes to anthelmintic drugs, P1
Wang W, 2012, PARASITOL RES, V111, P1871, DOI 10.1007/s00436-012-3151-z
Wang W, 2010, PARASITOLOGY, V137, P1905, DOI 10.1017/S0031182010001204
Ward MH, 2005, ENVIRON HEALTH PERSP, V113, P1607, DOI 10.1289/ehp.8043
WARREN KS, 1981, ANNU REV PUBL HEALTH, V2, P101, DOI 10.1146/annurev.pu.02.050181.000533
Webster JP, 2008, EVOL APPL, V1, P66, DOI 10.1111/j.1752-4571.2007.00012.x
Webster JP, 1998, EVOLUTION, V52, P1627, DOI [10.2307/2411336, 10.1111/j.1558-5646.1998.tb02243.x]
Wenz-Mücke A, 2013, PARASITOLOGY, V140, P709, DOI 10.1017/S003118201200203X
WHO, 2014, GLOBAL STATUS REPORT ON VIOLENCE PREVENTION 2014, P1
Wilcox Bruce A., 2005, EcoHealth, V2, P244, DOI 10.1007/s10393-005-8961-3

Wilson MS, 2004, CLIN REV ALLERG IMMU, V26, P35, DOI 10.1385/CRIAI:26:1:35
Wiria AE, 2014, PLOS PATHOG, V10, DOI 10.1371/journal.ppat.1004140
Wiria AE, 2012, SEMIN IMMUNOPATHOL, V34, P889, DOI 10.1007/s00281-012-0358-0
Wolinska J, 2009, TRENDS PARASITOL, V25, P236, DOI 10.1016/j.pt.2009.02.004
WONGRATANACHEEWIN S, 1987, J HELMINTHOL, V61, P151, DOI 10.1017/S0022149X00009913
Woolhouse MEJ, 1999, NAT MED, V5, P1225, DOI 10.1038/15169
WYKOFF DE, 1965, J PARASITOL, V51, P207, DOI 10.2307/3276083
Yazdanbakhsh M, 2001, TRENDS IMMUNOL, V22, P372, DOI 10.1016/S1471-4906(01)01958-5
Young ND, 2014, NAT COMMUN, V5, DOI 10.1038/ncomms5378
Ziegler A.D., 2016, ECOHEALTH
NR 195
TC 21
Z9 22
U1 1
U2 44
PU ELSEVIER
PI AMSTERDAM
PA RADARWEG 29, 1043 NX AMSTERDAM, NETHERLANDS
SN 1567-1348
EI 1567-7257
J9 INFECT GENET EVOL
JI Infect. Genet. Evol.
PD SEP
PY 2016
VL 43
BP 381
EP 397
DI 10.1016/j.meegid.2016.05.019
PG 17
WC Infectious Diseases
WE Science Citation Index Expanded (SCI-EXPANDED); Social Science Citation Index (SSCI)
SC Infectious Diseases
GA DR1YP
UT WOS:000379701100052
PM 27197053
OA Green Accepted
DA 2025-05-07
ER

PT J
AU Dornburg, A
Yoder, JA
AF Dornburg, Alex
Yoder, Jeffrey A.
TI On the relationship between extant innate immune receptors and the evolutionary origins of jawed vertebrate adaptive immunity
SO IMMUNOGENETICS
LA English

DT Review
DE Bowfin and gar (Holostei); Immune system evolution; Joining domains; Origins of V(D)J recombination; Zebrafish
ID B-CELL-RECEPTOR; VARIABLE LYMPHOCYTE RECEPTORS; CONSTANT-REGION GENES; ANTIGEN RECEPTOR; IMMUNOGLOBULIN GENES; GENOMIC ORGANIZATION; HAGFISH LEUKOCYTES; CHANNEL CATFISH; SPOTTED GAR; VH-GENE
AB For over half a century, deciphering the origins of the genomic loci that form the jawed vertebrate adaptive immune response has been a major topic in comparative immunogenetics. Vertebrate adaptive immunity relies on an extensive and highly diverse repertoire of tandem arrays of variable (V), diversity (D), and joining (J) gene segments that recombine to produce different immunoglobulin (Ig) and T cell receptor (TCR) genes. The current consensus is that a recombination-activating gene (RAG)-like transposon invaded an exon of an ancient innate immune VJ-bearing receptor, giving rise to the extant diversity of Ig and TCR loci across jawed vertebrates. However, a model for the evolutionary relationships between extant non-recombining innate immune receptors and the V(D)J receptors of the jawed vertebrate adaptive immune system has only recently begun to come into focus. In this review, we provide an overview of non-recombining VJ genes, including CD8 beta, CD79b, natural cytotoxicity receptor 3 (NCR3/NKp30), putative remnants of an antigen receptor precursor (PRARPs), and the multigene family of signal-regulatory proteins (SIRPs), that play a wide range of roles in immune function. We then focus in detail on the VJ-containing novel immune-type receptors (NITRs) from ray-finned fishes, as recent work has indicated that these genes are at least 50 million years older than originally thought. We conclude by providing a conceptual model of the evolutionary origins and phylogenetic distribution of known VJ-containing innate immune receptors, highlighting opportunities for future comparative research that are empowered by this emerging evolutionary perspective.
C1 [Dornburg, Alex] Univ North Carolina Charlotte, Dept Bioinformat & Genom, Charlotte, NC 28223 USA.
[Yoder, Jeffrey A.] North Carolina State Univ, Dept Mol Biomed Sci, 1060 William Moore Dr, Raleigh, NC 27606 USA.
[Yoder, Jeffrey A.] North Carolina State Univ, Comparat Med Inst, Raleigh, NC 27606 USA.
[Yoder, Jeffrey A.] North Carolina State Univ, Ctr Human Hlth & Environm, Raleigh, NC 27606 USA.
C3 University of North Carolina; University of North Carolina Charlotte; North Carolina State University; North Carolina State University; North
Carolina State University
RP Dornburg, A (autor correspondente), Univ North Carolina Charlotte, Dept Bioinformat & Genom, Charlotte, NC 28223 USA.; Yoder, JA (autor correspondente), North Carolina State Univ, Dept Mol Biomed Sci, 1060 William Moore Dr, Raleigh, NC 27606 USA.; Yoder, JA (autor correspondente), North Carolina State Univ, Comparat Med Inst, Raleigh, NC 27606 USA.; Yoder, JA (autor correspondente), North Carolina State Univ, Ctr Human Hlth & Environm, Raleigh, NC 27606 USA.
EM adornbur@uncc.edu; jeff_yoder@ncsu.edu
RI Yoder, Jeffrey/I-5329-2019; Dornburg, Alex/CAF-1206-2022
OI Yoder, Jeff/0000-0002-6083-1311
FU National Science Foundation [IOS-1755242, IOS-1755330]; Triangle Center

for Evolutionary Medicine (TriCEM)
FX This report was supported, in part, by grants from the National Science
Foundation (IOS-1755242 to AD and IOS-1755330 to JAY) and from the
Triangle Center for Evolutionary Medicine (TriCEM) to AD and JAY.
CR Agrawal A, 1998, NATURE, V394, P744, DOI 10.1038/29457
Alfaro ME, 2009, P NATL ACAD SCI USA, V106, P13410, DOI 10.1073/pnas.0811087106
Allcock RJN, 2003, EUR J IMMUNOL, V33, P567, DOI 10.1002/immu.200310033
ALLISON JP, 1982, J IMMUNOL, V129, P2293
Barclay AN, 2006, NAT REV IMMUNOL, V6, P457, DOI 10.1038/nri1859
Barrow AD, 2006, EUR J IMMUNOL, V36, P1646, DOI 10.1002/eji.200636195
Boehm T, 2018, ANNU REV IMMUNOL, V36, P19, DOI 10.1146/annurev-immunol-042617-053028
Braasch I, 2016, NAT GENET, V48, P427, DOI 10.1038/ng.3526
Brown EJ, 2001, TRENDS CELL BIOL, V11, P130, DOI 10.1016/S0962-8924(00)01906-1
Cannon JP, 2008, IMMUNITY, V29, P228, DOI 10.1016/j.immuni.2008.05.018
Castro R, 2011, FISH SHELLFISH IMMUN, V31, P644, DOI 10.1016/j.fsi.2010.08.016
Chen R, 2018, J IMMUNOL, V200, P2869, DOI 10.4049/jimmunol.1700906
CHIEN Y, 1984, NATURE, V312, P31, DOI 10.1038/312031a0
CHOTHIA C, 1987, J MOL BIOL, V196, P901, DOI 10.1016/0022-2836(87)90412-8
Cowman PF, 2017, BIOL REV, V92, P2112, DOI 10.1111/brv.12323
Danilova N, 2009, ANN NY ACAD SCI, V1168, P130, DOI 10.1111/j.1749-6632.2009.04881.x
Davis AM, 2012, J EVOLUTION BIOL, V25, P1163, DOI 10.1111/j.1420-9101.2012.02504.x
Desai S, 2008, BMC EVOL BIOL, V8, DOI 10.1186/1471-2148-8-177
Dong ML, 2005, FISH SHELLFISH IMMUN, V19, P165, DOI 10.1016/j.fsi.2004.11.001
Dooley H, 2006, DEV COMP IMMUNOL, V30, P43, DOI 10.1016/j.dci.2005.06.022
Dornburg A, 2021, ANNU REV ECOL EVOL S, V52, P427, DOI 10.1146/annurev-ecolsys-122120-122554
Dornburg A, 2021, IMMUNOGENETICS, V73, P479, DOI 10.1007/s00251-021-01225-6
Dornburg A, 2021, GENOME BIOL EVOL, V13, DOI 10.1093/gbe/evaa228
Dornburg A, 2017, NAT ECOL EVOL, V1, P1379, DOI 10.1038/s41559-017-0239-y
Dornburg A, 2015, EVOLUTION, V69, P146, DOI 10.1111/evo.12562
Eason DD, 2004, SEMIN IMMUNOL, V16, P215, DOI 10.1016/j.smim.2004.08.001
EDELMAN GM, 1969, ANNU REV BIOCHEM, V38, P415, DOI 10.1146/annurev.bi.38.070169.002215
Evenhuis J, 2007, IMMUNOGENETICS, V59, P661, DOI 10.1007/s00251-007-0230-x
Fellah JS, 2002, GENE, V288, P95, DOI 10.1016/S0378-1119(02)00437-7
Fernández R, 2020, NAT ECOL EVOL, V4, P524, DOI 10.1038/s41559-019-1069-x
Ferraresso S, 2009, IMMUNOGENETICS, V61, P773, DOI 10.1007/s00251-009-0398-3

Flajnik MF, 2018, NAT REV IMMUNOL, V18, P438, DOI 10.1038/s41577-018-0003-9
Flajnik MF, 2012, IMMUNOGENETICS, V64, P571, DOI 10.1007/s00251-012-0616-2
Fu YB, 2019, J IMMUNOL, V202, P1992, DOI 10.4049/jimmunol.1801305
Ghaffari SH, 1999, J IMMUNOL, V162, P1519
Guselnikov SV, 2003, IMMUNOGENETICS, V55, P472, DOI 10.1007/s00251-003-0599-0
Haruta C, 2006, IMMUNOGENETICS, V58, P216, DOI 10.1007/s00251-006-0098-1
HASKINS K, 1984, ANNU REV IMMUNOL, V2, P51, DOI 10.1146/annurev.iy.02.040184.000411
Hawke NA, 2001, P NATL ACAD SCI USA, V98, P13832, DOI 10.1073/pnas.231418598
HEDRICK SM, 1984, NATURE, V308, P153, DOI 10.1038/308153a0
HEDRICK SM, 1984, NATURE, V308, P149, DOI 10.1038/308149a0
Hirano M, 2013, NATURE, V501, P435, DOI 10.1038/nature12467
Holland SJ, 2014, P NATL ACAD SCI USA, V111, P14834, DOI 10.1073/pnas.1415655111
Hsu E., 2018, REFERENCE MODULE LIF, DOI [10.1016/B978-0-12-809633-8.20721-8, DOI 10.1016/B978-0-12-809633-8.20721-8]
Hsu E, 2006, J IMMUNOL, V177, P2452, DOI 10.4049/jimmunol.177.4.2452
Hsu E, 2009, ADV EXP MED BIOL, V650, P166
Inoue J, 2015, P NATL ACAD SCI USA, V112, P14918, DOI 10.1073/pnas.1507669112
Isakov N, 1997, IMMUNOL RES, V16, P85, DOI 10.1007/BF02786325
Katsukura H, 2001, IMMUNOGENETICS, V53, P770, DOI 10.1007/s00251-001-0385-9
Khalturin K, 2004, MOL IMMUNOL, V41, P1077, DOI 10.1016/j.molimm.2004.06.010
Kinlein A, 2021, IMMUNOGENETICS, V73, P333, DOI 10.1007/s00251-021-01209-6
KOKUBU F, 1987, P NATL ACAD SCI USA, V84, P5868, DOI 10.1073/pnas.84.16.5868
KOKUBU F, 1988, EMBO J, V7, P1979, DOI 10.1002/j.1460-2075.1988.tb03036.x
KOKUBU F, 1988, EMBO J, V7, P3413, DOI 10.1002/j.1460-2075.1988.tb03215.x
Kruse PH, 2014, IMMUNOL CELL BIOL, V92, P221, DOI 10.1038/icb.2013.98
Lanier LL, 1998, ANNU REV IMMUNOL, V16, P359, DOI 10.1146/annurev.immunol.16.1.359
Laun K, 2006, PLOS GENET, V2, P707, DOI 10.1371/journal.pgen.0020073
Lee SS, 2000, J EXP MED, V191, P1637, DOI 10.1084/jem.191.10.1637
Li JX, 2013, P NATL ACAD SCI USA, V110, P15013, DOI 10.1073/pnas.1314540110
Li RG, 2013, FISH SHELLFISH IMMUN, V34, P1404, DOI 10.1016/j.fsi.2013.02.015
Litman GW, 2010, NAT REV IMMUNOL, V10, P543, DOI 10.1038/nri2807
Litman GW, 2001, IMMUNOL REV, V181, P250, DOI 10.1034/j.1600-065X.2001.1810121.x
LITMAN GW, 1971, J IMMUNOL, V106, P747
LITMAN GW, 1971, J IMMUNOL, V107, P881
Mashoof Sara, 2016, Biology-Basel, V5, P45, DOI 10.3390/biology5040045
Matlung HL, 2017, IMMUNOL REV, V276, P145, DOI 10.1111/imr.12527

Meng FQ, 2014, DEV COMP IMMUNOL, V45, P269, DOI 10.1016/j.dci.2014.03.016
MEUER SC, 1983, J EXP MED, V157, P705, DOI 10.1084/jem.157.2.705
Montgomery BC, 2011, DEV COMP IMMUNOL, V35, P1223, DOI 10.1016/j.dci.2011.03.010
Moretta A, 2001, ANNU REV IMMUNOL, V19, P197, DOI 10.1146/annurev.immunol.19.1.197
Murphy K., 2016, Janeways Immunobiology, DOI DOI 10.1201/9781315533247
Nakatani M, 2011, BMC EVOL BIOL, V11, DOI 10.1186/1471-2148-11-177
Nakatani Y, 2021, NAT COMMUN, V12, DOI 10.1038/s41467-021-24573-z
Near TJ, 2013, P NATL ACAD SCI USA, V110, P12738, DOI 10.1073/pnas.1304661110
Near TJ, 2012, P NATL ACAD SCI USA, V109, P13698, DOI 10.1073/pnas.1206625109
Nei M, 2005, ANNU REV GENET, V39, P121, DOI 10.1146/annurev.genet.39.073003.112240
North B, 2011, J MOL BIOL, V406, P228, DOI 10.1016/j.jmb.2010.10.030
Ohta Y, 2006, J IMMUNOL, V176, P3674, DOI 10.4049/jimmunol.176.6.3674
Ohta Y, 2019, J IMMUNOL, V203, P1882, DOI 10.4049/jimmunol.1900597
Oronsky B, 2020, SEMIN ONCOL, V47, P117, DOI 10.1053/j.seminoncol.2020.05.009
Ott JA, 2021, IMMUNOGENETICS, V73, P17, DOI 10.1007/s00251-020-01183-5
Pancer Z, 2005, P NATL ACAD SCI USA, V102, P9224, DOI 10.1073/pnas.0503792102
Pancer Z, 2004, NATURE, V430, P174, DOI 10.1038/nature02740
Pancer Z, 2004, P NATL ACAD SCI USA, V101, P13273, DOI 10.1073/pnas.0405529101
PAPERMASTER BW, 1964, J EXP MED, V119, P105, DOI 10.1084/jem.119.1.105
Pende D, 1999, J EXP MED, V190, P1505, DOI 10.1084/jem.190.10.1505
Pessino A, 1998, J EXP MED, V188, P953, DOI 10.1084/jem.188.5.953
Pettinello R, 2014, BIOMOLECULES, V4, P1045, DOI 10.3390/biom4041045
Piyaviriyakul P, 2007, FISH SHELLFISH IMMUN, V22, P467, DOI 10.1016/j.fsi.2006.05.007
Prum RO, 2015, NATURE, V526, P569, DOI 10.1038/nature15697
RAST JP, 1994, IMMUNOGENETICS, V40, P83
RAST JP, 1995, IMMUNOGENETICS, V42, P204, DOI 10.1007/BF00191226
Rast JP, 1998, IMMUNOL REV, V166, P79, DOI 10.1111/j.1600-065X.1998.tb01254.x
REYNAUD CA, 1989, CELL, V59, P171, DOI 10.1016/0092-8674(89)90879-9
Sahoo M, 2008, DEV COMP IMMUNOL, V32, P1385, DOI 10.1016/j.dci.2008.05.008
SAKAGUCHI N, 1993, ADV IMMUNOL, V54, P337, DOI 10.1016/S0065-2776(08)60538-6
Sato A, 2003, IMMUNOGENETICS, V55, P423, DOI 10.1007/s00251-003-0589-2
Sayegh CE, 2000, IMMUNOL REV, V175, P187, DOI 10.1034/j.1600-065X.2000.017507.x
SIGEL MM, 1972, COMP BIOCHEM PHYSIOL, V42, P249, DOI 10.1016/0300-9629(72)90384-2
Simakov O, 2020, NAT ECOL EVOL, V4, P820, DOI 10.1038/s41559-020-1156-z
Siqueira AC, 2019, J BIOGEOGR, V46, P1611, DOI 10.1111/jbi.13631
Strong SJ, 1999, P NATL ACAD SCI USA, V96, P15080, DOI 10.1073/pnas.96.26.15080

Suetake H, 2007, FISH SHELLFISH IMMUN, V23, P1107, DOI 10.1016/j.fsi.2007.05.005
Sun Y, 2013, DEV COMP IMMUNOL, V39, P103, DOI 10.1016/j.dci.2012.02.008
Sutoh Y, 2021, IMMUNOGENETICS, V73, P5, DOI 10.1007/s00251-020-01182-6
Suzuki T, 2005, J IMMUNOL, V174, P2885, DOI 10.4049/jimmunol.174.5.2885
Suzuki T, 2004, DEV COMP IMMUNOL, V28, P993, DOI 10.1016/j.dci.2004.04.005
Taddese B, 2021, BIOINFORMATICS, V37, P2483, DOI 10.1093/bioinformatics/btab002
Tanaka S, 2020, ADV EXP MED BIOL, V1254, P23, DOI 10.1007/978-981-15-3532-1_2
Thompson AW, 2021, NAT GENET, V53, P1373, DOI 10.1038/s41588-021-00914-y
Timms JF, 1998, MOL CELL BIOL, V18, P3838, DOI 10.1128/MCB.18.7.3838
TONEGAWA S, 1983, NATURE, V302, P575, DOI 10.1038/302575a0
TREGASKES CA, 1995, J IMMUNOL, V154, P4485
TSENG J, 1994, IMMUNOL RES, V13, P299, DOI 10.1007/BF02935621
Tsuchiya Y, 2016, PROTEIN SCI, V25, P815, DOI 10.1002/pro.2874
Tukwasibwe S, 2020, CELL MOL IMMUNOL, V17, P799, DOI 10.1038/s41423-020-0482-z
Uhrberg M, 2002, IMMUNOGENETICS, V54, P221, DOI 10.1007/s00251-002-0463-7
van Beek EM, 2005, J IMMUNOL, V175, P7781, DOI 10.4049/jimmunol.175.12.7781
van den Berg TK, 2004, TRENDS IMMUNOL, V25, P11, DOI 10.1016/j.it.2003.11.006
Vernon-Wilson EF, 2000, EUR J IMMUNOL, V30, P2130
Viertlboeck BC, 2006, IMMUNOGENETICS, V58, P180, DOI 10.1007/s00251-006-0091-8
Viertlboeck BC, 2011, VET IMMUNOL IMMUNOP, V144, P1, DOI 10.1016/j.vetimm.2011.07.001
Vilches C, 2002, ANNU REV IMMUNOL, V20, P217, DOI 10.1146/annurev.immunol.20.092501.134942
Vitallé J, 2019, EUR J IMMUNOL, V49, P364, DOI 10.1002/eji.201847951
Wcisel DJ, 2020, BMC EVOL BIOL, V20, DOI 10.1186/s12862-020-01603-w
Wcisel DJ, 2017, J EXP ZOOL PART B, V328, P666, DOI 10.1002/jez.b.22738
Wcisel DJ, 2016, FISH SHELLFISH IMMUN, V53, P24, DOI 10.1016/j.fsi.2016.03.034
Wei S, 2007, IMMUNOGENETICS, V59, P813, DOI 10.1007/s00251-007-0250-6
Yamanoue Y, 2011, PLOS ONE, V6, DOI 10.1371/journal.pone.0017410
YANAGI Y, 1984, NATURE, V308, P145, DOI 10.1038/308145a0
Yoder JA, 2004, P NATL ACAD SCI USA, V101, P15706, DOI 10.1073/pnas.0405242101
Yoder JA, 2002, IMMUNOGENETICS, V54, P662, DOI 10.1007/s00251-002-0511-3
Yoder JA, 2001, P NATL ACAD SCI USA, V98, P6771, DOI 10.1073/pnas.121101598
Yoder JA, 2000, CURR TOP MICROBIOL, V248, P271
Yoder JA, 2008, IMMUNOGENETICS, V60, P257, DOI 10.1007/s00251-008-0285-3

   Yoder JA, 2011, IMMUNOGENETICS, V63, P123, DOI 10.1007/s00251-010-0506-4
   Yoder JA, 2009, DEV COMP IMMUNOL, V33, P135, DOI 10.1016/j.dci.2008.09.004
   Zhang YN, 2014, P NATL ACAD SCI USA, V111, P397, DOI 10.1073/pnas.1318843111
NR 138
TC 21
Z9 23
U1 1
U2 18
PU SPRINGER
PI NEW YORK
PA ONE NEW YORK PLAZA, SUITE 4600, NEW YORK, NY, UNITED STATES
SN 0093-7711
EI 1432-1211
J9 IMMUNOGENETICS
JI Immunogenetics
PD FEB
PY 2022
VL 74
IS 1
SI SI
BP 111
EP 128
DI 10.1007/s00251-021-01232-7
EA JAN 2022
PG 18
WC Genetics & Heredity; Immunology
WE Science Citation Index Expanded (SCI-EXPANDED)
SC Genetics & Heredity; Immunology
GA YS7IU
UT WOS:000737692200001
PM 34981186
DA 2025-05-07
ER

PT J
AU McCoy, DE
   Haig, D
   Kotler, J
AF McCoy, Dakota E.
   Haig, David
   Kotler, Jennifer
TI Egg donation and gestational surrogacy: Pregnancy is riskier with an
   unrelated embryo
SO EARLY HUMAN DEVELOPMENT
LA English
DT Article
DE Surrogacy; Gestational carrier; Egg donation; Egg donor; Ova donor;
   Oocyte donor; IVF; Immunology; Pregnancy; Microchimerism; Evolutionary
   medicine; Allogeneic pregnancies; Shared motherhood; Embryo donation
ID OOCYTE DONATION; KIDNEY EXCHANGE; OBSTETRIC COMPLICATIONS;
HYPERTENSIVE

DISORDERS; FETAL MICROCHIMERISM; OUTCOMES; PREECLAMPSIA; DONOR; HEALTH;
TRIPLET
AB Modern medicine has revolutionized family planning. Remarkably, women1 1 can carry to term embryos with whom they share no genetic connection, a feat made possible through egg donation and/or gestational surrogacy. Our reproductive systems evolved to accommodate embryos that are 50% related to the carrier, not 0% related. Here, we apply evolutionary theory to explain how and why pregnancy is riskier with an unrelated embryo. When a woman gestates an unrelated embryo, she is significantly more likely to develop preeclampsia and other diseases above and beyond the known risks associated with advanced maternal age, IVF, multiple gestation, and subfertility. Such "allogeneic pregnancies" are riskier even in fertile, healthy, commercial surrogates and when the egg is donated by a young, healthy donor. We propose that unrelated embryos present a special immune challenge to the gestational carrier, because they have fewer matching genes to the maternal body-therefore exacerbating symptoms of evolutionary maternal-fetal conflict. Indeed, maternal risks seem lower when the embryo is more related to the carrier, e.g., if a sister donates the egg. Finally, we discuss microchimerism in egg donation pregnancies, whereby wholly foreign cells pass from mother to embryo and vice-versa. We conclude with several medical proposals. First, egg donors and surrogates should be informed of the increased health risks they would face. In considerations of risk, these young, fertile women should not be compared to older, infertile women undergoing IVF; the proper comparison group is other young, fertile women. Second, contrary to some medical advice, perhaps genetically-related egg donors and surrogates should be preferred, all else equal. An immunological matching scheme, like what is used for organ transplants, could improve surrogate pregnancy outcomes. Third, more research is needed on microchimerism, sperm exposure, and the long-term impacts of allogeneic pregnancies on maternal and child health.
C1 [McCoy, Dakota E.] Univ Chicago, Dept Ecol & Evolut, Chicago, IL 60637 USA.
[McCoy, Dakota E.] Marine Biol Lab, Woods Hole, MA 02543 USA.
[Haig, David; Kotler, Jennifer] Harvard Univ, Dept Organism & Evolutionary Biol, Cambridge, MA 02136 USA.
C3 University of Chicago; Marine Biological Laboratory - Woods Hole; Harvard University
RP Kotler, J (autor correspondente), Harvard Univ, Dept Organism & Evolutionary Biol, Cambridge, MA 02136 USA.
EM dakota.e.mccoy@gmail.com; jlkotler@gmail.com
OI Kotler, Jennifer/0000-0001-6802-8980
FU Stanford Science Fellowship; NSF Postdoctoral Research Fellowships in Biology PRFB Program [2109465]
FX DEM is supported by the Stanford Science Fellowship and the NSF Postdoctoral Research Fellowships in Biology PRFB Program, grant 2109465.
CR Adams DH, 2016, J DEV ORIG HLTH DIS, V7, P257, DOI 10.1017/S2040174415007898
[Anonymous], 2024, National ART Summary | CDC Internet
[Anonymous], 2017, J Gynecol Reprod Med, V1, P1
Ashlagi I, 2021, MANAGE SCI, V67, P5455, DOI 10.1287/mnsc.2020.3954
Bartnik P, 2016, TWIN RES HUM GENET, V19, P377, DOI 10.1017/thg.2016.17

Boddy AM, 2015, BIOESSAYS, V37, P1106, DOI 10.1002/bies.201500059
Bodri D, 2008, REPROD BIOMED ONLINE, V17, P237, DOI 10.1016/S1472-6483(10)60200-3
Brandao P, 2022, J ASSIST REPROD GEN, V39, P2061, DOI 10.1007/s10815-022-02560-7
Brännström M, 2015, LANCET, V385, P607, DOI 10.1016/S0140-6736(14)61728-1
Campbell DM, 1999, HYPERTENS PREGNANCY, V18, P197, DOI 10.3109/10641959909016193
Daar J, 2018, FERTIL STERIL, V110, P1017, DOI 10.1016/j.fertnstert.2018.08.029
Dawe GS, 2007, CELL ADHES MIGR, V1, P19, DOI 10.4161/cam.1.1.4082
Duffy DA, 2005, FERTIL STERIL, V83, P749, DOI 10.1016/j.fertnstert.2004.08.023
Schwarze JE, 2018, J BRAS REPROD ASSIST, V22, P15, DOI 10.5935/1518-0557.20180001
Fiorentzis S., 2018, Multiple Pregnancy-New Challenges
Galliano D, 2015, FERTIL STERIL, V104, P1411, DOI 10.1016/j.fertnstert.2015.08.013
Gammill HS, 2013, HYPERTENSION, V62, P1062, DOI 10.1161/HYPERTENSIONAHA.113.01486
Gundogan F, 2010, FERTIL STERIL, V93, P397, DOI 10.1016/j.fertnstert.2008.12.144
Haig David, 2014, Chimerism, V5, P53
Hasson J, 2016, FERTIL STERIL, V106, P1696, DOI 10.1016/j.fertnstert.2016.08.042
Hernández ER, 2009, FERTIL STERIL, V91, P1358, DOI 10.1016/j.fertnstert.2008.03.077
Holzgreve W, 1998, OBSTET GYNECOL, V91, P669, DOI 10.1016/S0029-7844(98)00068-4
Hoskins R. E., 1995, Acta Geneticae Medicae et Gemellologiae, V44, P11
Iranifard E, 2024, REPROD HEALTH, V21, DOI 10.1186/s12978-024-01804-2
Jones BP, 2019, BJOG-INT J OBSTET GY, V126, P1310, DOI 10.1111/1471-0528.15863
Kallianidis AF, 2023, OBSTET GYNECOL, V141, P35, DOI 10.1097/AOG.0000000000005022
Kapfhamer J, 2016, FERTIL STERIL, V106, P270, DOI 10.1016/j.fertnstert.2016.04.028
Keukens A, 2022, HUM REPROD, V37, P586, DOI 10.1093/humrep/deab267
양광문, 2005, Obstetrics & Gynecology Science, V48, P112
Kinder JM, 2017, NAT REV IMMUNOL, V17, P483, DOI 10.1038/nri.2017.38
Klatsky PC, 2010, OBSTET GYNECOL, V116, P1387, DOI 10.1097/AOG.0b013e3181fb8e59
KLONOFFCOHEN HS, 1989, JAMA-J AM MED ASSOC, V262, P3143, DOI 10.1001/jama.262.22.3143
Lashley LEELO, 2015, J REPROD IMMUNOL, V112, P29, DOI 10.1016/j.jri.2015.05.006
Laufer-Ukeles P., 2018, The Disembodied Womb: Pregnancy, Informed Consent and Surrogate Motherhood Internet
LEETON J, 1986, J IN VITRO FERTIL EM, V3, P379, DOI 10.1007/BF01133252
LEETON J, 1988, J IN VITRO FERTIL EM, V5, P245, DOI 10.1007/BF01132171
Levron Y, 2014, AM J OBSTET GYNECOL, V211, DOI 10.1016/j.ajog.2014.03.044

Lucovnik M, 2016, HYPERTENS PREGNANCY, V35, P542, DOI 10.1080/10641955.2016.1197936
Maria D., 2022, Ann Romanian Soc Cell Biol., V26, P2025
Martínez-Varea A, 2014, J IMMUNOL RES, V2014, DOI 10.1155/2014/210241
Masoudian P, 2016, AM J OBSTET GYNECOL, V214, P328, DOI 10.1016/j.ajog.2015.11.020
Mastrobattista JM, 1997, AM J PERINAT, V14, P263, DOI 10.1055/s-2007-994140
Matorras R, 2023, HUM REPROD, V38, P895, DOI 10.1093/humrep/dead035
Maxwell CV, 2001, AM J OBSTET GYNECOL, V185, P819, DOI 10.1067/mob.2001.117352
Maxwell KN, 2008, FERTIL STERIL, V90, P2165, DOI 10.1016/j.fertnstert.2007.10.065
McCartney SA, 2023, AM J REPROD IMMUNOL, V89, DOI 10.1111/aji.13666
MCFARLANE A, 1976, J MED GENET, V13, P208, DOI 10.1136/jmg.13.3.208
MICHELOW MC, 1988, J IN VITRO FERTIL EM, V5, P31, DOI 10.1007/BF01138867
Miller DA, 1997, OBSTET GYNECOL, V89, P671, DOI 10.1016/S0029-7844(97)00073-2
Mochtar M, 2022, HUM REPROD, V37, P1693, DOI 10.1093/humrep/deac117
Pavlovic Z, 2020, J ASSIST REPROD GEN, V37, P953, DOI 10.1007/s10815-020-01728-3
Pecks U, 2011, DTSCH ARZTEBL INT, V108, P23, DOI 10.3238/arztebl.2011.0023
Perkins KM, 2016, FERTIL STERIL, V106, P435, DOI 10.1016/j.fertnstert.2016.03.050
Perni SC, 2005, J PERINAT MED, V33, P27, DOI 10.1515/JPM.2005.004
Pinborg A, 2023, FERTIL STERIL, V120, P449, DOI 10.1016/j.fertnstert.2023.04.022
Pohjonen EM, 2022, EUR J OBSTET GYN R B, V274, P210, DOI 10.1016/j.ejogrb.2022.05.031
ROBILLARD PY, 1994, LANCET, V344, P973, DOI 10.1016/S0140-6736(94)91638-1
Rodriguez-Wallberg KA, 2019, GYNECOL ENDOCRINOL, V35, P314, DOI 10.1080/09513590.2018.1528577
Roth AE, 2005, AM ECON REV, V95, P376, DOI 10.1257/0002828053828632
Roth AE, 2007, AM ECON REV, V97, P828, DOI 10.1257/aer.97.3.828
Savasi VM, 2016, HUM REPROD UPDATE, V22, P620, DOI 10.1093/humupd/dmw012
Shah A, 2019, J OBSTET GYN INDIA, V69, P395, DOI 10.1007/s13224-019-01211-9
Skupski DW, 1996, AM J OBSTET GYNECOL, V175, P1029, DOI 10.1016/S0002-9378(96)80047-0
Söderström-Anttila V, 2016, HUM REPROD UPDATE, V22, P260, DOI 10.1093/humupd/dmv046
Sparks TN, 2013, J MATERN-FETAL NEO M, V26, P1273, DOI 10.3109/14767058.2013.777701
Sridev Smisha, 2013, J Hum Reprod Sci, V6, P158, DOI 10.4103/0974-1208.117164
Storgaard M, 2017, BJOG-INT J OBSTET GY, V124, P561, DOI 10.1111/1471-0528.14257
Sung L, 1997, FERTIL STERIL, V67, P912, DOI 10.1016/S0015-0282(97)81406-9

Takemoto S, 2004, HUM IMMUNOL, V65, P1489, DOI
10.1016/j.humimm.2004.06.008
Tanderup M, 2015, ACTA OBSTET GYN SCAN, V94, P465, DOI
10.1111/aogs.12576
Taneja Jyoti, 2016, Case Rep Womens Health, V12, P1, DOI
10.1016/j.crwh.2016.10.001
Trombetta G, 2022, BMC PREGNANCY CHILDB, V22, DOI 10.1186/s12884-022-
04866-x
Tulay P, 2019, J TURK-GER GYNECOL A, V20, P236, DOI
10.4274/jtgga.galenos.2018.2018.0110
van Bentem K, 2020, J REPROD IMMUNOL, V137, DOI
10.1016/j.jri.2019.103074
van der Hoorn MLP, 2010, HUM REPROD UPDATE, V16, P704, DOI
10.1093/humupd/dmq017
van Nieuwenhoven ALV, 2003, HUM REPROD UPDATE, V9, P347, DOI
10.1093/humupd/dmg026
von Versen-Höynck F, 2022, HUM REPROD, V37, P1692, DOI
10.1093/humrep/deac116
Wang JX, 2002, LANCET, V359, P673, DOI 10.1016/S0140-6736(02)07804-2
Wang Y, 2021, RISK MANAG HEALTHC P, V14, P1999, DOI
10.2147/RMHP.S304040
Wen SW, 2004, AM J OBSTET GYNECOL, V191, P254, DOI
10.1016/j.ajog.2003.12.003
Williams Z, 2009, FERTIL STERIL, V91, P2593, DOI
10.1016/j.fertnstert.2008.02.008
Woo I, 2017, FERTIL STERIL, V108, P993, DOI
10.1016/j.fertnstert.2017.09.014
Yau A, 2021, AM J OBSTET GYNECOL, V225, P264, DOI
10.1016/j.ajog.2021.04.213
NR 83
TC 5
Z9 5
U1 0
U2 1
PU ELSEVIER IRELAND LTD
PI CLARE
PA ELSEVIER HOUSE, BROOKVALE PLAZA, EAST PARK SHANNON, CO, CLARE, 00000,
IRELAND
SN 0378-3782
EI 1872-6232
J9 EARLY HUM DEV
JI Early Hum. Dev.
PD SEP
PY 2024
VL 196
AR 106072
DI 10.1016/j.earlhumdev.2024.106072
EA AUG 2024
PG 10
WC Obstetrics & Gynecology; Pediatrics
WE Science Citation Index Expanded (SCI-EXPANDED)
SC Obstetrics & Gynecology; Pediatrics
GA C5Z7Z
UT WOS:001290157500001

PM 39106717
DA 2025-05-07
ER

PT J
AU Kuipers, RS
   Luxwolda, MF
   Dijck-Brouwer, DAJ
   Eaton, SB
   Crawford, MA
   Cordain, L
   Muskiet, FAJ
AF Kuipers, Remko S.
   Luxwolda, Martine F.
   Dijck-Brouwer, D. A. Janneke
   Eaton, S. Boyd
   Crawford, Michael A.
   Cordain, Loren
   Muskiet, Frits A. J.
TI Estimated macronutrient and fatty acid intakes from an East African
   Paleolithic diet
SO BRITISH JOURNAL OF NUTRITION
LA English
DT Article
DE Paleolithic diet; Land-water ecosystem; Hunter-gatherers; Evolutionary
   medicine; Macronutrients; Arachidonic acid; Linoleic acid;
   alpha-Linolenic acid; Docosahexaenoic acid; Cholesterol; Long-chain
PUFA
ID ARACHIDONIC-ACID; DOCOSAHEXAENOIC ACID; LINOLEIC-ACID; NUTRITIONAL
   COMPOSITION; CARDIOVASCULAR-DISEASE; EICOSAPENTAENOIC ACID;
   PHYSICAL-ACTIVITY; ADIPOSE-TISSUE; SERUM-LIPIDS; BREAST-MILK
AB Our genome adapts slowly to changing conditions of existence. Many diseases of civilisation result from mismatches between our Paleolithic genome and the rapidly changing environment, including our diet. The objective of the present study was to reconstruct multiple Paleolithic diets to estimate the ranges of nutrient intakes upon which humanity evolved. A database of, predominantly East African, plant and animal foods (meat/fish) was used to model multiple Paleolithic diets, using two pathophysiological constraints (i.e. protein,35 energy % (en%) and linoleic acid (LA) > 1.0 en%), at known hunter-gatherer plant/animal food intake ratios (range 70/30-30/70 en%/en%). We investigated selective and non-selective savannah, savannah/aquatic and aquatic hunter-gatherer/scavenger foraging strategies. We found (range of medians in en%) intakes of moderate-to-high protein (25-29), moderate-to-high fat (30-39) and moderate carbohydrates (39-40). The fatty acid composition was SFA (11.4-12.0), MUFA (5.6-18.5) and PUFA (8.6-15.2). The latter was high in a-linolenic acid (ALA) (3.7-4.7 en%), low in LA (2.3-3.6 en%), and high in long-chain PUFA (LCP; 4.75-25.8 g/d), LCP n-3 (2.26-17.0 g/d), LCP n-6 (2.54-8.84 g/d), ALA/LA ratio (1.12-1.64 g/g) and LCP n-3/LCP n-6 ratio (0.84-1.92 g/g). Consistent with the wide range of employed variables, nutrient intakes showed wide ranges. We conclude that compared with Western diets, Paleolithic diets contained consistently higher protein and LCP, and lower LA. These are likely to contribute to the known beneficial effects of Paleolithic-like diets, e. g. through

increased satiety/satiation. Disparities between Paleolithic, contemporary and recommended intakes might be important factors underlying the aetiology of common Western diseases. Data on Paleolithic diets and lifestyle, rather than the investigation of single nutrients, might be useful for the rational design of clinical trials.
C1 [Kuipers, Remko S.; Luxwolda, Martine F.; Dijck-Brouwer, D. A. Janneke; Muskiet, Frits A. J.] Univ Groningen, Dept Lab Med, Univ Med Ctr Groningen, NL-9700 RB Groningen, Netherlands.
[Eaton, S. Boyd] Emory Univ, Dept Anthropol, Atlanta, GA 30322 USA.
[Eaton, S. Boyd] Emory Univ, Dept Radiol, Atlanta, GA 30322 USA.
[Crawford, Michael A.] Univ London Imperial Coll Sci Technol & Med, Dept Biomol Med, London, England.
[Cordain, Loren] Colorado State Univ, Dept Hlth & Exercise Sci, Ft Collins, CO 80523 USA.
C3 University of Groningen; Emory University; Emory University; Imperial College London; Colorado State University System; Colorado State University Fort Collins
RP Kuipers, RS (autor correspondente), Univ Groningen, Dept Lab Med, Univ Med Ctr Groningen, POB 30-001, NL-9700 RB Groningen, Netherlands.
EM remkokuipers@hotmail.com
RI Kuipers, Remko/KBC-7216-2024; CRAWFOR, MICHAEL/MEP-1319-2025
OI Dijck-Brouwer, Janneke/0000-0003-1346-8254
CR ACKMAN RG, 1989, PROG FOOD NUTR SCI, V13, P161
AIELLO LC, 1995, CURR ANTHROPOL, V36, P199, DOI 10.1086/204350
Ailhaud G, 2006, PROG LIPID RES, V45, P203, DOI 10.1016/j.plipres.2006.01.003
Alhassan S, 2008, INT J OBESITY, V32, P985, DOI 10.1038/ijo.2008.8
Astorg P, 2004, LIPIDS, V39, P527, DOI 10.1007/s11745-004-1259-6
Barclay AW, 2008, AM J CLIN NUTR, V87, P627, DOI 10.1093/ajcn/87.3.627
Batterham RL, 2006, CELL METAB, V4, P223, DOI 10.1016/j.cmet.2006.08.001
Beasley JM, 2009, AM J EPIDEMIOL, V169, P893, DOI 10.1093/aje/kwn415
Bensinger SJ, 2008, NATURE, V454, P470, DOI 10.1038/nature07202
Berglund L, 2007, AM J CLIN NUTR, V86, P1611
Bhaskar N, 2004, Z NATURFORSCH C, V59, P310
BLUMENSCHINE RJ, 1992, SCI AM, V267, P90, DOI 10.1038/scientificamerican1092-90
BLUMENSCHINE RJ, 1993, J ARCHAEOL SCI, V20, P555, DOI 10.1006/jasc.1993.1034
Brand-Miller JC, 1998, NUTR RES REV, V11, P5, DOI 10.1079/NRR19980003
Broadhurst CL, 1998, BRIT J NUTR, V79, P3, DOI 10.1079/BJN19980004
Broadhurst CL, 2002, COMP BIOCHEM PHYS B, V131, P653, DOI 10.1016/S1096-4959(02)00002-7
BUNN HT, 1993, J ARCHAEOL SCI, V20, P365, DOI 10.1006/jasc.1993.1023
Burr GO, 1930, J BIOL CHEM, V86, P0587
Calder PC, 2010, CLIN NUTR, V29, P5, DOI 10.1016/j.clnu.2009.11.003
Calder PC, 2009, BIOCHIMIE, V91, P791, DOI 10.1016/j.biochi.2009.01.008
Caspi A, 2007, P NATL ACAD SCI USA, V104, P18860, DOI 10.1073/pnas.0704292104
*CDC, 2004, TRENDS INT EN MACR U
Chawla A, 2001, SCIENCE, V294, P1866, DOI 10.1126/science.294.5548.1866
Clarke R, 1997, BRIT MED J, V314, P112, DOI 10.1136/bmj.314.7074.112

COLLINS FD, 1971, NUTR METAB, V13, P150
Conquer JA, 1997, LIPIDS, V32, P341, DOI 10.1007/s11745-997-0043-y
Cordain L, 1998, INT J SPORTS MED, V19, P328, DOI 10.1055/s-2007-971926
Cordain L, 2002, EUR J CLIN NUTR, V56, P181, DOI 10.1038/sj.ejcn.1601307
Cordain L, 2001, WORLD REV NUTR DIET, V90, P144
Cordain L, 2000, AM J CLIN NUTR, V71, P682, DOI 10.1093/ajcn/71.3.682
Crawford MA, 1999, LIPIDS, V34, pS39, DOI 10.1007/BF02562227
CRAWFORD MA, 1968, LANCET, V1, P1329
CRAWFORD MA, 1971, INT J BIOCHEM, V2, P493, DOI 10.1016/0020-711X(71)90017-6
CRAWFORD MA, 1969, BIOCHEM J, V115, P25, DOI 10.1042/bj1150025
CRAWFORD MA, 1970, INT J BIOCHEM, V1, P295, DOI 10.1016/0020-711X(70)90072-8
CRAWFORD MA, 1970, INT J BIOCHEM, V1, P654, DOI 10.1016/0020-711X(70)90039-X
CRAWFORDMA, 2002, NUTR HLTH, V16, P29
Cunnane SC, 2006, PEDIATR RES, V59, P107, DOI 10.1203/01.pdr.0000190569.07991.ed
Cunnane SC, 2005, AM J CLIN NUTR, V82, P483, DOI 10.1093/ajcn/82.2.483
Cunnane SC, 2003, PROG LIPID RES, V42, P544, DOI 10.1016/S0163-7827(03)00038-9
Cunnane SC, 2003, COMP BIOCHEM PHYS A, V136, P17, DOI 10.1016/S1095-6433(03)00048-5
CUTHBERTSON WFJ, 1976, AM J CLIN NUTR, V29, P559, DOI 10.1093/ajcn/29.5.559
Davis JE, 2009, HORM METAB RES, V41, P523, DOI 10.1055/s-0029-1202852
de Lorgeril M, 1999, CIRCULATION, V99, P779, DOI 10.1161/01.CIR.99.6.779
DEMBITSKY VM, 1991, PHYTOCHEMISTRY, V30, P2279, DOI 10.1016/0031-9422(91)83630-4
Djousse L, 2005, CIRCULATION, V111, pE190
DUNCAN WRH, 1968, COMP BIOCHEM PHYSIOL, V25, P319, DOI 10.1016/0010-406X(68)90939-0
Eaton SB, 1998, WORLD REV NUTR DIET, V83, P12
Eaton SB, 2000, EUR J NUTR, V39, P67, DOI 10.1007/s003940070032
Eaton SB, 1996, J NUTR, V126, P1732, DOI 10.1093/jn/126.6.1732
Eaton SB, 2003, COMP BIOCHEM PHYS A, V136, P153, DOI 10.1016/S1095-6433(03)00208-3
EATON SB, 1992, LIPIDS, V27, P814, DOI 10.1007/BF02535856
Eaton SB, 1997, EUR J CLIN NUTR, V51, P207, DOI 10.1038/sj.ejcn.1600389
EATON SB, 1985, NEW ENGL J MED, V312, P283, DOI 10.1056/NEJM198501313120505
Erlandson JM, 2001, J ARCHAEOL RES, V9, P287, DOI 10.1023/A:1013062712695
Ervin R Bethene, 2004, Adv Data, P1
Ezeagu IE, 1998, J AM OIL CHEM SOC, V75, P1031, DOI 10.1007/s11746-998-0282-6
Ferretti A, 1997, LIPIDS, V32, P435, DOI 10.1007/s11745-997-0057-5
FESKENS EJM, 1993, ANN NY ACAD SCI, V683, P9, DOI 10.1111/j.1749-6632.1993.tb35688.x
Frassetto L, 2001, EUR J NUTR, V40, P200, DOI 10.1007/s394-001-8347-4

Frassetto LA, 2009, EUR J CLIN NUTR, V63, P947, DOI 10.1038/ejcn.2009.4
Freiberger CE, 1998, PLANT FOOD HUM NUTR, V53, P57, DOI 10.1023/A:1008080508028
Gardner CD, 2007, JAMA-J AM MED ASSOC, V297, P969, DOI 10.1001/jama.297.9.969
Givens DI, 2008, P NUTR SOC, V67, P273, DOI 10.1017/S0029665108007167
Goldstone AP, 2009, EUR J NEUROSCI, V30, P1625, DOI 10.1111/j.1460-9568.2009.06949.x
Guil JL, 1996, J CHROMATOGR A, V719, P229, DOI 10.1016/0021-9673(95)00414-9
HANSEN HS, 1986, BIOCHIM BIOPHYS ACTA, V878, P284, DOI 10.1016/0005-2760(86)90158-X
Harris WS, 2009, CIRCULATION, V119, P902, DOI 10.1161/CIRCULATIONAHA.108.191627
HART AR, 2009, GUT
Hawkes K, 1998, P NATL ACAD SCI USA, V95, P1336, DOI 10.1073/pnas.95.3.1336
Hibbeln Joseph R., 2009, V99, P17, DOI 10.1159/000192992
Hibbeln JR, 2002, J AFFECT DISORDERS, V69, P15, DOI 10.1016/S0165-0327(01)00374-3
Hill K, 2007, J HUM EVOL, V52, P443, DOI 10.1016/j.jhevol.2006.11.003
Holman R.T., 1971, Progress in the Chemistry of Fats and other Lipids, V9, P607
HOLMAN RT, 1982, AM J CLIN NUTR, V35, P617, DOI 10.1093/ajcn/35.3.617
Hsieh AT, 2009, PROSTAG LEUKOTR ESS, V81, P105, DOI 10.1016/j.plefa.2009.05.012
Hudgins LC, 2003, J LIPID RES, V44, P1489, DOI 10.1194/jlr.M200440-JLR200
Hunt JR, 2009, AM J CLIN NUTR, V89, P1357, DOI 10.3945/ajcn.2008.27238
INNIS SM, 1988, EARLY HUM DEV, V18, P185, DOI 10.1016/0378-3782(88)90055-2
Institute of Medicine, 2005, DIET REF INT EN CARB, DOI DOI 10.17226/10490
Isaac B., 1987, The African Archaeological Review, V5, P3, DOI [DOI 10.1007/BF01117078, 10.1007/BF01117078]
Jönsson T, 2009, CARDIOVASC DIABETOL, V8, DOI 10.1186/1475-2840-8-35
Kelley DS, 1998, LIPIDS, V33, P125, DOI 10.1007/s11745-998-0187-9
Kelley DS, 1997, LIPIDS, V32, P449, DOI 10.1007/s11745-997-0059-3
KHOTIMCHENKO SV, 1991, PHYTOCHEMISTRY, V30, P2639, DOI 10.1016/0031-9422(91)85113-E
KLEIN CJ, 2006, SCI EVIDENCE APPROAC
Koletzko B, 2008, J PERINAT MED, V36, P5, DOI 10.1515/JPM.2008.001
Kuipers RS, 2007, PROSTAG LEUKOTR ESS, V76, P221, DOI 10.1016/j.plefa.2007.01.004
Kuipers RS, 2005, PROSTAG LEUKOTR ESS, V72, P279, DOI 10.1016/j.plefa.2004.12.001
KUNSMAN JE, 1981, J FOOD SCI, V46, P1785, DOI 10.1111/j.1365-2621.1981.tb04485.x
Kusumoto A, 2007, BRIT J NUTR, V98, P626, DOI 10.1017/S0007114507734566
Last AR, 2006, AM FAM PHYSICIAN, V73, P1942
Le HD, 2009, PROSTAG LEUKOTR ESS, V81, P165, DOI 10.1016/j.plefa.2009.05.020

LEDGER HP, 1969, S ZOOL SOC LOND, V21, P280
Lee R.E., 1968, Man the Hunter
Leonard WR, 2003, COMP BIOCHEM PHYS A, V136, P5, DOI 10.1016/S1095-6433(03)00132-6
LEONARD WR, 1992, AM J HUM BIOL, V4, P179, DOI 10.1002/ajhb.1310040204
Lieb CW, 1929, J AMER MED ASSOC, V96, P20
Lien EL, 2009, PROSTAG LEUKOTR ESS, V81, P125, DOI 10.1016/j.plefa.2009.05.004
Lindeberg S, 2007, DIABETOLOGIA, V50, P1795, DOI 10.1007/s00125-007-0716-y
Liou YA, 2009, PROSTAG LEUKOTR ESS, V80, P201, DOI 10.1016/j.plefa.2009.02.003
Lupo KD, 1998, J ARCHAEOL SCI, V25, P657, DOI 10.1006/jasc.1997.0261
MAKRIDES M, 1994, AM J CLIN NUTR, V60, P189, DOI 10.1093/ajcn/60.2.189
MANN GV, 1965, LANCET, V2, P1308
MANN GV, 1962, J CHRON DIS, V15, P341, DOI 10.1016/0021-9681(62)90082-6
Mann N, 1997, LIPIDS, V32, P635, DOI 10.1007/s11745-997-0081-5
Marchioli R, 2002, CIRCULATION, V105, P1897, DOI 10.1161/01.CIR.0000014682.14181.F2
Matalas AL, 2006, INT J FOOD SCI NUTR, V57, P529, DOI 10.1080/09637480601041037
McCann JC, 2005, AM J CLIN NUTR, V82, P281, DOI 10.1093/ajcn/82.2.281
MEAD JF, 1960, AM J CLIN NUTR, V8, P55, DOI 10.1093/ajcn/8.1.55
Mensink RP, 2003, AM J CLIN NUTR, V77, P1146, DOI 10.1093/ajcn/77.5.1146
Mente A, 2009, ARCH INTERN MED, V169, P659, DOI 10.1001/archinternmed.2009.38
Milton K, 2003, J NUTR, V133, p3886S, DOI 10.1093/jn/133.11.3886S
Mozaffarian D, 2004, AM J CLIN NUTR, V80, P1175, DOI 10.1093/ajcn/80.5.1175
Mozaffarian D, 2006, JAMA-J AM MED ASSOC, V296, P1885, DOI 10.1001/jama.296.15.1885
Murdock GP, 1967, Ethnographic atlas
NAUGHTON JM, 1986, LIPIDS, V21, P684, DOI 10.1007/BF02537241
Nelson GJ, 1997, LIPIDS, V32, P421, DOI 10.1007/s11745-997-0055-7
Niwano Y, 2009, J NUTR SCI VITAMINOL, V55, P201, DOI 10.3177/jnsv.55.201
Norziah MH, 2000, FOOD CHEM, V68, P69, DOI 10.1016/S0308-8146(99)00161-2
O'Keefe JH, 2004, J AM COLL CARDIOL, V43, P2142, DOI 10.1016/j.jacc.2004.03.046
ODEA K, 1985, J NUTR SCI VITAMINOL, V31, P441, DOI 10.3177/jnsv.31.441
Österdahl M, 2008, EUR J CLIN NUTR, V62, P682, DOI 10.1038/sj.ejcn.1602790
Oyarzun SE, 1996, ZOO BIOL, V15, P509, DOI 10.1002/(SICI)1098-2361(1996)15:5<509::AID-ZOO7>3.0.CO;2-F
Pauletto P, 1996, LIPIDS, V31, pS309, DOI 10.1007/BF02637098
Plourde M, 2007, APPL PHYSIOL NUTR ME, V32, P619, DOI 10.1139/H07-034
Psota TL, 2006, AM J CARDIOL, V98, p3I, DOI 10.1016/j.amjcard.2005.12.022
Ramakers JD, 2008, BRIT J NUTR, V100, P347, DOI 10.1017/S0007114507901257
Rao JS, 2007, MOL PSYCHIATR, V12, P151, DOI 10.1038/sj.mp.4001887

Richards MP, 2003, NATURE, V425, P366, DOI 10.1038/425366a
ROBERTS MB, 1994, NATURE, V369, P311, DOI 10.1038/369311a0
Rocquelin G, 1998, INT J FOOD SCI NUTR, V49, P343, DOI 10.3109/09637489809089409
Rolls BJ, 2009, PHYSIOL BEHAV, V97, P609, DOI 10.1016/j.physbeh.2009.03.011
RUDMAN D, 1973, J CLIN INVEST, V52, P2241, DOI 10.1172/JCI107410
Sailer L.D., 1985, Primates, V26, P14, DOI 10.1007/BF02389044
Sanders TAB, 2009, PROSTAG LEUKOTR ESS, V81, P137, DOI 10.1016/j.plefa.2009.05.013
Sanigorski AJ, 1996, LIPIDS, V31, P729, DOI 10.1007/BF02522889
Sargent JR, 1997, BRIT J NUTR, V78, pS5, DOI 10.1079/BJN19970131
Schaeffler A, 2009, IMMUNOLOGY, V126, P233, DOI 10.1111/j.1365-2567.2008.02892.x
Sear R, 2000, P ROY SOC B-BIOL SCI, V267, P1641, DOI 10.1098/rspb.2000.1190
Serhan CN, 2008, PROSTAG LEUKOTR ESS, V79, P157, DOI 10.1016/j.plefa.2008.09.012
SEYBERTH HW, 1975, CLIN PHARMACOL THER, V18, P521
Simopoulos AP, 2008, EXP BIOL MED, V233, P674, DOI 10.3181/0711-MR-311
SINCLAIR AJ, 1982, J SCI FOOD AGR, V33, P771, DOI 10.1002/jsfa.2740330814
Smit E, 1999, J AM DIET ASSOC, V99, P813, DOI 10.1016/S0002-8223(99)00193-5
SPETH JD, 1983, J ANTHROPOL ARCHAEOL, V2, P1, DOI 10.1016/0278-4165(83)90006-5
STANLEYSAMUELSON DW, 1983, INSECT BIOCHEM, V13, P549, DOI 10.1016/0020-1790(83)90014-8
Stark AH, 2008, NUTR REV, V66, P326, DOI 10.1111/j.1753-4887.2008.00040.x
Steward J.H., 1968, MAN HUNTER, P321
STEWART KM, 1994, J HUM EVOL, V27, P229, DOI 10.1006/jhev.1994.1044
Stringer C, 2000, NATURE, V405, P24, DOI 10.1038/35011166
Sugano M, 2000, AM J CLIN NUTR, V71, p189S, DOI 10.1093/ajcn/71.1.189S
Tunstall-Pedoe H, 1999, LANCET, V353, P1547, DOI 10.1016/S0140-6736(99)04021-0
Ulijaszek SJ, 2002, P NUTR SOC, V61, P517, DOI 10.1079/PNS2002180
UNITED STATES DEPARTMENT OF AGRICULTURE - USDA, 2008, NAT NUTR DAT STAND R
van Goor SA, 2008, PROSTAG LEUKOTR ESS, V78, P159, DOI 10.1016/j.plefa.2008.01.004
VINCENT AS, 1985, WORLD ARCHAEOL, V17, P131, DOI 10.1080/00438243.1985.9979958
Volek JS, 2008, PROG LIPID RES, V47, P307, DOI 10.1016/j.plipres.2008.02.003
Wang S, 2007, PLOS GENET, V3, P2049, DOI 10.1371/journal.pgen.0030185
Washburn S.L., 1968, Man the Hunter, P293
Wehmeyer A S, 1969, S Afr Med J, V43, P1529
Wells JCK, 2006, BIOL REV, V81, P183, DOI 10.1017/S1464793105006974
Westerterp-Plantenga MS, 2009, ANNU REV NUTR, V29, P21, DOI 10.1146/annurev-nutr-080508-141056
Whelan J, 1997, ADV EXP MED BIOL, V400, P897
White TD, 2009, SCIENCE, V326, P87, DOI 10.1126/science.1175822

Wolfe AR, 2009, PROG NEURO-PSYCHOPH, V33, P972, DOI
10.1016/j.pnpbp.2009.05.002
Woodburn J., 1968, MAN HUNTER, DOI DOI 10.4324/9780203786567
Xie L, 2008, J NUTR, V138, P2222, DOI 10.3945/jn.108.096156
Yam D, 1996, ISRAEL J MED SCI, V32, P1134
Zhao GX, 2007, AM J CLIN NUTR, V85, P385, DOI 10.1093/ajcn/85.2.385
Zhou BF, 2003, J HUM HYPERTENS, V17, P623, DOI 10.1038/sj.jhh.1001605
NR 171
TC 100
Z9 111
U1 0
U2 78
PU CAMBRIDGE UNIV PRESS
PI CAMBRIDGE
PA EDINBURGH BLDG, SHAFTESBURY RD, CB2 8RU CAMBRIDGE, ENGLAND
SN 0007-1145
EI 1475-2662
J9 BRIT J NUTR
JI Br. J. Nutr.
PD DEC
PY 2010
VL 104
IS 11
BP 1666
EP 1687
DI 10.1017/S0007114510002679
PG 22
WC Nutrition & Dietetics
WE Science Citation Index Expanded (SCI-EXPANDED); Social Science
Citation Index (SSCI)
SC Nutrition & Dietetics
GA 696TI
UT WOS:000285463900014
PM 20860883
OA Bronze, Green Submitted
DA 2025-05-07
ER

PT J
AU Amoroso, CR
Nunn, CL
AF Amoroso, Caroline R.
Nunn, Charles L.
TI Human parasitism in a comparative context: Are humans exceptionally
parasitized?
SO AMERICAN JOURNAL OF PHYSICAL ANTHROPOLOGY
LA English
DT Meeting Abstract
CT 86th Annual Meeting of the
American-Association-of-Physical-Anthropologists (AAPA)
CY APR 19-22, 2017
CL New Orleans, LA
C1 [Amoroso, Caroline R.; Nunn, Charles L.] Duke Univ, Evolutionary
Anthropol, Durham, NC 27706 USA.

[Nunn, Charles L.] Duke Univ, Global Hlth Inst, Durham, NC 27706 USA.
C3 Duke University; Duke University
FU National Science Foundation [BCS-1355902]; National Evolutionary Synthesis Center and Triangle Center for Evolutionary Medicine [EF-0905606]
FX Funding for this research was provided by the National Science Foundation (BCS-1355902) and by the National Evolutionary Synthesis Center and Triangle Center for Evolutionary Medicine (EF-0905606).
NR 0
TC 0
Z9 0
U1 0
U2 0
PU WILEY
PI HOBOKEN
PA 111 RIVER ST, HOBOKEN 07030-5774, NJ USA
SN 0002-9483
EI 1096-8644
J9 AM J PHYS ANTHROPOL
JI Am. J. Phys. Anthropol.
PD APR
PY 2017
VL 162
SU 64
BP 99
EP 100
PG 2
WC Anthropology; Evolutionary Biology
WE Science Citation Index Expanded (SCI-EXPANDED); Social Science Citation Index (SSCI); Conference Proceedings Citation Index - Science (CPCI-S)
SC Anthropology; Evolutionary Biology
GA FT3QT
UT WOS:000423063101028
DA 2025-05-07
ER

PT J
AU Nicholas, LM
Ozanne, SE
AF Nicholas, Lisa M.
Ozanne, Susan E.
TI Early life programming in mice by maternal overnutrition: mechanistic insights and interventional approaches
SO PHILOSOPHICAL TRANSACTIONS OF THE ROYAL SOCIETY B-BIOLOGICAL SCIENCES
LA English
DT Article
DE maternal overnutrition; animal models; epigenetics; intervention studies
ID DIET-INDUCED OBESITY; INSULIN-RESISTANCE; MITOCHONDRIAL DYSFUNCTION; EPIGENETIC MODIFICATIONS; METABOLIC DYSFUNCTION; SEX-DIFFERENCES; PREGNANT-WOMEN; FATTY LIVER; FOLLOW-UP; EXERCISE
AB Animal models have been indispensable in elucidating the potential causative mechanisms underlying the effects of maternal diet on offspring

health. Of these, the mouse has been widely used to model maternal overnutrition and/or maternal obesity and to study its effects across one or more generations. This review discusses recent findings from mouse models, which resemble the human situation, i.e. overnutrition/obesity across pregnancy and lactation. It also highlights the importance of embryo transfer models in identifying critical developmental period(s) during which specific metabolic changes are programmed in the offspring. The mouse is also an excellent tool for maternal intervention studies aimed at elucidating the longer-term effects on the offspring and for defining possible maternal factors underling the programming of metabolic adversity in offspring. While knowledge of the mouse genome and the molecular tools available have allowed great progress to be made in the field, it is clear that we need to define if the effects on the offspring are mediated by maternal obesity per se or if specific components of the maternal metabolic environment are more important. We can then begin to identify at-risk offspring and to design more effective interventions for the mother and/or her child.

This article is part of the theme issue 'Developing differences: early-life effects and evolutionary medicine'.
C1 [Nicholas, Lisa M.; Ozanne, Susan E.] Univ Cambridge, Addenbrookes Hosp, Wellcome Trust MRC Inst Metab Sci, Metab Res Labs,MRC Metab Dis Unit, Cambridge, England.
C3 University of Cambridge; Cambridge University Hospitals NHS Foundation Trust; Addenbrooke's Hospital
RP Nicholas, LM; Ozanne, SE (autor correspondente), Univ Cambridge, Addenbrookes Hosp, Wellcome Trust MRC Inst Metab Sci, Metab Res Labs,MRC Metab Dis Unit, Cambridge, England.
EM ln305@medschl.cam.ac.uk; seo10@cam.ac.uk
OI Ozanne, Susan/0000-0001-8753-5144; Nicholas, Lisa/0000-0003-1976-1953
FU Isaac Newton Trust [17.37(l)]; Medical Research Council [MC_UU_12012/4];
British Heart Foundation [RG/17/12/33167]; MRC [MC_UU_12012/5, MC_UU_00014/4, MC_UU_12012/4] Funding Source: UKRI
FX L.M.N. is funded by the Isaac Newton Trust [17.37(l)] and S.E.O. is funded by the Medical Research Council (MC_UU_12012/4) and the British Heart Foundation (RG/17/12/33167).
CR Aiken CE, 2013, REPRODUCTION, V145, pR1, DOI 10.1530/REP-11-0489
Alfaradhi MZ, 2014, AM J PHYSIOL-REG I, V307, pR26, DOI 10.1152/ajpregu.00049.2014
Aye ILMH, 2015, P NATL ACAD SCI USA, V112, P12858, DOI 10.1073/pnas.1515484112
Bateson P., 1999, Design for a life: How behavior develops
Boots CE, 2016, HUM REPROD, V31, P2090, DOI 10.1093/humrep/dew181
Boudoures AL, 2016, REPRODUCTION, V151, P261, DOI 10.1530/REP-15-0419
Bruce KD, 2009, HEPATOLOGY, V50, P1796, DOI 10.1002/hep.23205
Chiswick C, 2015, LANCET DIABETES ENDO, V3, P778, DOI 10.1016/S2213-8587(15)00219-3
Dabelea D, 2000, DIABETES, V49, P2208, DOI 10.2337/diabetes.49.12.2208
Dickinson H, 2016, J DEV ORIG HLTH DIS, V7, P449, DOI 10.1017/S2040174416000477
Dodd JM, 2016, ACTA OBSTET GYN SCAN, V95, P309, DOI 10.1111/aogs.12832
Dudele A, 2017, INT J OBESITY, V41, P1420, DOI 10.1038/ijo.2017.136
DUHL DMJ, 1994, NAT GENET, V8, P59, DOI 10.1038/ng0994-59

Duque-Guimaraes DE, 2013, TRENDS ENDOCRIN MET, V24, P525, DOI 10.1016/j.tem.2013.05.006
Ferland-McCollough D, 2012, CELL DEATH DIFFER, V19, P1003, DOI 10.1038/cdd.2011.183
Fernandez-Twinn DS, 2017, SCI REP-UK, V7, DOI 10.1038/srep44650
Fernandez-Twinn DS, 2014, MOL METAB, V3, P325, DOI 10.1016/j.molmet.2014.01.007
Fernandez-Twinn DS, 2012, ENDOCRINOLOGY, V153, P5961, DOI 10.1210/en.2012-1508
Fex M, 2018, J ENDOCRINOL, V236, pR145, DOI 10.1530/JOE-17-0367
Finan B, 2012, NAT MED, V18, P1847, DOI 10.1038/nm.3009
Friedman JR, 2014, NATURE, V505, P335, DOI 10.1038/nature12985
Gluckman PD, 2007, AM J HUM BIOL, V19, P1, DOI 10.1002/ajhb.20590
Gregorio BM, 2010, AM J OBSTET GYNECOL, V203, DOI 10.1016/j.ajog.2010.06.042
Han LS, 2017, J PINEAL RES, V63, DOI 10.1111/jpi.12431
Hanem LGE, 2018, J CLIN ENDOCR METAB, V103, P1612, DOI 10.1210/jc.2017-02419
Hartil K, 2009, PEDIATR RES, V66, P368, DOI 10.1203/PDR.0b013e3181b33375
Hochner H, 2012, CIRCULATION, V125, P1381, DOI 10.1161/CIRCULATIONAHA.111.070060
Huypens P, 2016, NAT GENET, V48, P497, DOI 10.1038/ng.3527
Isagawa T, 2011, PLOS ONE, V6, DOI 10.1371/journal.pone.0026052
Jiménez-Chillarón JC, 2012, BIOCHIMIE, V94, P2242, DOI 10.1016/j.biochi.2012.06.012
Jonscher KR, 2017, FASEB J, V31, P1434, DOI 10.1096/fj.201600906R
Jungheim ES, 2010, ENDOCRINOLOGY, V151, P4039, DOI 10.1210/en.2010-0098
Kang SS, 2014, J NEUROINFLAMM, V11, DOI 10.1186/s12974-014-0156-9
Kelley DE, 2002, DIABETES, V51, P2944, DOI 10.2337/diabetes.51.10.2944
King V, 2013, ENDOCRINOLOGY, V154, P2514, DOI 10.1210/en.2013-1013
Kobayashi H, 2012, PLOS GENET, V8, DOI 10.1371/journal.pgen.1002440
Laker RC, 2014, DIABETES, V63, P1605, DOI [10.2337/db13-1614, 10.2337/db14-0135]
Lawlor DA, 2011, CIRCULATION, V123, P258, DOI 10.1161/CIRCULATIONAHA.110.980169
Li CCY, 2013, EPIGENETICS-US, V8, P602, DOI 10.4161/epi.24656
Li CCY, 2011, PLOS GENET, V7, DOI [10.1371/journal.pgen.1001380, 10.1371/journal.pgen.1002196]
Liang XW, 2016, J PHYSIOL-LONDON, V594, P4453, DOI 10.1113/JP272123
Louet Jean-Francois, 2004, Curr Atheroscler Rep, V6, P180, DOI 10.1007/s11883-004-0030-9
Luzzo KM, 2012, PLOS ONE, V7, DOI 10.1371/journal.pone.0049217
Margueron R, 2010, NAT REV GENET, V11, P285, DOI 10.1038/nrg2752
Matthews PA, 2011, J PHYSIOL-LONDON, V589, P3969, DOI 10.1113/jphysiol.2011.212324
McCormick H, 2017, BMC GENOMICS, V18, DOI 10.1186/s12864-017-4350-x
McPherson NO, 2015, AM J PHYSIOL-ENDOC M, V309, pE568, DOI 10.1152/ajpendo.00230.2015
Minge CE, 2008, ENDOCRINOLOGY, V149, P2646, DOI 10.1210/en.2007-1570
Monks J, 2018, NUTR DIABETES, V8, DOI 10.1038/s41387-018-0027-z
Morgan HD, 2005, HUM MOL GENET, V14, pR47, DOI 10.1093/hmg/ddi114
Nicholas LM, 2016, INT J OBESITY, V40, P229, DOI 10.1038/ijo.2015.178

Oben JA, 2010, J HEPATOL, V52, P913, DOI 10.1016/j.jhep.2009.12.042
Oostvogels AJJM, 2014, J CLIN ENDOCR METAB, V99, P3845, DOI 10.1210/jc.2014-1561
Poston L, 2015, LANCET DIABETES ENDO, V3, P767, DOI 10.1016/S2213-8587(15)00227-2
Saben JL, 2016, CELL REP, V16, P1, DOI 10.1016/j.celrep.2016.05.065
Samuelsson AM, 2008, HYPERTENSION, V51, P383, DOI 10.1161/HYPERTENSIONAHA.107.101477
Sasaki A, 2013, NEUROSCIENCE, V240, P1, DOI 10.1016/j.neuroscience.2013.02.044
Sasson IE, 2015, DIABETOLOGIA, V58, P615, DOI 10.1007/s00125-014-3466-7
Semple RK, 2006, J CLIN INVEST, V116, P581, DOI 10.1172/JCI28003
Smith J, 2009, J CLIN ENDOCR METAB, V94, P4275, DOI 10.1210/jc.2009-0709
Stanford KI, 2017, DIABETES, V66, P2124, DOI 10.2337/db17-0098
Stanford KI, 2015, DIABETES, V64, P427, DOI 10.2337/db13-1848
Sun ZY, 2013, CELL REP, V3, P567, DOI 10.1016/j.celrep.2013.01.001
Wang Q, 2009, MOL ENDOCRINOL, V23, P1603, DOI 10.1210/me.2009-0033
Wankhade UD, 2017, PLOS ONE, V12, DOI 10.1371/journal.pone.0175675
Waterland RA, 2008, INT J OBESITY, V32, P1373, DOI 10.1038/ijo.2008.100
Yang QY, 2013, DIABETES, V62, P3727, DOI 10.2337/db13-0433
Yokomizo H, 2014, AM J PHYSIOL-ENDOC M, V306, pE1163, DOI 10.1152/ajpendo.00688.2013
NR 68
TC 24
Z9 27
U1 0
U2 4
PU ROYAL SOC
PI LONDON
PA 6-9 CARLTON HOUSE TERRACE, LONDON SW1Y 5AG, ENGLAND
SN 0962-8436
EI 1471-2970
J9 PHILOS T R SOC B
JI Philos. Trans. R. Soc. B-Biol. Sci.
PD APR 15
PY 2019
VL 374
IS 1770
AR 20180116
DI 10.1098/rstb.2018.0116
PG 8
WC Biology
WE Science Citation Index Expanded (SCI-EXPANDED)
SC Life Sciences & Biomedicine - Other Topics
GA HN8ZI
UT WOS:000460487000007
PM 30966886
OA Bronze, Green Published
DA 2025-05-07
ER

PT J
AU Dantzer, B
Dubuc, C
Braga Goncalves, I
Cram, DL
Bennett, NC
Ganswindt, A
Heistermann, M
Duncan, C
Gaynor, D
Clutton-Brock, TH
AF Dantzer, Ben
Dubuc, Constance
Braga Goncalves, Ines
Cram, Dominic L.
Bennett, Nigel C.
Ganswindt, Andre
Heistermann, Michael
Duncan, Chris
Gaynor, David
Clutton-Brock, Tim H.
TI The development of individual differences in cooperative behaviour:
maternal glucocorticoid hormones alter helping behaviour of offspring in
wild meerkats
SO PHILOSOPHICAL TRANSACTIONS OF THE ROYAL SOCIETY B-BIOLOGICAL SCIENCES
LA English
DT Article
DE cooperation; early life adversity; glucocorticoids; growth; maternal stress
ID REPRODUCTIVE SUCCESS; SEX-DIFFERENCES; FUTURE FITNESS; LIFE-HISTORY;
EVOLUTION; HELPERS; GROWTH; HYMENOPTERA; PLASTICITY; SOCIALITY
AB The phenotype of parents can have long-lasting effects on the development of offspring as well as on their behaviour, physiology and morphology as adults. In some cases, these changes may increase offspring fitness but, in others, they can elevate parental fitness at a cost to the fitness of their offspring. We show that in Kalahari meerkats (Suricata suricatta), the circulating glucocorticoid (GC) hormones of pregnant females affect the growth and cooperative behaviour of their offspring. We performed a 3-year experiment in wild meerkats to test the hypothesis that GC-mediated maternal effects reduce the potential for offspring to reproduce directly and therefore cause them to exhibit more cooperative behaviour. Daughters (but not sons) born to mothers treated with cortisol during pregnancy grew more slowly early in life and exhibited significantly more of two types of cooperative behaviour (pup rearing and feeding) once they were adults compared to offspring from control mothers. They also had lower measures of GCs as they aged, which could explain the observed increases in cooperative behaviour. Because early life growth is a crucial determinant of fitness in female meerkats, our results indicate that GC-mediated maternal effects may reduce the fitness of offspring, but may elevate parental fitness as a consequence of increasing the cooperative behaviour of their daughters.
This article is part of the theme issue 'Developing differences: early-life effects and evolutionary medicine'.

C1 [Dantzer, Ben; Dubuc, Constance; Cram, Dominic L.; Duncan, Chris; Clutton-Brock, Tim H.] Univ Cambridge, Dept Zool, Cambridge CB2 3EJ, England.
[Dantzer, Ben; Dubuc, Constance; Braga Goncalves, Ines; Cram, Dominic L.; Duncan, Chris; Gaynor, David; Clutton-Brock, Tim H.] Kuruman River Reserve, Kalahari Meerkat Project, Northern Cape, South Africa.
[Dantzer, Ben] Univ Michigan, Dept Psychol, Ann Arbor, MI 48109 USA.
[Dantzer, Ben] Univ Michigan, Dept Ecol & Evolutionary Biol, Ann Arbor, MI 48109 USA.
[Braga Goncalves, Ines] Univ Bristol, Sch Biol Sci, Bristol BS8 1TQ, Avon, England.
[Bennett, Nigel C.; Ganswindt, Andre; Gaynor, David; Clutton-Brock, Tim H.] Univ Pretoria, Mammal Res Inst, Dept Zool & Entomol, ZA-0002 Pretoria, South Africa.
[Ganswindt, Andre] Univ Pretoria, Dept Anat & Physiol, Endocrine Res Lab, ZA-0110 Onderstepoort, South Africa.
[Heistermann, Michael] Leibniz Inst Primate Res, German Primate Ctr, Endocrinol Lab, Kellnerweg 4, D-37077 Gottingen, Germany.
C3 University of Cambridge; University of Michigan System; University of Michigan; University of Michigan System; University of Michigan; University of Bristol; University of Pretoria; University of Pretoria; Leibniz Association; Deutsches Primatenzentrum (DPZ)
RP Dantzer, B (autor correspondente), Univ Cambridge, Dept Zool, Cambridge CB2 3EJ, England.; Dantzer, B (autor correspondente), Kuruman River Reserve, Kalahari Meerkat Project, Northern Cape, South Africa.; Dantzer, B (autor correspondente), Univ Michigan, Dept Psychol, Ann Arbor, MI 48109 USA.; Dantzer, B (autor correspondente), Univ Michigan, Dept Ecol & Evolutionary Biol, Ann Arbor, MI 48109 USA.
EM dantzer@umich.edu
RI Goncalves, Ines/F-5859-2012; Cram, Dom/H-5974-2019; Duncan, Chris/S-5735-2019; Dantzer, Ben/E-9779-2011; Bennett, Nigel/E-4238-2010;
Ganswindt, Andre/G-9856-2014
OI Cram, Dominic/0000-0002-8790-8294; Dantzer, Ben/0000-0002-3058-265X; Bennett, Nigel/0000-0001-9748-2947; Ganswindt, Andre/0000-0002-1474-7602
FU National Environment Research Council [RG53472]; European Research Council [294494]; Swiss National Science Foundation [31003A_13676]; DST-NRF SARCHl chair of Mammal Behavioural Ecology and Physiology; University of Cambridge; University of Zurich
FX Research was supported by grants from National Environment Research Council (RG53472) and the European Research Council (294494) to T.H.C.-B. I.B.G. was funded by the Swiss National Science Foundation (grant no. 31003A_13676 to M. Manser). N.C.B. was supported by a DST-NRF
SARCHl chair of Mammal Behavioural Ecology and Physiology. The Kalahari
Meerkat Project (KMP) has also been financed by the University of Cambridge (T.H.C.-B.) and University of Zurich (M. Manser).
CR Agrawal AA, 1999, NATURE, V401, P60, DOI 10.1038/43425
[Anonymous], ANN REV ECOLOGY SYST
[Anonymous], DRYAD DIGITAL REPOSI
[Anonymous], 1998, MATERNAL EFFECTS ADA

Bates D., 2014, LME4 LINEAR MIXED EF, V17, P685, DOI DOI 10.18637/JSS.V067.I01
Bell MBV, 2014, NAT COMMUN, V5, DOI 10.1038/ncomms5499
Berghänel A, 2017, P NATL ACAD SCI USA, V114, pE10658, DOI 10.1073/pnas.1707152114
Bourke AFG, 1995, Social evolution in ants
Braga Goncalves I, 2016, PLOS ONE, V11, DOI 10.1371/journal.pone.0153161
Brotherton PNM, 2001, BEHAV ECOL, V12, P590, DOI 10.1093/beheco/12.5.590
Cant MA, 2001, P ROY SOC B-BIOL SCI, V268, P1959, DOI 10.1098/rspb.2001.1754
Carlson AA, 2006, P ROY SOC B-BIOL SCI, V273, P571, DOI 10.1098/rspb.2005.3087
Carlson AA, 2004, HORM BEHAV, V46, P141, DOI 10.1016/j.yhbeh.2004.01.009
Carter C. Sue, 1997, P231
Clutton-Brock TH, 2003, ANIM BEHAV, V66, P531, DOI 10.1006/anbe.2003.2209
Clutton-Brock TH, 1998, P ROY SOC B-BIOL SCI, V265, P185, DOI 10.1098/rspb.1998.0281
Clutton-Brock TH, 2000, P ROY SOC B-BIOL SCI, V267, P301, DOI 10.1098/rspb.2000.1000
Clutton-Brock TH, 2002, SCIENCE, V297, P253, DOI 10.1126/science.1071412
Clutton-Brock TH, 2001, ANIM BEHAV, V61, P705, DOI 10.1006/anbe.2000.1631
Clutton-Brock TH, 2001, SCIENCE, V293, P2446, DOI 10.1126/science.1061274
Clutton-Brock Tim, 2016, P294
Dantzer B, 2017, P ROY SOC B-BIOL SCI, V284, DOI 10.1098/rspb.2017.1248
Dantzer B, 2017, BEHAV ECOL, V28, P1131, DOI 10.1093/beheco/arx077
Dantzer B, 2013, SCIENCE, V340, P1215, DOI 10.1126/science.1235765
De Fraipont M, 2000, J ANIM ECOL, V69, P404, DOI 10.1046/j.1365-2656.2000.00405.x
Duckworth RA, 2015, SCIENCE, V347, P875, DOI 10.1126/science.1260154
English S, 2008, BIOL LETTERS, V4, P334, DOI 10.1098/rsbl.2008.0200
English S, 2014, J ANIM ECOL, V83, P332, DOI 10.1111/1365-2656.12149
English S, 2013, ECOL EVOL, V3, P4401, DOI 10.1002/ece3.820
Field J, 2006, NATURE, V441, P214, DOI 10.1038/nature04560
FOX J, 1992, J AM STAT ASSOC, V87, P178, DOI 10.2307/2290467
González-Forero M, 2013, AM NAT, V182, P439, DOI 10.1086/671932
Griffin AS, 2005, AM NAT, V166, P628, DOI 10.1086/491662
HAMILTON WD, 1964, J THEOR BIOL, V7, P1, DOI [10.1016/0022-5193(64)90038-4, 10.1016/0022-5193(64)90039-6]
Harris A, 2011, HORM BEHAV, V59, P279, DOI 10.1016/j.yhbeh.2010.06.007
Hatchwell BJ, 2000, ANIM BEHAV, V59, P1079, DOI 10.1006/anbe.2000.1394
Heistermann M, 2006, AM J PRIMATOL, V68, P257, DOI 10.1002/ajp.20222
HEISTERMANN M, 1995, AM J PRIMATOL, V37, P275, DOI 10.1002/ajp.1350370402
Hodge SJ, 2008, J ANIM ECOL, V77, P92, DOI 10.1111/j.1365-2656.2007.01318.x
Huchard E, 2016, NATURE, V533, P532, DOI 10.1038/nature17986

KIRKPATRICK M, 1989, EVOLUTION, V43, P485, DOI 10.1111/j.1558-5646.1989.tb04247.x
Kuijper B, 2018, EVOLUTION, V72, P220, DOI 10.1111/evo.13403
Kunc HP, 2007, AM NAT, V170, P617, DOI 10.1086/521233
Kutsukake N, 2006, BEHAV ECOL SOCIOBIOL, V59, P541, DOI 10.1007/s00265-005-0079-7
Lesnoff M., 2013, aods3: analysis of overdispersed data using S3 methods
Linksvayer TA, 2005, Q REV BIOL, V80, P317, DOI 10.1086/432266
Marshall DJ, 2007, OIKOS, V116, P1957, DOI 10.1111/j.2007.0030-1299.16203.x
Marshall HH, 2017, ECOL EVOL, V7, P1712, DOI 10.1002/ece3.2747
Metzger DCH, 2016, P ROY SOC B-BIOL SCI, V283, DOI 10.1098/rspb.2016.1734
MICHENER CD, 1974, P NATL ACAD SCI USA, V71, P671, DOI 10.1073/pnas.71.3.671
Mueller BR, 2008, J NEUROSCI, V28, P9055, DOI 10.1523/JNEUROSCI.1424-08.2008
Müller W, 2007, AM NAT, V169, pE84, DOI 10.1086/511962
R Core Team R, 2013, R LANG ENV STAT COMP, DOI [10.1007/978-3-540-74686-7, DOI 10.1007/978-3-540-74686-7]
Raulo A, 2018, ECOL EVOL, V8, P7697, DOI 10.1002/ece3.4059
Russell AF, 2007, P R SOC B, V274, P513, DOI 10.1098/rspb.2006.3698
Russell AF, 2003, P NATL ACAD SCI USA, V100, P3333, DOI 10.1073/pnas.0636503100
Russell AF, 2002, J ANIM ECOL, V71, P700, DOI 10.1046/j.1365-2656.2002.00636.x
Russell AF, 2009, PHILOS T R SOC B, V364, P1143, DOI 10.1098/rstb.2008.0298
Schwarzkopf L, 2012, HERPETOLOGICA, V68, P147
Sheriff MJ, 2017, INTEGR COMP BIOL, V57, P437, DOI 10.1093/icb/icx105
Taborsky B, 2017, ADV STUD BEHAV, V49, P49, DOI 10.1016/bs.asb.2016.12.002
Thornton A, 2008, ANIM BEHAV, V76, P1411, DOI 10.1016/j.anbehav.2008.07.007
TRIVERS RL, 1971, Q REV BIOL, V46, P35, DOI 10.1086/406755
Uller T, 2013, J EVOLUTION BIOL, V26, P2161, DOI 10.1111/jeb.12212
Uller T, 2008, TRENDS ECOL EVOL, V23, P432, DOI 10.1016/j.tree.2008.04.005
Uller T, 2011, EVOLUTION, V65, P2075, DOI 10.1111/j.1558-5646.2011.01282.x
Wade MJ, 2001, EVOLUTION, V55, P453, DOI 10.1554/0014-3820(2001)055[0453:MEGATE]2.0.CO;2
Weinstock M, 2007, NEUROCHEM RES, V32, P1730, DOI 10.1007/s11064-007-9339-4
Wilkin TA, 2009, CURR BIOL, V19, P1998, DOI 10.1016/j.cub.2009.09.065
Wilson AJ, 2005, EVOLUTION, V59, P451
Wolf JB, 2009, PHILOS T R SOC B, V364, P1107, DOI 10.1098/rstb.2008.0238
Zöttl M, 2013, BIOL LETTERS, V9, DOI 10.1098/rsbl.2012.0878
Zuur AF, 2010, METHODS ECOL EVOL, V1, P3, DOI 10.1111/j.2041-210X.2009.00001.x
NR 73
TC 16

Z9 17
U1 6
U2 42
PU ROYAL SOC
PI LONDON
PA 6-9 CARLTON HOUSE TERRACE, LONDON SW1Y 5AG, ENGLAND
SN 0962-8436
EI 1471-2970
J9 PHILOS T R SOC B
JI Philos. Trans. R. Soc. B-Biol. Sci.
PD APR 15
PY 2019
VL 374
IS 1770
AR 20180117
DI 10.1098/rstb.2018.0117
PG 16
WC Biology
WE Science Citation Index Expanded (SCI-EXPANDED)
SC Life Sciences & Biomedicine - Other Topics
GA HN8ZI
UT WOS:000460487000008
PM 30966876
OA Bronze, Green Published, Green Submitted
DA 2025-05-07
ER

PT J
AU Aktipis, A
AF Aktipis, Athena
TI Life history theory and breast cancer risk: methodological and
theoretical challenges Response to "Is estrogen receptor negative breast
cancer risk associated with a fast life history strategy?"
SO EVOLUTION MEDICINE AND PUBLIC HEALTH
LA English
DT Editorial Material
AB In a meta-analysis published by myself and co-authors, we report differences in the life history risk factors for estrogen receptor negative (ER-) and estrogen receptor positive (ER+) breast cancers. Our meta-analysis did not find the association of ER- breast cancer risk with fast life history characteristics that Hidaka and Boddy suggest in their response to our article. There are a number of possible explanations for the differences between their conclusions and the conclusions we drew from our meta-analysis, including limitations of our meta-analysis and methodological challenges in measuring and categorizing estrogen receptor status. These challenges, along with the association of ER+ breast cancer with slow life history characteristics, may make it challenging to find a clear signal of ER- breast cancer with fast life history characteristics, even if that relationship does exist. The contradictory results regarding breast cancer risk and life history characteristics illustrate a more general challenge in evolutionary medicine: often different sub-theories in evolutionary biology make contradictory predictions about disease risk. In this case, life history models predict that breast cancer risk

should increase with faster life history characteristics, while the
evolutionary mismatch hypothesis predicts that breast cancer risk should
increase with delayed reproduction. Whether life history tradeoffs
contribute to ER- breast cancer is still an open question, but current
models and several lines of evidence suggest that it is a possibility.
C1 [Aktipis, Athena] Arizona State Univ, Dept Psychol, Ctr Evolut & Med,
Ctr Social Dynam & Complex, Tempe, AZ 85287 USA.
C3 Arizona State University; Arizona State University-Tempe
RP Aktipis, A (autor correspondente), Arizona State Univ, Dept Psychol,
Ctr Evolut & Med, Ctr Social Dynam & Complex, Tempe, AZ 85287 USA.
EM aktipis@asu.edu
CR Archey WB, 2002, ONCOGENE, V21, P7034, DOI 10.1038/sj.onc.1205844
   Badve SS, 2008, J CLIN ONCOL, V26, P2473, DOI 10.1200/JCO.2007.13.6424
   Bauer KR, 2007, CANCER, V109, P1721, DOI 10.1002/cncr.22618
   Boddy AM, 2015, BIOESSAYS, V15, P21
   Boddy AM, 2015, PHILOS T R SOC B, V370, DOI 10.1098/rstb.2014.0220
   Dhimolea E, 2013, INT J CANCER, V133, P835, DOI 10.1002/ijc.28077
   Harbeck N, 2012, J CLIN ONCOL, V30, P686, DOI 10.1200/JCO.2011.38.9619
   Hidaka Brandon H., 2016, EVOLUTION MED PUBLIC, V2016, P17
   Rhodes A, 2000, J CLIN PATHOL, V53, P292, DOI 10.1136/jcp.53.4.292
NR 9
TC 4
Z9 4
U1 0
U2 4
PU OXFORD UNIV PRESS
PI OXFORD
PA GREAT CLARENDON ST, OXFORD OX2 6DP, ENGLAND
SN 2050-6201
J9 EVOL MED PUBLIC HLTH
JI Evol. Med. Public Health.
PY 2016
IS 1
BP 177
EP 179
DI 10.1093/emph/eov035
PG 3
WC Evolutionary Biology; Public, Environmental & Occupational Health
WE Science Citation Index Expanded (SCI-EXPANDED)
SC Evolutionary Biology; Public, Environmental & Occupational Health
GA DZ5TY
UT WOS:000385925600016
PM 26874356
OA Green Published, Green Submitted, gold
DA 2025-05-07
ER

PT J
AU Adams, DR
   Barbarin, AM
   Reiskind, MH
AF Adams, Dayvion R.
   Barbarin, Alexis M.
   Reiskind, Michael H.

TI New report of Haemaphysalis longicornis (Ixodida: Ixodidae) in
   Mecklenburg County, Virginia from field collections
SO JOURNAL OF MEDICAL ENTOMOLOGY
LA English
DT Article
DE hard tick; invasive species; distribution; range expansion; vector
   ecology
ID THEILERIA-ORIENTALIS; ACARI IXODIDAE; PICTORIAL KEY; TICKS;
ASSOCIATION;
   ADULTS
AB Haemaphysalis longicornis (Neumann) was first established in New Jersey and has rapidly spread across most of the eastern United States. This tick has the potential to infest a wide variety of hosts and can reproduce quickly via parthenogenesis, presenting a new threat to animal health. Here we report the first record of a single H. longicornis tick in Mecklenburg County, Virginia, from incidental field collections of ticks. In addition to H. longicornis, we collected 787 Amblyomma americanum, 25 Dermacentor variabilis, 6 Ixodes affinis, 1 Haemaphysalis leporispalustris, and 1 Amblyomma maculatum using standard dragging and flagging techniques. The expansion of H. longicornis will have economic consequences for livestock producers in south-central Virginia, who must now manage this species. Enhanced surveillance is needed to fully understand its growing geographic distribution in the United States and the subsequent consequences of its spread.
C1 [Adams, Dayvion R.; Reiskind, Michael H.] North Carolina State Univ, Dept Entomol & Plant Pathol, Raleigh, NC 27695 USA.
   [Barbarin, Alexis M.] North Carolina Dept Hlth & Human Serv, Div Publ Hlth, Communicable Dis Branch, Raleigh, NC 27699 USA.
C3 North Carolina State University
RP Reiskind, MH (autor correspondente), North Carolina State Univ, Dept Entomol & Plant Pathol, Raleigh, NC 27695 USA.
EM mhreiski@ncsu.edu
RI Reiskind, Michael/I-5614-2012
OI Adams, Dayvion/0000-0001-7690-3650; Reiskind,
   Michael/0000-0001-7307-9416
FU United States Department of Agriculture Multistate Project [NE-1943];
   Triangle Center of Evolutionary Medicine (TriCEM)
FX This work was supported by the United States Department of Agriculture
   Multistate Project NE-1943, "Biology, Ecology & Management of Emerging
   Disease Vectors." This work was also funded by a graduate student
   research grant from the Triangle Center of Evolutionary Medicine
   (TriCEM).
CR [Anonymous], 2020, Guide to the Surveillance of Metastriate Ticks (Acari: Ixodidae) and their Pathogens in the United States
   Aziati ID, 2023, TICKS TICK-BORNE DIS, V14, DOI 10.1016/j.ttbdis.2022.102080
   Ben Beard C, 2018, MMWR-MORBID MORTAL W, V67, P1310, DOI 10.15585/mmwr.mm6747a3
   Bickerton M, 2020, INT J ACAROL, V46, P373, DOI 10.1080/01647954.2020.1805004
   Butler RA, 2023, J MED ENTOMOL, V60, P1374, DOI 10.1093/jme/tjad121
   Clifford C. M., 1961, Miscellaneous Publications of the Entomological Society of America, V2, P213
   Cumbie AN, 2022, J MED ENTOMOL, V59, P1443, DOI 10.1093/jme/tjac067

Dinkel KD, 2021, PARASITE VECTOR, V14, DOI 10.1186/s13071-021-04659-9
Dubie TR, 2017, SOUTHWEST ENTOMOL, V42, P1, DOI 10.3958/059.042.0101
Durden LA., 1996, NYMPHS GENUS IXODES, DOI 10.4182/MAMR9602.1996.5
Eamens GJ, 2013, VET PARASITOL, V191, P209, DOI 10.1016/j.vetpar.2012.09.007
Egizi A, 2020, ZOONOSES PUBLIC HLTH, V67, P637, DOI 10.1111/zph.12743
Egizi AM, 2019, ZOOKEYS, P117, DOI 10.3897/zookeys.818.30448
Eleftheriou A, 2023, J MED ENTOMOL, V60, P1126, DOI 10.1093/jme/tjad104
Ferreira FC, 2023, INT J PARASITOL, V53, P809, DOI 10.1016/j.ijpara.2023.06.003
Foley N, 2023, J MED ENTOMOL, V60, P708, DOI 10.1093/jme/tjad039
Fryxell RTT, 2023, PLOS ONE, V18, DOI 10.1371/journal.pone.0292595
Fryxell RTT, 2021, INT J ENV RES PUB HE, V18, DOI 10.3390/ijerph18157980
KEIRANS JE, 1989, J MED ENTOMOL, V26, P435, DOI 10.1093/jmedent/26.5.435
Li ZF, 2016, PLOS NEGLECT TROP D, V10, DOI 10.1371/journal.pntd.0004574
Maestas LP, 2020, NORTHEAST NAT, V27, pN6, DOI 10.1656/045.027.0106
Nadolny RM, 2021, J MED ENTOMOL, V58, P1941, DOI 10.1093/jme/tjab058
Orr JM, 2013, SYST APPL ACAROL-UK, V18, P105, DOI 10.11158/saa.18.2.1
Price KJ, 2021, J MED ENTOMOL, V58, P1433, DOI 10.1093/jme/tjaa274
Raghavan RK, 2019, PLOS ONE, V14, DOI 10.1371/journal.pone.0209082
Rainey T, 2018, J MED ENTOMOL, V55, P757, DOI 10.1093/jme/tjy006
Raney WR, 2022, FRONT CELL INFECT MI, V12, DOI 10.3389/fcimb.2022.923914
Raney WR, 2022, EMERG INFECT DIS, V28, P726, DOI 10.3201/eid2803.210973
Rochlin I, 2023, TICKS TICK-BORNE DIS, V14, DOI 10.1016/j.ttbdis.2022.102088
Rochlin I, 2019, J MED ENTOMOL, V56, P384, DOI 10.1093/jme/tjy210
Romer Y, 2022, EMERG INFECT DIS, V28, P786, DOI 10.3201/eid2804.211540
Stanley HM, 2020, J MED ENTOMOL, V57, P1635, DOI 10.1093/jme/tjaa076
Thompson AT, 2021, TICKS TICK-BORNE DIS, V12, DOI 10.1016/j.ttbdis.2021.101773
Thompson AT, 2020, TICKS TICK-BORNE DIS, V11, DOI 10.1016/j.ttbdis.2020.101450
Tufts DM, 2021, PARASITE VECTOR, V14, DOI 10.1186/s13071-021-04887-z
Tufts DM, 2021, INT J PARASITOL, V51, P149, DOI 10.1016/j.ijpara.2020.08.008
USDA, 2023, NATL HAEMAPHYSALIS L, P15
Watts JG, 2016, NEW ZEAL VET J, V64, P3, DOI 10.1080/00480169.2015.1064792
Wormser GP, 2020, CLIN INFECT DIS, V70, P314, DOI 10.1093/cid/ciz449
Zhuang L, 2018, EMERG INFECT DIS, V24, P868, DOI 10.3201/eid2405.151435
NR 40
TC 1
Z9 1
U1 3
U2 5
PU OXFORD UNIV PRESS INC
PI CARY

PA JOURNALS DEPT, 2001 EVANS RD, CARY, NC 27513 USA
SN 0022-2585
EI 1938-2928
J9 J MED ENTOMOL
JI J. Med. Entomol.
PD JUL 17
PY 2024
VL 61
IS 5
BP 1261
EP 1265
DI 10.1093/jme/tjae090
EA JUL 2024
PG 5
WC Entomology; Veterinary Sciences
WE Science Citation Index Expanded (SCI-EXPANDED)
SC Entomology; Veterinary Sciences
GA F3K8U
UT WOS:001270381900001
PM 39021151
DA 2025-05-07
ER

PT J
AU Wells, JCK
AF Wells, Jonathan C. K.
TI Developmental plasticity as adaptation: adjusting to the external
   environment under the imprint of maternal capital
SO PHILOSOPHICAL TRANSACTIONS OF THE ROYAL SOCIETY B-BIOLOGICAL SCIENCES
LA English
DT Article
DE developmental plasticity; adaptation; parental investment;
   parent-offspring conflict; life-history theory
ID THRIFTY PHENOTYPE; LIFE-HISTORY; BODY-COMPOSITION; EARLY GROWTH;
   ORIGINS; BIRTH; EXPOSURE; FAMINE; HEALTH; CONSEQUENCES
AB Plasticity is assumed to enable beneficial adjustment to the environment. In this context, developmental plasticity is generally approached within a two-stage framework, whereby adjustments to ecological cues in stage 1 are exposed to selection in stage 2. This conceptual approach may have limitations, because in species providing parental investment, particularly placental mammals such as humans, initial adjustments are not to the environment directly, but rather to the niche generated by parental phenotype (in mammals, primarily that of the mother). Only as maternal investment is withdrawn is the developing organism exposed directly to prevailing ecological conditions. A three-stage model may therefore be preferable, where developmental trajectory first adjusts to maternal investment, then to the external environment. Each offspring experiences a trade-off, benefitting from maternal investment during the most vulnerable stages of development, at the cost of exposure to investment strategies that maximize maternal fitness. Maternal life-history trade-offs impact the magnitude and schedule of her investment in her offspring, generating lifelong effects on traits related to health outcomes. Understanding the imprint of maternal capital on offspring is particularly important in species demonstrating social

hierarchy. Interventions targeting maternal capital might offer new opportunities to improve health outcomes of both mother and offspring.
This article is part of the theme issue 'Developing differences: early-life effects and evolutionary medicine'.
C1 [Wells, Jonathan C. K.] UCL, Great Ormond St Inst Child Hlth, Childhood Nutr Res Ctr, 30 Guilford St, London WC1N 1EH, England.
C3 University of London; University College London
RP Wells, JCK (autor correspondente), UCL, Great Ormond St Inst Child Hlth, Childhood Nutr Res Ctr, 30 Guilford St, London WC1N 1EH, England.
EM jonathan.wells@ucl.ac.uk
RI Wells, Jonathan/A-4604-2009
OI Wells, Jonathan/0000-0003-0411-8025
CR Alpert P, 2002, EVOL ECOL, V16, P285, DOI 10.1023/A:1019684612767
[Anonymous], 2011, Plasticity, Robustness, Development and Evolution
[Anonymous], 2018, LANCET, DOI DOI 10.1016/S0140-6736(18)30312-X
[Anonymous], 1992, EVOLUTION LIFE HIST
[Anonymous], MATERN CHILD NUTR
Barker DJP, 2004, ACTA PAEDIATR, V93, P26, DOI 10.1080/08035320410022730
Bateson P, 2004, NATURE, V430, P419, DOI 10.1038/nature02725
Bedu-Addo G, 2017, J INFECTION, V75, P455, DOI 10.1016/j.jinf.2017.08.010
Bernstein RM, 2016, AM J PRIMATOL, V78, P838, DOI 10.1002/ajp.22544
Bogin B, 2007, AM J HUM BIOL, V19, P631, DOI 10.1002/ajhb.20666
Carrapato MRG, 2003, J PERINAT MED, V31, P5, DOI 10.1515/JPM.2003.001
Cnattingius S, 2012, INT J OBESITY, V36, P1320, DOI 10.1038/ijo.2011.248
Dabelea D, 2000, DIABETES, V49, P2208, DOI 10.2337/diabetes.49.12.2208
Dawkins R., 1982, pi
Del Giudice M, 2011, NEUROSCI BIOBEHAV R, V35, P1562, DOI 10.1016/j.neubiorev.2010.11.007
Dominguez-Salas P, 2014, NAT COMMUN, V5, DOI 10.1038/ncomms4746
Forrester TE, 2012, PLOS ONE, V7, DOI 10.1371/journal.pone.0035907
Gibson MA, 2006, PLOS MED, V3, P476, DOI 10.1371/journal.pmed.0030087
Gluckman PD, 2004, PEDIATR RES, V56, P311, DOI 10.1203/01.PDR.0000135998.08025.FB
Gluckman PD, 2007, AM J HUM BIOL, V19, P1, DOI 10.1002/ajhb.20590
Godfrey KM, 2011, DIABETES, V60, P1528, DOI 10.2337/db10-0979
HAIG D, 1993, Q REV BIOL, V68, P495, DOI 10.1086/418300
HALES CN, 1992, DIABETOLOGIA, V35, P595, DOI 10.1093/ije/dyt133
Hayward AD, 2013, P NATL ACAD SCI USA, V110, P13886, DOI 10.1073/pnas.1301817110
Hinde CA, 2009, P ROY SOC B-BIOL SCI, V276, P2787, DOI 10.1098/rspb.2009.0375
Hrdy S.B., 2009, Mothers and others: The evolutionary origins of mutual understanding
Inzani EL, 2016, SCI REP-UK, V6, DOI 10.1038/srep20013
Jones JH, 2005, AM J HUM BIOL, V17, P22, DOI 10.1002/ajhb.20099
Jonsson KI, 1997, OIKOS, V78, P57, DOI 10.2307/3545800
Kaplan H, 2003, LIFE SPAN: EVOLUTIONARY, ECOLOGICAL, AND DEMOGRAPHIC PERSPECTIVES, P152
Kuijper B, 2018, EVOLUTION, V72, P220, DOI 10.1111/evo.13403
Kuzawa CW, 2005, AM J HUM BIOL, V17, P5, DOI 10.1002/ajhb.20091
Lacey EP, 1998, MATERNAL EFFECTS AS ADAPTATIONS, P54

Lea AJ, 2017, EVOL MED PUBLIC HLTH, P162, DOI 10.1093/emph/eox019
Lindström J, 1999, TRENDS ECOL EVOL, V14, P343, DOI 10.1016/S0169-5347(99)01639-0
Love OP, 2008, AM NAT, V172, pE135, DOI 10.1086/590959
Lumey LH, 2015, LANCET DIABETES ENDO, V3, P787, DOI 10.1016/S2213-8587(15)00279-X
Marphatia AA, 2016, GLOB HEALTH EPIDEM G, V1, DOI 10.1017/gheg.2016.1
Marshall DJ, 2007, OIKOS, V116, P1957, DOI 10.1111/j.2007.0030-1299.16203.x
McMillen IC, 2005, PHYSIOL REV, V85, P571, DOI 10.1152/physrev.00053.2003
Monaghan P, 2008, PHILOS T R SOC B, V363, P1635, DOI 10.1098/rstb.2007.0011
Mousseau TA, 1998, TRENDS ECOL EVOL, V13, P403, DOI 10.1016/S0169-5347(98)01472-4
Nettle D, 2015, P ROY SOC B-BIOL SCI, V282, P23, DOI 10.1098/rspb.2015.1005
Nettle D, 2013, P ROY SOC B-BIOL SCI, V280, DOI 10.1098/rspb.2013.1343
Odling-Smee F.J. L., 2003, NICHE CONSTRUCTION
Özaltin E, 2010, JAMA-J AM MED ASSOC, V303, P1507, DOI 10.1001/jama.2010.450
Pittet F, 2017, AM J PHYS ANTHROPOL, V164, P457, DOI 10.1002/ajpa.23286
Rayco-Solon P, 2005, AM J CLIN NUTR, V81, P134
SHINE R, 1978, J THEOR BIOL, V75, P417, DOI 10.1016/0022-5193(78)90353-3
Siervo M, 2010, PLOS ONE, V5, DOI 10.1371/journal.pone.0013907
Smith J, 2009, J CLIN ENDOCR METAB, V94, P4275, DOI 10.1210/jc.2009-0709
Stein AD, 2004, INT J EPIDEMIOL, V33, P831, DOI 10.1093/ije/dyh083
Stein Z., 1975, FAMINE HUMAN DEV DUT
TRIVERS RL, 1974, AM ZOOL, V14, P249
VIEGAS OAC, 1982, BMJ-BRIT MED J, V285, P592, DOI 10.1136/bmj.285.6342.592
Wells JCK, 2016, METABOLIC GHETTO: AN EVOLUTIONARY PERSPECTIVE ON NUTRITION, POWER RELATIONS AND CHRONIC DISEASE
Wells JCK, 2017, EUR J CLIN NUTR, V71, P881, DOI 10.1038/ejcn.2017.31
Wells JCK, 2003, J THEOR BIOL, V221, P143, DOI 10.1006/jtbi.2003.3183
Wells JCK., 2017, The Arc of Life: Evolution and Health Across the Life Course, P21
Wells JCK, 2007, TRENDS ENDOCRIN MET, V18, P331, DOI 10.1016/j.tem.2007.07.006
Wells JCK, 2007, BIOL REV, V82, P143, DOI 10.1111/j.1469-185X.2006.00007.x
Wells JCK, 2018, EVOL MED PUBLIC HLTH, P153, DOI 10.1093/emph/eoy014
Wells JCK, 2017, EVOL MED PUBLIC HLTH, P185, DOI 10.1093/emph/eox023
Wells JCK, 2017, LANCET, V390, P500, DOI 10.1016/S0140-6736(17)30572-X
Wells JCK, 2016, EVOL MED PUBLIC HLTH, P133, DOI 10.1093/emph/eow011
Wells Jonathan C. K., 2014, Evolution Medicine and Public Health, P109, DOI 10.1093/emph/eou019
Wells JCK, 2012, AM J HUM BIOL, V24, P261, DOI 10.1002/ajhb.22253
Wells JCK, 2011, AM J EPIDEMIOL, V174, P1028, DOI 10.1093/aje/kwr232
Wells JCK, 2011, AM J HUM BIOL, V23, P65, DOI 10.1002/ajhb.21100
Wells JCK, 2010, AM J HUM BIOL, V22, P1, DOI 10.1002/ajhb.20994

NR 70
TC 26
Z9 30
U1 0
U2 17
PU ROYAL SOC
PI LONDON
PA 6-9 CARLTON HOUSE TERRACE, LONDON SW1Y 5AG, ENGLAND
SN 0962-8436
EI 1471-2970
J9 PHILOS T R SOC B
JI Philos. Trans. R. Soc. B-Biol. Sci.
PD APR 15
PY 2019
VL 374
IS 1770
AR 20180122
DI 10.1098/rstb.2018.0122
PG 8
WC Biology
WE Science Citation Index Expanded (SCI-EXPANDED)
SC Life Sciences & Biomedicine - Other Topics
GA HN8ZI
UT WOS:000460487000013
PM 30966888
OA Green Published, Bronze, Green Submitted
DA 2025-05-07
ER

PT J
AU Lei, YT
   Anders, HJ
AF Lei, Yutian
   Anders, Hans-Joachim
TI Evolutionary trade-offs in kidney injury and repair
SO HISTOLOGY AND HISTOPATHOLOGY
LA English
DT Review
DE Regeneration; Fibrosis; Coagulation; Inflammation; Healing; Repair
ID TOLL-LIKE RECEPTORS; PARIETAL EPITHELIAL-CELLS; IMMUNE-COMPLEX
   GLOMERULONEPHRITIS; GLOMERULAR ENDOTHELIAL-CELLS;
   SYSTEMIC-LUPUS-ERYTHEMATOSUS; URINARY-TRACT-INFECTIONS; MARROW-DERIVED
   CELLS; ACUTE-RENAL-FAILURE; INNATE IMMUNITY; TISSUE-INJURY
AB Evolutionary medicine has proven helpful to understand the origin of human disease, e. g. in identifying causal roles of recent environmental changes impacting on human physiology (environmentphenotype mismatch). In contrast, diseases affecting only a limited number of members of a species often originate from evolutionary trade-offs for usually physiologic adaptations assuring reproductive success in the context of extrinsic threats. For example, the G1 and G2 variants of the APOL1 gene supporting control of Trypanosoma infection come with the trade-off that they promote the progression of kidney disease. In this review we extend the concept of evolutionary nephrology by discussing how the physiologic adaptations (danger responses) to tissue injury create evolutionary

trade-offs that drive histopathological changes underlying acute and chronic kidney diseases. The evolution of multicellular organisms positively selected a number of danger response programs for their overwhelming benefits in assuring survival such as clotting, inflammation, epithelial healing and mesenchymal healing, i. e. fibrosis and sclerosis. Upon kidney injury these danger programs often present as pathomechanisms driving persistent nephron loss and renal failure. We explore how classic kidney disease entities involve insufficient or overshooting activation of these danger response programs for which the underlying genetic basis remains largely to be defined. Dissecting the causative and hierarchical relationships between danger programs should help to identify molecular targets to control kidney injury and to improve disease outcomes.
C1 [Lei, Yutian; Anders, Hans-Joachim] Ludwig Maximilians Univ Munchen, Nephrol Zentrum, Med Klin & Poliklin 4, Munich, Germany.
C3 University of Munich
RP Anders, HJ (autor correspondente), Klinikum Univ Munchen, Med Klin & Poliklin 4, Ziemssenstr 1, D-80336 Munich, Germany.
EM hjanders@med.uni-muenchen.de
RI Anders, Hans-Joachim/ABD-6247-2021
OI Anders, Hans-Joachim/0000-0003-2434-2956
FU Deutsche Forschungsgemeinschaft [AN372/11-2, AN372/14-3, AN372/17-1, AN372/20-1, AN372/23-1, AN372/24-1]; European Union [305736, 634086, 668036]; Chinese Scholarship Council; Academy of Finland (AKA) [305736]
Funding Source: Academy of Finland (AKA)
FX H.J.A was supported by the Deutsche Forschungsgemeinschaft (AN372/11-2; 14-3, 17-1, 20-1, 23-1, 24-1) and by European Union's research and innovation programmes (under grant agreements 7. FP, REDDSTAR No. 305736 and Horizon 2020, NEPHSTROM No. 634086 and RELENT No. 668036). The views expressed here are the responsibility of the authors only. The EU Commission takes no responsibility for any use made of the information set out. Y.L. was supported by the Chinese Scholarship Council.
CR Abais JM, 2014, J BIOL CHEM, V289, P27159, DOI 10.1074/jbc.M114.567537
Abraham SN, 2015, NAT REV IMMUNOL, V15, P655, DOI 10.1038/nri3887
Ahronowitz I., 2012, AM J CLIN DERMATOL
Alikhan MA, 2011, AM J PATHOL, V179, P1243, DOI 10.1016/j.ajpath.2011.05.037
Allam R, 2008, CURR OPIN RHEUMATOL, V20, P538, DOI 10.1097/BOR.0b013e3283025ed4
Allam R, 2014, J MOL MED, V92, P465, DOI 10.1007/s00109-014-1148-z
Allam R, 2013, EUR J IMMUNOL, V43, P3336, DOI 10.1002/eji.201243224
Allam R, 2012, J AM SOC NEPHROL, V23, P1375, DOI 10.1681/ASN.2011111077
Allam R, 2009, J AM SOC NEPHROL, V20, P1986, DOI 10.1681/ASN.2008101067
Allam R, 2008, EUR J IMMUNOL, V38, P3487, DOI 10.1002/eji.200838604
Anders HJ, 2007, KIDNEY INT, V72, P1051, DOI 10.1038/sj.ki.5002436
Anders HJ, 2007, CURR OPIN NEPHROL HY, V16, P177, DOI 10.1097/MNH.0b013e32803fb767

Anders HJ, 2016, J AM SOC NEPHROL, V27, P2564, DOI 10.1681/ASN.2016020177
Anders HJ, 2014, J AM SOC NEPHROL, V25, P1387, DOI 10.1681/ASN.2014010117
Anders HJ, 2014, NAT REV NEPHROL, V10, P347, DOI 10.1038/nrneph.2014.68
Anders HJ, 2012, ORGANOGENESIS, V8, P29, DOI 10.4161/org.20342
Anders HJ, 2011, KIDNEY INT, V80, P915, DOI 10.1038/ki.2011.217
Anders HJ, 2011, J AM SOC NEPHROL, V22, P1007, DOI 10.1681/ASN.2010080798
Anders HJ, 2010, J AM SOC NEPHROL, V21, P1270, DOI 10.1681/ASN.2010030233
Anders HJ, 2010, KIDNEY INT, V77, P848, DOI 10.1038/ki.2010.71
Anders HJ, 2009, TRENDS MOL MED, V15, P553, DOI 10.1016/j.molmed.2009.10.004
Anders HJ, 2006, KIDNEY INT, V69, P29, DOI 10.1038/sj.ki.5000053
Anders HJ, 2005, NEPHROL DIAL TRANSPL, V20, P1529, DOI 10.1093/ndt/gfh922
Anders HJ, 2004, FASEB J, V18, P534, DOI 10.1096/fj.03-0646fje
Anders HJ, 2004, J AM SOC NEPHROL, V15, P854, DOI 10.1097/01.ASN.0000121781.89599.16
Anders HJ, 2003, KIDNEY INT, V63, P401, DOI 10.1046/j.1523-1755.2003.00750.x
Anders HJ, 2003, J AM SOC NEPHROL, V14, P317, DOI 10.1097/01.ASN.0000042169.23931.73
Anders HJ, 2002, J CLIN INVEST, V109, P251, DOI 10.1172/JCI14040
Andersen K, 2014, KIDNEY INT, V86, P965, DOI 10.1038/ki.2014.161
Angelotti ML, 2012, STEM CELLS, V30, P1714, DOI 10.1002/stem.1130
Babel N, 2011, NAT REV NEPHROL, V7, P399, DOI 10.1038/nrneph.2011.59
Behzadi E, 2016, CENT EUR J UROL, V69, P404, DOI 10.5173/ceju.2016.871
Böger CA, 2011, PLOS GENET, V7, DOI 10.1371/journal.pgen.1002292
BOHLE A, 1992, PATHOL RES PRACT, V188, P908, DOI 10.1016/S0344-0338(11)80252-9
Bollée G, 2011, NAT MED, V17, P1242, DOI 10.1038/nm.2491
Bomback AS, 2012, NAT REV NEPHROL, V8, P634, DOI 10.1038/nrneph.2012.213
Bonventre JV, 2003, J AM SOC NEPHROL, V14, pS55, DOI 10.1097/01.ASN.0000067652.51441.21
Brähler S, 2012, AM J PHYSIOL-RENAL, V303, pF1473, DOI 10.1152/ajprenal.00031.2012
Braga TT, 2012, MOL MED, V18, P1231, DOI 10.2119/molmed.2012.00131
Braun RK, 2008, INFLAMMATION, V31, P167, DOI 10.1007/s10753-008-9062-6
Brinkmann V, 2004, SCIENCE, V303, P1532, DOI 10.1126/science.1092385
Buchmann K, 2014, FRONT IMMUNOL, V5, DOI 10.3389/fimmu.2014.00459
Bussone G, 2011, CURR RHEUMATOL REP, V13, P37, DOI 10.1007/s11926-010-0145-7
Cao Q, 2015, PHYSIOLOGY, V30, P183, DOI 10.1152/physiol.00046.2014
Chan JK, 2012, J CLIN INVEST, V122, P2711, DOI 10.1172/JCI62423
Chapman K, 2012, SEMIN THROMB HEMOST, V38, P47, DOI 10.1055/s-0031-1300951
Chen GC, 2012, PLOS ONE, V7, DOI 10.1371/journal.pone.0033626
Chen L, 2013, J INVEST DERMATOL, V133, P258, DOI 10.1038/jid.2012.267
Chevalier RL, 2017, KIDNEY INT REP, V2, P302, DOI 10.1016/j.ekir.2017.01.012

Clauss S, 2009, J PATHOL, V218, P40, DOI 10.1002/path.2505
Damman J, 2015, TRANSPLANTATION, V99, P1293, DOI 10.1097/TP.0000000000000500
Daniel C, 2008, LAB INVEST, V88, P387, DOI 10.1038/labinvest.2008.5
Darisipudi MN, 2011, AM J PATHOL, V179, P116, DOI 10.1016/j.ajpath.2011.03.004
Darisipudi MN, 2012, J AM SOC NEPHROL, V23, P1783, DOI 10.1681/ASN.2012040338
Delvaeye M, 2009, BLOOD, V114, P2367, DOI 10.1182/blood-2009-05-199208
Duffield JS, 2005, J CLIN INVEST, V115, P1743, DOI 10.1172/JCI22593
Duffield JS, 2005, J CLIN INVEST, V115, P56, DOI 10.1172/JCI200522675
Ebihara N, 2011, INVEST OPHTH VIS SCI, V52, P8549, DOI 10.1167/iovs.11-7956
Ebrahim M, 2015, HISTOL HISTOPATHOL, V30, P1271, DOI 10.14670/HH-11-636
Eitner F, 1997, KIDNEY INT, V51, P69, DOI 10.1038/ki.1997.9
Engelmann B, 2013, NAT REV IMMUNOL, V13, P34, DOI 10.1038/nri3345
Falke LL, 2015, NAT REV NEPHROL, V11, P233, DOI 10.1038/nrneph.2014.246
Flür K, 2009, AM J PATHOL, V175, P2014, DOI 10.2353/ajpath.2009.080585
Fukuda A, 2012, J AM SOC NEPHROL, V23, P1351, DOI 10.1681/ASN.2012030271
Furuichi K, 2006, AM J PATHOL, V169, P372, DOI 10.2353/ajpath.2006.060043
Gaikwad AB, 2010, AM J PATHOL, V176, P1079, DOI 10.2353/ajpath.2010.090528
Garimella PS, 2017, CURR OPIN NEPHROL HY, V26, P136, DOI 10.1097/MNH.0000000000000299
Godaly G, 2015, CURR OPIN INFECT DIS, V28, P88, DOI 10.1097/QCO.0000000000000127
Günthner R, 2013, INT J MOL SCI, V14, P18124, DOI 10.3390/ijms140918124
Gurtner GC, 2008, NATURE, V453, P314, DOI 10.1038/nature07039
Gutiérrez E, 2012, NEPHRON CLIN PRACT, V121, pC42, DOI 10.1159/000342385
Hägele H, 2009, AM J PATHOL, V175, P1896, DOI 10.2353/ajpath.2009.090182
Hägele H, 2009, NEPHROL DIAL TRANSPL, V24, P3312, DOI 10.1093/ndt/gfp339
Hagemann JH, 2013, INT J MOL SCI, V14, P11319, DOI 10.3390/ijms140611319
Hagen M, 2016, CELL CYCLE, V15, P1929, DOI 10.1080/15384101.2016.1191710
Helal I, 2012, NAT REV NEPHROL, V8, P293, DOI 10.1038/nrneph.2012.19
Hickey MJ, 2009, NAT REV IMMUNOL, V9, P364, DOI 10.1038/nri2532
Hodgin JB, 2015, J AM SOC NEPHROL, V26, P3162, DOI 10.1681/ASN.2014080752
Hotchkiss RS, 2009, NAT MED, V15, P496, DOI 10.1038/nm0509-496
Hugo C, 1997, J CLIN INVEST, V100, P786, DOI 10.1172/JCI119592
Humphreys BD, 2011, P NATL ACAD SCI USA, V108, P9226, DOI 10.1073/pnas.1100629108
Humphreys BD, 2010, AM J PATHOL, V176, P85, DOI 10.2353/ajpath.2010.090517

Ikarashi K, 2005, KIDNEY INT, V67, P1925, DOI 10.1111/j.1523-1755.2005.00291.x
Imasawa T, 2001, J AM SOC NEPHROL, V12, P1401, DOI 10.1681/ASN.V1271401
Ishida Y, 2008, J IMMUNOL, V180, P569, DOI 10.4049/jimmunol.180.1.569
Iwata Y, 2012, J IMMUNOL, V188, P4568, DOI 10.4049/jimmunol.1102154
Jennewein C, 2011, CURR OPIN ANESTHESIO, V24, P375, DOI 10.1097/ACO.0b013e3283489ac0
Kaczmarek A, 2013, IMMUNITY, V38, P209, DOI 10.1016/j.immuni.2013.02.003
Kramann R, 2017, J AM SOC NEPHROL, V28, P776, DOI 10.1681/ASN.2016030297
Kramann R, 2015, CELL STEM CELL, V16, P51, DOI 10.1016/j.stem.2014.11.004
Kriz W, 1999, CURR OPIN NEPHROL HY, V8, P489, DOI 10.1097/00041552-199907000-00014
Kroeze KL, 2012, J INVEST DERMATOL, V132, P216, DOI 10.1038/jid.2011.245
Kulkarni O, 2007, J AM SOC NEPHROL, V18, P2350, DOI 10.1681/ASN.2006121348
Kulkarni O, 2009, J PHARMACOL EXP THER, V328, P371, DOI 10.1124/jpet.108.142711
Kumar SVR, 2015, J AM SOC NEPHROL, V26, P2399, DOI 10.1681/ASN.2014070673
Kurts C, 2013, NAT REV IMMUNOL, V13, P738, DOI 10.1038/nri3523
Lai WF, 2014, J BIOSCIENCES, V39, P519, DOI 10.1007/s12038-014-9421-4
Lasagni L, 2013, CURR MOL MED, V13, P13, DOI 10.2174/156652413804486250
Lasagni L, 2015, STEM CELL REP, V5, P248, DOI 10.1016/j.stemcr.2015.07.003
Lasagni L, 2010, J AM SOC NEPHROL, V21, P1612, DOI 10.1681/ASN.2010010048
Lasagni L, 2010, STEM CELLS, V28, P1673, DOI 10.1002/stem.492
Lassen S, 2010, J IMMUNOL, V185, P1976, DOI 10.4049/jimmunol.0904207
Leadbetter EA, 2002, NATURE, V416, P603, DOI 10.1038/416603a
LeBleu V, 2009, J AM SOC NEPHROL, V20, P2359, DOI 10.1681/ASN.2009010123
Lech M, 2007, KIDNEY INT, V72, P182, DOI 10.1038/sj.ki.5002293
Lech M, 2014, J AM SOC NEPHROL, V25, P292, DOI 10.1681/ASN.2013020152
Lech M, 2012, MEDIAT INFLAMM, V2012, DOI 10.1155/2012/951390
Lech M, 2010, J PATHOL, V220, P596, DOI 10.1002/path.2678
Lee S, 2011, J AM SOC NEPHROL, V22, P317, DOI 10.1681/ASN.2009060615
Leemans JC, 2014, NAT REV NEPHROL, V10, P398, DOI 10.1038/nrneph.2014.91
Leventhal JS, 2012, KIDNEY INT, V81, P826, DOI 10.1038/ki.2011.498
Liapis H, 2013, AM J PATHOL, V183, P1364, DOI 10.1016/j.ajpath.2013.06.033
Lichtnekert J, 2016, J AM SOC NEPHROL, V27, P3611, DOI 10.1681/ASN.2015080877
Lichtnekert J, 2011, PLOS ONE, V6, DOI 10.1371/journal.pone.0026778
Lin SL, 2008, AM J PATHOL, V173, P1617, DOI 10.2353/ajpath.2008.080433
Linkermann A, 2014, NAT REV IMMUNOL, V14, P759, DOI 10.1038/nri3743
Linkermann A, 2014, NEW ENGL J MED, V370, P455, DOI 10.1056/NEJMra1310050

Martinod K, 2014, BLOOD, V123, P2768, DOI 10.1182/blood-2013-10-463646
Mauri C, 2017, J CLIN INVEST, V127, P772, DOI 10.1172/JCI85113
McCarthy CG, 2014, AM J PHYSIOL-HEART C, V306, pH184, DOI 10.1152/ajpheart.00328.2013
Mcgee HM, 2013, J INVEST DERMATOL, V133, P1321, DOI 10.1038/jid.2012.463
Medzhitov R, 2008, NATURE, V454, P428, DOI 10.1038/nature07201
Menke J, 2009, J CLIN INVEST, V119, P2330, DOI 10.1172/JCI39087
Migliorini A, 2013, AM J PATHOL, V183, P431, DOI 10.1016/j.ajpath.2013.04.017
Migliorini A, 2013, J NEPHROL, V26, P437, DOI 10.5301/jn.5000247
Migliorini A, 2012, NAT REV NEPHROL, V8, P183, DOI 10.1038/nrneph.2011.197
Minton K, 2015, NAT REV IMMUNOL, V15, P726, DOI 10.1038/nri3934
Mudaliar H, 2013, AM J PHYSIOL-RENAL, V305, pF143, DOI 10.1152/ajprenal.00398.2012
Mulay SR, 2017, NAT REV NEPHROL, V13, P226, DOI 10.1038/nrneph.2017.10
Mulay SR, 2016, SEMIN NEPHROL, V36, P162, DOI 10.1016/j.semnephrol.2016.03.004
Mulay SR, 2016, NEW ENGL J MED, V374, P2465, DOI 10.1056/NEJMra1601611
Mulay SR, 2016, AM J PATHOL, V186, P1442, DOI 10.1016/j.ajpath.2016.01.017
Mulay SR, 2016, J AM SOC NEPHROL, V27, P27, DOI 10.1681/ASN.2015040405
Mulay SR, 2013, J PATHOL, V230, P322, DOI 10.1002/path.4193
Mulay SR, 2012, KIDNEY INT, V81, P1199, DOI 10.1038/ki.2011.482
Nair AR, 2015, EXP CELL RES, V335, P238, DOI 10.1016/j.yexcr.2015.05.011
Nakazawa D, 2017, J AM SOC NEPHROL, V28, P1753, DOI 10.1681/ASN.2016080925
Nelson PJ, 2012, J AM SOC NEPHROL, V23, P194, DOI 10.1681/ASN.2011070680
Niedermeier M, 2009, P NATL ACAD SCI USA, V106, P17892, DOI 10.1073/pnas.0906070106
Niessen F, 2008, NATURE, V452, P654, DOI 10.1038/nature06663
Ninichuk V, 2005, J AM SOC NEPHROL, V16, P977, DOI 10.1681/ASN.2004100871
Ninichuk V, 2008, AM J PATHOL, V172, P628, DOI 10.2353/ajpath.2008.070601
Ninichuk V, 2007, AM J PATHOL, V170, P1267, DOI 10.2353/ajpath.2007.060937
Noris M, 2013, SEMIN NEPHROL, V33, P479, DOI 10.1016/j.semnephrol.2013.08.001
Noris M, 2009, J IMMUNOL, V183, P4249, DOI 10.4049/jimmunol.0803549
Nurden AT, 2011, THROMB HAEMOSTASIS, V105, pS13, DOI 10.1160/THS10-11-0720
PALABRICA T, 1992, NATURE, V359, P848, DOI 10.1038/359848a0
Patole PS, 2005, KIDNEY INT, V68, P2582, DOI 10.1111/j.1523-1755.2005.00729.x
Pawar RD, 2006, AM J PHYSIOL-RENAL, V291, pF509, DOI 10.1152/ajprenal.00453.2005
Pawar RD, 2009, IMMUNOLOGY, V128, pe206, DOI 10.1111/j.1365-2567.2008.02948.x
Peired A, 2013, J AM SOC NEPHROL, V24, P1756, DOI 10.1681/ASN.2012090950

Pickert G, 2009, J EXP MED, V206, P1465, DOI 10.1084/jem.20082683
Plikus MV, 2012, SEMIN CELL DEV BIOL, V23, P946, DOI 10.1016/j.semcdb.2012.10.001
Prodromidi EI, 2006, STEM CELLS, V24, P2448, DOI 10.1634/stemcells.2006-0201
Rabelink TJ, 2013, NAT REV NEPHROL, V9, P747, DOI 10.1038/nrneph.2013.152
Reich B, 2013, KIDNEY INT, V84, P78, DOI 10.1038/ki.2013.84
Reiser J, 2013, ANNU REV MED, V64, P357, DOI 10.1146/annurev-med-050311-163340
Ribeiro A, 2012, KIDNEY INT, V81, P100, DOI 10.1038/ki.2011.311
Rock KL, 2010, ANNU REV IMMUNOL, V28, P321, DOI 10.1146/annurev-immunol-030409-101311
Romagnani P, 2013, NAT REV NEPHROL, V9, P137, DOI 10.1038/nrneph.2012.290
Romagnani P, 2011, AM J PATHOL, V178, P490, DOI 10.1016/j.ajpath.2010.11.044
Romagnani P, 2009, STEM CELLS, V27, P2247, DOI 10.1002/stem.140
Ryu M, 2012, J PATHOL, V228, P482, DOI 10.1002/path.4046
Ryu M, 2011, KIDNEY INT, V79, P189, DOI 10.1038/ki.2010.373
Sakai N, 2006, P NATL ACAD SCI USA, V103, P14098, DOI 10.1073/pnas.0511200103
Sallustio F, 2013, KIDNEY INT, V83, P392, DOI 10.1038/ki.2012.413
Sayyed SG, 2011, KIDNEY INT, V80, P68, DOI 10.1038/ki.2011.102
Sayyed SG, 2010, NEPHROL DIAL TRANSPL, V25, P1811, DOI 10.1093/ndt/gfp730
Schrimpf C, 2012, J AM SOC NEPHROL, V23, P868, DOI 10.1681/ASN.2011080851
Semple JW, 2011, NAT REV IMMUNOL, V11, P264, DOI 10.1038/nri2956
Seok SJ, 2013, NEPHROL DIAL TRANSPL, V28, P1700, DOI 10.1093/ndt/gfs555
Sethi S, 2012, NEW ENGL J MED, V366, P1119, DOI 10.1056/NEJMra1108178
Shahzad K, 2015, KIDNEY INT, V87, P74, DOI 10.1038/ki.2014.271
Shankland SJ, 2017, CURR OPIN NEPHROL HY, V26, P154, DOI 10.1097/MNH.0000000000000311
Shankland SJ, 2013, CURR OPIN NEPHROL HY, V22, P302, DOI 10.1097/MNH.0b013e32835fefd4
Smeets B, 2011, J AM SOC NEPHROL, V22, P1262, DOI 10.1681/ASN.2010090970
Smeets B, 2009, J AM SOC NEPHROL, V20, P2593, DOI 10.1681/ASN.2009020132
Smith SA, 2015, CRIT REV BIOCHEM MOL, V50, P326, DOI 10.3109/10409238.2015.1050550
Smith SW, 2012, HISTOL HISTOPATHOL, V27, P1503, DOI 10.14670/HH-27.1503
Sörensen I, 2011, KIDNEY INT, V80, P1035, DOI 10.1038/ki.2011.214
Srivastava A, 2013, CLIN RHEUMATOL, V32, P1317, DOI 10.1007/s10067-013-2268-5
Starke C, 2015, J AM SOC NEPHROL, V26, P48, DOI 10.1681/ASN.2014030265
Stokman MF, 2016, NAT REV NEPHROL, V12, P472, DOI 10.1038/nrneph.2016.87
STOUT LC, 1993, HUM PATHOL, V24, P77
Suárez-Alvarez B, 2016, LAB INVEST, V96, P378, DOI 10.1038/labinvest.2015.164

Sugimoto H, 2006, P NATL ACAD SCI USA, V103, P7321, DOI 10.1073/pnas.0601436103
Sugimoto H, 2012, NAT MED, V18, P396, DOI 10.1038/nm.2629
Sun BK, 2014, SCIENCE, V346, P941, DOI 10.1126/science.1253836
Thomasova D, 2015, NEPHROL DIAL TRANSPL, V30, P1622, DOI 10.1093/ndt/gfu395
Thomasova D, 2012, NEOPLASIA, V14, P1097, DOI 10.1593/neo.121534
Urushihara M, 2011, HYPERTENSION, V57, P586, DOI 10.1161/HYPERTENSIONAHA.110.165704
Vaughan AE, 2015, NATURE, V517, P621, DOI 10.1038/nature14112
Velnar T, 2009, J INT MED RES, V37, P1528, DOI 10.1177/147323000903700531
Vielhauer V, 2004, KIDNEY INT, V66, P2264, DOI 10.1111/j.1523-1755.2004.66038.x
Villanueva E, 2011, J IMMUNOL, V187, P538, DOI 10.4049/jimmunol.1100450
Vivante A, 2013, CURR OPIN NEPHROL HY, V22, P325, DOI 10.1097/MNH.0b013e32835f7241
Vize PD, 2004, ANAT REC PART A, V277A, P344, DOI 10.1002/ar.a.20017
Wanner N, 2014, J AM SOC NEPHROL, V25, P707, DOI 10.1681/ASN.2013050452
Weiclenbusch M, 2012, J INNATE IMMUN, V4, P463, DOI 10.1159/000336717
Wen ZK, 2007, RHEUMATOLOGY, V46, P1796, DOI 10.1093/rheumatology/kem275
Werner S, 2003, PHYSIOL REV, V83, P835, DOI 10.1152/physrev.2003.83.3.835
WILLIAMS GC, 1991, Q REV BIOL, V66, P1, DOI 10.1086/417048
Wu H. P. J. M., 2010, J AM SOC NEPHROL, V21
Wu Y, 2015, THROMB J, V13, DOI 10.1186/s12959-015-0048-y
Wynn TA, 2007, J CLIN INVEST, V117, P524, DOI 10.1172/JCI31487
Xia YF, 2014, KIDNEY INT, V86, P327, DOI 10.1038/ki.2014.64
Yang CW, 2006, KIDNEY INT, V69, P815, DOI 10.1038/sj.ki.5000119
Yang L, 2010, NAT MED, V16, P535, DOI 10.1038/nm.2144
Yuste C, 2015, PLOS ONE, V10, DOI 10.1371/journal.pone.0128575
Zeisberg M, 2010, J AM SOC NEPHROL, V21, P1819, DOI 10.1681/ASN.2010080793
Zeisberg M, 2010, J AM SOC NEPHROL, V21, P1247, DOI 10.1681/ASN.2010060616
Zoja C, 2012, AM J PHYSIOL-RENAL, V303, pF1370, DOI 10.1152/ajprenal.00057.2012
NR 212
TC 6
Z9 6
U1 1
U2 22
PU F HERNANDEZ
PI MURCIA
PA PLAZA FUENSANTA 2-7 C, 30008 MURCIA, SPAIN
SN 0213-3911
EI 1699-5848
J9 HISTOL HISTOPATHOL
JI Histol. Histopath.
PD NOV
PY 2017

VL 32
IS 11
BP 1099
EP 1113
DI 10.14670/HH-11-900
PG 15
WC Cell Biology; Pathology
WE Science Citation Index Expanded (SCI-EXPANDED)
SC Cell Biology; Pathology
GA FM0CT
UT WOS:000414627200001
PM 28481398
DA 2025-05-07
ER

PT J
AU Somarelli, JA
   Boddy, AM
   Gardner, HL
   DeWitt, SB
   Tuohy, J
   Megquier, K
   Sheth, MU
   Hsu, SD
   Thorne, JL
   London, CA
   Eward, WC
AF Somarelli, Jason A.
   Boddy, Amy M.
   Gardner, Heather L.
   DeWitt, Suzanne Bartholf
   Tuohy, Joanne
   Megquier, Kate
   Sheth, Maya U.
   Hsu, Shiaowen David
   Thorne, Jeffrey L.
   London, Cheryl A.
   Eward, William C.
TI Improving Cancer Drug Discovery by Studying Cancer across the Tree of
   Life
SO MOLECULAR BIOLOGY AND EVOLUTION
LA English
DT Article
DE veterinary oncology; cross-species studies; cancer drug discovery;
   evolutionary biology
ID PROGRESSION; EVOLUTION; DOGS
AB Despite a considerable expenditure of time and resources and
significant advances in experimental models of disease, cancer research
continues to suffer from extremely low success rates in translating
preclinical discoveries into clinical practice. The continued failure of
cancer drug development, particularly late in the course of human
testing, not only impacts patient outcomes, but also drives up the cost
for those therapies that do succeed. It is clear that a paradigm shift is
necessary if improvements in this process are to occur. One promising

direction for increasing translational success is comparative oncology- the study of cancer across species, often involving veterinary patients that develop naturally-occurring cancers. Comparative oncology leverages the power of cross-species analyses to understand the fundamental drivers of cancer protective mechanisms, as well as factors contributing to cancer initiation and progression. Clinical trials in veterinary patients with cancer provide an opportunity to evaluate novel therapeutics in a setting that recapitulates many of the key features of human cancers, including genomic aberrations that underly tumor development, response and resistance to treatment, and the presence of comorbidities that can affect outcomes. With a concerted effort from basic scientists, human physicians and veterinarians, comparative oncology has the potential to enhance the cost-effectiveness and efficiency of pipelines for cancer drug discovery and other cancer treatments.
C1 [Somarelli, Jason A.; Sheth, Maya U.; Hsu, Shiaowen David] Duke Univ, Med Ctr, Dept Med, Durham, NC 27710 USA.
[Somarelli, Jason A.; Sheth, Maya U.; Hsu, Shiaowen David; Eward, William C.] Duke Canc Inst, Durham, NC 27710 USA.
[Boddy, Amy M.] Univ Calif Santa Barbara, Dept Anthropol, Santa Barbara, CA 93106 USA.
[Gardner, Heather L.; London, Cheryl A.] Tufts Univ, Cummings Sch Vet Med, Boston, MA 02111 USA.
[DeWitt, Suzanne Bartholf; Eward, William C.] Duke Univ, Med Ctr, Dept Orthopaed, Durham, NC USA.
[Tuohy, Joanne] Virginia Maryland Reg Coll Vet Med, Blacksburg, VA USA.
[Megquier, Kate] MIT, Broad Inst, Boston, MA USA.
[Megquier, Kate] Harvard Univ, Boston, MA 02115 USA.
[Thorne, Jeffrey L.] North Carolina State Univ, Dept Biol Sci, Raleigh, NC USA.
[Thorne, Jeffrey L.] North Carolina State Univ, Dept Stat, Raleigh, NC USA.
C3 Duke University; Duke University; University of California System; University of California Santa Barbara; Tufts University; Duke University; Harvard University; Massachusetts Institute of Technology (MIT); Broad Institute; Harvard University; North Carolina State University; North Carolina State University
RP Somarelli, JA (autor correspondente), Duke Univ, Med Ctr, Dept Med, Durham, NC 27710 USA.; Somarelli, JA (autor correspondente), Duke Canc Inst, Durham, NC 27710 USA.
EM jason.somarelli@duke.edu
RI London, Cheryl/E-6561-2012; Bartholf DeWitt, Suzanne/KVB-3549-2024
OI Somarelli, Jason/0000-0003-1510-9343; Sheth, Maya/0000-0003-1466-8899
FU Society for Molecular Biology and Evolution; Triangle Center for Evolutionary Medicine; Consortium for Canine Comparative Oncology
FX JAS acknowledges funding from the Society for Molecular Biology and Evolution and the Triangle Center for Evolutionary Medicine. JAS and WCE
acknowledge support from the Consortium for Canine Comparative Oncology.
CR Abegglen LM, 2015, JAMA-J AM MED ASSOC, V314, P1850, DOI 10.1001/jama.2015.13134
Adams MD, 2000, SCIENCE, V287, P2185, DOI 10.1126/science.287.5461.2185

Akamatsu S, 2015, CELL REP, V12, P922, DOI 10.1016/j.celrep.2015.07.012
Aktipis CA, 2015, PHILOS T R SOC B, V370, DOI 10.1098/rstb.2014.0219
Arrowsmith J, 2011, NAT REV DRUG DISCOV, V10, P1, DOI 10.1038/nrd3439
Arrowsmith J, 2011, NAT REV DRUG DISCOV, V10, P1, DOI 10.1038/nrd3375
Atlas RM, 2013, CURR TOP MICROBIOL, V365, P1, DOI 10.1007/82_2012_223
Ben-David U, 2017, NAT GENET, V49, P1567, DOI 10.1038/ng.3967
Bracken-Grissom H, 2014, J HERED, V105, P1, DOI 10.1093/jhered/est084
C elegans Sequencing Consortium, 1998, SCIENCE, V282, P2012, DOI 10.1126/science.282.5396.2012
Caulin AF, 2015, PHILOS T R SOC LON B, V370, P1850
D'Souza Alaric W., 2014, Evolution Medicine and Public Health, P136, DOI 10.1093/emph/eou022
Day CP, 2015, CELL, V163, P39, DOI 10.1016/j.cell.2015.08.068
Dunn KA, 2000, VET J, V160, P77, DOI 10.1053/tvjl.2000.0495
EDIDIN M, 1986, HUM IMMUNOL, V15, P357, DOI 10.1016/0198-8859(86)90012-1
Elvers I, 2015, GENOME RES, V25, P1634, DOI 10.1101/gr.194449.115
Ericsson Aaron C, 2013, Mo Med, V110, P201
Evans JD, 2013, J HERED, V104, P595, DOI 10.1093/jhered/est050
Friedberg MW, 2017, JAMA INTERN MED, V177, P1058, DOI 10.1001/jamainternmed.2017.2142
Gardner HL, 2019, COMMUN BIOL, V2, DOI 10.1038/s42003-019-0487-2
Gardner HL, 2016, ANNU REV ANIM BIOSCI, V4, P199, DOI 10.1146/annurev-animal-022114-110911
Guo LH, 2019, BMC CANCER, V19, DOI 10.1186/s12885-019-5555-y
Hai PF, 2019, NAT BIOMED ENG, V3, P381, DOI 10.1038/s41551-019-0376-5
Hoffman M, 2019, CANCER RES, V79, P2111, DOI 10.1158/0008-5472.CAN-18-3441
Jia QZ, 2018, NAT COMMUN, V9, DOI 10.1038/s41467-018-07767-w
Koepfli KP, 2015, ANNU REV ANIM BIOSCI, V3, P57, DOI 10.1146/annurev-animal-090414-014900
Lewin HA, 2018, P NATL ACAD SCI USA, V115, P4325, DOI 10.1073/pnas.1720115115
Li XR, 2016, INT J ONCOL, V48, P1933, DOI 10.3892/ijo.2016.3406
Maekawa N, 2014, PLOS ONE, V9, DOI 10.1371/journal.pone.0098415
Maki RG, 2005, CANCER-AM CANCER SOC, V103, P1431, DOI 10.1002/cncr.20968
McDonald OG, 2017, NAT GENET, V49, P367, DOI 10.1038/ng.3753
Naghavi M, 2017, LANCET, V390, P1151, DOI 10.1016/S0140-6736(17)32152-9
Paul SM, 2010, NAT REV DRUG DISCOV, V9, P203, DOI 10.1038/nrd3078
Peng YP, 2017, J EXP CLIN CANC RES, V36, DOI 10.1186/s13046-017-0500-x
PETO R, 1975, BRIT J CANCER, V32, P411, DOI 10.1038/bjc.1975.242
Reiter JG, 2018, SCIENCE, V361, P1033, DOI 10.1126/science.aat7171
Risom T, 2018, NAT COMMUN, V9, DOI 10.1038/s41467-018-05729-w
Ryser MD, 2018, SCI REP-UK, V8, DOI 10.1038/s41598-018-35621-y
Sakthikumar S, 2018, CANCER RES, V78, P3421, DOI 10.1158/0008-5472.CAN-17-3558
Scott MC, 2018, CANCER RES, V78, P326, DOI 10.1158/0008-5472.CAN-17-0576
Seim I, 2013, NAT COMMUN, V4, DOI 10.1038/ncomms3212
Somarelli JA, 2020, MOL BIOL EVOL, V37, P320, DOI 10.1093/molbev/msz242

Sulak M, 2016, ELIFE, V5, DOI [10.7554/eLife.11994, 10.7554/eLife.24307]
Tollis M, 2019, MOL BIOL EVOL, V36, P1746, DOI 10.1093/molbev/msz099
Tollis M, 2017, CURR OPIN GENET DEV, V42, P40, DOI 10.1016/j.gde.2016.12.004
Wang D, 2019, CANCER RES, V79, P7, DOI 10.1158/0008-5472.CAN-18-1086
Waterston RH, 2002, NATURE, V420, P520, DOI 10.1038/nature01262
Wu HJ, 2018, CANCER LETT, V438, P133, DOI 10.1016/j.canlet.2018.09.017
Yates LR, 2017, CANCER CELL, V32, P169, DOI 10.1016/j.ccell.2017.07.005
Zhang XN, 2019, J INVEST DERMATOL, V139, P1526, DOI 10.1016/j.jid.2019.01.019
Zhao MQ, 2017, ONCOL LETT, V14, P2919, DOI 10.3892/ol.2017.6498
NR 51
TC 23
Z9 25
U1 1
U2 10
PU OXFORD UNIV PRESS
PI OXFORD
PA GREAT CLARENDON ST, OXFORD OX2 6DP, ENGLAND
SN 0737-4038
EI 1537-1719
J9 MOL BIOL EVOL
JI Mol. Biol. Evol.
PD JAN
PY 2020
VL 37
IS 1
BP 11
EP 17
DI 10.1093/molbev/msz254
PG 7
WC Biochemistry & Molecular Biology; Evolutionary Biology; Genetics & Heredity
WE Science Citation Index Expanded (SCI-EXPANDED)
SC Biochemistry & Molecular Biology; Evolutionary Biology; Genetics & Heredity
GA KN8UR
UT WOS:000515121200003
PM 31688937
OA Green Published
DA 2025-05-07
ER

PT J
AU Williams, TC
Drake, AJ
AF Williams, Thomas C.
Drake, Amanda J.
TI Preterm birth in evolutionary context: a predictive adaptive response?
SO PHILOSOPHICAL TRANSACTIONS OF THE ROYAL SOCIETY B-BIOLOGICAL SCIENCES
LA English
DT Article

DE preterm birth; early life effects; predictive adaptive response;
evolution
ID MATERNAL STRESS; SYSTEMATIC ANALYSIS; ECONOMIC MOBILITY;
GESTATIONAL-AGE; BLOOD-PRESSURE; RISK-FACTOR; WEIGHT; BORN; DISEASE;
MENARCHE
AB Preterm birth is a significant public health problem worldwide, leading to substantial mortality in the newborn period, and a considerable burden of complications longer term, for affected infants and their carers. The fact that it is so common, and rates vary between different populations, raising the question of whether in some circumstances it might be an adaptive trait. In this review, we outline some of the evolutionary explanations put forward for preterm birth. We specifically address the hypothesis of the predictive adaptive response, setting it in the context of the Developmental Origins of Health and Disease, and explore the predictions that this hypothesis makes for the potential causes and consequences of preterm birth. We describe how preterm birth can be triggered by a range of adverse environmental factors, including nutrition, stress and relative socioeconomic status. Examining the literature for any associated longer-term phenotypic changes, we find no strong evidence for a marked temporal shift in the reproductive life-history trajectory, but more persuasive evidence for a re-programming of the cardiovascular and endocrine system, and a range of effects on neurodevelopment. Distinguishing between preterm birth as a predictive, rather than immediate adaptive response will depend on the demonstration of a positive effect of these alterations in developmental trajectories on reproductive fitness.

This article is part of the theme issue 'Developing differences: early-life effects and evolutionary medicine'.
C1 [Williams, Thomas C.] Univ Edinburgh, Inst Genet & Mol Med, Human Genet Unit, MRC, Edinburgh EH4 2XU, Midlothian, Scotland.

[Drake, Amanda J.] Univ Edinburgh, Queens Med Res Inst, British Heart Fdn Ctr Cardiovasc Sci, Edinburgh EH16 4TJ, Midlothian, Scotland.
C3 University of Edinburgh; University of Edinburgh
RP Williams, TC (autor correspondente), Univ Edinburgh, Inst Genet & Mol Med, Human Genet Unit, MRC, Edinburgh EH4 2XU, Midlothian, Scotland.
EM thomaschristiewilliams@gmail.com
RI Williams, Thomas/AFM-7862-2022
OI drake, amanda/0000-0001-8633-333X; Williams, Thomas/0000-0002-3866-1344
FU Wellcome Trust PhD Fellowship as part of the Edinburgh Clinical Academic
Track scheme [204802/Z/16/Z]
FX T.C.W. is supported by a Wellcome Trust PhD Fellowship as part of the Edinburgh Clinical Academic Track scheme (204802/Z/16/Z).
CR Ahlsén AK, 2015, ARCH DIS CHILD-FETAL, V100, pF150, DOI 10.1136/archdischild-2013-305877
[Anonymous], 1977, Acta Obstet Gynecol Scand, V56, P247
[Anonymous], CD-10: international statistical classification of diseases and related health problems
[Anonymous], 1948, MAN INT STAT CLASS D
Ballantyne JW, 1902, BMJ-BRIT MED J, V1902, P1196, DOI 10.1136/bmj.1.2159.1196
Barker DJP, 2012, PUBLIC HEALTH, V126, P185, DOI 10.1016/j.puhe.2011.11.014

BARKER DJP, 1989, LANCET, V2, P577
Bateson P, 2014, J PHYSIOL-LONDON, V592, P2357, DOI 10.1113/jphysiol.2014.271460
Björkenstam E, 2018, PSYCHOL MED, V48, P498, DOI 10.1017/S0033291717001933
Blencowe H, 2013, REPROD HEALTH, V10, DOI 10.1186/1742-4755-10-S1-S2
Blencowe H, 2013, PEDIATR RES, V74, P17, DOI 10.1038/pr.2013.204
Blencowe H, 2012, LANCET, V379, P2162, DOI 10.1016/S0140-6736(12)60820-4
Bloomfield FH, 2003, SCIENCE, V300, P606, DOI 10.1126/science.1080803
Bonamy AKE, 2005, PEDIATR RES, V58, P845, DOI 10.1203/01.PDR.0000181373.29290.80
Braveman P, 2014, PUBLIC HEALTH REP, V129, P19, DOI 10.1177/00333549141291S206
Capper A, 1928, AM J DIS CHILD, V35, P262, DOI 10.1001/archpedi.1928.01920200094012
Carlo WA, 2010, PEDIATRICS, V126, pE1072, DOI 10.1542/peds.2010-1183
Chisholm JS, 2005, HUM NATURE-INT BIOS, V16, P233, DOI 10.1007/s12110-005-1009-0
Cleal JK, 2007, PEDIATR RES, V62, P422, DOI 10.1203/PDR.0b013e31813cbe60
Coall DA, 2003, SOC SCI MED, V57, P1771, DOI 10.1016/S0277-9536(03)00022-4
Collins JW, 2015, MATERN CHILD HLTH J, V19, P1601, DOI 10.1007/s10995-015-1670-9
Collins JW, 2011, AM J PUBLIC HEALTH, V101, P714, DOI 10.2105/AJPH.2010.195024
Collins JW, 2004, AM J PUBLIC HEALTH, V94, P2132, DOI 10.2105/AJPH.94.12.2132
Copper RL, 1996, AM J OBSTET GYNECOL, V175, P1286, DOI 10.1016/S0002-9378(96)70042-X
Cruickshank MN, 2013, GENOME MED, V5, DOI 10.1186/gm500
Dole N, 2003, AM J EPIDEMIOL, V157, P14, DOI 10.1093/aje/kwf176
Dossus L, 2012, ANN EPIDEMIOL, V22, P723, DOI 10.1016/j.annepidem.2012.07.007
Forrester TE, 2012, PLOS ONE, V7, DOI 10.1371/journal.pone.0035907
FORSDAHL A, 1977, BRIT J PREV SOC MED, V31, P91
Francis-Emmanuel PM, 2014, J CLIN ENDOCR METAB, V99, P2233, DOI 10.1210/jc.2013-3511
Gardosi JO, 2005, EARLY HUM DEV, V81, P43, DOI 10.1016/j.earlhumdev.2004.10.015
Gluckman P, 2016, PRINCIPLES OF EVOLUTIONARY MEDICINE, 2ND EDITION, P1, DOI 10.1093/acprof:oso/9780199663927.001.0001
Gluckman PD, 2004, SCIENCE, V305, P1733, DOI 10.1126/science.1095292
Gluckman PD, 2007, AM J HUM BIOL, V19, P1, DOI 10.1002/ajhb.20590
Goldenberg RL, 2008, LANCET, V371, P75, DOI 10.1016/S0140-6736(08)60074-4
Grunau RE, 2007, J PEDIATR-US, V150, P151, DOI 10.1016/j.jpeds.2006.10.053
Haig D., 2008, EVOLUTION HLTH DIS, V2, P65
Hanson MA, 2014, PHYSIOL REV, V94, P1027, DOI 10.1152/physrev.00029.2013
Harville E, 2010, OBSTET GYNECOL SURV, V65, P713, DOI 10.1097/OGX.0b013e31820eddbe

Hughes MM, 2017, MATERN CHILD HLTH J, V21, P283, DOI 10.1007/s10995-016-2131-9
Hughson M, 2003, KIDNEY INT, V63, P2113, DOI 10.1046/j.1523-1755.2003.00018.x
Huxley R, 2007, AM J CLIN NUTR, V85, P1244, DOI 10.1093/ajcn/85.5.1244
James E, 2018, BMC PEDIATR, V18, DOI 10.1186/s12887-017-0976-8
Johnson S, 2011, PEDIATR RES, V69, p11R, DOI 10.1203/PDR.0b013e318212faa0
Kermack W, 1933, LANCET, V223, P80
KRAMER MS, 1988, JAMA-J AM MED ASSOC, V260, P3306, DOI 10.1001/jama.260.22.3306
Kundakovic M, 2017, GENES-BASEL, V8, DOI 10.3390/genes8030104
Lee ACC, 2013, LANCET GLOB HEALTH, V1, pE26, DOI 10.1016/S2214-109X(13)70006-8
LEE TM, 1988, AM J PHYSIOL, V255, pR831, DOI 10.1152/ajpregu.1988.255.5.R831
Lewandowski AJ, 2015, HYPERTENSION, V65, P607, DOI 10.1161/HYPERTENSIONAHA.114.04662
Mahoney AD, 2013, CLIN PERINATOL, V40, P665, DOI 10.1016/j.clp.2013.07.004
Mañalich R, 2000, KIDNEY INT, V58, P770, DOI 10.1046/j.1523-1755.2000.00225.x
Menon G, 2013, ARCH DIS CHILD-FETAL, V98, pF559, DOI 10.1136/archdischild-2012-303582
Murray CJL, 2012, LANCET, V380, P2197, DOI 10.1016/S0140-6736(12)61729-2
Parets SE, 2013, PLOS ONE, V8, DOI 10.1371/journal.pone.0067489
Parkinson JRC, 2013, PEDIATRICS, V131, pE1240, DOI 10.1542/peds.2012-2177
Phillips Julie Baker, 2015, Evolution Medicine and Public Health, P136, DOI 10.1093/emph/eov010
Pike IL, 2005, AM J HUM BIOL, V17, P55, DOI 10.1002/ajhb.20093
Piyasena C, 2016, FRONT ENDOCRINOL, V7, DOI 10.3389/fendo.2016.00158
Reynolds RM, 2013, PSYCHONEUROENDOCRINO, V38, P1, DOI 10.1016/j.psyneuen.2012.08.012
Sapolsky RM, 2005, SCIENCE, V308, P648, DOI 10.1126/science.1106477
SCHLESINGER ER, 1955, PEDIATRICS, V15, P698
Shaw GM, 2014, PAEDIATR PERINAT EP, V28, P302, DOI 10.1111/ppe.12125
Singhal A, 2003, LANCET, V361, P1089, DOI 10.1016/S0140-6736(03)12895-4
Sloboda DM, 2007, J CLIN ENDOCR METAB, V92, P46, DOI 10.1210/jc.2006-1378
Smith LK, 2007, ARCH DIS CHILD-FETAL, V92, pF11, DOI 10.1136/adc.2005.090308
Sparrow S, 2016, TRANSL PSYCHIAT, V6, DOI 10.1038/tp.2015.210
TRIVERS RL, 1974, AM ZOOL, V14, P249
Vinturache A, 2017, BMJ OPEN, V7, DOI 10.1136/bmjopen-2016-015258
Wells JCK, 2007, TRENDS ENDOCRIN MET, V18, P331, DOI 10.1016/j.tem.2007.07.006
WHO, 2017, WHO PRET BIRTH
Wildman DE, 2011, PLOS ONE, V6, DOI 10.1371/journal.pone.0024509
Ylppo Arvo, 1919, EUR J PEDIAT, V20, P212
NR 73
TC 18

Z9 19
U1 0
U2 13
PU ROYAL SOC
PI LONDON
PA 6-9 CARLTON HOUSE TERRACE, LONDON SW1Y 5AG, ENGLAND
SN 0962-8436
EI 1471-2970
J9 PHILOS T R SOC B
JI Philos. Trans. R. Soc. B-Biol. Sci.
PD APR 15
PY 2019
VL 374
IS 1770
AR 20180121
DI 10.1098/rstb.2018.0121
PG 9
WC Biology
WE Science Citation Index Expanded (SCI-EXPANDED); Social Science Citation Index (SSCI)
SC Life Sciences & Biomedicine - Other Topics
GA HN8ZI
UT WOS:000460487000012
PM 30966892
OA Green Published, hybrid
DA 2025-05-07
ER

PT J
AU Nunn, CL
   Samson, DR
   Krystal, AD
AF Nunn, Charles L.
   Samson, David R.
   Krystal, Andrew D.
TI Shining evolutionary light on human sleep and sleep disorders
SO EVOLUTION MEDICINE AND PUBLIC HEALTH
LA English
DT Review
ID SEASONAL AFFECTIVE-DISORDER; PAN-TROGLODYTES-SCHWEINFURTHII; SEMLIKI WILDLIFE RESERVE; ADAPTIVE SIGNIFICANCE; METABOLIC SYNDROME; MAMMALIAN SLEEP; GROWTH-HORMONE; INFANT SLEEP; SHIFT WORK; CHIMPANZEES
AB Sleep is essential to cognitive function and health in humans, yet the ultimate reasons for sleep-i.e. 'why' sleep evolved-remain mysterious. We integrate findings from human sleep studies, the ethnographic record, and the ecology and evolution of mammalian sleep to better understand sleep along the human lineage and in the modern world. Compared to other primates, sleep in great apes has undergone substantial evolutionary change, with all great apes building a sleeping platform or 'nest'. Further evolutionary change characterizes human sleep, with humans having the shortest sleep duration, yet the highest proportion of rapid eye movement sleep among primates. These changes likely reflect that our ancestors experienced fitness benefits from being active for a greater portion of the 24-h cycle than other primates, potentially related to

advantages arising from learning, socializing and defending against predators and hostile conspecifics. Perspectives from evolutionary medicine have implications for understanding sleep disorders; we consider these perspectives in the context of insomnia, narcolepsy, seasonal affective disorder, circadian rhythm disorders and sleep apnea. We also identify how human sleep today differs from sleep through most of human evolution, and the implications of these changes for global health and health disparities. More generally, our review highlights the importance of phylogenetic comparisons in understanding human health, including well-known links between sleep, cognitive performance and health in humans.
C1 [Nunn, Charles L.; Samson, David R.] Duke Univ, Dept Evolutionary Anthropol, Durham, NC 27708 USA.
[Nunn, Charles L.] Duke Global Hlth Inst, Durham, NC 27710 USA.
[Nunn, Charles L.] Triangle Ctr Evolutionary Med, Durham, NC 27708 USA.
[Krystal, Andrew D.] Duke Univ, Sch Med, Dept Psychiat & Behav Sci, Durham, NC 27710 USA.
C3 Duke University; Duke University; Duke University
RP Nunn, CL (autor correspondente), Duke Univ, Dept Evolutionary Anthropol, Durham, NC 27708 USA.
EM clnunn@duke.edu
RI Samson, David/B-6879-2011; Krystal, Andrew/J-7109-2013
CR Alhola Paula, 2007, Neuropsychiatr Dis Treat, V3, P553
Altevogt BM, 2006, SLEEP DISORDERS SLEE, DOI DOI 10.17226/11617
Anderson JR, 2000, SLEEP MED REV, V4, P355, DOI 10.1053/smrv.2000.0105
[Anonymous], THESIS
[Anonymous], 1989, SLEEP ALERTNESS CHRO
[Anonymous], COMP PRIMATE BIOL SY
[Anonymous], PERFORMANCE STRESS
[Anonymous], SLEEP CIRCUITS FUNCT
[Anonymous], 2009, RISE HOMOSAPIENS EVO
[Anonymous], AM J HUMAN BIOL
[Anonymous], 2014, EVOL MED PUBLIC HLTH, DOI DOI 10.1093/EMPH/EOU005
[Anonymous], FOREIGN AFFAIRS
[Anonymous], 1985, Ancestors: The hard evidence
BALDWIN P J, 1981, Primates, V22, P474, DOI 10.1007/BF02381239
Besedovsky L, 2012, PFLUG ARCH EUR J PHY, V463, P121, DOI 10.1007/s00424-011-1044-0
Busse C., 1980, Botswana Notes and Records, V12, P15
Capellini I, 2008, EVOLUTION, V62, P1764, DOI 10.1111/j.1558-5646.2008.00392.x
Capellini Isabella, 2010, P12
Capellini I, 2009, PLOS ONE, V4, DOI 10.1371/journal.pone.0004609
Carpenter R, 2013, BMJ OPEN, V3, DOI 10.1136/bmjopen-2012-002299
Carter MJ, 2014, THER RECREAT J, V48, P275
Chaput JP, 2013, PREV MED, V57, P872, DOI 10.1016/j.ypmed.2013.09.022
Chen LC, 2009, CNS NEUROL DISORD-DR, V8, P296, DOI 10.2174/187152709788921717
Cheyne SM, 2012, FOLIA PRIMATOL, V83, P299, DOI 10.1159/000342145
Coolidge FL., 2006, FARMING, V4, P1, DOI DOI 10.3828/BFARM.2006.4.11
Covert HH, 1995, CREATURES OF THE DARK, P495
CROOK JH, 1966, NATURE, V210, P1200, DOI 10.1038/2101200a0

Davis C, 2005, J AFFECT DISORDERS, V87, P3, DOI 10.1016/j.jad.2005.03.006
de la Iglesia HO, 2015, J BIOL RHYTHM, V30, P342, DOI 10.1177/0748730415590702
Dinges DF, 1997, SLEEP, V20, P267
Duda P, 2013, J HUM EVOL, V65, P424, DOI 10.1016/j.jhevol.2013.07.009
Durmer JS, 2005, SEMIN NEUROL, V25, P117, DOI 10.1055/s-0029-1237117
Eagles JM, 2004, MED HYPOTHESES, V63, P767, DOI 10.1016/j.mehy.2004.07.002
Eckert DJ, 2007, CHEST, V131, P595, DOI 10.1378/chest.06.2287
Edinger JD, 2008, SLEEP, V31, P599, DOI 10.1093/sleep/31.5.599
Edinger JD, 2013, SLEEP, V36, P1179, DOI 10.5665/sleep.2882
EkiRCH A. R., 2006, At Day's Close: Night in Times Past
Ekirch AR, 2016, SLEEP, V39, P715, DOI 10.5665/sleep.5558
ELGAR MA, 1988, ANIM BEHAV, V36, P1407, DOI 10.1016/S0003-3472(88)80211-2
Evans DS, 2011, SLEEP, V34, P661, DOI 10.1093/sleep/34.5.661
Everett DanielL., 2009, Don't sleep, there are snakes: Life and language in the Amazonian jungle
Faraco J, 2013, PLOS GENET, V9, DOI 10.1371/journal.pgen.1003270
Faraut B, 2012, SLEEP MED REV, V16, P137, DOI 10.1016/j.smrv.2011.05.001
Friborg O, 2012, J SLEEP RES, V21, P176, DOI 10.1111/j.1365-2869.2011.00982.x
Fruth Barbara, 1996, P225, DOI 10.1017/CBO9780511752414.019
Gangwisch JE, 2014, AM J HYPERTENS, V27, P1235, DOI 10.1093/ajh/hpu071
Gettlert LT, 2011, AM J PHYS ANTHROPOL, V144, P454, DOI 10.1002/ajpa.21426
GOLD DR, 1992, AM J PUBLIC HEALTH, V82, P1011, DOI 10.2105/AJPH.82.7.1011
GOODALL JM, 1962, ANN NY ACAD SCI, V102, P455, DOI 10.1111/j.1749-6632.1962.tb13652.x
Grandner MA, 2013, J CLIN SLEEP MED, V9, P897, DOI 10.5664/jcsm.2990
Grandner MA, 2012, BEHAV SLEEP MED, V10, P235, DOI 10.1080/15402002.2012.654548
Hale L, 2007, SLEEP, V30, P1096, DOI 10.1093/sleep/30.9.1096
Han F, 2011, ANN NEUROL, V70, P410, DOI 10.1002/ana.22587
Haus EL, 2013, SLEEP MED REV, V17, P273, DOI 10.1016/j.smrv.2012.08.003
Hewlett B.S., 2014, Ancestral landscapes in human evolution, P129
HEYMANN EW, 1995, J ZOOL, V237, P211, DOI 10.1111/j.1469-7998.1995.tb02759.x
Hill TD, 2016, SLEEP MED, V18, P56, DOI 10.1016/j.sleep.2014.12.003
Jones CR, 2013, EXP NEUROL, V243, P28, DOI 10.1016/j.expneurol.2012.07.012
JONES NGB, 1987, ETHOL SOCIOBIOL, V8, P135
Kappeler PM, 1998, AM J PRIMATOL, V46, P7
Knutson KL, 2014, AM J HUM BIOL, V26, P80, DOI 10.1002/ajhb.22481
Koops K, 2007, AM J PRIMATOL, V69, P407, DOI 10.1002/ajp.20358
Koops K, 2012, AM J PHYS ANTHROPOL, V148, P351, DOI 10.1002/ajpa.22056
Kornum BR, 2011, NAT GENET, V43, P66, DOI 10.1038/ng.734
Krystal AD, 2007, J CLIN SLEEP MED, V3, P63
Lam RW, 2000, J PSYCHIATR NEUROSCI, V25, P469

Lehnkering H, 2007, CHRONOBIOL INT, V24, P875, DOI 10.1080/07420520701648259
Lesku JA, 2008, SLEEP MED REV, V12, P229, DOI 10.1016/j.smrv.2007.10.003
Lesku JA, 2006, AM NAT, V168, P441, DOI 10.1086/506973
Lesku JA, 2012, SCIENCE, V337, P1654, DOI 10.1126/science.1220939
Lima SL, 2007, ANIM BEHAV, V74, P189, DOI 10.1016/j.anbehav.2006.12.007
Loredo JS, 2010, SLEEP, V33, P962, DOI 10.1093/sleep/33.7.962
Lyamin OI, 2008, NEUROSCI BIOBEHAV R, V32, P1451, DOI 10.1016/j.neubiorev.2008.05.023
Maestripieri D, 2014, EVOL PSYCHOL-US, V12, P130
Martin-Ordas G, 2011, BIOL LETTERS, V7, P829, DOI 10.1098/rsbl.2011.0437
MCKENNA J, 1994, EARLY HUM DEV, V38, P187, DOI 10.1016/0378-3782(94)90211-9
McKenna J, 1999, EVOLUTIONARY MEDICI NE, P53
McKenna JJ, 2007, YEARB PHYS ANTHROPOL, V50, P133, DOI 10.1002/ajpa.20736
MCKENNA JJ, 1993, SLEEP, V16, P263, DOI 10.1093/sleep/16.3.263
McNamara P., 2010, EVOLUTION SLEEP PHYL, P107
MEDDIS R, 1975, ANIM BEHAV, V23, P676, DOI 10.1016/0003-3472(75)90144-X
Meddis R., 1983, SLEEP MECH FUNCTIONS, P57
Mignot E, 1998, NEUROLOGY, V50, pS16, DOI 10.1212/WNL.50.2_Suppl_1.S16
Mitani Y, 2010, BIOL LETTERS, V6, P163, DOI 10.1098/rsbl.2009.0719
Mizuno Y, 2006, INFANCY, V9, P221, DOI 10.1207/s15327078in0902_7
Moore SJ, 2007, J MED ENTOMOL, V44, P624, DOI 10.1603/0022-2585(2007)44[624:FEOTUP]2.0.CO;2
Nunn C.L., 2014, Modern Phylogenetic Comparative Methods and Their Application in Evolutionary Biology, P481
Nunn Charles L., 2002, P159
Nunn Charles L., 2000, P388, DOI 10.1017/CBO9780511542312.018
Nunn CharlesL., 2011, COMP APPROACH EVOLUT
Ohayon MM, 2010, J PSYCHOSOM RES, V69, P565, DOI 10.1016/j.jpsychores.2010.03.010
Ohayon MM, 2010, SLEEP MED, V11, P236, DOI 10.1016/j.sleep.2009.11.004
Ohayon MM, 2009, SLEEP MED, V10, P952, DOI 10.1016/j.sleep.2009.07.008
Overeem Sebastiaan, 2002, Sleep Med, V3, P471, DOI 10.1016/S1389-9457(02)00037-0
Partinen M, 2014, LANCET NEUROL, V13, P600, DOI 10.1016/S1474-4422(14)70075-4
Patel NP, 2010, BMC PUBLIC HEALTH, V10, DOI 10.1186/1471-2458-10-475
Paul MA, 2015, PHYSIOL BEHAV, V141, P199, DOI 10.1016/j.physbeh.2015.01.021
Perlman RL, 2013, Evolution and Medicine, P1, DOI 10.1093/acprof:oso/9780199661718.001.0001
Prather AA, 2015, SLEEP, V38, P1353, DOI 10.5665/sleep.4968
Preston BT, 2009, BMC EVOL BIOL, V9, DOI 10.1186/1471-2148-9-7
Pruetz JD, 2008, AM J PRIMATOL, V70, P393, DOI 10.1002/ajp.20508
Randler C, 2014, ANIM BEHAV, V94, P161, DOI 10.1016/j.anbehav.2014.05.001
Reichard U, 1998, AM J PRIMATOL, V46, P35, DOI 10.1002/(SICI)1098-2345(1998)46:1<35::AID-AJP4>3.0.CO;2-W

Revonsuo A, 2000, BEHAV BRAIN SCI, V23, P877, DOI 10.1017/S0140525X00004015
ROFFWARG HP, 1966, SCIENCE, V152, P604, DOI 10.1126/science.152.3722.604
Rosekind MR, 2010, J OCCUP ENVIRON MED, V52, P91, DOI 10.1097/JOM.0b013e3181c78c30
Rosekind MR, 2005, SLEEP MED, V6, pS21, DOI 10.1016/S1389-9457(05)80005-X
ROSENTHAL NE, 1987, PSYCHIAT ANN, V17, P670, DOI 10.3928/0048-5713-19871001-10
Samson DR, 2015, EVOL ANTHROPOL, V24, P225, DOI 10.1002/evan.21464
Samson DR, 2014, PLOS ONE, V9, DOI 10.1371/journal.pone.0095361
Samson DR, 2013, BEHAVIOUR, V150, P845, DOI 10.1163/1568539X-00003082
Samson DR, 2013, PRIMATES, V54, P73, DOI 10.1007/s10329-012-0329-z
Samson DR, 2012, PRIMATES, V53, P357, DOI 10.1007/s10329-012-0310-x
Samson DR, 2012, AM J PRIMATOL, V74, P811, DOI 10.1002/ajp.22031
Singh AK, 2013, J AUTOIMMUN, V43, P26, DOI 10.1016/j.jaut.2013.02.003
Sivertsen B, 2011, AM J EPIDEMIOL, V174, P147, DOI 10.1093/aje/kwr052
Slopen N, 2016, SLEEP MED, V18, P88, DOI 10.1016/j.sleep.2015.01.012
Stearns S.C., 1999, Evolution in health and disease
Stewart FA, 2011, AM J PHYS ANTHROPOL, V146, P313, DOI 10.1002/ajpa.21580
Stranges S, 2012, SLEEP, V35, P1173, DOI 10.5665/sleep.2012
TAKAHASHI Y, 1968, J CLIN INVEST, V47, P2079, DOI 10.1172/JCI105893
Tsoukalas I, 2012, DREAMING, V22, P253, DOI 10.1037/a0030790
van Casteren A, 2013, PLOS ONE, V8, DOI 10.1371/journal.pone.0067877
van Casteren A, 2012, P NATL ACAD SCI USA, V109, P6873, DOI 10.1073/pnas.1200902109
van Lawick-Goodall J., 1968, Animal Behaviour Monographs, V1, P161
Van Veen MM, 2010, BIOL PSYCHIAT, V67, P1091, DOI 10.1016/j.biopsych.2009.12.032
Vennemann M M, 2009, Pediatrics, V123, pe406, DOI 10.1542/peds.2008-2145
Vincent F., 1978, P181
Walch OJ, 2016, SCI ADV, V2, DOI 10.1126/sciadv.1501705
WEHR TA, 1992, J SLEEP RES, V1, P103, DOI 10.1111/j.1365-2869.1992.tb00019.x
Wehr TA, 1999, LUNG BIOL HEALTH DIS, V133, P263
WEITZMAN ED, 1975, ACTA ENDOCRINOL-COP, V78, P65, DOI 10.1530/acta.0.0780065
Wilson HJ, 2015, AM J PHYS ANTHROPOL, V157, P641, DOI 10.1002/ajpa.22761
Worthman C.M., 2002, ADOLESCENT SLEEP PAT, P69, DOI [DOI 10.1017/CBO9780511499999.009, DOI 10.1017/CBO9780511499999]
Worthman CM, 2013, SOC SCI MED, V79, P31, DOI 10.1016/j.socscimed.2012.03.048
Worthman CM., 2008, DARK EVOLUTIONARY EC, P291
Wrangham R, 2010, EVOL ANTHROPOL, V19, P187, DOI 10.1002/evan.20275
Wu MC, 2012, PREV MED, V55, P305, DOI 10.1016/j.ypmed.2012.07.013
Xie LL, 2013, SCIENCE, V342, P373, DOI 10.1126/science.1241224
Yamagiwa J, 2001, J HUM EVOL, V40, P99, DOI 10.1006/jhev.2000.0444
Yetish G, 2015, CURR BIOL, V25, P2862, DOI 10.1016/j.cub.2015.09.046
Young T, 2004, JAMA-J AM MED ASSOC, V291, P2013, DOI 10.1001/jama.291.16.2013

ZEPELIN H, 1974, BRAIN BEHAV EVOLUT, V10, P425, DOI 10.1159/000124330
NR 143
TC 54
Z9 64
U1 4
U2 42
PU OXFORD UNIV PRESS
PI OXFORD
PA GREAT CLARENDON ST, OXFORD OX2 6DP, ENGLAND
EI 2050-6201
J9 EVOL MED PUBLIC HLTH
JI Evol. Med. Public Health
PY 2016
IS 1
BP 227
EP 243
DI 10.1093/emph/eow018
PG 17
WC Evolutionary Biology; Public, Environmental & Occupational Health
WE Science Citation Index Expanded (SCI-EXPANDED)
SC Evolutionary Biology; Public, Environmental & Occupational Health
GA DZ5TY
UT WOS:000385925600023
PM 27470330
OA Green Published, Green Submitted, gold
DA 2025-05-07
ER

PT J
AU Reser, JE
Reser, WW
AF Reser, Jared E.
Reser, William W.
TI Does rheumatoid arthritis represent an adaptive, thrifty condition?
SO MEDICAL HYPOTHESES
LA English
DT Article
ID ADRENAL STRESS AXIS; DIABETES-MELLITUS; RISK-FACTORS; EVOLUTION;
PREVALENCE; DEPRESSION; PREGNANCY; INDIANS; PAIN
AB The present article presents epidemiological, and comparative evidence Supporting the hypothesis that rheumatoid arthritis (RA) may represent a thrifty adaptation selected to compel animals to minimize voluntary energy expenditure. The autoimmune, pathophysiological manifestations underlying RA are framed here as constituting an evolved, protective mechanism that would have influenced animals to avoid exertion and maintain a sedentary lifestyle in order to minimize metabolic output and ultimately escape starvation. Arthritic pain is characterized here as a defensive, innate signal much like fatigue, fever, nausea and reflexive pain, and like these, is seen on a continuum varying between imperceptible encumbrance and debilitating disability. The epigenetic relationship between acute psychological stress and flare-up of arthritic symptoms is examined and taken to suggest that arthritis may be a predictive, adaptive response to severe stress allowing reductions in metabolism to follow adverse conditions or nutritional scarcity. The

close associations between rheumatoid arthritis and the metabolic syndrome are also explored along with potential ties to the "thrifty genotype" and "thrifty phenotype" phenomena. This hypothesis is examined in the contexts of evolutionary medicine, phenotypic plasticity, the stress response and the bioenergetics of thrift. A brief and exploratory review of pertinent evidence suggests that RA, its subclinical manifestations, and even other forms of arthropathy may possibly represent adaptations that promoted metabolic thrift during our evolutionary past. (C) 2009 Elsevier Ltd. All rights reserved.
C1 [Reser, Jared E.] Univ So Calif, Encino, CA 91436 USA.
[Reser, William W.] Univ Calif Davis, Davis, CA 95616 USA.
C3 University of Southern California; University of California System; University of California Davis
RP Reser, JE (autor correspondente), Univ So Calif, 16380 Meadow Ridge Rd, Encino, CA 91436 USA.
EM jared@jaredreser.com; williamwreser@sbcglobal.net
OI Reser, Jared/0000-0003-0580-3403
CR Bateson P, 2004, NATURE, V430, P419, DOI 10.1038/nature02725
Bobe R, 2002, J HUM EVOL, V42, P475, DOI 10.1006/jhev.2001.0535
Chung CP, 2008, ATHEROSCLEROSIS, V196, P756, DOI 10.1016/j.atherosclerosis.2007.01.004
Crofford LJ, 1998, Z RHEUMATOL, V57, P67
De Man YA, 2008, ARTHRIT RHEUM-ARTHR, V59, P1241, DOI 10.1002/art.24003
DELPUENTE A, 1989, AM J EPIDEMIOL, V129, P1170
DeWitt TJ, 2004, PHENOTYPIC PLASTICIT
Diamond J, 2003, NATURE, V423, P599, DOI 10.1038/423599a
Dickens C, 2002, PSYCHOSOM MED, V64, P52, DOI 10.1097/00006842-200201000-00008
EKDAHL C, 1992, ANN RHEUM DIS, V51, P35, DOI 10.1136/ard.51.1.35
Ferucci ED, 2005, SEMIN ARTHRITIS RHEU, V34, P662, DOI 10.1016/j.semarthrit.2004.08.003
FRANK RG, 1988, J RHEUMATOL, V15, P920
Gio-Fitman J, 1996, Medsurg Nurs, V5, P422
Grossman JM, 1997, J WOMENS HEALTH, V6, P627, DOI 10.1089/jwh.1997.6.627
HALES CN, 1992, DIABETOLOGIA, V35, P595, DOI 10.1093/ije/dyt133
Hales CN, 2001, BRIT MED BULL, V60, P5, DOI 10.1093/bmb/60.1.5
Heininger K, 2002, AGEING RES REV, V1, P481, DOI 10.1016/S1568-1637(02)00015-6
Hill K.R., 1996, Ache life history: The ecology and demography of a foraging people
JONES NGB, 1992, AM J PHYS ANTHROPOL, V89, P159, DOI 10.1002/ajpa.1330890204
Kaplan H, 2000, EVOL ANTHROPOL, V9, P156, DOI 10.1002/1520-6505(2000)9:4<156::AID-EVAN5>3.0.CO;2-7
KAPLAN H, 2003, POPUL DEV REV, V2
Khasar SG, 2008, J NEUROSCI, V28, P5721, DOI 10.1523/JNEUROSCI.0256-08.2008
Majithia V, 2007, AM J MED, V120, P936, DOI 10.1016/j.amjmed.2007.04.005
Mastorakos G, 2000, ANN NY ACAD SCI, V900, P95
McBeth J, 2007, ARTHRITIS RHEUM-US, V56, P360, DOI 10.1002/art.22336
McBeth J, 2005, ARTHRITIS RES THER, V7, pR992, DOI 10.1186/ar1772

NEEL JV, 1962, AM J HUM GENET, V14, P353
Nesse R.M., 1995, WHY WE GET SICK
Nesse RM, 1998, SCI AM, V279, P86, DOI 10.1038/scientificamerican1198-86
Nesse RM., 1999, EVOLUTIONARY MED
Noll RB, 2000, ARTHRITIS RHEUM, V43, P1387, DOI 10.1002/1529-0131(200006)43:6<1387::AID-ANR24>3.0.CO;2-C
Nunn CL, 2007, J EVOLUTION BIOL, V20, P460, DOI 10.1111/j.1420-9101.2006.01276.x
Parker JC, 1995, ARTHRITIS RHEUM-US, V38, P1807, DOI 10.1002/art.1780381214
Pigliucci M., 2001, SYN ECO EVO, P306
Rall LC, 2004, RHEUMATOLOGY, V43, P1219, DOI 10.1093/rheumatology/keh321
Ravelo AC, 2004, NATURE, V429, P263, DOI 10.1038/nature02567
Reed KE, 1997, J HUM EVOL, V32, P289, DOI 10.1006/jhev.1996.0106
Roubenoff R, 2002, AM J CLIN NUTR, V76, P774, DOI 10.1093/ajcn/76.4.774
Sánchez-Pernaute O, 2008, J AUTOIMMUN, V30, P12, DOI 10.1016/j.jaut.2007.11.006
Silman AJ, 2002, ARTHRITIS RES THER, V4, pS265, DOI 10.1186/ar578
Straub RH, 2003, FASEB J, V17, P2176, DOI 10.1096/fj.03-0433hyp
Turesson C, 2003, ANN RHEUM DIS, V62, P722, DOI 10.1136/ard.62.8.722
Valencia ME, 1999, NUTR REV, V57, pS55, DOI 10.1111/j.1753-4887.1999.tb01789.x
Vanden Berghe W, 2006, BIOCHEM PHARMACOL, V72, P1114, DOI 10.1016/j.bcp.2006.07.012
VIA S, 1985, EVOLUTION, V39, P505, DOI 10.1111/j.1558-5646.1985.tb00391.x
Vierboom MPM, 2005, ARTHRITIS RES THER, V7, P145, DOI 10.1186/ar1773
Villamor E, 2009, AM J EPIDEMIOL, V169, P1337, DOI 10.1093/aje/kwp054
Walker JG, 1999, RHEUMATOLOGY, V38, P1050, DOI 10.1093/rheumatology/38.11.1050
Wells JCK, 2003, J THEOR BIOL, V221, P143, DOI 10.1006/jtbi.2003.3183
WILLIAMS DRR, 1993, EYE, V7, P202, DOI 10.1038/eye.1993.48
WILLIAMS GC, 1991, Q REV BIOL, V66, P1, DOI 10.1086/417048
Zafon C, 2006, MED HYPOTHESES, V67, P15, DOI 10.1016/j.mehy.2006.01.055
NR 52
TC 3
Z9 3
U1 0
U2 12
PU CHURCHILL LIVINGSTONE
PI EDINBURGH
PA JOURNAL PRODUCTION DEPT, ROBERT STEVENSON HOUSE, 1-3 BAXTERS PLACE, LEITH WALK, EDINBURGH EH1 3AF, MIDLOTHIAN, SCOTLAND
SN 0306-9877
EI 1532-2777
J9 MED HYPOTHESES
JI Med. Hypotheses
PD JAN
PY 2010
VL 74

IS 1
BP 189
EP 194
DI 10.1016/j.mehy.2009.07.041
PG 6
WC Medicine, Research & Experimental
WE Science Citation Index Expanded (SCI-EXPANDED); Social Science
Citation Index (SSCI)
SC Research & Experimental Medicine
GA 547VU
UT WOS:000273918500054
PM 19716239
DA 2025-05-07
ER

PT J
AU Reyes-Contreras, M
Glauser, G
Rennison, DJ
Taborsky, B
AF Reyes-Contreras, Maria
Glauser, Gaetan
Rennison, Diana J.
Taborsky, Barbara
TI Early-life manipulation of cortisol and its receptor alters stress axis
programming and social competence
SO PHILOSOPHICAL TRANSACTIONS OF THE ROYAL SOCIETY B-BIOLOGICAL SCIENCES
LA English
DT Article
DE early-life effects; corticosteroid receptor; developmental plasticity;
HPA/HPI axis; mifepristone; cichlids
ID PITUITARY-ADRENAL AXIS; MULTIPLE CORTICOSTEROID RECEPTORS; CICHLID FISH;
GLUCOCORTICOID-RECEPTORS; NEOLAMPROLOGUS-PULCHER; BROODCARE HELPERS;
ALLOSTATIC LOAD; RAINBOW-TROUT; MESSENGER-RNA; MATERNAL-CARE
AB In many vertebrate species, early social experience generates long-term effects on later life social behaviour. These effects are accompanied by persistent modifications in the expression of genes implicated in the stress axis. It is unknown, however, whether stress axis programming can affect the development of social competence, and if so, by which mechanism(s). Here, we used pharmacological manipulations to persistently reprogramme the hypothalamic-pituitary-interrenal axis of juvenile cooperatively breeding cichlids, Neolamprologus pulcher. During the first two months of life, juveniles were repeatedly treated with cortisol, mifepristone or control treatments. Three months after the last manipulation, we tested for treatment effects on (i) social competence, (ii) the expression of genes coding for corticotropin-releasing factor (crf), glucocorticoid receptor (gr1) and mineralocorticoid receptor (mr) in the telencephalon and hypothalamus and (iii) cortisol levels. Social competence in a social challenge was reduced in cortisol-treated juveniles, which is in accordance with previous work applying early-life manipulations using different social experiences. During early life, both cortisol and mifepristone treatments induced a persistent downregulation

of crf and upregulation of mr in the telencephalon. We suggest that these persistent changes in stress gene expression may represent an effective physiological mechanism for coping with stress. This article is part of the theme issue 'Developing differences: early-life effects and evolutionary medicine'.
C1 [Reyes-Contreras, Maria; Taborsky, Barbara] Univ Bern, Inst Ecol & Evolut, Div Behav Ecol, Wohlenstr 50A, CH-3032 Hinterkappelen, Switzerland.
[Glauser, Gaetan] Univ Neuchatel, Inst Chem, Neuchatel Platform Analyt Chem, Ave Bellevaux 51, CH-2009 Neuchatel, Switzerland.
[Rennison, Diana J.] Univ Bern, Inst Ecol & Evolut, Div Evolutionary Ecol, Baltzerstr 6, CH-3012 Bern, Switzerland.
C3 University of Bern; University of Neuchatel; University of Bern
RP Reyes-Contreras, M (autor correspondente), Univ Bern, Inst Ecol & Evolut, Div Behav Ecol, Wohlenstr 50A, CH-3032 Hinterkappelen, Switzerland.
EM maria.reyes-contreras@iee.unibe.ch
RI ; Glauser, Gaetan/K-7574-2017
OI Reyes Contreras, Maria/0000-0002-0862-7436; Taborsky, Barbara/0000-0003-1690-8155; Rennison, Diana/0000-0002-5944-0743; Glauser, Gaetan/0000-0002-0983-8614
FU Swiss National Science Foundation [31003A_156881]; Swiss National Science Foundation (SNF) [31003A_156881] Funding Source: Swiss National
Science Foundation (SNF)
FX B.T. acknowledges financial support by the Swiss National Science Foundation (SNSF; grant 31003A_156881).
CR Adkins-Regan E, 2000, ANIM BEHAV, V60, P47, DOI 10.1006/anbe.2000.1448
Alderman SL, 2012, GEN COMP ENDOCR, V176, P79, DOI 10.1016/j.ygcen.2011.12.031
Archard GA, 2012, FUNCT ECOL, V26, P637, DOI 10.1111/j.1365-2435.2012.01968.x
Arnold C, 2010, ANIM BEHAV, V79, P621, DOI 10.1016/j.anbehav.2009.12.008
Auperin B, 2008, GEN COMP ENDOCR, V158, P234, DOI 10.1016/j.ygcen.2008.07.002
Backström T, 2013, FRONT NEUROSCI-SWITZ, V7, DOI 10.3389/fnins.2013.00117
Banerjee SB, 2012, P ROY SOC B-BIOL SCI, V279, P759, DOI 10.1098/rspb.2011.1265
Bannier F, 2017, ETHOLOGY, V123, P712, DOI 10.1111/eth.12646
Barton BA, 2002, INTEGR COMP BIOL, V42, P517, DOI 10.1093/icb/42.3.517
Bates D, 2015, J STAT SOFTW, V67, P1, DOI 10.18637/jss.v067.i01
Bender N, 2008, PHYSIOL BEHAV, V94, P349, DOI 10.1016/j.physbeh.2008.02.002
Bender N, 2006, HORM BEHAV, V50, P173, DOI 10.1016/j.yhbeh.2006.02.009
Bergmüller R, 2005, P ROY SOC B-BIOL SCI, V272, P325, DOI 10.1098/rspb.2004.2960
Bergmüller R, 2005, ANIM BEHAV, V69, P19, DOI 10.1016/j.anbehav.2004.05.009
Bernier NJ, 2009, FISH PHYSIOL, V28, P235, DOI 10.1016/S1546-5098(09)28006-X
Bernier NJ, 1999, GEN COMP ENDOCR, V116, P461, DOI 10.1006/gcen.1999.7386

Blüthgen N, 2013, AQUAT TOXICOL, V144, P96, DOI 10.1016/j.aquatox.2013.09.030
Bolker BM, 2009, TRENDS ECOL EVOL, V24, P127, DOI 10.1016/j.tree.2008.10.008
Bommae K., 2015, Understanding Diagnostic Plots for Linear Regression Analysis
Boulton K, 2015, ECOL EVOL, V5, P618, DOI 10.1002/ece3.1395
Branchi I, 2006, BIOL PSYCHIAT, V60, P690, DOI 10.1016/j.biopsych.2006.01.005
Branchi I, 2013, PSYCHONEUROENDOCRINO, V38, P522, DOI 10.1016/j.psyneuen.2012.07.010
Bruintjes R, 2011, ANIM BEHAV, V81, P387, DOI 10.1016/j.anbehav.2010.10.004
Cant MA, 2010, P ROY SOC B-BIOL SCI, V277, P2219, DOI 10.1098/rspb.2009.2097
Castanheira MF, 2013, APPL ANIM BEHAV SCI, V143, P75, DOI 10.1016/j.applanim.2012.11.008
Crawley M. J., 2013, R BOOK
Curley JP, 2011, PSYCHONEUROENDOCRINO, V36, P352, DOI 10.1016/j.psyneuen.2010.06.005
de Kloet ER, 2013, J CHEM NEUROANAT, V54, P42, DOI 10.1016/j.jchemneu.2013.10.003
Emmerson MG, 2017, HORM BEHAV, V90, P48, DOI 10.1016/j.yhbeh.2017.02.004
Engqvist L, 2005, ANIM BEHAV, V70, P967, DOI 10.1016/j.anbehav.2005.01.016
Field J, 2009, PHILOS T R SOC B, V364, P3181, DOI 10.1098/rstb.2009.0110
Fischer S, 2017, P NATL ACAD SCI USA, V114, pE9300, DOI 10.1073/pnas.1705934114
Fischer S, 2015, AM NAT, V186, P123, DOI 10.1086/681636
Fischer S, 2014, P ROY SOC B-BIOL SCI, V281, DOI 10.1098/rspb.2014.0184
Fox J., 2011, An R companion to applied regression, V2
Goymann W, 2004, ANIM BEHAV, V67, P591, DOI 10.1016/j.anbehav.2003.08.007
Grace JK, 2017, PHYSIOL BEHAV, V177, P82, DOI 10.1016/j.physbeh.2017.04.018
Grantner A, 1998, J COMP PHYSIOL B, V168, P427, DOI 10.1007/s003600050162
Greenwood AK, 2003, ENDOCRINOLOGY, V144, P4226, DOI 10.1210/en.2003-0566
Groothuis TGG, 2015, FRONT ZOOL, V12, DOI 10.1186/1742-9994-12-S1-S6
Heg D, 2004, P ROY SOC B-BIOL SCI, V271, P2367, DOI 10.1098/rspb.2004.2855
Hess S, 2016, ANIM BEHAV, V113, P229, DOI 10.1016/j.anbehav.2016.01.008
Hillegass JM, 2007, TOXICOL SCI, V100, P168, DOI 10.1093/toxsci/kfm192
Hothorn T, 2008, BIOMETRICAL J, V50, P346, DOI 10.1002/bimj.200810425
IMAKI T, 1991, J NEUROSCI, V11, P585
Jeffrey JD, 2014, GEN COMP ENDOCR, V196, P8, DOI 10.1016/j.ygcen.2013.11.010
Joels M, 2008, TRENDS NEUROSCI, V31, P1, DOI 10.1016/j.tins.2007.10.005

Joëls M, 2006, TRENDS PHARMACOL SCI, V27, P244, DOI 10.1016/j.tips.2006.03.007
Kalafatakis K, 2016, NEUROSCI BIOBEHAV R, V61, P12, DOI 10.1016/j.neubiorev.2015.11.009
Kasper C, 2018, PHYSIOL BEHAV, V195, P37, DOI 10.1016/j.physbeh.2018.07.025
Kasper C, 2017, MOL ECOL, V26, P4364, DOI 10.1111/mec.14208
Korte SM, 2005, NEUROSCI BIOBEHAV R, V29, P3, DOI 10.1016/j.neubiorev.2004.08.009
Maccari S, 2014, J NEUROENDOCRINOL, V26, P707, DOI 10.1111/jne.12175
Marasco V, 2016, J NEUROENDOCRINOL, V28, DOI 10.1111/jne.12387
Marasco V, 2012, J EXP BIOL, V215, P3955, DOI 10.1242/jeb.071423
Meaney MJ, 2005, TRENDS NEUROSCI, V28, P456, DOI 10.1016/j.tins.2005.07.006
Medeiros LR, 2013, COMP BIOCHEM PHYS A, V164, P612, DOI 10.1016/j.cbpa.2013.01.014
Mileva VR, 2009, PHYSIOL BIOCHEM ZOOL, V82, P720, DOI 10.1086/605937
Silva PIM, 2010, APPL ANIM BEHAV SCI, V124, P75, DOI 10.1016/j.applanim.2010.01.008
Nyman C, 2018, P ROY SOC B-BIOL SCI, V285, DOI 10.1098/rspb.2017.2344
Nyman C, 2017, MOL ECOL, V26, P3186, DOI 10.1111/mec.14113
O'Connell LA, 2012, SCIENCE, V336, P1154, DOI 10.1126/science.1218889
O'Connell LA, 2011, FRONT NEUROENDOCRIN, V32, P320, DOI 10.1016/j.yfrne.2010.12.004
O'Regan D, 2001, Semin Neonatol, V6, P319, DOI 10.1053/siny.2001.0067
Oliveira RF, 2009, INTEGR COMP BIOL, V49, P423, DOI 10.1093/icb/icp055
Overli O, 2004, HORM BEHAV, V45, P324, DOI 10.1016/j.yhbeh.2004.01.001
Pakkala JJ, 2016, FUNCT ECOL, V30, P1149, DOI 10.1111/1365-2435.12603
Panchanathan K, 2016, P ROY SOC B-BIOL SCI, V283, DOI 10.1098/rspb.2015.2439
Pavlidis M, 2011, BEHAV BRAIN RES, V225, P529, DOI 10.1016/j.bbr.2011.08.022
Pepels PPLM, 2004, J ENDOCRINOL, V180, P425, DOI 10.1677/joe.0.1800425
Prunet P, 2006, GEN COMP ENDOCR, V147, P17, DOI 10.1016/j.ygcen.2006.01.015
Raulo A, 2018, ECOL EVOL, V8, P7697, DOI 10.1002/ece3.4059
Ros AFH, 2012, HORM BEHAV, V61, P37, DOI 10.1016/j.yhbeh.2011.09.013
Sandi C, 2015, NAT REV NEUROSCI, V16, P290, DOI 10.1038/nrn3918
Sapolsky RM, 2000, ENDOCR REV, V21, P55, DOI 10.1210/er.21.1.55
Schjolden J, 2009, PHYSIOL BEHAV, V98, P625, DOI 10.1016/j.physbeh.2009.09.018
Scott AP, 2007, GEN COMP ENDOCR, V153, P392, DOI 10.1016/j.ygcen.2006.11.006
Shackelford T.K., 2017, Encyclopedia of Evolutionary Psychological Science, DOI [DOI 10.1007/978-3-319-19650-3, 10.1007/978-3-319-16999-6_1687-1, 10.1007/978-3-319-16999-61687-1, DOI 10.1007/978-3-319-16999-61687-1]
Sloman KA, 2010, HORM BEHAV, V58, P433, DOI 10.1016/j.yhbeh.2010.05.010
Sopinka NM, 2015, ANIM BEHAV, V104, P115, DOI 10.1016/j.anbehav.2015.03.011
Spencer KA, 2007, HORM BEHAV, V51, P273, DOI 10.1016/j.yhbeh.2006.11.001
Spencer KA, 2017, PHILOS T R SOC B, V372, DOI 10.1098/rstb.2016.0242

Stiver KA, 2004, J FISH BIOL, V65, P91, DOI 10.1111/j.0022-1112.2004.00427.x
Stolte EH, 2008, MOL IMMUNOL, V46, P70, DOI 10.1016/j.molimm.2008.07.022
Taborsky B, 2016, ETHOLOGY, V122, P267, DOI 10.1111/eth.12473
Taborsky B, 2013, P ROY SOC B-BIOL SCI, V280, DOI 10.1098/rspb.2012.2605
Taborsky B, 2012, TRENDS ECOL EVOL, V27, P679, DOI 10.1016/j.tree.2012.09.003
Taborsky B, 2012, ANIM BEHAV, V83, P1067, DOI 10.1016/j.anbehav.2012.01.037
TABORSKY M, 1984, ANIM BEHAV, V32, P1236, DOI 10.1016/S0003-3472(84)80241-9
TABORSKY M, 1985, BEHAVIOUR, V95, P45, DOI 10.1163/156853985X00046
Tsalafouta A, 2014, SCI REP-UK, V4, DOI 10.1038/srep05525
Veenit V, 2013, FRONT BEHAV NEUROSCI, V7, DOI 10.3389/fnbeh.2013.00026
Veillette PA, 2007, GEN COMP ENDOCR, V154, P105, DOI 10.1016/j.ygcen.2007.05.033
Veillette PA, 2007, GEN COMP ENDOCR, V151, P116, DOI 10.1016/j.ygcen.2006.12.019
Venables WN., 2002, Modern applied statistics with s, V4, DOI 10.1007/978-0-387-21706-2
Vindas MA, 2018, SCI REP-UK, V8, DOI 10.1038/s41598-018-23950-x
Welberg LAM, 2001, NEUROSCIENCE, V104, P71, DOI 10.1016/S0306-4522(01)00065-3
Wommack JC, 2007, HORM BEHAV, V51, P306, DOI 10.1016/j.yhbeh.2006.11.007
Wong MYL, 2012, BEHAV ECOL, V23, P521, DOI 10.1093/beheco/arr218
Wong SC, 2008, BEHAVIOUR, V145, P1283, DOI 10.1163/156853908785765863
Wooddell LJ, 2017, PSYCHONEUROENDOCRINO, V84, P109, DOI 10.1016/j.psyneuen.2017.06.018
Woodruff JA, 2013, HORM BEHAV, V64, P566, DOI 10.1016/j.yhbeh.2013.07.008
Zalachoras I, 2013, P NATL ACAD SCI USA, V110, P7910, DOI 10.1073/pnas.1219411110
Zimmer C, 2014, J NEUROENDOCRINOL, V26, P853, DOI 10.1111/jne.12228
Zimmer C, 2017, SCI REP-UK, V7, DOI 10.1038/srep46125
Ziv L, 2013, MOL PSYCHIATR, V18, P681, DOI 10.1038/mp.2012.64
Zöttl M, 2013, NAT COMMUN, V4, DOI 10.1038/ncomms2344
NR 107
TC 30
Z9 30
U1 0
U2 36
PU ROYAL SOC
PI LONDON
PA 6-9 CARLTON HOUSE TERRACE, LONDON SW1Y 5AG, ENGLAND
SN 0962-8436
EI 1471-2970
J9 PHILOS T R SOC B
JI Philos. Trans. R. Soc. B-Biol. Sci.
PD APR 15
PY 2019
VL 374

IS 1770
AR 20180119
DI 10.1098/rstb.2018.0119
PG 13
WC Biology
WE Science Citation Index Expanded (SCI-EXPANDED)
SC Life Sciences & Biomedicine - Other Topics
GA HN8ZI
UT WOS:000460487000010
PM 30966879
OA Green Published, Bronze
DA 2025-05-07
ER

PT J
AU Sear, R
   Sheppard, P
   Coall, DA
AF Sear, Rebecca
   Sheppard, Paula
   Coall, David A.
TI Cross-cultural evidence does not support universal acceleration of
   puberty in father-absent households
SO PHILOSOPHICAL TRANSACTIONS OF THE ROYAL SOCIETY B-BIOLOGICAL SCIENCES
LA English
DT Article
DE puberty; father absence; cross-cultural; menarche; family structure;
   early-life environment
ID EARLY-CHILDHOOD MALTREATMENT; EARLY-LIFE CONDITIONS; REPRODUCTIVE
   SUCCESS; EVOLUTIONARY PERSPECTIVE; MEDIATING ROLE; 1ST BIRTH; AGE;
   FAMILY; MENARCHE; HISTORY
AB Father absence in early life has been shown to be associated with
accelerated reproductive development in girls. Evolutionary social
scientists have proposed several adaptive hypotheses for this finding.
Though there is variation in the detail of these hypotheses, they all
assume that family environment in early life influences the development
of life-history strategy, and, broadly, that early reproductive
development is an adaptive response to father absence. Empirical evidence
to support these hypotheses, however, has been derived from WEIRD
(Western, Educated, Industrialized, Rich and Democratic) populations.
Data from a much broader range of human societies are necessary in order
to properly test adaptive hypotheses. Here, we review the empirical
literature on father absence and puberty in both sexes, focusing on
recent studies that have tested this association beyond the WEIRD world.
We find that relationships between father absence and age at puberty are
more varied in contexts beyond WEIRD societies, and when relationships
beyond the father-daughter dyad are considered. This has implications for
our understanding of how early-life environment is linked to life-history
strategies, and for our understanding of pathways to adult health
outcomes, given that early reproductive development may be linked to
negative health outcomes in later life
   This article is part of the theme issue 'Developing differences:
early-life effects and evolutionary medicine'.

C1 [Sear, Rebecca] London Sch Hyg & Trop Med, Populat Hlth, London, England.
[Sheppard, Paula] Univ Oxford, Dept Sociol, Oxford, England.
[Coall, David A.] Edith Cowan Univ, Med & Hlth Sci, Joondalup, Australia.
C3 University of London; London School of Hygiene & Tropical Medicine; University of Oxford; Edith Cowan University
RP Sear, R (autor correspondente), London Sch Hyg & Trop Med, Populat Hlth, London, England.
EM rebecca.sear@lshtm.ac.uk
RI Coall, David/AAR-4761-2020; Sear, Rebecca/K-9616-2019
OI Coall, David A./0000-0002-0488-2683; Sear, Rebecca/0000-0002-4315-0223
FU Edith Cowan University; European Research Council [StG-2010 263760-FAMMAT]
FX This study was funded by Edith Cowan University (Visiting Fellow and Capability Enhancement Grants) and the European Research Council (StG-2010 263760-FAMMAT).
CR Allal N, 2004, P ROY SOC B-BIOL SCI, V271, P465, DOI 10.1098/rspb.2003.2623
Amir D, 2016, PLOS ONE, V11, DOI 10.1371/journal.pone.0155883
Anderson KG, 2015, HUM NATURE-INT BIOS, V26, P401, DOI 10.1007/s12110-015-9243-6
[Anonymous], EVOLUTION LIFE HIST, DOI [10.1101/510826, DOI 10.1101/510826]
Apicella CL, 2016, CURR OPIN PSYCHOL, V7, P92, DOI 10.1016/j.copsyc.2015.08.015
Baldini R., 2015, BIORXIV, DOI [10.1101/014647, DOI 10.1101/014647]
Barbaro N, 2017, EVOL HUM BEHAV, V38, P357, DOI 10.1016/j.evolhumbehav.2016.11.007
BARKOW JH, 1986, J ANTHROPOL RES, V42, P373, DOI 10.1086/jar.42.3.3630041
BELSKY J, 1991, CHILD DEV, V62, P647, DOI 10.2307/1131166
Belsky J, 2007, CHILD DEV, V78, P1302, DOI 10.1111/j.1467-8624.2007.01067.x
Belsky J, 2012, CURR DIR PSYCHOL SCI, V21, P310, DOI 10.1177/0963721412453588
Biswas S, 2013, HUM BIOL, V85, P607, DOI 10.3378/027.085.0407
Charpentier MJE, 2008, P NATL ACAD SCI USA, V105, P1988, DOI 10.1073/pnas.0711219105
CHISHOLM JS, 1993, CURR ANTHROPOL, V34, P1, DOI 10.1086/204131
Clutton-Brock TH, 2012, MOL ECOL, V21, P472, DOI 10.1111/j.1365-294X.2011.05232.x
Coall DA, 2016, PHILOS T R SOC B, V371, DOI 10.1098/rstb.2015.0146
Coall DA, 2003, SOC SCI MED, V57, P1771, DOI 10.1016/S0277-9536(03)00022-4
Coall DA, 2019, HDB PARENTING BIOL E, V2, P57
Comings DE, 2002, CHILD DEV, V73, P1046, DOI 10.1111/1467-8624.00456
Cowan CSM, DEV PSYCHOBIOL
Day FR, 2015, SCI REP-UK, V5, DOI 10.1038/srep11208
Del Giudice M, 2009, BEHAV BRAIN SCI, V32, P1, DOI 10.1017/S0140525X09000016
DRAPER P, 1982, J ANTHROPOL RES, V38, P255, DOI 10.1086/jar.38.3.3629848

Ellis BJ, 2004, PSYCHOL BULL, V130, P920, DOI 10.1037/0033-2909.130.6.920
Ellis BJ, 2000, CHILD DEV, V71, P485, DOI 10.1111/1467-8624.00159
Ellis BJ, 2019, ANNU REV PSYCHOL, V70, P111, DOI 10.1146/annurev-psych-122216-011732
Ellis BJ, 2009, HUM NATURE-INT BIOS, V20, P204, DOI 10.1007/s12110-009-9063-7
ELLISON PT, 1990, AM ANTHROPOL, V92, P933, DOI 10.1525/aa.1990.92.4.02a00050
Flinn M.V., 1988, HUMAN REPROD BEHAV, P189
Gaydosh L, 2018, DEMOGRAPHY, V55, P1245, DOI 10.1007/s13524-018-0696-1
Geary DC, 2000, PSYCHOL BULL, V126, P55, DOI 10.1037/0033-2909.126.1.55
Geronimus Arline T., 1992, Ethnicity and Disease, V2, P207
Goldberg RE, 2013, POPUL DEV REV, V39, P231, DOI 10.1111/j.1728-4457.2013.00590.x
Gray PeterB., 2012, Fatherhood: Evolution and Human Paternal Behavior
Henrich J, 2010, BEHAV BRAIN SCI, V33, P111, DOI 10.1017/S0140525X10000725
Henrichs KL, 2014, INT J PEDIATR ENDOCR, DOI 10.1186/1687-9856-2014-14
Hewlett BS, 2000, MARRIAGE FAM REV, V29, P59, DOI 10.1300/J002v29n02_05
Hilbrand S., 2016, Grandfathers: Global Perspectives, P21, DOI [10.1057/978-1-137-56338-52, DOI 10.1057/978-1-137-56338-52]
Horak P, 2015, BIOL LETTERS, V11, DOI 10.1098/rsbl.2015.0752
Hrdy S., 2005, Grandmotherhood: The Evolutionary Significance of the Second Half of Female Life, P295
Hrdy S.B., 2009, Mothers and others: The evolutionary origins of mutual understanding
Hrdy SB, 2008, NEUROBIOLOGY OF THE PARENTAL BRAIN, P407
Hrdy SB, 2000, KINSEY INST, V6, P33
Huffman MC, 2017, AM J PRIMATOL, V79, DOI 10.1002/ajp.22588
Kaplan H, 1996, YEARB PHYS ANTHROPOL, V39, P91
Kearney MS, 2012, J ECON PERSPECT, V26, P141, DOI 10.1257/jep.26.2.141
Kim K, 1997, EVOL HUM BEHAV, V18, P109, DOI 10.1016/S1090-5138(96)00114-6
Lahdenperä M, 2007, P ROY SOC B-BIOL SCI, V274, P2437, DOI 10.1098/rspb.2007.0688
Lawson DW, 2017, AM J HUM BIOL, V29, DOI 10.1002/ajhb.22938
Lehmann L, 2010, PHILOS T R SOC B, V365, P2599, DOI 10.1098/rstb.2010.0138
LeVine RA, 2014, DIFFERENT FACES OF ATTACHMENT: CULTURAL VARIATIONS ON A UNIVERSAL HUMAN NEED, P50
Lian QG, 2018, PEERJ, V6, DOI 10.7717/peerj.5085
Lummaa V, 2002, TRENDS ECOL EVOL, V17, P141, DOI 10.1016/S0169-5347(01)02414-4
Magnus MC, 2018, J EPIDEMIOL COMMUN H, V72, P34, DOI 10.1136/jech-2017-209488
Matchock RL, 2006, AM J HUM BIOL, V18, P481, DOI 10.1002/ajhb.20508
Mathot KJ, 2018, BEHAV ECOL SOCIOBIOL, V72, DOI 10.1007/s00265-018-2459-9
Mattison SM, 2019, ARCH SEX BEHAV, V48, P1329, DOI 10.1007/s10508-018-1350-0
Mattison SM, 2014, AM ANTHROPOL, V116, P591, DOI 10.1111/aman.12125

Mell H, 2018, EVOL HUM BEHAV, V39, P1, DOI 10.1016/j.evolhumbehav.2017.08.006
Mendle J, 2006, DEV PSYCHOL, V42, P533, DOI 10.1037/0012-1649.42.3.533
Mendle J, 2016, J RES ADOLESCENCE, V26, P595, DOI 10.1111/jora.12201
Mishra GD, 2009, WOMENS HEALTH, V5, P175, DOI 10.2217/17455057.5.2.175
MOFFITT TE, 1992, CHILD DEV, V63, P47, DOI 10.1111/j.1467-8624.1992.tb03594.x
Montiglio PO, 2018, BEHAV ECOL SOCIOBIOL, V72, DOI 10.1007/s00265-018-2526-2
Moya C, INTERGENERATIO UNPUB
Moya C, 2014, PEERJ, V2, DOI 10.7717/peerj.512
MULDER MB, 1989, BEHAV ECOL SOCIOBIOL, V24, P145, DOI 10.1007/BF00292097
Nettle D, 2013, P ROY SOC B-BIOL SCI, V280, DOI 10.1098/rspb.2013.1343
Nettle D, 2011, P ROY SOC B-BIOL SCI, V278, P1721, DOI 10.1098/rspb.2010.1726
Nettle D, 2011, PHILOS T R SOC B, V366, P357, DOI 10.1098/rstb.2010.0073
Oli MK, 2004, BASIC APPL ECOL, V5, P449, DOI 10.1016/j.baae.2004.06.002
Osemwenkha AP., 2014, Nigerian J Experimental Clin Biosci, V2, P95, DOI [10.4103/2348-0149.144844, DOI 10.4103/2348-0149.144844]
Otto H, 2014, DIFFERENT FACES OF ATTACHMENT: CULTURAL VARIATIONS ON A UNIVERSAL HUMAN NEED, P1, DOI 10.1017/CBO9781139226684
Palermo T, 2009, STUD FAMILY PLANN, V40, P101, DOI 10.1111/j.1728-4465.2009.00193.x
PROMISLOW DEL, 1990, J ZOOL, V220, P417, DOI 10.1111/j.1469-7998.1990.tb04316.x
Quinlan RJ, 2001, HUM NATURE-INT BIOS, V12, P169, DOI 10.1007/s12110-001-1005-y
Rickard IJ, 2014, PERSPECT PSYCHOL SCI, V9, P3, DOI 10.1177/1745691613513467
Rosenbaum S, 2018, PHYSIOL BEHAV, V193, P12, DOI 10.1016/j.physbeh.2017.12.027
Ryan RM, 2015, J ADOLESCENT HEALTH, V57, P342, DOI 10.1016/j.jadohealth.2015.06.005
Scelza BA, 2010, CURR ANTHROPOL, V51, P295, DOI 10.1086/651051
Schlomer GL, 2017, EVOL HUM BEHAV, V38, P761, DOI 10.1016/j.evolhumbehav.2017.06.002
Sear R., 2018, Population Horizons, V14, P18, DOI [DOI 10.1515/POPHZN-2017-0006, 10.1515/pophzn-2017-0006]
Sear R, 2008, EVOL HUM BEHAV, V29, P1, DOI 10.1016/j.evolhumbehav.2007.10.001
Sear R, 2006, HUM NATURE-INT BIOS, V17, P405, DOI 10.1007/s12110-006-1003-1
Sear R, 2016, CURR OPIN PSYCHOL, V7, P98, DOI 10.1016/j.copsyc.2015.08.013
Sear R, 2011, POPUL DEV REV, V37, P81, DOI 10.1111/j.1728-4457.2011.00379.x
Shenk MK, 2013, HUM NATURE-INT BIOS, V24, P76, DOI 10.1007/s12110-013-9160-5
Sheppard Paula, 2015, Evolution Medicine and Public Health, P332, DOI 10.1093/emph/eov028

Sheppard P, 2014, HUM NATURE-INT BIOS, V25, P213, DOI 10.1007/s12110-014-9195-2
Sheppard P, 2012, BIOL LETTERS, V8, P237, DOI 10.1098/rsbl.2011.0747
Sloboda DM, 2007, J CLIN ENDOCR METAB, V92, P46, DOI 10.1210/jc.2006-1378
Smith D, 2017, BIOL LETTERS, V13, DOI 10.1098/rsbl.2017.0464
Sohn K, 2017, HUM NATURE-INT BIOS, V28, P407, DOI 10.1007/s12110-017-9299-6
Stearns S.C., 1992, pi
Stiver KA, 2009, ETHOLOGY, V115, P1101, DOI 10.1111/j.1439-0310.2009.01707.x
SURBEY MK, 1990, MG PRIMATOL, V13, P11
Surbey MK, 1998, HUM NATURE-INT BIOS, V9, P67, DOI 10.1007/s12110-998-1012-3
Thomas F, 2001, HUM BIOL, V73, P271, DOI 10.1353/hub.2001.0029
Tither JM, 2008, DEV PSYCHOL, V44, P1409, DOI 10.1037/a0013065
Voland E, 2017, EVOL HUM BEHAV, V38, P125, DOI 10.1016/j.evolhumbehav.2016.08.001
WANG ZX, 1992, J COMP PSYCHOL, V106, P163, DOI 10.1037/0735-7036.106.2.163
Waynforth D, 1998, EVOL HUM BEHAV, V19, P369, DOI 10.1016/S1090-5138(98)00031-2
Waynforth D., 2002, HDB FATHER INVOLVEME, P337
Webster GD, 2014, EVOL PSYCHOL, V12, DOI [10.1177/, DOI 10.1177/]
Weichold K., 2003, Gender differences at puberty, P241, DOI [DOI 10.1017/CBO9780511489716, DOI 10.1017/CBO9780511489716.013]
Wells JCK, 2000, J THEOR BIOL, V202, P65, DOI 10.1006/jtbi.1999.1044
Wells JCK, 2010, AM J HUM BIOL, V22, P1, DOI 10.1002/ajhb.20994
Whiting JWM., 1965, SEXUAL BEHAV, P221
Wilson ME, 2013, PSYCHONEUROENDOCRINO, V38, P67, DOI 10.1016/j.psyneuen.2012.04.021
Winking J, 2011, EVOL HUM BEHAV, V32, P79, DOI 10.1016/j.evolhumbehav.2010.08.002
Xu Y, 2018, EVOL HUM BEHAV, V39, P40, DOI 10.1016/j.evolhumbehav.2017.08.005
Yermachenko A, 2014, BIOMED RES INT-UK, V2014, DOI 10.1155/2014/371583
NR 112
TC 71
Z9 76
U1 0
U2 19
PU ROYAL SOC
PI LONDON
PA 6-9 CARLTON HOUSE TERRACE, LONDON SW1Y 5AG, ENGLAND
SN 0962-8436
EI 1471-2970
J9 PHILOS T R SOC B
JI Philos. Trans. R. Soc. B-Biol. Sci.
PD APR 15
PY 2019
VL 374
IS 1770
AR 20180124
DI 10.1098/rstb.2018.0124

PG 14
WC Biology
WE Science Citation Index Expanded (SCI-EXPANDED); Social Science Citation Index (SSCI)
SC Life Sciences & Biomedicine - Other Topics
GA HN8ZI
UT WOS:000460487000015
PM 30966893
OA hybrid, Green Submitted, Green Published, Green Accepted
DA 2025-05-07
ER

PT J
AU Groothuis, TGG
   Hsu, BY
   Kumar, N
   Tschirren, B
AF Groothuis, Ton G. G.
   Hsu, Bin-Yan
   Kumar, Neeraj
   Tschirren, Barbara
TI Revisiting mechanisms and functions of prenatal hormone-mediated maternal effects using avian species as a model
SO PHILOSOPHICAL TRANSACTIONS OF THE ROYAL SOCIETY B-BIOLOGICAL SCIENCES
LA English
DT Article
DE parental effects; phenotypic plasticity; context dependency; parent-offspring conflict; steroids; yolk hormones
ID YOLK TESTOSTERONE; THYROID-HORMONES; EGG-YOLK; SEXUAL CONFLICT; JAPANESE-QUAIL; OFFSPRING PHENOTYPE; MALE ATTRACTIVENESS; BIRD EGGS; ANDROGENS; DEPOSITION
AB Maternal effects can adaptively modulate offspring developmental trajectories in variable but predictable environments. Hormone synthesis is sensitive to environmental factors, and maternal hormones are thus a powerful mechanism to transfer environmental cues to the next generation. Birds have become a key model for the study of hormone-mediated maternal effects because the embryo develops outside the mother's body, facilitating the measurement and manipulation of prenatal hormone exposure. At the same time, birds are excellent models for the integration of both proximate and ultimate approaches, which is key to a better understanding of the evolution of hormone-mediated maternal effects. Over the past two decades, a surge of studies on hormone-mediated maternal effects has revealed an increasing number of discrepancies. In this review, we discuss the role of the environment, genetic factors and social interactions in causing these discrepancies and provide a framework to resolve them. We also explore the largely neglected role of the embryo in modulating the maternal signal, as well as costs and benefits of hormone transfer and expression for the different family members. We conclude by highlighting fruitful avenues for future research that have opened up thanks to new theoretical insights and technical advances in the field.
   This article is part of the theme issue 'Developing differences: early-life effects and evolutionary medicine'.

C1 [Groothuis, Ton G. G.; Hsu, Bin-Yan; Kumar, Neeraj] Univ Groningen, Groningen Inst Evolutionary Life Sci, Behav Biol, NL-9747 AG Groningen, Netherlands.
[Hsu, Bin-Yan] Univ Turku, Dept Biol, Turku, Finland.
[Tschirren, Barbara] Univ Exeter, Ctr Ecol & Conservat, Penryn, England.
C3 University of Groningen; University of Turku; University of Exeter
RP Groothuis, TGG (autor correspondente), Univ Groningen, Groningen Inst Evolutionary Life Sci, Behav Biol, NL-9747 AG Groningen, Netherlands.
EM a.g.g.groothuis@rug.nl
RI Hsu, Bin-Yan/AAF-6513-2019; Kumar, Neeraj/S-5654-2018; Kumar, Neeraj/M-3279-2015; Tschirren, Barbara/F-8202-2011
OI Kumar, Neeraj/0000-0001-6713-2129; Kumar, Neeraj/0000-0003-0769-1564; Tschirren, Barbara/0000-0003-4806-4102; Hsu, Bin-Yan/0000-0002-3799-0509
CR Adkins-Regan E., 2005, Hormones and animal social behavior
Albergotti LC, 2009, PLOS ONE, V4, DOI 10.1371/journal.pone.0005452
[Anonymous], 1998, MATERNAL EFFECTS ADA
Barnett CA, 2011, ANIM BEHAV, V81, P113, DOI 10.1016/j.anbehav.2010.09.019
Bateson P, 2004, NATURE, V430, P419, DOI 10.1038/nature02725
Benowitz-Fredericks ZM, 2013, GEN COMP ENDOCR, V193, P48, DOI 10.1016/j.ygcen.2013.07.005
Bertin A, 2008, HORM BEHAV, V54, P396, DOI 10.1016/j.yhbeh.2008.04.012
Bertin A, 2013, PLOS ONE, V8, DOI 10.1371/journal.pone.0057670
Bertin A, 2009, ETHOLOGY, V115, P879, DOI 10.1111/j.1439-0310.2009.01672.x
BURLEY N, 1986, AM NAT, V127, P415, DOI 10.1086/284493
De Baere S, 2015, ANAL BIOANAL CHEM, V407, P4351, DOI 10.1007/s00216-014-8269-7
Del Giudice M, 2012, PSYCHONEUROENDOCRINO, V37, P1614, DOI 10.1016/j.psyneuen.2012.05.014
DeWitt TJ, 1998, TRENDS ECOL EVOL, V13, P77, DOI 10.1016/S0169-5347(97)01274-3
Egbert JR, 2013, GEN COMP ENDOCR, V183, P53, DOI 10.1016/j.ygcen.2012.12.001
Estramil N, 2017, HORM BEHAV, V87, P129, DOI 10.1016/j.yhbeh.2016.11.005
Frésard L, 2014, NUCLEIC ACIDS RES, V42, P3768, DOI 10.1093/nar/gkt1390
Gil D, 2008, ADV STUD BEHAV, V38, P337, DOI 10.1016/S0065-3454(08)00007-7
Giordano M, 2014, BEHAV ECOL, V25, P1459, DOI 10.1093/beheco/aru149
Giraudeau M, 2016, BIOL LETTERS, V12, DOI 10.1098/rsbl.2016.0676
Giraudeau M, 2017, BEHAV ECOL, V28, P31, DOI 10.1093/beheco/arw127
Gluckman PD, 2007, AM J HUM BIOL, V19, P1, DOI 10.1002/ajhb.20590
Godsave SF, 2002, J COMP NEUROL, V453, P57, DOI 10.1002/cne.10391
Griffith OW, 2017, GEN COMP ENDOCR, V244, P19, DOI 10.1016/j.ygcen.2016.04.017
Groothuis TGG, 2005, BIOL LETTERS, V1, P78, DOI 10.1098/rsbl.2004.0233
Groothuis TGG, 2005, NEUROSCI BIOBEHAV R, V29, P329, DOI 10.1016/j.neubiorev.2004.12.002
Groothuis TGG, 2008, PHILOS T R SOC B, V363, P1647, DOI 10.1098/rstb.2007.0007

Groothuis TGG, 2015, FRONT ZOOL, V12, DOI 10.1186/1742-9994-12-S1-S6
Groothuis TGG, 2011, DEV PSYCHOBIOL, V53, P641, DOI 10.1002/dev.20574
Henriksen R, 2011, NEUROSCI BIOBEHAV R, V35, P1484, DOI 10.1016/j.neubiorev.2011.04.010
Horváthová T, 2012, P ROY SOC B-BIOL SCI, V279, P163, DOI 10.1098/rspb.2011.0663
Hsu BY, 2017, GEN COMP ENDOCR, V240, P174, DOI 10.1016/j.ygcen.2016.10.011
Hsu BY, 2016, ECOL EVOL, V6, P397, DOI 10.1002/ece3.1845
Hsu BY, COEVOLVED MATE UNPUB
Immler S, 2018, HEREDITY, V121, P239, DOI 10.1038/s41437-018-0111-0
Jenkins TG, 2015, MOL HUM REPROD, V21, P313, DOI 10.1093/molehr/gau115
Kuijper B, 2018, EVOLUTION, V72, P220, DOI 10.1111/evo.13403
Kumar N, STEROID RECEPT UNPUB
Kumar N, TESTOSTERONE CONCENT
Kumar N, 2018, GEN COMP ENDOCR, V269, P53, DOI 10.1016/j.ygcen.2018.08.014
Kumar N, 2018, SCI REP-UK, V8, DOI 10.1038/s41598-018-29478-4
Laaksonen T, 2011, BEHAV ECOL SOCIOBIOL, V65, P257, DOI 10.1007/s00265-010-1034-9
Larsen TR, 2015, J AVIAN BIOL, V46, P643, DOI 10.1111/jav.00735
Lessells CM, 1999, MG BEH ECOL, P75
Lipar JL, 1999, GEN COMP ENDOCR, V115, P220, DOI 10.1006/gcen.1999.7296
Lipar JL, 2001, J AVIAN BIOL, V32, P231, DOI 10.1111/j.0908-8857.2001.320305.x
Marshall DJ, 2007, OIKOS, V116, P1957, DOI 10.1111/j.2007.0030-1299.16203.x
Martin TE, 2008, PHILOS T R SOC B, V363, P1663, DOI 10.1098/rstb.2007.0009
MCNABB FMA, 1985, DOMEST ANIM ENDOCRIN, V2, P35, DOI 10.1016/0739-7240(85)90023-2
Merrill L, 2019, CAN J ZOOL, V97, P1, DOI 10.1139/cjz-2017-0351
Moog NK, 2017, NEUROSCIENCE, V342, P68, DOI 10.1016/j.neuroscience.2015.09.070
MOORE T, 1991, TRENDS GENET, V7, P45, DOI 10.1016/0168-9525(91)90230-N
Moreno-Rueda G, 2007, BEHAV ECOL, V18, P496, DOI 10.1093/beheco/arl106
Morinha F, 2012, THERIOGENOLOGY, V78, P703, DOI 10.1016/j.theriogenology.2012.04.015
Müller MS, 2012, PLOS ONE, V7, DOI 10.1371/journal.pone.0047763
Müller W, 2007, AM NAT, V169, pE84, DOI 10.1086/511962
Müller W, 2010, BEHAV ECOL, V21, P493, DOI 10.1093/beheco/arq010
Muriel J, 2015, J EXP BIOL, V218, P2241, DOI 10.1242/jeb.118257
Navara KJ, 2006, PHYSIOL BIOCHEM ZOOL, V79, P1098, DOI 10.1086/507661
Noguera JC, 2013, HORM BEHAV, V64, P19, DOI 10.1016/j.yhbeh.2013.04.008
Okuliarova M, 2018, GEN COMP ENDOCR, V256, P23, DOI 10.1016/j.ygcen.2017.05.004
Okuliarova M, 2014, PLOS ONE, V9, DOI 10.1371/journal.pone.0112817
Okuliarova M, 2011, AM NAT, V177, P824, DOI 10.1086/659996
Paitz RT, 2013, INTEGR COMP BIOL, V53, P895, DOI 10.1093/icb/ict027
Paitz RT, 2011, P ROY SOC B-BIOL SCI, V278, P2005, DOI 10.1098/rspb.2010.2128

Paitz RT, 2011, P ROY SOC B-BIOL SCI, V278, P99, DOI 10.1098/rspb.2010.0813
Paquet M, 2016, BEHAV ECOL, V27, P685, DOI 10.1093/beheco/arv230
Pedernera Enrique, 2017, BMC Physiology, V17, P7, DOI 10.1186/s12899-017-0033-8
Pick JL, 2016, HEREDITY, V116, P542, DOI 10.1038/hdy.2016.16
Pick JL, 2016, FRONT ZOOL, V13, DOI 10.1186/s12983-016-0172-y
Podmokla E, 2018, BIOL REV, V93, P1499, DOI 10.1111/brv.12406
Poisbleau M, 2011, PLOS ONE, V6, DOI 10.1371/journal.pone.0027765
Postma E, 2014, OECOLOGIA, V174, P631, DOI 10.1007/s00442-013-2803-8
Quillfeldt P, 2011, GEN COMP ENDOCR, V173, P11, DOI 10.1016/j.ygcen.2011.05.005
Reed WL, 2011, INTEGR COMP BIOL, V51, P73, DOI 10.1093/icb/icr032
Rettenbacher S, 2013, WIEN TIERARZTL MONAT, V100, P283
Rettenbacher S, 2009, GEN COMP ENDOCR, V164, P125, DOI 10.1016/j.ygcen.2009.05.019
Roff Derek A., 1992
Royle NJ, 2001, BEHAV ECOL, V12, P381, DOI 10.1093/beheco/12.4.381
Ruuskanen S, 2018, PHYSIOL BIOCHEM ZOOL, V91, P904, DOI 10.1086/697380
Ruuskanen S, 2016, HORM BEHAV, V81, P38, DOI 10.1016/j.yhbeh.2016.03.006
Ruuskanen S, 2013, BEHAV ECOL SOCIOBIOL, V67, P361, DOI 10.1007/s00265-012-1456-7
Ruuskanen S, 2009, HORM BEHAV, V55, P514, DOI 10.1016/j.yhbeh.2009.01.010
Schwabl H, 1997, NATURE, V386, P231, DOI 10.1038/386231a0
SCHWABL H, 1993, P NATL ACAD SCI USA, V90, P11446, DOI 10.1073/pnas.90.24.11446
Schwabl H, 1996, COMP BIOCHEM PHYS A, V114, P271, DOI 10.1016/0300-9629(96)00009-6
Smiseth PT, 2011, ANIM BEHAV, V81, P507, DOI 10.1016/j.anbehav.2010.11.029
Tobler M, 2007, BIOLOGY LETT, V3, P408, DOI 10.1098/rsbl.2007.0127
Tobler M, 2009, J EXP BIOL, V212, P89, DOI 10.1242/jeb.020826
Treidel LA, 2013, BIOL LETTERS, V9, DOI 10.1098/rsbl.2013.0684
TRIVERS RL, 1974, AM ZOOL, V14, P249
Tschirren B, 2005, J ANIM ECOL, V74, P675, DOI 10.1111/j.1365-2656.2005.00963.x
Tschirren B, 2004, P ROY SOC B-BIOL SCI, V271, P1371, DOI 10.1098/rspb.2004.2730
Tschirren B, 2008, ANIM BEHAV, V75, P1989, DOI 10.1016/j.anbehav.2008.01.007
Tschirren B, 2016, PHYSIOL BIOCHEM ZOOL, V89, P448, DOI 10.1086/687571
Tschirren B, 2015, PLOS ONE, V10, DOI 10.1371/journal.pone.0133673
Tschirren B, 2014, ECOL LETT, V17, P1310, DOI 10.1111/ele.12339
Tuiskula-Haavisto M, 2007, CYTOGENET GENOME RES, V117, P305, DOI 10.1159/000103192
Uller T, 2013, J EVOLUTION BIOL, V26, P2161, DOI 10.1111/jeb.12212
Vassallo BG, 2014, BIOL LETTERS, V10, DOI 10.1098/rsbl.2014.0502
Verboven N, 2003, P ROY SOC B-BIOL SCI, V270, P2223, DOI 10.1098/rspb.2003.2496
Vergauwen J, 2012, GEN COMP ENDOCR, V176, P112, DOI 10.1016/j.ygcen.2012.01.003

von Engelhardt N, 2011, HORMONES AND REPRODUCTION OF VERTEBRATES, VOL 4: BIRDS, P91
von Engelhardt N, 2009, GEN COMP ENDOCR, V163, P175, DOI 10.1016/j.ygcen.2009.04.004
Williams TD, 2015, NESTS EGGS INCUBATIO
Wilson CM, 1997, GEN COMP ENDOCR, V107, P153, DOI 10.1006/gcen.1997.6906
NR 105
TC 91
Z9 93
U1 2
U2 38
PU ROYAL SOC
PI LONDON
PA 6-9 CARLTON HOUSE TERRACE, LONDON SW1Y 5AG, ENGLAND
SN 0962-8436
EI 1471-2970
J9 PHILOS T R SOC B
JI Philos. Trans. R. Soc. B-Biol. Sci.
PD APR 15
PY 2019
VL 374
IS 1770
AR 20180115
DI 10.1098/rstb.2018.0115
PG 9
WC Biology
WE Science Citation Index Expanded (SCI-EXPANDED)
SC Life Sciences & Biomedicine - Other Topics
GA HN8ZI
UT WOS:000460487000006
PM 30966885
OA Green Published, Green Accepted, Bronze
DA 2025-05-07
ER

PT J
AU Qurollo, BA
Larsen, PA
Rakotondrainibe, HH
Mahefarisoa, K
Rajaonarivelo, T
Razafindramanana, J
Breitschwerdt, EB
Junge, RE
Williams, CV
AF Qurollo, Barbara A.
Larsen, Peter A.
Rakotondrainibe, Hajanirina H.
Mahefarisoa, Karine
Rajaonarivelo, Tsiky
Razafindramanana, Josia
Breitschwerdt, Edward B.
Junge, Randall E.

Williams, Cathy V.
TI Molecular surveillance of novel tick-borne organisms in Madagascar's lemurs
SO TICKS AND TICK-BORNE DISEASES
LA English
DT Article
DE Babesia; Borrelia; Neoehrlichia; Lemurs; Madagascar; One health
ID EMERGING INFECTIOUS-DISEASES; IXODIDAE; FEVER; BLOOD; PATHOGENS; BABESIA; HEALTH; VIRUS; ACARI; DNA
AB The discovery and characterization of emerging tick-borne organisms are critical for global health initiatives to improve animal and human welfare (One Health). It is possible that unknown tick-borne organisms underlie a subset of undiagnosed illness in wildlife, domesticated species, and humans. Our study lends support to the One Health concept by highlighting the prevalence of three blood-borne organisms in wild lemurs living in close proximity to domesticated species and humans. Previously, our team identified three novel, presumably tick-borne, intravascular organisms, belonging to the genera Babesia, Borrelia, and Neoehrlichia, circulating in two of Madagascar's lemur species. Here, we extend our previous observation by developing a targeted molecular surveillance approach aimed at determining the prevalence of these organisms in lemurs. Using quantitative PCR, we provide Babesia, Borrelia, and Neoehrlichia prevalence data for 76 individuals comprising four lemur species located in eastern Madagascar. Our results indicate a high prevalence (96%) of Babesia across sampled individuals with lower prevalences for Neoehrlichia (36%) and Borrelia (14.5%). In light of our results, we recommend additional studies of these tick-borne organisms to determine pathogenicity and assess zoonotic potency to other animals and humans in Madagascar.
C1 [Qurollo, Barbara A.; Breitschwerdt, Edward B.] North Carolina State Univ, Coll Vet Med, Dept Clin Sci, Raleigh, NC 27695 USA.
[Larsen, Peter A.] Duke Univ, Dept Biol, Durham, NC USA.
[Larsen, Peter A.; Williams, Cathy V.] Duke Univ, Duke Lemur Ctr, Durham, NC USA.
[Rakotondrainibe, Hajanirina H.; Razafindramanana, Josia] Ambatovy Minerals SA, Antananarivo, Madagascar.
[Mahefarisoa, Karine] VetCare, Clin Vet, Antananarivo, Madagascar.
[Rajaonarivelo, Tsiky] Vet Clin Ampandrianomby, Antananarivo, Madagascar.
[Junge, Randall E.] Columbus Zoo & Aquarium, Dept Anim Hlth, Columbus, OH USA.
C3 North Carolina State University; Duke University; Duke University
RP Qurollo, BA (autor correspondente), North Carolina State Univ, Coll Vet Med, Dept Clin Sci, Raleigh, NC 27695 USA.
OI Larsen, Peter/0000-0002-3634-3625; Breitschwerdt, Edward/0000-0002-3506-0279; Mahefarisoa, Karine Lalaina/0000-0003-0165-9088; Qurollo, Barbara/0000-0002-9849-2511
FU Triangle Center for Evolutionary Medicine (TriCEM) [(1950) 2016-2411]; Vector-Borne Disease Diagnostic Laboratory (VBDDL) at North Carolina State University
FX This work was supported by the Triangle Center for Evolutionary Medicine
(TriCEM) [proposal number (1950) 2016-2411] and the Vector-Borne Disease

Diagnostic Laboratory (VBDDL) at North Carolina State University.
CR [Anonymous], B MUS COMP ZOOL
Bardsley EL, 2014, TROP ANIM HEALTH PRO, V46, P133, DOI 10.1007/s11250-013-0463-7
Birkenheuer AJ, 2008, PARASITOLOGY, V135, P33, DOI 10.1017/S0031182007003538
Birkenheuer AJ, 2003, J CLIN MICROBIOL, V41, P4172, DOI 10.1128/JCM.41.9.4172-4177.2003
Blanco MB, 2013, J PARASITOL, V99, P11, DOI 10.1645/GE-3183.1
Briggs BJ, 2011, EMERG INFECT DIS, V17, P876, DOI 10.3201/eid1705.101183
Crowley BE, 2010, QUATERNARY SCI REV, V29, P2591, DOI 10.1016/j.quascirev.2010.06.030
Daszak P, 2000, SCIENCE, V287, P443, DOI 10.1126/science.287.5452.443
Dewar RE, 2013, P NATL ACAD SCI USA, V110, P12583, DOI 10.1073/pnas.1306100110
Gommery D, 2011, CR PALEVOL, V10, P271, DOI 10.1016/j.crpv.2011.01.006
Hagen RM, 2018, ACTA TROP, V177, P127, DOI 10.1016/j.actatropica.2017.10.002
Jones KE, 2008, NATURE, V451, P990, DOI 10.1038/nature06536
Klompen H., 2003, The Natural History of Madagascar, P588
Klompen H, 2015, J MED ENTOMOL, V52, P315, DOI 10.1093/jme/tjv032
Larsen PA, 2016, BIOL LETTERS, V12, DOI 10.1098/rsbl.2015.0829
MATHIOT CC, 1988, ANN INST PASTEUR VIR, V139, P239, DOI 10.1016/S0769-2617(88)80022-4
Matysiak A, 2016, VET MED-CZECH, V61, P516, DOI 10.17221/59/2016-VETMED
Moutailler S, 2016, PLOS NEGLECT TROP D, V10, DOI 10.1371/journal.pntd.0004539
Ogden NH, 2004, J MED ENTOMOL, V41, P622, DOI 10.1603/0022-2585-41.4.622
Rahajarison P, 2014, INFECT GENET EVOL, V28, P666, DOI 10.1016/j.meegid.2014.06.022
Roger F, 2001, EXP APPL ACAROL, V25, P263, DOI 10.1023/A:1010687502145
Savage AF, 2009, J WILDLIFE DIS, V45, P907, DOI 10.7589/0090-3558-45.4.907
Silaghi C, 2012, PARASITE VECTOR, V5, DOI 10.1186/1756-3305-5-285
Springer A, 2015, INT J PARASITOL-PAR, V4, P385, DOI 10.1016/j.ijppaw.2015.10.006
Uilenberg G, 1979, TIQUES IXODOIDEA MAD, P1
Vayssier-Taussat M, 2015, FUTURE MICROBIOL, V10, P2033, DOI 10.2217/fmb.15.114
NR 26
TC 4
Z9 4
U1 0
U2 12
PU ELSEVIER GMBH, URBAN & FISCHER VERLAG
PI JENA
PA OFFICE JENA, P O BOX 100537, 07705 JENA, GERMANY
SN 1877-959X
EI 1877-9603
J9 TICKS TICK-BORNE DIS
JI Ticks Tick-Borne Dis.

PD MAR
PY 2018
VL 9
IS 3
BP 672
EP 677
DI 10.1016/j.ttbdis.2018.02.012
PG 6
WC Infectious Diseases; Microbiology; Parasitology
WE Science Citation Index Expanded (SCI-EXPANDED)
SC Infectious Diseases; Microbiology; Parasitology
GA GH0YP
UT WOS:000433128600032
PM 29477959
DA 2025-05-07
ER

PT J
AU Ash, JR
Hughes-Oliver, JM
AF Ash, Jeremy R.
Hughes-Oliver, Jacqueline M.
TI Confidence bands and hypothesis tests for hit enrichment curves
SO JOURNAL OF CHEMINFORMATICS
LA English
DT Article
DE Virtual screening; Enrichment factor; Lift curve; Early enrichment; Ranking algorithm; Empirical process
ID OPTIMIZATION; RECOMMENDATIONS; DIFFERENCE; DISCOVERY; INTERVALS; DOCKING; MODELS; SETS; TOOL
AB In virtual screening for drug discovery, hit enrichment curves are widely used to assess the performance of ranking algorithms with regard to their ability to identify early enrichment. Unfortunately, researchers almost never consider the uncertainty associated with estimating such curves before declaring differences between performance of competing algorithms. Uncertainty is often large because the testing fractions of interest to researchers are small. Appropriate inference is complicated by two sources of correlation that are often overlooked: correlation across different testing fractions within a single algorithm, and correlation between competing algorithms. Additionally, researchers are often interested in making comparisons along the entire curve, not only at a few testing fractions. We develop inferential procedures to address both the needs of those interested in a few testing fractions, as well as those interested in the entire curve. For the former, four hypothesis testing and (pointwise) confidence intervals are investigated, and a newly developed EmProc approach is found to be most effective. For inference along entire curves, EmProc-based confidence bands are recommended for simultaneous coverage and minimal width. While we focus on the hit enrichment curve, this work is also appropriate for lift curves that are used throughout the machine learning community. Our inferential procedures trivially extend to enrichment factors, as well.
C1 [Ash, Jeremy R.; Hughes-Oliver, Jacqueline M.] North Carolina State Univ, Dept Stat, Bioinformat Res Ctr, Raleigh, NC 27695 USA.
[Ash, Jeremy R.] SAS Inst, JMP Div, Cary, NC 27513 USA.

C3 North Carolina State University; SAS Institute Inc
RP Ash, JR (autor correspondente), North Carolina State Univ, Dept Stat, Bioinformat Res Ctr, Raleigh, NC 27695 USA.; Ash, JR (autor correspondente), SAS Inst, JMP Div, Cary, NC 27513 USA.
EM jrash@ncsu.edu
RI Ash, Jeremy/ABA-5317-2020
FU NIH [T32ES007329]; Triangle Center for Evolutionary Medicine; SAS Institute
FX This work was supported by NIH training grant T32ES007329; the Triangle Center for Evolutionary Medicine; and a graduate research assistantship from SAS Institute.
CR Agarwala R, 2018, NUCLEIC ACIDS RES, V46, pD8, DOI [10.1093/nar/gks1189, 10.1093/nar/gkx1095, 10.1093/nar/gkq1172]
Agresti A, 1998, AM STAT, V52, P119, DOI 10.2307/2685469
Agresti A., 2007, An Introduction to Categorical Data Analysis, V2nd, DOI 10.10029780470114759.ch2
[Anonymous], 2007, Nonparametric Econometrics: Theory and Practice
[Anonymous], 1989, JMP
Ash JR, 2020, Methods development for quantitative structure-activity relationships
Bauer MR, 2013, J CHEM INF MODEL, V53, P1447, DOI [10.1021/ci400115b, 10.1021/ci400115bl]
BENJAMINI Y, 1995, J R STAT SOC B, V57, P289, DOI 10.1111/j.2517-6161.1995.tb02031.x
Bonett DG, 2012, J EDUC BEHAV STAT, V37, P479, DOI 10.3102/1076998611411915
Empereur-Mot C, 2016, J CHEM INF MODEL, V56, P2281, DOI 10.1021/acs.jcim.6b00283
Fagerland MW, 2013, BMC MED RES METHODOL, V13, DOI 10.1186/1471-2288-13-91
Friesner RA, 2004, J MED CHEM, V47, P1739, DOI 10.1021/jm0306430
Gaulton A, 2017, NUCLEIC ACIDS RES, V45, pD945, DOI 10.1093/nar/gkw1074
Geppert H, 2010, J CHEM INF MODEL, V50, P205, DOI 10.1021/ci900419k
Good AC, 2008, J COMPUT AID MOL DES, V22, P169, DOI 10.1007/s10822-007-9167-2
Hawkins PCD, 2008, J COMPUT AID MOL DES, V22, P179, DOI 10.1007/s10822-007-9166-3
Irwin JJ, 2008, J COMPUT AID MOL DES, V22, P193, DOI 10.1007/s10822-008-9189-4
Jain AN, 2008, J COMPUT AID MOL DES, V22, P133, DOI 10.1007/s10822-008-9196-5
Jiang W., 2014, Some Technical Details on Confidence Intervals for LIFT Measures in Data Mining; tech. rep
Jiang WX, 2015, J AM STAT ASSOC, V110, P1717, DOI 10.1080/01621459.2014.993080
Kuhn M, 2008, J STAT SOFTW, V28, P1, DOI 10.18637/jss.v028.i05
KULLBACK S, 1951, ANN MATH STAT, V22, P79, DOI 10.1214/aoms/1177729694
Maechler Martin, 2024, CRAN
Mysinger MM, 2012, J MED CHEM, V55, P6582, DOI 10.1021/jm300687e
NCBI, 2021, ABOUT US

Newcombe RG, 1998, STAT MED, V17, P2635, DOI 10.1002/(SICI)1097-0258(19981130)17:22<2635::AID-SIM954>3.0.CO;2-C
Nicholls A, 2014, J COMPUT AID MOL DES, V28, P887, DOI 10.1007/s10822-014-9753-z
Olea JLM, 2019, J APPL ECONOMET, V34, P1, DOI 10.1002/jae.2656
Robinson MC, 2020, J COMPUT AID MOL DES, V34, P717, DOI 10.1007/s10822-019-00274-0
Rodriguezde Gil P, 2013, P SE SAS USERS GROUP
Rohrer SG, 2009, J CHEM INF MODEL, V49, P169, DOI 10.1021/ci8002649
Rosset S, 2001, Evaluation of prediction models for marketing campaigns, P456, DOI 10.1145/502512.502581
Saito T, 2015, PLOS ONE, V10, DOI 10.1371/journal.pone.0118432
SAS Institute Inc, 2020, SAS ENT MIN 15 1
Sheridan RP, 2001, J CHEM INF COMP SCI, V41, P1395, DOI 10.1021/ci0100144
Sterling T, 2015, J CHEM INF MODEL, V55, P2324, DOI 10.1021/acs.jcim.5b00559
Stumpfe D, 2011, APPL VIRTUAL SCREENI, P291, DOI [10.1002/9783527633326.ch11, DOI 10.1002/9783527633326.CH11]
Truchon JF, 2007, J CHEM INF MODEL, V47, P488, DOI 10.1021/ci600426e
Xia J, 2015, METHODS, V71, P146, DOI 10.1016/j.ymeth.2014.11.015
Zhu T, 2013, J MED CHEM, V56, P6560, DOI 10.1021/jm301916b
NR 40
TC 1
Z9 1
U1 0
U2 3
PU BMC
PI LONDON
PA CAMPUS, 4 CRINAN ST, LONDON N1 9XW, ENGLAND
SN 1758-2946
J9 J CHEMINFORMATICS
JI J. Cheminformatics
PD JUL 28
PY 2022
VL 14
IS 1
AR 50
DI 10.1186/s13321-022-00629-0
PG 17
WC Chemistry, Multidisciplinary; Computer Science, Information Systems; Computer Science, Interdisciplinary Applications
WE Science Citation Index Expanded (SCI-EXPANDED)
SC Chemistry; Computer Science
GA 3I4FP
UT WOS:000832675200001
PM 35902962
OA Green Published, gold
DA 2025-05-07
ER

PT J
AU Sadhir, S
Mcgrosky, A

Swanson, ZS
Tavormina, A
Tomechko, K
Pontzer, H
AF Sadhir, Srishti
Mcgrosky, Amanda
Swanson, Zane S.
Tavormina, Anna
Tomechko, Keri
Pontzer, Herman
TI Physical activity and heat stress shape water needs in pregnant endurance athletes
SO EVOLUTION MEDICINE AND PUBLIC HEALTH
LA English
DT Article
DE water turnover; pregnancy; physical activity; heat index; climate change
ID ENERGY-EXPENDITURE; HYDRATION; EVOLUTION; TURNOVER; FLUID; WOMEN
AB Background and objectives Pregnancy, heat stress, and physical activity (PA) are all known to independently increase human water requirements. We hypothesize that climate conditions and behavioral strategies interact to shape water needs in highly active pregnancies.Methodology We recruited 20 female endurance runners who were pregnant (8-16 weeks gestational age; n = 13) or planning to be pregnant (n = 7) for an observational, prospective cohort study. At three timepoints in the study (preconception, 8-16 weeks, and 32-35 weeks), we measured water turnover (WT) using the deuterium dilution and elimination technique, PA using ActiGraph wGT3X-BT accelerometers, and heat index (HI) using historical temperature and humidity data. We also compared athletes to nonathletes from a previously published study.Results Athletes maintained high WT from preconception through the end of pregnancy. PA was positively associated with WT among athletes for preconception and early pregnancy time periods but not for the third trimester. HI weakly moderated the relationship between PA and WT in predicting a more positive slope in hotter and more humid weather conditions. WT in athletes was higher than in nonathletes, but this difference attenuated during the third trimester, as nonathletes increased their WT.Conclusions and implications Athletes experience higher WT with greater levels of PA, and this relationship is somewhat stronger in higher HI conditions. With the threat of climate change expected to exacerbate extreme heat conditions, evidence-based, global policies are required for particularly vulnerable populations.

Physical activity and environmental conditions shape water needs in pregnancy. Endurance athletes experience higher water turnover with more exercise during pregnancy, which is somewhat related to hotter and more humid weather conditions. Because of climate change, highly active pregnant populations may be particularly vulnerable to the effects of warming temperatures.
C1 [Sadhir, Srishti; Mcgrosky, Amanda; Tavormina, Anna; Tomechko, Keri; Pontzer, Herman] Duke Univ, Dept Evolutionary Anthropol, Durham, NC 27708 USA.

[Mcgrosky, Amanda] Elon Univ, Dept Biol, Elon, NC USA.

[Swanson, Zane S.] Ctr Strateg & Int Studies, Global Food & Water Secur Program, Washington, DC USA.

[Tomechko, Keri] Univ Calif San Diego, Sch Med, La Jolla, CA USA.
[Pontzer, Herman] Duke Univ, Duke Global Hlth Inst, Durham, NC 27708 USA.
C3 Duke University; Elon University; Centre for Strategic & International Studies; University of California System; University of California San Diego; Duke University
RP Sadhir, S (autor correspondente), Duke Univ, Dept Evolutionary Anthropol, Durham, NC 27708 USA.
EM srishti.sadhir@duke.edu
RI Sadhir, Srishti/JDX-0199-2023; McGrosky, Amanda/ABM-9253-2022
FU National Science Foundation [DGE 2139754, BA-DDRIG 2316583, BCS-2316555]; Leakey Foundation; Triangle Center for Evolutionary Medicine; Wenner-Gren Foundation
FX We acknowledge several funding organizations, including the National Science Foundation (DGE 2139754, BA-DDRIG 2316583, BCS-2316555), the Leakey Foundation, the Triangle Center for Evolutionary Medicine, and the Wenner-Gren Foundation.
CR Aldea D, 2021, P NATL ACAD SCI USA, V118, DOI 10.1073/pnas.2021722118
Andersen MB, 2020, AM J OBST GYNEC MFM, V2, DOI 10.1016/j.ajogmf.2020.100182
[Anonymous], 2004, DIETARY REFERENCE IN
Armstrong LE, 2018, NUTRIENTS, V10, DOI 10.3390/nu10121928
Beenackers MA, 2012, INT J BEHAV NUTR PHY, V9, DOI 10.1186/1479-5868-9-116
Beis LY, 2012, CLIN J SPORT MED, V22, P254, DOI 10.1097/JSM.0b013e31824a55d7
Bonell A, 2022, LANCET PLANET HEALTH, V6, pE968, DOI 10.1016/S2542-5196(22)00242-X
Brown EA, 2013, TRENDS GENET, V29, P585, DOI 10.1016/j.tig.2013.03.001
Butte NF, 2004, AM J CLIN NUTR, V79, P1078
Choi L., 2021, Package "PhysicalActivity.
Ciss G., 2022, Climate Change 2022: Impacts, Adaptation and Vulnerability Contribution of Working Group II to the Sixth Assessment Report of the Intergovernmental Panel on Climate Change, P1041
El-Sharkawy AM, 2015, NUTR REV, V73, P97, DOI 10.1093/nutrit/nuv038
Evenson KR, 2014, AM J LIFESTYLE MED, V8, P102, DOI 10.1177/1559827613498204
Gibbs BB, 2021, J PHYS ACT HEALTH, V18, P254, DOI 10.1123/jpah.2020-0398
Hampo CC, 2024, HELIYON, V10, DOI 10.1016/j.heliyon.2024.e25801
Hesketh Kathryn R, 2018, Prev Med Rep, V10, P337, DOI 10.1016/j.pmedr.2018.04.012
Hrdy SB, 2016, HORM BEHAV, V77, P272, DOI 10.1016/j.yhbeh.2015.10.016
International Atomic Energy Agency, 2009, IAEA Human Health Series, V3
Jiao A, 2023, JAMA NETW OPEN, V6, DOI 10.1001/jamanetworkopen.2023.32780
Kamberov YG, 2018, J HUM EVOL, V125, P99, DOI 10.1016/j.jhevol.2018.10.008
Kraus WE, 2019, MED SCI SPORT EXER, V51, P1206, DOI 10.1249/MSS.0000000000001932
Löf M, 2011, EUR J CLIN NUTR, V65, P1295, DOI 10.1038/ejcn.2011.129
Mastrucci A, 2019, ENERG BUILDINGS, V186, P405, DOI 10.1016/j.enbuild.2019.01.015

McKay AKA, 2022, INT J SPORT PHYSIOL, V17, P317, DOI 10.1123/ijspp.2021-0451
McKenzie AL, 2017, EUR J NUTR, V56, P2161, DOI 10.1007/s00394-016-1256-3
Migueles JH., 2019, J Meas Phys Behav, V2, P188, DOI [DOI 10.1123/JMPB.2018-0063, 10.1123/jmpb.2018-0063, https://doi.org/10.1123/jmpb.2018-0063]
Mottola MF, 2018, BRIT J SPORT MED, V52, P1339, DOI 10.1136/bjsports-2018-100056
Mottola MF, 2018, J OBSTET GYNAECOL CA, V40, P1528, DOI 10.1016/j.jogc.2018.07.001
Pazhayattil GS, 2015, AM J KIDNEY DIS, V65, P623, DOI 10.1053/j.ajkd.2014.09.027
Pontzer H, 2021, CURR BIOL, V31, P1804, DOI 10.1016/j.cub.2021.02.045
Popkin BM, 2010, NUTR REV, V68, P439, DOI 10.1111/j.1753-4887.2010.00304.x
Powell L., 2004, EVIDENCE BASED PREVE, V1, P135
R Core Team, 2024, R LANG ENV STAT COMP
Raman A, 2004, AM J PHYSIOL-RENAL, V286, pF394, DOI 10.1152/ajprenal.00295.2003
Rasmussen KM, 2009, WEIGHT GAIN DURING PREGNANCY: REEXAMINING THE GUIDELINES, P1
Rosinger AY, 2022, EUR J NUTR, V61, P127, DOI 10.1007/s00394-021-02616-x
Rosinger AY, 2020, AM J HUM BIOL, V32, DOI 10.1002/ajhb.23338
Rothfusz L.P., 1990, The Heat Index "Equation" (or, More Than You Ever Wanted to Know About Heat Index), V2
Rousham EK, 2006, EUR J CLIN NUTR, V60, P393, DOI 10.1038/sj.ejcn.1602329
Rzewnicki R, 2003, PUBLIC HEALTH NUTR, V6, P299, DOI 10.1079/PHN2002427
Samuels L, 2022, INT J BIOMETEOROL, V66, P1505, DOI 10.1007/s00484-022-02301-6
Sandborg J, 2022, MED SCI SPORT EXER, V54, P2054, DOI 10.1249/MSS.0000000000002996
Santos-Lozano A, 2013, INT J SPORTS MED, V34, P975, DOI 10.1055/s-0033-1337945
Shimamoto Hideki, 2000, Journal of Physiological Anthropology and Applied Human Science, V19, P207, DOI 10.2114/jpa.19.207
Song YY, 2023, NUTRIENTS, V15, DOI 10.3390/nu15071739
Spencer S, 2022, FRONT PUBLIC HEALTH, V10, DOI 10.3389/fpubh.2022.785254
Swanson ZS, 2020, AM J HUM BIOL, V32, DOI 10.1002/ajhb.23365
THEUNISSEN IM, 1994, CLIN OBSTET GYNECOL, V37, P3, DOI 10.1097/00003081-199403000-00005
Troiano RP, 2014, BRIT J SPORT MED, V48, DOI 10.1136/bjsports-2014-093546
van Hees VT, 2011, PLOS ONE, V6, DOI 10.1371/journal.pone.0022922
Vietheer A, 2021, SLEEP MED, V83, P89, DOI 10.1016/j.sleep.2021.04.028
Yamada Y, 2022, SCIENCE, V378, P909, DOI 10.1126/science.abm8668
NR 52
TC 0
Z9 0
U1 1

U2 1
PU OXFORD UNIV PRESS
PI OXFORD
PA GREAT CLARENDON ST, OXFORD OX2 6DP, ENGLAND
EI 2050-6201
J9 EVOL MED PUBLIC HLTH
JI Evol. Med. Public Health
PD MAR 1
PY 2025
VL 13
IS 1
BP 25
EP 34
DI 10.1093/emph/eoaf003
PG 10
WC Evolutionary Biology; Public, Environmental & Occupational Health
WE Science Citation Index Expanded (SCI-EXPANDED)
SC Evolutionary Biology; Public, Environmental & Occupational Health
GA Z6V4R
UT WOS:001440256000001
PM 40041548
OA gold
DA 2025-05-07
ER

PT J
AU Spreadbury, I
Samis, AJW
AF Spreadbury, Ian
Samis, Andrew J. W.
TI Evolutionary Aspects of Obesity, Insulin Resistance, and Cardiovascular
Risk
SO CURRENT CARDIOVASCULAR RISK REPORTS
LA English
DT Article
DE Obesity; Metabolic syndrome; Type 2 diabetes; Cardiovascular disease;
Leptin resistance; Evolutionary medicine; Paleolithic diet; Nutrition
transition; Western disease; Carbohydrate density; Acellular
carbohydrates; Kitava study
ID ISCHEMIC-HEART-DISEASE; DIET-INDUCED OBESITY; LEPTIN RESISTANCE;
PHYSICAL-ACTIVITY; GUT MICROBIOTA; FATTY-ACIDS; INTESTINAL MICROBIOTA;
MYOCARDIAL-INFARCTION; PALEOLITHIC DIET; GLYCEMIC INDEX
AB Cardiovascular disease (CVD) is still virtually absent in those rare populations with minimal Western dietary influence. To date, exercise, altered fats, fibre, anti-oxidants or Mediterranean diet do not appear to overcome the discrepancy in CVD between hunter-gatherer and Western populations. The CVD risk factors of obesity and diabetes are driven by increased caloric intake, with carbohydrates potentially implicated. Paradoxically, non-Westernized diets vary widely in macronutrients, glycemic and insulinemic indices, yet apparently produce no obesity or CVD regardless, even with abundant food. 'Ancestral' grain-free whole-food diet may represent the best lifestyle intervention for obesity and CVD. Such diets are composed of the cells of living organisms, while

Western grains, flour and sugar are dense, acellular powders. Bacterial inflammation of the small intestine and vagal afferents appears a crucial step in leptin-resistance and obesity. Therefore it may be important that the Western diet resembles a bacterial growth medium.
C1 [Spreadbury, Ian; Samis, Andrew J. W.] Queens Univ, Kingston Gen Hosp, Gastrointestinal Dis Res Unit, 76 Stuart St, Kingston, ON K7L 2V7, Canada.
C3 Queens University - Canada; Queens University Hospital
RP Spreadbury, I (autor correspondente), Queens Univ, Kingston Gen Hosp, Gastrointestinal Dis Res Unit, 76 Stuart St, Kingston, ON K7L 2V7, Canada.
EM is15@queensu.ca
CR Abramovitch RB, 2006, NAT REV MOL CELL BIO, V7, P601, DOI 10.1038/nrm1984
Alvheim AR, 2012, OBESITY, V20, P1984, DOI 10.1038/oby.2012.38
[Anonymous], J ATHEROSCLER THROMB
[Anonymous], AGR RES SERV NAT AGR
[Anonymous], REQUIRED READING BOT
[Anonymous], CONDITIONS CAUSED TA
Apolzan JW, 2013, APPETITE, V60, P65, DOI 10.1016/j.appet.2012.09.020
Baum SJ, 2012, J CLIN LIPIDOL, V6, P216, DOI 10.1016/j.jacl.2012.04.077
Baumgartner S, 2009, J PERIODONTOL, V80, P759, DOI 10.1902/jop.2009.080376
Berer K, 2011, NATURE, V479, P538, DOI 10.1038/nature10554
Berthoud HR, 2012, ANN NY ACAD SCI, V1264, P36, DOI 10.1111/j.1749-6632.2012.06573.x
BISS K, 1971, NEW ENGL J MED, V284, P694, DOI 10.1056/NEJM197104012841304
Bjerregaard P, 2003, ATHEROSCLEROSIS, V166, P351, DOI 10.1016/S0021-9150(02)00364-7
Boeing H, 2012, EUR J NUTR, V51, P637, DOI 10.1007/s00394-012-0380-y
Bribiescas RG, 2006, NUTR METAB, V3, DOI 10.1186/1743-7075-3-34
Brinkworth GD, 2009, AM J CLIN NUTR, V90, P23, DOI 10.3945/ajcn.2008.27326
Bruckdorfer KR, 2008, P NUTR SOC, V67, P214, DOI 10.1017/S0029665108007052
Bumaschny VF, 2012, J CLIN INVEST, V122, P4203, DOI 10.1172/JCI62543
Caesar R, 2012, GUT, V61, P1701, DOI 10.1136/gutjnl-2011-301689
Cordain L, 2002, EUR J CLIN NUTR, V56, pS42, DOI 10.1038/sj.ejcn.1601353
Danby FW, 2010, CLIN DERMATOL, V28, P598, DOI 10.1016/j.clindermatol.2010.03.017
De Filippo C, 2010, P NATL ACAD SCI USA, V107, P14691, DOI 10.1073/pnas.1005963107
de La Serre CB, 2010, AM J PHYSIOL-GASTR L, V299, pG440, DOI 10.1152/ajpgi.00098.2010
de Lartigue G, 2011, AM J PHYSIOL-ENDOC M, V301, pE187, DOI 10.1152/ajpendo.00056.2011
Desmarchelier C, 2013, BRIT J NUTR, V109, P1518, DOI 10.1017/S0007114512003340
Dewulf EM, 2013, GUT, V62, P1112, DOI 10.1136/gutjnl-2012-303304
Diamond J, 2003, NATURE, V423, P599, DOI 10.1038/423599a
Ding SL, 2010, PLOS ONE, V5, DOI 10.1371/journal.pone.0012191

Ebbeling CB, 2012, JAMA-J AM MED ASSOC, V307, P2627, DOI 10.1001/jama.2012.6607
Foster-Powell K, 2002, AM J CLIN NUTR, V76, P5, DOI 10.1093/ajcn/76.1.5
Frassetto LA, 2009, EUR J CLIN NUTR, V63, P947, DOI 10.1038/ejcn.2009.4
Gardner CD, 2007, JAMA-J AM MED ASSOC, V297, P969, DOI 10.1001/jama.297.9.969
Gautron L, 2011, J CLIN INVEST, V121, P2087, DOI 10.1172/JCI45888
Gross LS, 2004, AM J CLIN NUTR, V79, P774
Gurven M, 2009, PLOS ONE, V4, DOI 10.1371/journal.pone.0006590
Harris WS, 2009, CIRCULATION, V119, P902, DOI 10.1161/CIRCULATIONAHA.108.191627
Holt SHA, 1997, AM J CLIN NUTR, V66, P1264, DOI 10.1093/ajcn/66.5.1264
Hooper L., 2004, Cochrane Database of Systematic Reviews, DOI [DOI 10.1002/14651858.CD003177.PUB2, 10.1002/14651858.CD003177.pub2.]
Hooper L, 2012, COCHRANE DB SYST REV, DOI 10.1002/14651858.CD002137.pub3
Hoppe C, 2009, EUR J CLIN NUTR, V63, P1076, DOI 10.1038/ejcn.2009.34
Howard BV, 2006, JAMA-J AM MED ASSOC, V295, P655, DOI 10.1001/jama.295.6.655
Hujoel P, 2009, J DENT RES, V88, P490, DOI 10.1177/0022034509337700
Jonsson Tommy, 2005, BMC Endocr Disord, V5, P10, DOI 10.1186/1472-6823-5-10
Jönsson T, 2010, NUTR METAB, V7, DOI 10.1186/1743-7075-7-85
Jönsson T, 2009, CARDIOVASC DIABETOL, V8, DOI 10.1186/1475-2840-8-35
Jorgensen ME, 2008, ATHEROSCLEROSIS, V196, P772, DOI 10.1016/j.atherosclerosis.2007.01.008
Kadooka Y, 2010, EUR J CLIN NUTR, V64, P636, DOI 10.1038/ejcn.2010.19
Kantorová E, 2011, NEUROENDOCRINOL LETT, V32, P716
KJELSBERG MO, 1982, JAMA-J AM MED ASSOC, V248, P1465
Koopman JJE, 2012, PLOS ONE, V7, DOI 10.1371/journal.pone.0046855
Lagiou P, 2012, BMJ-BRIT MED J, V344, DOI 10.1136/bmj.e4026
Lam V, 2012, FASEB J, V26, P1727, DOI 10.1096/fj.11-197921
Lee IM, 1997, AGING CLIN EXP RES, V9, P2, DOI 10.1007/BF03340123
Lindeberg S, 1997, AM J CLIN NUTR, V66, P845, DOI 10.1093/ajcn/66.4.845
Lindeberg S, 2007, DIABETOLOGIA, V50, P1795, DOI 10.1007/s00125-007-0716-y
LINDEBERG S, 1993, J INTERN MED, V233, P269, DOI 10.1111/j.1365-2796.1993.tb00986.x
Lindeberg S, 2003, SCAND J CLIN LAB INV, V63, P175, DOI 10.1080/00365510310000268
Lindeberg S, 2001, J INTERN MED, V249, P553, DOI 10.1046/j.1365-2796.2001.00845.x
LINDEBERG S, 1994, J INTERN MED, V236, P331, DOI 10.1111/j.1365-2796.1994.tb00804.x
Lindeberg SA, 1999, METABOLISM, V48, P1216, DOI 10.1016/S0026-0495(99)90258-5
Lindeberg S, 2012, AM J HUM BIOL, V24, P110, DOI 10.1002/ajhb.22218
Lindgärde F, 2004, METABOLISM, V53, P1355, DOI 10.1016/j.metabol.2004.04.012
Lockhart PB, 2012, CIRCULATION, V125, P2520, DOI 10.1161/CIR.0b013e31825719f3

Lustig RH, 2012, NATURE, V482, P27, DOI 10.1038/482027a
Malik VS, 2010, DIABETES CARE, V33, P2477, DOI 10.2337/dc10-1079
Mancuso P, 2012, J IMMUNOL, V189, P867, DOI 10.4049/jimmunol.1200465
MANN GV, 1978, ATHEROSCLEROSIS, V29, P265, DOI 10.1016/0021-9150(78)90014-X
Mekary RA, 2010, OBESITY, V18, P167, DOI 10.1038/oby.2009.170
Mozaffarian D, 2006, NEW ENGL J MED, V354, P1601, DOI 10.1056/NEJMra054035
Musso G, 2011, ANNU REV MED, V62, P361, DOI 10.1146/annurev-med-012510-175505
Myers MG, 2010, TRENDS ENDOCRIN MET, V21, P643, DOI 10.1016/j.tem.2010.08.002
Nasidze I, 2011, PLOS ONE, V6, DOI 10.1371/journal.pone.0023352
ODEA K, 1984, DIABETES, V33, P596, DOI 10.2337/diabetes.33.6.596
Österdahl M, 2008, EUR J CLIN NUTR, V62, P682, DOI 10.1038/sj.ejcn.1602790
PAGE LB, 1974, CIRCULATION, V49, P1132, DOI 10.1161/01.CIR.49.6.1132
Pawlak DB, 2004, LANCET, V364, P778, DOI 10.1016/S0140-6736(04)16937-7
Pontzer H, 2012, PLOS ONE, V7, DOI 10.1371/journal.pone.0040503
Rabot S, 2010, FASEB J, V24, P4948, DOI 10.1096/fj.10-164921
Rajan A, 2012, CELL, V151, P123, DOI 10.1016/j.cell.2012.08.019
Redman LM, 2009, PLOS ONE, V4, DOI 10.1371/journal.pone.0004377
Reed AS, 2010, DIABETES, V59, P894, DOI 10.2337/db09-1024
Ridley EV, 2012, PLOS ONE, V7, DOI 10.1371/journal.pone.0036765
SCHAEFER O, 1959, Can Med Assoc J, V81, P386
Sesso HD, 2000, CIRCULATION, V102, P975, DOI 10.1161/01.CIR.102.9.975
Shin SC, 2011, SCIENCE, V334, P670, DOI 10.1126/science.1212782
Simopoulos AP, 1999, PROSTAG LEUKOTR ESS, V60, P421, DOI 10.1016/S0952-3278(99)80023-4
Sjögren P, 2010, AM J CLIN NUTR, V92, P967, DOI 10.3945/ajcn.2010.29345
Spreadbury I, 2012, DIABETES METAB SYNDR, V5, P175, DOI 10.2147/DMSO.S33473
Ströhle A, 2011, NUTR RES, V31, P429, DOI 10.1016/j.nutres.2011.05.003
Struijk EA, 2013, NUTR METAB CARDIOVAS, V23, P822, DOI 10.1016/j.numecd.2012.05.011
Szostak J, 2011, CLIN SCI, V121, P91, DOI 10.1042/CS20100520
Thompson PD, 2003, ARTERIOSCL THROM VAS, V23, pE42, DOI 10.1161/01.ATV.0000089628.63625.D4
Thornley S, 2012, INTERN MED J, V42, P46, DOI 10.1111/j.1445-5994.2012.02902.x
Vrieze A, 2012, GASTROENTEROLOGY, V143, P913, DOI 10.1053/j.gastro.2012.06.031
Wanders AJ, 2011, OBES REV, V12, P724, DOI 10.1111/j.1467-789X.2011.00895.x
Warensjö E, 2010, AM J CLIN NUTR, V92, P194, DOI 10.3945/ajcn.2009.29054
Williams PG, 2012, NUTR REV, V70, P80, DOI 10.1111/j.1753-4887.2011.00452.x
World Health Organization, 2012, WORLD HLTH STAT 2012
Zienczuk N, 2012, NUTR J, V11, DOI 10.1186/1475-2891-11-73
Zimmet PZ, 1997, J DIABETES COMPLICAT, V11, P60, DOI 10.1016/S1056-8727(96)00090-6
NR 100

TC 1
Z9 2
U1 0
U2 3
PU SPRINGER
PI NEW YORK
PA ONE NEW YORK PLAZA, SUITE 4600, NEW YORK, NY, UNITED STATES
SN 1932-9520
EI 1932-9563
J9 CURR CARDIOVASC RISK
JI Curr. Cardiovascu. Risk Rep.
PD APR
PY 2013
VL 7
IS 2
BP 136
EP 146
DI 10.1007/s12170-013-0293-1
PG 11
WC Cardiac & Cardiovascular Systems
WE Emerging Sources Citation Index (ESCI)
SC Cardiovascular System & Cardiology
GA V5T8Z
UT WOS:000219934800009
DA 2025-05-07
ER

PT J
AU Lozano, GA
AF Lozano, George A.
TI Evolutionary explanations in medicine: How do they differ and how to
   benefit from them
SO MEDICAL HYPOTHESES
LA English
DT Article
ID DARWINIAN MEDICINE
AB Evolutionary explanations, many of which have appeared on the pages of this journal, are becoming more pervasive and influential in medicine, so it is becoming more important to understand how these types of explanations differ from the proximate approach that is more common in medicine, and how the evolutionary approach can contribute to medicine. Understanding of any biological phenomenon can occur at four levels: (1) ontogeny (2) causation, (3) function and (4) evolution. These approaches are not mutually exclusive, and whereas the first two are more common in medical practice, a complete explanation requires all four levels of analysis. Two major differences among these approaches are the apparent degree of immediacy associated with them, and the extent to which they apply to individuals rather than populations. Criticisms of adaptive explanations often arise from a failure to understand the complementary nature of these four types of explanations. Other unwarranted criticisms result from a failure to appreciate that adaptive explanations often apply to populations, not individuals. A third type of criticism is driven by the mistaken belief that adaptive explanations somehow justify

morally reprehensible behaviours. Finally, evolutionary explanations sometimes face the criticism of "personal incredulity".
Adaptive explanations must be consistent with basic evolutionary concepts and must adhere to the physical reality of the phenomenon in question. Their value, however, comes not in devising a seemingly rational explanation, but in their predictions. Testable predictions must be explicitly stated and clearly articulated. They must differ from those of arising from other hypotheses and must not only be interesting to evolutionary biologists, but also useful to medical practitioners. Integration of the proximate and the ultimate approaches is possible and potentially beneficial to both evolutionists and physicians, but it requires some basic understanding of our differences and a desire to co-operate. (C) 2009 Elsevier Ltd. All rights reserved.
C1 Univ Tartu, Inst Ecol & Earth Sci, Dept Zool, EE-51014 Tartu, Estonia.
C3 University of Tartu; Tartu University Institute of Ecology & Earth Sciences
RP Lozano, GA (autor correspondente), Univ Tartu, Inst Ecol & Earth Sci, Dept Zool, Vanemuise 46, EE-51014 Tartu, Estonia.
EM george.lozano@ut.ee
RI Lozano, George A/E-8881-2012
OI Lozano, George A/0000-0002-1369-1408
FU Estonian Ministry of Education and Science [0180004s09]; European Union
FX I thank Drs. M. Kery, A. Lichtenwalner and D. LaFleur for offering their views on this paper, from an ecological, veterinary and medical perspective, respectively. G.A.L. was supported by the Estonian Ministry of Education and Science (target-financing project No. 0180004s09) and the European Union through the European Regional Development Fund (Center of Excellence FIBIR).
CR [Anonymous], 1986, The blind watchmaker
Archer J, 2009, BEHAV BRAIN SCI, V32, P249, DOI 10.1017/S0140525X09990951
Charlton BG, 1997, J ROY SOC MED, V90, P397, DOI 10.1177/014107689709000710
CHIAPPEDI M, 2009, MED HYPOTHESES, V72, P473
Daly M., 1988, HOMICIDE
Dewar CS, 2003, MED HYPOTHESES, V60, P225, DOI 10.1016/S0306-9877(02)00363-8
Eagles JM, 2004, MED HYPOTHESES, V63, P767, DOI 10.1016/j.mehy.2004.07.002
Erren TC, 2009, MED HYPOTHESES, V73, P278, DOI 10.1016/j.mehy.2009.05.002
Gofrit ON, 2006, MED HYPOTHESES, V67, P1245, DOI 10.1016/j.mehy.2006.04.052
Horrobin DF, 2004, MED HYPOTHESES, V62, P3, DOI 10.1016/S0306-9877(03)00277-9
Lozano GA, 2008, MED HYPOTHESES, V71, P933, DOI 10.1016/j.mehy.2008.07.013
Montefiore Hugh., 1985, PROBABILITY GOD
Nesse R.M., 2007, Evolutionary medicine, second edition, V2nd, P416, DOI DOI 10.1098/rspb.2007.1028

   Nesse R.M., 1995, Why we get sick: The new science of Darwinian medicine
   Nesse RM, 2008, EVOL APPL, V1, P28, DOI 10.1111/j.1752-4571.2007.00006.x
   Pasca SP, 2008, MED HYPOTHESES, V71, P472, DOI 10.1016/j.mehy.2008.04.009
   PLATT JR, 1964, SCIENCE, V146, P347, DOI 10.1126/science.146.3642.347
   Saad G, 2007, MED HYPOTHESES, V68, P692, DOI 10.1016/j.mehy.2006.08.012
   THORNHILL R, 1983, ETHOL SOCIOBIOL, V4, P137, DOI 10.1016/0162-3095(83)90027-4
   Tinbergen N., 1963, Zeitschrift fuer Tierpsychologie, V20, P410
   WILLIAMS GC, 1991, Q REV BIOL, V66, P1, DOI 10.1086/417048
NR 22
TC 7
Z9 8
U1 0
U2 21
PU CHURCHILL LIVINGSTONE
PI EDINBURGH
PA JOURNAL PRODUCTION DEPT, ROBERT STEVENSON HOUSE, 1-3 BAXTERS PLACE,
   LEITH WALK, EDINBURGH EH1 3AF, MIDLOTHIAN, SCOTLAND
SN 0306-9877
J9 MED HYPOTHESES
JI Med. Hypotheses
PD APR
PY 2010
VL 74
IS 4
BP 746
EP 749
DI 10.1016/j.mehy.2009.11.003
PG 4
WC Medicine, Research & Experimental
WE Science Citation Index Expanded (SCI-EXPANDED)
SC Research & Experimental Medicine
GA 583RV
UT WOS:000276697800034
PM 19963325
DA 2025-05-07
ER

PT J
AU Lee, DB
   Calendo, G
   Kopec, K
   Henry, R
   Coutts, S
   McCarthy, D
   Murphy, HM
AF Lee, Debbie
   Calendo, Gennaro
   Kopec, Kristin
   Henry, Rebekah

Coutts, Scott
McCarthy, David
Murphy, Heather M.
TI The Impact of Pipe Material on the Diversity of Microbial Communities in
Drinking Water Distribution Systems
SO FRONTIERS IN MICROBIOLOGY
LA English
DT Article
DE pipe material; annular reactor; drinking water; microbiome; biofilm
ID BIOFILM FORMATION; LEGIONELLA-PNEUMOPHILA; ESCHERICHIA-COLI; CHLORINE DIOXIDE; STAINLESS-STEEL; HOUSEHOLD TAPS; BULK WATER; CAST-IRON; BACTERIAL; DISINFECTION
AB As many cities around the world face the prospect of replacing aging drinking water distribution systems (DWDS), water utilities must make careful decisions on new pipe material (e.g., cement-lined or PVC) for these systems. These decisions are informed by cost, physical integrity, and impact on microbiological and physicochemical water quality. Indeed, pipe material can impact the development of biofilm in DWDS that can harbor pathogens and impact drinking water quality. Annular reactors (ARs) with cast iron and cement coupons fed with chloraminated water from a municipal DWDS were used to investigate the impact of pipe material on biofilm development and composition over 16 months. The ARs were plumbed as closely as possible to the water main in the basement of an academic building to simulate distribution system conditions. Biofilm communities on coupons were characterized using 16S rRNA sequencing. In the cast iron reactors, beta-proteobacteria, Actinobacteria, and alpha-proteobacteria were similarly relatively abundant (24.1, 22.5, and 22.4%, respectively) while in the cement reactors, alpha-proteobacteria and Actinobacteria were more relatively abundant (36.3 and 35.2%, respectively) compared to beta-proteobacteria (12.8%). Mean alpha diversity (estimated with Shannon H and Faith's Phylogenetic Difference indices) was greater in cast iron reactors (Shannon: 5.00 +/- 0.41; Faith's PD: 15.40 +/- 2.88) than in cement reactors (Shannon: 4.16 +/- 0.78; Faith's PD: 13.00 +/- 2.01). PCoA of Bray-Curtis dissimilarities indicated that communities in cast iron ARs, cement ARs, bulk distribution system water, and distribution system pipe biofilm were distinct. The mean relative abundance of Mycobacterium spp. was greater in the cement reactors (34.8 +/- 18.6%) than in the cast iron reactors (21.7 +/- 11.9%). In contrast, the mean relative abundance of Legionella spp. trended higher in biofilm from cast iron reactors (0.5 +/- 0.7%) than biofilm in cement reactors (0.01 +/- 0.01%). These results suggest that pipe material is associated with differences in the diversity, bacterial composition, and opportunistic pathogen prevalence in biofilm of DWDS.
C1 [Lee, Debbie; Calendo, Gennaro; Kopec, Kristin; Murphy, Heather M.] Temple Univ, Coll Publ Hlth, Dept Epidemiol & Biostat, Water Hlth & Appl Microbiol Lab WHAM Lab, Philadelphia, PA 19122 USA.
[Henry, Rebekah; McCarthy, David] Monash Univ, Dept Civil Engn, Environm & Publ Hlth Microbiol Lab EPHM Lab, Clayton, Vic, Australia.
[Coutts, Scott] Monash Univ, Dept Microbiol, Micromon, Clayton, Vic, Australia.
[Murphy, Heather M.] Univ Guelph, Ontario Vet Coll, Dept Pathobiol, Water Hlth & Appl Microbiol Lab WHAM Lab, Guelph, ON, Canada.
C3 Pennsylvania Commonwealth System of Higher Education (PCSHE); Temple

University; Monash University; Monash University; University of Guelph;
Ontario Veterinary College
RP Murphy, HM (autor correspondente), Temple Univ, Coll Publ Hlth, Dept Epidemiol & Biostat, Water Hlth & Appl Microbiol Lab WHAM Lab, Philadelphia, PA 19122 USA.; Murphy, HM (autor correspondente), Univ Guelph, Ontario Vet Coll, Dept Pathobiol, Water Hlth & Appl Microbiol Lab WHAM Lab, Guelph, ON, Canada.
EM heather.murphy@uoguelph.ca
RI Calendo, Gennaro/KBA-0187-2024
OI Murphy, Heather/0000-0002-0435-8515; Henry, Rebekah/0000-0001-6530-8557;
McCarthy, David/0000-0001-8845-6501
FU Institute for Genomics and Evolutionary Medicine (iGEM) at Temple Universit; Canada Research Chairs Program [950-232787]
FX This work was funded by the Institute for Genomics and Evolutionary Medicine (iGEM) at Temple University. The authors would like to acknowledge funding from the Canada Research Chairs Program (Grant #950-232787).
CR Aggarwal S, 2018, ENVIRON SCI TECHNOL, V52, P13077, DOI 10.1021/acs.est.8b02607
American Water Works Association (AWWA), 2017, BUR NO LONG CONFR AM
Best Corliss A, 2009, Hand (N Y), V4, P165, DOI 10.1007/s11552-008-9147-6
Boamah DK, 2017, FRONT CELL INFECT MI, V7, DOI 10.3389/fcimb.2017.00477
Bolyen E, 2019, NAT BIOTECHNOL, V37, P852, DOI 10.1038/s41587-019-0209-9
Bouam A, 2017, GENOME ANNOUNCEMENTS, V5, DOI 10.1128/genomeA.00292-17
Bruaset S, 2018, WATER-SUI, V10, DOI 10.3390/w10040411
Minh BQ, 2013, MOL BIOL EVOL, V30, P1188, DOI 10.1093/molbev/mst024
Burmolle M, 2006, APPL ENVIRON MICROB, V72, P3916, DOI 10.1128/AEM.03022-05
Buse HY, 2014, FEMS MICROBIOL ECOL, V88, P280, DOI 10.1111/1574-6941.12294
Callahan BJ, 2016, NAT METHODS, V13, P581, DOI [10.1038/NMETH.3869, 10.1038/nmeth.3869]
Camper A.K, 1995, FACTORS INFLUENCING
Chao YQ, 2015, SCI REP-UK, V5, DOI 10.1038/srep10044
Collier SA, 2021, EMERG INFECT DIS, V27, P140, DOI 10.3201/eid2701.190676
Cruz MC, 2020, WATER RES, V176, DOI 10.1016/j.watres.2020.115738
Da Re S, 2013, PLOS ONE, V8, DOI 10.1371/journal.pone.0061628
Davis NM, 2018, MICROBIOME, V6, DOI 10.1186/s40168-018-0605-2
Donohue MJ, 2019, APPL ENVIRON MICROB, V85, DOI 10.1128/AEM.01942-19
Douglas GM, 2020, PICRUSt2: An improved and extensible approach for metagenome inference, DOI DOI 10.1101/672295
Douterelo I, 2016, APPL ENVIRON MICROB, V82, P4155, DOI 10.1128/AEM.00109-16
Douterelo I, 2014, WATER RES, V54, P100, DOI 10.1016/j.watres.2014.01.049
Douterelo I, 2013, WATER RES, V47, P503, DOI 10.1016/j.watres.2012.09.053
Eaton A.D., 1995, AM J PUBLIC HEALTH, Vnineteenth

Elvers KT, 2002, J IND MICROBIOL BIOT, V29, P331, DOI 10.1038/sj.jim.7000318
Fish KE, 2018, FRONT MICROBIOL, V9, DOI 10.3389/fmicb.2018.02519
Fish KE, 2015, PLOS ONE, V10, DOI 10.1371/journal.pone.0115824
Flemming HC, 2010, NAT REV MICROBIOL, V8, P623, DOI 10.1038/nrmicro2415
Flemming HC, 2002, WA SCI TECHNOL, V2, P271, DOI 10.2166/ws.2002.0032
Folkman S., 2018, WATER MAIN BREAK RAT
Fu YL, 2021, SCI TOTAL ENVIRON, V764, DOI 10.1016/j.scitotenv.2020.142851
Gagnon GA, 2005, WATER RES, V39, P1809, DOI 10.1016/j.watres.2005.02.004
Gomes IB, 2014, WATER RES, V62, P63, DOI 10.1016/j.watres.2014.05.039
Guerrieri E, 2008, CURR MICROBIOL, V57, P532, DOI 10.1007/s00284-008-9237-2
Haig SJ, 2020, ENVIRON SCI-WAT RES, V6, P3032, DOI [10.1039/D0EW00723D, 10.1039/d0ew00723d]
Henne K, 2012, APPL ENVIRON MICROB, V78, P3530, DOI 10.1128/AEM.06373-11
Ingerson-Mahar M., 2012, MICROBES PIPES MIP M, DOI 10.1128/AAMCol.Apr.2012
Ji P, 2015, PLOS ONE, V10, DOI 10.1371/journal.pone.0141087
Kalyaanamoorthy S, 2017, NAT METHODS, V14, P587, DOI [10.1038/NMETH.4285, 10.1038/nmeth.4285]
Katoh K, 2013, MOL BIOL EVOL, V30, P772, DOI 10.1093/molbev/mst010
Kerr CJ, 1999, J APPL MICROBIOL, V85, p29S, DOI 10.1111/j.1365-2672.1998.tb05280.x
Krishna KCB, 2013, WATER RES, V47, P4666, DOI 10.1016/j.watres.2013.04.035
Nguyen LT, 2015, MOL BIOL EVOL, V32, P268, DOI 10.1093/molbev/msu300
Lautenschlager K, 2010, WATER RES, V44, P4868, DOI 10.1016/j.watres.2010.07.032
LeChevallier Mark W., 2003, Journal of Water and Health, V1, P3
LECHEVALLIER MW, 1993, J AM WATER WORKS ASS, V85, P111
Lehtola MJ, 2007, APPL ENVIRON MICROB, V73, P2854, DOI 10.1128/AEM.02916-06
Li WY, 2020, CHEMOSPHERE, V242, DOI 10.1016/j.chemosphere.2019.125310
Lin WF, 2013, APPL MICROBIOL BIOT, V97, P8393, DOI 10.1007/s00253-012-4557-3
Ling FQ, 2018, ISME J, V12, P1520, DOI [10.1038/s41396-018-0101-5, 10.1038/S41396-018-0101-5]
Ling FQ, 2013, MICROBES ENVIRON, V28, P50, DOI 10.1264/jsme2.ME12095
Liu G, 2014, ENVIRON SCI TECHNOL, V48, P5467, DOI 10.1021/es5009467
Liu RY, 2014, J ENVIRON SCI, V26, P865, DOI 10.1016/S1001-0742(13)60481-7
Lührig K, 2015, MICROBES ENVIRON, V30, P99, DOI 10.1264/jsme2.ME14123
Mandal Siddhartha, 2015, Microbial Ecology in Health and Disease, V26, P27663, DOI 10.3402/mehd.v26.27663
Manuel CM, 2007, WATER RES, V41, P551, DOI 10.1016/j.watres.2006.11.007
Martin M., 2011, EMBnet.journal, V17, P10, DOI DOI 10.14806/EJ.17.1.200
Martiny AC, 2003, APPL ENVIRON MICROB, V69, P6899, DOI 10.1128/AEM.69.11.6899-6907.2003

Morton SC, 2005, WATER RES, V39, P2883, DOI 10.1016/j.watres.2005.05.024
Murga R, 2001, MICROBIOL-SGM, V147, P3121, DOI 10.1099/00221287-147-11-3121
Murphy HM, 2008, WATER RES, V42, P2083, DOI 10.1016/j.watres.2007.12.020
Nadell CD, 2016, NAT REV MICROBIOL, V14, P589, DOI 10.1038/nrmicro.2016.84
Neu L, 2019, FRONT MICROBIOL, V10, DOI 10.3389/fmicb.2019.02446
Nguyen C, 2012, WATER RES, V46, P611, DOI 10.1016/j.watres.2011.11.006
Niquette P, 2000, WATER RES, V34, P1952, DOI 10.1016/S0043-1354(99)00307-3
Norton CD, 2000, APPL ENVIRON MICROB, V66, P268, DOI 10.1128/AEM.66.1.268-276.2000
Nygård K, 2007, INT J EPIDEMIOL, V36, P873, DOI 10.1093/ije/dym029
Oksanen Jari, 2024, CRAN
Perrin Y, 2019, WATER RES, V149, P375, DOI 10.1016/j.watres.2018.11.013
Proctor CR, 2017, MICROBIOME, V5, DOI 10.1186/s40168-017-0348-5
Quast C, 2013, NUCLEIC ACIDS RES, V41, pD590, DOI 10.1093/nar/gks1219
Rendueles O, 2015, MICROBIOL SPECTR, V3, DOI 10.1128/microbiolspec.MB-0009-2014
Renwick DV, 2019, J AM WATER WORKS ASS, V111, P42, DOI 10.1002/awwa.1235
Revetta RP, 2016, J APPL MICROBIOL, V121, P294, DOI 10.1111/jam.13150
Shen Y, 2015, ENVIRON SCI TECHNOL, V49, P4274, DOI 10.1021/es505842v
Shields-Cutler RR, 2018, FRONT MICROBIOL, V9, DOI 10.3389/fmicb.2018.00785
Simoes LC, 2010, APPL ENVIRON MICROB, V76, P6673, DOI 10.1128/AEM.00872-10
Smith CM, 2009, APPL ENVIRON MICROB, V75, P5284, DOI 10.1128/AEM.00456-09
Stewart CR, 2012, PLOS ONE, V7, DOI 10.1371/journal.pone.0050560
Taylor RH, 2000, APPL ENVIRON MICROB, V66, P1702, DOI 10.1128/AEM.66.4.1702-1705.2000
United States Environmental Protection Agency (USEPA), 2009, FACTOIDS DRINK WAT G
United States Environmental Protection Agency (USEPA), 2009, BAS INF CHLOR
van der Kooij D, 2005, WATER RES, V39, P2789, DOI 10.1016/j.watres.2005.04.075
van der Kooij D, 2018, APPL ENVIRON MICROB, V84, DOI 10.1128/AEM.01732-18
Wang H, 2014, ENVIRON SCI TECHNOL, V48, P1426, DOI 10.1021/es402636u
Wang H, 2012, ENVIRON SCI TECHNOL, V46, P11566, DOI 10.1021/es303212a
WILKINSON HW, 1985, J CLIN MICROBIOL, V22, P488, DOI 10.1128/JCM.22.4.488-489.1985
Wingender J, 2011, INT J HYG ENVIR HEAL, V214, P417, DOI 10.1016/j.ijheh.2011.05.009
Yu J, 2010, WATER SCI TECHNOL, V61, P163, DOI 10.2166/wst.2010.813
Zhu ZB, 2014, APPL BIOCHEM BIOTECH, V173, P1564, DOI 10.1007/s12010-014-0935-x
NR 89
TC 25

Z9 27
U1 5
U2 64
PU FRONTIERS MEDIA SA
PI LAUSANNE
PA AVENUE DU TRIBUNAL FEDERAL 34, LAUSANNE, CH-1015, SWITZERLAND
EI 1664-302X
J9 FRONT MICROBIOL
JI Front. Microbiol.
PD DEC 21
PY 2021
VL 12
AR 779016
DI 10.3389/fmicb.2021.779016
PG 14
WC Microbiology
WE Science Citation Index Expanded (SCI-EXPANDED)
SC Microbiology
GA YB5VE
UT WOS:000739078900001
PM 34992587
OA gold, Green Published
DA 2025-05-07
ER

PT J
AU Guo, ZM
Yuan, MY
Chai, JN
AF Guo, Zhimin
Yuan, Mengyao
Chai, Jiannan
TI Mini review advantages and limitations of lytic phages compared with chemical antibiotics to combat bacterial infections
SO HELIYON
LA English
DT Review
DE Bacteria; Antimicrobial resistance; Phage; Lytic phage; Phage therapy; Antibiotic
ID BACTERIOPHAGE THERAPY; COMBINATION THERAPY; ANTIBODY-RESPONSE; ORAL APPLICATION; RESISTANCE; VOLUNTEERS; EVOLUTION; COCKTAIL; DELIVERY; SYSTEMS
AB The overuse of antibiotics has caused the emergence of antibiotic-resistant strains, such as multidrug-resistant, extensively drug-resistant, and pandrug-resistant bacteria. The treatment of infections caused by such strains has become a formidable challenge. In the post-antibiotic era, phage therapy is an attractive solution for this problem and some successful phase 1 and 2 studies have demonstrated the efficacy and safety of phage therapy over the last decade. It is a form of evolutionary medicine, phages exhibit immunomodulatory and anti-inflammatory properties. However, phage therapy is limited by factors, such as the narrow spectrum of host strains, the special pharmacokinetics and pharmacodynamics in vivo, immune responses, and the development of phage resistance. The aim of this minireview was to compare the potencies

of lytic phages and chemical antibiotics to treat bacterial infections. The advantages of phage therapy has fewer side effects, self-replication, evolution, bacterial biofilms eradication, immunomodulatory and anti-inflammatory properties compared with chemical antibiotics. Meanwhile, the disadvantages of phage therapy include the narrow spectrum of available host strains, the special pharmacokinetics and pharmacodynamics in vivo, immune responses, and phage resistance hurdles. Recently, some researchers continue to make efforts to overcome these limitations of phage therapy. Phage therapy will be a welcome addition to the gamut of options available for treating antibiotic-resistant bacterial infections. We focus on the advantages and limitations of phage therapy with the intention of exploiting the advantages and overcoming the limitations.
C1 [Guo, Zhimin; Yuan, Mengyao] First Hosp Jilin Univ, Infect Dis & Pathogen Biol Ctr, Dept Lab Med, Changchun 130021, Peoples R China.
[Chai, Jiannan] First Hosp Jilin Univ, Dept Lab Med, Changchun 130021, Peoples R China.
C3 Jilin University; Jilin University
RP Guo, ZM (autor correspondente), First Hosp Jilin Univ, Infect Dis & Pathogen Biol Ctr, Dept Lab Med, Changchun 130021, Peoples R China.
EM amily@jlu.edu.cn
FU National Natural Science Foundation of China [81802056]; China Postdoctoral Science Foundation [2019M651215]; Scientific Research Fundation of the Education Department of Jilin Province [JJKH20231216KJ]; Achievement Transformation Project of the First Hospital of Jilin University [JDYYZH-2022006, JDYY2021-A0012, 2020-ZL-05]; Science and Technology Innovation Platform of Jilin Province [YDZJ202202CXJD050]
FX <BOLD>Acknowledgements</BOLD> This work was supported by grants from the
National Natural Science Foundation of China (No. 81802056) , China Postdoctoral Science Foundation (No. 2019M651215) , the Scientific Research Fundation of the Education Department of Jilin Province (No. JJKH20231216KJ) , the Achievement Transformation Project of the First Hospital of Jilin University (No. JDYYZH-2022006, No. JDYY2021-A0012, No. yjs2022007 and No. 2020-ZL-05) and the Science and Technology Innovation Platform of Jilin Province (YDZJ202202CXJD050) .
CR Alseth EO, 2019, NATURE, V574, P549, DOI 10.1038/s41586-019-1662-9
Anand T, 2020, J GLOB ANTIMICROB RE, V21, P34, DOI 10.1016/j.jgar.2019.09.018
ARONOW R, 1964, J EXP MED, V120, P943, DOI 10.1084/jem.120.5.943
Aslam B, 2018, INFECT DRUG RESIST, V11, P1645, DOI 10.2147/IDR.S173867
BAKER A G, 1963, Pa Med J, V66, P25
BAKER A G, 1958, Am Pract Dig Treat, V9, P591
BARFOOT R, 1989, IMMUNOLOGY, V68, P233
Barr JJ, 2017, IMMUNOL REV, V279, P106, DOI 10.1111/imr.12565
Barr JJ, 2013, P NATL ACAD SCI USA, V110, P10771, DOI 10.1073/pnas.1305923110
Biswas B, 2002, INFECT IMMUN, V70, P204, DOI 10.1128/IAI.70.1.204-210.2002
Borysowski J, 2017, VIRUSES-BASEL, V9, DOI 10.3390/v9020036
Bretin A, 2018, AM J PHYSIOL-ENDOC M, V315, pE1, DOI 10.1152/ajpendo.00014.2018
Bruttin A, 2005, ANTIMICROB AGENTS CH, V49, P2874, DOI 10.1128/AAC.49.7.2874-2878.2005

Bruynoghe R, 1921, CR SOC BIOL, V85, P1120
Bundoc VG, 2007, EXP EYE RES, V85, P575, DOI 10.1016/j.exer.2007.07.005
Camara-Wilpert S, 2023, NATURE, V623, P601, DOI 10.1038/s41586-023-06612-5
Cars O, 2014, UPSALA J MED SCI, V119, P209, DOI 10.3109/03009734.2014.912700
Chabas H, 2016, P ROY SOC B-BIOL SCI, V283, DOI 10.1098/rspb.2016.0721
Chan BK, 2018, EVOL MED PUBLIC HLTH, P60, DOI 10.1093/emph/eoy005
Chan BK, 2016, SCI REP-UK, V6, DOI 10.1038/srep26717
Chaudhry WN, 2017, PLOS ONE, V12, DOI 10.1371/journal.pone.0168615
Coates ARM, 2020, EXPERT REV ANTI-INFE, V18, P5, DOI 10.1080/14787210.2020.1705155
Colom J, 2015, APPL ENVIRON MICROB, V81, P4841, DOI 10.1128/AEM.00812-15
Comeau AM, 2007, PLOS ONE, V2, DOI 10.1371/journal.pone.0000799
d'Herelle F, 1917, CR HEBD ACAD SCI, V165, P373
Dabrowska K, 2014, J VIROL, V88, P12551, DOI 10.1128/JVI.02043-14
De Oliveira DMP, 2020, CLIN MICROBIOL REV, V33, DOI 10.1128/CMR.00181-19
De Paepe M, 2014, PLOS GENET, V10, DOI 10.1371/journal.pgen.1004181
Di Giovine M, 2001, VIROLOGY, V282, P102, DOI 10.1006/viro.2000.0809
Fabijan AP, 2020, MED J AUSTRALIA, V212, P279, DOI 10.5694/mja2.50355
Fauconnier A, 2017, EMBO REP, V18, P198, DOI 10.15252/embr.201643250
Febvre HP, 2019, NUTRIENTS, V11, DOI 10.3390/nu11030666
Fillol-Salom A, 2022, CELL, V185, P3248, DOI 10.1016/j.cell.2022.07.014
Frenkel D, 2002, P NATL ACAD SCI USA, V99, P5675, DOI 10.1073/pnas.072027199
GEIER MR, 1973, NATURE, V246, P221, DOI 10.1038/246221a0
Gindin M, 2019, J AM COLL NUTR, V38, P68, DOI 10.1080/07315724.2018.1483783
Ginn AN, 2017, PATHOLOGY, V49, P304, DOI 10.1016/j.pathol.2016.10.014
Golembo M, 2022, Skin Health Dis, V2, pe93, DOI 10.1002/ski2.93
Gorski A, 2006, TRANSPL P, V38, P331, DOI 10.1016/j.transproceed.2005.12.073
Gorski A, 2003, TRANSPLANT P, V35, P2372, DOI 10.1016/S0041-1345(03)00811-X
Górski A, 2017, FRONT IMMUNOL, V8, DOI 10.3389/fimmu.2017.01783
Gorski Andrzej, 2003, Med Immunol, V2, P2, DOI 10.1186/1476-9433-2-2
Grygorcewicz B, 2022, CURR ISSUES MOL BIOL, V44, P1316, DOI 10.3390/cimb44030088
Guo ZM, 2020, MICROB PATHOGENESIS, V142, DOI 10.1016/j.micpath.2020.104048
Guo ZM, 2017, FRONT MICROBIOL, V8, DOI 10.3389/fmicb.2017.01460
HAJEK P, 1966, FOLIA MICROBIOL, V11, P282, DOI 10.1007/BF02878898
Hodyra-Stefaniak K, 2015, SCI REP-UK, V5, DOI 10.1038/srep14802
Hughes KA, 1998, MICROBIOL-UK, V144, P3039, DOI 10.1099/00221287-144-11-3039
Humphreys G, 2016, B WORLD HEALTH ORGAN, V94, P638, DOI 10.2471/BLT.16.020916
Hutchings MI, 2019, CURR OPIN MICROBIOL, V51, P72, DOI 10.1016/j.mib.2019.10.008

Jablonska J, 2022, ADV APPL MICROBIOL, V121, P27, DOI 10.1016/bs.aambs.2022.08.002
Jault P, 2019, LANCET INFECT DIS, V19, P35, DOI 10.1016/S1473-3099(18)30482-1
Jennes S, 2017, CRIT CARE, V21, DOI 10.1186/s13054-017-1709-y
JERNE NK, 1952, NATURE, V169, P117, DOI 10.1038/169117b0
Jonczyk-Matysiak E, 2015, J IMMUNOL RES, V2015, DOI 10.1155/2015/482863
Jung LS, 2017, ANN CLIN MICROB ANTI, V16, DOI 10.1186/s12941-017-0237-6
KANTOCH M., 1958, SCHWEIZ ZEITSCHR ALLG PATH U BAKT, V21, P1106
Kim JS, 2018, SCIENCE, V359, P993, DOI 10.1126/science.aas9430
Kim M, 2018, APPL ENVIRON MICROB, V84, DOI 10.1128/AEM.02085-18
Koo J, 2000, J FOOD PROTECT, V63, P1049, DOI 10.4315/0362-028X-63.8.1049
KUCHAREWICZKRUKOWSKA A, 1987, ARCH IMMUNOL THER EX, V35, P553
Kurzepa A, 2009, CLIN EXP MED, V9, P93, DOI 10.1007/s10238-008-0027-8
Kutateladze M, 2015, VIROL SIN, V30, P80, DOI 10.1007/s12250-014-3557-0
Laanto E, 2017, NAT COMMUN, V8, DOI 10.1038/s41467-017-00158-7
Labrie SJ, 2019, SCI REP-UK, V9, DOI 10.1038/s41598-019-49975-4
Leitner L, 2021, LANCET INFECT DIS, V21, P427, DOI 10.1016/S1473-3099(20)30330-3
Levy A, 2015, NATURE, V520, P505, DOI 10.1038/nature14302
Li XH, 2021, INT J BIOL SCI, V17, P3573, DOI 10.7150/ijbs.60551
Lin Y, 2018, INT J PHARMACEUT, V551, P158, DOI 10.1016/j.ijpharm.2018.09.024
Liu CG, 2020, MBIO, V11, DOI 10.1128/mBio.01462-20
Lusiak-Szelachowska M, 2017, GUT PATHOG, V9, DOI 10.1186/s13099-017-0196-7
Lusiak-Szelachowska M, 2014, VIRAL IMMUNOL, V27, P295, DOI 10.1089/vim.2013.0128
Ma YS, 2012, FOOD HYDROCOLLOID, V26, P434, DOI 10.1016/j.foodhyd.2010.11.017
Majewska J, 2015, VIRUSES-BASEL, V7, P4783, DOI 10.3390/v7082845
Marek S, 2022, BRATISL MED J, V123, P724, DOI 10.4149/BLL_2022_116
McCallin S, 2013, VIROLOGY, V443, P187, DOI 10.1016/j.virol.2013.05.022
Merabishvili M, 2009, PLOS ONE, V4, DOI 10.1371/journal.pone.0004944
Merril CR, 1996, P NATL ACAD SCI USA, V93, P3188, DOI 10.1073/pnas.93.8.3188
Miedzybrodzki R, 2017, FRONT MICROBIOL, V8, DOI 10.3389/fmicb.2017.00467
Miedzybrodzki R, 2009, CLIN EXP MED, V9, P303, DOI 10.1007/s10238-009-0044-2
Miernikiewicz P, 2016, FRONT MICROBIOL, V7, DOI 10.3389/fmicb.2016.01112
NELSTROP AE, 1968, IMMUNOLOGY, V14, P339
Nguyen S, 2017, MBIO, V8, DOI 10.1128/mBio.01874-17
OCHS HD, 1971, J CLIN INVEST, V50, P2559, DOI 10.1172/JCI106756
Oechslin F, 2017, J INFECT DIS, V215, P703, DOI 10.1093/infdis/jiw632
Ooi ML, 2019, JAMA OTOLARYNGOL, V145, P723, DOI 10.1001/jamaoto.2019.1191

Organization WH, 2014, ANT RES GLOB REP SUR, DOI [10.1016/j.giec.2020.06.004, DOI 10.1007/S13312-014-0374-3]
Pabary R, 2016, ANTIMICROB AGENTS CH, V60, P744, DOI 10.1128/AAC.01426-15
Payne RJH, 2000, CLIN PHARMACOL THER, V68, P225, DOI 10.1067/mcp.2000.109520
Pelfrene E, 2016, J ANTIMICROB CHEMOTH, V71, P2071, DOI 10.1093/jac/dkw083
Peters DL, 2022, CURR PROTOC, V2, DOI 10.1002/cpz1.594
Fabijan AP, 2020, NAT MICROBIOL, V5, P465, DOI 10.1038/s41564-019-0634-z
Pirnay JP, 2015, PHARM RES-DORDR, V32, P2173, DOI 10.1007/s11095-014-1617-7
Pouillot F, 2012, ANTIMICROB AGENTS CH, V56, P3568, DOI 10.1128/AAC.06330-11
Rostol JT, 2019, CELL HOST MICROBE, V25, P184, DOI 10.1016/j.chom.2019.01.009
Roychoudhury P, 2014, P ROY SOC B-BIOL SCI, V281, DOI 10.1098/rspb.2013.2563
Rubalskii E, 2019, SCI REP-UK, V9, DOI 10.1038/s41598-018-38318-4
Rudolf MP, 1998, J IMMUNOL, V160, P3315
Ryan EM, 2012, FEMS IMMUNOL MED MIC, V65, P395, DOI 10.1111/j.1574-695X.2012.00977.x
Sarhan WA, 2015, EXPERT REV ANTI-INFE, V13, P91, DOI 10.1586/14787210.2015.990383
Sarker SA, 2016, EBIOMEDICINE, V4, P124, DOI 10.1016/j.ebiom.2015.12.023
Sarker SA, 2012, VIROLOGY, V434, P222, DOI 10.1016/j.virol.2012.09.002
Scanlan PD, 2011, MOL ECOL, V20, P981, DOI 10.1111/j.1365-294X.2010.04903.x
Scanlan PD, 2012, ISME J, V6, P1148, DOI 10.1038/ismej.2011.174
Schneider G, 2018, BIOMED RES INT, V2018, DOI 10.1155/2018/7569645
Secor PR, 2017, INFECT IMMUN, V85, DOI 10.1128/IAI.00648-16
Smith J, 1924, BRIT MED J, V1924, P47, DOI 10.1136/bmj.2.3315.47
Sokoloff AV, 2000, MOL THER, V2, P131, DOI 10.1006/mthe.2000.0110
Sun YH, 2017, ACTA BIOMATER, V58, P527, DOI 10.1016/j.actbio.2017.06.019
Tacconelli E, 2018, LANCET INFECT DIS, V18, P318, DOI [10.1016/s1473-3099(17)30753-3, 10.1016/S1473-3099(17)30753-3]
Tkhilaishvili T, 2018, INT J ANTIMICROB AG, V52, P842, DOI 10.1016/j.ijantimicag.2018.09.006
Twort FW, 1915, LANCET, V2, P1241, DOI 10.1016/s0140-6736(01)20383-3
Van Belleghem JD, 2017, SCI REP-UK, V7, DOI 10.1038/s41598-017-08336-9
Vashisth M, 2022, CAN J MICROBIOL, V68, P731, DOI 10.1139/cjm-2022-0080
Verbeken G, 2014, ARCH IMMUNOL THER EX, V62, P117, DOI 10.1007/s00005-014-0269-y
Xiao YC, 2020, ADV EXP MED BIOL, V1207, P413, DOI 10.1007/978-981-15-4272-5_29
Yang X, 2021, FRONT MICROBIOL, V12, DOI 10.3389/fmicb.2021.682255
Zaczek M, 2016, FRONT MICROBIOL, V7, DOI 10.3389/fmicb.2016.01681
Zhang LL, 2018, FRONT MICROBIOL, V9, DOI 10.3389/fmicb.2018.01614
Zhang T, 2022, NATURE, DOI 10.1038/s41586-022-05444-z
Zhang YF, 2018, APPL ENVIRON MICROB, V84, DOI 10.1128/AEM.00886-18

   Zimecki M, 2010, MED MICROBIOL IMMUN, V199, P71, DOI 10.1007/s00430-009-0135-4
NR 122
TC 4
Z9 4
U1 11
U2 15
PU CELL PRESS
PI CAMBRIDGE
PA 50 HAMPSHIRE ST, FLOOR 5, CAMBRIDGE, MA 02139 USA
EI 2405-8440
J9 HELIYON
JI Heliyon
PD JUL 30
PY 2024
VL 10
IS 14
AR e34849
DI 10.1016/j.heliyon.2024.e34849
EA JUL 2024
PG 13
WC Multidisciplinary Sciences
WE Science Citation Index Expanded (SCI-EXPANDED)
SC Science & Technology - Other Topics
GA ZO5R0
UT WOS:001276257000001
PM 39148970
OA gold
DA 2025-05-07
ER

PT J
AU Uysal, H
   Bilge, U
   Ilhanli, N
   Gromicho, M
   Grosskreutz, J
   Kuzma-Kozakiewicz, M
   Pinto, S
   Petri, S
   Szacka, K
   Nieporecki, K
   De Carvalho, M
AF Uysal, Hilmi
   Bilge, Ugur
   Ilhanli, Nevruz
   Gromicho, Marta
   Grosskreutz, Julian
   Kuzma-Kozakiewicz, Magdalena
   Pinto, Susana
   Petri, Susanne
   Szacka, Katarzyna
   Nieporecki, Krzysztof
   De Carvalho, Mamede

TI ALS and fertility: does ALS affect number of children patients have?
SO AMYOTROPHIC LATERAL SCLEROSIS AND FRONTOTEMPORAL DEGENERATION
LA English
DT Article
DE Amyotrophic lateral sclerosis; number of offspring; fertility; selection
pressure
ID AMYOTROPHIC-LATERAL-SCLEROSIS; DISEASE
AB Objective Amyotrophic Lateral Sclerosis is one major disease in the group of neurodegenerative conditions. As with most other neurodegenerative diseases, clinical signs of the disease usually show among the elderly population, and most commonly around 60-65 years of age. Therefore the disease is not expected to impact the fertility of ALS patients. When examined from an evolutionary medicine and evolutionary biology perspective, there should be no selection pressure on the patient population due to the late onset of ALS.Methods: In this study, we tested the hypothesis that ALS does not affect fertility on a group of patients with ALS that we collected in a multi-center study. We recruited 511 patients diagnosed with ALS according to the revised El Escorial criteria, and 236 control cases without a neurodegenerative disease. We compared the ALS group's number of offspring with the control group in three consecutive generations.Results: No statistically significant difference was found between the number of siblings of ALS and control groups ($p = 0.44$). A statistically significant difference was found between the number of children of ALS and control groups ($p < 0.001$), indicating ALS patients had more children than controls. When the number of children is assessed by gender, for women, there was no statistically significant difference between the number of children of ALS and control groups ($p = 0.067$).Conclusions: This finding supports the view that ALS does not have a negative selection pressure on the patient population's fertility.
C1 [Uysal, Hilmi] Akdeniz Univ, Dept Neurol & Clin Neurophysiol, Fac Med, Antalya, Turkey.
[Bilge, Ugur; Ilhanli, Nevruz] Akdeniz Univ, Dept Biostat & Med Informat, Fac Med, Antalya, Turkey.
[Gromicho, Marta; Pinto, Susana; De Carvalho, Mamede] Univ Lisbon, Fac Med, Inst Physiol, Inst Med Mol, Lisbon, Portugal.
[Grosskreutz, Julian] Jena Univ Hosp, Hans Berger Dept Neurol, Jena, Germany.
[Kuzma-Kozakiewicz, Magdalena] Med Univ Warsaw, Dept Neurol, Warsaw, Poland.
[Kuzma-Kozakiewicz, Magdalena] Med Univ Warsaw, Neurodegenerat Dis Res Grp, Warsaw, Poland.
[Kuzma-Kozakiewicz, Magdalena; Szacka, Katarzyna; Nieporecki, Krzysztof] Med Univ Warsaw, Univ Clin Ctr, Dept Neurol, Warsaw, Poland.
[Petri, Susanne] Hannover Med Sch, Dept Neurol, Hannover, Germany.
[De Carvalho, Mamede] Ctr Hosp Univ Lisboa Norte, Dept Neurosci & Mental Hlth, Lisbon, Portugal.
C3 Akdeniz University; Akdeniz University; Universidade de Lisboa; Friedrich Schiller University of Jena; Medical University of Warsaw; Medical University of Warsaw; Medical University of Warsaw; Hannover Medical School
RP Uysal, H (autor correspondente), Akdeniz Univ, Dept Neurol, Fac Med, Dumlupinar Bulvari, TR-07070 Antalya, Turkey.

EM uysalh@akdeniz.edu.tr
RI Nieporęcki, Krzysztof/AAB-2597-2021; Bilge, Ugur/C-1044-2016; Pinto, Susana/L-8394-2019; İlhanlı, Nevruz/I-7106-2017; Szacka, Katarzyna/N-1906-2018; Petri, Susanne/AID-7665-2022; Gromicho, Marta/B-2171-2018; Grosskreutz, Julian/G-2293-2010; Uysal, Hilmi/C-3651-2016
OI Gromicho, Marta/0000-0003-2111-4579; Ilhanli, Nevruz/0000-0002-4777-4025; Bilge, Ugur/0000-0002-5186-1092; Grosskreutz, Julian/0000-0001-9525-1424; Kuzma-Kozakiewicz, Magdalena/0000-0002-9676-1263; Uysal, Hilmi/0000-0002-6063-377X
FU OnWebDuals project; JPND: Bundesministerium fur Bildung und Forschung (BMBF), Germany; Narodowe Centrum Badani Rozwoju [NCBiR], Poland [JNPD 01ED1511B, DZP/2/JPND-III/2015]; Fundacao a Ciencia e a Tecnologia (FCT), Poland; Vetenskapsradet (VR), Sweden
FX This study was supported by OnWebDuals project. This is an EU Joint ProgrammeNeurodegenerative Disease Research (JPND) project. The project is supported through the following funding organization under the aegis of JPND-www.jpnd.eu: Bundesministerium fur Bildung und Forschung (BMBF), Germany; Narodowe Centrum Badani Rozwoju [NCBiR; JNPD 01ED1511B; DZP/2/JPND-III/2015], Poland; Fundacao a Ciencia e a Tecnologia (FCT), Poland; Vetenskapsradet (VR), Sweden.
CR Alavi A, 2014, NEUROBIOL AGING, V35, DOI 10.1016/j.neurobiolaging.2013.07.016
[Anonymous], 2019, Human Development Report 2019: Beyond Income, Beyond Averages, Beyond Today: Inequalities in Human Development in the 21st Century
Arthur KC, 2016, NAT COMMUN, V7, DOI 10.1038/ncomms12408
Benjaminsen E, 2018, AMYOTROPH LAT SCL FR, V19, P522, DOI 10.1080/21678421.2018.1513534
Camu W, 1999, J NEUROL SCI, V165, pS21, DOI 10.1016/S0022-510X(99)00022-2
Carleton GD, 1987, VIRUSES IMMUNITY MEN
Crow JF, 2009, INTRO POPULATION GEN
Dorst J, 2019, J NEUROL, V266, P1516, DOI 10.1007/s00415-019-09290-4
Goutman SA, 2019, J NEUROL NEUROSUR PS, V90, P907, DOI 10.1136/jnnp-2018-319785
Henderson RD, 2019, J NEUROL NEUROSUR PS, V90, P570, DOI 10.1136/jnnp-2017-317245
JOHNSON WG, 1995, AM J MED GENET, V59, P149, DOI 10.1002/ajmg.1320590206
Jun KY, 2019, J NEUROL NEUROSUR PS, V90, P395, DOI 10.1136/jnnp-2018-318974
Karlsson EK, 2014, NAT REV GENET, V15, P379, DOI 10.1038/nrg3734
Konno T, 2013, J NEUROL NEUROSUR PS, V84, P398, DOI 10.1136/jnnp-2012-302272
Leighton DJ, 2019, J NEUROL, V266, P817, DOI 10.1007/s00415-019-09190-7
LI TM, 1988, J NEUROL NEUROSUR PS, V51, P778, DOI 10.1136/jnnp.51.6.778
Longinetti E, 2019, CURR OPIN NEUROL, V32, P771, DOI 10.1097/WCO.0000000000000730

Longinetti E, 2018, AMYOTROPH LAT SCL FR, V19, P528, DOI 10.1080/21678421.2018.1497065
Marin B, 2017, INT J EPIDEMIOL, V46, P57, DOI 10.1093/ije/dyw061
Medawar P.B., 1957, UNIQUENESS INDIVIDUA
MURROS K, 1983, ACTA NEUROL SCAND, V67, P41, DOI 10.1111/j.1600-0404.1983.tb04543.x
Gonçalves FCNI, 2017, Meridiano 47, V18, DOI 10.20889/M47e18021
Null R.C.T.R., 2011, Computing, V1, P12, DOI [DOI 10.1890/0012-9658(2002)0833097:CFHIWS2.0.CO;2, DOI 10.1890/0012-9658(2002)083[3097:CFHIWS]2.0.CO;2]
Palese F, 2019, AMYOTROPH LAT SCL FR, V20, P90, DOI 10.1080/21678421.2018.1511732
Pliner HA, 2014, ACTA NEUROPATHOL, V127, P391, DOI 10.1007/s00401-014-1250-x
Rogers CA, 2014, TRIALS, V15, DOI 10.1186/1745-6215-15-53
Rühli FJ, 2013, BMC MED, V11, DOI 10.1186/1741-7015-11-115
Ryan M, 2019, J NEUROL NEUROSUR PS, V90, P659, DOI 10.1136/jnnp-2018-319838
Turgut N, 2019, AMYOTROPH LAT SCL FR, V20, P100, DOI 10.1080/21678421.2018.1525403
Ying W, 1996, MED HYPOTHESES, V47, P307, DOI 10.1016/S0306-9877(96)90071-7
2018, EUR UROL SUPPL, V17
NR 31
TC 1
Z9 1
U1 0
U2 5
PU TAYLOR & FRANCIS LTD
PI ABINGDON
PA 2-4 PARK SQUARE, MILTON PARK, ABINGDON OR14 4RN, OXON, ENGLAND
SN 2167-8421
EI 2167-9223
J9 AMYOTROPH LAT SCL FR
JI Amyotroph. Lateral Scher. Frontotemp. Degenerat.
PD JAN 2
PY 2021
VL 22
IS 1-2
BP 94
EP 100
DI 10.1080/21678421.2020.1813313
EA SEP 2020
PG 7
WC Clinical Neurology
WE Science Citation Index Expanded (SCI-EXPANDED)
SC Neurosciences & Neurology
GA RL5DF
UT WOS:000567563000001
PM 32897105
OA hybrid
DA 2025-05-07
ER

PT J
AU Pepper, I
   Vinik, A
   Lattanzio, F
   McPheat, W
   Dobrian, A
AF Pepper, Ian
   Vinik, Aaron
   Lattanzio, Frank
   McPheat, William
   Dobrian, Anca
TI Countering the Modern Metabolic Disease Rampage With Ancestral
   Endocannabinoid System Alignment
SO FRONTIERS IN ENDOCRINOLOGY
LA English
DT Article
DE cannabinoids; evolution; obesity; energy balance; type 2 diabetes;
NAFLD
ID HIGH-FAT DIET; CB1 RECEPTOR ANTAGONIST; BROWN ADIPOSE-TISSUE; INDUCED
   INSULIN-RESISTANCE; CANNABINOID RECEPTORS; DIABETES-MELLITUS;
   CIRCULATING ENDOCANNABINOIDS; ENERGY-EXPENDITURE; GLYCEMIC CONTROL;
   LIFE-STYLE
AB When primitive vertebrates evolved from ancestral members of the animal kingdom and acquired complex locomotive and neurological toolsets, a constant supply of energy became necessary for their continued survival. To help fulfill this need, the endocannabinoid (eCB) system transformed drastically with the addition of the cannabinoid-1 receptor (CB1R) to its gene repertoire. This established an eCB/CB1R signaling mechanism responsible for governing the whole organism's energy balance, with its activation triggering a shift toward energy intake and storage in the brain and the peripheral organs (i.e., liver and adipose). Although this function was of primal importance for humans during their pre-historic existence as hunter-gatherers, it became expendable following the successive lifestyle shifts of the Agricultural and Industrial Revolutions. Modernization of the world has further increased food availability and decreased energy expenditure, thus shifting the eCB/CB1R system into a state of hyperactive deregulated signaling that contributes to the 21st century metabolic disease pandemic. Studies from the literature supporting this perspective come from a variety of disciplines, including biochemistry, human medicine, evolutionary/comparative biology, anthropology, and developmental biology. Consideration of both biological and cultural evolution justifies the design of improved pharmacological treatments for obesity and Type 2 diabetes (T2D) that focus on peripheral CB1R antagonism. Blockade of peripheral CB1Rs, which universally promote energy conservation across the vertebrate lineage, represents an evolutionary medicine strategy for clinical management of present-day metabolic disorders.
C1 [Pepper, Ian; Lattanzio, Frank; McPheat, William; Dobrian, Anca] Eastern Virginia Med Sch, Dept Physiol Sci, Norfolk, VA 23501 USA.
   [Vinik, Aaron] Eastern Virginia Med Sch, Strelitz Diabet Ctr, Norfolk, VA 23501 USA.
C3 Eastern Virginia Medical School; Eastern Virginia Medical School

RP Pepper, I (autor correspondente), Eastern Virginia Med Sch, Dept Physiol Sci, Norfolk, VA 23501 USA.
EM pepperi@evms.edu
CR Abdulnour J, 2014, OBESITY, V22, P211, DOI 10.1002/oby.20498
Adams LA, 2006, POSTGRAD MED J, V82, P315, DOI 10.1136/pgmj.2005.042200
AIELLO LC, 1995, CURR ANTHROPOL, V36, P199, DOI 10.1086/204350
Alger BE, 2012, J PHYSIOL-LONDON, V590, P2203, DOI 10.1113/jphysiol.2011.220855
Almeida MM, 2017, BRIT J NUTR, V118, P788, DOI 10.1017/S0007114517002884
Anderson JW, 2003, J AM COLL NUTR, V22, P331, DOI 10.1080/07315724.2003.10719316
Argueta DA, 2017, PHYSIOL BEHAV, V171, P32, DOI 10.1016/j.physbeh.2016.12.044
Aronson D, 2002, CARDIOVASC DIABETOL, V1, DOI 10.1186/1475-2840-1-1
Azeez OI, 2014, LIPIDS HEALTH DIS, V13, DOI 10.1186/1476-511X-13-71
Baglioni S, 2012, PLOS ONE, V7, DOI 10.1371/journal.pone.0036569
Banni S, 2011, NUTR METAB, V8, DOI 10.1186/1743-7075-8-7
Barnard RJ, 1998, J APPL PHYSIOL, V84, P1311, DOI 10.1152/jappl.1998.84.4.1311
Bazwinsky-Wutschke I, 2017, INT J MOL SCI, V18, DOI 10.3390/ijms18091844
Beatrice M, 2006, FEBS LETT, V580, P1941, DOI 10.1016/j.febslet.2006.02.057
Bennetzen MF, 2011, INT J OBESITY, V35, P1377, DOI 10.1038/ijo.2011.8
Berge K, 2013, LIPIDS HEALTH DIS, V12, DOI 10.1186/1476-511X-12-78
Bermúdez-Silva FJ, 2009, BEST PRACT RES CL EN, V23, P87, DOI 10.1016/j.beem.2008.10.012
Berry EM, 2002, PHARMACOL THERAPEUT, V95, P185, DOI 10.1016/S0163-7258(02)00257-7
Bifulco M, 2007, MOL PHARMACOL, V71, P1445, DOI 10.1124/mol.106.033118
Blevins JE, 2009, AM J PHYSIOL-REG I, V296, pR476, DOI 10.1152/ajpregu.90544.2008
Bordicchia M, 2010, METABOLISM, V59, P734, DOI 10.1016/j.metabol.2009.09.018
Bowles NP, 2015, P NATL ACAD SCI USA, V112, P285, DOI 10.1073/pnas.1421420112
Brand-Miller JC, 2012, J OBES, V2012, DOI 10.1155/2012/258624
Breunig E, 2010, J NEUROSCI, V30, P8965, DOI 10.1523/JNEUROSCI.4030-09.2010
Brocato B, 2013, PLACENTA, V34, P983, DOI 10.1016/j.placenta.2013.08.007
Broccatelli F, 2012, MOL PHARMACEUT, V9, P570, DOI 10.1021/mp2004302
Chorvat RJ, 2012, BIOORG MED CHEM LETT, V22, P6173, DOI 10.1016/j.bmcl.2012.08.004
Christopoulou FD, 2011, J CLIN PHARM THER, V36, P10, DOI 10.1111/j.1365-2710.2010.01164.x
Comelli F, 2010, EUR J PHARMACOL, V637, P62, DOI 10.1016/j.ejphar.2010.03.061
Cottone E, 2009, NEUROREPORT, V20, P595, DOI 10.1097/WNR.0b013e32832a0a5f
Dardeno TA, 2010, FRONT NEUROENDOCRIN, V31, P377, DOI 10.1016/j.yfrne.2010.06.002

Martins CJD, 2015, PLOS ONE, V10, DOI 10.1371/journal.pone.0142728
De Petrocellis L, 1999, NEUROSCIENCE, V92, P377, DOI 10.1016/S0306-4522(98)00749-0
Del Raye G, 2013, P ROY SOC B-BIOL SCI, V280, DOI 10.1098/rspb.2013.0836
Demas GE, 2011, FUNCT ECOL, V25, P29, DOI 10.1111/j.1365-2435.2010.01738.x
DEVANE WA, 1992, SCIENCE, V258, P1946, DOI 10.1126/science.1470919
Di Marzo V, 2009, DIABETOLOGIA, V52, P213, DOI 10.1007/s00125-008-1178-6
Di Marzo V, 2001, NATURE, V410, P822, DOI 10.1038/35071088
Di Marzo V, 2012, PHILOS T R SOC B, V367, P3216, DOI 10.1098/rstb.2011.0382
Di Marzo V, 2011, HANDB EXP PHARMACOL, V203, P75, DOI 10.1007/978-3-642-17214-4_4
Dias-Rocha CP, 2018, J NUTR BIOCHEM, V51, P56, DOI 10.1016/j.jnutbio.2017.09.019
DiPatrizio NV, 2015, J CLIN INVEST, V125, P891, DOI 10.1172/JCI76302
DiPatrizio NV, 2012, TRENDS NEUROSCI, V35, P403, DOI 10.1016/j.tins.2012.04.006
Dodd GT, 2015, CELL, V160, P88, DOI 10.1016/j.cell.2014.12.022
Donahue SW, 2006, J EXP BIOL, V209, P1630, DOI 10.1242/jeb.02185
Eckardt K, 2009, DIABETOLOGIA, V52, P664, DOI 10.1007/s00125-008-1240-4
Elphick MR, 2012, PHILOS T R SOC B, V367, P3201, DOI 10.1098/rstb.2011.0394
Elphick MR, 2003, GENE, V302, P95, DOI 10.1016/S0378-1119(02)01094-6
Fedonidis C, 2014, NUTR DIABETES, V4, DOI 10.1038/nutd.2014.24
Fride E., 2002, Journal of Cannabis Therapeutics, V2, P51, DOI 10.1300/J175v02n03_04
Gamelin FX, 2016, J PHYSIOL BIOCHEM, V72, P183, DOI 10.1007/s13105-016-0469-5
Gandhi K, 2018, AM J PHYSIOL-ENDOC M, V314, pE322, DOI 10.1152/ajpendo.00119.2017
GAONI Y, 1964, J AM CHEM SOC, V86, P1646, DOI 10.1021/ja01062a046
Gelfand EV, 2006, J AM COLL CARDIOL, V47, P1919, DOI 10.1016/j.jacc.2005.12.067
Gertsch J, 2017, BRIT J PHARMACOL, V174, P1464, DOI 10.1111/bph.13676
Gesta S, 2007, CELL, V131, P242, DOI 10.1016/j.cell.2007.10.004
González-Mariscal I, 2016, SCI REP-UK, V6, DOI 10.1038/srep33302
Graja A, 2015, GERONTOLOGY, V61, P211, DOI 10.1159/000366557
Grant WF, 2011, PLOS ONE, V6, DOI 10.1371/journal.pone.0017261
Gruden G, 2016, BRIT J PHARMACOL, V173, P1116, DOI 10.1111/bph.13226
American Heart Association, 2005, Cardiol Rev, V13, P322
HAFFNER SM, 1992, DIABETES, V41, P715, DOI 10.2337/diabetes.41.6.715
Hall LML, 2010, PLOS ONE, V5, DOI 10.1371/journal.pone.0014197
Hanlon EC, 2015, J CLIN ENDOCR METAB, V100, P220, DOI 10.1210/jc.2014-3455
Hanlon EC, 2016, SLEEP, V39, P653, DOI 10.5665/sleep.5546
Hedges SB, 2015, MOL BIOL EVOL, V32, P835, DOI 10.1093/molbev/msv037
Heerwagen MJR, 2013, PLOS ONE, V8, DOI 10.1371/journal.pone.0067791
Heinitz S, 2018, J CLIN ENDOCR METAB, V103, P1077, DOI 10.1210/jc.2017-02257

Henneberg M, 1998, Human Evolution, V13, P229, DOI DOI 10.1007/BF02436507
Hill JW, 2017, NEUROENDOCRINOLOGY, V104, P330, DOI 10.1159/000450649
Hill MN, 2011, J NEUROSCI, V31, P10506, DOI 10.1523/JNEUROSCI.0496-11.2011
Hillard CJ, 2018, NEUROPSYCHOPHARMACOL, V43, P155, DOI 10.1038/npp.2017.130
Hollander P., 2007, Diabetes Spectrum, V20, P159, DOI 10.2337/diaspect.20.3.159
Hollander PA, 2010, DIABETES CARE, V33, P605, DOI 10.2337/dc09-0455
Howlett AC, 2010, CURR MED CHEM, V17, P1382, DOI 10.2174/092986710790980023
Hryhorczuk C, 2013, FRONT NEUROSCI-SWITZ, V7, DOI 10.3389/fnins.2013.00177
Hua T, 2016, CELL, V167, P750, DOI 10.1016/j.cell.2016.10.004
HUMPHREYS WF, 1979, J ANIM ECOL, V48, P427, DOI 10.2307/4171
Ibrahim MM, 2010, OBES REV, V11, P11, DOI 10.1111/j.1467-789X.2009.00623.x
Jones BJ, 2015, DRUGS, V75, P935, DOI 10.1007/s40265-015-0410-1
Jumpertz R, 2011, J CLIN ENDOCR METAB, V96, P787, DOI 10.1210/jc.2010-2028
Kabir M, 2015, AM J PHYSIOL-ENDOC M, V309, pE747, DOI 10.1152/ajpendo.00196.2015
Kantae V, 2017, SCI REP-UK, V7, DOI 10.1038/s41598-017-07980-5
Kasuga M, 2006, J CLIN INVEST, V116, P1756, DOI 10.1172/JCI29189
Kaufman F., 2005, Diabesity: The obesity-diabetes epidemic that threatens Americaand what we must do to stop it
Kim SP, 2012, AM J PHYSIOL-ENDOC M, V302, pE1261, DOI 10.1152/ajpendo.00496.2011
Kimberly WT, 2017, JCI INSIGHT, V2, DOI 10.1172/jci.insight.92989
Klumpers LE, 2013, BRIT J CLIN PHARMACO, V76, P846, DOI 10.1111/bcp.12141
Kowaleski M, 2014, SPECULUM, V89, P573, DOI 10.1017/S0038713414000815
Krashes MJ, 2016, NAT NEUROSCI, V19, P206, DOI 10.1038/nn.4202
Kronfeld-Schor N, 2000, AM J PHYSIOL-REG I, V279, pR1277, DOI 10.1152/ajpregu.2000.279.4.R1277
Krug RG, 2018, PLOS ONE, V13, DOI 10.1371/journal.pone.0190897
Laprairie RB, 2012, BRIT J PHARMACOL, V167, P1583, DOI 10.1111/j.1476-5381.2012.02175.x
Liu J, 2012, GASTROENTEROLOGY, V142, P1218, DOI 10.1053/j.gastro.2012.01.032
Liu LY, 2016, DEVELOPMENT, V143, P609, DOI 10.1242/dev.121731
Liu WJ, 2010, EUR J PHARMACOL, V637, P70, DOI 10.1016/j.ejphar.2010.04.004
Liu Y, 2018, INT J BIOCHEM CELL B, V99, P114, DOI 10.1016/j.biocel.2018.04.003
Ma HM, 2018, FRONT PHARMACOL, V9, DOI 10.3389/fphar.2018.00156
Malagoli D, 2010, COMP BIOCHEM PHYS A, V155, P134, DOI 10.1016/j.cbpa.2009.10.031
Malenczyk K, 2015, P NATL ACAD SCI USA, V112, pE6185, DOI 10.1073/pnas.1519040112
Martella A, 2016, ENDOCRINOLOGY, V157, P1751, DOI 10.1210/en.2015-1384
Martin RD, 1996, NEWS PHYSIOL SCI, V11, P149, DOI 10.1152/physiologyonline.1996.11.4.149

Matheson T, 2002, ELS, DOI [10.1038/npg.els.0003637, DOI 10.1038/NPG.ELS.0003637]
Matias I, 2005, J NEUROCHEM, V93, P1141, DOI 10.1111/j.1471-4159.2005.03103.x
Matias I, 2007, TRENDS ENDOCRIN MET, V18, P27, DOI 10.1016/j.tem.2006.11.006
Matias I, 2012, PLOS ONE, V7, DOI 10.1371/journal.pone.0042399
Mazier W, 2015, TRENDS ENDOCRIN MET, V26, P524, DOI 10.1016/j.tem.2015.07.007
McPartland J, 2001, J COMP NEUROL, V436, P423, DOI 10.1002/cne.1078
McPartland J., 2002, Journal of Cannabis Therapeutics, V2, P73, DOI DOI 10.1300/J175V02N01_
McPartland JM, 2006, J EVOLUTION BIOL, V19, P366, DOI 10.1111/j.1420-9101.2005.01028.x
McPartland JM, 2003, GENE, V312, P297, DOI 10.1016/S0378-1119(03)00638-3
McPartland JM, 2014, PLOS ONE, V9, DOI 10.1371/journal.pone.0089566
McPartland JM, 2009, PLOS ONE, V4, DOI 10.1371/journal.pone.0005092
MECHOULAM R, 1995, BIOCHEM PHARMACOL, V50, P83, DOI 10.1016/0006-2952(95)00109-D
Mehrpouya-Bahrami P, 2017, SCI REP-UK, V7, DOI 10.1038/s41598-017-15154-6
Mela V, 2016, J PHYSIOL BIOCHEM, V73, P349, DOI 10.1007/s13105-017-0558-0
Micale V, 2019, FRONT PHARMACOL, V10, DOI 10.3389/fphar.2019.00207
Muccioli GG, 2010, MOL SYST BIOL, V6, DOI 10.1038/msb.2010.46
Murillo-Rodniguez E, 2006, LIFE SCI, V79, P30, DOI 10.1016/j.lfs.2005.12.028
NEEL JV, 1962, AM J HUM GENET, V14, P353
Nomura T, 2014, NEUROSCI RES, V86, P25, DOI 10.1016/j.neures.2014.03.004
OBRIEN WJ, 1990, AM SCI, V78, P152
Ogurtsova K, 2017, DIABETES RES CLIN PR, V128, P40, DOI 10.1016/j.diabres.2017.03.024
Oh CM, 2016, DIABETES METAB J, V40, P89, DOI 10.4093/dmj.2016.40.2.89
Osei-Hyiaman D, 2005, J CLIN INVEST, V115, P1298, DOI 10.1172/JCI200523057
Pai WY, 2013, TRANSGENIC RES, V22, P823, DOI 10.1007/s11248-012-9685-0
Penner EA, 2013, AM J MED, V126, P583, DOI 10.1016/j.amjmed.2013.03.002
PEROUTKA SJ, 1994, NEUROPHARMACOLOGY, V33, P319, DOI 10.1016/0028-3908(94)90060-4
Perwitz N, 2010, DIABETES OBES METAB, V12, P158, DOI 10.1111/j.1463-1326.2009.01133.x
Quarta C, 2010, CELL METAB, V11, P273, DOI 10.1016/j.cmet.2010.02.015
Raichlen DA, 2012, J EXP BIOL, V215, P1331, DOI 10.1242/jeb.063677
REAVEN GM, 1988, DIABETES, V37, P1595, DOI 10.2337/diabetes.37.12.1595
Rhea EM, 2017, AAPS J, V19, P921, DOI 10.1208/s12248-017-0079-3
Richter D, 2017, NATURE, V546, P293, DOI 10.1038/nature22335
Rosenstock J, 2008, DIABETES CARE, V31, P2169, DOI 10.2337/dc08-0386
Russo E, 2005, MIL DRUG TH, P1
Ryberg E, 2005, FEBS LETT, V579, P259, DOI 10.1016/j.febslet.2004.11.085
Saito M, 2009, DIABETES, V58, P1526, DOI 10.2337/db09-0530

Salzet M, 2002, PROSTAG LEUKOTR ESS, V66, P353, DOI 10.1054/plef.2001.0347
Sanford AE, 2008, BRAIN RES, V1222, P141, DOI 10.1016/j.brainres.2008.05.048
Sanyal Arun J, 2002, Gastroenterology, V123, P1705, DOI 10.1053/gast.2002.36572
Sarzani R, 2009, METABOLISM, V58, P361, DOI 10.1016/j.metabol.2008.10.009
Seltzman HH, 2017, METHOD ENZYMOL, V593, P199, DOI 10.1016/bs.mie.2017.06.025
Silvestri C, 2015, J HEPATOL, V62, P1382, DOI 10.1016/j.jhep.2015.01.001
Silvestri C, 2013, CELL METAB, V17, P475, DOI 10.1016/j.cmet.2013.03.001
Simiand J, 1998, BEHAV PHARMACOL, V9, P179
Simopoulos AP, 2016, NUTRIENTS, V8, DOI 10.3390/nu8030128
Sohn JW, 2015, BMB REP, V48, P229, DOI 10.5483/BMBRep.2015.48.4.272
Song CK, 2008, AM J PHYSIOL-REG I, V295, pR417, DOI 10.1152/ajpregu.00174.2008
Sulaiman N, 2017, DIABETOL METAB SYNDR, V9, DOI 10.1186/s13098-017-0287-0
Tabesh M, 2018, J DIABETES, V10, P744, DOI 10.1111/1753-0407.12659
Tam J, 2012, CELL METAB, V16, P167, DOI 10.1016/j.cmet.2012.07.002
Ramírez-López MT, 2016, PLOS ONE, V11, DOI 10.1371/journal.pone.0165432
Topol EJ, 2010, LANCET, V376, P517, DOI 10.1016/S0140-6736(10)60935-X
Trillou CR, 2003, AM J PHYSIOL-REG I, V284, pR345, DOI 10.1152/ajpregu.00545.2002
Tuomilehto J, 2001, NEW ENGL J MED, V344, P1343, DOI 10.1056/NEJM200105033441801
Ulrich-Lai YM, 2014, CELL METAB, V19, P910, DOI 10.1016/j.cmet.2014.01.020
Valenta I, 2018, JACC-CARDIOVASC IMAG, V11, P320, DOI 10.1016/j.jcmg.2017.11.019
van Diepen H, 2008, N-S ARCH PHARMACOL, V378, P345, DOI 10.1007/s00210-008-0327-2
van Eyk HJ, 2018, NUTR DIABETES, V8, DOI 10.1038/s41387-017-0016-7
Varga ZV, 2015, BBA-MOL BASIS DIS, V1852, P232, DOI 10.1016/j.bbadis.2014.06.030
Vaughn LK, 2010, BRIT J PHARMACOL, V160, P530, DOI 10.1111/j.1476-5381.2010.00790.x
Vera G, 2012, PHARMACOL BIOCHEM BE, V102, P335, DOI 10.1016/j.pbb.2012.05.008
Worley NB, 2018, PROG NEURO-PSYCHOPH, V85, P180, DOI 10.1016/j.pnpbp.2017.04.004
Yan ZC, 2007, BIOCHEM BIOPH RES CO, V354, P427, DOI 10.1016/j.bbrc.2006.12.213
You TJ, 2011, LIPIDS HEALTH DIS, V10, DOI 10.1186/1476-511X-10-194
Zhang F, 2009, NEUROPHARMACOLOGY, V57, P88, DOI 10.1016/j.neuropharm.2009.04.017
Zimmet Paul Z, 2017, Clin Diabetes Endocrinol, V3, P1, DOI 10.1186/s40842-016-0039-3
2008, J CLIN INVEST, V118, P3160, DOI DOI 10.1172/JCI34827

2005, J PHARMACOL EXP THER, V312, P554, DOI DOI
10.1124/JPET.104.075903
2017, MOL METAB, V6, P1113, DOI DOI 10.1016/J.MOLMET.2017.06.010
2010, TRENDS ENDOCRIN MET, V21, P643, DOI DOI
10.1016/J.TEM.2010.08.002
1991, J NEUROSCI, V11, P563
NR 173
TC 11
Z9 11
U1 0
U2 6
PU FRONTIERS MEDIA SA
PI LAUSANNE
PA AVENUE DU TRIBUNAL FEDERAL 34, LAUSANNE, CH-1015, SWITZERLAND
SN 1664-2392
J9 FRONT ENDOCRINOL
JI Front. Endocrinol.
PD MAY 17
PY 2019
VL 10
AR 311
DI 10.3389/fendo.2019.00311
PG 18
WC Endocrinology & Metabolism
WE Science Citation Index Expanded (SCI-EXPANDED)
SC Endocrinology & Metabolism
GA HY5AQ
UT WOS:000468140100001
PM 31156558
OA Green Published, gold
DA 2025-05-07
ER

PT J
AU Featherston, J
Durand, PM
AF Featherston, Jonathan
Durand, Pierre M.
TI Cooperation and conflict in cancer: An evolutionary perspective
SO SOUTH AFRICAN JOURNAL OF SCIENCE
LA English
DT Review
ID TRANSPOSABLE ELEMENTS; NATURAL-SELECTION; GENOME; CELL; DIFFERENTIATION;
RETROELEMENTS; MECHANISMS; DIVERSITY; PROFILES; SEQUENCE
AB Evolutionary approaches to carcinogenesis have gained prominence in the literature and enhanced our understanding of cancer. However, an appreciation of neoplasia in the context of evolutionary transitions, particularly the transition from independent genes to a fully integrated genome, is largely absent. In the gene-genome evolutionary transition, mobile genetic elements (MGEs) can be studied as the extant exemplars of selfish autonomous lower-level units that cooperated to form a higher-level, functionally integrated genome. Here, we discuss levels of selection in cancer cells. In particular, we examine the tension between

gene and genome units of selection by examining the expression profiles of MGE domains in an array of human cancers. Overall, across diverse cancers, there is an aberrant expression of several families of mobile elements, including the most common MGE in the human genome, retrotransposon LINE 1. These results indicate an alternative life-history strategy for MGEs in the cancers studied. Whether the aberrant expression is the cause or effect of tumourigenesis is unknown, although some evidence suggests that dysregulation of MGEs can play a role in cancer origin and progression. These data are interpreted in combination with phylostratigraphic reports correlating the origin of cancer genes with multicellularity and other potential increases in complexity in cancer cell populations. Cooperation and conflict between individuals at the gene, genome and cell level provide an evolutionary medicine perspective of cancer that enhances our understanding of disease pathogenesis and treatment.
C1 [Featherston, Jonathan; Durand, Pierre M.] Univ Witwatersrand, Dept Mol Med & Haematol, Johannesburg, South Africa.
[Featherston, Jonathan; Durand, Pierre M.] Natl Hlth Lab Serv, Johannesburg, South Africa.
C3 University of Witwatersrand; National Health Laboratory Service
RP Featherston, J (autor correspondente), Univ Witwatersrand, Sch Med, Dept Mol Med & Haematol, 7 York Rd, ZA-2193 Parktown, South Africa.
EM jonathan.featherston@nhls.ac.za
OI Durand, Pierre M./0000-0002-9614-1371
FU Department of Molecular Medicine and Haematology, University of the Witwatersrand; National Research Foundation Bioinformatics and Functional Genomics Programme grant; University of the Witwatersrand Faculty of Health Sciences Individual Research Grant; IBM
FX J.F. is supported by the Department of Molecular Medicine and Haematology, University of the Witwatersrand and a National Research Foundation Bioinformatics and Functional Genomics Programme grant. P.M.D. is supported by the University of the Witwatersrand Faculty of Health Sciences Individual Research Grant and IBM.
CR Belancio VP, 2010, SEMIN CANCER BIOL, V20, P200, DOI 10.1016/j.semcancer.2010.06.001
Boehm T, 1997, NATURE, V390, P404, DOI 10.1038/37126
Brosius J, 1999, GENETICA, V107, P209, DOI 10.1023/A:1004018519722
Burt A., 2006, Genes in Conflict: The Biology of Selfish Genetic Elements
Buss L.W., 1987, The evolution of individuality
Crespi B, 2005, TRENDS ECOL EVOL, V20, P545, DOI 10.1016/j.tree.2005.07.007
Deming SL, 2000, BRIT J CANCER, V83, P1688, DOI 10.1054/bjoc.2000.1522
Domazet-Loso T, 2007, TRENDS GENET, V23, P533, DOI 10.1016/j.tig.2007.08.014
Domazet-Loso T, 2010, BMC BIOL, V8, DOI 10.1186/1741-7007-8-66
Durand PM, 2011, DRUG DISCOVERY AND DEVELOPMENT - PRESENT AND FUTURE, P25
Durand PM, 2010, EVOLUTION, V64, P1533, DOI 10.1111/j.1558-5646.2009.00907.x
Gatenby RA, 2003, CANCER RES, V63, P6212
Goodnight CJ, 1997, AM NAT, V150, pS59, DOI 10.1086/286050
Griesemer J. R., 2000, Selection, V1, P67, DOI [10.1556/Select.1.2000.1-3.7, DOI 10.1556/SELECT.1.2000.1-3.7]

Hauptmann S, 2006, MED HYPOTHESES, V66, P580, DOI 10.1016/j.mehy.2005.08.051
Heng HHO, 2011, ADV CANCER RES, V112, P217, DOI 10.1016/B978-0-12-387688-1.00008-9
Heng HHQ, 2011, GENOMICS, V98, P242, DOI 10.1016/j.ygeno.2011.05.008
Heng HHQ, 2009, BIOESSAYS, V31, P512, DOI 10.1002/bies.200800182
Jones S, 2008, P NATL ACAD SCI USA, V105, P4283, DOI 10.1073/pnas.0712345105
Kidwell MG, 2001, EVOLUTION, V55, P1, DOI 10.1111/j.0014-3820.2001.tb01268.x
Kim DS, 2007, GENES GENET SYST, V82, P145, DOI 10.1266/ggs.82.145
Korkola JE, 2006, CANCER RES, V66, P820, DOI 10.1158/0008-5472.CAN-05-2445
Lamprecht B, 2010, NAT MED, V16, P571, DOI 10.1038/nm.2129
Le Rouzic A, 2005, GENETICS, V169, P1033, DOI 10.1534/genetics.104.031211
Merlo LMF, 2006, NAT REV CANCER, V6, P924, DOI 10.1038/nrc2013
Michod R. E., 1999, DARWINIAN DYNAMICS E
Michod RE, 2005, BIOL PHILOS, V20, P967, DOI 10.1007/s10539-005-9018-2
Michod RE, 2003, INTEGR COMP BIOL, V43, P64, DOI 10.1093/icb/43.1.64
Michod RE, 2007, P NATL ACAD SCI USA, V104, P8613, DOI 10.1073/pnas.0701489104
MIKI Y, 1992, CANCER RES, V52, P643
MORSE B, 1988, NATURE, V333, P87, DOI 10.1038/333087a0
Murchison EP, 2010, SCIENCE, V327, P84, DOI 10.1126/science.1180616
Murgia C, 2006, CELL, V126, P477, DOI 10.1016/j.cell.2006.05.051
Nagy JD, 2004, B MATH BIOL, V66, P663, DOI 10.1016/j.bulm.2003.10.001
Okasha S., 2006, Evolution and the levels of selection, DOI [DOI 10.1093/ACPROF:OSO/9780199267972.001.0001, 10.1093/acprof:oso/9780199267972.001.0001]
Pellman D, 2007, NATURE, V446, P38, DOI 10.1038/446038a
Pepper JW, 2009, EVOL APPL, V2, P62, DOI 10.1111/j.1752-4571.2008.00063.x
Rand David M, 2005, Sci Aging Knowledge Environ, V2005, pre5, DOI 10.1126/sageke.2005.45.re5
Rhodes DR, 2004, NEOPLASIA, V6, P1, DOI 10.1016/S1476-5586(04)80047-2
Romanish MT, 2010, SEMIN CANCER BIOL, V20, P246, DOI 10.1016/j.semcancer.2010.05.005
Siddle HV, 2007, P NATL ACAD SCI USA, V104, P16221, DOI 10.1073/pnas.0704580104
Sinzelle L, 2009, CELL MOL LIFE SCI, V66, P1073, DOI 10.1007/s00018-009-8376-3
Skotheim RI, 2005, CANCER RES, V65, P5588, DOI 10.1158/0008-5472.CAN-05-0153
Stribinskis V, 2006, CANCER RES, V66, P2616, DOI 10.1158/0008-5472.CAN-05-3478
SZATHMARY E, 1995, NATURE, V374, P227, DOI 10.1038/374227a0
Thompson NS, 2000, BEHAV PHILOS, V28, P83
Ting DT, 2011, SCIENCE, V331, P593, DOI 10.1126/science.1200801
Weinberg R.A., 2008, BIOL CANC
Wurmbach E, 2007, HEPATOLOGY, V45, P938, DOI 10.1002/hep.21622
NR 49
TC 0
Z9 1

U1 0
U2 26
PU ACAD SCIENCE SOUTH AFRICA A S S AF
PI LYNWOOD RIDGE
PA PO BOX 72135, LYNWOOD RIDGE 0040, SOUTH AFRICA
SN 0038-2353
EI 1996-7489
J9 S AFR J SCI
JI S. Afr. J. Sci.
PD SEP-OCT
PY 2012
VL 108
IS 9-10
BP 31
EP 37
DI 10.4102/sajs.v108i9/10.1002
PG 7
WC Multidisciplinary Sciences
WE Science Citation Index Expanded (SCI-EXPANDED)
SC Science & Technology - Other Topics
GA 091PM
UT WOS:000315062300008
OA Green Submitted, gold
DA 2025-05-07
ER

PT J
AU Hasan, M
   Ahn, J
AF Hasan, Mahadi
   Ahn, Juhee
TI Evolutionary Dynamics between Phages and Bacteria as a Possible
Approach
   for Designing Effective Phage Therapies against Antibiotic-Resistant
   Bacteria
SO ANTIBIOTICS-BASEL
LA English
DT Review
DE phage resistance; trade-off; superinfection exclusion; restriction
   modification; abortive infection; CRISPR-Cas
ID COEVOLUTIONARY ARMS RACES; ESCHERICHIA-COLI K-12; MEMBRANE-PROTEIN
OMPA;
   EFFLUX PUMPS; PSEUDOMONAS-AERUGINOSA; MULTIDRUG-RESISTANCE;
   CAMPYLOBACTER-JEJUNI; SUPERINFECTION EXCLUSION;
   RESTRICTION-ENDONUCLEASE; ANTIMICROBIAL RESISTANCE
AB With the increasing global threat of antibiotic resistance, there is
an urgent need to develop new effective therapies to tackle antibiotic-
resistant bacterial infections. Bacteriophage therapy is considered as a
possible alternative over antibiotics to treat antibiotic-resistant
bacteria. However, bacteria can evolve resistance towards bacteriophages
through antiphage defense mechanisms, which is a major limitation of
phage therapy. The antiphage mechanisms target the phage life cycle,
including adsorption, the injection of DNA, synthesis, the assembly of
phage particles, and the release of progeny virions. The non-specific

bacterial defense mechanisms include adsorption inhibition, superinfection exclusion, restriction-modification, and abortive infection systems. The antiphage defense mechanism includes a clustered regularly interspaced short palindromic repeats (CRISPR)-CRISPR-associated (Cas) system. At the same time, phages can execute a counterstrategy against antiphage defense mechanisms. However, the antibiotic susceptibility and antibiotic resistance in bacteriophage-resistant bacteria still remain unclear in terms of evolutionary trade-offs and trade-ups between phages and bacteria. Since phage resistance has been a major barrier in phage therapy, the trade-offs can be a possible approach to design effective bacteriophage-mediated intervention strategies. Specifically, the trade-offs between phage resistance and antibiotic resistance can be used as therapeutic models for promoting antibiotic susceptibility and reducing virulence traits, known as bacteriophage steering or evolutionary medicine. Therefore, this review highlights the synergistic application of bacteriophages and antibiotics in association with the pleiotropic trade-offs of bacteriophage resistance.
C1 [Hasan, Mahadi; Ahn, Juhee] Kangwon Natl Univ, Dept Biomed Sci, Chunchon 24341, Gangwon, South Korea.
[Ahn, Juhee] Kangwon Natl Univ, Inst Biosci & Biotechnol, Chunchon 24341, Gangwon, South Korea.
C3 Kangwon National University; Kangwon National University
RP Ahn, J (autor correspondente), Kangwon Natl Univ, Dept Biomed Sci, Chunchon 24341, Gangwon, South Korea.; Ahn, J (autor correspondente), Kangwon Natl Univ, Inst Biosci & Biotechnol, Chunchon 24341, Gangwon, South Korea.
EM 202216366@kangwon.ac.kr; juheeahn@kangwon.ac.kr
RI Ahn, Juhee/G-1959-2014
OI Ahn, Juhee/0000-0001-9341-009X
FU Basic Science Research Program through the National Research Foundation
of Korea (NRF) - Ministry of Education [NRF-2016R1D1A3B01008304]
FX This research was supported by Basic Science Research Program through the National Research Foundation of Korea (NRF) funded by the Ministry of Education (NRF-2016R1D1A3B01008304).
CR ACHTMAN M, 1977, P NATL ACAD SCI USA, V74, P5104, DOI 10.1073/pnas.74.11.5104
Ackermann HW, 1999, ADV VIRUS RES, V51, P135
Aksyuk AA, 2009, EMBO J, V28, P821, DOI 10.1038/emboj.2009.36
Allen RC, 2017, MBIO, V8, DOI [10.1128/mBio.01341-17, 10.1128/mbio.01341-17]
Alós JI, 2015, ENFERM INFEC MICR CL, V33, P692, DOI 10.1016/j.eimc.2014.10.004
Alseth EO, 2019, NATURE, V574, P549, DOI 10.1038/s41586-019-1662-9
Altamirano F.G., 2020, NAT MICROBIOL, V6, P157
Aminov RI, 2010, FRONT MICROBIOL, V1, DOI 10.3389/fmicb.2010.00134
Arguijo-Hernández ES, 2018, ARCH VIROL, V163, P2959, DOI 10.1007/s00705-018-3954-z
Augustyniak D, 2022, VIRUSES-BASEL, V14, DOI 10.3390/v14010121
Bair CL, 2007, J MOL BIOL, V366, P779, DOI 10.1016/j.jmb.2006.11.049
Bair CL, 2007, J MOL BIOL, V366, P768, DOI 10.1016/j.jmb.2006.11.051
Barrangou R, 2007, SCIENCE, V315, P1709, DOI 10.1126/science.1138140

Bayliss CD, 2012, NUCLEIC ACIDS RES, V40, P5876, DOI 10.1093/nar/gks246
Bidnenko E, 2002, FEMS MICROBIOL LETT, V214, P283, DOI 10.1111/j.1574-6968.2002.tb11360.x
Bikard D, 2012, CURR OPIN IMMUNOL, V24, P15, DOI 10.1016/j.coi.2011.10.005
Blower Tim R, 2012, Bacteriophage, V2, P234
Bohannan BJM, 2002, ANTON LEEUW INT J G, V81, P107, DOI 10.1023/A:1020585711378
Bondy-Denomy J, 2013, NATURE, V493, P429, DOI 10.1038/nature11723
BRAUN V, 1994, J BACTERIOL, V176, P4710, DOI 10.1128/JB.176.15.4710-4717.1994
Broeker NK, 2017, MOL MICROBIOL, V105, P353, DOI 10.1111/mmi.13729
Brouns SJJ, 2008, SCIENCE, V321, P960, DOI 10.1126/science.1159689
Brüssow H, 2002, CELL, V108, P13, DOI 10.1016/S0092-8674(01)00637-7
Bull JJ, 2014, PLOS ONE, V9, DOI 10.1371/journal.pone.0094690
Burmeister A.R., 2021, MICROBIOLOGY, V167, DOI [10.1099/mic.0.001063, DOI 10.1099/MIC.0.001063]
Burmeister AR, 2020, CURR BIOL, V30, pR1120, DOI 10.1016/j.cub.2020.07.036
Burmeister AR, 2020, P NATL ACAD SCI USA, V117, P11207, DOI 10.1073/pnas.1919888117
Cai RP, 2019, FRONT MICROBIOL, V10, DOI 10.3389/fmicb.2019.01189
Capparelli R, 2010, PLOS ONE, V5, DOI 10.1371/journal.pone.0011720
Card KJ, 2021, P NATL ACAD SCI USA, V118, DOI 10.1073/pnas.2016886118
Castledine M, 2022, ELIFE, V11, DOI [10.7554/eLife.73679, 10.7554/eLife.73679.sa0, 10.7554/eLife.73679.sa1, 10.7554/eLife.73679.sa2]
Ceyssens PJ, 2009, ENVIRON MICROBIOL, V11, P2874, DOI 10.1111/j.1462-2920.2009.02030.x
Chan BK, 2018, EVOL MED PUBLIC HLTH, P60, DOI 10.1093/emph/eoy005
Chan BK, 2016, SCI REP-UK, V6, DOI 10.1038/srep26717
Chao KL, 2022, SCI REP-UK, V12, DOI 10.1038/s41598-022-06073-2
Chatterjee A, 2019, INFECT IMMUN, V87, DOI 10.1128/IAI.00085-19
Cheng XG, 2004, VIROLOGY, V326, P340, DOI 10.1016/j.virol.2004.06.001
Chung IY, 2014, P NATL ACAD SCI USA, V111, P11503, DOI 10.1073/pnas.1403537111
Chung PY, 2014, PATHOG DIS, V70, P231, DOI 10.1111/2049-632X.12141
Cunrath O, 2019, EBIOMEDICINE, V41, P479, DOI 10.1016/j.ebiom.2019.02.061
Czaplewski L, 2016, LANCET INFECT DIS, V16, P239, DOI 10.1016/S1473-3099(15)00466-1
Destoumieux-Garzón D, 2005, BIOCHEM J, V389, P869, DOI 10.1042/BJ20042107
Domingues S, 2008, VIROLOGY, V373, P14, DOI 10.1016/j.virol.2007.11.004
Dowah Ahmed S A, 2018, Biophys Rev, V10, P535, DOI 10.1007/s12551-017-0382-3
Dy RL, 2014, ANNU REV VIROL, V1, P307, DOI 10.1146/annurev-virology-031413-085500
Egido JE, 2022, FEMS MICROBIOL REV, V46, DOI 10.1093/femsre/fuab048
Fang QQ, 2022, COMMUN BIOL, V5, DOI 10.1038/s42003-022-03001-y
Fauconnier A, 2017, EMBO REP, V18, P198, DOI 10.15252/embr.201643250

Ferguson AD, 2001, STRUCTURE, V9, P707, DOI 10.1016/S0969-2126(01)00631-1
Ferriol-González C, 2020, ANTIBIOTICS-BASEL, V9, DOI 10.3390/antibiotics9050268
Fineran PC, 2009, P NATL ACAD SCI USA, V106, P894, DOI 10.1073/pnas.0808832106
Folimonova SY, 2012, J VIROL, V86, P5554, DOI 10.1128/JVI.00310-12
Fukuyo M, 2012, SCI REP-UK, V2, DOI 10.1038/srep00238
Gaynes R, 2017, EMERG INFECT DIS, V23, P849, DOI 10.3201/eid2305.161556
German GJ, 2001, J MOL BIOL, V308, P579, DOI 10.1006/jmbi.2001.4578
Gleditzsch D, 2019, RNA BIOL, V16, P504, DOI 10.1080/15476286.2018.1504546
Goldfarb T, 2015, EMBO J, V34, P169, DOI 10.15252/embj.201489455
Golkar Z, 2014, J INFECT DEV COUNTR, V8, P129, DOI 10.3855/jidc.3573
Altamirano FG, 2021, NAT MICROBIOL, V6, P157, DOI 10.1038/s41564-020-00830-7
Grant GR, 1997, CHEST, V112, P7, DOI 10.1378/chest.112.1.7
Green ER, 2016, VIRULENCE MECHANISMS OF BACTERIAL PATHOGENS, 5 EDITION, P215, DOI 10.1128/microbiolspec.VMBF-0012-2015
Grissa I, 2008, NUCLEIC ACIDS RES, V36, pW145, DOI 10.1093/nar/gkn228
Gurney J, 2020, EVOL MED PUBLIC HLTH, P148, DOI 10.1093/emph/eoaa026
Hall AR, 2011, ECOL LETT, V14, P635, DOI 10.1111/j.1461-0248.2011.01624.x
Hallet B, 2001, CURR OPIN MICROBIOL, V4, P570, DOI 10.1016/S1369-5274(00)00253-8
Hampton HG, 2020, NATURE, V577, P327, DOI 10.1038/s41586-019-1894-8
Haq IU, 2012, VIROL J, V9, DOI 10.1186/1743-422X-9-9
Hendrix RW, 1999, P NATL ACAD SCI USA, V96, P2192, DOI 10.1073/pnas.96.5.2192
Hermoso JA, 2007, CURR OPIN MICROBIOL, V10, P461, DOI 10.1016/j.mib.2007.08.002
Hernandez-Morales AC, 2018, J VIROL, V92, DOI 10.1128/JVI.01064-17
Hernández-Sánchez J, 2008, ARCH VIROL, V153, P1271, DOI 10.1007/s00705-008-0111-0
Hernando-Amado S, 2022, MOL BIOL EVOL, V39, DOI 10.1093/molbev/msac049
Hernando-Amado S, 2020, SCI ADV, V6, DOI 10.1126/sciadv.aba5493
Hesse S, 2020, MBIO, V11, DOI 10.1128/mBio.02530-19
Hill C, 2018, FUTURE MICROBIOL, V13, P711, DOI 10.2217/fmb-2017-0261
Hoque MM, 2016, SCI REP-UK, V6, DOI 10.1038/srep37956
Howlett R, 2018, MICROBIOL-SGM, V164, P614, DOI 10.1099/mic.0.000636
IIDA S, 1987, VIROLOGY, V157, P156, DOI 10.1016/0042-6822(87)90324-2
Isaev AB, 2021, BIOCHEMISTRY-MOSCOW+, V86, P319, DOI 10.1134/S0006297921030081
Iwasaki T, 2018, GENES CELLS, V23, P528, DOI 10.1111/gtc.12594
Jia N, 2021, NAT REV MOL CELL BIO, V22, P563, DOI 10.1038/s41580-021-00371-9
Jinek M, 2012, SCIENCE, V337, P816, DOI 10.1126/science.1225829
KADNER RJ, 1980, J BACTERIOL, V143, P256, DOI 10.1128/JB.143.1.256-264.1980
Kameyama L, 1999, VIROLOGY, V263, P100, DOI 10.1006/viro.1999.9888
Kang CM, 2008, MICROBIOL-SGM, V154, P725, DOI 10.1099/mic.0.2007/014076-0
Karginov FV, 2010, MOL CELL, V37, P7, DOI 10.1016/j.molcel.2009.12.033

Kasman L.M., 2022, StatPearls Internet
Kesik-Szeloch A, 2013, VIROL J, V10, DOI 10.1186/1743-422X-10-100
Kim M, 2012, MOL MICROBIOL, V86, P411, DOI 10.1111/j.1365-2958.2012.08202.x
KIRNOS MD, 1977, NATURE, V270, P369, DOI 10.1038/270369a0
Ko CC, 2018, MOL MICROBIOL, V108, P443, DOI 10.1111/mmi.13946
Koebnik R, 1999, J BACTERIOL, V181, P3688, DOI 10.1128/JB.181.12.3688-3694.1999
Koga M, 2011, GENETICS, V187, P123, DOI 10.1534/genetics.110.121798
Kortright KE, 2022, APPL ENVIRON MICROB, V88, DOI 10.1128/AEM.01514-21
Kortright KE, 2021, FRONT MICROBIOL, V12, DOI 10.3389/fmicb.2021.658374
Kortright KE, 2020, P NATL ACAD SCI USA, V117, P18670, DOI 10.1073/pnas.2001888117
Kortright KE, 2019, CELL HOST MICROBE, V25, P219, DOI 10.1016/j.chom.2019.01.014
Koskella B, 2014, FEMS MICROBIOL REV, V38, P916, DOI 10.1111/1574-6976.12072
Labrie SJ, 2010, NAT REV MICROBIOL, V8, P317, DOI 10.1038/nrmicro2315
Langenscheid J, 2004, FEMS MICROBIOL LETT, V234, P133, DOI 10.1016/j.femsle.2004.03.019
Lee YJ, 2018, P NATL ACAD SCI USA, V115, pE3116, DOI 10.1073/pnas.1714812115
Leon LM, 2018, CURR OPIN MICROBIOL, V42, P87, DOI 10.1016/j.mib.2017.11.005
León M, 2015, FRONT MICROBIOL, V6, DOI 10.3389/fmicb.2015.00343
LeRoux M, 2022, ANNU REV MICROBIOL, V76, P21, DOI 10.1146/annurev-micro-020722-013730
Leungtongkam U, 2020, SCI REP-UK, V10, DOI 10.1038/s41598-020-73123-y
Levin AS, 1999, CLIN INFECT DIS, V28, P1008, DOI 10.1086/514732
Li XH, 2021, INT J BIOL SCI, V17, P3573, DOI 10.7150/ijbs.60551
Liu YN, 2019, PEERJ, V7, DOI 10.7717/peerj.6173
Loc-Carrillo Catherine, 2011, Bacteriophage, V1, P111
Lopatina A, 2020, ANNU REV VIROL, V7, P371, DOI 10.1146/annurev-virology-011620-040628
Lu Meng-Ji, 1994, Trends in Microbiology, V2, P137, DOI 10.1016/0966-842X(94)90601-7
Luong T, 2020, CLIN THER, V42, P1659, DOI 10.1016/j.clinthera.2020.07.014
Majkowska-Skrobek G, 2021, ENVIRON MICROBIOL, V23, P7723, DOI 10.1111/1462-2920.15476
Makarova KS, 2015, NAT REV MICROBIOL, V13, P722, DOI 10.1038/nrmicro3569
Mangalea MR, 2020, INFECT IMMUN, V88, DOI 10.1128/IAI.00926-19
Markwitz P, 2022, ISME J, V16, P533, DOI 10.1038/s41396-021-01096-5
Masuda N, 2000, ANTIMICROB AGENTS CH, V44, P3322, DOI 10.1128/AAC.44.12.3322-3327.2000
Maynard ND, 2010, PLOS GENET, V6, DOI 10.1371/journal.pgen.1001017
Meeske AJ, 2020, SCIENCE, V369, P54, DOI 10.1126/science.abb6151
Melnyk AH, 2015, EVOL APPL, V8, P273, DOI 10.1111/eva.12196
Meyer JR, 2012, SCIENCE, V335, P428, DOI 10.1126/science.1214449
Mohammed Manal, 2015, BMC Res Notes, V8, P728, DOI 10.1186/s13104-015-1687-6
Mohanraju P, 2016, SCIENCE, V353, DOI 10.1126/science.aad5147

Moldovan R, 2007, BIOPHYS J, V93, P303, DOI 10.1529/biophysj.106.102962
MOLINEUX IJ, 1989, J MOL BIOL, V207, P563, DOI 10.1016/0022-2836(89)90465-8
Morehead MS, 2018, PRIMARY CARE, V45, P467, DOI 10.1016/j.pop.2018.05.006
MORONA R, 1984, J BACTERIOL, V159, P570, DOI 10.1128/JB.159.2.570-578.1984
Moulton-Brown CE, 2018, EVOL APPL, V11, P1630, DOI 10.1111/eva.12653
Murphy J, 2013, APPL ENVIRON MICROB, V79, P7547, DOI 10.1128/AEM.02229-13
Nachega JB, 2003, CLIN INFECT DIS, V36, pS24, DOI 10.1086/344657
NAKAGAWA H, 1985, J VIROL, V54, P460, DOI 10.1128/JVI.54.2.460-466.1985
Nishino K, 2009, BBA-PROTEINS PROTEOM, V1794, P834, DOI 10.1016/j.bbapap.2009.02.002
Nwodo UU, 2012, INT J MOL SCI, V13, P14002, DOI 10.3390/ijms131114002
Oechslin F, 2018, VIRUSES-BASEL, V10, DOI 10.3390/v10070351
Oechslin F, 2017, J INFECT DIS, V215, P703, DOI 10.1093/infdis/jiw632
OFARRELL PH, 1980, MOL GEN GENET, V179, P421, DOI 10.1007/BF00425473
Ofir G, 2018, NAT MICROBIOL, V3, DOI 10.1038/s41564-017-0051-0
Ojala V, 2013, EVOL APPL, V6, P925, DOI 10.1111/eva.12076
Oliveira H, 2014, PLOS ONE, V9, DOI 10.1371/journal.pone.0108376
Otsuka Y, 2005, GENETICS, V169, P13, DOI 10.1534/genetics.104.033290
Page R, 2016, NAT CHEM BIOL, V12, P208, DOI [10.1038/NCHEMBIO.2044, 10.1038/nchembio.2044]
PARMA DH, 1992, GENE DEV, V6, P497, DOI 10.1101/gad.6.3.497
Pawluk A, 2018, NAT REV MICROBIOL, V16, P12, DOI 10.1038/nrmicro.2017.120
Pearson JP, 1999, J BACTERIOL, V181, P1203, DOI 10.1128/JB.181.4.1203-1210.1999
Petrova OE, 2012, J BACTERIOL, V194, P2413, DOI 10.1128/JB.00003-12
Piddock LJV, 2006, NAT REV MICROBIOL, V4, P629, DOI 10.1038/nrmicro1464
Pokharel S, 2019, BMJ GLOB HEALTH, V4, DOI 10.1136/bmjgh-2019-002104
Poole J, 2018, NAT REV MICROBIOL, V16, P440, DOI 10.1038/s41579-018-0007-2
Poole K, 2007, ANN MED, V39, P162, DOI 10.1080/07853890701195262
Qimron U, 2006, P NATL ACAD SCI USA, V103, P19039, DOI 10.1073/pnas.0609428103
Quiberoni A, 2004, J APPL MICROBIOL, V96, P340, DOI 10.1046/j.1365-2672.2003.02147.x
Ram G, 2012, P NATL ACAD SCI USA, V109, P16300, DOI 10.1073/pnas.1204615109
Ravin V, 2002, J BACTERIOL, V184, P2455, DOI 10.1128/JB.184.9.2455-2459.2002
Ricci V, 2010, APPL ENVIRON MICROB, V76, P1704, DOI 10.1128/AEM.02681-09
Rice CJ, 2021, MICROORGANISMS, V9, DOI 10.3390/microorganisms9102172
RIEDE I, 1987, MOL GEN GENET, V206, P110, DOI 10.1007/BF00326544
Rifat D, 2008, J MOL BIOL, V375, P720, DOI 10.1016/j.jmb.2007.10.064
Riou M, 2016, INT J ANTIMICROB AG, V47, P77, DOI 10.1016/j.ijantimicag.2015.11.004

Roberts RJ, 2003, NUCLEIC ACIDS RES, V31, P1805, DOI 10.1093/nar/gkg274
Rossolini GM, 2008, CLIN MICROBIOL INFEC, V14, P2, DOI 10.1111/j.1469-0691.2008.02126.x
Rostol JT, 2019, CELL HOST MICROBE, V25, P184, DOI 10.1016/j.chom.2019.01.009
Safari F, 2020, CRIT REV BIOTECHNOL, V40, P119, DOI 10.1080/07388551.2019.1674774
Salazar KC, 2021, MBIO, V12, DOI 10.1128/mBio.00211-21
Samson JE, 2013, NAT REV MICROBIOL, V11, P675, DOI 10.1038/nrmicro3096
Scanlan PD, 2015, MOL BIOL EVOL, V32, P1425, DOI 10.1093/molbev/msv032
Scanlan PD, 2011, MOL ECOL, V20, P981, DOI 10.1111/j.1365-294X.2010.04903.x
Scanlan PD, 2012, ISME J, V6, P1148, DOI 10.1038/ismej.2011.174
Schmidt AK, 2022, MBIO, V13, DOI 10.1128/mbio.02441-21
SCHMITT CK, 1991, J BACTERIOL, V173, P6507, DOI 10.1128/jb.173.20.6507-6514.1991
SCHMITT CK, 1991, J BACTERIOL, V173, P1536, DOI 10.1128/jb.173.4.1536-1543.1991
Schuster S, 2017, INT J ANTIMICROB AG, V50, P477, DOI 10.1016/j.ijantimicag.2017.03.023
Scott AE, 2007, PLOS PATHOG, V3, P1142, DOI 10.1371/journal.ppat.0030119
Shabbir MAB, 2016, FRONT MICROBIOL, V7, DOI 10.3389/fmicb.2016.01292
Shen Y, 2021, CURR OPIN BIOTECH, V68, P166, DOI 10.1016/j.copbio.2020.11.004
Shi K, 2020, COMMUN BIOL, V3, DOI 10.1038/s42003-020-01412-3
SHINEDLING S, 1987, J VIROL, V61, P3790, DOI 10.1128/JVI.61.12.3790-3794.1987
Shkoporov AN, 2021, BMC BIOL, V19, DOI 10.1186/s12915-021-01084-3
SIDEN I, 1983, J BACTERIOL, V154, P170
Silva JB, 2016, FEMS MICROBIOL LETT, V363, DOI 10.1093/femsle/fnw002
Slavcev RA, 2003, MOL GENET GENOMICS, V269, P40, DOI 10.1007/s00438-002-0787-x
Slavcev RA, 2002, J BACTERIOL, V184, P857, DOI 10.1128/JB.184.3.857-858.2002
SNYDER L, 1989, GENE, V81, P17, DOI 10.1016/0378-1119(89)90332-6
SNYDER L, 1995, MOL MICROBIOL, V15, P415, DOI 10.1111/j.1365-2958.1995.tb02255.x
Song S, 2020, FRONT MICROBIOL, V11, DOI 10.3389/fmicb.2020.01895
Sorensen MCH, 2021, CELL REP, V35, DOI 10.1016/j.celrep.2021.109214
Sorensen MCH, 2011, J BACTERIOL, V193, P6742, DOI 10.1128/JB.05276-11
Stanley SY, 2019, CELL, V178, P1452, DOI 10.1016/j.cell.2019.07.046
Stern A, 2011, BIOESSAYS, V33, P43, DOI 10.1002/bies.201000071
Stewart CR, 2009, J MOL BIOL, V388, P48, DOI 10.1016/j.jmb.2009.03.009
Suárez VB, 2008, INT J FOOD MICROBIOL, V128, P401, DOI 10.1016/j.ijfoodmicro.2008.09.007
Summers William C, 2012, Bacteriophage, V2, P130
SUTHERLAND IW, 1995, FEMS MICROBIOL REV, V16, P323, DOI 10.1016/0168-6445(95)00020-D
Tamer YT, 2021, MOL BIOL EVOL, V38, P4493, DOI 10.1093/molbev/msab190
Tan DM, 2020, MICROORGANISMS, V8, DOI 10.3390/microorganisms8030378
Thanner S, 2016, MBIO, V7, DOI 10.1128/mBio.02227-15

Tock MR, 2005, CURR OPIN MICROBIOL, V8, P466, DOI 10.1016/j.mib.2005.06.003
Uc-Mass A, 2004, VIROLOGY, V329, P425, DOI 10.1016/j.virol.2004.09.005
Uchiyama J, 2011, PLOS ONE, V6, DOI 10.1371/journal.pone.0026648
Uddin MJ, 2020, MICROORGANISMS, V8, DOI 10.3390/microorganisms8050670
Uddin MJ, 2019, ARCH MICROBIOL, V201, P983, DOI 10.1007/s00203-019-01667-3
Unterholzner Simon J, 2013, Mob Genet Elements, V3, pe26219
Valappil SK, 2021, FRONT MICROBIOL, V12, DOI 10.3389/fmicb.2021.783722
Vale PF, 2015, P ROY SOC B-BIOL SCI, V282, P41, DOI 10.1098/rspb.2015.1270
van Houte S, 2016, NATURE, V532, P385, DOI 10.1038/nature17436
Varble A, 2021, NAT MICROBIOL, V6, P1516, DOI 10.1038/s41564-021-00996-8
Vila J, 2007, J ANTIMICROB CHEMOTH, V59, P1210, DOI 10.1093/jac/dkl509
Vincent PA, 2009, CURR MED CHEM, V16, P538, DOI 10.2174/092986709787458461
VOVIS GF, 1974, P NATL ACAD SCI USA, V71, P3810, DOI 10.1073/pnas.71.10.3810
Walter M, 2008, J VIROL, V82, P2265, DOI 10.1128/JVI.01641-07
Wang XQ, 2021, EMERG MICROBES INFEC, V10, P2205, DOI 10.1080/22221751.2021.2002671
Weitz JS, 2005, P NATL ACAD SCI USA, V102, P9535, DOI 10.1073/pnas.0504062102
Westra ER, 2015, CURR BIOL, V25, P1043, DOI 10.1016/j.cub.2015.01.065
Whitney CG, 2000, NEW ENGL J MED, V343, P1917, DOI 10.1056/NEJM200012283432603
Wiedenheft B, 2011, P NATL ACAD SCI USA, V108, P10092, DOI 10.1073/pnas.1102716108
Wong S, 2021, CURR GENET, V67, P739, DOI 10.1007/s00294-021-01183-2
Wright RCT, 2018, PLOS BIOL, V16, DOI 10.1371/journal.pbio.2006057
Wu XR, 2021, MBIO, V12, DOI 10.1128/mBio.01361-21
WURTZ M, 1992, ELECTRON MICROSC REV, V5, P283, DOI 10.1016/0892-0354(92)90013-G
Xia GQ, 2014, INFECT GENET EVOL, V21, P593, DOI 10.1016/j.meegid.2013.04.022
Xu J, 2018, MICROB BIOTECHNOL, V11, P1112, DOI 10.1111/1751-7915.13042
You JJ, 2018, NAT COMMUN, V9, DOI 10.1038/s41467-018-04232-6
Zago M, 2017, FOOD MICROBIOL, V66, P110, DOI 10.1016/j.fm.2017.04.014
Zaleski P, 2005, MICROBIOL-SGM, V151, P3361, DOI 10.1099/mic.0.28184-0
Zgurskaya HI, 1999, J MOL BIOL, V285, P409, DOI 10.1006/jmbi.1998.2313
NR 224
TC 53
Z9 53
U1 9
U2 57
PU MDPI
PI BASEL
PA ST ALBAN-ANLAGE 66, CH-4052 BASEL, SWITZERLAND
SN 2079-6382
J9 ANTIBIOTICS-BASEL
JI Antibiotics-Basel
PD JUL
PY 2022

VL 11
IS 7
AR 915
DI 10.3390/antibiotics11070915
PG 20
WC Infectious Diseases; Pharmacology & Pharmacy
WE Science Citation Index Expanded (SCI-EXPANDED)
SC Infectious Diseases; Pharmacology & Pharmacy
GA 3G5HG
UT WOS:000831384900001
PM 35884169
OA gold, Green Published
DA 2025-05-07
ER

PT J
AU Hollon, SD
   Andrews, PW
   Thomson, JA
AF Hollon, Steven D.
   Andrews, Paul W.
   Thomson, J. Anderson, Jr.
TI Cognitive Behavior Therapy for Depression From an Evolutionary
   Perspective
SO FRONTIERS IN PSYCHIATRY
LA English
DT Article
DE depression; evolution; rumination; cognitive behavior therapy;
   antidepressant medications
ID NATIONAL COMORBIDITY SURVEY; ANTIDEPRESSANT MEDICATION;
   GENDER-DIFFERENCES; RANDOMIZED-TRIAL; SYMPTOM CHANGE; RISK;
PREVENTION;
   ADAPTATION; SEVERITY; MODERATE
AB Evolutionary medicine attempts to solve a problem with which traditional medicine has struggled historically; how do we distinguish between diseased states and "healthy" responses to disease states? Fever and diarrhea represent classic examples of evolved adaptations that increase the likelihood of survival in response to the presence of pathogens in the body. Whereas, the severe mental disorders like psychotic mania or the schizophrenias may involve true "disease" states best treated pharmacologically, most non-psychotic "disorders" that revolve around negative affects like depression or anxiety are likely adaptations that evolved to serve a function that increased inclusive fitness in our ancestral past. What this likely means is that the proximal mechanisms underlying the non-psychotic "disorders" are "species typical" and neither diseases nor disorders. Rather, they are coordinated "whole body" responses that prepare the individual to respond in a maximally functional fashion to the variety of different challenges that our ancestors faced. A case can be made that depression evolved to facilitate a deliberate cognitive style (rumination) in response to complex (often social) problems. What this further suggests is that those interventions that best facilitate the functions that those adaptations evolved to serve (such as rumination) are likely to be preferred over those like medications that simply anesthetize the distress. We consider

the mechanisms that evolved to generate depression and the processes utilized in cognitive behavior therapy to facilitate those functions from an adaptationist evolutionary perspective.
C1 [Hollon, Steven D.] Vanderbilt Univ, Dept Psychol, Nashville, TN 37240 USA.
[Andrews, Paul W.] McMaster Univ, Dept Psychol Neurosci & Behav, Hamilton, ON, Canada.
[Thomson, J. Anderson, Jr.] Univ Virginia, Counseling & Psychol Serv, Student Hlth, Charlottesville, VA USA.
[Thomson, J. Anderson, Jr.] Univ Virginia, Inst Law Psychiat & Publ Policy, Charlottesville, VA USA.
C3 Vanderbilt University; McMaster University; University of Virginia; University of Virginia
RP Hollon, SD (autor correspondente), Vanderbilt Univ, Dept Psychol, Nashville, TN 37240 USA.
EM steven.d.hollon@vanderbilt.edu
CR Alloy LB, 2006, J ABNORM PSYCHOL, V115, P145, DOI 10.1037/0021-843X.115.1.145
Ambady N, 2002, J PERS SOC PSYCHOL, V83, P947, DOI 10.1037//0022-3514.83.4.947
American Psychiatric Association (APA), 2013, Diagnostic and statistical manual of mental disorders, V5th, DOI [10.1176/appi.books.9780890425596.744053, DOI 10.1176/APPI.BOOKS.9780890425596.744053, DOI 10.1176/APPI.BOOKS.9780890425596]
Andrews PW, 2021, EVOL HUM BEHAV, V42, P441, DOI 10.1016/j.evolhumbehav.2021.03.007
Andrews PW, 2020, CLIN PSYCHOL REV, V82, DOI 10.1016/j.cpr.2020.101927
Andrews PW, 2015, NEUROSCI BIOBEHAV R, V51, P164, DOI 10.1016/j.neubiorev.2015.01.018
Andrews PW, 2012, FRONT PSYCHOL, V3, DOI 10.3389/fpsyg.2012.00117
Andrews PW, 2011, FRONT PSYCHOL, V2, DOI 10.3389/fpsyg.2011.00159
Andrews PW, 2009, PSYCHOL REV, V116, P620, DOI 10.1037/a0016242
Andrews PW, 2002, BEHAV BRAIN SCI, V25, P534
[Anonymous], 2000, Am J Psychiatry, V157, P1
[Anonymous], 2014, MERRIAM WEBSTERS NEW, V11th
[Anonymous], 1970, The Structure of Scientific Revolutions
[Anonymous], 2016, Merriam-Webster's Medical Dictionary
BARBER JP, 1989, COGNITIVE THER RES, V13, P441, DOI 10.1007/BF01173905
Barbic SP, 2014, PLOS ONE, V9, DOI 10.1371/journal.pone.0112077
Barton DA, 2008, ARCH GEN PSYCHIAT, V65, P38, DOI 10.1001/archgenpsychiatry.2007.11
Bartoskova M, 2018, EVOL HUM BEHAV, V39, P277, DOI 10.1016/j.evolhumbehav.2018.01.005
Bateson M, 2011, CAN J PSYCHIAT, V56, P707, DOI 10.1177/070674371105601202
Beck AT., 2004, ASS COGNITIVE THERAP, V2nd
Belmaker RH, 2008, NEW ENGL J MED, V358, P55, DOI 10.1038/nrdp.2016.65
Buss DM, 2018, PERSPECT PSYCHOL SCI, V13, P155, DOI 10.1177/1745691617698225
Caveney S, 2006, J EXP BIOL, V209, P4858, DOI 10.1242/jeb.02607
Cosmides L., 2015, The Handbook of Evolutionary Psychology, V1, P3
Crook RJ, 2014, CURR BIOL, V24, P1121, DOI 10.1016/j.cub.2014.03.043
Cuijpers P, 2013, BMJ OPEN, V3, DOI 10.1136/bmjopen-2012-002542

Cuijpers P, 2012, CLIN PSYCHOL REV, V32, P280, DOI 10.1016/j.cpr.2012.01.003
Dawkins R., 2016, The selfish gene, V40th
deCatanzaro D, 1995, ETHOL SOCIOBIOL, V16, P385, DOI 10.1016/0162-3095(95)00055-0
DERUBEIS RJ, 1990, COGNITIVE THER RES, V14, P469, DOI 10.1007/BF01172968
DERUBEIS RJ, 1990, J CONSULT CLIN PSYCH, V58, P862, DOI 10.1037/0022-006X.58.6.862
DeRubeis RJ, 2005, ARCH GEN PSYCHIAT, V62, P409, DOI 10.1001/archpsyc.62.4.409
DeRubeis RJ, 2008, NAT REV NEUROSCI, V9, P788, DOI 10.1038/nrn2345
DeRubeis RJ, 2014, PLOS ONE, V9, DOI 10.1371/journal.pone.0083875
Diefendorf A.R., 1907, Clinical psychiatry: A textbook for students and physicians, abstracted and adapted from the 7th German edition of Kraepelin's lehrbuch der psychiatrie, V7th
Dimidjian S, 2006, J CONSULT CLIN PSYCH, V74, P658, DOI 10.1037/0022-006X.74.4.658
Dobson KS, 2008, J CONSULT CLIN PSYCH, V76, P468, DOI 10.1037/0022-006X.76.3.468
Driessen E, 2010, J CONSULT CLIN PSYCH, V78, P668, DOI 10.1037/a0020570
Durisko Z, 2015, J AFFECT DISORDERS, V172, P315, DOI 10.1016/j.jad.2014.09.032
ELKIN I, 1995, J CONSULT CLIN PSYCH, V63, P841, DOI 10.1037/0022-006X.63.5.841
Evans JST, 2013, PERSPECT PSYCHOL SCI, V8, P223, DOI 10.1177/1745691612460685
Feeley M, 1999, J CONSULT CLIN PSYCH, V67, P578, DOI 10.1037/0022-006X.67.4.578
Flinn MV, 2005, EVOL HUM BEHAV, V26, P10, DOI 10.1016/j.evolhumbehav.2004.08.005
Fournier JC, 2008, BRIT J PSYCHIAT, V192, P124, DOI 10.1192/bjp.bp.107.037234
Fournier JC, 2010, JAMA-J AM MED ASSOC, V303, P47, DOI 10.1001/jama.2009.1943
Frances AJ, 2013, BRIT J PSYCHIAT, V203, P1, DOI 10.1192/bjp.bp.113.127647
Frijda N. H., 1986, EMOTIONS
Gazzaley A, 2016, DISTRACTED MIND: ANCIENT BRAINS IN A HIGH-TECH WORLD, P1
GRAY JA, 1990, COGNITION EMOTION, V4, P269, DOI 10.1080/02699939008410799
Hagen EH, 2016, EVOL MED PUBLIC HLTH, P117, DOI 10.1093/emph/eow006
Hagen EH, 2011, CAN J PSYCHIAT, V56, P716, DOI 10.1177/070674371105601203
HAMILTON WD, 1964, J THEOR BIOL, V7, P17, DOI 10.1016/0022-5193(64)90039-6
Hammen C, 2018, ANNU REV CLIN PSYCHO, V14, P1, DOI [10.1146/annurev-clinpsy-050817-084811, 10.1146/annurev-clinpsy-050817084811]
Hankin BL, 2001, PSYCHOL BULL, V127, P773, DOI 10.1037//0033-2909.127.6.773
Hayes SC, 2004, BEHAV THER, V35, P639, DOI 10.1016/S0005-7894(04)80013-3

HOLLON SD, 1987, PSYCHOL BULL, V102, P139, DOI 10.1037/0033-2909.102.1.139
HOLLON SD, 1990, PERS SOC PSYCHOL B, V16, P58, DOI 10.1177/0146167290161005
Hollon SD, 2005, ARCH GEN PSYCHIAT, V62, P417, DOI 10.1001/archpsyc.62.4.417
Hollon SD, 2002, PSYCHOL SCI, P39, DOI 10.1111/1529-1006.00008
Hollon SD., HDB PSYCHOTHERAPY BE, V7th
Hollon SD, 2021, BEHAV RES THER, V143, DOI 10.1016/j.brat.2021.103849
Hollon SD, 2020, AM PSYCHOL, V75, P1207, DOI 10.1037/amp0000728
Hollon SD, 2021, COGNITIVE THER RES, V45, P402, DOI 10.1007/s10608-020-10132-1
Hyman SE, 1996, AM J PSYCHIAT, V153, P151
Jamison K., 1999, Night falls fast: Understanding suicide
Joiner TE, 2002, J SOC CLIN PSYCHOL, V21, P531, DOI 10.1521/jscp.21.5.531.22624
Kahneman D., 2013, Thinking, fast and slow, V1st pbk. ed.
Keller MC, 2007, AM J PSYCHIAT, V164, P1521, DOI 10.1176/appi.ajp.2007.06091564
Kessler RC, 2005, ARCH GEN PSYCHIAT, V62, P593, DOI 10.1001/archpsyc.62.6.593
Kirkpatrick L.A., 2003, BLACKWELL HDB SOCIAL, P411, DOI DOI 10.1002/9780470998557.CH16
Krishnan V, 2008, NATURE, V455, P894, DOI 10.1038/nature07455
Leary MR, 2000, ADV EXP SOC PSYCHOL, V32, P1, DOI 10.1016/S0065-2601(00)80003-9
Levenson RW, 1999, COGNITION EMOTION, V13, P481, DOI 10.1080/026999399379159
Lewis R., 1974, New Scientist, V63, P322
Li Y, 2014, PSYCHOL MED, V44, P3275, DOI 10.1017/S0033291714000749
Luoma JB, 2002, AM J PUBLIC HEALTH, V92, P1518, DOI 10.2105/AJPH.92.9.1518
Maier Steven F, 2006, Dialogues Clin Neurosci, V8, P397
Maslej M, 2020, EVOL PSYCHOL SCI, V6, P119, DOI 10.1007/s40806-019-00219-8
Maslej MM, 2020, CAN J BEHAV SCI, V52, P85, DOI 10.1037/cbs0000167
Maslej MM, 2017, PSYCHOTHER PSYCHOSOM, V86, P268, DOI 10.1159/000477940
Matza LS, 2003, ARCH GEN PSYCHIAT, V60, P817, DOI 10.1001/archpsyc.60.8.817
McGrath CL, 2013, JAMA PSYCHIAT, V70, P821, DOI 10.1001/jamapsychiatry.2013.143
Monroe SM, 2019, PSYCHOL REV, V126, P791, DOI 10.1037/rev0000157
Moore TJ, 2017, JAMA INTERN MED, V177, P274, DOI 10.1001/jamainternmed.2016.7507
Nademin E, 2008, ARCH SUICIDE RES, V12, P309, DOI 10.1080/13811110802324847
Nesse R.M, 2019, GOOD REASONS BAD FEE
Nesse RM, 2005, SPOUSAL BEREAVEMENT IN LATE LIFE, P195
Nisbett R.E., 1980, HUMAN INTERFACE STRA
Nolen-Hoeksema S, 2012, ANNU REV CLIN PSYCHO, V8, P161, DOI 10.1146/annurev-clinpsy-032511-143109
Platt J.R., 1966, The step to man

Roese NJ, 2017, ADV EXP SOC PSYCHOL, V56, P1, DOI 10.1016/bs.aesp.2017.02.001
Salkovskis Paul M., 1996, P48
Sell A, 2017, COGNITION, V168, P110, DOI 10.1016/j.cognition.2017.06.002
Sevcikova M, 2020, FRONT PSYCHOL, V11, DOI 10.3389/fpsyg.2020.01344
Shephard AM, 2016, Q REV BIOL, V91, P419, DOI 10.1086/689482
Sneddon LU, 2014, ANIM BEHAV, V97, P201, DOI 10.1016/j.anbehav.2014.09.007
Spitzer RL, 1999, AM J PSYCHIAT, V156, P1856
Strunk DR, 2007, J CONSULT CLIN PSYCH, V75, P523, DOI 10.1037/0022-006X.75.4.523
Syme KL, 2020, AM J PHYS ANTHROPOL, V171, P87, DOI 10.1002/ajpa.23965
Tang TZ, 2007, J CONSULT CLIN PSYCH, V75, P404, DOI 10.1037/0022-006X.75.3.404
TAYLOR SE, 1988, PSYCHOL BULL, V103, P193, DOI 10.1037/0033-2909.103.2.193
Thieleman K, 2014, RES SOCIAL WORK PRAC, V24, P114, DOI 10.1177/1049731512474695
Tooby J, 2003, PSYCHOL BULL, V129, P858, DOI 10.1037/0033-2909.129.6.858
TOOBY J, 1990, J PERS, V58, P17, DOI 10.1111/j.1467-6494.1990.tb00907.x
Tooby J, 2020, EVOL BEHAV SCI, V14, P390, DOI 10.1037/ebs0000250
Volk AA, 2013, EVOL HUM BEHAV, V34, P182, DOI 10.1016/j.evolhumbehav.2012.11.007
Wakefield JC, 2016, HIST PHIL THEOR LIFE, V15, P173, DOI 10.1007/978-94-017-7423-9_12
Wakefield JC, 2013, CLIN PSYCHOL REV, V33, P825, DOI 10.1016/j.cpr.2013.03.007
West SA, 2013, CURR BIOL, V23, pR577, DOI 10.1016/j.cub.2013.05.031
NR 109
TC 6
Z9 6
U1 0
U2 25
PU FRONTIERS MEDIA SA
PI LAUSANNE
PA AVENUE DU TRIBUNAL FEDERAL 34, LAUSANNE, CH-1015, SWITZERLAND
SN 1664-0640
J9 FRONT PSYCHIATRY
JI Front. Psychiatry
PD JUL 5
PY 2021
VL 12
AR 667592
DI 10.3389/fpsyt.2021.667592
PG 20
WC Psychiatry
WE Science Citation Index Expanded (SCI-EXPANDED); Social Science Citation Index (SSCI)
SC Psychiatry
GA TK6MF
UT WOS:000674269500001

PM 34290628
OA gold, Green Published
DA 2025-05-07
ER

PT J
AU Dorsey, AF
Roach, J
Burten, RB
Azcarate-Peril, MA
Thompson, AL
AF Dorsey, Achsah F.
Roach, Jeff
Burten, Rachel B.
Azcarate-Peril, M. Andrea
Thompson, Amanda L.
TI Intestinal microbiota composition and efficacy of iron supplementation in Peruvian children
SO AMERICAN JOURNAL OF HUMAN BIOLOGY
LA English
DT Article
ID GUT MICROBIOTA; FORTIFICATION; DEFICIENCY; ILLNESS; ANEMIA; HYPOTHESIS;
SIGNATURE; MALARIA; DISEASE; PROTEIN
AB ObjectiveDespite repeated public health interventions, anemia prevalence among children remains a concern. We use an evolutionary medicine perspective to examine the intestinal microbiome as a pathway underlying the efficacy of iron-sulfate treatment. This study explores whether gut microbiota composition differs between anemic children who respond and do not respond to treatment at baseline and posttreatment and if specific microbiota taxa remain associated with response to iron supplementation after controlling for relevant inflammatory and pathogenic variables.MethodsData come from 49 pre-school-aged anemic children living in San Juan de Lurigancho, Lima, Peru. We tested for differences in alpha and beta diversity using QIIME 2 and performed differential abundance testing in DESeq2 in R. We ran multivariate regression models to assess associations between abundance of specific taxa and response while controlling for relevant variables in Stata 17.ResultsWhile we found no evidence for gut microbiota diversity associated with child response to iron treatment, we observed several differential abundance patterns between responders and non-responders at both timepoints. Additionally, we present support for a nonzero relationship between lower relative abundance of Barnesiellaceae and response to iron supplementation in samples collected before and after treatment.ConclusionWhile larger studies and more specific approaches are needed to understand the relationship between microbes and anemia in an epidemiological context, this study suggests that investigating nutritional status and pathogen exposure is key to better understanding the gut microbiome and impact of iron fortification.
C1 [Dorsey, Achsah F.] Univ Massachusetts, Dept Anthropol, Amherst, MA USA.
[Roach, Jeff; Azcarate-Peril, M. Andrea] Univ North Carolina, Ctr Gastrointestinal Biol & Dis CGIBD, UNC Microbiome Core, Sch Med,Dept Med,Div Gastroenterol & Hepatol, Chapel Hill, NC USA.

[Burten, Rachel B.] Univ Massachusetts, Grad Program Organism & Evolutionary Biol, Amherst, MA USA.
[Azcarate-Peril, M. Andrea; Thompson, Amanda L.] Univ North Carolina, Gillings Sch Global Publ Hlth, Dept Nutr, Chapel Hill, NC USA.
[Thompson, Amanda L.] Univ North Carolina, Dept Anthropol, Chapel Hill, NC USA.
[Thompson, Amanda L.] Univ North Carolina, Carolina Populat Ctr, Chapel Hill, NC USA.
[Dorsey, Achsah F.] Univ Massachusetts, Dept Anthropol, 240 Hicks Way,Machmer Hall Rm 104, Amherst, MA 01003 USA.
C3 University of Massachusetts System; University of Massachusetts Amherst;
University of North Carolina; University of North Carolina Chapel Hill;
University of North Carolina School of Medicine; University of Massachusetts System; University of Massachusetts Amherst; University of
North Carolina; University of North Carolina Chapel Hill; University of
North Carolina; University of North Carolina Chapel Hill; University of
North Carolina; University of North Carolina Chapel Hill; University of
Massachusetts System; University of Massachusetts Amherst
RP Dorsey, AF (autor correspondente), Univ Massachusetts, Dept Anthropol, 240 Hicks Way,Machmer Hall Rm 104, Amherst, MA 01003 USA.
EM adorsey@umass.edu
RI Azcarate-Peril, M. Andrea/AFO-7157-2022
OI Burten, Rachel/0000-0001-5727-6591; Thompson, Amanda/0000-0002-5872-738X; Dorsey, Achsah/0000-0001-8860-0307; Azcarate-Peril, M. Andrea/0000-0003-0325-1289; ROACH, Jeffrey/0000-0001-9817-5877
FU National Science Foundation; Fulbright Association; Eunice Kennedy Shriver National Institute of Child Health and Human Development
FX National Science Foundation; Fulbright Association; Eunice Kennedy Shriver National Institute of Child Health and Human Development
CR Aeberli I, 2009, INT J OBESITY, V33, P1111, DOI 10.1038/ijo.2009.146
Alcazar L., 2013, The economic impact of anemia on Peru
Allali I, 2017, BMC MICROBIOL, V17, DOI 10.1186/s12866-017-1101-8
Andrews SC, 2003, FEMS MICROBIOL REV, V27, P215, DOI 10.1016/S0168-6445(03)00055-X
[Anonymous], 2009, GLOBAL HEALTH RISKS: MORTALITY AND BURDEN OF DISEASE ATTRIBUTABLE TO SELECTED MAJOR RISKS, P1
[Anonymous], 2022, Federal Policy for the Protection of Human Subjects ('Common Rule')
[Anonymous], 2016, GUIDELINE DAILY IRON
Arnold JW, 2021, MICROBIOME, V9, DOI 10.1186/s40168-020-00980-0
Balarajan Y, 2011, LANCET, V378, P2123, DOI 10.1016/S0140-6736(10)62304-5
BALLOU SP, 1992, ADV INTERNAL MED, V37, P313
Baltussen R, 2004, J NUTR, V134, P2678, DOI 10.1093/jn/134.10.2678
Björkstén B, 1999, CLIN EXP ALLERGY, V29, P342, DOI 10.1046/j.1365-2222.1999.00560.x

Bolyen E, 2019, NAT BIOTECHNOL, V37, P852, DOI 10.1038/s41587-019-0209-9
Braun V, 2011, CURR OPIN CHEM BIOL, V15, P328, DOI 10.1016/j.cbpa.2011.01.005
Caminiti C, 2016, J PEDIATR ENDOCR MET, V29, P543, DOI 10.1515/jpem-2014-0526
Caporaso JG, 2012, ISME J, V6, P1621, DOI 10.1038/ismej.2012.8
Chaparro CM, 2019, ANN NY ACAD SCI, V1450, P15, DOI 10.1111/nyas.14092
Cheng HL, 2012, OBES REV, V13, P150, DOI 10.1111/j.1467-789X.2011.00938.x
Cherayil BJ, 2011, CURR OPIN GASTROEN, V27, P523, DOI 10.1097/MOG.0b013e32834a4cd1
Collins JF., 2018, Physiology of the Gastrointestinal Tract, P1451, DOI [DOI 10.1016/B978-0-12-809954-4.00060-8, 10.1016/B978-0-12-809954-4.00060-8]
de Onis M, 2007, B WORLD HEALTH ORGAN, V85, P660, DOI 10.2471/BLT.07.043497
de Salud M., 2015, ANALISIS SITUACION S, P2015
Díaz-Rodríguez K, 2022, CHILDREN-BASEL, V9, DOI 10.3390/children9111615
Dorsey AF, 2021, AM J PHYS ANTHROPOL, V176, P54, DOI 10.1002/ajpa.24287
Dorsey AF, 2021, AM J HUM BIOL, V33, DOI 10.1002/ajhb.23538
Dorsey AF, 2018, AM J HUM BIOL, V30, DOI 10.1002/ajhb.23114
Eftekhari MH, 2009, PUBLIC HEALTH NUTR, V12, P2377, DOI 10.1017/S1368980009005187
Ewald P W, 1994, Evolution of Infectious Diseases
Gera T, 2002, BMJ-BRIT MED J, V325, P1142, DOI 10.1136/bmj.325.7373.1142
Gleason G., 2007, Nutritional Anemia, P45
Hadley C, 2015, AM J PHYS ANTHROPOL, V157, P675, DOI 10.1002/ajpa.22756
Jaeggi T, 2015, GUT, V64, P731, DOI 10.1136/gutjnl-2014-307720
Kassebaum NJ, 2014, BLOOD, V123, P615, DOI 10.1182/blood-2013-06-508325
Kortman GAM, 2014, FEMS MICROBIOL REV, V38, P1202, DOI 10.1111/1574-6976.12086
Kozich JJ, 2013, APPL ENVIRON MICROB, V79, P5112, DOI 10.1128/AEM.01043-13
Larsen N, 2010, PLOS ONE, V5, DOI 10.1371/journal.pone.0009085
Ley RE, 2005, P NATL ACAD SCI USA, V102, P11070, DOI 10.1073/pnas.0504978102
Litvak Y, 2017, CURR OPIN MICROBIOL, V39, P1, DOI 10.1016/j.mib.2017.07.003
Liu BD, 2020, FASEB J, V34, P3006, DOI 10.1096/fj.201901635RR
Manichanh C, 2006, GUT, V55, P205, DOI 10.1136/gut.2005.073817
Marini A., 2017, Standing Tall Peru's Success in Overcoming Its Stunting Crisis
McClorry S, 2018, AM J CLIN NUTR, V108, P1238, DOI 10.1093/ajcn/nqy249
Muleviciene A, 2018, ACTA MICROBIOL IMM H, V65, P551, DOI 10.1556/030.65.2018.045
MURRAY MJ, 1978, BRIT MED J, V2, P1113, DOI 10.1136/bmj.2.6145.1113
Naikare H, 2006, INFECT IMMUN, V74, P5433, DOI 10.1128/IAI.00052-06
Ogura Y, 2001, NATURE, V411, P603, DOI 10.1038/35079114

Oppenheimer SJ, 2001, J NUTR, V131, p616S, DOI 10.1093/jn/131.2.616S
Paganini D, 2017, GUT, V66, P1956, DOI 10.1136/gutjnl-2017-314418
Pasricha SR, 2018, BMJ-BRIT MED J, V362, DOI 10.1136/bmj.k3165
PAYNE SM, 1988, CRC CR REV MICROBIOL, V16, P81, DOI 10.3109/10408418809104468
Petry N, 2012, AM J CLIN NUTR, V96, P325, DOI 10.3945/ajcn.112.035717
Rogerson SJ, 2000, AM J TROP MED HYG, V62, P335, DOI 10.4269/ajtmh.2000.62.335
Rousham EK, 1998, BRIT J NUTR, V80, P451, DOI 10.1017/S0007114598001524
Salovaara S, 2003, J AGR FOOD CHEM, V51, P7820, DOI 10.1021/jf030177n
Sazawal S, 2006, LANCET, V367, P133, DOI 10.1016/S0140-6736(06)67962-2
Senn N, 2010, PLOS ONE, V5, DOI 10.1371/journal.pone.0009375
Shin NR, 2015, TRENDS BIOTECHNOL, V33, P496, DOI 10.1016/j.tibtech.2015.06.011
Smith RJ, 2018, AM J PHYS ANTHROPOL, V166, P236, DOI 10.1002/ajpa.23399
Gerós AS, 2020, IMMUNOLOGY, V161, P186, DOI 10.1111/imm.13236
Stoltzfus R.J., 2004, COMP QUANTIFICIATION, V1, P163
Stoltzfus R.J., 1998, Guidelines for the use of iron supplements to prevent and treat iron deficiency anemia
Tako E, 2008, BRIT J NUTR, V99, P472, DOI 10.1017/S0007114507825128
Turroni S, 2016, SCI REP-UK, V6, DOI 10.1038/srep32826
Valeggia CR, 2022, AM J BIOL ANTHROPOL, V177, P193, DOI 10.1002/ajpa.24444
Wander K, 2017, EVOL MED PUBLIC HLTH, P109, DOI 10.1093/emph/eox010
Wander K, 2009, AM J HUM BIOL, V21, P172, DOI 10.1002/ajhb.20839
WILLIAMS GC, 1991, Q REV BIOL, V66, P1, DOI 10.1086/417048
World Health Organization, 2002, The World Health Report 2002: Reducing risks, promoting healthy life
Wu GD, 2013, ANAEROBE, V24, P117, DOI 10.1016/j.anaerobe.2013.03.011
Xi RH, 2019, BMC ORAL HEALTH, V19, DOI 10.1186/s12903-019-0947-6
Zavaleta N, 2000, J NUTR, V130, p462S, DOI 10.1093/jn/130.2.462S
Zimmermann MB, 2007, LANCET, V370, P511, DOI 10.1016/S0140-6736(07)61235-5
Zimmermann MB, 2010, AM J CLIN NUTR, V92, P1406, DOI 10.3945/ajcn.110.004564
NR 73
TC 1
Z9 1
U1 0
U2 3
PU WILEY
PI HOBOKEN
PA 111 RIVER ST, HOBOKEN 07030-5774, NJ USA
SN 1042-0533
EI 1520-6300
J9 AM J HUM BIOL
JI Am. J. Hum. Biol.
PD AUG
PY 2024
VL 36
IS 8
DI 10.1002/ajhb.24058

EA FEB 2024
PG 15
WC Anthropology; Biology
WE Science Citation Index Expanded (SCI-EXPANDED); Social Science Citation Index (SSCI)
SC Anthropology; Life Sciences & Biomedicine - Other Topics
GA A5Y5X
UT WOS:001178040400001
PM 38420749
DA 2025-05-07
ER

PT J
AU Fall, CHD
   Kumaran, K
AF Fall, Caroline H. D.
   Kumaran, Kalyanaraman
TI Metabolic programming in early life in humans
SO PHILOSOPHICAL TRANSACTIONS OF THE ROYAL SOCIETY B-BIOLOGICAL SCIENCES
LA English
DT Article
DE birth weight; childhood weight gain; cardiovascular disease; type 2 diabetes; developmental origins of health and disease; programming
ID MULTIPLE MICRONUTRIENT SUPPLEMENTATION; RANDOMIZED CONTROLLED-TRIAL; CARDIOVASCULAR RISK-FACTORS; IMPAIRED GLUCOSE-TOLERANCE; BLOOD-PRESSURE
   LEVELS; SYMPOSIUM II INFANT; CHILDHOOD OBESITY; MATERNAL SMOKING; FOLLOW-UP; NUTRITIONAL SUPPLEMENTATION
AB An association of low birth weight with an increased risk of adult cardiovascular disease and diabetes led to the developmental origins of health and disease (DOHaD) hypothesis, which proposes that undernutrition during early development permanently 'programmes' organ structure and metabolism, leading to vulnerability to later cardio-metabolic disease. High birth weight caused by maternal gestational diabetes is also associated with later diabetes, suggesting that fetal over-nutrition also has programming effects. Post-natal factors (excess weight gain/obesity, smoking, poor diets and physical inactivity) interact with fetal exposures to increase disease risk. Animal studies have shown permanent metabolic effects in offspring after alterations to maternal or early post-natal diets but evidence in humans is largely limited to observational and quasi-experimental situations such as maternal famine exposure. Randomized trials of maternal nutritional interventions during pregnancy have so far had limited follow-up of the offspring. Moreover, interventions usually started after the first trimester and therefore missed key peri-conceptional or early pregnancy events such as epigenetic changes, placentation and fetal organogenesis. Recent and ongoing trials intervening pre-conceptionally and powered for long-term offspring follow-up will address these issues. While current preventive strategies for cardio-metabolic disease focus on high-risk individuals in mid-life, DOHaD concepts offer a 'primordial' preventive strategy to reduce disease in future generations by improving fetal and infant development.
   This article is part of the theme issue 'Developing differences: early-life effects and evolutionary medicine'.

C1 [Fall, Caroline H. D.; Kumaran, Kalyanaraman] Univ Southampton, Southampton Gen Hosp, Lifecourse Epidemiol Unit, MRC, Tremona Rd, Southampton SO16 6YD, Hants, England.
[Kumaran, Kalyanaraman] CSI Holdsworth Mem Hosp, Epidemiol Res Unit, Mysore, Karnataka, India.
C3 University of Southampton
RP Fall, CHD (autor correspondente), Univ Southampton, Southampton Gen Hosp, Lifecourse Epidemiol Unit, MRC, Tremona Rd, Southampton SO16 6YD, Hants, England.
EM chdf@mrc.soton.ac.uk
RI Kumaran, Kalyanaraman/KCK-5741-2024
FU Medical Research Council (UK); University of Southampton; MRC [MC_UP_A620_1016, MR/M005186/1, MR/N006208/1, G0400519, MC_UU_12011/3, MR/J000094/1] Funding Source: UKRI
FX Both authors are supported by the Medical Research Council (UK) and the
University of Southampton.
CR Adair LS, 2013, LANCET, V382, P525, DOI 10.1016/S0140-6736(13)60103-8
Al-Khudairy L, 2017, COCHRANE DB SYST REV, DOI 10.1002/14651858.CD012691
Alastalo H, 2013, J HUM HYPERTENS, V27, P90, DOI 10.1038/jhh.2012.6
[Anonymous], 2004, EUR J ENDOCRINOL S3
Bansal A, 2018, AM J PHYSIOL-ENDOC M, V315, pE15, DOI 10.1152/ajpendo.00424.2017
Behl M, 2013, ENVIRON HEALTH PERSP, V121, P170, DOI 10.1289/ehp.1205404
Bhargava SK, 2004, NEW ENGL J MED, V350, P865, DOI 10.1056/NEJMoa035698
Black RE, 2008, LANCET, V371, P243, DOI 10.1016/S0140-6736(13)60937-X
Blake-Lamb TL, 2016, AM J PREV MED, V50, P780, DOI 10.1016/j.amepre.2015.11.010
Catalano PM, 2009, DIABETES CARE, V32, P1076, DOI 10.2337/dc08-2077
Conlisk AJ, 2004, J NUTR, V134, P890, DOI 10.1093/jn/134.4.890
Dabelea D, 2000, DIABETES, V49, P2208, DOI 10.2337/diabetes.49.12.2208
Dalziel SR, 2005, LANCET, V365, P1856, DOI 10.1016/S0140-6736(05)66617-2
Devakumar D, 2014, LANCET GLOB HEALTH, V2, pE654, DOI 10.1016/S2214-109X(14)70314-6
Ehrlich SF, 2013, AM J EPIDEMIOL, V177, P768, DOI 10.1093/aje/kws312
Ekström EC, 2016, INT J EPIDEMIOL, V45, P1656, DOI 10.1093/ije/dyw199
Eriksson JG, 2001, BRIT MED J, V322, P949, DOI 10.1136/bmj.322.7292.949
Eriksson JG, 2003, DIABETOLOGIA, V46, P190, DOI 10.1007/s00125-002-1012-5
Fall CHD, 2011, ANN HUM BIOL, V38, P410, DOI 10.3109/03014460.2011.592513
Fall CHD, 2011, INT J EPIDEMIOL, V40, P47, DOI 10.1093/ije/dyq155
Fall CHD, 2008, DIABETES CARE, V31, P2349, DOI 10.2337/dc08-0911
Federation ID, 2019, IDF DIABETES ATLAS, DOI DOI 10.1016/S0140-6736(16)31679-8
Fernandez-Twinn DS, 2010, ANN NY ACAD SCI, V1212, P78, DOI 10.1111/j.1749-6632.2010.05798.x
Fewtrell MS, 2011, P NUTR SOC, V70, P472, DOI 10.1017/S0029665111000589

Fleming TP, 2018, LANCET, V391, P1842, DOI [10.1016/S0140-6736(18)30312-X, 10.1016/s0140-6736(18)30312-x]
FREINKEL N, 1980, DIABETES, V29, P1023, DOI 10.2337/diabetes.29.12.1023
Gaillard R, 2016, ANN NUTR METAB, V69, P171, DOI 10.1159/000453077
Gillman MW, 2010, DIABETES CARE, V33, P964, DOI 10.2337/dc09-1810
Godfrey KM, 2017, LANCET DIABETES ENDO, V5, P53, DOI 10.1016/S2213-8587(16)30107-3
Guillemette L, 2017, INT J OBESITY, V41, P999, DOI 10.1038/ijo.2017.65
Hambidge KM, 2014, BMC PREGNANCY CHILDB, V14, DOI 10.1186/1471-2393-14-111
Hamilton JK, 2010, OBESITY, V18, P340, DOI 10.1038/oby.2009.231
Hawkesworth S, 2013, J NUTR, V143, P728, DOI 10.3945/jn.112.168518
Hawkesworth S, 2011, AM J CLIN NUTR, V94, p1853S, DOI 10.3945/ajcn.110.000877
Heindel JJ, 2017, REPROD TOXICOL, V68, P3, DOI 10.1016/j.reprotox.2016.10.001
Hillier TA, 2007, DIABETES CARE, V30, P2287, DOI 10.2337/dc06-2361
Horta BL, 2015, ACTA PAEDIATR, V104, P30, DOI 10.1111/apa.13133
Huda SS, 2010, SEMIN FETAL NEONAT M, V15, P70, DOI 10.1016/j.siny.2009.09.006
Hult M, 2010, PLOS ONE, V5, DOI 10.1371/journal.pone.0013582
James P, 2018, INT J EPIDEMIOL, V47, P1910, DOI 10.1093/ije/dyy153
Khan AI, 2013, ACTA PAEDIATR, V102, P815, DOI 10.1111/apa.12282
Khan AI, 2015, MATERN CHILD NUTR, V11, P385, DOI 10.1111/mcn.12021
Kinra S, 2008, BMJ-BRIT MED J, V337, DOI 10.1136/bmj.a605
Krishnaveni GV, 2010, DIABETES CARE, V33, P402, DOI 10.2337/dc09-1393
Kühnen P, 2016, CELL METAB, V24, P502, DOI 10.1016/j.cmet.2016.08.001
Kumaran Kalyanaraman, 2017, BMC Nutr, V3, P41, DOI 10.1186/s40795-017-0143-5
LangleyEvans SC, 1996, PLACENTA, V17, P169, DOI 10.1016/S0143-4004(96)80010-5
Law CM, 2002, CIRCULATION, V105, P1088, DOI 10.1161/hc0902.104677
Li YP, 2015, BMJ-BRIT MED J, V351, DOI 10.1136/bmj.h3672
Li YP, 2011, J HYPERTENS, V29, P1085, DOI 10.1097/HJH.0b013e328345d969
Li YP, 2010, DIABETES, V59, P2400, DOI 10.2337/db10-0385
Lillycrop KA, 2012, BEST PRACT RES CL EN, V26, P667, DOI 10.1016/j.beem.2012.03.009
Luyckx VA, 2013, LANCET, V382, P273, DOI 10.1016/S0140-6736(13)60311-6
Martin RM, 2017, JAMA PEDIATR, V171, DOI 10.1001/jamapediatrics.2017.0698
Mattsson K, 2013, DIABETOLOGIA, V56, P1689, DOI 10.1007/s00125-013-2936-7
McMullen S, 2012, MED HYPOTHESES, V78, P88, DOI 10.1016/j.mehy.2011.09.047
Mead E, 2017, COCHRANE DB SYST REV, DOI 10.1002/14651858.CD012651
Metzger BE, 2009, DIABETES, V58, P453, DOI 10.2337/db08-1112
Nguyen PH, 2017, BMC WOMENS HEALTH, V17, DOI 10.1186/s12905-017-0401-3
Oken E, 2008, INT J OBESITY, V32, P201, DOI 10.1038/sj.ijo.0803760
Oliver M H, 2007, Soc Reprod Fertil Suppl, V64, P397
Osmond C, 2000, ENVIRON HEALTH PERSP, V108, P545, DOI 10.1289/ehp.00108s3545
Owen CG, 2011, P NUTR SOC, V70, P478, DOI 10.1017/S0029665111000590

Painter RC, 2008, BJOG-INT J OBSTET GY, V115, P1243, DOI 10.1111/j.1471-0528.2008.01822.x
Patel N, 2017, INT J OBESITY, V41, P1018, DOI 10.1038/ijo.2017.44
PEDERSEN J, 1954, ACTA ENDOCRINOL-COP, V16, P330, DOI 10.1530/acta.0.0160330
Pembrey M, 2014, J MED GENET, V51, P563, DOI 10.1136/jmedgenet-2014-102577
Philips EM, 2017, REPROD TOXICOL, V68, P105, DOI 10.1016/j.reprotox.2016.08.015
Phillips DIW, 2011, WORLD J DIABETES, V2, P211, DOI 10.4239/wjd.v2.i12.211
Phillips DIW, 2000, HYPERTENSION, V35, P1301, DOI 10.1161/01.HYP.35.6.1301
Potdar RD, 2014, AM J CLIN NUTR, V100, P1257, DOI 10.3945/ajcn.114.084921
RAVELLI GP, 1976, NEW ENGL J MED, V295, P349, DOI 10.1056/NEJM197608122950701
Rayfield S, 2017, J EPIDEMIOL COMMUN H, V71, P162, DOI 10.1136/jech-2016-207376
Reynolds RM, 2013, CLIN OBSTET GYNECOL, V56, P602, DOI 10.1097/GRF.0b013e31829939f7
Roseboom TJ, 2011, MATURITAS, V70, P141, DOI 10.1016/j.maturitas.2011.06.017
Smith J, 2009, J CLIN ENDOCR METAB, V94, P4275, DOI 10.1210/jc.2009-0709
Stein AD, 2006, AM J EPIDEMIOL, V164, P1160, DOI 10.1093/aje/kwj328
Stewart CP, 2009, J NUTR, V139, P1575, DOI 10.3945/jn.109.106666
Stewart CP, 2009, AM J CLIN NUTR, V90, P132, DOI 10.3945/ajcn.2008.27368
Summerbell CD, 2005, COCHRANE DB SYST REV, DOI [10.1002/14651858.CD001871.pub4, 10.1002/14651858.CD001871.pub2, 10.1002/14651858.CD001871.pub3]
Tanvig M, 2015, J CLIN ENDOCR METAB, V100, P175, DOI 10.1210/jc.2014-2675
Tanvig M, 2014, PLOS ONE, V9, DOI 10.1371/journal.pone.0089590
Vaidya A, 2008, LANCET, V371, P492, DOI 10.1016/S0140-6736(08)60172-5
Webb AL, 2005, INT J EPIDEMIOL, V34, P898, DOI 10.1093/ije/dyi097
Yehuda R, 2005, J CLIN ENDOCR METAB, V90, P4115, DOI 10.1210/jc.2005-0550
NR 85
TC 96
Z9 110
U1 1
U2 52
PU ROYAL SOC
PI LONDON
PA 6-9 CARLTON HOUSE TERRACE, LONDON SW1Y 5AG, ENGLAND
SN 0962-8436
EI 1471-2970
J9 PHILOS T R SOC B
JI Philos. Trans. R. Soc. B-Biol. Sci.
PD APR 15
PY 2019
VL 374

IS 1770
AR 20180123
DI 10.1098/rstb.2018.0123
PG 9
WC Biology
WE Science Citation Index Expanded (SCI-EXPANDED)
SC Life Sciences & Biomedicine - Other Topics
GA HN8ZI
UT WOS:000460487000014
PM 30966889
OA Green Accepted, Bronze, Green Published
DA 2025-05-07
ER

PT J
AU Daschner, A
   Cuéllar, C
AF Daschner, Alvaro
   Cuellar, Carmen
TI The hidden sense of symptoms: Urticaria can be beneficial
SO MEDICAL HYPOTHESES
LA English
DT Article
ID ANISAKIS-SIMPLEX; PARASITES; ALLERGY
AB Evolutionary Medicine can be useful when analysing the origin of disease and symptoms. Acute urticaria or anaphylaxis is bothersome and potentially life-threatening. We analyse this symptom-complex in the context of Gastro-allergic Anisakiasis (GAA), where the human is an incidental host for the cosmopolitan fish-nematode Anisakis simplex (A. simplex). The immunological response against this nematode resembles that against other helminths, but overt expression of allergic symptoms is by far more frequent in GAA. This could be due to the missing co-evolutionary relationship between host and parasite. Features of acute gastric parasitism with and without overt allergic type 1 hypersensitivity symptoms are compared with the abdominal complications in intestinal Anisakiasis, where clinically visible IgE mediated symptoms are missing. In GAA, parasite induced chronic abdominal complications are missing. We postulate that urticaria in GAA can be considered the price for rapidly expelling the live larva of A. simplex in those subjects whose evolutionary history made them more resistant to other helminth parasites. Further, urticaria is explained as a possibly exaggerated immunopathological feature in this special type of acute parasitism, where sensitized mast-cells are not only present in the skin, but also in the gastro-intestinal mucosa. This evolutionary analysis of clinical observations is the first known report that addresses the possible beneficial feature of hypersensitivity type 1 response with overt allergic urticaria-anaphylaxis in GAA and confers an evolutionary based sense to the acute IgE-mediated reaction. (C) 2010 Elsevier Ltd. All rights reserved.
C1 [Daschner, Alvaro] Hosp Univ La Princesa, Serv Alergia, E-28006 Madrid, Spain.
   [Cuellar, Carmen] Univ Complutense Madrid, Fac Farm, Dept Parasitol, Madrid, Spain.
C3 Hospital de La Princesa; Complutense University of Madrid

RP Daschner, A (autor correspondente), Hosp Univ La Princesa, Serv Alergia, C Diego de Leon 62, E-28006 Madrid, Spain.
EM alvarodaschner@gmail.com
RI Daschner, Alvaro/H-7639-2018; Cuellar, Carmen/H-1669-2015
OI Cuellar, Carmen/0000-0001-7948-9889
CR Alonso-Gómez A, 2004, PARASITOL RES, V93, P378, DOI 10.1007/s00436-004-1085-9
ASAISHI K, 1980, Gastroenterologia Japonica, V15, P128
Castán B, 2002, REV ESP ENFERM DIG, V94, P468
Cooper PJ, 2009, CURR OPIN ALLERGY CL, V9, P29, DOI 10.1097/ACI.0b013e32831f44a6
Daschner A, 2002, PARASITE IMMUNOL, V24, P243, DOI 10.1046/j.1365-3024.2002.00458.x
Daschner A, 1998, BRIT J DERMATOL, V139, P822, DOI 10.1046/j.1365-2133.1998.02507.x
Daschner A, 1999, CLIN EXP ALLERGY, V29, P1260, DOI 10.1046/j.1365-2222.1999.00569.x
Daschner A, 2000, J ALLERGY CLIN IMMUN, V105, P176, DOI 10.1016/S0091-6749(00)90194-5
Graham AL, 2002, Q REV BIOL, V77, P409, DOI 10.1086/344414
KUIPERS F C, 1960, Ned Tijdschr Geneeskd, V104, P422
Le Souëf PN, 2000, LANCET, V356, P242
Nesse R.M., 1994, WHY WE GET SICK NEW
Nesse RM, 2008, B WORLD HEALTH ORGAN, V86, P83, DOI 10.2471/BLT.07.049601
Olveira A, 1999, REV ESP ENFERM DIG, V91, P70
Peñas DL, 2000, MED CLIN-BARCELONA, V114, P177, DOI 10.1016/S0025-7753(00)71235-8
Puente P, 2008, EXP PARASITOL, V118, P271, DOI 10.1016/j.exppara.2007.07.002
Repiso Ortega A, 2003, Gastroenterol Hepatol, V26, P341, DOI 10.1157/13048887
Rook GAW, 2009, IMMUNOLOGY, V126, P3, DOI 10.1111/j.1365-2567.2008.03007.x
URBAN JF, 1992, IMMUNOL REV, V127, P205, DOI 10.1111/j.1600-065X.1992.tb01415.x
Yazdanbakhsh M, 2002, SCIENCE, V296, P490, DOI 10.1126/science.296.5567.490
NR 20
TC 23
Z9 23
U1 0
U2 6
PU CHURCHILL LIVINGSTONE
PI EDINBURGH
PA JOURNAL PRODUCTION DEPT, ROBERT STEVENSON HOUSE, 1-3 BAXTERS PLACE, LEITH WALK, EDINBURGH EH1 3AF, MIDLOTHIAN, SCOTLAND
SN 0306-9877
EI 1532-2777
J9 MED HYPOTHESES
JI Med. Hypotheses
PD DEC
PY 2010
VL 75

IS 6
BP 623
EP 626
DI 10.1016/j.mehy.2010.07.050
PG 4
WC Medicine, Research & Experimental
WE Science Citation Index Expanded (SCI-EXPANDED)
SC Research & Experimental Medicine
GA 715VO
UT WOS:000286918300043
PM 20729003
DA 2025-05-07
ER

PT J
AU Lev, I
   Bril, R
   Liu, YN
   Ceré, LI
   Rechavi, O
AF Lev, Itamar
   Bril, Roberta
   Liu, Yunan
   Cere, Lucila Ines
   Rechavi, Oded
TI Inter-generational consequences for growing Caenorhabditis
   elegans in liquid
SO PHILOSOPHICAL TRANSACTIONS OF THE ROYAL SOCIETY B-BIOLOGICAL SCIENCES
LA English
DT Article
DE trans-generational; inter-generational; liquid; inheritance;
   epigenetics; C. elegans
ID EPIGENETIC INHERITANCE; TRANSGENERATIONAL INHERITANCE; METHYLATION;
   NEMATODE; CULTURE; GENES; RNAS
AB In recent years, studies in Caenorhabditis elegans nematodes have shown that different stresses can generate multigenerational changes. Here, we show that worms that grow in liquid media, and also their plate-grown progeny, are different from worms whose ancestors were grown on plates. It has been suggested that C. elegans might encounter liquid environments in nature, although actual observations in the wild are few and far between. By contrast, in the laboratory, growing worms in liquid is commonplace, and often used as an alternative to growing worms on agar plates, to control the composition of the worms' diet, to starve (and synchronize) worms or to grow large populations for biochemical assays. We found that plate-grown descendants of M9 liquid medium-grown worms were longer than control worms, and the heritable effects were already apparent very early in development. We tested for the involvement of different known epigenetic inheritance mechanisms, but could not find a single mutant in which these inter-generational effects are cancelled. While we found that growing in liquid always leads to inter-generational changes in the worms' size, trans-generational effects were found to be variable, and in some cases, the effects were gone after one to two generations. These results demonstrate that standard cultivation

conditions in early life can dramatically change the worms' physiology in adulthood, and can also affect the next generations.
This article is part of the theme issue 'Developing differences: early-life effects and evolutionary medicine'.
C1 [Lev, Itamar; Bril, Roberta; Liu, Yunan; Cere, Lucila Ines; Rechavi, Oded] Tel Aviv Univ, Wise Fac Life Sci, Dept Neurobiol, IL-69978 Tel Aviv, Israel.
[Rechavi, Oded] Tel Aviv Univ, Sagol Sch Neurosci, IL-69978 Tel Aviv, Israel.
Tufts Univ, Allen Discovery Ctr, Medford, MA 02155 USA.
C3 Tel Aviv University; Tel Aviv University; Tufts University
RP Lev, I; Rechavi, O (autor correspondente), Tel Aviv Univ, Wise Fac Life Sci, Dept Neurobiol, IL-69978 Tel Aviv, Israel.; Rechavi, O (autor correspondente), Tel Aviv Univ, Sagol Sch Neurosci, IL-69978 Tel Aviv, Israel.
EM itamai.et@gmail.com; odedrechavi@gmail.com
RI Lev, Itamar/ABF-4276-2020
OI Bril Paroz, Roberta/0000-0002-0390-4764; Lev, Itamar/0000-0002-9100-5100
FU NIH Office of Research Infrastructure Programs [P40 OD010440]; ERC [335624]; Israel Science Foundation [1339/17]; Allen Discovery Center through the Paul G. Allen Frontiers Group [12171]; Adelis foundation [01430001000]
FX Some strains were provided by the CGC, which is funded by NIH Office of
Research Infrastructure Programs (P40 OD010440). This work was supported
by the ERC (grant no. 335624) and the Israel Science Foundation (grant no. 1339/17) and O.R. gratefully acknowledges support by the Allen Discovery Center through the Paul G. Allen Frontiers Group (12171) and the support of the Adelis foundation (no. 01430001000).
CR Anava Sarit, 2014, Worm, V3, pe989798, DOI 10.4161/21624054.2014.989798
BARGMANN CI, 1993, CELL, V74, P515, DOI 10.1016/0092-8674(93)80053-H
Ben-David U, 2018, NATURE, V560, P325, DOI 10.1038/s41586-018-0409-3
Buckley BA, 2012, NATURE, V489, P447, DOI 10.1038/nature11352
Burton NO, 2017, NAT CELL BIOL, V19, P252, DOI 10.1038/ncb3470
Burton NO, 2011, P NATL ACAD SCI USA, V108, P19683, DOI 10.1073/pnas.1113310108
Campos EI, 2014, TRENDS CELL BIOL, V24, P664, DOI 10.1016/j.tcb.2014.08.004
Capes-Davis A, 2010, INT J CANCER, V127, P1, DOI 10.1002/ijc.25242
Çelen I, 2018, BMC GENOMICS, V19, DOI 10.1186/s12864-018-4948-7
Demoinet E, 2017, P NATL ACAD SCI USA, V114, pE2689, DOI 10.1073/pnas.1616171114
Doh Jung H, 2016, J Biol Methods, V3, pe44, DOI 10.14440/jbm.2016.109
Ehlers RU, 1998, BIOCONTROL, V43, P77, DOI 10.1023/A:1009965922794
Frokjær-Jensen C, 2016, CELL, V166, P343, DOI 10.1016/j.cell.2016.05.072
Gammon DB, 2017, CURR BIOL, V27, P795, DOI 10.1016/j.cub.2017.02.004
Gialitakis M, 2010, MOL CELL BIOL, V30, P2046, DOI 10.1128/MCB.00906-09
GOLDEN JW, 1985, MOL GEN GENET, V198, P534, DOI 10.1007/BF00332953
Gu SG, 2012, NAT GENET, V44, P157, DOI 10.1038/ng.1039

Guang S, 2008, SCIENCE, V321, P537, DOI 10.1126/science.1157647
Guo XY, 2012, J VIROL, V86, P11645, DOI 10.1128/JVI.01501-12
Han RC, 2000, J INVERTEBR PATHOL, V75, P55, DOI 10.1006/jipa.1999.4900
Heard E, 2014, CELL, V157, P95, DOI 10.1016/j.cell.2014.02.045
Hodgkin J, 2013, CURR BIOL, V23, P2157, DOI 10.1016/j.cub.2013.08.060
Hu P. J., 2007, DAUER, V8, P1, DOI [10.1895/wormbook.1.144.1, DOI 10.1895/WORMBOOK.1.144.1]
Jobson MA, 2015, C ELEGANS GENETICS, V201, P1, DOI [10.1534/genetics.115.178699, DOI 10.1534/GENETICS.115.178699]
Kaplan F, 2011, PLOS ONE, V6, DOI 10.1371/journal.pone.0017804
Kiontke Karin, 2005, P1
Kishimoto S, 2017, NAT COMMUN, V8, DOI 10.1038/ncomms14031
Klosin A, 2017, SCIENCE, V356, P316, DOI 10.1126/science.aah6412
Laranjeiro R, 2017, BMC BIOL, V15, DOI 10.1186/s12915-017-0368-4
Lev I, 2018, BIORXIV, DOI [10.1101/338582, DOI 10.1101/338582, 10.1101/338582.]
Lev I, 2017, CURR BIOL, V27, P1138, DOI 10.1016/j.cub.2017.03.008
LEWIS JA, 1977, J COMP NEUROL, V172, P489, DOI 10.1002/cne.901720306
Light WH, 2013, PLOS BIOL, V11, DOI 10.1371/journal.pbio.1001524
Ludewig AH, 2017, PLOS GENET, V13, DOI 10.1371/journal.pgen.1006717
McGrath PT, 2011, NATURE, V477, P321, DOI 10.1038/nature10378
McMurchy AN, 2017, ELIFE, V6, DOI [10.7554/eLife.21666, 10.7554/eLife.32516]
Merrill GF, 1998, METHOD CELL BIOL, V57, P229
Moore BT, 2013, PLOS ONE, V8, DOI 10.1371/journal.pone.0057142
Murphy Coleen T, 2013, WormBook, P1, DOI 10.1895/wormbook.1.164.1
Ni JZ, 2016, EPIGENET CHROMATIN, V9, DOI 10.1186/s13072-016-0052-x
Niida H, 1998, NAT GENET, V19, P203, DOI 10.1038/580
PERKINS LA, 1986, DEV BIOL, V117, P456, DOI 10.1016/0012-1606(86)90314-3
Peters A, 2017, MICROBIAL CONTROL OF INSECT AND MITE PESTS: FROM THEORY TO PRACTICE, P157, DOI 10.1016/B978-0-12-803527-6.00010-X
Radick G, 2015, SCIENCE, V350, P159, DOI 10.1126/science.aab3846
Rechavi O, 2017, CURR BIOL, V27, pR720, DOI 10.1016/j.cub.2017.05.043
Rechavi O, 2014, CELL, V158, P277, DOI 10.1016/j.cell.2014.06.020
Rechavi O, 2011, CELL, V147, P1248, DOI 10.1016/j.cell.2011.10.042
Sapetschnig A, 2015, PLOS GENET, V11, DOI 10.1371/journal.pgen.1005078
Schott D, 2014, SCI REP-UK, V4, DOI 10.1038/srep07387
Schulenburg H, 2017, GENETICS, V206, P55, DOI 10.1534/genetics.116.195511
Spracklin G, 2017, GENETICS, V206, P1, DOI [10.20944/, DOI 10.20944/]
Srinivasan J, 2008, NATURE, V454, P1115, DOI 10.1038/nature07168
Stiernagle Theresa, 2006, WormBook, P1
Strauch O, 1998, APPL MICROBIOL BIOT, V50, P369, DOI 10.1007/s002530051306
Surrey MR, 1996, J INVERTEBR PATHOL, V67, P92, DOI 10.1006/jipa.1996.0013
Szewczyk NJ, 2003, BMC BIOTECHNOL, V3, DOI 10.1186/1472-6750-3-19
Tauffenberger A, 2014, PLOS GENET, V10, DOI 10.1371/journal.pgen.1004346
Timmons L, 2003, MOL BIOL CELL, V14, P2972, DOI 10.1091/mbc.E03-01-0858
Towbin BD, 2012, CELL, V150, P934, DOI 10.1016/j.cell.2012.06.051

Upadhyay D, 2015, AM J BIOSCI BIOENG, V3, P203, DOI [10.11648/j.bio.20150306.19, DOI 10.11648/J.BIO.20150306.19]
von Reuss SH, 2012, J AM CHEM SOC, V134, P1817, DOI 10.1021/ja210202y
Wang XR, 2013, BMC PLANT BIOL, V13, DOI 10.1186/1471-2229-13-77
Webster AK, 2018, GENETICS, V210, P263, DOI 10.1534/genetics.118.301250
Werner MS, 2017, SCI REP-UK, V7, DOI 10.1038/s41598-017-07455-7
NR 64
TC 11
Z9 14
U1 1
U2 26
PU ROYAL SOC
PI LONDON
PA 6-9 CARLTON HOUSE TERRACE, LONDON SW1Y 5AG, ENGLAND
SN 0962-8436
EI 1471-2970
J9 PHILOS T R SOC B
JI Philos. Trans. R. Soc. B-Biol. Sci.
PD APR 15
PY 2019
VL 374
IS 1770
AR 20180125
DI 10.1098/rstb.2018.0125
PG 8
WC Biology
WE Science Citation Index Expanded (SCI-EXPANDED)
SC Life Sciences & Biomedicine - Other Topics
GA HN8ZI
UT WOS:000460487000016
PM 30966881
OA hybrid, Green Published
DA 2025-05-07
ER

PT J
AU Jamison, CS
Wallace, M
Jamison, PL
AF Jamison, CS
Wallace, M
Jamison, PL
TI Contemporary work characteristics, stress, and ill health
SO AMERICAN JOURNAL OF HUMAN BIOLOGY
LA English
DT Article
ID INCOME INEQUALITY; HEART-DISEASE; CARDIOVASCULAR-DISEASE; SOCIOECONOMIC-STATUS; JOB-SATISFACTION; RISK-FACTORS; FOLLOW-UP; MORTALITY; MORBIDITY; EDUCATION
AB This study investigates the relationship between work and ill health resulting from stressors originating in the work environment. The association between work and health has been well-documented. Factors in the work environment leading to ill health have included generalized

psychological stress and/or specific physical environmental characteristics. Data for this study come from a 1998 telephone survey, the Indiana Survey of Work in a Polarized Economy, under the auspices of the Center for Survey Research at Indiana University. The dependent variable is an index that measures ill health among workers; control variables include sociodemographic and lifestyle characteristics of these workers as well as their job characteristics; and the four key independent variables are occupational status, organizational disruption, layoff experience, and educational discordance of the workers. Multiple regression analysis on the entire sample (n = 919) demonstrates that occupational status is negatively associated with ill health, while organizational disruption, layoff experience, and educational discordance are positively associated with it. Separate analyses for male (n = 474) and female (n = 445) respondents find significant sex differences in the effects of the key variables: for males, organizational disruption is positively related to ill health while for women occupational status is negatively related and layoff experience and educational discordance are positively related to ill health. The findings from the present research provide insight into the interrelationship of occupational environment and health within the framework of evolutionary medicine. (C) 2003 Wiley-Liss, Inc.
C1 Indiana Univ, Inst Social Res, Bloomington, IN 47405 USA.
Indiana Univ, Dept Anthropol, Bloomington, IN 47405 USA.
Univ Connecticut, Dept Sociol, Storrs, CT 06268 USA.
C3 Indiana University System; Indiana University Bloomington; Indiana University System; Indiana University Bloomington; University of Connecticut
RP Indiana Univ, Inst Social Res, 1022 E 3rd St, Bloomington, IN 47405 USA.
EM cjamison@indiana.edu
CR Aasland OG, 1997, SOC SCI MED, V45, P1615, DOI 10.1016/S0277-9536(97)00093-2
Adler NE, 2000, HEALTH PSYCHOL, V19, P586, DOI 10.1037/0278-6133.19.6.586
ADLER NE, 1994, AM PSYCHOL, V49, P15, DOI 10.1037/0003-066X.49.1.15
Anderson R, 1999, EVOLUTIONARY MEDICI NE, P333
ANGEL R, 1988, AM SOCIOL REV, V53, P464, DOI 10.2307/2095653
[Anonymous], 1991, DICT OCCUPATIONAL TI
[Anonymous], 1956, The stress of life
Bekker MHJ, 2000, INT J BEHAV MED, V7, P28, DOI 10.1207/S15327558IJBM0701_3
BOWLIN SJ, 1993, J CLIN EPIDEMIOL, V46, P561, DOI 10.1016/0895-4356(93)90129-O
BRIDGES W, 1994, FORTUNE 0919, P62
Bridges W., 1994, JOB SHIFT PROSPER WO
Burke R.J., 2000, The Organization in Crisis: Downsizing, Restructuring, and Privatization
Butterworth T, 1999, STRESS MEDICINE, V15, P27
Cahill J, 1996, INT J HEALTH SERV, V26, P731, DOI 10.2190/PUHN-DLH6-C33X-EFLF
CHILDE VG, 1950, TOWN PLANN REV, P213
Cohen S, 1999, PSYCHOSOM MED, V61, P175, DOI 10.1097/00006842-199903000-00009
COHEN S, 1991, PSYCHOL BULL, V109, P5, DOI 10.1037/0033-2909.109.1.5

DeVellis R.F., 1991, SCALE DEV THEORY APP, DOI DOI 10.1037/0022-006X.56.5.754
Eisenberg J., 1986, DOCTORS DECISIONS CO
EISENBERG JM, 1979, ANN INTERN MED, V90, P957, DOI 10.7326/0003-4819-90-6-957
ETTNER SL, 2001, J OCCUP HEALTH PSYCH, V2, P101
Ewald PaulW., 2000, PLAGUE TIME STEALTH
Ferraro KF, 1999, AM SOCIOL REV, V64, P303, DOI 10.2307/2657534
Ferraro KF, 2000, AM J PUBLIC HEALTH, V90, P103, DOI 10.2105/AJPH.90.1.103
Ferrie JE, 1998, AM J PUBLIC HEALTH, V88, P1030, DOI 10.2105/AJPH.88.7.1030
Franceschi C, 2000, ANN NY ACAD SCI, V908, P244
Gerber LM, 1999, EVOLUTIONARY MEDICI NE, P443
GUST D, 1993, HORM BEHAV, V7, P229
Hartung J., 1988, Self-deception: An adaptive mechanism?, P170
HAUSER RM, 1997, SOCIOLOGICAL METHODO
Johnson A.W., 2000, EVOLUTION HUMAN SOC
Joplin JRW, 1999, J ORGAN BEHAV, V20, P783, DOI 10.1002/(SICI)1099-1379(199911)20:6<783::AID-JOB923>3.0.CO;2-P
KAPLAN GA, 1983, AM J EPIDEMIOL, V117, P292, DOI 10.1093/oxfordjournals.aje.a113541
KARASEK R, 1990, J ORGAN BEHAV, V11, P171, DOI 10.1002/job.4030110302
Kawachi I, 1999, AM J PUBLIC HEALTH, V89, P1187, DOI 10.2105/AJPH.89.8.1187
Kiecolt-Glaser JK, 2002, ANNU REV PSYCHOL, V53, P83, DOI 10.1146/annurev.psych.53.100901.135217
Kivimäki M, 2000, BRIT MED J, V320, P971, DOI 10.1136/bmj.320.7240.971
Kivimaki M, 1997, J APPL PSYCHOL, V82, P858, DOI 10.1037/0021-9010.82.6.858
Landsbergis P A, 1993, New Solut, V3, P42, DOI 10.2190/NS3.4.d
Lynch J, 1997, AM J PUBLIC HEALTH, V87, P617, DOI 10.2105/AJPH.87.4.617
Mack DA, 1998, APPL PSYCHOL-INT REV, V47, P219, DOI 10.1080/026999498377999
MACKENBACH JP, 2000, BMJ-BRIT MED J, V324, P1
Mast MS, 2002, PERS SOC PSYCHOL B, V28, P29, DOI 10.1177/0146167202281003
MATTIASSON I, 1990, BMJ-BRIT MED J, V301, P461, DOI 10.1136/bmj.301.6750.461
McEwen BS, 2000, BRAIN RES, V886, P172, DOI 10.1016/S0006-8993(00)02950-4
MORGAN D, 2002, NAT NEUROSCI 0122, P1, DOI DOI 10.1038/NN.798
MOSSEY JM, 1982, AM J PUBLIC HEALTH, V72, P800, DOI 10.2105/AJPH.72.8.800
Muller A, 2002, BRIT MED J, V324, P23, DOI 10.1136/bmj.324.7328.23
Nesse R.M., 1994, WHY WE GET SICK NEW
NETTERSTROM B, 1993, INT J EPIDEMIOL, V22, P51, DOI 10.1093/ije/22.1.51
Osler M, 2002, BRIT MED J, V324, P13, DOI 10.1136/bmj.324.7328.13
Ostrove JM, 1999, ANN NY ACAD SCI, V896, P335, DOI 10.1111/j.1749-6632.1999.tb08133.x
PACKER C, 1995, NATURE, V373, P60, DOI 10.1038/373060a0
Panter-Brick C., 1999, HORMONES HLTH BEHAV, P139

PATEL P, 1995, BRIT MED J, V311, P711, DOI 10.1136/bmj.311.7007.711
Pollard TM, 1997, YEARB PHYS ANTHROPOL, V40, P1
Pratto F, 1997, BRIT J SOC PSYCHOL, V36, P49, DOI 10.1111/j.2044-
8309.1997.tb01118.x
Probst T M, 2001, J Occup Health Psychol, V6, P139, DOI 10.1037/1076-
8998.6.2.139
Pusey A, 1997, SCIENCE, V277, P828, DOI 10.1126/science.277.5327.828
Quick JC, 2001, STRESS HEALTH, V17, P147, DOI 10.1002/smi.893
RAY JC, 1992, AM J PRIMATOL, V28, P231, DOI 10.1002/ajp.1350280402
Reboul P, 2001, ARTHRITIS RHEUM, V44, P73, DOI 10.1002/1529-
0131(200101)44:1<73::AID-ANR11>3.0.CO;2-E
Sapolsky RM, 1996, SCIENCE, V273, P749, DOI
10.1126/science.273.5276.749
SAPOLSKY RM, 2001, HDB PHYSL, P517
Sapolsky RM., 1999, HORMONES HLTH BEHAV, P18
Sapolsky RM., 1998, WHY ZEBRAS DONT GET
Schaubroeck J, 2001, J APPL PSYCHOL, V86, P265, DOI 10.1037//0021-
9010.86.2.265
Schernhammer ES, 2001, J NATL CANCER I, V93, P1563, DOI
10.1093/jnci/93.20.1563
Sturm R, 2002, BMJ-BRIT MED J, V324, P20, DOI 10.1136/bmj.324.7328.20
Taylor SE, 2000, PSYCHOL REV, V107, P411, DOI 10.1037/0033-
295X.107.3.411
Trevathan WR, 1999, EVOLUTIONARY MEDICI NE, P183
Vahtera J, 1997, LANCET, V350, P1124, DOI 10.1016/S0140-6736(97)03216-
9
Vgontzas AN, 2001, J CLIN ENDOCR METAB, V86, P1489, DOI
10.1210/jc.86.4.1489
Walters J.R., 1987, P306
WATTENMAKER P, 1998, HOUSEHOLD STATE MESO
WILLIAMS GC, 1991, Q REV BIOL, V66, P1, DOI 10.1086/417048
YOSHIO M, 1998, OCCUP ENVIRON MED, V56, P41
Zimmer C, 2001, SCIENCE, V293, P1974, DOI
10.1126/science.293.5537.1974
NR 78
TC 13
Z9 17
U1 0
U2 5
PU WILEY
PI HOBOKEN
PA 111 RIVER ST, HOBOKEN 07030-5774, NJ USA
SN 1042-0533
EI 1520-6300
J9 AM J HUM BIOL
JI Am. J. Hum. Biol.
PD JAN-FEB
PY 2004
VL 16
IS 1
BP 43
EP 56
DI 10.1002/ajhb.10237
PG 14

WC Anthropology; Biology
WE Science Citation Index Expanded (SCI-EXPANDED); Social Science Citation Index (SSCI)
SC Anthropology; Life Sciences & Biomedicine - Other Topics
GA 758WU
UT WOS:000187675200005
PM 14689515
DA 2025-05-07
ER

PT J
AU Vinogradov, AE
   Anatskaya, O
AF Vinogradov, Alexander E.
   Anatskaya, Olga, V
TI Cellular Biogenetic Law and Its Distortion by Protein Interactions: A
   Possible Unified Framework for Cancer Biology and Regenerative
Medicine
SO INTERNATIONAL JOURNAL OF MOLECULAR SCIENCES
LA English
DT Article
DE cell differentiation; gene phylostratigraphy; gene expression;
   interactome; embryonic stem cells; induced pluripotent stem cells;
   recapitulation law; Heckel's law; humans; whole genome duplication;
   evolutionary medicine
ID TRANSCRIPTOME; METAANALYSIS; PLASTICITY; EVOLUTION; CELLS; SET
AB The biogenetic law (recapitulation law) states that ontogenesis recapitulates phylogenesis. However, this law can be distorted by the modification of development. We showed the recapitulation of phylogenesis during the differentiation of various cell types, using a meta-analysis of human single-cell transcriptomes, with the control for cell cycle activity and the improved phylostratigraphy (gene dating). The multipotent progenitors, differentiated from pluripotent embryonic stem cells (ESC), showed the downregulation of unicellular (UC) genes and the upregulation of multicellular (MC) genes, but only in the case of those originating up to the Euteleostomi (bony vertebrates). This picture strikingly resembles the evolutionary profile of regulatory gene expansion due to gene duplication in the human genome. The recapitulation of phylogenesis in the induced pluripotent stem cells (iPSC) during their differentiation resembles the ESC pattern. The unipotent erythroblasts differentiating into erythrocytes showed the downregulation of UC genes and the upregulation of MC genes originating after the Euteleostomi. The MC interactome neighborhood of a protein encoded by a UC gene reverses the gene expression pattern. The functional analysis showed that the evolved environment of the UC proteins is typical for protein modifiers and signaling-related proteins. Besides a fundamental aspect, this approach can provide a unified framework for cancer biology and regenerative/rejuvenation medicine because oncogenesis can be defined as an atavistic reversal to a UC state, while regeneration and rejuvenation require an ontogenetic reversal.
C1 [Vinogradov, Alexander E.; Anatskaya, Olga, V] Russian Acad Sci, Inst Cytol, St Petersburg 194064, Russia.
C3 Russian Academy of Sciences; St. Petersburg Scientific Centre of the
   Russian Academy of Sciences; Institute of Cytology RAS

RP Vinogradov, AE (autor correspondente), Russian Acad Sci, Inst Cytol, St Petersburg 194064, Russia.
EM aevin@incras.ru
RI Vinogradov, Alexander/R-3923-2016; Anatskaya, Olga/R-4042-2016
OI Anatskaya, Olga/0000-0003-4538-8056
FU Ministry of Science and Higher Education of the Russian Federation [075-15-2021-1075]
FX This work was funded by the Ministry of Science and Higher Education of the Russian Federation (Agreement No. 075-15-2021-1075, signed 28 September 2021).
CR Abzhanov A, 2013, TRENDS GENET, V29, P712, DOI 10.1016/j.tig.2013.09.004
Aktipis CA, 2015, PHILOS T R SOC B, V370, DOI 10.1098/rstb.2014.0219
Anatskaya OV, 2021, MOL BIOL+, V55, P813, DOI [10.31857/S0026898421060021, 10.1134/S0026893321050022]
Anatskaya OV, 2022, INT J MOL SCI, V23, DOI 10.3390/ijms23073542
[Anonymous], 2002, STEM CELLS FUT REG M
Assou S, 2007, STEM CELLS, V25, P961, DOI 10.1634/stemcells.2006-0352
Bussey KJ, 2021, PROG BIOPHYS MOL BIO, V165, P49, DOI 10.1016/j.pbiomolbio.2021.08.002
Bussey KJ, 2017, P NATL ACAD SCI USA, V114, P6160, DOI 10.1073/pnas.1706990114
Camp JG, 2017, NATURE, V546, P533, DOI 10.1038/nature22796
Chu LF, 2016, GENOME BIOL, V17, DOI 10.1186/s13059-016-1033-x
Corradetti B, 2021, BIOESSAYS, V43, DOI 10.1002/bies.202000339
Court F, 2017, ONCOTARGET, V8, P4110, DOI 10.18632/oncotarget.13746
Davies PCW, 2011, PHYS BIOL, V8, DOI 10.1088/1478-3975/8/1/015001
Dobreva MP, 2022, J EXP ZOOL PART B, V338, P87, DOI 10.1002/jez.b.23103
Greaves M, 2015, CANCER DISCOV, V5, P806, DOI 10.1158/2159-8290.CD-15-0439
Heng JL, 2022, SEMIN CANCER BIOL, V81, P160, DOI 10.1016/j.semcancer.2020.11.003
Hu H, 2019, NUCLEIC ACIDS RES, V47, pD33, DOI 10.1093/nar/gky822
Huang S, 2021, TRENDS CANCER, V7, P309, DOI 10.1016/j.trecan.2020.12.007
Irie N, 2018, ZOOL LETT, V4, DOI 10.1186/s40851-018-0114-y
Khyeam Sheamin, 2021, Adv Genet (Hoboken), V2, DOI 10.1002/ggn2.10042
Lambert SA, 2018, CELL, V172, P650, DOI 10.1016/j.cell.2018.01.029
Liberzon A, 2015, CELL SYST, V1, P417, DOI 10.1016/j.cels.2015.12.004
Lineweaver CH, 2021, BIOESSAYS, V43, DOI 10.1002/bies.202000305
Lineweaver CH, 2014, BIOESSAYS, V36, P827, DOI 10.1002/bies.201400070
Mackay A, 2017, CANCER CELL, V32, P520, DOI 10.1016/j.ccell.2017.08.017
Mononen MM, 2020, STEM CELLS, V38, P1267, DOI 10.1002/stem.3236
Moroz LL, 2021, FRONT CELL DEV BIOL, V9, DOI 10.3389/fcell.2021.726563
Nguyen PD, 2021, CELL MOL LIFE SCI, V78, P5107, DOI 10.1007/s00018-021-03831-9
Ossipova O, 2018, GENESIS, V56, DOI 10.1002/dvg.23108
Petropoulos S, 2016, CELL, V165, P1012, DOI [10.1016/j.cell.2016.03.023, 10.1016/j.cell.2016.08.009]
Pisco AO, 2015, BRIT J CANCER, V112, P1725, DOI 10.1038/bjc.2015.146

Postovit LM, 2008, P NATL ACAD SCI USA, V105, P4329, DOI 10.1073/pnas.0800467105
Ritchie ME, 2015, NUCLEIC ACIDS RES, V43, DOI 10.1093/nar/gkv007
Sarkans U, 2021, NUCLEIC ACIDS RES, V49, pD1502, DOI 10.1093/nar/gkaa1062
Sayers EW, 2022, NUCLEIC ACIDS RES, V50, pD20, DOI 10.1093/nar/gkab1112
Scudellari M, 2016, NATURE, V534, P310, DOI 10.1038/534310a
Sha QQ, 2020, NAT COMMUN, V11, DOI 10.1038/s41467-020-18680-6
Singh PP, 2020, NUCLEIC ACIDS RES, V48, pD724, DOI 10.1093/nar/gkz909
Sonnenschein C, 2015, JNCI-J NATL CANCER I, V107, DOI 10.1093/jnci/djv236
Storey JD, 2003, P NATL ACAD SCI USA, V100, P9440, DOI 10.1073/pnas.1530509100
Szklarczyk D, 2021, NUCLEIC ACIDS RES, V49, pD605, DOI 10.1093/nar/gkaa1074
Talbert PB, 2019, NAT REV GENET, V20, P283, DOI 10.1038/s41576-019-0105-7
Tate JG, 2019, NUCLEIC ACIDS RES, V47, pD941, DOI 10.1093/nar/gky1015
Thompson D., 1917, On Growth and Form
Torday JS, 2018, PROG BIOPHYS MOL BIO, V135, P1, DOI 10.1016/j.pbiomolbio.2017.12.003
Trigos AS, 2019, ELIFE, V8, DOI 10.7554/eLife.40947
Trigos AS, 2018, BRIT J CANCER, V118, P145, DOI 10.1038/bjc.2017.398
Trigos AS, 2017, P NATL ACAD SCI USA, V114, P6406, DOI 10.1073/pnas.1617743114
Uesaka M, 2022, J EXP ZOOL PART B, V338, P9, DOI 10.1002/jez.b.23116
Uesaka M, 2022, J EXP ZOOL PART B, V338, P76, DOI 10.1002/jez.b.23027
Uesaka M, 2019, ZOOL LETT, V5, DOI 10.1186/s40851-019-0148-9
Vincent M, 2012, BIOESSAYS, V34, P72, DOI 10.1002/bies.201100049
Vinogradov AE, 2019, BIOSYSTEMS, V181, P82, DOI 10.1016/j.biosystems.2019.05.004
Vinogradov AE, 2017, STEM CELLS INT, V2017, DOI 10.1155/2017/2362630
Vinogradov AE, 2006, NUCLEIC ACIDS RES, V34, P5906, DOI 10.1093/nar/gkl773
Vinogradov AE, 2021, INT J MOL SCI, V22, DOI 10.3390/ijms222111640
Vinogradov AE, 2020, FEBS J, V287, P4427, DOI 10.1111/febs.15257
Willnow D, 2021, NATURE, V597, P87, DOI 10.1038/s41586-021-03844-1
Xing QR, 2020, GENOME RES, V30, P1027, DOI 10.1101/gr.257840.119
Yan LY, 2013, NAT STRUCT MOL BIOL, V20, P1131, DOI 10.1038/nsmb.2660
Zhao M, 2016, NUCLEIC ACIDS RES, V44, pD1023, DOI 10.1093/nar/gkv1268
NR 61
TC 4
Z9 4
U1 0
U2 1
PU MDPI
PI BASEL
PA MDPI AG, Grosspeteranlage 5, CH-4052 BASEL, SWITZERLAND
SN 1661-6596
EI 1422-0067
J9 INT J MOL SCI
JI Int. J. Mol. Sci.
PD OCT

PY 2022
VL 23
IS 19
AR 11486
DI 10.3390/ijms231911486
PG 17
WC Biochemistry & Molecular Biology; Chemistry, Multidisciplinary
WE Science Citation Index Expanded (SCI-EXPANDED)
SC Biochemistry & Molecular Biology; Chemistry
GA 5H6PZ
UT WOS:000867800400001
PM 36232785
OA Green Published, gold
DA 2025-05-07
ER

PT J
AU Eppenberger, PE
Cavka, M
Habicht, ME
Galassi, FM
Rühli, F
AF Eppenberger, Patrick E.
Cavka, Mislav
Habicht, Michael E.
Galassi, Francesco M.
Ruehli, Frank
TI Radiological findings in ancient Egyptian canopic jars: comparing three
standard clinical imaging modalities (x-rays, CT and MRI)
SO EUROPEAN RADIOLOGY EXPERIMENTAL
LA English
DT Article
DE Ancient Egyptian canopic jars; Computed tomography; Magnetic resonance imaging; Radiography; Paleoradiology
ID TECHNICAL NOTE; MUMMIES; DIFFERENTIATION
AB Background The aim of our study was to evaluate the potential and the limitations of standard clinical imaging modalities for the examination of ancient Egyptian canopic jars and the mummified visceral organs (putatively) contained within them. Methods A series of four ancient Egyptian canopic jars was imaged comparing the three standard clinical imaging modalities: x-rays, computed tomography (CT) and magnetic resonance imaging (MRI). Additionally, imaging-data-based volumetric calculations were performed for quantitative assessment of the jar contents. Results The image contrast of the x-ray images was limited by the thickness and high density of the calcite mineral constituting the examined jars. CT scans showed few artefacts and revealed hyperdense structures of organ-specific morphology, surrounded by a hypodense homogeneous material. The image quality of MRI scans was limited by the low amount of water present in the desiccated jar contents. Nevertheless, areas of pronounced signal intensity coincided well with hyperdense structures previously identified on CT scans. CT-based volumetric calculations revealed holding capacities of the jars of 626-1319 cm(3) and content volumes of 206-1035 cm(3). Conclusions CT is the modality of

choice for non-invasive examination of ancient Egyptian canopic jars. However, despite its limitations, x-ray imaging will often remain the only practicable method for on-site investigations. Overall, the presented radiological findings are more compatible with contained small organ fragments rather than entire mummified organs, as originally expected, with consequent implications for envisioned future sampling for chemical and genetic analysis.
C1 [Eppenberger, Patrick E.; Habicht, Michael E.; Galassi, Francesco M.; Ruehli, Frank] Univ Zurich, Inst Evolutionary Med, Winterthurerstr 190, CH-8057 Zurich, Switzerland.
[Cavka, Mislav] Univ Zagreb, Sch Med, Salata 3, Zagreb 10000, Croatia.
[Habicht, Michael E.] Flinders Univ S Australia, Sch Humanities & Creat Art, Dept Archeol, Sturt Rd, Bedford Pk, SA 5042, Australia.
C3 University of Zurich; University of Zagreb; Flinders University South Australia
RP Eppenberger, PE (autor correspondente), Univ Zurich, Inst Evolutionary Med, Winterthurerstr 190, CH-8057 Zurich, Switzerland.
EM patrick.eppenberger@iem.uzh.ch
RI Habicht, Michael/AAF-4033-2020; Ruhli, Frank/H-8789-2017
OI Eppenberger, Patrick/0000-0002-9526-8709; Galassi, Francesco Maria/0000-0001-8902-3142; Ruhli, Frank/0000-0001-7937-001X; Habicht, Michael/0000-0002-0193-9672
FU Canopic Jar Project at the Institute of Evolutionary Medicine, University of Zurich - Swiss National Science Foundation; Maxi Foundation [162803]
FX This study is part of the Canopic Jar Project at the Institute of Evolutionary Medicine, University of Zurich, and is funded by the Swiss
National Science Foundation and the Maxi Foundation under grant number 162803.
CR Allam AH, 2011, JACC-CARDIOVASC IMAG, V4, P315, DOI 10.1016/j.jcmg.2011.02.002
Bolliger SA, 2009, J FORENSIC SCI, V54, P1119, DOI 10.1111/j.1556-4029.2009.01100.x
Cavka M, 2012, RADIOGRAPHICS, V32, P2151, DOI 10.1148/rg.327115744
Cavka M, 2010, COLLEGIUM ANTROPOL, V34, P797
Cox SL, 2015, ANAT REC, V298, P1099, DOI 10.1002/ar.23149
David R, 2003, HDB LIFE ANCIENT EGY, pS417
Dodson A, 2008, TOMB ANCIENT EGYPT R, P368
Dodson A., 1994, STUDIES EGYPTOLOGY
Galassi FM, 2017, CIPEG J ANCIENT EGYP, DOI [10.11588/cipeg.2017.1.44165, DOI 10.11588/CIPEG.2017.1.44165]
Ikram S., 1998, MUMMY ANCIENT EGYPT, P352
Koenig W, 1896, 14 PHOTOGRAPHIEN ROE
Lewin PK., 1977, PALEOPATHOL NEWSL, P17
Öhrström L, 2010, AM J PHYS ANTHROPOL, V142, P497, DOI 10.1002/ajpa.21292
Ruder TD, 2013, FORENSIC SCI MED PAT, V9, P163, DOI 10.1007/s12024-012-9398-y
Ruder TD, 2012, FORENSIC SCI INT, V220, P219, DOI 10.1016/j.forsciint.2012.03.004
Rühli FJ, 2015, ANAT REC, V298, P1111, DOI 10.1002/ar.23150
Rühli FJ, 2004, CAN ASSOC RADIOL J, V55, P218
Rühli FJ, 2002, AM J PHYS ANTHROPOL, V117, P272, DOI 10.1002/ajpa.2003

   Rühli FJ, 2007, JAMA-J AM MED ASSOC, V298, P2618, DOI
10.1001/jama.298.22.2618-b
   Rühli FJ, 2016, CLIN ANAT, V29, P816, DOI 10.1002/ca.22709
   Saab G., 2008, Paleoradiology: Imaging Mummies and Fossils pp, P15
   Universitat Zurich Anatomisches Institut, 2011, MUM MENSCH MED MAG A,
P141
   Uranic I, 2007, CATALOGUE MONOGRAPHS
   Wade AD, 2011, HOMO, V62, P248, DOI 10.1016/j.jchb.2011.01.004
   Weissleder R, 2007, PRIMER DIAGNOSTIC IM
NR 25
TC 11
Z9 11
U1 0
U2 18
PU SPRINGERNATURE
PI LONDON
PA CAMPUS, 4 CRINAN ST, LONDON, N1 9XW, ENGLAND
EI 2509-9280
J9 EUR RADIOL EXP
JI Eur. Radiol. Exp.
PD JUN 20
PY 2018
VL 2
IS 1
AR 12
DI 10.1186/s41747-018-0048-3
PG 9
WC Radiology, Nuclear Medicine & Medical Imaging
WE Emerging Sources Citation Index (ESCI)
SC Radiology, Nuclear Medicine & Medical Imaging
GA VK0UD
UT WOS:000656325300001
PM 29951641
OA gold, Green Published
DA 2025-05-07
ER

PT J
AU Kuron, D
   Pohlmann, A
   Angenendt, L
   Kessler, T
   Mesters, R
   Berdel, WE
   Stelljes, M
   Lenz, G
   Schliemann, C
   Mikesch, JH
AF Kuron, David
   Pohlmann, Alexander
   Angenendt, Linus
   Kessler, Torsten
   Mesters, Rolf
   Berdel, Wolfgang E. E.

Stelljes, Matthias
Lenz, Georg
Schliemann, Christoph
Mikesch, Jan-Henrik
TI Amsacrine-based induction therapy in AML patients with cardiac comorbidities: a retrospective single-center analysis
SO ANNALS OF HEMATOLOGY
LA English
DT Article
DE Acute myeloid leukemia; AML; Cardiac comorbidities; Induction therapy; Amsacrine
ID ACUTE MYELOID-LEUKEMIA; OLDER PATIENTS; DAUNORUBICIN; CYTARABINE; VENETOCLAX; CHEMOTHERAPY; FLUDARABINE; COMBINATION; DYSFUNCTION; THIOGUANINE
AB Intensive chemotherapy is the backbone of induction treatment in patients with acute myeloid leukemia (AML). However, AML patients with concomitant cardiac disease may not be eligible for anthracycline-based therapies. In a small cohort of patients, we have previously shown that anthracycline-free, amsacrine-based chemotherapy TAA (thioguanine, cytarabine, amsacrine) may be as effective as cytarabine/daunorubicin for induction therapy in these patients. In this systematic retrospective single-center analysis, we documented the outcome of 31 patients with significant cardiac comorbidities including coronary heart disease or cardiomyopathy receiving TAA as induction chemotherapy. Median (range) ejection fraction (EF) was 48% (30-67%) in this cohort. Patients with EF below 30% were considered unfit for intensive induction therapy. Event-free survival (EFS), overall survival (OS), and relapse-free survival (RFS) were 1.61, 5.46, and 13.6 months respectively. Poor outcome was primarily related to a high early mortality rate within the first 30 days of therapy, mainly caused by infectious complications. TAA cannot be recommended as a substitute of standard induction for AML patients with significant concomitant cardiac disease. In the era of novel agents, alternative strategies (e.g., hypomethylating agents plus venetoclax) should be considered when anthracycline-based regimens are not suitable.
C1 [Kuron, David; Pohlmann, Alexander; Angenendt, Linus; Kessler, Torsten; Mesters, Rolf; Berdel, Wolfgang E. E.; Stelljes, Matthias; Lenz, Georg; Schliemann, Christoph; Mikesch, Jan-Henrik] Univ Hosp Munster, Dept Med A, D-48149 Munster, Germany.
[Kuron, David] Univ Hosp Schleswig Holstein, Dept Med 2, Arnold Heller Str 3, D-24105 Kiel, Germany.
C3 University of Munster; University of Kiel; Schleswig Holstein University
Hospital
RP Kuron, D (autor correspondente), Univ Hosp Munster, Dept Med A, D-48149 Munster, Germany.; Kuron, D (autor correspondente), Univ Hosp Schleswig Holstein, Dept Med 2, Arnold Heller Str 3, D-24105 Kiel, Germany.
EM David.kuron@uksh.de
RI A, L/K-2711-2017
OI Baden, David/0000-0002-4519-1002
FU Projekt DEAL; Deutsche Forschungsgemeinschaft (DFG, German Research Foundation) [413490537]; Kiel Clinician Scientist Program in Evolutionary Medicine (CSEM)
FX Open Access funding enabled and organized by Projekt DEAL. David Kuron

is funded by the Deutsche Forschungsgemeinschaft (DFG, German Research Foundation) - project number 413490537 within the Kiel Clinician Scientist Program in Evolutionary Medicine (CSEM).
CR Appelbaum FR, 2006, BLOOD, V107, P3481, DOI 10.1182/blood-2005-09-3724
ARLIN ZA, 1991, CANCER, V68, P1198, DOI 10.1002/1097-0142(19910915)68:6<1198::AID-CNCR2820680603>3.0.CO;2-E
Arslan S, 2020, BLOOD, V136, DOI 10.1182/blood-2020-142780
Bashey A, 2006, LEUKEMIA RES, V30, P503, DOI 10.1016/j.leukres.2005.09.002
BERMAN E, 1989, LEUKEMIA, V3, P115
Bosch X, 2013, J AM COLL CARDIOL, V61, P2355, DOI 10.1016/j.jacc.2013.02.072
Brandwein JM, 2013, AM J BLOOD RES, V3, P141
Burnett AK, 2015, BLOOD, V125, P3878, DOI 10.1182/blood-2015-01-623447
Cortes JE, 2020, J HEMATOL ONCOL, V13, DOI 10.1186/s13045-020-00929-8
Cortes JE, 2019, LEUKEMIA, V33, P379, DOI 10.1038/s41375-018-0312-9
Dhopeshwarkar N, 2019, CL LYMPH MYELOM LEUK, V19, pE436, DOI 10.1016/j.clml.2019.04.012
DiNardo CD, 2020, NEW ENGL J MED, V383, P617, DOI 10.1056/NEJMoa2012971
DiNardo CD, 2021, J CLIN ONCOL, V39, P57, DOI 10.1200/JCO.20.01632
DiNardo CD, 2019, BLOOD, V134, DOI 10.1182/blood-2019-130362
DiNardo CD, 2020, BLOOD, V135, P85, DOI 10.1182/blood.2019001239
DiNardo CD, 2019, BLOOD, V133, P7, DOI 10.1182/blood-2018-08-868752
Döhner H, 2017, BLOOD, V129, P424, DOI 10.1182/blood-2016-08-733196
Dohner H, 2021, NAT REV CLIN ONCOL, V18, P577, DOI 10.1038/s41571-021-00509-w
Fong CY, 2013, LEUKEMIA LYMPHOMA, V54, P336, DOI 10.3109/10428194.2012.713479
Georgakopoulos P, 2010, AM J HEMATOL, V85, P894, DOI 10.1002/ajh.21840
Hahn A, 2015, LEUKEMIA RES, V39, P505, DOI 10.1016/j.leukres.2015.02.003
Harkness A, 2020, ECHO RES PRACT, V7, pG1, DOI 10.1530/ERP-19-0050
Heuser M, 2021, ANN HEMATOL, V100, P1181, DOI 10.1007/s00277-021-04465-4
Johnson IM, 2022, BLOOD ADV, V6, P5227, DOI 10.1182/bloodadvances.2022007333
Juliusson G, 2009, BLOOD, V113, P4179, DOI 10.1182/blood-2008-07-172007
Kennedy VE, 2020, BLOOD, V136, DOI 10.1182/blood-2020-143002
Kessler T, 2008, LEUKEMIA RES, V32, P491, DOI 10.1016/j.leukres.2007.06.015
LINKESCH W, 1989, ONKOLOGIE, V12, P8, DOI 10.1159/000216587
Liu CJ, 2020, CANCER MED-US, V9, P1572, DOI 10.1002/cam4.2740
Löwenberg B, 2009, NEW ENGL J MED, V361, P1235, DOI 10.1056/NEJMoa0901409
Ofran Y, 2016, BLOOD, V128, P488, DOI 10.1182/blood-2016-01-635060
Ostgård LSG, 2015, LEUKEMIA, V29, P548, DOI 10.1038/leu.2014.234
Perl AE, 2019, NEW ENGL J MED, V381, P1728, DOI 10.1056/NEJMoa1902688
Pfrepper C, 2016, J CANCER RES CLIN, V142, P317, DOI 10.1007/s00432-015-2050-y
Pollyea DA, 2021, AM J HEMATOL, V96, P208, DOI 10.1002/ajh.26039
Sandhu KS, 2020, BIOL BLOOD MARROW TR, V26, pE322, DOI 10.1016/j.bbmt.2020.08.027

Shallis RM, 2019, BLOOD REV, V36, P70, DOI 10.1016/j.blre.2019.04.005
Solomon SR, 2020, BONE MARROW TRANSPL, V55, DOI 10.1038/s41409-019-0675-1
Stein EM, 2021, BLOOD, V137, P1792, DOI 10.1182/blood.2020007233
van Dalen EC, 2016, COCHRANE DB SYST REV, DOI 10.1002/14651858.CD005008.pub4
Wei AH, 2019, J CLIN ONCOL, V37, P1277, DOI 10.1200/JCO.18.01600
Yin J, 2019, BLOOD ADV, V3, P1714, DOI 10.1182/bloodadvances.2018026112
Zeidan AM, 2019, BLOOD, V134, DOI 10.1182/blood-2019-124034
NR 43
TC 3
Z9 3
U1 4
U2 7
PU SPRINGER
PI NEW YORK
PA ONE NEW YORK PLAZA, SUITE 4600, NEW YORK, NY, UNITED STATES
SN 0939-5555
EI 1432-0584
J9 ANN HEMATOL
JI Ann. Hematol.
PD APR
PY 2023
VL 102
IS 4
BP 755
EP 760
DI 10.1007/s00277-023-05111-x
EA FEB 2023
PG 6
WC Hematology
WE Science Citation Index Expanded (SCI-EXPANDED)
SC Hematology
GA 9T0YY
UT WOS:000934241800004
PM 36749402
OA Green Published, hybrid
DA 2025-05-07
ER

PT J
AU Tobler, R
Souilmi, Y
Huber, CD
Bean, N
Turney, CSM
Grey, ST
Cooper, A
AF Tobler, Raymond
Souilmi, Yassine
Huber, Christian D.
Bean, Nigel
Turney, Chris S. M.

Grey, Shane T.
Cooper, Alan
TI The role of genetic selection and climatic factors in the dispersal of anatomically modern humans out of Africa
SO PROCEEDINGS OF THE NATIONAL ACADEMY OF SCIENCES OF THE UNITED STATES OF
AMERICA
LA English
DT Article
DE adaptation; ancient DNA; hard sweeps; human migrations
ID SOFT SWEEPS; GRISCELLI-SYNDROME; HUMAN ADAPTATION; GENOMIC HISTORY; SIGNATURES; MUTATIONS; PIGMENTATION; POPULATIONS; SPECTRUM; DELETION
AB The evolutionarily recent dispersal of anatomically modern humans (AMH) out of Africa (OoA) and across Eurasia provides a unique opportunity to examine the impacts of genetic selection as humans adapted to multiple new environments. Analysis of ancient Eurasian genomic datasets (similar to 1,000 to 45,000 y old) reveals sig-natures of strong selection, including at least 57 hard sweeps after the initial AMH movement OoA, which have been obscured in modern populations by extensive admixture during the Holocene. The spatiotemporal patterns of these hard sweeps provide a means to reconstruct early AMH population dispersals OoA. We identify a previously unsuspected extended period of genetic adaptation lasting similar to 30,000 y, potentially in the Arabian Peninsula area, prior to a major Neandertal genetic intro-gression and subsequent rapid dispersal across Eurasia as far as Australia. Consistent functional targets of selection initiated during this period, which we term the Arabian Standstill, include loci involved in the regulation of fat storage, neural development, skin physiology, and cilia function. Similar adaptive signatures are also evident in introgressed archaic hominin loci and modern Arctic human groups, and we suggest that this signal represents selection for cold adaptation. Surprisingly, many of the candidate selected loci across these groups appear to directly interact and coordinately regulate biological processes, with a number associated with major modern diseases including the ciliopathies, metabolic syndrome, and neurodegenerative disorders. This expands the potential for ancestral human adaptation to directly impact modern diseases, providing a platform for evolutionary medicine.
C1 [Tobler, Raymond] Australian Natl Univ, Coll Asia & Pacific, Evolut Cultural Divers Initiat, Canberra, ACT 2600, Australia.
[Huber, Christian D.] Penn State Univ, Dept Biol, University Pk, PA 16802 USA.
[Tobler, Raymond; Souilmi, Yassine; Huber, Christian D.; Cooper, Alan] Univ Adelaide, Australian Ctr Ancient DNA, Adelaide, SA 5005, Australia.
[Souilmi, Yassine; Cooper, Alan] Univ Adelaide, Environm Inst, Adelaide, SA 5005, Australia.
[Bean, Nigel] Univ Adelaide, Australian Res Council Ctr Excellence Math & Stat, Adelaide, SA 5005, Australia.
[Bean, Nigel] Univ Adelaide, Sch Math Sci, Adelaide, SA 5005, Australia.
[Huber, Christian D.] Univ Technol Sydney, Div Res, Ultimo, NSW 2007, Australia.
[Grey, Shane T.] Univ New South Wales, Fac Sci, Sch Biotechnol & Biomol Sci, Sydney, NSW 2052, Australia.

[Grey, Shane T.] Garvan Inst Med Res, Transplantat Immunol Grp, Translat Sci Pillar, Darlinghurst, NSW 2010, Australia.
[Cooper, Alan] Blue Sky Genet, Ashton, SA 5137, Australia.
C3 Australian National University; Pennsylvania Commonwealth System of Higher Education (PCSHE); Pennsylvania State University; Penn State Behrend; Pennsylvania State University - University Park; University of Adelaide; University of Adelaide; University of Adelaide; University of Adelaide; University of Technology Sydney; University of New South Wales Sydney; Garvan Institute of Medical Research
RP Tobler, R; Souilmi, Y; Cooper, A (autor correspondente), Univ Adelaide, Australian Ctr Ancient DNA, Adelaide, SA 5005, Australia.; Souilmi, Y; Cooper, A (autor correspondente), Univ Adelaide, Environm Inst, Adelaide, SA 5005, Australia.; Grey, ST (autor correspondente), Univ New South Wales, Fac Sci, Sch Biotechnol & Biomol Sci, Sydney, NSW 2052, Australia.; Grey, ST (autor correspondente), Garvan Inst Med Res, Transplantat Immunol Grp, Translat Sci Pillar, Darlinghurst, NSW 2010, Australia.; Cooper, A (autor correspondente), Blue Sky Genet, Ashton, SA 5137, Australia.
EM ray.tobler@anu.edu.au; yassine.souilmi@adelaide.edu.au; s.grey@garvan.org.au; alanjcooper42@gmail.com
RI Cooper, Alan/E-8171-2012; Tobler, Ray/AAE-6745-2020; Huber, Christian/GRF-1881-2022; Turney, Chris/P-8701-2018; Grey, Shane/B-3020-2008; Bean, Nigel/I-2261-2014
OI Grey, Shane/0000-0003-2160-1625; Tobler, Raymond/0000-0002-4603-1473; Cooper, Alan/0000-0002-7738-7851; Souilmi, Yassine/0000-0001-7543-4864; Bean, Nigel/0000-0002-5351-3104
FU ARC [DE190101069, DP190103705, FL140100260, DE180100883, CE140100049]; NHMRC [GNT1189235, GNT1140691]; Australian Research Council [DE180100883, DE190101069] Funding Source: Australian Research Council
FX The use of the term "Arabian Gulf" rather than the alternative "Persian Gulf" follows recent studies and does not reflect the political view of any author. We thank Murray Cox, Angad Johar, Jeremy Austin, Chris Auricht, and many colleagues who have provided valuable support and suggestions; This project was supported by the ARC; R.T. (DE190101069) , Y.S. (DP190103705) , A.C. (FL140100260) , C.D.H. (DE180100883) , N.B. (CE140100049) , and NHMRC; S.T.G. (GNT1189235; GNT1140691) .
CR Akbari A, 2018, NAT METHODS, V15, P279, DOI [10.1038/nmeth.4606, 10.1038/NMETH.4606]
Aksu G, 2003, AM J MED GENET A, V116A, P329, DOI 10.1002/ajmg.a.10836
Ambrose S. H., 2017, Modern Human Origins and Dispersal, P171
Armitage SJ, 2011, SCIENCE, V331, P453, DOI 10.1126/science.1199113
Banks WE, 2019, QUATERNARY SCI REV, V220, P188, DOI 10.1016/j.quascirev.2019.07.025
Barak Y, 1999, MOL CELL, V4, P585, DOI 10.1016/S1097-2765(00)80209-9
Benton ML, 2021, NAT REV GENET, V22, P269, DOI 10.1038/s41576-020-00305-9
Bergström A, 2021, NATURE, V590, P229, DOI 10.1038/s41586-021-03244-5

Bergström A, 2020, SCIENCE, V367, P1339, DOI 10.1126/science.aay5012
Bersaglieri T, 2004, AM J HUM GENET, V74, P1111, DOI 10.1086/421051
Beyer RM, 2021, NAT COMMUN, V12, DOI 10.1038/s41467-021-24779-1
Canty JT, 2021, ANNU REV BIOPHYS, V50, P549, DOI 10.1146/annurev-biophys-111020-101511
Cardona A, 2014, PLOS ONE, V9, DOI 10.1371/journal.pone.0098076
Clarkson C, 2020, NAT COMMUN, V11, DOI 10.1038/s41467-020-14668-4
Cooper A, 2021, SCIENCE, V371, P811, DOI 10.1126/science.abb8677
Corbetta M, 2008, NEURON, V58, P306, DOI 10.1016/j.neuron.2008.04.017
Crawford NG, 2017, SCIENCE, V358, P887, DOI 10.1126/science.aan8433
Enard D, 2018, CELL, V175, P360, DOI 10.1016/j.cell.2018.08.034
Evans RM, 2004, NAT MED, V10, P355, DOI 10.1038/nm1025
Fang L, 2016, PPAR RES, V2016, DOI 10.1155/2016/6042162
Fernandes DM, 2018, SCI REP-UK, V8, DOI 10.1038/s41598-018-33067-w
Ferreira JC, 2021, GENOME BIOL EVOL, V13, DOI 10.1093/gbe/evab194
Fritzius T, 2008, EMBO J, V27, P1399, DOI 10.1038/emboj.2008.67
Fu QM, 2016, NATURE, V534, P200, DOI 10.1038/nature17993
Fumagalli M, 2015, SCIENCE, V349, P1343, DOI 10.1126/science.aab2319
Gittelman RM, 2016, CURR BIOL, V26, P3375, DOI 10.1016/j.cub.2016.10.041
Gower G, 2021, ELIFE, V10, DOI 10.7554/eLife.64669
Greenbaum G, 2019, NAT COMMUN, V10, DOI 10.1038/s41467-019-12862-7
Groucutt HS, 2021, NATURE, V597, P376, DOI [10.1038/s41586-021-03863-y, 10.1038/s41586-021-04289-2]
Günther T, 2015, P NATL ACAD SCI USA, V112, P11917, DOI 10.1073/pnas.1509851112
Haak W, 2015, NATURE, V522, P207, DOI 10.1038/nature14317
Hajdinjak M, 2021, NATURE, V592, P253, DOI 10.1038/s41586-021-03335-3
Hamblin MT, 2002, AM J HUM GENET, V70, P369, DOI 10.1086/338628
Hebert LE, 2013, NEUROLOGY, V80, P1778, DOI 10.1212/WNL.0b013e31828726f5
Hermisson J, 2005, GENETICS, V169, P2335, DOI 10.1534/genetics.104.036947
Hernandez RD, 2011, SCIENCE, V331, P920, DOI 10.1126/science.1198878
Higham T, 2011, J HUM EVOL, V61, P549, DOI 10.1016/j.jhevol.2011.06.005
Huber CD, 2016, MOL ECOL, V25, P142, DOI 10.1111/mec.13351
Hublin JJ, 2020, NATURE, V581, P299, DOI 10.1038/s41586-020-2259-z
Hume AN, 2007, J CELL SCI, V120, P3111, DOI 10.1242/jcs.010207
Ishida Y, 2006, GENOMICS, V88, P698, DOI 10.1016/j.ygeno.2006.06.006
Jablonski NG, 2010, P NATL ACAD SCI USA, V107, P8962, DOI 10.1073/pnas.0914628107
Karczewski KJ, 2020, NATURE, V581, P434, DOI 10.1038/s41586-020-2308-7
Khrunin AV, 2020, PLOS ONE, V15, DOI 10.1371/journal.pone.0228778
Kratochwil CF, 2019, PIGM CELL MELANOMA R, V32, P719, DOI 10.1111/pcmr.12799
Lamason RL, 2005, SCIENCE, V310, P1782, DOI 10.1126/science.1116238
Lazaridis I, 2018, CURR OPIN GENET DEV, V53, P21, DOI 10.1016/j.gde.2018.06.007
Lazaridis I, 2014, NATURE, V513, P409, DOI 10.1038/nature13673
Lee H, 2014, J MED FOOD, V17, P189, DOI 10.1089/jmf.2013.2917
LEE ST, 1994, NEW ENGL J MED, V330, P529, DOI 10.1056/NEJM199402243300803
Lesko SL, 2020, DEV GROWTH DIFFER, V62, P423, DOI 10.1111/dgd.12669

Lipson M, 2017, MOL BIOL EVOL, V34, P889, DOI 10.1093/molbev/msw293
Ma HR, 2019, GENES-BASEL, V10, DOI 10.3390/genes10040254
Mäkinen TM, 2007, AM J HUM BIOL, V19, P155, DOI 10.1002/ajhb.20627
Mathieson I, 2020, CURR OPIN GENET DEV, V62, P97, DOI 10.1016/j.gde.2020.06.003
Mathieson I, 2018, NATURE, V555, P197, DOI 10.1038/nature25778
Ménasché G, 2003, J CLIN INVEST, V112, P450, DOI 10.1172/JCI200318264
Milla CE, 2016, CURR OPIN PEDIATR, V28, P339, DOI 10.1097/MOP.0000000000000358
Miller TEX, 2020, ECOLOGY, V101, DOI 10.1002/ecy.3139
Mittnik A, 2018, NAT COMMUN, V9, DOI 10.1038/s41467-018-02825-9
O'Connell JF, 2018, P NATL ACAD SCI USA, V115, P8482, DOI 10.1073/pnas.1808385115
Ohbayashi Norihiko, 2020, F1000Res, V9, DOI 10.12688/f1000research.24625.1
Oike Y, 2005, NAT MED, V11, P400, DOI 10.1038/nm1214
Olalde I, 2019, SCIENCE, V363, P1230, DOI 10.1126/science.aav4040
Peter B., 2020, bioRxiv, DOI [DOI 10.1101/2020.03.13.990523, DOI 10.1101/2020.03.13.990523V1]
Petraglia M D., 2019, Geological Setting, Palaeoenvironment and Archaeology of the Red Sea, P675, DOI DOI 10.1007/978-3-319-99408-630
Pritchard JK, 2010, CURR BIOL, V20, pR208, DOI 10.1016/j.cub.2009.11.055
Rasmussen SO, 2014, QUATERNARY SCI REV, V106, P14, DOI 10.1016/j.quascirev.2014.09.007
Reiter JF, 2017, NAT REV MOL CELL BIO, V18, P533, DOI 10.1038/nrm.2017.60
Reynolds AW, 2019, P NATL ACAD SCI USA, V116, P9312, DOI 10.1073/pnas.1819467116
Rose J. I, 2022, INTRO HUMAN PREHISTO
Saitoh Y, 2019, HISTOCHEM CELL BIOL, V151, P385, DOI 10.1007/s00418-018-1745-y
Saklayen MG, 2018, CURR HYPERTENS REP, V20, DOI 10.1007/s11906-018-0812-z
Schrider DR, 2017, MOL BIOL EVOL, V34, P1863, DOI 10.1093/molbev/msx154
Setter D, 2020, PLOS GENET, V16, DOI 10.1371/journal.pgen.1008867
Shen JJ, 1996, J CLIN INVEST, V98, P352, DOI 10.1172/JCI118799
Shoaee MJ, 2021, J ANTHROPOL ARCHAEOL, V62, DOI 10.1016/j.jaa.2021.101292
Skoglund P, 2018, ANNU REV GENOM HUM G, V19, P381, DOI 10.1146/annurev-genom-083117-021749
Slimak L, 2022, SCI ADV, V8, DOI 10.1126/sciadv.abj9496
Souilmi Y, 2022, NAT ECOL EVOL, V6, P2003, DOI 10.1038/s41559-022-01914-9
Stewart M, 2020, SCI ADV, V6, DOI 10.1126/sciadv.aba8940
Stubbs JL, 2012, NAT CELL BIOL, V14, P140, DOI 10.1038/ncb2406
Sun XF, 2021, P NATL ACAD SCI USA, V118, DOI 10.1073/pnas.2019158118
Szklarczyk D, 2021, NUCLEIC ACIDS RES, V49, pD605, DOI 10.1093/nar/gkaa1074
Szpak M, 2019, FEBS LETT, V593, P1431, DOI 10.1002/1873-3468.13447
Szucs M, 2017, P NATL ACAD SCI USA, V114, P13501, DOI 10.1073/pnas.1712934114
Tan CH, 2016, NATURE, V536, P460, DOI 10.1038/nature19074

   Teixeira JC, 2019, P NATL ACAD SCI USA, V116, P15327, DOI 10.1073/pnas.1904824116
   Tierney JE, 2017, GEOLOGY, V45, P1023, DOI 10.1130/G39457.1
   Tobler R., SUPPLEMENTAL DATASET, DOI [10.25909/22359865, DOI 10.25909/22359865]
   Tobler R., SUPPLEMENTAL DATASET, DOI [10.25909/22359874, DOI 10.25909/22359874]
   Williams MAJ, 2009, PALAEOGEOGR PALAEOCL, V284, P295, DOI 10.1016/j.palaeo.2009.10.009
   Yang MA, 2022, Hum Popul Genet Genomics, V2, DOI [10.47248/hpgg2202010001, DOI 10.47248/HPGG2202010001]
   Yoneyama S, 2014, HUM MOL GENET, V23, P2498, DOI 10.1093/hmg/ddt626
   Yousaf S, 2020, PIGM CELL MELANOMA R, V33, P556, DOI 10.1111/pcmr.12879
   Yudin NS, 2017, BMC GENET, V18, DOI 10.1186/s12863-017-0580-9
   Zammit NW, 2019, NAT IMMUNOL, V20, P1299, DOI 10.1038/s41590-019-0492-0
   Zhang L, 2015, CYTOSKELETON, V72, P171, DOI 10.1002/cm.21214
   Zhou SR, 2019, P NATL ACAD SCI USA, V116, P16012, DOI 10.1073/pnas.1810388116
NR 99
TC 8
Z9 8
U1 1
U2 15
PU NATL ACAD SCIENCES
PI WASHINGTON
PA 2101 CONSTITUTION AVE NW, WASHINGTON, DC 20418 USA
SN 0027-8424
EI 1091-6490
J9 P NATL ACAD SCI USA
JI Proc. Natl. Acad. Sci. U. S. A.
PD MAY 30
PY 2023
VL 120
IS 22
AR e2213061120
DI 10.1073/pnas.2213061120
PG 12
WC Multidisciplinary Sciences
WE Science Citation Index Expanded (SCI-EXPANDED)
SC Science & Technology - Other Topics
GA N8PL8
UT WOS:001039568200005
PM 37220274
OA hybrid
DA 2025-05-07
ER

PT J
AU Vinogradov, AE
   Anatskaya, OV
AF Vinogradov, Alexander E.
   Anatskaya, Olga, V

TI Growth of Biological Complexity from Prokaryotes to Hominids Reflected
   in the Human Genome
SO INTERNATIONAL JOURNAL OF MOLECULAR SCIENCES
LA English
DT Article
DE evolution of complexity; gene phylostratigraphy; transcription factors;
   epigenetic factors; nervous system; cancer; diseases; aging
ID TRANSCRIPTION FACTORS; EVOLUTION; EXPRESSION; CANCER; COMMON; CELLS; DNA
AB The growth of complexity in evolution is a most intriguing phenomenon. Using gene phylostratigraphy, we showed this growth (as reflected in regulatory mechanisms) in the human genome, tracing the path from prokaryotes to hominids. Generally, the different regulatory gene families expanded at different times, yet only up to the Euteleostomi (bony vertebrates). The only exception was the expansion of transcription factors (TF) in placentals; however, we argue that this was not related to increase in general complexity. Surprisingly, although TF originated in the Prokaryota while chromatin appeared only in the Eukaryota, the expansion of epigenetic factors predated the expansion of TF. Signaling receptors, tumor suppressors, oncogenes, and aging- and disease-associated genes (indicating vulnerabilities in terms of complex organization and strongly enrichment in regulatory genes) also expanded only up to the Euteleostomi. The complexity-related gene properties (protein size, number of alternative splicing mRNA, length of untranslated mRNA, number of biological processes per gene, number of disordered regions in a protein, and density of TF-TF interactions) rose in multicellular organisms and declined after the Euteleostomi, and possibly earlier. At the same time, the speed of protein sequence evolution sharply increased in the genes that originated after the Euteleostomi. Thus, several lines of evidence indicate that molecular mechanisms of complexity growth were changing with time, and in the phyletic lineage leading to humans, the most salient shift occurred after the basic vertebrate body plan was fixed with bony skeleton. The obtained results can be useful for evolutionary medicine.
C1 [Vinogradov, Alexander E.; Anatskaya, Olga, V] Russian Acad Sci, Inst Cytol, St Petersburg 194064, Russia.
C3 Russian Academy of Sciences; St. Petersburg Scientific Centre of the
   Russian Academy of Sciences; Institute of Cytology RAS
RP Vinogradov, AE (autor correspondente), Russian Acad Sci, Inst Cytol, St Petersburg 194064, Russia.
EM aevin@incras.ru; anatskaya@incras.ru
RI Vinogradov, Alexander/R-3923-2016; Anatskaya, Olga/R-4042-2016
FU Ministry of Science and Higher Education of the Russian Federation
   [075-15-2021-1075]
FX Funding This research was funded by the Ministry of Science and Higher
   Education of the Russian Federation (Agreement No. 075-15-2021-1075,
   signed 28 September 2021). No conflicts of interest are declared.
CR Altenhoff AM, 2021, NUCLEIC ACIDS RES, V49, pD373, DOI 10.1093/nar/gkaa1007
   Amin S, 2020, FRONT CELL DEV BIOL, V8, DOI 10.3389/fcell.2020.604448
   [Anonymous], 2016, RETROGRADE EVOLUTION
   [Anonymous], 2002, The structure of evolutionary theory, DOI [10.4159/9780674417922, DOI 10.4159/9780674417922]

Assou S, 2007, STEM CELLS, V25, P961, DOI 10.1634/stemcells.2006-0352
Bains William, 2016, Life-Basel, V6, P25, DOI 10.3390/life6030025
Berg J, 2018, SCIENCE, V359, P1309, DOI 10.1126/science.aat5956
Bizzarri M, 2020, ENTROPY-SWITZ, V22, DOI 10.3390/e22080885
Bussey KJ, 2017, P NATL ACAD SCI USA, V114, P6160, DOI 10.1073/pnas.1706990114
Carbon S, 2021, NUCLEIC ACIDS RES, V49, pD325, DOI 10.1093/nar/gkaa1113
Chen CY, 2012, BIOINFORMATICS, V28, P3178, DOI 10.1093/bioinformatics/bts623
Chen L, 2014, MOL BIOL EVOL, V31, P1402, DOI 10.1093/molbev/msu083
Christenhusz Maarten J. M., 2020, Megataxa, V1, P67, DOI 10.11646/megataxa.1.1.14
Claverie JM, 2001, SCIENCE, V291, P1255, DOI 10.1126/science.1058969
Core L, 2019, GENE DEV, V33, P960, DOI 10.1101/gad.325142.119
Court F, 2017, ONCOTARGET, V8, P4110, DOI 10.18632/oncotarget.13746
Davies PCW, 2011, PHYS BIOL, V8, DOI 10.1088/1478-3975/8/1/015001
Dicke U, 2016, PHILOS T R SOC B, V371, DOI 10.1098/rstb.2015.0180
Domazet-Loso T, 2007, TRENDS GENET, V23, P533, DOI 10.1016/j.tig.2007.08.014
Drongitis D, 2019, INT J MOL SCI, V20, DOI 10.3390/ijms20225755
Enriquez-Gasca R, 2020, VIRUSES-BASEL, V12, DOI 10.3390/v12101089
Erwin DH, 2020, DEVELOPMENT, V147, DOI 10.1242/dev.182899
Fields C, 2020, PHYSIOLOGY, V35, P16, DOI 10.1152/physiol.00027.2019
Fukuda K, 2020, VIRUSES-BASEL, V12, DOI 10.3390/v12060596
Gates AJ, 2016, SCI REP-UK, V6, DOI 10.1038/srep24456
Hahn MW, 2002, EVOL DEV, V4, P73, DOI 10.1046/j.1525-142X.2002.01069.x
Howe KL, 2021, NUCLEIC ACIDS RES, V49, pD884, DOI 10.1093/nar/gkaa942
Hu H, 2019, NUCLEIC ACIDS RES, V47, pD33, DOI 10.1093/nar/gky822
Khrameeva E, 2020, GENOME RES, V30, P776, DOI 10.1101/gr.256958.119
Khyeam Sheamin, 2021, Adv Genet (Hoboken), V2, DOI 10.1002/ggn2.10042
Kindler S, 2005, ANNU REV CELL DEV BI, V21, P223, DOI 10.1146/annurev.cellbio.21.122303.120653
Kyriazis M, 2020, J MOL EVOL, V88, P57, DOI 10.1007/s00239-019-09896-2
Lambert SA, 2018, CELL, V172, P650, DOI 10.1016/j.cell.2018.01.029
Lineweaver CH, 2013, COMPLEXITY AND THE ARROW OF TIME, P1, DOI 10.1017/CBO9781139225700
Lineweaver CH, 2014, BIOESSAYS, V36, P827, DOI 10.1002/bies.201400070
Litman T, 2019, SEMIN ONCOL, V46, P3, DOI 10.1053/j.seminoncol.2018.11.002
Liu YX, 2020, INTEGR COMP BIOL, V60, P991, DOI 10.1093/icb/icaa109
Loehlin DW, 2019, P NATL ACAD SCI USA, V116, P12383, DOI 10.1073/pnas.1904071116
Löytynoja A, 2014, METHODS MOL BIOL, V1079, P155, DOI 10.1007/978-1-62703-646-7_10
Ma'ayan A, 2017, J R SOC INTERFACE, V14, DOI 10.1098/rsif.2017.0391
Machnik M, 2020, CELLS-BASEL, V9, DOI 10.3390/cells9030653
Mackeh Rafah, 2018, Nucl Recept Signal, V15, p1550762918801071, DOI 10.1177/1550762918801071
MacKintosh Carol, 2017, F1000Res, V6, P1623, DOI 10.12688/f1000research.11792.2
Martin A, 2013, EVOLUTION, V67, P1235, DOI 10.1111/evo.12081
Maupetit-Mehouas S, 2020, CELLS-BASEL, V9, DOI 10.3390/cells9051172
Medvedeva YA, 2015, DATABASE-OXFORD, DOI 10.1093/database/bav067

Müller FJ, 2008, NATURE, V455, P401, DOI 10.1038/nature07213
Nguyen PD, 2021, CELL MOL LIFE SCI, V78, P5107, DOI 10.1007/s00018-021-03831-9
Nie YM, 2020, GENOMICS, V112, P3427, DOI 10.1016/j.ygeno.2020.06.029
Nieuwkoop T, 2020, MOL CELL, V80, P193, DOI 10.1016/j.molcel.2020.09.014
Niklas KJ, 2018, J EXP BOT, V69, P1437, DOI 10.1093/jxb/erx493
Piñero J, 2020, NUCLEIC ACIDS RES, V48, pD845, DOI 10.1093/nar/gkz1021
Poelmann RE, 2019, DEV DYNAM, V248, P634, DOI 10.1002/dvdy.45
Remm M, 2001, J MOL BIOL, V314, P1041, DOI 10.1006/jmbi.2000.5197
Repana D, 2019, GENOME BIOL, V20, DOI 10.1186/s13059-018-1612-0
Sasakura Y, 2018, WIRES DEV BIOL, V7, DOI 10.1002/wdev.304
Sayers EW, 2021, NUCLEIC ACIDS RES, V49, pD10, DOI 10.1093/nar/gkaa892
Shemesh N, 2021, NAT COMMUN, V12, DOI 10.1038/s41467-021-22369-9
Singh PP, 2020, NUCLEIC ACIDS RES, V48, pD724, DOI 10.1093/nar/gkz909
Smith SA, 2017, BRIEF BIOINFORM, V18, P451, DOI 10.1093/bib/bbw034
Stearns SC, 2020, J MOL EVOL, V88, P3, DOI 10.1007/s00239-019-09893-5
Storey JD, 2003, P NATL ACAD SCI USA, V100, P9440, DOI 10.1073/pnas.1530509100
Suter DM, 2020, TRENDS CELL BIOL, V30, P491, DOI 10.1016/j.tcb.2020.03.003
Szklarczyk D, 2021, NUCLEIC ACIDS RES, V49, pD605, DOI 10.1093/nar/gkaa1074
Tacutu R, 2018, NUCLEIC ACIDS RES, V46, pD1083, DOI 10.1093/nar/gkx1042
Taft RJ, 2007, BIOESSAYS, V29, P288, DOI 10.1002/bies.20544
Talbert PB, 2019, NAT REV GENET, V20, P283, DOI 10.1038/s41576-019-0105-7
Tate JG, 2019, NUCLEIC ACIDS RES, V47, pD941, DOI 10.1093/nar/gky1015
Torres-Paz J, 2018, BIOL LETTERS, V14, DOI 10.1098/rsbl.2018.0101
Trigos AS, 2019, ELIFE, V8, DOI 10.7554/eLife.40947
Trigos AS, 2018, BRIT J CANCER, V118, P145, DOI 10.1038/bjc.2017.398
Trigos AS, 2017, P NATL ACAD SCI USA, V114, P6406, DOI 10.1073/pnas.1617743114
Uesaka M, 2022, J EXP ZOOL PART B, V338, P76, DOI 10.1002/jez.b.23027
Uhlen M, 2015, SCIENCE, V347, DOI 10.1126/science.1260419
Vallender EJ, 2019, PROG BRAIN RES, V250, P3, DOI 10.1016/bs.pbr.2019.01.003
Vincent M, 2012, BIOESSAYS, V34, P72, DOI 10.1002/bies.201100049
Vinogradov AE, 2019, BIOSYSTEMS, V181, P82, DOI 10.1016/j.biosystems.2019.05.004
Vinogradov AE, 2019, GENOMICS, V111, P115, DOI 10.1016/j.ygeno.2018.02.015
Vinogradov AE, 2005, CHROMOSOMA, V113, P362, DOI 10.1007/s00412-004-0323-3
Vinogradov AE, 2007, NUCLEIC ACIDS RES, V35, P6350, DOI 10.1093/nar/gkm723
Vinogradov AE, 2006, NUCLEIC ACIDS RES, V34, P5906, DOI 10.1093/nar/gkl773
Vinogradov AE, 2020, BIOSYSTEMS, V198, DOI 10.1016/j.biosystems.2020.104256
Vinogradov AE, 2020, FEBS J, V287, P4427, DOI 10.1111/febs.15257
Vinogradov AE, 2013, GENE, V527, P55, DOI 10.1016/j.gene.2013.05.039

   Vinogradov AE, 2010, GENOMICS, V95, P345, DOI
10.1016/j.ygeno.2010.03.004
   Yang ZH, 2007, MOL BIOL EVOL, V24, P1586, DOI 10.1093/molbev/msm088
   Yruela I, 2017, GENOME BIOL EVOL, V9, P1248, DOI 10.1093/gbe/evx073
   Zdobnov EM, 2021, NUCLEIC ACIDS RES, V49, pD389, DOI
10.1093/nar/gkaa1009
   Zhang LY, 2019, SCI REP-UK, V9, DOI 10.1038/s41598-019-40023-9
   Zhao M, 2016, NUCLEIC ACIDS RES, V44, pD1023, DOI 10.1093/nar/gkv1268
NR 90
TC 10
Z9 10
U1 0
U2 11
PU MDPI
PI BASEL
PA ST ALBAN-ANLAGE 66, CH-4052 BASEL, SWITZERLAND
EI 1422-0067
J9 INT J MOL SCI
JI Int. J. Mol. Sci.
PD NOV
PY 2021
VL 22
IS 21
AR 11640
DI 10.3390/ijms222111640
PG 25
WC Biochemistry & Molecular Biology; Chemistry, Multidisciplinary
WE Science Citation Index Expanded (SCI-EXPANDED)
SC Biochemistry & Molecular Biology; Chemistry
GA XB1XB
UT WOS:000721126200001
PM 34769071
OA gold, Green Published
DA 2025-05-07
ER

PT J
AU Kuijper, B
   Johnstone, RA
AF Kuijper, Bram
   Johnstone, Rufus A.
TI The evolution of early-life effects on social behaviour-why should
   social adversity carry over to the future?
SO PHILOSOPHICAL TRANSACTIONS OF THE ROYAL SOCIETY B-BIOLOGICAL SCIENCES
LA English
DT Article
DE predictive adaptive response; developmental plasticity; inclusive
   fitness; maternal effect; parental effect; intergenerational effect
ID INTERGENERATIONAL TRANSMISSION; DEVELOPMENTAL PLASTICITY;
   INDIVIDUAL-DIFFERENCES; KIN SELECTION; INFORMATION; MODEL;
   SPECIALIZATION; EXPERIENCES; COMPETENCE; PROMOTE
AB Numerous studies have shown that social adversity in early life can
have long-lasting consequences for social behaviour in adulthood,
consequences that may in turn be propagated to future generations. Given

these intergenerational effects, it is puzzling why natural selection might favour such sensitivity to an individual's early social environment. To address this question, we model the evolution of social sensitivity in the development of helping behaviours, showing that natural selection indeed favours individuals whose tendency to help others is dependent on early-life social experience. In organisms with non-overlapping generations, we find that natural selection can favour positive social feedbacks, in which individuals who received more help in early life are also more likely to help others in adulthood, while individuals who received no early-life help develop low tendencies to help others later in life. This positive social sensitivity is favoured because of an intergenerational relatedness feedback: patches with many helpers tend to be more productive, leading to higher relatedness within the local group, which in turn favours higher levels of help in the next generation. In organisms with overlapping generations, this positive feedback is less likely to occur, and those who received more help may instead be less likely to help others (negative social feedback). We conclude that early-life social influences can lead to strong between-individual differences in helping behaviour, which can take different forms dependent on the life history in question.
This article is part of the theme issue 'Developing differences: early-life effects and evolutionary medicine'.
C1 [Kuijper, Bram] Univ Exeter, Environm & Sustainabil Inst, Cornwall Campus, Penryn TR10 9FE, England.
[Johnstone, Rufus A.] Univ Cambridge, Dept Zool, Downing St, Cambridge CB2 3EJ, England.
C3 University of Exeter; University of Cambridge
RP Kuijper, B (autor correspondente), Univ Exeter, Environm & Sustainabil Inst, Cornwall Campus, Penryn TR10 9FE, England.
EM a.l.w.kuijper@exeter.ac.uk
RI Kuijper, Bram/E-9409-2013
OI Kuijper, Bram/0000-0002-7263-2846
FU Leverhulme Trust Early Career Research Fellowship [ECF 2015-273]
FX B.K. is funded by a Leverhulme Trust Early Career Research Fellowship (ECF 2015-273).
CR [Anonymous], 2007, A biologist's guide to mathematical modeling in ecology and evolution
Baig U, 2011, J OBES, V2011, DOI 10.1155/2011/861049
Blazei RW, 2006, APPL PREV PSYCHOL, V11, P230, DOI 10.1016/j.appsy.2006.07.001
Burgess SC, 2014, OIKOS, V123, P769, DOI 10.1111/oik.01235
Champagne FA, 2016, DEV PSYCHOPATHOL, V28, P1219, DOI 10.1017/S0954579416000808
Croft DP, 2015, TRENDS ECOL EVOL, V30, P407, DOI 10.1016/j.tree.2015.04.011
Curley JP, 2009, FRONT BEHAV NEUROSCI, V3, DOI 10.3389/neuro.08.025.2009
English S, 2016, AM NAT, V187, P620, DOI 10.1086/685644
English S, 2015, ANIM BEHAV, V106, P37, DOI 10.1016/j.anbehav.2015.05.006
English S, 2015, PLOS ONE, V10, DOI 10.1371/journal.pone.0116996
Fawcett TW, 2015, FRONT ZOOL, V12, DOI 10.1186/1742-9994-12-S1-S3
Fischer B, 2014, AM NAT, V183, P108, DOI 10.1086/674008
Fischer S, 2015, AM NAT, V186, P123, DOI 10.1086/681636

Frankenhuis WE, 2019, PHILOS T R SOC B, V374, DOI 10.1098/rstb.2018.0110
Frankenhuis WE, 2016, DEVELOPMENTAL SCI, V19, P251, DOI 10.1111/desc.12309
Frankenhuis WE, 2011, P ROY SOC B-BIOL SCI, V278, P3558, DOI 10.1098/rspb.2011.0055
French JA, 2016, CURR OPIN BEHAV SCI, V7, P40, DOI 10.1016/j.cobeha.2015.11.004
Gardner A, 2011, J EVOLUTION BIOL, V24, P1020, DOI 10.1111/j.1420-9101.2011.02236.x
HAMILTON WD, 1964, J THEOR BIOL, V7, P1, DOI [10.1016/0022-5193(64)90038-4, 10.1016/0022-5193(64)90039-6]
Holman L, 2014, BEHAV ECOL, V25, P1173, DOI 10.1093/beheco/aru100
Johnstone RA, 2012, P ROY SOC B-BIOL SCI, V279, P787, DOI 10.1098/rspb.2011.1257
Kasumovic MM, 2011, Q REV BIOL, V86, P181, DOI 10.1086/661119
Kuijper B, 2016, J EVOLUTION BIOL, V29, P265, DOI 10.1111/jeb.12778
Kuijper B, 2013, J EVOLUTION BIOL, V26, P1488, DOI 10.1111/jeb.12156
Kuijper B, 2018, EVOLUTION, V72, P220, DOI 10.1111/evo.13403
Lehmann L, 2007, J EVOLUTION BIOL, V20, P181, DOI 10.1111/j.1420-9101.2006.01202.x
Lehmann L, 2008, P ROY SOC B-BIOL SCI, V275, P1887, DOI 10.1098/rspb.2008.0276
Lehmann L, 2008, EVOLUTION, V62, P549, DOI 10.1111/j.1558-5646.2007.00291.x
Lehmann L, 2014, PHILOS T R SOC B, V369, DOI 10.1098/rstb.2013.0357
Lehmann L, 2010, PHILOS T R SOC B, V365, P2599, DOI 10.1098/rstb.2010.0138
Lehtonen J, 2012, PHILOS T R SOC B, V367, P211, DOI 10.1098/rstb.2011.0177
Maestripieri D, 2005, P NATL ACAD SCI USA, V102, P9726, DOI 10.1073/pnas.0504122102
Nettle D, 2017, PEERJ, V5, DOI 10.7717/peerj.3528
Nettle D, 2015, P ROY SOC B-BIOL SCI, V282, P23, DOI 10.1098/rspb.2015.1005
Nettle D, 2013, P ROY SOC B-BIOL SCI, V280, DOI 10.1098/rspb.2013.1343
Perkeybile AM, 2017, J EXP BIOL, V220, P114, DOI 10.1242/jeb.142182
Rodrigues AMM, 2016, PHILOS T R SOC B, V371, DOI 10.1098/rstb.2015.0088
Russell AF, 2009, PHILOS T R SOC B, V364, P1143, DOI 10.1098/rstb.2008.0298
Shea N, 2011, J EVOLUTION BIOL, V24, P1178, DOI 10.1111/j.1420-9101.2011.02235.x
Stamps JA, 2014, AM NAT, V184, P647, DOI 10.1086/678116
Taborsky B, 2017, ADV STUD BEHAV, V49, P49, DOI 10.1016/bs.asb.2016.12.002
Taborsky B, 2016, ETHOLOGY, V122, P267, DOI 10.1111/eth.12473
Taborsky B, 2012, TRENDS ECOL EVOL, V27, P679, DOI 10.1016/j.tree.2012.09.003
Taylor PD, 2007, J EVOLUTION BIOL, V20, P301, DOI 10.1111/j.1420-9101.2006.01196.x
Taylor PD, 1996, J THEOR BIOL, V180, P27, DOI 10.1006/jtbi.1996.0075
TAYLOR PD, 1990, AM NAT, V135, P95, DOI 10.1086/285034

   Vitikainen EIK, 2019, PHILOS T R SOC B, V374, DOI
10.1098/rstb.2018.0114
   Wells JCK., 2017, The Arc of Life: Evolution and Health Across the
Life Course, P21
   Wells JCK, 2007, TRENDS ENDOCRIN MET, V18, P331, DOI
10.1016/j.tem.2007.07.006
   Wolf M, 2008, P NATL ACAD SCI USA, V105, P15825, DOI
10.1073/pnas.0805473105
   Wolf M, 2007, NATURE, V447, P581, DOI 10.1038/nature05835
   Wright S, 1931, GENETICS, V16, P0097
NR 52
TC 15
Z9 16
U1 0
U2 22
PU ROYAL SOC
PI LONDON
PA 6-9 CARLTON HOUSE TERRACE, LONDON SW1Y 5AG, ENGLAND
SN 0962-8436
EI 1471-2970
J9 PHILOS T R SOC B
JI Philos. Trans. R. Soc. B-Biol. Sci.
PD APR 15
PY 2019
VL 374
IS 1770
AR 20180111
DI 10.1098/rstb.2018.0111
PG 8
WC Biology
WE Science Citation Index Expanded (SCI-EXPANDED)
SC Life Sciences & Biomedicine - Other Topics
GA HN8ZI
UT WOS:000460487000003
PM 30966877
OA Green Submitted, Green Published, Bronze
DA 2025-05-07
ER

PT J
AU Little, MA
   Garruto, RM
AF Little, MA
   Garruto, RM
TI Human adaptability research into the beginning of the third millennium
SO HUMAN BIOLOGY
LA English
DT Article; Proceedings Paper
CT Symposium on Anthropological Genetics in the 21st Century at the 14th
   International Congress of Anthropological and Ethnological Sciences
CY JUL 27, 1998
CL WILLIAMSBURG, VA
DE human adaptability; International Biological Program; history of
   research; future research directions

ID PHYSIOLOGICAL STRESS; REPRODUCTIVE ECOLOGY; TIBETAN HIGHLANDERS; OXYGEN-SATURATION; GROWTH; DISEASE; ANTHROPOLOGY; PREVALENCE; MALES

AB Human adaptability, as a field of inquiry within human biology, became defined during the research activities of the International Biological Program (IBP) (1964-1974). During this period, research was focused on ecological, physiological, and genetic studies of human populations within the theoretical frameworks of adaptation and evolution. Other defining characteristics of the IBP human adaptability research were standardization of methods, multidisciplinary projects, international cooperation, and a concern with human health issues. Some observers suggest that this research contributed to the ongoing transformation of physical anthropology and related fields from a largely descriptive to an analytical science. During the 25 years between the end of the IBP and the present, a number of research trends have continued: Several new multidisciplinary projects were initiated and completed; a subfield of demography within human biology has matured; nutrition, infant and child growth, and health studies have proliferated; and molecular generics and DNA analysis have superseded the earlier population genetics. International programs today are geared toward more practical and applied studies with less emphasis on basic science. Continuation of human adapt ability research into the 21st century is likely to make contributions in 3 broad areas: population, environment, and health. Productive research is likely to contribute to these 3 areas in the following categories: reproduction, psychosocial stress, life span approaches to health, effects of losses in biodiversity on health, a human biology of poverty, emerging infectious diseases, epidemiology of modernization, evolutionary medicine, and aging. The success of much of this research in its contribution to knowledge will come from the integrated perspectives of a biobehavioral framework of inquiry.

C1 SUNY Binghamton, Dept Anthropol, Binghamton, NY 13902 USA.
C3 State University of New York (SUNY) System; Binghamton University, SUNY
RP SUNY Binghamton, Dept Anthropol, Binghamton, NY 13902 USA.

CR [Anonymous], INT BIOL PROGRAMME G
[Anonymous], 1999, CAMBRIDGE STUDIES BI
[Anonymous], 2010, RUZHYA MIKROBY STALI
[Anonymous], 1990, AYMARA STRATEGIES HU
Bailey RobertC., 1988, COPING UNCERTAINTY F, P88
Baker P.T., 1967, The Biology of Human Adaptability
Baker P. T., 1986, CHANGING SAMOANS HLT
BAKER PT, 1972, ANNU REV ANTHROPOL, V1, P151, DOI 10.1146/annurev.an.01.100172.001055
BAKER PT, 1991, J HUM ECOL, V1, P39
Baker PT., 1976, MEASURES MAN, P230
BAKER PT, 1977, HUMAN POPULATION PRO, P11
Baker PT, 1965, INT BIOL PROGRAMME G, P63
Barker DJP, 1998, Mothers, babies, and health in later life
Barr R G, 1990, Hum Nat, V1, P355, DOI 10.1007/BF02734051
Beall CM, 1999, AM J PHYS ANTHROPOL, V108, P41, DOI 10.1002/(SICI)1096-8644(199901)108:1<41::AID-AJPA2>3.3.CO;2-B
BEALL CM, 1994, AM J PHYS ANTHROPOL, V95, P271, DOI 10.1002/ajpa.1330950303
Beall CM, 1997, HUM BIOL, V69, P597
BLANGERO J, 1993, HUM BIOL, V65, P941

Bogin B, 1997, YEARB PHYS ANTHROPOL, V40, P63
Botkin Daniel., 1990, Discordant Harmonies: A New Ecology for the Twenty-First Century
BOWEN ETW, 1977, LANCET, V1, P571
BOYCE AJ, 1984, SOC STUDY HUMAN BIOL, V23
BROWN DE, 1982, ANN HUM BIOL, V9, P553, DOI 10.1080/03014468200006071
Campbell BC, 1995, YEARB PHYS ANTHROPOL, V38, P1
CAMPBELL BC, 1999, TURKANA HERDERS DRY
Cavalli-Sforza L.L., 1994, The history and geography of human genes
Cernea M.M., 1985, PUTTING PEOPLE 1 SOC
CHAMBERS E, 1987, ANNU REV ANTHROPOL, V16, P309
Collee JG, 1997, LANCET, V349, P636, DOI 10.1016/S0140-6736(96)01310-4
Collins K.J., 1977, Human Adaptability: A History and Compendium of Research in the International Biological Programme
Crews D.E., 1994, BIOL ANTHR AGING PER
CUMMINGS SR, 1985, EPIDEMIOL REV, V7, P178, DOI 10.1093/oxfordjournals.epirev.a036281
Daltabuit Magali., 1998, BUILDING NEW BIOCULT
DAWSON DA, 1987, VITAL HLTH STAT, V10
Dill David B., 1964, HDB PHYSL 4
Dressler WW, 1995, YEARB PHYS ANTHROPOL, V38, P27
DRESSLER WW, 1987, AM ANTHROPOL, V89, P398, DOI 10.1525/aa.1987.89.2.02a00080
DURST M, 1983, P NATL ACAD SCI-BIOL, V80, P3812, DOI 10.1073/pnas.80.12.3812
Ellison PeterThorpe., 1991, APPL BIOLOGICAL ANTH, P14
ELLISON PT, 1994, ANNU REV ANTHROPOL, V23, P255, DOI 10.1146/annurev.an.23.100194.001351
Erlich P.R., 1988, BIODIVERSITY, P21
Ewald P W, 1994, Evolution of Infectious Diseases
FREGLY MJ, 1996, HDB PHYSL 4
GAJDUSEK DC, 1977, SCIENCE, V197, P943, DOI 10.1126/science.142303
Garrett L., 1994, The Coming Plague: Newly Emerging Diseases in a World Out of Balance
Garruto RM, 1999, P NATL ACAD SCI USA, V96, P10536, DOI 10.1073/pnas.96.18.10536
GARRUTO RM, 1994, BIOL INT, V32, P42
Gray SJ, 1999, RES MG HUM, P165
Greene L. S., 1980, SOCIAL BIOL PREDICTO
GREENE LS, 1973, AM J PHYS ANTHROPOL, V38, P119, DOI 10.1002/ajpa.1330380126
HAAS JD, 1999, S NEW TRENDS BIOL AN
HANNA JM, 1972, HUM BIOL, V44, P381
Harrison G. Ainsworth., 1997, HUMAN ADAPTABILITY P, P17
HARRISON GA, 1967, BIOL HIGH ALTITUDE P, P509
Harrison GA., 1995, HUMAN BIOL ENGLISH V
HARRISON GA, 1977, HUMAN POPULATION PRO, P65
HARRISON GA, 1990, SOC STUDY HUMAN BIOL, V30
HAUB C, 1997, POPULATION TODAY, V25, P1
HAUPT A, 1991, POPULATION HDB
HEDIGER ML, 1986, HUM BIOL, V58, P585
HENRY CJK, 1996, SOC STUDY HUMAN BIOL, V37
HOMER D, 1987, ANN NEUROL, V22, P245, DOI 10.1002/ana.410220209
HOROWITZ MM, 1990, ENTHNOLOGISCHE BEITR, V2, P189

HUGHES JM, 1993, SCIENCE, V262, P850, DOI 10.1126/science.8235607
HUSSASHMORE R, 1988, RES PAPERS SCI ARCHE, V5
James GD, 1997, ANNU REV ANTHROPOL, V26, P313, DOI 10.1146/annurev.anthro.26.1.313
JOHNS T, 1990, THESE BITTER HERBS T
JOHNSON KM, 1977, LANCET, V1, P569
KOTTAK CP, 1998, INT C ANTHR ETHN SCI
LAMPL M, 1992, SCIENCE, V258, P801, DOI 10.1126/science.1439787
LAMPL M, 1993, AM J HUM BIOL, V5, P641, DOI 10.1002/ajhb.1310050607
LASKER GW, 1969, SCIENCE, V166, P1480, DOI 10.1126/science.166.3912.1480
LEDERBERG J, 1998, EMERG INFECT DIS, V4, P7
Leidy LE, 1996, AM J HUM BIOL, V8, P699, DOI 10.1002/(SICI)1520-6300(1996)8:6<699::AID-AJHB1>3.0.CO;2-P
Leslie P.W., 1989, HUMAN POPULATION BIO, P15
Little M.A., 1999, Turkana herders of the dry savanna: ecology and biobehavioral response of nomads to an uncertain environment
Little MA., 1993, RES STRATEGIES HUMAN, P62
Little MA., 1988, Biological aspects of human migration, P167
LITTLE MA, 1991, J INDIAN ANTHR, V26, P9
Martorell R., 1980, SOCIAL BIOLOGICAL PR, P81
McKenna JJ, 1996, ANNU REV ANTHROPOL, V25, P201, DOI 10.1146/annurev.anthro.25.1.201
MCKENNA JJ, 1994, ACTA PAEDIATR, V83, P94, DOI 10.1111/j.1651-2227.1994.tb13271.x
Milan F.A., 1980, The human biology of circumpolar populations
Miles Toni P., 1994, P3
Morse Stephen S., 1993, P10
Nesse R M, 1990, Hum Nat, V1, P261, DOI 10.1007/BF02733986
Nesse R.M., 1994, WHY WE GET SICK NEW
Panter-Brick C., 1999, HORMONES HLTH BEHAV
PEARSON JD, 1993, AM J HUM BIOL, V5, P49, DOI 10.1002/ajhb.1310050109
Peters R.L. II, 1988, P450
PFEFFER RI, 1987, AM J EPIDEMIOL, V125, P420, DOI 10.1093/oxfordjournals.aje.a114548
POPOVIC M, 1984, SCIENCE, V224, P497, DOI 10.1126/science.6200935
PRIOR IAM, 1977, POPULATION STRUCTURE, P165
Raffel M.W., 1997, Health Care and Reform in Industrialized Countries
SCHELL LM, 1991, YEARB PHYS ANTHROPOL, V34, P157
SCHELL LM, 1993, SOC STUDY HUMAN BIOL, V32
Shephard R., 1996, HLTH CONSEQUENCES MO
STEERE AC, 1989, NEW ENGL J MED, V321, P586, DOI 10.1056/NEJM198908313210906
STRICKLAND SS, 1998, SOC STUDY HUMAN BIOL, V39
Stuart-Macadam Patricia., 1995, BREASTFEEDING BIOCUL
Thomas B.R., 1989, HUMAN POPULATION BIO, P296
Thomas B.R., 1998, BUILDING NEW BIOCULT, P43
Thomas R.Brooke., 1997, HUMAN ADAPTABILITY P, P183
THOMAS RB, 1988, SOC STUDY HUMAN BIOL, V26, P249
TORREY BB, 1987, INT POPULATION P, V25
Ulijaszek S., 1997, HUMAN ADAPTABILITY
Ulijaszek S.J., 1997, HUMAN ADAPTABILITY, P261
ULIJASZEK SJ, 1993, SOC STUDY HUMAN BIOL, V35
Ulijaszek SJ., 1997, Human adaptability: Past, present and future, P7

Vitzthum Virginia J., 1994, Yearbook of Physical Anthropology, V37, P307
Vitzthum VJ, 1998, AM J HUM BIOL, V10, P145, DOI 10.1002/(SICI)1520-6300(1998)10:2<145::AID-AJHB1>3.0.CO;2-K
Webster Robert G., 1993, P37
Weiner J.S., 1977, HUMAN ADAPTABILITY, P1
Weiner J. S., 1969, IBP HDB, V9
WEINER JS, 1967, BIOL HUMAN ADAPTABIL, P1
Weiner JS., 1981, Practical Human Biology
Weiss K. M., 1973, Demographic Models for Anthropology, Memoir, V27
WIDDOWSON EM, 1974, SIZE BIRTH, P65
WILLIAMS A, 1997, BEIN REASONABLE EC H
WILLIAMS GC, 1991, Q REV BIOL, V66, P1, DOI 10.1086/417048
Williams-Blangero S., 1992, American Journal of Human Genetics, V51, pA163
Wilson E.O., 1988, P3
Wolff E.N., 1996, TOP HEAVY INCREASING
Wood J.W., 1994, DYNAMICS HUMAN REPRO
Yoshimura H., 1966, HUMAN ADAPTABILITY I
ZEMEL BS, 1986, AM J PHYS ANTHROPOL, V71, P459, DOI 10.1002/ajpa.1330710409
Zubrow EzraB.W., 1976, DEMOGRAPHIC ANTHR QU
1996, DIVERSITAS INT PROGR
1990, US MAN BIOSPHERE PRO
NR 129
TC 7
Z9 11
U1 0
U2 10
PU WAYNE STATE UNIV PRESS
PI DETROIT
PA 4809 WOODWARD AVE, DETROIT, MI 48201-1309 USA
SN 0018-7143
EI 1534-6617
J9 HUM BIOL
JI Hum. Biol.
PD FEB
PY 2000
VL 72
IS 1
BP 179
EP 199
PG 21
WC Anthropology; Biology; Genetics & Heredity
WE Science Citation Index Expanded (SCI-EXPANDED); Social Science Citation Index (SSCI); Conference Proceedings Citation Index - Science (CPCI-S)
SC Anthropology; Life Sciences & Biomedicine - Other Topics; Genetics & Heredity
GA 287KQ
UT WOS:000085505400008
PM 10721617
DA 2025-05-07
ER

PT J
AU Tollis, M
Schneider-Utaka, AK
Maley, CC
AF Tollis, Marc
Schneider-Utaka, Aika K.
Maley, Carlo C.
TI The Evolution of Human Cancer Gene Duplications across Mammals
SO MOLECULAR BIOLOGY AND EVOLUTION
LA English
DT Article
DE mammals; genome; tumor suppressor genes; oncogenes; cancer; life history
ID NAKED MOLE-RAT; RISK; RESISTANCE; DNA; EXTINCTION; ELEPHANTS; INSIGHTS;
MUTATION; BROWSER; HEIGHT
AB Cancer is caused by genetic alterations that affect cellular fitness, and multicellular organisms have evolved mechanisms to suppress cancer such as cell cycle checkpoints and apoptosis. These pathways may be enhanced by the addition of tumor suppressor gene paralogs or deletion of oncogenes. To provide insights to the evolution of cancer suppression across the mammalian radiation, we estimated copy numbers for 548 human tumor suppressor gene and oncogene homologs in 63 mammalian genome assemblies. The naked mole rat contained the most cancer gene copies, consistent with the extremely low rates of cancer found in this species. We found a positive correlation between a species' cancer gene copy number and its longevity, but not body size, contrary to predictions from Peto's Paradox. Extremely long-lived mammals also contained more copies of caretaker genes in their genomes, suggesting that the maintenance of genome integrity is an essential form of cancer prevention in long-lived species. We found the strongest association between longevity and copy numbers of genes that are both germline and somatic tumor suppressor genes, suggesting that selection has acted to suppress both hereditary and sporadic cancers. We also found a strong relationship between the number of tumor suppressor genes and the number of oncogenes in mammalian genomes, suggesting that complex regulatory networks mediate the balance between cell proliferation and checks on tumor progression. This study is the first to investigate cancer gene expansions across the mammalian radiation and provides a springboard for potential human therapies based on evolutionary medicine.
C1 [Tollis, Marc] No Arizona Univ, Sch Informat Comp & Cyber Syst, Flagstaff, AZ 86011 USA.
[Tollis, Marc; Maley, Carlo C.] Arizona State Univ, Arizona Canc Evolut Ctr, Tempe, AZ 85281 USA.
[Schneider-Utaka, Aika K.; Maley, Carlo C.] Arizona State Univ, Sch Life Sci, Tempe, AZ USA.
C3 Northern Arizona University; Arizona State University; Arizona State University-Tempe; Arizona State University; Arizona State University-Tempe
RP Tollis, M (autor correspondente), No Arizona Univ, Sch Informat Comp & Cyber Syst, Flagstaff, AZ 86011 USA.; Tollis, M (autor correspondente), Arizona State Univ, Arizona Canc Evolut Ctr, Tempe, AZ 85281 USA.
EM marc.tollis@nau.edu

RI Tollis, Marc/AAX-2332-2020
OI Schneider-Utaka, Aika/0000-0002-6348-1521; Tollis,
   Marc/0000-0002-1917-2473
FU NIH [U54 CA217376, U2C CA233254, P01 CA91955, R01 CA149566, R01
   CA170595, R01 CA185138, R01 CA140657]; CDMRP Breast Cancer Research
   Program [BC132057]; Arizona Biomedical Research Commission
   [ADHS18-198847]
FX This work was supported in part by NIH (Grant Nos. U54 CA217376, U2C
   CA233254, P01 CA91955, R01 CA149566, R01 CA170595, R01 CA185138, and
R01
   CA140657) as well as CDMRP Breast Cancer Research Program (Award No.
   BC132057) and the Arizona Biomedical Research Commission (Grant No.
   ADHS18-198847 to C.C.M.). We would like to thank Brian Hanratty from
the
   Bioinformatics Core at Arizona State University and Michael I. Byars
   from Northern Arizona University. We also extend gratitude toward Dr
   Melissa Wilson (Arizona State University) for valuable comments during
   early manuscript preparations.
CR Abegglen LM, 2015, JAMA-J AM MED ASSOC, V314, P1850, DOI
10.1001/jama.2015.13134
   ADELMAN R, 1988, P NATL ACAD SCI USA, V85, P2706, DOI
10.1073/pnas.85.8.2706
   Aktipis CA, 2015, PHILOS T R SOC B, V370, DOI 10.1098/rstb.2014.0219
   Albanes D, 1998, BMJ-BRIT MED J, V317, P1331, DOI
10.1136/bmj.317.7169.1331
   ANDERVONT HB, 1962, JNCI-J NATL CANCER I, V28, P1153
   [Anonymous], 2018, R LANG ENV STAT COMP
   AUSTAD SN, 1991, J GERONTOL, V46, pB47, DOI 10.1093/geronj/46.2.B47
   Bodmer W, 2010, CURR OPIN GENET DEV, V20, P262, DOI
10.1016/j.gde.2010.04.016
   Boratyn GM, 2012, BIOL DIRECT, V7, DOI 10.1186/1745-6150-7-12
   Buffenstein R, 2005, J GERONTOL A-BIOL, V60, P1369, DOI
10.1093/gerona/60.11.1369
   Buffenstein R, 2008, J COMP PHYSIOL B, V178, P439, DOI 10.1007/s00360-
007-0237-5
   Caulin AF, 2015, PHILOS T R SOC B, V370, DOI 10.1098/rstb.2014.0222
   Caulin AF, 2011, TRENDS ECOL EVOL, V26, P175, DOI
10.1016/j.tree.2011.01.002
   Delaney MA, 2016, VET PATHOL, V53, P691, DOI 10.1177/0300985816630796
   Demuth JP, 2006, PLOS ONE, V1, DOI 10.1371/journal.pone.0000085
   Domazet-Loso T, 2010, BMC BIOL, V8, DOI 10.1186/1741-7007-8-66
   Dorus S, 2004, CELL, V119, P1027, DOI 10.1016/j.cell.2004.11.040
   Faith DP, 2007, PLOS ONE, V2, pe296
   Ferlay J, 2015, INT J CANCER, V136, pE359, DOI 10.1002/ijc.29210
   Foley NM, 2018, SCI ADV, V4, DOI 10.1126/sciadv.aao0926
   Frank S. A., 2007, Dynamics of Cancer: Incidence, Inheritance, and
Evolution
   Fritz SA, 2009, ECOL LETT, V12, P538, DOI 10.1111/j.1461-
0248.2009.01307.x
   Futreal PA, 2004, NAT REV CANCER, V4, P177, DOI 10.1038/nrc1299
   Gumal M. T., 1998, HORNBILL, V2, P82
   Hanahan D, 2011, CELL, V144, P646, DOI 10.1016/j.cell.2011.02.013
   Higginbotham S, 2014, PLOS ONE, V9, DOI 10.1371/journal.pone.0084549

Huang ZX, 2019, NAT ECOL EVOL, V3, P1110, DOI 10.1038/s41559-019-0913-3
Jones Kate E., 2009, Ecology (Washington D C), V90, P2648, DOI 10.1890/08-1494.1
Karolchik D, 2004, NUCLEIC ACIDS RES, V32, pD493, DOI 10.1093/nar/gkh103
Keane M, 2015, CELL REP, V10, P112, DOI 10.1016/j.celrep.2014.12.008
Kent WJ, 2002, GENOME RES, V12, P996, DOI 10.1101/gr.229102
Kent WJ, 2002, GENOME RES, V12, P656, DOI [10.1101/gr.229202. Article published online before March 2002, 10.1101/gr.229202]
Kinsella RJ, 2011, DATABASE-OXFORD, DOI 10.1093/database/bar030
Kinzler KW, 1997, NATURE, V386, P761, DOI 10.1038/386761a0
Koepfli KP, 2015, ANNU REV ANIM BIOSCI, V3, P57, DOI 10.1146/annurev-animal-090414-014900
Kumar S, 2017, MOL BIOL EVOL, V34, P1812, DOI 10.1093/molbev/msx116
Kumari Rajni, 2014, Mol Cell Oncol, V1, pe969653, DOI 10.4161/23723548.2014.969653
Lahmann PH, 2016, CANCER EPIDEMIOL, V40, P119, DOI 10.1016/j.canep.2015.12.006
Lee BR, 2015, J ZOO WILDLIFE MED, V46, P333, DOI 10.1638/2013-0258R1.1
Leroi AM, 2003, NAT REV CANCER, V3, P226, DOI 10.1038/nrc1016
Lewin HA, 2018, P NATL ACAD SCI USA, V115, P4325, DOI 10.1073/pnas.1720115115
Li Y, 2013, AGE, V35, P301, DOI 10.1007/s11357-011-9361-y
MacRae SL, 2015, AGING CELL, V14, P288, DOI 10.1111/acel.12314
Makashov AA, 2019, SCI REP, V9, P12
McDonald J.H., 2014, Handbook of Biological Statistics, VThird
McNab B. K., 1985, EVOLUTION ECOLOGY AR
Meredith RW, 2011, SCIENCE, V334, P521, DOI 10.1126/science.1211028
Meyers PR, 2020, ANIMAL DIVERSITY WEB
Mossman H.W., 1987, Vertebrate Fetal Membranes: Comparative Ontogeny and Morphology; Evolution; Phylogenetic Significance; Basic Functions; Research Opportunities
Munshi-South J, 2010, AGEING RES REV, V9, P12, DOI 10.1016/j.arr.2009.07.006
Myhrvold N.P., 2015, Ecology, V96, P3109, DOI [DOI 10.1890/15-0846R.1, 10.1890/15-0846r.1, 10.1890/15-0846R.1]
Nunney L, 1999, P ROY SOC B-BIOL SCI, V266, P493, DOI 10.1098/rspb.1999.0664
Nunney L, 2018, P BIOL SCI, V285, P1889
O'Leary NA, 2016, NUCLEIC ACIDS RES, V44, pD733, DOI 10.1093/nar/gkv1189
OHTA T, 1989, GENOME, V31, P304, DOI 10.1139/g89-048
Orme D., 2018, CAPER COMP ANAL PHYL
Pauli JN, 2016, AM NAT, V188, P196, DOI 10.1086/687032
PENCE DB, 1983, J COMP PATHOL, V93, P179, DOI 10.1016/0021-9975(83)90004-X
PETO R, 1975, BRIT J CANCER, V32, P411, DOI 10.1038/bjc.1975.242
Peto R., 1977, ORIGINS HUMAN CANC, P403
Roche B, 2012, BMC CANCER, V12, DOI 10.1186/1471-2407-12-387
Sayres MAW, 2011, EVOLUTION, V65, P2800, DOI 10.1111/j.1558-5646.2011.01337.x
Seluanov A, 2007, AGING CELL, V6, P45, DOI 10.1111/j.1474-9726.2006.00262.x

Seluanov A, 2018, NAT REV CANCER, V18, P433, DOI 10.1038/s41568-018-0004-9
Seluanov A, 2009, P NATL ACAD SCI USA, V106, P19352, DOI 10.1073/pnas.0905252106
Sieg AE, 2009, AM NAT, V174, P720, DOI 10.1086/606023
Siepel A, 2005, GENOME RES, V15, P1034, DOI 10.1101/gr.3715005
Simao FA, 2015, BIOINFORMATICS, V31, P3210, DOI 10.1093/bioinformatics/btv351
Sondka Z, 2018, NAT REV CANCER, V18, P696, DOI 10.1038/s41568-018-0060-1
Stelzer G., 2016, Curr Protoc Bioinformatics, V54, DOI [10.1002/cpbi.5, DOI 10.1002/CPBI.5]
Stratton MR, 2009, NATURE, V458, P719, DOI 10.1038/nature07943
Sulak M, 2016, ELIFE, V5, DOI [10.7554/eLife.11994, 10.7554/eLife.24307]
Tate JG, 2019, NUCLEIC ACIDS RES, V47, pD941, DOI 10.1093/nar/gky1015
Tian X, 2013, NATURE, V499, P346, DOI 10.1038/nature12234
Tollis M, 2019, MOL BIOL EVOL, V314, P1850
Tollis M, 2017, CURR OPIN GENET DEV, V42, P40, DOI 10.1016/j.gde.2016.12.004
Vazquez JM, 2018, CELL REP, V24, P1765, DOI 10.1016/j.celrep.2018.07.042
Venditti C, 2006, SYST BIOL, V55, P637, DOI 10.1080/10635150600865567
Vicens A, 2018, GENES-BASEL, V9, DOI 10.3390/genes9120582
Vogelstein B, 2004, NAT MED, V10, P789, DOI 10.1038/nm1087
Voskarides K, 2019, HUM GENOMICS, V13, DOI 10.1186/s40246-019-0210-x
White MC, 2014, AM J PREV MED, V46, pS7, DOI 10.1016/j.amepre.2013.10.029
Wu WKK, 2017, MOL BIOL EVOL, V34, P903, DOI 10.1093/molbev/msw295
Xie YL, 2004, CANCER RES, V64, P3096, DOI 10.1158/0008-5472.CAN-03-3834
Zdobnov EM, 2017, NUCLEIC ACIDS RES, V45, pD744, DOI 10.1093/nar/gkw1119
NR 85
TC 33
Z9 36
U1 1
U2 15
PU OXFORD UNIV PRESS
PI OXFORD
PA GREAT CLARENDON ST, OXFORD OX2 6DP, ENGLAND
SN 0737-4038
EI 1537-1719
J9 MOL BIOL EVOL
JI Mol. Biol. Evol.
PD OCT
PY 2020
VL 37
IS 10
BP 2875
EP 2886
DI 10.1093/molbev/msaa125
PG 12
WC Biochemistry & Molecular Biology; Evolutionary Biology; Genetics &

Heredity
WE Science Citation Index Expanded (SCI-EXPANDED)
SC Biochemistry & Molecular Biology; Evolutionary Biology; Genetics & Heredity
GA OW8FQ
UT WOS:000593115800008
PM 32421773
OA Green Submitted, Green Published, hybrid
DA 2025-05-07
ER

PT J
AU Funk, VA
AF Funk, Vicki A.
TI Collections-based science in the 21st Century
SO JOURNAL OF SYSTEMATICS AND EVOLUTION
LA English
DT Review
DE biogeography; biological-collections; cladistics; classification; collections-based research; evolution; phylogenetics; phylogenomics; systematics; tree-thinking
ID PLANT DNA BARCODES; CHLOROPLAST DNA; COMPARATIVE PHYLOGEOGRAPHY; PHYLOGENETIC STRUCTURE; HERBARIUM SPECIMENS; BIODIVERSITY; VITACEAE; AREAS; TREE; DIVERSIFICATION
AB Discoveries from collections-based science change the way we perceive ourselves, our environment, and our place in the universe. The 18(th) Century saw the beginning of formal classification with Linnaeus proposing a system to classify all of life. The 19(th) Century ushered in the age of exploration as naturalists undertook large-scale collecting expeditions leading to major scientific advances (the founding of Physical Geography, Meteorology, Ecology, Biogeography, and Evolution) and challenging long held beliefs about nature. In the 20(th) Century collections were central to paradigm shifts, including theories of Continental Drift and Phylogenetic Systematics; Molecular Phylogenetics added testable hypotheses, and computerized specimen records gave rise to the field of Biodiversity. In the first 15 years of the 21(st) Century we have seen tree-thinking pervade the life sciences, leading to the emergence of Evolutionary Medicine, Evolutionary Ecology, and new Food Safety methods. More advances are on the way: (i) Open access to large amounts of specimen data & images, (ii) Linking of collections and climate data to phylogenies on a global scale, and (iii) Production of vast quantities of genomic data allowing us to address big evolutionary questions. As a result of collections-based science people see themselves not as the center of all things but rather as part of a complex universe. It is essential that we integrate new discoveries with knowledge from the past (e.g., collections) in order to understand this planet we all inhabit. To ensure the health of collections-based science we must come together and plan for the future.
C1 [Funk, Vicki A.] Smithsonian Inst, Dept Bot, Natl Museum Nat Hist, MRC 166,POB 37012, Washington, DC 20013 USA.
C3 Smithsonian Institution; Smithsonian National Museum of Natural History
RP Funk, VA (autor correspondente), Smithsonian Inst, Dept Bot, Natl Museum Nat Hist, MRC 166,POB 37012, Washington, DC 20013 USA.

EM funkv@si.edu
CR Abraham ORTELIUS., 1570, Theatrum Orbis Terrarum
Adanson Michel., 1763, Familles naturelles des plantes
Allard MW, 2018, CURR OPIN BIOTECH, V49, P224, DOI 10.1016/j.copbio.2017.11.002
Allard MW, 2016, J CLIN MICROBIOL, V54, P1975, DOI 10.1128/JCM.00081-16
[Anonymous], COMPOSITAE NEWSLETTE
[Anonymous], GEOGRAPHIE PFLANZEN
[Anonymous], 1950, GRUNDZUGE THEORIE PH
[Anonymous], PLANTS ARE COOL TOO
[Anonymous], LICH GEN WORKSH
[Anonymous], HUMBOLDT DIGITAL LIB
[Anonymous], ADV CLADISTICS
[Anonymous], FDA WHOL GEN SEQ PRO
[Anonymous], GEOGRAPHICAL DISTRIB
[Anonymous], NATL PARKS PROTECTED
[Anonymous], 1964, On the Origin of Species
[Anonymous], ADV CLAD P 1 M W HEN
[Anonymous], 1995, HAWAIIAN BIOGEOGRAPH
[Anonymous], W AFRICAN PLANTS PHO
[Anonymous], INVENTION NATURE A V
[Anonymous], AUSTR BIOL
[Anonymous], PHOTO GUIDE PLANTS S
[Anonymous], AUTONOMOUS REEF MONI
[Anonymous], J SYSTEMATICS EVOLUT
[Anonymous], BARC LIF ID SPEC DNA
[Anonymous], INFL VIR GEN SEQ GEN
[Anonymous], CREATION SES MYSTERE
[Anonymous], FLORA ANTARCTICA BOT
[Anonymous], TROPICOS MISS BOT GA
[Anonymous], PLANT PRESS
Avise JC., 2000, PHYLOGEOGRAPHY HIST, DOI DOI 10.2307/J.CTV1NZFGJ7
Bartlett John M S, 2003, Methods Mol Biol, V226, P3
Baum D.A., 2012, Tree Thinking: An Introduction to Phylogenetic Biology
Beck JB, 2015, APPL PLANT SCI, V3, DOI 10.3732/apps.1500014
Beheregaray LB, 2017, MOL PHYLOGENET EVOL, V111, P65, DOI 10.1016/j.ympev.2017.03.021
Bell RC, 2015, EVOLUTION, V69, P904, DOI 10.1111/evo.12623
Bos KI, 2014, NATURE, V514, P494, DOI 10.1038/nature13591
Brundin L., 1966, Kungliga Svenska Vetenskapsakademiens Handlingar (4), V11, P1
Calinger KM, 2013, ECOL LETT, V16, P1037, DOI 10.1111/ele.12135
Carew ME, 2017, MOL ECOL RESOUR, V17, P915, DOI 10.1111/1755-0998.12644
Carey S.W., 1958, Continental drift. A symposium. Hobart, P177
Cavender-Bares J, 2003, ECOLOGY, V84, P592, DOI 10.1890/0012-9658(2003)084[0592:IMAMPI]2.0.CO;2
Crandall KA, 1999, AUST J ZOOL, V47, P199, DOI 10.1071/ZO99011
Darlington P.J., 1965, BIOGEOGRAPHY SO END, DOI DOI 10.4159/HARVARD.9780674492073
Darwin C. R., 1858, Journal of the proceedings of the Linnean Society. Zoology, V3, P46, DOI DOI 10.1111/J.1096-3642.1858.TB02500.X

Diazgranados M., 2015, Los pramos circundantes a la Sabana de Bogot (de Ernesto Guhl Nimtz), P175
Diazgranados M., 2012, THESIS
Eigenmann C.H., 1909, Report of the Princeton Expedition Patagonia, V3, P225
EXCOFFIER L, 1992, GENETICS, V131, P479
Faith DP, 1996, BIODIVERS CONSERV, V5, P399, DOI 10.1007/BF00056387
Feeley KJ, 2012, GLOBAL CHANGE BIOL, V18, P1335, DOI 10.1111/j.1365-2486.2011.02602.x
Ferrier S, 1999, TRZS NSW, P68
Ferrier S, 2000, BIOL CONSERV, V93, P303, DOI 10.1016/S0006-3207(99)00149-4
FUNK V.A., 2009, SYSTEMATICS EVOLUTIO
Funk V. A., 2017, Tropical Plant Collections: Legacies from the past? Essential tools for the future? The Royal Danish Academy of Sciences and Letters, P73
Gire SK, 2014, SCIENCE, V345, P1369, DOI 10.1126/science.1259657
Gluckman P, 2016, PRINCIPLES OF EVOLUTIONARY MEDICINE, 2ND EDITION, P1, DOI 10.1093/acprof:oso/9780199663927.001.0001
Goodwin ZA, 2015, CURR BIOL, V25, pR1066, DOI 10.1016/j.cub.2015.10.002
Gostel MR, 2015, J SYST EVOL, V53, P411, DOI 10.1111/jse.12173
Graham CH, 2004, TRENDS ECOL EVOL, V19, P497, DOI 10.1016/j.tree.2004.07.006
Habib S, 2018, J SYST EVOL, V56, P360, DOI 10.1111/jse.12309
Habib S, 2017, FRONT PLANT SCI, V8, DOI 10.3389/fpls.2017.00590
Heard E, 2014, CELL, V157, P95, DOI 10.1016/j.cell.2014.02.045
Hennig W., 1999, Phylogenetic systematics
Hillis D.M., 1990, SCIENCE, V250, P1022
Hillis D.M., 1990, MOL SYSTEMATICS, DOI DOI 10.2307/1447682
Holmes A., 1944, PRINCIPLES PHYS GEOL
Hull D.L., 1988, Science as a process: an evolutionary account of the social and conceptual development of science, DOI [10.7208/chicago/9780226360492.001.0001, DOI 10.7208/CHICAGO/9780226360492.001.0001]
HUMBOLDT AV, 1845, KOSMOS ENTWURF PHYS
JANSEN RK, 1987, P NATL ACAD SCI USA, V84, P5818, DOI 10.1073/pnas.84.16.5818
JANSEN RK, 1988, AM J BOT, V75, P753, DOI 10.2307/2444207
JANSEN RK, 1987, CURR GENET, V11, P553, DOI 10.1007/BF00384619
Jussieu A.L., 1789, Genera Plantarum, secundum ordines naturales disposita juxta methodum in Horto Regio Parisiensi exaratam
Kembel SW, 2006, ECOLOGY, V87, pS86, DOI 10.1890/0012-9658(2006)87[86:TPSOAN]2.0.CO;2
Kress WJ, 2017, J SYST EVOL, V55, P291, DOI 10.1111/jse.12254
Kress WJ, 2015, TRENDS ECOL EVOL, V30, P25, DOI 10.1016/j.tree.2014.10.008
Kress WJ, 2010, PLOS ONE, V5, DOI 10.1371/journal.pone.0015409
Kress WJ, 2009, P NATL ACAD SCI USA, V106, P18621, DOI 10.1073/pnas.0909820106
Kuzmina ML, 2017, APPL PLANT SCI, V5, DOI 10.3732/apps.1700079
Li Y, 2007, P NATL ACAD SCI USA, V104, P15787, DOI 10.1073/pnas.0609268104
Linnaeus C., 1753, SPECIES PLANTARUM

Liu XQ, 2016, MOL PHYLOGENET EVOL, V95, P217, DOI 10.1016/j.ympev.2015.10.013
Liu XQ, 2013, MOL PHYLOGENET EVOL, V66, P43, DOI 10.1016/j.ympev.2012.09.003
Losos JB, 1996, ECOLOGY, V77, P1344, DOI 10.2307/2265532
Lu LM, 2018, CLADISTICS, V34, P57, DOI 10.1111/cla.12191
Mandel JR, 2017, J SYST EVOL, V55, P405, DOI 10.1111/jse.12265
MARGULES CR, 1988, BIOL CONSERV, V43, P63, DOI 10.1016/0006-3207(88)90078-X
MARGULES CR, 1994, PHILOS T ROY SOC B, V344, P69, DOI 10.1098/rstb.1994.0053
Martin MD, 2018, MOL PHYLOGENET EVOL, V120, P335, DOI 10.1016/j.ympev.2017.12.023
Martin MD, 2014, MOL ECOL, V23, P1701, DOI 10.1111/mec.12675
Mishler BD, 2014, NAT COMMUN, V5, DOI 10.1038/ncomms5473
Moritz C, 1998, MOL ECOL, V7, P419, DOI 10.1046/j.1365-294x.1998.00317.x
Moritz C, 2001, P ROY SOC B-BIOL SCI, V268, P1875, DOI 10.1098/rspb.2001.1713
Mullis KB, 1987, US Patent, Patent No. [4,683,195, 4683195]
Nelson G., 1981, SYSTEMATICS BIOGEORA
Ottesen A, 2016, BMC MICROBIOL, V16, DOI 10.1186/s12866-016-0894-1
Pace MR, 2018, J SYST EVOL, V56, P394, DOI 10.1111/jse.12303
Panero JL, 2008, MOL PHYLOGENET EVOL, V47, P757, DOI 10.1016/j.ympev.2008.02.011
Pinto CM, 2010, EMERG INFECT DIS, V16, P356, DOI 10.3201/eid1602.090998
PRESSEY RL, 1993, TRENDS ECOL EVOL, V8, P124, DOI 10.1016/0169-5347(93)90023-I
Sawyer S, 2015, P NATL ACAD SCI USA, V112, P15696, DOI 10.1073/pnas.1519905112
Schuettpelz E, 2017, BIODIVERS DATA J, V5, DOI 10.3897/BDJ.5.e21139
Sharp PM, 2011, CSH PERSPECT MED, V1, DOI 10.1101/cshperspect.a006841
Shaw KL, 2016, P NATL ACAD SCI USA, V113, P7986, DOI 10.1073/pnas.1601078113
Smith Charles H., 2017, Linnean, V33, P18
Stearns S. C., 2015, Evolutionary Medicine
Still SM, 2015, NAT AREA J, V35, P106, DOI 10.3375/043.035.0115
Tank DC, 2015, NEW PHYTOL, V207, P454, DOI 10.1111/nph.13491
Taylor J., 2015, Body By Darwin: How Evolution Shapes Our Health And Transforms Medicine
Thiers B., 2025, Index Herbariorum: A global directory of public herbaria and associated staff
Thornhill AH, 2017, BMC BIOL, V15, DOI 10.1186/s12915-017-0435-x
Unger J, 2016, BMC EVOL BIOL, V16, DOI 10.1186/s12862-016-0827-5
VanBuren R, 2015, NATURE, V527, P508, DOI 10.1038/nature15714
Vanneste K, 2014, GENOME RES, V24, P1334, DOI 10.1101/gr.168997.113
Dang VC, 2017, SYST BOT, V42, P449, DOI [10.1600/036364417X696032, 10.1600/036364417x696032]
Wallace AlfredRussel., 1905, MY LIFE RECORD EVENT, V2
Webb CO, 2000, AM NAT, V156, P145, DOI 10.1086/303378
Wegener A., 1912, Geologische Rundschau, V3, P276, DOI DOI 10.1007/BF02202896
Wegener A, 1912, PETERMANNS MITT, V58, P185

   Wegener A, 1912, PETERMANNS MITT, V58, P253
   Wegener A, 1912, PETERMANNS MITT, V58, P305
   Wen J, 2017, J SYST EVOL, V55, P308, DOI 10.1111/jse.12270
   Wen J, 2015, J SYST EVOL, V53, P369, DOI 10.1111/jse.12175
   Wen J, 2015, J SYST EVOL, V53, P477, DOI 10.1111/jse.12181
   Wiley EO, 1991, U KANSAS SPECIAL PUB, V19
   Williams P, 1996, CONSERV BIOL, V10, P155, DOI 10.1046/j.1523-1739.1996.10010155.x
   Zhang N, 2017, PLANTA MED, V83, P1420, DOI 10.1055/s-0043-113449
   Zimmer EA, 2015, J SYST EVOL, V53, P371, DOI 10.1111/jse.12174
   Zúñiga JD, 2017, PHYTOKEYS, P119, DOI 10.3897/phytokeys.88.14607
NR 128
TC 94
Z9 117
U1 0
U2 58
PU WILEY
PI HOBOKEN
PA 111 RIVER ST, HOBOKEN 07030-5774, NJ USA
SN 1674-4918
EI 1759-6831
J9 J SYST EVOL
JI J. Syst. Evol.
PD MAY
PY 2018
VL 56
IS 3
BP 175
EP 193
DI 10.1111/jse.12315
PG 19
WC Plant Sciences
WE Science Citation Index Expanded (SCI-EXPANDED)
SC Plant Sciences
GA GH6WP
UT WOS:000433585100001
OA hybrid
DA 2025-05-07
ER

PT J
AU Vining, AQ
   Nunn, CL
AF Vining, Alexander Q.
   Nunn, Charles L.
TI Evolutionary change in physiological phenotypes along the human lineage
SO EVOLUTION MEDICINE AND PUBLIC HEALTH
LA English
DT Article
AB Background and Objectives: Research in evolutionary medicine provides many examples of how evolution has shaped human susceptibility to disease. Traits undergoing rapid evolutionary change may result in associated costs or reduce the energy available to other traits. We

hypothesize that humans have experienced more such changes than other primates as a result of major evolutionary change along the human lineage. We investigated 41 physiological traits across 50 primate species to identify traits that have undergone marked evolutionary change along the human lineage.

Methodology: We analysed the data using two Bayesian phylogenetic comparative methods. One approach models trait covariation in non-human primates and predicts human phenotypes to identify whether humans are evolutionary outliers. The other approach models adaptive shifts under an Ornstein-Uhlenbeck model of evolution to assess whether inferred shifts are more common on the human branch than on other primate lineages.

Results: We identified four traits with strong evidence for an evolutionary increase on the human lineage (amylase, haematocrit, phosphorus and monocytes) and one trait with strong evidence for decrease (neutrophilic bands). Humans exhibited more cases of distinct evolutionary change than other primates.

Conclusions and Implications: Human physiology has undergone increased evolutionary change compared to other primates. Long distance running may have contributed to increases in haematocrit and mean corpuscular haemoglobin concentration, while dietary changes are likely related to increases in amylase. In accordance with the pathogen load hypothesis, human monocyte levels were increased, but many other immune-related measures were not. Determining the mechanisms underlying conspicuous evolutionary change in these traits may provide new insights into human disease.
C1 [Vining, Alexander Q.; Nunn, Charles L.] Duke Univ, Dept Evolutionary Anthropol, Durham, NC USA.
[Nunn, Charles L.] Duke Univ, Duke Global Hlth Inst, Durham, NC USA.
[Nunn, Charles L.] Triangle Ctr Evolutionary Med, Durham, NC USA.
C3 Duke University; Duke University
RP Vining, AQ (autor correspondente), Duke Univ, 107 Biol Sci Bldg,Box 90383, Durham, NC 27709 USA.
EM av127@duke.edu
FU Direct For Social, Behav & Economic Scie; Division Of Behavioral and Cognitive Sci [1355902] Funding Source: National Science Foundation
CR [Anonymous], 1962, J CHEM EDUC
[Anonymous], 1991, COMP METHOD EVOLUTIO
Arnold C, 2010, EVOL ANTHROPOL, V19, P114, DOI 10.1002/evan.20251
Barrett R, 1998, ANNU REV ANTHROPOL, V27, P247, DOI 10.1146/annurev.anthro.27.1.247
BENJAMINI Y, 1995, J R STAT SOC B, V57, P289, DOI 10.1111/j.2517-6161.1995.tb02031.x
Blackwell AD, 2016, ANN HUM BIOL, V43, P382, DOI 10.1080/03014460.2016.1189963
Bramble DM, 2004, NATURE, V432, P345, DOI 10.1038/nature03052
BROTHERHOOD J, 1975, CLIN SCI MOL MED, V48, P139, DOI 10.1042/cs0480139
Deaner RO, 1999, P ROY SOC B-BIOL SCI, V266, P687, DOI 10.1098/rspb.1999.0690
FELSENSTEIN J, 1985, AM NAT, V125, P1, DOI 10.1086/284325
Fumagalli M, 2011, PLOS GENET, V7, DOI 10.1371/journal.pgen.1002355
Garamszegi LZ, 2014, MODERN PHYLOGENETIC
Garland T, 2000, AM NAT, V155, P346, DOI 10.1086/303327
Hansen TF, 2005, EVOLUTION, V59, P2063

Ho LST, 2014, METHODS ECOL EVOL, V5, P1133, DOI 10.1111/2041-210X.12285
HUEY RB, 1987, EVOLUTION, V41, P1098, DOI 10.1111/j.1558-5646.1987.tb05879.x
International Species Information System, 2002, PHYS REF VAL
Isler K, 2008, J HUM EVOL, V55, P967, DOI 10.1016/j.jhevol.2008.08.004
Lindenfors P, 2010, J EVOLUTION BIOL, V23, P1183, DOI 10.1111/j.1420-9101.2010.01983.x
MCPEEK MA, 1995, AM NAT, V145, P686, DOI 10.1086/285763
Meeusen ENT, 2000, PARASITOL TODAY, V16, P95, DOI 10.1016/S0169-4758(99)01607-5
Nunn C.L., 2014, Modern Phylogenetic Comparative Methods and Their Application in Evolutionary Biology, P481
Nunn CL, 2015, EVOLUTION, V69, P245, DOI 10.1111/evo.12520
Nunn CharlesL., 2011, COMP APPROACH EVOLUT
Organ C, 2011, P NATL ACAD SCI USA, V108, P14555, DOI 10.1073/pnas.1107806108
Organ CL, 2007, NATURE, V446, P180, DOI 10.1038/nature05621
Perry GH, 2007, NAT GENET, V39, P1256, DOI 10.1038/ng2123
R Development Core Team, 2014, A Language and Environment for Statistical Computing
Rodgers GP, 2014, CONNS CURRENT THERAP
Samson DR, 2015, EVOL ANTHROPOL, V24, P225, DOI 10.1002/evan.21464
Sivakumaran S, 2011, AM J HUM GENET, V89, P607, DOI 10.1016/j.ajhg.2011.10.004
STEARNS SC, 1989, FUNCT ECOL, V3, P259, DOI 10.2307/2389364
STEVENSON ET, 1994, J APPL PHYSIOL, V77, P1691, DOI 10.1152/jappl.1994.77.4.1691
Uyeda JC, 2014, SYST BIOL, V63, P902, DOI 10.1093/sysbio/syu057
West GB, 1997, SCIENCE, V276, P122, DOI 10.1126/science.276.5309.122
Witko-Sarsat V, 2000, LAB INVEST, V80, P617, DOI 10.1038/labinvest.3780067
Wolfe ND, 2007, NATURE, V447, P279, DOI 10.1038/nature05775
NR 37
TC 8
Z9 8
U1 0
U2 1
PU OXFORD UNIV PRESS
PI OXFORD
PA GREAT CLARENDON ST, OXFORD OX2 6DP, ENGLAND
SN 2050-6201
J9 EVOL MED PUBLIC HLTH
JI Evol. Med. Public Health.
PY 2016
IS 1
BP 312
EP 324
DI 10.1093/emph/eow026
PG 13
WC Evolutionary Biology; Public, Environmental & Occupational Health
WE Science Citation Index Expanded (SCI-EXPANDED)
SC Evolutionary Biology; Public, Environmental & Occupational Health
GA DZ5TY

UT WOS:000385925600029
PM 27615376
OA Green Submitted, gold, Green Published
DA 2025-05-07
ER

PT J
AU Gupta, MK
   Gouda, G
   Vadde, R
AF Gupta, Manoj Kumar
   Gouda, Gayatri
   Vadde, Ramakrishna
TI Relation Between Obesity and Type 2 Diabetes: Evolutionary Insights, Perspectives and Controversies
SO CURRENT OBESITY REPORTS
LA English
DT Review
DE Insulin resistance; Evolutionary origin; Thrifty hypothesis; Metabolic disorders; Drifty hypothesis
ID BODY-MASS INDEX; GENOME-WIDE ASSOCIATION; HIGH-FAT DIET; INDUCED INSULIN-RESISTANCE; ADIPOSE-TISSUE; PIMA-INDIANS; GLUCOSE-HOMEOSTASIS; THRIFTY PHENOTYPE; RISK-FACTORS; PHYSICAL-ACTIVITY
AB Purpose of ReviewSince the mid-twentieth century, obesity and its related comorbidities, notably insulin resistance (IR) and type 2 diabetes (T2D), have surged. Nevertheless, their underlying mechanisms remain elusive. Evolutionary medicine (EM) sheds light on these issues by examining how evolutionary processes shape traits and diseases, offering insights for medical practice. This review summarizes the pathogenesis and genetics of obesity-related IR and T2D. Subsequently, delving into their evolutionary connections. Addressing limitations and proposing future research directions aims to enhance our understanding of these conditions, paving the way for improved treatments and prevention strategies.Recent FindingsSeveral evolutionary hypotheses have been proposed to unmask the origin of obesity-related IR and T2D, e.g., the "thrifty genotype" hypothesis suggests that certain "thrifty genes" that helped hunter-gatherer populations efficiently store energy as fat during feast-famine cycles are now maladaptive in our modern obesogenic environment. The "drifty genotype" theory suggests that if thrifty genes were advantageous, they would have spread widely, but proposes genetic drift instead. The "behavioral switch" and "carnivore connection" hypotheses propose insulin resistance as an adaptation for a brain-dependent, low-carbohydrate lifestyle. The thrifty phenotype theory suggests various metabolic outcomes shaped by genes and environment during development. However, the majority of these hypotheses lack experimental validation. Understanding why ancestral advantages now predispose us to diseases may aid in drug development and prevention of disease.SummaryEM helps us to understand the evolutionary relation between obesity-related IR and T2D. But still gaps and contradictions persist. Further interdisciplinary research is required to elucidate complete mechanisms.
C1 [Gupta, Manoj Kumar; Vadde, Ramakrishna] Yogi Vemana Univ, Dept Biotechnol & Bioinformat, Kadapa 516005, Andhra Pradesh, India.

[Gouda, Gayatri] ICAR Natl Rice Res Inst, Cuttack 753006, Odisha, India.
C3 Yogi Vemana University; Indian Council of Agricultural Research (ICAR);
ICAR - National Rice Research Institute
RP Gupta, MK (autor correspondente), Yogi Vemana Univ, Dept Biotechnol & Bioinformat, Kadapa 516005, Andhra Pradesh, India.
EM mkgupta.bioinfo@gmail.com
RI Vadde, Ramakrishna/AAJ-5349-2020
FU MakerSpace Bonn e.V (Germany)
FX Authors would like to thank Mr. Jan Benzenberg, Operations Manager at Deutsche Telekom, (Germany), and MakerSpace Bonn e.V (Germany), for providing a computation facility for the literature survey.
CR Akhtar J, 2022, ISCIENCE, V25, DOI 10.1016/j.isci.2022.105561
Al-Jawadi AA, 2021, BMC RES NOTES, V14, DOI 10.1186/s13104-021-05823-1
Alcock J., 2011, Evol Educ Outreach, P574, DOI [DOI 10.1007/S12052-011-0362-1, https://doi.org/10.1007/s12052-011-0362-1]
Chavoya-Guardado MA, 2022, NUTRIENTS, V14, DOI 10.3390/nu14142887
Alves JGB, 2024, J PEDIAT-BRAZIL, V100, pS4, DOI 10.1016/j.jped.2023.08.007
Ang MY, 2023, J HUM GENET, V68, P823, DOI 10.1038/s10038-023-01189-3
[Anonymous], 2017, JAMA-J AM MED ASSOC, V318, P603, DOI 10.1001/jama.2017.10120
Aoshiba K, 2015, RESPIRATION, V89, P243, DOI 10.1159/000369861
Baker P., 2022, MIGRATION MOBILITY, P209, DOI [10.4324/9781003334019-13, DOI 10.4324/9781003334019-13]
Basile AJ, 2021, EVOL MED PUBLIC HLTH, V9, P157, DOI 10.1093/emph/eoab010
Basile KJ, 2014, INT J ENDOCRINOL, V2014, DOI 10.1155/2014/769671
Benton ML, 2021, NAT REV GENET, V22, P269, DOI 10.1038/s41576-020-00305-9
Berndt SI, 2013, NAT GENET, V45, P501, DOI 10.1038/ng.2606
Bertile F, 2023, COMMUN BIOL, V6, DOI 10.1038/s42003-023-05458-x
Bindon JR, 1997, AM J PHYS ANTHROPOL, V104, P201, DOI 10.1002/(SICI)1096-8644(199710)104:2<201::AID-AJPA6>3.3.CO;2-F
Blüher M, 2018, ENDOCRINOL SER, P1, DOI 10.1007/978-3-319-44433-8_1
BOGARDUS C, 1984, J CLIN INVEST, V74, P1238, DOI 10.1172/JCI111533
Bogardus C, 1990, Int J Obes, V14 Suppl 1, P5
BONADONNA RC, 1990, METABOLISM, V39, P452, DOI 10.1016/0026-0495(90)90002-T
Bonàs-Guarch S, 2018, NAT COMMUN, V9, DOI 10.1038/s41467-017-02380-9
Brand-Miller JC, 2012, J OBES, V2012, DOI 10.1155/2012/258624
Brüne M, 2013, BMC MED, V11, DOI 10.1186/1741-7015-11-116
BURGHEN GA, 1980, J CLIN ENDOCR METAB, V50, P113, DOI 10.1210/jcem-50-1-113
Chadt A, 2020, PFLUG ARCH EUR J PHY, V472, P1273, DOI 10.1007/s00424-020-02417-x
CHANG S, 1990, AM J PHYSIOL, V259, pR1103, DOI 10.1152/ajpregu.1990.259.6.R1103
Chang YC, 2010, OBESITY, V18, P1404, DOI 10.1038/oby.2009.390
Chen Y, 2018, ENDOCR CONNECT, V7, pR187, DOI 10.1530/EC-18-0121
Cheng WY, 2022, NUTRIENTS, V14, DOI 10.3390/nu14112250
Choi WG, 2021, J CLIN INVEST INTERN
Cooper MD, 2010, NAT REV IMMUNOL, V10, P2, DOI 10.1038/nri2686

Cuthill IC, 2000, BEHAV ECOL, V11, P189, DOI 10.1093/beheco/11.2.189
Dabelea D, 1998, DIABETOLOGIA, V41, P904, DOI 10.1007/s001250051006
Dale PO, 1998, HUM REPROD, V13, P567, DOI 10.1093/humrep/13.3.567
DEKABAN AS, 1978, ANN NEUROL, V4, P345, DOI 10.1002/ana.410040410
Dina C, 2007, NAT GENET, V39, P724, DOI 10.1038/ng2048
Djalalinia S, 2015, PAK J MED SCI, V31, P239, DOI 10.12669/pjms.311.7033
Dunaif Andrea, 1997, P249
El-Bakry HA, 1999, PHYSIOL BEHAV, V68, P87, DOI 10.1016/S0031-9384(99)00151-1
Ellison Peter T., 2014, Evolution Medicine and Public Health, P93, DOI 10.1093/emph/eou015
Esparza J, 2000, INT J OBESITY, V24, P55, DOI 10.1038/sj.ijo.0801085
Morales PE, 2017, J DIABETES RES, V2017, DOI 10.1155/2017/1789395
Ezzati M, 2017, LANCET, V390, P2627, DOI [10.1016/S0140-6736(17)32129-3, 10.1016/s0140-6736(17)32129-3]
Fitipaldi H, 2023, HUM MOL GENET, V32, P520, DOI 10.1093/hmg/ddac245
Frayling TM, 2007, SCIENCE, V316, P889, DOI 10.1126/science.1141634
Freathy RM, 2008, DIABETES, V57, P1419, DOI 10.2337/db07-1466
Frisancho AR, 2003, AM J HUM BIOL, V15, P522, DOI 10.1002/ajhb.10191
Genne-Bacon Elizabeth A., 2014, Yale Journal of Biology and Medicine, V87, P99
Girgis CM, 2012, TRENDS ENDOCRIN MET, V23, P372, DOI 10.1016/j.tem.2012.05.003
Domínguez-Cruz MG, 2018, GENE, V677, P324, DOI 10.1016/j.gene.2018.08.041
Goh KP, 2022, NUTR THERAPEUTIC INT, P193
Goodarzi MO, 2018, LANCET DIABETES ENDO, V6, P223, DOI 10.1016/S2213-8587(17)30200-0
Gorlov IP, 2011, CLIN GENET, V79, P199, DOI 10.1111/j.1399-0004.2010.01535.x
GOSLER AG, 1995, NATURE, V377, P621, DOI 10.1038/377621a0
Grant RW, 2015, AM J PHYSIOL-ENDOC M, V309, pE205, DOI 10.1152/ajpendo.00053.2015
Grant SFA, 2006, NAT GENET, V38, P320, DOI 10.1038/ng1732
Grarup N, 2014, DIABETOLOGIA, V57, P1528, DOI 10.1007/s00125-014-3270-4
Greisen S, 2001, HUM REPROD, V16, P2061, DOI 10.1093/humrep/16.10.2061
Grunspan DZ, 2018, EVOL MED PUBLIC HLTH, P13, DOI 10.1093/emph/eox025
Gupta MK, 2023, PHARMACOL THERAPEUT, V250, DOI 10.1016/j.pharmthera.2023.108508
Gupta MK, 2023, FRONT GENET, V14, DOI 10.3389/fgene.2023.1091575
Gupta MK, 2020, GENETICA, V148, P269, DOI 10.1007/s10709-020-00101-7
Gupta MK, 2021, J BIOMOL STRUCT DYN, V39, P1447, DOI 10.1080/07391102.2020.1733666
Gupta MK, 2020, J BIOMOL STRUCT DYN, V38, P137, DOI 10.1080/07391102.2019.1567391
Hales CN, 2001, BRIT MED BULL, V60, P5, DOI 10.1093/bmb/60.1.5
Hannon SC, 2021, NUTRIENTS, V13, DOI 10.3390/nu13124505
HANSON RL, 1995, AM J HUM GENET, V57, P160
He Q, 2011, AM J PHYSIOL-ENDOC M, V300, pE877, DOI 10.1152/ajpendo.00626.2010
Hochberg ME, 2013, EVOL APPL, V6, P134, DOI 10.1111/eva.12033

Hocking S, 2023, REV ENDOCR METAB DIS, V24, P951, DOI 10.1007/s11154-023-09808-2
Homburg Roy, 2003, Reprod Biol Endocrinol, V1, P109
Hood Ernie, 2008, Evolution (N Y), V1, P114, DOI 10.1007/s12052-008-0036-9
HOUGHTON P, 1990, ANN HUM BIOL, V17, P19, DOI 10.1080/03014469000000752
Inadera H, 2013, ENVIRON HEALTH PREV, V18, P185, DOI 10.1007/s12199-013-0328-8
Inandiklioglu N, 2021, J PEDIATR GENET, V10, P9, DOI 10.1055/s-0040-1713154
Itani SI, 2002, DIABETES, V51, P2005, DOI 10.2337/diabetes.51.7.2005
Ji H, 2007, METABOLISM, V56, P1124, DOI 10.1016/j.metabol.2007.04.006
Jiao JJ, 2023, NUTRIENTS, V15, DOI 10.3390/nu15112556
Joseph A, 2021, J DIABETES RES, V2021, DOI 10.1155/2021/5123241
Kagawa Y, 2002, BIOCHEM BIOPH RES CO, V295, P207, DOI 10.1016/S0006-291X(02)00680-0
Kahn BB, 2000, J CLIN INVEST, V106, P473, DOI 10.1172/JCI10842
Kalantari Naser, 2016, ARYA Atheroscler, V12, P281
Karin O, 2016, MOL SYST BIOL, V12, DOI 10.15252/msb.20167216
Kehl R J, 1996, J Matern Fetal Med, V5, P273
Kimura M., 1983, The neutral theory of molecular evolution, DOI [DOI 10.1017/CBO9780511623486, 10.1017/CBO9780511623486]
KNOWLER WC, 1990, DIABETES METAB REV, V6, P1, DOI 10.1002/dmr.5610060101
Koliaki C, 2023, CURR OBES REP, V12, P514, DOI 10.1007/s13679-023-00527-y
Kopp R, 2016, INT J OBESITY, V40, P824, DOI 10.1038/ijo.2015.240
Król E, 2005, J EXP BIOL, V208, P571, DOI 10.1242/jeb.01429
Kunz I, 2002, INT J OBESITY, V26, P150, DOI 10.1038/sj.ijo.0801869
Lenover MB, 2024, EVOL ANTHROPOL, V33, DOI 10.1002/evan.22010
Li YY, 2018, FRONT GENET, V9, DOI 10.3389/fgene.2018.00342
Lindsay R, 2001, BRIT MED BULL, V60, P21, DOI 10.1093/bmb/60.1.21
Litman GW, 2007, NAT IMMUNOL, V8, P547, DOI 10.1038/ni0607-547
Liu CT, 2013, PLOS GENET, V9, DOI 10.1371/journal.pgen.1003681
Locke AE, 2015, NATURE, V518, P197, DOI 10.1038/nature14177
Luo LP, 2016, J ENDOCRINOL, V231, pR77, DOI 10.1530/JOE-16-0211
Lyssenko V, 2008, NEW ENGL J MED, V359, P2220, DOI 10.1056/NEJMoa0801869
Marra M, 2004, ANN NUTR METAB, V48, P189, DOI 10.1159/000079556
McAllister EJ, 2009, CRIT REV FOOD SCI, V49, P868, DOI 10.1080/10408390903372599
Meeks KAC, 2021, HUM MOL GENET, V30, pR110, DOI 10.1093/hmg/ddaa238
Metzger BE, 1998, DIABETES CARE, V21, pB161
Meyre D, 2009, NAT GENET, V41, P157, DOI 10.1038/ng.301
MILLER JCB, 1994, DIABETOLOGIA, V37, P1280, DOI 10.1007/s001250050246
Minster RL, 2016, NAT GENET, V48, P1049, DOI 10.1038/ng.3620
Moller DE, 2005, ANNU REV MED, V56, P45, DOI 10.1146/annurev.med.56.082103.104751
Anderson JM, 2020, ECOL EVOL, V10, P10930, DOI 10.1002/ece3.6825
Monda KL, 2013, NAT GENET, V45, P690, DOI 10.1038/ng.2608
Musselman LP, 2018, J EXP BIOL, V221, DOI 10.1242/jeb.163881
Myles S, 2011, BMC MED GENET, V12, DOI 10.1186/1471-2350-12-10

Natterson-Horowitz B, 2023, Front Sci, V1, DOI 10.3389/fsci.2023.997136
NEEL JV, 1962, AM J HUM GENET, V14, P353
Nesse RM, 2019, PHILOS T R SOC B, V374, DOI 10.1098/rstb.2019.0288
Nesse RM, 2010, P NATL ACAD SCI USA, V107, P1800, DOI 10.1073/pnas.0906224106
Nesse RM, 2004, PHILOS T R SOC B, V359, P1333, DOI 10.1098/rstb.2004.1511
Ng MCY, 2008, DIABETES, V57, P2226, DOI 10.2337/db07-1583
Norris JM, 2012, ARTERIOSCL THROM VAS, V32, P2091, DOI 10.1161/ATVBAHA.112.255463
Nuttall Frank Q, 2015, Nutr Today, V50, P117
O'Dea K, 1995, Asia Pac J Clin Nutr, V4, P339
Okoduwa SIR, 2017, PLOS ONE, V12, DOI 10.1371/journal.pone.0170971
Oliveri A, 2024, NAT GENET, V56, P212, DOI 10.1038/s41588-023-01625-2
Ormazabal V, 2018, CARDIOVASC DIABETOL, V17, DOI 10.1186/s12933-018-0762-4
Park SS, 2020, INT J MOL SCI, V21, DOI 10.3390/ijms21061949
Paul HA, 2016, SCI REP-UK, V6, DOI 10.1038/srep20683
Peacock WL, 2001, PHYSIOL BEHAV, V74, P65, DOI 10.1016/S0031-9384(01)00533-9
Perry GH, 2021, ELIFE, V10, DOI 10.7554/eLife.69398
PETTITT DJ, 1993, DIABETES CARE, V16, P310, DOI 10.2337/diacare.16.1.310
Pierce WD, 2010, INT J OBESITY, V34, P589, DOI 10.1038/ijo.2009.294
Ramos-Molina B, 2016, PROG MOL BIOL TRANSL, V140, P47, DOI 10.1016/bs.pmbts.2015.12.001
Ravussin E, 1999, INT J OBESITY, V23, P37, DOI 10.1038/sj.ijo.0800793
Reaven GM, 1998, DIABETOLOGIA, V41, P482, DOI 10.1007/s001250050933
Reinehr T, 2014, HORM RES PAEDIAT, V81, P391, DOI 10.1159/000358328
Rosen ED, 2006, NATURE, V444, P847, DOI 10.1038/nature05483
Ruiz JR, 2010, ARCH PEDIAT ADOL MED, V164, P328, DOI 10.1001/archpediatrics.2010.29
Ruze R, 2023, FRONT ENDOCRINOL, V14, DOI 10.3389/fendo.2023.1161521
Schulz LO, 2006, DIABETES CARE, V29, P1866, DOI 10.2337/dc06-0138
Schulz LO, 2015, CURR OBES REP, V4, P92, DOI 10.1007/s13679-014-0132-9
Ségurel L, 2013, EUR J HUM GENET, V21, P1146, DOI 10.1038/ejhg.2012.295
Sellayah D, 2014, ENDOCRINOLOGY, V155, P1573, DOI 10.1210/en.2013-2103
Sherman A, 2011, J GEN PHYSIOL, V138, P13, DOI 10.1085/jgp.201110668
Shulman GI, 2014, NEW ENGL J MED, V371, P1131, DOI 10.1056/NEJMra1011035
Sivadas A, 2024, BMJ OPEN DIAB RES CA, V12, DOI 10.1136/bmjdrc-2023-003769
Skorobogatko Y, 2018, P NATL ACAD SCI USA, V115, P7819, DOI 10.1073/pnas.1801050115
Smith GI, 2019, J CLIN INVEST, V129, P3978, DOI 10.1172/JCI129186
Smolinska K, 2024, SCI REP-UK, V14, DOI 10.1038/s41598-024-53695-9
South A., 2017, rnaturalearth: World Map Data from Natural Earth. R package version 0.1.0
Speakman JR, 2008, INT J OBESITY, V32, P1611, DOI 10.1038/ijo.2008.161
Speakman JR, 2007, CELL METAB, V6, P5, DOI 10.1016/j.cmet.2007.06.004
Stijnen P, 2016, ENDOCR REV, V37, P347, DOI 10.1210/er.2015-1117
Stipp D, 2011, AGING-US, V3, P85, DOI 10.18632/aging.100286

Suzuki K., 2023, SERV HLTH SCI, DOI [10.1101/2023.03.31.23287839, DOI 10.1101/2023.03.31.23287839]
Suzuki K, 2024, NATURE, V627, DOI 10.1038/s41586-024-07019-6
Swinburn BA, 1996, DIABETIC MED, V13, P695, DOI 10.1002/(SICI)1096-9136(199608)13:8<695::AID-DIA170>3.0.CO;2-#
Takeuchi F, 2018, NAT COMMUN, V9, DOI 10.1038/s41467-018-07345-0
Tam V, 2019, NAT REV GENET, V20, P467, DOI 10.1038/s41576-019-0127-1
Tataranni PA, 1998, DIABETES METAB, V24, P108
Toprak S, 2001, HORM RES, V55, P65, DOI 10.1159/000049972
Tumova J, 2016, PHYSIOL RES, V65, P193, DOI 10.33549/physiolres.932993
Unger RH, 2006, NAT MED, V12, P56, DOI 10.1038/nm0106-56
Unoki H, 2008, NAT GENET, V40, P1098, DOI 10.1038/ng.208
Vähämiko S, 2019, EUR J NUTR, V58, P367, DOI 10.1007/s00394-017-1601-1
Vámos A, 2023, FRONT CELL DEV BIOL, V11, DOI 10.3389/fcell.2023.1155673
van Herpen NA, 2008, PHYSIOL BEHAV, V94, P231, DOI 10.1016/j.physbeh.2007.11.049
Venkatachalapathy P, 2021, PHARMACOGEN PERS MED, V14, P1441, DOI 10.2147/PGPM.S329787
Venniyoor A, 2020, FRONT NUTR, V7, DOI 10.3389/fnut.2020.00081
Wang CCL, 2004, DIABETES, V53, P2735, DOI 10.2337/diabetes.53.11.2735
Wang GY, 2014, J R SOC INTERFACE, V11, DOI 10.1098/rsif.2014.0892
Warbrick-Smith J, 2006, P NATL ACAD SCI USA, V103, P14045, DOI 10.1073/pnas.0605225103
Waterson MJ, 2015, CELL METAB, V22, P962, DOI 10.1016/j.cmet.2015.09.026
Watve MG, 2007, BMC EVOL BIOL, V7, DOI 10.1186/1471-2148-7-61
Wells JCK, 2007, BIOL REV, V82, P143, DOI 10.1111/j.1469-185X.2006.00007.x
Wen WQ, 2012, NAT GENET, V44, P307, DOI 10.1038/ng.1087
Westbury S, 2023, CURR OBES REP, V12, P10, DOI 10.1007/s13679-023-00495-3
WHO, 2022, Prevalence of obesity among adults, BMI ≥ 30, age-standardized Estimates by country
Wickham H, 2011, WIRES COMPUT STAT, V3, P180, DOI 10.1002/wics.147
WILLIAMS RC, 1992, AM J HUM GENET, V51, P101
Wojcik GL, 2019, NATURE, V570, P514, DOI 10.1038/s41586-019-1310-4
Wondmkun YT, 2020, DIABET METAB SYND OB, V13, P3611, DOI 10.2147/DMSO.S275898
World Health Organization, 2022, IND RAIS FAST BLOOD
Wu HZ, 2020, CIRC RES, V126, P1549, DOI 10.1161/CIRCRESAHA.119.315896
Wu YG, 2024, J BIOPHARM STAT, DOI 10.1080/10543406.2024.2358803
Xu LN, 2018, PHARMACOL RES, V130, P451, DOI 10.1016/j.phrs.2018.01.015
Xue A, 2018, NAT COMMUN, V9, DOI 10.1038/s41467-018-04951-w
Xue HX, 2015, INT J CLIN EXP PATHO, V8, P13505
Yamauchi T, 2010, NAT GENET, V42, P864, DOI 10.1038/ng.660
Yasuda K, 2008, NAT GENET, V40, P1092, DOI 10.1038/ng.207
Zhou B, 2016, LANCET, V387, P1513, DOI 10.1016/S0140-6736(16)00618-8
Zhu Y, 2021, NAT COMMUN, V12, DOI 10.1038/s41467-021-25025-4
ZIMMET PZ, 1992, DIABETES CARE, V15, P232, DOI 10.2337/diacare.15.2.232
ZURLO F, 1990, AM J PHYSIOL, V259, pE650
NR 190
TC 4

Z9 4
U1 7
U2 10
PU SPRINGER
PI NEW YORK
PA ONE NEW YORK PLAZA, SUITE 4600, NEW YORK, NY, UNITED STATES
SN 2162-4968
J9 CURR OBES REP
JI Curr. Obes. Rep.
PD SEP
PY 2024
VL 13
IS 3
BP 475
EP 495
DI 10.1007/s13679-024-00572-1
EA JUN 2024
PG 21
WC Endocrinology & Metabolism; Nutrition & Dietetics
WE Science Citation Index Expanded (SCI-EXPANDED)
SC Endocrinology & Metabolism; Nutrition & Dietetics
GA C0N6Z
UT WOS:001243261900002
PM 38850502
DA 2025-05-07
ER

PT J
AU Straub, RH
AF Straub, Rainer H.
TI Insulin resistance, selfish brain, and selfish immune system: an
   evolutionarily positively selected program used in chronic
inflammatory
   diseases
SO ARTHRITIS RESEARCH & THERAPY
LA English
DT Review
ID BETA-CELL FUNCTION; OBESITY-RELATED HYPERTENSION; ACTIVE
   RHEUMATOID-ARTHRITIS; NECROSIS-FACTOR-ALPHA; METABOLIC SYNDROME;
   GLUCOSE-METABOLISM; MENTAL STRESS; JOB STRAIN; INFLIXIMAB TREATMENT;
   DIABETES-MELLITUS
AB Insulin resistance (IR) is a general phenomenon of many physiological
states, disease states, and diseases. IR has been described in diabetes
mellitus, obesity, infection, sepsis, trauma, painful states such as
postoperative pain and migraine, schizophrenia, major depression, chronic
mental stress, and others. In arthritis, abnormalities of glucose
homeostasis were described in 1920; and in 1950 combined glucose and
insulin tests unmistakably demonstrated IR. The phenomenon is now
described in rheumatoid arthritis, systemic lupus erythematosus,
ankylosing spondylitis, polymyalgia rheumatica, and others. In chronic
inflammatory diseases, cytokine-neutralizing strategies normalize insulin
sensitivity. This paper delineates that IR is either based on
inflammatory factors (activation of the immune/repair system) or on the
brain (mental activation via stress axes). Due to the selfishness of the

immune system and the selfishness of the brain, both can induce IR
independent of each other. Consequently, the immune system can block the
brain (for example, by sickness behavior)and the brain can block the
immune system (for example, stress-induced immune system alterations).
Based on considerations of evolutionary medicine, it is discussed that
obesity per se is not a disease. Obesity-related IR depends on provoking
factors from either the immune system or the brain. Chronic inflammation
and/or stress axis activation are thus needed for obesity-related IR. Due
to redundant pathways in stimulating IR, a simple one factor-neutralizing
strategy might help in chronic inflammatory diseases (inflammation is the
key), but not in obesity-related IR. The new considerations towards IR
are interrelated to the published theories of IR (thrifty genotype,
thrifty phenotype, and others).
C1 Univ Hosp, Dept Internal Med, Div Rheumatol, Lab Expt Rheumatol &
Neuroendocrine Immunol, D-93042 Regensburg, Germany.
C3 University of Regensburg
RP Straub, RH (autor correspondente), Univ Hosp, Dept Internal Med, Div
Rheumatol, Lab Expt Rheumatol & Neuroendocrine Immunol, D-93042
Regensburg, Germany.
EM rainer.straub@ukr.de
OI Straub, Rainer H/0000-0003-1165-4555
FU Horizon Pharma Inc.
FX This supplement was proposed, developed and commissioned by Arthritis
Research & Therapy and was funded by an educational grant from Horizon
Pharma Inc. All published articles were independently prepared by the
authors and have undergone peer review in accordance with the
journal's
standard policies and processes. Horizon Pharma Inc had no input into
the topics covered or the articles themselves. The Supplement Editor
was
appointed by the journal and declares that they have no competing
interests.
CR Abdul-Ghani MA, 2007, DIABETES CARE, V30, P89, DOI 10.2337/dc06-1519
Agardh EE, 2003, DIABETES CARE, V26, P719, DOI
10.2337/diacare.26.3.719
Aggarwal B, 2009, J GEN INTERN MED, V24, P93, DOI 10.1007/s11606-008-
0852-1
Aletaha D, 2010, ARTHRITIS RHEUM-US, V62, P2569, DOI 10.1002/art.27584
[Anonymous], 1989, Energy metabolism in animals and man
ARENDT EC, 1956, J CLIN ENDOCR METAB, V16, P367, DOI 10.1210/jcem-16-
3-367
BERGMAN RN, 1979, AM J PHYSIOL, V236, pE667, DOI
10.1152/ajpendo.1979.236.6.E667
Björntorp P, 2001, LANCET, V358, P1006, DOI 10.1016/S0140-
6736(01)06110-4
Björntorp P, 1999, DIABETES-METAB RES, V15, P427
Block JP, 2009, AM J EPIDEMIOL, V170, P181, DOI 10.1093/aje/kwp104
Borai A, 2011, BMC MED RES METHODOL, V11, DOI 10.1186/1471-2288-11-158
Borella P, 1999, PSYCHONEUROENDOCRINO, V24, P613, DOI 10.1016/S0306-
4530(99)00016-5
Boyer D, 2010, PHILOS T R SOC A, V368, P5645, DOI
10.1098/rsta.2010.0275
Brunner EJ, 2007, AM J EPIDEMIOL, V165, P828, DOI 10.1093/aje/kwk058

Butterfield W. J. H., 1965, Diabetologia, V1, P43, DOI 10.1007/BF01338715
CAHILL GF, 1979, METABOLISM, V28, P389, DOI 10.1016/0026-0495(79)90043-X
Calder PC, 2007, CURR OPIN CLIN NUTR, V10, P531, DOI 10.1097/MCO.0b013e3281e72ad4
Capellino S, 2014, ANN NY ACAD SCI, V1317, P24, DOI 10.1111/nyas.12398
Capellino S, 2010, ANN RHEUM DIS, V69, P1853, DOI 10.1136/ard.2009.119701
Capistrant BD, 2014, AM J GERIAT PSYCHIAT, V22, P766, DOI 10.1016/j.jagp.2013.01.073
Castagnetta LA, 2003, J RHEUMATOL, V30, P2597
Chrousos GP, 2000, INT J OBESITY, V24, pS50, DOI 10.1038/sj.ijo.0801278
CHROUSOS GP, 2006, ANN NEW YORK ACAD SC
CIARALDI TP, 1982, DIABETES, V31, P1016, DOI 10.2337/diacare.31.11.1016
COLLINS JOHN, 1957, MED JOUR AUSTRALIA II, V44, P467
Dallman MF, 2010, TRENDS ENDOCRIN MET, V21, P159, DOI 10.1016/j.tem.2009.10.004
DEFRONZO RA, 1979, AM J PHYSIOL, V237, pE214
DEFRONZO RA, 1988, DIABETES, V37, P667, DOI 10.2337/diab.37.6.667
DeFronzo RA, 2009, DIABETES, V58, P773, DOI 10.2337/db09-9028
Dessein PH, 2006, ARTHRITIS RHEUM-US, V54, P2765, DOI 10.1002/art.22053
Dubreuil M, 2014, RHEUMATOLOGY, V53, P346, DOI 10.1093/rheumatology/ket343
Edwards C, 2012, J CLIN ENDOCR METAB, V97, P1443, DOI 10.1210/jc.2011-2879
Edwards EM, 2012, J OCCUP ENVIRON MED, V54, P1447, DOI 10.1097/JOM.0b013e3182783f27
Ehrhart-Bornstein M, 1998, ENDOCR REV, V19, P101, DOI 10.1210/er.19.2.101
Esquirol Y, 2009, CHRONOBIOL INT, V26, P544, DOI 10.1080/07420520902821176
Fall T, 2014, MOL CELL ENDOCRINOL, V382, P740, DOI 10.1016/j.mce.2012.08.018
FEINGOLD KR, 1992, DIABETES, V41, P97, DOI 10.2337/diab.41.2.S97
Fernández-Real JM, 1999, DIABETOLOGIA, V42, P1367, DOI 10.1007/s001250051451
Ferrer B, 2014, BRAIN BEHAV IMMUN, V40, P121, DOI 10.1016/j.bbi.2014.03.001
Fleischman A, 2008, DIABETES CARE, V31, P289, DOI 10.2337/dc07-1338
Fredman L, 2010, J GERONTOL A-BIOL, V65, P565, DOI 10.1093/gerona/glq025
GARVEY WT, 1986, DIABETES, V35, P258, DOI 10.2337/diabetes.35.3.258
*GEIG PHARM, 1973, WISS TAB
Glass CK, 2012, CELL METAB, V15, P635, DOI 10.1016/j.cmet.2012.04.001
Gluckman PD, 2004, TRENDS ENDOCRIN MET, V15, P183, DOI 10.1016/j.tem.2004.03.002
Goldfine AB, 2011, CLIN CHEM, V57, P162, DOI 10.1373/clinchem.2010.148833
Goldfine AB, 2010, ANN INTERN MED, V152, P346, DOI 10.7326/0003-4819-152-6-201003160-00004

Gonzalez-Gay MA, 2006, CLIN EXP RHEUMATOL, V24, P83
Graham G, 1940, Br Med J, V2, P479
Gregor MF, 2011, ANNU REV IMMUNOL, V29, P415, DOI 10.1146/annurev-immunol-031210-101322
Greisen J, 2001, ANESTHESIOLOGY, V95, P578, DOI 10.1097/00000542-200109000-00007
GRUNBERGER G, 1984, SCIENCE, V223, P932, DOI 10.1126/science.6141638
HALES CN, 1992, DIABETOLOGIA, V35, P595, DOI 10.1093/ije/dyt133
Hales CN, 2001, BRIT MED BULL, V60, P5, DOI 10.1093/bmb/60.1.5
Himsworth HP, 1936, LANCET, V1, P127
Hitze Britta, 2010, Front Neuroenergetics, V2, P7, DOI 10.3389/fnene.2010.00007
Hotamisligil GS, 2006, NATURE, V444, P860, DOI 10.1038/nature05485
Hotamisligil GS, 2008, NAT REV IMMUNOL, V8, P923, DOI 10.1038/nri2449
HOTAMISLIGIL GS, 1993, SCIENCE, V259, P87, DOI 10.1126/science.7678183
Iemitsu M, 2000, MED SCI SPORT EXER, V32, P2067, DOI 10.1097/00005768-200012000-00016
Ikmal SIQS, 2013, INT J ENDOCRINOL, V2013, DOI 10.1155/2013/698567
Jauch-Chara K, 2014, PROG NEUROBIOL, V114, P84, DOI 10.1016/j.pneurobio.2013.12.001
Jin CC, 2013, CELL METAB, V17, P873, DOI 10.1016/j.cmet.2013.05.011
Johnson AMF, 2013, CELL, V152, P673, DOI 10.1016/j.cell.2013.01.041
Joslin EP, 1916, CAN MED ASSOC J, V6, P673
Kaaja R, 2007, INT J CLIN PHARM TH, V45, P394
KASUGA M, 1982, NATURE, V298, P667, DOI 10.1038/298667a0
Keen-Rhinehart E, 2013, FRONT NEUROSCI-SWITZ, V7, DOI 10.3389/fnins.2013.00213
KeltikangasJarvinen L, 1996, METABOLISM, V45, P614, DOI 10.1016/S0026-0495(96)90033-5
Kiecolt-Glaser JK, 2003, P NATL ACAD SCI USA, V100, P9090, DOI 10.1073/pnas.1531903100
Kiortsis DN, 2006, J RHEUMATOL, V33, P921
Kiortsis DN, 2005, ANN RHEUM DIS, V64, P765, DOI 10.1136/ard.2004.026534
Kitano H, 2004, DIABETES, V53, pS6, DOI 10.2337/diabetes.53.suppl_3.S6
Kivimäki M, 2006, SCAND J WORK ENV HEA, V32, P431, DOI 10.5271/sjweh.1049
Korkeila M, 1998, INT J OBESITY, V22, P949, DOI 10.1038/sj.ijo.0800694
KRIEGER DR, 1988, AM J HYPERTENS, V1, P84, DOI 10.1093/ajh/1.1.84
Kuipers RS, 2010, BRIT J NUTR, V104, P1666, DOI 10.1017/S0007114510002679
Kuzawa CW, 1998, YEARB PHYS ANTHROPOL, V41, P177
Landsberg L, 1999, ANN NY ACAD SCI, V892, P84, DOI 10.1111/j.1749-6632.1999.tb07787.x
Landsberg L, 2013, J CLIN HYPERTENS, V15, P14, DOI 10.1111/jch.12049
Larsen CM, 2007, NEW ENGL J MED, V356, P1517, DOI 10.1056/NEJMoa065213
Levitan RD, 2013, CURR PSYCHIAT REP, V15, DOI 10.1007/s11920-013-0408-x
Ley RE, 2006, NATURE, V444, P1022, DOI 10.1038/4441022a
LIEFMANN R, 1950, ACTA MED SCAND, V136, P226
Lutgendorf SK, 1999, J GERONTOL A-BIOL, V54, pM434, DOI 10.1093/gerona/54.9.M434
Mahajan A, 2014, NAT GENET, V46, P234, DOI 10.1038/ng.2897

Mahfoud F, 2011, CIRCULATION, V123, P1940, DOI 10.1161/CIRCULATIONAHA.110.991869
McAlonan GM, 2007, CAN J PSYCHIAT, V52, P241, DOI 10.1177/070674370705200406
Meyer-Hermann ME, 2005, J IMMUNOL, V174, P2489, DOI 10.4049/jimmunol.174.5.2489
MOBERG E, 1994, DIABETOLOGIA, V37, P247
MOLLER DE, 1991, NEW ENGL J MED, V325, P938
Montague CT, 1997, NATURE, V387, P903, DOI 10.1038/43185
Müller N, 2013, PSYCHIAT DANUB, V25, P292
Müller N, 2002, AM J PSYCHIAT, V159, P1029, DOI 10.1176/appi.ajp.159.6.1029
Mullington JM, 2009, PROG CARDIOVASC DIS, V51, P294, DOI 10.1016/j.pcad.2008.10.003
Myers MG, 2012, NATURE, V491, P357, DOI 10.1038/nature11705
Nairne JS, 2010, COGNITIVE PSYCHOL, V61, P1, DOI 10.1016/j.cogpsych.2010.01.005
Nakae J, 2008, FEBS LETT, V582, P54, DOI 10.1016/j.febslet.2007.11.025
Neel JV, 1999, NUTR REV, V57, pS2, DOI 10.1111/j.1753-4887.1999.tb01782.x
NEEL JV, 1962, AM J HUM GENET, V14, P353
Osborn O, 2012, NAT MED, V18, P363, DOI 10.1038/nm.2627
PABST R, 1974, RES EXP MED, V164, P247, DOI 10.1007/BF01851943
Pemberton R, 1920, ARCH INTERN MED, V25, P243, DOI 10.1001/archinte.1920.00090320014003
Peters A, 2004, NEUROSCI BIOBEHAV R, V28, P143, DOI 10.1016/j.neubiorev.2004.03.002
Peters Achim, 2009, Front Neuroenergetics, V1, P2, DOI 10.3389/neuro.14.002.2009
Pongratz G, 2013, NAT REV RHEUMATOL, V9, P117, DOI 10.1038/nrrheum.2012.181
Quine S, 1993, Health Soc Work, V18, P114
Rabinowitch IM, 1924, CAN MED ASSOC J, V14, P481
RANDLE PJ, 1963, LANCET, V1, P785
Rassow J, 2008, BIOCHEMISTRY
REAVEN GM, 1988, DIABETES, V37, P1595, DOI 10.2337/diabetes.37.12.1595
Reaven GM, 1998, DIABETOLOGIA, V41, P482, DOI 10.1007/s001250050933
Reeves KW, 2012, BMC PUBLIC HEALTH, V12, DOI 10.1186/1471-2458-12-685
Rolfe DFS, 1997, PHYSIOL REV, V77, P731, DOI 10.1152/physrev.1997.77.3.731
Root HF, 1929, NEW ENGL J MED, V201, P0201, DOI 10.1056/NEJM192908012010501
Roseboom TJ, 2012, PLACENTA, V33, pE40, DOI 10.1016/j.placenta.2012.07.018
Rosenblat JD, 2014, PROG NEURO-PSYCHOPH, V53, P23, DOI 10.1016/j.pnpbp.2014.01.013
Schenk S, 2008, J CLIN INVEST, V118, P2992, DOI 10.1172/JCI34260
Schultz O, 2010, PLOS ONE, V5, DOI 10.1371/journal.pone.0014328
Schwartz MW, 2004, J CLIN ENDOCR METAB, V89, P5889, DOI 10.1210/jc.2004-0906
Sebert S, 2011, AM J CLIN NUTR, V94, p1953S, DOI 10.3945/ajcn.110.001040
Seematter G, 2000, AM J PHYSIOL-ENDOC M, V279, pE799, DOI 10.1152/ajpendo.2000.279.4.E799

Serlachius A, 2007, PHYSIOL BEHAV, V92, P548, DOI 10.1016/j.physbeh.2007.04.032
SHEN SW, 1970, J CLIN INVEST, V49, P2151, DOI 10.1172/JCI106433
Shoelson SE, 2007, GASTROENTEROLOGY, V132, P2169, DOI 10.1053/j.gastro.2007.03.059
Sjögren E, 2006, BRAIN BEHAV IMMUN, V20, P270, DOI 10.1016/j.bbi.2005.08.001
Spalding KL, 2008, NATURE, V453, P783, DOI 10.1038/nature06902
Stagakis I, 2012, ARTHRITIS RES THER, V14, DOI 10.1186/ar3874
Straub RH, 2010, J INTERN MED, V267, P543, DOI 10.1111/j.1365-2796.2010.02218.x
Straub RH, 2012, J MOL MED, V90, P523, DOI 10.1007/s00109-012-0861-8
Straub RH, 2002, ARTHRITIS RHEUM, V46, P654, DOI 10.1002/art.10177
Straub RH, 1998, J CLIN ENDOCR METAB, V83, P2012, DOI 10.1210/jc.83.6.2012
SVENSON KLG, 1987, METABOLISM, V36, P940, DOI 10.1016/0026-0495(87)90128-4
Taubes G, 2012, NATURE, V492, P155, DOI 10.1038/492155a
Thomsen V, 1936, ACTA MED SCAND, P918
Tsigos C, 1997, NEUROENDOCRINOLOGY, V66, P54, DOI 10.1159/000127219
Tso T, 2004, CLIN RHEUMATOL, V23, P416, DOI 10.1007/s10067-004-0908-5
UCHIDA I, 1988, BRIT J SURG, V75, P557, DOI 10.1002/bjs.1800750618
Vaisse C, 1998, NAT GENET, V20, P113, DOI 10.1038/2407
VANPRAAG HM, 1965, PSYCHOPHARMACOLOGIA, V8, P67, DOI 10.1007/BF00405362
von Känel R, 2011, J AM GERIATR SOC, V59, P2037, DOI 10.1111/j.1532-5415.2011.03634.x
Wolfe RR, 1997, BAILLIERE CLIN ENDOC, V11, P645, DOI 10.1016/S0950-351X(97)80926-3
YALOW RS, 1960, J CLIN INVEST, V39, P1157, DOI 10.1172/JCI104130
Zunhammer M, 2013, PLOS ONE, V8, DOI 10.1371/journal.pone.0084911
NR 144
TC 56
Z9 57
U1 1
U2 21
PU BMC
PI LONDON
PA CAMPUS, 4 CRINAN ST, LONDON N1 9XW, ENGLAND
SN 1478-6354
EI 1478-6362
J9 ARTHRITIS RES THER
JI Arthritis Res. Ther.
PY 2014
VL 16
SU 2
AR S4
DI 10.1186/ar4688
PG 15
WC Rheumatology
WE Science Citation Index Expanded (SCI-EXPANDED)
SC Rheumatology
GA AX9JC
UT WOS:000347218300005

PM 25608958
OA Green Published, gold
DA 2025-05-07
ER

PT J
AU Morris, R
AF Morris, Rick
TI Stranger in a strange land: an optimal-environments account of evolutionary mismatch
SO SYNTHESE
LA English
DT Article
DE Evolutionary mismatch; Developmental mismatch; Evolution; Biology; Mismatch; Adaptation; Environment
ID PALEOLITHIC NUTRITION; VITAMIN-D; ADAPTATIONISM; HYPOTHESIS; PLASTICITY;
ELEPHANTS; RESPONSES; BEHAVIOR; RUBISCO; MYOPIA
AB In evolutionary medicine, researchers characterize some outcomes as evolutionary mismatch. Mismatch problems arise as the result of organisms living in environments to which they are poorly adapted, typically as the result of some rapid environmental change. Depression, anxiety, obesity, myopia, insomnia, breast cancer, dental problems, and numerous other negative health outcomes have all been characterized as mismatch problems. The exact nature of evolutionary mismatch itself is unclear, however. This leads to a lack of clarity about the sorts of problems that evolutionary mismatch can actually explain. Resolving this challenge is important not only for the evolutionary health literature, but also because the notion of evolutionary mismatch involves central concepts in evolutionary biology: fitness, evolution in changing environments, and so forth. In this paper, I examine two characterizations of mismatch currently in the literature. I propose that we conceptualize mismatch as a relation between an optimal environment and an actual environment. Given an organism and its particular physiology, the optimal environment is the environment in which the organism's fitness is maximized: in other words, the optimal environment is that in which the organism's fitness is as high as it can possibly be. The actual environment is the environment in which the organism actually finds itself. To the extent that there is a discordance between the organism's actual and optimal environments, there is an evolutionary mismatch. In the paper, I show that this account of mismatch gives us the right result when other accounts fail, and provides useful targets for investigation.
C1 [Morris, Rick] Univ Calif Davis, Dept Philosophy, Davis, CA 95616 USA.
C3 University of California System; University of California Davis
RP Morris, R (autor correspondente), Univ Calif Davis, Dept Philosophy, Davis, CA 95616 USA.
EM jemorr@ucdavis.edu
CR Aktipis C. Athena, 2015, Evolution Medicine and Public Health, P52, DOI 10.1093/emph/eou028
[Anonymous], 2009, SCI AM, V300, P32
[Anonymous], 1982, ATTACHMENT
Barr RG, 1999, INFANT CRYING BEHAV
Boersma M, 1998, AM NAT, V152, P237, DOI 10.1086/286164

Bouchard F, 2011, STUD HIST PHI PART C, V42, P106, DOI 10.1016/j.shpsc.2010.11.002
Buckley TR, 2009, P ROY SOC B-BIOL SCI, V276, P1055, DOI 10.1098/rspb.2008.1552
Buller DJ, 2006, EVOLUTIONARY PSYCHOL, P197
Carlile Nicholas, 2009, Ecological Management & Restoration, V10, pS124, DOI 10.1111/j.1442-8903.2009.00450.x
Cofnas N, 2016, BIOL PHILOS, V31, P507, DOI 10.1007/s10539-016-9527-1
Cosmides L, 1997, MODULAR NATURE HUMAN
Cournoyea M, 2013, PERSPECT BIOL MED, V56, P36, DOI 10.1353/pbm.2013.0006
EATON SB, 1985, NEW ENGL J MED, V312, P283, DOI 10.1056/NEJM198501313120505
Garson J, 2015, BIOL MIND
Gluckman P., 2006, Mismatch: the lifestyle diseases timebomb
Gluckman PD, 2005, TRENDS ECOL EVOL, V20, P527, DOI 10.1016/j.tree.2005.08.001
Gluckman PD, 2011, BIRTH DEFECTS RES C, V93, P12, DOI 10.1002/bdrc.20198
Gluckman PeterD., 2009, Principles of evolutionary medicine
Hales CN, 2013, INT J EPIDEMIOL, V42, P1215, DOI 10.1093/ije/dyt133
Honan P, 2008, J INSECT CONSERV, V12, P399, DOI 10.1007/s10841-008-9162-5
JACHMANN H, 1995, AFR J ECOL, V33, P230, DOI 10.1111/j.1365-2028.1995.tb00800.x
Jasienska G., 2013, The Fragile Wisdom: An Evolut ionary View on Women's Biology and Health
Keller M. C., 2006, BEHAV BRAIN SCI
Keller MC, 2006, J PERS SOC PSYCHOL, V91, P316, DOI 10.1037/0022-3514.91.2.316
Konner M, 2010, NUTR CLIN PRACT, V25, P594, DOI 10.1177/0884533610385702
LEVINS R, 1962, AM NAT, V96, P361, DOI 10.1086/282245
LEVINS RICHARD, 1968
Li NP, 2018, CURR DIR PSYCHOL SCI, V27, P38, DOI 10.1177/0963721417731378
Lieberman D., 2013, The story of the human body : evolution, health, and disease, VFirst
Lindeberg S., 2010, Food and Western Disease: Health and Nutrition from an Evolutionary Perspective, DOI DOI 10.1002/9781444317176
Lloyd Elisabeth., 2011, Evolutionary Mismatch and What to Do About It: A Basic Tutorial
Lloyd Elisabeth A., 2015, Biology Theory, V10, P343, DOI 10.1007/s13752-015-0214-2
Maner JK, 2010, SOC ISS POLICY REV, V4, P111, DOI 10.1111/j.1751-2409.2010.01019.x
Matthewson J, 2017, J MED PHILOS, V42, P447, DOI 10.1093/jmp/jhx004
MILLIKAN RG, 1989, PHILOS SCI, V56, P288, DOI 10.1086/289488
MILLS SK, 1979, PHILOS SCI, V46, P263, DOI 10.1086/288865
Nesse R.M., 1994, WHY WE GET SICK NEW
Nisbet EG, 2007, GEOBIOLOGY, V5, P311, DOI 10.1111/j.1472-4669.2007.00127.x
ORZACK SH, 1994, AM NAT, V143, P361, DOI 10.1086/285608

   Owens MJ, 2009, AFR J ECOL, V47, P214, DOI 10.1111/j.1365-2028.2008.01001.x
   Pan CW, 2017, PHOTOCH PHOTOBIO SCI, V16, P426, DOI 10.1039/c6pp00292g
   POLLOCK JI, 1987, AM J PHYS ANTHROPOL, V73, P65, DOI 10.1002/ajpa.1330730106
   Priddel D, 2003, BIODIVERS CONSERV, V12, P1391, DOI 10.1023/A:1023625710011
   Ravelli ACJ, 1998, LANCET, V351, P173, DOI 10.1016/S0140-6736(97)07244-9
   Sih A, 2013, ANIM BEHAV, V85, P1077, DOI 10.1016/j.anbehav.2013.02.017
   Sih A, 2010, OIKOS, V119, P610, DOI 10.1111/j.1600-0706.2009.18039.x
   Tabita FR, 2007, MICROBIOL MOL BIOL R, V71, P576, DOI 10.1128/MMBR.00015-07
   Taylor J., 2015, Body By Darwin: How Evolution Shapes Our Health And Transforms Medicine
   Tideman JWL, 2016, EUR J EPIDEMIOL, V31, P491, DOI 10.1007/s10654-016-0128-8
   Tooby J, 2005, CONCEPTUAL FDN EVOLU, P5
   Trevathan WR, 1999, EVOLUTIONARY OBSTET
   Valles SA, 2012, BIOL PHILOS, V27, P241, DOI 10.1007/s10539-011-9305-z
NR 52
TC 3
Z9 3
U1 3
U2 54
PU SPRINGER
PI DORDRECHT
PA VAN GODEWIJCKSTRAAT 30, 3311 GZ DORDRECHT, NETHERLANDS
SN 0039-7857
EI 1573-0964
J9 SYNTHESE
JI Synthese
PD SEP
PY 2020
VL 197
IS 9
SI SI
BP 4021
EP 4046
DI 10.1007/s11229-018-01915-x
PG 26
WC History & Philosophy Of Science; Philosophy
WE Science Citation Index Expanded (SCI-EXPANDED); Social Science Citation Index (SSCI); Arts & Humanities Citation Index (A&HCI)
SC History & Philosophy of Science; Philosophy
GA NB4BY
UT WOS:000560460900018
DA 2025-05-07
ER

PT J
AU Raichlen, DA
   Pontzer, H
   Harris, JA

Mabulla, AZP
Marlowe, FW
Snodgrass, JJ
Eick, G
Berbesque, JC
Sancilio, A
Wood, BM
AF Raichlen, David A.
Pontzer, Herman
Harris, Jacob A.
Mabulla, Audax Z. P.
Marlowe, Frank W.
Snodgrass, J. Josh
Eick, Geeta
Berbesque, J. Colette
Sancilio, Amelia
Wood, Brian M.
TI Physical activity patterns and biomarkers of cardiovascular disease risk
in hunter-gatherers
SO AMERICAN JOURNAL OF HUMAN BIOLOGY
LA English
DT Article
DE exercise; heart-rate; cholesterol; C-reactive protein; blood pressure;
energetics; flex heart rate
ID TOTAL-ENERGY-EXPENDITURE; EVOLUTIONARY PERSPECTIVE; UNITED-STATES; HDL
CHOLESTEROL; HEALTH; ADULTS; POPULATION; EXERCISE; FITNESS; VARIABILITY
AB Objectives: Time spent in moderate-to-vigorous physical activity (MVPA) is a strong predictor of cardiovascular health, yet few humans living in industrialized societies meet current recommendations (150 min/week). Researchers have long suggested that human physiological requirements for aerobic exercise reflect an evolutionary shift to a hunting and gathering foraging strategy, and a recent transition to more sedentary lifestyles likely represents a mismatch with our past in terms of physical activity. The goal of this study is to explore this mismatch by characterizing MVPA and cardiovascular health in the Hadza, a modern hunting and gathering population living in Northern Tanzania.

Methods: We measured MVPA using continuous heart rate monitoring in 46 participants recruited from two Hadza camps. As part of a larger survey of health in the Hadza, we measured blood pressure (n = 198) and biomarkers of cardiovascular health (n = 23) including C-reactive protein, cholesterol (Total, HDL, and LDL), and triglycerides.

Results: We show that Hadza participants spend large amounts of time in MVPA (134.92 +/- 8.6 min/day), and maintain these activity levels across the lifespan. In fact, the Hadza engage in over 14 times as much MVPA as subjects participating in large epidemiological studies in the United States. We found no evidence of risk factors for cardiovascular disease in this population (low prevalence of hypertension across the lifespan, optimal levels for biomarkers of cardiovascular health).

Conclusions: Our results provide evidence that the hunting and gathering foraging strategy involves high levels of MVPA, supporting the evolutionary medicine model for the relationship between MVPA and cardiovascular health.

C1 [Raichlen, David A.] Univ Arizona, Sch Anthropol, Tucson, AZ 85721 USA.
[Pontzer, Herman] Hunter Coll, Dept Anthropol, New York, NY 10065 USA.
[Pontzer, Herman] New York Consortium Evolutionary Primatol, New York, NY USA.
[Harris, Jacob A.] Arizona State Univ, Inst Human Origins, Sch Human Evolut & Social Change, Tempe, AZ 85287 USA.
[Mabulla, Audax Z. P.] Natl Museums Tanzania, Dar Es Salaam, Tanzania.
[Marlowe, Frank W.] Univ Cambridge, Dept Archaeol & Anthropol, Cambridge CB2 1QH, England.
[Snodgrass, J. Josh; Eick, Geeta] Univ Oregon, Dept Anthropol, Eugene, OR 97403 USA.
[Berbesque, J. Colette] Roehampton Univ, Ctr Res Evolutionary Social & Interdisciplinary A, London, England.
[Sancilio, Amelia; Wood, Brian M.] Yale Univ, Dept Anthropol, New Haven, CT 06520 USA.
C3 University of Arizona; City University of New York (CUNY) System; Hunter College (CUNY); Arizona State University; Arizona State University-Tempe; University of Cambridge; University of Oregon; Roehampton University; Yale University
RP Raichlen, DA (autor correspondente), Univ Arizona, Sch Anthropol, Tucson, AZ 85721 USA.
EM raichlen@email.arizona.edu
RI Marlowe, Frank/G-9752-2012; Berbesque, J./AAX-9582-2020; Wood, Brian/AAQ-4410-2020; Mabulla, Audax/ABE-2455-2020; Berbesque, J. Colette/B-7203-2013
OI Pontzer, Herman/0000-0003-2397-6543; Berbesque, J. Colette/0000-0003-0517-9524; Harris, Jacob/0000-0001-5398-7871
FU National Science Foundation [NSF-BCS-0850815, NSF-BCS 1440867, NSF-BCS 1440841, NSF-BCS 1440671]; L.S.B. Leakey Foundation; University of Arizona Bio5 Institute; University of Oregon Richard A. Bray Faculty Fellowship; Division Of Behavioral and Cognitive Sci; Direct For Social, Behav & Economic Scie [1440867] Funding Source: National Science Foundation
FX The authors thank Hadza participants for their involvement in this study. We also thank Carla Wood, Fides Kirei, Shani Msafiri, Ibrahim and Audax Mabulla, Layne Vashro, Charis Jonathan, Ruth Matiyas, Holiness, and Herieth Cleophace for their invaluable assistance in the field. Funding was provided by the National Science Foundation [NSF-BCS-0850815 (HP), NSF-BCS 1440867 (DAR), NSF-BCS 1440841 (HP), NSF-BCS 1440671 (BMW)] The L.S.B. Leakey Foundation (CB, BMW), The University of Arizona Bio5 Institute (DAR), The University of Oregon Richard A. Bray Faculty Fellowship (JJS). The authors declare no conflicts of interest.
CR [Anonymous], 2010, The Hadza: Hunter-Gatherers of Tanzania, DOI DOI 10.1525/J.CTT1PP17Z
[Anonymous], 2008, EVOLUTION HLTH DIS
Archer E, 2011, PROG CARDIOVASC DIS, V53, P387, DOI 10.1016/j.pcad.2011.02.006

Barreira TV, 2016, J SCI MED SPORT, V19, P476, DOI 10.1016/j.jsams.2015.05.004
Blackwell AD, 2011, PLOS NEGLECT TROP D, V5, DOI 10.1371/journal.pntd.0001218
Carlson SA, 2015, PROG CARDIOVASC DIS, V57, P315, DOI 10.1016/j.pcad.2014.08.002
Cordain L, 1998, INT J SPORTS MED, V19, P328, DOI 10.1055/s-2007-971926
Cordain L, 2005, AM J CLIN NUTR, V82, P483, DOI 10.1093/ajcn/82.2.483a
Donato LJ, 2015, CLIN BIOCHEM, V48, P174, DOI 10.1016/j.clinbiochem.2014.11.003
Dufour DL, 2008, AM J HUM BIOL, V20, P249, DOI 10.1002/ajhb.20764
Dugas LR, 2014, BMC PUBLIC HEALTH, V14, DOI 10.1186/1471-2458-14-882
Eaton SB, 2003, COMP BIOCHEM PHYS A, V136, P153, DOI 10.1016/S1095-6433(03)00208-3
EATON SB, 1988, THE AMERICAN JOURNAL, V84, P739
Ekelund U, 2015, AM J CLIN NUTR, V101, P613, DOI 10.3945/ajcn.114.100065
Ferraro JV, 2013, PLOS ONE, V8, DOI 10.1371/journal.pone.0062174
Garber CE, 2011, MED SCI SPORT EXER, V43, P1334, DOI 10.1249/MSS.0b013e318213fefb
Gurven M, 2013, PLOS ONE, V8, DOI 10.1371/journal.pone.0055679
Hawkes K., 1989, COMP SOCIOECOLOGY HU, P341
HEATH GW, 1981, J APPL PHYSIOL, V51, P634, DOI 10.1152/jappl.1981.51.3.634
International Atomic Energy Agency (IAEA), 2009, IAEA HUM HLTH SER
KASHIWAZAKI H, 1995, THE AMERICAN JOURNAL, V62, P901
LAWRENCE M, 1988, EUR J CLIN NUTR, V42, P145
Leonard WR, 2003, AM J HUM BIOL, V15, P479, DOI 10.1002/ajhb.10187
Leonard WR, 1997, AM J PHYS ANTHROPOL, V102, P265, DOI 10.1002/(SICI)1096-8644(199702)102:2<265::AID-AJPA8>3.0.CO;2-X
Leonard WR, 1997, AM J PHYS ANTHROPOL, V103, P443, DOI 10.1002/(SICI)1096-8644(199708)103:4<443::AID-AJPA2>3.0.CO;2-O
Lieberman D., 2013, The story of the human body : evolution, health, and disease, VFirst
Lieberman DE, 2015, CURR SPORT MED REP, V14, P313, DOI 10.1249/JSR.0000000000000168
Lieberman DE, 2009, THE FIRST HUMANS ORI, P77
Liebert MA, 2013, ANN HUM BIOL, V40, P228, DOI 10.3109/03014460.2012.759621
Madimenos FC, 2011, AM J HUM BIOL, V23, P488, DOI 10.1002/ajhb.21163
Malina RM, 2008, AM J HUM BIOL, V20, P373, DOI 10.1002/ajhb.20772
MANNINEN V, 1992, CIRCULATION, V85, P37, DOI 10.1161/01.CIR.85.1.37
Mcdade TW, 2007, DEMOGRAPHY, V44, P899, DOI 10.1353/dem.2007.0038
McDade TW, 2012, AM J HUM BIOL, V24, P675, DOI 10.1002/ajhb.22296
Myers J, 2015, PROG CARDIOVASC DIS, V57, P306, DOI 10.1016/j.pcad.2014.09.011
Norton K, 2010, J SCI MED SPORT, V13, P496, DOI 10.1016/j.jsams.2009.09.008
Ong KL, 2007, HYPERTENSION, V49, P69, DOI 10.1161/01.HYP.0000252676.46043.18
Pollock ML, 1997, J APPL PHYSIOL, V82, P1508, DOI 10.1152/jappl.1997.82.5.1508
Pontzer H, 2016, CURR BIOL, V26, P410, DOI 10.1016/j.cub.2015.12.046

Pontzer H, 2015, AM J HUM BIOL, V27, P628, DOI 10.1002/ajhb.22711
Pontzer H, 2015, EXERC SPORT SCI REV, V43, P110, DOI 10.1249/JES.0000000000000048
Pontzer H, 2012, CURR ANTHROPOL, V53, pS346, DOI 10.1086/667402
Pontzer H, 2012, PLOS ONE, V7, DOI 10.1371/journal.pone.0040503
Raichlen DA, 2014, P NATL ACAD SCI USA, V111, P728, DOI 10.1073/pnas.1318616111
Raichlen DA, 2013, P ROY SOC B-BIOL SCI, V280, DOI 10.1098/rspb.2012.2250
Ridker PM, 2010, LANCET, V376, P333, DOI 10.1016/S0140-6736(10)60713-1
Tanaka H, 2001, J AM COLL CARDIOL, V37, P153, DOI 10.1016/S0735-1097(00)01054-8
Troiano RP, 2008, MED SCI SPORT EXER, V40, P181, DOI 10.1249/mss.0b013e31815a51b3
Tucker JM, 2011, AM J PREV MED, V40, P454, DOI 10.1016/j.amepre.2010.12.016
Vasunilashorn S, 2010, AM J HUM BIOL, V22, P731, DOI 10.1002/ajhb.21074
Warburton DER, 2006, CAN MED ASSOC J, V174, P801, DOI 10.1503/cmaj.051351
Wood BM, 2013, HUM NATURE-INT BIOS, V24, P280, DOI 10.1007/s12110-013-9173-0
NR 52
TC 101
Z9 113
U1 2
U2 39
PU WILEY
PI HOBOKEN
PA 111 RIVER ST, HOBOKEN 07030-5774, NJ USA
SN 1042-0533
EI 1520-6300
J9 AM J HUM BIOL
JI Am. J. Hum. Biol.
PD MAR-APR
PY 2017
VL 29
IS 2
SI SI
AR e22919
DI 10.1002/ajhb.22919
PG 13
WC Anthropology; Biology
WE Science Citation Index Expanded (SCI-EXPANDED); Social Science Citation Index (SSCI)
SC Anthropology; Life Sciences & Biomedicine - Other Topics
GA EP5GM
UT WOS:000397407000147
PM 27723159
OA Green Submitted
DA 2025-05-07
ER

PT J

AU Baadsvik, EL
   Weiger, M
   Froidevaux, R
   Rösler, MB
   Brunner, DO
   Öhrström, L
   Rühli, FJ
   Eppenberger, P
   Pruessmann, KP
AF Baadsvik, Emily Louise
   Weiger, Markus
   Froidevaux, Romain
   Rosler, Manuela Barbara
   Brunner, David Otto
   Ohrstrom, Lena
   Ruhli, Frank Jakobus
   Eppenberger, Patrick
   Pruessmann, Klaas Paul
TI High-resolution MRI of mummified tissues using advanced
   short-T2methodology and hardware
SO MAGNETIC RESONANCE IN MEDICINE
LA English
DT Article
DE ancient Egyptian mummy; high resolution; high-performance gradient;
   HYFI; short T-2; ZTE
ID RESONANCE; MUMMY; PERFORMANCE; REMAINS; CT
AB Purpose Evolutionary medicine aims to study disease development from a long-term perspective, and through the analysis of mummified tissue, timescales of several thousand years are unlocked. Due to the status of mummies as ancient relics, noninvasive techniques are preferable, and, currently, CT imaging is the most widespread method. However, CT images lack soft-tissue contrast, making complementary MRI data desirable. Unfortunately, the dehydrated nature and short T(2)times of mummified tissues render them practically invisible to standard MRI techniques. Specialized short-T(2)approaches have therefore been used, but currently suffer severe resolution limitations. The purpose of the present study is to improve resolution in MRI of mummified tissues. Methods The zero-TE-based hybrid filling technique, together with a high-performance magnetic field gradient, was used to image three ancient Egyptian mummified human body parts: a hand, a foot, and a head. A similar pairing has already been shown to increase resolution and image quality in MRI of short-T(2)tissues. Results MRI images of yet unparalleled image quality were obtained for all samples, reaching isotropic resolutions of 0.6 mm and SNR values above 100. The same general features as present in CT images were depicted but with different contrast, particularly for regions containing embalming substances. Conclusion Mummy MRI is a potentially valuable tool for (paleo)pathological studies, as well as for investigations into ancient mummification processes. The results presented here show sufficient improvement in the depiction of mummified tissues to clear new paths for the exploration of this field.
C1 [Baadsvik, Emily Louise; Weiger, Markus; Froidevaux, Romain; Rosler, Manuela Barbara; Brunner, David Otto; Pruessmann, Klaas Paul] Swiss Fed Inst Technol, Inst Biomed Engn, Gloriastr 35, CH-8092 Zurich, Switzerland.

[Baadsvik, Emily Louise; Weiger, Markus; Froidevaux, Romain; Rosler, Manuela Barbara; Brunner, David Otto; Pruessmann, Klaas Paul] Univ Zurich, Gloriastr 35, CH-8092 Zurich, Switzerland.
[Ohrstrom, Lena; Ruhli, Frank Jakobus; Eppenberger, Patrick] Univ Zurich, Inst Evolutionary Med, Zurich, Switzerland.
C3 Swiss Federal Institutes of Technology Domain; ETH Zurich; University of
Zurich; University of Zurich
RP Weiger, M (autor correspondente), Swiss Fed Inst Technol, Inst Biomed Engn, Gloriastr 35, CH-8092 Zurich, Switzerland.; Weiger, M (autor correspondente), Univ Zurich, Gloriastr 35, CH-8092 Zurich, Switzerland.
EM weiger@biomed.ee.ethz.ch
RI Pruessmann, Klaas/H-5385-2018; Ruhli, Frank/H-8789-2017
OI Froidevaux, Romain/0000-0001-7550-5554; Baadsvik, Emily Louise/0000-0001-5941-5532; Ruhli, Frank/0000-0001-7937-001X; Weiger, Markus/0000-0003-4758-3103
FU Aker Scholarship
FX E.L.B. wishes to acknowledge personal funding from the Aker Scholarship.
CR Appelboom T, 1999, LANCET, V354, P2153, DOI 10.1016/S0140-6736(99)07113-5
Aufderheide AC, 2000, JAMA-J AM MED ASSOC, V284, P2571, DOI 10.1001/jama.284.20.2571
Bock M, 2008, P INT MAG RESON MED, V16, P3666
Bourke JB, 1986, LINDOW MAN BODY BOG, P46
Brunner DO, 2016, J MAGN RESON, V263, P147, DOI 10.1016/j.jmr.2015.12.016
Buckley SA, 2001, NATURE, V413, P837, DOI 10.1038/35101588
Colombini MP, 2000, STUD CONSERV, V45, P19, DOI 10.2307/1506680
Dietrich BE, 2016, MAGN RESON MED, V75, P1831, DOI 10.1002/mrm.25770
Doran SJ, 2005, PHYS MED BIOL, V50, P1343, DOI 10.1088/0031-9155/50/7/001
Froidevaux R, 2018, P 26 ANN SCI M ISMRM, P3947
Froidevaux R, 2020, MAGN RESON MED, V84, P1933, DOI 10.1002/mrm.28254
Grodzki DM, 2012, J MAGN RESON, V214, P61, DOI 10.1016/j.jmr.2011.10.005
GUDBJARTSSON H, 1995, MAGNET RESON MED, V34, P910, DOI 10.1002/mrm.1910340618
Hunt DR, 1996, MAN IN ICE, V3, P15
[Institute of Evolutionary Medicine (IEM) University of Zurich Institute of Evolutionary Medicine (IEM)], IEM COD ETH
Konig W., 1896, 14 Photographien mit RontgenStrahlen: aufgenommen im physikalischen Verein zu Frankfurt AM
Lee HM, 2020, MAGN RESON MED, V83, P412, DOI 10.1002/mrm.27925
Li C, 2014, IEEE T MED IMAGING, V33, P961, DOI 10.1109/TMI.2014.2300500
Münnemann K, 2007, MAGN RESON IMAGING, V25, P1341, DOI 10.1016/j.mri.2007.03.023
NOTMAN DNH, 1986, AM J ROENTGENOL, V146, P93, DOI 10.2214/ajr.146.1.93
Öhrström LM, 2013, RADIOGRAPHICS, V33, P291, DOI 10.1148/rg.331125711
Özen AC, 2016, MAGN RESON MED, V75, P701, DOI 10.1002/mrm.25651
Öhrström L, 2020, CLIN ANAT, V33, P860, DOI 10.1002/ca.23568
PIEPENBRINK H, 1986, AM J PHYS ANTHROPOL, V70, P27, DOI 10.1002/ajpa.1330700107

Posh JC, 2015, ANAT REC, V298, P1116, DOI 10.1002/ar.23153
Rahmer J, 2006, MAGN RESON MED, V55, P1075, DOI 10.1002/mrm.20868
Rosler MB, 2019, P 27 ANN M ISMRM MON, P438
Rühli FJ, 2006, AM J PHYS ANTHROPOL, P156
Rühli FJ, 2007, JAMA-J AM MED ASSOC, V298, P2618, DOI 10.1001/jama.298.22.2618-b
Shin DH, 2010, J ANAT, V216, P329, DOI 10.1111/j.1469-7580.2009.01185.x
Tannus A, 1996, J MAGN RESON SER A, V120, P133, DOI 10.1006/jmra.1996.0110
Weiger M, 2012, EMAGRES, V1, P311, DOI 10.1002/9780470034590.emrstm1292
Weiger M, 2019, PROG NUCL MAG RES SP, V114, P237, DOI 10.1016/j.pnmrs.2019.07.001
Weiger M, 2018, MAGN RESON MED, V79, P3256, DOI 10.1002/mrm.26954
Weiger M, 2017, MAGN RESON MED, V78, P399, DOI 10.1002/mrm.26368
NR 35
TC 9
Z9 9
U1 0
U2 8
PU WILEY
PI HOBOKEN
PA 111 RIVER ST, HOBOKEN 07030-5774, NJ USA
SN 0740-3194
EI 1522-2594
J9 MAGN RESON MED
JI Magn. Reson. Med.
PD MAR
PY 2021
VL 85
IS 3
BP 1481
EP 1492
DI 10.1002/mrm.28530
EA OCT 2020
PG 12
WC Radiology, Nuclear Medicine & Medical Imaging
WE Science Citation Index Expanded (SCI-EXPANDED)
SC Radiology, Nuclear Medicine & Medical Imaging
GA WN7WL
UT WOS:000574660000001
PM 33009877
DA 2025-05-07
ER

PT J
AU Chevalier, RL
AF Chevalier, Robert L.
TI Bioenergetics: the evolutionary basis of progressive kidney disease
SO PHYSIOLOGICAL REVIEWS
LA English
DT Review
DE adaptation; aging; bioenergetics; chronic kidney disease; evolution

ID UNILATERAL URETERAL OBSTRUCTION; HEAT-SHOCK PROTEINS; CELL-CYCLE ARREST;
PROLYL-HYDROXYLASE INHIBITORS; ABNORMAL RENAL ARCHITECTURE; PODOCYTE ENERGY-METABOLISM; TUBULAR EPITHELIAL-CELLS; GROWTH-FACTOR-BETA; OXYGEN-CONSUMPTION; MITOCHONDRIAL DYSFUNCTION
AB Chronic kidney disease (CKD) affects >10% of the world population, with increasing prevalence in middle age. The risk for CKD is dependent on the number of functioning nephrons through the life cycle, and 50% of nephrons are lost through normal aging, revealing their vulnerability to internal and external stressors. Factors responsible for CKD remain poorly understood, with limited availability of biomarkers or effective therapy to slow progression. This review draws on the disciplines of evolutionary medicine and bioenergetics to account for the heterogeneous nephron injury that characterizes progressive CKD following episodes of acute kidney injury with incomplete recovery. The evolution of symbiosis in eukaryotes led to the efficiencies of oxidative phosphorylation and the rise of metazoa. Adaptations to ancestral environments are the products of natural selection that have shaped the mammalian nephron with its vulnerabilities to ischemic, hypoxic, and toxic injury. Reproductive fitness rather than longevity has served as the driver of evolution, constrained by available energy and its allocation to homeostatic responses through the life cycle. Metabolic plasticity has evolved in parallel with robustness necessary to preserve complex developmental programs, and adaptations that optimize survival through reproductive years can become maladaptive with aging, reflecting antagonistic pleiotropy. Consequently, environmental stresses promote trade-offs and mismatches that result in cell fate decisions that ultimately lead to nephron loss. Elucidation of the bioenergetic adaptations by the nephron to ancestral and contemporary environments may lead to the development of new biomarkers of kidney disease and new therapies to reduce the global burden of progressive CKD.
C1 [Chevalier, Robert L.] Univ Virginia, Dept Pediat, Charlottesville, VA 22903 USA.
C3 University of Virginia
RP Chevalier, RL (autor correspondente), Univ Virginia, Dept Pediat, Charlottesville, VA 22903 USA.
EM RLC2M@virginia.edu
OI Chevalier, Robert/0000-0002-7465-482X
CR Adler S, 2004, J AM SOC NEPHROL, V15, P52, DOI 10.1097/01.ASN.0000101032.21097.C5
Adler S, 2002, J AM SOC NEPHROL, V13, DOI 10.1097/01.ASN.0000019781.90630.0F
Alderman H, 2018, PLOS ONE, V13, DOI 10.1371/journal.pone.0195904
Alicic RZ, 2018, AM J KIDNEY DIS, V72, P267, DOI 10.1053/j.ajkd.2018.03.022
Ameisen JC, 2002, CELL DEATH DIFFER, V9, P367, DOI 10.1038/sj.cdd.4400950
Armon R, 2010, J GEN PHILOS SCI, V41, P173, DOI 10.1007/s10838-010-9117-x
Bai M, 2019, KIDNEY INT, V95, P1389, DOI 10.1016/j.kint.2018.12.028
Bajgar A, 2015, PLOS BIOL, V13, DOI 10.1371/journal.pbio.1002135
Bar-On YM, 2018, P NATL ACAD SCI USA, V115, P6506, DOI 10.1073/pnas.1711842115

Barraclough KA, 2017, KIDNEY INT, V92, P526, DOI 10.1016/j.kint.2017.03.047
Barutta F, 2021, J AM SOC NEPHROL, V32, P1114, DOI 10.1681/ASN.2020071076
Bateson P, 2013, TRENDS ECOL EVOL, V28, P712, DOI 10.1016/j.tree.2013.09.013
Bateson P, 2012, INT J EPIDEMIOL, V41, P219, DOI 10.1093/ije/dyr240
BEEUWKES R, 1975, AM J PHYSIOL, V229, P695, DOI 10.1152/ajplegacy.1975.229.3.695
Benton ML, 2021, NAT REV GENET, V22, P269, DOI 10.1038/s41576-020-00305-9
Bernard K, 2015, J BIOL CHEM, V290, P25427, DOI 10.1074/jbc.M115.646984
Bertram JF, 2011, PEDIATR NEPHROL, V26, P1529, DOI 10.1007/s00467-011-1843-8
Beyenbach KW, 2004, AM J PHYSIOL-RENAL, V286, pF811, DOI 10.1152/ajprenal.00351.2003
Bhargava P, 2017, NAT REV NEPHROL, V13, P629, DOI 10.1038/nrneph.2017.107
BHATHENA DB, 1985, AM J KIDNEY DIS, V5, P226, DOI 10.1016/S0272-6386(85)80113-X
Bhathena DB, 1996, J AM SOC NEPHROL, V7, P2600
Bikbov B, 2020, LANCET, V395, P709, DOI 10.1016/S0140-6736(20)30045-3
Bishop CM, 1999, P ROY SOC B-BIOL SCI, V266, P2275, DOI 10.1098/rspb.1999.0919
BLONDIN J, 1975, P SOC EXP BIOL MED, V150, P71
Bolisetty S, 2010, J AM SOC NEPHROL, V21, P1702, DOI 10.1681/ASN.2010030238
Bonventre JV, 2014, KIDNEY INT SUPPL, V4, P39, DOI 10.1038/kisup.2014.8
Brady SP, 2019, EVOL APPL, V12, P1229, DOI 10.1111/eva.12844
BRAND MD, 1994, BBA-BIOENERGETICS, V1187, P132, DOI 10.1016/0005-2728(94)90099-X
BRENNER BM, 1989, PERSPECT BIOL MED, V32, P434
BREZIS M, 1995, NEW ENGL J MED, V332, P647, DOI 10.1056/NEJM199503093321006
Burhans WC, 2009, FREE RADICAL BIO MED, V47, P1282, DOI 10.1016/j.freeradbiomed.2009.05.026
Canaud G, 2015, NEPHROL DIAL TRANSPL, V30, P575, DOI 10.1093/ndt/gfu230
Cantó C, 2015, CELL METAB, V22, P31, DOI 10.1016/j.cmet.2015.05.023
Cao HD, 2020, KIDNEY INT, V98, P686, DOI 10.1016/j.kint.2020.03.035
Cargill K, 2019, J AM SOC NEPHROL, V30, P1192, DOI 10.1681/ASN.2018111170
Cargill K, 2020, PEDIATR NEPHROL, V35, P1, DOI 10.1007/s00467-018-4157-2
CARGILL WH, 1949, J CLIN INVEST, V28, P526, DOI 10.1172/JCI102100
Carney EF, 2020, NAT REV NEPHROL, V16, P251, DOI 10.1038/s41581-020-0268-7
Casalena G, 2012, SEMIN NEPHROL, V32, P295, DOI 10.1016/j.semnephrol.2012.04.009
Chacko BK, 2014, CLIN SCI, V127, P367, DOI 10.1042/CS20140101
Chappell JC, 2019, J CLIN INVEST, V129, P442, DOI 10.1172/JCI120855

Che RC, 2014, AM J PHYSIOL-RENAL, V306, pF367, DOI 10.1152/ajprenal.00571.2013
Chebotareva N, 2017, CELL STRESS CHAPERON, V22, P319, DOI 10.1007/s12192-017-0790-0
Chen JC, 2006, J AM SOC NEPHROL, V17, P1615, DOI 10.1681/ASN.2005111163
Chen XZ, 2015, FREE RADICAL BIO MED, V79, P253, DOI 10.1016/j.freeradbiomed.2014.08.027
Chevalier RL, 2008, J AM SOC NEPHROL, V19, P197, DOI 10.1681/ASN.2007080862
Chevalier RL, 2020, KIDNEY360, V1, P863, DOI 10.34067/KID.0002012020
Chevalier RL, 2019, SEMIN CELL DEV BIOL, V91, P119, DOI 10.1016/j.semcdb.2018.05.024
Chevalier RL, 2017, KIDNEY INT REP, V2, P302, DOI 10.1016/j.ekir.2017.01.012
Chevalier RL, 2016, AM J PHYSIOL-RENAL, V311, pF145, DOI 10.1152/ajprenal.00164.2016
Chevalier RL, 2009, KIDNEY INT, V75, P1145, DOI 10.1038/ki.2009.86
Chiba T, 2019, J AM SOC NEPHROL, V30, P2384, DOI 10.1681/ASN.2019020163
Churchhouse C., 2013, NATURE, V506, P97, DOI DOI 10.1038/nature12828
Clark AJ, 2021, KIDNEY INT, V99, P828, DOI 10.1016/j.kint.2020.09.037
Cordain L, 2005, AM J CLIN NUTR, V81, P341, DOI 10.1093/ajcn.81.2.341
Craig PM, 2018, COMP BIOCHEM PHYS B, V224, P156, DOI 10.1016/j.cbpb.2017.11.001
Crespi BJ, 2000, HEREDITY, V84, P623, DOI 10.1046/j.1365-2540.2000.00746.x
CRICK F, 1970, NATURE, V227, P561, DOI 10.1038/227561a0
Csete M, 2004, TRENDS BIOTECHNOL, V22, P446, DOI 10.1016/j.tibtech.2004.07.007
da Silva JL, 2001, KIDNEY INT, V59, P1448, DOI 10.1046/j.1523-1755.2001.0590041448.x
Darwin Charles., 2009, ANNOTATED ORIGIN FAC
Davaapil H, 2017, DEVELOPMENT, V144, P106, DOI 10.1242/dev.138222
Davies KJA, 2016, MOL ASPECTS MED, V49, P1, DOI 10.1016/j.mam.2016.04.007
De Bels D, 2011, NEW ENGL J MED, V365, P1845, DOI [10.1056/NEJMra1011165, 10.1056/NEJMc1110602]
de Cavanagh EMV, 2015, AM J PHYSIOL-HEART C, V309, pH15, DOI 10.1152/ajpheart.00459.2014
DeBerardinis RJ, 2008, CELL METAB, V7, P11, DOI 10.1016/j.cmet.2007.10.002
Declèves AE, 2011, J AM SOC NEPHROL, V22, P1846, DOI 10.1681/ASN.2011010026
Denburg MR, 2021, CLIN J AM SOC NEPHRO, V16, P1178, DOI 10.2215/CJN.00220121
Denic A, 2017, NEW ENGL J MED, V376, P2349, DOI 10.1056/NEJMoa1614329
Denic A, 2017, J AM SOC NEPHROL, V28, P313, DOI 10.1681/ASN.2016020154
Devalaraja-Narashimha K, 2009, J AM SOC NEPHROL, V20, P95, DOI 10.1681/ASN.2008030325
Diamond J, 2002, J PHYSIOL-LONDON, V542, P337, DOI 10.1113/jphysiol.2002.018366
DiRocco DP, 2014, AM J PHYSIOL-RENAL, V306, pF379, DOI 10.1152/ajprenal.00475.2013

Dörr JR, 2013, NATURE, V501, P421, DOI 10.1038/nature12437
Doleris LM, 2000, KIDNEY INT, V58, P1851, DOI 10.1111/j.1523-1755.2000.00356.x
Donnelly S, 2003, ADV EXP MED BIOL, V543, P73
Dumanski SM, 2019, J NEPHROL, V32, P39, DOI 10.1007/s40620-018-00569-9
Dumas SJ, 2020, J AM SOC NEPHROL, V31, P118, DOI 10.1681/ASN.2019080832
Dybala MP, 2019, DIABETES, V68, P1230, DOI 10.2337/db19-0072
Eckardt KU, 2013, LANCET, V382, P158, DOI 10.1016/S0140-6736(13)60439-0
ELIA M, 1992, ENERGY METABOLISM, P61
Emma F, 2016, NAT REV NEPHROL, V12, P267, DOI 10.1038/nrneph.2015.214
Epstein FH, 1997, KIDNEY INT, V51, P381, DOI 10.1038/ki.1997.50
Evans RG, 2008, AM J PHYSIOL-RENAL, V295, pF1259, DOI 10.1152/ajprenal.90230.2008
Evans RG, 2020, ANAT REC, V303, P2544, DOI 10.1002/ar.24260
Fattah H, 2019, PHYSIOLOGY, V34, P189, DOI 10.1152/physiol.00052.2018
Fine LG, 2018, J AM SOC NEPHROL, V29, P2780, DOI 10.1681/ASN.2018080822
Fine LG, 2002, J AM SOC NEPHROL, V13, P1974, DOI 10.1097/01.ASN.0000024380.91444.C8
FINE LG, 1983, SEMIN NEPHROL, V3, P261
Fogel RW, 2003, PERSPECT BIOL MED, V46, pS24, DOI 10.1353/pbm.2003.0066
Folmes CDL, 2012, ANN NY ACAD SCI, V1254, P82, DOI 10.1111/j.1749-6632.2012.06487.x
Forbes JM, 2020, KIDNEY INT, V97, P855, DOI 10.1016/j.kint.2020.01.038
Forbes JM, 2016, TRENDS ENDOCRIN MET, V27, P441, DOI 10.1016/j.tem.2016.05.002
Forbes MS, 2012, AM J PHYSIOL-RENAL, V303, pF120, DOI 10.1152/ajprenal.00110.2012
Forbes MS, 2011, AM J PHYSIOL-RENAL, V301, pF110, DOI 10.1152/ajprenal.00022.2011
Ford ES, 2005, DIABETES CARE, V28, P1769, DOI 10.2337/diacare.28.7.1769
Franceschi C, 2000, ANN NY ACAD SCI, V908, P244
Frank SA, 2007, PLOS ONE, V2, DOI 10.1371/journal.pone.0001021
Frank SA, 2019, J THEOR BIOL, V463, P121, DOI 10.1016/j.jtbi.2018.12.023
Frasca D, 2021, MECH AGEING DEV, V194, DOI 10.1016/j.mad.2020.111428
Frenkel-Denkberg G, 1999, FEBS LETT, V462, P341, DOI 10.1016/S0014-5793(99)01552-5
Friederich M, 2008, BBA-BIOENERGETICS, V1777, P935, DOI 10.1016/j.bbabio.2008.03.030
Friedman DJ, 2011, J AM SOC NEPHROL, V22, P2098, DOI 10.1681/ASN.2011050519
Fujii Y, 2006, KIDNEY INT, V69, P1350, DOI 10.1038/sj.ki.5000317
Galarreta CI, 2014, AM J PATHOL, V184, P1957, DOI 10.1016/j.ajpath.2014.03.007
Gallipoli P, 2019, SCIENCE, V363, P1148, DOI 10.1126/science.aaw8373
Galluzzi L, 2008, CELL DEATH DIFFER, V15, P1113, DOI 10.1038/cdd.2008.28
Galvan DL, 2017, KIDNEY INT, V92, P1282, DOI 10.1016/j.kint.2017.05.015

Ganeshan K, 2019, CELL, V177, P399, DOI 10.1016/j.cell.2019.01.050
Ganeshan K, 2014, ANNU REV IMMUNOL, V32, P609, DOI 10.1146/annurev-immunol-032713-120236
GARLAND T, 1994, ANNU REV PHYSIOL, V56, P579, DOI 10.1146/annurev.ph.56.030194.003051
Geraci S, 2017, AM J PHYSIOL-RENAL, V313, pF576, DOI 10.1152/ajprenal.00329.2015
Gjerde A, 2020, NEPHROL DIAL TRANSPL, V35, P1157, DOI 10.1093/ndt/gfaa001
Glaser J, 2016, CLIN J AM SOC NEPHRO, V11, P1472, DOI 10.2215/CJN.13841215
Goda N, 2012, INT J HEMATOL, V95, P457, DOI 10.1007/s12185-012-1069-y
Gödel M, 2011, J CLIN INVEST, V121, P2197, DOI 10.1172/JCI44774
Gómez H, 2017, NAT REV NEPHROL, V13, P143, DOI 10.1038/nrneph.2016.186
Gomez H, 2014, SHOCK, V41, P3, DOI 10.1097/SHK.0000000000000052
GOMEZ RA, 1988, AM J PHYSIOL, V254, pF900, DOI 10.1152/ajprenal.1988.254.6.F900
Gooz MB, 2014, AM J PHYSIOL-RENAL, V307, pF551, DOI 10.1152/ajprenal.00218.2014
Gorin Y, 2005, J BIOL CHEM, V280, P39616, DOI 10.1074/jbc.M502412200
Grafflin AL, 1933, ANAT REC, V57, P59, DOI 10.1002/ar.1090570109
Graham AM, 2017, PLOS ONE, V12, DOI 10.1371/journal.pone.0179545
Granado D, 2017, J AM SOC NEPHROL, V28, P3226, DOI 10.1681/ASN.2016111220
Granata S, 2009, BMC GENOMICS, V10, DOI 10.1186/1471-2164-10-388
Grantham JJ, 2002, AM J PHYSIOL-RENAL, V282, pF1, DOI 10.1152/ajprenal.2002.282.1.F1
Grau-Bové X, 2022, NAT ECOL EVOL, V6, P1007, DOI 10.1038/s41559-022-01771-6
GREEN CJ, 1984, COMP BIOCHEM PHYS B, V79, P167, DOI 10.1016/0305-0491(84)90009-9
Green DR, 2014, SCIENCE, V345, P1466, DOI 10.1126/science.1250256
Greenberg JH, 2020, J AM SOC NEPHROL, V31, P1067, DOI 10.1681/ASN.2019070723
Grunspan DZ, 2018, EVOL MED PUBLIC HLTH, P13, DOI 10.1093/emph/eox025
Gujarati NA, 2020, AM J PHYSIOL-RENAL, V319, pF955, DOI 10.1152/ajprenal.00393.2020
Gunaratnam L, 2009, J AM SOC NEPHROL, V20, P1877, DOI 10.1681/ASN.2008070804
Guo DF, 2006, HYPERTENSION, V48, P453, DOI 10.1161/01.HYP.0000230664.32874.52
Guo YQ, 2018, J AM SOC NEPHROL, V29, P2529, DOI 10.1681/ASN.2018030324
Gupta N, 2022, SCI TRANSL MED, V14, DOI 10.1126/scitranslmed.abj4772
Hallows KR, 2010, AM J PHYSIOL-RENAL, V298, pF1067, DOI 10.1152/ajprenal.00005.2010
Hand SC, 1996, ANNU REV PHYSIOL, V58, P539, DOI 10.1146/annurev.ph.58.030196.002543
Hannken T, 1998, KIDNEY INT, V54, P1923, DOI 10.1046/j.1523-1755.1998.00212.x
Hansell P, 2013, CLIN EXP PHARMACOL P, V40, P123, DOI 10.1111/1440-1681.12034
Harms MJ, 2013, NAT REV GENET, V14, P559, DOI 10.1038/nrg3540
Harold FM., 2014, In Search of Cell History: The Evolution of Life's Building Blocks

HARRIS RH, 1977, KIDNEY INT, V12, P305, DOI 10.1038/ki.1977.117
Hasegawa S, 2020, KIDNEY INT, V97, P934, DOI 10.1016/j.kint.2019.12.007
He JC, 2012, KIDNEY INT, V81, P22, DOI 10.1038/ki.2011.314
He LY, 2017, KIDNEY INT, V92, P1071, DOI 10.1016/j.kint.2017.06.030
He WJ, 2022, CLIN J AM SOC NEPHRO, V17, P966, DOI 10.2215/CJN.15551121
Healy K, 2014, P ROY SOC B-BIOL SCI, V281, DOI 10.1098/rspb.2014.0298
Heiden MGV, 2009, SCIENCE, V324, P1029, DOI 10.1126/science.1160809
Hesp AC, 2020, KIDNEY INT, V98, P579, DOI 10.1016/j.kint.2020.02.041
Hill NR, 2016, PLOS ONE, V11, DOI 10.1371/journal.pone.0158765
Hirakawa Y, 2018, KIDNEY INT, V93, P1483, DOI 10.1016/j.kint.2018.01.015
HOSTETTER TH, 1981, AM J PHYSIOL, V241, pF85, DOI 10.1681/ASN.V1261315
Hoy WE, 2015, J AM SOC NEPHROL, V26, P3179, DOI 10.1681/ASN.2014080768
Hoy WE, 2011, AM J NEPHROL, V33, P15, DOI 10.1159/000327044
Huang LH, 2008, CELL RES, V18, P1074, DOI 10.1038/cr.2008.283
Hughes T, 2007, ADV EXP MED BIOL, V607, P111
Hulbert AJ, 2005, J EXP BIOL, V208, P1593, DOI 10.1242/jeb.01482
Humphreys BD, 2018, SCIENCE, V360, P709, DOI 10.1126/science.aat7271
Hunter P, 2002, PFLUG ARCH EUR J PHY, V445, P1, DOI 10.1007/s00424-002-0890-1
Huxley J., 1942, Evolution, the modern synthesis.
HWANG S, 1990, J AM SOC NEPHROL, V1, P822
Ichimura K, 2017, ANAT SCI INT, V92, P161, DOI 10.1007/s12565-015-0317-7
Imasawa T, 2017, FASEB J, V31, P294, DOI 10.1096/fj.201600293R
Imasawa T, 2013, INT J BIOCHEM CELL B, V45, P2109, DOI 10.1016/j.biocel.2013.06.013
Ishii K, 2021, KIDNEY INT, V99, P657, DOI 10.1016/j.kint.2020.10.013
Ix JH, 2010, J AM SOC NEPHROL, V21, P406, DOI 10.1681/ASN.2009080820
Jalal DI, 2010, J AM SOC NEPHROL, V21, P1543, DOI 10.1681/ASN.2009111111
Jang C, 2019, CELL METAB, V30, P594, DOI 10.1016/j.cmet.2019.06.002
Jani A, 2013, AM J PHYSIOL-RENAL, V305, pF1521, DOI 10.1152/ajprenal.00675.2012
Johnson RJ, 2020, J INTERN MED, V287, P252, DOI 10.1111/joim.12993
Johnson RJ, 2019, NEW ENGL J MED, V380, P1843, DOI 10.1056/NEJMra1813869
Johnson RJ, 2016, J AM SOC NEPHROL, V27, P2247, DOI 10.1681/ASN.2015121314
Johnson RJ, 2010, J AM SOC NEPHROL, V21, P2036, DOI 10.1681/ASN.2010050506
Johnson RJ, 2009, J COMP PHYSIOL B, V179, P67, DOI 10.1007/s00360-008-0291-7
Joyner MJ, 2015, PROG BIOPHYS MOL BIO, V117, P107, DOI 10.1016/j.pbiomolbio.2014.09.010
Ju WJ, 2015, SCI TRANSL MED, V7, DOI 10.1126/scitranslmed.aac7071
Kaissling B, 2013, BBA-MOL BASIS DIS, V1832, P931, DOI 10.1016/j.bbadis.2013.02.010
Kamel KS, 1998, KIDNEY INT, V53, P531, DOI 10.1046/j.1523-1755.1998.00803.x
Kanwar YS, 2008, AM J PHYSIOL-RENAL, V295, pF1301, DOI 10.1152/ajprenal.90506.2008
Katsyuba E, 2018, NATURE, V563, P354, DOI 10.1038/s41586-018-0645-6

Kawahara T, 2007, BMC EVOL BIOL, V7, DOI 10.1186/147-2148-7-109
Kawakami T, 2015, J AM SOC NEPHROL, V26, P1040, DOI 10.1681/ASN.2013111202
Keller BJ, 2012, KIDNEY INT, V81, P14, DOI 10.1038/ki.2011.359
Kellum JA, 2016, NEPHROL DIAL TRANSPL, V31, P16, DOI 10.1093/ndt/gfv130
Kelly JT, 2021, J AM SOC NEPHROL, V32, P239, DOI 10.1681/ASN.2020030384
Kikuchi H, 2019, KIDNEY INT, V95, P123, DOI 10.1016/j.kint.2018.08.030
Kim DH, 2020, EXP GERONTOL, V134, DOI 10.1016/j.exger.2020.110891
Kim JW, 2006, CELL METAB, V3, P177, DOI 10.1016/j.cmet.2006.02.002
Kim SM, 2012, PLOS ONE, V7, DOI 10.1371/journal.pone.0039739
Kirkwood TBL, 2005, MECH AGEING DEV, V126, P1011, DOI 10.1016/j.mad.2005.03.021
KIRKWOOD TBL, 1977, NATURE, V270, P301, DOI 10.1038/270301a0
Kitamura M, 2008, AM J PHYSIOL-RENAL, V295, pF323, DOI 10.1152/ajprenal.00050.2008
Kominsky DJ, 2010, J IMMUNOL, V184, P4062, DOI 10.4049/jimmunol.0903002
Koonin EV, 2002, CELL DEATH DIFFER, V9, P394, DOI 10.1038/sj.cdd.4400991
Kowaltowski AJ, 2001, FEBS LETT, V495, P12, DOI 10.1016/S0014-5793(01)02316-X
Kramer BA, 2022, SCIENCE, V377, P642, DOI 10.1126/science.abf4062
Kriz W, 1998, NEPHROL DIAL TRANSPL, V13, P2781, DOI 10.1093/ndt/13.11.2781
Kriz W, 2020, ANAT REC, V303, P2588, DOI 10.1002/ar.24291
Kriz W, 2017, PEDIATR NEPHROL, V32, P405, DOI 10.1007/s00467-016-3358-9
Kruzel-Davila E, 2016, NEPHROL DIAL TRANSPL, V31, P349, DOI 10.1093/ndt/gfu391
Kuro-o M, 2010, MECH AGEING DEV, V131, P270, DOI 10.1016/j.mad.2010.02.008
Lambeth JD, 2007, FREE RADICAL BIO MED, V43, P332, DOI 10.1016/j.freeradbiomed.2007.03.027
Lampl M, 2005, AM J HUM BIOL, V17, P66, DOI 10.1002/ajhb.20094
Lan RP, 2016, J AM SOC NEPHROL, V27, P3356, DOI 10.1681/ASN.2015020177
Landau SI, 2019, KIDNEY INT, V95, P797, DOI 10.1016/j.kint.2018.11.042
Lane N, 2003, J THEOR BIOL, V225, P531, DOI 10.1016/S0022-5193(03)00304-7
Lane N, 2010, NATURE, V467, P929, DOI 10.1038/nature09486
Lash LH, 2009, BIOFACTORS, V35, P500, DOI 10.1002/biof.65
Lazzeri E, 2019, TRENDS MOL MED, V25, P366, DOI 10.1016/j.molmed.2019.02.006
Lazzeri E, 2018, NAT COMMUN, V9, DOI 10.1038/s41467-018-03753-4
Le MK, 2022, bioRxiv, DOI [10.1101/2022.08.24.505188, 10.1101/2022.08.24.505188, DOI 10.1101/2022.08.24.505188]
Lee AM, 2022, J AM SOC NEPHROL, V33, P375, DOI 10.1681/ASN.2021040538
Lee H, 2014, KIDNEY INT, V85, P561, DOI 10.1038/ki.2013.397
Lee HE, 2022, KIDNEY RES CLIN PRAC, V41, pS89, DOI 10.23876/j.krcp.21.269
Lee HO, 2009, GENE DEV, V23, P2461, DOI 10.1101/gad.1829209
Leemans JC, 2014, NAT REV NEPHROL, V10, P398, DOI 10.1038/nrneph.2014.91

Legouis D, 2020, NAT METAB, V2, P732, DOI 10.1038/s42255-020-0238-1
Lei YT, 2017, HISTOL HISTOPATHOL, V32, P1099, DOI 10.14670/HH-11-900
LEMLEY KV, 1987, KIDNEY INT, V31, P538, DOI 10.1038/ki.1987.33
LeMoine CMR, 2010, J MOL EVOL, V70, P492, DOI 10.1007/s00239-010-9347-x
Lemons JMS, 2010, PLOS BIOL, V8, DOI 10.1371/journal.pbio.1000514
Levit GS, 2019, CURR BIOL, V29, pR1276, DOI 10.1016/j.cub.2019.10.064
Li H, 2015, AM J PHYSIOL-RENAL, V309, pF414, DOI 10.1152/ajprenal.00463.2014
Li ZY, 2016, FRONT PHARMACOL, V7, DOI 10.3389/fphar.2016.00307
Liang MY, 2005, KIDNEY INT, V67, P2114, DOI 10.1111/j.1523-1755.2005.00315.x
Libby E, 2014, SCIENCE, V346, P426, DOI 10.1126/science.1262053
Lieberthal W, 1998, AM J PHYSIOL-RENAL, V274, pF315, DOI 10.1152/ajprenal.1998.274.2.F315
Lin HYH, 2021, KIDNEY INT, V99, P870, DOI 10.1016/j.kint.2020.10.038
Little MH, 2014, CLIN J AM SOC NEPHRO, V9, P809, DOI 10.2215/CJN.10851013
Liu CX, 2015, NEPHRON, V130, P66, DOI 10.1159/000381858
Liu J, 2017, J AM SOC NEPHROL, V28, P3323, DOI 10.1681/ASN.2016111246
López-Lluch G, 2017, MECH AGEING DEV, V162, P108, DOI 10.1016/j.mad.2016.12.005
Lubbers DW, 1997, KIDNEY INT, V51, P372, DOI 10.1038/ki.1997.49
Lum JJ, 2007, GENE DEV, V21, P1037, DOI 10.1101/gad.1529107
Luyckx VA, 2020, ANAT REC, V303, P2613, DOI 10.1002/ar.24270
Ma MKM, 2018, TRANSPLANTATION, V102, pS32, DOI 10.1097/TP.0000000000001729
MacManes MD, 2017, AM J PHYSIOL-RENAL, V313, pF262, DOI 10.1152/ajprenal.00067.2017
Mallipattu SK, 2016, AM J PHYSIOL-RENAL, V311, pF46, DOI 10.1152/ajprenal.00184.2016
Marelli-Berg FM, 2012, IMMUNOLOGY, V136, P363, DOI 10.1111/j.1365-2567.2012.03583.x
Marescal O, 2020, DEV CELL, V55, P259, DOI 10.1016/j.devcel.2020.09.029
MARTIN ARTHUR W., 1955, PHYSIOL ZOOL, V28, P18
Martin B, 2010, P NATL ACAD SCI USA, V107, P6127, DOI 10.1073/pnas.0912955107
Martin SL, 2008, DIABETES VASC DIS RE, V5, P76, DOI 10.3132/dvdr.2008.013
Maslin MA, 2007, J HUM EVOL, V53, P443, DOI 10.1016/j.jhevol.2007.06.005
MASON J, 1983, KIDNEY INT, V24, P27, DOI 10.1038/ki.1983.122
Mathieson S, 2018, MOL BIOL EVOL, V35, P2957, DOI 10.1093/molbev/msy180
McEwen BS, 2015, METABOLISM, V64, pS2, DOI 10.1016/j.metabol.2014.10.029
McEwen BS, 2003, HORM BEHAV, V43, P2, DOI 10.1016/S0018-506X(02)00024-7
McKiernan SH, 2007, AM J PHYSIOL-RENAL, V292, pF1751, DOI 10.1152/ajprenal.00307.2006
Medley JK, 2022, ELIFE, V11, DOI 10.7554/eLife.74539
Megyesi J, 1999, P NATL ACAD SCI USA, V96, P10830, DOI 10.1073/pnas.96.19.10830

Mehr AP, 2018, NAT MED, V24, P1351, DOI 10.1038/s41591-018-0138-z
MelendezHevia E, 1996, J MOL EVOL, V43, P293, DOI 10.1007/BF02338838
Minster RL, 2016, NAT GENET, V48, P1049, DOI 10.1038/ng.3620
Molema G, 2012, SEMIN NEPHROL, V32, P145, DOI 10.1016/j.semnephrol.2012.02.001
Moorjani P, 2016, P NATL ACAD SCI USA, V113, P5652, DOI 10.1073/pnas.1514696113
Morigi M, 2018, J AM SOC NEPHROL, V29, P1799, DOI 10.1681/ASN.2017111218
Muñoz-Espín D, 2013, CELL, V155, P1104, DOI 10.1016/j.cell.2013.10.019
Munusamy S, 2009, FREE RADICAL BIO MED, V46, P1149, DOI 10.1016/j.freeradbiomed.2009.01.022
Naik AS, 2020, KIDNEY INT, V98, P699, DOI 10.1016/j.kint.2020.03.038
Naik S, 2022, NATURE, V607, P249, DOI 10.1038/s41586-022-04919-3
Nair V, 2019, KIDNEY INT, V95, P16, DOI 10.1016/j.kint.2018.11.013
Nakagawa T, 2020, J AM SOC NEPHROL, V31, P898, DOI 10.1681/ASN.2019101015
Needham J, 1926, BR J EXP BIOL, V4, P145
NEEDHAM JOSEPH, 1929, SCIENCE PROGR [LONDON], V23, P633
Nesse RM, 2019, EVOL MED PUBLIC HLTH, P2, DOI 10.1093/emph/eoy035
Nesse RM, 2019, EVOL MED PUBLIC HLTH, P1, DOI 10.1093/emph/eoy034
Nesse RM, 2005, Q REV BIOL, V80, P62, DOI 10.1086/431026
Neuhofer W, 2005, ANNU REV PHYSIOL, V67, P531, DOI 10.1146/annurev.physiol.67.031103.154456
Nicotera P, 2007, CELL DEATH DIFFER, V14, P1235, DOI 10.1038/sj.cdd.4402161
Noble D, 2008, EXP PHYSIOL, V93, P16, DOI 10.1113/expphysiol.2007.038695
Noble D, 2017, INTERFACE FOCUS, V7, DOI 10.1098/rsfs.2016.0159
Noble D, 2015, J EXP BIOL, V218, P7, DOI 10.1242/jeb.106310
Noble D, 2013, EXP PHYSIOL, V98, P1235, DOI 10.1113/expphysiol.2012.071134
Norin T, 2019, PHILOS T R SOC B, V374, DOI 10.1098/rstb.2018.0180
Oda M, 2002, MOL BIOL EVOL, V19, P640, DOI 10.1093/oxfordjournals.molbev.a004123
Odermatt A, 2011, AM J PHYSIOL-RENAL, V301, pF919, DOI 10.1152/ajprenal.00068.2011
Oliver J, 1934, ARCH PATHOL, V18, P777
Oliver J, 1935, ARCH PATHOL, V19, P1
OLIVER J, 1950, J UROLOGY, V63, P373, DOI 10.1016/S0022-5347(17)68776-7
Oliver J., 1939, ARCHITECTURE KIDNEY
Pacher P, 2008, AM J PHYSIOL-RENAL, V295, pF1303, DOI 10.1152/ajprenal.90343.2008
Packer M, 2020, J AM SOC NEPHROL, V31, P907, DOI 10.1681/ASN.2020010010
Paldi A, 2012, PROG BIOPHYS MOL BIO, V110, P41, DOI 10.1016/j.pbiomolbio.2012.04.003
Papadimitriou A, 2015, AM J PHYSIOL-RENAL, V308, pF209, DOI 10.1152/ajprenal.00252.2014
Papandreou I, 2006, CELL METAB, V3, P187, DOI 10.1016/j.cmet.2006.01.012
PAREKH N, 1981, KIDNEY INT, V19, P306, DOI 10.1038/ki.1981.21
Parikh SM, 2018, NATURE, V563, P332, DOI 10.1038/d41586-018-07088-4

Park J, 2018, SCIENCE, V360, P758, DOI 10.1126/science.aar2131
Parlakgül G, 2022, NATURE, V603, P736, DOI 10.1038/s41586-022-04488-5
Patel SA, 2022, NATURE, V606, P999, DOI 10.1038/s41586-022-04809-8
Pavenstädt H, 2003, PHYSIOL REV, V83, P253, DOI 10.1152/physrev.00020.2002
Perlman R, 2020, INTEGRATING EVOLUTIONARY BIOLOGY INTO MEDICAL EDUCATION: FOR MATERNAL AND CHILD HEALTHCARE STUDENTS, CLINICIANS, AND SCIENTISTS, P119, DOI 10.1093/oso/9780198814153.003.0007
Piccoli GB, 2015, J AM SOC NEPHROL, V26, P2011, DOI 10.1681/ASN.2014050459
Piret SE, 2021, KIDNEY INT, V100, P1250, DOI 10.1016/j.kint.2021.08.031
Pomatto LCD, 2017, J PHYSIOL-LONDON, V595, P7275, DOI 10.1113/JP275072
Pontzer H, 2021, SCIENCE, V373, P808, DOI 10.1126/science.abe5017
Priolo C, 2013, NAT MED, V19, P407, DOI 10.1038/nm.3140
Pruijm M, 2018, KIDNEY INT, V93, P932, DOI 10.1016/j.kint.2017.10.020
Puelles VG, 2011, CURR OPIN NEPHROL HY, V20, P7, DOI 10.1097/MNH.0b013e3283410a7d
Qiu CX, 2018, NAT MED, V24, P1721, DOI 10.1038/s41591-018-0194-4
Raghubar AM, 2022, FRONT MED-LAUSANNE, V9, DOI 10.3389/fmed.2022.873923
Rajaram RD, 2019, NEPHROL DIAL TRANSPL, V34, P567, DOI 10.1093/ndt/gfy161
Rao M, 2009, J AM SOC NEPHROL, V20, P189, DOI 10.1681/ASN.2007091031
Raymond J, 2006, SCIENCE, V311, P1764, DOI 10.1126/science.1118439
Rhinn M, 2019, DEVELOPMENT, V146, DOI 10.1242/dev.151837
Richter R, 1999, J CHROMATOGR B, V726, P25, DOI 10.1016/S0378-4347(99)00012-2
Riddle MR, 2018, NATURE, V555, P647, DOI 10.1038/nature26136
Robin JP, 1998, AM J PHYSIOL-REG I, V274, pR746, DOI 10.1152/ajpregu.1998.274.3.R746
Rodríguez-Trejo A, 2012, AM J PHYSIOL-ENDOC M, V302, pE1431, DOI 10.1152/ajpendo.00619.2011
ROSEN S, 1992, RENAL FAILURE, V14, P321, DOI 10.3109/08860229209106636
Rosenberger C, 2002, J AM SOC NEPHROL, V13, DOI 10.1097/01.ASN.0000017223.49823.2A
Rossier BC, 2015, PHYSIOL REV, V95, P297, DOI 10.1152/physrev.00011.2014
Roustan V, 2016, J EXP BOT, V67, P3897, DOI 10.1093/jxb/erw211
Rowe I, 2013, NAT MED, V19, P488, DOI 10.1038/nm.3092
Ruan XZ, 2008, AM J PHYSIOL-RENAL, V294, pF1032, DOI 10.1152/ajprenal.00152.2007
Ryan D, 2018, EBIOMEDICINE, V27, P275, DOI 10.1016/j.ebiom.2017.12.016
Rylander C, 2013, GLOBAL HEALTH ACTION, V6, DOI 10.3402/gha.v6i0.19538
Rys-Sikora KE, 2013, CLIN J AM SOC NEPHRO, V8, P1599, DOI 10.2215/CJN.03590413
Saeki S, 2020, KIDNEY INT, V98, P88, DOI 10.1016/j.kint.2020.02.024
Saleem MA, 2008, AM J PHYSIOL-RENAL, V295, pF959, DOI 10.1152/ajprenal.00559.2007
Saleem MA, 2015, NEPHROL DIAL TRANSPL, V30, P1266, DOI 10.1093/ndt/gfu363
Sas KM, 2016, JCI INSIGHT, V1, DOI 10.1172/jci.insight.86976
Schaub JA, 2021, KIDNEY360, V2, P355, DOI 10.34067/KID.0004772020

Schaub JA, 2020, CLIN J AM SOC NEPHRO, V15, P695, DOI 10.2215/CJN.09990819
Scheffers BR, 2016, SCIENCE, V354, DOI 10.1126/science.aaf7671
Schmitt R, 2017, KIDNEY INT, V92, P569, DOI 10.1016/j.kint.2017.02.036
Schuh CD, 2018, J AM SOC NEPHROL, V29, P2696, DOI 10.1681/ASN.2018050522
Schulte K, 2015, NEPHROL DIAL TRANSPL, V30, P713, DOI 10.1093/ndt/gfu275
Schumacher B, 2021, NATURE, V592, P695, DOI 10.1038/s41586-021-03307-7
Schwartz L, 2018, J CELL MOL MED, V22, P738, DOI 10.1111/jcmm.13417
Sebastian A, 2002, AM J CLIN NUTR, V76, P1308, DOI 10.1093/ajcn/76.6.1308
Sedeek M, 2013, J AM SOC NEPHROL, V24, P1512, DOI 10.1681/ASN.2012111112
See EJ, 2019, KIDNEY INT, V95, P160, DOI 10.1016/j.kint.2018.08.036
Seebacher F, 2018, COMP BIOCHEM PHYS B, V224, P195, DOI 10.1016/j.cbpb.2017.11.002
Selamet U, 2016, KIDNEY INT, V89, P176, DOI 10.1038/ki.2015.284
Seong SY, 2004, NAT REV IMMUNOL, V4, P469, DOI 10.1038/nri1372
Sequeira-Lopez MLS, 2021, CIRC RES, V128, P887, DOI 10.1161/CIRCRESAHA.121.318064
Shankland SJ, 2000, AM J PHYSIOL-RENAL, V278, pF515, DOI 10.1152/ajprenal.2000.278.4.F515
Shoji K, 2014, CURR OPIN NEPHROL HY, V23, P161, DOI 10.1097/01.mnh.0000441049.98664.6c
Singer MA, 2001, AM J KIDNEY DIS, V37, P164, DOI 10.1016/S0272-6386(01)80073-1
Skinner MK, 2015, GENOME BIOL EVOL, V7, P1296, DOI 10.1093/gbe/evv073
Smith E, 2004, P NATL ACAD SCI USA, V101, P13168, DOI 10.1073/pnas.0404922101
Smith H. W., 1953, From fish to philosopher
SMITH HOMER W., 1933, SIGMA XI QUART, V21, P141
Smith HW., 1943, LECT KIDNEY
Smith RL, 2018, ENDOCR REV, V39, P489, DOI 10.1210/er.2017-00211
Soeters MR, 2012, CLIN NUTR, V31, P1002, DOI 10.1016/j.clnu.2012.05.011
Sokolova IM, 2012, MAR ENVIRON RES, V79, P1, DOI 10.1016/j.marenvres.2012.04.003
SOLTOFF SP, 1986, ANNU REV PHYSIOL, V48, P9
Somero G.N., 2017, Biochemical Adaptation: Response to Environmental Challenges from Life's Origins to the Anthropocene
Sookoian S, 2013, PEDIATR RES, V73, P531, DOI 10.1038/pr.2013.2
Speakman JR, 2020, NATL SCI REV, V7, P1153, DOI 10.1093/nsr/nwaa078
Speakman JR, 2010, J ANIM ECOL, V79, P726, DOI 10.1111/j.1365-2656.2010.01689.x
STECKER JF, 1971, SURG GYNECOL OBSTETR, V133, P846
Stenvinkel P, 2013, AM J KIDNEY DIS, V62, P339, DOI 10.1053/j.ajkd.2012.11.051
Stenvinkel P, 2013, KIDNEY INT, V83, P207, DOI 10.1038/ki.2012.396
Stieger N, 2012, METABOLISM, V61, P1073, DOI 10.1016/j.metabol.2011.12.003
Stokman MF, 2016, NAT REV NEPHROL, V12, P472, DOI 10.1038/nrneph.2016.87
Storey K., 2004, Functional metabolism: regulation and adpatation

Storey KB, 2011, RES REP BIOL, V2, P57, DOI 10.2147/RRB.S13351
Straub RH, 2017, NAT REV RHEUMATOL, V13, P743, DOI 10.1038/nrrheum.2017.172
Straub RH, 2016, EVOL MED PUBLIC HLTH, P37, DOI 10.1093/emph/eow001
Su J, 2020, MOL BIOL REP, V47, P8023, DOI 10.1007/s11033-020-05749-0
Sun J, 2019, J AM SOC NEPHROL, V30, P1151, DOI 10.1681/ASN.2018111126
Szeto HH, 2017, J AM SOC NEPHROL, V28, P2856, DOI 10.1681/ASN.2017030247
Szeto HH, 2017, J AM SOC NEPHROL, V28, P1437, DOI 10.1681/ASN.2016070761
Szeto HH, 2011, J AM SOC NEPHROL, V22, P1041, DOI 10.1681/ASN.2010080808
TANAKA R, 1995, KIDNEY INT, V47, P131, DOI 10.1038/ki.1995.15
Tanaka T, 2009, J PHARMACOL SCI, V109, P24, DOI 10.1254/jphs.08R09FM
Tannahill GM, 2013, NATURE, V496, P238, DOI 10.1038/nature11986
Taylor SR, 2021, NATURE, V597, P263, DOI [10.1038/s41586-021-03827-2, https://doi.org/10.1038/d41586-021-02195-1]
Thompson CB, 2011, COLD SH Q B, V76, P23, DOI 10.1101/sqb.2012.76.010496
Tian ZM, 2021, NAT COMMUN, V12, DOI 10.1038/s41467-021-21301-5
Tierney JE, 2020, SCIENCE, V370, P680, DOI 10.1126/science.aay3701
Tinbergen N., 1963, Zeitschrift fuer Tierpsychologie, V20, P410
Torres R, 2016, J AM SOC NEPHROL, V27, P1102, DOI 10.1681/ASN.2015010079
Tortelote GG, 2021, PEDIATR NEPHROL, V36, P2155, DOI 10.1007/s00467-020-04752-8
Tran M, 2011, J CLIN INVEST, V121, P4003, DOI 10.1172/JCI58662
Tran MT, 2016, NATURE, V531, P528, DOI 10.1038/nature17184
Trewavas A, 2006, PLANT CELL, V18, P2420, DOI 10.1105/tpc.106.042267
UCHIDA S, 1988, AM J PHYSIOL, V255, pF977, DOI 10.1152/ajprenal.1988.255.5.F977
Valcourt JR, 2012, CELL CYCLE, V11, P1680, DOI 10.4161/cc.19879
VALTIN H, 1977, AM J PHYSIOL, V233, pF491, DOI 10.1152/ajprenal.1977.233.6.F491
VANCURA P, 1973, AM J PHYSIOL, V225, P281, DOI 10.1152/ajplegacy.1973.225.2.281
Vaziri ND, 2003, KIDNEY INT, V63, P179, DOI 10.1046/j.1523-1755.2003.00702.x
VELTRI KL, 1990, J CELL PHYSIOL, V143, P160, DOI 10.1002/jcp.1041430122
Verissimo T, 2022, J AM SOC NEPHROL, V33, P810, DOI 10.1681/ASN.2021050680
Visarius TM, 1996, BIOCHEM PHARMACOL, V52, P259, DOI 10.1016/0006-2952(96)00203-1
Volovelsky O, 2018, P NATL ACAD SCI USA, V115, P5998, DOI 10.1073/pnas.1712955115
Wallace DC, 2011, COLD SH Q B, V76, P1, DOI 10.1101/sqb.2011.76.010462
Wallace DC, 2016, NATURE, V535, P498, DOI 10.1038/nature18902
Wallace DC, 2013, J CLIN INVEST, V123, P1405, DOI 10.1172/JCI61398
Wallace DC, 2010, MITOCHONDRION, V10, P12, DOI 10.1016/j.mito.2009.09.006
Wang LL, 2012, J APPL PHYSIOL, V113, P1669, DOI 10.1152/japplphysiol.00658.2012

Wanner N, 2014, J AM SOC NEPHROL, V25, P707, DOI 10.1681/ASN.2013050452
Wells JCK, 2011, AM J HUM BIOL, V23, P65, DOI 10.1002/ajhb.21100
Wen B, 2022, LANCET REG HEALTH-AM, V6, DOI 10.1016/j.lana.2021.100101
Wen J, 2012, FERTIL STERIL, V97, P1331, DOI 10.1016/j.fertnstert.2012.02.053
Whitaker RM, 2015, KIDNEY INT, V88, P1336, DOI 10.1038/ki.2015.240
Wilcox CS, 2005, AM J PHYSIOL-REG I, V289, pR913, DOI 10.1152/ajpregu.00250.2005
Wilks A, 2002, ANTIOXID REDOX SIGN, V4, P603, DOI 10.1089/15230860260220102
WILLIAMS GC, 1957, EVOLUTION, V11, P398, DOI 10.1111/j.1558-5646.1957.tb02911.x
WILLIAMS GC, 1991, Q REV BIOL, V66, P1, DOI 10.1086/417048
WOLF G, 1993, J AM SOC NEPHROL, V3, P1531
Wolowczuk I, 2008, CLIN DEV IMMUNOL, P1, DOI 10.1155/2008/639803
Xie Y, 2018, KIDNEY INT, V94, P567, DOI 10.1016/j.kint.2018.04.011
Yamamoto S, 2020, J AM SOC NEPHROL, V31, P2855, DOI 10.1681/ASN.2020050580
Yang HC, 2009, J AM SOC NEPHROL, V20, P2380, DOI 10.1681/ASN.2008111138
Yang HC, 2010, J AM SOC NEPHROL, V21, P1436, DOI 10.1681/ASN.2010020205
Yang J, 2017, J SYST EVOL, V55, P71, DOI 10.1111/jse.12212
Yang KK, 2016, J RENIN-ANGIO-ALDO S, V17, DOI 10.1177/1470320316673231
Yang YH, 2003, KIDNEY INT, V64, P1302, DOI 10.1046/j.1523-1755.2003.00231.x
Youle RJ, 2019, SCIENCE, V365, P655, DOI 10.1126/science.aaw9855
Yu F, 2005, AM J PHYSIOL-RENAL, V289, pF514, DOI 10.1152/ajprenal.00101.2005
Zehra T, 2021, J AM SOC NEPHROL, V32, P1293, DOI 10.1681/ASN.2020040423
Zera AJ, 2011, J EXP BIOL, V214, P179, DOI 10.1242/jeb.046912
Zhang FJ, 2013, J AM SOC NEPHROL, V24, P209, DOI 10.1681/ASN.2012080795
Zhang Q, 2010, NATURE, V464, P104, DOI 10.1038/nature08780
Zheng SQ, 2018, INT J MOL SCI, V19, DOI 10.3390/ijms19041154
Zhou LL, 2019, KIDNEY INT, V95, P830, DOI 10.1016/j.kint.2018.10.032
Zou J, 2021, KIDNEY INT, V99, P620, DOI 10.1016/j.kint.2020.09.031
NR 423
TC 5
Z9 5
U1 8
U2 16
PU AMER PHYSIOLOGICAL SOC
PI Rockville
PA 6120 Executive Blvd, Suite 600, Rockville, MD, UNITED STATES
SN 0031-9333
EI 1522-1210
J9 PHYSIOL REV
JI Physiol. Rev.
PD OCT 1
PY 2023
VL 103

IS 4
BP 2451
EP 2506
DI 10.1152/physrev.00029.2022
PG 57
WC Physiology
WE Science Citation Index Expanded (SCI-EXPANDED)
SC Physiology
GA N0UN0
UT WOS:001034269500003
PM 36996412
DA 2025-05-07
ER

PT J
AU Vijendravarma, RK
AF Vijendravarma, Roshan K.
TI Experimental evolution demonstrates evolvability of preferential nutrient allocation to competing traits in response to chronic malnutrition
SO JOURNAL OF EVOLUTIONARY BIOLOGY
LA English
DT Article
DE Drosophila melanogaster; geometric framework of nutrition; mismatch hypothesis; Protein : carbohydrate ratio; Smurf phenotype
ID HISTORY TRADE-OFFS; RESOURCE-ALLOCATION; PHYSIOLOGY; CONSEQUENCES; ADAPTATION; SIZE
AB Investigating the evolutionary origins of disease vulnerability is an important aspect of evolutionary medicine that strongly complements our current understanding on proximate causes of disease. Life-history trade-offs mediated through evolutionary changes in resource allocation strategies could be one possible explanation to why suboptimal traits that leave bodies vulnerable to disease exist. For example, Drosophila melanogaster populations experimentally evolved to tolerate chronic larval malnutrition succumb to intestinal infection despite eliciting a competent immune response, owing to the loss of their intestinal integrity. Here, I test whether evolved changes in resource allocation underlies this trade-off, by assaying preferential allocation of dietary protein towards growth and tissue repair in the same populations. Using two phenotypic traits, regeneration of intestinal epithelium post-pathogenic infection and body weight, I show that in accordance with the dynamic energy budget theory (DEB) dietary protein acquired during the larval phase is allocated to both growth and adult tissue repair. Furthermore, by altering the ratio of protein and carbohydrates in the larval diets I demonstrate that in comparison with the control populations, the evolved (selected) populations differ in their protein allocation strategy towards these two traits. While the control populations stored away excess protein for tissue repair, the selected populations invested it towards immediate increase in body weight rather than towards an unanticipated tissue damage. Thus, I show how macronutrient availability and their allocation between traits can alter resistance, and provide empirical evidence that supports the 'mismatch hypothesis', wherein vulnerability to disease is proposed to stem from the differences between ancestral and current environment.

C1 [Vijendravarma, Roshan K.] Univ Lausanne, Dept Ecol & Evolut, CH-1015 Lausanne, Switzerland.
[Vijendravarma, Roshan K.] Inst Curie, Ctr Rech, Genet & Dev Biol Unit, INSERM,U934,CNRS,UMR3215, 26 Rue Ulm, F-75248 Paris 05, France.
C3 University of Lausanne; Universite PSL; UNICANCER; Institut Curie; Centre National de la Recherche Scientifique (CNRS); Institut National de la Sante et de la Recherche Medicale (Inserm)
RP Vijendravarma, RK (autor correspondente), Univ Lausanne, Dept Ecol & Evolut, CH-1015 Lausanne, Switzerland.; Vijendravarma, RK (autor correspondente), Inst Curie, Ctr Rech, Genet & Dev Biol Unit, INSERM,U934,CNRS,UMR3215, 26 Rue Ulm, F-75248 Paris 05, France.
EM roshankumar.vijendravarma@gmail.com
RI Vijendravarma, Roshan Kumar/E-7746-2018
OI Vijendravarma, Roshan Kumar/0000-0002-3274-0682; Vijendravarma, Roshan Kumar/0000-0002-4930-3040
FU Swiss National Science Foundation [31003A_143939]; Swiss National Science Foundation (SNF) [31003A_143939] Funding Source: Swiss National
Science Foundation (SNF)
FX R.K.V was supported by the Swiss National Science Foundation Grant (# 31003A_143939) awarded to T.J. Kawecki.
CR Alhagamhmad M. H., 2015, J GASTROEN HEPATOL, V4, P1805
Bourg S., 2017, 169904 BIORXIV
Clark RM, 2015, J EXP BIOL, V218, P298, DOI 10.1242/jeb.112888
Fellous S, 2010, MOL ECOL, V19, P1462, DOI 10.1111/j.1365-294X.2010.04567.x
Godfrey KM, 2007, PEDIATR RES, V61, p5R, DOI 10.1203/pdr.0b013e318045bedb
Katona P, 2008, CLIN INFECT DIS, V46, P1582, DOI 10.1086/587658
Kawecki TJ, 2012, TRENDS ECOL EVOL, V27, P547, DOI 10.1016/j.tree.2012.06.001
King EG, 2010, AM NAT, V175, P702, DOI 10.1086/652434
Kolss M, 2009, EVOLUTION, V63, P2389, DOI 10.1111/j.1558-5646.2009.00718.x
Kristensen TN, 2011, BIOL LETTERS, V7, P269, DOI 10.1098/rsbl.2010.0872
Lee KP, 2008, FUNCT ECOL, V22, P1052, DOI 10.1111/j.1365-2435.2008.01459.x
Leroi AM, 2001, TRENDS ECOL EVOL, V16, P24, DOI 10.1016/S0169-5347(00)02032-2
Llandres AL, 2015, ECOL MONOGR, V85, P353, DOI 10.1890/14-0976.1
McDade TW, 2005, ANNU REV ANTHROPOL, V34, P495, DOI 10.1146/annurev.anthro.34.081804.120348
Nesse RM, 2011, EVOL APPL, V4, P264, DOI 10.1111/j.1752-4571.2010.00181.x
Ng'oma E, 2017, P ROY SOC B-BIOL SCI, V284, DOI 10.1098/rspb.2017.0445
PARKER GA, 1990, NATURE, V348, P27, DOI 10.1038/348027a0
Raubenheimer D, 2012, PHILOS T R SOC B, V367, P1628, DOI 10.1098/rstb.2012.0007
Rauw Wendy M., 2012, Frontiers in Genetics, V3, P267, DOI 10.3389/fgene.2012.00267
Rera M, 2012, P NATL ACAD SCI USA, V109, P21528, DOI 10.1073/pnas.1215849110
Roff Derek, 2002, pi

Roff DA, 2012, EVOLUTION, V66, P2461, DOI 10.1111/j.1558-5646.2012.01634.x
Simmons FH, 1997, J INSECT PHYSIOL, V43, P779, DOI 10.1016/S0022-1910(97)00037-1
Simpson S.J., 2012, NATURE NUTR UNIFYING
Stearns S.C., 1992, pi
VAN NOORDWIJK AJ, 1986, AM NAT, V128, P137, DOI 10.1086/284547
Vijendravarma RK, 2012, J EVOLUTION BIOL, V25, P288, DOI 10.1111/j.1420-9101.2011.02419.x
Vijendravarma RK, 2011, J EVOLUTION BIOL, V24, P897, DOI 10.1111/j.1420-9101.2010.02225.x
Vijendravarma RK, 2015, ECOL LETT, V18, P1078, DOI 10.1111/ele.12490
Vijendravarma RK, 2013, NAT COMMUN, V4, DOI 10.1038/ncomms2744
Vijendravarma RK, 2012, P ROY SOC B-BIOL SCI, V279, P3540, DOI 10.1098/rspb.2012.0966
Vijendravarma RK, 2012, PLOS ONE, V7, DOI 10.1371/journal.pone.0030650
Vodovar N, 2005, P NATL ACAD SCI USA, V102, P11414, DOI 10.1073/pnas.0502240102
Worley AC, 2003, AM NAT, V161, P153, DOI 10.1086/345461
Zera AJ, 2001, ANNU REV ECOL SYST, V32, P95, DOI 10.1146/annurev.ecolsys.32.081501.114006
NR 35
TC 2
Z9 2
U1 0
U2 14
PU WILEY
PI HOBOKEN
PA 111 RIVER ST, HOBOKEN 07030-5774, NJ USA
SN 1010-061X
EI 1420-9101
J9 J EVOLUTION BIOL
JI J. Evol. Biol.
PD NOV
PY 2018
VL 31
IS 11
BP 1743
EP 1749
DI 10.1111/jeb.13359
PG 7
WC Ecology; Evolutionary Biology; Genetics & Heredity
WE Science Citation Index Expanded (SCI-EXPANDED)
SC Environmental Sciences & Ecology; Evolutionary Biology; Genetics & Heredity
GA HC4OP
UT WOS:000451783400013
PM 30075055
OA Green Submitted
DA 2025-05-07
ER

PT J
AU Smyth, K

Morton, C
Mathew, A
Karuturi, S
Haley, C
Zhang, M
Holzknecht, ZE
Swanson, C
Lin, SS
Parker, W
AF Smyth, Kendra
Morton, Claire
Mathew, Amanda
Karuturi, Sahil
Haley, Cliff
Zhang, Min
Holzknecht, Zoie E.
Swanson, Chelsea
Lin, Shu S.
Parker, William
TI Production and Use of Hymenolepis diminuta Cysticercoids as Anti-Inflammatory Therapeutics
SO JOURNAL OF CLINICAL MEDICINE
LA English
DT Article
DE helminthic therapy; helminth; biological therapeutic; inflammation; anti-inflammatory
ID EXPERIMENTAL HOOKWORM INFECTION; TRICHURIS-SUIS THERAPY; PARASITES; HELMINTHS; COLITIS; RECONSTITUTION; MULTIPLE; BIOME
AB Helminthic therapy has shown considerable promise as a means of alleviating some inflammatory diseases that have proven resistant to pharmaceutical intervention. However, research in the field has been limited by a lack of availability to clinician scientists of a helminth that is relatively benign, non-communicable, affordable, and effectively treats disease. Previous socio-medical studies have found that some individuals self-treating with helminths to alleviate various diseases are using the rat tapeworm (cysticercoid developmental stage of Hymenolepis diminuta; HDC). In this study, we describe the production and use of HDCs in a manner that is based on reports from individuals self-treating with helminths, individuals producing helminths for self-treatment, and physicians monitoring patients that are self-treating. The helminth may fit the criteria needed by clinical scientists for clinical trials, and the methodology is apparently feasible for any medical center to reproduce. It is hoped that future clinical trials using this organism may shed light on the potential for helminthic therapy to alleviate inflammatory diseases. Further, it is hoped that studies with HDCs may provide a stepping stone toward population-wide restoration of the biota of the human body, potentially reversing the inflammatory consequences of biota depletion that currently affect Western society.
C1 [Smyth, Kendra] Duke Univ, Univ Program Ecol, Durham, NC 27708 USA.
[Morton, Claire; Mathew, Amanda; Karuturi, Sahil; Haley, Cliff; Zhang, Min; Holzknecht, Zoie E.; Lin, Shu S.; Parker, William] Duke Univ, Med Ctr, Dept Surg, Durham, NC 27710 USA.
[Swanson, Chelsea] Duke Univ, Med Ctr, Duke Brain Imaging & Anal Ctr, Durham, NC 27710 USA.

[Swanson, Chelsea] Durham VA Med Ctr, Mental Illness Res Educ & Clin Ctr Post Deploymen, Durham, NC 27710 USA.
[Lin, Shu S.] Duke Univ, Med Ctr, Dept Immunol, Durham, NC 27710 USA.
[Lin, Shu S.] Duke Univ, Med Ctr, Dept Pathol, Durham, NC 27710 USA.
C3 Duke University; Duke University; Duke University; US Department of Veterans Affairs; Veterans Health Administration (VHA); Durham VA Medical Center; Duke University; Duke University
RP Parker, W (autor correspondente), Duke Univ, Med Ctr, Dept Surg, Durham, NC 27710 USA.
EM kendra.smyth@gmail.com; claire.morton@duke.edu; amanda.mathew@duke.edu;
sahilk2000@gmail.com; cliffhaley222@gmail.com; Min.zhang39@duke.edu;
zoie.holzknecht@duke.edu; chelsea.swanson@duke.edu; shu.lin@duke.edu;
william.parker@duke.edu
RI Zhang, Min/ABE-9844-2021
OI Morton, Claire/0000-0002-8855-3667
FU Immunity's Forge Foundation; SafeMinds; Triangle Center for Evolutionary Medicine; Duke University's Office of Undergraduate Research Support
FX This work was supported in part by funds from the Immunity's Forge Foundation and from SafeMinds, by a graduate fellowship to KS from the Triangle Center for Evolutionary Medicine, and by assistantship funding to CM from Duke University's Office of Undergraduate Research Support.
CR [Anonymous], 2016, CLIN RES REGUL AFF, DOI DOI 10.1080/10601333.2016.1210159
ARAI H.P., 1980, BIOL TAPEWORM HYMENO
Bethony J, 2006, LANCET, V367, P1521, DOI 10.1016/S0140-6736(06)68653-4
Bilbo SD, 2011, MED HYPOTHESES, V77, P494, DOI 10.1016/j.mehy.2011.06.019
Cheng A.M., 2015, J EVOLUT MED
Correale J, 2007, ANN NEUROL, V61, P97, DOI 10.1002/ana.21067
Correale J, 2011, J NEUROIMMUNOL, V233, P6, DOI 10.1016/j.jneuroim.2011.01.002
Croese J, 2015, J ALLERGY CLIN IMMUN, V135, P508, DOI 10.1016/j.jaci.2014.07.022
Daveson AJ, 2011, PLOS ONE, V6, DOI 10.1371/journal.pone.0017366
Elliott DE, 2005, CURR OPIN GASTROEN, V21, P51
Feary J, 2009, CLIN EXP ALLERGY, V39, P1060, DOI 10.1111/j.1365-2222.2009.03187.x
Feary JR, 2010, CLIN EXP ALLERGY, V40, P299, DOI 10.1111/j.1365-2222.2009.03433.x
Flohr C, 2009, CLIN EXP ALLERGY, V39, P20, DOI 10.1111/j.1365-2222.2008.03134.x
Flowers S, 2013, NATURE, V493, P163, DOI 10.1038/493163c
Fumagalli M, 2009, J EXP MED, V206, P1395, DOI 10.1084/jem.20082779
Hamad I, 2014, SCI REP-UK, V4, DOI 10.1038/srep04478
Helmby H, 2015, BMC IMMUNOL, V16, DOI 10.1186/s12865-015-0074-3
Hewitson JP, 2009, MOL BIOCHEM PARASIT, V167, P1, DOI 10.1016/j.molbiopara.2009.04.008
Hotez PJ, 2005, PLOS MED, V2, P187, DOI 10.1371/journal.pmed.0020067
Imai S, 2004, CURR TOP MED CHEM, V4, P539, DOI 10.2174/1568026043451285

Johnston MJG, 2010, INFECT IMMUN, V78, P1364, DOI 10.1128/IAI.01349-08
Khan AR, 2013, INT J PARASITOL, V43, P293, DOI 10.1016/j.ijpara.2012.12.002
Léon-Cabrera S, 2014, INT J BIOL SCI, V10, P948, DOI 10.7150/ijbs.9033
Liu J, 2017, J HELMINTHOL, V91, P267, DOI 10.1017/S0022149X16000316
Lukes J, 2014, TRENDS PARASITOL, V30, P377, DOI 10.1016/j.pt.2014.06.005
Maizels RM, 2005, CURR OPIN IMMUNOL, V17, P656, DOI 10.1016/j.coi.2005.09.001
McCafferty K., 2010, FIELD STREAM, V8, P22
McKay DM, 2015, PARASITE IMMUNOL, V37, P324, DOI 10.1111/pim.12160
Melon A, 2010, J BIOMED BIOTECHNOL, DOI 10.1155/2010/384523
Parker William, 2013, Evolution Medicine and Public Health, P89, DOI 10.1093/emph/eot008
Parker W, 2012, CURR MED RES OPIN, V28, P1193, DOI 10.1185/03007995.2012.695731
Pastille E, 2017, PLOS PATHOG, V13, DOI 10.1371/journal.ppat.1006649
Pi C, 2015, PLOS ONE, V10, DOI 10.1371/journal.pone.0120255
Rook GAW, 2014, ADV EXP MED BIOL, V817, P319, DOI 10.1007/978-1-4939-0897-4_15
Scavetta R., 2009, SURVIVAL SCH OUT YOU
Summers RW, 2005, GASTROENTEROLOGY, V128, P825, DOI 10.1053/j.gastro.2005.01.005
Summers RW, 2005, GUT, V54, P87, DOI 10.1136/gut.2004.041749
Summers RW, 2003, AM J GASTROENTEROL, V98, P2034, DOI [10.1016/S0002-9270(03)00623-3, 10.1111/j.1572-0241.2003.07660.x]
Tilp C, 2013, INT J PARASITOL, V43, P319, DOI 10.1016/j.ijpara.2012.12.003
TURTON JA, 1976, LANCET, V2, P686
van Huis A, 2013, EDIBLE INSECTS FUTUR, P171
Wang A, 2017, PARASITE IMMUNOL, V39, DOI 10.1111/pim.12490
Williamson LL, 2016, BRAIN BEHAV IMMUN, V51, P14, DOI 10.1016/j.bbi.2015.07.006
Wolff MJ, 2012, TRENDS PARASITOL, V28, P187, DOI 10.1016/j.pt.2012.02.008
Zaccone P, 2006, PARASITE IMMUNOL, V28, P515, DOI 10.1111/j.1365-3024.2006.00879.x
NR 45
TC 14
Z9 15
U1 1
U2 11
PU MDPI
PI BASEL
PA ST ALBAN-ANLAGE 66, CH-4052 BASEL, SWITZERLAND
EI 2077-0383
J9 J CLIN MED
JI J. Clin. Med.
PD OCT
PY 2017
VL 6
IS 10
AR 98
DI 10.3390/jcm6100098

PG 20
WC Medicine, General & Internal
WE Science Citation Index Expanded (SCI-EXPANDED)
SC General & Internal Medicine
GA FR7KA
UT WOS:000419245500008
PM 29064448
OA gold, Green Published, Green Submitted
DA 2025-05-07
ER

PT J
AU Chung, CJ
Hermes, BM
Gupta, Y
Ibrahim, S
Belheouane, M
Baines, JF
AF Chung, C. J.
Hermes, B. M.
Gupta, Y.
Ibrahim, S.
Belheouane, Meriem
Baines, John F.
TI Genome-wide mapping of gene-microbe interactions in the murine lung microbiota based on quantitative microbial profiling
SO ANIMAL MICROBIOME
LA English
DT Article
DE Mouse; Lung microbiota; Host genetics; QTL mapping; Quantitative microbial profiling; Lactobacillus; Interleukin 10
ID MAPKAP KINASE 2; RESPIRATORY-TRACT; CANCER CELLS; ASSOCIATION; EXPRESSION; MICE; RISK; IDENTIFICATION; DETERMINANTS; INFLAMMATION
AB Background Mammalian lungs comprise a complex microbial ecosystem that interacts with host physiology. Previous research demonstrates that the environment significantly contributes to bacterial community structure in the upper and lower respiratory tract. However, the influence of host genetics on the makeup of lung microbiota remains ambiguous, largely due to technical difficulties related to sampling, as well as challenges inherent to investigating low biomass communities. Thus, innovative approaches are warranted to clarify host-microbe interactions in the mammalian lung.

Results Here, we aimed to characterize host genomic regions associated with lung bacterial traits in an advanced intercross mouse line (AIL). By performing quantitative microbial profiling (QMP) using the highly precise method of droplet digital PCR (ddPCR), we refined 16S rRNA gene amplicon-based traits to identify and map candidate lung-resident taxa using a QTL mapping approach. In addition, the two abundant core taxa Lactobacillus and Pelomonas were chosen for independent microbial phenotyping using genus-specific primers. In total, this revealed seven significant loci involving eight bacterial traits. The narrow confidence intervals afforded by the AIL population allowed us to identify several promising candidate genes related to immune and inflammatory responses, cell apoptosis, DNA repair, and lung functioning and disease

susceptibility. Interestingly, one genomic region associated with Lactobacillus abundance contains the well-known anti-inflammatory cytokine Il10, which we confirmed through the analysis of Il10 knockout mice.

Conclusions Our study provides the first evidence for a role of host genetic variation contributing to variation in the lung microbiota. This was in large part made possible through the careful curation of 16S rRNA gene amplicon data and the incorporation of a QMP-based methods. This approach to evaluating the low biomass lung environment opens new avenues for advancing lung microbiome research using animal models.
C1 [Chung, C. J.; Hermes, B. M.; Belheouane, Meriem; Baines, John F.] Max Planck Inst Evolutionary Biol, August Thienemann Str 2, D-24306 Plon, Germany.

[Chung, C. J.; Hermes, B. M.; Belheouane, Meriem; Baines, John F.] Univ Kiel, Inst Expt Med, Sect Evolutionary Med, Arnold Heller Str 3, D-24105 Kiel, Germany.

[Gupta, Y.] Columbia Univ Irving Med Ctr, Dept Med, Div Nephrol, New York, NY 10032 USA.

[Ibrahim, S.] Khalifa Univ, Coll Med & Hlth Sci, Abu Dhabi, U Arab Emirates.

[Belheouane, Meriem] Res Ctr Borstel, Leibniz Lung Ctr, Evolut Resistome, Parkallee 1-40, D-23845 Borstel, Germany.

C3 Max Planck Society; University of Kiel; NewYork-Presbyterian Hospital; Khalifa University of Science & Technology; Leibniz Association; Forschungszentrum Borstel

RP Belheouane, M; Baines, JF (autor correspondente), Max Planck Inst Evolutionary Biol, August Thienemann Str 2, D-24306 Plon, Germany.; Belheouane, M; Baines, JF (autor correspondente), Univ Kiel, Inst Expt Med, Sect Evolutionary Med, Arnold Heller Str 3, D-24105 Kiel, Germany.; Belheouane, M (autor correspondente), Res Ctr Borstel, Leibniz Lung Ctr, Evolut Resistome, Parkallee 1-40, D-23845 Borstel, Germany.

EM mbelheouane@fz-borstel.de; baines@evolbio.mpg.de

OI Chung, Cecilia J./0000-0003-2392-1745
FU German Science Foundation (DFG) [EXC2167]; Leibniz Science Campus Evolutionary Medicine of the Lung (EvoLUNG); Projekt DEAL; [CRC 1182]

FX This work was supported by the German Science Foundation (DFG) grants EXC2167 "Precision Medicine in Chronic Inflammation (PMI)", CRC 1182, and the Leibniz Science Campus Evolutionary Medicine of the Lung (EvoLUNG). Open Access funding enabled and organized by Projekt DEAL.
CR Abellan-Schneyder I, 2021, BMC MICROBIOL, V21, DOI 10.1186/s12866-021-02391-z

Ashley SL, 2020, SCI TRANSL MED, V12, DOI 10.1126/scitranslmed.aau9959

Baker JM, 2021, MICROBIOME, V9, DOI 10.1186/s40168-021-01055-4

Barfod Kenneth Klingenberg, 2015, Open Microbiol J, V9, P167, DOI 10.2174/1874285801509010167

Barfod KK, 2013, BMC MICROBIOL, V13, DOI 10.1186/1471-2180-13-303

Barrios J, 2019, AM J RESP CELL MOL, V60, P687, DOI 10.1165/rcmb.2018-0179OC

Barton K., 2015, R Package Version, V15, P1

Bates D., 2012, Package lme4.

Beisswenger C, 2007, J BIOL CHEM, V282, P28700, DOI 10.1074/jbc.M703576200

Belheouane M, 2017, MICROBIOME, V5, DOI 10.1186/s40168-017-0275-5

BENJAMINI Y, 1995, J R STAT SOC B, V57, P289, DOI 10.1111/j.2517-6161.1995.tb02031.x
Benson AK, 2010, P NATL ACAD SCI USA, V107, P18933, DOI 10.1073/pnas.1007028107
Biesbroek G, 2014, AM J RESP CRIT CARE, V190, P298, DOI 10.1164/rccm.201401-0073OC
Blekhman R, 2015, GENOME BIOL, V16, DOI 10.1186/s13059-015-0759-1
Bonder MJ, 2016, NAT GENET, V48, P1407, DOI 10.1038/ng.3663
Brehm JM, 2015, AM J RESP CRIT CARE, V192, P634, DOI 10.1164/rccm.201501-0047LE
Callahan BJ, 2016, NAT METHODS, V13, P581, DOI [10.1038/NMETH.3869, 10.1038/nmeth.3869]
Campbell BJ, 2011, P NATL ACAD SCI USA, V108, P12776, DOI 10.1073/pnas.1101405108
Campbell JH, 2012, ISME J, V6, P2033, DOI 10.1038/ismej.2012.54
Carney SM, 2020, AM J RESP CELL MOL, V62, P283, DOI 10.1165/rcmb.2019-0273TR
Charlson ES, 2011, AM J RESP CRIT CARE, V184, P957, DOI 10.1164/rccm.201104-0655OC
Chaubey S, 2021, PEDIATR RES, V89, P1126, DOI 10.1038/s41390-020-0891-9
Cheng R, 2011, BMC GENET, V12, DOI 10.1186/1471-2156-12-66
Chiba E, 2013, INT IMMUNOPHARMACOL, V17, P373, DOI 10.1016/j.intimp.2013.06.024
Chillappagari S, 2021, J CYST FIBROS, V20, P140, DOI 10.1016/j.jcf.2020.05.006
Coburn B, 2015, SCI REP-UK, V5, DOI 10.1038/srep10241
Cole JR, 2014, NUCLEIC ACIDS RES, V42, pD633, DOI 10.1093/nar/gkt1244
Cougoul A, 2019, PLOS ONE, V14, DOI 10.1371/journal.pone.0200458
Danielsson J, 2016, LUNG, V194, P401, DOI 10.1007/s00408-016-9867-2
Davenport ER, 2015, PLOS ONE, V10, DOI 10.1371/journal.pone.0140301
Davis NM, 2018, MICROBIOME, V6, DOI 10.1186/s40168-018-0605-2
de Vries M, 2019, RESP RES, V20, DOI 10.1186/s12931-019-1222-8
Delroisse JM, 2008, MICROBIOL RES, V163, P663, DOI 10.1016/j.micres.2006.09.004
Dickson RP, 2018, AM J RESP CRIT CARE, V198, P497, DOI 10.1164/rccm.201711-2180OC
Dickson RP, 2016, NAT MICROBIOL, V1, DOI [10.1038/NMICROBIOL.2016.113, 10.1038/nmicrobiol.2016.113]
Ding N, 2022, INT ARCH ALLERGY IMM, V183, P1098, DOI 10.1159/000525063
Drengenes C, 2021, BMC GENOMICS, V22, DOI 10.1186/s12864-020-07252-z
Drengenes C, 2019, BMC MICROBIOL, V19, DOI 10.1186/s12866-019-1560-1
Ehlting C, 2016, J HEPATOL, V64, P380, DOI 10.1016/j.jhep.2015.08.012
Ehlting C, 2011, J BIOL CHEM, V286, P24113, DOI 10.1074/jbc.M111.235275
Eisenhofer R, 2019, TRENDS MICROBIOL, V27, P105, DOI 10.1016/j.tim.2018.11.003
Erb-Downward JR, 2011, PLOS ONE, V6, DOI 10.1371/journal.pone.0016384
Ezendam J, 2008, BRIT J NUTR, V99, P83, DOI 10.1017/S0007114507803412
Fan J, 2020, J INFECTION, V81, pE64, DOI 10.1016/j.jinf.2020.06.047
Forsythe P, 2007, AM J RESP CRIT CARE, V175, P561, DOI 10.1164/rccm.200606-821OC
Gaibani P, 2021, SCI REP-UK, V11, DOI 10.1038/s41598-021-89516-6

Gatti DM, 2014, G3-GENES GENOM GENET, V4, P1623, DOI 10.1534/g3.114.013748
Gobert G, 2018, J MICROBIOL METH, V148, P64, DOI 10.1016/j.mimet.2018.03.004
Goddard AF, 2012, P NATL ACAD SCI USA, V109, P13769, DOI 10.1073/pnas.1107435109
Goodale BC, 2017, TOXICOL APPL PHARM, V331, P154, DOI 10.1016/j.taap.2017.06.010
Goodrich JK, 2016, CELL HOST MICROBE, V19, P731, DOI 10.1016/j.chom.2016.04.017
Goodrich JK, 2014, CELL, V158, P250, DOI 10.1016/j.cell.2014.06.037
Grab Jessica, 2019, Microbiol Insights, V12, p1178636119864594, DOI 10.1177/1178636119864594
Guo MZ, 2019, NAT COMMUN, V10, DOI 10.1038/s41467-018-07770-1
Güre AO, 2000, P NATL ACAD SCI USA, V97, P4198, DOI 10.1073/pnas.97.8.4198
Heirali AA, 2017, MICROBIOME, V5, DOI 10.1186/s40168-017-0265-7
Hilty M, 2010, PLOS ONE, V5, DOI 10.1371/journal.pone.0008578
Hosgood HD, 2014, ENVIRON MOL MUTAGEN, V55, P643, DOI 10.1002/em.21878
Humphrey PT, 2020, NAT ECOL EVOL, V4, P221, DOI 10.1038/s41559-019-1085-x
Ishii J, 2014, PATHOL INT, V64, P415, DOI 10.1111/pin.12198
Ishii J, 2013, PATHOL INT, V63, P158, DOI 10.1111/pin.12042
Jervis-Bardy J, 2015, MICROBIOME, V3, DOI 10.1186/s40168-015-0083-8
Jiang P, 2021, RESP RES, V22, DOI 10.1186/s12931-021-01918-2
Karstens L, 2019, MSYSTEMS, V4, DOI 10.1128/mSystems.00290-19
Kostric M, 2017, MICROB ECOL, P1
Kotlyarov A, 1999, NAT CELL BIOL, V1, P94, DOI 10.1038/10061
Kovacs A, 2011, MICROB ECOL, V61, P423, DOI 10.1007/s00248-010-9787-2
Lee Y, 2013, CARCINOGENESIS, V34, P307, DOI 10.1093/carcin/bgs356
Lignitto L, 2019, CELL, V178, P316, DOI 10.1016/j.cell.2019.06.003
Lipinski JH, 2021, AM J PHYSIOL-LUNG C, V321, pL404, DOI 10.1152/ajplung.00002.2021
Liu AB, 2021, MOL THER ONCOLYTICS, V22, P195, DOI 10.1016/j.omto.2021.05.008
Liu B, 2014, INT J CLIN EXP PATHO, V7, P7863
Liu B, 2012, AM J HUM GENET, V91, P384, DOI 10.1016/j.ajhg.2012.07.003
Liu DD, 2019, J CANCER, V10, P3381, DOI 10.7150/jca.31553
Lu BX, 2007, CELL, V129, P371, DOI 10.1016/j.cell.2007.02.041
Lyon H, 2004, GENET EPIDEMIOL, V26, P155, DOI 10.1002/gepi.10298
Man WH, 2017, NAT REV MICROBIOL, V15, P259, DOI 10.1038/nrmicro.2017.14
Manichaikul A, 2007, GENETICS, V177, P1963, DOI 10.1534/genetics.107.080093
Männistö MK, 2013, FEMS MICROBIOL ECOL, V84, P47, DOI 10.1111/1574-6941.12035
McMillin MJ, 2013, AM J HUM GENET, V92, P150, DOI 10.1016/j.ajhg.2012.11.014
McMurdie PJ, 2013, PLOS ONE, V8, DOI 10.1371/journal.pone.0061217
Nguyen MH, 2012, INT J ONCOL, V41, P1285, DOI 10.3892/ijo.2012.1589
Murillo-Rincon AP, 2017, SCI REP-UK, V7, DOI 10.1038/s41598-017-16191-x
Nan YD, 2010, MED ONCOL, V27, P1398, DOI 10.1007/s12032-009-9393-7
Nazarian A, 2021, GENES-BASEL, V12, DOI 10.3390/genes12050686

Neu AT, 2021, P NATL ACAD SCI USA, V118, DOI 10.1073/pnas.2104429118
Nilsson AC, 2010, BMC INFECT DIS, V10, DOI 10.1186/1471-2334-10-39
Niu LM, 2021, THORAC CANCER, V12, P737, DOI 10.1111/1759-7714.13833
Papagiannakopoulos T, 2016, CELL METAB, V24, P324, DOI 10.1016/j.cmet.2016.07.001
Polineni D, 2018, AM J RESP CRIT CARE, V197, P79, DOI 10.1164/rccm.201701-0134OC
Qian F, 2016, CURR PROTEIN PEPT SC, V17, P332, DOI 10.2174/1389203716666150629121324
Racedo S, 2006, MICROBES INFECT, V8, P2359, DOI 10.1016/j.micinf.2006.04.022
Rausch P, 2017, FRONT MICROBIOL, V8, DOI 10.3389/fmicb.2017.00991
Redford PS, 2010, EUR J IMMUNOL, V40, P2200, DOI 10.1002/eji.201040433
Regalado J, 2020, ISME J, V14, P2116, DOI 10.1038/s41396-020-0665-8
Rello J, 2009, CHEST, V136, P832, DOI 10.1378/chest.09-0258
Ren Z, 2020, EXP THER MED, V20, DOI 10.3892/etm.2020.9296
Richmond BW, 2016, NAT COMMUN, V7, DOI 10.1038/ncomms11240
Risely A, 2021, FRONT MICROBIOL, P1128
Ronkina N, 2007, MOL CELL BIOL, V27, P170, DOI 10.1128/MCB.01456-06
Rydell-Törmänen K, 2019, METHODS MOL BIOL, V1940, P3, DOI 10.1007/978-1-4939-9086-3_1
Rylance J, 2016, BMC MICROBIOL, V16, DOI 10.1186/s12866-016-0803-7
Sabater L, 2008, J NEUROIMMUNOL, V201, P163, DOI 10.1016/j.jneuroim.2008.01.018
Saccone NL, 2010, GENES BRAIN BEHAV, V9, P741, DOI 10.1111/j.1601-183X.2010.00608.x
Salter SJ, 2014, BMC BIOL, V12, DOI 10.1186/s12915-014-0087-z
Schlosser-Brandenburg J, 2021, FRONT IMMUNOL, V12, DOI 10.3389/fimmu.2021.734153
Sibila O, 2019, AM J RESP CRIT CARE, V200, P33, DOI 10.1164/rccm.201809-1651OC
Singh N, 2017, MICROBIOME, V5, DOI 10.1186/s40168-017-0277-3
Song YJ, 2021, EXP CELL RES, V405, DOI 10.1016/j.yexcr.2021.112661
Srinivas G, 2013, NAT COMMUN, V4, DOI 10.1038/ncomms3462
Stämmler F, 2016, MICROBIOME, V4, DOI 10.1186/s40168-016-0175-0
Sui PF, 2018, SCIENCE, V360, P1086, DOI 10.1126/science.aan8546
Sze MA, 2014, PLOS ONE, V9, DOI 10.1371/journal.pone.0110351
Sze MA, 2012, AM J RESP CRIT CARE, V185, P1073, DOI 10.1164/rccm.201111-2075OC
Taylor SC, 2017, SCI REP-UK, V7, DOI 10.1038/s41598-017-02217-x
Taylor SC, 2015, J VIROL METHODS, V224, P58, DOI 10.1016/j.jviromet.2015.08.014
Tkacz A, 2018, MICROBIOME, V6, DOI 10.1186/s40168-018-0491-7
Tomosada Y, 2013, BMC IMMUNOL, V14, DOI 10.1186/1471-2172-14-40
Tsay JCJ, 2021, CANCER DISCOV, V11, P293, DOI 10.1158/2159-8290.CD-20-0263
Turkowski K, 2020, CELLS-BASEL, V9, DOI 10.3390/cells9081755
Turner J, 2002, J IMMUNOL, V169, P6343, DOI 10.4049/jimmunol.169.11.6343
Turpin W, 2016, NAT GENET, V48, P1413, DOI 10.1038/ng.3693
Tzeng A, 2021, GENOME MED, V13, DOI 10.1186/s13073-021-00874-2
Vahl JM, 2017, BRIT J CANCER, V117, P1644, DOI 10.1038/bjc.2017.336
Valette K, 2021, COMMUN BIOL, V4, DOI 10.1038/s42003-021-02227-6
Vandeputte D, 2017, NATURE, V551, P507, DOI 10.1038/nature24460

Villanueva S, 2015, PLOS ONE, V10, DOI 10.1371/journal.pone.0139284
Villena J, 2012, BMC IMMUNOL, V13, DOI 10.1186/1471-2172-13-53
Vogt J, 2008, AM J HUM GENET, V82, P222, DOI 10.1016/j.ajhg.2007.09.016
Vorobyev A, 2019, NAT COMMUN, V10, DOI 10.1038/s41467-019-11952-w
Wang J, 2015, NAT COMMUN, V6, DOI 10.1038/ncomms6957
Wang J, 2016, NAT GENET, V48, P1396, DOI 10.1038/ng.3695
Wang R, 2011, J EXP CLIN CANC RES, V30, DOI 10.1186/1756-9966-30-62
Wei W, 2020, LACTATE DEHYDROGENAS
Wei W, 2021, AGING-US, V13, P17499, DOI 10.18632/aging.203240
Weyrich LS, 2019, MOL ECOL RESOUR, V19, P982, DOI 10.1111/1755-0998.13011
White MJ, 2016, IMMUNOGENETICS, V68, P391, DOI 10.1007/s00251-016-0914-1
Whiteson KL, 2014, AM J RESP CRIT CARE, V189, P1309, DOI 10.1164/rccm.201312-2129PP
Wiel C, 2019, CELL, V178, P330, DOI 10.1016/j.cell.2019.06.005
Wijk SC, 2021, AM J RESP CELL MOL, V65, P103, DOI 10.1165/rcmb.2020-0464OC
Wu YJ, 2021, NPJ BIOFILMS MICROBI, V7, DOI 10.1038/s41522-021-00232-5
Xiang R, 2018, ONCOL REP, V40, P3040, DOI 10.3892/or.2018.6704
Yabumoto Y, 2008, MED MOL MORPHOL, V41, P20, DOI 10.1007/s00795-007-0391-6
Yildiz S, 2020, ELIFE, V9, DOI 10.7554/eLife.53581
Yin WG, 2018, NAT COMMUN, V9, DOI 10.1038/s41467-018-05043-5
Yu GQ, 2016, GENOME BIOL, V17, DOI 10.1186/s13059-016-1021-1
Zheng L., 2021, SCI REP-UK, V11, P1
Zhu ZZ, 2021, EUR RESPIR J, V58, DOI 10.1183/13993003.00199-2021
NR 148
TC 2
Z9 2
U1 1
U2 8
PU BMC
PI LONDON
PA CAMPUS, 4 CRINAN ST, LONDON N1 9XW, ENGLAND
EI 2524-4671
J9 ANIM MICROBIOME
JI Anim. Microbiome
PD JUN 1
PY 2023
VL 5
IS 1
AR 31
DI 10.1186/s42523-023-00250-y
PG 16
WC Microbiology; Veterinary Sciences
WE Emerging Sources Citation Index (ESCI)
SC Microbiology; Veterinary Sciences
GA I1HY8
UT WOS:001000368600001
PM 37264412
OA Green Published, gold
DA 2025-05-07

ER

PT J
AU Nieves-Colon, MA
   Pestle, WJ
   Benn-Torres, J
   Stone, AC
AF Nieves-Colon, Maria A.
   Pestle, William J.
   Benn-Torres, Jada
   Stone, Anne C.
TI Migration, admixture and genetic continuity in pre and post-contact
   Puerto Rico
SO AMERICAN JOURNAL OF PHYSICAL ANTHROPOLOGY
LA English
DT Meeting Abstract
CT 86th Annual Meeting of the
   American-Association-of-Physical-Anthropologists (AAPA)
CY APR 19-22, 2017
CL New Orleans, LA
C1 [Nieves-Colon, Maria A.; Stone, Anne C.] Arizona State Univ, Sch Human
Evolut & Social Change, Tempe, AZ 85287 USA.
   [Pestle, William J.] Univ Miami, Dept Anthropol, Coral Gables, FL
33124 USA.
   [Benn-Torres, Jada] Vanderbilt Univ, Dept Anthropol, 221 Kirkland
Hall, Nashville, TN 37235 USA.
C3 Arizona State University; Arizona State University-Tempe; University
of
   Miami; Vanderbilt University
RI Stone, Anne/B-5719-2009
FU National Science Foundation; Rust Family Foundation; Sigma Xi; ASU
   School of Human Evolution and Social Change Research Committee; ASU
   Center for Evolutionary Medicine
FX Funding provided by: National Science Foundation, Rust Family
   Foundation, Sigma Xi, ASU School of Human Evolution and Social Change
   Research Committee and ASU Center for Evolutionary Medicine.
NR 0
TC 0
Z9 0
U1 0
U2 1
PU WILEY
PI HOBOKEN
PA 111 RIVER ST, HOBOKEN 07030-5774, NJ USA
SN 0002-9483
EI 1096-8644
J9 AM J PHYS ANTHROPOL
JI Am. J. Phys. Anthropol.
PD APR
PY 2017
VL 162
SU 64
BP 300
EP 300

PG 1
WC Anthropology; Evolutionary Biology
WE Science Citation Index Expanded (SCI-EXPANDED); Social Science Citation Index (SSCI); Conference Proceedings Citation Index - Science (CPCI-S)
SC Anthropology; Evolutionary Biology
GA FT3QT
UT WOS:000423063103321
DA 2025-05-07
ER

PT J
AU Fourches, D
Ash, J
AF Fourches, Denis
Ash, Jeremy
TI 4D-quantitative structure-activity relationship modeling: making a comeback
SO EXPERT OPINION ON DRUG DISCOVERY
LA English
DT Review
DE 4D descriptors; cheminformatics; QSAR; molecular dynamics
ID QSAR MODELS; MULTIDIMENSIONAL QSAR; 4D-QSAR ANALYSIS; SIMPLEX REPRESENTATION; FREE-ENERGY; DRUG; INHIBITORS; BINDING; CONSTRUCTION; DOCKING
AB Introduction: Predictive Quantitative Structure-Activity Relationship (QSAR) modeling has become an essential methodology for rapidly assessing various properties of chemicals. The vast majority of these QSAR models utilize numerical descriptors derived from the two- and/or three-dimensional structures of molecules. However, the conformation-dependent characteristics of flexible molecules and their dynamic interactions with biological target(s) is/are not encoded by these descriptors, leading to limited prediction performances and reduced interpretability. 2D/3D QSAR models are successful for virtual screening, but typically suffer at lead optimization stages. That is why conformation-dependent 4D-QSAR modeling methods were developed two decades ago. However, these methods have always suffered from the associated computational cost. Recently, 4D-QSAR has been experiencing a significant come-back due to rapid advances in GPU-accelerated molecular dynamic simulations and modern machine learning techniques. Areas covered: Herein, the authors briefly review the literature regarding 4D-QSAR modeling and describe its modern workflow called MD-QSAR. Challenges and current limitations are also highlighted. Expert opinion: The development of hyper-predictive MD-QSAR models could represent a disruptive technology for analyzing, understanding, and optimizing dynamic protein-ligand interactions with countless applications for drug discovery and chemical toxicity assessment. Therefore, there has never been a better time and relevance for molecular modeling teams to engage in hyper-predictive MD-QSAR modeling.
C1 [Fourches, Denis; Ash, Jeremy] North Carolina State Univ, Dept Chem, Bioinformat Res Ctr, Box 8204, Raleigh, NC 27695 USA.
C3 North Carolina State University
RP Fourches, D (autor correspondente), North Carolina State Univ, Dept Chem, Bioinformat Res Ctr, Box 8204, Raleigh, NC 27695 USA.
EM dfourch@ncsu.edu

RI Fourches, Denis/H-4336-2019; Ash, Jeremy/ABA-5317-2020
OI Fourches, Denis/0000-0001-5642-8303; Ash, Jeremy/0000-0002-8041-8524
FU NC State Chancellor's Faculty Excellence Program; National Institute of
Health bioinformatics training grant [T32ES007329]; Triangle Center of
Evolutionary Medicine; SAS Institute
FX D Fourches is supported and funded by the NC State Chancellor's Faculty
Excellence Program. J Ash is supported by a National Institute of Health
bioinformatics training grant [T32ES007329], the Triangle Center of
Evolutionary Medicine, and the SAS Institute.
CR ALLINGER NL, 1977, J AM CHEM SOC, V99, P8127, DOI 10.1021/ja00467a001
Alves VM, 2015, TOXICOL APPL PHARM, V284, P273, DOI 10.1016/j.taap.2014.12.013
Andrade CH, 2010, MOLECULES, V15, P3281, DOI 10.3390/molecules15053281
Ash J, 2017, J CHEM INF MODEL, V57, P1286, DOI 10.1021/acs.jcim.7b00048
Bak A, 2016, RSC ADV, V6, P76183, DOI 10.1039/c6ra15820j
Bowers KJ, 2006, P ACM IEEE C SUP SC0, DOI [DOI 10.1145/1188455.1188544, 10.1145/1188455.1188544]
Braga RC, 2015, MOL INFORM, V34, P698, DOI 10.1002/minf.201500040
Carlson HA, 2016, J CHEM INF MODEL, V56, P1063, DOI 10.1021/acs.jcim.5b00523
Cherkasov A, 2014, J MED CHEM, V57, P4977, DOI 10.1021/jm4004285
CRAMER RD, 1988, J AM CHEM SOC, V110, P5959, DOI 10.1021/ja00226a005
Damale MG, 2014, MINI-REV MED CHEM, V14, P35, DOI 10.2174/13895575113136660104
Damm-Ganamet KL, 2013, J CHEM INF MODEL, V53, P1853, DOI 10.1021/ci400025f
Daré JK, 2019, CHEM BIOL DRUG DES, V93, P1096, DOI 10.1111/cbdd.13440
Doweyko AM, 2004, J COMPUT AID MOL DES, V18, P587, DOI 10.1007/s10822-004-4068-0
Dreher J, 2018, J CHEM INF MODEL, V58, P165, DOI 10.1021/acs.jcim.7b00419
Fourches D, 2016, J CHEM INF MODEL, V56, P1243, DOI 10.1021/acs.jcim.6b00129
Fourches D, 2015, J CHEM INF MODEL, V55, P63, DOI 10.1021/ci500519w
Fourches D, 2013, J CHEM INF MODEL, V53, P1915, DOI 10.1021/ci400216q
Fourches D, 2010, J CHEM INF MODEL, V50, P1189, DOI 10.1021/ci100176x
Gramatica P., 1997, SAR QSAR Environ. Res., V7, P89, DOI DOI 10.1080/10629369708039126
Hopfinger AJ, 1999, J CHEM INF COMP SCI, V39, P1151, DOI 10.1021/ci990032+
Hopfinger AJ, 1997, J AM CHEM SOC, V119, P10509, DOI 10.1021/ja9718937
Jagiello K, 2016, J NANOPART RES, V18, DOI 10.1007/s11051-016-3564-1
Kuenemann MA, 2018, MOL INFORM, V37, DOI 10.1002/minf.201700138
Kuenemann MA, 2018, MOL INFORM, V37, DOI 10.1002/minf.201800004
Kuz'min VE, 2005, J MOL MODEL, V11, P457, DOI 10.1007/s00894-005-0237-x
Kuz'min VE, 2002, ACTA BIOCHIM POL, V49, P157
Lill MA, 2007, DRUG DISCOV TODAY, V12, P1013, DOI 10.1016/j.drudis.2007.08.004
Low Y, 2011, CHEM RES TOXICOL, V24, P1251, DOI 10.1021/tx200148a

Martin YC, 2009, J COMPUT AID MOL DES, V23, P693, DOI 10.1007/s10822-009-9303-2
Martins JPA, 2009, J CHEM INF MODEL, V49, P1428, DOI 10.1021/ci900014f
Mazanetz MP, 2014, MOLECULES, V19, P9134, DOI 10.3390/molecules19079134
McClendon CL, 2014, P NATL ACAD SCI USA, V111, pE4623, DOI 10.1073/pnas.1418402111
Pan DH, 2004, J CHEM INF COMP SCI, V44, P2083, DOI 10.1021/ci0498057
Polanski J, 2009, CURR MED CHEM, V16, P3243, DOI 10.2174/092986709788803286
Ramírez D, 2018, MOLECULES, V23, DOI 10.3390/molecules23051038
Riniker S, 2017, J CHEM INF MODEL, V57, P726, DOI 10.1021/acs.jcim.6b00778
Romeiro NC, 2005, J COMPUT AID MOL DES, V19, P385, DOI 10.1007/s10822-005-7927-4
Santos OA, 2006, J CHEM INF MODEL, V46, P345, DOI 10.1021/ci050326x
Sedykh A, 2013, PHARM RES-DORDR, V30, P996, DOI 10.1007/s11095-012-0935-x
Sherman W, 2006, J MED CHEM, V49, P534, DOI 10.1021/jm050540c
Sutherland JJ, 2007, J CHEM INF MODEL, V47, P2293, DOI 10.1021/ci700253h
Teófilo RF, 2009, J CHEMOMETR, V23, P32, DOI 10.1002/cem.1192
Tetko IV, 2008, J CHEM INF MODEL, V48, P1733, DOI 10.1021/ci800151m
Todeschini R.C, 2000, Handbook of Molecular Descriptors: Handbook of Molecular Descriptors, DOI 10.1002/9783527613106
Tropsha A, 2010, MOL INFORM, V29, P476, DOI 10.1002/minf.201000061
Tseng YJ, 2012, J COMPUT AID MOL DES, V26, P39, DOI 10.1007/s10822-011-9511-4
Vedani A, 2005, J MED CHEM, V48, P3700, DOI 10.1021/jm050185q
Vedani A, 2002, J MED CHEM, V45, P2139, DOI 10.1021/jm011005p
Vedani A, 1998, J AM CHEM SOC, V120, P4471, DOI 10.1021/ja973976t
Venkatarangan P, 1999, J CHEM INF COMP SCI, V39, P1141, DOI 10.1021/ci9900332
Wieder M, 2017, J CHEM INF MODEL, V57, P365, DOI 10.1021/acs.jcim.6b00674
Yap CW, 2011, J COMPUT CHEM, V32, P1466, DOI 10.1002/jcc.21707
Zakharov AV, 2016, MOL PHARMACEUT, V13, P545, DOI 10.1021/acs.molpharmaceut.5b00762
Zhang LY, 2013, J CHEM INF MODEL, V53, P475, DOI 10.1021/ci300421n
Zhu H, 2008, J CHEM INF MODEL, V48, P766, DOI 10.1021/ci700443v
NR 56
TC 20
Z9 22
U1 1
U2 52
PU TAYLOR & FRANCIS LTD
PI ABINGDON
PA 2-4 PARK SQUARE, MILTON PARK, ABINGDON OR14 4RN, OXON, ENGLAND
SN 1746-0441
EI 1746-045X
J9 EXPERT OPIN DRUG DIS
JI Expert. Opin. Drug Discov.
PD DEC 2
PY 2019

VL 14
IS 12
BP 1227
EP 1235
DI 10.1080/17460441.2019.1664467
EA SEP 2019
PG 9
WC Pharmacology & Pharmacy
WE Science Citation Index Expanded (SCI-EXPANDED)
SC Pharmacology & Pharmacy
GA JJ1GF
UT WOS:000486354700001
PM 31513441
DA 2025-05-07
ER

PT J
AU Diogo, R
AF Diogo, Rui
TI Where is, in 2017, the evo in evo-devo (evolutionary developmental
   biology)?
SO JOURNAL OF EXPERIMENTAL ZOOLOGY PART B-MOLECULAR AND DEVELOPMENTAL
   EVOLUTION
LA English
DT Article
DE developmental biology; evolutionary biology; genetics; genomics;
   genotype; molecular biology; organismal biology; phenotype
ID REGULATORY NETWORKS; PHENOTYPE MAP; GENE; DYNAMICS; DISEASE;
PLEIOTROPY;
   SURVIVAL; ORIGIN; MODEL
AB After the inaugural Pan-American-Evo-Devo meeting (2015, Berkeley), I showed how major concerns about evo-devo (Evolutionary Developmental Biology) research were demonstrated by a simple, non-biased quantitative analysis of the titles/abstracts of that meeting's talks. Here, I apply the same methodology to the titles/abstracts of the recent Pan-American-Evo-Devo meeting (2017, Calgary). The aim is to evaluate if the concerns raised by me in that paper and by other authors have been addressed and/or if there are other types of differences between the two-meetings thatmay reflect trends within the field of evo-devo. This analysis shows that the proportion of presentations referring to "morphology", "organism", "selection", "adaptive", "phylogeny", and their derivatives was higher in the 2017 meeting, which therefore had a more "organismal" feel. However, there was a decrease in the use of "evolution"/its derivatives and of macroevolutionary terms related to the tempo and mode of evolution in the 2017 meeting. Moreover, the disproportionately high use of genetic/genomic terms clearly shows that evo-devo continues to be mainly focused on devo, and particularly on "Geno", that is, on molecular/genetic studies. Furthermore, the vast majority of animal evo-devo studies are focused only on hard tissues, which are just a small fraction of the whole organism-for example, only 15% of the tissue mass of the human body. The lack of an integrative approach is also evidenced by the lack of studies addressing conceptual/longstanding broader questions, including the links between ecology and particularly behavior

and developmental/evolutionary variability and between evo-devo and evolutionary medicine.
C1 [Diogo, Rui] Howard Univ, Dept Anat, Coll Med, Washington, DC 20059 USA.
C3 Howard University
RP Diogo, R (autor correspondente), Howard Univ, Dept Anat, Coll Med, Washington, DC 20059 USA.
EM rui.diogo@howard.edu
CR Alberts B, 2015, MOLECULAR BIOLOGY OF THE CELL, SIXTH EDITION, P1
[Anonymous], 2003, Niche Construction: The Neglected Process in Evolution
[Anonymous], 1993, The disorder of things: Metaphysical foundations of the disunity of science
[Anonymous], 2013, SEARCH MECH DISCOVER, DOI DOI 10.7208/CHICAGO/9780226039824.001.0001
[Anonymous], 1980, The evolutionary synthesis
[Anonymous], 1993, Discovery and Explanation in Biology and Medicine
[Anonymous], 2013, ONTOLOGY MATCHING, DOI DOI 10.1007/978-3-642-38721-0
[Anonymous], 1945, WHAT IS LIFE PHYS AS
Appasani K, 2008, MICRORNAS: FROM BASIC SCIENCE TO DISEASE BIOLOGY, P1
Arendt D, 2016, NAT REV GENET, V17, P744, DOI 10.1038/nrg.2016.127
Berardini TZ, 2010, NUCLEIC ACIDS RES, V38, pD331, DOI 10.1093/nar/gkp1018
Bergstrom C. T., 2016, Evolution, VSecond
Brachman R. J., 2004, Knowledge Representation and Reasoning
Coulon A, 2013, NAT REV GENET, V14, P572, DOI 10.1038/nrg3484
Coyne JA, 2004, Speciation
Daniels BC, 2012, P NATL ACAD SCI USA, V109, P14259, DOI 10.1073/pnas.1203021109
Davidson EH, 2006, SCIENCE, V311, P796, DOI 10.1126/science.1113832
Davidson EH, 2010, J EXP ZOOL PART B, V314B, P182, DOI 10.1002/jez.b.21329
De Brito RA, 2005, EVOLUTION, V59, P2333, DOI 10.1111/j.0014-3820.2005.tb00943.x
desChadarevian S., 2002, DESIGNS LIFE MOL BIO
Djuranovic S, 2011, SCIENCE, V331, P550, DOI 10.1126/science.1191138
Dvorák P, 2015, P NATL ACAD SCI USA, V112, pE2559, DOI 10.1073/pnas.1502924112
Eldredge N, 2005, PALEOBIOLOGY, V31, P133, DOI 10.1666/0094-8373(2005)031[0133:TDOES]2.0.CO;2
Endler J.A., 1986, Monographs in Population Biology, pviii
Erwin DH, 2002, DEVELOPMENT, V129, P3021
Erwin DH, 2009, NAT REV GENET, V10, P141, DOI 10.1038/nrg2499
Falconer D. S., 1989, Introduction to quantitative genetics.
Feil R, 2012, NAT REV GENET, V13, P97, DOI 10.1038/nrg3142
Fisher R.A., 1930, The Genetical Theory of Natural Selection-A Complete Variorum Edition
Fox C, 2006, EVOLUTIONARY GENETIC
Fox JW, 2015, PLOS BIOL, V13, DOI 10.1371/journal.pbio.1002185
Gerstein MB, 2007, GENOME RES, V17, P669, DOI 10.1101/gr.6339607
Gilbert S. F., 2015, Ecological developmental biology
Gligorijevic D, 2016, SCI REP-UK, V6, DOI 10.1038/srep32404

Godfrey-Smith P., 2003, Theory and Reality: An Introduction to the Philosophy of Science
Godfrey-Smith P., 2014, Philosophy of Biology
Gohlmann H., 2009, Gene Expression Studies using Affymetrix Microarrays
Grant P.R., 2010, In search of the causes of evolution: From field observations to mechanisms
Grant P.R., 2014, 40 Years of Evolution: Darwin's Finches on Daphne Major Island
Griffiths AJ, 2012, INTRO GENETIC ANAL
GRUBER TR, 1993, KNOWL ACQUIS, V5, P199, DOI 10.1006/knac.1993.1008
Hofbauer J., 1988, The Theory of Evolution and Dynamical Systems
Hu L., 2010, Computer Design and Applications (ICCDA), 2010 International Conference on, V1, pV1
Hunter A, 2010, KNOWL ENG REV, V25, P199, DOI 10.1017/S0269888910000019
Jarvis JP, 2009, J EVOLUTION BIOL, V22, P1658, DOI 10.1111/j.1420-9101.2009.01776.x
Jia YP, 2015, INT J BIOL SCI, V11, P1413, DOI 10.7150/ijbs.13436
Kawecki TJ, 2012, TRENDS ECOL EVOL, V27, P547, DOI 10.1016/j.tree.2012.06.001
KERR RA, 1994, SCIENCE, V266, P29
Krakauer DC, 2011, J THEOR BIOL, V276, P269, DOI 10.1016/j.jtbi.2011.01.051
Lahiry P, 2010, NAT REV GENET, V11, P60, DOI 10.1038/nrg2707
Laubichler MD, 2015, THEOR BIOSCI, V134, P143, DOI 10.1007/s12064-015-0215-5
Laubichler MD, 2015, J EXP ZOOL PART B, V324, P565, DOI 10.1002/jez.b.22631
Laubichler MD, 2013, CAMBRIDGE ENCYCLOPEDIA OF DARWIN AND EVOLUTIONARY THOUGHT, P375
Laubichler MD, 2000, PHILOS SCI, V67, pS289, DOI 10.1086/392826
Lenski RE, 2003, NATURE, V423, P139, DOI 10.1038/nature01568
Lenski RE, 2001, SCIENCE, V294, P533, DOI 10.1126/science.1066241
Löffler D, 2007, BLOOD, V110, P1330, DOI 10.1182/blood-2007-03-081133
Lynch M., 2007, ORIGINS GENOME ARCHI
Marsit CJ, 2015, J EXP BIOL, V218, P71, DOI 10.1242/jeb.106971
MAYR E, 1961, SCIENCE, V134, P1501, DOI 10.1126/science.134.3489.1501
Mitchell S. D., 2003, Biological Complexity and Integrative Pluralism, DOI 10.1007/s10539-004-5896-y
Mitteroecker P, 2016, GENETICS, V202, P1345, DOI 10.1534/genetics.115.181339
Morange M., 1998, A History of Molecular Biology
Odling-Smee J, 2013, Q REV BIOL, V88, P3, DOI 10.1086/669266
Otero-Cerdeira L, 2015, EXPERT SYST APPL, V42, P949, DOI 10.1016/j.eswa.2014.08.032
Pavlicev M, 2011, J EXP ZOOL PART B, V316B, P371, DOI 10.1002/jez.b.21410
Peter I. S., 2015, Genomic Control Process: Development and Evolution
Porto A, 2016, GENETICS, V204, P1601, DOI 10.1534/genetics.116.189431
Prohaska SJ, 2008, THEOR BIOSCI, V127, P215, DOI 10.1007/s12064-008-0025-0
Rappaport N, 2017, NUCLEIC ACIDS RES, V45, pD877, DOI 10.1093/nar/gkw1012

Sarkar S., 2005, Molecular Models of Life: Philosophical Papers on Molecular Biology
Sarkar S., 1998, Genetics and reductionism
Sayed D, 2011, PHYSIOL REV, V91, P827, DOI 10.1152/physrev.00006.2010
Sengupta B, 2013, PLOS COMPUT BIOL, V9, DOI 10.1371/journal.pcbi.1003157
Singh P, 2011, METHODS MOL BIOL, V733, P267, DOI 10.1007/978-1-61779-089-8_19
Smocovitis VassilikiBetty., 1996, Unifying Biology: The Evolutionary Synthesis and Evolutionary Biology
Spivak DI, 2012, PLOS ONE, V7, DOI 10.1371/journal.pone.0024274
Stadler PF, 2009, THEOR BIOSCI, V128, P165, DOI 10.1007/s12064-009-0067-y
Wagner GP, 1998, GENETICA, V102-3, P569, DOI 10.1023/A:1017088321094
Wagner GP, 2000, AM ZOOL, V40, P819, DOI 10.1668/0003-1569(2000)040[0819:DEAAMS]2.0.CO;2
Wagner GP, 2000, THEOR BIOSCI, V119, P20, DOI 10.1078/1431-7613-00003
Wagner GP, 2011, NAT REV GENET, V12, P204, DOI 10.1038/nrg2949
West G.B., 2017, Scale: the universal laws of life and death in organisms, cities and companies
Wilke CO, 2001, NATURE, V412, P331, DOI 10.1038/35085569
Wimsatt W. C., 2007, Re-engineering philosophy for limited beings: Piecewise approximations to reality
Wiser MJ, 2013, SCIENCE, V342, P1364, DOI 10.1126/science.1243357
WOLPERT DH, 2016, ENTROPY SWITZ, V18
Zhang JZ, 2013, TRENDS GENET, V29, P383, DOI 10.1016/j.tig.2013.05.002
Zimmermann A., 2006, P 4 INT C FORM ONT I, P277
NR 89
TC 5
Z9 8
U1 0
U2 7
PU WILEY
PI HOBOKEN
PA 111 RIVER ST, HOBOKEN 07030-5774, NJ USA
SN 1552-5007
EI 1552-5015
J9 J EXP ZOOL PART B
JI J. Exp. Zool. Part B
PD JAN
PY 2018
VL 330
IS 1
BP 15
EP 22
DI 10.1002/jez.b.22791
PG 8
WC Evolutionary Biology; Developmental Biology; Zoology
WE Science Citation Index Expanded (SCI-EXPANDED)
SC Evolutionary Biology; Developmental Biology; Zoology
GA FY0OL
UT WOS:000426511100002
PM 29393575
DA 2025-05-07

ER

PT J
AU Shattuck, EC
   Perrotte, JK
   Daniels, CL
   Xu, XH
   Sunil, TS
AF Shattuck, Eric C.
   Perrotte, Jessica K.
   Daniels, Colton L.
   Xu, Xiaohe
   Sunil, Thankam S.
TI Signaling sickness: the role of recalled sickness behavior and
   psychosocial factors in shaping communication style
SO EVOLUTION MEDICINE AND PUBLIC HEALTH
LA English
DT Article
DE sickness behavior; signaling; stoicism; health communication;
infectious
   disease
ID GENDER-DIFFERENCES; HEALTH; SYMPTOMS; PERCEPTION; COLLECTIVISM;
   MASCULINITY; EXPLORATION; SUPPORT; WOMEN; PAIN
AB Background and objectives: Active infection results in several outward signs in humans, including visible symptoms, changes in behavior and possible alterations in skin color and gait. A potential adaptive function of these indicators is to signal distress and elicit care from close others. We hypothesized that sickness behavior, a suite of stereotypical changes in mood and behavior, also serves to communicate health status to others. We further hypothesized that such outward signals/cues of health status would vary based on context and sociocultural norms.

Methodology: We explored self-reported, recalled sickness behavior, communication style, demographics and theoretically relevant cultural factors in a large national US sample (n = 1259) using multinomial probit regressions.

Results: In accordance with predictions, relatively few participants were willing to talk or complain about sickness to strangers. Self-reported, recalled sickness behavior was associated with some communication styles but attention received from others was more consistently associated with potential signaling. Several cultural factors, including stoicism and traditional machismo, were also associated with different sickness signaling styles.

Conclusions and implications: These preliminary, self-reported data lend some tentative support to the sickness behavior signaling hypothesis, though experimental or observational support is needed. The role of cultural norms in shaping how such signals are transmitted and received also deserves further attention as they may have important implications for disease transmission.

Lay Summary: Evolutionary medicine hypothesizes that signs and symptoms of infectious disease-including sickness behavior-have adaptive functions, one of which might be to reliably signal one's health status to others. Our results suggest that evolved signals like these are likely shaped by cultural factors.

C1 [Shattuck, Eric C.] Univ Texas San Antonio, Inst Hlth Dispar Res, One UTSA Circle, San Antonio, TX 78249 USA.
[Shattuck, Eric C.] Univ Texas San Antonio, Dept Publ Hlth, One UTSA Circle, San Antonio, TX 78249 USA.
[Perrotte, Jessica K.] Texas State Univ, Dept Psychol, 614 N Guadalupe St 253, San Marcos, TX USA.
[Daniels, Colton L.; Xu, Xiaohe] Univ Texas San Antonio, Dept Sociol, One UTSA Circle, San Antonio, TX 78249 USA.
[Xu, Xiaohe] Sichuan Univ, Sch Publ Adm, Yuzhang S Rd, Chengdu, Sichuan, Peoples R China.
[Sunil, Thankam S.] Univ Tennessee, Dept Publ Hlth, 1914 Andy Holt Ave, Knoxville, TN 37996 USA.
C3 University of Texas System; University of Texas at San Antonio (UTSA); University of Texas System; University of Texas at San Antonio (UTSA); Texas State University System; Texas State University San Marcos; University of Texas System; University of Texas at San Antonio (UTSA); Sichuan University; University of Tennessee System; University of Tennessee Knoxville
RP Shattuck, EC (autor correspondente), Inst Hlth Dispar Res, One UTSA Circle, San Antonio, TX 78249 USA.; Shattuck, EC (autor correspondente), Dept Publ Hlth, One UTSA Circle, San Antonio, TX 78249 USA.
EM eric.shattuck@utsa.edu
RI Sunil, Thankam/AAH-4340-2021; Daniels, Colton/HTR-4558-2023
OI Perrotte, Jessica/0000-0002-4091-7820; Shattuck, Eric/0000-0002-3615-5931
FU Institute for Health Disparities Research at UTSA; Department of Sociology at UTSA
FX This study was funded through the Institute for Health Disparities Research and the Department of Sociology at UTSA.
CR Andreasson A, 2018, J HEALTH PSYCHOL, V23, P1452, DOI 10.1177/1359105316659917
ANNANDALE E, 1990, SOCIOL HEALTH ILL, V12, P24, DOI 10.1111/1467-9566.ep10844865
[Anonymous], 2015, Stata Statistical Software: Release 14
Arciniega GM, 2008, J COUNS PSYCHOL, V55, P19, DOI 10.1037/0022-0167.55.1.19
Armstrong T.L., 2001, Handbook of Cultural Health Psychology, P63, DOI [10.1016/B978012402771-8/50005-2, DOI 10.1016/B978012402771-8/50005-2]
Ballering AV, 2020, SOC SCI MED, V253, DOI 10.1016/j.socscimed.2020.112968
Beauboeuf-Lafontant T, 2007, GENDER SOC, V21, P28, DOI 10.1177/0891243206294108
Berk M, 2012, J EVAL CLIN PRACT, V18, P360, DOI 10.1111/j.1365-2753.2010.01570.x
Carels RA, 2004, J PSYCHOSOM RES, V56, P95, DOI 10.1016/S0022-3999(03)00041-2
Cohn DWH, 2006, PHYSIOL BEHAV, V87, P932, DOI 10.1016/j.physbeh.2006.02.011
Conway LG, 2001, J CROSS CULT PSYCHOL, V32, P681, DOI 10.1177/0022022101032006003
Courtenay WH, 2000, SOC SCI MED, V50, P1385, DOI 10.1016/S0277-9536(99)00390-1
Gaines SO, 1997, J PERS SOC PSYCHOL, V72, P1460, DOI 10.1037/0022-3514.72.6.1460

GEERTSEN HR, 1970, J MARRIAGE FAM, V32, P638, DOI 10.2307/350257
Hammond WP, 2010, AM J COMMUN PSYCHOL, V45, P87, DOI 10.1007/s10464-009-9280-6
Haritatos J, 2007, SOC SCI MED, V64, P1192, DOI 10.1016/j.socscimed.2006.10.037
HART BL, 1988, NEUROSCI BIOBEHAV R, V12, P123, DOI 10.1016/S0149-7634(88)80004-6
Henderson AJ, 2017, BRAIN BEHAV IMMUN, V60, P312, DOI 10.1016/j.bbi.2016.11.008
Hunter JB, 2007, AM J MENS HEALTH, V1, P242, DOI 10.1177/1557988306294163
Hurter S, 2014, J PAIN, V15, P934, DOI 10.1016/j.jpain.2014.06.004
Inagaki TK, 2015, BRAIN BEHAV IMMUN, V44, P247, DOI 10.1016/j.bbi.2014.10.006
Jackson T, 2005, J PAIN, V6, P228, DOI 10.1016/j.jpain.2004.12.004
JAMES SA, 1994, CULT MED PSYCHIAT, V18, P163, DOI 10.1007/BF01379448
Kachel S, 2016, FRONT PSYCHOL, V7, DOI 10.3389/fpsyg.2016.00956
Laidre ME, 2013, CURR BIOL, V23, pR829, DOI 10.1016/j.cub.2013.07.070
Lasselin J, 2018, PSYCHONEUROENDOCRINO, V87, P188, DOI 10.1016/j.psyneuen.2017.10.024
Lindinger-Sternart S., 2014, International Journal of Social Science Studies, V3, P1, DOI [DOI 10.11114/IJSSS.V3I1.519, 10.11114/ijsss.v3i1.519]
Lopes PC, 2014, P ROY SOC B-BIOL SCI, V281, DOI 10.1098/rspb.2014.0218
Lopes PC, 2012, BEHAV ECOL SOCIOBIOL, V66, P1421, DOI 10.1007/s00265-012-1397-1
MacLean A, 2017, SOC SCI MED, V191, P134, DOI 10.1016/j.socscimed.2017.09.015
MacLean A, 2010, SOC SCI MED, V70, P597, DOI 10.1016/j.socscimed.2009.10.042
McCusker RH, 2013, J EXP BIOL, V216, P84, DOI 10.1242/jeb.073411
Miller AH, 2005, CLIN NEUROSCI RES, V4, P325, DOI 10.1016/j.cnr.2005.03.010
Pathak EB, 2017, BMJ OPEN, V7, DOI 10.1136/bmjopen-2016-015137
Roy SC, 2008, SOCIOL HEALTH ILL, V30, P463, DOI 10.1111/j.1467-9566.2007.01066.x
Schaller M, 2011, PHILOS T R SOC B, V366, P3418, DOI 10.1098/rstb.2011.0029
Schaller M, 2010, PSYCHOL SCI, V21, P649, DOI 10.1177/0956797610368064
Shakhar K, 2015, PLOS BIOL, V13, DOI 10.1371/journal.pbio.1002276
Shattuck EC, 2020, FRONT BEHAV NEUROSCI, V14, DOI 10.3389/fnbeh.2020.00004
Shattuck EC, 2015, AM J PHYS ANTHROPOL, V157, P1, DOI 10.1002/ajpa.22698
Silvia P.J., 2001, REV GEN PSYCHOL, V5, P241, DOI [DOI 10.1037/1089-2680.5.3.241, 10.1037/1089-2680.5.3.241]
Sivadas E, 2008, J BUS RES, V61, P201, DOI 10.1016/j.jbusres.2007.06.016
Steinkopf L, 2016, EVOL PSYCHOL-US, V14, DOI 10.1177/1474704916653964
Stockmaier S, 2021, SCIENCE, V371, P1007, DOI 10.1126/science.abc8881
Sundelin T, 2015, BRAIN BEHAV IMMUN, V48, P53, DOI 10.1016/j.bbi.2015.03.007
Tiokhin L, 2016, Q REV BIOL, V91, P177, DOI 10.1086/686811

Uchino BN, 1996, PSYCHOL BULL, V119, P488, DOI 10.1037/0033-2909.119.3.488
Willette AA, 2007, BRAIN BEHAV IMMUN, V21, P807, DOI 10.1016/j.bbi.2007.01.007
WILLIAMS GC, 1991, Q REV BIOL, V66, P1, DOI 10.1086/417048
Zylberberg M, 2013, BIOL LETTERS, V9, DOI 10.1098/rsbl.2012.0856
NR 50
TC 3
Z9 3
U1 1
U2 6
PU OXFORD UNIV PRESS
PI OXFORD
PA GREAT CLARENDON ST, OXFORD OX2 6DP, ENGLAND
EI 2050-6201
J9 EVOL MED PUBLIC HLTH
JI Evol. Med. Public Health
PY 2021
VL 9
IS 1
BP 221
EP 231
DI 10.1093/emph/eoab017
EA AUG 2021
PG 11
WC Evolutionary Biology; Public, Environmental & Occupational Health
WE Science Citation Index Expanded (SCI-EXPANDED); Social Science Citation Index (SSCI)
SC Evolutionary Biology; Public, Environmental & Occupational Health
GA YL3EM
UT WOS:000745777800001
PM 34408880
OA gold, Green Published
DA 2025-05-07
ER

PT S
AU Keller, MC
AF Keller, Matthew C.
BE Widiger, T
Cannon, TD
TI Evolutionary Perspectives on Genetic and Environmental Risk Factors for
Psychiatric Disorders
SO ANNUAL REVIEW OF CLINICAL PSYCHOLOGY, VOL 14
SE Annual Review of Clinical Psychology
LA English
DT Article; Book Chapter
DE evolution; psychiatric disorders; genetics; schizophrenia; depression
ID STRESSFUL LIFE EVENTS; DEPRESSIVE SYMPTOMS; MAJOR DEPRESSION; MISSING HERITABILITY; NATURAL-SELECTION; MENTAL-DISORDER; COMPLEX TRAITS; SCHIZOPHRENIA; MOOD; COMMON
AB Evolutionary medicine uses evolutionary theory to help elucidate why humans are vulnerable to disease and disorders. I discuss two different

types of evolutionary explanations that have been used to help understand human psychiatric disorders. First, a consistent finding is that psychiatric disorders are moderately to highly heritable, and many, such as schizophrenia, are also highly disabling and appear to decrease Darwinian fitness. Models used in evolutionary genetics to understand why genetic variation exists in fitness-related traits can be used to understand why risk alleles for psychiatric disorders persist in the population. The usual explanation for species-typical adaptations-natural selection-is less useful for understanding individual differences in genetic risk to disorders. Rather, two other types of models, mutation-selection-drift and balancing selection, offer frameworks for understanding why genetic variation in risk to psychiatric (and other) disorders exists, and each makes predictions that are now testable using whole-genome data. Second, species-typical capacities to mount reactions to negative events are likely to have been crafted by natural selection to minimize fitness loss. The pain reaction to tissue damage is almost certainly such an example, but it has been argued that the capacity to experience depressive symptoms such as sadness, anhedonia, crying, and fatigue in the face of adverse life situations may have been crafted by natural selection as well. I review the rationale and strength of evidence for this hypothesis. Evolutionary hypotheses of psychiatric disorders are important not only for offering explanations for why psychiatric disorders exist, but also for generating new, testable hypotheses and understanding how best to design studies and analyze data.
C1 [Keller, Matthew C.] Univ Colorado Boulder, Dept Psychol & Neurosci, Boulder, CO 80309 USA.
[Keller, Matthew C.] Univ Colorado Boulder, Inst Behav Genet, Boulder, CO 80309 USA.
C3 University of Colorado System; University of Colorado Boulder; University of Colorado System; University of Colorado Boulder
RP Keller, MC (autor correspondente), Univ Colorado Boulder, Dept Psychol & Neurosci, Boulder, CO 80309 USA.; Keller, MC (autor correspondente), Univ Colorado Boulder, Inst Behav Genet, Boulder, CO 80309 USA.
EM matthew.c.keller@gmail.com
RI Keller, Matthew/B-3033-2010
CR Akiskal H.S., 1997, DYSTHYMIA SPECTRUM C
Andrews PW, 2009, PSYCHOL REV, V116, P620, DOI 10.1037/a0016242
ANGST J, 1992, NEUROL PSYCHIAT BR, V1, P5
[Anonymous], ACTA PSYCHIAT SCAN S
Barrantes-Vidal N, 2004, J CONSCIOUSNESS STUD, V11, P58
Beaussart ML, 2012, J CREATIVE BEHAV, V46, P151, DOI 10.1002/jocb.11
Belsky J, 2014, WORLD PSYCHIATRY, V13, P87, DOI 10.1002/wps.20092
Boyle EA, 2017, CELL, V169, P1177, DOI 10.1016/j.cell.2017.05.038
Breivik H, 2006, EUR J PAIN, V10, P287, DOI 10.1016/j.ejpain.2005.06.009
Bremmer MA, 2008, J AFFECT DISORDERS, V106, P249, DOI 10.1016/j.jad.2007.07.002
Brown G.W., 1986, LIFE EVENTS PSYCHIAT, P107
Brown G.W., 1978, SOCIAL ORIGINS DEPRE
Bubb KL, 2006, GENETICS, V173, P2165, DOI 10.1534/genetics.106.055715
Bulik-Sullivan B, 2015, NAT GENET, V47, P1236, DOI 10.1038/ng.3406
Bulik-Sullivan BK, 2015, NAT GENET, V47, P291, DOI 10.1038/ng.3211
Bundy H, 2011, ACTA PSYCHIAT SCAND, V123, P98, DOI 10.1111/j.1600-0447.2010.01623.x

Cannon TD, 2006, ANNU REV CLIN PSYCHO, V2, P267, DOI 10.1146/annurev.clinpsy.2.022305.095232
Capuron L, 2000, J CLIN ONCOL, V18, P2143, DOI 10.1200/JCO.2000.18.10.2143
Capuron L, 2004, BRAIN BEHAV IMMUN, V18, P205, DOI 10.1016/j.bbi.2003.11.004
Carrera N, 2009, AM J MED GENET B, V150B, P369, DOI 10.1002/ajmg.b.30823
Carver C.S., 2001, Optimism and pessimism: Implications for theory, research, and practice, P31, DOI [10.1037/10385-002, DOI 10.1037/10385-002, https://doi.org/10.1037/10385-002]
CASSANO GB, 1989, PSYCHOPATHOLOGY, V22, P278, DOI 10.1159/000284608
Charlesworth B., 1999, Evolutionary genetics: from molecules to morphology, V1, P369
Charlesworth B, 2015, P NATL ACAD SCI USA, V112, P1662, DOI 10.1073/pnas.1423275112
Chun S, 2009, GENOME RES, V19, P1553, DOI 10.1101/gr.092619.109
Comeron JM, 2008, HEREDITY, V100, P19, DOI 10.1038/sj.hdy.6801059
Cramer AOJ, 2012, PSYCHOL MED, V42, P957, DOI 10.1017/S003329171100211X
Crespi B, 2007, P ROY SOC B-BIOL SCI, V274, P2801, DOI 10.1098/rspb.2007.0876
Crow TJ, 2000, BRAIN RES REV, V31, P118, DOI 10.1016/S0165-0173(99)00029-6
Danziger N, 2009, REV NEUROL-FRANCE, V165, P129, DOI 10.1016/j.neurol.2008.05.003
Farrell MS, 2015, MOL PSYCHIATR, V20, P555, DOI 10.1038/mp.2015.16
Fisher R., 1930, GENETICAL THEORY NAT
Gazal S, 2017, NAT GENET, V49, P1421, DOI 10.1038/ng.3954
Gratten J, 2014, NAT NEUROSCI, V17, P782, DOI 10.1038/nn.3708
Guisinger S, 2003, PSYCHOL REV, V110, P745, DOI 10.1037/0033-295X.110.4.745
Hagen EH, 1999, EVOL HUM BEHAV, V20, P325, DOI 10.1016/S1090-5138(99)00016-1
HAMILTON M, 1967, British Journal of Social and Clinical Psychology, V6, P278
Haukka J, 2003, AM J PSYCHIAT, V160, P460, DOI 10.1176/appi.ajp.160.3.460
Holden C, 1997, HUM BIOL, V69, P605
Horwitz Allan V., 2007, The Loss of Sadness: How Psychiatry Transformed Normal Sorrow into Depressive Disorder
Houle D, 1998, GENETICA, V102-3, P241, DOI 10.1023/A:1017034925212
HOULE D, 1992, GENETICS, V130, P195
Huber CD, 2016, MOL ECOL, V25, P142, DOI 10.1111/mec.13351
Janzen D.H., 1981, P145
Jensen PS, 1997, J AM ACAD CHILD PSY, V36, P1672, DOI 10.1097/00004583-199712000-00015
Johnson EC, 2017, BIOL PSYCHIAT, V82, P702, DOI 10.1016/j.biopsych.2017.06.033
Keller MC, 2007, AM J PSYCHIAT, V164, P1521, DOI 10.1176/appi.ajp.2007.06091564
Keller MC, 2006, J PERS SOC PSYCHOL, V91, P316, DOI 10.1037/0022-3514.91.2.316

Keller MC, 2006, BEHAV BRAIN SCI, V29, P385, DOI 10.1017/S0140525X06009095
Kendler KS, 2011, MOL PSYCHIATR, V16, P626, DOI 10.1038/mp.2011.22
Kendler KS, 2013, AM J PSYCHIAT, V170, P124, DOI 10.1176/appi.ajp.2012.12070995
Kendler KS, 2000, AM J PSYCHIAT, V157, P1243, DOI 10.1176/appi.ajp.157.8.1243
Kendler KS, 2002, AM J PSYCHIAT, V159, P1133, DOI 10.1176/appi.ajp.159.7.1133
Kendler KS, 2003, ARCH GEN PSYCHIAT, V60, P929, DOI 10.1001/archpsyc.60.9.929
Kendler KS, 1998, AM J PSYCHIAT, V155, P172
Kessler RC, 2005, ARCH GEN PSYCHIAT, V62, P593, DOI 10.1001/archpsyc.62.6.593
Kiezun A, 2013, PLOS GENET, V9, DOI 10.1371/journal.pgen.1003301
Kluger MJ, 1996, INFECT DIS CLIN N AM, V10, P1, DOI 10.1016/S0891-5520(05)70282-8
Kyaga S, 2011, BRIT J PSYCHIAT, V199, P373, DOI 10.1192/bjp.bp.110.085316
Lee SH, 2012, NAT GENET, V44, P247, DOI 10.1038/ng.1108
Lee S, 2014, AM J HUM GENET, V95, P5, DOI 10.1016/j.ajhg.2014.06.009
Loh PR, 2015, NAT GENET, V47, P1385, DOI 10.1038/ng.3431
Maes M, 2012, BMC MED, V10, DOI 10.1186/1741-7015-10-66
Maroufizadeh S, 2015, INT J GYNECOL OBSTET, V130, P253, DOI 10.1016/j.ijgo.2015.03.044
Meyers CA, 1999, ADV EXP MED BIOL, V461, P75
Mitchell-Olds T, 2007, NAT REV GENET, V8, P845, DOI 10.1038/nrg2207
MONROE SM, 1991, PSYCHOL BULL, V110, P406, DOI 10.1037/0033-2909.110.3.406
Naumova OY, 2013, CHILD DEV, V84, P76, DOI 10.1111/cdev.12014
Nesse R.M., 1994, WHY WE GET SICK NEW
Nesse RM, 2005, Q REV BIOL, V80, P62, DOI 10.1086/431026
Nesse RM, 2000, ARCH GEN PSYCHIAT, V57, P14, DOI 10.1001/archpsyc.57.1.14
Nesse RM, 1997, SCIENCE, V278, P63, DOI 10.1126/science.278.5335.63
Nettle D, 2012, CURR BIOL, V22, pR712, DOI 10.1016/j.cub.2012.06.020
Nielsen CS, 2008, PAIN, V136, P21, DOI 10.1016/j.pain.2007.06.008
OHTA T, 1973, NATURE, V246, P96, DOI 10.1038/246096a0
Pardinas A F., 2016, bioRxiv
PERRIS H, 1984, J AFFECT DISORDERS, V7, P25, DOI 10.1016/0165-0327(84)90061-2
Piel FB, 2010, NAT COMMUN, V1, DOI 10.1038/ncomms1104
Power RA, 2015, NAT NEUROSCI, V18, P953, DOI 10.1038/nn.4040
PRICE J, 1994, BRIT J PSYCHIAT, V164, P309, DOI 10.1192/bjp.164.3.309
PRICE J, 1967, LANCET, V2, P243
PRICE T, 1991, EVOLUTION, V45, P853, DOI [10.2307/2409693, 10.1111/j.1558-5646.1991.tb04354.x]
Rands CM, 2014, PLOS GENET, V10, DOI 10.1371/journal.pgen.1004525
Ripke S, 2014, NATURE, V511, P421, DOI 10.1038/nature13595
Saha S, 2005, PLOS MED, V2, P413, DOI 10.1371/journal.pmed.0020141
Salmela-Aro K, 2008, J RES PERS, V42, P988, DOI 10.1016/j.jrp.2008.02.007
Sankaranarayanan K, 2001, HEALTH PHYS, V80, P363, DOI 10.1097/00004032-200104000-00013

Sekar A, 2016, NATURE, V530, P177, DOI 10.1038/nature16549
Siewert KM, 2017, MOL BIOL EVOL, V34, P2996, DOI 10.1093/molbev/msx209
Simons YB, 2014, NAT GENET, V46, P220, DOI 10.1038/ng.2896
Srinivasan S, 2016, BIOL PSYCHIAT, V80, P284, DOI 10.1016/j.biopsych.2015.10.009
Stieglitz J, 2015, BRAIN BEHAV IMMUN, V49, P130, DOI 10.1016/j.bbi.2015.05.008
Taub DR, 2016, FRONT PSYCHOL, V7, DOI 10.3389/fpsyg.2016.00857
Tishkoff SA, 2007, NAT GENET, V39, P31, DOI 10.1038/ng1946
TOOBY J, 1990, J PERS, V58, P17, DOI 10.1111/j.1467-6494.1990.tb00907.x
Uher R, 2009, MOL PSYCHIATR, V14, P1072, DOI 10.1038/mp.2009.85
van Dongen J, 2013, AM J MED GENET B, V162B, P122, DOI 10.1002/ajmg.b.32135
van Esch RW, 2013, MED HYPOTHESES, V81, P343, DOI 10.1016/j.mehy.2013.03.042
Vitti JJ, 2013, ANNU REV GENET, V47, P97, DOI 10.1146/annurev-genet-111212-133526
WAKEFIELD JC, 1992, AM PSYCHOL, V47, P373, DOI 10.1037/0003-066X.47.3.373
Wakefield JC, 1999, J ABNORM PSYCHOL, V108, P374, DOI 10.1037/0021-843X.108.3.374
Watson PJ, 2002, J AFFECT DISORDERS, V72, P1, DOI 10.1016/S0165-0327(01)00459-1
WILLIAMS GC, 1991, Q REV BIOL, V66, P1, DOI 10.1086/417048
Wray NR, 2011, PLOS BIOL, V9, DOI 10.1371/journal.pbio.1000579
Wright CE, 2005, BRAIN BEHAV IMMUN, V19, P345, DOI 10.1016/j.bbi.2004.10.003
Wrosch C, 2009, J PERS SOC PSYCHOL, V96, P1181, DOI 10.1037/a0015172
Xu K, 2015, MOL BIOL EVOL, V32, P1148, DOI 10.1093/molbev/msv031
Yang JA, 2010, NAT GENET, V42, P565, DOI 10.1038/ng.608
Yeo RA, 1999, SCHIZOPHR RES, V39, P197, DOI 10.1016/S0920-9964(99)00074-2
Yirmiya R, 2000, ANN NY ACAD SCI, V917, P478
Zietsch BP, 2015, CURR OPIN BEHAV SCI, V2, P73, DOI 10.1016/j.cobeha.2014.09.005
Zuk O, 2014, P NATL ACAD SCI USA, V111, pE455, DOI 10.1073/pnas.1322563111
NR 112
TC 31
Z9 33
U1 1
U2 40
PU ANNUAL REVIEWS
PI PALO ALTO
PA 4139 EL CAMINO WAY, PO BOX 10139, PALO ALTO, CA 94303-0897 USA
SN 1548-5943
J9 ANNU REV CLIN PSYCHO
JI Annu. Rev. Clin. Psychol.
PY 2018
VL 14
BP 471
EP 493
DI 10.1146/annurev-clinpsy-050817084854

PG 23
WC Psychology, Clinical; Psychology
WE Book Citation Index– Social Sciences & Humanities (BKCI-SSH); Book Citation Index– Science (BKCI-S); Science Citation Index Expanded (SCI-EXPANDED); Social Science Citation Index (SSCI)
SC Psychology
GA BK2KR
UT WOS:000433057100018
PM 29401047
DA 2025-05-07
ER

PT J
AU Vinogradov, AE
   Anatskaya, OV
AF Vinogradov, Alexander E.
   Anatskaya, Olga V.
TI Systemic evolutionary changes in mammalian gene expression
SO BIOSYSTEMS
LA English
DT Article
DE Gene expression; Evolution; Longevity; Organismal integrity;
   Translation; Carcinogenesis; Neurological diseases; Cis-regulation;
   Protein interaction networks; Human; Primates; Mouse
ID REGULATORY CHANGES; NONCODING DNA; COMPLEXITY; DISEASE; HUMANS
AB Changes in gene expression play an important role in evolution and can be relevant to evolutionary medicine. In this work, a strong relationship was found between the statistical significance of evolutionary changes in the expression of orthologous genes in the five or six homologous mammalian tissues and the across-tissues unidirectionality of changes (i.e., they occur in the same direction in different tissues - all upward or all downward). In the area of highly significant changes, the fraction of unidirectionally changed genes (UCG) was above 0.9 (random expectation is 0.03). This observation indicates that the most pronounced evolutionary changes in mammalian gene expression are systemic (i.e., they operate at the whole-organism level). The UCG are strongly enriched in the housekeeping genes. More specifically, in the human-chimpanzee comparison, the UCG are enriched in the pathways belonging to gene expression (translation is prominent), cell cycle control, ubiquitin-dependent protein degradation (mostly related to cell cycle control), apoptosis, and Parkinson's disease. In the human-macaque comparison, the two other neurodegenerative diseases (Alzheimer's and Huntington's) are added to the enriched pathways. The consolidation of gene expression changes at the level of pathways indicates that they are not neutral but functional. The systemic expression changes probably maintain the across-tissues balance of basic physiological processes in the course of evolution (e.g., during the movement along the fast-slow life axis). These results can be useful for understanding the variation in longevity and susceptibility to cancer and widespread neurodegenerative diseases. This approach can also guide the choice of prospective genes for studies aiming to decipher cis-regulatory code (the gene list is provided).
C1 [Vinogradov, Alexander E.; Anatskaya, Olga V.] Russian Acad Sci, Inst Cytol, St Petersburg 194064, Russia.
C3 Russian Academy of Sciences; St. Petersburg Scientific Centre of the

Russian Academy of Sciences; Institute of Cytology RAS
RP Anatskaya, OV (autor correspondente), Russian Acad Sci, Inst Cytol, St Petersburg 194064, Russia.
EM aevin@incras.ru
RI Vinogradov, Alexander/R-3923-2016
FU Institute of Cytology (Russian Academy of Sciences)
FX We are very grateful to anonymous referees for valuable comments. This work was supported by the Institute of Cytology (Russian Academy of Sciences).
CR Ahmed J, 2007, GENOME INFORM SER, V18, P22
Akalin A, 2009, GENOME BIOL, V10, DOI 10.1186/gb-2009-10-4-r38
Amemori T, 2015, INT J MOL SCI, V16, P26417, DOI 10.3390/ijms161125961
Bailey J, 2009, ATLA-ALTERN LAB ANIM, V37, P399, DOI 10.1177/026119290903700410
Brawand D, 2011, NATURE, V478, P343, DOI 10.1038/nature10532
Bu XL, 2018, MOL PSYCHIATR, V23, DOI 10.1038/mp.2017.204
Cayuela H, 2020, J ANIM ECOL, V89, P1069, DOI 10.1111/1365-2656.13172
de Magalhaes JP, 2009, J EVOLUTION BIOL, V22, P1770, DOI 10.1111/j.1420-9101.2009.01783.x
Dinman JD, 2016, J MOL BIOL, V428, P2186, DOI 10.1016/j.jmb.2015.12.021
Eisenberg E, 2013, TRENDS GENET, V29, P569, DOI 10.1016/j.tig.2013.05.010
Farajzadeh L, 2013, BIOCHEM BIOPH RES CO, V438, P346, DOI 10.1016/j.bbrc.2013.07.074
Greene CS, 2015, NAT GENET, V47, P569, DOI 10.1038/ng.3259
Hall LS, 2021, MOL PSYCHIATR, V26, P2082, DOI 10.1038/s41380-020-0743-3
Hatton IA, 2019, P NATL ACAD SCI USA, V116, P21616, DOI 10.1073/pnas.1900492116
Ito N, 2018, DEV GROWTH DIFFER, V60, P241, DOI 10.1111/dgd.12538
Jia LF, 2020, NAT STRUCT MOL BIOL, V27, P814, DOI 10.1038/s41594-020-0465-x
Jia Q, 2010, SCI CHINA LIFE SCI, V53, P94, DOI 10.1007/s11427-010-0003-x
Jung M, 2015, NUCLEIC ACIDS RES, V43, pE100, DOI 10.1093/nar/gkv473
Kalyanaraman B, 2020, REDOX BIOL, V36, DOI 10.1016/j.redox.2020.101665
Keller SH, 2020, P NATL ACAD SCI USA, V117, P15096, DOI 10.1073/pnas.1917040117
Khuu P, 2007, P NATL ACAD SCI USA, V104, P16528, DOI 10.1073/pnas.0707203104
Kindler S, 2005, ANNU REV CELL DEV BI, V21, P223, DOI 10.1146/annurev.cellbio.21.122303.120653
Kriventseva EV, 2019, NUCLEIC ACIDS RES, V47, pD807, DOI 10.1093/nar/gky1053
Kyriazis M, 2020, J MOL EVOL, V88, P57, DOI 10.1007/s00239-019-09896-2
Loehlin DW, 2019, P NATL ACAD SCI USA, V116, P12383, DOI 10.1073/pnas.1904071116
Loehlin DW, 2012, SCIENCE, V335, P943, DOI 10.1126/science.1215193
Martin A, 2013, EVOLUTION, V67, P1235, DOI 10.1111/evo.12081
Metzger BPH, 2017, GENOME BIOL EVOL, V9, P843, DOI 10.1093/gbe/evx035
Nagy G, 2020, COMPUT STRUCT BIOTEC, V18, P2026, DOI 10.1016/j.csbj.2020.07.007
Nielsen R, 2005, PLOS BIOL, V3, P976, DOI 10.1371/journal.pbio.0030170

Nourmohammad A, 2017, CELL REP, V20, P1385, DOI 10.1016/j.celrep.2017.07.033
Novikova DD, 2020, GENES-BASEL, V11, DOI 10.3390/genes11060634
Preger-Ben Noon E, 2018, CELL REP, V22, P3021, DOI 10.1016/j.celrep.2018.02.073
Ritchie ME, 2015, NUCLEIC ACIDS RES, V43, DOI 10.1093/nar/gkv007
Sayers EW, 2020, NUCLEIC ACIDS RES, V48, pD9, DOI [10.1093/nar/gkz899, 10.1093/nar/gkaa892, 10.1093/nar/gkv1290]
Scholefield M, 2020, AGEING RES REV, V63, DOI 10.1016/j.arr.2020.101152
Signor SA, 2018, TRENDS GENET, V34, P532, DOI 10.1016/j.tig.2018.03.007
Slobodin B, 2020, EMBO REP, V21, DOI 10.15252/embr.202050799
Stearns SC, 2020, J MOL EVOL, V88, P3, DOI 10.1007/s00239-019-09893-5
Storey JD, 2003, P NATL ACAD SCI USA, V100, P9440, DOI 10.1073/pnas.1530509100
Su G, 2010, BIOINFORMATICS, V26, P3135, DOI 10.1093/bioinformatics/btq596
Szklarczyk D, 2019, NUCLEIC ACIDS RES, V47, pD607, DOI [10.1093/nar/gky1131, 10.1093/nar/gkac1000]
Vazquez-Martin A, 2016, ONCOTARGET, V7, P75235, DOI 10.18632/oncotarget.12118
Vinogradov AE, 2017, MAMM GENOME, V28, P455, DOI 10.1007/s00335-017-9713-8
Vinogradov AE, 2005, NUCLEIC ACIDS RES, V33, P559, DOI 10.1093/nar/gki184
Vinogradov AE, 2001, MOL BIOL EVOL, V18, P2195, DOI 10.1093/oxfordjournals.molbev.a003766
Vinogradov AE, 2007, NUCLEIC ACIDS RES, V35, P6350, DOI 10.1093/nar/gkm723
Vinogradov AE, 2006, NUCLEIC ACIDS RES, V34, P5906, DOI 10.1093/nar/gkl773
Vinogradov AE, 2020, FEBS J, V287, P4427, DOI 10.1111/febs.15257
Vinogradov AE, 2015, MAMM GENOME, V26, P609, DOI 10.1007/s00335-015-9605-8
Weis E, 2008, CYTOGENET GENOME RES, V122, P92, DOI 10.1159/000163086
Wong ES, 2017, NAT COMMUN, V8, DOI 10.1038/s41467-017-01037-x
Wright J, 2020, ECOL EVOL, V10, P3068, DOI 10.1002/ece3.6122
Xin YQ, 2020, P NATL ACAD SCI USA, V117, P20636, DOI 10.1073/pnas.2004003117
Yates AD, 2020, NUCLEIC ACIDS RES, V48, pD682, DOI 10.1093/nar/gkz966
Zhou X, 2015, J MOL CELL BIOL, V7, P92, DOI 10.1093/jmcb/mjv014
NR 56
TC 6
Z9 6
U1 0
U2 5
PU ELSEVIER SCI LTD
PI London
PA 125 London Wall, London, ENGLAND
SN 0303-2647
EI 1872-8324
J9 BIOSYSTEMS
JI Biosystems

PD DEC
PY 2020
VL 198
AR 104256
DI 10.1016/j.biosystems.2020.104256
PG 9
WC Biology; Mathematical & Computational Biology
WE Science Citation Index Expanded (SCI-EXPANDED)
SC Life Sciences & Biomedicine - Other Topics; Mathematical &
Computational
   Biology
GA PA9LC
UT WOS:000595948200006
PM 32976926
DA 2025-05-07
ER

PT J
AU Watve, M
   Diwekar-Joshi, M
AF Watve, Milind
   Diwekar-Joshi, Manawa
TI What to expect from an evolutionary hypothesis for a human disease:
The
   case of type 2 diabetes
SO HOMO-JOURNAL OF COMPARATIVE HUMAN BIOLOGY
LA English
DT Article
ID GENOME-WIDE ASSOCIATION; PREDICTIVE ADAPTIVE RESPONSE; THRIFTY
PHENOTYPE
   HYPOTHESIS; POLYCYSTIC-OVARY-SYNDROME; BETA-CELL FUNCTION;
   LOW-BIRTH-WEIGHT; INSULIN-RESISTANCE; CARDIOVASCULAR-DISEASE;
METABOLIC
   SYNDROME; SERUM-CHOLESTEROL
AB Evolutionary medicine has a promise to bring in a conceptual
revolution in medicine. However, as yet the field does not have the same
theoretical rigour as that of many other fields in evolutionary studies.
We discuss here with reference to type 2 diabetes mellitus (T2DM) what
role an evolutionary hypothesis should play in the development of
thinking in medicine. Starting with the thrifty gene hypothesis,
evolutionary thinking in T2DM has undergone several transitions,
modifications and refinements of the thrift family of hypotheses. In
addition alternative hypotheses independent of thrift are also suggested.
However, most hypotheses look at partial pictures; make selective use of
supportive data ignoring inconvenient truths. Most hypotheses look at a
superficial picture and avoid getting into the intricacies of underlying
molecular, neuronal and physiological processes. Very few hypotheses have
suggested clinical implications and none of them have been tested with
randomized clinical trials. In the meanwhile the concepts in the
pathophysiology of T2DM are undergoing radical changes and evolutionary
hypotheses need to take them into account. We suggest an approach and a
set of criteria to evaluate the relative merits of the alternative
hypotheses. A number of hypotheses are likely to fail when critically
evaluated against these criteria. It is possible that more than one

selective process are at work in the evolution of propensity to T2DM, but the intercompatibility of the alternative selective forces and their relative contribution needs to be examined. The approach we describe could potentially lead to a sound evolutionary theory that is clinically useful and testable by randomized controlled clinical trials. (C) 2016 Elsevier GmbH. All rights reserved.
C1 [Watve, Milind; Diwekar-Joshi, Manawa] Indian Inst Sci Educ & Res, Dr Homi Bhabha Rd, Pune 411008, Maharashtra, India.
C3 Indian Institute of Science Education & Research (IISER) Pune
RP Watve, M (autor correspondente), Indian Inst Sci Educ & Res, Dr Homi Bhabha Rd, Pune 411008, Maharashtra, India.
EM milind@iiserpune.ac.in
CR Alemzadeh R, 2002, EUR J ENDOCRINOL, V146, P871, DOI 10.1530/eje.0.1460871
Alemzadeh R, 2008, METABOLISM, V57, P1597, DOI 10.1016/j.metabol.2008.06.017
[Anonymous], 1932, The Wisdom of the Body
Baig U, 2011, J OBES, V2011, DOI 10.1155/2011/861049
Bajaj M, 2007, DIABETOLOGIA, V50, P1723, DOI 10.1007/s00125-007-0698-9
Bamford NJ, 2016, EQUINE VET J, V48, P368, DOI 10.1111/evj.12434
Barker DJP, 1998, CLIN SCI, V95, P115, DOI 10.1042/CS19980019
Bateson P, 2014, J PHYSIOL-LONDON, V592, P2357, DOI 10.1113/jphysiol.2014.271460
Belsare PV, 2010, MED HYPOTHESES, V74, P578, DOI 10.1016/j.mehy.2009.09.014
Benyshek DC, 2006, AM J PHYS ANTHROPOL, V131, P120, DOI 10.1002/ajpa.20334
Blagosklonny MV, 2013, CELL DEATH DIS, V4, DOI 10.1038/cddis.2013.506
Blagosklonny MV, 2009, AGING-US, V1, P357, DOI 10.18632/aging.100040
Bogin B, 2007, AM J HUM BIOL, V19, P631, DOI 10.1002/ajhb.20666
Boyko EJ, 2000, DIABETES CARE, V23, P1260, DOI 10.2337/diacare.23.9.1260
Butler AE, 2003, DIABETES, V52, P102, DOI 10.2337/diabetes.52.1.102
Carey M, 2013, J BIOL CHEM, V288, P34981, DOI 10.1074/jbc.R113.506782
Carnethon MR, 2003, DIABETES CARE, V26, P3035, DOI 10.2337/diacare.26.11.3035
Chakravarthy MV, 2008, DIABETES, V57, P2698, DOI 10.2337/db08-0404
CLARK A, 1990, DIABETOLOGIA, V33, P285, DOI 10.1007/BF00403322
Corbett S, 2013, MOL CELL ENDOCRINOL, V373, P39, DOI 10.1016/j.mce.2013.01.001
Corbett SJ, 2009, AM J HUM BIOL, V21, P587, DOI 10.1002/ajhb.20937
Cournoyea M., 2010, ANCESTRAL ASSUMPTION
DAVIES DP, 1991, BRIT MED J, V303, P1474, DOI 10.1136/bmj.303.6815.1474-b
DEWSBURY DA, 1990, ANIM BEHAV, V39, P284, DOI 10.1016/S0003-3472(05)80872-3
Drake AJ, 2004, J ENDOCRINOL, V180, P1, DOI 10.1677/joe.0.1800001
DUBUC PU, 1981, J NUTR, V111, P1742, DOI 10.1093/jn/111.10.1742
DUBUC PU, 1976, METABOLISM, V25, P1567, DOI 10.1016/0026-0495(76)90109-8
East ML, 2009, BEHAV ECOL, V20, P478, DOI 10.1093/beheco/arp020
Fernández-Real JM, 1999, DIABETOLOGIA, V42, P1367, DOI 10.1007/s001250051451

Fernández-Real JM, 2008, TRENDS ENDOCRIN MET, V19, P10, DOI 10.1016/j.tem.2007.10.004
Franckhauser S, 2002, DIABETES, V51, P624, DOI 10.2337/diabetes.51.3.624
FRISCH RE, 1987, HUM REPROD, V2, P521, DOI 10.1093/oxfordjournals.humrep.a136582
GARVEY WT, 1986, DIABETES, V35, P258, DOI 10.2337/diabetes.35.3.258
Gluckman PD, 2005, TRENDS ECOL EVOL, V20, P527, DOI 10.1016/j.tree.2005.08.001
Golomb BA, 2000, J PSYCHIATR RES, V34, P301, DOI 10.1016/S0022-3956(00)00024-8
Guglielmo CG, 2010, INTEGR COMP BIOL, V50, P336, DOI 10.1093/icb/icq097
HALES CN, 1992, DIABETOLOGIA, V35, P595, DOI 10.1093/ije/dyt133
HALES CN, 1991, BMJ-BRIT MED J, V303, P1019, DOI 10.1136/bmj.303.6809.1019
Hales CN, 2001, BRIT MED BULL, V60, P5, DOI 10.1093/bmb/60.1.5
Hardikar AA, 2015, CELL METAB, V22, P312, DOI 10.1016/j.cmet.2015.06.008
Hayward AD, 2013, P NATL ACAD SCI USA, V110, P13886, DOI 10.1073/pnas.1301817110
Hillbrand M, 2005, J BEHAV MED, V28, P295, DOI 10.1007/s10865-005-4665-y
HOLEKAMP KE, 1991, AM ZOOL, V31, P306
Holman RR, 2008, NEW ENGL J MED, V359, P1577, DOI 10.1056/NEJMoa0806470
JOHNSON JA, 1987, ANIM BEHAV, V35, P1694, DOI 10.1016/S0003-3472(87)80062-3
Kadowaki T, 2000, J CLIN INVEST, V106, P459, DOI 10.1172/JCI10830
Kaletsky R, 2010, DIS MODEL MECH, V3, P415, DOI 10.1242/dmm.001040
Kaufman JM, 2005, ENDOCR REV, V26, P833, DOI 10.1210/er.2004-0013
Kilpeläinen TO, 2011, NAT GENET, V43, P753, DOI 10.1038/ng.866
Kim JK, 2000, J CLIN INVEST, V105, P1791, DOI 10.1172/JCI8305
Kulkarni S., 2016, J THEOR BIO IN PRESS
Lacherade JC, 2007, INTENS CARE MED, V33, P814, DOI 10.1007/s00134-007-0543-0
Law DCG, 2007, HUM REPROD, V22, P414, DOI 10.1093/humrep/de1400
Lea AJ, 2015, AM NAT, V185, P809, DOI 10.1086/681016
Leeson CPM, 2001, CIRCULATION, V103, P1264
Li SX, 2010, AM J CLIN NUTR, V91, P184, DOI 10.3945/ajcn.2009.28403
Lord LD, 2009, HORM BEHAV, V56, P519, DOI 10.1016/j.yhbeh.2009.09.002
Lustig RH, 2004, INT J OBESITY, V28, P1344, DOI 10.1038/sj.ijo.0802753
Lustig RH, 2003, J CLIN ENDOCR METAB, V88, P2586, DOI 10.1210/jc.2002-030003
Lustig RH, 2006, NAT CLIN PRACT ENDOC, V2, P447, DOI 10.1038/ncpendmet0220
de Rezende LFM, 2014, PLOS ONE, V9, DOI 10.1371/journal.pone.0105620
Maianti JP, 2014, NATURE, V511, P94, DOI 10.1038/nature13297
MATTHEWS DR, 1985, DIABETOLOGIA, V28, P412, DOI 10.1007/BF00280883
MCCANCE DR, 1994, BRIT MED J, V308, P942, DOI 10.1136/bmj.308.6934.942
McCarthy MI, 2009, CURR DIABETES REP, V9, P164, DOI 10.1007/s11892-009-0027-4
McWilliams SR, 2004, J AVIAN BIOL, V35, P377, DOI 10.1111/j.0908-8857.2004.03378.x

Meigs JB, 2004, JAMA-J AM MED ASSOC, V291, P1978, DOI 10.1001/jama.291.16.1978
Michael MD, 2000, MOL CELL, V6, P87, DOI 10.1016/S1097-2765(00)00010-1
Miller AW, 1999, J CARDIOVASC PHARM, V33, P698, DOI 10.1097/00005344-199905000-00004
Montori V.M., 2008, EVID BASED MED, V13, P169
Montori VM, 2009, ANN INTERN MED, V150, P803, DOI 10.7326/0003-4819-150-11-200906020-00008
Moustaid N, 1996, J NUTR, V126, P865, DOI 10.1093/jn/126.4.865
Mutch DM, 2006, PLOS GENET, V2, P1956, DOI 10.1371/journal.pgen.0020188
Nair L, 2009, INDIAN PEDIATR, V46, pS48
NEEL JV, 1962, AM J HUM GENET, V14, P353
Nesse RM, 2001, WESTERN J MED, V174, P358, DOI 10.1136/ewjm.174.5.358
Nettle D, 2015, P ROY SOC B-BIOL SCI, V282, P23, DOI 10.1098/rspb.2015.1005
Nettle D, 2013, P ROY SOC B-BIOL SCI, V280, DOI 10.1098/rspb.2013.1343
Nguyen RHN, 2007, HUM REPROD, V22, P2488, DOI 10.1093/humrep/dem139
ODEA K, 1991, MED J AUSTRALIA, V155, P258, DOI 10.5694/j.1326-5377.1991.tb142236.x
Pardini VC, 1998, J CLIN ENDOCR METAB, V83, P503, DOI 10.1210/jc.83.2.503
Pasarica M, 2006, OBESITY, V14, P1905, DOI 10.1038/oby.2006.222
PASQUET P, 1992, AM J CLIN NUTR, V56, P483, DOI 10.1093/ajcn/56.3.483
Pickup J.C., 2002, TXB DIABETES
Polotsky AJ, 2010, FERTIL STERIL, V93, P2004, DOI 10.1016/j.fertnstert.2008.12.059
Pond CM., 1998, The fats of life
Porte D, 2001, DIABETES, V50, pS160, DOI 10.2337/diabetes.50.2007.S160
Prentice AM, 2008, INT J OBESITY, V32, P1607, DOI 10.1038/ijo.2008.147
Ramlau-Hansen CH, 2007, HUM REPROD, V22, P1634, DOI 10.1093/humrep/dem035
Rankinen T, 2006, OBESITY, V14, P529, DOI 10.1038/oby.2006.71
RATZMANN KP, 1983, INT J OBESITY, V7, P453
Read AF, 2009, EVOL APPL, V2, P40, DOI 10.1111/j.1752-4571.2008.00066.x
Read Andrew F., 1994, Trends in Microbiology, V2, P73, DOI 10.1016/0966-842X(94)90537-1
Reinhardt M, 2015, DIABETES, V64, P2859, DOI 10.2337/db14-1881
Ribeiro RT, 2008, J GERONTOL A-BIOL, V63, P560, DOI 10.1093/gerona/63.6.560
Rickard IJ, 2007, TRENDS ENDOCRIN MET, V18, P94, DOI 10.1016/j.tem.2007.02.004
Roberson LL, 2014, BMC PUBLIC HEALTH, V14, DOI 10.1186/1471-2458-14-14
Rosati AG, 2013, PLOS ONE, V8, DOI 10.1371/journal.pone.0063058
Rosati AG, 2012, ANIM BEHAV, V84, P869, DOI 10.1016/j.anbehav.2012.07.010
Sahlins Marshall., 1974, STONE AGE EC
Sallmén M, 2006, EPIDEMIOLOGY, V17, P520, DOI 10.1097/01.ede.0000229953.76862.e5
Schreuder T, 2005, DIABETES OBES METAB, V7, P239, DOI 10.1111/j.1463-1326.2004.00449.x
Schteingart D E, 1979, Adv Exp Med Biol, V119, P125
Schwartz MW, 2005, SCIENCE, V307, P375, DOI 10.1126/science.1104344

Scott LJ, 2007, SCIENCE, V316, P1341, DOI 10.1126/science.1142382
Shanik MH, 2008, DIABETES CARE, V31, pS262, DOI 10.2337/dc08-s264
Shaughnessy AF, 2003, BRIT MED J, V327, P266, DOI 10.1136/bmj.327.7409.266
Sladek R, 2007, NATURE, V445, P881, DOI 10.1038/nature05616
Speakman JR, 2008, INT J OBESITY, V32, P1611, DOI 10.1038/ijo.2008.161
Stipp D, 2011, AGING-US, V3, P85, DOI 10.18632/aging.100286
Terauchi Y, 2002, ENDOCR J, V49, P247, DOI 10.1507/endocrj.49.247
TEUSCHER T, 1987, LANCET, V1, P765
Thorleifsson G, 2009, NAT GENET, V41, P18, DOI 10.1038/ng.274
Turner N, 2007, DIABETES, V56, P2085, DOI 10.2337/db07-0093
Turner RC, 1998, LANCET, V352, P837, DOI 10.1016/s0140-6736(98)07019-6
TURNER RC, 1979, METABOLISM, V28, P1086, DOI 10.1016/0026-0495(79)90146-X
Vaag AA, 2012, DIABETOLOGIA, V55, P2085, DOI 10.1007/s00125-012-2589-y
Velasquez-Mieyer PA, 2003, INT J OBESITY, V27, P219, DOI 10.1038/sj.ijo.802227
Venn-Watson S, 2014, FRONT ENDOCRINOL, V5, DOI 10.3389/fendo.2014.00227
Virgin CE, 1997, AM J PRIMATOL, V42, P25, DOI 10.1002/(SICI)1098-2345(1997)42:1<25::AID-AJP2>3.0.CO;2-0
Watve M, 2013, DOVES DIPLOMATS DIAB
Watve M, 2014, MED HYPOTHESES, V82, P49, DOI 10.1016/j.mehy.2013.11.002
Weber JM, 2009, J EXP BIOL, V212, P593, DOI 10.1242/jeb.015024
Wells JCK, 2003, J THEOR BIOL, V221, P143, DOI 10.1006/jtbi.2003.3183
Wells JCK, 2007, TRENDS ENDOCRIN MET, V18, P331, DOI 10.1016/j.tem.2007.07.006
Wells JCK, 2007, BIOL REV, V82, P143, DOI 10.1111/j.1469-185X.2006.00007.x
Wells JCK, 2012, INT J EPIDEMIOL, V41, P229, DOI 10.1093/ije/dyr239
WILLIAMS GC, 1991, Q REV BIOL, V66, P1, DOI 10.1086/417048
Wong TY, 2006, BRIT J OPHTHALMOL, V90, P301, DOI 10.1136/bjo.2005.084400
Yilmaz N, 2009, J WOMENS HEALTH, V18, P633, DOI 10.1089/jwh.2008.1057
Zhang J, 2005, AM J EPIDEMIOL, V161, P691, DOI 10.1093/aje/kwi074
Zoncu R, 2011, NAT REV MOL CELL BIO, V12, P21, DOI 10.1038/nrm3025
NR 132
TC 6
Z9 6
U1 1
U2 23
PU ELSEVIER GMBH
PI MUNICH
PA HACKERBRUCKE 6, 80335 MUNICH, GERMANY
SN 0018-442X
EI 1618-1301
J9 HOMO
JI Homo
PY 2016
VL 67
IS 5
BP 349
EP 368

DI 10.1016/j.jchb.2016.07.001
PG 20
WC Anthropology
WE Social Science Citation Index (SSCI)
SC Anthropology
GA EB2OG
UT WOS:000387201100001
PM 27650853
DA 2025-05-07
ER

PT J
AU Vitikainen, EIK
   Thompson, FJ
   Marshall, HH
   Cant, MA
AF Vitikainen, Emma I. K.
   Thompson, Faye J.
   Marshall, Harry H.
   Cant, Michael A.
TI Live long and prosper: durable benefits of early-life care in banded
   mongooses
SO PHILOSOPHICAL TRANSACTIONS OF THE ROYAL SOCIETY B-BIOLOGICAL SCIENCES
LA English
DT Article
DE early-life effects; cooperative breeding; inclusive fitness; lifetime
   reproductive success; selective disappearance; social evolution
ID FEMALE REPRODUCTIVE COMPETITION; HELPERS INCREASE; SOCIAL-CONTROL;
   MATERNAL-CARE; EVOLUTION; EVICTION; BEHAVIOR; DEMOGRAPHY; MENOPAUSE;
   PARENTAGE
AB Kin selection theory defines the conditions for which altruism or 'helping' can be favoured by natural selection. Tests of this theory in cooperatively breeding animals have focused on the short-term benefits to the recipients of help, such as improved growth or survival to adulthood. However, research on early-life effects suggests that there may be more durable, lifelong fitness impacts to the recipients of help, which in theory should strengthen selection for helping. Here, we show in cooperatively breeding banded mongooses (Mungos mungo) that care received in the first 3 months of life has lifelong fitness benefits for both male and female recipients. In this species, adult helpers called 'escorts' form exclusive one-to-one caring relationships with specific pups (not their own offspring), allowing us to isolate the effects of being escorted on later reproduction and survival. Pups that were more closely escorted were heavier at sexual maturity, which was associated with higher lifetime reproductive success for both sexes. Moreover, for female offspring, lifetime reproductive success increased with the level of escorting received per se, over and above any effect on body mass. Our results suggest that early-life social care has durable benefits to offspring of both sexes in this species. Given the well-established developmental effects of early-life care in laboratory animals and humans, we suggest that similar effects are likely to be widespread in social animals more generally. We discuss some of the implications of durable fitness benefits for the evolution of intergenerational helping in cooperative animal societies, including humans.

This article is part of the theme issue 'Developing differences: early-life effects and evolutionary medicine'.
C1 [Vitikainen, Emma I. K.; Thompson, Faye J.; Marshall, Harry H.; Cant, Michael A.] Univ Exeter, Ctr Ecol & Conservat, Penryn Campus, Penryn, England.
[Vitikainen, Emma I. K.] Univ Helsinki, Fac Biol & Environm Sci, Organismal & Evolutionary Biol Res Programme, Helsinki, Finland.
[Marshall, Harry H.] Univ Roehampton, Dept Life Sci, London, England.
C3 University of Exeter; University of Helsinki; Roehampton University
RP Vitikainen, EIK; Cant, MA (autor correspondente), Univ Exeter, Ctr Ecol & Conservat, Penryn Campus, Penryn, England.; Vitikainen, EIK (autor correspondente), Univ Helsinki, Fac Biol & Environm Sci, Organismal & Evolutionary Biol Res Programme, Helsinki, Finland.
EM emma.vitikainen@helsinki.fi; m.a.cant@exeter.ac.uk
RI ; Vitikainen, Emma/F-2099-2010; Cant, Michael/B-1982-2009; Marshall, Harry/D-4945-2014
OI Thompson, Faye/0000-0001-7581-2204; Vitikainen, Emma/0000-0003-3718-0941; Cant, Michael/0000-0002-1530-3077; Marshall, Harry/0000-0003-2120-243X
FU European Research Council Starting Grant (SOCODEV) [309249]; Natural Environment Research Council (UK) [NE/J010278/1, NE/E015441/1, NE/N011171]; European Research Council (ERC) [309249] Funding Source: European Research Council (ERC); NERC [NE/J010278/1, NE/E015441/1] Funding Source: UKRI
FX This work was funded by a European Research Council Starting Grant (SOCODEV, grant number 309249) and Natural Environment Research Council (UK) Standard Grants (NE/J010278/1; NE/E015441/1; NE/N011171) awarded to M.C. The funders had no role in study design, data collection and analysis, decision to publish or preparation of the manuscript.
CR [Anonymous], E AFR WILDLIFE J
Bates D, 2015, J STAT SOFTW, V67, P1, DOI 10.18637/jss.v067.i01
Black MM, 2017, LANCET, V389, P77, DOI 10.1016/S0140-6736(16)31389-7
Blurton-Jones N., 2016, Deomgraphy and Evolutionary Ecology of Hadza Hunter-Gatherers
Brent LJN, 2015, CURR BIOL, V25, P746, DOI 10.1016/j.cub.2015.01.037
Burkart JM, 2009, EVOL ANTHROPOL, V18, P175, DOI 10.1002/evan.20222
Cameron NM, 2008, J NEUROENDOCRINOL, V20, P795, DOI 10.1111/j.1365-2826.2008.01725.x
Cameron NM, 2008, HORM BEHAV, V54, P178, DOI 10.1016/j.yhbeh.2008.02.013
Cant MA, 2000, ANIM BEHAV, V59, P147, DOI 10.1006/anbe.1999.1279
Cant MA, 2008, P NATL ACAD SCI USA, V105, P5332, DOI 10.1073/pnas.0711911105
Cant Michael A., 2016, P318
Cant MA, 2013, ADV STUD BEHAV, V45, P407, DOI 10.1016/B978-0-12-407186-5.00006-9
Cant MA, 2011, P ROY SOC B-BIOL SCI, V278, P170, DOI 10.1098/rspb.2010.1241
Cant MA, 2010, P ROY SOC B-BIOL SCI, V277, P2219, DOI 10.1098/rspb.2009.2097
Champagne FA, 2003, PHYSIOL BEHAV, V79, P359, DOI 10.1016/S0031-9384(03)00149-5

Choi JK, 2007, SCIENCE, V318, P636, DOI 10.1126/science.1144237
Croft DP, 2015, TRENDS ECOL EVOL, V30, P407, DOI 10.1016/j.tree.2015.04.011
Engqvist L, 2005, ANIM BEHAV, V70, P967, DOI 10.1016/j.anbehav.2005.01.016
Forstmeier W, 2011, BEHAV ECOL SOCIOBIOL, V65, P47, DOI 10.1007/s00265-010-1038-5
Freckleton RP, 2011, BEHAV ECOL SOCIOBIOL, V65, P91, DOI 10.1007/s00265-010-1045-6
Gardner A, 2011, J EVOLUTION BIOL, V24, P1020, DOI 10.1111/j.1420-9101.2011.02236.x
Garratt M, 2015, CURR BIOL, V25, P759, DOI 10.1016/j.cub.2014.11.071
Gilchrist JS, 2008, P ROY SOC B-BIOL SCI, V275, P2491, DOI 10.1098/rspb.2008.0597
Gilchrist JS, 2008, J ZOOL, V275, P41, DOI 10.1111/j.1469-7998.2007.00405.x
Gilchrist JS, 2006, BEHAV ECOL, V17, P664, DOI 10.1093/beheco/ark012
Gilchrist JS, 2004, BEHAV ECOL, V15, P952, DOI 10.1093/beheco/arh071
Hadfield JD, 2006, MOL ECOL, V15, P3715, DOI 10.1111/j.1365-294X.2006.03050.x
HAMILTON WD, 1963, AM NAT, V97, P354, DOI 10.1086/497114
HAMILTON WD, 1964, J THEOR BIOL, V7, P1, DOI [10.1016/0022-5193(64)90038-4, 10.1016/0022-5193(64)90039-6]
Hatchwell BJ, 2004, BEHAV ECOL, V15, P1, DOI 10.1093/beheco/arg091
Hawkes K, 1998, P NATL ACAD SCI USA, V95, P1336, DOI 10.1073/pnas.95.3.1336
Hill K.R., 1996, Ache life history: The ecology and demography of a foraging people
Hodge SJ, 2011, BIOL LETTERS, V7, P54, DOI 10.1098/rsbl.2010.0555
Inzani EL, 2016, SCI REP-UK, V6, DOI 10.1038/srep20013
Johnstone RA, 2012, P ROY SOC B-BIOL SCI, V279, P787, DOI 10.1098/rspb.2011.1257
Jones OR, 2010, MOL ECOL RESOUR, V10, P551, DOI 10.1111/j.1755-0998.2009.02787.x
Jordan NR, 2010, J ZOOL, V280, P72, DOI 10.1111/j.1469-7998.2009.00646.x
Koenig WD, 2016, COOPERATIVE BREEDING, DOI DOI 10.1017/CBO9781107338357
Kokko H, 2001, P ROY SOC B-BIOL SCI, V268, P187, DOI 10.1098/rspb.2000.1349
KOMDEUR J, 1994, BEHAV ECOL SOCIOBIOL, V34, P175, DOI 10.1007/BF00167742
Lahdenperä M, 2004, NATURE, V428, P178, DOI 10.1038/nature02367
Lahdenperä M, 2016, SCI REP-UK, V6, DOI 10.1038/srep27213
Lahdenperä M, 2012, ECOL LETT, V15, P1283, DOI 10.1111/j.1461-0248.2012.01851.x
Lehmann L, 2007, J EVOLUTION BIOL, V20, P181, DOI 10.1111/j.1420-9101.2006.01202.x
Lehmann L, 2010, PHILOS T R SOC B, V365, P2599, DOI 10.1098/rstb.2010.0138
Liu D, 1997, SCIENCE, V277, P1659, DOI 10.1126/science.277.5332.1659
Mace R., 2005, Grandmotherhood: The evolutionary significance of the second half of female life, P143

Mace R, 2012, P ROY SOC B-BIOL SCI, V279, P2219, DOI
10.1098/rspb.2011.2424
Marshall HH, 2016, BEHAV ECOL, V27, P978, DOI 10.1093/beheco/arw006
Müller CA, 2008, ANIM BEHAV, V75, P1683, DOI
10.1016/j.anbehav.2007.10.021
Nichols HJ, 2012, ANIM BEHAV, V83, P1377, DOI
10.1016/j.anbehav.2012.03.005
Nichols HJ, 2010, ANIM BEHAV, V80, P649, DOI
10.1016/j.anbehav.2010.06.025
Nowak MA, 2006, SCIENCE, V314, P1560, DOI 10.1126/science.1133755
Oster GeorgeF., 1979, CASTE ECOLOGY SOCIAL
Raihani NJ, 2012, TRENDS ECOL EVOL, V27, P288, DOI
10.1016/j.tree.2011.12.004
ROGERS AR, 1993, EVOL ECOL, V7, P406, DOI 10.1007/BF01237872
ROOD J P, 1975, East African Wildlife Journal, V13, P89
Russell AF, 2007, P R SOC B, V274, P513, DOI 10.1098/rspb.2006.3698
Sanderson JL, 2015, MOL ECOL, V24, P3738, DOI 10.1111/mec.13253
Schwander T, 2010, TRENDS ECOL EVOL, V25, P275, DOI
10.1016/j.tree.2009.12.001
Sheppard CE, 2018, CURR BIOL, V28, P1846, DOI
10.1016/j.cub.2018.05.001
Team RC, 2013, R: a language and environment for statistical computing
Thompson FJ, 2017, ANIM BEHAV, V134, P103, DOI
10.1016/j.anbehav.2017.10.009
Thompson FJ, 2017, P NATL ACAD SCI USA, V114, P5207, DOI
10.1073/pnas.1612235114
Thompson FJ, 2016, P ROY SOC B-BIOL SCI, V283, DOI
10.1098/rspb.2015.2607
van der Horst FCP, 2008, INTEGR PSYCHOL BEHAV, V42, P325, DOI
10.1007/s12124-008-9071-x
Vitikainen EIK, 2017, P ROY SOC B-BIOL SCI, V284, DOI
10.1098/rspb.2016.2384
Wells DA, 2018, MOL ECOL, V27, P2271, DOI 10.1111/mec.14570
Whittingham MJ, 2006, J ANIM ECOL, V75, P1182, DOI 10.1111/j.1365-
2656.2006.01141.x
NR 69
TC 18
Z9 18
U1 5
U2 15
PU ROYAL SOC
PI LONDON
PA 6-9 CARLTON HOUSE TERRACE, LONDON SW1Y 5AG, ENGLAND
SN 0962-8436
EI 1471-2970
J9 PHILOS T R SOC B
JI Philos. Trans. R. Soc. B-Biol. Sci.
PD APR 15
PY 2019
VL 374
IS 1770
AR 20180114
DI 10.1098/rstb.2018.0114
PG 9

WC Biology
WE Science Citation Index Expanded (SCI-EXPANDED)
SC Life Sciences & Biomedicine - Other Topics
GA HN8ZI
UT WOS:000460487000005
PM 30966878
OA Green Published, Green Submitted, hybrid
DA 2025-05-07
ER

PT J
AU Jeje, O
   Ewunkem, AJ
   Jeffers-Francis, LK
   Graves, JL
AF Jeje, Olusola
   Ewunkem, Akamu J.
   Jeffers-Francis, Liesl K.
   Graves Jr, Joseph L.
TI Serving Two Masters: Effect of Escherichia coli Dual Resistance
   on Antibiotic Susceptibility
SO ANTIBIOTICS-BASEL
LA English
DT Article
DE Escherichia coli; T7 bacteriophage; antibiotic; iron (III);
experimental
   evolution; antimicrobial-resistance
ID STRESS-RESPONSE; SALMONELLA COLONIZATION; SIGNAL-TRANSDUCTION;
OXIDATIVE
   STRESS; EVOLUTION; GROWTH; MUTANTS; PROTEIN; OPERON; GENES
AB The prevalence of multidrug-resistant bacteria and their increased pathogenicity has led to a growing interest in metallic antimicrobial materials and bacteriophages as potential alternatives to conventional antibiotics. This study examines how resistance to excess iron (III) influences the evolution of bacteriophage resistance in the bacterium Escherichia coli. We utilized experimental evolution in E. coli to test the effect of the evolution of phage T7 resistance on populations resistant to excess iron (III) and populations without excess iron resistance. Phage resistance evolved rapidly in both groups. Dual-resistant (iron (III)/phage) populations were compared to their controls (excess iron (III)-resistant, phage-resistant, no resistance to either) for their performance against each stressor, excess iron (III) and phage; and correlated resistances to excess iron (II), gallium (III), silver (I) and conventional antibiotics. Excess iron (III)/phage-resistant populations demonstrated superior 24 h growth compared to all other populations when exposed to increasing concentrations of iron (II, III), gallium (III), ampicillin, and tetracycline. No differences in 24 h growth were shown between excess iron (III)/phage-resistant and excess iron (III)-resistant populations in chloramphenicol, sulfonamide, and silver (I). The genomic analysis identified selective sweeps in the iron (III) resistant (rpoB, rpoC, yegB, yeaG), phage-resistant (clpX ->/-> lon, uvaB, yeaG, fliR, gatT, ypjF, waaC, rpoC, pgi, and yjbH) and iron (III)/phage resistant populations (rcsA, hldE, rpoB, and waaC). E. coli selected for resistance to both excess iron (III) and T7 phage showed

some evidence of a synergistic effect on various components of fitness. Dual selection resulted in correlated resistances to ionic metals {iron (II), gallium (III), and silver (I)} and several conventional antibiotics. There is a likelihood that this sort of combination antimicrobial treatment may result in bacterial variants with multiple resistances.
C1 [Jeje, Olusola; Jeffers-Francis, Liesl K.; Graves Jr, Joseph L.] North Carolina Agr & Tech State Univ, Biol Dept, 1601 E Market St, Greensboro, NC 27411 USA.
[Ewunkem, Akamu J.] Winston Salem State Univ, Dept Biol Sci, 601 S Martin Luther King Jr Dr, Winston Salem, NC 27110 USA.
C3 University of North Carolina; North Carolina A&T State University; Winston-Salem State University
RP Jeffers-Francis, LK (autor correspondente), North Carolina Agr & Tech State Univ, Biol Dept, 1601 E Market St, Greensboro, NC 27411 USA.
EM ljeffers@ncat.edu
OI Jeje, Olusola/0000-0003-0629-8664
FU NSF [IOS-2132240]; NSF BEACON Center for the Study of Evolution in Action [DBI-0939454]; Triangle Center for Evolutionary Medicine (TriCEM)
Graduate Student Award
FX We acknowledged funds from NSF IOS-2132240, NSF BEACON Center for the Study of Evolution in Action DBI-0939454, and the Triangle Center for Evolutionary Medicine (TriCEM) Graduate Student Award. The authors declare that they have no competing interests.
CR Al Mamun AM, 2012, SCIENCE, V338, P1344, DOI 10.1126/science.1226683
Aminov RI, 2007, FEMS MICROBIOL LETT, V271, P147, DOI 10.1111/j.1574-6968.2007.00757.x
Andrews JM, 2001, J ANTIMICROB CHEMOTH, V48, P5, DOI 10.1093/jac/48.suppl_1.5
Ansari SA, 2017, POL J CHEM TECHNOL, V19, P110, DOI 10.1515/pjct-2017-0076
BEHER MG, 1981, J BACTERIOL, V147, P972, DOI 10.1128/JB.147.3.972-985.1981
Berardi AE, 2021, PLANT CELL, V33, P2273, DOI 10.1093/plcell/koab114
Bin Zaman S, 2017, CUREUS J MED SCIENCE, V9, DOI 10.7759/cureus.1403
Borie C, 2008, AVIAN DIS, V52, P64, DOI 10.1637/8091-082007-Reg
Carrillo CL, 2005, APPL ENVIRON MICROB, V71, P6554, DOI 10.1128/AEM.71.11.6554-6563.2005
Chan BK, 2016, SCI REP-UK, V6, DOI 10.1038/srep26717
Conibear TCR, 2009, PLOS ONE, V4, DOI 10.1371/journal.pone.0006289
Cutugno L, 2020, MICROBIOL-SGM, V166, P1160, DOI 10.1099/mic.0.000991
Dedrick RM, 2019, NAT MED, V25, P730, DOI 10.1038/s41591-019-0437-z
Ewunkem AJ, 2021, NANOMATERIALS-BASEL, V11, DOI 10.3390/nano11030790
Fard RMN, 2011, VET MICROBIOL, V148, P276, DOI 10.1016/j.vetmic.2010.09.002
Ferenci T, 2016, TRENDS MICROBIOL, V24, P209, DOI 10.1016/j.tim.2015.11.009
Ferrières L, 2007, MICROBIOL-SGM, V153, P1070, DOI 10.1099/mic.0.2006/002907-0
Figueira R, 2015, SCI REP-UK, V5, DOI 10.1038/srep17524
Graves J.L, 2021, ANTIMICROBIAL NANOMA
Graves JL., 2020, EVOLUTION ACTION PRE, P91, DOI DOI 10.1007/978-3-030-39831-6_9

Graves JL, 2019, EVOL MED PUBLIC HLTH, P169, DOI 10.1093/emph/eoz025
Gu H, 2019, J BACTERIOL, V201, DOI 10.1128/JB.00034-19
Guisbert E, 2007, J BACTERIOL, V189, P1963, DOI 10.1128/JB.01243-06
Hall BG, 2014, MOL BIOL EVOL, V31, P232, DOI 10.1093/molbev/mst187
Hantke K, 2020, FEMS MICROBIOL LETT, V367, DOI 10.1093/femsle/fnaa013
Heinrichs DE, 1998, MOL MICROBIOL, V30, P221, DOI 10.1046/j.1365-2958.1998.01063.x
Hengge-Aronis R, 2002, MICROBIOL MOL BIOL R, V66, P373, DOI 10.1128/MMBR.66.3.373-395.2002
Hershberg R, 2017, TRENDS GENET, V33, P521, DOI 10.1016/j.tig.2017.05.003
Hirakawa H, 2003, J ANTIMICROB CHEMOTH, V52, P576, DOI 10.1093/jac/dkg406
Huff WE, 2002, POULTRY SCI, V81, P1486, DOI 10.1093/ps/81.10.1486
HUGOUVIEUXCOTTEPATTAT N, 1981, MOL GEN GENET, V182, P279, DOI 10.1007/BF00269671
Ibanez-Ruiz M, 2000, J BACTERIOL, V182, P5749, DOI 10.1128/JB.182.20.5749-5756.2000
Ionescu M, 2008, ARCH MICROBIOL, V189, P219, DOI 10.1007/s00203-007-0311-0
Kadrmas JL, 1998, J BIOL CHEM, V273, P2799, DOI 10.1074/jbc.273.5.2799
Kedziora Anna, 2013, Polim Med, V43, P129
Lee C, 2008, ENVIRON SCI TECHNOL, V42, P4927, DOI 10.1021/es800408u
Lenski RE, 2017, ISME J, V11, P2181, DOI 10.1038/ismej.2017.69
Meberg BM, 2001, J BACTERIOL, V183, P6148, DOI 10.1128/JB.183.20.6148-6149.2001
Neuwald AF, 1999, GENOME RES, V9, P27
NUNOSHIBA T, 1992, J BACTERIOL, V174, P6054, DOI 10.1128/JB.174.19.6054-6060.1992
PORTALIER RC, 1972, EUR J BIOCHEM, V26, P50, DOI 10.1111/j.1432-1033.1972.tb01738.x
Reyes-Cortés R, 2012, MICROBIOL-SGM, V158, P3063, DOI 10.1099/mic.0.060970-0
Reynolds MG, 2000, GENETICS, V156, P1471
Riley M, 2006, NUCLEIC ACIDS RES, V34, P1, DOI 10.1093/nar/gkj405
Rungrassamee W, 2008, ARCH MICROBIOL, V190, P41, DOI 10.1007/s00203-008-0361-y
Saez AC, 2011, FOODBORNE PATHOG DIS, V8, P1269, DOI 10.1089/fpd.2011.0905
Seo SW, 2014, NAT COMMUN, V5, DOI 10.1038/ncomms5910
Silva JB, 2016, FEMS MICROBIOL LETT, V363, DOI 10.1093/femsle/fnw002
Sugden R, 2016, NAT MICROBIOL, V1, DOI [10.1038/NMICROBIOL.2016.187, 10.1038/nmicrobiol.2016.187]
Sulakvelidze A, 2001, ANTIMICROB AGENTS CH, V45, P649, DOI 10.1128/AAC.45.3.649-659.2001
Thomas MD, 2021, EVOL MED PUBLIC HLTH, V9, P53, DOI 10.1093/emph/eoaa051
Tyerman JG, 2013, BMC EVOL BIOL, V13, DOI 10.1186/1471-2148-13-22
Vahjen W, 2015, GUT PATHOG, V7, DOI 10.1186/s13099-015-0071-3
Weber H, 2005, J BACTERIOL, V187, P1591, DOI 10.1128/JB.187.5.1591-1603.2005
Weichart D, 2003, J BACTERIOL, V185, P115, DOI 10.1128/JB.185.1.115-125.2003

Wrande M, 2008, P NATL ACAD SCI USA, V105, P11863, DOI
10.1073/pnas.0804739105
Yokoyama M, 2018, GENOME BIOL, V19, DOI 10.1186/s13059-018-1469-2
Zheng M, 1998, SCIENCE, V279, P1718, DOI 10.1126/science.279.5357.1718
NR 58
TC 1
Z9 1
U1 3
U2 13
PU MDPI
PI BASEL
PA ST ALBAN-ANLAGE 66, CH-4052 BASEL, SWITZERLAND
SN 2079-6382
J9 ANTIBIOTICS-BASEL
JI Antibiotics-Basel
PD MAR
PY 2023
VL 12
IS 3
AR 603
DI 10.3390/antibiotics12030603
PG 20
WC Infectious Diseases; Pharmacology & Pharmacy
WE Science Citation Index Expanded (SCI-EXPANDED)
SC Infectious Diseases; Pharmacology & Pharmacy
GA A7VD6
UT WOS:000957150000001
PM 36978471
OA gold, Green Published
DA 2025-05-07
ER

PT J
AU Young, HS
Wood, CL
Kilpatrick, AM
Lafferty, KD
Nunn, CL
Vincent, JR
AF Young, Hillary S.
Wood, Chelsea L.
Kilpatrick, A. Marm
Lafferty, Kevin D.
Nunn, Charles L.
Vincent, Jeffrey R.
TI Conservation, biodiversity and infectious disease: scientific evidence
and policy implications
SO PHILOSOPHICAL TRANSACTIONS OF THE ROYAL SOCIETY B-BIOLOGICAL SCIENCES
LA English
DT Editorial Material
ID HEALTH IMPACTS; DIVERSITY; RISK
C1 [Young, Hillary S.] UC Santa Barbara, Ecol Evolut & Marine Biol,
Goleta, CA 93106 USA.

[Wood, Chelsea L.] Univ Michigan, Dept Ecol & Evolutionary Biol, Ann Arbor, MI 48104 USA.
[Wood, Chelsea L.] Univ Michigan, Michiban Soc Fellows, Ann Arbor, MI 48104 USA.
[Wood, Chelsea L.] Univ Washington, Sch Aquat & Fishery Sci, Seattle, WA 98195 USA.
[Kilpatrick, A. Marm] UC Santa Cruz, EEB, Santa Cruz, CA USA.
[Lafferty, Kevin D.] Univ Calif Santa Barbara, Marine Sci Inst, Santa Barbara, CA 93106 USA.
[Nunn, Charles L.] Duke Univ, Duke Global Hlth Inst, Durham, NC USA.
[Nunn, Charles L.] Duke Univ, Dept Evolutionary Anthropol Duke, Durham, NC USA.
[Vincent, Jeffrey R.] Duke Univ, Nicholas Sch Environm, Durham, NC USA.
C3 University of California System; University of California Santa Barbara;
University of Michigan System; University of Michigan; University of Michigan System; University of Michigan; University of Washington; University of Washington Seattle; University of California System; University of California Santa Cruz; University of California System; University of California Santa Barbara; Duke University; Duke University; Duke University
RP Young, HS (autor correspondente), UC Santa Barbara, Ecol Evolut & Marine Biol, Goleta, CA 93106 USA.
EM hillary.young@lifesci.ucsb.edu
RI Young, Hillary/S-1514-2017; Lafferty, Kevin/B-3888-2009; Vincent, Jeffrey/JZU-1149-2024; Kilpatrick, A Marm/HOI-0422-2023
OI Young, Hillary/0000-0003-0449-8582; Wood, Chelsea/0000-0003-2738-3139; Kilpatrick, A. Marm/0000-0002-3612-5775
FU Triangle Center for Evolutionary Medicine (TriCEM); National Evolutionary Synthesis Center (NESCent, NSF) [EF-0905606]; Duke Tropical
Conservation Initiative
FX The Catalysis Meeting ('Biodiversity, Conservation and Infectious Disease') was sponsored by the Triangle Center for Evolutionary Medicine
(TriCEM) and the National Evolutionary Synthesis Center (NESCent, NSF #EF-0905606), along with the Duke Tropical Conservation Initiative.
CR [Anonymous], 2016, ECOLOGY, DOI DOI 10.1890/15-1503.1
Aronson JC, 2016, ECOL SOC, V21, DOI 10.5751/ES-08974-210439
Barrett CB, 2011, P NATL ACAD SCI USA, V108, P13907, DOI 10.1073/pnas.1011521108
Bauch SC, 2015, P NATL ACAD SCI USA, V112, P7414, DOI 10.1073/pnas.1406495111
Bernstein AS, 2014, ANNU REV PUBL HEALTH, V35, P153, DOI 10.1146/annurev-publhealth-032013-182348
Civitello DJ, 2015, P NATL ACAD SCI USA, V112, P8667, DOI 10.1073/pnas.1506279112
Dornelas M, 2014, SCIENCE, V344, P296, DOI 10.1126/science.1248484
Faust CL, 2017, PHILOS T R SOC B, V372, DOI 10.1098/rstb.2016.0173
Garchitorena A, 2017, PHILOS T R SOC B, V372, DOI 10.1098/rstb.2016.0128
Gottdenker NL, 2014, ECOHEALTH, V11, P619, DOI 10.1007/s10393-014-0941-z

Hill SLL, 2016, CONSERV LETT, V9, P405, DOI 10.1111/conl.12291
Hosseini PR, 2017, PHILOS T R SOC B, V372, DOI 10.1098/rstb.2016.0129
Kilpatrick AM, 2017, PHILOS T R SOC B, V372, DOI 10.1098/rstb.2016.0131
Kilpatrick AM, 2017, PHILOS T R SOC B, V372, DOI 10.1098/rstb.2016.0117
Lima JMT, 2017, PHILOS T R SOC B, V372, DOI 10.1098/rstb.2016.0125
McGill BJ, 2015, TRENDS ECOL EVOL, V30, P104, DOI 10.1016/j.tree.2014.11.006
Millins C, 2017, PHILOS T R SOC B, V372, DOI 10.1098/rstb.2016.0123
Myers SS, 2013, P NATL ACAD SCI USA, V110, P18753, DOI 10.1073/pnas.1218656110
Ostfeld RS, 2012, ANNU REV ECOL EVOL S, V43, P157, DOI 10.1146/annurev-ecolsys-102710-145022
Pattanayak SK, 2017, PHILOS T R SOC B, V372, DOI 10.1098/rstb.2016.0130
Redford KH, 2014, CONSERV BIOL, V28, P627, DOI 10.1111/cobi.12290
Romanelli C., 2015, Connecting global priorities: Biodiversity and human health: a state of knowledge review
Salkeld DJ, 2013, ECOL LETT, V16, P679, DOI 10.1111/ele.12101
Smith KR, 2005, ANNU REV ENV RESOUR, V30, P291, DOI 10.1146/annurev.energy.30.050504.144424
Sokolow SH, 2017, PHILOS T R SOC B, V372, DOI 10.1098/rstb.2016.0127
Velasquez-Manoff M., 2016, NY TIMES, pSR9
Vellend M, 2017, ECOLOGY, V98, P583, DOI 10.1002/ecy.1660
Vellend M, 2013, P NATL ACAD SCI USA, V110, P19456, DOI 10.1073/pnas.1312779110
Wood CL, 2017, PHILOS T R SOC B, V372, DOI 10.1098/rstb.2016.0122
Wu T, 2017, PHILOS T R SOC B, V372, DOI 10.1098/rstb.2016.0126
Young HS, 2017, PHILOS T R SOC B, V372, DOI 10.1098/rstb.2016.0116
Young HS, 2016, ANNU REV ECOL EVOL S, V47, P333, DOI 10.1146/annurev-ecolsys-112414-054142
NR 32
TC 22
Z9 22
U1 0
U2 39
PU ROYAL SOC
PI LONDON
PA 6-9 CARLTON HOUSE TERRACE, LONDON SW1Y 5AG, ENGLAND
SN 0962-8436
EI 1471-2970
J9 PHILOS T R SOC B
JI Philos. Trans. R. Soc. B-Biol. Sci.
PD JUN 5
PY 2017
VL 372
IS 1722
AR 20160124
DI 10.1098/rstb.2016.0124
PG 4
WC Biology
WE Science Citation Index Expanded (SCI-EXPANDED)
SC Life Sciences & Biomedicine - Other Topics

GA ET0ML
UT WOS:000399956400005
PM 28438913
OA Bronze, Green Published
DA 2025-05-07
ER

PT J
AU Au, EH
Fauci, C
Luo, YT
Mangan, RJ
Snellings, DA
Shoben, CR
Weaver, S
Simpson, SK
Lowe, CB
AF Au, Eric H.
Fauci, Christiana
Luo, Yanting
Mangan, Riley J.
Snellings, Daniel A.
Shoben, Chelsea R.
Weaver, Seth
Simpson, Shae K.
Lowe, Craig B.
TI Gonomics: uniting high performance and readability for genomics with Go
SO BIOINFORMATICS
LA English
DT Article
AB Many existing software libraries for genomics require researchers to pick between competing considerations: the performance of compiled languages and the accessibility of interpreted languages. Go, a modern compiled language, provides an opportunity to address this conflict. We introduce Gonomics, an open-source collection of command line programs and bioinformatic libraries implemented in Go that unites readability and performance for genomic analyses. Gonomics contains packages to read, write, and manipulate a wide array of file formats (e.g. FASTA, FASTQ, BED, BEDPE, SAM, BAM, and VCF), and can convert and interface between these formats. Furthermore, our modular library structure provides a flexible platform for researchers developing their own software tools to address specific questions. These commands can be combined and incorporated into complex pipelines to meet the growing need for high-performance bioinformatic resources.

Availability and implementation: Gonomics is implemented in the Go programming language. Source code, installation instructions, and documentation are freely available at https://github.com/vertgenlab/gonomics.
C1 [Au, Eric H.; Fauci, Christiana; Luo, Yanting; Mangan, Riley J.; Snellings, Daniel A.; Shoben, Chelsea R.; Weaver, Seth; Simpson, Shae K.; Lowe, Craig B.] Duke Univ, Sch Med, Dept Mol Genet & Microbiol, Durham, NC 27710 USA.

[Fauci, Christiana; Luo, Yanting; Shoben, Chelsea R.; Weaver, Seth; Lowe, Craig B.] Duke Univ, Univ Program Genet & Genom, Sch Med, Durham, NC 27710 USA.
[Au, Eric H.] Mammoth Biosci Inc, Brisbane, CA 94005 USA.
[Snellings, Daniel A.] Harvard Med Sch, Boston Childrens Hosp, Boston, MA 02115 USA.
C3 Duke University; Duke University; Harvard University; Harvard University
Medical Affiliates; Boston Children's Hospital; Harvard Medical School
RP Lowe, CB (autor correspondente), Duke Univ, Sch Med, Dept Mol Genet & Microbiol, Durham, NC 27710 USA.
EM craig.lowe@duke.edu
RI Au, Eric/I-9582-2019
OI Lowe, Craig/0000-0002-6838-1976; Snellings, Daniel/0000-0003-0615-9111;
Simpson, Shae/0009-0000-8966-6180; Mangan, Riley/0000-0003-3342-3934;
Fauci, Christiana/0000-0002-7458-813X
FU NHGRI of the National Institutes of Health [R35HG011332]; Duke Whitehead
Scholarship; Triangle Center for Evolutionary Medicine; John Templeton
Foundation [62220]
FX This work was supported by the NHGRI of the National Institutes of
Health under award number R35HG011332, the Duke Whitehead Scholarship,
and the Triangle Center for Evolutionary Medicine. We gratefully
acknowledge the financial support of the John Templeton Foundation (#
62220).The opinions expressed in this paper are those of the authorsand
not those of the funders.
CR Abraham A, 2023, BIOINFORMATICS, V39, DOI 10.1093/bioinformatics/btad037
Bonfield JK., 2021, Gigascience, V10, P1
Butler A, 2018, NAT BIOTECHNOL, V36, P411, DOI 10.1038/nbt.4096
Cock PJA, 2009, BIOINFORMATICS, V25, P1422, DOI 10.1093/bioinformatics/btp163
Costanza P, 2019, EVOL BIOINFORM, V15, DOI 10.1177/1176934319869015
Costanza P, 2019, BMC BIOINFORMATICS, V20, DOI 10.1186/s12859-019-2903-5
Hickey G, 2020, GENOME BIOL, V21, DOI 10.1186/s13059-020-1941-7
Kent WJ, 2010, BIOINFORMATICS, V26, P2204, DOI 10.1093/bioinformatics/btq351
Kortschak, 2017, J OPEN SOURCE SOFTW, V2, P167, DOI DOI 10.21105/JOSS.00167
Li H, 2009, BIOINFORMATICS, V25, P1754, DOI [10.1093/bioinformatics/btp698, 10.1093/bioinformatics/btp324, 10.1093/bioinformatics/btp352]
Mangan RJ, 2022, CELL, V185, P4587, DOI 10.1016/j.cell.2022.10.016
Mao CS, 2019, SCI REP-UK, V9, DOI 10.1038/s41598-019-41451-3
McLaren W, 2016, GENOME BIOL, V17, DOI 10.1186/s13059-016-0974-4
Mousavi N, 2021, BIOINFORMATICS, V37, P731, DOI 10.1093/bioinformatics/btaa736
Neph S, 2012, BIOINFORMATICS, V28, P1919, DOI 10.1093/bioinformatics/bts277
Quinlan AR, 2010, BIOINFORMATICS, V26, P841, DOI 10.1093/bioinformatics/btq033

   Ren AA, 2021, NATURE, V594, P271, DOI 10.1038/s41586-021-03562-8
   Saelens JW, 2022, CELL, V185, P4507, DOI 10.1016/j.cell.2022.10.019
   Shen W, 2016, PLOS ONE, V11, DOI 10.1371/journal.pone.0163962
   Snellings DA, 2022, NAT CARDIOVASC RES, V1, P246, DOI 10.1038/s44161-022-00035-7
NR 20
TC 1
Z9 1
U1 0
U2 0
PU OXFORD UNIV PRESS
PI OXFORD
PA GREAT CLARENDON ST, OXFORD OX2 6DP, ENGLAND
SN 1367-4803
EI 1367-4811
J9 BIOINFORMATICS
JI Bioinformatics
PD AUG 1
PY 2023
VL 39
IS 8
AR btad516
DI 10.1093/bioinformatics/btad516
EA AUG 2023
PG 4
WC Biochemical Research Methods; Biotechnology & Applied Microbiology;
   Computer Science, Interdisciplinary Applications; Mathematical &
   Computational Biology; Statistics & Probability
WE Science Citation Index Expanded (SCI-EXPANDED)
SC Biochemistry & Molecular Biology; Biotechnology & Applied Microbiology;
   Computer Science; Mathematical & Computational Biology; Mathematics
GA Q5UX2
UT WOS:001058183600004
PM 37624924
OA gold, Green Published
DA 2025-05-07
ER

PT J
AU Swanson, ZS
   Nzunza, R
   Bethancourt, HJ
   Ndiema, E
   Braun, DR
   Rosinger, AY
   Pontzer, H
AF Swanson, Zane S.
   Nzunza, Rosemary
   Bethancourt, Hilary J.
   Ndiema, Emmanuel
   Braun, David R.
   Rosinger, Asher Y.
   Pontzer, Herman

TI Variation in market integration and health affect early childhood growth
in Daasanach pastoralists living in northern Kenya
SO AMERICAN JOURNAL OF BIOLOGICAL ANTHROPOLOGY
LA English
DT Meeting Abstract
C1 [Swanson, Zane S.; Pontzer, Herman] Duke Univ, Evolutionary Anthropol, Durham, NC 27706 USA.
[Nzunza, Rosemary] Kenya Med Res Inst KEMRI, Nairobi, Kenya.
[Bethancourt, Hilary J.; Rosinger, Asher Y.] Penn State Univ, Dept Anthropol, University Pk, PA 16802 USA.
[Bethancourt, Hilary J.] Northwestern Univ, Dept Anthropol, Evanston, IL 60208 USA.
[Ndiema, Emmanuel] Natl Museums Kenya, Dept Earth Sci, Nairobi, Kenya.
[Braun, David R.] George Washington Univ, Ctr Adv Study Human Paleobiol, Dept Anthropol, Washington, DC 20052 USA.
[Braun, David R.] Max Planck Inst Evolutionary Anthropol, Dept Human Evolut, Leipzig, Germany.
[Rosinger, Asher Y.] Penn State Univ, Dept Biobehav Hlth, University Pk, PA 16802 USA.
[Pontzer, Herman] Duke Univ, Global Hlth Inst, Durham, NC 27706 USA.
C3 Duke University; Kenya Medical Research Institute; Pennsylvania Commonwealth System of Higher Education (PCSHE); Pennsylvania State University; Penn State Behrend; Pennsylvania State University - University Park; Northwestern University; George Washington University;
Max Planck Society; Pennsylvania Commonwealth System of Higher Education
(PCSHE); Pennsylvania State University; Penn State Behrend; Pennsylvania
State University - University Park; Duke University
RI Braun, David/C-9680-2009
FU NSF ARCH [1624398]; NSF REU [1930719]; PSU SSRI Human Health and Environment Seed Grant; Triangle Center for Evolutionary Medicine (TriCEM) Graduate Research Grant
FX NSF ARCH #1624398; NSF REU #1930719; This work was funded by the PSU SSRI Human Health and Environment Seed Grant, and the Triangle Center for Evolutionary Medicine (TriCEM) Graduate Research Grant.
NR 0
TC 0
Z9 0
U1 0
U2 0
PU WILEY
PI HOBOKEN
PA 111 RIVER ST, HOBOKEN 07030-5774, NJ USA
SN 2692-7691
J9 AMER J BIOL ANTHROPO
JI Amer. J. Biol. Anthropol.
PD APR
PY 2022
VL 177
SU 73
SI SI

BP 176
EP 177
PG 2
WC Anthropology; Evolutionary Biology
WE Science Citation Index Expanded (SCI-EXPANDED); Social Science Citation Index (SSCI)
SC Anthropology; Evolutionary Biology
GA ZX9XJ
UT WOS:000772245500663
DA 2025-05-07
ER

PT J
AU Shitikov, E
Guliaev, A
Bespyatykh, J
Malakhova, M
Kolchenko, S
Smirnov, G
Merker, M
Niemann, S
Mokrousov, I
Ilina, E
Govorun, V
AF Shitikov, Egor
Guliaev, Andrei
Bespyatykh, Julia
Malakhova, Maja
Kolchenko, Sergey
Smirnov, Georgy
Merker, Matthias
Niemann, Stefan
Mokrousov, Igor
Ilina, Elena
Govorun, Vadim
TI The role of IS6110 in micro- and macroevolution of Mycobacterium tuberculosis lineage 2
SO MOLECULAR PHYLOGENETICS AND EVOLUTION
LA English
DT Article
DE Mycobacterium tuberculosis; IS6110; Insertion sequence; Recombination; Lineage 2; Beijing
ID MULTIPLEX PCR ASSAY; BEIJING GENOTYPE; INSERTION-SEQUENCE; STRAINS; TRANSPOSITION; POLYMORPHISM; EVOLUTION; SITES; IDENTIFICATION; MUTATIONS
AB The insertion sequence 6110 (IS6110) is the most studied transposable element in the Mycobacterium tuberculosis complex species. The element plays a significant role in genome plasticity of this important human pathogen, but still many causes and consequences of its transposition have not been fully studied. Here, we analyzed insertion sites for 902 Mycobacterium tuberculosis lineage 2 strains using whole-genome sequencing data. In total, 17,972 insertions were found, corresponding to 827 independent positions in the genome of the reference strain H37Rv. To trace the history of IS6110 expansion since proto-Beijing strains up to

modern sublineages, we looked at the distribution of IS6110 across the genome-wide SNP-based phylogenetic tree. This analysis demonstrated a stepwise transposition of IS6110 that occurs by "copy-and-paste" mechanism. Additionally, we detected evolutionary-scale and sublineage-specific integration sites, which can be used for typing and for understanding the reasons for the success of the lineage. A significant part of such insertions affected the genes that are essential for the pathogen. Finally, we identified and confirmed deletions that occurred between differently oriented elements, which is uncommon for this family of insertion elements and appears to be another mechanism of genome variability.
C1 [Shitikov, Egor; Guliaev, Andrei; Bespyatykh, Julia; Malakhova, Maja; Kolchenko, Sergey; Smirnov, Georgy; Ilina, Elena; Govorun, Vadim] Fed Res & Clin Ctr Phys Chem Med, Moscow, Russia.
[Merker, Matthias; Niemann, Stefan] Leibniz Lung Ctr, Res Ctr Borstel, Mol & Expt Mycobacteriol, Borstel, Germany.
[Mokrousov, Igor] St Petersburg Pasteur Inst, St Petersburg, Russia.
[Merker, Matthias; Niemann, Stefan] German Ctr Infect Res, Partner Site Hamburg Lubeck Borstel Riems, Hamburg, Germany.
C3 Lopukhin Federal Research & Clinical Center of Physical-Chemical Medicine of Federal Medical Biological Agency; Leibniz Association; Forschungszentrum Borstel; Saint-Petersburg Pasteur Institute; German Center for Infection Research
RP Shitikov, E (autor correspondente), Fed Res & Clin Ctr Phys Chem Med, Moscow, Russia.
EM egorshtkv@gmail.com
RI Niemann, Stefan/C-9327-2011; Bespyatykh, Julia/Q-8132-2016; Guliaev, Andrei/S-2546-2019; Ilina, Elena/ABI-1172-2020; Govorun, Vadim/A-9043-2018; Mokrousov, Igor/J-3640-2014; Ilina, Elena/N-8190-2015; Shitikov, Egor/Q-2520-2017; Merker, Matthias/P-7399-2015; Smirnov, Alexey/AAB-2521-2020
OI Ilina, Elena/0000-0003-0130-5079; Niemann, Stefan/0000-0002-6604-0684; Shitikov, Egor/0000-0002-4865-6004; Merker, Matthias/0000-0003-1386-2331; Smirnov, Alexey/0000-0001-5361-8232; Guliaev, Andrei/0000-0002-6329-2421
FU RFBR [18-04-01035]; Russian Science Foundation [17-15-01412]; Leibniz Science Campus Evolutionary Medicine of the Lung (EvoLung); Russian Science Foundation [17-15-01412] Funding Source: Russian Science Foundation
FX This work was supported by RFBR according to the research project No 18-04-01035; analysis of bacterial DNA was funded by the Russian Science
Foundation (project No 17-15-01412) and by the Leibniz Science Campus Evolutionary Medicine of the Lung (EvoLung).
CR Alonso H, 2013, BMC GENOMICS, V14, DOI 10.1186/1471-2164-14-422
Ates LS, 2018, NAT MICROBIOL, V3, P181, DOI 10.1038/s41564-017-0090-6
Beggs ML, 2000, J CLIN MICROBIOL, V38, P2923, DOI 10.1128/JCM.38.8.2923-2928.2000
Brites D, 2015, IMMUNOL REV, V264, P6, DOI 10.1111/imr.12264
Casali N, 2014, NAT GENET, V46, P279, DOI 10.1038/ng.2878
Cortes T, 2013, CELL REP, V5, P1121, DOI 10.1016/j.celrep.2013.10.031
Dehal PS, 2010, NUCLEIC ACIDS RES, V38, pD396, DOI 10.1093/nar/gkp919
DeJesus MA, 2017, MBIO, V8, DOI 10.1128/mBio.02133-16

Fang Z, 1998, J BACTERIOL, V180, P2102, DOI 10.1128/JB.180.8.2102-2109.1998
Ghanekar K, 1999, MOL MICROBIOL, V33, P982, DOI 10.1046/j.1365-2958.1999.01539.x
Gonzalo-Asensio J, 2018, PLOS GENET, V14, DOI 10.1371/journal.pgen.1007282
Green E, 2013, J HEALTH POPUL NUTR, V31, P1
Gutacker MM, 2006, J INFECT DIS, V193, P121, DOI 10.1086/498574
Hanekom M, 2011, TUBERCULOSIS, V91, P510, DOI 10.1016/j.tube.2011.07.005
Hawkey J, 2015, BMC GENOMICS, V16, DOI 10.1186/s12864-015-1860-2
Kivi M, 2002, J CLIN MICROBIOL, V40, P2192, DOI 10.1128/JCM.40.6.2192-2198.2002
Lamichhane G, 2003, P NATL ACAD SCI USA, V100, P7213, DOI 10.1073/pnas.1231432100
Lemaitre N, 1999, ANTIMICROB AGENTS CH, V43, P1761, DOI 10.1128/AAC.43.7.1761
Letunic I, 2016, NUCLEIC ACIDS RES, V44, pW242, DOI 10.1093/nar/gkw290
Mao FL, 2009, NUCLEIC ACIDS RES, V37, pD459, DOI 10.1093/nar/gkn757
Maus CE, 2005, ANTIMICROB AGENTS CH, V49, P571, DOI 10.1128/AAC.49.2.571-577.2005
McEvoy CRE, 2007, TUBERCULOSIS, V87, P393, DOI 10.1016/j.tube.2007.05.010
Merker M, 2015, NAT GENET, V47, P242, DOI 10.1038/ng.3195
Mitra A, 2017, MBIO, V8, DOI 10.1128/mBio.01720-16
Mokrousov I, 2005, GENOME RES, V15, P1357, DOI 10.1101/gr.3840605
Mokrousov I, 2002, RES MICROBIOL, V153, P629, DOI 10.1016/S0923-2508(02)01374-8
Mokrousov I, 2012, J CLIN MICROBIOL, V50, P3757, DOI 10.1128/JCM.02001-12
Parwati I, 2010, LANCET INFECT DIS, V10, P103, DOI 10.1016/S1473-3099(09)70330-5
PLIKAYTIS BB, 1994, J CLIN MICROBIOL, V32, P1542, DOI 10.1128/JCM.32.6.1542-1546.1994
Raimunda D, 2012, MOL MICROBIOL, V84, P1139, DOI 10.1111/j.1365-2958.2012.08082.x
Reyes A, 2012, BMC GENOMICS, V13, DOI 10.1186/1471-2164-13-249
Roychowdhury T, 2015, SCI REP-UK, V5, DOI 10.1038/srep12567
Safi H, 2004, MOL MICROBIOL, V52, P999, DOI 10.1111/j.1365-2958.2004.04037.x
Sampson S, 2001, J CLIN MICROBIOL, V39, P3423, DOI 10.1128/JCM.39.9.3423-3424.2001
Sampson SL, 2003, J BACTERIOL, V185, P2856, DOI 10.1128/JB.185.9.2856-2866.2003
Sassetti CM, 2003, P NATL ACAD SCI USA, V100, P12989, DOI 10.1073/pnas.2134250100
Sassetti CM, 2003, MOL MICROBIOL, V48, P77, DOI 10.1046/j.1365-2958.2003.03425.x
Sharma D, 2016, FRONT MICROBIOL, V7, DOI 10.3389/fmicb.2016.01816
Shitikov E, 2017, SCI REP, V7, P92, DOI [10.1038/s41598-41017-10018-41595, DOI 10.1038/S41598-41017-10018-41595]
Soto CY, 2004, J CLIN MICROBIOL, V42, P212, DOI 10.1128/JCM.42.1.212-219.2004

Thabet S, 2014, INT J INFECT DIS, V29, P211, DOI
10.1016/j.ijid.2014.10.004
Thabet S, 2017, MOL BIOL REP, V44, P25, DOI 10.1007/s11033-016-4084-x
THIERRY D, 1990, J CLIN MICROBIOL, V28, P2668, DOI
10.1128/JCM.28.12.2668-2673.1990
Thorne N, 2011, INFECT GENET EVOL, V11, P132, DOI
10.1016/j.meegid.2010.09.011
Thorvaldsdóttir H, 2013, BRIEF BIOINFORM, V14, P178, DOI
10.1093/bib/bbs017
VANEMBDEN JDA, 1993, J CLIN MICROBIOL, V31, P406, DOI
10.1128/JCM.31.2.406-409.1993
Yang ZH, 2005, AM J RESP CRIT CARE, V171, P1436, DOI
10.1164/rccm.200408-1147OC
NR 47
TC 7
Z9 8
U1 1
U2 9
PU ACADEMIC PRESS INC ELSEVIER SCIENCE
PI SAN DIEGO
PA 525 B ST, STE 1900, SAN DIEGO, CA 92101-4495 USA
SN 1055-7903
EI 1095-9513
J9 MOL PHYLOGENET EVOL
JI Mol. Phylogenet. Evol.
PD OCT
PY 2019
VL 139
AR 106559
DI 10.1016/j.ympev.2019.106559
PG 7
WC Biochemistry & Molecular Biology; Evolutionary Biology; Genetics &
Heredity
WE Science Citation Index Expanded (SCI-EXPANDED)
SC Biochemistry & Molecular Biology; Evolutionary Biology; Genetics &
Heredity
GA IW5TW
UT WOS:000485041900043
PM 31302224
OA Green Published
DA 2025-05-07
ER

PT J
AU Saunders, J
Swanson, ZS
Bethancourt, H
Dhanasekar, S
Nzunza, R
Rosinger, AY
Pontzer, H
AF Saunders, Jessica
Swanson, Zane S.
Bethancourt, Hilary

Dhanasekar, Shiva
   Nzunza, Rosemary
   Rosinger, Asher Y.
   Pontzer, Herman
TI Human Biology and the Koobi Fora Field School: New Work with the
   Daasanach, a Small-Scale Pastoralist Population
SO AMERICAN JOURNAL OF PHYSICAL ANTHROPOLOGY
LA English
DT Meeting Abstract
CT 89th Annual Meeting of the
   American-Association-of-Physical-Anthropologists (AAPA)
CY APR 15-18, 2020
CL Los Angeles, CA
SP Amer Assoc Phys Anthropologists
C1 [Saunders, Jessica; Swanson, Zane S.; Pontzer, Herman] Duke Univ,
Evolutionary Anthropol, Durham, NC 27706 USA.
   [Bethancourt, Hilary; Rosinger, Asher Y.] Penn State Univ, Dept
Biobehav Hlth, University Pk, PA 16802 USA.
   [Dhanasekar, Shiva] Emory Univ, Dept Anthropol, Atlanta, GA 30322 USA.
   [Nzunza, Rosemary] KEMRI, Kenya Med Res Inst, Kilifi, Kenya.
   [Rosinger, Asher Y.] Penn State Univ, Dept Anthropol, University Pk,
PA 16802 USA.
   [Pontzer, Herman] Duke Univ, Global Hlth Inst, Durham, NC 27706 USA.
C3 Duke University; Pennsylvania Commonwealth System of Higher Education
   (PCSHE); Pennsylvania State University; Pennsylvania State University
-
   University Park; Penn State Behrend; Emory University; Kenya Medical
   Research Institute; Pennsylvania Commonwealth System of Higher
Education
   (PCSHE); Pennsylvania State University; Pennsylvania State University
-
   University Park; Penn State Behrend; Duke University
RI S, DHANASEKAR/GNH-2305-2022
FU NSF ARCH [1624398]; NSF REU [1930719]; PSU SSRI Human Health and
   Environment Seed Grant; Triangle Center for Evolutionary Medicine
   (TriCEM) Graduate Research Grant
FX NSF ARCH #1624398; NSF REU #1930719; This work was funded by the PSU
   SSRI Human Health and Environment Seed Grant, and the Triangle Center
   for Evolutionary Medicine (TriCEM) Graduate Research Grant.
NR 0
TC 0
Z9 0
U1 0
U2 1
PU WILEY
PI HOBOKEN
PA 111 RIVER ST, HOBOKEN 07030-5774, NJ USA
SN 0002-9483
EI 1096-8644
J9 AM J PHYS ANTHROPOL
JI Am. J. Phys. Anthropol.
PD MAR
PY 2020
VL 171

SU 69
BP 248
EP 248
PG 1
WC Anthropology; Evolutionary Biology
WE Science Citation Index Expanded (SCI-EXPANDED); Social Science Citation Index (SSCI); Conference Proceedings Citation Index - Science (CPCI-S)
SC Anthropology; Evolutionary Biology
GA KL2WI
UT WOS:000513288902352
DA 2025-05-07
ER

PT J
AU Aziz, A
Harrop, SP
Bishop, NE
AF Aziz, Azhari
Harrop, Sean P.
Bishop, Naomi E.
TI Characterization of the Deleted in Autism 1 Protein Family: Implications
for Studying Cognitive Disorders
SO PLOS ONE
LA English
DT Article
ID COMPARATIVE GENOMICS; SEQUENCE DIVERGENCE; SPECTRUM DISORDERS; VERTEBRATE GENOME; GENE DUPLICATION; AMPHIOXUS GENOME; NERVOUS-SYSTEM; MODEL ORGANISM; ANIMAL-MODELS; GOLGI PROTEIN
AB Autism spectrum disorders (ASDs) are a group of commonly occurring, highly-heritable developmental disabilities. Human genes c3orf58 or Deleted In Autism-1 (DIA1) and cXorf36 or Deleted in Autism-1 Related (DIA1R) are implicated in ASD and mental retardation. Both gene products encode signal peptides for targeting to the secretory pathway. As evolutionary medicine has emerged as a key tool for understanding increasing numbers of human diseases, we have used an evolutionary approach to study DIA1 and DIA1R. We found DIA1 conserved from cnidarians to humans, indicating DIA1 evolution coincided with the development of the first primitive synapses. Nematodes lack a DIA1 homologue, indicating Caenorhabditis elegans is not suitable for studying all aspects of ASD etiology, while zebrafish encode two DIA1 paralogues. By contrast to DIA1, DIA1R was found exclusively in vertebrates, with an origin coinciding with the whole-genome duplication events occurring early in the vertebrate lineage, and the evolution of the more complex vertebrate nervous system. Strikingly, DIA1R was present in schooling fish but absent in fish that have adopted a more solitary lifestyle. An additional DIA1-related gene we named DIA1-Like (DIA1L), lacks a signal peptide and is restricted to the genomes of the echinoderm Strongylocentrotus purpuratus and cephalochordate Branchiostoma floridae. Evidence for remarkable DIA1L gene expansion was found in B. floridae. Amino acid alignments of DIA1 family gene products revealed a potential Golgi-retention motif and a number of conserved motifs with unknown function. Furthermore, a glycine and three cysteine residues were absolutely

conserved in all DIA1-family proteins, indicating a critical role in protein structure and/or function. We have therefore identified a new metazoan protein family, the DIA1-family, and understanding the biological roles of DIA1-family members will have implications for our understanding of autism and mental retardation.
C1 [Aziz, Azhari; Harrop, Sean P.; Bishop, Naomi E.] La Trobe Univ, Dept Microbiol, Bundoora, Vic 3083, Australia.
C3 La Trobe University
RP Aziz, A (autor correspondente), La Trobe Univ, Dept Microbiol, Bundoora, Vic 3083, Australia.
EM N.Bishop@latrobe.edu.au
FU La Trobe University; Malaysian Ministry of Higher Education; Islamic Science University of Malaysia
FX NEB and SPH were supported by La Trobe University and AA by La Trobe University, the Malaysian Ministry of Higher Education, and the Islamic Science University of Malaysia. The funders had no role in study design, data collection and analysis, decision to publish, or preparation of the manuscript.
CR Adoutte A, 2000, P NATL ACAD SCI USA, V97, P4453, DOI 10.1073/pnas.97.9.4453
Altschul SF, 1997, NUCLEIC ACIDS RES, V25, P3389, DOI 10.1093/nar/25.17.3389
Anisimova M, 2006, SYST BIOL, V55, P539, DOI 10.1080/10635150600755453
[Anonymous], SULPHATION AUTISM WH
[Anonymous], BIOCH BIOPH IN PRESS
[Anonymous], NUCL ACIDS RES
[Anonymous], [No title captured]
[Anonymous], PLOS ONE IN PRESS
BAILEY A, 1995, PSYCHOL MED, V25, P63, DOI 10.1017/S0033291700028099
Bargmann CI., 1998, SCIENCE, V282, P2028, DOI [10.1126/science.282.5396.2028, DOI 10.1126/science.282.5396.2028]
Bendtsen JD, 2004, J MOL BIOL, V340, P783, DOI 10.1016/j.jmb.2004.05.028
Best J D, 2008, Neuropsychiatr Dis Treat, V4, P567
Birney E, 2004, GENOME RES, V14, P925, DOI 10.1101/gr.1860604
Bitto E, 2008, J BIOL CHEM, V283, P30184, DOI 10.1074/jbc.M804746200
Blomme T, 2006, GENOME BIOL, V7, DOI 10.1186/gb-2006-7-5-r43
Brockie PJ, 2001, NEURON, V31, P617, DOI 10.1016/S0896-6273(01)00394-4
Brockie PJ, 2001, J NEUROSCI, V21, P1510, DOI 10.1523/JNEUROSCI.21-05-01510.2001
Burbach JPH, 2009, TRENDS NEUROSCI, V32, P69, DOI 10.1016/j.tins.2008.11.002
Castagnola M, 2008, J PROTEOME RES, V7, P5327, DOI 10.1021/pr8004088
Castermans D, 2010, HUM MOL GENET, V19, P1368, DOI 10.1093/hmg/ddq013
Castresana J, 2000, MOL BIOL EVOL, V17, P540, DOI 10.1093/oxfordjournals.molbev.a026334
Chapleau CA, 2009, J NEURODEV DISORD, V1, P185, DOI 10.1007/s11689-009-9027-6
Cheung C, 2010, PLOS ONE, V5, DOI 10.1371/journal.pone.0012233
Coghlan A, 2002, GENOME RES, V12, P857, DOI 10.1101/gr.172702

Combet C, 2000, TRENDS BIOCHEM SCI, V25, P147, DOI 10.1016/S0968-0004(99)01540-6
Conant GC, 2008, NAT REV GENET, V9, P938, DOI 10.1038/nrg2482
Conant GC, 2003, GENOME RES, V13, P2052, DOI 10.1101/gr.1252603
Cossins AR, 2005, NAT REV GENET, V6, P324, DOI 10.1038/nrg1590
Crespi B, 2010, P NATL ACAD SCI USA, V107, P1736, DOI 10.1073/pnas.0906080106
D'Aniello S, 2008, MOL BIOL EVOL, V25, P1841, DOI 10.1093/molbev/msn132
D'Elia D, 2009, BMC BIOINFORMATICS, V10, DOI 10.1186/1471-2105-10-S6-S1
Dehal P, 2005, PLOS BIOL, V3, P1700, DOI 10.1371/journal.pbio.0030314
Dereeper A, 2008, NUCLEIC ACIDS RES, V36, pW465, DOI 10.1093/nar/gkn180
Douard V, 2008, BMC EVOL BIOL, V8, DOI 10.1186/1471-2148-8-336
Duan J, 2010, NUCLEIC ACIDS RES, V38, pD453, DOI 10.1093/nar/gkp801
Durand CM, 2007, NAT GENET, V39, P25, DOI 10.1038/ng1933
Eisen GE, 2009, DEV BIOL, V335, P78, DOI 10.1016/j.ydbio.2009.08.024
Emanuelsson O, 2007, NAT PROTOC, V2, P953, DOI 10.1038/nprot.2007.131
FELSENSTEIN J, 1985, EVOLUTION, V39, P783, DOI 10.1111/j.1558-5646.1985.tb00420.x
Feng JN, 2006, NEUROSCI LETT, V409, P10, DOI 10.1016/j.neulet.2006.08.017
Finn RN, 2007, PLOS ONE, V2, DOI 10.1371/journal.pone.0000169
Flicek P, 2010, NUCLEIC ACIDS RES, V38, pD557, DOI 10.1093/nar/gkp972
Flint J, 2008, CURR OPIN GENET DEV, V18, P235, DOI 10.1016/j.gde.2008.07.002
Folstein SE, 2001, NAT REV GENET, V2, P943, DOI 10.1038/35103559
Fontanillas E, 2007, BMC EVOL BIOL, V7, DOI 10.1186/1471-2148-7-95
Freitag CM, 2007, MOL PSYCHIATR, V12, P2, DOI 10.1038/sj.mp.4001896
Froschauer A, 2006, CURR GENOMICS, V7, P43, DOI 10.2174/138920206776389766
Fukuda M, 1999, J BIOL CHEM, V274, P31421, DOI 10.1074/jbc.274.44.31421
Furutani-Seiki M, 2004, MECH DEVELOP, V121, P629, DOI 10.1016/j.mod.2004.05.010
Galliot B, 2009, DEV BIOL, V332, P2, DOI 10.1016/j.ydbio.2009.05.563
Gerlach Daniel, 2007, In Silico Biology, V7, P151
Giannandrea M, 2010, AM J HUM GENET, V86, P185, DOI 10.1016/j.ajhg.2010.01.011
Glick BS, 2009, ANNU REV CELL DEV BI, V25, P113, DOI 10.1146/annurev.cellbio.24.110707.175421
Gould CM, 2010, NUCLEIC ACIDS RES, V38, pD167, DOI 10.1093/nar/gkp1016
Grimmelikhuijzen C.J.P., 1995, Experientia Supplementum (Basel), V72, P7
Guindon S, 2003, SYST BIOL, V52, P696, DOI 10.1080/10635150390235520
Guindon S, 2010, SYST BIOL, V59, P307, DOI 10.1093/sysbio/syq010
Halanych KM, 2001, AM ZOOL, V41, P629, DOI 10.1668/0003-1569(2001)041[0629:ABROMP]2.0.CO;2
Hardham AR, 2008, BMC PLANT BIOL, V8, DOI 10.1186/1471-2229-8-63
Harper RA, 1996, TRENDS BIOCHEM SCI, V21, P150, DOI 10.1016/0968-0004(96)30012-1
Hoegg S, 2007, BMC GENOMICS, V8, DOI 10.1186/1471-2164-8-317
Hofmann K., 1993, Biol. Chem. Hoppe-Seyler, V374, P166

Holland LZ, 2008, BRAIN BEHAV EVOLUT, V72, P91, DOI 10.1159/000151470
Holland LZ, 2008, GENOME RES, V18, P1100, DOI 10.1101/gr.073676.107
Holland LZ, 2004, CELL MOL LIFE SCI, V61, P2290, DOI 10.1007/s00018-004-4075-2
Holland LZ, 2003, BIOESSAYS, V25, P529, DOI 10.1002/bies.10302
Horton P, 1997, ISMB-97 - FIFTH INTERNATIONAL CONFERENCE ON INTELLIGENT SYSTEMS FOR MOLECULAR BIOLOGY, PROCEEDINGS, P147
Huang SF, 2008, GENOME RES, V18, P1112, DOI 10.1101/gr.069674.107
Huelsenbeck JP, 2001, BIOINFORMATICS, V17, P754, DOI 10.1093/bioinformatics/17.8.754
Hufton AL, 2008, GENOME RES, V18, P1582, DOI 10.1101/gr.080119.108
Jamain S, 2003, NAT GENET, V34, P27, DOI 10.1038/ng1136
Jamain S, 2002, MOL PSYCHIATR, V7, P302, DOI 10.1038/sj.mp.4000979
Kanehisa M, 1997, TRENDS BIOCHEM SCI, V22, P442, DOI 10.1016/S0968-0004(97)01130-4
Kasahara M, 2007, NATURE, V447, P714, DOI 10.1038/nature05846
Kasahara M, 2007, CURR OPIN IMMUNOL, V19, P547, DOI 10.1016/j.coi.2007.07.009
Kawano T, 1997, BIOSCI BIOTECH BIOCH, V61, P927, DOI 10.1271/bbb.61.927
Kiontke K, 2004, P NATL ACAD SCI USA, V101, P9003, DOI 10.1073/pnas.0403094101
Klee EW, 2005, BMC BIOINFORMATICS, V6, DOI 10.1186/1471-2105-6-256
Kortschak RD, 2003, CURR BIOL, V13, P2190, DOI 10.1016/j.cub.2003.11.030
Kumar RA, 2009, CURR NEUROL NEUROSCI, V9, P188, DOI 10.1007/s11910-009-0029-2
Kuraku S, 2009, MOL BIOL EVOL, V26, P47, DOI 10.1093/molbev/msn222
Kurosawa G, 1999, BIOCHEM BIOPH RES CO, V260, P66, DOI 10.1006/bbrc.1999.0834
Laviola G, 2009, NEUROSCI BIOBEHAV R, V33, P560, DOI 10.1016/j.neubiorev.2008.09.006
Ledent V, 2001, GENOME RES, V11, P754, DOI 10.1101/gr.177001
Li C, 2005, PARASITOLOGY, V131, pS109, DOI 10.1017/S0031182005009376
Li CH, 2008, SYST BIOL, V57, P519, DOI 10.1080/10635150802206883
Li L, 2003, GENOME RES, V13, P2178, DOI 10.1101/gr.1224503
Lichtenstein P, 2010, AM J PSYCHIAT, V167, P1357, DOI 10.1176/appi.ajp.2010.10020223
Lloyd TE, 2000, NEURON, V26, P45, DOI 10.1016/S0896-6273(00)81136-8
Losh M, 2008, J NEUROPATH EXP NEUR, V67, P829, DOI 10.1097/NEN.0b013e318184482d
Ma DQ, 2005, AM J HUM GENET, V77, P377, DOI 10.1086/433195
Marchler-Bauer A, 2009, NUCLEIC ACIDS RES, V37, pD205, DOI 10.1093/nar/gkn845
Marenholz I, 2004, BIOCHEM BIOPH RES CO, V322, P1111, DOI 10.1016/j.bbrc.2004.07.096
Markowitz VM, 2010, NUCLEIC ACIDS RES, V38, pD382, DOI 10.1093/nar/gkp887
Marques AC, 2008, GENOME BIOL, V9, DOI 10.1186/gb-2008-9-3-r54
Marshall CR, 2008, AM J HUM GENET, V82, P477, DOI 10.1016/j.ajhg.2007.12.009
Mathur P, 2010, NEUROBIOL DIS, V40, P66, DOI 10.1016/j.nbd.2010.05.016
McBride SMJ, 2005, NEURON, V45, P753, DOI 10.1016/j.neuron.2005.01.038
McCall KA, 2000, J NUTR, V130, p1437S, DOI 10.1093/jn/130.5.1437S

Menne KML, 2000, BIOINFORMATICS, V16, P741, DOI 10.1093/bioinformatics/16.8.741
Meyer A, 2005, BIOESSAYS, V27, P937, DOI 10.1002/bies.20293
Miller G, 2009, SCIENCE, V325, P24, DOI 10.1126/science.325_24
MILPETZ F, 1995, TRENDS BIOCHEM SCI, V20, P204, DOI 10.1016/S0968-0004(00)89009-X
Mitreva M, 2005, TRENDS GENET, V21, P573, DOI 10.1016/j.tig.2005.08.003
Morris JA, 2009, PROG BRAIN RES, V179, P97, DOI 10.1016/S0079-6123(09)17911-6
Morrow EM, 2008, SCIENCE, V321, P218, DOI 10.1126/science.1157657
Moy SS, 2008, MOL PSYCHIATR, V13, P4, DOI 10.1038/sj.mp.4002082
Mushegian AR, 1998, GENOME RES, V8, P590, DOI 10.1101/gr.8.6.590
Nesselhut J, 2001, FEBS LETT, V509, P469, DOI 10.1016/S0014-5793(01)03187-8
Newman M, 2007, BBA-MOL BASIS DIS, V1772, P285, DOI 10.1016/j.bbadis.2006.12.001
O'Meara BC, 2006, EVOLUTION, V60, P922
Oota S, 1999, MOL BIOL EVOL, V16, P856, DOI 10.1093/oxfordjournals.molbev.a026170
Parkinson J, 2004, NAT GENET, V36, P1259, DOI 10.1038/ng1472
Paxton John R., 1998, Encyclopedia of Fishes
Permanyer J, 2006, INT J BIOL SCI, V2, P48
Pinto D, 2010, NATURE, V466, P368, DOI 10.1038/nature09146
Ponting CP, 2008, NAT REV GENET, V9, P689, DOI 10.1038/nrg2413
Pruitt KD, 2007, NUCLEIC ACIDS RES, V35, pD61, DOI 10.1093/nar/gkl842
Putnam NH, 2008, NATURE, V453, P1064, DOI 10.1038/nature06967
Qian WF, 2009, GENOME BIOL EVOL, V1, P198, DOI 10.1093/gbe/evp021
Rapoport J, 2009, J AM ACAD CHILD PSY, V48, P10, DOI 10.1097/CHI.0b013e31818b1c63
Rice P, 2000, TRENDS GENET, V16, P276, DOI 10.1016/S0168-9525(00)02024-2
Ronald A, 2006, J AM ACAD CHILD PSY, V45, P691, DOI 10.1097/01.chi.0000215325.13058.9d
Rosso L, 2008, PLOS BIOL, V6, P1281, DOI 10.1371/journal.pbio.0060140
Rupp B, 1996, ANAT EMBRYOL, V194, P187
Rutter M, 2000, J ABNORM CHILD PSYCH, V28, P3, DOI 10.1023/A:1005113900068
Sadakata T, 2007, J CLIN INVEST, V117, P931, DOI 10.1172/JCI29031
Sadakata T, 2009, CEREBELLUM, V8, P312, DOI 10.1007/s12311-009-0097-5
SAITOU N, 1987, MOL BIOL EVOL, V4, P406, DOI 10.1093/oxfordjournals.molbev.a040454
Satoh N, 2006, GENOME DYN, V2, P198, DOI 10.1159/000095105
Sawa A, 2009, PROG BRAIN RES, V179, pVII, DOI 10.1016/S0079-6123(09)17919-0
Schiöth HB, 2005, ANN NY ACAD SCI, V1040, P460, DOI 10.1196/annals.1327.090
Schubert M, 2008, DEV GENES EVOL, V218, P651, DOI 10.1007/s00427-008-0251-y
Sebat J, 2007, SCIENCE, V316, P445, DOI 10.1126/science.1138659
Sebat J, 2009, TRENDS GENET, V25, P528, DOI 10.1016/j.tig.2009.10.004
Sodergren E, 2006, SCIENCE, V314, P941, DOI 10.1126/science.1133609
Srivastava M, 2008, NATURE, V454, P955, DOI 10.1038/nature07191
Takatalo MS, 2009, J DENT RES, V88, P534, DOI 10.1177/0022034509338452

Takatalo M, 2008, DEV DYNAM, V237, P2243, DOI 10.1002/dvdy.21646
Takatalo MS, 2006, PROTEOMICS, V6, P3502, DOI 10.1002/pmic.200500516
Talavera G, 2007, SYST BIOL, V56, P564, DOI 10.1080/10635150701472164
Talebizadeh Z, 2006, J MED GENET, V43, DOI 10.1136/jmg.2005.036897
Tamura K, 2013, MOL BIOL EVOL, V30, P2725, DOI [10.1093/molbev/mst197, 10.1093/molbev/msr121]
Taniai H, 2008, AM J MED GENET B, V147B, P844, DOI 10.1002/ajmg.b.30740
Taylor JS, 2004, ANNU REV GENET, V38, P615, DOI 10.1146/annurev.genet.38.072902.092831
THOMPSON JD, 1994, NUCLEIC ACIDS RES, V22, P4673, DOI 10.1093/nar/22.22.4673
Tordjman S, 2007, BEHAV GENET, V37, P61, DOI 10.1007/s10519-006-9120-5
Tropepe V, 2003, GENES BRAIN BEHAV, V2, P268, DOI 10.1034/j.1601-183X.2003.00038.x
Tusnády GE, 2001, BIOINFORMATICS, V17, P849, DOI 10.1093/bioinformatics/17.9.849
Van de Peer Y, 2001, J MOL EVOL, V53, P436, DOI 10.1007/s002390010233
Van de Peer Y, 2009, NAT REV GENET, V10, P725, DOI 10.1038/nrg2600
van der Zwaag B, 2009, PLOS ONE, V4, DOI 10.1371/journal.pone.0005324
VANEGAS H, 1983, BRAIN RES REV, V6, P117, DOI 10.1016/0165-0173(83)90036-X
Veenstra-VanderWeele J, 2004, MOL PSYCHIATR, V9, P819, DOI 10.1038/sj.mp.4001505
Veldman MB, 2008, PEDIATR RES, V64, P470, DOI 10.1203/PDR.0b013e318186e609
VONHEIJNE G, 1986, NUCLEIC ACIDS RES, V14, P4683, DOI 10.1093/nar/14.11.4683
Wägele JW, 2007, BMC EVOL BIOL, V7, DOI 10.1186/1471-2148-7-147
Wang YF, 2000, J MOL EVOL, V51, P88, DOI 10.1007/s002390010069
Weiss LA, 2009, EXPERT REV MOL DIAGN, V9, P795, DOI 10.1586/ERM.09.59
Werren JH, 2010, SCIENCE, V327, P343, DOI 10.1126/science.1178028
Wilkins MR, 1999, METH MOL B, V112, P531
WINNEPENNINCKX B, 1995, MOL BIOL EVOL, V12, P641
Wood JD, 2009, HUM MOL GENET, V18, P391, DOI 10.1093/hmg/ddn361
WULLIMANN MF, 1991, J COMP NEUROL, V312, P415, DOI 10.1002/cne.903120309
Xue HG, 2005, NEUROSCI LETT, V390, P104, DOI 10.1016/j.neulet.2005.08.007
Yoshihara Motojiro, 2001, Functional and Integrative Genomics, V1, P235, DOI 10.1007/s101420000029
Zhang JZ, 2003, TRENDS ECOL EVOL, V18, P292, DOI 10.1016/S0169-5347(03)00033-8
Zhao X, 2007, P NATL ACAD SCI USA, V104, P12831, DOI 10.1073/pnas.0705803104
ZIMMER DB, 1995, BRAIN RES BULL, V37, P417, DOI 10.1016/0361-9230(95)00040-2
ZUCKERKANDL EMILE, 1965, P97
NR 170
TC 19
Z9 21
U1 0
U2 10
PU PUBLIC LIBRARY SCIENCE

PI SAN FRANCISCO
PA 1160 BATTERY STREET, STE 100, SAN FRANCISCO, CA 94111 USA
SN 1932-6203
J9 PLOS ONE
JI PLoS One
PD JAN 19
PY 2011
VL 6
IS 1
AR e14547
DI 10.1371/journal.pone.0014547
PG 22
WC Multidisciplinary Sciences
WE Science Citation Index Expanded (SCI-EXPANDED)
SC Science & Technology - Other Topics
GA 710OH
UT WOS:000286520600002
PM 21283809
OA Green Published, gold, Green Submitted
DA 2025-05-07
ER

PT J
AU Straub, RH
   Bijlsma, JWJ
   Masi, A
   Cutolo, M
AF Straub, Rainer H.
   Bijlsma, Johannes W. J.
   Masi, Alfonse
   Cutolo, Maurizio
TI Role of neuroendocrine and neuroimmune mechanisms in chronic
   inflammatory rheumatic diseases-The 10-year update
SO SEMINARS IN ARTHRITIS AND RHEUMATISM
LA English
DT Review
DE Neuroendocrine immunology; Hormones; Neurotransmitters; Nervous system;
   Rheumatic diseases
ID REGULATORY T-CELLS; TUMOR-NECROSIS-FACTOR;
   VASOACTIVE-INTESTINAL-PEPTIDE; COLLAGEN-INDUCED ARTHRITIS;
   SYSTEMIC-LUPUS-ERYTHEMATOSUS; SYMPATHETIC-NERVOUS-SYSTEM;
   PITUITARY-ADRENAL AXIS; CORTICOTROPIN-RELEASING HORMONE; EXPERIMENTAL
   AUTOIMMUNE ENCEPHALOMYELITIS; NICOTINIC ACETYLCHOLINE-RECEPTOR
AB Background: Neuroendocrine immunology in musculoskeletal diseases is an emerging scientific field. It deals with the aspects of efferent neuronal and neurohormonal bearing on the peripheral immune and musculoskeletal systems. This review aims to add new information that appeared since 2001.
   Search strategy: The following PubMed search sentence was used to find a total of 15,462 references between 2001 and March 2013: "(rheum* OR SLE OR vasculitis) AND (nerve OR hormone OR neurotransmitter OR neuropeptide OR steroid)." In a continuous process, year by year, this search strategy

yielded relevant papers that were screened and collected in a database, which build the platform of this review.
Results: The main findings are the anti-inflammatory role of androgens, the loss of androgens (androgen drain), the bimodal role of estrogens (support B cells and inhibit macrophages and T cells), increased conversion of androgens to estrogens in inflammation (androgen drain), disturbances of the gonadal axis, inadequate amount of HPA axis hormones relative to inflammation (disproportion principle), biologics partly improve neuroendocrine axes, anti-corticotropin-releasing hormone therapies improve inflammation (antalarmin), bimodal role of the sympathetic nervous system (proinflammatory early, anti-inflammatory late most probably due to catecholamine-producing local cells), anti-inflammatory role of alpha melanocyte-stimulating hormone, vasoactive intestinal peptide, and the Vagus nerve via alpha 7 nicotinergic receptors. Circadian rhythms of hypothalamic origin are responsible for circadian rhythms of symptoms (neuroimmune link revealed). Important new pain-sensitizing immunological pathways were found in the last decade.
Conclusions: The last decade brought much new information that gave birth to the first therapies of chronic inflammatory diseases on the basis of neuroendocrine immune targets. In addition, a new theory linked evolutionary medicine, neuroendocrine regulation of distribution of energy-rich fuels, and volume regulation that can explain many disease sequelae in patients with chronic inflammatory diseases. (C) 2013 Elsevier Inc. All rights reserved.
C1 [Straub, Rainer H.] Univ Hosp, Dept Internal Med 1, Div Rheumatol, Lab Expt Rheumatol & Neuroendocrinoimmunol, Regensburg, Germany.
[Bijlsma, Johannes W. J.] Univ Med Ctr, Dept Rheumatol & Clin Immunol, Utrecht, Netherlands.
[Masi, Alfonse] Univ Illinois, Coll Med, Dept Med, Div Rheumatol, Peoria, IL 61656 USA.
[Cutolo, Maurizio] Univ Genoa, Res Labs, Genoa, Italy.
[Cutolo, Maurizio] Univ Genoa, Dept Internal Med, Postgrad Sch Rheumatol, Acad Unit Clin Rheumatol, I-16126 Genoa, Italy.
C3 University of Regensburg; Utrecht University; Utrecht University Medical
Center; University of Illinois System; University of Illinois Peoria; University of Genoa; University of Genoa
RP Straub, RH (autor correspondente), Univ Hosp, Dept Internal Med 1, Div Rheumatol, Lab Expt Rheumatol & Neuroendocrinoimmunol, Regensburg, Germany.
EM rainer.straub@klinik.uni-regensburg.de
OI Straub, Rainer H/0000-0003-1165-4555
CR [Anonymous], 1938, MAYO CLIN PROC
Auriemma M, 2012, J INVEST DERMATOL, V132, P1814, DOI 10.1038/jid.2012.59
Azab NA, 2008, CLIN IMMUNOL, V127, P151, DOI 10.1016/j.clim.2007.12.010
Baeke F, 2011, J IMMUNOL, V186, P132, DOI 10.4049/jimmunol.1000695
Barros PO, 2011, J CLIN IMMUNOL, V31, P51, DOI 10.1007/s10875-010-9466-6
Bhowmick S, 2009, J LEUKOCYTE BIOL, V86, P1275, DOI 10.1189/jlb.0209107
Boettger MK, 2010, ARTHRITIS RHEUM-US, V62, P1308, DOI 10.1002/art.27380

Borovikova LV, 2000, NATURE, V405, P458, DOI 10.1038/35013070
Bosmann M, 2013, AM J PATHOL, V182, P1124, DOI 10.1016/j.ajpath.2013.01.005
Boyle DL, 2006, PLOS MED, V3, P1616, DOI 10.1371/journal.pmed.0030338
Braitch M, 2009, ACTA NEUROL SCAND, V119, P239, DOI 10.1111/j.1600-0404.2008.01090.x
Brenn D, 2007, ARTHRITIS RHEUM, V56, P351, DOI 10.1002/art.22282
Brzoska T, 2008, ENDOCR REV, V29, P581, DOI 10.1210/er.2007-0027
Buttgereit F, 2008, LANCET, V371, P205, DOI 10.1016/S0140-6736(08)60132-4
Buttgereit F, 2011, ARTHRITIS RHEUM-US, V63, P1, DOI 10.1002/art.30070
Capellino S, 2012, ARTHRITIS RHEUM-US, V64, P1110, DOI 10.1002/art.33431
Capellino S, 2010, ANN RHEUM DIS, V69, P1853, DOI 10.1136/ard.2009.119701
CARLSTEN H, 1992, CELL IMMUNOL, V144, P190, DOI 10.1016/0008-8749(92)90236-I
Castagnetta LA, 2003, J RHEUMATOL, V30, P2597
Ceriani Giuliana, 1994, Neuroimmunomodulation, V1, P28, DOI 10.1159/000097087
Chang DM, 2002, ARTHRITIS RHEUM, V46, P2924, DOI 10.1002/art.10615
Chang SH, 2010, J BIOL CHEM, V285, P38751, DOI 10.1074/jbc.C110.185777
Chen X, 2006, EUR J IMMUNOL, V36, P2139, DOI 10.1002/eji.200635873
Christianson CA, 2010, PAIN, V151, P394, DOI 10.1016/j.pain.2010.07.030
Clarke L, 2011, INT J CLIN RHEUMATOL, V6, P241
Colin EM, 2010, ARTHRITIS RHEUM-US, V62, P132, DOI 10.1002/art.25043
Cooper MS, 2003, NEW ENGL J MED, V348, P727, DOI 10.1056/NEJMra020529
CROFFORD LJ, 1993, J IMMUNOL, V151, P1587
Crofford LJ, 1997, J CLIN ENDOCR METAB, V82, P1279, DOI 10.1210/jc.82.4.1279
Cunin P, 2011, J IMMUNOL, V186, P4175, DOI 10.4049/jimmunol.1002535
Cutolo M, 2008, RHEUMATOLOGY, V47, P2, DOI 10.1093/rheumatology/ken150
Cutolo M, 2005, ANN RHEUM DIS, V64, P1109, DOI 10.1136/ard.2005.038588
Cutolo M, 2003, CLIN EXP RHEUMATOL, V21, P687
Cutolo M, 2008, ANN RHEUM DIS, V67, P905, DOI 10.1136/ard.2008.088955
Daniel C, 2008, J PHARMACOL EXP THER, V324, P23, DOI 10.1124/jpet.107.127209
Dantzer R, 2007, BRAIN BEHAV IMMUN, V21, P153, DOI 10.1016/j.bbi.2006.09.006
de Mos M, 2009, J PAIN, V10, P1161, DOI 10.1016/j.jpain.2009.04.012
De Rosa V, 2007, IMMUNITY, V26, P241, DOI 10.1016/j.immuni.2007.01.011
Dekkers JC, 2004, CLIN EXP RHEUMATOL, V22, P63
Dekkers JC, 2001, J RHEUMATOL, V28, P1496
del Rey A, 2008, ARTHRITIS RHEUM-US, V58, P3090, DOI 10.1002/art.23869
Deng J, 2012, ARTHRITIS RHEUM-US, V64, P3564, DOI 10.1002/art.34637
Deng SH, 2010, CELL IMMUNOL, V265, P105, DOI 10.1016/j.cellimm.2010.07.010
Ding WH, 2012, EUR J IMMUNOL, V42, P901, DOI 10.1002/eji.201141958
Dominguez E, 2010, J NEUROSCI, V30, P5754, DOI 10.1523/JNEUROSCI.5007-09.2010
Dulos J, 2004, ARTHRITIS RHEUM-US, V50, P3346, DOI 10.1002/art.20509
Ebbinghaus M, 2012, ANN RHEUM DIS, V71, P253, DOI 10.1136/ard.2011.150318

El Maghraoui A, 2005, CLIN RHEUMATOL, V24, P346, DOI 10.1007/s10067-004-1039-8
Elenkov IJ, 2002, ANN NY ACAD SCI, V966, P290, DOI 10.1111/j.1749-6632.2002.tb04229.x
Ernestam S, 2007, J RHEUMATOL, V34, P1451
ESTEBAN NV, 1991, J CLIN ENDOCR METAB, V72, P39, DOI 10.1210/jcem-72-1-39
Evers AWM, 2011, NAT REV RHEUMATOL, V7, P409, DOI 10.1038/nrrheum.2011.80
Fassold A, 2009, ARTHRITIS RHEUM-US, V60, P2892, DOI 10.1002/art.24860
Fernandez-Martin A, 2006, EUR J IMMUNOL, V36, P318, DOI 10.1002/eji.200535430
Fijak M, 2011, J IMMUNOL, V186, P5162, DOI 10.4049/jimmunol.1001958
Forrest CM, 2007, BRIT J CLIN PHARMACO, V64, P517, DOI 10.1111/j.1365-2125.2007.02911.x
Forsblad-d'Elia H, 2009, J CLIN ENDOCR METAB, V94, P2044, DOI 10.1210/jc.2009-0106
Ganesan K, 2012, RHEUMATOL INT, V32, P3181, DOI 10.1007/s00296-011-2146-x
Gao YJ, 2010, NEUROTHERAPEUTICS, V7, P482, DOI 10.1016/j.nurt.2010.05.016
Giltay EJ, 1998, BRIT J RHEUMATOL, V37, P705
Goldstein RS, 2007, MOL MED, V13, P210, DOI 10.2119/2006-00108.Goldstein
Gonzalez-Rey E, 2006, ARTHRITIS RHEUM-US, V54, P864, DOI 10.1002/art.21652
Gonzalez-Rey E, 2007, ANN RHEUM DIS, V66, P70, DOI 10.1136/ard.2007.078519
GORDON D, 1986, Q J MED, V60, P671
Härle P, 2008, ARTHRITIS RHEUM-US, V58, P2347, DOI 10.1002/art.23628
Hanna FS, 2007, SEMIN ARTHRITIS RHEU, V37, P56, DOI 10.1016/j.semarthrit.2006.12.008
Härle P, 2006, ANN RHEUM DIS, V65, P51, DOI 10.1136/ard.2005.038059
Härle P, 2005, ARTHRITIS RHEUM-US, V52, P1305, DOI 10.1002/art.20987
Hartkamp A, 2008, ANN RHEUM DIS, V67, P91, DOI 10.1136/ard.2007.071563
Hartkamp A, 2010, ANN RHEUM DIS, V69, P1144, DOI 10.1136/ard.2009.117036
Heijnen CJ, 1996, J NEUROIMMUNOL, V71, P223, DOI 10.1016/S0165-5728(96)00125-7
Heikkilä R, 1998, ANN RHEUM DIS, V57, P281, DOI 10.1136/ard.57.5.281
Herrada AA, 2010, J IMMUNOL, V184, P191, DOI 10.4049/jimmunol.0802886
Herrmann M, 2002, ANN NY ACAD SCI, V966, P166, DOI 10.1111/j.1749-6632.2002.tb04213.x
Huang SA, 2008, NAT CLIN PRACT ENDOC, V4, P148, DOI 10.1038/ncpendmet0727
Imrich R, 2005, ANN RHEUM DIS, V64, P202, DOI 10.1136/ard.2003.019844
Inoue K, 2004, J PHARMACOL SCI, V94, P112, DOI 10.1254/jphs.94.112
JABLONS DM, 1989, J IMMUNOL, V142, P1542
Jacobs JWG, 2006, ARTHRITIS RHEUM, V54, P1422, DOI 10.1002/art.21809
Jara LJ, 2011, CLIN REV ALLERG IMMU, V40, P50, DOI 10.1007/s12016-009-8185-3
Jeffery LE, 2009, J IMMUNOL, V183, P5458, DOI 10.4049/jimmunol.0803217
Jessop DS, 2005, RHEUMATOLOGY, V44, P1097, DOI 10.1093/rheumatology/keh644

Johnson EO, 2000, ANN NY ACAD SCI, V917, P797
Joshi S, 2011, MOL CELL BIOL, V31, P3653, DOI 10.1128/MCB.05020-11
Juarranz Y, 2005, ARTHRITIS RES THER, V7, pR1034, DOI 10.1186/ar1779
Kang SW, 2012, J IMMUNOL, V188, P5276, DOI 10.4049/jimmunol.1101211
Karlson EW, 2009, ARTHRITIS RES THER, V11, DOI 10.1186/ar2742
Katsura H, 2006, J NEUROSCI, V26, P8680, DOI 10.1523/JNEUROSCI.1771-06.2006
Kim BJ, 2010, BRAIN BEHAV IMMUN, V24, P1126, DOI 10.1016/j.bbi.2010.05.003
Kipnis J, 2004, J NEUROSCI, V24, P6133, DOI 10.1523/JNEUROSCI.0600-04.2004
Kirkwood TBL, 2000, NATURE, V408, P233, DOI 10.1038/35041682
Kirwan J.R., 2007, COCHRANE DB SYST REV, V24, P1
Köller MD, 2004, ANN RHEUM DIS, V63, P1677, DOI 10.1136/ard.2003.018325
KONTTINEN YT, 1994, ARTHRITIS RHEUM, V37, P965, DOI 10.1002/art.1780370701
Kumar K, 2012, RHEUMATOL INT, V32, P73, DOI 10.1007/s00296-010-1556-5
Lee JH, 2012, EUR J IMMUNOL, V42, P2683, DOI 10.1002/eji.201142317
Lee JH, 2011, J IMMUNOL, V187, P1778, DOI 10.4049/jimmunol.1003919
Lee YC, 2009, ARTHRITIS RHEUM, V60, P1250, DOI 10.1002/art.24468
Lélu K, 2011, J IMMUNOL, V187, P2386, DOI 10.4049/jimmunol.1101578
Li N, 2012, EUR J IMMUNOL, V42, P1140, DOI 10.1002/eji.201142088
Li QX, 2011, CELL IMMUNOL, V272, P53, DOI 10.1016/j.cellimm.2011.09.007
Lin Q, 1996, J NEUROSCI, V16, P3026
Ling Y, 2007, EUR J HAEMATOL, V79, P310, DOI 10.1111/j.1600-0609.2007.00917.x
Liu M, 2009, ACTA NEUROL SCAND, V120, P235, DOI 10.1111/j.1600-0404.2009.01158.x
Lombardi MS, 2001, J IMMUNOL, V166, P1635, DOI 10.4049/jimmunol.166.3.1635
Lorton D, 2005, J COMP NEUROL, V489, P260, DOI 10.1002/cne.20640
Lubahn CL, 2004, BRAIN BEHAV IMMUN, V18, P563, DOI 10.1016/j.bbi.2004.02.004
Luo CY, 2011, CELL MOL IMMUNOL, V8, P50, DOI 10.1038/cmi.2010.54
Man HW, 2009, J MED CHEM, V52, P1522, DOI 10.1021/jm900210d
Manni M, 2011, CYTOKINE, V55, P380, DOI 10.1016/j.cyto.2011.05.013
Mao GP, 2010, ENDOCRINOLOGY, V151, P5477, DOI 10.1210/en.2010-0426
Masi AT, 2003, J RHEUMATOL, V30, P2302
Masi AT, 1999, J RHEUMATOL, V26, P247
Matera L, 2000, J NEUROIMMUNOL, V109, P47, DOI 10.1016/S0165-5728(00)00302-7
McCann FE, 2010, ARTHRITIS RES THER, V12, DOI 10.1186/ar3041
Mei Q, 2002, DIABETES, V51, P2997, DOI 10.2337/diabetes.51.10.2997
Meyer-Hermann M, 2009, ARTHRITIS RHEUM, V60, P2585, DOI 10.1002/art.24797
Mikami N, 2012, INT IMMUNOL, V24, P681, DOI 10.1093/intimm/dxs075
Miljkovic Z, 2009, J NEUROINFLAMM, V6, DOI 10.1186/1742-2094-6-37
Miller LE, 2004, ARTHRITIS RHEUM-US, V50, P1156, DOI 10.1002/art.20110
Miller LE, 2000, FASEB J, V14, P2097, DOI 10.1096/fj.99-1082com
Milligan ED, 2009, NAT REV NEUROSCI, V10, P23, DOI 10.1038/nrn2533
Muls N, 2012, J NEUROIMMUNOL, V243, P73, DOI 10.1016/j.jneuroim.2011.12.010

Murphy EP, 2001, ARTHRITIS RHEUM-US, V44, P782, DOI 10.1002/1529-0131(200104)44:4<782::AID-ANR134>3.0.CO;2-H
Nakagome K, 2011, J IMMUNOL, V186, P5975, DOI 10.4049/jimmunol.1001274
Nakano K, 2011, J IMMUNOL, V186, P3745, DOI 10.4049/jimmunol.1002475
Nizri E, 2009, J IMMUNOL, V183, P6681, DOI 10.4049/jimmunol.0902212
Petri MA, 2004, ARTHRITIS RHEUM-US, V50, P2858, DOI 10.1002/art.20427
Plum SM, 2009, BMC MUSCULOSKEL DIS, V10, DOI 10.1186/1471-2474-10-46
Polanczyk MJ, 2004, J IMMUNOL, V173, P2227, DOI 10.4049/jimmunol.173.4.2227
Pongratz G, 2013, NAT REV RHEUMATOL, V9, P117, DOI 10.1038/nrrheum.2012.181
Pool AJ, 2004, RHEUMATOLOGY, V43, P43, DOI 10.1093/rheumatology/keg425
Prado C, 2012, J IMMUNOL, V188, P3062, DOI 10.4049/jimmunol.1103096
Prieto GA, 2006, IMMUNOLOGY, V118, P58, DOI 10.1111/j.1365-2567.2006.02339.x
Raterman HG, 2011, J RHEUMATOL, V38, P247, DOI 10.3899/jrheum.100488
Relloso M, 2012, J LEUKOCYTE BIOL, V91, P159, DOI 10.1189/jlb.1110645
RIVIER C, 1989, ENDOCRINOLOGY, V124, P2105, DOI 10.1210/endo-124-5-2105
Schett G, 2012, ARTHRITIS RHEUM-US, V64, P3156, DOI 10.1002/art.34627
Schmidt M, 2005, ARTHRITIS RES THER, V7, pR938, DOI 10.1186/ar1769
Schmidt M, 2009, ARTHRITIS RHEUM-US, V60, P2913, DOI 10.1002/art.24859
Schmidt-Wilcke T, 2011, NAT REV RHEUMATOL, V7, P518, DOI 10.1038/nrrheum.2011.98
Silva CA, 2011, RHEUMATOL INT, V31, P1037, DOI 10.1007/s00296-010-1389-2
Sluka KA, 1997, EXP BRAIN RES, V115, P15, DOI 10.1007/PL00005675
Soares PMF, 2007, ARTHRITIS RHEUM-US, V56, P2352, DOI 10.1002/art.22660
Sorkin LS, 2009, NEUROSCIENCE, V162, P462, DOI 10.1016/j.neuroscience.2009.05.008
Srinivasan V, 2008, NEUROIMMUNOMODULAT, V15, P93, DOI 10.1159/000148191
Stark K., 2013, Brain, Behavior and Immunity, V29, pS12, DOI 10.1016/j.bbi.2013.01.039
Stary G, 2011, J IMMUNOL, V186, P103, DOI 10.4049/jimmunol.1002485
Stein C, 2003, NAT MED, V9, P1003, DOI 10.1038/nm908
Stepien T, 2010, NEUROENDOCRINOL LETT, V31, P852
STERNBERG EM, 1989, P NATL ACAD SCI USA, V86, P2374, DOI 10.1073/pnas.86.7.2374
Straub R, 2011, CLIN EXP RHEUMAT S68, V29, pS15
Straub RH, 2008, GUT, V57, P911, DOI 10.1136/gut.2007.125401
Straub RH, 2010, J INTERN MED, V267, P543, DOI 10.1111/j.1365-2796.2010.02218.x
Straub RH, 2008, ARTHRITIS RHEUM, V58, P3450, DOI 10.1002/art.24030
Straub RH, 2008, ARTHRITIS RHEUM, V58, P976, DOI 10.1002/art.23385
Straub RH, 2007, ENDOCR REV, V28, P521, DOI 10.1210/er.2007-0001
Straub RH, 2007, ARTHRITIS RHEUM, V56, P399, DOI 10.1002/art.22368
Straub RH, 2006, J RHEUMATOL, V33, P1219
Straub RH, 2006, ARTHRITIS RHEUM-US, V54, P1778, DOI 10.1002/art.21826
Straub RH, 2012, J MOL MED, V90, P523, DOI 10.1007/s00109-012-0861-8
Straub RH, 2011, ARTHRITIS RHEUM-US, V63, P3234, DOI 10.1002/art.30516
Straub RH, 2011, BRAIN BEHAV IMMUN, V25, P1, DOI 10.1016/j.bbi.2010.08.002

Straub RH, 2000, FASEB J, V14, P1380, DOI 10.1096/fj.14.10.1380
Straub RH, 2003, FASEB J, V17, P2176, DOI 10.1096/fj.03-0433hyp
Straub RH, 2003, ARTHRITIS RHEUM, V48, P1504, DOI 10.1002/art.11036
Straub RH, 2002, J NEUROIMMUNOL, V126, P116, DOI 10.1016/S0165-5728(02)00047-4
Straub RH, 2002, ARTHRITIS RHEUM, V46, P654, DOI 10.1002/art.10177
Straub RH, 2001, ARTHRITIS RHEUM-US, V44, P493, DOI 10.1002/1529-0131(200103)44:3<493::AID-ANR95>3.0.CO;2-U
Suehiro RM, 2008, RHEUMATOLOGY, V47, P1692, DOI 10.1093/rheumatology/ken338
Tai P, 2008, J CELL PHYSIOL, V214, P456, DOI 10.1002/jcp.21221
Tan YV, 2009, P NATL ACAD SCI USA, V106, P2012, DOI 10.1073/pnas.0812257106
Tang J, 2009, J IMMUNOL, V182, P4624, DOI 10.4049/jimmunol.0801543
Taylor A, 2011, J TRANSPLANT, V2011, DOI 10.1155/2011/246856
Tengstrand B, 2002, RHEUMATOLOGY, V41, P285, DOI 10.1093/rheumatology/41.3.285
Tengstrand B, 2009, J RHEUMATOL, V36, P887, DOI 10.3899/jrheum.080558
Tian Y, 2012, INVEST OPHTH VIS SCI, V53, P6434, DOI 10.1167/iovs.12-10398
Tiittanen M, 2006, DIABETES, V55, P3446, DOI 10.2337/db06-0132
Tracey KJ, 2007, J CLIN INVEST, V117, P289, DOI 10.1172/JCI30555
Tsigos C, 1997, NEUROENDOCRINOLOGY, V66, P54, DOI 10.1159/000127219
Tyagi AM, 2012, PLOS ONE, V7, DOI 10.1371/journal.pone.0044552
van der Aar AMG, 2011, J ALLERGY CLIN IMMUN, V127, P1532, DOI 10.1016/j.jaci.2011.01.068
van Hamburg JP, 2012, ANN RHEUM DIS, V71, P606, DOI 10.1136/annrheumdis-2011-200424
van Maanen MA, 2009, ARTHRITIS RHEUM-US, V60, P114, DOI 10.1002/art.24177
Vanegas HN, 2001, PROG NEUROBIOL, V64, P327, DOI 10.1016/S0301-0082(00)00063-0
Villiger PM, 2010, ANN RHEUM DIS, V69, P1842, DOI 10.1136/ard.2009.127423
Wahle M, 2006, ARTHRITIS RES THER, V8, DOI 10.1186/ar2028
Waldburger JM, 2008, ARTHRITIS RHEUM, V58, P3439, DOI 10.1002/art.23987
Walker SE, 2000, RHEUM DIS CLIN N AM, V26, P713, DOI 10.1016/S0889-857X(05)70166-6
Wang C, 2009, IMMUNOLOGY, V126, P329, DOI 10.1111/j.1365-2567.2008.03051.x
Wang DW, 2010, J PHARMACOL EXP THER, V335, P553, DOI 10.1124/jpet.110.169961
Wang H, 2003, NATURE, V421, P384, DOI 10.1038/nature01339
Webster EL, 2002, J RHEUMATOL, V29, P1252
Weidler C, 2005, ARTHRITIS RHEUM-US, V52, P1721, DOI 10.1002/art.21112
Xie YH, 2009, ACTA BIOCH BIOPH SIN, V41, P781, DOI 10.1093/abbs/gmp067
Yadav M, 2008, J IMMUNOL, V180, P2772, DOI 10.4049/jimmunol.180.5.2772
Yates MA, 2010, J NEUROIMMUNOL, V220, P136, DOI 10.1016/j.jneuroim.2010.01.013
NR 197
TC 62
Z9 65
U1 0

U2 20
PU W B SAUNDERS CO-ELSEVIER INC
PI PHILADELPHIA
PA 1600 JOHN F KENNEDY BOULEVARD, STE 1800, PHILADELPHIA, PA 19103-2899
USA
SN 0049-0172
EI 1532-866X
J9 SEMIN ARTHRITIS RHEU
JI Semin. Arthritis Rheum.
PD DEC
PY 2013
VL 43
IS 3
BP 392
EP 404
DI 10.1016/j.semarthrit.2013.04.008
PG 13
WC Rheumatology
WE Science Citation Index Expanded (SCI-EXPANDED)
SC Rheumatology
GA 277DV
UT WOS:000328800000013
PM 23731531
DA 2025-05-07
ER

PT J
AU Spreadbury, I
AF Spreadbury, Ian
TI Comparison with ancestral diets suggests dense acellular carbohydrates promote an inflammatory microbiota, and may be the primary dietary cause of leptin resistance and obesity
SO DIABETES METABOLIC SYNDROME AND OBESITY-TARGETS AND THERAPY
LA English
DT Article
DE carbohydrate density; metabolic syndrome; nutrition transition; Paleolithic diet
AB A novel hypothesis of obesity is suggested by consideration of diet-related inflammation and evolutionary medicine. The obese homeostatically guard their elevated weight. In rodent models of high-fat diet-induced obesity, leptin resistance is seen initially at vagal afferents, blunting the actions of satiety mediators, then centrally, with gastrointestinal bacterial-triggered SOCS3 signaling implicated. In humans, dietary fat and fructose elevate systemic lipopolysaccharide, while dietary glucose also strongly activates SOCS3 signaling. Crucially however, in humans, low-carbohydrate diets spontaneously decrease weight in a way that low-fat diets do not. Furthermore, nutrition transition patterns and the health of those still eating diverse ancestral diets with abundant food suggest that neither glycemic index, altered fat, nor carbohydrate intake can be intrinsic causes of obesity, and that human energy homeostasis functions well without Westernized foods containing flours, sugar, and refined fats. Due to being made up of cells, virtually all "ancestral foods" have markedly lower carbohydrate densities than flour-and sugar-

containing foods, a property quite independent of glycemic index. Thus the "forgotten organ" of the gastrointestinal microbiota is a prime candidate to be influenced by evolutionarily unprecedented postprandial luminal carbohydrate concentrations. The present hypothesis suggests that in parallel with the bacterial effects of sugars on dental and periodontal health, acellular flours, sugars, and processed foods produce an inflammatory microbiota via the upper gastrointestinal tract, with fat able to effect a "double hit" by increasing systemic absorption of lipopolysaccharide. This model is consistent with a broad spectrum of reported dietary phenomena. A diet of grain-free whole foods with carbohydrate from cellular tubers, leaves, and fruits may produce a gastrointestinal microbiota consistent with our evolutionary condition, potentially explaining the exceptional macronutrient-independent metabolic health of non-Westernized populations, and the apparent efficacy of the modern " Paleolithic" diet on satiety and metabolism.
C1 [Spreadbury, Ian] Queens Univ, Gastrointestinal Dis Res Unit, Kingston, ON, Canada.
C3 Queens University - Canada
RP Spreadbury, I (autor correspondente), Kingston Gen Hosp, GIDRU Wing, 76 Stuart St, Kingston, ON K7L 2V7, Canada.
EM is15@queensu.ca
CR Accurso A, 2008, NUTR METAB, V5, DOI 10.1186/1743-7075-5-9
Aljada A, 2006, METABOLISM, V55, P1177, DOI 10.1016/j.metabol.2006.04.016
Bäckhed F, 2011, ANN NUTR METAB, V58, P44, DOI 10.1159/000328042
Baumgartner S, 2009, J PERIODONTOL, V80, P759, DOI 10.1902/jop.2009.080376
Bergheim I, 2008, J HEPATOL, V48, P983, DOI 10.1016/j.jhep.2008.01.035
BISS K, 1971, NEW ENGL J MED, V284, P694, DOI 10.1056/NEJM197104012841304
Bribiescas RG, 2001, AM J PHYS ANTHROPOL, V115, P297, DOI 10.1002/ajpa.1085
Bribiescas RG, 2006, NUTR METAB, V3, DOI 10.1186/1743-7075-3-34
Brinkworth GD, 2009, AM J CLIN NUTR, V90, P23, DOI 10.3945/ajcn.2008.27326
Bruckdorfer KR, 2008, P NUTR SOC, V67, P214, DOI 10.1017/S0029665108007052
Caesar R, 2012, GUT
Cani PD, 2008, DIABETES, V57, P1470, DOI 10.2337/db07-1403
Cani PD, 2007, DIABETES, V56, P1761, DOI 10.2337/db06-1491
CERQUEIRA MT, 1979, AM J CLIN NUTR, V32, P905, DOI 10.1093/ajcn/32.4.905
Chakravarthy MV, 2004, J APPL PHYSIOL, V96, P3, DOI 10.1152/japplphysiol.00757.2003
Cleave T. L., 1974, The Saccharine Disease: Conditions Caused by the Taking of Refined Carbohydrate Such as Sugar and White Flour
CONNOR WE, 1978, AM J CLIN NUTR, V31, P1131, DOI 10.1093/ajcn/31.7.1131
Cordain L, 2002, EUR J CLIN NUTR, V56, pS42, DOI 10.1038/sj.ejcn.1601353
De Filippo C, 2010, P NATL ACAD SCI USA, V107, P14691, DOI 10.1073/pnas.1005963107
de La Serre CB, 2010, AM J PHYSIOL-GASTR L, V299, pG440, DOI 10.1152/ajpgi.00098.2010

de Souza RJ, 2012, AM J CLIN NUTR, V95, P614, DOI 10.3945/ajcn.111.026328
Deopurkar R, 2010, DIABETES CARE, V33, P991, DOI 10.2337/dc09-1630
Diamond J, 2003, NATURE, V423, P599, DOI 10.1038/423599a
Ding SL, 2010, PLOS ONE, V5, DOI 10.1371/journal.pone.0012191
Donnison CP, 1929, LANCET, V1, P6
EATON SB, 1985, NEW ENGL J MED, V312, P283, DOI 10.1056/NEJM198501313120505
Eguchi K, 2012, CELL METAB, V15, P518, DOI 10.1016/j.cmet.2012.01.023
Erridge C, 2011, ATHEROSCLEROSIS, V216, P1, DOI 10.1016/j.atherosclerosis.2011.02.043
FINCHER GB, 1989, ANNU REV PLANT PHYS, V40, P305, DOI 10.1146/annurev.pp.40.060189.001513
Foster GD, 2010, ANN INTERN MED, V153, P147, DOI 10.7326/0003-4819-153-3-201008030-00005
Foster GD, 2003, NEW ENGL J MED, V348, P2082, DOI 10.1056/NEJMoa022207
Foster-Powell K, 2002, AM J CLIN NUTR, V76, P5, DOI 10.1093/ajcn/76.1.5
Frassetto LA, 2009, EUR J CLIN NUTR, V63, P947, DOI 10.1038/ejcn.2009.4
Gardner CD, 2007, JAMA-J AM MED ASSOC, V297, P969, DOI 10.1001/jama.297.9.969
Ghanim H, 2007, DIABETOLOGIA, V50, P278, DOI 10.1007/s00125-006-0508-9
Ghanim H, 2009, DIABETES CARE, V32, P2281, DOI 10.2337/dc09-0979
Goodson JM, 2009, J DENT RES, V88, P519, DOI 10.1177/0022034509338353
Harris KA, 2010, CURR ATHEROSCLER REP, V12, P368, DOI 10.1007/s11883-010-0136-1
Hite AH, 2011, NUTR CLIN PRACT, V26, P300, DOI 10.1177/0884533611405791
HO KJ, 1972, AM J CLIN NUTR, V25, P737
Hopping BN, 2010, J HUM NUTR DIET, V23, P110, DOI 10.1111/j.1365-277X.2010.01096.x
Hujoel P, 2009, J DENT RES, V88, P490, DOI 10.1177/0022034509337700
Jialal I, 2012, DIABETES CARE, V35, P900, DOI 10.2337/dc11-2375
JOHNSON A, 1982, HUM ECOL, V10, P167, DOI 10.1007/BF01531239
Jonsson Tommy, 2005, BMC Endocr Disord, V5, P10, DOI 10.1186/1472-6823-5-10
Jönsson T, 2010, NUTR METAB, V7, DOI 10.1186/1743-7075-7-85
Jönsson T, 2009, CARDIOVASC DIABETOL, V8, DOI 10.1186/1475-2840-8-35
Klaus HD, 2010, AM J PHYS ANTHROPOL, V141, P594, DOI 10.1002/ajpa.21179
Kuipers RS, 2010, BRIT J NUTR, V104, P1666, DOI 10.1017/S0007114510002679
Lam V, 2012, FASEB J, V26, P1727, DOI 10.1096/fj.11-197921
Lartigue de, 2011, AM J PHYSIOL-ENDOC M, V301, pE187
Lartigue de, 2012, PLOS ONE, V7
Lassenius MI, 2011, DIABETES CARE, V34, P1809, DOI 10.2337/dc10-2197
Laugerette F, 2012, AM J PHYSIOL-ENDOC M, V302, pE374, DOI 10.1152/ajpendo.00314.2011
Lee JY, 2001, J BIOL CHEM, V276, P16683, DOI 10.1074/jbc.M011695200
Lim EL, 2011, DIABETOLOGIA, V54, P2506, DOI 10.1007/s00125-011-2204-7
Lim JS, 2010, NAT REV GASTRO HEPAT, V7, P251, DOI 10.1038/nrgastro.2010.41

Lindeberg S, 1997, AM J CLIN NUTR, V66, P845, DOI 10.1093/ajcn/66.4.845
Lindeberg S, 2007, DIABETOLOGIA, V50, P1795, DOI 10.1007/s00125-007-0716-y
LINDEBERG S, 1993, J INTERN MED, V233, P269, DOI 10.1111/j.1365-2796.1993.tb00986.x
Lindeberg S, 2001, J INTERN MED, V249, P553, DOI 10.1046/j.1365-2796.2001.00845.x
LINDEBERG S, 1994, J INTERN MED, V236, P331, DOI 10.1111/j.1365-2796.1994.tb00804.x
Lindeberg S, 1997, THROMB HAEMOSTASIS, V77, P94
Lindeberg S., 2010, Food and Western Disease: Health and Nutrition from an Evolutionary Perspective, DOI DOI 10.1002/9781444317176
Lindeberg SA, 1999, METABOLISM, V48, P1216, DOI 10.1016/S0026-0495(99)90258-5
Lindeberg S, 2012, AM J HUM BIOL, V24, P110, DOI 10.1002/ajhb.22218
Lindgärde F, 2004, METABOLISM, V53, P1355, DOI 10.1016/j.metabol.2004.04.012
LITTLE MA, 1983, HUM BIOL, V55, P811
Lockhart PB, 2012, CIRCULATION, V125, P2520, DOI 10.1161/CIR.0b013e31825719f3
Lumeng CN, 2011, J CLIN INVEST, V121, P2111, DOI 10.1172/JCI57132
Lustig RH, 2012, NATURE, V482, P27, DOI 10.1038/482027a
McAuley KA, 2005, DIABETOLOGIA, V48, P8, DOI 10.1007/s00125-004-1603-4
Mekary RA, 2010, OBESITY, V18, P167, DOI 10.1038/oby.2009.170
Membrez M, 2008, FASEB J, V22, P2416, DOI 10.1096/fj.07-102723
Mente A, 2010, DIABETES CARE, V33, P1629, DOI 10.2337/dc09-1392
Mercader J, 2009, SCIENCE, V326, P1680, DOI 10.1126/science.1173966
Monte SV, 2012, SURGERY, V151, P587, DOI 10.1016/j.surg.2011.09.038
MURRAY MJ, 1980, AM J CLIN NUTR, V33, P697, DOI 10.1093/ajcn/33.3.697
Ng A, 1997, J SCI FOOD AGR, V73, P503, DOI 10.1002/(SICI)1097-0010(199704)73:4<503::AID-JSFA762>3.3.CO;2-Q
ODEA K, 1984, DIABETES, V33, P596, DOI 10.2337/diabetes.33.6.596
Österdahl M, 2008, EUR J CLIN NUTR, V62, P682, DOI 10.1038/sj.ejcn.1602790
PAGE LB, 1974, CIRCULATION, V49, P1132, DOI 10.1161/01.CIR.49.6.1132
Parker CC, 2001, J AGR FOOD CHEM, V49, P4364, DOI 10.1021/jf0104228
Pendyala S, 2012, GASTROENTEROLOGY, V142, P1100, DOI 10.1053/j.gastro.2012.01.034
Piperno DR, 2004, NATURE, V430, P670, DOI 10.1038/nature02734
Poggi M, 2007, DIABETOLOGIA, V50, P1267, DOI 10.1007/s00125-007-0654-8
Reed AS, 2010, DIABETES, V59, P894, DOI 10.2337/db09-1024
Revedin A, 2010, P NATL ACAD SCI USA, V107, P18815, DOI 10.1073/pnas.1006993107
Roman-Gutierrez AD, 2002, LEBENSM-WISS TECHNOL, V35, P730, DOI 10.1006/fstl.2002.0932
Sampey BP, 2011, OBESITY, V19, P1109, DOI 10.1038/oby.2011.18
SCHAEFER O, 1959, Can Med Assoc J, V81, P386
Schulz LO, 2006, DIABETES CARE, V29, P1866, DOI 10.2337/dc06-0138
Shapiro A, 2008, AM J PHYSIOL-REG I, V295, pR1370, DOI 10.1152/ajpregu.00195.2008
Shin SC, 2011, SCIENCE, V334, P670, DOI 10.1126/science.1212782
Smith CJ, 1996, J AM DIET ASSOC, V96, P778, DOI 10.1016/S0002-8223(96)00216-7

Spruss A, 2009, HEPATOLOGY, V50, P1094, DOI 10.1002/hep.23122
Ströhle A, 2011, NUTR RES, V31, P429, DOI 10.1016/j.nutres.2011.05.003
Ströhle A, 2010, AM J CLIN NUTR, V92, P940, DOI 10.3945/ajcn.2010.29815
Suganami T, 2007, ARTERIOSCL THROM VAS, V27, P84, DOI 10.1161/01.ATV.0000251608.09329.9a
Tanaka M, 2005, AM J HUM BIOL, V17, P696, DOI 10.1002/ajhb.20445
Thuy S, 2008, J NUTR, V138, P1452, DOI 10.1093/jn/138.8.1452
TRUSWELL AS, 1972, AM HEART J, V84, P5, DOI 10.1016/0002-8703(72)90299-2
Tsukumo DML, 2007, DIABETES, V56, P1986, DOI 10.2337/db06-1595
Turnbaugh PJ, 2009, NATURE, V457, P480, DOI 10.1038/nature07540
Vessby B, 2012, NUTR METAB CARDIOVAS, V22, P176, DOI 10.1016/j.numecd.2010.07.011
Volynets V, 2012, DIG DIS SCI
Wanders AJ, 2011, OBES REV, V12, P724, DOI 10.1111/j.1467-789X.2011.00895.x
Williams PG, 2012, NUTR REV, V70, P80, DOI 10.1111/j.1753-4887.2011.00452.x
Wilsey J, 2003, AM J PHYSIOL-REG I, V285, pR1011, DOI 10.1152/ajpregu.00193.2003
Wood N, 2003, J CLIN PERIODONTOL, V30, P321, DOI 10.1034/j.1600-051X.2003.00353.x
Zimmet PZ, 1997, J DIABETES COMPLICAT, V11, P60, DOI 10.1016/S1056-8727(96)00090-6
NR 111
TC 81
Z9 87
U1 0
U2 14
PU DOVE MEDICAL PRESS LTD
PI ALBANY
PA PO BOX 300-008, ALBANY, AUCKLAND 0752, NEW ZEALAND
SN 1178-7007
J9 DIABETES METAB SYNDR
JI Diabetes Metab. Syndr. Obes.
PY 2012
VL 5
BP 175
EP 189
DI 10.2147/DMSO.S33473
PG 15
WC Endocrinology & Metabolism
WE Emerging Sources Citation Index (ESCI)
SC Endocrinology & Metabolism
GA V06KQ
UT WOS:000213939500018
PM 22826636
OA Green Submitted, Green Published, gold
DA 2025-05-07
ER

PT J
AU Straub, RH

Cutolo, M
AF Straub, Rainer H.
Cutolo, Maurizio
TI A History of Psycho-Neuro-Endocrine Immune Interactions in Rheumatic Diseases
SO NEUROIMMUNOMODULATION
LA English
DT Review
DE Neuroendocrine immunology; Rheumatic diseases; Clinical immunology
ID SYSTEMIC-LUPUS-ERYTHEMATOSUS; VAGUS NERVE-STIMULATION; TUMOR-NECROSIS-FACTOR; COLLAGEN-INDUCED ARTHRITIS; BETA-ADRENERGIC-RECEPTORS; PITUITARY-ADRENAL AXIS; NORADRENERGIC SYMPATHETIC INNERVATION; CHOLINERGIC ANTIINFLAMMATORY PATHWAY; VASOACTIVE-INTESTINAL-PEPTIDE; INDUCED CARTILAGE DEGRADATION
AB Background: All active scientists stand on the shoulders of giants and many other more anonymous scientists, and this is not different in our field of psycho-neuro-endocrine immunology in rheumatic diseases. Too often, the modern world of publishing forgets about the collective enterprise of scientists. Some journals advise the authors to present only literature from the last decade, and it has become a natural attitude of many scientists to present only the latest publications. In order to work against this general unempirical behavior, neuroimmunomodulation devotes the 30th anniversary issue to the history of medical science in psycho-neuro-endocrine immunology. Summary: Keywords were derived from the psycho-neuro-endocrine immunology research field very well known to the authors (R.H.S. has collected a list of keywords since 1994). We screened PubMed, the Cochran Library of Medicine, Embase, Scopus database, and the ORCID database to find relevant historical literature. The Snowballing procedure helped find related work. According to the historical appearance of discoveries in the field, the order of presentation follows the subsequent scheme: (1) the sensory nervous system, (2) the sympathetic nervous system, (3) the vagus nerve, (4) steroid hormones (glucocorticoids, androgens, progesterone, estrogens, and the vitamin D hormone), (5) afferent pathways involved in fatigue, anxiety, insomnia, and depression (includes pathophysiology), and (6) evolutionary medicine and energy regulation - an umbrella theory. Key Messages: A brief history on psycho-neuro-endocrine immunology cannot address all relevant aspects of the field. The authors are aware of this shortcoming. The reader must see this review as a viewpoint through the biased eyes of the authors. Nevertheless, the text gives an overview of the history in psycho-neuro-endocrine immunology of rheumatic diseases. (c) 2024 The Author(s).Published by S. Karger AG, Basel
C1 [Straub, Rainer H.] Univ Hosp Regensburg, Dept Internal Med, Lab Expt Rheumatol & Neuroendocrine Immunol, Regensburg, Germany.
[Cutolo, Maurizio] Univ Genoa, Postgrad Sch Rheumatol, Res Lab & Acad Div Clin Rheumatol, Dept Internal Med DIMI, Genoa, Italy.
C3 University of Regensburg; University of Genoa
RP Straub, RH (autor correspondente), Univ Hosp Regensburg, Dept Internal Med, Lab Expt Rheumatol & Neuroendocrine Immunol, Regensburg, Germany.
EM rainer.straub@ukr.de
CR Aalbers MW, 2012, NEUROIMMUNOMODULAT, V19, P352, DOI 10.1159/000341402
ABE E, 1981, P NATL ACAD SCI-BIOL, V78, P4990, DOI 10.1073/pnas.78.8.4990

Abel J., 1898, Proc Am Physiol Soc, P3
Ackerknecht E H, 1974, Med Hist, V18, P1
Adlan AM, 2017, J PHYSIOL-LONDON, V595, P967, DOI 10.1113/JP272944
ALEXANDER WRM, 1950, LANCET, V258, P297
AMARA SG, 1982, NATURE, V298, P240, DOI 10.1038/298240a0
Amrani Y, 2017, ADV IMMUNOL, V136, P1, DOI 10.1016/bs.ai.2017.06.003
Avitsur R, 1999, BRAIN BEHAV IMMUN, V13, P14, DOI 10.1006/brbi.1999.0555
Axelrod J, 1969, Prog Brain Res, V31, P21
AXELROD J, 1959, J PHARMACOL EXP THER, V127, P251
BAERWALD C, 1992, J RHEUMATOL, V19, P204
Baerwald CGO, 1997, BRIT J RHEUMATOL, V36, P1262
Baerwald CGO, 1999, IMMUNOBIOLOGY, V200, P77, DOI 10.1016/S0171-2985(99)80034-5
BAKER AR, 1988, P NATL ACAD SCI USA, V85, P3294, DOI 10.1073/pnas.85.10.3294
Baker MC, 2023, ARTHRITIS RHEUMATOL, V75, P2107, DOI 10.1002/art.42637
Bao L, 2001, NEUROREPORT, V12, P3905, DOI 10.1097/00001756-200112210-00010
Barendregt PJ, 2002, ANN RHEUM DIS, V61, P232, DOI 10.1136/ard.61.3.232
BARNES PJ, 1980, NATURE, V285, P569, DOI 10.1038/285569a0
Barone L, 2008, NEUROIMMUNOMODULAT, V14, P331, DOI 10.1159/000127360
BARSHAVIT Z, 1981, CALCIFIED TISSUE INT, V33, P673, DOI 10.1007/BF02409507
Bas DB, 2012, ARTHRITIS RHEUM-US, V64, P3886, DOI 10.1002/art.37686
Bassi GS, 2018, PROG NEURO-PSYCHOPH, V84, P201, DOI 10.1016/j.pnpbp.2018.02.013
Bassi GS, 2017, BRAIN BEHAV IMMUN, V64, P330, DOI 10.1016/j.bbi.2017.04.003
Bayliss WM, 1901, J PHYSIOL-LONDON, V26, P173
BELVISI MG, 1990, J NEUROSCI METH, V32, P159, DOI 10.1016/0165-0270(90)90173-D
Benner MH., 1968, J Allergy, V41
Bernik TR, 2002, J EXP MED, V195, P781, DOI 10.1084/jem.20011714
BESEDOVSKY H, 1983, SCIENCE, V221, P564, DOI 10.1126/science.6867729
BESEDOVSKY HO, 1979, CELL IMMUNOL, V48, P346, DOI 10.1016/0008-8749(79)90129-1
BIJLSMA JWJ, 1987, ANN RHEUM DIS, V46, P777, DOI 10.1136/ard.46.10.777
BLALOCK SJ, 1989, ARTHRITIS RHEUM-US, V32, P991, DOI 10.1002/anr.1780320808
BLAND JH, 1968, ARTHRITIS RHEUM-US, V11, P72, DOI 10.1002/art.1780110110
BLUTHE RM, 1991, EUR J PHARMACOL, V209, P281, DOI 10.1016/0014-2999(91)90184-R
BLUTHE RM, 1994, CR ACAD SCI III-VIE, V317, P499
BLUTHE RM, 1992, BRAIN RES, V573, P318, DOI 10.1016/0006-8993(92)90779-9
Boettger MK, 2010, ARTHRITIS RHEUM-US, V62, P1308, DOI 10.1002/art.27380
BOGNAR IT, 1994, N-S ARCH PHARMACOL, V349, P497, DOI 10.1007/BF00169139
BOISSETPIORO MH, 1995, ARTHRITIS RHEUM, V38, P235, DOI 10.1002/art.1780380212

Bonaz B, 2016, NEUROGASTROENT MOTIL, V28, P948, DOI 10.1111/nmo.12792
Booij A, 1996, ANN RHEUM DIS, V55, P811, DOI 10.1136/ard.55.11.811
Bordin G, 2006, RHEUMATOLOGY, V45, P1578, DOI 10.1093/rheumatology/kel334
Borovikova LV, 2000, NATURE, V405, P458, DOI 10.1038/35013070
BOURNE HR, 1971, J PHARMACOL EXP THER, V178, P1
Boyle DL, 2006, PLOS MED, V3, P1616, DOI 10.1371/journal.pmed.0030338
Bratton BO, 2012, EXP PHYSIOL, V97, P1180, DOI 10.1113/expphysiol.2011.061531
Breslauer F., 1919, Chir Deut Z, V150, P50
Brinkman DJ, 2022, J NEUROINFLAMM, V19, DOI 10.1186/s12974-022-02504-z
Brock C, 2021, MEDIAT INFLAMM, V2021, DOI 10.1155/2021/9933532
Brock C., 2017, Neurogastroenterol Motil, V29
BROWN AG, 1971, J PHYSIOL-LONDON, V219, P103, DOI 10.1113/jphysiol.1971.sp009652
Bruce AN, 1910, ARCH EXP PATHOL PHAR, V63, P424
Bruce AN, 1913, Q J EXP PHYSIOL, V6, P339, DOI 10.1113/expphysiol.1913.sp000144
BRUMBAUGH PF, 1974, J BIOL CHEM, V249, P1251
BUCALA R, 1985, J CLIN ENDOCR METAB, V60, P841, DOI 10.1210/jcem-60-5-841
Butenandt A., 1935, Biol Chem, V237, P89
Buttgereit F, 2008, LANCET, V371, P205, DOI 10.1016/S0140-6736(08)60132-4
Buttgereit F, 2023, LANCET RHEUMATOL, V5, pE368, DOI 10.1016/S2665-9913(23)00159-5
Buttgereit F, 2013, ANN RHEUM DIS, V72, P204, DOI 10.1136/annrheumdis-2011-201067
Buttgereit F, 2011, ARTHRITIS RHEUM-US, V63, P1, DOI 10.1002/art.30070
Buyon JP, 2005, ANN INTERN MED, V142, P953, DOI 10.7326/0003-4819-142-12_Part_1-200506210-00004
Camoretti-Mercado B, 2019, J ALLERGY CLIN IMMUN, V144, P1166, DOI 10.1016/j.jaci.2019.07.010
Cao YT, 2020, MEDIAT INFLAMM, V2020, DOI 10.1155/2020/2076328
Capellino S, 2012, ARTHRITIS RHEUM-US, V64, P1110, DOI 10.1002/art.33431
Capellino S, 2010, ANN RHEUM DIS, V69, P1853, DOI 10.1136/ard.2009.119701
CARCASSI A, 1981, MINERVA MED, V72, P951
CARDENAS DD, 1982, NEPHRON, V30, P336, DOI 10.1159/000182512
CARLSTEN H, 1989, IMMUNOLOGY, V68, P209
CARLSTEN H, 1992, CELL IMMUNOL, V144, P190, DOI 10.1016/0008-8749(92)90236-I
CARR DJJ, 1992, CHEM IMMUNOL, V52, P84
Castagnetta LA, 2003, J RHEUMATOL, V30, P2597
CHAPEL TA, 1971, AM J OBSTET GYNECOL, V110, P366, DOI 10.1016/0002-9378(71)90730-7
CHAPMAN L F, 1960, Trans Am Neurol Assoc, V85, P42
Chick H, 1922, LANCET, V2, P7
CODERRE TJ, 1991, BRAIN RES, V544, P325, DOI 10.1016/0006-8993(91)90073-5
COFFEY RG, 1975, ENDOCR RES COMMUN, V2, P179, DOI 10.1080/07435807509053848
Connor H., 2022, Eur J Hist Med Health, V80, P3

CONOLLY ME, 1977, BRIT J PHARMACOL, V59, P17, DOI 10.1111/j.1476-5381.1977.tb06971.x
Corcoran C, 2005, NEUROIMMUNOMODULAT, V12, P307, DOI 10.1159/000087109
Covic T, 2006, J PSYCHOSOM RES, V60, P469, DOI 10.1016/j.jpsychores.2005.09.011
Creamer P, 1999, ARTHRIT CARE RES, V12, P3, DOI 10.1002/1529-0131(199902)12:1<3::AID-ART2>3.0.CO;2-K
CUCHACOVICH M, 1988, J RHEUMATOL, V15, P561
Cutolo M, 2013, CLIN EXP RHEUMATOL, V31, P498
CUTOLO M, 1991, ARTHRITIS RHEUM, V34, P1, DOI 10.1002/art.1780340102
CUTOLO M, 1992, ARTHRITIS RHEUM-US, V35, P1007, DOI 10.1002/art.1780350905
CUTOLO M, 1993, ARTHRITIS RHEUM, V36, P1087, DOI 10.1002/art.1780360809
CUTOLO M, 1988, ARTHRITIS RHEUM-US, V31, P1314, DOI 10.1002/art.1780311015
Cutolo M, 2023, NUTRIENTS, V15, DOI 10.3390/nu15224760
Cutolo M, 2023, NAT REV RHEUMATOL, V19, P265, DOI 10.1038/s41584-023-00944-2
Cutolo M, 2020, RMD OPEN, V6, DOI 10.1136/rmdopen-2020-001454
Cutolo M, 2020, NAT REV RHEUMATOL, V16, P628, DOI 10.1038/s41584-020-0503-4
Cutolo M, 2011, VITAM HORM, V86, P327, DOI 10.1016/B978-0-12-386960-9.00014-9
DAHLSTRO.AB, 1965, SCIENCE, V147, P1583, DOI 10.1126/science.147.3665.1583
Dantzer R, 2008, NAT REV NEUROSCI, V9, P46, DOI 10.1038/nrn2297
DASILVA JAP, 1993, ANN RHEUM DIS, V52, P285, DOI 10.1136/ard.52.4.285
DASILVA JAP, 1994, J RHEUMATOL, V21, P330
DASILVA JAP, 1990, J RHEUMATOL, V17, P1592
DASILVA JAP, 1993, ARTHRITIS RHEUM, V36, P1007, DOI 10.1002/art.1780360719
David K., 1935, Biol Chem, V233, P281, DOI 10.1515/bchm2.1935.233.5-6.281
DAVISON RA, 1947, J CLIN ENDOCR METAB, V7, P201, DOI 10.1210/jcem-7-3-201
Dawes JM, 2018, NEURON, V97, P806, DOI 10.1016/j.neuron.2018.01.033
DE NEEF J C, 1958, Ned Tijdschr Geneeskd, V102, P246
Dekkers JC, 2004, CLIN EXP RHEUMATOL, V22, P63
Delgado M, 2001, NAT MED, V7, P563, DOI 10.1038/87887
DELREY A, 1982, AM J PHYSIOL, V242, pR30, DOI 10.1152/ajpregu.1982.242.1.R30
DELREY A, 1981, CELL IMMUNOL, V63, P329, DOI 10.1016/0008-8749(81)90012-5
DeVellis BM, 1995, ARTHRIT CARE RES, V8, P284, DOI 10.1002/art.1790080413
DEVIS R, 1950, Ann Endocrinol (Paris), V11, P637
DICK WC, 1971, CLIN SCI, V40, P197, DOI 10.1042/cs0400197
Dietrich P, 2013, J HEPATOL, V58, P254, DOI 10.1016/j.jhep.2012.09.027
Dirmeier M, 2008, RHEUMATOLOGY, V47, P36, DOI 10.1093/rheumatology/kem301
DOLAN AL, 1995, ANN RHEUM DIS, V54, P532, DOI 10.1136/ard.54.6.532
DRASKOCI M, 1964, NATURE, V202, P408, DOI 10.1038/202408a0

Drewes AM, 2021, SCAND J RHEUMATOL, V50, P20, DOI 10.1080/03009742.2020.1764617
DUNNE JV, 1978, ARTHRITIS RHEUM-US, V21, P767, DOI 10.1002/art.1780210705
DUVIC M, 1978, ARTHRITIS RHEUM, V21, P414, DOI 10.1002/art.1780210403
Emala CW, 1997, AM J PHYSIOL-LUNG C, V272, pL644, DOI 10.1152/ajplung.1997.272.4.L644
ENGEL GL, 1959, AM J MED, V26, P899, DOI 10.1016/0002-9343(59)90212-8
Ernestam S, 2007, J RHEUMATOL, V34, P1451
EULER USV, 1956, NATURE, V177, P44, DOI 10.1038/177044b0
Exton MS, 1998, J NEUROIMMUNOL, V88, P182, DOI 10.1016/S0165-5728(98)00122-2
FALCK B, 1961, MED EXP, V5, P429
FEHER GK, 1979, ENDOKRINOLOGIE, V73, P167
FELTEN DL, 1992, EUR J CLIN INVEST, V22, P37
FELTEN DL, 1981, BRAIN RES BULL, V7, P595, DOI 10.1016/0361-9230(81)90010-1
FELTEN DL, 1985, J IMMUNOL, V135, pS755
FELTEN DL, 1987, IMMUNOL REV, V100, P225, DOI 10.1111/j.1600-065X.1987.tb00534.x
FELTEN SY, 1987, J NEUROSCI RES, V18, P37, DOI 10.1002/jnr.490180108
Ferrero S, 2010, FERTIL STERIL, V94, P2817, DOI 10.1016/j.fertnstert.2010.06.069
Filippa MG, 2022, EUR J CLIN INVEST, V52, DOI 10.1111/eci.13635
Fitzcharles MA, 2021, LANCET, V397, P2098, DOI 10.1016/S0140-6736(21)00392-5
Fleischmann R, 2004, EXPERT OPIN BIOL TH, V4, P1333, DOI 10.1517/14712598.4.8.1333
Fleshner M, 1998, J NEUROIMMUNOL, V86, P134, DOI 10.1016/S0165-5728(98)00026-5
FRIES JF, 1980, ARTHRITIS RHEUM-US, V23, P137, DOI 10.1002/art.1780230202
Fujita T, 2007, LANGENBECK ARCH SURG, V392, P127, DOI 10.1007/s00423-006-0099-4
Ganesan K, 2008, BONE, V43, P758, DOI 10.1016/j.bone.2008.05.025
Garwolinska H, 1972, Reumatologia, V10, P259
Ge ML, 2023, CELL COMMUN SIGNAL, V21, DOI 10.1186/s12964-023-01358-z
Ghia JE, 2007, AM J PHYSIOL-GASTR L, V293, pG560, DOI 10.1152/ajpgi.00098.2007
Ghilardi JR, 2012, ARTHRITIS RHEUM-US, V64, P2223, DOI 10.1002/art.34385
GIRON LT, 1980, ANN NEUROL, V8, P520, DOI 10.1002/ana.410080509
GLICK EN, 1967, BRIT MED J, V3, P26, DOI 10.1136/bmj.3.5556.26
Glisson F., 1651, TREATISE RICKETS BEI
GLYNN JJ, 1976, ANN RHEUM DIS, V35, P534, DOI 10.1136/ard.35.6.534
Goebel A, 2021, J CLIN INVEST, V131, DOI 10.1172/JCI144201
Gonzalez-Rey E, 2007, ANN RHEUM DIS, V66, P70, DOI 10.1136/ard.2007.078519
Gowers W R, 1904, Br Med J, V1, P117
GRIECO MH, 1976, J ALLERGY CLIN IMMUN, V58, P149, DOI 10.1016/0091-6749(76)90150-0
GUEST CM, 1950, JAMA-J AM MED ASSOC, V143, P338, DOI 10.1001/jama.1950.02910390010003

Günther F, 2021, Z RHEUMATOL, V80, P140, DOI 10.1007/s00393-020-00845-9
Haas S, 2010, J DERMATOL SCI, V58, P193, DOI 10.1016/j.jdermsci.2010.03.020
Hadden J W, 1970, Cell Immunol, V1, P583, DOI 10.1016/0008-8749(70)90024-9
HADDEN JW, 1971, INT ARCH ALLER A IMM, V40, P526, DOI 10.1159/000230436
Härle P, 2008, ARTHRITIS RHEUM-US, V58, P2347, DOI 10.1002/art.23628
HAMBERGER B, 1971, ACTA PHYSIOL SCAND, V82, P107, DOI 10.1111/j.1748-1716.1971.tb04947.x
HAMILTON S, 1983, J CAN ASSOC RADIOL, V34, P49
Härle P, 2006, ANN RHEUM DIS, V65, P51, DOI 10.1136/ard.2005.038059
Härle P, 2005, ARTHRITIS RHEUM-US, V52, P1305, DOI 10.1002/art.20987
Harris VM, 2016, CLIN IMMUNOL, V168, P25, DOI 10.1016/j.clim.2016.04.002
Harrison S, 2001, INT J BIOCHEM CELL B, V33, P555, DOI 10.1016/S1357-2725(01)00031-0
HART BL, 1987, VET CLIN N AM-FOOD A, V3, P383, DOI 10.1016/S0749-0720(15)31159-2
HART BL, 1988, NEUROSCI BIOBEHAV R, V12, P123, DOI 10.1016/S0149-7634(88)80004-6
Haussler M.R., 2011, Vitamin D, P137
HAUSSLER MR, 1968, J BIOL CHEM, V243, P4055
HAWLEY DJ, 1993, J RHEUMATOL, V20, P2025
HAWLEY DJ, 1988, J RHEUMATOL, V15, P932
Hazes J M, 1989, Br J Rheumatol, V28 Suppl 1, P28
Heider TR, 2004, AM SURGEON, V70, P342
HEINE B E, 1969, Acta Psychiatrica Scandinavica, V45, P307, DOI 10.1111/j.1600-0447.1969.tb07132.x
Hench P., 1938, MAYO CLIN PROC, V13, P161
HENCH PS, 1949, ANN RHEUM DIS, V8, P97, DOI 10.1136/ard.8.2.97
Hench PS, 1934, ANN INTERN MED, V7, P1278, DOI 10.7326/0003-4819-7-10-1278
HENOCH MJ, 1978, ARTHRITIS RHEUM-US, V21, P229, DOI 10.1002/art.1780210209
HERFORT RA, 1957, J AM GERIATR SOC, V5, P904, DOI 10.1111/j.1532-5415.1957.tb00490.x
Hess AF, 1925, J BIOL CHEM, V63, P305
Hess AF, 1924, J BIOL CHEM, V62, P301
Hess A, 2011, P NATL ACAD SCI USA, V108, P3731, DOI 10.1073/pnas.1011774108
HILLARP NA, 1953, ACTA PHYSIOL SCAND, V30, P55
Holmdahl R, 1989, Br J Rheumatol, V28 Suppl 1, P54
Holzmann B, 2013, AMINO ACIDS, V45, P1, DOI 10.1007/s00726-011-1161-2
Hopkins F.Gowland., 1906, Analyst, V31, p385b, DOI DOI 10.1039/AN906310385B
HOWARD RP, 1950, CAN MED ASSOC J, V63, P340
Howlett LA, 2021, EXP GERONTOL, V150, DOI 10.1016/j.exger.2021.111339
HUANG HT, 1993, ANAT EMBRYOL, V188, P363
Hughes GC, 2014, NAT REV RHEUMATOL, V10, P740, DOI 10.1038/nrrheum.2014.144
Hughes GC, 2009, ARTHRITIS RHEUM, V60, P1775, DOI 10.1002/art.24548
Huston JM, 2006, J EXP MED, V203, P1623, DOI 10.1084/jem.20052362

Hwang YG, 2015, NEUROIMMUNOMODULAT, V22, P72, DOI 10.1159/000362727
Ishigami T., 1919, AM REV TUBERC, V2, P470
ISHMAEL W K, 1949, J Okla State Med Assoc, V42, P434
JACQUELINE F, 1953, Rev Rhum Mal Osteoartic, V20, P323
JANCSO N, 1967, BRIT J PHARM CHEMOTH, V31, P138, DOI 10.1111/j.1476-5381.1967.tb01984.x
JANSSON L, 1989, J REPROD IMMUNOL, V15, P141, DOI 10.1016/0165-0378(89)90033-8
Jenei-Lanzl Z, 2015, BRAIN BEHAV IMMUN, V50, P266, DOI 10.1016/j.bbi.2015.07.020
Jenei-Lanzl Z, 2015, ANN RHEUM DIS, V74, P444, DOI 10.1136/annrheumdis-2013-203925
Jenei-Lanzl Z, 2014, ARTHRITIS RHEUMATOL, V66, P2472, DOI 10.1002/art.38695
JESSEPH JM, 1984, ANAT REC, V208, pA81
Jimenez-Andrade JM, 2012, ARTHRITIS RES THER, V14, DOI 10.1186/ar3826
JOH TH, 1973, P NATL ACAD SCI USA, V70, P2767, DOI 10.1073/pnas.70.10.2767
JOLLIFFE VA, 1995, ANN RHEUM DIS, V54, P251, DOI 10.1136/ard.54.4.251
JUNGERS P, 1982, ARTHRITIS RHEUM, V25, P454, DOI 10.1002/art.1780250415
Kane D, 2005, ANN RHEUM DIS, V64, P325, DOI 10.1136/ard.2004.022277
KELLY M, 1951, Med J Aust, V1, P859
KENT S, 1992, P NATL ACAD SCI USA, V89, P9117, DOI 10.1073/pnas.89.19.9117
Keyszer G, 2004, ANN RHEUM DIS, V63, P1349, DOI 10.1136/ard.2003.016410
Khalkhali-Ellis Z, 2000, J RHEUMATOL, V27, P1622
KIDD BL, 1989, LANCET, V2, P1128
Kirwan JR, 2007, COCHRANE DB SYST REV, DOI 10.1002/14651858.CD006356
Klatt S, 2016, BRAIN BEHAV IMMUN, V54, P201, DOI 10.1016/j.bbi.2016.02.006
KLING D H, 1950, Ann West Med Surg, V4, P378
Koeck FX, 2009, J ORTHOP RES, V27, P736, DOI 10.1002/jor.20807
Komegae EN, 2018, BRAIN BEHAV IMMUN, V73, P441, DOI 10.1016/j.bbi.2018.06.005
Konsman JP, 2000, EUR J NEUROSCI, V12, P4434, DOI 10.1046/j.0953-816X.2000.01319.x
Koopman FA, 2016, P NATL ACAD SCI USA, V113, P8284, DOI 10.1073/pnas.1605635113
Kopec JA, 2004, CAN J PUBLIC HEALTH, V95, P361, DOI 10.1007/BF03405147
KOPIN IJ, 1972, PHARMACOL REV, V24, P245
Korte SM, 2019, RHEUMATOLOGY, V58, P35, DOI 10.1093/rheumatology/kez413
Kosek E, 2016, PAIN, V157, P1382, DOI 10.1097/j.pain.0000000000000507
KOWALSKI ML, 1989, AM REV RESPIR DIS, V140, P101, DOI 10.1164/ajrccm/140.1.101
KRUPP LB, 1989, ARCH NEUROL-CHICAGO, V46, P1121, DOI 10.1001/archneur.1989.00520460115022
Kuis W, 1996, BRAIN BEHAV IMMUN, V10, P387, DOI 10.1006/brbi.1996.0034
KYLE LH, 1950, ANN INTERN MED, V32, P878, DOI 10.7326/0003-4819-32-5-878
LADURON P, 1968, NATURE, V217, P1155, DOI 10.1038/2171155a0

LAHITA RG, 1979, ARTHRITIS RHEUM, V22, P1195, DOI 10.1002/art.1780221106
LAHITA RG, 1987, ARTHRITIS RHEUM-US, V30, P241, DOI 10.1002/art.1780300301
LAM FY, 1993, NEUROSCIENCE, V52, P443, DOI 10.1016/0306-4522(93)90170-K
Langely JN., 1921, The autonomic nervous system
Lapadula G, 1997, J RHEUMATOL, V24, P747
LEDEN I, 1983, SCAND J RHEUMATOL, V12, P166, DOI 10.3109/03009748309102905
Lee JCT, 1997, J RHEUMATOL, V24, P2476
LEMBECK F, 1953, N-S ARCH EX PATH PH, V219, P197
LEVINE JD, 1988, P NATL ACAD SCI USA, V85, P4553, DOI 10.1073/pnas.85.12.4553
LEVINE JD, 1985, J IMMUNOL, V135, pS843
LEVINE JD, 1984, SCIENCE, V226, P547, DOI 10.1126/science.6208609
LEVINE JD, 1986, J RHEUMATOL, V13, P1040
Levine YA, 2014, PLOS ONE, V9, DOI 10.1371/journal.pone.0104530
Lewis T, 1936, CLIN SCI, V2, P373
Li T, 2010, J CLIN IMMUNOL, V30, P213, DOI 10.1007/s10875-009-9346-0
Liao KP, 2014, CIRC-CARDIOVASC IMAG, V7, P575, DOI 10.1161/CIRCIMAGING.114.002235
LILLY GD, 1966, ANN SURG, V163, P856, DOI 10.1097/00000658-196606000-00006
LINDVALL O, 1973, HISTOCHEMISTRY, V35, P31, DOI 10.1007/BF00303662
Lombardi MS, 1999, FASEB J, V13, P715, DOI 10.1096/fasebj.13.6.715
Lombardi MS, 2001, J IMMUNOL, V166, P1635, DOI 10.4049/jimmunol.166.3.1635
Lorton D, 1996, J NEUROIMMUNOL, V64, P103, DOI 10.1016/0165-5728(95)00150-6
LORTON D, 1990, BRAIN BEHAV IMMUN, V4, P198, DOI 10.1016/0889-1591(90)90023-J
Lorton D, 1999, BRAIN BEHAV IMMUN, V13, P315, DOI 10.1006/brbi.1999.0564
Lorton D, 2015, INT J MOL SCI, V16, P5635, DOI 10.3390/ijms16035635
Lorton D, 2013, CLIN DEV IMMUNOL, DOI 10.1155/2013/764395
Lorton D, 2009, BRAIN BEHAV IMMUN, V23, P276, DOI 10.1016/j.bbi.2008.10.004
LUNDBERG JM, 1983, P NATL ACAD SCI-BIOL, V80, P1120, DOI 10.1073/pnas.80.4.1120
LUNDBERG JM, 1989, NEUROSCIENCE, V28, P475, DOI 10.1016/0306-4522(89)90193-0
MACMANUS JP, 1969, EXP CELL RES, V58, P188, DOI 10.1016/0014-4827(69)90135-9
Maini RN, 1998, ARTHRITIS RHEUM, V41, P1552, DOI 10.1002/1529-0131(199809)41:9<1552::AID-ART5>3.0.CO;2-W
Majoie HJM, 2011, NEUROIMMUNOMODULAT, V18, P52, DOI 10.1159/000315530
Malfait AM, 1999, J IMMUNOL, V162, P6278
MAPP PI, 1994, ANN RHEUM DIS, V53, P240, DOI 10.1136/ard.53.4.240
MAPP PI, 1993, NEUROSCIENCE, V57, P1091, DOI 10.1016/0306-4522(93)90051-G
Marsal S, 2021, LANCET RHEUMATOL, V3, pE262, DOI 10.1016/S2665-9913(20)30425-2

Martelli D, 2014, AUTON NEUROSCI-BASIC, V182, P65, DOI 10.1016/j.autneu.2013.12.007
Martelli D, 2014, J PHYSIOL-LONDON, V592, P1677, DOI 10.1113/jphysiol.2013.268573
MASI AT, 1984, SEMIN ARTHRITIS RHEU, V14, P1, DOI 10.1016/0049-0172(84)90005-2
Masi AT, 1999, J RHEUMATOL, V26, P247
Mason HL, 1938, J BIOL CHEM, V124, P0459
Mays S, 2018, AM J PHYS ANTHROPOL, V167, P484, DOI 10.1002/ajpa.23646
Mazelin L, 1998, J AUTONOM NERV SYST, V73, P38, DOI 10.1016/S0165-1838(98)00113-1
MCCLARY AR, 1955, PSYCHOSOM MED, V17, P311, DOI 10.1097/00006842-195507000-00006
McCollum EV, 1922, J BIOL CHEM, V53, P293
MCDONALD DM, 1988, J NEUROCYTOL, V17, P605, DOI 10.1007/BF01260989
MCDONALD DM, 1988, AM REV RESPIR DIS, V137, P1432, DOI 10.1164/ajrccm/137.6.1432
MCDONNELL DP, 1987, SCIENCE, V235, P1214, DOI 10.1126/science.3029866
McDougall JJ, 2001, AM J PHYSIOL-REG I, V281, pR821, DOI 10.1152/ajpregu.2001.281.3.R821
MEENAN RF, 1980, ARTHRITIS RHEUM-US, V23, P146, DOI 10.1002/art.1780230203
Meeus M, 2012, SEMIN ARTHRITIS RHEU, V41, P556, DOI 10.1016/j.semarthrit.2011.08.001
Mehta PK, 2023, AM HEART J PLUS, V27, DOI 10.1016/j.ahjo.2023.100267
MELEZ KA, 1980, ARTHRITIS RHEUM-US, V23, P41, DOI 10.1002/art.1780230108
Mellanby E, 1919, LANCET, V1, P407
Meltzer SJ, 1904, J MED RES, V10, P135
MELZACK R, 1965, SCIENCE, V150, P971, DOI 10.1126/science.150.3699.971
Metalnikov S., 1926, Ann Linst Pasteur, V40, P893
Miller LE, 2000, FASEB J, V14, P2097, DOI 10.1096/fj.99-1082com
MIZOGUCHI Y, 1985, HEPATO-GASTROENTEROL, V32, P109
MOLDOFSKY H, 1970, PSYCHOSOM MED, V32, P309, DOI 10.1097/00006842-197005000-00009
MOOS RH, 1964, J PSYCHOSOM RES, V8, P17, DOI 10.1016/0022-3999(64)90018-2
Moreland LW, 2006, ARTHRIT RHEUM-ARTHR, V55, P287, DOI 10.1002/art.21838
Moreland LW, 2004, PHARMACOECONOMICS, V22, P39, DOI 10.2165/00019053-200422001-00005
Musselman DL, 2001, NEW ENGL J MED, V344, P961, DOI 10.1056/NEJM200103293441303
NAGATSU T, 1964, J BIOL CHEM, V239, P2910
Nakamura K, 1994, Ryumachi, V34, P656
NICASSIO PM, 1992, J ABNORM PSYCHOL, V101, P514, DOI 10.1037/0021-843X.101.3.514
NORMAN AW, 1982, ENDOCR REV, V3, P331, DOI 10.1210/edrv-3-4-331
NORRIS A A, 1990, Pulmonary Pharmacology, V3, P180, DOI 10.1016/0952-0600(90)90014-A
Oakes PC, 2016, CHILD NERV SYST, V32, P2309, DOI 10.1007/s00381-016-3247-3
Oakes PC, 2016, CHILD NERV SYST, V32, P2303, DOI 10.1007/s00381-016-3246-4

Oliver G, 1895, J Physiol, V18, P230
Omdal R, 2005, RHEUMATOL INT, V25, P481, DOI 10.1007/s00296-004-0463-z
Ossyssek B, 2011, J ORTHOP RES, V29, P297, DOI 10.1002/jor.21233
OTSUKA M, 1976, NATURE, V264, P83, DOI 10.1038/264083a0
PERRY F, 1989, PAIN, V39, P77, DOI 10.1016/0304-3959(89)90177-2
Petri M, 2005, NEW ENGL J MED, V353, P2550, DOI 10.1056/NEJMoa051135
Petri MA, 2004, ARTHRITIS RHEUM-US, V50, P2858, DOI 10.1002/art.20427
Petri MA, 2002, ARTHRITIS RHEUM, V46, P1820, DOI 10.1002/art.10364
PICKEL VM, 1975, J HISTOCHEM CYTOCHEM, V23, P1, DOI 10.1177/23.1.234988
Pincus T, 2015, NEUROIMMUNOMODULAT, V22, P89, DOI 10.1159/000362735
Pongratz G, 2014, ARTHRITIS RES THER, V16, DOI 10.1186/s13075-014-0504-2
Pongratz G, 2013, NAT REV RHEUMATOL, V9, P117, DOI 10.1038/nrrheum.2012.181
Poupon R, 2012, CLIN RES HEPATOL GAS, V36, pS3, DOI 10.1016/S2210-7401(12)70015-3
PROVVEDINI DM, 1983, SCIENCE, V221, P1181, DOI 10.1126/science.6310748
Rao Y, 2019, TRENDS ENDOCRIN MET, V30, P331, DOI 10.1016/j.tem.2019.03.005
REES H, 1994, NEUROREPORT, V5, P821, DOI 10.1097/00001756-199403000-00021
Robinson E T, 1977, J R Coll Gen Pract, V27, P423
ROBINSON H, 1971, J CHRON DIS, V23, P791, DOI 10.1016/0021-9681(71)90009-9
Rojas-Villarraga A, 2014, PLOS ONE, V9, DOI 10.1371/journal.pone.0104303
Rosas-Ballina M, 2008, P NATL ACAD SCI USA, V105, P11008, DOI 10.1073/pnas.0803237105
Rosas-Ballina M, 2011, SCIENCE, V334, P98, DOI 10.1126/science.1209985
Rosenheim O, 1927, BIOCHEM J, V21, P389, DOI 10.1042/bj0210389
Rosenheim O.W., 1926, J. Soc. Chem. Ind, V45, P932
Rovensky J, 2010, ANN NY ACAD SCI, V1193, P1, DOI 10.1111/j.1749-6632.2009.05292.x
Saeed RW, 2005, J EXP MED, V201, P1113, DOI 10.1084/jem.20040463
SAMBROOK P, 1992, J RHEUMATOL, V19, P357
Sanders VM., 2001, PSYCHONEUROIMMUNOLOG, V2, P161
Santiago T, 2015, NEUROIMMUNOMODULAT, V22, P57, DOI 10.1159/000362726
SARIA A, 1985, ACTA PHYSIOL SCAND, V125, P407, DOI 10.1111/j.1748-1716.1985.tb07736.x
Sarzi-Puttini P, 2024, AUTOIMMUN REV, V23, DOI 10.1016/j.autrev.2024.103516
SATO A, 1986, J PHYSIOL-LONDON, V375, P611, DOI 10.1113/jphysiol.1986.sp016136
SATO Y, 1985, J AUTONOM NERV SYST, V12, P1, DOI 10.1016/0165-1838(85)90036-0
Schaible HG, 2024, J NEUROCHEM, V168, P3644, DOI 10.1111/jnc.15742
Schaible HG, 2014, ARTHRITIS RES THER, V16, DOI 10.1186/s13075-014-0470-8
Schaible HG, 2014, AUTON NEUROSCI-BASIC, V182, P55, DOI 10.1016/j.autneu.2013.12.004
Schmidt M, 2005, ARTHRITIS RES THER, V7, pR938, DOI 10.1186/ar1769
Schwartz N, 2019, NAT REV RHEUMATOL, V15, P137, DOI 10.1038/s41584-018-0156-8

Seminog OO, 2015, AUTOIMMUNITY, V48, P125, DOI 10.3109/08916934.2014.968918
SERGEEVA V E, 1974, Bulletin of Experimental Biology and Medicine, V77, P456
SETHI S, 1990, ANN RHEUM DIS, V49, P999, DOI 10.1136/ard.49.12.999
Sherrington CS, 1915, J PHYSIOL-LONDON, V49, P331
Skare TL, 2023, MEDITERR J RHEUMATOL, V34, DOI 10.31138/mjr.20230825.dd
SLUKA KA, 1993, NEUROSCI LETT, V149, P99, DOI 10.1016/0304-3940(93)90357-Q
Smarr KL, 2000, ARTHRIT CARE RES, V13, P23, DOI 10.1002/1529-0131(200002)13:1<23::AID-ART5>3.0.CO;2-W
Smedstad LM, 1997, BRIT J RHEUMATOL, V36, P1304
SMEDSTAD LM, 1995, J RHEUMATOL, V22, P514
SMITH J W, 1969, Clinical Research, V17, P549
SMITH RD, 1979, ARTHRITIS RHEUM, V22, P1419, DOI 10.1002/art.1780221225
Snow MH, 2005, CURR OPIN RHEUMATOL, V17, P234
Söderlin MK, 2000, SCAND J RHEUMATOL, V29, P177
SOKOL WN, 1975, J ALLERGY CLIN IMMUN, V55, P310, DOI 10.1016/0091-6749(75)90003-2
SPENGLER RN, 1994, J IMMUNOL, V152, P3024
Spies CM, 2012, ARTHRITIS RES THER, V14, DOI 10.1186/ar3885
STEAD RH, 1987, P NATL ACAD SCI USA, V84, P2975, DOI 10.1073/pnas.84.9.2975
Steenbock H, 1924, Science, V60, P224, DOI 10.1126/science.60.1549.224
STEINBERG AD, 1979, ARTHRITIS RHEUM, V22, P1170, DOI 10.1002/art.1780221103
STERN R, 1977, ARTHRITIS RHEUM-US, V20, P18, DOI 10.1002/art.1780200103
Stoff E, 1989, Arthritis Care Res, V2, P49, DOI 10.1002/anr.1790020205
Straub RH, 2008, GUT, V57, P911, DOI 10.1136/gut.2007.125401
Straub RH, 2011, CLIN EXP RHEUMATOL, V29, pS23
Straub RH, 2010, J INTERN MED, V267, P543, DOI 10.1111/j.1365-2796.2010.02218.x
Straub RH, 2007, ENDOCR REV, V28, P521, DOI 10.1210/er.2007-0001
Straub RH, 2006, ARTHRITIS RHEUM-US, V54, P1778, DOI 10.1002/art.21826
Straub RH, 2020, NEUROIMMUNOMODULAT, V27, P58, DOI 10.1159/000508109
Straub RH, 2017, NAT REV RHEUMATOL, V13, P743, DOI 10.1038/nrrheum.2017.172
Straub RH, 2017, CLIN RHEUMATOL, V36, P1041, DOI 10.1007/s10067-016-3286-x
Straub RH, 2016, EVOL MED PUBLIC HLTH, P37, DOI 10.1093/emph/eow001
Straub RH, 2015, SEMIN ARTHRITIS RHEU, V45, P220, DOI 10.1016/j.semarthrit.2015.04.014
Straub RH, 2014, ARTHRITIS RES THER, V16, DOI 10.1186/ar4688
Straub RH, 2014, ARTHRITIS RES THER, V16, DOI 10.1186/ar4484
Straub RH, 2013, SEMIN ARTHRITIS RHEU, V43, P392, DOI 10.1016/j.semarthrit.2013.04.008
Straub RH, 2012, J MOL MED, V90, P523, DOI 10.1007/s00109-012-0861-8
Straub RH, 2011, ARTHRITIS RHEUM-US, V63, P3234, DOI 10.1002/art.30516
Straub RH, 2011, BRAIN BEHAV IMMUN, V25, P1, DOI 10.1016/j.bbi.2010.08.002

STRAUB RH, 1995, J NEUROIMMUNOL, V61, P53, DOI 10.1016/0165-5728(95)00073-B
Straub RH, 1996, J NEUROIMMUNOL, V71, P37, DOI 10.1016/S0165-5728(96)00126-9
Straub RH, 1997, J NEUROCHEM, V68, P1633
Straub RH, 2004, TRENDS PHARMACOL SCI, V25, P640, DOI 10.1016/j.tips.2004.10.007
Straub RH, 1998, AM J PHYSIOL-REG I, V274, pR997, DOI 10.1152/ajpregu.1998.274.4.R997
Straub RH, 2002, FASEB J, V16, P993, DOI 10.1096/fj.02-0085com
Straub RH, 2002, J NEUROIMMUNOL, V126, P116, DOI 10.1016/S0165-5728(02)00047-4
Straub RH., 2023, Early trauma as the origin of chronic inflammation-a psychoneuroimmunological perspective
Stricker S., 1876, Ber Akad Wiss Wien, V3, P173
STURGESS AD, 1984, J CLIN LAB IMMUNOL, V13, P11
SUDA T, 1970, BIOCHEMISTRY-US, V9, P4776, DOI 10.1021/bi00826a022
Suvas S, 2017, J IMMUNOL, V199, P1543, DOI 10.4049/jimmunol.1601751
Svensson CI, 2003, NEUROREPORT, V14, P1153, DOI 10.1097/00001756-200306110-00010
SZENTIVANYI A, 1968, J ALLERGY, V42, P203, DOI 10.1016/S0021-8707(68)90117-2
SZENTIVANYI ANDOR, 1958, ANN ALLERGY, V16, P143
SZENTIVANYI ANDOR, 1958, ANN ALLERGY, V16, P389
Takamine J., 1901, Am J Pharm, V73, P523
TALAL N, 1981, ARTHRITIS RHEUM, V24, P1054, DOI 10.1002/art.1780240811
Tanaka H, 1996, BRAIN RES BULL, V39, P33, DOI 10.1016/0361-9230(95)02037-3
TARKOWSKI E, 1995, STROKE, V26, P57, DOI 10.1161/01.STR.26.1.57
TARZY BJ, 1972, LANCET, V2, P501
Teymoori-Rad M, 2019, REV MED VIROL, V29, DOI 10.1002/rmv.2032
THOMPSON M, 1962, ANN RHEUM DIS, V21, P370, DOI 10.1136/ard.21.4.370
TOIVANEN P, 1967, MED PHARMACOL EXP, V17, P33
Toussirot E, 1999, ANN RHEUM DIS, V58, P481, DOI 10.1136/ard.58.8.481
TSAI HC, 1973, J BIOL CHEM, V248, P5967
TSOUKAS CD, 1984, SCIENCE, V224, P1438, DOI 10.1126/science.6427926
Tyring S, 2006, LANCET, V367, P29, DOI 10.1016/S0140-6736(05)67763-X
UENO Y, 1983, ARTHRITIS RHEUM, V26, P118, DOI 10.1002/art.1780260124
UNGAR F, 1954, J BIOL CHEM, V206, P597
van der Goes MC, 2012, ANN RHEUM DIS, V71, P1552, DOI 10.1136/annrheumdis-2011-201019
van Maanen MA, 2010, ANN RHEUM DIS, V69, P1717, DOI 10.1136/ard.2009.118554
van Maanen MA, 2009, ARTHRITIS RHEUM-US, V60, P114, DOI 10.1002/art.24177
van Vollenhoven RF, 1998, J RHEUMATOL, V25, P285
VANVOLLENHOVEN RF, 1994, ARTHRITIS RHEUM, V37, P1305, DOI 10.1002/art.1780370906
vanVollenhoven RF, 1995, ARTHRITIS RHEUM, V38, P1826, DOI 10.1002/art.1780381216
VEALE D, 1993, BRIT J RHEUMATOL, V32, P413
Velayos E E, 1972, Md State Med J, V21, P38
Venborg J, 2021, PHARMACEUTICALS-BASE, V14, DOI 10.3390/ph14111166
VON EULER U S, 1946, J Physiol, V105, P26

von Euler US, 1931, J PHYSIOL-LONDON, V72, P74, DOI 10.1113/jphysiol.1931.sp002763
VONEULER US, 1951, ACTA PHYSIOL SCAND, V24, P212
VONEULER US, 1946, NATURE, V157, P369
Wahle M, 2001, ANN RHEUM DIS, V60, P505, DOI 10.1136/ard.60.5.505
Wahle M, 2006, ARTHRITIS RES THER, V8, DOI 10.1186/ar2028
Waldburger JM, 2008, ARTHRITIS RHEUM, V58, P3439, DOI 10.1002/art.23987
WALKER SE, 1993, TRENDS ENDOCRIN MET, V4, P147, DOI 10.1016/1043-2760(93)90103-L
Wallace DJ, 1997, ANN MED, V29, P9, DOI 10.3109/07853899708998739
Wang H, 2003, NATURE, V421, P384, DOI 10.1038/nature01339
WARE JE, 1992, MED CARE, V30, P473, DOI 10.1097/00005650-199206000-00002
WATKINS LR, 1995, NEUROSCI LETT, V183, P27, DOI 10.1016/0304-3940(94)11105-R
Watkins LR, 2002, PHYSIOL REV, V82, P981, DOI 10.1152/physrev.00011.2002
Weidler C, 2005, ANN RHEUM DIS, V64, P13, DOI 10.1136/ard.2003.016154
Weidler C, 2004, J RHEUMATOL, V31, P489
WEIHE E, 1991, INT J NEUROSCI, V59, P1
Whitacre CC, 2001, NAT IMMUNOL, V2, P777, DOI 10.1038/ni0901-777
White J C., 1952, The autonomic nervous system: Anatomy, Physiology and Surgical Application, V3rd
WIDENFALK B, 1991, SCAND J PLAST RECONS, V25, P31, DOI 10.3109/02844319109034920
Willemze RA, 2018, NEUROGASTROENT MOTIL, V30, DOI 10.1111/nmo.13163
WILLIAMS LT, 1976, J CLIN INVEST, V57, P149, DOI 10.1172/JCI108254
Williams RM, 1997, NEUROIMMUNOMODULAT, V4, P266, DOI 10.1159/000097346
WILLIS WD, 1988, PROG BRAIN RES, V77, P1
Wimalawansa SJ, 2023, NUTRIENTS, V15, DOI 10.3390/nu15173842
Windaus A., 1926, NACHRICHTEN GESELLSC, P175
Wolf G, 2004, J NUTR, V134, P1299, DOI 10.1093/jn/134.6.1299
Wu PH, 2024, BRAIN BEHAV IMMUN, V117, P412, DOI 10.1016/j.bbi.2024.02.006
YAGHMAI I, 1977, AM J ROENTGENOL, V128, P299, DOI 10.2214/ajr.128.2.299
YUNUS M, 1981, SEMIN ARTHRITIS RHEU, V11, P151, DOI 10.1016/0049-0172(81)90096-2
ZAPHIROPOULOS G, 1973, ANN RHEUM DIS, V32, P593, DOI 10.1136/ard.32.6.593-b
NR 436
TC 2
Z9 2
U1 0
U2 0
PU KARGER
PI BASEL
PA ALLSCHWILERSTRASSE 10, CH-4009 BASEL, SWITZERLAND
SN 1021-7401
EI 1423-0216
J9 NEUROIMMUNOMODULAT
JI Neuroimmunomodulation
PD JAN-DEC

PY 2024
VL 31
IS 1
BP 183
EP 210
DI 10.1159/000540959
PG 28
WC Endocrinology & Metabolism; Immunology; Neurosciences
WE Science Citation Index Expanded (SCI-EXPANDED)
SC Endocrinology & Metabolism; Immunology; Neurosciences & Neurology
GA Q0A1H
UT WOS:001381412600001
PM 39168106
OA gold
DA 2025-05-07
ER

PT J
AU Cotter, MM
   Loomis, DA
   Simpson, SW
   Latimer, B
   Hernandez, CJ
AF Cotter, Meghan M.
   Loomis, David A.
   Simpson, Scott W.
   Latimer, Bruce
   Hernandez, Christopher J.
TI Human Evolution and Osteoporosis-Related Spinal Fractures
SO PLOS ONE
LA English
DT Article
ID FINITE-ELEMENT MODELS; BONE-MINERAL DENSITY; CANCELLOUS BONE;
VERTEBRAL
   BODY; TRABECULAR MICROARCHITECTURE; JOINT; AGE; RISK; SIZE; STRENGTH
AB The field of evolutionary medicine examines the possibility that some diseases are the result of trade-offs made in human evolution. Spinal fractures are the most common osteoporosis-related fracture in humans, but are not observed in apes, even in cases of severe osteopenia. In humans, the development of osteoporosis is influenced by peak bone mass and strength in early adulthood as well as age-related bone loss. Here, we examine the structural differences in the vertebral bodies (the portion of the vertebra most commonly involved in osteoporosis-related fractures) between humans and apes before age-related bone loss occurs. Vertebrae from young adult humans and chimpanzees, gorillas, orangutans, and gibbons (T8 vertebrae, n = 8-14 per species, male and female, humans: 20-40 years of age) were examined to determine bone strength (using finite element models), bone morphology (external shape), and trabecular microarchitecture (micro-computed tomography). The vertebrae of young adult humans are not as strong as those from apes after accounting for body mass (p < 0.01). Human vertebrae are larger in size (volume, cross-sectional area, height) than in apes with a similar body mass. Young adult human vertebrae have significantly lower trabecular bone volume fraction (0.26 +/- 0.04 in humans and 0.37 +/- 0.07 in apes, mean +/- SD,

$p < 0.01$) and thinner vertebral shells than apes (after accounting for body mass, $p < 0.01$). Since human vertebrae are more porous and weaker than those in apes in young adulthood (after accounting for bone mass), even modest amounts of age-related bone loss may lead to vertebral fracture in humans, while in apes, larger amounts of bone loss would be required before a vertebral fracture becomes likely. We present arguments that differences in vertebral bone size and shape associated with reduced bone strength in humans is linked to evolutionary adaptations associated with bipedalism.
C1 [Cotter, Meghan M.; Simpson, Scott W.] Case Western Reserve Univ, Sch Med, Dept Anat, Cleveland, OH 44106 USA.
[Cotter, Meghan M.; Loomis, David A.] Case Western Reserve Univ, Musculoskeletal Mech & Mat Lab, Dept Mech & Aerosp Engn, Cleveland, OH 44106 USA.
[Cotter, Meghan M.; Simpson, Scott W.; Latimer, Bruce; Hernandez, Christopher J.] Case Western Reserve Univ, Ctr Human Origins, Cleveland, OH 44106 USA.
[Latimer, Bruce] Case Western Reserve Univ, Dept Anthropol, Cleveland, OH 44106 USA.
[Hernandez, Christopher J.] Cornell Univ, Sibley Sch Mech & Aerosp Engn, Ithaca, NY 14853 USA.
C3 University System of Ohio; Case Western Reserve University; University System of Ohio; Case Western Reserve University; University System of Ohio; Case Western Reserve University; University System of Ohio; Case Western Reserve University; Cornell University
RP Cotter, MM (autor correspondente), Case Western Reserve Univ, Sch Med, Dept Anat, Cleveland, OH 44106 USA.
EM cjh275@cornell.edu
RI Simpson, Scott/GRS-0081-2022; Hernandez, Christopher/B-5290-2009
OI Hernandez, Christopher John/0000-0002-0712-6533
FU Case Western Reserve University
FX The study was funded by Case Western Reserve University. The funders had
no role in study design, data collection and analysis, decision to publish, or preparation of the manuscript.
CR Alexander C, 2001, ANN RHEUM DIS, V60, P554, DOI 10.1136/ard.60.6.554
Banse X, 2001, BONE, V28, P563, DOI 10.1016/S8756-3282(01)00425-2
BOBBERT MF, 1992, J BIOMECH, V25, P223, DOI 10.1016/0021-9290(92)90022-S
Brown KAR, 2002, J HUM EVOL, V43, P189, DOI 10.1006/jhev.2002.0568
Buckley JM, 2007, BONE, V40, P767, DOI 10.1016/j.bone.2006.10.025
Carlson K, 2008, INT J PRIMATOL, V29, P1401, DOI 10.1007/s10764-008-9297-y
Cerroni AM, 2000, AM J PHYS ANTHROPOL, V113, P389, DOI 10.1002/1096-8644(200011)113:3<389::AID-AJPA9>3.0.CO;2-I
Cooper Cyrus, 1996, P419
Cotter MM, 2009, ANAT REC, V292, P1098, DOI 10.1002/ar.20932
Cotter MM., 2011, Gross Morphology, Microarchitecture, Strength and Evolution of the Hominoid Vertebral Body
Crawford RP, 2003, BONE, V33, P744, DOI 10.1016/S8756-3282(03)00210-2
Crawford RP, 2003, J BIOMECH ENG-T ASME, V125, P434, DOI 10.1115/1.1589772
Currey J. D., 2002, Bones: Structure and Mechanics

Duan YB, 2001, J BONE MINER RES, V16, P2276, DOI 10.1359/jbmr.2001.16.12.2276
EASTELL R, 1991, J BONE MINER RES, V6, P207
Eswaran SK, 2007, BONE, V41, P733, DOI 10.1016/j.bone.2007.05.017
Fleagle J.G., 1999, PRIMATE ADAPTATION E
GARLAND T, 1994, PHYSIOL ZOOL, V67, P797, DOI 10.1086/physzool.67.4.30163866
Glantz S. A., 2001, Primer of applied regression & analysis of variance, V2nd
Gunji H, 2003, PRIMATES, V44, P145, DOI 10.1007/s10329-002-0030-8
Heaney RP, 2003, BONE, V33, P457, DOI 10.1016/S8756-3282(03)00236-9
Hernandez CJ, 2009, J HUM EVOL, V56, P462, DOI 10.1016/j.jhevol.2008.12.007
Hernandez CJ, 2003, OSTEOPOROSIS INT, V14, P843, DOI 10.1007/s00198-003-1454-8
Hildebrand TOR, 1997, Comput Methods Biomech Biomed Engin, V1, P15, DOI 10.1080/01495739708936692
Homminga J, 2001, SPINE, V26, P1555, DOI 10.1097/00007632-200107150-00010
Homminga J, 2004, BONE, V34, P510, DOI 10.1016/j.bone.2003.12.001
Hussein A.I., 2009, PREDICTIONS VERTEBRA
Ismail AA, 1999, OSTEOPOROSIS INT, V9, P206, DOI 10.1007/s001980050138
Iwamoto M, 2007, DEV BIOL, V305, P40, DOI 10.1016/j.ydbio.2007.01.037
JUNGERS WL, 1988, J HUM EVOL, V17, P247, DOI 10.1016/0047-2484(88)90056-5
Jurmain R, 2000, J HUM EVOL, V39, P185, DOI 10.1006/jhev.2000.0413
Karasik D, 2008, HUM GENET, V124, P349, DOI 10.1007/s00439-008-0559-8
Ketcham RA, 2004, J MICROSC-OXFORD, V213, P158, DOI 10.1111/j.1365-2818.2004.01277.x
KEYAK JH, 1994, J BIOMED MATER RES, V28, P1329, DOI 10.1002/jbm.820281111
Kneissel M, 1997, CALCIFIED TISSUE INT, V61, P95
KNEISSEL M, 1994, BONE, V15, P539, DOI 10.1016/8756-3282(94)90278-X
LATIMER B, 1989, AM J PHYS ANTHROPOL, V78, P369, DOI 10.1002/ajpa.1330780306
Latimer B, 2005, ANN BIOMED ENG, V33, P3, DOI 10.1007/s10439-005-8957-8
Lentle BC, 2007, CAN ASSOC RADIOL J, V58, P27
Lieberman DE, 2001, AM J PHYS ANTHROPOL, V116, P266, DOI 10.1002/ajpa.1123
Lovejoy CO, 2005, GAIT POSTURE, V21, P95, DOI 10.1016/j.gaitpost.2004.01.001
Lovell N.C., 1990, PATTERNS INJURY ILLN
Maga M, 2006, AM J PHYS ANTHROPOL, V129, P410, DOI 10.1002/ajpa.20276
Mays S, 1998, INT J OSTEOARCHAEOL, V8, P97, DOI 10.1002/(SICI)1099-1212(199803/04)8:2<97::AID-OA412>3.0.CO;2-U
MCHENRY HM, 1992, AM J PHYS ANTHROPOL, V87, P407, DOI 10.1002/ajpa.1330870404
Oxnard C.E., 1981, Symposia of the Zoological Society of London, P127
Ritchie RO, 2008, BONE, V43, P798, DOI 10.1016/j.bone.2008.04.027
Rizzoli R, 2001, J MOL ENDOCRINOL, V26, P79, DOI 10.1677/jme.0.0260079
Rizzoli R, 2010, BONE, V46, P294, DOI 10.1016/j.bone.2009.10.005
RUFF C, 1988, J HUM EVOL, V17, P687, DOI 10.1016/0047-2484(88)90025-5

Ruff C. B., 2005, Journal of Musculoskeletal & Neuronal Interactions, V5, P202
Sambrook P, 2006, LANCET, V367, P2010, DOI 10.1016/S0140-6736(06)68891-0
Settle SH, 2003, DEV BIOL, V254, P116, DOI 10.1016/S0012-1606(02)00022-2
SHAPIRO LJ, 1994, AM J PHYS ANTHROPOL, V93, P491, DOI 10.1002/ajpa.1330930408
Sokol RR., 1995, BIOMETRY
SUMNER DR, 1989, AM J PHYS ANTHROPOL, V79, P225, DOI 10.1002/ajpa.1330790210
Trevathan WR., 2008, Evolutionary Medicine and Health: New Perspectives
ZIHLMAN AL, 1990, J ZOOL, V221, P37, DOI 10.1111/j.1469-7998.1990.tb03773.x
NR 58
TC 23
Z9 25
U1 0
U2 25
PU PUBLIC LIBRARY SCIENCE
PI SAN FRANCISCO
PA 1160 BATTERY STREET, STE 100, SAN FRANCISCO, CA 94111 USA
SN 1932-6203
J9 PLOS ONE
JI PLoS One
PD OCT 19
PY 2011
VL 6
IS 10
AR e26658
DI 10.1371/journal.pone.0026658
PG 11
WC Multidisciplinary Sciences
WE Science Citation Index Expanded (SCI-EXPANDED)
SC Science & Technology - Other Topics
GA 841JB
UT WOS:000296507500104
PM 22028933
OA Green Published, Green Submitted, gold
DA 2025-05-07
ER

PT J
AU Andrés, AM
Soldevila, M
Lao, O
Volpini, V
Saitou, N
Jacobs, HT
Hayasaka, I
Calafell, F
Bertranpetit, J
AF Andrés, AM

Soldevila, M
Lao, O
Volpini, V
Saitou, N
Jacobs, HT
Hayasaka, I
Calafell, F
Bertranpetit, J
TI Comparative genetics of functional trinucleotide tandem repeats in humans and apes
SO JOURNAL OF MOLECULAR EVOLUTION
LA English
DT Article
DE spinocerebellar ataxia; trinucleotide repeat expansion; short tandem repeat evolution; primates
ID SPINOCEREBELLAR ATAXIA TYPE-1; MICROSATELLITE EVOLUTION; CAG REPEATS; POLYGLUTAMINE TRACT; BALANCING SELECTION; SEQUENCE DIVERSITY; ANDROGEN RECEPTOR; ALLELE SIZE; CGG REPEAT; LOCI
AB Several human neurodegenerative disorders are caused by the expansion of polymorphic trinucleotide repeat regions. Many of these loci are functional short tandem repeats (STRs) located in brain-expressed genes, and their study is thus relevant from both a medical and an evolutionary point of view. The aims of our study are to infer the comparative pattern of variation and evolution of this set of loci in order to show species-specific features in this group of STRs and on their potential for expansion (therefore, an insight into evolutionary medicine) and to unravel whether any human-specific feature may be identified in brain-expressed genes involved in human disease. We analyzed the variability of the normal range of seven expanding STR CAG/CTG loci (SCA1, SCA2, SCA3-MJD, SCA6, SCA8, SCA12, and DRPLA) and two nonexpanding polymorphic CAG loci (KCNN3 and NCOA3) in humans, chimpanzees, gorillas, and orangutans. The study showed a general conservation of the repetitive tract and of the polymorphism in the four species and high heterogeneity among loci distributions. Humans present slightly larger alleles than the rest of species but a more relevant difference appears in variability levels: Humans are the species with the largest variance, although only for the expanding loci, suggesting a relationship between variability levels and expansion potential. The sequence analysis shows high levels of sequence conservation among species, a lack of correspondence between interruption patterns and variability levels, and signs of conservative selective pressure for some of the STR loci. Only two loci (SCA1 and SCA8) show a human specific distribution, with larger alleles than the rest of species. This could account, at the same time, for a human-specific trait and a predisposition to disease through expansion.
C1 Univ Pompeu Fabra, Fac Ciencies Salut & Vida, Unitat Biol Evolut, Barcelona, Spain.
Canc Res Inst Barcelona, Dept Mol Genet, Barcelona, Spain.
Natl Inst Genet, Div Populat Genet, Mishima, Shizuoka 411, Japan.
Univ Tampere, Inst Med Technol, FIN-33101 Tampere, Finland.
Univ Tampere, Tampere Univ Hosp, FIN-33101 Tampere, Finland.
Kenkyusho Kumamoto Primate Pk, Sanwa Kagaku, Misumi, Japan.
C3 Pompeu Fabra University; Research Organization of Information & Systems
(ROIS); National Institute of Genetics (NIG) - Japan; Tampere

University; Tampere University; Tampere University Hospital
RP Univ Pompeu Fabra, Fac Ciencies Salut & Vida, Unitat Biol Evolut, Barcelona, Spain.
EM jaume.bertran-petit@upf.edu
RI Jacobs, Howard/G-2434-2011; Andres, Aida/B-4088-2014; Calafell, Francesc/C-8595-2014; Lao, Oscar/N-9930-2017; Bertranpetit, Jaume/F-8550-2012
OI Jacobs, Howard/0000-0003-1895-6003; Andres, Aida/0000-0002-8590-9672; Calafell, Francesc/0000-0002-1083-9438; Lao, Oscar/0000-0002-8525-9649;
Bertranpetit, Jaume/0000-0003-0100-0590
CR Albà MM, 1999, MOL BIOL EVOL, V16, P1641, DOI 10.1093/oxfordjournals.molbev.a026077
Andrés AM, 2003, HUM MUTAT, V21, P61, DOI 10.1002/humu.10151
[Anonymous], 2012, Biometry
Bamshad MJ, 2002, P NATL ACAD SCI USA, V99, P10539, DOI 10.1073/pnas.162046399
Chakraborty R, 1997, P NATL ACAD SCI USA, V94, P1041, DOI 10.1073/pnas.94.3.1041
Chen FC, 2001, AM J HUM GENET, V68, P444, DOI 10.1086/318206
Choong CS, 1998, J MOL EVOL, V47, P334, DOI 10.1007/PL00006391
Cooper G, 1998, HUM MOL GENET, V7, P1425, DOI 10.1093/hmg/7.9.1425
CrouauRoy B, 1996, HUM MOL GENET, V5, P1131, DOI 10.1093/hmg/5.8.1131
Deka R, 1999, MOL BIOL EVOL, V16, P1166, DOI 10.1093/oxfordjournals.molbev.a026207
Di Rienzo A, 1998, GENETICS, V148, P1269
Djian P, 1996, P NATL ACAD SCI USA, V93, P417, DOI 10.1073/pnas.93.1.417
Dror V, 1999, MOL PSYCHIATR, V4, P254, DOI 10.1038/sj.mp.4000508
ELLEGREN H, 1995, NAT GENET, V11, P360, DOI 10.1038/ng1295-360
Ellegren H, 1997, MOL BIOL EVOL, V14, P854, DOI 10.1093/oxfordjournals.molbev.a025826
Enard W, 2002, NATURE, V418, P869, DOI 10.1038/nature01025
Figueroa KP, 2001, ARCH NEUROL-CHICAGO, V58, P1649, DOI 10.1001/archneur.58.10.1649
FU YH, 1991, CELL, V67, P1047, DOI 10.1016/0092-8674(91)90283-5
Gagneux P, 2001, MOL PHYLOGENET EVOL, V18, P2, DOI 10.1006/mpev.2000.0799
GARZA JC, 1995, MOL BIOL EVOL, V12, P594
González-Cabo P, 1999, MOL BIOL EVOL, V16, P880, DOI 10.1093/oxfordjournals.molbev.a026173
Hacia JG, 2001, TRENDS GENET, V17, P637, DOI 10.1016/S0168-9525(01)02494-5
HEDRICK PW, 1983, GENETICS, V104, P449
HUGHES AL, 1988, NATURE, V335, P167, DOI 10.1038/335167a0
Ingman M, 2000, NATURE, V408, P708, DOI 10.1038/35047064
Jodice C, 1997, ANN HUM GENET, V61, P425, DOI 10.1046/j.1469-1809.1997.6150425.x
Jorde LB, 2000, AM J HUM GENET, V66, P979, DOI 10.1086/302825
Justice CM, 2001, J MOL EVOL, V52, P232, DOI 10.1007/s002390010151
Kaessmann H, 1999, SCIENCE, V286, P1159, DOI 10.1126/science.286.5442.1159
Kaessmann H, 2001, NAT GENET, V27, P155, DOI 10.1038/84773
Kawasaki T, 2001, PAC REV, V14, P221

Koob MD, 1999, NAT GENET, V21, P379, DOI 10.1038/7710
Lai CSL, 2001, NATURE, V413, P519, DOI 10.1038/35097076
Limprasert P, 1997, AM J MED GENET, V74, P488, DOI 10.1002/(SICI)1096-8628(19970919)74:5<488::AID-AJMG6>3.0.CO;2-K
Limprasert P, 1996, HUM MOL GENET, V5, P207, DOI 10.1093/hmg/5.2.207
Messler W, 1997, NATURE, V385, P151, DOI 10.1038/385151a0
NAGAFUCHI S, 1994, NAT GENET, V6, P14, DOI 10.1038/ng0194-14
Noda R, 2001, J HERED, V92, P490, DOI 10.1093/jhered/92.6.490
Nolin SL, 2003, AM J HUM GENET, V72, P454, DOI 10.1086/367713
ORR HT, 1993, NAT GENET, V4, P221, DOI 10.1038/ng0793-221
Pulst SM, 1996, NAT GENET, V14, P269, DOI 10.1038/ng1196-269
RUBINSZTEIN DC, 1995, HUM MOL GENET, V4, P1585, DOI 10.1093/hmg/4.9.1585
RUBINSZTEIN DC, 1995, NAT GENET, V10, P337, DOI 10.1038/ng0795-337
Ruvolo M, 1997, MOL BIOL EVOL, V14, P248, DOI 10.1093/oxfordjournals.molbev.a025761
Saleem Q, 2001, HUM GENET, V109, P136, DOI 10.1007/s004390100550
Schneider S., 2000, SOFTWARE POPULATION
WATKINS WS, 1995, HUM MOL GENET, V4, P1485, DOI 10.1093/hmg/4.9.1485
Webster MT, 2002, P NATL ACAD SCI USA, V99, P8748, DOI 10.1073/pnas.122067599
Wise CA, 1997, MOL BIOL EVOL, V14, P707, DOI 10.1093/oxfordjournals.molbev.a025810
Yue S, 2001, HUM MOL GENET, V10, P25, DOI 10.1093/hmg/10.1.25
Zhuchenko O, 1997, NAT GENET, V15, P62, DOI 10.1038/ng0197-62
NR 51
TC 22
Z9 27
U1 0
U2 6
PU SPRINGER
PI NEW YORK
PA 233 SPRING ST, NEW YORK, NY 10013 USA
SN 0022-2844
EI 1432-1432
J9 J MOL EVOL
JI J. Mol. Evol.
PD SEP
PY 2004
VL 59
IS 3
BP 329
EP 339
DI 10.1007/s00239-004-2628-5
PG 11
WC Biochemistry & Molecular Biology; Evolutionary Biology; Genetics & Heredity
WE Science Citation Index Expanded (SCI-EXPANDED)
SC Biochemistry & Molecular Biology; Evolutionary Biology; Genetics & Heredity
GA 850OG
UT WOS:000223621700005
PM 15553088
DA 2025-05-07

ER

PT J
AU Oladunni, FS
   Oyekunle, MA
   Talabi, AO
   Ojo, OE
   Takeet, MI
   Adam, M
   Raufu, IA
AF Oladunni, Fatai S.
   Oyekunle, Mufutau A.
   Talabi, Adewale O.
   Ojo, Olufemi E.
   Takeet, Michael I.
   Adam, Mohammed
   Raufu, Ibrahim A.
TI Phylogenetic analysis of Dermatophilus congolensis isolated from
   naturally infected cattle in Abeokuta and Ilorin, Nigeria
SO VETERINARY MEDICINE AND SCIENCE
LA English
DT Article
DE pleomorphic; sequences; conserved; gene; epidermis; primers; clade
AB Dermatophilus congolensis, the aetiological agent of dermatophilosis, is a pleomorphic, Gram-positive actinomycete, which infects animals and humans. Often, there is a wrong diagnosis of the infection in animals because of the close resemblance of the organism with other members of the family Actinomycetaceae. In this study, molecular tools were applied to suspected isolates of D. congolensis obtained from naturally infected cattle in Nigeria for confirmation of dermatophilosis. DNA extraction from 54 suspected pure colonies of D. congolensis was carried out using the QIAamp (R) DNA Mini extraction kit. PCR targeted at the 16S rRNA gene was employed for the confirmation of D. congolensis using 5'-ACATGCAAGTCGAACGATGA-3' and 5'-ACGCTCGCACCCTACGTATT-3' as forward and reverse primers, respectively. Positive amplicons were then sequenced directly using Big Dye Terminator Cycle Sequencing Kit with the forward primers and Ampli-Taq-FS DNA Polymerase. Nucleotide sequences were aligned using BIOEDIT (Ibis Biosciences Carlsbad, CA USA) and the phylogenetic analysis was carried out using MEGA 5.2 (Center for Evolutionary Medicine and Informatics, The Biodesign Institute, Tempe, Arizona, USA) software programme. The aligned nucleotide sequences of 10 positive D. congolensis isolates had between 94% to 99% homology with the sequences of D. congolensis satellite DNA in GenBank. This result also revealed that the sequenced D. congolensis are of different strains. Phylogenetic analysis revealed that D. congolensis, though closely related to Nocardia brasiliensis (NR 074743.01) and Streptomyces sp. (JN 400114.1), belongs to different genus. In conclusion, molecular tools employed in the study were able to confirm the identity of the test organisms as D. congolensis. It can also be concluded that two strains of D. congolensis obtained from the study can still be accommodated within the previously listed strains available in GenBank while the remaining eight may be different strains of D. congolensis not yet listed in GenBank.

C1 [Oladunni, Fatai S.; Raufu, Ibrahim A.] Univ Ilorin, Dept Vet Microbiol, PMB 1515, Ilorin, Nigeria.
[Oyekunle, Mufutau A.; Ojo, Olufemi E.; Takeet, Michael I.] Fed Univ Agr, Dept Vet Microbiol & Parasitol, PMB 2240, Abeokuta, Nigeria.
[Talabi, Adewale O.] Fed Univ Agr, Dept Vet Med, PMB 2240, Abeokuta, Nigeria.
[Adam, Mohammed] Univ Ilorin, Dept Vet Pathol, PMB 1515, Ilorin, Nigeria.
C3 University of Ilorin; University of Agriculture, Abeokuta; University of
Agriculture, Abeokuta; University of Ilorin
RP Oladunni, FS (autor correspondente), Univ Ilorin, Dept Vet Microbiol, PMB 1515, Ilorin, Nigeria.
EM kanmi01@gmail.com
RI Raufu, Ibrahim/ITU-2023-2023
OI Adam, Mohammed/0000-0002-5811-4604; Talabi, Oladele/0000-0002-9910-1439;
Ojo, Olufemi Ernest/0000-0002-3640-1688
CR ALTSCHUL SF, 1990, J MOL BIOL, V215, P403, DOI 10.1006/jmbi.1990.9999
Ambrose N, 1999, PARASITOL TODAY, V15, P295, DOI 10.1016/S0169-4758(99)01470-2
Aning KG, 1996, TROP ANIM HEALTH PRO, V28, pS39, DOI 10.1007/BF02310697
[Anonymous], MAN STAND DIAGN TEST
Gordon MA, 1976, DERMATOPHILUS INFECT, P49
Han W, 2007, ESTABLISHMENT POLYME
Hirsh D. C., 2004, VET MICROBIOL, P220
MACADAM I, 1971, Tropical Animal Health and Production, V3, P225, DOI 10.1007/BF02359584
Makinde A.A., 1991, NIG J IMMUN, V5, P56
Makinde AA, 2001, VET REC, V149, P154, DOI 10.1136/vr.149.5.154
Pal M., 1993, VERHANDLUNGSBERICHT, V35, P167
Shaibu SJ, 2011, AFR J MICROBIOL RES, V5, P467
Shaibu SJ, 2010, J ANIM VET ADV, V9, P1034, DOI 10.3923/javaa.2010.1034.1036
van SACEGHEM RENE, 1934, BULL AGRIC CONGO BEIGE, V25, P590
ZARIA LT, 1993, COMP IMMUNOL MICROB, V16, P179, DOI 10.1016/0147-9571(93)90148-X
NR 15
TC 3
Z9 3
U1 1
U2 7
PU WILEY
PI HOBOKEN
PA 111 RIVER ST, HOBOKEN, NJ 07030 USA
SN 2053-1095
J9 VET MED SCI
JI Vet. Med. Sci.
PD MAY
PY 2016
VL 2
IS 2
BP 136

EP 142
DI 10.1002/vms3.23
PG 7
WC Veterinary Sciences
WE Science Citation Index Expanded (SCI-EXPANDED)
SC Veterinary Sciences
GA EC1WG
UT WOS:000387898800006
PM 29067187
OA Green Published, gold
DA 2025-05-07
ER

PT J
AU Andrews, PW
Thomson, JA
Amstadter, A
Neale, MC
AF Andrews, Paul W.
Thomson, J. Anderson, Jr.
Amstadter, Ananda
Neale, Michael C.
TI Primum non nocere: an evolutionary analysis of whether antidepressants do more harm than good
SO FRONTIERS IN PSYCHOLOGY
LA English
DT Review
DE antidepressant medications; depression; mortality; placebo; rebound; risks; safety; side effects
ID SEROTONIN-REUPTAKE INHIBITORS; CHRONIC FLUOXETINE TREATMENT; DORSAL RAPHE NUCLEUS; SELECTIVE SEROTONIN; HIPPOCAMPAL NEUROGENESIS; MAJOR DEPRESSION; EXTRACELLULAR SEROTONIN; MYOCARDIAL-INFARCTION; VIGILANCE PERFORMANCE; CLINICAL-SIGNIFICANCE
AB Antidepressant medications are the first-line treatment for people meeting current diagnostic criteria for major depressive disorder. Most antidepressants are designed to perturb the mechanisms that regulate the neurotransmitter serotonin - an evolutionarily ancient biochemical found in plants, animals, and fungi. Many adaptive processes evolved to be regulated by serotonin, including emotion, development, neuronal growth and death, platelet activation and the clotting process, attention, electrolyte balance, and reproduction. It is a principle of evolutionary medicine that the disruption of evolved adaptations will degrade biological functioning. Because serotonin regulates many adaptive processes, antidepressants could have many adverse health effects. For instance, while antidepressants are modestly effective in reducing depressive symptoms, they increase the brain's susceptibility to future episodes after they have been discontinued. Contrary to a widely held belief in psychiatry, studies that purport to show that antidepressants promote neurogenesis are flawed because they all use a method that cannot, by itself, distinguish between neurogenesis and neuronal death. In fact, antidepressants cause neuronal damage and mature neurons to revert to an immature state, both of which may explain why antidepressants also cause neurons to undergo apoptosis (programmed death). Antidepressants can also cause developmental problems, they have

adverse effects on sexual and romantic life, and they increase the risk of hyponatremia (low sodium in the blood plasma), bleeding, stroke, and death in the elderly. Our review supports the conclusion that antidepressants generally do more harm than good by disrupting a number of adaptive processes regulated by serotonin. However, there may be specific conditions for which their use is warranted (e.g., cancer, recovery from stroke). We conclude that altered informed consent practices and greater caution in the prescription of antidepressants are warranted.
C1 [Andrews, Paul W.] McMaster Univ, Dept Psychol Neurosci & Behav, Hamilton, ON L8S 4K1, Canada.
[Andrews, Paul W.; Amstadter, Ananda; Neale, Michael C.] Virginia Commonwealth Univ, Virginia Inst Psychiat & Behav Genet, Richmond, VA USA.
[Thomson, J. Anderson, Jr.] Univ Virginia, Charlottesville, VA USA.
[Thomson, J. Anderson, Jr.] Univ Virginia, Inst Law Psychiat & Publ Policy, Charlottesville, VA USA.
C3 McMaster University; Virginia Commonwealth University; University of Virginia; University of Virginia
RP Andrews, PW (autor correspondente), McMaster Univ, Dept Psychol Neurosci & Behav, 1280 Main St West, Hamilton, ON L8S 4K1, Canada.
EM pandrews@mcmaster.ca
RI Neale, Michael/AAD-5056-2020
OI Andrews, Paul/0000-0002-5794-1747
CR Adell A, 2005, DRUG DISCOV TODAY, V10, P578, DOI 10.1016/S1359-6446(05)03398-2
Almeida OP, 2010, PLOS ONE, V5, DOI 10.1371/journal.pone.0011266
Amat J, 2005, NAT NEUROSCI, V8, P365, DOI 10.1038/nn1399
Amat J, 2006, J NEUROSCI, V26, P13264, DOI 10.1523/JNEUROSCI.3630-06.2006
Andrews P.W., 2007, EVOL PSYCHOL-US, V5, P584
Andrews PW, 2011, FRONT PSYCHOL, V2, DOI 10.3389/fpsyg.2011.00159
Andrews PW, 2009, PSYCHOL REV, V116, P620, DOI 10.1037/a0016242
Angell M., 2011, The epidemic of mental illness: Why
ANGST J, 1993, HUM PSYCHOPHARM CLIN, V8, P311, DOI 10.1002/hup.470080503
[Anonymous], 2000, HDB PSYCHIAT MEASURE
[Anonymous], DEPR MAN DEPR PRIM S
[Anonymous], DSM 4 TR DIAGN STAT
[Anonymous], DEPRESSION
Appenheimer MM, 2005, IMMUNOL INVEST, V34, P295, DOI 10.1081/IMM-200064501
Azmitia EC, 2001, BRAIN RES BULL, V56, P413, DOI 10.1016/S0361-9230(01)00614-1
Azmitia EC, 2007, INT REV NEUROBIOL, V77, P31, DOI 10.1016/S0074-7742(06)77002-7
Azmitia EC, 2010, HBK BEHAV NEUROSCI, V21, P3, DOI 10.1016/S1569-7339(10)70069-2
Bagby RM, 2004, AM J PSYCHIAT, V161, P2163, DOI 10.1176/appi.ajp.161.12.2163
Bakkaloglu B, 2008, TURK J BIOCHEM, V33, P97
Baldessarini RJ, 2007, HARVARD REV PSYCHIAT, V15, P133, DOI 10.1080/10673220701551102

Bar-Oz B, 2007, CLIN THER, V29, P918, DOI 10.1016/j.clinthera.2007.05.003
Bartholomä P, 2002, BIOCHEM PHARMACOL, V63, P1507, DOI 10.1016/S0006-2952(02)00882-1
Berger M, 2009, ANNU REV MED, V60, P355, DOI 10.1146/annurev.med.60.042307.110802
Bernard GR, 1997, NEW ENGL J MED, V336, P912, DOI 10.1056/NEJM199703273361303
Best J, 2010, THEOR BIOL MED MODEL, V7, DOI 10.1186/1742-4682-7-34
Blatteis CM, 2003, J THERM BIOL, V28, P1, DOI 10.1016/S0306-4565(02)00034-7
Bockting CLH, 2008, PSYCHOTHER PSYCHOSOM, V77, P17, DOI 10.1159/000110056
Bonin R., 2012, 60 MINUTES
Brambilla P, 2005, PHARMACOPSYCHIATRY, V38, P69, DOI 10.1055/s-2005-837806
Brandts CH, 1997, LANCET, V350, P704, DOI 10.1016/S0140-6736(97)02255-1
Bresalier RS, 2005, NEW ENGL J MED, V352, P1092, DOI 10.1056/NEJMoa050493
BRYANT RE, 1971, ARCH INTERN MED, V127, P120, DOI 10.1001/archinte.127.1.120
Bull SA, 2002, ANN PHARMACOTHER, V36, P578, DOI 10.1345/aph.1A254
Bymaster FP, 2002, PSYCHOPHARMACOLOGY, V160, P353, DOI 10.1007/s00213-001-0986-x
Byrne SE, 1998, J CLIN PSYCHIAT, V59, P279, DOI 10.4088/JCP.v59n0602
Carter O. L., 2005, J COGNITIVE NEUROSCI, V17, P1497
CHEN L, 1994, NEUROSCI RES, V19, P111, DOI 10.1016/0168-0102(94)90014-0
Cheng HC, 2010, ANN NEUROL, V67, P715, DOI 10.1002/ana.21995
Chollet F, 2011, LANCET NEUROL, V10, P123, DOI 10.1016/S1474-4422(10)70314-8
Cloonan SM, 2011, INT J CANCER, V128, P1712, DOI 10.1002/ijc.25477
Cohen HW, 2000, AM J MED, V108, P2, DOI 10.1016/S0002-9343(99)00301-0
Cohen Z, 1996, PROG NEUROBIOL, V50, P335, DOI 10.1016/S0301-0082(96)00033-0
COHN JB, 1992, J CLIN PSYCHIAT, V53, P52
Cole JA, 2007, PHARMACOEPIDEM DR S, V16, P1075, DOI 10.1002/pds.1463
Cosgrove L, 2011, PLOS ONE, V6, DOI 10.1371/journal.pone.0018210
Cotran RamziS., 1998, ROBBINS PATHOLOGIC B
Coupland C, 2011, BMJ-BRIT MED J, V343, DOI 10.1136/bmj.d4551
Croen LA, 2011, ARCH GEN PSYCHIAT, V68, P1104, DOI 10.1001/archgenpsychiatry.2011.73
Cryan JF, 2004, P NATL ACAD SCI USA, V101, P8186, DOI 10.1073/pnas.0401080101
Cumming RG, 1998, DRUG AGING, V12, P43, DOI 10.2165/00002512-199812010-00005
Dall M, 2009, CLIN GASTROENTEROL H, V7, P1314, DOI 10.1016/j.cgh.2009.08.019
Demyttenaere K, 2001, J CLIN PSYCHIAT, V62, P30
Dong HX, 2003, J NEUROSCI, V23, P1742
DORAN TF, 1989, J PEDIATR-US, V114, P1045, DOI 10.1016/S0022-3476(89)80461-5

Duman RS, 2004, BIOL PSYCHIAT, V56, P140, DOI 10.1016/j.biopsych.2004.02.033
El-Mallakh RS, 2011, MED HYPOTHESES, V76, P769, DOI 10.1016/j.mehy.2011.01.020
Elgot A, 2009, NEUROSCI LETT, V466, P6, DOI 10.1016/j.neulet.2009.08.058
Ellfolk M, 2010, REPROD TOXICOL, V30, P249, DOI 10.1016/j.reprotox.2010.04.015
Encinas JM, 2006, P NATL ACAD SCI USA, V103, P8233, DOI 10.1073/pnas.0601992103
Fava GA, 2011, PROG NEURO-PSYCHOPH, V35, P1593, DOI 10.1016/j.pnpbp.2010.07.026
Felton TM, 2003, N-S ARCH PHARMACOL, V367, P297, DOI 10.1007/s00210-002-0688-x
Fergusson D, 2005, BMJ-BRIT MED J, V330, P396, DOI 10.1136/bmj.330.7488.396
Fisher Helen E., 2006, EVOLUTIONARY COGNITI, P245
Flöck A, 2010, THROMB RES, V126, pE83, DOI 10.1016/j.thromres.2010.04.020
Fonseca FV, 2009, EXP NEUROL, V217, P353, DOI 10.1016/j.expneurol.2009.03.015
Fournier JC, 2010, JAMA-J AM MED ASSOC, V303, P47, DOI 10.1001/jama.2009.1943
Ganzel BL, 2010, PSYCHOL REV, V117, P134, DOI 10.1037/a0017773
Gawaz M, 2005, J CLIN INVEST, V115, P3378, DOI 10.1172/JCI27196
Gershon MD, 2004, ALIMENT PHARM THER, V20, P3, DOI 10.1111/j.1365-2036.2004.02180.x
Gershon MD, 2007, GASTROENTEROLOGY, V132, P397, DOI 10.1053/j.gastro.2006.11.002
Ghaemi SN, 2010, INT J CLIN PRACT, V64, P1009, DOI 10.1111/j.1742-1241.2010.02438.x
Gibson JE, 2009, AM J EPIDEMIOL, V169, P761, DOI 10.1093/aje/kwn364
Gortner EM, 2006, BEHAV THER, V37, P292, DOI 10.1016/j.beth.2006.01.004
Goveas JS, 2011, J AM GERIATR SOC, V59, P57, DOI 10.1111/j.1532-5415.2010.03233.x
Graf MC, 2008, PSYCHOTHER RES, V18, P389, DOI 10.1080/10503300701691664
GRAHAM NMH, 1990, J INFECT DIS, V162, P1277, DOI 10.1093/infdis/162.6.1277
Hagen EH, 2003, DAHL WS ENV, P95
HAMILTON M, 1960, J NEUROL NEUROSUR PS, V23, P56, DOI 10.1136/jnnp.23.1.56
Hanson ND, 2011, NEUROPSYCHOPHARMACOL, V36, P2589, DOI 10.1038/npp.2011.220
Hasday JD, 2000, MICROBES INFECT, V2, P1891, DOI 10.1016/S1286-4579(00)01337-X
Hata M, 2011, SURG TODAY, V41, P791, DOI 10.1007/s00595-010-4357-2
Hayes AM, 2007, J CONSULT CLIN PSYCH, V75, P409, DOI 10.1037/0022-006X.75.3.409
Hayes AM, 2005, INT J BEHAV MED, V12, P111, DOI 10.1207/s15327558ijbm1202_9
Heger CD, 2004, MOL INTERV, V4, P79, DOI 10.1124/mi.4.2.3

Heisler LK, 1998, P NATL ACAD SCI USA, V95, P15049, DOI 10.1073/pnas.95.25.15049
Hendrick V, 2000, PSYCHONEUROENDOCRINO, V25, P37, DOI 10.1016/S0306-4530(99)00038-4
Hengartner MO, 2000, NATURE, V407, P770, DOI 10.1038/35037710
Hernández-Cueto C, 2000, AM J FOREN MED PATH, V21, P21, DOI 10.1097/00000433-200003000-00004
Herrup K, 2004, J NEUROSCI, V24, P9232, DOI 10.1523/JNEUROSCI.3347-04.2004
Hindmarch I, 2009, INT J CLIN PRACT, V63, P1085, DOI 10.1111/j.1742-1241.2009.02085.x
Hochochka P.W., 2002, Biochemical adaptation: Mechanism and Process in Physiological Evolution
Holt A, 1996, N-S ARCH PHARMACOL, V354, P17
Holtzer R, 2007, NEUROPSYCHOLOGY, V21, P540, DOI 10.1037/0894-4105.21.5.540
Homberg JR, 2010, TRENDS PHARMACOL SCI, V31, P60, DOI 10.1016/j.tips.2009.11.003
Honig G, 2009, PLOS ONE, V4, DOI 10.1371/journal.pone.0006797
Horwitz Allan V., 2007, The Loss of Sadness: How Psychiatry Transformed Normal Sorrow into Depressive Disorder
Hranilovic D, 2011, NEUROCHEM INT, V59, P202, DOI 10.1016/j.neuint.2011.05.003
Hyman SE, 1996, AM J PSYCHIAT, V153, P151
Insel TR, 2009, PSYCHIAT SERV, V60, P1466, DOI 10.1176/appi.ps.60.11.1466
Jackson G., 2009, Drug-induced dementia: A perfect crime
Jackson GE, 2008, MED HYPOTHESES, V70, P572, DOI 10.1016/j.mehy.2007.06.019
Jacob S, 2006, ANN PHARMACOTHER, V40, P1618, DOI 10.1345/aph.1G293
Jacobs BL, 1999, NEUROPSYCHOPHARMACOL, V21, pS9, DOI 10.1038/sj.npp.1395336
Jacobs BL, 1995, SEMIN NEUROSCI, V7, P401, DOI 10.1016/1044-5765(95)90004-7
Jiménez-Trejo F, 2007, J ANDROL, V28, P136, DOI 10.2164/jandrol.106.000653
Johnson AK, 1997, FRONT NEUROENDOCRIN, V18, P292, DOI 10.1006/frne.1997.0153
Jorge RE, 2004, AM J PSYCHIAT, V161, P1507, DOI 10.1176/appi.ajp.161.8.1507
Jorge RE, 2003, AM J PSYCHIAT, V160, P1823, DOI 10.1176/appi.ajp.160.10.1823
Kalia M, 2000, BRAIN RES, V858, P92, DOI 10.1016/S0006-8993(99)02430-0
KEARNS NP, 1982, BRIT J PSYCHIAT, V141, P45, DOI 10.1192/bjp.141.1.45
Kelly O, 2007, DEPRESS ANXIETY, V24, P233, DOI 10.1002/da.20236
Kessler RC, 2007, ANNU REV CLIN PSYCHO, V3, P137, DOI 10.1146/annurev.clinpsy.3.022806.091444
Keszthelyi D, 2009, NEUROGASTROENT MOTIL, V21, P1239, DOI 10.1111/j.1365-2982.2009.01370.x
Khan A, 2005, J PSYCHIAT RES, V39, P145, DOI 10.1016/j.jpsychires.2004.06.005
Khan A, 2002, J CLIN PSYCHOPHARM, V22, P40, DOI 10.1097/00004714-200202000-00007

Khan A, 2011, PSYCHOPHARMACOLOGY, V214, P961, DOI 10.1007/s00213-010-2107-1
Kirsch I, 2008, PLOS MED, V5, P260, DOI 10.1371/journal.pmed.0050045
Kluger Matthew J., 1997, P255
Kobayashi K, 2010, P NATL ACAD SCI USA, V107, P8434, DOI 10.1073/pnas.0912690107
Kovacevic T, 2010, SYNAPSE, V64, P231, DOI 10.1002/syn.20721
KRAMER PD, 2011, NY TIMES
Lehrer J., 2010, NEW YORK TIMES 0228
Lesurtel M, 2008, CELL MOL LIFE SCI, V65, P940, DOI 10.1007/s00018-007-7377-3
Levkovitz Y, 2005, J MOL NEUROSCI, V27, P29, DOI 10.1385/JMN:27:1:029
Linder AE, 2007, J PHARMACOL EXP THER, V323, P415, DOI 10.1124/jpet.107.122630
MACKOWIAK PA, 1980, AM J MED SCI, V280, P73, DOI 10.1097/00000441-198009000-00002
Matondo RB, 2009, AM J PHYSIOL-GASTR L, V296, pG963, DOI 10.1152/ajpgi.90709.2008
Mayorga AJ, 2001, J PHARMACOL EXP THER, V298, P1101
McEwen BS, 2000, BRAIN RES, V886, P172, DOI 10.1016/S0006-8993(00)02950-4
McGrath PJ, 2006, AM J PSYCHIAT, V163, P1542, DOI 10.1176/appi.ajp.163.9.1542
Meier CR, 2001, BRIT J CLIN PHARMACO, V52, P179, DOI 10.1046/j.0306-5251.2001.01426.x
Meijer WEE, 2004, ARCH INTERN MED, V164, P2367, DOI 10.1001/archinte.164.21.2367
Mercado CP, 2010, MOL INTERV, V10, P231, DOI 10.1124/mi.10.4.6
Monster TBM, 2004, AM J MED, V117, P732, DOI 10.1016/j.amjmed.2004.06.027
Moret C, 2009, J PSYCHOPHARMACOL, V23, P967, DOI 10.1177/0269881108093582
MORROW J, 1990, J PERS SOC PSYCHOL, V58, P519, DOI 10.1037/0022-3514.58.3.519
Musselman DL, 2000, ARCH GEN PSYCHIAT, V57, P875, DOI 10.1001/archpsyc.57.9.875
Mutlu O, 2011, CHINESE J PHYSIOL, V54, P36, DOI 10.4077/CJP.2011.AMK077
Nesse R.M., 1994, WHY WE GET SICK NEW
NOLENHOEKSEMA S, 1993, COGNITION EMOTION, V7, P561, DOI 10.1080/02699939308409206
Nordlind K, 2008, EXP DERMATOL, V17, P301, DOI 10.1111/j.1600-0625.2007.00670.x
O'Hanlon JF, 1998, J CLIN PSYCHOPHARM, V18, P212, DOI 10.1097/00004714-199806000-00006
Olfson M, 2002, JAMA-J AM MED ASSOC, V287, P203, DOI 10.1001/jama.287.2.203
Paciaroni M, 2007, STROKE, V38, P423, DOI 10.1161/01.STR.0000254600.92975.1f
Park RJ, 2004, J CHILD PSYCHOL PSYC, V45, P996, DOI 10.1111/j.1469-7610.2004.t01-1-00291.x
Paulmann N, 2009, PLOS BIOL, V7, DOI 10.1371/journal.pbio.1000229
Perera TD, 2011, PLOS ONE, V6, DOI 10.1371/journal.pone.0017600

Pigott HE, 2010, PSYCHOTHER PSYCHOSOM, V79, P267, DOI 10.1159/000318293
Pincus HA, 1999, ARCH GEN PSYCHIAT, V56, P441, DOI 10.1001/archpsyc.56.5.441
Popa D, 2010, EUR J PHARMACOL, V628, P83, DOI 10.1016/j.ejphar.2009.11.037
Post A, 2000, BIOL PSYCHIAT, V47, p138S, DOI 10.1016/S0006-3223(00)00721-6
Pozuelo L, 2009, CLEV CLIN J MED, V76, P59, DOI 10.3949/ccjm.75a.08011
RAMAEKERS JG, 1995, BRIT J CLIN PHARMACO, V39, P397, DOI 10.1111/j.1365-2125.1995.tb04468.x
Rantala S, 2009, J INFECTION, V58, P266, DOI 10.1016/j.jinf.2009.01.015
Reeves RR, 2010, CNS NEUROSCI THER, V16, P227, DOI 10.1111/j.1755-5949.2010.00160.x
Riedel WJ, 2005, J PSYCHOPHARMACOL, V19, P12, DOI 10.1177/0269881105048887
Romanovsky AA, 2005, FRONT BIOSCI-LANDMRK, V10, P2193, DOI 10.2741/1690
Romanovsky AA, 2007, AM J PHYSIOL-REG I, V292, pR37, DOI 10.1152/ajpregu.00668.2006
Rush AJ, 2006, AM J PSYCHIAT, V163, P1905, DOI 10.1176/appi.ajp.163.11.1905
RUTTER JJ, 1993, J PHARMACOL EXP THER, V265, P1319
Sairanen M, 2005, J NEUROSCI, V25, P1089, DOI 10.1523/JNEUROSCI.3741-04.2005
Sandercock PAG, 2008, COCHRANE DB SYST REV, DOI 10.1002/14651858.CD000024.pub3
Santarelli L, 2003, SCIENCE, V301, P805, DOI 10.1126/science.1083328
Sapolsky R.M., 2004, Why zebras don't get ulcers: The acclaimed guide to stress, stressrelated diseases, and coping
Sapolsky RM, 2001, P NATL ACAD SCI USA, V98, P12320, DOI 10.1073/pnas.231475998
Sauer WH, 2003, CIRCULATION, V108, P32, DOI 10.1161/01.CIR.0000079172.43229.CD
Schlienger RG, 2004, DRUG SAFETY, V27, P1157, DOI 10.2165/00002018-200427140-00006
Schmaling KB, 2002, J ABNORM PSYCHOL, V111, P350, DOI 10.1037//0021-843X.111.2.350
Schmitt JAJ, 2002, J PSYCHOPHARMACOL, V16, P207, DOI 10.1177/026988110201600303
Schmitt JAJ, 2001, J PSYCHOPHARMACOL, V15, P173, DOI 10.1177/026988110101500304
Schulman Carl I, 2005, Surg Infect (Larchmt), V6, P369
Serretti A, 2009, J CLIN PSYCHOPHARM, V29, P259, DOI 10.1097/JCP.0b013e3181a5233f
Smoller JW, 2009, ARCH INTERN MED, V169, P2128, DOI 10.1001/archinternmed.2009.436
Sonis J, 2004, AM J PSYCHIAT, V161, P1506, DOI 10.1176/appi.ajp.161.8.1506-a
Stahl S.M., 2008, Stahl's Essential Psychopharmacology, V3rd
STANLEY ED, 1975, JAMA-J AM MED ASSOC, V231, P1248, DOI 10.1001/jama.231.12.1248

Stergiou L, 2004, CELL DEATH DIFFER, V11, P21, DOI 10.1038/sj.cdd.4401340
Stone M, 2009, BMJ-BRIT MED J, V339, DOI 10.1136/bmj.b2880
Swenson BR, 2007, J AM COLL SURGEONS, V204, P815, DOI 10.1016/j.jamcollsurg.2007.01.033
Tanrikut C, 2010, FERTIL STERIL, V94, P1021, DOI 10.1016/j.fertnstert.2009.04.039
Tata LJ, 2005, HEART, V91, P465, DOI 10.1136/hrt.2004.037457
Taupin P, 2007, BRAIN RES REV, V53, P198, DOI 10.1016/j.brainresrev.2006.08.002
Trifirò G, 2010, J CLIN PSYCHOPHARM, V30, P252, DOI 10.1097/JCP.0b013e3181dca10a
Turner Erick H, 2008, N Engl J Med, V358, P252, DOI 10.1056/NEJMsa065779
Ulak G, 2006, DRUG DEVELOP RES, V67, P456, DOI 10.1002/ddr.20106
Van der Kooy K, 2007, INT J GERIATR PSYCH, V22, P613, DOI 10.1002/gps.1723
van Haelst IMM, 2010, ANESTHESIOLOGY, V112, P631, DOI 10.1097/ALN.0b013e3181cf8fdf
Vickers KS, 2003, COGNITIVE THER RES, V27, P331, DOI 10.1023/A:1023970501448
WAKEFIELD JC, 1992, AM PSYCHOL, V47, P373, DOI 10.1037/0003-066X.47.3.373
Wakefield JC, 1999, J ABNORM PSYCHOL, V108, P374, DOI 10.1037/0021-843X.108.3.374
Wakefield JC, 2010, AM J PSYCHIAT, V167, P298, DOI 10.1176/appi.ajp.2009.09040553
Walker A J, 2011, Br J Cancer, V104, P193, DOI 10.1038/sj.bjc.6605996
Warner-Schmidt JL, 2006, HIPPOCAMPUS, V16, P239, DOI 10.1002/hipo.20156
Watson PJ, 2002, J AFFECT DISORDERS, V72, P1, DOI 10.1016/S0165-0327(01)00459-1
Weber C, 2005, CIRC RES, V96, P612, DOI 10.1161/01.RES.0000160077.17427.57
WEINSTEIN MP, 1978, AM J MED, V64, P592, DOI 10.1016/0002-9343(78)90578-8
WEINSTEIN MP, 1983, REV INFECT DIS, V5, P54
Wenzlaff RM, 2003, COGNITIVE THER RES, V27, P293, DOI 10.1023/A:1023966400540
Wilson CC, 1998, BRAIN RES, V782, P235, DOI 10.1016/S0006-8993(97)01284-5
Wingen M, 2005, J CLIN PSYCHIAT, V66, P436, DOI 10.4088/JCP.v66n0405
Woods H. A., 2009, Phenotypic plasticity of insects: mechanisms and consequences, P655
Zimmerman M, 2010, J CLIN PSYCHIAT, V71, P484, DOI 10.4088/JCP.08m04978blu
NR 202
TC 83
Z9 96
U1 1
U2 43
PU FRONTIERS MEDIA SA
PI LAUSANNE
PA AVENUE DU TRIBUNAL FEDERAL 34, LAUSANNE, CH-1015, SWITZERLAND

SN 1664-1078
J9 FRONT PSYCHOL
JI Front. Psychol.
PY 2012
VL 3
AR 117
DI 10.3389/fpsyg.2012.00117
PG 19
WC Psychology, Multidisciplinary
WE Social Science Citation Index (SSCI)
SC Psychology
GA V31DI
UT WOS:000208863900129
PM 22536191
OA gold, Green Published
DA 2025-05-07
ER

PT J
AU Crespi, B
AF Crespi, Bernard
TI Evolutionary medical insights into the SARS-CoV-2 pandemic
SO EVOLUTION MEDICINE AND PUBLIC HEALTH
LA English
DT Editorial Material
DE COVID-19; SARS-CoV-2; inflammation; interferon; coronavirus; amlodipine
ID AMLODIPINE; INFECTION; CORONAVIRUSES; EPIDEMIOLOGY; PATHOGENESIS;
   INFLAMMATION; LONGEVITY; COVID-19; VIRUSES; 229E
AB The author apply concepts and tools from evolutionary medicine to understanding the SARS-CoV-2 pandemic. The pandemic represents a mismatched conflict, with dynamics and pathology apparently driven by three main factors: (i) bat immune systems that rely on low inflammation but high efficacy of interferon-based defenses; (ii) viral tactics that differentially target the human interferon system, leading to substantial asymptomatic and pre-symptomatic transmission; and (ii) high mortality caused by hyper-inflammatory and hyper-coagulatory phenotypes, that represent dysregulated tradeoffs whereby collateral immune-induced damage becomes systemic and severe. This framework can explain the association of mortality with age (which involves immune life-history shifts towards higher inflammation and coagulation and reduced adaptive immunity), and sex (since males senesce faster than females). Genetic-risk factors for COVID-19 mortality can be shown, from a phenome-wide association analysis of the relevant SNPs, to be associated with inflammation and coagulation; the phenome-wide association study also provides evidence, consistent with several previous studies, that the calcium channel blocking drug amlodipine mediates risk of mortality.
   Lay Summary: SARS-CoV-2 is a bat virus that jumped into humans. The virus is adapted to bat immune systems, where it evolved to suppress the immune defenses (interferons) that mammals use to tell that they are infected. In humans, the virus can apparently spread effectively in the body with a delay in the production of symptoms and the initiation of immune responses. This delay may then promote overactive immune responses, when the virus is detected, that damage the body as a side

effect. Older people are more vulnerable to the virus because they are less adapted to novel infectious agents, and invest less in immune defense, compared to younger people. Genes that increase risk of mortality from SARS-CoV-2 are functionally associated with a drug called amlodipine, which may represent a useful treatment.
C1 [Crespi, Bernard] Simon Fraser Univ, Dept Biol Sci, 8888 Univ Dr, Burnaby, BC V5A 1S6, Canada.
C3 Simon Fraser University
RP Crespi, B (autor correspondente), Simon Fraser Univ, Dept Biol Sci, 8888 Univ Dr, Burnaby, BC V5A 1S6, Canada.
EM crespi@sfu.ca
FU Natural Sciences and Engineering Research Council of Canada [2019-04208]
FX Supported by Natural Sciences and Engineering Research Council of Canada
Discovery Grant 2019-04208.
CR Ahn M, 2016, SCI REP-UK, V6, DOI 10.1038/srep21722
Banerjee A., 2020, EARLY TEMPORAL DYNAM, DOI [10.1101/2020.06.18.158154., DOI 10.1101/2020.06.18.158154]
Banerjee A, 2020, ISCIENCE, V23, DOI 10.1016/j.isci.2020.100958
Banerjee A, 2020, FRONT IMMUNOL, V11, DOI 10.3389/fimmu.2020.00026
Banerjee A, 2017, SCI REP-UK, V7, DOI 10.1038/s41598-017-01513-w
Bell DM, 2004, EMERG INFECT DIS, V10, P1900, DOI 10.3201/eid1011.040729
Bell G, 2018, TRENDS MICROBIOL, V26, P471, DOI 10.1016/j.tim.2017.11.005
Brook CE, 2020, ELIFE, V9, DOI 10.7554/eLife.48401
Calisher C, 2016, MEM I O CRUZ, V110, P1
Chen XJ, 2020, CELLS-BASEL, V9, DOI 10.3390/cells9010094
Ciminelli G, 2020, VOXEU CTR EC POLICY, V20
Daugherty MD, 2012, ANNU REV GENET, V46, P677, DOI 10.1146/annurev-genet-110711-155522
Day T, 2020, CURR BIOL, V30, pR849, DOI 10.1016/j.cub.2020.06.031
Denison MR, 2011, RNA BIOL, V8, P270, DOI 10.4161/rna.8.2.15013
Dijkman R, 2008, J CLIN MICROBIOL, V46, P2368, DOI 10.1128/JCM.00533-08
Ellinghaus D, 2020, NEW ENGL J MED, V383, P1522, DOI 10.1056/NEJMoa2020283
Esmon CT, 2011, J THROMB HAEMOST, V9, P182, DOI 10.1111/j.1538-7836.2011.04323.x
Ewald PW, 2011, ECOL RES, V26, P1017, DOI 10.1007/s11284-011-0874-8
Ferron F, 2018, P NATL ACAD SCI USA, V115, pE162, DOI 10.1073/pnas.1718806115
Franceschi C, 2018, NAT REV ENDOCRINOL, V14, P576, DOI 10.1038/s41574-018-0059-4
García-Sastre A, 2017, CELL HOST MICROBE, V22, P176, DOI 10.1016/j.chom.2017.07.012
Goodwin K, 2006, VACCINE, V24, P1159, DOI 10.1016/j.vaccine.2005.08.105
Guth S, 2019, PHILOS T R SOC B, V374, DOI 10.1098/rstb.2019.0296
Hawkins JA, 2019, P NATL ACAD SCI USA, V116, P11351, DOI 10.1073/pnas.1814995116
HERNANDEZ R, 1993, CLIN THER, V15, P304
Herz J, 2016, IMMUNITY, V44, P718, DOI 10.1016/j.immuni.2016.03.010

Hoffmann HH, 2015, TRENDS IMMUNOL, V36, P124, DOI 10.1016/j.it.2015.01.004
Holmes EC, 2003, TRENDS MICROBIOL, V11, P543, DOI 10.1016/j.tim.2003.10.006
Huang ZX, 2019, NAT ECOL EVOL, V3, P1110, DOI 10.1038/s41559-019-0913-3
Jamilloux Y, 2020, AUTOIMMUN REV, V19, DOI 10.1016/j.autrev.2020.102567
Jin JM, 2020, FRONT PUBLIC HEALTH, V8, DOI 10.3389/fpubh.2020.00152
Joachim RB, 2018, FUTURE MICROBIOL, V13, P1549, DOI 10.2217/fmb-2018-0221
Joachim RB, 2018, MOL SYST BIOL, V14, DOI 10.15252/msb.20177998
Kacprzyk J, 2017, ACTA CHIROPTEROL, V19, P219, DOI [10.3161/15081109ACC2017.19.2.001, 10.3161/15081109acc2017.19.2.001]
Kataoka C, 2004, AM J PHYSIOL-HEART C, V286, pH768, DOI 10.1152/ajpheart.00937.2002
Korber B, 2020, CELL, V182, P812, DOI 10.1016/j.cell.2020.06.043
Kuo Chia-Ling, 2020, medRxiv, DOI 10.1101/2020.07.10.20147777
Ledford H, 2020, NATURE, V580, P311, DOI 10.1038/d41586-020-01056-7
Leisman DE, 2020, INTENS CARE MED, V46, P1105, DOI 10.1007/s00134-020-06059-6
Lenart P, 2019, SCI REP-UK, V9, DOI 10.1038/s41598-019-47111-w
Levi M, 2003, CARDIOVASC RES, V60, P26, DOI 10.1016/S0008-6363(02)00857-X
Lu C., 2020, GENETIC RISK FACTORS, DOI 10.1101/2020.07.01.20144592
McDade TW, 2016, EVOL MED PUBLIC HLTH, P1, DOI 10.1093/emph/eov033
McDade TW, 2003, YEARB PHYS ANTHROPOL, V46, P100, DOI 10.1002/ajpa.10398
Merad M, 2020, NAT REV IMMUNOL, V20, P355, DOI 10.1038/s41577-020-0331-4
Miller IF, 2019, J R SOC INTERFACE, V16, DOI 10.1098/rsif.2019.0642
Moghadas SM, 2020, P NATL ACAD SCI USA, V117, P17513, DOI 10.1073/pnas.2008373117
Montenont E, 2019, CURR OPIN HEMATOL, V26, P336, DOI 10.1097/MOH.0000000000000526
Mueller AL, 2020, AGING-US, V12, P9959, DOI 10.18632/aging.103344
Oh M, 2019, CLIN CARDIOL, V42, P241, DOI 10.1002/clc.23133
Okin D, 2012, CURR BIOL, V22, pR733, DOI 10.1016/j.cub.2012.07.029
Opal SM, 2005, CLIN INFECT DIS, V41, pS504, DOI 10.1086/432007
Pavlovich SS, 2018, CELL, V173, P1098, DOI 10.1016/j.cell.2018.03.070
Poletti P, 200608471 ARXIV
Randolph HE, 2018, IMMUNITY, V48, P1074, DOI 10.1016/j.immuni.2018.05.016
Read AF, 2015, PLOS BIOL, V13, DOI 10.1371/journal.pbio.1002198
Regan JC, 2013, BEST PRACT RES CL EN, V27, P467, DOI 10.1016/j.beem.2013.05.016
Rottoli M, 2020, LANCET
Schountz T, 2017, FRONT IMMUNOL, V8, DOI 10.3389/fimmu.2017.01098
Sen GC, 2001, ANNU REV MICROBIOL, V55, P255, DOI 10.1146/annurev.micro.55.1.255
Shang, 2020, CALCIUM CHANNEL BLOC, DOI 10.1101/2020.04.08.20047134
Shoelson SE, 2007, GASTROENTEROLOGY, V132, P2169, DOI 10.1053/j.gastro.2007.03.059
Shokri S, 2019, J CELL PHYSIOL, V234, P2143, DOI 10.1002/jcp.27155

Siddiqi HK, 2020, J HEART LUNG TRANSPL, V39, P405, DOI
10.1016/j.healun.2020.03.012
Solaimanzadeh I, 2020, CUREUS J MED SCIENCE, V12, DOI
10.7759/cureus.8069
Straub RH, 2016, EVOL MED PUBLIC HLTH, P37, DOI 10.1093/emph/eow001
Straus MR, 2020, BIORXIV, DOI [10.1101/2020.07.21.214577v1, DOI
10.1101/2020.07.21.214577V1]
Stromberg SP, 2006, PLOS COMPUT BIOL, V2, P1475, DOI
10.1371/journal.pcbi.0020160
Su S, 2016, TRENDS MICROBIOL, V24, P490, DOI 10.1016/j.tim.2016.03.003
Subudhi S, 2019, VIRUSES-BASEL, V11, DOI 10.3390/v11020192
Totura AL, 2012, CURR OPIN VIROL, V2, P264, DOI
10.1016/j.coviro.2012.04.004
Walsh EE, 2013, J INFECT DIS, V208, P1634, DOI 10.1093/infdis/jit393
Watanabe K, 2019, NAT GENET, V51, P1339, DOI 10.1038/s41588-019-0481-0
Weinberger B, 2018, IMMUN AGEING, V15, DOI 10.1186/s12979-017-0107-2
Weitz JS, 2019, VIRUS EVOL, V5, DOI 10.1093/ve/vez006
WILLIAMS GC, 1991, Q REV BIOL, V66, P1, DOI 10.1086/417048
Wong LYR, 2016, VIROL SIN, V31, P12, DOI 10.1007/s12250-015-3683-3
Worobey M, 2007, ANNU REV ECOL EVOL S, V38, P515, DOI
10.1146/annurev.ecolsys.38.091206.095722
Yurkovetskiy Leonid, 2020, Cell, V183, P739, DOI
[10.1101/2020.07.04.187757, 10.1016/j.cell.2020.09.032]
Zeberg H, 2020, MAJOR GENETIC RISK F, V2020, DOI
[10.1101/2020.07.03.186296, DOI 10.1101/2020.07.03.186296]
Zhou WM, 2013, BMC INFECT DIS, V13, DOI 10.1186/1471-2334-13-433
Zhu LH, 2020, CELL METAB, V31, P1068, DOI 10.1016/j.cmet.2020.04.021
NR 82
TC 16
Z9 18
U1 0
U2 15
PU OXFORD UNIV PRESS
PI OXFORD
PA GREAT CLARENDON ST, OXFORD OX2 6DP, ENGLAND
EI 2050-6201
J9 EVOL MED PUBLIC HLTH
JI Evol. Med. Public Health
PD OCT 14
PY 2020
IS 1
BP 314
EP 322
DI 10.1093/emph/eoaa036
PG 9
WC Evolutionary Biology; Public, Environmental & Occupational Health
WE Science Citation Index Expanded (SCI-EXPANDED)
SC Evolutionary Biology; Public, Environmental & Occupational Health
GA PO5SQ
UT WOS:000605229500001
PM 33335737
OA gold, Green Published
DA 2025-05-07
ER

PT J
AU Chevalier, RL
AF Chevalier, Robert L.
TI Bioenergetic Evolution Explains Prevalence of Low Nephron Number at Birth: Risk Factor for CKD
SO KIDNEY360
LA English
DT Review
ID INTRAUTERINE GROWTH RESTRICTION; GLOMERULAR NUMBER; KIDNEY-DISEASE; MITOCHONDRIAL-DNA; PRENATAL EXPOSURE; AMPK KNOCKDOWN; METABOLIC-RATE; RENAL-DISEASE; SELF-RENEWAL; STEM-CELLS
AB There is greater than tenfold variation in nephron number of the human kidney at birth. Although low nephron number is a recognized risk factor for CKD, its determinants are poorly understood. Evolutionary medicine represents a new discipline that seeks evolutionary explanations for disease, broadening perspectives on research and public health initiatives. Evolution of the kidney, an organ rich in mitochondria, has been driven by natural selection for reproductive fitness constrained by energy availability. Over the past 2 million years, rapid growth of an energy-demanding brain in Homo sapiens enabled hominid adaptation to environmental extremes through selection for mutations in mitochondrial and nuclear DNA epigenetically regulated by allocation of energy to developing organs. Maternal undernutrition or hypoxia results in intrauterine growth restriction or preterm birth, resulting in low birth weight and low nephron number. Regulated through placental transfer, environmental oxygen and nutrients signal nephron progenitor cells to reprogram metabolism from glycolysis to oxidative phosphorylation. These processes are modulated by counterbalancing anabolic and catabolic metabolic pathways that evolved from prokaryote homologs and by hypoxia-driven and autophagy pathways that evolved in eukaryotes. Regulation of nephron differentiation by histone modifications and DNA methyltransferases provide epigenetic control of nephron number in response to energy available to the fetus. Developmental plasticity of nephrogenesis represents an evolved life history strategy that prioritizes energy to early brain growth with adequate kidney function through reproductive years, the trade-off being increasing prevalence of CKD delayed until later adulthood. The research implications of this evolutionary analysis are to identify regulatory pathways of energy allocation directing nephrogenesis while accounting for the different life history strategies of animal models such as the mouse. The clinical implications are to optimize nutrition and minimize hypoxic/toxic stressors in childbearing women and children in early postnatal development.
C1 [Chevalier, Robert L.] Univ Virginia, Dept Pediat, POB 800386, Charlottesville, VA 22908 USA.
C3 University of Virginia
RP Chevalier, RL (autor correspondente), Univ Virginia, Dept Pediat, POB 800386, Charlottesville, VA 22908 USA.
EM RLC2M@virginia.edu
OI Chevalier, Robert/0000-0002-7465-482X
CR AIELLO LC, 1995, CURR ANTHROPOL, V36, P199, DOI 10.1086/204350
[Anonymous], 2018, 2018 USRDS Annual Data Report: Epidemiology of Kidney Disease in the United States

Baker CN., 2013, OA Biotechnol, V2, P16, DOI DOI 10.13172/2052-0069-2-2-571
Baldelomar EJ, 2018, AM J PHYSIOL-RENAL, V314, pF399, DOI 10.1152/ajprenal.00399.2017
Barker DJP, 2007, J INTERN MED, V261, P412, DOI 10.1111/j.1365-2796.2007.01809.x
Barker DJP, 2012, PLACENTA, V33, pE30, DOI 10.1016/j.placenta.2012.06.009
BARKER DJP, 1986, LANCET, V1, P1077
Bates CM, 2014, CLIN J AM SOC NEPHRO, V9, P1141, DOI 10.2215/CJN.00860114
Bennett KM, 2021, J DEV ORIG HLTH DIS, V12, P179, DOI 10.1017/S204017442000001X
Bernhardt WM, 2006, KIDNEY INT, V69, P114, DOI 10.1038/sj.ki.5000062
Bertram JF, 2011, PEDIATR NEPHROL, V26, P1529, DOI 10.1007/s00467-011-1843-8
Bhargava P, 2017, NAT REV NEPHROL, V13, P629, DOI 10.1038/nrneph.2017.107
Bock T, 2005, DIABETES, V54, P133, DOI 10.2337/diabetes.54.1.133
Bonduriansky R., 2018, Extended Heredity: A New Understanding of Inheritance and Evolution
Bowe B, 2018, JAMA NETW OPEN, V1, DOI 10.1001/jamanetworkopen.2018.4412
BRENNER BM, 1988, AM J HYPERTENS, V1, P335, DOI 10.1093/ajh/1.4.335
Calderon-Margalit R, 2018, NEW ENGL J MED, V378, P428, DOI 10.1056/NEJMoa1700993
Carey EAK, 2014, STEM CELLS DEV, V23, P2921, DOI 10.1089/scd.2014.0092
Cargill K, 2019, J AM SOC NEPHROL, V30, P1192, DOI 10.1681/ASN.2018111170
Carreau A, 2011, J CELL MOL MED, V15, P1239, DOI 10.1111/j.1582-4934.2011.01258.x
Centers for Disease Control and Prevention, 2018, CHRON KIDN DIS CKD S
Charlton JR, 2016, NANOMED-NANOTECHNOL, V12, P1735, DOI 10.1016/j.nano.2016.03.007
Chen LG, 2010, STEM CELLS, V28, P57, DOI 10.1002/stem.251
Chen XZ, 2015, FREE RADICAL BIO MED, V79, P253, DOI 10.1016/j.freeradbiomed.2014.08.027
Chevalier RL, 2019, SEMIN CELL DEV BIOL, V91, P119, DOI 10.1016/j.semcdb.2018.05.024
Chevalier RL, 2018, J AM SOC NEPHROL, V29, P706, DOI 10.1681/ASN.2018010013
Chevalier RL, 2017, KIDNEY INT REP, V2, P302, DOI 10.1016/j.ekir.2017.01.012
Cox LA, 2006, J PHYSIOL-LONDON, V572, P67, DOI 10.1113/jphysiol.2006.106872
Craig PM, 2018, COMP BIOCHEM PHYS B, V224, P156, DOI 10.1016/j.cbpb.2017.11.001
Crump C, 2019, BMJ-BRIT MED J, V365, DOI 10.1136/bmj.l1346
Darwin C, 2003, On the origin of species
Denic A, 2017, J AM SOC NEPHROL, V28, P313, DOI 10.1681/ASN.2016020154
Dimasuay KG, 2016, FRONT PHYSIOL, V7, DOI 10.3389/fphys.2016.00012
Douglas-Denton R, 2002, J AM SOC NEPHROL, V13, P406, DOI 10.1681/ASN.V132406
Du A, 2018, P ROY SOC B-BIOL SCI, V285, DOI 10.1098/rspb.2017.2738

Dumesic PA, 2019, CELL METAB, V30, P190, DOI 10.1016/j.cmet.2019.04.013
Dybala MP, 2019, ISLETS, V11, P1, DOI 10.1080/19382014.2018.1557486
Erecinska M, 2001, RESP PHYSIOL, V128, P263, DOI 10.1016/S0034-5687(01)00306-1
Eriksson JG, 2018, AM J KIDNEY DIS, V71, P20, DOI 10.1053/j.ajkd.2017.06.030
FOTHERGILLGILMORE LA, 1993, PROG BIOPHYS MOL BIO, V59, P105, DOI 10.1016/0079-6107(93)90001-Z
Gemma C, 2006, OBESITY, V14, P2193, DOI 10.1038/oby.2006.257
Georgas KM, 2011, DEV DYNAM, V240, P1600, DOI 10.1002/dvdy.22640
Geraci S, 2017, AM J PHYSIOL-RENAL, V313, pF576, DOI 10.1152/ajprenal.00329.2015
Giussani DA, 2016, J PHYSIOL-LONDON, V594, P1215, DOI 10.1113/JP271099
Gjerde A, 2020, NEPHROL DIAL TRANSPL, V35, P1157, DOI 10.1093/ndt/gfaa001
Gluckman Peter., 2005, The Fetal Matrix: Evolution, Development and Disease
Goda N, 2012, INT J HEMATOL, V95, P457, DOI 10.1007/s12185-012-1069-y
Graham AM, 2017, PLOS ONE, V12, DOI 10.1371/journal.pone.0179545
Green DR, 2014, SCIENCE, V345, P1466, DOI 10.1126/science.1250256
Gutierrez-Mazariegos J, 2014, SUBCELL BIOCHEM, V70, P55, DOI 10.1007/978-94-017-9050-5_4
Haig D, 2019, CURR BIOL, V29, pR455, DOI 10.1016/j.cub.2019.04.040
Harder T, 2007, AM J EPIDEMIOL, V165, P849, DOI 10.1093/aje/kwk071
Heiden MGV, 2009, SCIENCE, V324, P1029, DOI 10.1126/science.1160809
Heijmans BT, 2008, P NATL ACAD SCI USA, V105, P17046, DOI 10.1073/pnas.0806560105
Hill NR, 2016, PLOS ONE, V11, DOI 10.1371/journal.pone.0158765
HINCHLIFFE SA, 1991, LAB INVEST, V64, P777
Hochberg Z, 2008, ARCH DIS CHILD, V93, P534, DOI 10.1136/adc.2008.137570
Holliday M.A., 1986, Human Growth: A Comprehensive,Treatise, P101, DOI 10.1007/978-1-4899-0522-2_5
HOUK JC, 1988, FASEB J, V2, P97, DOI 10.1096/fasebj.2.2.3277888
Hoy WE, 2005, J AM SOC NEPHROL, V16, P2557, DOI 10.1681/ASN.2005020172
Hoy WE, 2011, AM J NEPHROL, V33, P15, DOI 10.1159/000327044
Hughes T, 2007, ADV EXP MED BIOL, V607, P111
Hughson M, 2003, KIDNEY INT, V63, P2113, DOI 10.1046/j.1523-1755.2003.00018.x
Jurkowski TP, 2011, PLOS ONE, V6, DOI 10.1371/journal.pone.0028104
Kawakami T, 2015, J AM SOC NEPHROL, V26, P1040, DOI 10.1681/ASN.2013111202
Khalsa DDK, 2016, PEDIATR NEPHROL, V31, P1509, DOI 10.1007/s00467-016-3384-7
Kimball R, 2015, PHYSIOL REP, V3, DOI 10.14814/phy2.12651
Kirkwood TBL, 2005, MECH AGEING DEV, V126, P1011, DOI 10.1016/j.mad.2005.03.021
KIRKWOOD TBL, 1977, NATURE, V270, P301, DOI 10.1038/270301a0
Kucharski R, 2008, SCIENCE, V319, P1827, DOI 10.1126/science.1153069
Kuma A, 2004, NATURE, V432, P1032, DOI 10.1038/nature03029
Lambeth JD, 2007, FREE RADICAL BIO MED, V43, P332, DOI 10.1016/j.freeradbiomed.2007.03.027
Lampl M, 2005, AM J HUM BIOL, V17, P178, DOI 10.1002/ajhb.20109

Lampl M, 2005, AM J HUM BIOL, V17, P66, DOI 10.1002/ajhb.20094
Lane N, 2003, J THEOR BIOL, V225, P531, DOI 10.1016/S0022-5193(03)00304-7
Lea AJ, 2017, EVOL MED PUBLIC HLTH, P201, DOI 10.1093/emph/eoy007
Lea AJ, 2017, EVOL MED PUBLIC HLTH, P162, DOI 10.1093/emph/eox019
Lee DY, 2015, BMB REP, V48, P209, DOI 10.5483/BMBRep.2015.48.4.275
Lee YQ, 2018, NUTRIENTS, V10, DOI 10.3390/nu10020241
Lelièvre-Pégorier M, 1998, KIDNEY INT, V54, P1455, DOI 10.1046/j.1523-1755.1998.00151.x
LEONARD WR, 1992, AM J HUM BIOL, V4, P179, DOI 10.1002/ajhb.1310040204
Lewis RM, 2012, PLACENTA, V33, pS28, DOI 10.1016/j.placenta.2011.12.003
Little MH, 2019, GENE DEV, V33, P1319, DOI 10.1101/gad.329573.119
Little MH, 2015, CURR OPIN GENET DEV, V32, P135, DOI 10.1016/j.gde.2015.03.001
Little MH, 2014, CLIN J AM SOC NEPHRO, V9, P809, DOI 10.2215/CJN.10851013
Liu J, 2017, J AM SOC NEPHROL, V28, P3323, DOI 10.1681/ASN.2016111246
Luyckx VA, CLIN CONSEQUENCES DE
Luyckx VA, 2009, AM J PHYSIOL-RENAL, V297, pF1697, DOI 10.1152/ajprenal.00462.2009
Mandò C, 2014, AM J PHYSIOL-ENDOC M, V306, pE404, DOI 10.1152/ajpendo.00426.2013
McEwen BS, 2003, HORM BEHAV, V43, P2, DOI 10.1016/S0018-506X(02)00024-7
McKiernan SH, 2007, AM J PHYSIOL-RENAL, V292, pF1751, DOI 10.1152/ajprenal.00307.2006
McMahon AP, 2008, J AM SOC NEPHROL, V19, P667, DOI 10.1681/ASN.2007101078
Morita M, 2015, CELL CYCLE, V14, P473, DOI 10.4161/15384101.2014.991572
Moussaieff A, 2015, CELL METAB, V21, P392, DOI 10.1016/j.cmet.2015.02.002
Murugapoopathy V, 2020, CLIN J AM SOC NEPHRO, V15, P723, DOI 10.2215/CJN.12581019
Navarrete A, 2011, NATURE, V480, P91, DOI 10.1038/nature10629
Nijland MJ, 2007, J PHYSIOL-LONDON, V579, P643, DOI 10.1113/jphysiol.2006.122101
Nishimura H, 2007, AM J PHYSIOL-REG I, V293, pR2147, DOI 10.1152/ajpregu.00163.2007
Noble D., 2017, BIOLOGICAL RELATIVIT
Nye GA, 2018, J PHYSIOL-LONDON, V596, P5523, DOI 10.1113/JP275633
Painter RC, 2005, J AM SOC NEPHROL, V16, P189, DOI 10.1681/ASN.2004060474
Paldi A, 2012, PROG BIOPHYS MOL BIO, V110, P41, DOI 10.1016/j.pbiomolbio.2012.04.003
Pereira SP, 2015, AM J PHYSIOL-RENAL, V308, pF1217, DOI 10.1152/ajprenal.00419.2014
Perera F, 2011, REPROD TOXICOL, V31, P363, DOI 10.1016/j.reprotox.2010.12.055
Perlman R, 2020, INTEGRATING EVOLUTIONARY BIOLOGY INTO MEDICAL EDUCATION: FOR MATERNAL AND CHILD HEALTHCARE STUDENTS, CLINICIANS, AND SCIENTISTS, P119, DOI 10.1093/oso/9780198814153.003.0007
Perlman RL, 2016, EVOL MED PUBLIC HLTH, P170, DOI 10.1093/emph/eow014

Potter E.L., 1972, NORMAL ABNORMAL DEV
Poulose N, 2014, AGING DIS, V5, P101, DOI 10.14336/AD.2014.0500101
Puelles VG, 2011, CURR OPIN NEPHROL HY, V20, P7, DOI 10.1097/MNH.0b013e3283410a7d
Quinlan J, 2007, J AM SOC NEPHROL, V18, P1915, DOI 10.1681/ASN.2006101107
Ravera S, 2018, CELL MOL LIFE SCI, V75, P889, DOI 10.1007/s00018-017-2665-z
RODESCH F, 1992, OBSTET GYNECOL, V80, P283
Rodríguez MM, 2004, PEDIATR DEVEL PATHOL, V7, P17, DOI 10.1007/s10024-003-3029-2
Rodríguez-Trejo A, 2012, AM J PHYSIOL-ENDOC M, V302, pE1431, DOI 10.1152/ajpendo.00619.2011
Rolfe DFS, 1997, PHYSIOL REV, V77, P731, DOI 10.1152/physrev.1997.77.3.731
Roustan V, 2016, J EXP BOT, V67, P3897, DOI 10.1093/jxb/erw211
Rylander C, 2013, GLOBAL HEALTH ACTION, V6, DOI 10.3402/gha.v6i0.19538
Salomon R, 2001, KIDNEY INT, V59, P457, DOI 10.1046/j.1523-1755.2001.059002457.x
Sampogna RV, 2015, J AM SOC NEPHROL, V26, P2414, DOI 10.1681/ASN.2014090886
Schmitt R, 2008, AM J PHYSIOL-RENAL, V294, pF1265, DOI 10.1152/ajprenal.00543.2007
Schrdinger E., 1944
Seebacher F, 2018, COMP BIOCHEM PHYS B, V224, P195, DOI 10.1016/j.cbpb.2017.11.002
Sharma N, 2019, ENVIRON MOL MUTAGEN, V60, P668, DOI 10.1002/em.22319
Shen Q, 2010, PEDIATR NEPHROL, V25, P1063, DOI 10.1007/s00467-009-1437-x
Shyh-Chang N, 2015, CELL METAB, V21, P349, DOI 10.1016/j.cmet.2015.02.011
Singer MA, 2001, AM J KIDNEY DIS, V37, P164, DOI 10.1016/S0272-6386(01)80073-1
Sparrow DB, 2009, J AM SOC NEPHROL, V20, P777, DOI 10.1681/ASN.2008050547
Stangenberg S, 2015, AM J PHYSIOL-RENAL, V308, pF1189, DOI 10.1152/ajprenal.00638.2014
Stearns SC, 2020, J MOL EVOL, V88, P3, DOI 10.1007/s00239-019-09893-5
Stelloh C, 2012, TRANSL RES, V159, P80, DOI 10.1016/j.trsl.2011.10.004
Susztak K, 2014, J AM SOC NEPHROL, V25, P10, DOI 10.1681/ASN.2013050461
Sutherland MR, 2011, J AM SOC NEPHROL, V22, P1365, DOI 10.1681/ASN.2010121266
Thayer ZM, 2020, EVOL MED PUBLIC HLTH, P14, DOI 10.1093/emph/eoz037
Ungar PS, 2006, ANNU REV ANTHROPOL, V35, P209, DOI 10.1146/annurev.anthro.35.081705.123153
Volovelsky O, 2018, P NATL ACAD SCI USA, V115, P5998, DOI 10.1073/pnas.1712955115
Waker CA, 2017, STEM CELLS DEV, V26, P808, DOI 10.1089/scd.2016.0252
Wallace DC, 2011, COLD SH Q B, V76, P1, DOI 10.1101/sqb.2011.76.010462
Wallace DC, 2009, COLD SH Q B, V74, P383, DOI 10.1101/sqb.2009.74.031
Wallace DC, 2016, NATURE, V535, P498, DOI 10.1038/nature18902
Wallace DC, 2013, PHILOS T R SOC B, V368, DOI 10.1098/rstb.2012.0267
Wallace DC, 2013, J CLIN INVEST, V123, P1405, DOI 10.1172/JCI61398

   Wallace DC, 2010, P NATL ACAD SCI USA, V107, P8947, DOI
10.1073/pnas.0914635107
   Wallace DC, 2010, MITOCHONDRION, V10, P12, DOI
10.1016/j.mito.2009.09.006
   Wanner N, 2019, J AM SOC NEPHROL, V30, P63, DOI 10.1681/ASN.2018070736
   White SL, 2009, AM J KIDNEY DIS, V54, P248, DOI
10.1053/j.ajkd.2008.12.042
   WHO, 2018, WHO Falls Factsheet WHO
   WILLIAMS GC, 1991, Q REV BIOL, V66, P1, DOI 10.1086/417048
   Yim HE, 2014, PEDIATR RES, V75, P259, DOI 10.1038/pr.2013.223
NR 148
TC 12
Z9 12
U1 0
U2 1
PU AMER SOC NEPHROLOGY
PI WASHINGTON
PA 1401 H Street NW, Suite 900, WASHINGTON, DC 20005, UNITED STATES
SN 2641-7650
J9 KIDNEY360
JI Kidney360
PD AUG
PY 2020
VL 1
IS 8
BP 863
EP 879
DI 10.34067/KID.0002012020
PG 17
WC Urology & Nephrology
WE Emerging Sources Citation Index (ESCI)
SC Urology & Nephrology
GA VL8GY
UT WOS:000915413000020
PM 35372951
OA gold, Green Published
DA 2025-05-07
ER

PT J
AU Swanson, ZS
   Bethancourt, H
   Saunders, J
   Dhanasekar, S
   Nzunza, R
   Rosinger, AY
   Pontzer, H
AF Swanson, Z. S.
   Bethancourt, H.
   Saunders, J.
   Dhanasekar, S.
   Nzunza, R.
   Rosinger, A. Y.
   Pontzer, H.

TI The effect of lifestyle variation on health and life history in
   Daasanach pastoralists of Northern Kenya
SO AMERICAN JOURNAL OF HUMAN BIOLOGY
LA English
DT Meeting Abstract
C1 [Swanson, Z. S.; Saunders, J.; Pontzer, H.] Duke Univ, Dept
Evolutionary Anthropol, Durham, NC USA.
   [Bethancourt, H.; Rosinger, A. Y.] Penn State Univ, Dept Biobehav
Hlth, University Pk, PA 16802 USA.
   [Dhanasekar, S.] Emory Univ, Dept Anthropol, Atlanta, GA 30322 USA.
   [Nzunza, R.] Kenya Med Res Inst KEMRI, Nairobi, Kenya.
   [Rosinger, A. Y.] Penn State Univ, Dept Anthropol, State Coll, PA USA.
   [Pontzer, H.] Duke Univ, Global Hlth Inst, Durham, NC USA.
C3 Duke University; Pennsylvania Commonwealth System of Higher Education
   (PCSHE); Pennsylvania State University; Pennsylvania State University
-
   University Park; Penn State Behrend; Emory University; Kenya Medical
   Research Institute; Pennsylvania Commonwealth System of Higher
Education
   (PCSHE); Pennsylvania State University; Pennsylvania State University
-
   University Park; Duke University
RI S, DHANASEKAR/GNH-2305-2022
FU NSF ARCH [1624398]; NSF REU [1930719]; PSU SSRI Human Health and
   Environment Seed Grant; Triangle Center for Evolutionary Medicine
   (TriCEM) Graduate Research Grant
FX NSF ARCH #1624398; NSF REU #1930719; This work was funded by the PSU
   SSRI Human Health and Environment Seed Grant, and the Triangle Center
   for Evolutionary Medicine (TriCEM) Graduate Research Grant.
NR 0
TC 0
Z9 0
U1 0
U2 1
PU WILEY
PI HOBOKEN
PA 111 RIVER ST, HOBOKEN 07030-5774, NJ USA
SN 1042-0533
EI 1520-6300
J9 AM J HUM BIOL
JI Am. J. Hum. Biol.
PD APR
PY 2020
VL 32
SU 1
MA P/AM 65
PG 1
WC Anthropology; Biology
WE Science Citation Index Expanded (SCI-EXPANDED); Social Science
Citation Index (SSCI)
SC Anthropology; Life Sciences & Biomedicine - Other Topics
GA LB2WU
UT WOS:000524499200136
DA 2025-05-07

ER

PT J
AU Somarelli, JA
AF Somarelli, Jason A.
TI The Hallmarks of Cancer as Ecologically Driven Phenotypes
SO FRONTIERS IN ECOLOGY AND EVOLUTION
LA English
DT Review
DE ideal free distribution; metastasis; tumor microenvironment; fitness;
niche construction theory
ID METASTATIC NICHE FORMATION; TUMOR-INDUCED IMMUNOSUPPRESSION;
EXTRACELLULAR-MATRIX; TRADE-OFFS; EVOLUTION; CHEMOTHERAPY; SURVIVAL;
HYPOXIA; CELLS; MICROENVIRONMENT
AB Ecological fitness is the ability of individuals in a population to survive and reproduce. Individuals with increased fitness are better equipped to withstand the selective pressures of their environments. This paradigm pertains to all organismal life as we know it; however, it is also becoming increasingly clear that within multicellular organisms exist highly complex, competitive, and cooperative populations of cells under many of the same ecological and evolutionary constraints as populations of individuals in nature. In this review I discuss the parallels between populations of cancer cells and populations of individuals in the wild, highlighting how individuals in either context are constrained by their environments to converge on a small number of critical phenotypes to ensure survival and future reproductive success. I argue that the hallmarks of cancer can be distilled into key phenotypes necessary for cancer cell fitness: survival and reproduction. I posit that for therapeutic strategies to be maximally beneficial, they should seek to subvert these ecologically driven phenotypic responses.
C1 [Somarelli, Jason A.] Duke Univ, Med Ctr, Dept Med, Duke Canc Inst, Durham, NC 27710 USA.
C3 Duke University
RP Somarelli, JA (autor correspondente), Duke Univ, Med Ctr, Dept Med, Duke Canc Inst, Durham, NC 27710 USA.
EM jason.somarelli@duke.edu
OI Somarelli, Jason/0000-0003-1510-9343
FU Triangle Center for Evolutionary Medicine (TriCEM); Department of
Defense [W81XWH-18-1-0189]; National Cancer Institute [5R01CA233585-02]
FX JS receives funding support from the Triangle Center for Evolutionary
Medicine (TriCEM), the Department of Defense (W81XWH-18-1-0189), and the
National Cancer Institute (5R01CA233585-02).
CR Aguirre-Ghiso JA, 2003, CANCER RES, V63, P1684
Aktipis CA, 2015, PHILOS T R SOC B, V370, DOI 10.1098/rstb.2014.0219
Alkasalias T, 2018, INT J MOL SCI, V19, DOI 10.3390/ijms19051532
Alumkal JJ, 2020, P NATL ACAD SCI USA, V117, P12315, DOI 10.1073/pnas.1922207117
Andreoiu M, 2010, HUM PATHOL, V41, P781, DOI 10.1016/j.humpath.2010.02.011
Armstrong AJ, 2019, J CLIN ONCOL, V37, P1120, DOI 10.1200/JCO.18.01731
Augoff K, 2020, ANN TRANSL MED, V8, DOI 10.21037/atm.2020.02.157

Baghdadi M, 2016, CANCER RES, V76, P6030, DOI 10.1158/0008-5472.CAN-16-1170
Bandari SK, 2018, MATRIX BIOL, V65, P104, DOI 10.1016/j.matbio.2017.09.001
Banh RS, 2020, CELL, V183, P1202, DOI 10.1016/j.cell.2020.10.016
Bashashati A, 2013, J PATHOL, V231, P21, DOI 10.1002/path.4230
Ben-Baruch A, 2006, SEMIN CANCER BIOL, V16, P38, DOI 10.1016/j.semcancer.2005.07.006
Bergers G, 2003, NAT REV CANCER, V3, P401, DOI 10.1038/nrc1093
Binnewies M, 2018, NAT MED, V24, P541, DOI 10.1038/s41591-018-0014-x
Bragado P, 2013, NAT CELL BIOL, V15, P1351, DOI 10.1038/ncb2861
Caswell-Jin JL, 2019, NAT COMMUN, V10, DOI 10.1038/s41467-019-08593-4
Chen J, 2011, PLOS ONE, V6, DOI 10.1371/journal.pone.0017933
Childers CL, 2019, PEERJ, V7, DOI 10.7717/peerj.7587
Chin DD, 2016, J EXP BIOL, V219, P920, DOI 10.1242/jeb.042317
Cipponi A, 2020, SCIENCE, V368, P1127, DOI 10.1126/science.aau8768
Costa-Silva B, 2015, NAT CELL BIOL, V17, P816, DOI 10.1038/ncb3169
Cunningham J, 2020, PLOS ONE, V15, DOI 10.1371/journal.pone.0243386
Das A, 2017, SCI REP-UK, V7, DOI 10.1038/s41598-017-14340-w
De Saedeleer CJ, 2014, ONCOGENE, V33, P4060, DOI 10.1038/onc.2013.454
Ding L, 2012, NATURE, V481, P506, DOI 10.1038/nature10738
Doglioni G, 2019, FRONT ONCOL, V9, DOI 10.3389/fonc.2019.00219
Eddy SF, 2007, CELL BIOCHEM FUNCT, V25, P759, DOI 10.1002/cbf.1416
Enriquez-Navas PM, 2016, SCI TRANSL MED, V8, DOI 10.1126/scitranslmed.aad7842
Erler JT, 2009, CANCER CELL, V15, P35, DOI 10.1016/j.ccr.2008.11.012
Fish FE, 2017, J EXP BIOL, V220, P4351, DOI 10.1242/jeb.149617
Fong MY, 2015, NAT CELL BIOL, V17, P183, DOI 10.1038/ncb3094
Foo W.-C., 2020, CONVERGENT EVOLUTION, DOI [10.1101/2020.04.22.050385, DOI 10.1101/2020.04.22.050385]
Fortunato A, 2017, CSH PERSPECT MED, V7, DOI 10.1101/cshperspect.a029652
FRETWELL S D, 1969, Acta Biotheoretica, V19, P16, DOI 10.1007/BF01601953
Friman VP, 2008, P ROY SOC B-BIOL SCI, V275, P1625, DOI 10.1098/rspb.2008.0174
Gardner Renee V., 1999, Frontiers in Bioscience, V4, pe47, DOI 10.2741/Gardner
Gatenby RA, 2011, NAT REV CANCER, V11, P237, DOI 10.1038/nrc3036
Gérard M, 2019, FRONT MED-LAUSANNE, V6, DOI 10.3389/fmed.2019.00117
Goldman A, 2019, SCI SIGNAL, V12, DOI 10.1126/scisignal.aas8779
Grange C, 2011, CANCER RES, V71, P5346, DOI 10.1158/0008-5472.CAN-11-0241
Gremke N, 2020, NAT COMMUN, V11, DOI 10.1038/s41467-020-18504-7
Griffiths JI, 2020, P NATL ACAD SCI USA, V117, P16072, DOI 10.1073/pnas.1918937117
Grignon DJ, 1996, CANCER RES, V56, P1654
Gupta S, 2020, GENE CHROMOSOME CANC, V59, P225, DOI 10.1002/gcc.22824
Gupta S, 2017, CLIN CANCER RES, V23, P1346, DOI 10.1158/1078-0432.CCR-16-1211
Hamanoue M, 2016, SCI REP-UK, V6, DOI 10.1038/srep24279
Hanahan D, 2011, CELL, V144, P646, DOI 10.1016/j.cell.2011.02.013
Heaster TM, 2019, FRONT ONCOL, V9, DOI 10.3389/fonc.2019.01144
Hida K, 2018, INT J MOL SCI, V19, DOI 10.3390/ijms19051272

Hori SS, 2011, SCI TRANSL MED, V3, DOI 10.1126/scitranslmed.3003110
Hoshino A, 2015, NATURE, V527, P329, DOI 10.1038/nature15756
Huang H, 2018, SENSORS-BASEL, V18, DOI 10.3390/s18103249
Hutley L.B., 2018, Encyclopedia of ecology, P623
Ignatiadis M, 2021, NAT REV CLIN ONCOL, V18, P297, DOI 10.1038/s41571-020-00457-x
Jin MZ, 2020, SIGNAL TRANSDUCT TAR, V5, DOI 10.1038/s41392-020-00280-x
Johnson BE, 2014, SCIENCE, V343, P189, DOI 10.1126/science.1239947
Jolly MK, 2019, PHARMACOL THERAPEUT, V194, P161, DOI 10.1016/j.pharmthera.2018.09.007
Juárez P, 2012, CANCER RES, V72, P6247, DOI 10.1158/0008-5472.CAN-12-1444
Kam Y, 2014, WOMENS HEALTH, V10, P423, DOI [10.2217/WHE.14.23, 10.2217/whe.14.23]
Kaplan RN, 2005, NATURE, V438, P820, DOI 10.1038/nature04186
Kelly DW, 2000, PARASITOLOGY, V120, P319, DOI 10.1017/S0031182099005442
Kim R, 2006, CANCER RES, V66, P5527, DOI 10.1158/0008-5472.CAN-05-4128
Klein CA, 2020, NAT REV CANCER, V20, P681, DOI 10.1038/s41568-020-00300-6
Klein CA, 2011, CURR OPIN GENET DEV, V21, P42, DOI 10.1016/j.gde.2010.10.011
Krause M, 2015, CELL ADHES MIGR, V9, P357, DOI 10.1080/19336918.2015.1061173
Kurppa KJ, 2020, CANCER CELL, V37, P104, DOI 10.1016/j.ccell.2019.12.006
Kyriakis JM, 1996, J BIOL CHEM, V271, P24313, DOI 10.1074/jbc.271.40.24313
Laland K, 2016, EVOL ECOL, V30, P191, DOI 10.1007/s10682-016-9821-z
Laroche F, 2016, P ROY SOC B-BIOL SCI, V283, DOI 10.1098/rspb.2016.0548
Li H, 2017, ONCOL LETT, V14, P5865, DOI 10.3892/ol.2017.6924
Li Y, 2021, CANCER LETT, V497, P212, DOI 10.1016/j.canlet.2020.10.032
Liang XJ, 2014, J LEUKOCYTE BIOL, V95, P179, DOI 10.1189/jlb.0613330
Liu YF, 2016, CANCER CELL, V30, P243, DOI 10.1016/j.ccell.2016.06.021
Loud JT, 2017, SEMIN ONCOL NURS, V33, P121, DOI 10.1016/j.soncn.2017.02.002
Lue HW, 2017, GENE DEV, V31, P2067, DOI 10.1101/gad.305292.117
Matthysen E, 2005, ECOGRAPHY, V28, P403, DOI 10.1111/j.0906-7590.2005.04073.x
Milosevic MF, 1999, INT J RADIAT ONCOL, V43, P1111, DOI 10.1016/S0360-3016(98)00512-4
Moses MM, 2013, EVOL ECOL RES, V15, P809
Mu W, 2013, NEOPLASIA, V15, P875, DOI 10.1593/neo.13786
NAIMAN RJ, 1988, BIOSCIENCE, V38, P753, DOI 10.2307/1310784
Niven JE, 2008, CURR BIOL, V18, pR27, DOI 10.1016/j.cub.2007.11.020
Odling-Smee J, 2013, Q REV BIOL, V88, P3, DOI 10.1086/669266
Opzoomer JW, 2019, FRONT IMMUNOL, V10, DOI 10.3389/fimmu.2019.01654
Pardoll DM, 2012, NAT REV CANCER, V12, P252, DOI 10.1038/nrc3239
Peinado H, 2017, NAT REV CANCER, V17, P302, DOI 10.1038/nrc.2017.6
Pellissier L, 2015, FRONT ECOL EVOL, V3, DOI 10.3389/fevo.2015.00052
Protas ME, 2006, NAT GENET, V38, P107, DOI 10.1038/ng1700
Psaila B, 2009, NAT REV CANCER, V9, P285, DOI 10.1038/nrc2621
Rana S, 2013, NEOPLASIA, V15, P281, DOI 10.1593/neo.122010

Recasens A, 2019, TRENDS PHARMACOL SCI, V40, P128, DOI 10.1016/j.tips.2018.12.004
Rijken PFJW, 2000, INT J RADIAT ONCOL, V48, P571, DOI 10.1016/S0360-3016(00)00686-6
Roper N, 2020, CELL REP MED, V1, DOI 10.1016/j.xcrm.2020.100007
Sahai E, 2020, NAT REV CANCER, V20, P174, DOI 10.1038/s41568-019-0238-1
Schaper S, 2014, PLOS ONE, V9, DOI 10.1371/journal.pone.0086635
Schmitz Oswald, 2017, F1000Res, V6, P1767, DOI 10.12688/f1000research.11813.1
Schmitz OJ, 2016, CURR OPIN BEHAV SCI, V12, P6, DOI 10.1016/j.cobeha.2016.08.003
Shah PP, 2017, ONCOTARGET, V8, P22625, DOI 10.18632/oncotarget.15150
Shen SS, 2020, CELL, V183, P860, DOI 10.1016/j.cell.2020.10.027
Shieh Y, 2016, NAT REV CLIN ONCOL, V13, P550, DOI 10.1038/nrclinonc.2016.50
Sier CFM, 1996, BRIT J CANCER, V74, P413, DOI 10.1038/bjc.1996.374
Silva AS, 2012, CANCER RES, V72, P6362, DOI 10.1158/0008-5472.CAN-12-2235
Somarelli JA, 2020, MOL BIOL EVOL, V37, P320, DOI 10.1093/molbev/msz242
Somarelli JA, 2017, BBA-REV CANCER, V1867, P101, DOI 10.1016/j.bbcan.2016.10.006
Sun X, 2017, ONCOTARGET, V8, P15168, DOI 10.18632/oncotarget.14834
Sun Y, 2012, NAT MED, V18, P1359, DOI 10.1038/nm.2890
Swann JB, 2007, J CLIN INVEST, V117, P1137, DOI 10.1172/JCI31405
Takahashi Y, 2018, P ROY SOC B-BIOL SCI, V285, DOI 10.1098/rspb.2017.2045
Takenaka K, 1998, SCIENCE, V280, P599, DOI 10.1126/science.280.5363.599
Takeuchi S, 2015, CANCER RES, V75, P2629, DOI 10.1158/0008-5472.CAN-14-2921
Ware KE, 2016, ONCOTARGET, V7, P50520, DOI 10.18632/oncotarget.10476
Wen SW, 2016, CANCER RES, V76, P6816, DOI 10.1158/0008-5472.CAN-16-0868
West J, 2018, J THEOR BIOL, V455, P249, DOI 10.1016/j.jtbi.2018.07.028
Willis CKR, 2017, INTEGR COMP BIOL, V57, P1214, DOI 10.1093/icb/icx087
Winkler J, 2020, NAT COMMUN, V11, DOI 10.1038/s41467-020-18794-x
Wojtowicz-Praga S, 2003, INVEST NEW DRUG, V21, P21, DOI 10.1023/A:1022951824806
Wu JJ, 2018, CANCER LETT, V419, P210, DOI 10.1016/j.canlet.2018.01.050
Xu S, 2019, J CLIN MED, V8, DOI 10.3390/jcm8020205
Yang MH, 2008, NAT CELL BIOL, V10, P295, DOI 10.1038/ncb1691
Yawata Y, 2014, P NATL ACAD SCI USA, V111, P5622, DOI 10.1073/pnas.1318943111
Zaidi M, 2019, FRONT BIOENG BIOTECH, V7, DOI 10.3389/fbioe.2019.00397
Zeng ZC, 2018, NAT COMMUN, V9, DOI 10.1038/s41467-018-07810-w
Zhang X, 2017, NAT COMMUN, V8, DOI [10.1038/ncomms14542, 10.1038/ncomms15280]
Zhou WY, 2014, CANCER CELL, V25, P501, DOI 10.1016/j.ccr.2014.03.007
NR 123
TC 25
Z9 26
U1 2

U2 8
PU FRONTIERS MEDIA SA
PI LAUSANNE
PA AVENUE DU TRIBUNAL FEDERAL 34, LAUSANNE, CH-1015, SWITZERLAND
SN 2296-701X
J9 FRONT ECOL EVOL
JI Front. Ecol. Evol.
PD APR 28
PY 2021
VL 9
AR 661583
DI 10.3389/fevo.2021.661583
PG 11
WC Ecology
WE Science Citation Index Expanded (SCI-EXPANDED)
SC Environmental Sciences & Ecology
GA SA2AQ
UT WOS:000649102900001
PM 34703824
OA Green Accepted, gold
DA 2025-05-07
ER

PT J
AU Swanson, ZS
   Bethancourt, H
   Saunders, J
   Nzunza, R
   Rosinger, AY
   Pontzer, H
AF Swanson, Zane S.
   Bethancourt, Hilary
   Saunders, Jessica
   Nzunza, Rosemary
   Rosinger, Asher Y.
   Pontzer, Herman
TI Distinct early growth trajectory among Daasanach pastoralists of
   Northern Kenya
SO AMERICAN JOURNAL OF PHYSICAL ANTHROPOLOGY
LA English
DT Meeting Abstract
CT 89th Annual Meeting of the
   American-Association-of-Physical-Anthropologists (AAPA)
CY APR 15-18, 2020
CL Los Angeles, CA
SP Amer Assoc Phys Anthropologists
C1 [Swanson, Zane S.; Saunders, Jessica; Pontzer, Herman] Duke Univ,
Evolutionary Anthropol, Durham, NC 27706 USA.
   [Bethancourt, Hilary; Rosinger, Asher Y.] Penn State Univ, Biobehav
Hlth, University Pk, PA 16802 USA.
   [Nzunza, Rosemary] Kenya Govt Med Res Ctr, KEMRI, Nairobi, Kenya.
   [Rosinger, Asher Y.] Penn State Univ, Anthropol, University Pk, PA
16802 USA.
   [Pontzer, Herman] Duke Univ, Global Hlth Inst, Durham, NC 27706 USA.

C3 Duke University; Pennsylvania Commonwealth System of Higher Education
   (PCSHE); Pennsylvania State University; Pennsylvania State University
-
   University Park; Penn State Behrend; Kenya Medical Research Institute;
   Pennsylvania Commonwealth System of Higher Education (PCSHE);
   Pennsylvania State University; Pennsylvania State University -
   University Park; Penn State Behrend; Duke University
FU NSF ARCH [1624398]; NSF REU [1930719]; PSU SSRI Human Health and
   Environment Seed Grant; Triangle Center for Evolutionary Medicine
   (TriCEM) Graduate Research Grant
FX NSF ARCH #1624398; NSF REU #1930719; This work was funded by the PSU
   SSRI Human Health and Environment Seed Grant, and the Triangle Center
   for Evolutionary Medicine (TriCEM) Graduate Research Grant.
NR 0
TC 0
Z9 0
U1 0
U2 3
PU WILEY
PI HOBOKEN
PA 111 RIVER ST, HOBOKEN 07030-5774, NJ USA
SN 0002-9483
EI 1096-8644
J9 AM J PHYS ANTHROPOL
JI Am. J. Phys. Anthropol.
PD MAR
PY 2020
VL 171
SU 69
BP 278
EP 278
PG 1
WC Anthropology; Evolutionary Biology
WE Science Citation Index Expanded (SCI-EXPANDED); Social Science
Citation Index (SSCI); Conference Proceedings Citation Index - Science
(CPCI-S)
SC Anthropology; Evolutionary Biology
GA KL2WI
UT WOS:000513288903114
DA 2025-05-07
ER

PT J
AU Chitadze, G
   Wehkamp, U
   Janssen, O
   Brüggemann, M
   Lettau, M
AF Chitadze, Guranda
   Wehkamp, Ulrike
   Janssen, Ottmar
   Bruggemann, Monika
   Lettau, Marcus
TI The Serine Protease CD26/DPP4 in Non-Transformed and Malignant T Cells

SO CANCERS
LA English
DT Review
DE CD26; DPP4; dipeptidyl peptidase 4; T cells; cytotoxic granules; cutaneous T-cell lymphoma; mycosis fungoides; Sezary syndrome
ID DIPEPTIDYL PEPTIDASE-IV; MYCOSIS-FUNGOIDES; CD26 EXPRESSION; SEZARY-SYNDROME; ADENOSINE-DEAMINASE; FAS LIGAND; MONOCLONAL-ANTIBODY; DENDRITIC CELLS; FLOW-CYTOMETRY; CANCER-RISK
AB CD26/Dipeptidylpeptidase 4 is a transmembrane serine protease that cleaves off N-terminal dipeptides. CD26/DPP4 is expressed on several immune cell types including T and NK cells, dendritic cells, and activated B cells. A catalytically active soluble form of CD26/DPP4 can be released from the plasma membrane. Given its wide array of substrates and interaction partners CD26/DPP4 has been implicated in numerous biological processes and effects can be dependent or independent of its enzymatic activity and are exerted by the transmembrane protein and/or the soluble form. CD26/DPP4 has been implicated in the modulation of T-cell activation and proliferation and CD26/DPP4-positive T cells are characterized by remarkable anti-tumor properties rendering them interesting candidates for T cell-based immunotherapies. Moreover, especially in cutaneous T-cell lymphoma CD26/DPP4 expression patterns emerged as an established marker for diagnosis and treatment monitoring. Surprisingly, besides a profound knowledge on substrates, interaction partners, and associated signal transduction pathways, the precise role of CD26/DPP4 for T cell-based immune responses is only partially understood.
C1 [Chitadze, Guranda; Bruggemann, Monika; Lettau, Marcus] Univ Hosp Schleswig Holstein, Dept Internal Med 2, Unit Hematol Diagnost, Langer Segen 8-10, D-24105 Kiel, Germany.
[Wehkamp, Ulrike] Univ Hosp Schleswig Holstein, Dept Dermatol, Arnold Heller Str 3,Bldg C, D-24105 Kiel, Germany.
[Janssen, Ottmar; Lettau, Marcus] Christian Albrechts Univ Kiel, Univ Hosp Schleswig Holstein, Inst Immunol, Arnold Heller Str 3,Bldg U30, D-24105 Kiel, Germany.
C3 University of Kiel; Schleswig Holstein University Hospital; University of Kiel; Schleswig Holstein University Hospital; University of Kiel; Schleswig Holstein University Hospital
RP Lettau, M (autor correspondente), Univ Hosp Schleswig Holstein, Dept Internal Med 2, Unit Hematol Diagnost, Langer Segen 8-10, D-24105 Kiel, Germany.; Lettau, M (autor correspondente), Christian Albrechts Univ Kiel, Univ Hosp Schleswig Holstein, Inst Immunol, Arnold Heller Str 3,Bldg U30, D-24105 Kiel, Germany.
EM Guranda.Chitadze@uksh.de; uwehkamp@dermatology.uni-kiel.de; Ottmar.Janssen@uksh.de; m.brueggemann@med2.uni-kiel.de; marcus.lettau@uksh.de
RI Lettau, Marcus/H-7595-2019; Chitadze, Guranda/ABA-4545-2020; Janssen, Ottmar/E-9735-2010
OI Lettau, Marcus/0000-0002-3824-9923; Janssen, Ottmar/0000-0002-9612-8900
FU Deutsche Krebshilfe [70113252]; Deutsche Jose Carreras Leukamie-Stiftung
[DJCLS 22R/2019]; Deutsche Forschungsgemeinschaft [JA 610/7-3]; clinician scientist program in evolutionary medicine [413490537]
FX This research was funded by Deutsche Krebshilfe, grant number 70113252

(to G.C., M.B.), Deutsche Jose Carreras Leukamie-Stiftung, grant number
DJCLS 22R/2019 (to G.C., M.B., O.J.), the Deutsche Forschungsgemeinschaft, grant number JA 610/7-3 (to O.J.) and the "clinician scientist program in evolutionary medicine", grant number 413490537 (to M.B.).
CR ABBOTT CA, 1994, IMMUNOGENETICS, V40, P331, DOI 10.1007/BF01246674
Abeni D, 2005, BRIT J DERMATOL, V153, P324, DOI 10.1111/j.1365-2133.2005.06755.x
Ali A, 2019, MOL CLIN ONCOL, V10, P118, DOI 10.3892/mco.2018.1766
Angevin E, 2017, BRIT J CANCER, V116, P1126, DOI 10.1038/bjc.2017.62
Apetoh L, 2007, NAT MED, V13, P1050, DOI 10.1038/nm1622
Arscott WT, 2009, ONCOGENE, V28, P479, DOI 10.1038/onc.2008.402
Bailey SR, 2017, NAT COMMUN, V8, DOI 10.1038/s41467-017-01867-9
Baum W, 2005, J BIOL CHEM, V280, P40012, DOI 10.1074/jbc.M502222200
Beckenkamp A, 2016, TUMOR BIOL, V37, P7059, DOI 10.1007/s13277-016-5005-2
Bernengo MG, 2001, BRIT J DERMATOL, V144, P125, DOI 10.1046/j.1365-2133.2001.04014.x
Bishnoi R, 2019, CANCER MED-US, V8, P3918, DOI 10.1002/cam4.2278
Boniol M, 2018, DIABETES CARE, V41, P286, DOI 10.2337/dc17-0280
Boonacker E, 2003, EUR J CELL BIOL, V82, P53, DOI 10.1078/0171-9335-00302
Britton C, 2021, FASEB J, V35, DOI 10.1096/fj.202001273R
CARBONE A, 1995, BLOOD, V86, P4617, DOI 10.1182/blood.V86.12.4617.bloodjournal86124617
Casey TM, 2007, MOL CELL PROTEOMICS, V6, P767, DOI 10.1074/mcp.M600365-MCP200
Casrouge A, 2018, CLIN EXP IMMUNOL, V194, P166, DOI 10.1111/cei.13163
Cedeno-Laurent F, 2017, J CUTAN PATHOL, V44, P462, DOI 10.1111/cup.12899
Cekic C, 2016, NAT REV IMMUNOL, V16, P177, DOI 10.1038/nri.2016.4
Cheng HC, 2003, J BIOL CHEM, V278, P24600, DOI 10.1074/jbc.M303424200
Choi YJ, 2019, CANCER MANAG RES, V11, P7427, DOI 10.2147/CMAR.S215107
Chung KM, 2010, PROTEIN SCI, V19, P1627, DOI 10.1002/pro.443
Cordero OJ, 2015, PLOS ONE, V10, DOI 10.1371/journal.pone.0131992
Cordero OJ, 2009, CANCER IMMUNOL IMMUN, V58, P1725, DOI 10.1007/s00262-009-0728-1
Coutinho MF, 2012, MOL GENET METAB, V105, P542, DOI 10.1016/j.ymgme.2011.12.012
Crump MP, 1997, EMBO J, V16, P6996, DOI 10.1093/emboj/16.23.6996
Dang NH, 2002, HISTOL HISTOPATHOL, V17, P1213, DOI 10.14670/HH-17.1213
Davoodi J, 2007, PROTEOMICS, V7, P2300, DOI 10.1002/pmic.200600654
De Andrade CFCG, 2009, J ENZYM INHIB MED CH, V24, P708, DOI 10.1080/14756360802334800
De Zutter A, 2021, CANCERS, V13, DOI 10.3390/cancers13174247
Decalf J, 2016, EMBO MOL MED, V8, P679, DOI 10.15252/emmm.201506145
Dicembrini I, 2020, ACTA DIABETOL, V57, P689, DOI 10.1007/s00592-020-01479-8
DINJENS WNM, 1989, J BIOL CHEM, V264, P19215
Dong RP, 1996, J IMMUNOL, V156, P1349
Durinx C, 2000, EUR J BIOCHEM, V267, P5608, DOI 10.1046/j.1432-1327.2000.01634.x

Enz N, 2019, PHARMACOL THERAPEUT, V198, P135, DOI 10.1016/j.pharmthera.2019.02.015
FUKUI Y, 1990, CELL STRUCT FUNCT, V15, P117, DOI 10.1247/csf.15.117
Gdynia G, 2016, NAT COMMUN, V7, DOI 10.1038/ncomms10764
Gdynia G, 2010, CANCER RES, V70, P8558, DOI 10.1158/0008-5472.CAN-10-0204
GRIFFITHS GM, 1993, J CELL BIOL, V120, P885, DOI 10.1083/jcb.120.4.885
Hatano R, 2013, IMMUNOLOGY, V138, P165, DOI 10.1111/imm.12028
Hegen M, 1997, IMMUNOLOGY, V90, P257, DOI 10.1046/j.1365-2567.1997.00053.x
Herrera C, 2001, J BIOL CHEM, V276, P19532, DOI 10.1074/jbc.M004586200
HONG WJ, 1989, EXP CELL RES, V182, P256, DOI 10.1016/0014-4827(89)90296-6
Hristov AC, 2011, AM J CLIN PATHOL, V136, P944, DOI 10.1309/AJCP09OTJOYAVZZK
Ikushima H, 2000, P NATL ACAD SCI USA, V97, P8439, DOI 10.1073/pnas.97.15.8439
Ikushima H, 2002, CELL IMMUNOL, V215, P106, DOI 10.1016/S0008-8749(02)00010-2
Ishii T, 2001, P NATL ACAD SCI USA, V98, P12138, DOI 10.1073/pnas.211439098
Iwata S, 1999, INT IMMUNOL, V11, P417, DOI 10.1093/intimm/11.3.417
Jones D, 2001, AM J CLIN PATHOL, V115, P885
KAMEOKA J, 1993, SCIENCE, V261, P466, DOI 10.1126/science.8101391
Kelemen K, 2008, AM J CLIN PATHOL, V129, P146, DOI 10.1309/05GFG3LY3VYCDMEY
Kitagawa N, 2018, J CLIN BIOCHEM NUTR, V63, P106, DOI 10.3164/jcbn.17-64
Klemann C, 2016, CLIN EXP IMMUNOL, V185, P1, DOI 10.1111/cei.12781
Klune JR, 2008, MOL MED, V14, P476, DOI 10.2119/2008-00034.Klune
Knochelmann HM, 2020, INT J RADIAT ONCOL, V106, P1132
Kwak MS, 2020, FRONT IMMUNOL, V11, DOI 10.3389/fimmu.2020.01189
Lam CSC, 2014, PLOS ONE, V9, DOI 10.1371/journal.pone.0098582
Lankas GR, 2005, DIABETES, V54, P2988, DOI 10.2337/diabetes.54.10.2988
Lee M, 2019, DIABETES CARE, V42, P2057, DOI 10.2337/dc18-2195
Lettau M, 2006, P NATL ACAD SCI USA, V103, P5911, DOI 10.1073/pnas.0508562103
Lettau M, 2015, SCAND J IMMUNOL, V82, P235, DOI 10.1111/sji.12337
Lettau M, 2020, CELL MOL LIFE SCI, V77, P751, DOI 10.1007/s00018-019-03207-0
LOSTER K, 1995, BIOCHEM BIOPH RES CO, V217, P341, DOI 10.1006/bbrc.1995.2782
Lotze MT, 2005, NAT REV IMMUNOL, V5, P331, DOI 10.1038/nri1594
Lu GW, 2013, NATURE, V500, P227, DOI 10.1038/nature12328
Marchetti C, 2012, DIABETOLOGIA, V55, P236, DOI 10.1007/s00125-011-2213-6
Martinez-Navio JM, 2011, J LEUKOCYTE BIOL, V89, P127, DOI 10.1189/jlb.1009696
MATTERN T, 1995, IMMUNOLOGY, V84, P595
Miyagaki T, 2013, J EUR ACAD DERMATOL, V27, P19, DOI 10.1111/j.1468-3083.2011.04340.x
Moffitt LR, 2020, INT J MOL SCI, V21, DOI 10.3390/ijms21218110
Mulvihill EE, 2017, CELL METAB, V25, P152, DOI 10.1016/j.cmet.2016.10.007

Mulvihill EE, 2014, ENDOCR REV, V35, P992, DOI 10.1210/er.2014-1035
Namburi S, 2021, LEUKEMIA, V35, P1925, DOI 10.1038/s41375-020-01047-7
Narducci MG, 2006, BLOOD, V107, P1108, DOI 10.1182/blood-2005-04-1492
Nargis T, 2017, MOL METAB, V6, P1529, DOI 10.1016/j.molmet.2017.09.004
Nelson MH, 2020, SCI ADV, V6, DOI 10.1126/sciadv.aba7443
Nishikawa H, 2010, INT J CANCER, V127, P759, DOI 10.1002/ijc.25429
Novelli M, 2003, ADV EXP MED BIOL, V524, P223
Novelli M, 2015, AM J CLIN PATHOL, V143, P57, DOI 10.1309/AJCP1NA3YCHCDEIG
Oderup C, 2006, IMMUNOLOGY, V118, P240, DOI 10.1111/j.1365-2567.2006.02362.x
Ohnuma K, 2004, P NATL ACAD SCI USA, V101, P14186, DOI 10.1073/pnas.0405266101
Ohnuma K, 2008, TRENDS IMMUNOL, V29, P295, DOI 10.1016/j.it.2008.02.010
Ohnuma K, 2007, J BIOL CHEM, V282, P10117, DOI 10.1074/jbc.M609157200
Ohnuma K, 2009, BIOCHEM BIOPH RES CO, V386, P327, DOI 10.1016/j.bbrc.2009.06.027
Ohta A, 2014, FRONT IMMUNOL, V5, P1, DOI 10.3389/fimmu.2014.00304
Olsen E, 2007, BLOOD, V110, P1713, DOI 10.1182/blood-2007-03-055749
Pacheco R, 2005, P NATL ACAD SCI USA, V102, P9583, DOI 10.1073/pnas.0501050102
Pan K, 2021, CUREUS J MED SCIENCE, V13, DOI 10.7759/cureus.14712
Pearce Erika L, 2013, Science, V342, P1242454, DOI 10.1126/science.1242454
POULSEN MD, 1993, J HISTOCHEM CYTOCHEM, V41, P81, DOI 10.1177/41.1.8093256
Proost P, 1998, J BIOL CHEM, V273, P7222, DOI 10.1074/jbc.273.13.7222
Qian J, 2006, CELL SIGNAL, V18, P1327, DOI 10.1016/j.cellsig.2005.10.015
Röhrborn D, 2015, FRONT IMMUNOL, V6, P1, DOI 10.3389/fimmu.2015.00386
Röhrborn D, 2014, FEBS LETT, V588, P3870, DOI 10.1016/j.febslet.2014.08.029
Rudy W, 1997, INT IMMUNOL, V9, P853, DOI 10.1093/intimm/9.6.853
Saïdi H, 2008, PLOS ONE, V3, DOI 10.1371/journal.pone.0003601
Salgado FJ, 2012, CYTOM PART A, V81A, P843, DOI 10.1002/cyto.a.22117
Sanchez J, 2020, METABOLITES, V10, DOI 10.3390/metabo10110461
Sato T, 2005, CANCER RES, V65, P6950, DOI 10.1158/0008-5472.CAN-05-0647
Sauer AV, 2012, FRONT IMMUNOL, V3, DOI 10.3389/fimmu.2012.00265
Saulite I, 2020, ONCOIMMUNOLOGY, V9, DOI 10.1080/2162402X.2020.1738797
Scala E, 1999, J INVEST DERMATOL, V113, P622, DOI 10.1046/j.1523-1747.1999.00718.x
Scarisbrick JJ, 2018, EUR J CANCER, V93, P47, DOI 10.1016/j.ejca.2018.01.076
Schmidt H, 2011, J PROTEOME RES, V10, P1603, DOI 10.1021/pr100967v
Schmidt H, 2011, CELL COMMUN SIGNAL, V9, DOI 10.1186/1478-811X-9-4
SCHON E, 1989, SCAND J IMMUNOL, V29, P127, DOI 10.1111/j.1365-3083.1989.tb01108.x
Shioda T, 1998, P NATL ACAD SCI USA, V95, P6331, DOI 10.1073/pnas.95.11.6331
Sokolowska-Wojdylo M, 2005, CLIN EXP DERMATOL, V30, P702, DOI 10.1111/j.1365-2230.2005.01904.x
Stinchcombe J, 2004, SCIENCE, V305, P55, DOI 10.1126/science.1095291

Takeda M, 2019, LUNG CANCER, V137, P64, DOI 10.1016/j.lungcan.2019.09.010
Theodoraki MN, 2018, CLIN EXP IMMUNOL, V194, P67, DOI 10.1111/cei.13157
Tian L, 2010, ENDOCRINOLOGY, V151, P3049, DOI 10.1210/en.2010-0068
TORIMOTO Y, 1991, J IMMUNOL, V147, P2514
Torrealba MP, 2018, ONCOTARGET, V9, P3497, DOI 10.18632/oncotarget.23334
Tseng CH, 2016, ONCOTARGET, V7, P24871, DOI 10.18632/oncotarget.8399
Ueki K, 2021, BMJ OPEN DIAB RES CA, V9, DOI 10.1136/bmjdrc-2020-001787
Vandersee S, 2015, J DTSCH DERMATOL GES, V13, P30, DOI 10.1111/ddg.12549
Vankadari N, 2020, EMERG MICROBES INFEC, V9, P601, DOI 10.1080/22221751.2020.1739565
Vonderheid EC, 2018, LEUKEMIA LYMPHOMA, V59, P330, DOI 10.1080/10428194.2017.1334123
Vora KA, 2009, BMC IMMUNOL, V10, DOI 10.1186/1471-2172-10-19
Voss Matthias, 2008, Cell Commun Signal, V6, P11, DOI 10.1186/1478-811X-6-11
Vural S, 2018, TURK J HEMATOL, V35, P35, DOI 10.4274/tjh.2016.0502
Wang HC, 1999, SCIENCE, V285, P248, DOI 10.1126/science.285.5425.248
Wei H, 2021, INT J BIOL SCI, V17, P163, DOI 10.7150/ijbs.53671
Wei J, 2017, FRONT IMMUNOL, V8, DOI 10.3389/fimmu.2017.00247
Wenzel J, 2001, FEBS LETT, V509, P255, DOI 10.1016/S0014-5793(01)03174-X
Wesley UV, 2004, INT J CANCER, V109, P855, DOI 10.1002/ijc.20091
Willheim M, 1997, J ALLERGY CLIN IMMUN, V100, P348, DOI 10.1016/S0091-6749(97)70248-3
WOOD GS, 1990, J AM ACAD DERMATOL, V22, P602, DOI 10.1016/0190-9622(90)70080-2
Wragg KM, 2020, CELL REP, V31, DOI 10.1016/j.celrep.2020.107773
Wyatt M.M., 2020, J IMMUNOL, V1, P72
Yang D, 2007, J LEUKOCYTE BIOL, V81, P59, DOI 10.1189/jlb.0306180
Yazbeck R, 2018, PROTOPLASMA, V255, P375, DOI 10.1007/s00709-017-1129-5
Yu DMT, 2011, SCAND J IMMUNOL, V73, P102, DOI 10.1111/j.1365-3083.2010.02488.x
Zetterström CK, 2002, PEDIATR RES, V52, DOI 10.1023/01.PDR.0000023175.89966.9E
Zhang Y, 2019, CELL BIOSCI, V9, DOI 10.1186/s13578-019-0282-2
Zhao XL, 2021, J IMMUNOL RES, V2021, DOI 10.1155/2021/6671410
Zheng Y, 2004, J IMMUNOL, V172, P2778, DOI 10.4049/jimmunol.172.5.2778
Zuccato E, 2007, J CELL SCI, V120, P191, DOI 10.1242/jcs.03315
NR 140
TC 15
Z9 15
U1 0
U2 2
PU MDPI
PI BASEL
PA ST ALBAN-ANLAGE 66, CH-4052 BASEL, SWITZERLAND
EI 2072-6694
J9 CANCERS
JI Cancers

PD DEC
PY 2021
VL 13
IS 23
AR 5947
DI 10.3390/cancers13235947
PG 18
WC Oncology
WE Science Citation Index Expanded (SCI-EXPANDED)
SC Oncology
GA ZJ1NY
UT WOS:000762080200001
PM 34885056
OA Green Published, gold
DA 2025-05-07
ER

PT J
AU Sheth, MU
   Kwartler, SK
   Schmaltz, ER
   Hoskinson, SM
   Martz, EJ
   Dunphy-Daly, MM
   Schultz, TF
   Read, AJ
   Eward, WC
   Somarelli, JA
AF Sheth, Maya U.
   Kwartler, Sarah K.
   Schmaltz, Emma R.
   Hoskinson, Sarah M.
   Martz, E. J.
   Dunphy-Daly, Meagan M.
   Schultz, Thomas F.
   Read, Andrew J.
   Eward, William C.
   Somarelli, Jason A.
TI Bioengineering a Future Free of Marine Plastic Waste
SO FRONTIERS IN MARINE SCIENCE
LA English
DT Review
DE bioremediation; PETase; polymer; degradation; pollution
ID CUTINASE-CATALYZED HYDROLYSIS; HIGH-DENSITY POLYETHYLENE; POLYESTER
   POLYURETHANE; ENZYMATIC DEGRADATION; BACTERIAL STRAINS; BRANCH
COMPOST;
   VINYL-CHLORIDE; BIODEGRADATION; TEREPHTHALATE;
   POLY(ETHYLENE-TEREPHTHALATE)
AB Plastic waste has reached epidemic proportions worldwide, and the
production of plastic continues to rise steadily. Plastic represents a
diverse array of commonly used synthetic polymers that are extremely
useful as durable, economically beneficial alternatives to other
materials; however, despite the wide-ranging utility of plastic, the
increasing accumulation of plastic waste in the environment has had

numerous detrimental impacts. In particular, plastic marine debris can transport invasive species, entangle marine organisms, and cause toxic chemical bioaccumulation in the marine food web. The negative impacts of plastic waste have motivated research on new ways to reduce and eliminate plastic. One unique approach to tackle the plastic waste problem is to turn to nature's solutions for degrading polymers by leveraging the biology of naturally occurring organisms to degrade plastic. Advances in metagenomics, next generation sequencing, and bioengineering have provided new insights and new opportunities to identify and optimize organisms for use in plastic bioremediation. In this review, we discuss the plastic waste problem and possible solutions, with a focus on potential mechanisms for plastic bioremediation. We pinpoint two key habitats to identify plastic-biodegrading organisms: (1) habitats with distinct enrichment of plastic waste, such as those near processing or disposal sites, and (2) habitats with naturally occurring polymers, such as cutin, lignin, and wax. Finally, we identify directions of future research for the isolation and optimization of these methods for widespread bioremediation applications.
C1 [Sheth, Maya U.; Kwartler, Sarah K.; Schmaltz, Emma R.; Somarelli, Jason A.] Duke Univ, Dept Med, Durham, NC 27708 USA.
[Hoskinson, Sarah M.; Eward, William C.] Duke Univ, Dept Orthopaed, Durham, NC USA.
[Hoskinson, Sarah M.; Eward, William C.; Somarelli, Jason A.] Duke Univ, Duke Canc Inst, Durham, NC 27708 USA.
[Martz, E. J.] North Carolina State Univ, Dept Biol Sci, Raleigh, NC USA.
[Dunphy-Daly, Meagan M.; Schultz, Thomas F.; Read, Andrew J.] Duke Univ, Nicholas Sch Environm, Div Marine Sci & Conservat, Durham, NC 27708 USA.
C3 Duke University; Duke University; Duke University; North Carolina State
University; Duke University
RP Somarelli, JA (autor correspondente), Duke Univ, Dept Med, Durham, NC 27708 USA.; Somarelli, JA (autor correspondente), Duke Univ, Duke Canc Inst, Durham, NC 27708 USA.
EM jason.somarelli@duke.edu
RI Read, Andrew/AAE-8386-2019
OI Sheth, Maya/0000-0003-1466-8899; Martz, E/0000-0002-0183-7705;
Dunphy-Daly, Meagan/0009-0006-1995-5909
FU Department of Defense [W81XWH-18-1-0189]; Seaworld & Busch Gardens
Conservation Fund; Paul and Shirley Friedland Fund; Triangle Center for
Evolutionary Medicine
FX JS is supported by the Department of Defense (W81XWH-18-1-0189), the Seaworld & Busch Gardens Conservation Fund, Meg and Bill Lindenberger, the Paul and Shirley Friedland Fund, the Triangle Center for Evolutionary Medicine, and funds raised in memory of Muriel E. Rudershausen (riding4research.org).
CR Acero EH, 2013, BIOTECHNOL BIOENG, V110, P2581, DOI 10.1002/bit.24930
Alok S, 2008, J MICROBIOL BIOTECHN, V18, P477
Andrady AL, 2009, PHILOS T R SOC B, V364, P1977, DOI 10.1098/rstb.2008.0304
Anonymous, 2008, V97, P311
[Anonymous], 1986, IARC Monogr Eval Carcinog Risk Chem Hum, V39, P347

[Anonymous], 2014, IARC Monographs, V106, P35
[Anonymous], 2006, IARC Monographs, V88, P37
[Anonymous], IARC MONOGR EVAL CAR
Apriceno A, 2019, CHEMOSPHERE, V215, P535, DOI 10.1016/j.chemosphere.2018.10.086
Astrup T, 2009, WASTE MANAGE RES, V27, P789, DOI 10.1177/0734242X09343774
Austin AT, 2006, NATURE, V442, P555, DOI 10.1038/nature05038
Austin HP, 2018, P NATL ACAD SCI USA, V115, pE4350, DOI 10.1073/pnas.1718804115
Barnes DKA, 2009, PHILOS T R SOC B, V364, P1985, DOI 10.1098/rstb.2008.0205
Barth M, 2016, BIOTECHNOL J, V11, P1082, DOI 10.1002/biot.201600008
Barth M, 2015, BIOCHEM ENG J, V93, P222, DOI 10.1016/j.bej.2014.10.012
Bombelli P, 2017, CURR BIOL, V27, pR292, DOI 10.1016/j.cub.2017.02.060
Bosetti C, 2003, EUR J CANCER PREV, V12, P427, DOI 10.1097/00008469-200310000-00012
Brandt-Rauf Paul Wesley, 2012, J Carcinog, V11, P5, DOI 10.4103/1477-3163.93700
Brooijmans RJW, 2009, MICROB BIOTECHNOL, V2, P587, DOI 10.1111/j.1751-7915.2009.00151.x
Carlton JT, 2017, SCIENCE, V357, P1402, DOI 10.1126/science.aao1498
Chiba S, 2018, MAR POLICY, V96, P204, DOI 10.1016/j.marpol.2018.03.022
Christopher LP, 2014, FRONT ENERGY RES, DOI 10.3389/fenrg.2014.00012
Cox KD, 2019, ENVIRON SCI TECHNOL, V53, P7068, DOI 10.1021/acs.est.9b01517
Cózar A, 2017, SCI ADV, V3, DOI 10.1126/sciadv.1600582
Cromack K, 1981, Q REV BIOL, V56, P96
Dashtban Mehdi, 2010, Int J Biochem Mol Biol, V1, P36
Deguchi T, 1998, APPL ENVIRON MICROB, V64, P1366
Deguchi T, 1997, APPL ENVIRON MICROB, V63, P329, DOI 10.1128/AEM.63.1.329-331.1997
Dirzo R, 2003, ANNU REV ENV RESOUR, V28, P137, DOI 10.1146/annurev.energy.28.050302.105532
Eberl A, 2009, J BIOTECHNOL, V143, P207, DOI 10.1016/j.jbiotec.2009.07.008
Eriksen M, 2014, PLOS ONE, V9, DOI 10.1371/journal.pone.0111913
Eriksson C, 2003, AMBIO, V32, P380, DOI 10.1639/0044-7447(2003)032[0380:OABAOS]2.0.CO;2
Esmaeili A, 2013, PLOS ONE, V8, DOI 10.1371/journal.pone.0071720
Espino-Rammer L, 2013, APPL ENVIRON MICROB, V79, P4230, DOI 10.1128/AEM.01132-13
Fich EA, 2016, ANNU REV PLANT BIOL, V67, P207, DOI 10.1146/annurev-arplant-043015-111929
Friedrich J, 2007, CHEMOSPHERE, V67, P2089, DOI 10.1016/j.chemosphere.2006.09.038
Fujisawa M, 2001, J POLYM ENVIRON, V9, P103, DOI 10.1023/A:1020472426516
Gewert B, 2015, ENVIRON SCI-PROC IMP, V17, P1513, DOI 10.1039/c5em00207a
Geyer R, 2017, SCI ADV, V3, DOI 10.1126/sciadv.1700782
Gilan I, 2004, APPL MICROBIOL BIOT, V65, P97, DOI 10.1007/s00253-004-1584-8
Gilpin R. K, 2005, DIOXINS HLTH

Gómez EF, 2013, POLYM DEGRAD STABIL, V98, P2583, DOI 10.1016/j.polymdegradstab.2013.09.018
Gregory MR, 2009, PHILOS T R SOC B, V364, P2013, DOI 10.1098/rstb.2008.0265
Hadad D, 2005, J APPL MICROBIOL, V98, P1093, DOI 10.1111/j.1365-2672.2005.02553.x
Han X, 2017, NAT COMMUN, V8, DOI 10.1038/s41467-017-02255-z
Hoornweg D., 2012, Urban development series; knowledge papers, V15, DOI DOI 10.1111/FEBS.13058
Hopewell J, 2009, PHILOS T R SOC B, V364, P2115, DOI 10.1098/rstb.2008.0311
Horner-Devine MC, 2004, P ROY SOC B-BIOL SCI, V271, P113, DOI 10.1098/rspb.2003.2549
I.A.R.C, 1993, IARC MONOGRAPHS EVAL, V58, P41
Ibrahim I. N., 2009, Advances in Environmental Biology, V3, P162
INFANTE PF, 1976, MUTAT RES, V41, P131, DOI 10.1016/0027-5107(76)90083-X
International Agencey for Research on Cancer, 2018, IARC MONOGRAPHS EVAL, P191
International Agencey for Research on Cancer, 1994, IARC MON EV CARC RIS, V60, P215
International Agencey for Research on Cancer, 2010, IARC MONOGRAPHS EVAL, V99, P325
International Agencey for Research on Cancer, 1995, CANC CAUS CONTROL, V7, P289, DOI [10.1007/bf00051307, DOI 10.1007/BF00051307]
International Agencey for Research on Cancer, 2000, IARC MON EV CARC RIS, V77, P41
International Agencey for Research on Cancer, 1977, IARC MONOGRAPHS EVAL, V14, P29
International Agencey for Research on Cancer, 1990, IARC MONOGR EVAL CAR, V48, P45
International Agencey for Research on Cancer, 2017, IARC MONOGRAPHS EVAL, V110, P111
International Agency for Research on Cancer, 1999, IARC MONOGRAPHS EVAL
International Agency for Research on Cancer, 2013, IARC MONOGR EVAL CAR, V101, P141
International Agency for Research on Cancer (IARC), 2012, Arsenic, Metals, VVolume 100C, P219
International Agency for Research on Cancer (IARC) Working Group on the Evaluation of Carcinogenic Risks to Humans, 2012, Chemical agents and related occupations, P309
Jambeck JR, 2015, SCIENCE, V347, P768, DOI 10.1126/science.1260352
Jang M, 2018, MAR POLLUT BULL, V131, P365, DOI 10.1016/j.marpolbul.2018.04.017
Joo S, 2018, NAT COMMUN, V9, DOI 10.1038/s41467-018-02881-1
Alava JJ, 2019, PREDICTING FUTURE OCEANS: SUSTAINABILITY OF OCEAN AND HUMAN SYSTEMS AMIDST GLOBAL ENVIRONMENTAL CHANGE, P495, DOI 10.1016/B978-0-12-817945-1.00046-0
Kawai F, 2014, APPL MICROBIOL BIOT, V98, P10053, DOI 10.1007/s00253-014-5860-y
Klun U, 2003, POLYM DEGRAD STABIL, V79, P99, DOI 10.1016/S0141-3910(02)00260-4
Kong HG, 2019, CELL REP, V26, P2451, DOI 10.1016/j.celrep.2019.02.018

Koutny M, 2009, INT BIODETER BIODEGR, V63, P354, DOI 10.1016/j.ibiod.2008.11.003
Kowalczyk A, 2016, ENVIRON SCI POLLUT R, V23, P11349, DOI 10.1007/s11356-016-6563-y
Kumar AK, 2013, ARCH ENVIRON CON TOX, V64, P652, DOI 10.1007/s00244-012-9857-1
Law KL, 2017, ANNU REV MAR SCI, V9, P205, DOI 10.1146/annurev-marine-010816-060409
Leonowicz A, 1999, FUNGAL GENET BIOL, V27, P175, DOI 10.1006/fgbi.1999.1150
Lopez V, 2013, BMJ OPEN, V3, DOI 10.1136/bmjopen-2013-002785
Lucas N, 2008, CHEMOSPHERE, V73, P429, DOI 10.1016/j.chemosphere.2008.06.064
Luo Q, 2018, ENVIRON SCI TECHNOL, V52, P10617, DOI 10.1021/acs.est.8b00839
Mcdonough W., 2010, Cradle to Cradle: Remaking the Way We Make Things
Mukherjee K, 2011, BIODEGRADATION, V22, P377, DOI 10.1007/s10532-010-9409-1
Müller RJ, 2005, MACROMOL RAPID COMM, V26, P1400, DOI 10.1002/marc.200500410
Naghdi M, 2018, PROCESS BIOCHEM, V67, P147, DOI 10.1016/j.procbio.2018.02.009
Nair DN, 2014, SCI WORLD J, DOI 10.1155/2014/250693
National Research Council, 2003, Oil in the sea III: inputs, fates, and effects
Neely AN, 2000, J CLIN MICROBIOL, V38, P724, DOI 10.1128/JCM.38.2.724-726.2000
Neufeld F. S, 2016, NEW PLASTICS EC RETH
Ojha N, 2017, SCI REP-UK, V7, DOI 10.1038/srep39515
Placido J., 2015, Bioresources and Bioprocessing, V2, P23, DOI DOI 10.1186/S40643-015-0049-5
Prosser JI, 2007, NAT REV MICROBIOL, V5, P384, DOI 10.1038/nrmicro1643
Ribitsch D, 2015, APPL ENVIRON MICROB, V81, P3586, DOI 10.1128/AEM.04111-14
Rochman CM, 2016, ECOLOGY, V97, P302, DOI 10.1890/14-2070.1
Rochman CM, 2013, SCI REP-UK, V3, DOI 10.1038/srep03263
Ronkvist ÅM, 2009, MACROMOLECULES, V42, P5128, DOI 10.1021/ma9005318
Royer SJ, 2018, PLOS ONE, V13, DOI 10.1371/journal.pone.0200574
Rubin BS, 2011, J STEROID BIOCHEM, V127, P27, DOI 10.1016/j.jsbmb.2011.05.002
Russell JR, 2011, APPL ENVIRON MICROB, V77, P6076, DOI 10.1128/AEM.00521-11
Scott AR, 2015, NATURE, V519, pS12, DOI 10.1038/519S12a
Setälä O, 2014, ENVIRON POLLUT, V185, P77, DOI 10.1016/j.envpol.2013.10.013
Shah Z, 2016, POLYM DEGRAD STABIL, V134, P349, DOI 10.1016/j.polymdegradstab.2016.11.003
Shah Z, 2013, BIODEGRADATION, V24, P865, DOI 10.1007/s10532-013-9634-5
Shirke AN, 2018, BIOCHEMISTRY-US, V57, P1190, DOI 10.1021/acs.biochem.7b01189
Silva C, 2011, BIOTECHNOL J, V6, P1230, DOI 10.1002/biot.201000391
Skariyachan S, 2017, ENVIRON SCI POLLUT R, V24, P8443, DOI 10.1007/s11356-017-8537-0

Skariyachan S, 2016, ENVIRON SCI POLLUT R, V23, P18307, DOI 10.1007/s11356-016-7000-y
Sogin ML, 2006, P NATL ACAD SCI USA, V103, P12115, DOI 10.1073/pnas.0605127103
Song JH, 2009, PHILOS T R SOC B, V364, P2127, DOI 10.1098/rstb.2008.0289
Sudhakar M, 2007, INT BIODETER BIODEGR, V60, P144, DOI 10.1016/j.ibiod.2007.02.002
Sulaiman S, 2012, APPL ENVIRON MICROB, V78, P1556, DOI 10.1128/AEM.06725-11
Tachibana K, 2010, POLYM DEGRAD STABIL, V95, P912, DOI 10.1016/j.polymdegradstab.2010.03.031
Teuten EL, 2009, PHILOS T R SOC B, V364, P2027, DOI 10.1098/rstb.2008.0284
Thompson RC, 2009, PHILOS T R SOC B, V364, P2153, DOI 10.1098/rstb.2009.0053
Tribedi P., 2015, Bio resources and Bioprocessing, V2, P1, DOI DOI 10.1186/S40643-015-0044-X
Tripathi BM, 2016, FRONT MICROBIOL, V7, DOI 10.3389/fmicb.2016.00376
TULLOCH AP, 1971, CHEM PHYS LIPIDS, V6, P235, DOI 10.1016/0009-3084(71)90063-6
Wang Z, 2017, ADV SCI, V4, DOI 10.1002/advs.201600248
Wei R, 2016, BIOTECHNOL BIOENG, V113, P1658, DOI 10.1002/bit.25941
Yang J, 2014, ENVIRON SCI TECHNOL, V48, P13776, DOI 10.1021/es504038a
Yoon MoonGyung Yoon MoonGyung, 2012, Journal of Bioremediation and Biodegradation, V3, P1000145
Yoshida S, 2016, SCIENCE, V351, P1196, DOI 10.1126/science.aad6359
NR 119
TC 29
Z9 31
U1 2
U2 119
PU FRONTIERS MEDIA SA
PI LAUSANNE
PA AVENUE DU TRIBUNAL FEDERAL 34, LAUSANNE, CH-1015, SWITZERLAND
EI 2296-7745
J9 FRONT MAR SCI
JI Front. Mar. Sci.
PD OCT 11
PY 2019
VL 6
AR 624
DI 10.3389/fmars.2019.00624
PG 10
WC Environmental Sciences; Marine & Freshwater Biology
WE Science Citation Index Expanded (SCI-EXPANDED)
SC Environmental Sciences & Ecology; Marine & Freshwater Biology
GA JD7HN
UT WOS:000490151900001
OA gold
DA 2025-05-07
ER

PT J

AU Dawson, D
Rasmussen, D
Peng, XX
Lanzas, C
AF Dawson, Daniel
Rasmussen, David
Peng, Xinxia
Lanzas, Cristina
TI Inferring environmental transmission using phylodynamics: a case-study using simulated evolution of an enteric pathogen
SO JOURNAL OF THE ROYAL SOCIETY INTERFACE
LA English
DT Article
DE environmental transmission; disease transmission modelling; phylodynamics; whole-genome sequencing
AB Indirect (environmental) and direct (host-host) transmission pathways cannot easily be distinguished when they co-occur in epidemics, particularly when they occur on similar time scales. Phylodynamic reconstruction is a potential approach to this problem that combines epidemiological information (temporal, spatial information) with pathogen whole-genome sequencing data to infer transmission trees of epidemics. However, factors such as differences in mutation and transmission rates between host and non-host environments may obscure phylogenetic inference from these methods. In this study, we used a network-based transmission model that explicitly models pathogen evolution to simulate epidemics with both direct and indirect transmission. Epidemics were simulated according to factorial combinations of direct/indirect transmission proportions, host mutation rates and conditions of environmental pathogen growth. Transmission trees were then reconstructed using the phylodynamic approach SCOTTI (structured coalescent transmission tree inference) and evaluated. We found that although insufficient diversity sets a lower bound on when accurate phylodynamic inferences can be made, transmission routes and assumed pathogen lifestyle affected pathogen population structure and subsequently influenced both reconstruction success and the likelihood of direct versus indirect pathways being reconstructed. We conclude that prior knowledge of the likely ecology and population structure of pathogens in host and non-host environments is critical to fully using phylodynamic techniques.
C1 [Dawson, Daniel; Lanzas, Cristina] North Carolina State Univ, Coll Vet Med, Dept Populat Hlth & Pathobiol, Raleigh, NC 27695 USA.
[Rasmussen, David; Peng, Xinxia] North Carolina State Univ, Bioinformat Res Ctr, Raleigh, NC 27695 USA.
[Peng, Xinxia] North Carolina State Univ, Coll Vet Med, Dept Mol Biomed Sci, Raleigh, NC 27695 USA.
[Rasmussen, David] North Carolina State Univ, Dept Entomol & Plant Pathol, Raleigh, NC 27695 USA.
C3 North Carolina State University; North Carolina State University; North
Carolina State University; North Carolina State University
RP Dawson, D (autor correspondente), North Carolina State Univ, Coll Vet Med, Dept Populat Hlth & Pathobiol, Raleigh, NC 27695 USA.
EM daniel.dawson1081@gmail.com
RI Dawson, Daniel/JVZ-2105-2024
OI Rasmussen, David/0000-0001-9457-7561; Dawson,

Daniel/0000-0001-9622-4495; Lanzas, Cristina/0000-0002-4039-0336; Peng,
Xinxia/0000-0002-0117-3582
FU Triangle Center for Evolutionary Medicine (TriCEM); US National
Institutes of Health (NIH) [R01GM117618, R35GM134934]
FX This work was supported by the Triangle Center for Evolutionary Medicine
(TriCEM) and the US National Institutes of Health (NIH) (grant nos.
R01GM117618 and R35GM134934). The funders had no role in study design,
data collection and analysis, decision to publish, or preparation of the
manuscript.
CR Anttila J, 2013, PLOS ONE, V8, DOI 10.1371/journal.pone.0071621
Bouckaert R, 2019, PLOS COMPUT BIOL, V15, DOI 10.1371/journal.pcbi.1006650
Breban R, 2013, J MATH BIOL, V66, P535, DOI 10.1007/s00285-012-0520-2
Breban R, 2010, J THEOR BIOL, V264, P729, DOI 10.1016/j.jtbi.2010.03.005
Brouwer AF, 2017, PLOS COMPUT BIOL, V13, DOI 10.1371/journal.pcbi.1005481
Campbell F, 2018, PLOS PATHOG, V14, DOI 10.1371/journal.ppat.1006885
Cortez MH, 2013, AM NAT, V181, pE43, DOI 10.1086/668826
Dawson DE, 2019, EPIDEMICS-NETH, V26, P32, DOI 10.1016/j.epidem.2018.08.003
De Maio N, 2018, PLOS COMPUT BIOL, V14, DOI 10.1371/journal.pcbi.1006117
De Maio N, 2016, PLOS COMPUT BIOL, V12, DOI 10.1371/journal.pcbi.1005130
Didelot X, 2014, MOL BIOL EVOL, V31, P1869, DOI 10.1093/molbev/msu121
Eisenberg MC, 2013, J THEOR BIOL, V324, P84, DOI 10.1016/j.jtbi.2012.12.021
Firestone SM, 2019, SCI REP-UK, V9, DOI 10.1038/s41598-019-41103-6
Frenoy A, 2018, PLOS BIOL, V16, DOI 10.1371/journal.pbio.2005056
Gaunle K., 2018, CALCULATE SPECIES EV
Gibson B, 2018, P ROY SOC B-BIOL SCI, V285, DOI 10.1098/rspb.2018.0789
Handel A, 2013, PLOS COMPUT BIOL, V9, DOI 10.1371/journal.pcbi.1002989
HASEGAWA M, 1985, J MOL EVOL, V22, P160, DOI 10.1007/BF02101694
Jombart T, 2014, PLOS COMPUT BIOL, V10, DOI 10.1371/journal.pcbi.1003457
JUKES T H, 1969, P21
Kendall M, 2018, STAT SCI, V33, P70, DOI 10.1214/17-STS637
Klinkenberg D, 2017, PLOS COMPUT BIOL, V13, DOI 10.1371/journal.pcbi.1005495
Krasovec R, 2014, NAT COMMUN, V5, DOI 10.1038/ncomms4742
Lanzas C, 2020, INTERFACE FOCUS, V10, DOI 10.1098/rsfs.2019.0056
Lee H, 2012, P NATL ACAD SCI USA, V109, pE2774, DOI 10.1073/pnas.1210309109
Maharjan RP, 2018, ENV MICROBIOL REP, V10, P626, DOI 10.1111/1758-2229.12661
O'Leary NA, 2016, NUCLEIC ACIDS RES, V44, pD733, DOI 10.1093/nar/gkv1189
PIELOU EC, 1966, J THEOR BIOL, V13, P131, DOI 10.1016/0022-5193(66)90013-0
Rambaut A, 2018, SYST BIOL, V67, P901, DOI 10.1093/sysbio/syy032

   Roche B, 2011, ECOL LETT, V14, P569, DOI 10.1111/j.1461-0248.2011.01619.x
   Rohani P, 2009, P NATL ACAD SCI USA, V106, P10365, DOI 10.1073/pnas.0809026106
   Snitkin ES, 2017, SCI TRANSL MED, V9, DOI 10.1126/scitranslmed.aan0093
   Walther BA, 2004, BIOL REV, V79, P849, DOI 10.1017/S1464793104006475
   Worby CJ, 2015, PLOS ONE, V10, DOI 10.1371/journal.pone.0129745
   Worby CJ, 2014, PLOS COMPUT BIOL, V10, DOI 10.1371/journal.pcbi.1003549
   Wrande M, 2008, P NATL ACAD SCI USA, V105, P11863, DOI 10.1073/pnas.0804739105
   Ypma RJF, 2013, GENETICS, V195, P1055, DOI 10.1534/genetics.113.154856
NR 37
TC 5
Z9 5
U1 2
U2 11
PU ROYAL SOC
PI LONDON
PA 6-9 CARLTON HOUSE TERRACE, LONDON SW1Y 5AG, ENGLAND
SN 1742-5689
EI 1742-5662
J9 J R SOC INTERFACE
JI J. R. Soc. Interface
PD JUN 9
PY 2021
VL 18
IS 179
AR 20210041
DI 10.1098/rsif.2021.0041
PG 10
WC Multidisciplinary Sciences
WE Science Citation Index Expanded (SCI-EXPANDED)
SC Science & Technology - Other Topics
GA SS1HQ
UT WOS:000661492700001
PM 34102084
OA Green Published, Bronze
DA 2025-05-07
ER

PT J
AU Wcisel, DJ
   Ota, T
   Litman, GW
   Yoder, JA
AF Wcisel, Dustin J.
   Ota, Tatsuya
   Litman, Gary W.
   Yoder, Jeffrey A.
TI Spotted Gar and the Evolution of Innate Immune Receptors
SO JOURNAL OF EXPERIMENTAL ZOOLOGY PART B-MOLECULAR AND DEVELOPMENTAL
   EVOLUTION
LA English

DT Article
ID TOLL-LIKE RECEPTORS; MHC CLASS-I; IMMUNOGLOBULIN-LIKE TRANSCRIPTS; GENOME DUPLICATION; CHANNEL CATFISH; RAINBOW-TROUT; BONY FISH; FUNCTIONAL-CHARACTERIZATION; LIPOPOLYSACCHARIDE LPS; ICTALURUS-PUNCTATUS
AB The resolution of the gar genome affords an opportunity to examine the diversification and functional specialization of immune effector molecules at a distant and potentially informative point in phylogenetic development. Although innate immunity is effected by a particularly large number of different families of molecules, the focus here is to provide detailed characterization of several families of innate receptors that are encoded in large multigene families, for which orthologous forms can be identified in other species of bony fish but not in other vertebrate groups as well as those for which orthologs are present in other vertebrate species. The results indicate that although teleost fish and the gar, as a holostean reference species, share gene families thought previously to be restricted to the teleost fish, the manner in which the members of the multigene families of innate immune receptors have undergone diversification is different in these two major phylogenetic radiations. It appears that both the total genome duplication and different patterns of genetic selection have influenced the derivation and stabilization of innate immune genes in a substantial manner during the course of vertebrate evolution.
C1 [Wcisel, Dustin J.; Yoder, Jeffrey A.] North Carolina State Univ, Dept Mol Biomed Sci, 1060 William Moore Dr, Raleigh, NC 27607 USA.
[Ota, Tatsuya] SOKENDAI, Dept Evolutionary Studies Biosyst, Hayama, Japan.
[Litman, Gary W.] Univ S Florida, Morsani Coll Med, Dept Pediat, St Petersburg, FL USA.
[Yoder, Jeffrey A.] North Carolina State Univ, Comparat Med Inst, Raleigh, NC 27607 USA.
[Yoder, Jeffrey A.] N Carolina State Univ, Ctr Human Hlth & Environm, Raleigh, NC 27607 USA.
C3 North Carolina State University; Graduate University for Advanced Studies - Japan; State University System of Florida; University of South Florida; North Carolina State University; North Carolina State University
RP Yoder, JA (autor correspondente), North Carolina State Univ, Dept Mol Biomed Sci, 1060 William Moore Dr, Raleigh, NC 27607 USA.
EM jeff_yoder@ncsu.edu
RI Yoder, Jeffrey/I-5329-2019
OI Yoder, Jeff/0000-0002-6083-1311; Ota, Tatsuya/0000-0002-1115-5169; Wcisel, Dustin/0000-0001-6858-643X
FU National Institutes of Health [R01 AI057559]; National Evolutionary Synthesis Center [EF-0905606]; National Science Foundation; Triangle Center for Evolutionary Medicine
FX Grant sponsor: National Institutes of Health; Grant number: R01 AI057559; Grant sponsor: National Evolutionary Synthesis Center; Grant number: EF-0905606, National Science Foundation; Grant sponsor: Triangle Center for Evolutionary Medicine.
CR Aderem A, 2000, NATURE, V406, P782, DOI 10.1038/35021228
Aken BL, 2017, NUCLEIC ACIDS RES, V45, pD635, DOI 10.1093/nar/gkw1104

ALTSCHUL SF, 1990, J MOL BIOL, V215, P403, DOI 10.1006/jmbi.1990.9999
Amores A, 1998, SCIENCE, V282, P1711, DOI 10.1126/science.282.5394.1711
Amores A, 2011, GENETICS, V188, P799, DOI 10.1534/genetics.111.127324
Barrow AD, 2006, EUR J IMMUNOL, V36, P1646, DOI 10.1002/eji.200636195
BERCZI I, 1966, CAN J MICROBIOL, V12, P1070, DOI 10.1139/m66-143
Beutler B, 2000, CURR OPIN IMMUNOL, V12, P20, DOI 10.1016/S0952-7915(99)00046-1
Beutler B., 2002, EVOLUTION TIR TOLLS, P1
Borrego F, 2013, BLOOD, V121, P1951, DOI 10.1182/blood-2012-09-435057
Boudinot P, 2014, J EXP ZOOL PART B, V322, P415, DOI 10.1002/jez.b.22559
Braasch I, 2016, NAT GENET, V48, P427, DOI 10.1038/ng.3526
Brawand D, 2014, NATURE, V513, P375, DOI 10.1038/nature13726
Buonocore F, 2016, MOL IMMUNOL, V69, P157, DOI 10.1016/j.molimm.2015.09.003
Camacho C, 2009, BMC BIOINFORMATICS, V10, DOI 10.1186/1471-2105-10-421
Cannon JP, 2008, IMMUNITY, V29, P228, DOI 10.1016/j.immuni.2008.05.018
Carrillo-Bustamante P, 2016, IMMUNOGENETICS, V68, P3, DOI 10.1007/s00251-015-0869-7
Dehal P, 2005, PLOS BIOL, V3, P1700, DOI 10.1371/journal.pbio.0030314
Desai S, 2008, BMC EVOL BIOL, V8, DOI 10.1186/1471-2148-8-177
Dirscherl H, 2014, DEV COMP IMMUNOL, V46, P11, DOI 10.1016/j.dci.2014.02.018
Dirscherl H, 2014, IMMUNOGENETICS, V66, P185, DOI 10.1007/s00251-013-0748-z
Eschmeyer WN, 2016, CATALOG FISHES ON LI
Ferraresso S, 2009, IMMUNOGENETICS, V61, P773, DOI 10.1007/s00251-009-0398-3
Flajnik MF, 2001, IMMUNITY, V15, P351, DOI 10.1016/S1074-7613(01)00198-4
Gay NJ, 2007, ANNU REV BIOCHEM, V76, P141, DOI 10.1146/annurev.biochem.76.060305.151318
Glasauer SMK, 2014, MOL GENET GENOMICS, V289, P1045, DOI 10.1007/s00438-014-0889-2
Grimholt U, 2015, BMC EVOL BIOL, V15, DOI 10.1186/s12862-015-0309-1
Guethlein LA, 2015, IMMUNOL REV, V267, P259, DOI 10.1111/imr.12326
Haire RN, 2012, GENOMICS, V99, P282, DOI 10.1016/j.ygeno.2012.02.004
Hansen JD, 2011, DEV COMP IMMUNOL, V35, P886, DOI 10.1016/j.dci.2011.01.008
Huang XN, 2011, FISH SHELLFISH IMMUN, V31, P98, DOI 10.1016/j.fsi.2011.04.009
Jack RS, 2015, RESULTS PROBL CELL D, V57, P1, DOI 10.1007/978-3-319-20819-0_1
Jamil KM, 2011, J BIOMED BIOTECHNOL, DOI 10.1155/2011/298348
Jault C, 2004, MOL IMMUNOL, V40, P759, DOI 10.1016/j.molimm.2003.10.001
JONES DT, 1992, COMPUT APPL BIOSCI, V8, P275, DOI 10.1093/bioinformatics/8.3.275
Kanwal Z, 2014, DEV COMP IMMUNOL, V46, P35, DOI 10.1016/j.dci.2014.02.003
Kawai T, 2006, CELL DEATH DIFFER, V13, P816, DOI 10.1038/sj.cdd.4401850
Keestra AM, 2010, J IMMUNOL, V185, P460, DOI 10.4049/jimmunol.0901921

Kim JI, 2005, J BIOL CHEM, V280, P11347, DOI 10.1074/jbc.M414607200
Kock H, 2008, MOL IMMUNOL, V45, P1612, DOI 10.1016/j.molimm.2007.10.005
Kortum AN, 2014, IMMUNOGENETICS, V66, P267, DOI 10.1007/s00251-014-0759-4
Kumar S, 2016, MOL BIOL EVOL, V33, P1870, DOI [10.1093/molbev/msw054, 10.1093/molbev/msv279]
Kuraku S, 2010, EVOLUTION AFTER GENE DUPLICATION, P299
Lane RP, 2002, GENOME RES, V12, P81, DOI 10.1101/gr.197901
Letunic I, 2015, NUCLEIC ACIDS RES, V43, pD257, DOI 10.1093/nar/gku949
Leulier F, 2008, NAT REV GENET, V9, P165, DOI 10.1038/nrg2303
Louis A, 2015, NUCLEIC ACIDS RES, V43, pD682, DOI 10.1093/nar/gku1112
Lu YL, 2013, DEV COMP IMMUNOL, V41, P498, DOI 10.1016/j.dci.2013.07.009
Malmstrom M, 2016, NAT GENET, V48, P1204, DOI 10.1038/ng.3645
Manser AR, 2015, IMMUNOL REV, V267, P178, DOI 10.1111/imr.12316
Matsuo A, 2008, J IMMUNOL, V181, P3474, DOI 10.4049/jimmunol.181.5.3474
Matsushima N, 2005, CELL MOL LIFE SCI, V62, P2771, DOI 10.1007/s00018-005-5187-z
Matsushima N, 2007, BMC GENOMICS, V8, DOI 10.1186/1471-2164-8-124
Miggin SM, 2016, J LEUKOCYTE BIOL, V80, P220
MORRISON DC, 1987, ANNU REV MED, V38, P417, DOI 10.1146/annurev.me.38.020187.002221
Muñoz I, 2013, FISH SHELLFISH IMMUN, V35, P36, DOI 10.1016/j.fsi.2013.03.374
Nelson J.S., 2016, Fishes of the World, V5th edition
Ostergaard AE, 2010, BMC GENOMICS, V11, DOI 10.1186/1471-2164-11-697
Ostergaard AE, 2009, DEV COMP IMMUNOL, V33, P525, DOI 10.1016/j.dci.2008.10.002
Palti Y, 2011, DEV COMP IMMUNOL, V35, P1263, DOI 10.1016/j.dci.2011.03.006
Pasquier J, 2016, BMC GENOMICS, V17, DOI 10.1186/s12864-016-2709-z
Quiniou SMA, 2013, IMMUNOGENETICS, V65, P511, DOI 10.1007/s00251-013-0694-9
Rahim MMA, 2015, IMMUNOL REV, V267, P137, DOI 10.1111/imr.12318
Rauta PR, 2014, IMMUNOL LETT, V158, P14, DOI 10.1016/j.imlet.2013.11.013
Roach JC, 2005, P NATL ACAD SCI USA, V102, P9577, DOI 10.1073/pnas.0502272102
Rodriguez-Nunez I, 2016, IMMUNOGENETICS, V68, P295, DOI 10.1007/s00251-016-0901-6
Rodríguez-Nunez I, 2014, DEV COMP IMMUNOL, V46, P24, DOI 10.1016/j.dci.2014.02.004
Saha NR, 2014, J EXP ZOOL PART B, V322, P438, DOI 10.1002/jez.b.22558
SAITOU N, 1987, MOL BIOL EVOL, V4, P406, DOI 10.1093/oxfordjournals.molbev.a040454
Samanta M, 2013, MOL BIOL REP, V40, P225, DOI 10.1007/s11033-012-2053-6
Schartl M, 2014, DIS MODEL MECH, V7, P181, DOI 10.1242/dmm.012245
Sepulcre MP, 2009, J IMMUNOL, V182, P1836, DOI 10.4049/jimmunol.0801755
Sievers F, 2011, MOL SYST BIOL, V7, DOI 10.1038/msb.2011.75

Stafford JL, 2007, IMMUNOGENETICS, V59, P77, DOI 10.1007/s00251-006-0169-3
Stafford JL, 2006, IMMUNOGENETICS, V58, P758, DOI 10.1007/s00251-006-0134-1
Star B, 2012, BIOESSAYS, V34, P648, DOI 10.1002/bies.201200005
Star B, 2011, NATURE, V477, P207, DOI 10.1038/nature10342
Stet RJM, 2005, IMMUNOGENETICS, V57, P77, DOI 10.1007/s00251-005-0771-9
Strong SJ, 1999, P NATL ACAD SCI USA, V96, P15080, DOI 10.1073/pnas.96.26.15080
Su GL, 2000, HEPATOLOGY, V31, P932, DOI 10.1053/he.2000.5634
Sullivan C, 2009, J IMMUNOL, V183, P5896, DOI 10.4049/jimmunol.0803285
Takeuchi O, 2002, J IMMUNOL, V169, P10, DOI 10.4049/jimmunol.169.1.10
Taylor EB, 2016, J IMMUNOL, V196, P2677, DOI 10.4049/jimmunol.1502166
Taylor JS, 2003, GENOME RES, V13, P382, DOI 10.1101/gr.640303
Thaiss CA, 2016, NATURE, V535, P65, DOI 10.1038/nature18847
Tsujita T, 2004, J BIOL CHEM, V279, P48588, DOI 10.1074/jbc.M407634200
Vilella AJ, 2009, GENOME RES, V19, P327, DOI 10.1101/gr.073585.107
Wang JL, 2015, FISH SHELLFISH IMMUN, V45, P940, DOI 10.1016/j.fsi.2015.06.017
Wcisel DJ, 2016, FISH SHELLFISH IMMUN, V53, P24, DOI 10.1016/j.fsi.2016.03.034
Wei S, 2007, IMMUNOGENETICS, V59, P813, DOI 10.1007/s00251-007-0250-6
WRIGHT SD, 1990, SCIENCE, V249, P1431, DOI 10.1126/science.1698311
Yeh DW, 2013, P NATL ACAD SCI USA, V110, P20711, DOI 10.1073/pnas.1305273110
Yoder JA, 2004, P NATL ACAD SCI USA, V101, P15706, DOI 10.1073/pnas.0405242101
Yoder JA, 2001, P NATL ACAD SCI USA, V98, P6771, DOI 10.1073/pnas.121101598
Yoder JA, 2008, IMMUNOGENETICS, V60, P257, DOI 10.1007/s00251-008-0285-3
Yoder JA, 2011, IMMUNOGENETICS, V63, P123, DOI 10.1007/s00251-010-0506-4
Yoder JA, 2010, IMMUNOGENETICS, V62, P117, DOI 10.1007/s00251-009-0416-5
Yoder JA, 2009, DEV COMP IMMUNOL, V33, P135, DOI 10.1016/j.dci.2008.09.004
Zhang J, 2014, FISH SHELLFISH IMMUN, V41, P380, DOI 10.1016/j.fsi.2014.09.022
NR 99
TC 21
Z9 23
U1 0
U2 13
PU WILEY
PI HOBOKEN
PA 111 RIVER ST, HOBOKEN 07030-5774, NJ USA
SN 1552-5007
EI 1552-5015
J9 J EXP ZOOL PART B
JI J. Exp. Zool. Part B
PD NOV
PY 2017

VL 328
IS 7
SI SI
BP 666
EP 684
DI 10.1002/jez.b.22738
PG 19
WC Evolutionary Biology; Developmental Biology; Zoology
WE Science Citation Index Expanded (SCI-EXPANDED)
SC Evolutionary Biology; Developmental Biology; Zoology
GA FK6AX
UT WOS:000413586500008
PM 28544607
OA Bronze, Green Accepted
DA 2025-05-07
ER

PT J
AU Voskarides, K
AF Voskarides, Konstantinos
TI Combination of 247 Genome-Wide Association Studies Reveals High Cancer Risk as a Result of Evolutionary Adaptation
SO MOLECULAR BIOLOGY AND EVOLUTION
LA English
DT Article
DE evolutionary medicine; antagonistic pleiotropy; natural selection; extreme environmental conditions; cancer evolution; tumor suppressor genes; population genetics; meta-analysis; genomics; average annual temperature; cancer epidemiology; high altitude; breast cancer; GWAS
ID HIGH-ALTITUDE ADAPTATION; GALLBLADDER CANCER; GENETIC SIGNATURES; ESCHERICHIA-COLI; SIGNALS; POPULATIONS; SELECTION; PEOPLE; LINK; P53
AB Analysis of GLOBOCAN-2012 data shows clearly here that cancer incidence worldwide is highly related with low average annual temperatures and extreme low temperatures. This applies for all cancers together or separately formany frequent or rare cancer types (all cancers P = 9.49 x 10(-18)). Supporting fact is that Inuit people, living at extreme low temperatures, have the highest cancer rates today. Hypothesizing an evolutionary explanation, 240 cancer genome-wide association studies, and seven genome-wide association studies for cold and high-altitude adaptation were combined. A list of 1,377 cancer-associated genes was created to initially investigate whether cold selected genes are enriched with cancerassociated genes. Among Native Americans, Inuit and Eskimos, the highest association was observed for Native Americans (P = 6.7 x 10(-5)). An overall or a meta-analysis approach confirmed further this result. Similar approach for three populations living at extreme high altitude, revealed high association for Andeans-Tibetans (P = 1.3 x 10(-11)). Overall analysis or a meta-analysis was also significant. A separate analysis showed special selection for tumor suppressor genes. These results can be viewed along with those of previous functional studies that showed that reduced apoptosis potential due to specific p53 variants (the most important tumor suppressor gene) is beneficial in high-altitude and cold environments. In conclusion, this study shows that genetic variants selected for adaptation at extreme environmental conditions can increase

cancer risk later on age. This is in accordance with antagonistic pleiotropy hypothesis.

C1 [Voskarides, Konstantinos] Univ Cyprus, Med Sch, Nicosia, Cyprus.
C3 University of Cyprus
RP Voskarides, K (autor correspondente), Univ Cyprus, Med Sch, Nicosia, Cyprus.
EM kvoskar@ucy.ac.cy
RI Voskarides, Konstantinos/A-4669-2013
OI Voskarides, Konstantinos/0000-0002-3705-3451

CR Abate S M., 2016, Int J Cancer Res Mol Mech, V2, P1
Alkorta-Aranburu G, 2012, PLOS GENET, V8, DOI 10.1371/journal.pgen.1003110
Amorim CEG, 2017, P NATL ACAD SCI USA, V114, P2195, DOI 10.1073/pnas.1620541114
Amundadottir LT, 2004, PLOS MED, V1, P229, DOI 10.1371/journal.pmed.0010065
Ashur-Fabian C, 2004, P NATL ACAD SCI USA, V101, P12236, DOI 10.1073/pnas.0404998101
Bigham A, 2010, PLOS GENET, V6, DOI 10.1371/journal.pgen.1001116
Bigham AW, 2016, CURR OPIN GENET DEV, V41, P8, DOI 10.1016/j.gde.2016.06.018
Bui DT, 2015, PLOS GENET, V11, DOI 10.1371/journal.pgen.1005407
Byrne RT, 2014, ELIFE, V3, DOI 10.7554/eLife.01322
Cardona A, 2014, PLOS ONE, V9, DOI 10.1371/journal.pone.0098076
Carter AJR, 2011, BMC MED GENET, V12, DOI 10.1186/1471-2350-12-160
Crespi B, 2005, TRENDS ECOL EVOL, V20, P545, DOI 10.1016/j.tree.2005.07.007
Day GE, 2010, INT J CIRCUMPOL HEAL, V69, P72, DOI 10.3402/ijch.v69i1.17388
de Martel C, 2017, INT J CANCER, V141, P664, DOI 10.1002/ijc.30716
Eisenberg E, 2013, TRENDS GENET, V29, P569, DOI 10.1016/j.tig.2013.05.010
Ferlay J, 2015, INT J CANCER, V136, pE359, DOI 10.1002/ijc.29210
Foll M, 2014, AM J HUM GENET, V95, P394, DOI 10.1016/j.ajhg.2014.09.002
Foote Mary, 2016, WMJ, V115, P11
Friborg JT, 2008, LANCET ONCOL, V9, P892, DOI 10.1016/S1470-2045(08)70231-6
Fumagalli M, 2015, SCIENCE, V349, P1343, DOI 10.1126/science.aab2319
Grazielle-Silva V, 2015, PLOS NEGLECT TROP D, V9, DOI 10.1371/journal.pntd.0003870
Harboe TL, 2009, FAM CANCER, V8, P413, DOI 10.1007/s10689-009-9257-5
Harris RJ, 2008, STATA J, V8, P3, DOI 10.1177/1536867X0800800102
Healey KR, 2016, NAT COMMUN, V7, DOI 10.1038/ncomms11128
Howe K, 2013, NATURE, V496, P498, DOI 10.1038/nature12111
Huang DW, 2009, NAT PROTOC, V4, P44, DOI 10.1038/nprot.2008.211
Huang DW, 2009, NUCLEIC ACIDS RES, V37, P1, DOI 10.1093/nar/gkn923
Huerta-Sánchez E, 2013, MOL BIOL EVOL, V30, P1877, DOI 10.1093/molbev/mst089
Jeggo PA, 2016, NAT REV CANCER, V16, P35, DOI 10.1038/nrc.2015.4
Jemal A, 2005, CA-CANCER J CLIN, V55, P10, DOI 10.3322/canjclin.55.1.10
Lanikova L, 2017, ONCOTARGET, V8, P11739, DOI 10.18632/oncotarget.14340

LeClerc JE, 1996, SCIENCE, V274, P1208, DOI 10.1126/science.274.5290.1208
Lemrow SM, 2008, CANCER-AM CANCER SOC, V113, P1266, DOI 10.1002/cncr.23737
Liu YN, 2017, J GENET GENOMICS, V44, P119, DOI 10.1016/j.jgg.2016.12.004
Lorenzo FR, 2014, NAT GENET, V46, P951, DOI 10.1038/ng.3067
Luján AM, 2011, PLOS ONE, V6, DOI 10.1371/journal.pone.0027842
MacArthur J, 2017, NUCLEIC ACIDS RES, V45, pD896, DOI 10.1093/nar/gkw1133
Mhatre S, 2017, LANCET ONCOL, V18, P535, DOI 10.1016/S1470-2045(17)30167-5
Moore SP, 2014, CANCER MED-US, V3, P70, DOI 10.1002/cam4.134
Nesse RM, 2011, EVOL APPL, V4, P264, DOI 10.1111/j.1752-4571.2010.00181.x
Notley-McRobb L, 2002, J BACTERIOL, V184, P739, DOI 10.1128/JB.184.3.739-745.2002
Peng Y, 2017, MOL BIOL EVOL, V34, P818, DOI 10.1093/molbev/msw280
Peng Y, 2011, MOL BIOL EVOL, V28, P1075, DOI 10.1093/molbev/msq290
Perdue DG, 2014, AM J PUBLIC HEALTH, V104, pS404, DOI 10.2105/AJPH.2013.301654
Randall H, 2017, ROY SOC OPEN SCI, V4
RIOSDALENZ J, 1981, INT J CANCER, V28, P307, DOI 10.1002/ijc.2910280309
Scheinfeldt LB, 2012, GENOME BIOL, V13, DOI 10.1186/gb-2012-13-1-r1
Sharma A, 2015, TUMOR BIOL, V36, P5953, DOI 10.1007/s13277-015-3270-0
Simonson TS, 2010, SCIENCE, V329, P72, DOI 10.1126/science.1189406
Tashi T, 2017, J MOL MED, V95, P665, DOI 10.1007/s00109-017-1519-3
Tekola-Ayele F, 2015, EUR J HUM GENET, V23, P1085, DOI 10.1038/ejhg.2014.233
Valverde G, 2015, PLOS ONE, V10, DOI 10.1371/journal.pone.0125444
Vittecoq M, 2013, TRENDS ECOL EVOL, V28, P628, DOI 10.1016/j.tree.2013.07.005
Wang ZM, 2017, NAT GENET, V49, P1141, DOI 10.1038/ng.3879
WILLIAMS GC, 1957, EVOLUTION, V11, P398, DOI 10.1111/j.1558-5646.1957.tb02911.x
Xu SH, 2011, MOL BIOL EVOL, V28, P1003, DOI 10.1093/molbev/msq277
Yi X, 2010, SCIENCE, V329, P75, DOI 10.1126/science.1190371
Young TK, 2016, INT J CIRCUMPOL HEAL, V75, DOI 10.3402/ijch.v75.29787
ZARIDZE DG, 1993, INT J CANCER, V54, P889, DOI 10.1002/ijc.2910540602
Zhang B, 2014, NAT GENET, V46, P533, DOI 10.1038/ng.2985
Zhao M, 2016, NUCLEIC ACIDS RES, V44, pD1023, DOI 10.1093/nar/gkv1268
Zhao Y, 2013, P NATL ACAD SCI USA, V110, P20639, DOI 10.1073/pnas.1320369110
NR 62
TC 45
Z9 45
U1 0
U2 33
PU OXFORD UNIV PRESS
PI OXFORD
PA GREAT CLARENDON ST, OXFORD OX2 6DP, ENGLAND
SN 0737-4038
EI 1537-1719

J9 MOL BIOL EVOL
JI Mol. Biol. Evol.
PD FEB
PY 2018
VL 35
IS 2
BP 473
EP 485
DI 10.1093/molbev/msx305
PG 13
WC Biochemistry & Molecular Biology; Evolutionary Biology; Genetics & Heredity
WE Science Citation Index Expanded (SCI-EXPANDED)
SC Biochemistry & Molecular Biology; Evolutionary Biology; Genetics & Heredity
GA FU2XK
UT WOS:000423713100016
PM 29220501
OA Green Published, hybrid
DA 2025-05-07
ER

PT J
AU Ghodousi, A
Tagliani, E
Karunaratne, E
Niemann, S
Perera, J
Köser, CU
Cirillo, DM
AF Ghodousi, Arash
Tagliani, Elisa
Karunaratne, Eranga
Niemann, Stefan
Perera, Jennifer
Koser, Claudio U.
Cirillo, Daniela Maria
TI Isoniazid Resistance in Mycobacterium tuberculosis Is a Heterogeneous Phenotype Composed of Overlapping MIC Distributions with Different Underlying Resistance Mechanisms
SO ANTIMICROBIAL AGENTS AND CHEMOTHERAPY
LA English
DT Article
DE MGIT 960; MIC; Mycobacterium tuberculosis; isoniazid resistance; whole-genome sequencing
ID MUTATION
AB MIC testing using the Bactec mycobacteria growth indicator tube system 960 of 70 phylogenetically diverse, isoniazid-resistant clinical strains of Mycobacterium tuberculosis revealed a complex pattern of overlapping MIC distributions. Whole-genome sequencing explained most of the levels of resistance observed. The MIC distribution of strains with only inhA promoter mutations was split by the current concentration endorsed by the Clinical and Laboratory Standards Institute to detect low-level resistance to isoniazid and is, consequently, likely not optimally set.

C1 [Ghodousi, Arash; Tagliani, Elisa; Cirillo, Daniela Maria] IRCCS San Raffaele Sci Inst, Div Immunol Transplantat & Infect Dis, Emerging Bacterial Pathogens Unit, Milan, Italy.
[Karunaratne, Eranga; Perera, Jennifer] Univ Colombo, Fac Med, Colombo, Sri Lanka.
[Niemann, Stefan] Res Ctr Borstel, Mol & Expt Mycohacteriol, Borstel, Germany.
[Niemann, Stefan] Partner Site Hamburg Lubeck Borstel Riems, German Ctr Infect Res, Riems, Germany.
[Koser, Claudio U.] Univ Cambridge, Dept Genet, Cambridge, England.
C3 Vita-Salute San Raffaele University; IRCCS Ospedale San Raffaele; University of Colombo; Leibniz Association; Forschungszentrum Borstel; German Center for Infection Research; University of Cambridge
RP Cirillo, DM (autor correspondente), IRCCS San Raffaele Sci Inst, Div Immunol Transplantat & Infect Dis, Emerging Bacterial Pathogens Unit, Milan, Italy.
EM cirillo.daniela@hsr.it
RI Tagliani, Elisa/AAN-1955-2020; Niemann, Stefan/C-9327-2011; Cirillo, Daniela/AAB-3646-2019; Ghodousi, Arash/AAB-8698-2022; Koser, Claudio/I-3740-2013
OI Koser, Claudio/0000-0002-0232-846X; Niemann, Stefan/0000-0002-6604-0684;
Ghodousi, Arash/0000-0001-9334-1573
FU German Center for Infection Research; Deutsche Forschungsgemeinschaft (German Research Foundation) under Germany's Excellence Strategy [EXC 22167-390884018]; Leibniz Science Campus EvoLUNG (Evolutionary Medicine of the Lung)
FX S.N. received support by the German Center for Infection Research, the Deutsche Forschungsgemeinschaft (German Research Foundation) under Germany's Excellence Strategy (EXC 22167-390884018), and the Leibniz Science Campus EvoLUNG (Evolutionary Medicine of the Lung).
CR Ando H, 2014, MOL MICROBIOL, V91, P538, DOI 10.1111/mmi.12476
[Anonymous], WHOCDSTB201820
[Anonymous], 2003, Susceptibility Testing of Mycobacteria, Nocardiae, and Other Aerobic Actinomycetes; Approved Standard. NCCLS document M24-A. NCCLS, 940 West Valley Road
Chesov D, 2017, EUR RESPIR J, V50, DOI 10.1183/13993003.01752-2017
CLSI, 2018, SUSCEPTIBILITY TESTI
CLSI, 2018, M24 CLSI, V3
Dominguez J, 2016, INT J TUBERC LUNG D, V20, P24, DOI 10.5588/ijtld.15.0221
Goutelle S, 2018, J ANTIMICROB CHEMOTH, V73, P987, DOI [10.1093/jac/dkx477, 10.1093/jac/dky131]
Heyckendorf J, 2018, ANTIMICROB AGENTS CH, V62, DOI 10.1128/AAC.01550-17
Kahlmeter G, 2015, J ANTIMICROB CHEMOTH, V70, P2427, DOI 10.1093/jac/dkv145
Karunaratne GHRE, 2018, MICROB DRUG RESIST, V24, P844, DOI 10.1089/mdr.2017.0330
Kohl TA, 2018, PEERJ, V6, DOI 10.7717/peerj.5895
Köser CU, 2019, INT J INFECT DIS, V80, pS32, DOI 10.1016/j.ijid.2019.02.027
Lempens P, 2018, SCI REP-UK, V8, DOI 10.1038/s41598-018-21378-x

Machado D, 2013, J ANTIMICROB CHEMOTH, V68, P1728, DOI 10.1093/jac/dkt090
Miotto P, 2017, EUR RESPIR J, V50, DOI 10.1183/13993003.01354-2017
Mouton JW, 2018, J ANTIMICROB CHEMOTH, V73, P2585, DOI 10.1093/jac/dky195
Mouton JW, 2018, J ANTIMICROB CHEMOTH, V73, P564, DOI 10.1093/jac/dkx427
Schön T, 2019, CLIN MICROBIOL INFEC, V25, P403, DOI 10.1016/j.cmi.2019.01.019
Van Deun A, 2018, INT J TUBERC LUNG D, V22, P239, DOI 10.5588/ijtld.17.0660
Vilchèze C, 2006, NAT MED, V12, P1027, DOI 10.1038/nm1466
World Health Organization, 2018, WHOCDSTB201824
World Health Organization, 2016, WHOHTMTB201612
World Health Organization, 2018, Call for experts
Zignol M, 2018, LANCET INFECT DIS, V18, P675, DOI 10.1016/S1473-3099(18)30073-2
NR 25
TC 26
Z9 27
U1 0
U2 8
PU AMER SOC MICROBIOLOGY
PI WASHINGTON
PA 1752 N ST NW, WASHINGTON, DC 20036-2904 USA
SN 0066-4804
EI 1098-6596
J9 ANTIMICROB AGENTS CH
JI Antimicrob. Agents Chemother.
PD JUL
PY 2019
VL 63
IS 7
AR e00092-19
DI 10.1128/AAC.00092-19
PG 4
WC Microbiology; Pharmacology & Pharmacy
WE Science Citation Index Expanded (SCI-EXPANDED)
SC Microbiology; Pharmacology & Pharmacy
GA IE8KP
UT WOS:000472622600005
PM 31010866
OA hybrid, Green Published, Green Submitted
DA 2025-05-07
ER

PT J
AU Swanson, ZS
Bethancourt, H
Saunders, J
Nzunza, R
Ndiema, E
Braun, D
Rosinger, AY

Pontzer, H
AF Swanson, Zane S.
Bethancourt, Hilary
Saunders, Jessica
Nzunza, Rosemary
Ndiema, Emmanuel
Braun, David
Rosinger, Asher Y.
Pontzer, Herman
TI Distinct patterns of early childhood growth represent variation in life
history strategy among Daasanach pastoralists living in Northern Kenya
SO AMERICAN JOURNAL OF PHYSICAL ANTHROPOLOGY
LA English
DT Meeting Abstract
C1 [Swanson, Zane S.; Saunders, Jessica; Pontzer, Herman] Duke Univ, Evolutionary Anthropol, Durham, NC 27706 USA.
[Bethancourt, Hilary; Rosinger, Asher Y.] Penn State Univ, Dept Biobehav Hlth, University Pk, PA 16802 USA.
[Nzunza, Rosemary] Kenya Govt Med Res Ctr, KEMRI, Nairobi, Kenya.
[Ndiema, Emmanuel] Natl Museums Kenya, Dept Earth Sci, Nairobi, Kenya.
[Braun, David] George Washington Univ, Ctr Adv Study Human Paleobiol, Dept Anthropol, Washington, DC 20052 USA.
[Braun, David] Max Planck Inst Evolutionary Anthropol, Dept Human Evolut, Leipzig, Germany.
[Rosinger, Asher Y.] Penn State Univ, Dept Anthropol, University Pk, PA 16802 USA.
[Pontzer, Herman] Duke Univ, Global Hlth Inst, Durham, NC 27706 USA.
C3 Duke University; Pennsylvania Commonwealth System of Higher Education
(PCSHE); Pennsylvania State University; Pennsylvania State University -
University Park; Penn State Behrend; Kenya Medical Research Institute;
George Washington University; Max Planck Society; Pennsylvania
Commonwealth System of Higher Education (PCSHE); Pennsylvania State
University; Penn State Behrend; Pennsylvania State University -
University Park; Duke University
RI Braun, David/C-9680-2009
FU NSF ARCH [1624398]; NSF REU [1930719]; NSF CNH2-S [1924322]; PSU SSRI
Human Health and Environment Seed Grant; Triangle Center for
Evolutionary Medicine Graduate Research Grant
FX NSF ARCH #1624398; NSF REU #1930719; NSF CNH2-S #1924322; also funded by
the PSU SSRI Human Health and Environment Seed Grant, and the Triangle
Center for Evolutionary Medicine Graduate Research Grant.
NR 0
TC 0
Z9 0
U1 0
U2 2
PU WILEY
PI HOBOKEN
PA 111 RIVER ST, HOBOKEN 07030-5774, NJ USA
SN 0002-9483
EI 1096-8644

J9 AM J PHYS ANTHROPOL
JI Am. J. Phys. Anthropol.
PD MAR
PY 2021
VL 174
SU 71
SI SI
BP 101
EP 102
PG 2
WC Anthropology; Evolutionary Biology
WE Science Citation Index Expanded (SCI-EXPANDED); Social Science Citation Index (SSCI)
SC Anthropology; Evolutionary Biology
GA QR4KM
UT WOS:000625180200387
DA 2025-05-07
ER

PT J
AU Bornbusch, SL
Greene, LK
Rahobilalaina, S
Calkins, S
Rothman, RS
Clarke, TA
LaFleur, M
Drea, CM
AF Bornbusch, Sally L.
Greene, Lydia K.
Rahobilalaina, Sylvia
Calkins, Samantha
Rothman, Ryan S.
Clarke, Tara A.
LaFleur, Marni
Drea, Christine M.
TI Gut microbiota of ring-tailed lemurs (Lemur catta) vary across natural and captive populations and correlate with environmental microbiota
SO ANIMAL MICROBIOME
LA English
DT Article
ID VERTICAL TRANSMISSION; HOST GENETICS; WILD; BACTERIA; DIVERSITY; AGE; BIODIVERSITY; ACQUISITION; MADAGASCAR; EMERGENCE
AB Background Inter-population variation in host-associated microbiota reflects differences in the hosts' environments, but this characterization is typically based on studies comparing few populations. The diversity of natural habitats and captivity conditions occupied by any given host species has not been captured in these comparisons. Moreover, intraspecific variation in gut microbiota, generally attributed to diet, may also stem from differential acquisition of environmental microbes-an understudied mechanism by which host microbiomes are directly shaped by environmental microbes. To more comprehensively characterize gut microbiota in an ecologically flexible host, the ring-tailed lemur

(Lemur catta; n = 209), while also investigating the role of environmental acquisition, we used 16S rRNA sequencing of lemur gut and soil microbiota sampled from up to 13 settings, eight in the wilderness of Madagascar and five in captivity in Madagascar or the U.S. Based on matched fecal and soil samples, we used microbial source tracking to examine covariation between the two types of consortia. Results The diversity of lemur gut microbes varied markedly within and between settings. Microbial diversity was not consistently greater in wild than in captive lemurs, indicating that this metric is not necessarily an indicator of host habitat or environmental condition. Variation in microbial composition was inconsistent both with a single, representative gut community for wild conspecifics and with a universal 'signal of captivity' that homogenizes the gut consortia of captive animals. Despite the similar, commercial diets of captive lemurs on both continents, lemur gut microbiomes within Madagascar were compositionally most similar, suggesting that non-dietary factors govern some of the variability. In particular, soil microbial communities varied across geographic locations, with the few samples from different continents being the most distinct, and there was significant and context-specific covariation between gut and soil microbiota. Conclusions As one of the broadest, single-species investigations of primate microbiota, our study highlights that gut consortia are sensitive to multiple scales of environmental differences. This finding begs a reevaluation of the simple 'captive vs. wild' dichotomy. Beyond the important implications for animal care, health, and conservation, our finding that environmental acquisition may mediate aspects of host-associated consortia further expands the framework for how host-associated and environmental microbes interact across different microbial landscapes.
C1 [Bornbusch, Sally L.; Drea, Christine M.] Duke Univ, Dept Evolutionary Anthropol, Durham, NC 27708 USA.
[Greene, Lydia K.] Duke Lemur Ctr, Durham, NC USA.
[Rahobilalaina, Sylvia] Univ Antananarivo, Fac Sci, Antananarivo, Madagascar.
[Calkins, Samantha] Hunter Coll, Dept Psychol, Program Anim Behav & Conservat, New York, NY USA.
[Rothman, Ryan S.] SUNY Stony Brook, Inst Conservat Trop Environm, Interdept Doctoral Program Anthropol Sci, Stony Brook, NY 11794 USA.
[Clarke, Tara A.] North Carolina State Univ, Dept Sociol & Anthropol, Raleigh, NC USA.
[LaFleur, Marni] Univ San Diego, Dept Anthropol, 5998 Alcala Pk, San Diego, CA 92110 USA.
C3 Duke University; University Antananarivo; City University of New York (CUNY) System; Hunter College (CUNY); State University of New York (SUNY) System; Stony Brook University; North Carolina State University;
University of San Diego
RP Bornbusch, SL (autor correspondente), Duke Univ, Dept Evolutionary Anthropol, Durham, NC 27708 USA.
EM sally.bornbusch@gmail.com
OI Drea, Christine/0000-0002-3750-3344; Rothman, Ryan/0000-0002-8985-6168;
Bornbusch, Sally/0000-0001-9996-8369
FU National Science Foundation [BCS 1749465]; Triangle Center for Evolutionary Medicine; Kenan Institute for Ethics at Duke University;

Margot Marsh Biodiversity Fund
FX Funding was provided by awards from the National Science Foundation (BCS 1749465 to CMD), the Triangle Center for Evolutionary Medicine (Graduate Student Research Award to SLB), the Kenan Institute for Ethics at Duke University (Anthropocene Graduate Research Grant to SLB). During collections, ML and TC were funded by the Margot Marsh Biodiversity Fund.
CR Ahmed HI, 2019, FRONT MICROBIOL, V10, DOI 10.3389/fmicb.2019.00975
Altschul DM, 2019, PLOS ONE, V14, DOI 10.1371/journal.pone.0223675
Amato KR, 2019, ISME J, V13, P576, DOI 10.1038/s41396-018-0175-0
Amato KR, 2013, ISME J, V7, P1344, DOI 10.1038/ismej.2013.16
[Anonymous], 2001, MACH LEARN
Arora N, 2017, CELL HOST MICROBE, V21, P561, DOI 10.1016/j.chom.2017.04.007
Bahram M, 2018, NATURE, V560, P233, DOI 10.1038/s41586-018-0386-6
Baniel A, 2021, MICROBIOME, V9, DOI 10.1186/s40168-020-00977-9
Barelli C, 2020, MSYSTEMS, V5, DOI 10.1128/mSystems.00061-20
Barelli C, 2015, SCI REP-UK, V5, DOI 10.1038/srep14862
Ben Maamar S, 2020, J EXPO SCI ENV EPID, V30, P1, DOI 10.1038/s41370-019-0171-0
Bennett G, 2016, AM J PRIMATOL, V78, P883, DOI 10.1002/ajp.22555
Borbón-García A, 2017, FRONT MICROBIOL, V8, DOI 10.3389/fmicb.2017.01316
Bornbusch SL, 2021, ANIM MICROBIOME, V3, DOI 10.1186/s42523-021-00126-z
Bornbusch SL, 2019, AM J PRIMATOL, V81, DOI 10.1002/ajp.22986
Bornbusch SL, 2020, FEMS MICROBIOL ECOL, V96, DOI 10.1093/femsec/fiaa090
Borruso L, 2021, MICROB ECOL, V82, P215, DOI 10.1007/s00248-020-01677-5
Cameron A, 2013, DEV PRIMATOL-PROG PR, P227, DOI 10.1007/978-1-4614-8839-2_16
Campbell TP, 2020, ISME J, V14, P1584, DOI 10.1038/s41396-020-0634-2
Caporaso JG, 2012, ISME J, V6, P1621, DOI 10.1038/ismej.2012.8
Caravaggi A, 2018, J ZOO AQUAR RES, V6, P41
Cardona C, 2018, MSYSTEMS, V3, DOI 10.1128/mSystems.00052-18
Charpentier MJE, 2008, CONSERV GENET, V9, P1605, DOI 10.1007/s10592-007-9499-4
Chen CY, 2017, PLOS ONE, V12, DOI [10.1371/journal.pone.0181427, 10.1371/journal.pone.0182834]
Cheng YY, 2015, MICROBIOME, V3, DOI 10.1186/s40168-015-0143-0
Chi XW, 2019, BMC VET RES, V15, DOI 10.1186/s12917-019-1993-7
Chomel BB, 2007, EMERG INFECT DIS, V13, P6, DOI 10.3201/eid1301.060480
Choo JM, 2015, SCI REP-UK, V5, DOI 10.1038/srep16350
Clayton JB, 2018, SCI REP-UK, V8, DOI 10.1038/s41598-018-29277-x
Clayton JB, 2018, AM J PRIMATOL, V80, DOI 10.1002/ajp.22867
Clayton JB, 2016, P NATL ACAD SCI USA, V113, P10376, DOI 10.1073/pnas.1521835113
David LA, 2014, NATURE, V505, P559, DOI 10.1038/nature12820
Dietrich M, 2014, MOL ECOL, V23, P2783, DOI 10.1111/mec.12777
Dishman DL, 2009, APPL ANIM BEHAV SCI, V116, P88, DOI 10.1016/j.applanim.2008.06.012

DURRER S, 1994, P ROY SOC B-BIOL SCI, V258, P299, DOI 10.1098/rspb.1994.0176
Fedarko Marcus W, 2020, NAR Genom Bioinform, V2, plqaa023, DOI 10.1093/nargab/lqaa023
Fierer N, 2017, NAT REV MICROBIOL, V15, P579, DOI 10.1038/nrmicro.2017.87
Fogel AT, 2015, FOLIA PRIMATOL, V86, P85, DOI 10.1159/000369971
Frankel JS, 2019, AM J PRIMATOL, DOI 10.1002/ajp.23061
Fujimura KE, 2010, EXPERT REV ANTI-INFE, V8, P435, DOI [10.1586/eri.10.14, 10.1586/ERI.10.14]
Funkhouser LJ, 2013, PLOS BIOL, V11, DOI 10.1371/journal.pbio.1001631
Gabriel DN, 2013, INT J PRIMATOL, V34, P388, DOI 10.1007/s10764-013-9668-x
Ganzhorn JU, 2001, ORYX, V35, P346, DOI 10.1046/j.1365-3008.2001.00201.x
Gibson KM, 2019, SCI REP-UK, V9, DOI 10.1038/s41598-019-43875-3
Goodman SM, 2005, ORYX, V39, P73, DOI 10.1017/S0030605305000128
Gould Lisa, 2006, P255, DOI 10.1007/978-0-387-34586-4_12
Greene LK, 2021, ANIM MICROBIOME, V3, DOI 10.1186/s42523-021-00093-5
Greene LK, 2020, ISME J, V14, P1675, DOI 10.1038/s41396-020-0640-4
Greene LK, 2019, AM J PRIMATOL, V81, DOI 10.1002/ajp.22974
Greene LK, 2018, SCI REP-UK, V8, DOI 10.1038/s41598-018-32759-7
Grieneisen LE, 2019, P ROY SOC B-BIOL SCI, V286, DOI 10.1098/rspb.2019.0431
Griffiths SM, 2018, ISME J, V12, P2506, DOI 10.1038/s41396-018-0167-0
Grogan KE, 2017, ECOL EVOL, V7, P7638, DOI 10.1002/ece3.3317
Guarner F, 2006, NAT CLIN PRACT GASTR, V3, P275, DOI 10.1038/ncpgasthep0471
Guiyoule A, 1997, J CLIN MICROBIOL, V35, P2826, DOI 10.1128/JCM.35.11.2826-2833.1997
Hale VL, 2019, AM J PRIMATOL, V81, DOI 10.1002/ajp.22989
Hansen J, 2010, CURR OPIN GASTROEN, V26, P564, DOI 10.1097/MOG.0b013e32833f1195
Hartmann EM, 2016, ENVIRON SCI TECHNOL, V50, P9807, DOI 10.1021/acs.est.6b00262
Hicks AL, 2018, NAT COMMUN, V9, DOI 10.1038/s41467-018-04204-w
Hyde ER, 2016, MSYSTEMS, V1, DOI 10.1128/mSystems.00046-16
Inoue R, 2003, FEMS MICROBIOL ECOL, V46, P213, DOI 10.1016/S0168-6496(03)00215-0
Jeffries CL, 2018, SCI REP-UK, V8, DOI 10.1038/s41598-018-35658-z
JOHNS T, 1991, AM J CLIN NUTR, V53, P448, DOI 10.1093/ajcn/53.2.448
Jolly Alison, 2006, P3
Kaakoush NO, 2015, FRONT CELL INFECT MI, V5, DOI [10.3389/fcimb.2015.00084, 10.3309/fcimb.2015.00004]
Kartzinel TR, 2019, P NATL ACAD SCI USA, V116, P23588, DOI 10.1073/pnas.1905666116
Kikuchi Y, 2007, APPL ENVIRON MICROB, V73, P4308, DOI 10.1128/AEM.00067-07
Kohl KD, 2014, CONSERV PHYSIOL, V2, DOI 10.1093/conphys/cou009
Kohl KD, 2014, ENV MICROBIOL REP, V6, P191, DOI 10.1111/1758-2229.12118
Krishnamani R, 2000, ANIM BEHAV, V59, P899, DOI 10.1006/anbe.1999.1376
Kulkarni S, 2007, BEHAV PROCESS, V74, P88, DOI 10.1016/j.beproc.2006.10.005

Kuthyar S, 2019, CURR OPIN MICROBIOL, V50, P8, DOI 10.1016/j.mib.2019.09.001
LaFleur M, 2021, EMERG INFECT DIS, V27, P977, DOI 10.3201/eid2703.202924
LaFleur M, 2019, FOLIA PRIMATOL, V90, P199, DOI 10.1159/000496970
Larsen PA, 2016, BIOL LETTERS, V12, DOI 10.1098/rsbl.2015.0829
Leclaire S, 2014, BEHAV ECOL, V25, P996, DOI 10.1093/beheco/aru074
Leftwich PT, 2020, P ROY SOC B-BIOL SCI, V287, DOI 10.1098/rspb.2020.0820
Li H, 2016, FRONT MICROBIOL, V7, DOI 10.3389/fmicb.2016.01269
Linnenbrink M, 2013, MOL ECOL, V22, P1904, DOI 10.1111/mec.12206
Liu J, 2015, CURR MICROBIOL, V70, P67, DOI 10.1007/s00284-014-0672-y
Liu J, 2014, FEMS MICROBIOL ECOL, V87, P576, DOI 10.1111/1574-6941.12246
Ma T, 2020, ISME J, V14, P2223, DOI 10.1038/s41396-020-0678-3
Martínez-Mota R, 2020, ISME J, V14, P67, DOI 10.1038/s41396-019-0497-6
Mason GJ, 2010, TRENDS ECOL EVOL, V25, P713, DOI 10.1016/j.tree.2010.08.011
McKenney Erin A., 2017, Microbial Ecology in Health and Disease, V28, P1335165, DOI 10.1080/16512235.2017.1335165
McKenzie VJ, 2017, INTEGR COMP BIOL, V57, P690, DOI 10.1093/icb/icx090
McPherson F.J., 2013, J PRIMATOL, V2, P1, DOI [10.4172/2167-6801.1000e121, DOI 10.4172/2167-6801.1000112]
Melfi V, 2005, APPL ANIM BEHAV SCI, V90, P97, DOI 10.1016/j.applanim.2004.08.017
Milani C., 2020, Applied and Environmental Microbiology, V86
Mills JG, 2017, RESTOR ECOL, V25, P866, DOI 10.1111/rec.12610
Mittermeier R.A., 1988, P145
Moeller AH, 2015, MOL ECOL, V24, P690, DOI 10.1111/mec.13057
Morton JT, 2019, NAT COMMUN, V10, DOI 10.1038/s41467-019-10656-5
Mowry CB., 2001, TAG SSP HUSBANDRY NO
Mushegian AA, 2019, APPL ENVIRON MICROB, V85, DOI 10.1128/AEM.01547-18
Narat V, 2020, SCI REP-UK, V10, DOI 10.1038/s41598-020-75847-3
Nelson TM, 2013, ENVIRON MICROBIOL, V15, P1132, DOI 10.1111/1462-2920.12022
Nishida AH, 2019, NAT REV GENET, V20, P195, DOI 10.1038/s41576-018-0085-z
Orsted M, 2021, POPULATION BOTTLENEC
Peccia J, 2016, TRENDS MICROBIOL, V24, P595, DOI 10.1016/j.tim.2016.04.007
Pedersen AB, 2009, ECOHEALTH, V6, P496, DOI 10.1007/s10393-010-0284-3
Quast C, 2013, NUCLEIC ACIDS RES, V41, pD590, DOI 10.1093/nar/gks1219
REED KE, 1995, P NATL ACAD SCI USA, V92, P7874, DOI 10.1073/pnas.92.17.7874
Ren TT, 2017, MICROBIOME, V5, DOI 10.1186/s40168-017-0382-3
Rothschild D, 2018, NATURE, V555, P210, DOI 10.1038/nature25973
Sarkar A, 2020, NAT ECOL EVOL, V4, P1020, DOI 10.1038/s41559-020-1220-8
Schmidt E, 2019, ISME J, V13, P1293, DOI 10.1038/s41396-019-0345-8
Schwitzer C, 2014, SCIENCE, V343, P842, DOI 10.1126/science.1245783
Scott TM, 2002, APPL ENVIRON MICROB, V68, P5796, DOI 10.1128/AEM.68.12.5796-5803.2002
Seedorf H, 2014, CELL, V159, P253, DOI 10.1016/j.cell.2014.09.008
Selway CA, 2020, ENVIRON INT, V145, DOI 10.1016/j.envint.2020.106084

Shenhav L, 2019, NAT METHODS, V16, P627, DOI 10.1038/s41592-019-0431-x
Smith CCR, 2015, ISME J, V9, P2515, DOI 10.1038/ismej.2015.64
Song SJ, 2016, MSYSTEMS, V1, DOI 10.1128/mSystems.00021-16
Sun YW, 2020, FRONT MICROBIOL, V10, DOI 10.3389/fmicb.2019.03156
Suzuki TA, 2021, CODIVERSIFICATION GU
Sylvain FÉ, 2017, SCI REP-UK, V7, DOI 10.1038/s41598-017-05662-w
Tasnim N, 2017, FRONT MICROBIOL, V8, DOI 10.3389/fmicb.2017.01935
Tenhumberg B, 2004, CONSERV BIOL, V18, P1304, DOI 10.1111/j.1523-1739.2004.00246.x
Theis KR, 2012, SCI REP-UK, V2, DOI 10.1038/srep00615
Thompson LR, 2017, NATURE, V551, P457, DOI 10.1038/nature24621
Tong Q, 2020, FRONT MICROBIOL, V11, DOI 10.3389/fmicb.2020.02096
Tout J, 2017, ENV MICROBIOL REP, V9, P750, DOI 10.1111/1758-2229.12591
Trevelline BK, 2019, P ROY SOC B-BIOL SCI, V286, DOI 10.1098/rspb.2018.2448
Trosvik P, 2018, SCI REP-UK, V8, DOI 10.1038/s41598-017-18435-2
Tsukayama P, 2018, MSYSTEMS, V3, DOI 10.1128/mSystems.00016-18
Tung J, 2015, ELIFE, V4, DOI 10.7554/eLife.05224
Vernier CL, 2020, SCI ADV, V6, DOI 10.1126/sciadv.abd3431
Walke JB, 2014, ISME J, V8, P2207, DOI 10.1038/ismej.2014.77
Watson SE, 2019, ISME J, V13, P2916, DOI 10.1038/s41396-019-0480-2
Webster TMU, 2018, APPL ENVIRON MICROB, V84, DOI 10.1128/AEM.00691-18
Wei LM, 2020, ANIMALS-BASEL, V10, DOI 10.3390/ani10112125
Won YJ, 2003, APPL ENVIRON MICROB, V69, P6785, DOI 10.1128/AEM.69.11.6785-6792.2003
Xiao YH, 2019, PEERJ, V7, DOI 10.7717/peerj.6844
Yao R, 2019, GLOB ECOL CONSERV, V18, DOI 10.1016/j.gecco.2019.e00644
Yarza P, 2014, NAT REV MICROBIOL, V12, P635, DOI 10.1038/nrmicro3330
Yatsunenko T, 2012, NATURE, V486, P222, DOI 10.1038/nature11053
Youngblut ND, 2019, NAT COMMUN, V10, DOI 10.1038/s41467-019-10191-3
Yuan ML, 2015, MOL ECOL, V24, P2521, DOI 10.1111/mec.13169
Zaneveld JR, 2017, NAT MICROBIOL, V2, DOI 10.1038/nmicrobiol.2017.121
NR 140
TC 33
Z9 36
U1 3
U2 44
PU BMC
PI LONDON
PA CAMPUS, 4 CRINAN ST, LONDON N1 9XW, ENGLAND
EI 2524-4671
J9 ANIM MICROBIOME
JI Anim. Microbiome
PD APR 28
PY 2022
VL 4
IS 1
AR 29
DI 10.1186/s42523-022-00176-x
PG 19
WC Microbiology; Veterinary Sciences
WE Emerging Sources Citation Index (ESCI)
SC Microbiology; Veterinary Sciences
GA 0V8HB

UT WOS:000788579600001
PM 35484581
OA gold, Green Published, Green Submitted
DA 2025-05-07
ER

PT J
AU van Schaik, K
   Vinichenko, D
   Rühli, F
AF van Schaik, Katherine
   Vinichenko, Dmitry
   Ruehli, Frank
TI Health Is Not Always Written in Bone: Using a Modern Comorbidity Index to Assess Disease Load in Paleopathology
SO AMERICAN JOURNAL OF PHYSICAL ANTHROPOLOGY
LA English
DT Article
DE osteological paradox; chronic disease; multimorbidity
ID RISK ADJUSTMENT; CARE
AB Paleopathology has revealed much about disease in the past but is usually limited to conditions with osteological manifestations; this often excludes acute soft tissue infections and causes of death for most individuals in the past and present. Our understanding of the evolution of disease is essential for contextualizing and predicting the epidemiological shifts that are happening in modern society, as high rates of infectious disease coexist alongside high rates of chronic disease in rates unlike those observed previously in human history. Moreover, many physiological states not previously classified as disease (obesity) have become pathologized, influencing our conception of disease and what defines health. By using the Galler Collection, a pre-antibiotic and pre-chemotherapeutic osteological series with modern autopsy records, our research quantifies disease burden of the past using the Charlson Index (CI), a modern comorbidity index of disease severity. Galler Collection remains and autopsy records were scored with the Charlson Index to correlate bone findings with soft tissue findings, and statistical analysis was performed for cumulative scores and absolute diagnosis counts, with patients stratified by sex and cause of death (pneumonia or cancer). Osteological diagnosis counts were more predictive of soft-tissue autopsy disease counts than were associated cumulative CI scores. Diagnosis counts and CI scores for osteological data were more closely related to associated soft tissue data for cancer patients than for pneumonia patients. This research indicates how interdisciplinary paleopathological analysis assists in making more reliable assessments of health and mortality in the past, with implications for trending and predicting future epidemiological shifts. Am J Phys Anthropol 154:215-221, 2014. (c) 2014 Wiley Periodicals, Inc.
C1 [van Schaik, Katherine] Harvard Univ, Sch Med, Cambridge, MA 02138 USA.
   [van Schaik, Katherine] Harvard Dept Class, Cambridge, MA USA.
   [Vinichenko, Dmitry] Harvard Univ, Dept Chem & Chem Biol, Cambridge, MA 02138 USA.
   [Ruehli, Frank] Univ Zurich, Ctr Evolutionary Med, Inst Anat, CH-8006 Zurich, Switzerland.

C3 Harvard University; Harvard University; University of Zurich
RP van Schaik, K (autor correspondente), 501 Oak Brook Dr, Columbia, SC 29223 USA.
EM katherine_vanschaik@hms.harvard.edu; frank.ruhli@anatom.uzh.ch
RI Ruhli, Frank/H-8789-2017
OI Ruhli, Frank/0000-0001-7937-001X
FU Harvard Department of the Classics; Harvard Medical School MD-PhD Social
Sciences Program; Harvard Graduate School of Arts and Sciences; Centre for Evolutionary Medicine at the University of Zurich, Maxi Foundation
FX Grant sponsors: Harvard Department of the Classics, the Harvard Medical
School MD-PhD Social Sciences Program, Harvard Graduate School of Arts and Sciences, Centre for Evolutionary Medicine at the University of Zurich, Maxi Foundation.
CR Amer Diabet Assoc, 2011, DIABETES CARE, V34, pS11, DOI [10.2337/dc10-S011, 10.2337/dc13-S067, 10.2337/dc12-s064, 10.2337/dc11-S062, 10.2337/dc11-S011, 10.2337/dc10-S062, 10.2337/dc14-S081, 10.2337/dc12-s011, 10.2337/dc13-S011]
Aufderheide A.C., 1998, CAMBRIDGE ENCY HUMAN
CHARLSON ME, 1987, J CHRON DIS, V40, P373, DOI 10.1016/0021-9681(87)90171-8
DaVita V.T., 2005, Cancer Principles and Practice of Oncology, V7th
KNAUS WA, 1991, CHEST, V100, P1619, DOI 10.1378/chest.100.6.1619
Moynihan R, 2002, BMJ-BRIT MED J, V324, P859, DOI 10.1136/bmj.324.7342.859
Needham DM, 2005, J CRIT CARE, V20, P12, DOI 10.1016/j.jcrc.2004.09.007
Quan HD, 2011, AM J EPIDEMIOL, V173, P676, DOI 10.1093/aje/kwq433
Rühli FJ, 2003, AM J PHYS ANTHROPOL, V121, P15, DOI 10.1002/ajpa.10219
SAVAGE DG, 1982, ANN INTERN MED, V96, P47, DOI 10.7326/0003-4819-96-1-47
Smith R, 2002, BRIT MED J, V324, P883, DOI 10.1136/bmj.324.7342.883
Tinetti ME, 2012, JAMA-J AM MED ASSOC, V307, P2493, DOI 10.1001/jama.2012.5265
Vital Statistics of the US, 1980, VIT STAT US 1980 198
WOOD JW, 1992, CURR ANTHROPOL, V33, P343, DOI 10.1086/204084
World Health Organization, 2008, TOP 10 CAUS DEATH 20
Wright LE., 2003, Journal of Archaeological Research, V11, P43
NR 16
TC 19
Z9 23
U1 2
U2 26
PU WILEY
PI HOBOKEN
PA 111 RIVER ST, HOBOKEN 07030-5774, NJ USA
SN 0002-9483
EI 1096-8644
J9 AM J PHYS ANTHROPOL
JI Am. J. Phys. Anthropol.
PD JUN
PY 2014
VL 154

IS 2
BP 215
EP 221
DI 10.1002/ajpa.22494
PG 7
WC Anthropology; Evolutionary Biology
WE Science Citation Index Expanded (SCI-EXPANDED); Social Science Citation Index (SSCI)
SC Anthropology; Evolutionary Biology
GA AH9VM
UT WOS:000336492900004
PM 24936606
DA 2025-05-07
ER

PT J
AU Blawas, AM
Ware, KE
Schmaltz, E
Zheng, L
Spruance, J
Allen, AS
West, N
Devos, N
Corcoran, DL
Nowacek, DP
Eward, WC
Fahlman, A
Somarelli, JA
AF Blawas, Ashley M.
Ware, Kathryn E.
Schmaltz, Emma
Zheng, Larry
Spruance, Jacob
Allen, Austin S.
West, Nicole
Devos, Nicolas
Corcoran, David L.
Nowacek, Douglas P.
Eward, William C.
Fahlman, Andreas
Somarelli, Jason A.
TI An integrated comparative physiology and molecular approach pinpoints mediators of breath-hold capacity in dolphins
SO EVOLUTION MEDICINE AND PUBLIC HEALTH
LA English
DT Article
DE ischemic stress tolerance; cetaceans; diving physiology; oceans and human health; ALOX5; lipoxygenase
ID EVOLUTIONARY GENETICS; ISCHEMIA-REPERFUSION; HYPOXIA TOLERANCE; DIVING SEALS; BLOOD-FLOW; APOPTOSIS; IDENTIFICATION; ADAPTATIONS; RESPIRATION;
ATTENUATION

AB Background and objectives: Ischemic events, such as ischemic heart disease and stroke, are the number one cause of death globally. Ischemia prevents blood, carrying essential nutrients and oxygen, from reaching tissues, leading to cell and tissue death, and eventual organ failure. While humans are relatively intolerant to ischemic events, other species, such as marine mammals, have evolved a unique tolerance to chronic ischemia/reperfusion during apneic diving. To identify possible molecular features of an increased tolerance for apnea, we examined changes in gene expression in breath-holding dolphins.

Methodology: Here, we capitalized on the adaptations possesed by bottlenose dolphins (Tursiops truncatus) for diving as a comparative model of ischemic stress and hypoxia tolerance to identify molecular features associated with breath holding. Given that signals in the blood may influence physiological changes during diving, we used RNA-Seq and enzyme assays to examine time-dependent changes in gene expression in the blood of breath-holding dolphins.

Results: We observed time-dependent upregulation of the arachidonate 5-lipoxygenase (ALOX5) gene and increased lipoxygenase activity during breath holding. ALOX5 has been shown to be activated during hypoxia in rodent models, and its metabolites, leukotrienes, induce vasoconstriction.

Conclusions and implications: The upregulation of ALOX5 mRNA occurred within the calculated aerobic dive limit of the species, suggesting that ALOX5 may play a role in the dolphin's physiological response to diving, particularly in a pro-inflammatory response to ischemia and in promoting vasoconstriction. These observations pinpoint a potential molecular mechanism by which dolphins, and perhaps other marine mammals, respond to the prolonged breath holds associated with diving.

Lay Summary: Reductions in blood flow are associated with tissue damage in humans; however, marine mammals have evolved remarkable tolerance to reductions in tissue blood flow during diving. We found that changes in gene expression in breath-holding dolphins may support a response to diving highlighting a potential molecular underpinning for apnea tolerance.
C1 [Blawas, Ashley M.; Schmaltz, Emma; Zheng, Larry; Allen, Austin S.; Nowacek, Douglas P.] Duke Univ, Marine Lab, Nicholas Sch Environm, Beaufort, NC 28516 USA.

[Ware, Kathryn E.; Spruance, Jacob; Somarelli, Jason A.] Duke Univ, Dept Med, Med Ctr, Durham, NC 27710 USA.

[West, Nicole] Dolphin Quest, Honolulu, HI USA.

[Devos, Nicolas; Corcoran, David L.] Duke Univ, Duke Ctr Genom & Computat Biol, Durham, NC 27710 USA.

[Nowacek, Douglas P.] Duke Univ, Pratt Sch Engn, Durham, NC 27710 USA.

[Eward, William C.] Duke Univ, Dept Orthopaed Surg, Med Ctr, Durham, NC 27710 USA.

[Eward, William C.; Somarelli, Jason A.] Duke Univ, Duke Canc Inst, Med Ctr, Durham, NC 27710 USA.

[Fahlman, Andreas] Global Diving Res Inc, Ottawa, ON K2J 5E8, Canada.

[Fahlman, Andreas] Fdn Oceanog Comunitat Valenciana, Res Dept, Valencia, Spain.

C3 Duke University; Duke University; Duke University; Duke University; Duke
University; Duke University

RP Somarelli, JA (autor correspondente), Duke Univ, Duke Canc Inst, Med Ctr, Durham, NC 27710 USA.; Fahlman, A (autor correspondente), Global Diving Res Inc, Ottawa, ON K2J 5E8, Canada.
EM andreas.fahlman@duke.edu; jason.-somarelli@duke.edu
RI Fahlman, Andreas/A-2901-2011; Allen, Austin/IWU-4480-2023; Devos, Nicolas/E-7490-2015
OI Somarelli, Jason/0000-0003-1510-9343; Spruance, Jacob/0000-0003-2744-8123
FU Dolphin Quest, Oahu; Triangle Center for Evolutionary Medicine; Bureau of Ocean Energy Management (BOEM) Environmental Study; E. Bayard Halsted Scholarship in Science, History and Journalism
FX This work was supported through Dolphin Quest, Oahu (J.A.S.) and the Triangle Center for Evolutionary Medicine (J.A.S.). A.M.B. was supported by a Bureau of Ocean Energy Management (BOEM) Environmental Study and the E. Bayard Halsted Scholarship in Science, History and Journalism.
CR Allen KN, 2019, FRONT PHYSIOL, V10, DOI 10.3389/fphys.2019.01199
Anders S, 2015, BIOINFORMATICS, V31, P166, DOI 10.1093/bioinformatics/btu638
Andersson JPA, 2004, J APPL PHYSIOL, V96, P1005, DOI 10.1152/japplphysiol.01057.2002
[Anonymous], 2018, Global Health Estimates 2016: Deaths by Cause, Age, Sex, by Country and by Region, 2000-2016
ARNOLD RW, 1985, UNDERSEA BIOMED RES, V12, P183
Bagchi A, 2018, J EXP BIOL, V221, DOI 10.1242/jeb.178491
Bartels K, 2013, P NATL ACAD SCI USA, V110, P18351, DOI 10.1073/pnas.1318345110
Bi JL, 2015, MAR BIOL, V162, P1201, DOI 10.1007/s00227-015-2662-4
Blank U, 2011, LEUKEMIA, V25, P1379, DOI 10.1038/leu.2011.95
Blawas AM, 2021, J EXP BIOL, V224, DOI 10.1242/jeb.234096
BLIX AS, 1983, ACTA PHYSIOL SCAND, V118, P109, DOI 10.1111/j.1748-1716.1983.tb07250.x
BLIX AS, 1976, ACTA PHYSIOL SCAND, V96, P277, DOI 10.1111/j.1748-1716.1976.tb10196.x
Bona E, 1999, PEDIATR RES, V45, P500, DOI 10.1203/00006450-199904010-00008
Bostjancic E, 2009, DIS MARKERS, V27, P255, DOI 10.3233/DMA-2009-0671
Chen W, 2019, AM J TRANSL RES, V11, P7364
Cheong HS, 2005, J HUM GENET, V50, P628, DOI 10.1007/s10038-005-0304-2
Choi DW, 1996, CURR OPIN NEUROBIOL, V6, P667, DOI 10.1016/S0959-4388(96)80101-2
Davis RW, 1999, J EXP BIOL, V202, P1091
Dobin A, 2013, BIOINFORMATICS, V29, P15, DOI 10.1093/bioinformatics/bts635
ELSNER R, 1985, AM J PHYSIOL, V249, P1119, DOI 10.1152/ajpheart.1985.249.6.H1119
Elsner R, 1998, COMP BIOCHEM PHYS A, V119, P975, DOI 10.1016/S1095-6433(98)00012-9
Eltzschig HK, 2011, NEW ENGL J MED, V364, P656, DOI 10.1056/NEJMra0910283
Fahlman A, 2009, RESP PHYSIOL NEUROBI, V165, P28, DOI 10.1016/j.resp.2008.09.013
Fahlman A, 2019, SCI REP-UK, V9, DOI 10.1038/s41598-019-45064-8

Fahlman A, 2020, J EXP BIOL, V223
Fahlman A, 2020, FRONT PHYSIOL, V11, DOI 10.3389/fphys.2020.604018
Fahlman A, 2019, J EXP BIOL, V222, DOI 10.1242/jeb.192211
Fahlman A, 2018, FRONT PHYSIOL, V9, DOI 10.3389/fphys.2018.00838
Fala Loretta, 2016, Am Health Drug Benefits, V9, P106
Favilla AB, 2020, FRONT ECOL EVOL, V8, DOI 10.3389/fevo.2020.555509
FISH FE, 1993, J EXP BIOL, V185, P179
Folkow LP, 2008, NEUROSCI LETT, V446, P147, DOI 10.1016/j.neulet.2008.09.040
Franz M, 2018, NUCLEIC ACIDS RES, V46, pW60, DOI 10.1093/nar/gky311
FRIEDMAN Z, 1984, PROSTA LEUKOTR MED, V14, P279, DOI 10.1016/0262-1746(84)90211-7
Gaschler MM, 2017, BIOCHEM BIOPH RES CO, V482, P419, DOI 10.1016/j.bbrc.2016.10.086
Gottlieb RA, 1999, ANN NY ACAD SCI, V874, P412, DOI 10.1111/j.1749-6632.1999.tb09255.x
Grocott MPW, 2009, NEW ENGL J MED, V360, P140, DOI 10.1056/NEJMoa0801581
Guo LL, 2020, EUR REV MED PHARMACO, V24, P9041, DOI 10.26355/eurrev_202009_22849
Hindle AG, 2020, J APPL PHYSIOL, V128, P1439, DOI 10.1152/japplphysiol.00846.2019
HOCHACHKA PW, 1981, SCIENCE, V212, P509, DOI 10.1126/science.7209545
Hoff MLM, 2017, PLOS ONE, V12, DOI 10.1371/journal.pone.0169366
Huber W, 2015, NAT METHODS, V12, P115, DOI [10.1038/NMETH.3252, 10.1038/nmeth.3252]
Ichinose F, 2001, CIRC RES, V88, P832, DOI 10.1161/hh0801.089177
Romero JI, 2015, BBA-GEN SUBJECTS, V1850, P1274, DOI 10.1016/j.bbagen.2015.02.015
Irving L, 1941, J CELL COMPAR PHYSL, V17, P145, DOI 10.1002/jcp.1030170203
Johnson P, 2005, FREE RADICAL BIO MED, V39, P205, DOI 10.1016/j.freeradbiomed.2005.03.008
Johnson P, 2004, FREE RADICAL RES, V38, P847, DOI 10.1080/10715760410001725526
Kersey PJ, 2012, NUCLEIC ACIDS RES, V40, pD91, DOI 10.1093/nar/gkr895
Kooyman GL, 1998, ANNU REV PHYSIOL, V60, P19, DOI 10.1146/annurev.physiol.60.1.19
Krueger F., 2015, Babraham Bioinformatics6.10 (Version 0.6.10), DOI DOI 10.1093/BIOINFORMATICS/BTU170
Lentjes MHFM, 2016, EXPERT REV MOL MED, V18, DOI 10.1017/erm.2016.2
Li CJ, 2013, BMC MED GENOMICS, V6, DOI 10.1186/1755-8794-6-16
Lopes R, 2017, METHODS MOL BIOL, V1543, P45, DOI 10.1007/978-1-4939-6716-2_3
Love MI, 2014, GENOME BIOL, V15, DOI 10.1186/s13059-014-0550-8
Lu C, 2015, J MOL CELL CARDIOL, V89, P87, DOI 10.1016/j.yjmcc.2015.10.011
Martin M., 2011, EMBnet.journal, V17, P10, DOI DOI 10.14806/EJ.17.1.200
McKnight JC, 2019, PLOS BIOL, V17, DOI 10.1371/journal.pbio.3000306
Meir JU, 2009, AM J PHYSIOL-REG I, V297, pR927, DOI 10.1152/ajpregu.00247.2009
Mirceta S, 2013, SCIENCE, V340, DOI 10.1126/science.1234192
Mitsios N, 2006, PATHOBIOLOGY, V73, P159, DOI 10.1159/000096017

Mootha VK, 2003, NAT GENET, V34, P267, DOI 10.1038/ng1180
Mougey E, 2013, CLIN EXP ALLERGY, V43, P512, DOI 10.1111/cea.12076
Murdoch C, 2005, J IMMUNOL, V175, P6257, DOI 10.4049/jimmunol.175.10.6257
Nery MF, 2013, J MOL EVOL, V76, P380, DOI 10.1007/s00239-013-9572-1
Noren SR, 2012, J EXP BIOL, V215, P2735, DOI 10.1242/jeb.069583
Nowbar AN, 2019, CIRC-CARDIOVASC QUAL, V12, DOI 10.1161/CIRCOUTCOMES.118.005375
Pabst DA., 1999, BIOL MARINE MAMMALS
Panneton WM, 2013, PHYSIOLOGY, V28, P284, DOI 10.1152/physiol.00020.2013
Pavord ID, 2012, LANCET, V380, P651, DOI 10.1016/S0140-6736(12)60988-X
Poeckel D, 2010, CARDIOVASC RES, V86, P243, DOI 10.1093/cvr/cvq016
Porter KM, 2014, PLOS ONE, V9, DOI 10.1371/journal.pone.0098532
R Core Team, 2020, R: a language and environment for statistical computing. R Foundation for Statistical Computing v.4.3.0
RIDGWAY SH, 1969, SCIENCE, V166, P1651, DOI 10.1126/science.166.3913.1651
Scholander P. F., 1940, Hvalradets Skrifter, Vno. 22, P1
Scholander PF, 1942, J BIOL CHEM, V142, P431
Shannon P, 2003, GENOME RES, V13, P2498, DOI 10.1101/gr.1239303
Shattock MJ, 2012, J PHYSIOL-LONDON, V590, P3219, DOI 10.1113/jphysiol.2012.229864
Subramanian A, 2005, P NATL ACAD SCI USA, V102, P15545, DOI 10.1073/pnas.0506580102
Tian R, 2017, FRONT GENET, V8, DOI 10.3389/fgene.2017.00205
Tian R, 2016, GENOME BIOL EVOL, V8, P827, DOI 10.1093/gbe/evw037
Tift MS, 2014, J EXP BIOL, V217, P1752, DOI 10.1242/jeb.100677
VANCITTERS RL, 1965, COMP BIOCHEM PHYSIOL, V16, P267, DOI 10.1016/0010-406X(65)90324-5
Vázquez-Medina JP, 2011, CAN J ZOOL, V89, P206, DOI 10.1139/Z10-107
Vázquez-Medina JP, 2007, J EXP MAR BIOL ECOL, V345, P110, DOI 10.1016/j.jembe.2007.02.003
Vázquez-Medina JP, 2006, COMP BIOCHEM PHYS C, V142, P198, DOI 10.1016/j.cbpc.2005.09.004
Wang R, 2018, INT J BIOL SCI, V14, P1791, DOI 10.7150/ijbs.25352
Wilhelm D, 2002, COMP BIOCHEM PHYS A, V133, P885, DOI 10.1016/S1095-6433(02)00253-2
Witten A, 2020, PLOS ONE, V15, DOI 10.1371/journal.pone.0237928
Yim HS, 2014, NAT GENET, V46, P88, DOI 10.1038/ng.2835
ZAPOL WM, 1979, J APPL PHYSIOL, V47, P968, DOI 10.1152/jappl.1979.47.5.968
Zenteno-Savín T, 2002, COMP BIOCHEM PHYS C, V133, P527, DOI 10.1016/S1532-0456(02)00075-3
Zheng DF, 2018, CELL PHYSIOL BIOCHEM, V49, P2060, DOI 10.1159/000493716
Zhou XM, 2018, GENOME BIOL EVOL, V10, P967, DOI 10.1093/gbe/evy062
Zhu KL, 2018, J MOL EVOL, V86, P618, DOI 10.1007/s00239-018-9870-8
NR 94
TC 4
Z9 5
U1 2
U2 10
PU OXFORD UNIV PRESS

PI OXFORD
PA GREAT CLARENDON ST, OXFORD OX2 6DP, ENGLAND
EI 2050-6201
J9 EVOL MED PUBLIC HLTH
JI Evol. Med. Public Health
PY 2021
VL 9
IS 1
BP 420
EP 430
DI 10.1093/emph/eoab036
EA DEC 2021
PG 11
WC Evolutionary Biology; Public, Environmental & Occupational Health
WE Science Citation Index Expanded (SCI-EXPANDED)
SC Evolutionary Biology; Public, Environmental & Occupational Health
GA YL3PE
UT WOS:000745805600001
PM 35169481
OA Green Submitted, gold, Green Published
DA 2025-05-07
ER

PT J
AU Somarelli, JA
   Rupprecht, G
   Altunel, E
   Flamant, EM
   Rao, S
   Sivaraj, D
   Lazarides, AL
   Hoskinson, SM
   Sheth, MU
   Cheng, S
   Kim, SY
   Ware, KE
   Agarwal, A
   Cullen, MM
   Selmic, LE
   Everitt, JI
   McCall, SJ
   Eward, C
   Eward, WC
   Hsu, DS
AF Somarelli, Jason A.
   Rupprecht, Gabrielle
   Altunel, Erdem
   Flamant, Etienne M.
   Rao, Sneha
   Sivaraj, Dharshan
   Lazarides, Alexander L.
   Hoskinson, Sarah M.
   Sheth, Maya U.
   Cheng, Serene

Kim, So Young
Ware, Kathryn E.
Agarwal, Anika
Cullen, Mark M.
Selmic, Laura E.
Everitt, Jeffrey I.
McCall, Shannon J.
Eward, Cindy
Eward, William C.
Hsu, David S.
TI A Comparative Oncology Drug Discovery Pipeline to Identify and Validate
New Treatments for Osteosarcoma
SO CANCERS
LA English
DT Article
DE comparative oncology; patient-derived xenografts; precision medicine;
CRM1; proteasome; sarcoma
ID PATIENT-DERIVED ORGANOIDS; COLORECTAL-CANCER; NUCLEAR EXPORT; CANINE
OSTEOSARCOMA; MULTIPLE-MYELOMA; MODEL; CHEMOTHERAPY; EURAMOS-1
AB Simple Summary
Osteosarcoma is a rare bone cancer that occurs primarily in children. The discovery of new treatments for osteosarcoma and other rare cancer types has been severely limited by access to patient samples to study these often-complex diseases. Here we capitalize on naturally-occurring cancers in pet dogs to study the biology of these rare cancers. Using living cells from canine and human patients to test thousands of drugs simultaneously, we identify a unique combination of drugs that disrupts protein degradation and protein trafficking in cancer cells. This drug combination represents a promising new treatment to treat both dogs and people with osteosarcoma.

Background: Osteosarcoma is a rare but aggressive bone cancer that occurs primarily in children. Like other rare cancers, treatment advances for osteosarcoma have stagnated, with little improvement in survival for the past several decades. Developing new treatments has been hampered by extensive genomic heterogeneity and limited access to patient samples to study the biology of this complex disease. Methods: To overcome these barriers, we combined the power of comparative oncology with patient-derived models of cancer and high-throughput chemical screens in a cross-species drug discovery pipeline. Results: Coupling in vitro high-throughput drug screens on low-passage and established cell lines with in vivo validation in patient-derived xenografts we identify the proteasome and CRM1 nuclear export pathways as therapeutic sensitivities in osteosarcoma, with dual inhibition of these pathways inducing synergistic cytotoxicity. Conclusions: These collective efforts provide an experimental framework and set of new tools for osteosarcoma and other rare cancers to identify and study new therapeutic vulnerabilities.
C1 [Somarelli, Jason A.; Rupprecht, Gabrielle; Altunel, Erdem; Flamant, Etienne M.; Sivaraj, Dharshan; Sheth, Maya U.; Cheng, Serene; Ware, Kathryn E.; Agarwal, Anika; Hsu, David S.] Duke Univ, Dept Med, Med Ctr, Durham, NC 27710 USA.
[Somarelli, Jason A.; Everitt, Jeffrey I.; McCall, Shannon J.; Eward, William C.; Hsu, David S.] Duke Canc Inst, Durham, NC 27710 USA.

[Rao, Sneha; Lazarides, Alexander L.; Hoskinson, Sarah M.; Cullen, Mark M.; Eward, William C.] Duke Univ, Dept Orthopaed, Med Ctr, Durham, NC 27710 USA.
[Kim, So Young] Duke Univ, Dept Mol Genet & Microbiol, Med Ctr, Durham, NC 27710 USA.
[Selmic, Laura E.] Ohio State Univ, Coll Vet Med, Columbus, OH 43210 USA.
[Everitt, Jeffrey I.; McCall, Shannon J.] Duke Univ, Dept Pathol, Med Ctr, Durham, NC 27710 USA.
[Eward, Cindy] Triangle Vet Referral Hosp, Surg Serv, Durham, NC 27710 USA.
C3 Duke University; Duke University; Duke University; Duke University; University System of Ohio; Ohio State University; Duke University
RP Somarelli, JA (autor correspondente), Duke Univ, Dept Med, Med Ctr, Durham, NC 27710 USA.; Somarelli, JA (autor correspondente), Duke Canc Inst, Durham, NC 27710 USA.
EM jason.somarelli@duke.edu; gabrielle.rupprecht@duke.edu; erdemaltunel90@gmail.com; etienne.flamant@duke.edu; sneha.rao@duke.edu; ds311@stanford.edu; alexander.lazarides@duke.edu; sarah.hoskinson@duke.edu; maya.sheth@duke.edu; serene.cheng@duke.edu; soyoung.kim@duke.edu; kathryn.ware@duke.edu; anika.agarwal@duke.edu; mark.cullen@duke.edu; selmic.1@osu.edu; jeffrey.everitt@duke.edu; Shannon.mccall@duke.edu; cindyewarddvm@tvrhdurham.com; william.eward@duke.edu; shiaowen.hsu@duke.edu
RI Nikova, Alexandrina/L-6263-2019
OI Everitt, Jeffrey/0000-0003-0273-6284; Cullen, Mark/0000-0003-1322-4858; Sheth, Maya/0000-0003-1466-8899; Agarwal, Anika/0000-0002-8820-7297; Altunel, Erdem/0000-0003-1821-0156; Somarelli, Jason/0000-0003-1510-9343; McCall, Shannon/0000-0003-3957-061X; Kim, So Young/0000-0002-5671-1878; Lazarides, Alexander/0000-0003-0704-1529; Sivaraj, Dharshan/0000-0001-7453-0045
FU Triangle Center for Evolutionary Medicine; Hyundai Hope on Wheels; Ryan Foundation; Consortium for Canine Comparative Oncology; Orthopedic Research and Education Foundation Resident Research Grant
FX This work was supported by the Triangle Center for Evolutionary Medicine (J.A.S.), Hyundai Hope on Wheels (W.C.E.), the Ryan Foundation (W.C.E.), the Consortium for Canine Comparative Oncology (W.C.E./J.A.S.), and an Orthopedic Research and Education Foundation Resident Research Grant (A.L.L.).
CR Alexander TB, 2016, J CLIN ONCOL, V34, P4094, DOI 10.1200/JCO.2016.67.5066
Altunel E, 2020, BMC CANCER, V20, DOI 10.1186/s12885-020-07090-y
Angstadt AY, 2012, CANCER GENET-NY, V205, P572, DOI 10.1016/j.cancergen.2012.09.005
Angstadt AY, 2011, GENE CHROMOSOME CANC, V50, P859, DOI 10.1002/gcc.20908
Armenian Saro, 2018, Am Soc Clin Oncol Educ Book, V38, P3, DOI 10.1200/EDBK_100015

August KJ, 2020, PEDIATR BLOOD CANCER, V67, DOI 10.1002/pbc.28062
Barretina J, 2012, NATURE, V483, P603, DOI 10.1038/nature11003
Bruun J, 2020, CLIN CANCER RES, V26, P4107, DOI 10.1158/1078-0432.CCR-19-3637
Chanukuppa V, 2019, J PROTEOMICS, V209, DOI 10.1016/j.jprot.2019.103504
Dolgin E, 2019, CANCER DISCOV, V9, P1150, DOI 10.1158/2159-8290.CD-NB2019-085
Egas-Bejar Daniela, 2014, Oncoscience, V1, P167
Ferrari S, 2012, J CLIN ONCOL, V30, P2112, DOI 10.1200/JCO.2011.38.4420
Friebele Jill C, 2015, Am J Orthop (Belle Mead NJ), V44, P547
Gao H, 2015, NAT MED, V21, P1318, DOI 10.1038/nm.3954
He HT, 2014, ONCOL LETT, V7, P1352, DOI 10.3892/ol.2014.1935
Kane RC, 2006, CLIN CANCER RES, V12, P2955, DOI 10.1158/1078-0432.CCR-06-0170
Karlsson EK, 2013, GENOME BIOL, V14, DOI 10.1186/gb-2013-14-12-r132
Kashyap T, 2016, ONCOTARGET, V7, P78883, DOI 10.18632/oncotarget.12428
Kim W, 2011, MOL CELL, V44, P325, DOI 10.1016/j.molcel.2011.08.025
Koga Y, 2019, CELLS-BASEL, V8, DOI 10.3390/cells8050418
Lindblad-Toh K, 2005, NATURE, V438, P803, DOI 10.1038/nature04338
Lu M, 2017, PLOS ONE, V12, DOI 10.1371/journal.pone.0169439
Makielski KM, 2019, VET SCI, V6, DOI 10.3390/vetsci6020048
Marina NM, 2016, LANCET ONCOL, V17, P1396, DOI 10.1016/S1470-2045(16)30214-5
Markovina S, 2010, MOL CANCER, V9, DOI 10.1186/1476-4598-9-176
Mirabello L, 2009, INT J CANCER, V125, P229, DOI 10.1002/ijc.24320
Mofers A, 2017, CANCER METAST REV, V36, P635, DOI 10.1007/s10555-017-9697-6
Nagarajan R, 2011, CANCER-AM CANCER SOC, V117, P625, DOI 10.1002/cncr.25446
Neal JT, 2018, CELL, V175, P1972, DOI 10.1016/j.cell.2018.11.021
Obeng EA, 2006, BLOOD, V107, P4907, DOI 10.1182/blood-2005-08-3531
Ooft SN, 2019, SCI TRANSL MED, V11, DOI 10.1126/scitranslmed.aay2574
Paoloni M, 2009, BMC GENOMICS, V10, DOI 10.1186/1471-2164-10-625
Pauli C, 2017, CANCER DISCOV, V7, P462, DOI 10.1158/2159-8290.CD-16-1154
Rao SR, 2020, FRONT ONCOL, V10, DOI 10.3389/fonc.2020.00117
Rodriguez CO, 2014, ADV EXP MED BIOL, V804, P237, DOI 10.1007/978-3-319-04843-7_13
Schiffman JD, 2015, PHILOS T R SOC B, V370, DOI 10.1098/rstb.2014.0231
Scognamiglio G, 2019, BRIT J CANCER, V121, P979, DOI 10.1038/s41416-019-0616-1
Somarelli JA, 2020, MOL CANCER THER, V19, P2516, DOI 10.1158/1535-7163.MCT-20-0454
Somarelli JA, 2020, MOL BIOL EVOL, V37, P11, DOI 10.1093/molbev/msz254
Stade K, 1997, CELL, V90, P1041, DOI 10.1016/S0092-8674(00)80370-0
Sun QX, 2016, SIGNAL TRANSDUCT TAR, V1, DOI 10.1038/sigtrans.2016.10
Taran SJ, 2017, INDIAN J MED PAEDIAT, V38, P33, DOI 10.4103/0971-5851.203513
Taylor J, 2019, CANCER DISCOV, V9, P1452, DOI 10.1158/2159-8290.CD-19-0298
Theodoropoulos N, 2020, TARGET ONCOL, V15, P697, DOI 10.1007/s11523-020-00758-2

   Turner JG, 2016, ONCOTARGET, V7, P78896, DOI 10.18632/oncotarget.12969
   van de Wetering M, 2015, CELL, V161, P933, DOI 10.1016/j.cell.2015.03.053
   Vlachogiannis G, 2018, SCIENCE, V359, P920, DOI 10.1126/science.aao2774
   Whelan JS, 2015, ANN ONCOL, V26, P407, DOI 10.1093/annonc/mdu526
   Wu TY, 2017, MOL CANCER THER, V16, P717, DOI 10.1158/1535-7163.MCT-16-0553
   Xu S, 2019, J CLIN MED, V8, DOI 10.3390/jcm8020205
NR 50
TC 13
Z9 13
U1 3
U2 15
PU MDPI
PI BASEL
PA ST ALBAN-ANLAGE 66, CH-4052 BASEL, SWITZERLAND
EI 2072-6694
J9 CANCERS
JI Cancers
PD NOV
PY 2020
VL 12
IS 11
AR 3335
DI 10.3390/cancers12113335
PG 14
WC Oncology
WE Science Citation Index Expanded (SCI-EXPANDED)
SC Oncology
GA OW2KN
UT WOS:000592722600001
PM 33187254
OA gold, Green Submitted, Green Published
DA 2025-05-07
ER

PT J
AU Chitadze, G
   Stengel, A
   John-Klaua, C
   Bruckmüller, J
   Trautmann, H
   Kotrova, M
   Darzentas, F
   Kelm, M
   Pal, K
   Darzentas, N
   Bastian, L
   Kehden, B
   Wessels, W
   Ströh, AS
   Oberg, HH
   Altrock, PM

Baer, C
Meggendorfer, M
Gökbuget, N
Baldus, CD
Haferlach, C
Brüggemann, M
AF Chitadze, Guranda
Stengel, Anna
John-Klaua, Cathrin
Bruckmueller, Julien
Trautmann, Heiko
Kotrova, Michaela
Darzentas, Franziska
Kelm, Miriam
Pal, Karol
Darzentas, Nikos
Bastian, Lorenz
Kehden, Britta
Wessels, Wiebke
Stroeh, Aeint-Steffen
Oberg, Hans -Heinrich
Altrock, Philipp M.
Baer, Constance
Meggendorfer, Manja
Goekbuget, Nicola
Baldus, Claudia D.
Haferlach, Claudia
Brueggemann, Monika
TI Somatic TP53 mutations are preleukemic events in acute lymphoblastic leukemia
SO BLOOD
LA English
DT Article
ID MINIMAL RESIDUAL DISEASE; DELETIONS; ADULT
C1 [Chitadze, Guranda; John-Klaua, Cathrin; Bruckmueller, Julien; Trautmann, Heiko; Kotrova, Michaela; Darzentas, Franziska; Kelm, Miriam; Pal, Karol; Darzentas, Nikos; Bastian, Lorenz; Kehden, Britta; Wessels, Wiebke; Stroeh, Aeint-Steffen; Baldus, Claudia D.; Brueggemann, Monika] Christian Albrechts Univ Kiel, Med Dept 2, Hematol & Oncol, Kiel, Germany.
[Chitadze, Guranda; John-Klaua, Cathrin; Bruckmueller, Julien; Trautmann, Heiko; Kotrova, Michaela; Darzentas, Franziska; Kelm, Miriam; Pal, Karol; Darzentas, Nikos; Bastian, Lorenz; Kehden, Britta; Wessels, Wiebke; Stroeh, Aeint-Steffen; Baldus, Claudia D.; Brueggemann, Monika] Univ Hosp Schleswig Holstein, Kiel, Germany.
[Chitadze, Guranda; Trautmann, Heiko; Kotrova, Michaela; Darzentas, Franziska; Kelm, Miriam; Pal, Karol; Darzentas, Nikos; Bastian, Lorenz; Kehden, Britta; Wessels, Wiebke; Stroeh, Aeint-Steffen; Baldus, Claudia D.; Brueggemann, Monika] Univ Hosp Schleswig Holstein, Univ Canc Ctr Schleswig Holstein, Kiel, Germany.
[Chitadze, Guranda; Trautmann, Heiko; Kotrova, Michaela; Darzentas, Franziska; Kelm, Miriam; Darzentas, Nikos; Bastian, Lorenz; Kehden, Britta; Wessels, Wiebke; Stroeh, Aeint-Steffen; Baldus, Claudia D.;

Brueggemann, Monika] Univ Hosp Schleswig Holstein, Univ Canc Ctr Schleswig Holstein, Lubeck, Germany.
[Chitadze, Guranda; Pal, Karol; Bastian, Lorenz; Baldus, Claudia D.; Brueggemann, Monika] Christian Albrechts Univ Kiel, Clin Res Unit CATCH ALL, Kiel, Germany.
[Stengel, Anna; Pal, Karol; Baer, Constance; Meggendorfer, Manja; Haferlach, Claudia] Munich Leukemia Lab, Munich, Germany.
[Bruckmueller, Julien; Pal, Karol] Solana Res GmbH, Windeby, Germany.
[Pal, Karol] Masaryk Univ, Cent European Inst Technol, Brno, Czech Republic.
[Oberg, Hans -Heinrich] Christian Albrechts Univ Kiel, Univ Hosp Schleswig Holstein, Inst Immunol, Kiel, Germany.
[Altrock, Philipp M.] Max Planck Inst Evolutionary Biol, Dept Evolutionary Theory, Plon, Germany.
[Goekbuget, Nicola] Goethe Univ Hosp, Dept Med 2, Frankfurt, Germany.
[Brueggemann, Monika] Univ Hosp Schleswig Holstein, Med Dept 2, Hematol Lab Kiel, Hematol & Oncol, Langer Segen 8-10, D-24105 Kiel, Germany.
C3 University of Kiel; University of Kiel; Schleswig Holstein University Hospital; University of Kiel; Schleswig Holstein University Hospital; University of Kiel; Schleswig Holstein University Hospital; University of Kiel; MLL Munich Leukemia Laboratory; Masaryk University Brno; University of Kiel; Schleswig Holstein University Hospital; Max Planck Society; Goethe University Frankfurt; Goethe University Frankfurt Hospital; University of Kiel; Schleswig Holstein University Hospital
RP Brüggemann, M (autor correspondente), Univ Hosp Schleswig Holstein, Med Dept 2, Hematol Lab Kiel, Hematol & Oncol, Langer Segen 8-10, D-24105 Kiel, Germany.
RI Chitadze, Guranda/ABA-4545-2020; Oberg, Hans-Heinrich/C-3994-2019; Pál,
Karol/E-3031-2012; Bastian, Lorenz/AAX-6249-2021; Bruckmuller, Julien/C-8973-2011; Altrock, Philipp/X-9063-2018
OI Bastian, Lorenz/0000-0002-1487-9437; Chitadze, Guranda/0000-0001-9609-4643; Bruckmuller, Julien/0000-0002-3900-9833; Trautmann, Heiko/0000-0002-9000-4747; Altrock, Philipp/0000-0001-7731-3345
FU Deutsche For-schungsgemeinschaft (German Research Foundation) [444949889, KFO 5010/1]; Clinician Scientist Program in Evolutionary Medicine? [413490537]
FX Acknowledgments The authors thank Henrik Knecht, Dietrich Hermann, and Martin Schwarz for their expert help. They also thank Petra Chall and Sandra Ussat for providing technical assistance.This work was supported
in part by the Deutsche For-schungsgemeinschaft (German Research Foundation) (project number 444949889) (KFO 5010/1 Clinical Research Unit ?CATCH-ALL? to G.C., L.B., C.D.B., and M.B) , and through the ?Clinician Scientist Program in Evolutionary Medicine? (project number 413490537 to G.C. and A-S.S.) .
CR Brüggemann M, 2017, BLOOD ADV, V1, P2456, DOI 10.1182/bloodadvances.2017009845
Chiaretti S, 2013, HAEMATOLOGICA, V98, pE59, DOI 10.3324/haematol.2012.076786
Comeaux EQ, 2017, CSH PERSPECT MED, V7, DOI 10.1101/cshperspect.a026286

   Hof J, 2011, J CLIN ONCOL, V29, P3185, DOI 10.1200/JCO.2011.34.8144
   Lal R, 2017, BLOOD, V129, P2587, DOI 10.1182/blood-2016-11-751008
   Mühlbacher V, 2014, GENE CHROMOSOME CANC, V53, P524, DOI
10.1002/gcc.22163
   Mullighan CG, 2008, SCIENCE, V322, P1377, DOI 10.1126/science.1164266
   Prochazka KT, 2019, HAEMATOLOGICA, V104, P516, DOI
10.3324/haematol.2018.205013
   Salmoiraghi S, 2016, HAEMATOLOGICA, V101, P245
   Salmoiraghi S, 2020, GENES-BASEL, V11, DOI 10.3390/genes11090960
   Schroeder MP, 2019, SCI REP-UK, V9, DOI 10.1038/s41598-019-40786-1
   Stengel A, 2017, LEUKEMIA, V31, P705, DOI 10.1038/leu.2016.263
   Stengel A, 2014, BLOOD, V124, P251, DOI 10.1182/blood-2014-02-558833
   van der Velden VHJ, 2007, LEUKEMIA, V21, P604, DOI
10.1038/sj.leu.2404586
   Velardi E, 2021, NAT REV IMMUNOL, V21, P277, DOI 10.1038/s41577-020-
00457-z
   Yang Q, 2010, IMMUNOL REV, V238, P12, DOI 10.1111/j.1600-
065X.2010.00956.x
NR 16
TC 7
Z9 7
U1 2
U2 9
PU ELSEVIER
PI AMSTERDAM
PA RADARWEG 29, 1043 NX AMSTERDAM, NETHERLANDS
SN 0006-4971
EI 1528-0020
J9 BLOOD
JI Blood
PD MAR 30
PY 2023
VL 141
IS 13
BP 1640
EP 1644
DI 10.1182/blood.2022017249
EA MAR 2023
PG 5
WC Hematology
WE Science Citation Index Expanded (SCI-EXPANDED)
SC Hematology
GA D4ZW9
UT WOS:000968843100001
PM 36450137
OA hybrid, Green Published
DA 2025-05-07
ER

PT J
AU Niemuth, JN
   Harms, CA
   Macdonald, JM
   Stoskopf, MK

AF Niemuth, Jennifer N.
   Harms, Craig A.
   Macdonald, Jeffrey M.
   Stoskopf, Michael K.
TI Propylene Glycol in Free-Ranging Green Sea Turtle Plasma
SO JOURNAL OF FISH AND WILDLIFE MANAGEMENT
LA English
DT Article
DE antifreeze; cold adaptation; green sea turtles; metabolomics; nuclear magnetic resonance spectroscopy; propylene glycol
ID GLYCEROL METABOLISM; CEREBROSPINAL-FLUID; SPECTROSCOPY; METABOLOMICS; PATTERNS; SERUM; BAY
AB Metabolomics is the study of metabolites, the small-molecular-weight end products of metabolism. Propylene glycol is a synthetic diol commonly used as antifreeze, as a humectant, and in the production of polyester compounds. In otherwise healthy animals, propylene glycol has generally been considered a contaminant, iatrogenic, or unexplained. We demonstrate the presence of propylene glycol in plasma of free-ranging apparently healthy green sea turtles Chelonia mydas and individuals impacted by cold stun syndrome, without iatrogenic administration or known sample processing contamination, using one- and two-dimensional proton nuclear magnetic resonance spectroscopy techniques. There was not a statistically significant difference in relative propylene glycol concentration between the two cohorts (two-sided random sampling two-sample permutation test, P = 0.842, R = 1,000). The presence of this metabolite raises important questions about sea turtle physiology and potential latent environmental contamination and serves as a starting point for future characterization of lipid metabolism and glycolysis in green sea turtles.
C1 [Niemuth, Jennifer N.; Harms, Craig A.; Macdonald, Jeffrey M.; Stoskopf, Michael K.] North Carolina State Univ, Environm Med Consortium, Raleigh, NC 27607 USA.
   [Niemuth, Jennifer N.; Harms, Craig A.; Stoskopf, Michael K.] North Carolina State Univ, Coll Vet Med, Dept Clin Sci, Raleigh, NC 27607 USA.
   [Niemuth, Jennifer N.; Harms, Craig A.; Stoskopf, Michael K.] North Carolina State Univ, Fisheries Wildlife & Conservat Biol Ctr Marine Sc, Raleigh, NC 27695 USA.
   [Macdonald, Jeffrey M.] Univ N Carolina, Dept Biomed Engn, Chapel Hill, NC 27599 USA.
C3 North Carolina State University; North Carolina State University; North
   Carolina State University; University of North Carolina; University of North Carolina Chapel Hill
RP Niemuth, JN (autor correspondente), North Carolina State Univ, Environm Med Consortium, Raleigh, NC 27607 USA.; Niemuth, JN (autor correspondente), North Carolina State Univ, Coll Vet Med, Dept Clin Sci, Raleigh, NC 27607 USA.; Niemuth, JN (autor correspondente), North Carolina State Univ, Fisheries Wildlife & Conservat Biol Ctr Marine Sc, Raleigh, NC 27695 USA.
EM jennifer_niemuth@ncsu.edu
RI Niemuth, Jennifer/AAJ-6051-2020
OI Niemuth, Jennifer/0000-0003-3412-475X
FU Bernice Barbour Foundation, Inc.; National Evolutionary Synthesis Center; Triangle Center for Evolutionary Medicine

FX We thank Dr Matthew Godfrey, Sarah Finn, and the North Carolina Wildlife
Resources Commission, and Dr Larisa Avens, Joanne Braun McNeill, and the
National Marine Fisheries Service for access to samples, as well as
Heather Broadhurst and the North Carolina State University College of
Veterinary Medicine Zoological Medicine residents for assistance in
sample collection. This research was supported by a grant from the
Bernice Barbour Foundation, Inc. and a graduate fellowship (J.N.N.) from
the National Evolutionary Synthesis Center and the Triangle Center for
Evolutionary Medicine. We thank the Associate Editor and Journal
reviewers for their feedback on this manuscript.
CR AHLQUIST DA, 1984, J COMP PHYSIOL B, V155, P75, DOI 10.1007/BF00688794
[Anonymous], SEA TURTL STRAND SAL
[Anonymous], TOX PROF PROP GLYC
[Anonymous], 1978, FED REGISTER, V43, P32800
[Anonymous], 5 INT C AN REPR ART
[Anonymous], FED REG
[Anonymous], BIOL CONSERVATION SE
Bahado-Singh RO, 2012, J MATERN-FETAL NEO M, V25, P1840, DOI 10.3109/14767058.2012.680254
Bailey SAB, 2017, ROY SOC OPEN SCI, V4, DOI 10.1098/rsos.171433
Bentivegna Flegra, 2002, Marine Turtle Newsletter, V97, P1
BURKE VJ, 1991, COPEIA, P1136
CARR A, 1980, BIOL CONSERV, V19, P7, DOI 10.1016/0006-3207(80)90011-7
Enebo L., 1954, STUDIES CELLULOSE DE
FELGER RS, 1976, SCIENCE, V191, P283, DOI 10.1126/science.191.4224.283
Innis CJ., 2017, Sea turtle health and rehabilitation, P675
KRAUSE D, 1967, J REPROD FERTIL, V14, P139, DOI 10.1530/jrf.0.0140139
KRIAT M, 1992, NMR BIOMED, V5, P179, DOI 10.1002/nbm.1940050404
KUKAL O, 1988, J COMP PHYSIOL B, V158, P175, DOI 10.1007/BF01075831
MORREALE SJ, 1992, J HERPETOL, V26, P301, DOI 10.2307/1564885
MORSHED KM, 1988, BIOCHEM MED METAB B, V39, P90, DOI 10.1016/0885-4505(88)90062-X
Nielsen NI, 2004, ANIM FEED SCI TECH, V115, P191, DOI 10.1016/j.anifeedsci.2004.03.008
Niemuth J.N., 2017, J Integr OMICS, V7, P1
O'Sullivan A, 2013, J PROTEOME RES, V12, P481, DOI 10.1021/pr3009176
Psychogios N, 2011, PLOS ONE, V6, DOI 10.1371/journal.pone.0016957
R Core Team, 2016, R: a language and environment for statistical computing
Roberts K, 2014, PHYS GEOGR, V35, P134, DOI 10.1080/02723646.2014.898573
RUDDICK JA, 1972, TOXICOL APPL PHARM, V21, P102, DOI 10.1016/0041-008X(72)90032-4
Saxena RK, 2010, INDIAN J MICROBIOL, V50, P2, DOI 10.1007/s12088-010-0017-x
Schock TB, 2013, CURR METABOL, V1, P279, DOI 10.2174/2213235X01999140101150107
SHULL KH, 1960, J BIOL CHEM, V235, P551
Still BM, 2005, CHELONIAN CONSERV BI, V4, P883
STORY JM, 1983, J COMP PHYSIOL, V149, P495, DOI 10.1007/BF00690008
Stringer KA, 2015, SHOCK, V44, P200, DOI 10.1097/SHK.0000000000000406

SUZUKI T, 1968, AGR BIOL CHEM TOKYO, V32, P888, DOI
10.1080/00021369.1968.10859143
Takeda I, 2009, NMR BIOMED, V22, P577, DOI 10.1002/nbm.1369
Wishart DS, 2008, J CHROMATOGR B, V871, P164, DOI
10.1016/j.jchromb.2008.05.001
Wishart DS, 2013, NUCLEIC ACIDS RES, V41, pD801, DOI
10.1093/nar/gks1065
NR 37
TC 4
Z9 5
U1 0
U2 7
PU U S FISH & WILDLIFE SERVICE
PI SHEPHERDSTOWN
PA NATL CONSERVATION TRAINING CENTER, CONSERVATION LIBRARY, 698
CONSERVATION WAY, SHEPHERDSTOWN, WV 25443 USA
SN 1944-687X
J9 J FISH WILDL MANAG
JI J. Fish Wildl. Manag.
PD DEC
PY 2018
VL 9
IS 2
BP 598
EP 604
DI 10.3996/122017-JFWM-099
PG 7
WC Biodiversity Conservation; Ecology
WE Science Citation Index Expanded (SCI-EXPANDED)
SC Biodiversity & Conservation; Environmental Sciences & Ecology
GA HE1PX
UT WOS:000453046200022
OA gold
DA 2025-05-07
ER

PT J
AU Kapitza, M
Tueffers, L
Schulenburg, H
Kremer, K
AF Kapitza, Martina
Tueffers, Leif
Schulenburg, Hinrich
Kremer, Kerstin
TI Tracking down resistances: a co-design approach for a biomedical
educational program on antibiotic resistance
SO SCIENCE ACTIVITIES-PROJECTS AND CURRICULUM IDEAS IN STEM CLASSROOMS
LA English
DT Article
DE Science outreach; authentic science teaching; antibiotic resistance;
cystic fibrosis; design-based research
ID EPISTEMOLOGICAL BELIEFS; EVOLUTION

AB Professional societies and institutes have stressed the importance of a dialogue between scientists and the public over the past decades. Especially the life sciences include many highly relevant research topics pertaining not only to scientific progress but also to decision-making in society. Surprisingly, few educational researchers and biological scientists make interdisciplinary efforts to communicate life science topics to society in an adequate manner. In our project, scientists from evolutionary biology, medicine and biology education worked in an interdisciplinary manner to design an educational program to make scientific research on antibiotic resistance and cystic fibrosis accessible to students (ninth, tenth and eleventh graders). In this article, we explain the communication objectives from the science education researcher's and the life scientist's perspectives. We provide a systematic description of the co-design process, a step-by-step procedure and the link to the open-source-materials used in the course. We conclude with a reflection about the implementation in consideration of the two perspectives. In sum, our results highlight that the co-design approach provides an effective method of communicating current biomedical research to school students. Our description of the co-design-process can help future applications of this co-design approach when designing science outreach activities.
C1 [Kapitza, Martina] Univ Kiel, Kiel Sci Outreach Campus KiSOC, Kiel, Germany.
[Kapitza, Martina; Kremer, Kerstin] Univ Kiel, IPN Leibniz Inst Sci & Math Educ, Kiel, Germany.
[Tueffers, Leif; Schulenburg, Hinrich] Univ Kiel, Dept Evolutionary Ecol & Genet, Kiel, Germany.
[Kremer, Kerstin] Leibniz Univ Hannover, IDN Inst Sci Educ, Hannover, Germany.
C3 University of Kiel; Leibniz Association; Leibniz Institut fur die Padagogik der Naturwissenschaften und Mathematik an der Universitat Kiel (IPN); University of Kiel; University of Kiel; Leibniz University Hannover
RP Kapitza, M (autor correspondente), IPN Leibniz Inst Sci & Math Educ, Olshausenstr 62, D-24118 Kiel, Germany.
EM kapitza@leibniz-ipn.de
RI ; Kremer, Kerstin/HJI-0511-2023
OI Tuffers, Leif/0000-0001-5496-9456; Kremer, Kerstin/0000-0003-2305-3650
FU Leibniz Science Campus "Kiel Science Outreach Campus" (KiSOC); Leibniz Science Campus "Evolutionary Medicine of the Lung" (EvoLUNG); Deutsche Forschungsgemeinschaft (DFG, German Research Foundation) under Germany's Excellence Initiative [EXC 22167-390884018]
FX Leibniz Science Campus "Kiel Science Outreach Campus" (KiSOC); Leibniz Science Campus "Evolutionary Medicine of the Lung" (EvoLUNG); Deutsche Forschungsgemeinschaft (DFG, German Research Foundation) under Germany's Excellence Initiative EXC 22167-390884018 (Excellence cluster Precision Medicine in chronic Inflammation)
CR Allchin D, 2011, SCI EDUC, V95, P518, DOI 10.1002/sce.20432
Andersson DI, 2014, NAT REV MICROBIOL, V12, P465, DOI 10.1038/nrmicro3270

Baram-Tsabari A, 2015, J RES SCI TEACH, V52, P135, DOI 10.1002/tea.21202
Barbosa C, 2018, PLOS BIOL, V16, DOI 10.1371/journal.pbio.2004356
Barbosa C, 2017, MOL BIOL EVOL, V34, P2229, DOI 10.1093/molbev/msx158
Bendixen LD, 2004, EDUC PSYCHOL-US, V39, P69, DOI 10.1207/s15326985ep3901_7
Braund M, 2006, INT J SCI EDUC, V28, P1373, DOI 10.1080/09500690500498419
Bromme R., 2014, Padagogische PsychologieKienhues, Dorothe, V6th, P55
Bromme R, 2015, J RES SCI TEACH, V52, P36, DOI 10.1002/tea.21172
Bromme R, 2014, EDUC PSYCHOL-US, V49, P59, DOI 10.1080/00461520.2014.921572
Bybee R.W., 2012, Science/ Environment/ Health: Towards a Renewed Pedagogy for Science Education, P49, DOI DOI 10.1007/978-90-481-3949-1_4
Conley AM, 2004, CONTEMP EDUC PSYCHOL, V29, P186, DOI 10.1016/j.cedpsych.2004.01.004
Fensham P.J., 2012, Science| Environment| Health, P7, DOI DOI 10.1007/978-90-481-3949-1_2
Fischhoff B, 2013, P NATL ACAD SCI USA, V110, P14033, DOI 10.1073/pnas.1213273110
Hughes JM, 2011, JAMA-J AM MED ASSOC, V305, P1027, DOI 10.1001/jama.2011.279
Imamovic L, 2018, CELL, V172, P121, DOI 10.1016/j.cell.2017.12.012
Imamovic L, 2013, SCI TRANSL MED, V5, DOI 10.1126/scitranslmed.3006609
Kampa N, 2016, CONTEMP EDUC PSYCHOL, V46, P81, DOI 10.1016/j.cedpsych.2016.04.007
Kapitza, 2020, MNU J, V73, P36
Kienhues D., 2016, DENKEN WISSEN WISSEN, P157
Kienhues D, 2012, BMC PUBLIC HEALTH, V12, DOI 10.1186/1471-2458-12-759
Kirschner PA, 2006, EDUC PSYCHOL-US, V41, P75, DOI 10.1207/s15326985ep4102_1
Kremer K., 2020, HDB FORSCHEN SCHULER, P79
Kyburz-Graber, 2012, SCI ENV HLTH RENEWED, P175
Laherto A, 2018, PROBL EDUC 21ST CENT, V76, P43
Lázár V, 2014, NAT COMMUN, V5, DOI 10.1038/ncomms5352
Lázár V, 2013, MOL SYST BIOL, V9, DOI 10.1038/msb.2013.57
Mason L, 2010, METACOGN LEARN, V5, P1, DOI 10.1007/s11409-009-9050-8
McKenney S, 2012, CONDUCTING EDUCATIONAL DESIGN RESEARCH, P1
Moschner, 2017, WISSEN LERNEN WIE EP, P99
Muis KR, 2006, EDUC PSYCHOL REV, V18, P3, DOI 10.1007/s10648-006-9003-6
O\"Neill, 2014, ANTIMICROBIAL RESIST
Organization WH, 2014, ANT RES GLOB REP SUR, DOI [10.1016/j.giec.2020.06.004, DOI 10.1007/S13312-014-0374-3]
Oz T, 2014, MOL BIOL EVOL, V31, P2387, DOI 10.1093/molbev/msu191
Phipps M, 2010, VISIT STUD, V13, P3, DOI 10.1080/10645571003618717
Podnecky NL, 2018, NAT COMMUN, V9, DOI 10.1038/s41467-018-06143-y
Roemhild R, 2019, EVOL MED PUBLIC HLTH, P37, DOI 10.1093/emph/eoz008
Roemhild R, 2015, EVOL APPL, V8, P945, DOI 10.1111/eva.12330
Rule D.C., 2010, Personal epistemology in the classroom : Theory, research, and implications for practice, P94, DOI DOI 10.1017/CBO9780511691904.004
Trevors GJ, 2017, CONTEMP EDUC PSYCHOL, V49, P107, DOI 10.1016/j.cedpsych.2017.01.005

   van Dijk EM, 2011, SCI EDUC, V95, P1086, DOI 10.1002/sce.20458
   Varner J, 2014, BIOSCIENCE, V64, P333, DOI 10.1093/biosci/biu021
   Woods-Townsend K, 2015, TRIALS, V16, DOI 10.1186/s13063-015-0890-z
   YEN P, 2017, TRIALS, V15
   Yoshida M, 2017, NAT COMMUN, V8, DOI 10.1038/ncomms15589
   Zeyer A., 2015, M HLTH INF NEEDS OUT, P235
NR 46
TC 0
Z9 0
U1 1
U2 5
PU ROUTLEDGE JOURNALS, TAYLOR & FRANCIS LTD
PI ABINGDON
PA 2-4 PARK SQUARE, MILTON PARK, ABINGDON OX14 4RN, OXON, ENGLAND
SN 0036-8121
EI 1940-1302
J9 SCI ACT
JI Sci. Act.
PD APR 3
PY 2021
VL 58
IS 2
BP 52
EP 64
DI 10.1080/00368121.2021.1906201
EA MAR 2021
PG 13
WC Education & Educational Research
WE Emerging Sources Citation Index (ESCI)
SC Education & Educational Research
GA SO3PZ
UT WOS:000646838300001
OA hybrid
DA 2025-05-07
ER

PT J
AU O'Keeffe, KR
   Jones, CD
AF O'Keeffe, Kayleigh R.
   Jones, Corbin D.
TI Challenges and solutions for analysing dual RNA-seq data for non-model
   host-pathogen systems
SO METHODS IN ECOLOGY AND EVOLUTION
LA English
DT Article
DE disease ecology; dual RNA-seq; gene expression; host-pathogen
   interactions; transcriptomics
ID READ ALIGNMENT; TRANSCRIPTOMES
AB Dual RNA-seq simultaneously profiles the transcriptomes of a host and
of a pathogen during infection and may reveal the mechanisms underlying
host-pathogen interactions. Dual RNA-seq is inherently a mixture of
transcripts from at least two species (host and pathogen), so this
mixture must be computationally sorted into host and pathogen components.

Sorting relies on aligning reads to respective reference genomes, which may be unavailable for both species in non-model host-pathogen pairs. This lack of genomic resources may present challenges to applying dual RNA-seq to non-model systems. We assessed the accuracy of alignments of dual RNA-seq when using the genomic resources of a closely related species to the species of interest by simulating datasets of mixed transcripts from a host and pathogen. Specifically, we compared how different aligners performed across different proportions of pathogen-to-host transcripts and across variation in the genetic distance between the pathogen genome and reference genome. We performed extensive analyses for a host plant with a fungal pathogen, and then, we extended the plant-fungus results by repeating key analyses in vertebrate (human)-fungus and vertebrate-bacterium systems. Aligners that were able to map pathogen transcripts to the reference genome of a species closely related to the pathogen (a 'related reference genome') also mismapped transcripts originating from the host to the pathogen's related reference genome. This resulted in regions where this occurred being quantified as overexpressed. If a host reference genome was available, we show that, to minimize host transcript mismapping and retain the ability to map pathogen transcripts, one could concatenate the host genome with the pathogen's related reference genome, then map transcripts to the concatenated genomes. If a host genome was unavailable, assembling reads de novo prior to aligning substantially decreased host read mismapping, while retaining the ability to map pathogen transcripts to a related reference genome. The application of dual RNA-seq to organisms without reference genomes is currently limited. We propose an analytical workflow that leverages the genomic resources of species closely related to species of interest to facilitate the application of dual RNA-seq to reveal the mechanisms of host-pathogen interactions across a wider array of systems.
C1 [O'Keeffe, Kayleigh R.; Jones, Corbin D.] Univ North Carolina Chapel Hill, Dept Biol, Chapel Hill, NC 27599 USA.
[Jones, Corbin D.] Univ North Carolina Chapel Hill, Integrat Program Biol & Genome Sci, Chapel Hill, NC USA.
C3 University of North Carolina; University of North Carolina Chapel Hill;
University of North Carolina School of Medicine; University of North Carolina School of Medicine; University of North Carolina; University of
North Carolina Chapel Hill
RP O'Keeffe, KR (autor correspondente), Univ North Carolina Chapel Hill, Dept Biol, Chapel Hill, NC 27599 USA.
EM kokeeffe@live.unc.edu
RI O'Keeffe, Kayleigh/AAG-8135-2019
OI O'Keeffe, Kayleigh/0000-0002-8259-6439
FU NSF-USDA joint program in Ecology and Evolution of Infectious Diseases [2016-67013-25762]; University Cancer Research Fund; Triangle Center for
Evolutionary Medicine; National Science Foundation; NIFA [914333, 2016-67013-25762] Funding Source: Federal RePORTER
FX NSF-USDA joint program in Ecology and Evolution of Infectious Diseases,
Grant/Award Number: 2016-67013-25762; University Cancer Research Fund; Triangle Center for Evolutionary Medicine; the National Science

Foundation

CR Aprianto R, 2016, GENOME BIOL, V17, DOI 10.1186/s13059-016-1054-5
Baddal B, 2015, MBIO, V6, DOI 10.1128/mBio.01765-15
Baruzzo G, 2017, NAT METHODS, V14, P135, DOI [10.1038/NMETH.4106, 10.1038/nmeth.4106]
Benjamin AM, 2014, BMC GENOMICS, V15, DOI 10.1186/1471-2164-15-570
Choi YJ, 2014, PLOS NEGLECT TROP D, V8, DOI 10.1371/journal.pntd.0002905
Dobin A, 2013, BIOINFORMATICS, V29, P15, DOI 10.1093/bioinformatics/bts635
Ekblom R, 2011, HEREDITY, V107, P1, DOI 10.1038/hdy.2010.152
Ellison AR, 2017, G3-GENES GENOM GENET, V7, P269, DOI 10.1534/g3.116.035873
Grabherr MG, 2011, NAT BIOTECHNOL, V29, P644, DOI 10.1038/nbt.1883
Griebel T, 2012, NUCLEIC ACIDS RES, V40, P10073, DOI 10.1093/nar/gks666
Haas BJ, 2013, NAT PROTOC, V8, P1494, DOI 10.1038/nprot.2013.084
Hayden KJ, 2014, TREE GENET GENOMES, V10, P489, DOI 10.1007/s11295-014-0698-0
Kawahara Y, 2012, PLOS ONE, V7, DOI 10.1371/journal.pone.0049423
Langmead B, 2012, NAT METHODS, V9, P357, DOI [10.1038/NMETH.1923, 10.1038/nmeth.1923]
Li H, 2010, BIOINFORMATICS, V26, P589, DOI 10.1093/bioinformatics/btp698
Li H, 2009, BIOINFORMATICS, V25, P1754, DOI [10.1093/bioinformatics/btp698, 10.1093/bioinformatics/btp324, 10.1093/bioinformatics/btp352]
Liao Y, 2013, NUCLEIC ACIDS RES, V41, DOI 10.1093/nar/gkt214
Love MI, 2014, GENOME BIOL, V15, DOI 10.1186/s13059-014-0550-8
Mortazavi A, 2008, NAT METHODS, V5, P621, DOI 10.1038/nmeth.1226
O'Keeffe K. R., 2018, DRYAD DIGITAL REPOSI, DOI [10. 5061/dryad. t40nj78, DOI 10.5061/DRYAD.T40NJ78]
Teixeira PJPL, 2014, PLANT CELL, V26, P4245, DOI 10.1105/tpc.114.130807
Quinlan AR, 2010, BIOINFORMATICS, V26, P841, DOI 10.1093/bioinformatics/btq033
Rhind N, 2011, SCIENCE, V332, P930, DOI 10.1126/science.1203357
Sedlazeck FJ, 2013, BIOINFORMATICS, V29, P2790, DOI 10.1093/bioinformatics/btt468
Trapnell C, 2009, BIOINFORMATICS, V25, P1105, DOI 10.1093/bioinformatics/btp120
Wang K, 2010, NUCLEIC ACIDS RES, V38, DOI 10.1093/nar/gkq622
Westermann AJ, 2016, NATURE, V529, P496, DOI 10.1038/nature16547
Westermann AJ, 2012, NAT REV MICROBIOL, V10, P618, DOI 10.1038/nrmicro2852
NR 28
TC 4
Z9 6
U1 3
U2 27
PU WILEY
PI HOBOKEN
PA 111 RIVER ST, HOBOKEN 07030-5774, NJ USA
SN 2041-210X

EI 2041-2096
J9 METHODS ECOL EVOL
JI Methods Ecol. Evol.
PD MAR
PY 2019
VL 10
IS 3
BP 401
EP 414
DI 10.1111/2041-210X.13135
PG 14
WC Ecology
WE Science Citation Index Expanded (SCI-EXPANDED)
SC Environmental Sciences & Ecology
GA HO5PI
UT WOS:000460979800011
OA Bronze, Green Submitted
DA 2025-05-07
ER

PT B
AU Lewis, S
AF Lewis, Stephen
BE Komorowska, MA
   OlsztynskaJanus, S
TI Conceptual Models of the Human Organism: Towards a New Biomedical
   Understanding of the Individual
SO BIOMEDICAL ENGINEERING, TRENDS, RESEARCH AND TECHNOLOGIES
LA English
DT Article; Book Chapter
ID DARWINIAN MEDICINE; DISEASE; HEALTH; ILLNESS
C1 [Lewis, Stephen] Univ Chester, Chester, Cheshire, England.
C3 University of Chester
RP Lewis, S (autor correspondente), Univ Chester, Chester, Cheshire, England.
CR BOORSE C, 1977, PHILOS SCI, V44, P542, DOI 10.1086/288768
   BOORSE C, 1975, PHILOS PUBLIC AFF, V5, P49
   Boorse Christopher., 1997, What is disease?, P3
   CAMPBELL EJM, 1979, BMJ-BRIT MED J, V2, P757, DOI 10.1136/bmj.2.6193.757
   Davey Basiro, 1996, EXPERIENCING EXPLAIN
   Downie RS., 1996, HLTH PROMOTION MODEL
   ENGEL GL, 1981, J MED PHILOS, V6, P101, DOI 10.1093/jmp/6.2.101
   Engel GL, 1997, PSYCHOSOMATICS, V38, P521, DOI 10.1016/S0033-3182(97)71396-3
   ENGEL GL, 1977, SCIENCE, V196, P129, DOI 10.1126/science.847460
   Grobstein Clifford, 1965, THE STRATEGY OF LIFE
   Keating Peter., 2003, Biomedical Platforms: Realigning the Normal and the Pathological in Late-Twentieth-Century Medicine
   Lewis S., 2007, ILLNESS RATED BIOL P, V34, P688
   Lewis S, 2007, PUTTING ILLNESS ITS
   Lewis S, 2009, J EVAL CLIN PRACT, V15, P745, DOI 10.1111/j.1365-2753.2009.01226.x

Malmgren Helge., 2005, Biopsychosocial medicine, P21, DOI DOI 10.1093/MED:PSYCH/9780198530343.003.0002
MARINKER M, 1975, J MED ETHICS, V1, P81, DOI 10.1136/jme.1.2.81
Maslow AH, 1943, PSYCHOL REV, V50, P370, DOI 10.1037/h0054346
Nesse R., 1999, Life Sci. Res. Life Sci. Res. Publ. China, V3, P1
Nesse R., 2001, NEW PHYS, P8
Nesse R.M., 1995, EVOLUTION HEALING NE
Nesse RM, 2006, SCIENCE, V311, P1071, DOI 10.1126/science.1125956
Nesse RM, 2001, WESTERN J MED, V174, P358, DOI 10.1136/ewjm.174.5.358
NORDENFELT L, 1986, J EPIDEMIOL COMMUN H, V40, P281, DOI 10.1136/jech.40.4.281
Nordenfelt L., 2007, Medicine, Health Care, and Philosophy, V10, P29
Nordenfelt Lennart, 2007, Med Health Care Philos, V10, P5, DOI 10.1007/s11019-006-9017-3
Reznek Lawrie., 1987, The Nature of Disease
Sadegh-Zadeh K, 2000, J MED PHILOS, V25, P605, DOI 10.1076/0360-5310(200010)25:5;1-W;FT605
SEEDHOUSE D., 2001, HLTH FDN ACHIEVEMENT, V2nd
Smith R, 2002, BRIT MED J, V324, P883, DOI 10.1136/bmj.324.7342.883
Smith R., 2008, BMJ Group Blogs
Tinetti ME, 2004, AM J MED, V116, P179, DOI 10.1016/j.amjmed.2003.09.031
White P., 2005, BIOPSYCHOSOC MED
WILLIAMS GC, 1991, Q REV BIOL, V66, P1, DOI 10.1086/417048
World Health Organization, 1948, PREAMBL CONST WORLD
NR 34
TC 10
Z9 10
U1 0
U2 1
PU INTECH EUROPE
PI RIJEKA
PA JANEZA TRDINE9, RIJEKA, 51000, CROATIA
BN 978-953-307-514-3
PY 2011
BP 3
EP 22
PG 20
WC Engineering, Biomedical
WE Book Citation Index- Science (BKCI-S)
SC Engineering
GA BE3PF
UT WOS:000371132600002
DA 2025-05-07
ER

PT J
AU Saffer, A
Tateosian, L
Saville, AC
Yang, YP
Ristaino, JB
AF Saffer, Ariel
Tateosian, Laura

Saville, Amanda C.
Yang, Yi-Peng
Ristaino, Jean B.
TI Reconstructing historic and modern potato late blight outbreaks using text analytics
SO SCIENTIFIC REPORTS
LA English
DT Article
ID PHYTOPHTHORA-INFESTANS; FAMINE PATHOGEN; ORIGIN; MITOCHONDRIAL
AB In 1843, a hitherto unknown plant pathogen entered the US and spread to potato fields in the northeast. By 1845, the pathogen had reached Ireland leading to devastating famine. Questions arose immediately about the source of the outbreaks and how the disease should be managed. The pathogen, now known as Phytophthora infestans, still continues to threaten food security globally. A wealth of untapped knowledge exists in both archival and modern documents, but is not readily available because the details are hidden in descriptive text. In this work, we (1) used text analytics of unstructured historical reports (1843-1845) to map US late blight outbreaks; (2) characterized theories on the source of the pathogen and remedies for control; and (3) created modern late blight intensity maps using Twitter feeds. The disease spread from 5 to 17 states and provinces in the US and Canada between 1843 and 1845. Crop losses, Andean sources of the pathogen, possible causes and potential treatments were discussed. Modern disease discussion on Twitter included near-global coverage and local disease observations. Topic modeling revealed general disease information, published research, and outbreak locations. The tools described will help researchers explore and map unstructured text to track and visualize pandemics.
C1 [Saffer, Ariel; Tateosian, Laura; Yang, Yi-Peng] North Carolina State Univ, Ctr Geospatial Analyt, Raleigh, NC USA.
[Saville, Amanda C.; Ristaino, Jean B.] North Carolina State Univ, Dept Entomol & Plant Pathol, Raleigh, NC 27695 USA.
[Ristaino, Jean B.] North Carolina State Univ, Emerging Plant Dis & Global Food Secur Cluster, Raleigh, NC 27695 USA.
C3 North Carolina State University; North Carolina State University; North
Carolina State University
RP Ristaino, JB (autor correspondente), North Carolina State Univ, Dept Entomol & Plant Pathol, Raleigh, NC 27695 USA.; Ristaino, JB (autor correspondente), North Carolina State Univ, Emerging Plant Dis & Global Food Secur Cluster, Raleigh, NC 27695 USA.
EM jean_ristaino@ncsu.edu
RI ; Ristaino, Jean/N-3616-2018
OI Saffer, Ariel/0000-0002-7920-6997; Tateosian, Laura/0000-0002-3578-3696;
Ristaino, Jean/0000-0002-9458-0514
FU NSF Predictive Intelligence for Pandemic Preparedness; Triangle Center for Evolutionary Medicine Seed Grant [2015-2370, 2022-1191]; USDA NIFA;
Center for Geospatial Analytics
FX Funding was provided by the Triangle Center for Evolutionary Medicine Seed Grant, USDA NIFA Grant Number 2015-2370 and NSF PIPP Grant Number 2022-1191. Appreciation to Ross Meentemeyer and the Center for Geospatial Analytics for funding a portion of AS time for the work.

Additional thanks to John Polo for Twitter data validation.
CR Abad ZG, 1997, PLANT DIS, V81, P682, DOI 10.1094/PDIS.1997.81.6.682
Anderson PK, 2004, TRENDS ECOL EVOL, V19, P535, DOI 10.1016/j.tree.2004.07.021
Andrivon D, 1996, PLANT PATHOL, V45, P1027, DOI 10.1046/j.1365-3059.1996.d01-196.x
Berkeley MJ., 1846, J Hort Soc London, V1, P9
Blei DM, 2003, J MACH LEARN RES, V3, P993, DOI 10.1162/jmlr.2003.3.4-5.993
BOURKE PMA, 1964, NATURE, V203, P805, DOI 10.1038/203805a0
Bourke PMA., 1993, The Visitation of God?: The Potato and the Great Irish Famine
Charles LE, 2018, Advanced Data Analytics in Health. Smart Innovation, P117, DOI [10.1007/978-3-319-77911-9_7, DOI 10.1007/978-3-319-77911-9_7]
Collier N, 2012, GLOB PUBLIC HEALTH, V7, P731, DOI 10.1080/17441692.2012.699975
De Bary A., 1863, Ann. Sci. Nat, V1, P143
De Bary A., 1876, J R AGR SOC ENGL, V12, P239
DIgnazio C., 2014, News KDD: Data Science for News
Dimmick C., 1874, The Potato Disease and its Prevention
Ellsworth H., 1844, House No. 151
Ellsworth H., 1845, House Document No. 140
Ellsworth H., 1843, House Document No. 177
Gomez-Alpizar L, 2007, P NATL ACAD SCI USA, V104, P3306, DOI 10.1073/pnas.0611479104
Goss EM, 2014, P NATL ACAD SCI USA, V111, P8791, DOI 10.1073/pnas.1401884111
Grünwald NJ, 2005, ANNU REV PHYTOPATHOL, V43, P171, DOI 10.1146/annurev.phyto.43.040204.135906
Hu Xuke, 2022, arXiv
Karimzadeh M, 2019, T GIS, V23, P118, DOI 10.1111/tgis.12510
Lindley J., 1845, Chronicle, V38, P648
Lindley J., 1846, Chronicle, V10, P152
Loper E., 2002, ACL, P63, DOI [DOI 10.3115/1118108.1118117, 10.3115/1225403.1225421]
Martin MD, 2016, MOL BIOL EVOL, V33, P478, DOI 10.1093/molbev/msv241
Martin MD, 2014, MOL BIOL EVOL, V31, P1414, DOI 10.1093/molbev/msu086
Martin MD, 2013, NAT COMMUN, V4, DOI 10.1038/ncomms3172
May KJ, 2004, MYCOL RES, V108, P471, DOI 10.1017/s0953756204009876
Michel JB, 2011, SCIENCE, V331, P176, DOI 10.1126/science.1199644
Miller P. R., 1955, Plant Disease Reporter, P3
Norvig Peter, 2009, Beautiful data, P219
O'Shea J, 2017, INT J MED INFORM, V101, P15, DOI 10.1016/j.ijmedinf.2017.01.019
Pasteur L., 1880, Comptes Rendus de l'Academie des Sciences, V90, P033
Pedregosa F, 2011, J MACH LEARN RES, V12, P2825
REDDICK D., 1939, CHRON BOT, V5, P410
Ristaino JB, 2001, NATURE, V411, P695, DOI 10.1038/35079606
Ristaino JB., 2009, USABlight: A disease surveillance system for late blight in the USA
Ristaino JB, 2020, EMERGING PLANT DIS G, P101, DOI DOI 10.1094/9780890546383.006
Ristaino JB, 2021, P NATL ACAD SCI USA, V118, DOI 10.1073/pnas.2022239118

Ristaino JB, 2020, FRONT ECOL EVOL, V7, DOI 10.3389/fevo.2019.00521
Saville AC, 2021, SCI REP-UK, V11, DOI 10.1038/s41598-021-90937-6
Scherm H, 2014, ANNU REV PHYTOPATHOL, V52, P453, DOI 10.1146/annurev-phyto-102313-050214
Stevens N., 1933, J. Wash. Academy Sci, V33, P435
Tateosian L., 2017, Free and Open Source Software for Geospatial, P46
Tateosian LG, 2023, COMPUT ENVIRON URBAN, V100, DOI 10.1016/j.compenvurbsys.2022.101922
Tateosian LG, 2017, MATH VIS, P129, DOI 10.1007/978-3-319-47024-5_8
Teschemacher JE, 1845, GARDENERS CHRONICLE, P125
Thomas Carla S, 2011, Emerg Health Threats J, V4, P7157, DOI 10.3402/ehtj.v4i0.7157
Vermeren I., 2015, Brandwith Blog
Weiss A., 2015, The Complete Guide to Using Google in Libraries: Instruction, Administration, and Staff Productivity, V1, P183
Woodham-Smith Cecil., 1962, GREAT HUNGER
Yoshida K, 2013, ELIFE, V2, DOI 10.7554/eLife.00731
NR 52
TC 4
Z9 4
U1 3
U2 10
PU NATURE PORTFOLIO
PI BERLIN
PA HEIDELBERGER PLATZ 3, BERLIN, 14197, GERMANY
SN 2045-2322
J9 SCI REP-UK
JI Sci Rep
PD FEB 15
PY 2024
VL 14
IS 1
AR 2523
DI 10.1038/s41598-024-52870-2
PG 13
WC Multidisciplinary Sciences
WE Science Citation Index Expanded (SCI-EXPANDED)
SC Science & Technology - Other Topics
GA IA0I9
UT WOS:001163476700069
PM 38360880
OA Green Published, gold
DA 2025-05-07
ER

PT J
AU Richards, MP
AF Richards, MP
TI A brief review of the archaeological evidence for Palaeolithic and Neolithic subsistence
SO EUROPEAN JOURNAL OF CLINICAL NUTRITION
LA English
DT Article
DE subsistence; archaeology; Palaeolithic; Neolithic; Palaeodiet; stable

isotopes
ID ISOTOPIC EVIDENCE; STABLE-ISOTOPES; DIETARY CHANGE; BONE-COLLAGEN; CARBON; BRAIN; EVOLUTION; PATTERNS; HOMINID; ORIGIN
AB Knowledge of our ancestor's diets is becoming increasingly important in evolutionary medicine, as researchers have argued that we have evolved to specific type of 'Palaeolithic' diet, and many modern nutritional disorders relate to the mismatch between the diet to which we have evolved, and the relatively newer agricultural-based 'Neolithic' diets. However, what is the archaeological evidence for pre-agricultural diets and how have they changed over the four million years of hominid evolution? This paper briefly introduces the three lines of evidence we have for Palaeolithic and Neolithic diets; morphological changes, archaeological material evidence, and direct measurement of diet from bone chemistry. The morphological changes, increasing gracilization of the mandible and increasing brain size have been interpreted (based on analogies with living primates) as the move from plants to higher-quality, more digestible, animal meat, although this is debated. The archaeological evidence is especially weak, as many organic materials, especially plants, do not survive well, and are therefore invisible in the archaeological record. Artefacts, such as stone tools which are likely to be used for hunting and animal bones with evidence of human processing and butchering do indicate that hunting did occur at many times in the past, but it is impossible to judge the frequency. Direct evidence from bone chemistry, such as the measurement of the stable isotopes of carbon and nitrogen, do provide direct evidence of past diet, and limited studies on five Neanderthals from three sites, as well as a number of modern Palaeolithic and Mesolithic humans indicates the importance of animal protein in diets. There is a significant change in the archaeological record associated with the introduction of agriculture worldwide, and an associated general decline in health in some areas. However, there is an rapid increase in population associated with domestication of plants, so although in some regions individual health suffers after the Neolithic revolution, as a species humans have greatly expanded their population worldwide.
C1 Univ Bradford, Dept Archaeol Sci, Bradford BD7 1DP, W Yorkshire, England.
C3 University of Bradford
RP Univ Bradford, Dept Archaeol Sci, Bradford BD7 1DP, W Yorkshire, England.
EM m.p.richards@bradford.ac.uk
RI Richards, Michael/O-3717-2016; Richards, Michael/A-9374-2008
OI Richards, Michael/0000-0001-5274-8887
CR AIELLO LC, 1995, CURR ANTHROPOL, V36, P199, DOI 10.1086/204350
Allison MarvinJ., 1984, Paleopathology at the Origins of Agriculture, P515
Ambrose S., 2000, Biogeochemical Approaches to Paleodietary Analysis, P243
Ambrose SH, 1998, NEANDERTALS AND MODERN HUMANS IN WESTERN ASIA, P277
Ambrose SH., 1993, Molecular Archaeology of Prehistoric Human Bone (Prehistoric Human Bone-Archaeology at the Molecular Level), P1, DOI [DOI 10.1007/978-3-662-02894-01, DOI 10.1007/978-3-662-02894-0_1]
AMBROSE SH, 1993, CHEM ANAL ANTHR, P59
[Anonymous], 1984, Paleopathology at the origins of agriculture
[Anonymous], 1992, Crops and man

[Anonymous], 1997, Journal ofEuropean Archaeology, DOI DOI 10.1179/096576697800703575
[Anonymous], 1984, Paleopathology at the Origins of Agriculture
ATKINS RC, 1999, ATKINS NEW DIET REVO
Bar-Yosef O., 1992, The transition to agriculture, P21
Bellwood P.S., 1996, ORIGINS SPREAD AGR P, P465
Blake Michael., 1992, TRANSITIONS AGR PREH, P133
Bocherens H, 1999, J ARCHAEOL SCI, V26, P599, DOI 10.1006/jasc.1998.0377
BOCHERENS H, 1991, J HUM EVOL, V20, P481, DOI 10.1016/0047-2484(91)90021-M
Boëda E, 1999, ANTIQUITY, V73, P394, DOI 10.1017/S0003598X00088335
BURGER RL, 1990, AM ANTHROPOL, V92, P85, DOI 10.1525/aa.1990.92.1.02a00060
Cai L.Z., 1984, Archaeol. (kaogu), V10, P949
Chamberlain JG, 1998, BRIT J NUTR, V80, P301
Chamberlain JG, 1996, PERSPECT BIOL MED, V39, P436
CHISHOLM B, 1996, INTERDISCIPLINARY PE, P199
Cohen M.N., 1984, Paleopathology at the Origins of Agriculture
Cook DC., 1984, PALEOPATHOLOGY ORIGI, P237
Cordain L, 1999, World Rev Nutr Diet, V84, P19
Cordain L, 2001, WORLD REV NUTR DIET, V90, P144
Cordain L, 2000, AM J CLIN NUTR, V71, P682, DOI 10.1093/ajcn/71.3.682
Crawford G.W., 1992, TRANSITIONS AGR PREH, P117
DENIRO MJ, 1981, GEOCHIM COSMOCHIM AC, V45, P341, DOI 10.1016/0016-7037(81)90244-1
DENIRO MJ, 1987, AM SCI, V75, P182
DENIRO MJ, 1985, NATURE, V317, P806, DOI 10.1038/317806a0
EADES MR, 2000, PROTEIN POWER LIFEPL
EATON SB, 1985, NEW ENGL J MED, V312, P283, DOI 10.1056/NEJM198501313120505
FIZET M, 1995, J ARCHAEOL SCI, V22, P67, DOI 10.1016/S0305-4403(95)80163-4
FLANNERY KV, 1973, ANNU REV ANTHROPOL, V2, P271, DOI 10.1146/annurev.an.02.100173.001415
Fox CL, 1996, CURR ANTHROPOL, V37, P689, DOI 10.1086/204544
Gamble C., 1999, The Palaeolithic Societies of Europe
Gebauer A.B., 1992, Transitions to Agriculture in Prehistory
Hillman G., 1996, ORIGINS SPREAD AGR P, P159
Imamura K., 1996, The Origins and Spread of Agriculture and Pastoralism in Eurasia, P442
Isaac G., 1984, Adv World Archaeol, V3, P1
Jacob-Friesen K. H., 1956, Jahrbuch des Romisch-Germanischen Zentralmuseums Mainz, V3, P1, DOI [10.11588/jrgzm.1956.0.32797, DOI 10.11588/JRGZM.1956.0.32797]
JURMAIN R, 2000, INTRO PHYSICAL ANTHR
Keeley L.H., 1980, Experimental Determinations of Stone Tool Uses: A Microwear Analisis
Kennedy KAR., 1984, PALEOPATHOLOGY ORIGI, P169
Koch PL, 1997, J ARCHAEOL SCI, V24, P417, DOI 10.1006/jasc.1996.0126
LARSEN C.S., 1984, Paleopathology at the Origins of Agriculture, P367
Larsen ClarkSpencer., 1997, BIOARCHAEOLOGY
LARSEN CS, 1992, AM J PHYS ANTHROPOL, V89, P197, DOI 10.1002/ajpa.1330890206

Leakey M.D., 1971, OLDUVAI GORGE
Leakey MG, 2001, NATURE, V410, P433, DOI 10.1038/35068500
Lee-Thorp J, 2000, J HUM EVOL, V39, P565, DOI 10.1006/jhev.2000.0436
LEETHORP JA, 1989, J ARCHAEOL SCI, V16, P585, DOI 10.1016/0305-4403(89)90024-1
LEETHORP JA, 1994, J HUM EVOL, V23, P495
LEONARD WR, 1994, AM J HUM BIOL, V6, P77, DOI 10.1002/ajhb.1310060111
Lieberman DE, 2001, NATURE, V410, P419, DOI 10.1038/35068648
LUBELL D, 1994, J ARCHAEOL SCI, V21, P201, DOI 10.1006/jasc.1994.1022
Lukacs JR, 1996, CURR ANTHROPOL, V37, P147, DOI 10.1086/204481
Lyman R. L., 1994, Vertebrate taphonomy
Mellars P., 1989, HUMAN REVOLUTION
Mellars P., 1996, NEANDERTHAL LEGACY
Miller NF, 1996, CURR ANTHROPOL, V37, P521, DOI 10.1086/204514
Milton K, 2000, AM J CLIN NUTR, V71, P665, DOI 10.1093/ajcn/71.3.665
MILTON K, 1993, SCI AM, V269, P86, DOI 10.1038/scientificamerican0893-86
MOLLESON T, 1993, J HUM EVOL, V24, P455, DOI 10.1006/jhev.1993.1031
MOLLESON T, 1994, SCI AM, V271, P70, DOI 10.1038/scientificamerican0894-70
Nestle M, 1999, P NUTR SOC, V58, P211, DOI 10.1017/S0029665199000300
Nielsen-Marsh CM, 2000, J ARCHAEOL SCI, V27, P1151, DOI 10.1006/jasc.1999.0538
Norr L., 1984, PALEOPATHOLOGY ORIGI, P463
O'Connor T.P., 2004, TEXAS A M U ANTHR SE
Patou-Mathis M, 2000, INT J OSTEOARCHAEOL, V10, P379, DOI 10.1002/1099-1212(200009/10)10:5<379::AID-OA558>3.0.CO;2-4
Pitts M.W., 1997, Fairweather Eden: Life in Britain Half a Million Years Ago as Revealed by the Excavations at Boxgrove
Price T.D., 2000, EUROPES 1 FARMERS
Richards MP, 2000, P NATL ACAD SCI USA, V97, P7663, DOI 10.1073/pnas.120178997
Richards MP, 2000, J ARCHAEOL SCI, V27, P1, DOI 10.1006/jasc.1999.0520
Richards MP, 1999, ANTIQUITY, V73, P891, DOI 10.1017/S0003598X00065649
Richards MP, 1998, ANTIQUITY, V72, P178, DOI 10.1017/S0003598X00086373
Richards MP, 2001, P NATL ACAD SCI USA, V98, P6528, DOI 10.1073/pnas.111155298
RILEY TJ, 1994, AM ANTIQUITY, V59, P490, DOI 10.2307/282461
Roberts M.B., 1986, Proceedings of the Prehistoric Society, V52, P215
Rose JeromeC., 1984, PALEOPATHOLOGY ORIGI, P393
Rose L, 1996, CURR ANTHROPOL, V37, P307, DOI 10.1086/204494
Schoeninger Margaret J., 1995, Evolutionary Anthropology, V4, P83, DOI 10.1002/evan.1360040305
SCHOENINGER MJ, 1982, NATURE, V297, P577, DOI 10.1038/297577a0
SCHWARCZ HP, 1991, YEARB PHYS ANTHROPOL, V34, P283
Semaw S, 2000, J ARCHAEOL SCI, V27, P1197, DOI 10.1006/jasc.1999.0592
Smith FH, 1999, P NATL ACAD SCI USA, V96, P12281, DOI 10.1073/pnas.96.22.12281
Sponheimer M, 1999, SCIENCE, V283, P368, DOI 10.1126/science.283.5400.368
Sponheimer M, 1999, J HUM EVOL, V36, P705, DOI 10.1006/jhev.1999.0300
Stiner M.C., 1994, Honor Among Thieves: A Zooarchaeological Study of Neandertal Ecology
STRINGER CB, 1988, SCIENCE, V239, P1263, DOI 10.1126/science.3125610

SUSMAN RL, 1994, SCIENCE, V265, P1570, DOI 10.1126/science.8079169
SUSMAN RL, 1991, J ANTHROPOL RES, V47, P129, DOI 10.1086/jar.47.2.3630322
TAUBER H, 1981, NATURE, V292, P332, DOI 10.1038/292332a0
Thomas Julian., 1996, The Origins and Spread of Agriculture and Pastoralism in Eurasia, P310
THORNE AG, 1992, SCI AM, V266, P76, DOI 10.1038/scientificamerican0492-76
Ubelaker DH., 1984, PALEOPATHOLOGY ORIGI, P491
VANDERMERWE NJ, 1981, NATURE, V292, P536, DOI 10.1038/292536a0
VANDERMERWE NJ, 1978, NATURE, V276, P815, DOI 10.1038/276815a0
WHITE CD, 1989, J ARCHAEOL SCI, V16, P451, DOI 10.1016/0305-4403(89)90068-X
WORM N, 2002, SYNDROM X MAMMUT TEL
Zvelebil M., 2000, EUROPES 1 FARMERS ED, P57, DOI DOI 10.1017/CBO9780511607851.004
NR 102
TC 85
Z9 101
U1 0
U2 63
PU NATURE PUBLISHING GROUP
PI LONDON
PA MACMILLAN BUILDING, 4 CRINAN ST, LONDON N1 9XW, ENGLAND
SN 0954-3007
EI 1476-5640
J9 EUR J CLIN NUTR
JI Eur. J. Clin. Nutr.
PD DEC
PY 2002
VL 56
IS 12
DI 10.1038/sj.ejcn.1601646
PG 9
WC Nutrition & Dietetics
WE Science Citation Index Expanded (SCI-EXPANDED)
SC Nutrition & Dietetics
GA 638LL
UT WOS:000180572400013
PM 12494313
DA 2025-05-07
ER

PT J
AU McKinnon, L
Shattuck, EC
Samson, DR
AF McKinnon, Leela
Shattuck, Eric C.
Samson, David R.
TI Sound reasons for unsound sleep: Comparative support for the sentinel hypothesis in industrial and nonindustrial groups
SO EVOLUTION MEDICINE AND PUBLIC HEALTH
LA English

DT Article
DE sleep; sentinel hypothesis; human evolution; evolutionary mismatch; health disparities
ID HEALTH; DURATION; LIGHT; RACE/ETHNICITY; CONSEQUENCES; METAANALYSIS; DISPARITIES; EVOLUTION; AMERICANS; EPIDEMIC
AB Lay Summary Sleep disruption may have been an evolved mechanism to increase alertness and safety in human ancestors that is still reflected in sleep patterns today. We found increased nighttime awakenings in nonindustrial Hadza and Malagasy compared to industrial groups, and that Hispanic participants experience more disruption than NHWs. Results suggest the need for improving sleep environment safety and addressing social sources of health disparities.

Background and objectives Sleep is a vulnerable state in which individuals are more susceptible to threat, which may have led to evolved mechanisms for increasing safety. The sentinel hypothesis proposes that brief awakenings during sleep may be a strategy for detecting and responding to environmental threats. Observations of sleep segmentation and group sentinelization in hunter-gatherer and small-scale communities support this hypothesis, but to date it has not been tested in comparisons with industrial populations characterized by more secure sleep environments. Methodology Here, we compare wake after sleep onset (WASO), a quantitative measure of nighttime awakenings, between two nonindustrial and two industrial populations: Hadza hunter-gatherers (n = 33), Malagasy small-scale agriculturalists (n = 38), and Hispanic (n = 1,531) and non-Hispanic White (NHW) (n = 347) Americans. We compared nighttime awakenings between these groups using actigraphically-measured sleep data. We fit linear models to assess whether WASO varies across groups, controlling for sex and age. Results We found that WASO varies significantly by group membership and is highest in Hadza (2.44 h) and Malagasy (1.93 h) and lowest in non-Hispanic Whites (0.69 h). Hispanics demonstrate intermediate WASO (0.86 h), which is significantly more than NHW participants. After performing supplementary analysis within the Hispanic sample, we found that WASO is significantly and positively associated with increased perception of neighborhood violence. Conclusions and implications Consistent with principles central to evolutionary medicine, we propose that evolved mechanisms to increase vigilance during sleep may now be mismatched with relatively safer environments, and in part responsible for driving poor sleep health.
C1 [McKinnon, Leela; Shattuck, Eric C.; Samson, David R.] Univ Toronto Mississauga, Dept Anthropol, Mississauga L5L 1C6, ON, Canada.

[Shattuck, Eric C.] Univ Texas San Antonio, Inst Hlth Dispar Res, San Antonio, TX 78249 USA.

[Samson, David R.] Univ Texas San Antonio, Dept Publ Hlth, San Antonio, TX 78249 USA.

[McKinnon, Leela] 9 Ursula Franklin St, Toronto, ON M5S 2S2, Canada.
C3 University of Toronto; University Toronto Mississauga; University of Texas System; University of Texas at San Antonio (UTSA); University of Texas System; University of Texas at San Antonio (UTSA)
RP McKinnon, L (autor correspondente), 9 Ursula Franklin St, Toronto, ON M5S 2S2, Canada.
EM l.mckinnon@mail.utoronto.ca
RI Samson, David/B-6879-2011
OI McKinnon, Leela/0000-0003-1557-7438

CR Alcántara C, 2017, SSM-POPUL HLTH, V3, P713, DOI 10.1016/j.ssmph.2017.08.004
Blume C, 2018, NEUROIMAGE, V178, P638, DOI 10.1016/j.neuroimage.2018.05.056
Carnethon MR, 2016, SLEEP MED, V18, P50, DOI 10.1016/j.sleep.2015.07.005
Coolidge FL., 2006, FARMING, V4, P1, DOI DOI 10.3828/BFARM.2006.4.11
Crittenden AN, 2018, SLEEP HEALTH, V4, P527, DOI 10.1016/j.sleh.2018.10.005
Czeisler C, 2013, NATURE, V497, pS13, DOI 10.1038/497S13a
de la Iglesia HO, 2015, J BIOL RHYTHM, V30, P342, DOI 10.1177/0748730415590702
Durmer JS, 2005, SEMIN NEUROL, V25, P117, DOI 10.1055/s-0029-1237117
Faraut B, 2012, SLEEP MED REV, V16, P137, DOI 10.1016/j.smrv.2011.05.001
Feagin J, 2014, SOC SCI MED, V103, P7, DOI 10.1016/j.socscimed.2013.09.006
Grandner MA, 2013, J CLIN SLEEP MED, V9, P897, DOI 10.5664/jcsm.2990
HCHS/SOL Coordinating Center, 2015, COD SUEN ANC STUD SL
Hill TD, 2016, SLEEP MED, V18, P56, DOI 10.1016/j.sleep.2014.12.003
Hwang JY, 2018, J CLIN MED, V7, DOI 10.3390/jcm7090268
Irwin MR, 2016, BIOL PSYCHIAT, V80, P40, DOI 10.1016/j.biopsych.2015.05.014
Koops K, 2012, AM J PHYS ANTHROPOL, V148, P351, DOI 10.1002/ajpa.22056
LENDREM DW, 1984, ANIM BEHAV, V32, P243, DOI 10.1016/S0003-3472(84)80343-7
Li NP, 2018, CURR DIR PSYCHOL SCI, V27, P38, DOI 10.1177/0963721417731378
Lima SL, 2005, ANIM BEHAV, V70, P723, DOI 10.1016/j.anbehav.2005.01.008
MacDonald K, 2021, P NATL ACAD SCI USA, V118, DOI 10.1073/pnas.2101108118
Marino M, 2013, SLEEP, V36, P1747, DOI 10.5665/sleep.3142
Marlowe FW, 2005, EVOL ANTHROPOL, V14, P54, DOI 10.1002/evan.20046
McKinnon II, 2022, SOC SCI MED, V310, DOI 10.1016/j.socscimed.2022.115269
McKinnon L., 2022, PLOS ONE, V17, pe0277416
Mezick EJ, 2008, PSYCHOSOM MED, V70, P410, DOI 10.1097/PSY.0b013e31816fdf21
Mong JA, 2016, PHILOS T R SOC B, V371, DOI 10.1098/rstb.2015.0110
Moreno CRC, 2015, SCI REP-UK, V5, DOI 10.1038/srep14074
Nesse RM, 2005, EVOL HUM BEHAV, V26, P88, DOI 10.1016/j.evolhumbehav.2004.08.002
Nunes J, 2008, J NATL MED ASSOC, V100, P317, DOI 10.1016/S0027-9684(15)31244-X
Nunn CL, 2018, AM J PHYS ANTHROPOL, V166, P601, DOI 10.1002/ajpa.23427
Nunn CL, 2016, EVOL MED PUBLIC HLTH, P227, DOI 10.1093/emph/eow018
Ohayon MM, 2004, SLEEP, V27, P1255, DOI 10.1093/sleep/27.7.1255
Patel SR, 2015, SLEEP, V38, P1497, DOI 10.5665/sleep.4998
Perogamvros L, 2020, SLEEP MED REV, V51, DOI 10.1016/j.smrv.2020.101277
R Core Team, 2020, R: a language and environment for statistical computing. R Foundation for Statistical Computing v.4.3.0
Radler BT, 2014, Open Health Data, V2, P3, DOI DOI 10.5334/OHD.AI

Roenneberg T, 2013, NATURE, V498, P427, DOI 10.1038/498427a
Ryff C., 2018, SLEEP DATA DOCUMENTA
Ryff C. D., 2021, Midlife in the United States (MIDUS 2), 2004-2006
Samson DR, 2021, AM J HUM BIOL, V33, DOI 10.1002/ajhb.23541
Samson DR, 2017, J HUM EVOL, V113, P91, DOI 10.1016/j.jhevol.2017.08.005
Samson DR, 2017, AM J HUM BIOL, V29, DOI 10.1002/ajhb.22979
Samson DR, 2017, P ROY SOC B-BIOL SCI, V284, DOI 10.1098/rspb.2017.0967
Samson DR, 2017, AM J PHYS ANTHROPOL, V162, P573, DOI 10.1002/ajpa.23160
Samson DR, 2015, EVOL ANTHROPOL, V24, P225, DOI 10.1002/evan.21464
Santos-Silva R, 2010, SLEEP MED, V11, P520, DOI 10.1016/j.sleep.2009.12.011
Shattuck EC, 2022, AM J HUM BIOL, V34, DOI 10.1002/ajhb.23574
Simonelli G, 2017, SLEEP, V40, DOI 10.1093/sleep/zsw025
Simonelli G, 2015, SLEEP HEALTH, V1, P98, DOI 10.1016/j.sleh.2015.02.006
Smit AN, 2019, SCI REP-UK, V9, DOI 10.1038/s41598-019-53635-y
SNYDER F, 1966, AM J PSYCHIAT, V123, P121, DOI 10.1176/ajp.123.2.121
Sorlie PD, 2010, ANN EPIDEMIOL, V20, P629, DOI 10.1016/j.annepidem.2010.03.015
Stranges S, 2012, SLEEP, V35, P1173, DOI 10.5665/sleep.2012
Trevathan WR, 2007, ANNU REV ANTHROPOL, V36, P139, DOI 10.1146/annurev.anthro.36.081406.094321
Van Cauter E, 2008, EUR J ENDOCRINOL, V159, pS59, DOI 10.1530/EJE-08-0298
Voss U, 2004, REV NEUROSCIENCE, V15, P33
Weinstein M., 2019, MIDLIFE US MIDUS REF
Wiessner PW, 2014, P NATL ACAD SCI USA, V111, P14027, DOI 10.1073/pnas.1404212111
Williams NJ, 2015, SLEEP HEALTH, V1, P28, DOI 10.1016/j.sleh.2014.12.004
Williams NJ, 2016, SLEEP MED, V18, P103, DOI 10.1016/j.sleep.2015.02.533
Xie LL, 2013, SCIENCE, V342, P373, DOI 10.1126/science.1241224
Yamagiwa J, 2001, J HUM EVOL, V40, P99, DOI 10.1006/jhev.2000.0444
NR 62
TC 3
Z9 3
U1 0
U2 6
PU OXFORD UNIV PRESS
PI OXFORD
PA GREAT CLARENDON ST, OXFORD OX2 6DP, ENGLAND
EI 2050-6201
J9 EVOL MED PUBLIC HLTH
JI Evol. Med. Public Health
PD JAN 1
PY 2023
VL 11
IS 1
BP 53
EP 66

DI 10.1093/emph/eoac039
EA MAR 2023
PG 14
WC Evolutionary Biology; Public, Environmental & Occupational Health
WE Science Citation Index Expanded (SCI-EXPANDED)
SC Evolutionary Biology; Public, Environmental & Occupational Health
GA A1QW2
UT WOS:000952956100002
PM 36945298
OA Green Published, gold
DA 2025-05-07
ER

PT J
AU Botelho, J
Tüffers, L
Fuss, J
Buchholz, F
Utpatel, C
Klockgether, J
Niemann, S
Tümmler, B
Schulenburg, H
AF Botelho, Joao
Tueffers, Leif
Fuss, Janina
Buchholz, Florian
Utpatel, Christian
Klockgether, Jens
Niemann, Stefan
Tuemmler, Burkhard
Schulenburg, Hinrich
TI Phylogroup-specific variation shapes the clustering of antimicrobial resistance genes and defence systems across regions of genome plasticity in Pseudomonas aeruginosa
SO EBIOMEDICINE
LA English
DT Article
DE Pangenome; Pseudomonas aeruginosa; Regions of genome plasticity; Antibiotic resistance; CRISPR-Cas systems; Defence systems
ID BACTERIAL; PANGENOME; EVOLUTION; SEQUENCE; ELEMENTS; ISLANDS; ECOLOGY; CRISPR; CORE
AB Background Pseudomonas aeruginosa is an opportunistic pathogen consisting of three phylogroups (hereafter named A, B, and C). Here, we assessed phylogroup-specific evolutionary dynamics across available and also new P. aeruginosa genomes.Methods In this genomic analysis, we first generated new genome assemblies for 18 strains of the major P. aeruginosa clone type (mPact) panel, comprising a phylogenetically diverse collection of clinical and environmental isolates for this species. Thereafter, we combined these new genomes with 1991 publicly available P. aeruginosa genomes for a phylogenomic and comparative analysis. We specifically explored to what extent antimicrobial resistance (AMR) genes, defence systems, and virulence genes vary in their distribution

across regions of genome plasticity (RGPs) and "masked" (RGP-free) genomes, and to what extent this variation differs among the phylogroups.Findings We found that members of phylogroup B possess larger genomes, contribute a comparatively larger number of pangenome families, and show lower abundance of CRISPR-Cas systems. Furthermore, AMR and defence systems are pervasive in RGPs and integrative and conjugative/mobilizable elements (ICEs/IMEs) from phylogroups A and B, and the abundance of these cargo genes is often significantly correlated. Moreover, inter -and intra-phylogroup interactions occur at the accessory genome level, suggesting frequent recombination events. Finally, we provide here the mPact panel of diverse P. aeruginosa strains that may serve as a valuable reference for functional analyses.Interpretation Altogether, our results highlight distinct pangenome characteristics of the P. aeruginosa phylogroups, which are possibly influenced by variation in the abundance of CRISPR-Cas systems and are shaped by the differential distribution of other defence systems and AMR genes.Funding German Science Foundation, Max-Planck Society, Leibniz ScienceCampus Evolutionary Medicine of the Lung, BMBF program Medical Infection Genomics, Kiel Life Science Postdoc Award.Copyright (c) 2023 The Author(s). Published by Elsevier B.V. This is an open access article under the CC BY-NC-ND license (http://creativecommons.org/licenses/by-nc-nd/4.0/).2023;90: Published 2023 https://doi.org/10. 1016/j.ebiom.2023. 104532
C1 [Botelho, Joao; Schulenburg, Hinrich] Max Planck Inst Evolutionary Biol, Antibiot Resistance Grp, Plon, Germany.
[Botelho, Joao; Tueffers, Leif; Buchholz, Florian; Schulenburg, Hinrich] Univ Kiel, Evolutionary Ecol & Genet, Kiel, Germany.
[Tueffers, Leif] Univ Lubeck, Dept Infect Dis & Microbiol, Lubeck, Germany.
[Fuss, Janina] Univ Kiel, Inst Clin Mol Biol, Kiel, Germany.
[Fuss, Janina] Univ Hosp Schleswig Holstein, Kiel, Germany.
[Utpatel, Christian; Niemann, Stefan] Res Ctr Borstel, Mol & Expt Mycobacteriol, Borstel, Germany.
[Utpatel, Christian; Niemann, Stefan] German Ctr Infect Res, Partner Site Hamburg Lubeck Borstel Riems, Borstel, Germany.
[Klockgether, Jens; Tuemmler, Burkhard] Hannover Med Sch MHH, Clin Paediat Pneumol Allergol & Neonatol, Hannover, Germany.
[Tuemmler, Burkhard] Hannover Med Sch, German Ctr Lung Res, Biomed Res Endstage & Obstruct Lung Dis Hannover, Hannover, Germany.
C3 Max Planck Society; University of Kiel; University of Lubeck; University
of Kiel; University of Kiel; Schleswig Holstein University Hospital; Leibniz Association; Forschungszentrum Borstel; German Center for Infection Research; Hannover Medical School; Hannover Medical School
RP Botelho, J; Schulenburg, H (autor correspondente), Max Planck Inst Evolutionary Biol, Antibiot Resistance Grp, Plon, Germany.
EM botelho@evolbio.mpg.de; hschulenburg@zoologie.uni-kiel.de
RI ; Botelho, Joao/T-9066-2017
OI Buchholz, Florian/0000-0003-3882-9132; Botelho, Joao/0000-0002-2771-2345
FU Max-Planck Society; German Science Foundation; German ministry for education and research; Kiel university
FX The funders (Max-Planck Society, German Science Foundation, German

ministry for education and research, Kiel university) had no role in the study design, data collection, data analysis, data interpretation, or writing of the report. The corresponding authors had full access to all the data and take final responsibility for the decision to submit for publication. None of the contributing authors was denied access to the data of this study.

CR Acman M, 2020, NAT COMMUN, V11, DOI 10.1038/s41467-020-16282-w
Arnold BJ, 2022, NAT REV MICROBIOL, V20, P206, DOI 10.1038/s41579-021-00650-4
Arora SK, 2001, P NATL ACAD SCI USA, V98, P9342, DOI 10.1073/pnas.161249198
Bazin A, 2020, BIOINFORMATICS, V36, pI651, DOI 10.1093/bioinformatics/btaa792
Blanchet FG, 2020, ECOL LETT, V23, P1050, DOI 10.1111/ele.13525
Botelho J, 2023, NUCLEIC ACIDS RES, V51, P236, DOI 10.1093/nar/gkac1220
Botelho J, 2020, MICROB GENOMICS, V6, DOI 10.1099/mgen.0.000424
Botelho J, 2020, GENES-BASEL, V11, DOI 10.3390/genes11080889
Botelho J, 2021, TRENDS MICROBIOL, V29, P8, DOI 10.1016/j.tim.2020.05.011
Botelho J, 2019, DRUG RESIST UPDATE, V44, P26, DOI 10.1016/j.drup.2019.07.002
Brockhurst MA, 2019, CURR BIOL, V29, pR1094, DOI 10.1016/j.cub.2019.08.012
Cantalapiedra CP, 2021, MOL BIOL EVOL, V38, P5825, DOI 10.1093/molbev/msab293
Cohen D, 2019, NATURE, V574, P691, DOI 10.1038/s41586-019-1605-5
Collins RE, 2012, MOL BIOL EVOL, V29, P3413, DOI 10.1093/molbev/mss163
Cury J, 2018, MOL BIOL EVOL, V35, P2230, DOI 10.1093/molbev/msy123
Cury J, 2017, NUCLEIC ACIDS RES, V45, P8943, DOI 10.1093/nar/gkx607
De Oliveira DMP, 2020, CLIN MICROBIOL REV, V33, DOI 10.1128/CMR.00181-19
Didelot X, 2015, PLOS COMPUT BIOL, V11, DOI 10.1371/journal.pcbi.1004041
Doron S, 2018, SCIENCE, V359, DOI 10.1126/science.aar4120
Feldgarden M, 2019, ANTIMICROB AGENTS CH, V63, DOI 10.1128/AAC.00483-19
Filloux A, 2011, FRONT MICROBIOL, V2, DOI 10.3389/fmicb.2011.00155
Fischer S, 2020, MSYSTEMS, V5, DOI 10.1128/mSystems.01007-20
Freschi L, 2019, GENOME BIOL EVOL, V11, P109, DOI 10.1093/gbe/evy259
Gautreau G, 2020, PLOS COMPUT BIOL, V16, DOI 10.1371/journal.pcbi.1007732
Ghaly TM, 2020, TRENDS MICROBIOL, V28, P455, DOI 10.1016/j.tim.2019.12.002
Goldfarb T, 2015, EMBO J, V34, P169, DOI 10.15252/embj.201489455
Guglielmini J, 2011, PLOS GENET, V7, DOI 10.1371/journal.pgen.1002222
Hilker R, 2015, ENVIRON MICROBIOL, V17, P29, DOI 10.1111/1462-2920.12606
Horcajada JP, 2019, CLIN MICROBIOL REV, V32, DOI 10.1128/CMR.00031-19
Horesh G, 2021, MICROB GENOMICS, V7, DOI 10.1099/mgen.0.000670
Horna G, 2019, SCI REP-UK, V9, DOI 10.1038/s41598-019-47303-4
Hussain FA, 2021, SCIENCE, V374, P488, DOI 10.1126/science.abb1083

Jain C, 2018, NAT COMMUN, V9, DOI 10.1038/s41467-018-07641-9
Koonin EV, 2020, NAT REV GENET, V21, P119, DOI 10.1038/s41576-019-0172-9
Koonin EV, 2008, NUCLEIC ACIDS RES, V36, P6688, DOI 10.1093/nar/gkn668
Labrie SJ, 2010, NAT REV MICROBIOL, V8, P317, DOI 10.1038/nrmicro2315
Lassalle F, 2015, RES MICROBIOL, V166, P729, DOI 10.1016/j.resmic.2015.06.008
LeGault KN, 2021, SCIENCE, V373, P534, DOI 10.1126/science.abg2166
León LM, 2021, NUCLEIC ACIDS RES, V49, P2114, DOI 10.1093/nar/gkab006
Letunic I, 2021, NUCLEIC ACIDS RES, V49, pW293, DOI 10.1093/nar/gkab301
Liu B, 2019, NUCLEIC ACIDS RES, V47, pD687, DOI 10.1093/nar/gky1080
Liu M, 2019, NUCLEIC ACIDS RES, V47, pD660, DOI 10.1093/nar/gky1123
Makarova KS, 2011, J BACTERIOL, V193, P6039, DOI 10.1128/JB.05535-11
Matilla MA, 2021, MICROBIOL MOL BIOL R, V85, DOI 10.1128/MMBR.00151-20
Minh BQ, 2020, MOL BIOL EVOL, V37, P1530, DOI 10.1093/molbev/msaa015
Morimoto Y, 2020, INT J SYST EVOL MICR, V70, P5958, DOI 10.1099/ijsem.0.004468
Moya-Beltrán A, 2021, CRISPR J, V4, P656, DOI 10.1089/crispr.2021.0051
Murray CJL, 2022, LANCET, V399, P629, DOI 10.1016/S0140-6736(21)02724-0
Ofir G, 2018, NAT MICROBIOL, V3, DOI 10.1038/s41564-017-0051-0
Ozer EA, 2019, GENOME BIOL EVOL, V11, P1780, DOI 10.1093/gbe/evz119
Partridge SR, 2018, CLIN MICROBIOL REV, V31, DOI 10.1128/CMR.00088-17
Perrin A, 2021, NAR GENOM BIOINFORM, V3, DOI DOI 10.1093/nargab/lqaa106
Pinilla-Redondo R, 2020, NUCLEIC ACIDS RES, V48, P2000, DOI [DOI 10.1093/nar/gkz1197, 10.1093/nar/gkz1197]
Pinilla-Redondo R, 2022, NUCLEIC ACIDS RES, V50, P4315, DOI 10.1093/nar/gkab859
Pursey E, 2022, PHILOS T R SOC B, V377, DOI 10.1098/rstb.2020.0464
Quinlan AR, 2010, BIOINFORMATICS, V26, P841, DOI 10.1093/bioinformatics/btq033
Reboud E, 2017, TOXINS, V9, DOI 10.3390/toxins9110364
Rocha EPC, 2022, PLOS BIOL, V20, DOI 10.1371/journal.pbio.3001514
Rodrigues YC, 2020, PLOS ONE, V15, DOI 10.1371/journal.pone.0238741
Roy PH, 2010, PLOS ONE, V5, DOI 10.1371/journal.pone.0008842
Russel J, 2020, CRISPR J, V3, P462, DOI 10.1089/crispr.2020.0059
Saati-Santamaría Z, 2022, MICROBIOL SPECTR, V10, DOI 10.1128/spectrum.02370-22
Seemann T, 2014, BIOINFORMATICS, V30, P2068, DOI 10.1093/bioinformatics/btu153
Steinegger M, 2017, NAT BIOTECHNOL, V35, P1026, DOI 10.1038/nbt.3988
Stover CK, 2000, NATURE, V406, P959, DOI 10.1038/35023079
Tacconelli E, 2018, LANCET INFECT DIS, V18, P318, DOI [10.1016/s1473-3099(17)30753-3, 10.1016/S1473-3099(17)30753-3]
Tatusov RL, 2000, NUCLEIC ACIDS RES, V28, P33, DOI 10.1093/nar/28.1.33
Tesson F, 2022, NAT COMMUN, V13, DOI 10.1038/s41467-022-30269-9
Trouillon J, 2022, NAT COMMUN, V13, DOI 10.1038/s41467-022-28849-w
Trouillon J, 2021, NUCLEIC ACIDS RES, V49, P11476, DOI 10.1093/nar/gkab928
van Belkum A, 2015, MBIO, V6, DOI 10.1128/mBio.01796-15
van Vliet AHM, 2021, MICROB GENOMICS, V7, DOI 10.1099/mgen.0.000729
Wheatley RM, 2021, ISME J, V15, P1420, DOI 10.1038/s41396-020-00860-3

Whelan FJ, 2021, MOL BIOL EVOL, V38, P3697, DOI 10.1093/molbev/msab139
Wick RR, 2017, PLOS COMPUT BIOL, V13, DOI 10.1371/journal.pcbi.1005595
Wick Ryan R, 2015, Bioinformatics, V31, P3350, DOI 10.1093/bioinformatics/btv383
Wiehlmann L, 2007, P NATL ACAD SCI USA, V104, P8101, DOI 10.1073/pnas.0609213104
Wiehlmann L, 2015, ENV MICROBIOL REP, V7, P955, DOI 10.1111/1758-2229.12340
Zhao XF, 2019, BIOINFORMATICS, V35, P671, DOI 10.1093/bioinformatics/bty651
NR 79
TC 19
Z9 19
U1 0
U2 6
PU ELSEVIER
PI AMSTERDAM
PA RADARWEG 29, 1043 NX AMSTERDAM, NETHERLANDS
SN 2352-3964
J9 EBIOMEDICINE
JI EBioMedicine
PD APR
PY 2023
VL 90
AR 104532
DI 10.1016/j.ebiom.2023.104532
EA MAR 2023
PG 15
WC Medicine, General & Internal; Medicine, Research & Experimental
WE Science Citation Index Expanded (SCI-EXPANDED)
SC General & Internal Medicine; Research & Experimental Medicine
GA H2XT1
UT WOS:000994654600001
PM 36958270
OA Green Published
DA 2025-05-07
ER

PT J
AU Dornburg, A
Wcisel, DJ
Zapfe, K
Ferraro, E
Roupe-Abrams, L
Thompson, AW
Braasch, I
Ota, T
Yoder, JA
AF Dornburg, Alex
Wcisel, Dustin J.
Zapfe, Katerina
Ferraro, Emma
Roupe-Abrams, Lindsay
Thompson, Andrew W.

Braasch, Ingo
Ota, Tatsuya
Yoder, Jeffrey A.
TI Holosteans contextualize the role of the teleost genome duplication in promoting the rise of evolutionary novelties in the ray-finned fish innate immune system
SO IMMUNOGENETICS
LA English
DT Article
DE Bowfin and gar; Multigene families; Diverse immunoglobulin domain-containing proteins (DICPs); Novel immune-type receptors (NITRs);
V(D)J recombination; MHC
ID ADAPTIVE RADIATION; GENE-CLUSTER; SPOTTED GAR; RECEPTOR; MODEL; DIVERSIFICATION; PHYLOGENY; CONSERVATION; GENERATION; RESOLUTION
AB Over 99% of ray-finned fishes (Actinopterygii) are teleosts, a clade that comprises half of all living vertebrate species that have diversified across virtually all fresh and saltwater ecosystems. This ecological breadth raises the question of how the immunogenetic diversity required to persist under heterogeneous pathogen pressures evolved. The teleost genome duplication (TGD) has been hypothesized as the evolutionary event that provided the substrate for rapid genomic evolution and innovation. However, studies of putative teleost-specific innate immune receptors have been largely limited to comparisons either among teleosts or between teleosts and distantly related vertebrate clades such as tetrapods. Here we describe and characterize the receptor diversity of two clustered innate immune gene families in the teleost sister lineage: Holostei (bowfin and gars). Using genomic and transcriptomic data, we provide a detailed investigation of the phylogenetic history and conserved synteny of gene clusters encoding diverse immunoglobulin domain-containing proteins (DICPs) and novel immune-type receptors (NITRs). These data demonstrate an ancient linkage of DICPs to the major histocompatibility complex (MHC) and reveal an evolutionary origin of NITR variable-joining (VJ) exons that predate the TGD by at least 50 million years. Further characterizing the receptor diversity of Holostean DICPs and NITRs illuminates a sequence diversity that rivals the diversity of these innate immune receptor families in many teleosts. Taken together, our findings provide important historical context for the evolution of these gene families that challenge prevailing expectations concerning the consequences of the TGD during actinopterygiian evolution.
C1 [Dornburg, Alex; Zapfe, Katerina] Univ N Carolina, Dept Bioinformat & Genom, Charlotte, NC 28223 USA.
[Wcisel, Dustin J.; Yoder, Jeffrey A.] North Carolina State Univ, Dept Mol Biomed Sci, Raleigh, NC 27695 USA.
[Ferraro, Emma; Roupe-Abrams, Lindsay] North Carolina Museum Nat Sci, Raleigh, NC USA.
[Thompson, Andrew W.; Braasch, Ingo] Michigan State Univ, Dept Integrat Biol, E Lansing, MI USA.
[Thompson, Andrew W.] Michigan State Univ, Ecol Evolut Sr Behav Program, E Lansing, MI 48824 USA.
[Ota, Tatsuya] Grad Univ Adv Studies, Dept Evolutionary Studies Biosyst, SOKENDAI, Hayama, Kanagawa, Japan.

[Yoder, Jeffrey A.] North Carolina State Univ, Comparat Med Inst, Raleigh, NC 27695 USA.
[Yoder, Jeffrey A.] North Carolina State Univ, Ctr Human Hlth & Environm, Raleigh, NC 27695 USA.
C3 University of North Carolina; University of North Carolina Charlotte; North Carolina State University; Michigan State University; Michigan State University; Graduate University for Advanced Studies - Japan; North Carolina State University; North Carolina State University
RP Dornburg, A (autor correspondente), Univ N Carolina, Dept Bioinformat & Genom, Charlotte, NC 28223 USA.; Yoder, JA (autor correspondente), North Carolina State Univ, Dept Mol Biomed Sci, Raleigh, NC 27695 USA.; Yoder, JA (autor correspondente), North Carolina State Univ, Comparat Med Inst, Raleigh, NC 27695 USA.; Yoder, JA (autor correspondente), North Carolina State Univ, Ctr Human Hlth & Environm, Raleigh, NC 27695 USA.
EM adornbur@uncc.edu; jeff_yoder@ncsu.edu
RI Braasch, Ingo/A-6948-2010; Dornburg, Alex/CAF-1206-2022; Yoder, Jeffrey/I-5329-2019; Ferraro, Elisabetta/AAS-4975-2020; Thompson, Andrew/W-1443-2017
OI Yoder, Jeff/0000-0002-6083-1311; Ota, Tatsuya/0000-0002-1115-5169; Wcisel, Dustin/0000-0001-6858-643X; Thompson, Andrew/0000-0002-1198-6130; Braasch, Ingo/0000-0003-4766-611X
FU National Science Foundation [IOS-1755242, IOS-1755330]; Triangle Center for Evolutionary Medicine (TriCEM); National Institutes of Health [R01OD011116]
FX This research was supported, in part, by grants from the National Science Foundation (IOS-1755242 to AD and IOS-1755330 to JAY), a grant from the Triangle Center for Evolutionary Medicine (TriCEM) to AD and JAY, and funding from the National Institutes of Health (R01OD011116 to IB).
CR Alfaro ME, 2009, P NATL ACAD SCI USA, V106, P13410, DOI 10.1073/pnas.0811087106
Aoki T, 2013, FISH SHELLFISH IMMUN, V35, P1689, DOI 10.1016/j.fsi.2013.02.005
Armenteros JJA, 2019, NAT BIOTECHNOL, V37, P420, DOI 10.1038/s41587-019-0036-z
Berv JS, 2018, SYST BIOL, V67, P1, DOI 10.1093/sysbio/syx064
Betancur-R R, 2017, BMC EVOL BIOL, V17, DOI 10.1186/s12862-017-0958-3
Boudinot P, 2014, J EXP ZOOL PART B, V322, P415, DOI 10.1002/jez.b.22559
Braasch I., 2012, Polyploidy and genome evolution, P341, DOI [10.1007/978-3-642-31442-117, DOI 10.1007/978-3-642-31442-117]
Braasch I, 2016, NAT GENET, V48, P427, DOI 10.1038/ng.3526
Brawand D, 2014, NATURE, V513, P375, DOI 10.1038/nature13726
Minh BQ, 2013, MOL BIOL EVOL, V30, P1188, DOI 10.1093/molbev/mst024
Burns MD, 2019, EVOLUTION, V73, P569, DOI 10.1111/evo.13658
Cannon JP, 2008, IMMUNITY, V29, P228, DOI 10.1016/j.immuni.2008.05.018
Carmona SJ, 2017, GENOME RES, V27, P451, DOI 10.1101/gr.207704.116
Conant GC, 2020, PLOS ONE, V15, DOI 10.1371/journal.pone.0231356
Daane JM, 2019, NAT ECOL EVOL, V3, P1102, DOI 10.1038/s41559-019-0914-2
Davis AM, 2012, J EVOLUTION BIOL, V25, P1163, DOI 10.1111/j.1420-9101.2012.02504.x

Davis MP, 2014, MAR BIOL, V161, P1139, DOI 10.1007/s00227-014-2406-x
Desai S, 2008, BMC EVOL BIOL, V8, DOI 10.1186/1471-2148-8-177
Hoang DT, 2018, MOL BIOL EVOL, V35, P518, DOI 10.1093/molbev/msx281
Dirscherl H, 2014, DEV COMP IMMUNOL, V46, P11, DOI 10.1016/j.dci.2014.02.018
Dornburg A, 2017, NAT ECOL EVOL, V1, P1379, DOI 10.1038/s41559-017-0239-y
Dornburg A, 2014, BMC EVOL BIOL, V14, DOI 10.1186/s12862-014-0169-0
Dornburg A, 2011, EVOLUTION, V65, P1912, DOI 10.1111/j.1558-5646.2011.01275.x
Fabbri M, 2017, NAT ECOL EVOL, V1, P1543, DOI 10.1038/s41559-017-0288-2
Fernández R, 2020, NAT ECOL EVOL, V4, P524, DOI 10.1038/s41559-019-1069-x
Ferraresso S, 2009, IMMUNOGENETICS, V61, P773, DOI 10.1007/s00251-009-0398-3
Friedman M, 2013, P ROY SOC B-BIOL SCI, V280, DOI 10.1098/rspb.2013.1733
Friedman ST, 2019, EVOLUTION, V73, P1873, DOI 10.1111/evo.13755
Gajdzik L, 2019, ECOL LETT, V22, P572, DOI 10.1111/ele.13219
Gao FX, 2018, DEV COMP IMMUNOL, V84, P396, DOI 10.1016/j.dci.2018.03.013
Glasauer SMK, 2014, MOL GENET GENOMICS, V289, P1045, DOI 10.1007/s00438-014-0889-2
Grande L, 2010, COPEIA, P1
Haire RN, 2012, GENOMICS, V99, P282, DOI 10.1016/j.ygeno.2012.02.004
Höhne C, 2021, REV AQUACULT, V13, P1709, DOI 10.1111/raq.12542
Holt C, 2011, BMC BIOINFORMATICS, V12, DOI 10.1186/1471-2105-12-491
Hughes LC, 2018, P NATL ACAD SCI USA, V115, P6249, DOI 10.1073/pnas.1719358115
Iglesias TL, 2018, J EVOLUTION BIOL, V31, P1082, DOI 10.1111/jeb.13299
Inoue J, 2015, P NATL ACAD SCI USA, V112, P14918, DOI 10.1073/pnas.1507669112
JONES DT, 1992, COMPUT APPL BIOSCI, V8, P275, DOI 10.1093/bioinformatics/8.3.275
Kalyaanamoorthy S, 2017, NAT METHODS, V14, P587, DOI [10.1038/NMETH.4285, 10.1038/nmeth.4285]
Kapustin Y, 2008, BIOL DIRECT, V3, DOI 10.1186/1745-6150-3-20
Kasahara M, 1999, IMMUNOL REV, V167, P17, DOI 10.1111/j.1600-065X.1999.tb01379.x
Kasahara M, 2019, IMMUNOGENETICS, V71, P251, DOI 10.1007/s00251-019-01105-0
Katoh K, 2014, METHODS MOL BIOL, V1079, P131, DOI 10.1007/978-1-62703-646-7_8
Langevin C, 2013, J MOL BIOL, V425, P4904, DOI 10.1016/j.jmb.2013.09.033
Lefranc MP, 2015, NUCLEIC ACIDS RES, V43, pD413, DOI 10.1093/nar/gku1056
Letunic I, 2018, NUCLEIC ACIDS RES, V46, pD493, DOI 10.1093/nar/gkx922
Litman GW, 2003, INTEGR COMP BIOL, V43, P331, DOI 10.1093/icb/43.2.331
Litman GW, 2001, IMMUNOL REV, V181, P250, DOI 10.1034/j.1600-065X.2001.1810121.x
Montgomery BC, 2011, DEV COMP IMMUNOL, V35, P1223, DOI 10.1016/j.dci.2011.03.010

Nakamura T, 2018, BIOINFORMATICS, V34, P2490, DOI 10.1093/bioinformatics/bty121
Nakatani M, 2011, BMC EVOL BIOL, V11, DOI 10.1186/1471-2148-11-177
Near TJ, 2021, MOL PHYLOGENET EVOL, V161, DOI 10.1016/j.ympev.2021.107156
Near TJ, 2013, P NATL ACAD SCI USA, V110, P12738, DOI 10.1073/pnas.1304661110
Near TJ, 2012, P NATL ACAD SCI USA, V109, P13698, DOI 10.1073/pnas.1206625109
Near TJ, 2012, P NATL ACAD SCI USA, V109, P3434, DOI 10.1073/pnas.1115169109
Nei M, 2005, ANNU REV GENET, V39, P121, DOI 10.1146/annurev.genet.39.073003.112240
Nesbitt SJ, 2003, P ROY SOC B-BIOL SCI, V270, pS234, DOI 10.1098/rsbl.2003.0066
Nguyen Lam-Tung, 2015, Mol Biol Evol, V32, P268, DOI 10.1093/molbev/msu300
Pasquier J, 2016, BMC GENOMICS, V17, DOI 10.1186/s12864-016-2709-z
Pietretti D, 2014, DEV COMP IMMUNOL, V43, P205, DOI 10.1016/j.dci.2013.08.010
Price SA, 2014, P ROY SOC B-BIOL SCI, V281, DOI 10.1098/rspb.2014.0321
Prum RO, 2015, NATURE, V526, P569, DOI 10.1038/nature15697
Quang LS, 2008, BIOINFORMATICS, V24, P2317, DOI 10.1093/bioinformatics/btn445
Rached L. Abi, 1999, Immunological Reviews, V167, P33, DOI 10.1111/j.1600-065X.1999.tb01380.x
Rebl A, 2010, VET IMMUNOL IMMUNOP, V134, P139, DOI 10.1016/j.vetimm.2009.09.021
Rodriguez-Nunez I, 2016, IMMUNOGENETICS, V68, P295, DOI 10.1007/s00251-016-0901-6
Rodríguez-Nunez I, 2014, DEV COMP IMMUNOL, V46, P24, DOI 10.1016/j.dci.2014.02.004
Salzburger W, 2018, NAT REV GENET, V19, P705, DOI 10.1038/s41576-018-0043-9
Seehausen O, 2006, P ROY SOC B-BIOL SCI, V273, P1987, DOI 10.1098/rspb.2006.3539
Shah RN, 2012, ADV HEMATOL, V2012
Sibert E, 2018, P ROY SOC B-BIOL SCI, V285, DOI 10.1098/rspb.2018.1194
Sievers F, 2018, PROTEIN SCI, V27, P135, DOI 10.1002/pro.3290
Siqueira AC, 2019, J BIOGEOGR, V46, P1611, DOI 10.1111/jbi.13631
Smithwick FM, 2018, EVOLUTION, V72, P348, DOI 10.1111/evo.13421
Soubrier J, 2012, MOL BIOL EVOL, V29, P3345, DOI 10.1093/molbev/mss140
Strong SJ, 1999, P NATL ACAD SCI USA, V96, P15080, DOI 10.1073/pnas.96.26.15080
Takezaki N, 2018, GENOME BIOL EVOL, V10, P1803, DOI 10.1093/gbe/evy125
Thompson AW, 2021, NAT GENET, V53, P1373, DOI 10.1038/s41588-021-00914-y
Traver D, 2020, AM COLL LAB, P191, DOI 10.1016/B978-0-12-812431-4.00019-1
Tukwasibwe S, 2020, CELL MOL IMMUNOL, V17, P799, DOI 10.1038/s41423-020-0482-z
Uhrberg M, 2002, IMMUNOGENETICS, V54, P221, DOI 10.1007/s00251-002-0463-7

Vilches C, 2002, ANNU REV IMMUNOL, V20, P217, DOI 10.1146/annurev.immunol.20.092501.134942
Wang Y, 2012, NUCLEIC ACIDS RES, V40, DOI 10.1093/nar/gks248
Wcisel DJ, 2020, BMC EVOL BIOL, V20, DOI 10.1186/s12862-020-01603-w
Wcisel DJ, 2017, J EXP ZOOL PART B, V328, P666, DOI 10.1002/jez.b.22738
Wcisel DJ, 2016, FISH SHELLFISH IMMUN, V53, P24, DOI 10.1016/j.fsi.2016.03.034
Wei S, 2007, IMMUNOGENETICS, V59, P813, DOI 10.1007/s00251-007-0250-6
Whelan S, 2001, MOL BIOL EVOL, V18, P691, DOI 10.1093/oxfordjournals.molbev.a003851
Wickham H, 2011, WIRES COMPUT STAT, V3, P180, DOI 10.1002/wics.147
WILLIAMS AF, 1988, ANNU REV IMMUNOL, V6, P381, DOI 10.1146/annurev.iy.06.040188.002121
Yamaguchi T, 2019, CELLS-BASEL, V8, DOI 10.3390/cells8040378
Yamanoue Y, 2011, PLOS ONE, V6, DOI 10.1371/journal.pone.0017410
YANG ZH, 1994, J MOL EVOL, V39, P306, DOI 10.1007/BF00160154
Yoder JA, 2004, P NATL ACAD SCI USA, V101, P15706, DOI 10.1073/pnas.0405242101
Yoder JA, 2008, IMMUNOGENETICS, V60, P257, DOI 10.1007/s00251-008-0285-3
Yoder JA, 2011, IMMUNOGENETICS, V63, P123, DOI 10.1007/s00251-010-0506-4
Yoder JA, 2010, IMMUNOGENETICS, V62, P117, DOI 10.1007/s00251-009-0416-5
Yoder JA, 2009, DEV COMP IMMUNOL, V33, P135, DOI 10.1016/j.dci.2008.09.004
NR 99
TC 8
Z9 9
U1 0
U2 6
PU SPRINGER
PI NEW YORK
PA ONE NEW YORK PLAZA, SUITE 4600, NEW YORK, NY, UNITED STATES
SN 0093-7711
EI 1432-1211
J9 IMMUNOGENETICS
JI Immunogenetics
PD DEC
PY 2021
VL 73
IS 6
BP 479
EP 497
DI 10.1007/s00251-021-01225-6
EA SEP 2021
PG 19
WC Genetics & Heredity; Immunology
WE Science Citation Index Expanded (SCI-EXPANDED)
SC Genetics & Heredity; Immunology
GA WS1FB
UT WOS:000695158000001
PM 34510270

OA Green Accepted, Green Submitted
DA 2025-05-07
ER

PT J
AU Wang, YQ
   Batra, A
   Schulenburg, H
   Dagan, T
AF Wang, Yiqing
   Batra, Aditi
   Schulenburg, Hinrich
   Dagan, Tal
TI Gene sharing among plasmids and chromosomes reveals barriers for
   antibiotic resistance gene transfer
SO PHILOSOPHICAL TRANSACTIONS OF THE ROYAL SOCIETY B-BIOLOGICAL SCIENCES
LA English
DT Article
DE horizontal gene transfer; antibiotic resistance; Escherichia;
   Klebsiella; Salmonella
ID TETRACYCLINE RESISTANCE; SULFONAMIDE RESISTANCE; ESCHERICHIA-COLI;
   EFFLUX PUMPS; DETERMINANTS; MECHANISMS; EVOLUTION; EXPRESSION;
   OLAQUINDOX; MOBILITY
AB The emergence of antibiotic resistant bacteria is a major threat to modern medicine. Rapid adaptation to antibiotics is often mediated by the acquisition of plasmids carrying antibiotic resistance (ABR) genes. Nonetheless, the determinants of plasmid-mediated ABR gene transfer remain debated. Here, we show that the propensity of ABR gene transfer via plasmids is higher for accessory chromosomal ABR genes in comparison with core chromosomal ABR genes, regardless of the resistance mechanism. Analysing the pattern of ABR gene occurrence in the genomes of 2635 Enterobacteriaceae isolates, we find that 33% of the 416 ABR genes are shared between chromosomes and plasmids. Phylogenetic reconstruction of ABR genes occurring on both plasmids and chromosomes supports their evolution by lateral gene transfer. Furthermore, accessory ABR genes (encoded in less than 10% of the chromosomes) occur more abundantly in plasmids in comparison with core ABR genes (encoded in greater than or equal to 90% of the chromosomes). The pattern of ABR gene occurrence in plasmids and chromosomes is similar to that in the total Escherichia genome. Our results thus indicate that the previously recognized barriers for gene acquisition by lateral gene transfer apply also to ABR genes. We propose that the functional complexity of the underlying ABR mechanism is an important determinant of ABR gene transferability. This article is part of the theme issue 'The secret lives of microbial mobile genetic elements'.
C1 [Wang, Yiqing; Dagan, Tal] Univ Kiel, Inst Gen Microbiol, Kiel, Germany.
   [Batra, Aditi; Schulenburg, Hinrich] Univ Kiel, Zool Inst, Kiel, Germany.
C3 University of Kiel; University of Kiel
RP Dagan, T (autor correspondente), Univ Kiel, Inst Gen Microbiol, Kiel, Germany.
EM tdagan@ifam.uni-kiel.de
RI Wang, Yiqing/MFJ-0189-2025; Schulenburg, Hinrich/A-3886-2009

OI Batra, Aditi/0000-0001-6910-3532; Wang, Yiqing/0000-0002-4816-5176
FU German Science Foundation [RTG 2501 TransEvo, SCHU 1415/12]; Leibniz
Science Campus Evolutionary Medicine of the Lung (EvoLUNG); Chinese
Scholarship Council (CSC)
FX We are grateful for financial support from the German Science
Foundation
(projects 3 and 4 to T.D. and H.S. within the RTG 2501 TransEvo and
grant no. SCHU 1415/12 to H.S.), the Leibniz Science Campus
Evolutionary
Medicine of the Lung (EvoLUNG to T.D. and H.S.), and Chinese
Scholarship
Council (CSC scholarship to Y.W.).
CR Abraham EP, 1940, NATURE, V146, P837, DOI 10.1038/146837a0
Kirit HA, 2020, BMC MICROBIOL, V20, DOI 10.1186/s12866-020-01983-5
Alcock BP, 2020, NUCLEIC ACIDS RES, V48, pD517, DOI 10.1093/nar/gkz935
Andersson DI, 2020, FEMS MICROBIOL REV, V44, P171, DOI
10.1093/femsre/fuaa001
BALL PR, 1980, BIOCHEM BIOPH RES CO, V93, P74, DOI 10.1016/S0006-
291X(80)80247-6
Baltrus DA, 2013, TRENDS ECOL EVOL, V28, P489, DOI
10.1016/j.tree.2013.04.002
Baquero F, 2021, NAT REV MICROBIOL, V19, P123, DOI 10.1038/s41579-020-
00443-1
BENJAMINI Y, 1995, J R STAT SOC B, V57, P289, DOI 10.1111/j.2517-
6161.1995.tb02031.x
Blanco P, 2016, MICROORGANISMS, V4, DOI 10.3390/microorganisms4010014
Blau K, 2018, MBIO, V9, DOI 10.1128/mBio.01300-18
Che Y, 2021, P NATL ACAD SCI USA, V118, DOI [10.1073/pnas.2008731118,
10.1073/pnas.2104557118]
Cohen O, 2011, MOL BIOL EVOL, V28, P1481, DOI 10.1093/molbev/msq333
D'Costa VM, 2011, NATURE, V477, P457, DOI 10.1038/nature10388
Daily J, 2016, BMC BIOINFORMATICS, V16, DOI 10.1186/s12859-016-0930-z
DATTA N, 1965, NATURE, V208, P239, DOI 10.1038/208239a0
De Oliveira DMP, 2020, CLIN MICROBIOL REV, V33, DOI 10.1128/CMR.00181-
19
Domingues S, 2012, PLOS PATHOG, V8, DOI 10.1371/journal.ppat.1002837
El Ghachi M, 2004, J BIOL CHEM, V279, P30106, DOI
10.1074/jbc.M401701200
Enright AJ, 2002, NUCLEIC ACIDS RES, V30, P1575, DOI
10.1093/nar/30.7.1575
Evans K, 1998, J BACTERIOL, V180, P5443, DOI 10.1128/JB.180.20.5443-
5447.1998
Fahmy A., 2016, Efflux-mediated antimicrobial resistance in bacteria,
DOI [10.1007/978-3-319-39658-325, DOI 10.1007/978-3-319-39658-325]
Fard RMN, 2011, LETT APPL MICROBIOL, V52, P559, DOI 10.1111/j.1472-
765X.2011.03043.x
Gallagher LA, 2017, MBIO, V8, DOI 10.1128/mBio.01655-17
García-León G, 2014, APPL ENVIRON MICROB, V80, P4559, DOI
10.1128/AEM.01058-14
González-Leiza SM, 2011, J BACTERIOL, V193, P6887, DOI
10.1128/JB.05764-11
Guo QL, 2016, J ANTIMICROB CHEMOTH, V71, P2460, DOI 10.1093/jac/dkw177
Hall JPJ, 2016, P NATL ACAD SCI USA, V113, P8260, DOI
10.1073/pnas.1600974113

Hansen LH, 2005, MICROB DRUG RESIST, V11, P378, DOI 10.1089/mdr.2005.11.378
Hansen LH, 2004, ANTIMICROB AGENTS CH, V48, P3332, DOI 10.1128/AAC.48.9.3332-3337.2004
Heuer H, 2011, CURR OPIN MICROBIOL, V14, P236, DOI 10.1016/j.mib.2011.04.009
Hinchliffe P, 2017, SCI REP-UK, V7, DOI 10.1038/srep39392
Hülter NF, 2020, FRONT MICROBIOL, V11, DOI 10.3389/fmicb.2020.02062
Jacoby GA, 2014, MICROBIOL SPECTR, V2, DOI [10.1128/microbiolspec.PLAS-0006.2013, 10.1128/microbiolspec.PLAS-0006-2013]
Jacoby GA, 2009, CLIN MICROBIOL REV, V22, P161, DOI 10.1128/CMR.00036-08
Jain R, 1999, P NATL ACAD SCI USA, V96, P3801, DOI 10.1073/pnas.96.7.3801
Jorgensen TS, 2014, PLOS ONE, V9, DOI 10.1371/journal.pone.0087924
Katoh K, 2013, MOL BIOL EVOL, V30, P772, DOI 10.1093/molbev/mst010
Kazama H, 1999, FEMS MICROBIOL LETT, V174, P379, DOI 10.1016/S0378-1097(99)00167-6
Laxminarayan R, 2013, LANCET INFECT DIS, V13, P1057, DOI 10.1016/S1473-3099(13)70318-9
Le SQ, 2008, MOL BIOL EVOL, V25, P1307, DOI 10.1093/molbev/msn067
Le SQ, 2012, MOL BIOL EVOL, V29, P2921, DOI 10.1093/molbev/mss112
Letunic I, 2019, NUCLEIC ACIDS RES, V47, pW256, DOI 10.1093/nar/gkz239
Li J, 2019, ANTIMICROB RESIST IN, V8, DOI 10.1186/s13756-019-0489-3
Li X.-Z., 2016, Efflux-Mediated Antimicrobial Resistance in Bacteria
Liu YY, 2016, LANCET INFECT DIS, V16, P161, DOI 10.1016/S1473-3099(15)00424-7
LIVERMORE DM, 1995, CLIN MICROBIOL REV, V8, P557, DOI 10.1128/CMR.8.4.557
MCMURRY L, 1980, P NATL ACAD SCI-BIOL, V77, P3974, DOI 10.1073/pnas.77.7.3974
Minh BQ, 2020, MOL BIOL EVOL, V37, P1530, DOI 10.1093/molbev/msaa015
Nies DH, 2003, FEMS MICROBIOL REV, V27, P313, DOI 10.1016/S0168-6445(03)00048-2
Novick A, 2020, BIOL PHILOS, V35, DOI 10.1007/s10539-019-9727-6
O'Leary NA, 2016, NUCLEIC ACIDS RES, V44, pD733, DOI 10.1093/nar/gkv1189
Poole K, 2007, ANN MED, V39, P162, DOI 10.1080/07853890701195262
Popa O, 2017, ISME J, V11, P543, DOI 10.1038/ismej.2016.116
RADSTROM P, 1988, ANTIMICROB AGENTS CH, V32, P1684, DOI 10.1128/AAC.32.11.1684
Redondo-Salvo S, 2020, NAT COMMUN, V11, DOI 10.1038/s41467-020-17278-2
Roberts MC, 1996, FEMS MICROBIOL REV, V19, P1, DOI 10.1111/j.1574-6976.1996.tb00251.x
Rodríguez-Beltrán J, 2020, P NATL ACAD SCI USA, V117, P15755, DOI 10.1073/pnas.2001240117
Rojas A, 2001, J BACTERIOL, V183, P3967, DOI 10.1128/JB.183.13.3967-3973.2001
Rousset F, 2021, NAT MICROBIOL, V6, P301, DOI 10.1038/s41564-020-00839-y
Ruiz C, 2014, J ANTIMICROB CHEMOTH, V69, P390, DOI 10.1093/jac/dkt352
Ruiz J, 2003, J ANTIMICROB CHEMOTH, V51, P1109, DOI 10.1093/jac/dkg222

Salverda MLM, 2010, FEMS MICROBIOL REV, V34, P1015, DOI 10.1111/j.1574-6976.2010.00222.x
Seemann T, 2014, BIOINFORMATICS, V30, P2068, DOI 10.1093/bioinformatics/btu153
SKOLD O, 1976, ANTIMICROB AGENTS CH, V9, P49, DOI 10.1128/AAC.9.1.49
Sköld O, 2000, DRUG RESIST UPDATE, V3, P155, DOI 10.1054/drup.2000.0146
Socha RD, 2019, J MOL BIOL, V431, P1172, DOI 10.1016/j.jmb.2019.01.041
Sorek R, 2007, SCIENCE, V318, P1449, DOI 10.1126/science.1147112
Steinegger M, 2017, NAT BIOTECHNOL, V35, P1026, DOI 10.1038/nbt.3988
SWEDBERG G, 1983, J BACTERIOL, V153, P1228, DOI 10.1128/JB.153.3.1228-1237.1983
Turner PE, 2014, EVOLUTION, V68, P3368, DOI 10.1111/evo.12537
Varga M, 2012, FEMS MICROBIOL LETT, V332, P146, DOI 10.1111/j.1574-6968.2012.02589.x
Wang Y., 2021, GENE SHARING PLASMID
Warinner C, 2014, NAT GENET, V46, P336, DOI 10.1038/ng.2906
Wein T, 2021, PLOS GENET, V17, DOI 10.1371/journal.pgen.1009656
Wein T, 2020, CURR BIOL, V30, pR1158, DOI 10.1016/j.cub.2020.07.003
Wein T, 2020, CURR BIOL, V30, P3841, DOI 10.1016/j.cub.2020.07.019
Wein T, 2019, NAT COMMUN, V10, DOI 10.1038/s41467-019-10600-7
Werren JH, 2011, P NATL ACAD SCI USA, V108, P10863, DOI 10.1073/pnas.1102343108
Wilson DN, 2020, NAT REV MICROBIOL, V18, P637, DOI 10.1038/s41579-020-0386-z
NR 79
TC 31
Z9 35
U1 4
U2 49
PU ROYAL SOC
PI LONDON
PA 6-9 CARLTON HOUSE TERRACE, LONDON SW1Y 5AG, ENGLAND
SN 0962-8436
EI 1471-2970
J9 PHILOS T R SOC B
JI Philos. Trans. R. Soc. B-Biol. Sci.
PD JAN 17
PY 2022
VL 377
IS 1842
AR 20200467
DI 10.1098/rstb.2020.0467
PG 11
WC Biology
WE Science Citation Index Expanded (SCI-EXPANDED)
SC Life Sciences & Biomedicine - Other Topics
GA XE1MT
UT WOS:000723160300009
PM 34839702
OA hybrid, Green Published
DA 2025-05-07
ER

PT J
AU Hopperstad, KA
Sallam, MF
Reiskind, MH
AF Hopperstad, Kristen A.
Sallam, Mohamed F.
Reiskind, Michael H.
TI Estimations of Fine-Scale Species Distributions of Aedes aegypti and Aedes albopictus (Diptera: Culicidae) in Eastern Florida
SO JOURNAL OF MEDICAL ENTOMOLOGY
LA English
DT Article
DE habitat suitability model; maximum entropy model; MaxEnt; Aedes aegypti;
Aedes albopictus
ID STEGOMYIA-AEGYPTI; HABITAT SEGREGATION; ARBOVIRUS VECTORS; SOUTH FLORIDA; MOSQUITO; DISPERSAL; ABUNDANCE; NICHE; RISK; EPIDEMIOLOGY
AB Many species distribution maps indicate the ranges of Aedes aegypti (Linnaeus) and Aedes albopictus (Skuse) overlap in Florida despite the well-documented range reduction of Ae. aegypti. Within the last 30 yr, competitive displacement of Ae. aegypti by Ae. albopictus has resulted in partial spatial segregation of the two species, with Ae. aegypti persisting primarily in urban refugia. We modeled fine-scale distributions of both species, with the goal of capturing the outcome of interspecific competition across space by building habitat suitability maps. We empirically parameterized models by sampling 59 sites in south and central Florida over time and incorporated climatic, landscape, and human population data to identify predictors of habitat suitability for both species. Our results show human density, precipitation, and urban land cover drive Ae. aegypti habitat suitability, compared with exclusively climatic variables driving Ae. albopictus habitat suitability. Remotely sensed variables (macrohabitat) were more predictive than locally collected metrics (microhabitat), although recorded minimum daily temperature showed significant, inverse relationships with both species. We detected minor Aedes habitat segregation; some periurban areas that were highly suitable for Ae. albopictus were unsuitable for Ae. aegypti. Fine-scale empirical models like those presented here have the potential for precise risk assessment and the improvement of operational applications to control container-breeding Aedes mosquitoes.
C1 [Hopperstad, Kristen A.; Reiskind, Michael H.] North Carolina State Univ, Dept Entomol & Plant Pathol, Campus Box 7613, Raleigh, NC 27696 USA.
[Sallam, Mohamed F.] Univ Nevada, Dept Biol, Reno, NV 89557 USA.
C3 North Carolina State University; Nevada System of Higher Education (NSHE); University of Nevada Reno
RP Hopperstad, KA (autor correspondente), North Carolina State Univ, Dept Entomol & Plant Pathol, Campus Box 7613, Raleigh, NC 27696 USA.
EM kahopper@ncsu.edu
RI Reiskind, Michael/I-5614-2012; (Hopperstad) Breaux, Kristen/U-4728-2018
OI (Hopperstad) Breaux, Kristen/0000-0002-3622-153X; Reiskind, Michael/0000-0001-7307-9416; Sallam, Mohamed/0000-0003-2817-6190

FU National Science Foundation Graduate Research Fellowship [DGE-1252376];
USDA Multistate Project [NE-1943]; Triangle Center for Evolutionary Medicine
FX We would like to thank Dr. L. Phil Lounibos and Dr. Walter Tabachnick of the Florida Medical Entomology laboratory in Vero Beach, FL for sharing their facility and expertise and Kyle Jones for his field assistance. We also thank Thomas Pleasant and Cole Keenan for digitizing site-level land cover. This material is based upon work supported by the National Science Foundation Graduate Research Fellowship under Grant No. (DGE-1252376), USDA Multistate Project NE-1943, and the Triangle Center for Evolutionary Medicine.
CR Anderson J.R., 1976, A Land Use and Land Cover Classification System for Use with Remote Sensor Data, P964
Bargielowski IE, 2013, P NATL ACAD SCI USA, V110, P2888, DOI 10.1073/pnas.1219599110
Benedict MQ, 2007, VECTOR-BORNE ZOONOT, V7, P76, DOI 10.1089/vbz.2006.0562
Braks MAH, 2003, J MED ENTOMOL, V40, P785, DOI 10.1603/0022-2585-40.6.785
Burkett-Cadena ND, 2013, ECOSPHERE, V4, DOI 10.1890/ES13-00009.1
Costanzo KS, 2005, ECOLOGY, V86, P3289, DOI 10.1890/05-0583
Damal K, 2013, AM J TROP MED HYG, V89, P482, DOI 10.4269/ajtmh.13-0102
de Resende MC, 2013, MEM I OSWALDO CRUZ, V108, P1024, DOI 10.1590/0074-0276130128
Ding FY, 2018, ACTA TROP, V178, P155, DOI 10.1016/j.actatropica.2017.11.020
Elith J, 2011, DIVERS DISTRIB, V17, P43, DOI 10.1111/j.1472-4642.2010.00725.x
Eritja R, 2017, SCI REP-UK, V7, DOI 10.1038/s41598-017-12652-5
Guagliardo SA, 2015, PLOS NEGLECT TROP D, V9, DOI 10.1371/journal.pntd.0003648
Hahn MB, 2016, J MED ENTOMOL, V53, P1169, DOI 10.1093/jme/tjw072
Harrington LC, 2014, PLOS NEGLECT TROP D, V8, DOI 10.1371/journal.pntd.0003048
Hopperstad KA, 2016, J MED ENTOMOL, V53, P836, DOI 10.1093/jme/tjw050
HUTCHINSON GE, 1957, COLD SPRING HARB SYM, V22, P415, DOI 10.1101/SQB.1957.022.01.039
Johnson TL, 2017, J MED ENTOMOL, V54, P1605, DOI 10.1093/jme/tjx163
Juliano SA, 2002, OECOLOGIA, V130, P458, DOI 10.1007/s004420100811
Kitron U, 1998, J MED ENTOMOL, V35, P435, DOI 10.1093/jmedent/35.4.435
Kraemer MUG, 2015, ELIFE, V4, DOI 10.7554/eLife.08347
Landau KI, 2012, J VECTOR ECOL, V37, P407, DOI 10.1111/j.1948-7134.2012.00245.x
Leisnham PT, 2008, J MED ENTOMOL, V45, P210, DOI 10.1603/0022-2585(2008)45[210:GVIASA]2.0.CO;2
Liu BY, 2019, SCI TOTAL ENVIRON, V664, P203, DOI 10.1016/j.scitotenv.2019.01.301
Lounibos LP, 2018, BIOL INVASIONS, V20, P1913, DOI 10.1007/s10530-018-1674-7

Lounibos LP, 2016, J MED ENTOMOL, V53, P1385, DOI 10.1093/jme/tjw122
Lozano-Fuentes S, 2014, J MED ENTOMOL, V51, P742, DOI 10.1603/ME14015
Madden H., 1945, Fla Entomol, V28, P1
Marini F, 2010, MED VET ENTOMOL, V24, P361, DOI 10.1111/j.1365-2915.2010.00898.x
Medley KA, 2015, MOL ECOL, V24, P284, DOI 10.1111/mec.12925
Medley KA, 2010, GLOBAL ECOL BIOGEOGR, V19, P122, DOI 10.1111/j.1466-8238.2009.00497.x
Merow C, 2013, ECOGRAPHY, V36, P1058, DOI 10.1111/j.1600-0587.2013.07872.x
Monaghan A.J., 2016, PLOS CURRENTS OUTBRE, V8, P1, DOI 10.1371/currents.outbreaks.50dfc7f46798675fc63e7d7da563da76
MORLAN HB, 1965, AM J TROP MED HYG, V14, P892, DOI 10.4269/ajtmh.1965.14.892
Obenauer JF, 2017, TROP MED HEALTH, V45, DOI 10.1186/s41182-017-0078-1
OMEARA GF, 1995, J MED ENTOMOL, V32, P554, DOI 10.1093/jmedent/32.4.554
Padmanabha H, 2012, PLOS NEGLECT TROP D, V6, DOI 10.1371/journal.pntd.0001799
Pavlovskii˘ E., 1966, Natural Nidality of Transmissible Diseases; With Special Reference to the Landscape Epidemiology of Zooanthroponoses
Phillips SJ, 2006, ECOL MODEL, V190, P231, DOI 10.1016/j.ecolmodel.2005.03.026
Reisen WK, 2010, ANNU REV ENTOMOL, V55, P461, DOI 10.1146/annurev-ento-112408-085419
Reiskind MH, 2013, MED VET ENTOMOL, V27, P421, DOI 10.1111/mve.12000
Reiskind MH, 2009, MED VET ENTOMOL, V23, P62, DOI 10.1111/j.1365-2915.2008.00782.x
REITER P, 1995, AM J TROP MED HYG, V52, P177, DOI 10.4269/ajtmh.1995.52.177
Rey JR, 2006, J MED ENTOMOL, V43, P1134, DOI 10.1603/0022-2585(2006)43[1134:HSOMAV]2.0.CO;2
Richards SL, 2006, J MED ENTOMOL, V43, P543, DOI 10.1603/0022-2585(2006)43[543:HPOAAD]2.0.CO;2
Rochlin I, 2016, NAT COMMUN, V7, DOI 10.1038/ncomms13604
Sallam MF, 2017, INT J ENV RES PUB HE, V14, DOI 10.3390/ijerph14101230
Sallam MF, 2017, INT J ENV RES PUB HE, V14, DOI 10.3390/ijerph14080892
Sallam MF, 2016, PARASITE VECTOR, V9, DOI 10.1186/s13071-016-1646-7
SAS Institute Inc, 2018, Using JMP 14
Soberón J, 2007, ECOL LETT, V10, P1115, DOI 10.1111/j.1461-0248.2007.01107.x
Soberón J, 2009, P NATL ACAD SCI USA, V106, P19644, DOI 10.1073/pnas.0901637106
Terando AJ, 2014, PLOS ONE, V9, DOI 10.1371/journal.pone.0102261
Vora Neil, 2008, Medscape J Med, V10, P238
Wijayanti SPM, 2016, PLOS NEGLECT TROP D, V10, DOI 10.1371/journal.pntd.0004964
Yee DA, 2014, MED VET ENTOMOL, V28, P60, DOI 10.1111/mve.12007
Zitko T, 2014, J MED ENTOMOL, V51, P760, DOI 10.1603/ME13071
NR 56
TC 15
Z9 20
U1 0
U2 22

PU OXFORD UNIV PRESS INC
PI CARY
PA JOURNALS DEPT, 2001 EVANS RD, CARY, NC 27513 USA
SN 0022-2585
EI 1938-2928
J9 J MED ENTOMOL
JI J. Med. Entomol.
PD MAR
PY 2021
VL 58
IS 2
BP 699
EP 707
DI 10.1093/jme/tjaa216
PG 9
WC Entomology; Veterinary Sciences
WE Science Citation Index Expanded (SCI-EXPANDED)
SC Entomology; Veterinary Sciences
GA RJ0UX
UT WOS:000637320200023
PM 33128447
OA hybrid
DA 2025-05-07
ER

PT J
AU Lapp, Z
   Obala, AA
   Abel, L
   Rasmussen, DA
   Sumner, KM
   Freedman, E
   Taylor, SM
   Prudhomme-O'Meara, W
AF Lapp, Zena
   Obala, Andrew A.
   Abel, Lucy
   Rasmussen, David A.
   Sumner, Kelsey M.
   Freedman, Elizabeth
   Taylor, Steve M.
   Prudhomme-O'Meara, Wendy
TI Plasmodium falciparum Genetic Diversity in Coincident Human and
   Mosquito Hosts
SO MBIO
LA English
DT Article
DE malaria; Plasmodium falciparum; transmission; comparative genomics;
   genetic diversity
ID R PACKAGE; MALARIA; ANOPHELES; POLYMORPHISMS; TRANSMISSION;
RESISTANCE;
   PARASITES; REVEALS; RISK
AB Population genetic diversity of Plasmodium falciparum antigenic loci
is high despite large bottlenecks in population size during the parasite

life cycle. The prevalence of genetically distinct haplotypes at these loci, while well characterized in humans, has not been thoroughly compared between human and mosquito hosts. We assessed parasite haplotype prevalence, diversity, and evenness using human and mosquito P. falciparum infections collected from the same households during a 14-month longitudinal cohort study using amplicon deep sequencing of two antigenic gene fragments (ama1 and csp). To a prior set of infected humans (n = 1,175/2,813; 86.2% sequencing success) and mosquito abdomens (n = 199/1,448; 95.5% sequencing success), we added sequences from infected mosquito heads (n = 134/1,448; 98.5% sequencing success). The overall and sample-level parasite populations were more diverse in mosquitoes than in humans. Additionally, haplotype prevalences were more even in the P. falciparum human population than in the mosquito population, consistent with balancing selection occurring at these loci in humans. In contrast, we observed that infections in humans were more likely to harbor a dominant haplotype than infections in mosquitoes, potentially due to removal of unfit strains by the human immune system. Finally, within a given mosquito, there was little overlap in genetic composition of abdomen and head infections, suggesting that infections may be cleared from the abdomen during a mosquito's lifespan. Taken together, our observations provide evidence for the mosquito vector acting as a reservoir of sequence diversity in malaria parasite populations.

IMPORTANCE Plasmodium falciparum is the deadliest human malaria parasite, and infections consisting of concurrent, multiple strains are common in regions of high endemicity. During transitions within and between the parasite's mosquito and human hosts, these strains are subject to population bottlenecks, and distinct parasite strains may have differential fitness in the various environments encountered. These bottlenecks and fitness differences may lead to differences in strain prevalence and diversity between hosts. We investigated differences in genetic diversity and evenness between P. falciparum parasites in human and mosquito hosts collected from the same households during a 14-month longitudinal study in Kenya. Compared to human parasite populations and infections, P. falciparum parasites observed in mosquito populations and infections were more diverse by multiple population genetic metrics. This suggests that the mosquito vector acts as a reservoir of sequence diversity in malaria parasite populations.
C1 [Lapp, Zena; Taylor, Steve M.; Prudhomme-O'Meara, Wendy] Duke Univ, Duke Global Hlth Inst, Durham, NC 27708 USA.

[Obala, Andrew A.] Moi Univ, Coll Hlth Sci, Sch Med, Eldoret, Kenya.

[Abel, Lucy] Acad Model Providing Access Healthcare AMPATH, Eldoret, Kenya.

[Rasmussen, David A.] North Carolina State Univ, Dept Entomol & Plant Pathol, Raleigh, NC USA.

[Rasmussen, David A.] North Carolina State Univ, Bioinformat Res Ctr, Raleigh, NC USA.

[Sumner, Kelsey M.] Univ N Carolina, Gillings Sch Global Publ Hlth, Dept Epidemiol, Chapel Hill, NC USA.

[Sumner, Kelsey M.; Freedman, Elizabeth; Taylor, Steve M.; Prudhomme-O'Meara, Wendy] Duke Univ, Div Infect Dis, Sch Med, Durham, NC 27708 USA.

C3 Duke University; Moi University; North Carolina State University; North

Carolina State University; University of North Carolina; University of

North Carolina Chapel Hill; Duke University
RP Taylor, SM (autor correspondente), Duke Univ, Duke Global Hlth Inst, Durham, NC 27708 USA.; Taylor, SM (autor correspondente), Duke Univ, Div Infect Dis, Sch Med, Durham, NC 27708 USA.
EM steve.taylor@duke.edu
RI Abel, Lucy/AAF-9225-2019
OI Taylor, Steve/0000-0002-2783-0990; Rasmussen, David/0000-0001-9457-7561;
Lapp, Zena/0000-0003-4674-2176
FU NIAID [R21AI126024, R01AI146849]; Triangle Center for Evolutionary Medicine; National Human Genome Research Institute [T32HG000040] Funding
Source: NIH RePORTER
FX This work was supported by NIAID (R21AI126024 to W.P.O. and R01AI146849
to W.P.O. and S.M.T.) and the Triangle Center for Evolutionary Medicine
(Graduate Student Award to K.M.S.).
CR Berry A, 2021, MICROBIOL SPECTR, V9, DOI 10.1128/Spectrum.00852-21
Callahan BJ, 2016, NAT METHODS, V13, P581, DOI [10.1038/NMETH.3869, 10.1038/nmeth.3869]
Chang HH, 2013, P NATL ACAD SCI USA, V110, P20129, DOI 10.1073/pnas.1319857110
Chang HH, 2012, MOL BIOL EVOL, V29, P3427, DOI 10.1093/molbev/mss161
Chao AN, 2021, METHODS ECOL EVOL, V12, P1926, DOI 10.1111/2041-210X.13682
Chao AN, 2020, ECOL RES, V35, P292, DOI 10.1111/1440-1703.12102
Chao A, 2019, ECOLOGY, V100, DOI 10.1002/ecy.2852
Cohuet A, 2010, TRENDS PARASITOL, V26, P130, DOI 10.1016/j.pt.2009.12.001
Conrad MD, 2017, AM J TROP MED HYG, V97, P1576, DOI 10.4269/ajtmh.17-0351
Demas A, 2011, J CLIN MICROBIOL, V49, P2411, DOI 10.1128/JCM.02603-10
Duke Health, 2020, PLASMODIUM FALCIPARU
Early AM, 2019, MALARIA J, V18, DOI 10.1186/s12936-019-2856-1
Graumans W, 2020, TRENDS PARASITOL, V36, P705, DOI 10.1016/j.pt.2020.05.011
Greenhouse B, 2011, J INFECT DIS, V204, P19, DOI 10.1093/infdis/jir223
Grubaugh ND, 2016, CELL HOST MICROBE, V19, P481, DOI 10.1016/j.chom.2016.03.002
HILL MO, 1973, ECOLOGY, V54, P427, DOI 10.2307/1934352
Jaccard P., 1912, New Phytologist, V11, P37, DOI DOI 10.1111/J.1469-8137.1912.TB05611.X
Koella JC, 1998, P ROY SOC B-BIOL SCI, V265, P763, DOI 10.1098/rspb.1998.0358
Lapp Z., 2022, DATA CODE PLASMODIUM
Malaguarnera L, 2002, LANCET INFECT DIS, V2, P472, DOI 10.1016/S1473-3099(02)00344-4
Mendes C, 2013, MALARIA J, V12, DOI 10.1186/1475-2875-12-114
Mharakurwa S, 2013, MALARIA J, V12, DOI 10.1186/1475-2875-12-453
Mharakurwa S, 2011, P NATL ACAD SCI USA, V108, P18796, DOI 10.1073/pnas.1116162108
Molina-Cruz A, 2015, P NATL ACAD SCI USA, V112, P15178, DOI 10.1073/pnas.1520426112

Morlais I, 2015, PLOS ONE, V10, DOI 10.1371/journal.pone.0123777
Mu JB, 2005, PLOS BIOL, V3, P1734, DOI 10.1371/journal.pbio.0030335
Paradis E, 2019, BIOINFORMATICS, V35, P526, DOI 10.1093/bioinformatics/bty633
Paradis E, 2018, METHODS ECOL EVOL, V9, P1308, DOI 10.1111/2041-210X.12969
Paradis E, 2010, BIOINFORMATICS, V26, P419, DOI 10.1093/bioinformatics/btp696
Pollitt LC, 2015, PLOS PATHOG, V11, DOI 10.1371/journal.ppat.1005003
Raats M. M., 1992, Food Quality and Preference, V3, P89, DOI 10.1016/0950-3293(91)90028-D
Rigby RA, 2005, J ROY STAT SOC C, V54, P507, DOI 10.1111/j.1467-9876.2005.00510.x
Scott TW, 2012, TRENDS PARASITOL, V28, P114, DOI 10.1016/j.pt.2012.01.001
Sumner KM, 2021, NAT COMMUN, V12, DOI 10.1038/s41467-021-21269-2
Taylor SM, 2019, AM J TROP MED HYG, V101, P1416, DOI 10.4269/ajtmh.19-0558
Wickham H., 2019, J. Open Source Softw, V4, P1686, DOI DOI 10.21105/JOSS.01686
Wickham H., 2016, ggplot2: Elegant Graphics for Data Analysis, DOI DOI 10.1007/978-3-319-24277-4
Wiernik Brenton M, 2022, CRAN
Wilke Claus O, 2024, CRAN
Yu GC, 2017, METHODS ECOL EVOL, V8, P28, DOI 10.1111/2041-210X.12628
NR 40
TC 5
Z9 6
U1 1
U2 3
PU AMER SOC MICROBIOLOGY
PI WASHINGTON
PA 1752 N ST NW, WASHINGTON, DC 20036-2904 USA
SN 2150-7511
J9 MBIO
JI mBio
PD OCT 26
PY 2022
VL 13
IS 5
DI 10.1128/mbio.02277-22
EA SEP 2022
PG 11
WC Microbiology
WE Science Citation Index Expanded (SCI-EXPANDED)
SC Microbiology
GA 5X2SZ
UT WOS:000854184500007
PM 36073811
OA gold, Green Submitted, Green Published
DA 2025-05-07
ER

PT J

AU Xu, S
Ware, KE
Ding, YT
Kim, SY
Sheth, MU
Rao, S
Chan, W
Armstrong, AJ
Eward, WC
Jolly, MK
Somarelli, JA
AF Xu, Shengnan
Ware, Kathryn E.
Ding, Yuantong
Kim, So Young
Sheth, Maya U.
Rao, Sneha
Chan, Wesley
Armstrong, Andrew J.
Eward, William C.
Jolly, Mohit Kumar
Somarelli, Jason A.
TI An Integrative Systems Biology and Experimental Approach Identifies Convergence of Epithelial Plasticity, Metabolism, and Autophagy to Promote Chemoresistance
SO JOURNAL OF CLINICAL MEDICINE
LA English
DT Article
DE evolution; systems biology; autophagy; lung cancer; epithelial-mesenchymal transition; tumor invasiveness; metabolism
ID TO-MESENCHYMAL TRANSITION; TGF-BETA; CANCER EVOLUTION; TUMOR-GROWTH; STEM-CELLS; RESISTANCE; METASTASIS; INVASION; MECHANISMS; EXPRESSION
AB The evolution of therapeutic resistance is a major cause of death for cancer patients. The development of therapy resistance is shaped by the ecological dynamics within the tumor microenvironment and the selective pressure of the host immune system. These selective forces often lead to evolutionary convergence on pathways or hallmarks that drive progression. Thus, a deeper understanding of the evolutionary convergences that occur could reveal vulnerabilities to treat therapy-resistant cancer. To this end, we combined phylogenetic clustering, systems biology analyses, and molecular experimentation to identify convergences in gene expression data onto common signaling pathways. We applied these methods to derive new insights about the networks at play during transforming growth factor-beta (TGF-beta)-mediated epithelial-mesenchymal transition in lung cancer. Phylogenetic analyses of gene expression data from TGF-beta-treated cells revealed convergence of cells toward amine metabolic pathways and autophagy during TGF-beta treatment. Knockdown of the autophagy regulatory, ATG16L1, re-sensitized lung cancer cells to cancer therapies following TGF-beta-induced resistance, implicating autophagy as a TGF-beta-mediated chemoresistance mechanism. In addition, high ATG16L expression was found to be a poor prognostic marker in multiple cancer types. These analyses reveal the usefulness of combining evolutionary and systems biology methods with experimental validation to illuminate new therapeutic vulnerabilities for cancer.

C1 [Xu, Shengnan; Ware, Kathryn E.; Sheth, Maya U.; Chan, Wesley; Armstrong, Andrew J.; Somarelli, Jason A.] Duke Univ, Duke Canc Inst, Med Ctr, Durham, NC 27710 USA.
[Xu, Shengnan; Ware, Kathryn E.; Sheth, Maya U.; Chan, Wesley; Armstrong, Andrew J.; Somarelli, Jason A.] Duke Univ, Dept Med, Med Ctr, Durham, NC 27710 USA.
[Ding, Yuantong] Duke Univ, Dept Biol, Med Ctr, Durham, NC 27710 USA.
[Kim, So Young] Duke Univ, Dept Mol Genet & Microbiol, Med Ctr, Durham, NC 27710 USA.
[Rao, Sneha; Eward, William C.] Duke Univ, Dept Orthopaed Surg, Med Ctr, Durham, NC 27710 USA.
[Armstrong, Andrew J.] Duke Univ, Solid Tumor Program, Med Ctr, Durham, NC 27710 USA.
[Armstrong, Andrew J.] Duke Univ, Duke Prostate & Urol Canc Ctr, Med Ctr, Durham, NC 27710 USA.
[Jolly, Mohit Kumar] Rice Univ, Ctr Theoret Biol Phys, Houston, TX 77005 USA.
[Jolly, Mohit Kumar] Indian Inst Sci, Ctr BioSyst Sci & Engn, Bangalore 560012, Karnataka, India.
C3 Duke University; Duke University; Duke University; Duke University; Duke
University; Duke University; Duke University; Rice University; Indian Institute of Science (IISC) - Bangalore
RP Somarelli, JA (autor correspondente), Duke Univ, Duke Canc Inst, Med Ctr, Durham, NC 27710 USA.; Somarelli, JA (autor correspondente), Duke Univ, Dept Med, Med Ctr, Durham, NC 27710 USA.; Jolly, MK (autor correspondente), Rice Univ, Ctr Theoret Biol Phys, Houston, TX 77005 USA.
EM shengnan.xu@duke.edu; kathryn.ware@duke.edu; yuantong.ding@duke.edu;
soyoung.kim@duke.edu; maya.sheth@duke.edu; sneha.rao@duke.edu;
wesley.chan@duke.edu; andrew.armstrong@duke.edu; william.eward@duke.edu;
mkjolly.15@gmail.com; jason.somarelli@duke.edu
RI Jolly, Mohit Kumar/A-1826-2014
OI Jolly, Mohit Kumar/0000-0002-6631-2109; Armstrong, Andrew/0000-0001-7012-1754; Sheth, Maya/0000-0003-1466-8899; Kim, So Young/0000-0002-5671-1878; Somarelli, Jason/0000-0003-1510-9343
FU Paul and Shirley Friedland Fund; Triangle Center for Evolutionary Medicine; Gulf Coast Consortia on Computational Cancer Biology Training
Program (CPRIT) [RP170593]
FX J.A.S. wishes to acknowledge support from Meg and Bill Lindenberger, the
Paul and Shirley Friedland Fund, the Triangle Center for Evolutionary Medicine, and funds raised in memory of Muriel E. Rudershausen (riding4research.org). The authors wish to thank Jeffrey Townsend and Herbert Levine for many helpful discussions in preparation of the manuscript. M.K.J. is supported by a training fellowship from the Gulf Coast Consortia on Computational Cancer Biology Training Program (CPRIT
Grant No. RP170593).
CR Anderson ARA, 2008, NAT REV CANCER, V8, P227, DOI 10.1038/nrc2329
Barrow-McGee R, 2016, NAT COMMUN, V7, DOI 10.1038/ncomms11942
Berriz GF, 2003, BIOINFORMATICS, V19, P2502, DOI 10.1093/bioinformatics/btg363

Brown WS, 2016, MOL CANCER THER, V15, P2096, DOI 10.1158/1535-7163.MCT-16-0136
Brunen D, 2013, CELL CYCLE, V12, P2960, DOI 10.4161/cc.26034
Carthy JM, 2018, J CELL PHYSIOL, V233, P98, DOI 10.1002/jcp.25879
Casasent AK, 2017, J PATHOL, V241, P208, DOI 10.1002/path.4840
Chaiwanon J, 2016, CELL, V164, P1257, DOI 10.1016/j.cell.2016.01.044
Cheong H, 2012, AUTOPHAGY, V8, P122, DOI 10.4161/auto.8.1.18078
Du BW, 2016, MOLECULES, V21, DOI 10.3390/molecules21070965
Eng CH, 2010, SCI SIGNAL, V3, DOI 10.1126/scisignal.2000911
Feinberg AP, 2014, J INTERN MED, V276, P5, DOI 10.1111/joim.12224
Fischer KR, 2015, NATURE, V527, P472, DOI 10.1038/nature15748
Fuxe J, 2010, CELL CYCLE, V9, P2363, DOI 10.4161/cc.9.12.12050
Gatenby R, 2018, CSH PERSPECT MED, V8, DOI 10.1101/cshperspect.a033415
Gerlinger M, 2014, ANNU REV GENET, V48, P215, DOI 10.1146/annurev-genet-120213-092314
Gigante M, 2012, CURR PHARM DESIGN, V18, P4126, DOI 10.2174/138161212802430378
Guido C, 2012, CELL CYCLE, V11, P3019, DOI 10.4161/cc.21384
Hanahan D, 2011, CELL, V144, P646, DOI 10.1016/j.cell.2011.02.013
Hazelbag S, 2002, HUM PATHOL, V33, P1193, DOI 10.1053/hupa.2002.130109
HILLIS DM, 1993, SYST BIOL, V42, P182, DOI 10.2307/2992540
HILLIS DM, 1994, SCIENCE, V264, P671, DOI 10.1126/science.8171318
Huang SS, 2005, J CELL BIOCHEM, V96, P447, DOI 10.1002/jcb.20558
Iqbal W, 2016, ONCOTARGET, V7, P76337, DOI 10.18632/oncotarget.10942
Janda E, 2002, J CELL BIOL, V156, P299, DOI 10.1083/jcb.200109037
Janda E, 2002, ONCOGENE, V21, P5148, DOI 10.1038/sj.onc.1205661
Jiang L, 2015, ONCOGENE, V34, P3908, DOI 10.1038/onc.2014.321
Kitisin Krit, 2007, Sci STKE, V2007, pcm1, DOI 10.1126/stke.3992007cm1
Kiyono K, 2009, CANCER RES, V69, P8844, DOI 10.1158/0008-5472.CAN-08-4401
Kogan-Sakin I, 2011, CELL DEATH DIFFER, V18, P271, DOI 10.1038/cdd.2010.94
KURTZ I, 1990, MINER ELECTROL METAB, V16, P331
Lipinski KA, 2016, TRENDS CANCER, V2, P49, DOI 10.1016/j.trecan.2015.11.003
Lovisa S, 2015, NAT MED, V21, P998, DOI 10.1038/nm.3902
Magi Shigeyuki, 2017, Current Opinion in Systems Biology, V2, P39, DOI 10.1016/j.coisb.2017.02.008
Maley CC, 2017, NAT REV CANCER, V17, P605, DOI 10.1038/nrc.2017.69
Moustakas A, 2002, IMMUNOL LETT, V82, P85, DOI 10.1016/S0165-2478(02)00023-8
Mross K, 2012, CLIN CANCER RES, V18, P2658, DOI 10.1158/1078-0432.CCR-11-1900
Nawshad A, 2005, CELLS TISSUES ORGANS, V179, P11, DOI 10.1159/000084505
Neal JW, 2016, LANCET ONCOL, V17, P1661, DOI 10.1016/S1470-2045(16)30561-7
NOWELL PC, 1976, SCIENCE, V194, P23, DOI 10.1126/science.959840
Ostrow SL, 2014, PLOS GENET, V10, DOI 10.1371/journal.pgen.1004239
Papageorgis P, 2015, J ONCOL, V2015, DOI 10.1155/2015/587193
Paradis E, 2004, BIOINFORMATICS, V20, P289, DOI [10.1093/bioinformatics/btg412, 10.1093/bioinformatics/bty633]
Pasquini G, 2018, EXPERT OPIN INV DRUG, V27, P363, DOI 10.1080/13543784.2018.1462336

Pawson CT, 2010, NAT STRUCT MOL BIOL, V17, P653, DOI 10.1038/nsmb.1843
Rastogi I, 2016, BIOCHEM BIOPH RES CO, V477, P937, DOI 10.1016/j.bbrc.2016.07.003
Razani B, 2001, J BIOL CHEM, V276, P6727, DOI 10.1074/jbc.M008340200
SAITOU N, 1987, MOL BIOL EVOL, V4, P406, DOI 10.1093/oxfordjournals.molbev.a040454
Sartor MA, 2010, BIOINFORMATICS, V26, P456, DOI 10.1093/bioinformatics/btp683
Schiller JH, 2009, J CLIN ONCOL, V27, P3836, DOI 10.1200/JCO.2008.20.8355
Schliep KP, 2011, BIOINFORMATICS, V27, P592, DOI 10.1093/bioinformatics/btq706
Sever R, 2015, CSH PERSPECT MED, V5, DOI 10.1101/cshperspect.a006098
Seyfried Thomas N., 2013, Critical Reviews in Oncogenesis, V18, P43
Shiroto T, 2014, PLOS ONE, V9, DOI 10.1371/journal.pone.0087871
Singh A, 2010, ONCOGENE, V29, P4741, DOI 10.1038/onc.2010.215
Somarelli JA, 2017, BBA-REV CANCER, V1867, P101, DOI 10.1016/j.bbcan.2016.10.006
Somarelli JA, 2016, MOL CELL BIOL, V36, P2503, DOI 10.1128/MCB.00373-16
Sui X, 2013, CELL DEATH DIS, V4, DOI 10.1038/cddis.2013.350
Takahashi E, 2010, J BIOL CHEM, V285, P4060, DOI 10.1074/jbc.M109.056523
van Golen KL, 2002, CLIN EXP METASTAS, V19, P301, DOI 10.1023/A:1015518114931
Vega S, 2004, GENE DEV, V18, P1131, DOI 10.1101/gad.294104
Walton KL, 2017, FRONT PHARMACOL, V8, DOI 10.3389/fphar.2017.00461
Wiens JJ, 1995, SYST BIOL, V44, P482, DOI 10.2307/2413656
Yang ZNJ, 2011, MOL CANCER THER, V10, P1533, DOI 10.1158/1535-7163.MCT-11-0047
Yao Z, 2010, P NATL ACAD SCI USA, V107, P15535, DOI 10.1073/pnas.1009472107
Zheng XF, 2015, NATURE, V527, P525, DOI 10.1038/nature16064
NR 66
TC 13
Z9 15
U1 0
U2 3
PU MDPI
PI BASEL
PA ST ALBAN-ANLAGE 66, CH-4052 BASEL, SWITZERLAND
EI 2077-0383
J9 J CLIN MED
JI J. Clin. Med.
PD FEB
PY 2019
VL 8
IS 2
AR 205
DI 10.3390/jcm8020205
PG 15
WC Medicine, General & Internal
WE Science Citation Index Expanded (SCI-EXPANDED)
SC General & Internal Medicine

GA HN6KR
UT WOS:000460295400078
PM 30736412
OA gold, Green Submitted, Green Accepted, Green Published
DA 2025-05-07
ER

PT J
AU Diel, R
   Meywald-Walter, K
   Schwarzbach, C
   Voss, K
   Dreyer, V
   Niemann, S
AF Diel, Roland
   Meywald-Walter, Karen
   Schwarzbach, Christian
   Voss, Klaas
   Dreyer, Viola
   Niemann, Stefan
TI Risk of tuberculosis transmission by children in Hamburg, Germany
SO RESPIRATORY MEDICINE
LA English
DT Article
DE Children; Tuberculosis; Transmission; Genotyping; Whole-genome
   sequencing; Epidemiology; IGRA
ID MYCOBACTERIUM-TUBERCULOSIS
AB Background: Data from a prospective molecular-epidemiologic study (1997-2021) in Hamburg, Germany, were evaluated to assess the transmission risk of Mycobacterium tuberculosis complex (Mtbc) by children <15 years in a low-incidence setting.

Methods: Isolates of Mtbc were genotyped whole genome sequencing, applying a core genome multilocus sequence typing scheme. Close contacts of culture-confirmed children were examined for latent Mtbc infections (LTBI) with particular focus on IGRA testing.

Results: Out of 3154 culture-confirmed tuberculosis (TB) cases, 79 (2.5%) were children <15 years. Of those, 52 (58%) had pulmonary TB. Genotyping revealed that 35 of the 52 children (67%) were epidemiologically confirmed secondary cluster members; all of their source cases were adults. Six immigrant children presented without a presumed source case; their TB diagnoses came on average 48 weeks (interquartile range [IQR] 71) after their arrival in Germany. Three German-born children were determined to have been infected by adult relatives while visiting their parents' home country. Of the 317 children's close contacts tested with QuantiFERON-TB Gold-In Tube for LTBI, only 21 (6.6%) were positive. Absent a history of prior exposure or immigration from a high-incidence country, none of the contacts of younger (<10 years) TB-afflicted children was latently infected, whereas 2 older children infected 12 of their contacts, children and adults. During a mean observational period of 551 weeks (IQR 735) on average, no secondary TB cases appeared.

Conclusions: Children with pulmonary TB disease, especially those aged below 10 years, rarely transmit Mtbc to their close contacts in a low-incidence setting.

C1 [Diel, Roland] Univ Med Hosp Schleswig Holstein, Inst Epidemiol, Kiel, Germany.
[Diel, Roland] German Ctr Lung Res DZL, Airway Res Ctr North ARCN, LungClin Grosshansdorf, Kiel, Germany.
[Meywald-Walter, Karen; Schwarzbach, Christian; Voss, Klaas] Publ Hlth Dept Hamburg Cent, Hamburg, Germany.
[Dreyer, Viola; Niemann, Stefan] Natl Reference Lab Mycobacteria, Res Ctr Borstel, Mol & Expt Mycobacteriol Grp, Borstel, Germany.
[Dreyer, Viola; Niemann, Stefan] German Ctr Infect Res, Borstel, Germany.
C3 Leibniz Association; Forschungszentrum Borstel; German Center for Infection Research
RP Diel, R (autor correspondente), Univ Med Hosp Schleswig Holstein, Inst Epidemiol, Kiel, Germany.
EM roland.diel@epi.uni-kiel.de
RI Niemann, Stefan/C-9327-2011
OI Schwarzbach, Christian/0000-0003-1468-678X; Niemann, Stefan/0000-0002-6604-0684; Dreyer, Viola/0000-0003-2997-3693
FU Deutsche Forschungsgemeinschaft (DFG, German Research Foundation) under Germanys Excellence Strategy [EXC 2167]; Leibniz Science Campus Evolutionary Medicine of the LUNG (EvoLUNG)
FX Parts of this work have been supported by the Deutsche Forschungsgemeinschaft (DFG, German Research Foundation) under Germanys Excellence Strategy - EXC 2167, and Leibniz Science Campus Evolutionary Medicine of the LUNG (EvoLUNG) . The funders had no role in study design; in the collection, analysis, and interpretation of the data; in the writing of the report; and in the decision to submit the paper for publication.
CR Cardona M, 1999, J PAEDIATR CHILD H, V35, P375, DOI 10.1046/j.1440-1754.1999.00385.x
Cruz AT, 2010, PEDIATR REV, V31, P13, DOI 10.1542/pir.31-1-13
Detjen A K, 2009, Pneumologie, V63, P207, DOI 10.1055/s-0028-1100827
Diel R., GESUNDHEITSWESEN
Diel R, 2019, EUR RESPIR J, V54, DOI 10.1183/13993003.01154-2019
Diel R, 2018, ERJ OPEN RES, V4, DOI 10.1183/23120541.00161-2017
Kohl TA, 2018, EBIOMEDICINE, V34, P131, DOI 10.1016/j.ebiom.2018.07.030
Kohl TA, 2014, J CLIN MICROBIOL, V52, P2479, DOI 10.1128/JCM.00567-14
Mathema B, 2017, J INFECT DIS, V216, pS644, DOI 10.1093/infdis/jix354
Newton SM, 2008, LANCET INFECT DIS, V8, P498, DOI 10.1016/S1473-3099(08)70182-8
RABALAIS G, 1991, LANCET, V338, P825, DOI 10.1016/0140-6736(91)90719-6
Reynolds DL, 2006, INT J TUBERC LUNG D, V10, P1051
Robert Koch-Institut, 2021, BERICHT EPIDEMIOLOGI
Swaminathan S, 2010, CLIN INFECT DIS, V50, pS184, DOI 10.1086/651490
World Health Organization (WHO), 2022, WHO operational handbook on tuberculosis:. module 5: management of tuberculosis in children and adolescents
NR 15
TC 3

Z9 3
U1 0
U2 1
PU W B SAUNDERS CO LTD
PI LONDON
PA 32 JAMESTOWN RD, LONDON NW1 7BY, ENGLAND
SN 0954-6111
EI 1532-3064
J9 RESP MED
JI Respir. Med.
PD APR
PY 2023
VL 209
AR 107152
DI 10.1016/j.rmed.2023.107152
EA FEB 2023
PG 7
WC Cardiac & Cardiovascular Systems; Respiratory System
WE Science Citation Index Expanded (SCI-EXPANDED)
SC Cardiovascular System & Cardiology; Respiratory System
GA 9N3TZ
UT WOS:000942839400001
PM 36781053
DA 2025-05-07
ER

PT J
AU Lea, AJ
   Tung, J
   Archie, EA
   Alberts, SC
AF Lea, Amanda J.
   Tung, Jenny
   Archie, Elizabeth A.
   Alberts, Susan C.
TI Developmental plasticity research in evolution and human health
SO EVOLUTION MEDICINE AND PUBLIC HEALTH
LA English
DT Editorial Material
C1 [Lea, Amanda J.; Tung, Jenny; Alberts, Susan C.] Duke Univ, Dept Biol, Box 90338, Durham, NC 27708 USA.
   [Tung, Jenny; Archie, Elizabeth A.; Alberts, Susan C.] Natl Museums Kenya, Inst Primate Res, Nairobi, Kenya.
   [Tung, Jenny; Alberts, Susan C.] Duke Univ, Populat Res Inst, Durham, NC 27708 USA.
   [Tung, Jenny; Alberts, Susan C.] Duke Univ, Dept Evolutionary Anthropol, Box 90338, Durham, NC 27708 USA.
   [Archie, Elizabeth A.] Univ Notre Dame, Dept Biol Sci, Notre Dame, IN 46556 USA.
   [Lea, Amanda J.] Princeton Univ, Carl Icahn Lab, Lewis Sigler Inst Integrat Genom, Washington Rd, Princeton, NJ 08544 USA.
C3 Duke University; Duke University; Duke University; University of Notre
   Dame; Princeton University

RP Alberts, SC (autor correspondente), Duke Univ, Dept Biol, Box 90338, Durham, NC 27708 USA.; Alberts, SC (autor correspondente), Duke Univ, Dept Evolutionary Anthropol, Box 90338, Durham, NC 27708 USA.
EM alberts@duke.edu
RI Archie, Elizabeth/AAE-7456-2022
FU NIH [P01-AG031719]; NSF [IOS-1456832, R21-AG049936, 1R01GM102562, BCS-1455808]; Leakey Foundation; Triangle Center for Evolutionary Medicine Fellowship
FX This work was supported by NIH P01-AG031719 and NSF IOS-1456832 to S.C.A., R21-AG049936 and 1R01GM102562 to J.T., NSF BCS-1455808 to J.T. and A.J.L., a Leakey Foundation Research Grant to A.J.L. and S.C.A., and
a Triangle Center for Evolutionary Medicine Fellowship to A.J.L.
CR Altmann J, 2003, OFFSPRING, P140
Barrès R, 2012, CELL METAB, V15, P405, DOI 10.1016/j.cmet.2012.01.001
Berghanel A, 2017, P NATL ACAD SCI USA, V114
Boersma M, 1998, AM NAT, V152, P237, DOI 10.1086/286164
Chen E, 2011, MOL PSYCHIATR, V16, P729, DOI 10.1038/mp.2010.53
Chen YY, 2007, J HEALTH ECON, V26, P659, DOI 10.1016/j.jhealeco.2006.12.006
de Rooij SR, 2006, DIABETES CARE, V29, P1897, DOI 10.2337/dc06-0460
Douhard M, 2014, P ROY SOC B-BIOL SCI, V281, DOI 10.1098/rspb.2014.0276
Eriksson A, 2012, P NATL ACAD SCI USA, V109, P16089, DOI 10.1073/pnas.1209494109
Galobardes B, 2006, ANN EPIDEMIOL, V16, P91, DOI 10.1016/j.annepidem.2005.06.053
Gertz J, 2011, PLOS GENET, V7, DOI 10.1371/journal.pgen.1002228
Gluckman P., 2008, Mismatch: the Lifestyle Diseases Timebomb
Gluckman PD, 2008, NEW ENGL J MED, V359, P61, DOI 10.1056/NEJMra0708473
Godfrey KM, 2007, PEDIATR RES, V61, p5R, DOI 10.1203/pdr.0b013e318045bedb
Godfrey KM, 2010, TRENDS ENDOCRIN MET, V21, P199, DOI 10.1016/j.tem.2009.12.008
HALES CN, 1992, DIABETOLOGIA, V35, P595, DOI 10.1093/ije/dyt133
Hales CN, 2001, BRIT MED BULL, V60, P5, DOI 10.1093/bmb/60.1.5
Hayward Adam D., 2013, Evolution Medicine and Public Health, P106, DOI 10.1093/emph/eot007
Hayward AD, 2013, P NATL ACAD SCI USA, V110, P13886, DOI 10.1073/pnas.1301817110
Heard E, 2014, CELL, V157, P95, DOI 10.1016/j.cell.2014.02.045
Heijmans BT, 2008, P NATL ACAD SCI USA, V105, P17046, DOI 10.1073/pnas.0806560105
Henry C.J.K., 1996, LONG TERM CONSEQUENC
Hoddinott J, 2013, MATERN CHILD NUTR, V9, P69, DOI 10.1111/mcn.12080
Jaenisch R, 2003, NAT GENET, V33, P245, DOI 10.1038/ng1089
Jirtle RL, 2007, NAT REV GENET, V8, P253, DOI 10.1038/nrg2045
Jones JH, 2011, CURR BIOL, V21, pR708, DOI 10.1016/j.cub.2011.08.025
Jones JH, 2009, EVOL HUM BEHAV, V30, P305, DOI 10.1016/j.evolhumbehav.2009.01.005
Jones OR, 2008, ECOL LETT, V11, P664, DOI 10.1111/j.1461-0248.2008.01187.x
JONES P, 1994, ARCH GEN PSYCHIAT, V51, P333

Klose RJ, 2006, TRENDS BIOCHEM SCI, V31, P89, DOI 10.1016/j.tibs.2005.12.008
Kuzawa CW, 2012, PLOS ONE, V7, DOI 10.1371/journal.pone.0040905
Laforsch C, 2006, LIMNOL OCEANOGR, V51, P1466, DOI 10.4319/lo.2006.51.3.1466
Lea AJ, 2015, AM NAT, V185, P809, DOI 10.1086/681016
Lindström J, 1999, TRENDS ECOL EVOL, V14, P343, DOI 10.1016/S0169-5347(99)01639-0
Martin RD, 1996, NEWS PHYSIOL SCI, V11, P149, DOI 10.1152/physiologyonline.1996.11.4.149
Martin RD, 2007, YEARB PHYS ANTHROPOL, V50, P59, DOI 10.1002/ajpa.20734
MCDONALD DB, 1993, BEHAV ECOL, V4, P297, DOI 10.1093/beheco/4.4.297
Miller CA, 2007, NEURON, V53, P857, DOI 10.1016/j.neuron.2007.02.022
Monaghan P, 2008, PHILOS T R SOC B, V363, P1635, DOI 10.1098/rstb.2007.0011
Mumby HS, 2015, SCI REP-UK, V5, DOI 10.1038/srep13946
NEEL JV, 1962, AM J HUM GENET, V14, P353
Netea MG, 2016, SCIENCE, V352, DOI 10.1126/science.aaf1098
Nettle D, 2015, P ROY SOC B-BIOL SCI, V282, P23, DOI 10.1098/rspb.2015.1005
Nussey DH, 2007, CURR BIOL, V17, pR1000, DOI 10.1016/j.cub.2007.10.005
Pacis A, 2015, GENOME RES, V25, P1801, DOI 10.1101/gr.192005.115
Pesonen AK, 2012, PHYSIOL BEHAV, V106, P722, DOI 10.1016/j.physbeh.2011.10.030
Pigeon G, 2017, P ROY SOC B-BIOL SCI, V284, DOI 10.1098/rspb.2017.0222
Rickard IJ, 2012, P ROY SOC B-BIOL SCI, V279, P4253, DOI 10.1098/rspb.2012.1363
Roseboom T, 2006, EARLY HUM DEV, V82, P485, DOI 10.1016/j.earlhumdev.2006.07.001
Sih A, 2011, EVOL APPL, V4, P367, DOI 10.1111/j.1752-4571.2010.00166.x
Sih A, 2010, OIKOS, V119, P610, DOI 10.1111/j.1600-0706.2009.18039.x
Smith GD, 1998, BMJ-BRIT MED J, V316, P1631, DOI 10.1136/bmj.316.7145.1631
Solís CB, 2015, P NATL ACAD SCI USA, V112, pE738, DOI 10.1073/pnas.1417325112
Suzuki MM, 2008, NAT REV GENET, V9, P465, DOI 10.1038/nrg2341
Tobi EW, 2014, NAT COMMUN, V5, DOI 10.1038/ncomms6592
Tobi EW, 2009, HUM MOL GENET, V18, P4046, DOI 10.1093/hmg/ddp353
Tung J, 2012, P NATL ACAD SCI USA, V109, P6490, DOI 10.1073/pnas.1202734109
Uller T, 2013, J EVOLUTION BIOL, V26, P2161, DOI 10.1111/jeb.12212
Wachs TD, 2014, ANN NY ACAD SCI, V1308, P89, DOI 10.1111/nyas.12314
Walton A, 1938, PROC R SOC SER B-BIO, V125, P311, DOI 10.1098/rspb.1938.0029
Wells Jonathan C. K., 2014, Evolution Medicine and Public Health, P109, DOI 10.1093/emph/eou019
NR 61
TC 5
Z9 7
U1 1
U2 3
PU OXFORD UNIV PRESS
PI OXFORD

PA GREAT CLARENDON ST, OXFORD OX2 6DP, ENGLAND
SN 2050-6201
J9 EVOL MED PUBLIC HLTH
JI Evol. Med. Public Health.
PY 2017
IS 1
BP 201
EP 205
DI 10.1093/emph/eoy007
PG 5
WC Evolutionary Biology; Public, Environmental & Occupational Health
WE Science Citation Index Expanded (SCI-EXPANDED)
SC Evolutionary Biology; Public, Environmental & Occupational Health
GA GC3AB
UT WOS:000429654800001
PM 29645009
OA gold, Green Submitted, Green Published
DA 2025-05-07
ER

PT J
AU Merker, M
   Rasigade, JP
   Barbier, M
   Cox, H
   Feuerriegel, S
   Kohl, TA
   Shitikov, E
   Klaos, K
   Gaudin, C
   Antoine, R
   Diel, R
   Borrell, S
   Gagneux, S
   Nikolayevskyy, V
   Andres, S
   Crudu, V
   Supply, P
   Niemann, S
   Wirth, T
AF Merker, Matthias
   Rasigade, Jean-Philippe
   Barbier, Maxime
   Cox, Helen
   Feuerriegel, Silke
   Kohl, Thomas A.
   Shitikov, Egor
   Klaos, Kadri
   Gaudin, Cyril
   Antoine, Rudy
   Diel, Roland
   Borrell, Sonia
   Gagneux, Sebastien
   Nikolayevskyy, Vladyslav

Andres, Soenke
Crudu, Valeriu
Supply, Philip
Niemann, Stefan
Wirth, Thierry
TI Transcontinental spread and evolution of Mycobacterium tuberculosis W148 European/Russian Glade toward extensively drug resistant tuberculosis
SO NATURE COMMUNICATIONS
LA English
DT Article
ID NEWLY INDEPENDENT STATES; COMPENSATORY MUTATIONS; TRANSMISSION; EMERGENCE; HISTORY; EPIDEMICS; VIRULENCE
AB Transmission-driven multi-/extensively drug resistant (M/XDR) tuberculosis (TB) is the largest single contributor to human mortality due to antimicrobial resistance. A few major clades of the Mycobacterium tuberculosis complex belonging to lineage 2, responsible for high prevalence of MDR-TB in Eurasia, show outstanding transnational distributions. Here, we determined factors underlying the emergence and epidemic spread of the W148 Glade by genome sequencing and Bayesian demogenetic analyses of 720 isolates from 23 countries. We dated a common ancestor around 1963 and identified two successive epidemic expansions in the late 1980s and late 1990s, coinciding with major socio-economic changes in the post-Soviet Era. These population expansions favored accumulation of resistance mutations to up to 11 anti-TB drugs, with MDR evolving toward additional resistances to fluoroquinolones and second-line injectable drugs within 20 years on average. Timescaled haplotypic density analysis revealed that widespread acquisition of compensatory mutations was associated with transmission success of XDR strains. Virtually all W148 strains harbored a hypervirulence-associated ppe38 gene locus, and incipient recurrent emergence of prpR mutation-mediated drug tolerance was detected. The outstanding genetic arsenal of this geographically widespread M/XDR strain Glade represents a "perfect storm" that jeopardizes the successful introduction of new anti-M/XDR-TB antibiotic regimens.
C1 [Merker, Matthias; Feuerriegel, Silke; Kohl, Thomas A.; Niemann, Stefan] Res Ctr Borstel, Mol & Expt Mycobacteriol, Borstel, Germany.
[Merker, Matthias; Feuerriegel, Silke; Kohl, Thomas A.] German Ctr Infect Res, Partner Site Hamburg Lubeck Borstel Riems, Borstel, Germany.
[Merker, Matthias] Res Ctr Borstel, Evolut Resistome, Borstel, Germany.
[Rasigade, Jean-Philippe; Barbier, Maxime; Wirth, Thierry] PSL Univ, EPHE, Paris, France.
[Rasigade, Jean-Philippe; Barbier, Maxime; Wirth, Thierry] Sorbonne Univ, Univ Antilles, EPHE,Museum Natl Hist Nat, CNRS,Inst Systemat,Evolut,Biodiversite,ISYEB, Paris, France.
[Rasigade, Jean-Philippe] Univ Lyon 1, ENS Lyon, Ctr Int Rech Infectiol, INSERM U1111,CNRS UMR5308, Lyon, France.
[Cox, Helen] Univ Cape Town, Div Med Microbiol, Cape Town, South Africa.
[Cox, Helen] Univ Cape Town, Inst Infect Dis & Mol Med, Cape Town, South Africa.
[Shitikov, Egor] Fed Res & Clin Ctr Phys Chem Med, Moscow, Russia.
[Klaos, Kadri] SA TUH United Labs, Mycobacteriol, Tartu, Estonia.

[Gaudin, Cyril] Genoscreen, Lille, France.
[Antoine, Rudy; Supply, Philip] Univ Lille, Inst Pasteur Lille, CHU Lille,CNRS,Inserm, U1019,UMR 9017,CIIL Ctr Infect & Immunite Lille, F-59000 Lille, France.
[Diel, Roland] Schleswig Holstein Univ Hosp, Inst Epidemiol, Kiel, Germany.
[Diel, Roland] German Ctr Lung Res DZL, Airway Res Ctr North ARCN, Lung Clin Grosshansdorf, D-22927 Grosshansdorf, Germany.
[Borrell, Sonia; Gagneux, Sebastien] Swiss Trop & Publ Hlth Inst, Allschwil, Switzerland.
[Borrell, Sonia; Gagneux, Sebastien] Univ Basel, Basel, Switzerland.
[Nikolayevskyy, Vladyslav] Imperial Coll London, London, England.
[Andres, Soenke] Res Ctr Borstel, Natl & Who Supranatl Reference Ctr Mycobacteria, Borstel, Germany.
[Crudu, Valeriu] Inst Phthisiopneumol, Natl TB Reference Lab, Kishinev, Moldova.
C3 Leibniz Association; Forschungszentrum Borstel; German Center for Infection Research; Leibniz Association; Forschungszentrum Borstel; Universite PSL; Ecole Pratique des Hautes Etudes (EPHE); Universite PSL;
Ecole Pratique des Hautes Etudes (EPHE); Museum National d'Histoire Naturelle (MNHN); Sorbonne Universite; Centre National de la Recherche Scientifique (CNRS); Centre National de la Recherche Scientifique (CNRS); CNRS - National Institute for Biology (INSB); Ecole Normale Superieure de Lyon (ENS de LYON); Universite Claude Bernard Lyon 1; Institut National de la Sante et de la Recherche Medicale (Inserm); University of Cape Town; University of Cape Town; Lopukhin Federal Research & Clinical Center of Physical-Chemical Medicine of Federal Medical Biological Agency; Institut National de la Sante et de la Recherche Medicale (Inserm); Centre National de la Recherche Scientifique (CNRS); Pasteur Network; Universite de Lille; Institut Pasteur Lille; CHU Lille; University of Kiel; Schleswig Holstein University Hospital; University of Basel; Swiss Tropical & Public Health
Institute; University of Basel; Imperial College London; Leibniz Association; Forschungszentrum Borstel
RP Niemann, S (autor correspondente), Res Ctr Borstel, Mol & Expt Mycobacteriol, Borstel, Germany.; Wirth, T (autor correspondente), PSL Univ, EPHE, Paris, France.; Wirth, T (autor correspondente), Sorbonne Univ, Univ Antilles, EPHE,Museum Natl Hist Nat, CNRS,Inst Systemat,Evolut,Biodiversite,ISYEB, Paris, France.; Supply, P (autor correspondente), Univ Lille, Inst Pasteur Lille, CHU Lille,CNRS,Inserm, U1019,UMR 9017,CIIL Ctr Infect & Immunite Lille, F-59000 Lille, France.
EM philip.supply@ibl.cnrs.fr; sniemann@fz-borstel.de; wirth@mnhn.fr
RI Nikolayevskyy, Vladyslav/HFZ-7738-2022; Kohl, Thomas/AAB-9782-2020; Cox,
Helen/ADS-6247-2022; Antoine, Rudy/CAH-6653-2022; Gagneux, Sebastien/ABE-7909-2020; Gaudin, Christophe/F-4576-2012; Niemann, Stefan/C-9327-2011; Rasigade, Jean-Philippe/AAE-1667-2021; Antoine, Rudy/L-1630-2018; Merker, Matthias/P-7399-2015; Supply, Philip/A-9580-2019; Wirth, Thierry/B-4915-2008; Shitikov, Egor/Q-2520-2017
OI Gagneux, Sebastien/0000-0001-7783-9048; Niemann,

Stefan/0000-0002-6604-0684; Gaudin, Cyril/0009-0002-1833-1193; Antoine,
Rudy/0000-0002-7369-0626; Merker, Matthias/0000-0003-1386-2331; Supply,
Philip/0000-0003-3690-3853; Wirth, Thierry/0000-0002-7371-0172; Cox,
Helen/0000-0002-6538-7192; Shitikov, Egor/0000-0002-4865-6004; Klaos,
Kadri/0000-0002-0055-755X
FU German Ministry of Education and Research (BMBF); Leibniz Science Campus
of Evolutionary Medicine of the Lung (Evolung)
FX We thank the Institut Convergences Migrations for travel grants to T.W.
Parts of the work were funded by the German Ministry of Education and
Research (BMBF) for the German Center of Infection Research (DZIF) and
the Leibniz Science Campus of Evolutionary Medicine of the Lung
(Evolung). We would also like to thank Ulrich Nubel, Qin Yi and Emilie
Rousseau for constructive comments and help on the paper.
CR Anderson MJ, 2001, AUST NZ J STAT, V43, P75, DOI 10.1111/1467-842X.00156
Antoine R, 2021, MICROBIOL SPECTR, V9, DOI 10.1128/Spectrum.00019-21
Ates LS, 2018, NAT MICROBIOL, V3, P181, DOI 10.1038/s41564-017-0090-6
Barbier M, 2018, SCI REP-UK, V8, DOI 10.1038/s41598-018-24034-6
Battaglia S, 2020, J CLIN MICROBIOL, V58, DOI 10.1128/JCM.01304-20
Bifani PJ, 2002, TRENDS MICROBIOL, V10, P45, DOI 10.1016/S0966-842X(01)02277-6
Bouckaert R, 2014, PLOS COMPUT BIOL, V10, DOI 10.1371/journal.pcbi.1003537
Brandis G, 2012, MOL MICROBIOL, V85, P142, DOI 10.1111/j.1365-2958.2012.08099.x
Brown TS, 2019, P NATL ACAD SCI USA, V116, P23284, DOI 10.1073/pnas.1906636116
Brynildsrud OB, 2018, SCI ADV, V4, DOI 10.1126/sciadv.aat5869
Casali N, 2014, NAT GENET, V46, P279, DOI 10.1038/ng.2878
Cohen KA, 2015, PLOS MED, V12, DOI 10.1371/journal.pmed.1001880
Coll F, 2018, NAT GENET, V50, P307, DOI 10.1038/s41588-017-0029-0
Cooke GS, 2011, EMERG INFECT DIS, V17, P2035, DOI 10.3201/eid1711.110291
Crispell J, 2019, MICROB GENOMICS, V5, DOI 10.1099/mgen.0.000245
Dheda K, 2017, LANCET RESP MED, V5, P291, DOI [10.1016/S2213-2600(17)30079-6, 10.1016/s2213-2600(17)30079-6]
Eldholm V, 2016, P NATL ACAD SCI USA, V113, P13881, DOI 10.1073/pnas.1611283113
Eldholm V, 2015, NAT COMMUN, V6, DOI 10.1038/ncomms8119
Farhat MR, 2019, NAT COMMUN, V10, DOI 10.1038/s41467-019-10110-6
Ford CB, 2013, NAT GENET, V45, P784, DOI 10.1038/ng.2656
Guindon S, 2010, SYST BIOL, V59, P307, DOI 10.1093/sysbio/syq010
Gygli SM, 2021, NAT MED, V27, P1171, DOI 10.1038/s41591-021-01358-x
Handel A, 2006, PLOS COMPUT BIOL, V2, P1262, DOI 10.1371/journal.pcbi.0020137
Hardy IRB, 1996, LANCET, V347, P1739, DOI 10.1016/S0140-6736(96)90811-9
Hicks ND, 2020, PLOS PATHOG, V16, DOI 10.1371/journal.ppat.1009063
Hicks ND, 2018, NAT MICROBIOL, V3, P1032, DOI 10.1038/s41564-018-0218-3

Kadura S, 2020, J ANTIMICROB CHEMOTH, V75, P2031, DOI 10.1093/jac/dkaa136
Kendall EA, 2015, LANCET RESP MED, V3, P963, DOI 10.1016/S2213-2600(15)00458-0
Kohl TA, 2018, PEERJ, V6, DOI 10.7717/peerj.5895
Lange C, 2018, J INTERN MED, V284, P163, DOI 10.1111/joim.12780
Langmead B, 2012, NAT METHODS, V9, P357, DOI [10.1038/NMETH.1923, 10.1038/nmeth.1923]
Letunic I, 2016, NUCLEIC ACIDS RES, V44, pW242, DOI 10.1093/nar/gkw290
Li QJ, 2016, ANTIMICROB AGENTS CH, V60, P2807, DOI 10.1128/AAC.02358-15
McEvoy CRE, 2009, BMC EVOL BIOL, V9, DOI 10.1186/1471-2148-9-237
Merker M, 2020, GENOME MED, V12, DOI 10.1186/s13073-020-00726-5
Merker M, 2018, ELIFE, V7, DOI 10.7554/eLife.38200
Merker M, 2015, NAT GENET, V47, P242, DOI 10.1038/ng.3195
Mokrousov I, 2013, CLIN MICROBIOL REV, V26, P342, DOI 10.1128/CMR.00087-12
Moura A, 2021, SCI ADV, V7, DOI 10.1126/sciadv.abj9805
Paradis E, 2004, BIOINFORMATICS, V20, P289, DOI [10.1093/bioinformatics/btg412, 10.1093/bioinformatics/bty633]
Paradis E, 2010, BIOINFORMATICS, V26, P419, DOI 10.1093/bioinformatics/btp696
Parish T, 2003, INFECT IMMUN, V71, P1134, DOI 10.1128/IAI.71.3.1134-1140.2003
Price AL, 2006, NAT GENET, V38, P904, DOI 10.1038/ng1847
Rambaut A, 2016, VIRUS EVOL, V2, DOI 10.1093/ve/vew007
Rasigade JP, 2017, SCI REP-UK, V7, DOI 10.1038/srep45326
Renton AM, 1998, SEX TRANSM INFECT, V74, P165, DOI 10.1136/sti.74.3.165
Rieux A, 2017, MOL ECOL RESOUR, V17, P608, DOI 10.1111/1755-0998.12603
Sardesai AA, 2001, J BACTERIOL, V183, P86, DOI 10.1128/JB.183.1.86-93.2001
Shitikov EA, 2014, PLOS ONE, V9, DOI 10.1371/journal.pone.0084971
Skrahina A, 2013, B WORLD HEALTH ORGAN, V91, P36, DOI 10.2471/BLT.12.104588
Stucki D, 2016, NAT GENET, V48, P1535, DOI 10.1038/ng.3704
Thawornwattana Y, 2021, MICROB GENOMICS, V7, DOI 10.1099/mgen.0.000697
Wirth T, 2021, NAT MICROBIOL, V6, P1215, DOI 10.1038/s41564-021-00967-z
Wirth T, 2020, EVOL APPL, V13, P1513, DOI 10.1111/eva.12991
Wirth T, 2020, NAT MICROBIOL, V5, P735, DOI 10.1038/s41564-020-0676-2
Wirth T, 2015, NAT GENET, V47, P565, DOI 10.1038/ng.3318
Yablonskii PK, 2015, AM J RESP CRIT CARE, V191, P372, DOI 10.1164/rccm.201305-0926OE
Zhang HT, 2013, NAT GENET, V45, P1255, DOI 10.1038/ng.2735
NR 58
TC 37
Z9 37
U1 2
U2 12
PU NATURE PORTFOLIO
PI BERLIN
PA HEIDELBERGER PLATZ 3, BERLIN, 14197, GERMANY
EI 2041-1723

J9 NAT COMMUN
JI Nat. Commun.
PD AUG 30
PY 2022
VL 13
IS 1
AR 5105
DI 10.1038/s41467-022-32455-1
PG 11
WC Multidisciplinary Sciences
WE Science Citation Index Expanded (SCI-EXPANDED)
SC Science & Technology - Other Topics
GA 4E4LE
UT WOS:000847797500017
PM 36042200
OA Green Published, gold, Green Accepted
DA 2025-05-07
ER

PT J
AU Feuerriegel, S
   Kohl, TA
   Utpatel, C
   Andres, S
   Maurer, FP
   Heyckendorf, J
   Jouet, A
   Badalato, N
   Foray, L
   Kamara, RF
   Conteh, OS
   Supply, P
   Niemann, S
AF Feuerriegel, Silke
   Kohl, Thomas A.
   Utpatel, Christian
   Andres, Soenke
   Maurer, Florian P.
   Heyckendorf, Jan
   Jouet, Agathe
   Badalato, Nelly
   Foray, Lynda
   Kamara, Rashidatu Fouad
   Conteh, Osman S.
   Supply, Philip
   Niemann, Stefan
TI Rapid genomic first- and second-line drug resistance prediction from
   clinical Mycobacterium tuberculosis specimens using Deeplex-MycTB
SO EUROPEAN RESPIRATORY JOURNAL
LA English
DT Letter
ID SUSCEPTIBILITY

C1 [Feuerriegel, Silke; Kohl, Thomas A.; Utpatel, Christian; Niemann, Stefan] Res Ctr Borstel, Mol & Expt Mycobacteriol, D-23845 Borstel, Germany.
[Feuerriegel, Silke; Kohl, Thomas A.; Utpatel, Christian; Heyckendorf, Jan; Niemann, Stefan] German Ctr Infect Res DZIF, Partner Site Hamburg Lubeck Borstel, Borstel, Germany.
[Andres, Soenke; Maurer, Florian P.] Res Ctr Borstel, Natl & WHO Supranat Reference Ctr Mycobacteria, Borstel, Germany.
[Maurer, Florian P.] Univ Med Ctr Hamburg Eppendorf, Inst Med Microbiol Virol & Hyg, Hamburg, Germany.
[Heyckendorf, Jan] Res Ctr Borstel, Clin Infect Dis, Borstel, Germany.
[Heyckendorf, Jan] Univ Lubeck, Int Hlth Infect Dis, Lubeck, Germany.
[Jouet, Agathe; Badalato, Nelly] Campus Inst Pasteur Lille, Genoscreen, Lille, France.
[Foray, Lynda; Kamara, Rashidatu Fouad] Minist Hlth & Sanitat, Natl TB Control Programme, Freetown, Sierra Leone.
[Conteh, Osman S.] Lakka Govt Hosp, Natl Reference Lab, Freetown, Sierra Leone.
[Supply, Philip] Univ Lille, CNRS, INSERM, CHU Lille,Inst Pasteur Lille,U1019 UMR 9017 CIIL, Lille, France.
C3 Leibniz Association; Forschungszentrum Borstel; German Center for Infection Research; Leibniz Association; Forschungszentrum Borstel; University of Hamburg; University Medical Center Hamburg-Eppendorf; Leibniz Association; Forschungszentrum Borstel; University of Lubeck; Ministry of Health & Sanitation Sierra Leone; Centre National de la Recherche Scientifique (CNRS); Institut National de la Sante et de la Recherche Medicale (Inserm); Universite de Lille; Pasteur Network; Institut Pasteur Lille; CHU Lille
RP Niemann, S (autor correspondente), Res Ctr Borstel, Mol & Expt Mycobacteriol, D-23845 Borstel, Germany.
EM sniemann@fz-borstel.de
RI Niemann, Stefan/C-9327-2011; Maurer, Florian/ABA-1918-2020; Heyckendorf,
Jan/AAA-8782-2021; Kohl, Thomas Andreas/AAB-9782-2020; Maurer, Florian/J-9687-2014; Supply, Philip/A-9580-2019
OI Kohl, Thomas Andreas/0000-0002-1126-6803; Maurer, Florian/0000-0001-8353-5964; Supply, Philip/0000-0003-3690-3853; Niemann, Stefan/0000-0002-6604-0684; Jouet, Agathe/0000-0003-4998-9596;
Badalato, Nelly/0000-0002-1994-5894
FU European Commission [FP7-278864]; Deutsches Zentrum fur Infektionsforschung; Joachim Herz Stiftung; Leibniz Science Campus Evolutionary Medicine of the LUNG; Deutsche Forschungsgemeinschaft [EXC
216]
FX This work was supported by the European Commission (grant: FP7-278864),
Deutsches Zentrum fur Infektionsforschung, Joachim Herz Stiftung, Leibniz Science Campus Evolutionary Medicine of the LUNG and Deutsche Forschungsgemeinschaft (grant: Precision Medicine in Chronic Inflammation EXC 216). Funding information for this article has been deposited with the Crossref Funder Registry.
CR Andres S, 2020, AM J RESP CRIT CARE, V201, P1564, DOI 10.1164/rccm.201909-1819LE

   Brown AC, 2015, J CLIN MICROBIOL, V53, P2230, DOI 10.1128/JCM.00486-15
   Dheda K, 2017, LANCET RESP MED, V5, P291, DOI [10.1016/S2213-2600(17)30079-6, 10.1016/s2213-2600(17)30079-6]
   Di Angelantonio E, 2019, LANCET GLOB HEALTH, V7, pE1332, DOI 10.1016/S2214-109X(19)30318-3
   Feuerriegel Silke, 2012, BMC Microbiol, V12, P90, DOI 10.1186/1471-2180-12-90
   Kandler JL, 2018, ANTIMICROB AGENTS CH, V62, DOI [10.1128/AAC.00974-18, 10.1128/aac.00974-18]
   Köser CU, 2013, NEW ENGL J MED, V369, P290, DOI 10.1056/NEJMc1215305
   Kohl TA, 2018, PEERJ, V6, DOI 10.7717/peerj.5895
   Makhado NA, 2018, LANCET INFECT DIS, V18, P1350, DOI 10.1016/S1473-3099(18)30496-1
   Meehan CJ, 2019, NAT REV MICROBIOL, V17, P533, DOI 10.1038/s41579-019-0214-5
   Miotto P, 2017, EUR RESPIR J, V50, DOI 10.1183/13993003.01354-2017
   Nikam C, 2016, INT J TUBERC LUNG D, V20, P154, DOI 10.5588/ijtld.15.0319
   Rigouts L, 2019, SCI REP-UK, V9, DOI 10.1038/s41598-019-48289-9
   Rigouts L, 2013, J CLIN MICROBIOL, V51, P2641, DOI 10.1128/JCM.02741-12
   Sirgel FA, 2012, J ANTIMICROB CHEMOTH, V67, P1088, DOI 10.1093/jac/dks033
   Tagliani E, 2017, SCI REP-UK, V7, DOI 10.1038/s41598-017-17705-3
   Walker TM, 2015, LANCET INFECT DIS, V15, P1193, DOI 10.1016/S1473-3099(15)00062-6
NR 17
TC 48
Z9 51
U1 0
U2 13
PU EUROPEAN RESPIRATORY SOC JOURNALS LTD
PI SHEFFIELD
PA 442 GLOSSOP RD, SHEFFIELD S10 2PX, ENGLAND
SN 0903-1936
EI 1399-3003
J9 EUR RESPIR J
JI Eur. Resp. J.
PD JAN 1
PY 2021
VL 57
IS 1
AR 2001796
DI 10.1183/13993003.01796-2020
PG 4
WC Respiratory System
WE Science Citation Index Expanded (SCI-EXPANDED)
SC Respiratory System
GA PT1UY
UT WOS:000608406300004
PM 32764113
OA Green Published, Bronze
DA 2025-05-07
ER

PT J
AU Hao, Y
   Lee, HJ
   Baraboo, M
   Burch, K
   Maurer, T
   Somarelli, JA
   Conant, GC
AF Hao, Yue
   Lee, Hyuk Jin
   Baraboo, Michael
   Burch, Katherine
   Maurer, Taylor
   Somarelli, Jason A.
   Conant, Gavin C.
TI Baby Genomics: Tracing the Evolutionary Changes That Gave Rise to
   Placentation
SO GENOME BIOLOGY AND EVOLUTION
LA English
DT Article
DE comparative genomics; orthology inference; mammalian pregnancy;
   placental mammals
ID R PACKAGE; TRANSPOSABLE ELEMENTS; HIERARCHICAL CATALOG; SEMANTIC
   SIMILARITY; IMMUNE MODULATION; PREGNANCY; GENES; CANCER; FAMILY; CELLS
AB It has long been challenging to uncover the molecular mechanisms behind striking morphological innovations such as mammalian pregnancy. We studied the power of a robust comparative orthology pipeline based on gene synteny to address such problems. We inferred orthology relations between human genes and genes from each of 43 other vertebrate genomes, resulting in similar to 18,000 orthologous pairs for each genome comparison. By identifying genes that first appear coincident with origin of the placental mammals, we hypothesized that we would define a subset of the genome enriched for genes that played a role in placental evolution. We thus pinpointed orthologs that appeared before and after the divergence of eutherian mammals from marsupials. Reinforcing previous work, we found instead that much of the genetic toolkit of mammalian pregnancy evolved through the repurposing of preexisting genes to new roles. These genes acquired regulatory controls for their novel roles from a group of regulatory genes, many of which did in fact originate at the appearance of the eutherians. Thus, orthologs appearing at the origin of the eutherians are enriched in functions such as transcriptional regulation by Kruppel-associated box-zinc-finger proteins, innate immune responses, keratinization, and the melanoma-associated antigen protein class. Because the cellular mechanisms of invasive placentae are similar to those of metastatic cancers, we then used our orthology inferences to explore the association between placenta invasion and cancer metastasis. Again echoing previous work, we find that genes that are phylogenetically older are more likely to be implicated in cancer development.
C1 [Hao, Yue; Conant, Gavin C.] North Carolina State Univ, Bioinformat Res Ctr, Raleigh, NC 27695 USA.
   [Lee, Hyuk Jin] Univ Missouri Columbia, Div Biol Sci, Columbia, MO USA.

[Baraboo, Michael] Truman State Univ, Dept Comp Sci, Kirksville, MO USA.
[Burch, Katherine] Truman State Univ, Dept Psychol, Kirksville, MO USA.
[Maurer, Taylor] Kenyon Coll, Gambier, OH 43022 USA.
[Somarelli, Jason A.] Duke Univ, Med Ctr, Duke Canc Inst, Durham, NC 27706 USA.
[Somarelli, Jason A.] Duke Univ, Sch Med, Dept Med, Durham, NC 27706 USA.
[Conant, Gavin C.] Univ Missouri Columbia, Div Anim Sci, Columbia, MO USA.
[Conant, Gavin C.] North Carolina State Univ, Program Genet, Raleigh, NC USA.
[Conant, Gavin C.] North Carolina State Univ, Dept Biol Sci, Raleigh, NC USA.
C3 North Carolina State University; University of Missouri System; University of Missouri Columbia; University System of Ohio; Kenyon College; Duke University; Duke University; University of Missouri System; University of Missouri Columbia; North Carolina State University; North Carolina State University
RP Hao, Y (autor correspondente), North Carolina State Univ, Bioinformat Res Ctr, Raleigh, NC 27695 USA.
EM yhao7@ncsu.edu
RI Conant, Gavin/R-7022-2019; Hoffmann, Federico/B-7134-2009; Hao, Yue/G-8856-2019
OI Hoffmann, Federico/0000-0001-5056-4929; Hao, Yue/0000-0002-5065-8906; Maurer, Taylor (Maggie)/0009-0002-2330-7980; Conant, Gavin/0000-0002-8677-4933; Somarelli, Jason/0000-0003-1510-9343
FU National Science Foundation [NSF-IOS-1339156, NSF-DBI-1358997, NSF-CCF-1421765]; European Union [613689]; Triangle Comparative and Evolutionary Medicine Center
FX We would like to thank V. Lynch, J.C. Pires, M. Lucy, T. Spencer, and J.
Thorne for helpful conversations and H. Truong for computational advice.
This work was supported by National Science Foundation Grants NSF-IOS-1339156 (Y.H. and G.C.C.), NSF-DBI-1358997 (M.B., H.J.L., K.B.,
T.M., and G.C.C.), NSF-CCF-1421765 (G.C.C.), European Union's Seventh Framework Programme Grant 613689 (Y.H. and G.C.C.), and by the Triangle
Comparative and Evolutionary Medicine Center (Y.H.).
CR [Anonymous], VERTEBRATE FETALMEMB
[Anonymous], 2003, GENOME BIOL
[Anonymous], 2018, GGRIDGES RIDGELINE P
[Anonymous], 2019, Genome wide annotation for Human
[Anonymous], OBSTET GYNECOLOGY IN
Azuaje F., 2005, P ISMB 2005 SIG M BI, V2005, P9
Bekaert M, 2014, PLOS ONE, V9, DOI 10.1371/journal.pone.0087115
Bekaert M, 2011, MOL BIOL EVOL, V28, P1111, DOI 10.1093/molbev/msq296
BENJAMINI Y, 1995, J R STAT SOC B, V57, P289, DOI 10.1111/j.2517-6161.1995.tb02031.x
Brandt J, 2005, CYTOGENET GENOME RES, V110, P307, DOI 10.1159/000084963

Chavan AR, 2016, PLACENTA, V40, P40, DOI 10.1016/j.placenta.2016.02.012
Chomez P, 2001, CANCER RES, V61, P5544
Churakov G, 2009, GENOME RES, V19, P868, DOI 10.1101/gr.090647.108
Conant GC, 2008, NAT REV GENET, V9, P938, DOI 10.1038/nrg2482
Conant GC, 2009, TRENDS GENET, V25, P377, DOI 10.1016/j.tig.2009.07.004
Conant GC, 2002, NUCLEIC ACIDS RES, V30, P3378, DOI 10.1093/nar/gkf449
Cosby RL, 2019, GENE DEV, V33, P1098, DOI 10.1101/gad.327312.119
Costanzo V, 2018, OPEN BIOL, V8, DOI 10.1098/rsob.180081
Cuman C, 2014, REPRODUCTION, V147, pR75, DOI 10.1530/REP-13-0474
D'Souza Alaric W., 2014, Evolution Medicine and Public Health, P136, DOI 10.1093/emph/eou022
Domazet-Loso T, 2008, MOL BIOL EVOL, V25, P2699, DOI 10.1093/molbev/msn214
dos Reis M, 2012, P ROY SOC B-BIOL SCI, V279, P3491, DOI 10.1098/rspb.2012.0683
Dunwell TL, 2017, P ROY SOC B-BIOL SCI, V284, DOI 10.1098/rspb.2017.1357
Ecco G, 2016, DEV CELL, V36, P611, DOI 10.1016/j.devcel.2016.02.024
Emera D, 2012, BRIEF FUNCT GENOMICS, V11, P267, DOI 10.1093/bfgp/els013
Faas MM, 2018, PLACENTA, V69, P125, DOI 10.1016/j.placenta.2018.04.012
Faas MM, 2017, PLACENTA, V56, P44, DOI 10.1016/j.placenta.2017.03.001
Ferretti C, 2007, HUM REPROD UPDATE, V13, P121, DOI 10.1093/humupd/dml048
Forbes SA, 2017, NUCLEIC ACIDS RES, V45, pD777, DOI 10.1093/nar/gkw1121
Gould S.J., 1980, The Pandas Thumb: More Reflections in Natural History, P19
Guernsey MW, 2017, ELIFE, V6, DOI 10.7554/eLife.27450
Hanahan D, 2011, CELL, V144, P646, DOI 10.1016/j.cell.2011.02.013
Hedges SB, 2015, MOL BIOL EVOL, V32, P835, DOI 10.1093/molbev/msv037
Holtan SG, 2009, MAYO CLIN PROC, V84, P985, DOI 10.1016/S0025-6196(11)60669-1
Huang DW, 2007, GENOME BIOL, V8, DOI 10.1186/gb-2007-8-9-r183
Huang DW, 2009, NAT PROTOC, V4, P44, DOI 10.1038/nprot.2008.211
Huntley S, 2006, GENOME RES, V16, P669, DOI 10.1101/gr.4842106
Kim M, 2016, NUCLEIC ACIDS RES, V44, pD908, DOI 10.1093/nar/gkv1137
Knowles DG, 2009, GENOME RES, V19, P1752, DOI 10.1101/gr.095026.109
Knox K, 2008, GENOME RES, V18, P695, DOI 10.1101/gr.071407.107
Koonin EV, 2005, ANNU REV GENET, V39, P309, DOI 10.1146/annurev.genet.39.073003.114725
Koonin EV, 2003, SEQUENCE EVOLUTION F, DOI DOI 10.1007/978-1-4757-3783-7
Kriegs JO, 2006, PLOS BIOL, V4, P537, DOI 10.1371/journal.pbio.0040091
Kristensen DM, 2011, BRIEF BIOINFORM, V12, P379, DOI 10.1093/bib/bbr030
Kriventseva EV, 2008, NUCLEIC ACIDS RES, V36, pD271, DOI 10.1093/nar/gkm845
Kriventseva EV, 2015, NUCLEIC ACIDS RES, V43, pD250, DOI 10.1093/nar/gku1220
Kumar S, 2017, MOL BIOL EVOL, V34, P1812, DOI 10.1093/molbev/msx116
Lee A, 2013, PHILOS T R SOC B, V368, DOI 10.1098/rstb.2012.0503

Lefèvre CM, 2010, ANNU REV GENOM HUM G, V11, P219, DOI 10.1146/annurev-genom-082509-141806
Liu H, 2014, GENOME BIOL EVOL, V6, P510, DOI 10.1093/gbe/evu030
Long MY, 2013, ANNU REV GENET, V47, P307, DOI 10.1146/annurev-genet-111212-133301
Lynch VJ, 2015, CELL REP, V10, P551, DOI 10.1016/j.celrep.2014.12.052
Lynch VJ, 2011, NAT GENET, V43, P1154, DOI 10.1038/ng.917
MANN HB, 1947, ANN MATH STAT, V18, P50, DOI 10.1214/aoms/1177730491
Meredith RW, 2011, SCIENCE, V334, P521, DOI 10.1126/science.1211028
Mi HY, 2019, NUCLEIC ACIDS RES, V47, pD419, DOI 10.1093/nar/gky1038
Mincheva-Nilsson L, 2014, AM J REPROD IMMUNOL, V72, P440, DOI 10.1111/aji.12311
Mossman HW, 1937, CONTRIB EMBRYOL, V26, P133
Nejadmoghaddam MR, 2017, SCI REP-UK, V7, DOI 10.1038/s41598-017-13682-9
Niimura Y, 2014, GENOME RES, V24, P1485, DOI 10.1101/gr.169532.113
Notredame C, 2000, J MOL BIOL, V302, P205, DOI 10.1006/jmbi.2000.4042
O'Leary MA, 2013, SCIENCE, V339, P662, DOI 10.1126/science.1229237
Oftedal OT, 2012, ANIMAL, V6, P355, DOI 10.1017/S1751731111001935
Ogg SL, 2004, P NATL ACAD SCI USA, V101, P10084, DOI 10.1073/pnas.0402930101
Paradis E, 2004, BIOINFORMATICS, V20, P289, DOI [10.1093/bioinformatics/btg412, 10.1093/bioinformatics/bty633]
R Core Team, 2018, R: a language and environment for statistical computing
Revell LJ, 2012, METHODS ECOL EVOL, V3, P217, DOI 10.1111/j.2041-210X.2011.00169.x
Roberts RM, 2016, REPRODUCTION, V152, pR179, DOI 10.1530/REP-16-0325
Shi H, 2019, EPIGENET CHROMATIN, V12, DOI 10.1186/s13072-019-0295-4
Smith Kathleen K., 2015, P205
Sondka Z, 2018, NAT REV CANCER, V18, P696, DOI 10.1038/s41568-018-0060-1
Stearns SC., 2016, Evolutionary Medicine
Syvanen M, 2012, ANNU REV GENET, V46, P341, DOI 10.1146/annurev-genet-110711-155529
Tarver JE, 2016, GENOME BIOL EVOL, V8, P330, DOI 10.1093/gbe/evv261
Trigos AS, 2019, ELIFE, V8, DOI 10.7554/eLife.40947
Van Dyke JU, 2014, REPRODUCTION, V147, pR15, DOI 10.1530/REP-13-0309
Vicens A, 2018, GENES-BASEL, V9, DOI 10.3390/genes9120582
Vogel P, 2005, PLACENTA, V26, P591, DOI 10.1016/j.placenta.2004.11.005
Vorbach C, 2002, GENE DEV, V16, P3223, DOI 10.1101/gad.1032702
Wagner GP, 2014, INT J DEV BIOL, V58, P117, DOI 10.1387/ijdb.130335gw
Wang JZ, 2007, BIOINFORMATICS, V23, P1274, DOI 10.1093/bioinformatics/btm087
Ward N, 2014, PLOS ONE, V9, DOI 10.1371/journal.pone.0101850
Weinstein JN, 2013, NAT GENET, V45, P1113, DOI 10.1038/ng.2764
Weon JL, 2015, CURR OPIN CELL BIOL, V37, P1, DOI 10.1016/j.ceb.2015.08.002
Wickham H., 2016, GGPLOT2 ELEGANT GRAP, DOI DOI 10.1007/978-3-319-24277-4
Wildman DE, 2006, P NATL ACAD SCI USA, V103, P3203, DOI 10.1073/pnas.0511344103
Wildman DE, 2016, PLACENTA, V48, pS31, DOI 10.1016/j.placenta.2016.04.004

   Wu JQ, 2017, CURR BIOL, V27, P3025, DOI 10.1016/j.cub.2017.08.043
   Yang P, 2017, TRENDS GENET, V33, P871, DOI 10.1016/j.tig.2017.08.006
   Yang ZH, 2007, MOL BIOL EVOL, V24, P1586, DOI 10.1093/molbev/msm088
   Yu GC, 2018, MOL BIOL EVOL, V35, P3041, DOI 10.1093/molbev/msy194
   Yu GC, 2017, METHODS ECOL EVOL, V8, P28, DOI 10.1111/2041-210X.12628
   Yu GC, 2010, BIOINFORMATICS, V26, P976, DOI
10.1093/bioinformatics/btq064
   Yuan HY, 2018, SCI REP-UK, V8, DOI 10.1038/s41598-018-24022-w
   Zerbino DR, 2018, NUCLEIC ACIDS RES, V46, pD754, DOI
10.1093/nar/gkx1098
   Zhang Y, 2010, PLOS BIOL, V8, DOI [10.1371/journal.pbio.1000430,
10.1371/journal.pbio.1000494]
NR 96
TC 9
Z9 10
U1 0
U2 3
PU OXFORD UNIV PRESS
PI OXFORD
PA GREAT CLARENDON ST, OXFORD OX2 6DP, ENGLAND
SN 1759-6653
J9 GENOME BIOL EVOL
JI Genome Biol. Evol.
PD MAR
PY 2020
VL 12
IS 3
BP 35
EP 47
DI 10.1093/gbe/evaa026
PG 13
WC Evolutionary Biology; Genetics & Heredity
WE Science Citation Index Expanded (SCI-EXPANDED)
SC Evolutionary Biology; Genetics & Heredity
GA LS6MH
UT WOS:000536495200003
PM 32053193
OA Green Published, gold
DA 2025-05-07
ER

PT J
AU Sumner, KM
   Freedman, E
   Mangeni, JN
   Obala, AA
   Abel, L
   Edwards, JK
   Emch, M
   Meshnick, SR
   Pence, BW
   Prudhomme-O'Meara, W
   Taylor, SM
AF Sumner, Kelsey M.

Freedman, Elizabeth
Mangeni, Judith N.
Obala, Andrew A.
Abel, Lucy
Edwards, Jessie K.
Emch, Michael
Meshnick, Steven R.
Pence, Brian W.
Prudhomme-O'Meara, Wendy
Taylor, Steve M.
TI Exposure to Diverse Plasmodium falciparum Genotypes Shapes the Risk of Symptomatic Malaria in Incident and Persistent Infections: A Longitudinal Molecular Epidemiologic Study in Kenya
SO CLINICAL INFECTIOUS DISEASES
LA English
DT Article
DE falciparum malaria; asymptomatic; adaptive immunity
ID ASYMPTOMATIC PARASITEMIA; CLINICAL MALARIA; DRY SEASON; TRANSMISSION; POPULATION; SEQUENCE
AB Background. Repeated exposure to malaria infections could protect against symptomatic progression as people develop adaptive immunity to infections acquired over time.

Methods. We investigated how new, recurrent, and persistent Plasmodium falciparum infections were associated with the odds of developing symptomatic compared with asymptomatic malaria. Using a 14-month longitudinal cohort in Western Kenya, we used amplicon deep sequencing of 2 polymorphic genes (pfama1 and pfcsp) to assess overlap of parasite genotypes (represented by haplotypes) acquired within an individual's successive infections. We hypothesized infections with novel haplotypes would increase the odds of symptomatic malaria.

Results. After excluding initial infections, we observed 534 asymptomatic and 88 symptomatic infections across 186 people. We detected 109 pfcsp haplotypes, and each infection was classified as harboring novel, recurrent, or persistent haplotypes. Incident infections with only new haplotypes had higher odds of symptomatic malaria when compared with infections with only recurrent haplotypes [odds ratio (OR): 3.24; 95% confidence interval (CI), 1.20-8.78], but infections with both new and recurrent haplotypes (OR: 0.64; 95% CI: 0.15-2.65) did not. Assessing persistent infections, those with mixed (persistent with new or recurrent) haplotypes (OR: 0.77; 95% CI: 0.21-2.75) had no association with symptomatic malaria compared with infections with only persistent haplotypes. Results were similar for pfama1.

Conclusions. These results confirm that incident infections with only novel haplotypes were associated with increased odds of symptomatic malaria compared with infections with only recurrent haplotypes but this relationship was not seen when haplotypes persisted over time in consecutive infections.
C1 [Sumner, Kelsey M.; Edwards, Jessie K.; Emch, Michael; Meshnick, Steven R.; Pence, Brian W.; Taylor, Steve M.] Univ N Carolina, Gillings Sch Global Publ Hlth, Dept Epidemiol, Chapel Hill, NC 27515 USA.

[Sumner, Kelsey M.; Freedman, Elizabeth; Prudhomme-O'Meara, Wendy; Taylor, Steve M.] Duke Univ, Sch Med, Div Infect Dis, Durham, NC 27710 USA.

[Mangeni, Judith N.; Prudhomme-O'Meara, Wendy] Moi Univ, Coll Hlth Sci, Sch Publ Hlth, Eldoret, Kenya.
[Obala, Andrew A.] Moi Univ, Coll Hlth Sci, Sch Med, Eldoret, Kenya.
[Abel, Lucy] Moi Teaching & Referral Hosp, Acad Model Providing Access Healthcare, Eldoret, Kenya.
[Emch, Michael] Univ N Carolina, Dept Geog, Chapel Hill, NC 27515 USA.
[Prudhomme-O'Meara, Wendy; Taylor, Steve M.] Duke Univ, Duke Global Hlth Inst, Durham, NC 27710 USA.
C3 University of North Carolina; University of North Carolina Chapel Hill;
Duke University; Moi University; Moi University; University of North Carolina; University of North Carolina Chapel Hill; Duke University
RP Taylor, SM (autor correspondente), Duke Univ, Box 102359 DUMC, Durham, NC 27710 USA.
EM steve.taylor@duke.edu
RI Abel, Lucy/AAF-9225-2019
OI Sumner, Kelsey/0000-0002-4479-897X
FU National Institute of Allergy and Infectious Diseases [R21AI126024, R01AI146849]; Triangle Center for Evolutionary Medicine (TriCEM) Graduate Student Award
FX This work was supported by the National Institute of Allergy and Infectious Diseases (R21AI126024 to W. P. O. and R01AI146849 to W. P. O.
and S. M. T.). This work was also partially supported by a Triangle Center for Evolutionary Medicine (TriCEM) Graduate Student Award.
CR AccessBio, 2019, CARESTART MAL
Adomako-Ankomah Y, 2017, PLOS ONE, V12, DOI 10.1371/journal.pone.0170948
[Anonymous], 2019, World Malaria Report
Babiker HA, 1998, AM J TROP MED HYG, V59, P582, DOI 10.4269/ajtmh.1998.59.582
Boutlis CS, 2006, TRENDS PARASITOL, V22, P371, DOI 10.1016/j.pt.2006.06.002
Buchwald AG, 2019, CLIN INFECT DIS, V68, P106, DOI 10.1093/cid/ciy427
Callahan BJ, 2016, NAT METHODS, V13, P581, DOI [10.1038/NMETH.3869, 10.1038/nmeth.3869]
Chaorattanakawee S, 2018, PLOS ONE, V13, DOI 10.1371/journal.pone.0190418
Contamin H, 1996, AM J TROP MED HYG, V54, P632, DOI 10.4269/ajtmh.1996.54.632
Daniels RF, 2015, P NATL ACAD SCI USA, V112, P7067, DOI 10.1073/pnas.1505691112
Early AM, 2019, MALARIA J, V18, DOI 10.1186/s12936-019-2856-1
Gardner MJ, 2002, NATURE, V419, P498, DOI 10.1038/nature01097
Giha HA, 1998, AM J TROP MED HYG, V58, P399, DOI 10.4269/ajtmh.1998.58.399
GUPTA S, 1994, PARASITOL TODAY, V10, P476, DOI 10.1016/0169-4758(94)90160-0
Hall N, 2002, NATURE, V419, P527, DOI 10.1038/nature01095
Illumina, 2018, ILL MIS
Juliano JJ, 2010, P NATL ACAD SCI USA, V107, P20138, DOI 10.1073/pnas.1007068107
Kun JFJ, 2002, AM J TROP MED HYG, V66, P653, DOI 10.4269/ajtmh.2002.66.653

Levitt B, 2017, SCI REP-UK, V7, DOI 10.1038/srep41108
MARSH K, 1986, SCIENCE, V231, P150, DOI 10.1126/science.2417315
Nelson CS, 2019, NAT COMMUN, V10, DOI 10.1038/s41467-019-13578-4
Newell K, 2016, MALARIA J, V15, DOI 10.1186/s12936-016-1128-6
Njama-Meya D, 2004, TROP MED INT HEALTH, V9, P862, DOI 10.1111/j.1365-3156.2004.01277.x
Nkhoma SC, 2013, MOL ECOL, V22, P273, DOI 10.1111/mec.12099
Nsobya SL, 2004, J INFECT DIS, V189, P2220, DOI 10.1086/421281
Ntoumi F., 1995, Acta Trop, V153, P111, DOI [DOI 10.1016/J.ACTATROPICA.2015.10, 10.1016/j.actatropica.2015.10]
OMeara WP, 2019, J INFECT DIS, V27708, P1
PLOWE CV, 1995, AM J TROP MED HYG, V52, P565, DOI 10.4269/ajtmh.1995.52.565
Portugal S, 2011, NAT MED, V17, P732, DOI 10.1038/nm.2368
R Core Team, 2020, R: a language and environment for statistical computing. R Foundation for Statistical Computing v.4.3.0
Read AF, 2001, SCIENCE, V292, P1099, DOI 10.1126/science.1059410
Roper C, 1998, PARASITOLOGY, V116, P501, DOI 10.1017/S0031182098002650
Sergent Ed., 1935, Arch Inst Pasteur Alger, V13, P279
Smith T, 1999, Trans R Soc Trop Med Hyg, V93 Suppl 1, P59, DOI 10.1016/S0035-9203(99)90329-2
Snow RW, 2002, ADV PARASIT, V52, P235, DOI 10.1016/S0065-308X(02)52013-3
Sondén K, 2015, J INFECT DIS, V212, P608, DOI 10.1093/infdis/jiv088
Sumner KM, 2021, NAT COMMUN, V12, DOI 10.1038/s41467-021-21269-2
Takala SL, 2009, SCI TRANSL MED, V1, DOI 10.1126/scitranslmed.3000257
Taylor SM, 2019, AM J TROP MED HYG, V101, P1416, DOI 10.4269/ajtmh.19-0558
NR 39
TC 7
Z9 9
U1 0
U2 0
PU OXFORD UNIV PRESS INC
PI CARY
PA JOURNALS DEPT, 2001 EVANS RD, CARY, NC 27513 USA
SN 1058-4838
EI 1537-6591
J9 CLIN INFECT DIS
JI Clin. Infect. Dis.
PD OCT 1
PY 2021
VL 73
IS 7
BP 1176
EP 1184
DI 10.1093/cid/ciab357
PG 9
WC Immunology; Infectious Diseases; Microbiology
WE Science Citation Index Expanded (SCI-EXPANDED)
SC Immunology; Infectious Diseases; Microbiology
GA XN9PN
UT WOS:000729829500180
PM 33904907

OA Green Published
DA 2025-05-07
ER

PT J
AU Anderson, JA
   Vilgalys, TP
   Tung, J
AF Anderson, Jordan A.
   Vilgalys, Tauras P.
   Tung, Jenny
TI Broadening primate genomics: new insights into the ecology and evolution
   of primate gene regulation
SO CURRENT OPINION IN GENETICS & DEVELOPMENT
LA English
DT Review
ID EXPRESSION EVOLUTION; TRANSCRIPTOMES; NEANDERTHAL; MECHANISMS;
   DIVERSITY; INFECTION; SEQUENCES; ADMIXTURE; VARIANTS; VIRUSES
AB Comparative analyses have played a key role in understanding how gene regulatory evolution contributes to primate phenotypic diversity. Recently, these studies have expanded to include a wider range of species, within-population as well as interspecific analyses, and research on wild as well as captive individuals. This expansion provides context for understanding genetic and environmental effects on gene regulation in humans, including the importance of the pathogen and social environments. Although taxonomic representation remains biased, inclusion of more species has also begun to reveal the evolutionary processes that explain whether and when gene regulation is conserved. Together, this work highlights how studies in other primates contribute to understanding evolution in our own lineage, and we conclude by identifying promising avenues for future work.
C1 [Anderson, Jordan A.; Vilgalys, Tauras P.; Tung, Jenny] Duke Univ, Dept Evolutionary Anthropol, Durham, NC 27708 USA.
   [Vilgalys, Tauras P.] Univ Chicago, Dept Med, Sect Genet Med, Chicago, IL 60637 USA.
   [Tung, Jenny] Duke Univ, Dept Biol, Durham, NC 27708 USA.
   [Tung, Jenny] Natl Museums Kenya, Inst Primate Res, Nairobi 00502, Kenya.
   [Tung, Jenny] Duke Univ, Duke Populat Res Inst, Durham, NC 27708 USA.
C3 Duke University; University of Chicago; Duke University; Duke University
RP Tung, J (autor correspondente), Duke Univ, Dept Evolutionary Anthropol, Durham, NC 27708 USA.; Tung, J (autor correspondente), Duke Univ, Dept Biol, Durham, NC 27708 USA.; Tung, J (autor correspondente), Natl Museums Kenya, Inst Primate Res, Nairobi 00502, Kenya.; Tung, J (autor correspondente), Duke Univ, Duke Populat Res Inst, Durham, NC 27708 USA.
EM jt5@duke.edu
RI Vilgalys, Tauras/ABA-8953-2021
OI Vilgalys, Tauras/0000-0002-0615-6888
FU National Science Foundation Graduate Research Fellowship [2018264636];
   Triangle Center for Evolutionary Medicine Graduate Student Award; NSF
   [BCS1751783]; National Institutes of Health [R01AG057235, R01HD088558]

FX This work was supported by National Science Foundation Graduate Research
Fellowship #2018264636 and the Triangle Center for Evolutionary Medicine
Graduate Student Award to J.A.A., NSF BCS1751783 to J.T. and T.P.V., and
National Institutes of Health R01AG057235 and R01HD088558 to J.T.
CR Aguet F, 2017, NATURE, V550, P204, DOI 10.1038/nature24277
Arnold C, 2010, EVOL ANTHROPOL, V19, P114, DOI 10.1002/evan.20251
Babbitt CC, 2010, GENOME BIOL EVOL, V2, P67, DOI 10.1093/gbe/evq002
Barnett KR, 2020, MOL CELL, V6, P1159
Berthelot C, 2018, NAT ECOL EVOL, V2, P152, DOI 10.1038/s41559-017-0377-2
Blake LE, 2020, GENOME RES, V30, P250, DOI 10.1101/gr.254904.119
Blekhman R, 2008, PLOS GENET, V4
Boffelli D, 2003, SCIENCE, V299, P1391, DOI 10.1126/science.1081331
Brawand D, 2011, NATURE, V478, P343, DOI 10.1038/nature10532
Chahroudi A, 2012, SCIENCE, V335, P1188, DOI 10.1126/science.1217550
Chen LY, 2019, LAB ANIMAL, V48, P57, DOI 10.1038/s41684-018-0217-2
Chun K, 2017, PSYCHONEUROENDOCRINO, V76, P107, DOI 10.1016/j.psyneuen.2016.10.029
Cole SW, 2012, P NATL ACAD SCI USA, V109, P20578, DOI 10.1073/pnas.1218253109
Danko CG, 2018, NAT ECOL EVOL, V2, P537, DOI 10.1038/s41559-017-0447-5
de Manuel M, 2016, SCIENCE, V354, P477, DOI 10.1126/science.aag2602
Dunn CW, 2018, P NATL ACAD SCI USA, V115, pE409, DOI 10.1073/pnas.1707515115
Enard D, 2018, CELL, V175, P360, DOI 10.1016/j.cell.2018.08.034
Enard D, 2016, ELIFE, V5, DOI 10.7554/eLife.12469
Enard W, 2002, SCIENCE, V296, P340, DOI 10.1126/science.1068996
Fan Y, 2019, GENOME BIOL, V20, DOI 10.1186/s13059-019-1813-1
Giger T, 2010, GENOME BIOL EVOL, V2, P284, DOI 10.1093/gbe/evq018
Gilad Y, 2005, GENOME RES, V15, P674, DOI 10.1101/gr.3335705
Glassberg EC, 2019, GENETICS, V211, P757, DOI 10.1534/genetics.118.301833
Gschwind AR, 2017, BMC GENOMICS, V18, DOI 10.1186/s12864-017-3531-y
Hernandez M, 2020, BIORXIV, DOI [10.1101/2020.04.16.045450, DOI 10.1101/2020.04.16.045450.]
Hernandez RD, 2019, NAT GENET, V51, P1349, DOI 10.1038/s41588-019-0487-7
Hernando-Herraez I, 2013, PLOS GENET, V9, DOI 10.1371/journal.pgen.1003763
Hong JW, 2008, SCIENCE, V321, P1314, DOI 10.1126/science.1160631
Huang YF, 2017, NAT GENET, V49, P618, DOI 10.1038/ng.3810
Jacquelin B, 2009, J CLIN INVEST, V119, P3544, DOI 10.1172/JCI40093
Jasinska AJ, 2017, NAT GENET, V49, P1714, DOI 10.1038/ng.3959
Khaitovich P, 2005, SCIENCE, V309, P1850, DOI 10.1126/science.1108296
King MC, 1975, SCIENCE, P188
Klein JC, 2018, GENOME BIOL, V19, DOI 10.1186/s13059-018-1473-6
Lea AJ, 2018, ELIFE, V7, DOI 10.7554/eLife.37513
Lea AJ, 2018, P NATL ACAD SCI USA, V115, pE12163, DOI 10.1073/pnas.1811967115
McCoy RC, 2017, CELL, V168, P916, DOI 10.1016/j.cell.2017.01.038
Melé M, 2015, SCIENCE, V348, P660, DOI 10.1126/science.aaa0355

Pacis A, 2019, P NATL ACAD SCI USA, V116, P6938, DOI 10.1073/pnas.1814700116
Perry GH, 2012, GENOME RES, V22, P602, DOI 10.1101/gr.130468.111
Pickrell JK, 2010, NATURE, V464, P768, DOI 10.1038/nature08872
Prabhakar S, 2006, GENOME RES, V16, P855, DOI 10.1101/gr.4717506
Prado-Martinez J, 2013, NATURE, V499, P471, DOI 10.1038/nature12228
Rogers J, 2019, SCI ADV, V5, DOI 10.1126/sciadv.aau6947
Rohlfs RV, 2014, MOL BIOL EVOL, V31, P201, DOI 10.1093/molbev/mst190
Romero IG, 2012, NAT REV GENET, V13, P505, DOI 10.1038/nrg3229
Sankararaman S, 2016, CURR BIOL, V26, P1241, DOI 10.1016/j.cub.2016.03.037
Sanz J, 2020, P NATL ACAD SCI USA, V117, P23317, DOI 10.1073/pnas.1820846116
Simons ND, 2019, GENOME BIOL EVOL, V11, P1630, DOI 10.1093/gbe/evz099
Snyder-Mackler N, 2020, SCIENCE, V368, P843, DOI 10.1126/science.aax9553
Snyder-Mackler N, 2019, P NATL ACAD SCI USA, V116, P1219, DOI 10.1073/pnas.1811758115
Snyder-Mackler N, 2016, SCIENCE, V354, P1041, DOI 10.1126/science.aah3580
Sundaram L, 2018, NAT GENET, V50, P1161, DOI 10.1038/s41588-018-0167-z
Svardal H, 2017, NAT GENET, V49, P1705, DOI 10.1038/ng.3980
Telis N., 2019, BIORXIV, DOI [10.1101/708230, DOI 10.1101/708230]
Tung J, 2017, CURR OPIN GENET DEV, V47, P61, DOI 10.1016/j.gde.2017.08.010
Tung J, 2015, ELIFE, V4, DOI 10.7554/eLife.04729
Tung J, 2012, P NATL ACAD SCI USA, V109, P6490, DOI 10.1073/pnas.1202734109
Vanderpool D., 2020, BIORXIV, DOI DOI 10.1101/2020.04.15.043786
Varki A, 2005, GENOME RES, V15, P1746, DOI 10.1101/gr.3737405
Vernot B, 2016, SCIENCE, V352, P235, DOI 10.1126/science.aad9416
Vilgalys TP, 2019, MOL BIOL EVOL, V36, P527, DOI 10.1093/molbev/msy227
Villar D, 2015, CELL, V160, P554, DOI 10.1016/j.cell.2015.01.006
Wall JD, 2016, MOL ECOL, V25, P3469, DOI 10.1111/mec.13684
Wittkopp PJ, 2012, NAT REV GENET, V13, P59, DOI 10.1038/nrg3095
Xue C, 2016, GENOME RES, V26, P1651, DOI 10.1101/gr.204255.116
NR 66
TC 10
Z9 13
U1 0
U2 15
PU CURRENT BIOLOGY LTD
PI LONDON
PA 84 THEOBALDS RD, LONDON WC1X 8RR, ENGLAND
SN 0959-437X
EI 1879-0380
J9 CURR OPIN GENET DEV
JI Curr. Opin. Genet. Dev.
PD JUN
PY 2020
VL 62
BP 16
EP 22
DI 10.1016/j.gde.2020.05.009

PG 7
WC Cell Biology; Genetics & Heredity
WE Science Citation Index Expanded (SCI-EXPANDED)
SC Cell Biology; Genetics & Heredity
GA NL7JD
UT WOS:000567586300004
PM 32569794
OA Bronze, Green Accepted
DA 2025-05-07
ER

PT B
AU Mertens, G
AF Mertens, Gerhard
BE Urbano, KV
TI Also Medicine Needs Evolution! What Clinical Sciences Can Learn from Evolution Biology
SO ADVANCES IN GENETICS RESEARCH, VOL 2
SE Advances in Genetics Research Series
LA English
DT Article; Book Chapter
ID CHLAMYDIA-PNEUMONIAE; NATURAL-SELECTION; ATHEROSCLEROSIS; RESISTANCE; HEMOCHROMATOSIS; SMOKING; HLA
AB In its population genetic sense, evolution is defined as the ongoing change of gene frequencies in populations due to one or several of the driving forces of evolution: selection, drift, mutation and migration. Evolution's role is central in the sub-discipline of biology that addresses health and disease in humans and training in evolutionary thinking can both help biomedical researchers and clinicians ask useful questions they might not otherwise pose.

The co-evolution of man and his environment of pathogenic micro-organisms, the rapidly shifting antibiotic resistance of these pathogens and our persistent vulnerability to chronic diseases should all be seen from an evolutionary perspective. These subjects form the core of "evolutionary medicine", which will be illustrated by a number of thought inspiring examples.

The hypothesis that allergy can be viewed like cough and pain as a defence mechanism evolved by natural selection, is gaining support from toxicological studies measuring lower levels of carcinogens in allergic individuals.

Recent research, combining the effects of genes and environment, has provided surprising clues to the cause of atherosclerosis, a major public health problem.

In medical microbiology, the combination of the short generation time of bacteria, the exchange of resistance genes between species and the swift transfer of bacteria from animals to humans and between humans, forms a life threatening cocktail with a critical role for evolutionary mechanisms.

The HLA system which encodes proteins of the immune response, shows the most extensive polymorphism of the whole human genome. The global distribution of HLA alleles illustrates evolution by migration, while the polymorphism itself is promoted by natural selection, operating through pre- and post-conceptual mechanisms.

An example of "recent" evolution in Homo sapiens by natural selection and a genetic bottleneck, comes from the relation between Yersinia pestis and hemochromatosis. The geographical distribution of the hemochromatosis gene correlates strictly with the area of the 14(th) century bubonic plague that raged through Europe, which can be explained by a protective mechanism of the hemochromatosis gene against bacterial infection.

The examples above make a strong case for recognizing evolution biology as a basic science for medicine.

C1 Univ Antwerp, Dept Biol Clin, B-2020 Antwerp, Belgium.
C3 University of Antwerp
RP Mertens, G (autor correspondente), Univ Antwerp, Dept Biol Clin, B-2020 Antwerp, Belgium.

CR Barnoya J, 2005, CIRCULATION, V111, P2684, DOI 10.1161/CIRCULATIONAHA.104.492215
Bywater RJ, 2004, J VET MED B, V51, P361, DOI 10.1111/j.1439-0450.2004.00791.x
Cayley WE, 2008, BRIT MED J, V336, P506, DOI 10.1136/bmj.39357.698889.94
Coenen S, 2007, QUAL SAF HEALTH CARE, V16, P440, DOI 10.1136/qshc.2006.021121
DAVIGNON J, 1988, ARTERIOSCLEROSIS, V8, P1, DOI 10.1161/01.ATV.8.1.1
Dobzhansky Theodosius, 1973, Zygon, V8, P261, DOI 10.1111/j.1467-9744.1973.tb00232.x
Ewald PW, 2000, J INFECT DIS, V181, pS394, DOI 10.1086/315602
FISHER RA, 1950, BIOMETRICS, V6, P353, DOI 10.2307/3001780
Hoymans VY, 2007, ACTA CARDIOL, V62, P565, DOI 10.2143/AC.62.6.2024015
Leung Donald Y. M., 1997, JAMA (Journal of the American Medical Association), V278, P1914, DOI 10.1001/jama.278.22.1914
Levy SB, 2004, NAT MED, V10, pS122, DOI 10.1038/nm1145
Levy SB, 2002, J ANTIMICROB CHEMOTH, V49, P25, DOI 10.1093/jac/49.1.25
Meyer D, 2006, GENETICS, V173, P2121, DOI 10.1534/genetics.105.052837
Moalem S, 2002, MED HYPOTHESES, V59, P325, DOI 10.1016/S0306-9877(02)00179-2
Mochida Yoji, 2007, Fukuoka Acta Medica, V98, P106
Nesse RM, 2008, B WORLD HEALTH ORGAN, V86, P83, DOI 10.2471/BLT.07.049601
Nesse RM, 2006, SCIENCE, V311, P1071, DOI 10.1126/science.1125956
Nesse RM, 2005, Q REV BIOL, V80, P62, DOI 10.1086/431026
OBER C, 1992, AM J HUM GENET, V50, P6
Pepin KM, 2007, EVOLUTION, V61, P1710, DOI 10.1111/j.1558-5646.2007.00143.x
PEPIN KM, 2008, EVOL BIOL, V8, P85
Sedlak TW, 2004, PEDIATRICS, V113, P1776, DOI 10.1542/peds.113.6.1776
Single RM, 2007, TISSUE ANTIGENS, V69, P185, DOI 10.1111/j.1399-0039.2006.00767.x
Watson C, 2008, CLIN SCI, V114, P509, DOI 10.1042/CS20070298
WEDEKIND C, 1995, P ROY SOC B-BIOL SCI, V260, P245, DOI 10.1098/rspb.1995.0087
Wong HR, 1997, AM J PHYSIOL-LUNG C, V273, pL1, DOI 10.1152/ajplung.1997.273.1.L1
YOUNG JZ, 1991, VISUAL NEUROSCI, V7, P1, DOI 10.1017/S0952523800010907
Zacharia B, 2003, MED HYPOTHESES, V60, P1, DOI 10.1016/S0306-9877(02)00217-7
NR 28

TC 0
Z9 0
U1 0
U2 6
PU NOVA SCIENCE PUBLISHERS, INC
PI HAUPPAUGE
PA 400 OSER AVE, STE 1600, HAUPPAUGE, NY 11788-3635 USA
BN 978-1-60876-100-5
J9 ADV GENET RES SER
PY 2010
VL 2
BP 213
EP 221
PG 9
WC Genetics & Heredity; Medicine, Research & Experimental
WE Book Citation Index– Science (BKCI-S)
SC Genetics & Heredity; Research & Experimental Medicine
GA BQQ34
UT WOS:000281539600017
DA 2025-05-07
ER

PT J
AU Nunn, CL
   Vining, AQ
   Chakraborty, D
   Reiskind, MH
   Young, HS
AF Nunn, Charles L.
   Vining, Alexander Q.
   Chakraborty, Debapriyo
   Reiskind, Michael H.
   Young, Hillary S.
TI Effects of host extinction and vector preferences on vector-borne
   disease risk in phylogenetically structured host-hector communities
SO PLOS ONE
LA English
DT Article
ID WEST-NILE-VIRUS; INFECTIOUS-DISEASE; BIODIVERSITY CHANGE; LAND-USE;
   DIVERSITY; TRANSMISSION; PARASITES; CONSEQUENCES; ASSOCIATIONS;
PATTERNS
AB Anthropogenic disturbance impacts the phylogenetic composition and diversity of ecological communities. While changes in diversity are known to dramatically change species interactions and alter disease dynamics, the effects of phylogenetic changes in host and vector communities on disease have been relatively poorly studied. Using a theoretical model, we investigated how phylogeny and extinction influence network structural characteristics relevant to disease transmission in disturbed environments. We modelled a multi-host, multi-vector community as a bipartite ecological network, where nodes represent host and vector species and edges represent connections among them through vector feeding, and we simulated vector preferences and threat status on host and parasite phylogenies. We then simulated loss of hosts, including phylogenetically clustered losses, to investigate how extinction

influences network structure. We compared effects of phylogeny and extinction to those of host specificity, which we predicted to strongly increase network modularity and reduce disease prevalence. The simulations revealed that extinction often increased modularity, with higher modularity as species loss increased, although not as much as increasing host specificity did. These results suggest that extinction itself, all else being equal, may reduce disease prevalence in disturbed communities. However, in real communities, systematic patterns in species loss (e.g. favoring high competence species) or changes in abundance may counteract these effects. Unexpectedly, we found that effects of phylogenetic signal in host and vector traits were relatively weak, and only important when phylogenetic signal of host and vector traits were similar, or when these traits both varied.
C1 [Nunn, Charles L.; Vining, Alexander Q.; Chakraborty, Debapriyo] Duke Univ, Dept Evolutionary Anthropol, Durham, NC 27708 USA.
[Nunn, Charles L.] Duke Global Hlth Inst, Durham, NC 27710 USA.
[Vining, Alexander Q.] Univ Calif Davis, Grad Program Anim Behav, Davis, CA USA.
[Chakraborty, Debapriyo] Natl Vet Sch Toulouse, INRAE ENVT IHAP, Toulouse, France.
[Reiskind, Michael H.] North Carolina State Univ, Dept Entomol & Plant Pathol, Raleigh, NC USA.
[Young, Hillary S.] Univ Calif Santa Barbara, Dept Ecol Evolut & Marine Biol, Santa Barbara, CA 93106 USA.
C3 Duke University; Duke University; University of California System; University of California Davis; INRAE; North Carolina State University; University of California System; University of California Santa Barbara
RP Nunn, CL (autor correspondente), Duke Univ, Dept Evolutionary Anthropol, Durham, NC 27708 USA.; Nunn, CL (autor correspondente), Duke Global Hlth Inst, Durham, NC 27710 USA.
EM clunn@duke.edu
RI Young, Hillary/S-1514-2017; Reiskind, Michael/I-5614-2012
OI Reiskind, Michael/0000-0001-7307-9416
FU National Science Foundation (NSF)via the National Evolutionary Synthesis Center [NSF EF0905606]; Triangle Center for Evolutionary Medicine; Fulbright Association; US-India Educational Foundation
FX This study was supported by the National Science Foundation (NSF) in the form of Catalysis Meeting funding awarded to CLN and HSY (via the National Evolutionary Synthesis Center, NSF EF0905606), the Triangle Center for Evolutionary Medicine in the form of funds awarded to CLN and HSY, the Fulbright Association in the form of funds awarded to DC, and the US-India Educational Foundation in the form of funds awarded to DC.
CR [Anonymous], 2015, R: a language and environment for statistical computing
Beaulieu MRS, 2019, PLOS ONE, V14, DOI 10.1371/journal.pone.0215485
Blomberg SP, 2003, EVOLUTION, V57, P717, DOI 10.1111/j.0014-3820.2003.tb00285.x
BLOWER SM, 1994, INT STAT REV, V62, P229, DOI 10.2307/1403510

BURNHAM K. A., 2002, Model selection and multimodel inference: a practical informationtheoretic approach, VSecond, DOI 10.1007/978-0-387-22456-5_5
Carnell R., 2009, lhs: Latin Hypercube Samples
Chaves LF, 2010, FRONT ZOOL, V7, DOI 10.1186/1742-9994-7-3
Civitello DJ, 2015, P NATL ACAD SCI USA, V112, P8667, DOI 10.1073/pnas.1506279112
Cohuet A, 2010, TRENDS PARASITOL, V26, P130, DOI 10.1016/j.pt.2009.12.001
Cooper N, 2012, ECOL LETT, V15, P1370, DOI 10.1111/j.1461-0248.2012.01858.x
D'agata S, 2014, CURR BIOL, V24, P555, DOI 10.1016/j.cub.2014.01.049
Dormann C.F., 2008, Networks, V8, DOI DOI 10.1159/000265935
Dormann CF, 2014, METHODS ECOL EVOL, V5, P90, DOI 10.1111/2041-210X.12139
Dornelas M, 2019, ECOL LETT, V22, P847, DOI 10.1111/ele.13242
Dornelas M, 2014, SCIENCE, V344, P296, DOI 10.1126/science.1248484
Dunn RR, 2009, P ROY SOC B-BIOL SCI, V276, P3037, DOI 10.1098/rspb.2009.0413
Estrada-Peña A, 2016, SCI DATA, V3, DOI 10.1038/sdata.2016.56
Estrada-Peña A, 2014, TRENDS PARASITOL, V30, P205, DOI 10.1016/j.pt.2014.02.003
Evans MV, 2017, ELIFE, V6, DOI 10.7554/eLife.22053
Ezenwa VO, 2006, P ROY SOC B-BIOL SCI, V273, P109, DOI 10.1098/rspb.2005.3284
FELSENSTEIN J, 1985, AM NAT, V125, P1, DOI 10.1086/284325
Fountain-Jones NM, 2018, BIOL REV, V93, P950, DOI 10.1111/brv.12380
Frank HK, 2017, AM NAT, V190, P200, DOI 10.1086/692534
Freckleton RP, 2002, AM NAT, V160, P712, DOI 10.1086/343873
Frishkoff LO, 2014, SCIENCE, V345, P1343, DOI 10.1126/science.1254610
Gibb R, 2020, NATURE, V584, P398, DOI 10.1038/s41586-020-2562-8
Gilbert GS, 2007, P NATL ACAD SCI USA, V104, P4979, DOI 10.1073/pnas.0607968104
Gilbert GS, 2016, ANNU REV PHYTOPATHOL, V54, P549, DOI 10.1146/annurev-phyto-102313-045959
Griffin RH, 2012, EVOL ECOL, V26, P779, DOI 10.1007/s10682-011-9526-2
Hadfield JD, 2014, AM NAT, V183, P174, DOI 10.1086/674445
Halliday FW, 2020, ECOL LETT, V23, P1611, DOI 10.1111/ele.13590
Harrison T, 2018, GLOBAL CHANGE BIOL, V24, P287, DOI 10.1111/gcb.13921
Hellgren O, 2008, MOL ECOL, V17, P1605, DOI 10.1111/j.1365-294X.2007.03680.x
Helmus MR, 2010, ECOL LETT, V13, P162, DOI 10.1111/j.1461-0248.2009.01411.x
Heym EC, 2019, MED VET ENTOMOL, V33, P203, DOI 10.1111/mve.12350
Hill CA, 2005, NAT REV MICROBIOL, V3, P262, DOI 10.1038/nrmicro1101
Hill SLL, 2016, CONSERV LETT, V9, P405, DOI 10.1111/conl.12291
Huang ZYX, 2019, J ANIM ECOL, V88, P1044, DOI 10.1111/1365-2656.12997
Johnson PTJ, 2010, J EXP BIOL, V213, P961, DOI 10.1242/jeb.037721
Johnson PTJ, 2019, P ROY SOC B-BIOL SCI, V286, DOI 10.1098/rspb.2019.0260
Jones FAM, 2020, ROY SOC OPEN SCI, V7, DOI 10.1098/rsos.192045
Keesing F, 2010, NATURE, V468, P647, DOI 10.1038/nature09575
Kilpatrick AM, 2006, P ROY SOC B-BIOL SCI, V273, P2327, DOI 10.1098/rspb.2006.3575

Kiszewski A, 2004, AM J TROP MED HYG, V70, P486, DOI 10.4269/ajtmh.2004.70.486
Krasnov BR, 2016, OIKOS, V125, P29, DOI 10.1111/oik.02178
Lacroix C, 2014, J R SOC INTERFACE, V11, DOI 10.1098/rsif.2013.0947
Larsen S, 2018, ECOLOGY, V99, P1316, DOI 10.1002/ecy.2213
Liu X, 2017, P ROY SOC B-BIOL SCI, V284, DOI 10.1098/rspb.2016.2621
Liu X, 2016, ECOLOGY, V97, P1680, DOI 10.1890/15-1784.1
Lyimo IN, 2009, TRENDS PARASITOL, V25, P189, DOI 10.1016/j.pt.2009.01.005
Malmqvist B, 2004, P ROY SOC B-BIOL SCI, V271, pS152, DOI 10.1098/rsbl.2003.0120
Martin LB, 2016, INTEGR COMP BIOL, V56, P1225, DOI 10.1093/icb/icw064
McCoy KD, 2013, FRONT CELL INFECT MI, V3, DOI 10.3389/fcimb.2013.00057
McGill BJ, 2015, TRENDS ECOL EVOL, V30, P104, DOI 10.1016/j.tree.2014.11.006
Miller E, 2013, PLOS ONE, V8, DOI 10.1371/journal.pone.0080279
Morens DM, 2004, NATURE, V430, P242, DOI 10.1038/nature02759
MuMIn Barton K., 2011, MULTIMODEL INFERENCE
NEE S, 1994, PHILOS T ROY SOC B, V344, P77, DOI 10.1098/rstb.1994.0054
Nee S, 2001, EVOLUTION, V55, P661, DOI 10.1554/0014-3820(2001)055[0661:ISRFP]2.0.CO;2
Nee S, 2006, ANNU REV ECOL EVOL S, V37, P1, DOI 10.1146/annurev.ecolsys.37.091305.110035
Newbold T, 2020, FUNCT ECOL, V34, P684, DOI 10.1111/1365-2435.13500
Newbold T, 2019, EMERG TOP LIFE SCI, V3, P207, DOI 10.1042/ETLS20180135
Newbold T, 2018, PLOS BIOL, V16, DOI 10.1371/journal.pbio.2006841
Newbold T, 2015, NATURE, V520, P45, DOI 10.1038/nature14324
Newman MEJ, 2004, EUR PHYS J B, V38, P321, DOI 10.1140/epjb/e2004-00124-y
Newman MEJ, 2004, PHYS REV E, V69, DOI 10.1103/PhysRevE.69.026113
Nowakowski AJ, 2018, P NATL ACAD SCI USA, V115, pE3454, DOI 10.1073/pnas.1714891115
Nunn CharlesL., 2011, COMP APPROACH EVOLUT
Omondi D, 2015, PLOS ONE, V10, DOI 10.1371/journal.pone.0134375
Ostfeld RS, 2012, ANNU REV ECOL EVOL S, V43, P157, DOI 10.1146/annurev-ecolsys-102710-145022
Pagel M, 1999, NATURE, V401, P877, DOI 10.1038/44766
Park AW, 2018, P ROY SOC B-BIOL SCI, V285, DOI 10.1098/rspb.2017.2613
Parker IM, 2015, NATURE, V520, P542, DOI 10.1038/nature14372
Previtali MA, 2012, OIKOS, V121, P1483, DOI 10.1111/j.1600-0706.2012.020215.x
Purvis A, 2000, P ROY SOC B-BIOL SCI, V267, P1947, DOI 10.1098/rspb.2000.1234
Purvis A, 2008, ANNU REV ECOL EVOL S, V39, P301, DOI 10.1146/annurev-ecolsys-063008-102010
R Development Core Team, 2014, R: a language and environment for statistical computing
Revell LJ, 2012, METHODS ECOL EVOL, V3, P217, DOI 10.1111/j.2041-210X.2011.00169.x
Ripple WJ, 2017, P NATL ACAD SCI USA, V114, P10678, DOI 10.1073/pnas.1702078114
Ross R., 1910, The prevention of malaria

Sah P, 2017, P NATL ACAD SCI USA, V114, P4165, DOI 10.1073/pnas.1613616114
Salathé M, 2010, PLOS COMPUT BIOL, V6, DOI 10.1371/journal.pcbi.1000736
SEAHOLM SK, 1988, INT J BIOMED COMPUT, V23, P97, DOI 10.1016/0020-7101(88)90067-0
SETTLE WH, 1990, ECOLOGY, V71, P1461, DOI 10.2307/1938283
Simpson JE, 2012, P ROY SOC B-BIOL SCI, V279, P925, DOI 10.1098/rspb.2011.1282
Smith DL, 2007, PLOS BIOL, V5, P531, DOI 10.1371/journal.pbio.0050042
Smith FA, 2018, SCIENCE, V360, P310, DOI 10.1126/science.aao5987
Smith KF, 2014, J R SOC INTERFACE, V11, DOI 10.1098/rsif.2014.0950
STEIN M, 1987, TECHNOMETRICS, V29, P143, DOI 10.2307/1269769
Streicker DG, 2010, SCIENCE, V329, P676, DOI 10.1126/science.1188836
Sumner KM, 2018, BEHAVIOUR, V155, P639, DOI [10.1163/1568539X-00003508, 10.1163/1568539x-00003508]
Takken W, 2013, ANNU REV ENTOMOL, V58, P433, DOI 10.1146/annurev-ento-120811-153618
Vellend M, 2017, ECOLOGY, V98, P583, DOI 10.1002/ecy.1660
Vellend M, 2013, P NATL ACAD SCI USA, V110, P19456, DOI 10.1073/pnas.1312779110
Wang LF, 2019, CURR OPIN VIROL, V34, P79, DOI 10.1016/j.coviro.2018.12.007
Wang YYXG, 2019, FUNCT ECOL, V33, P1332, DOI 10.1111/1365-2435.13311
Wood CL, 2013, TRENDS ECOL EVOL, V28, P239, DOI 10.1016/j.tree.2012.10.011
Young H, 2013, ECOL LETT, V16, P656, DOI 10.1111/ele.12094
Young HS, 2017, TRENDS ECOL EVOL, V32, P41, DOI 10.1016/j.tree.2016.09.008
Young HS, 2016, ANNU REV ECOL EVOL S, V47, P333, DOI 10.1146/annurev-ecolsys-112414-054142
NR 100
TC 1
Z9 1
U1 1
U2 12
PU PUBLIC LIBRARY SCIENCE
PI SAN FRANCISCO
PA 1160 BATTERY STREET, STE 100, SAN FRANCISCO, CA 94111 USA
SN 1932-6203
J9 PLOS ONE
JI PLoS One
PY 2021
VL 16
IS 8
AR e0256456
DI 10.1371/journal.pone.0256456
PG 24
WC Multidisciplinary Sciences
WE Science Citation Index Expanded (SCI-EXPANDED)
SC Science & Technology - Other Topics
GA UD7YU
UT WOS:000687421200033
PM 34424937

OA Green Published, gold
DA 2025-05-07
ER

PT J
AU O'Keeffe, KR
   Simha, A
   Mitchell, CE
AF O'Keeffe, Kayleigh R.
   Simha, Anita
   Mitchell, Charles E.
TI Indirect interactions among co-infecting parasites and a microbial
   mutualist impact disease progression
SO PROCEEDINGS OF THE ROYAL SOCIETY B-BIOLOGICAL SCIENCES
LA English
DT Article
DE indirect interactions; species interactions; co-infection; disease
   ecology; within-host microbial interactions; plant pathogens
ID YELLOW DWARF VIRUS; WITHIN-HOST COMPETITION; MULTIPLE INFECTION;
FUNGAL
   ENDOPHYTES; COINFECTION; PLANT; SYMBIONTS; PATHOGENS; BARLEY;
   SUSCEPTIBILITY
AB Interactions among parasites and other microbes within hosts can
impact disease progression, yet study of such interactions has been
mostly limited to pairwise combinations of microbes. Given the diversity
of microbes within hosts, indirect interactions among more than two
microbial species may also impact disease. To test this hypothesis, we
performed inoculation experiments that investigated interactions among
two fungal parasites, Rhizoctonia solani and Colletotrichum cereale, and
a systemic fungal endophyte, Epichloe coenophiala, within the grass, tall
fescue (Lolium arundinaceum). Both direct and indirect interactions
impacted disease progression. While the endophyte did not directly
influence R. solani disease progression or C. cereale symptom
development, the endophyte modified the interaction between the two
parasites. The magnitude of the facilitative effect of C. cereale on the
growth of R. solani tended to be greater when the endophyte was present.
Moreover, this interaction modification strongly affected leaf mortality.
For plants lacking the endophyte, parasite co-inoculation did not
increase leaf mortality compared to single-parasite inoculations. By
contrast, for endophyte-infected plants, parasite co-inoculation
increased leaf mortality compared to inoculation with R. solani or C.
cereale alone by 1.9 or 4.9 times, respectively. Together, these results
show that disease progression can be strongly impacted by indirect
interactions among microbial symbionts.
C1 [O'Keeffe, Kayleigh R.; Simha, Anita; Mitchell, Charles E.] Univ North
Carolina Chapel Hill, Dept Biol, Chapel Hill, NC 27599 USA.
   [O'Keeffe, Kayleigh R.] Univ Penn, Dept Biol, Philadelphia, PA 19104
USA.
   [Simha, Anita] Duke Univ, Dept Biol, Durham, NC USA.
   [Mitchell, Charles E.] Univ N Carolina, Environm Ecol & Energy
Program, Chapel Hill, NC 27515 USA.
C3 University of North Carolina School of Medicine; University of North
   Carolina; University of North Carolina Chapel Hill; University of

Pennsylvania; Duke University; University of North Carolina; University
of North Carolina Chapel Hill
RP O'Keeffe, KR (autor correspondente), Univ North Carolina Chapel Hill, Dept Biol, Chapel Hill, NC 27599 USA.; O'Keeffe, KR (autor correspondente), Univ Penn, Dept Biol, Philadelphia, PA 19104 USA.
EM kokeeffe@sas.upenn.edu
RI O'Keeffe, Kayleigh/AAG-8135-2019; Mitchell, Charles/I-3709-2014
OI O'Keeffe, Kayleigh/0000-0002-8259-6439; Mitchell,
Charles/0000-0002-1633-1993; Simha, Anita/0000-0002-8770-3029
FU NSF-USDA joint programme in Ecology and Evolution of Infectious Diseases
(USDA-NIFA AFRI) [2016-67013-25762]; Triangle Center for Evolutionary
Medicine; National Science Foundation; NIFA [914333, 2016-67013-25762]
Funding Source: Federal RePORTER
FX This work was supported by the NSF-USDA joint programme in Ecology and
Evolution of Infectious Diseases (USDA-NIFA AFRI grant no.
2016-67013-25762). K.R.O. was supported by graduate research fellowships
from the Triangle Center for Evolutionary Medicine and the National
Science Foundation.
CR Abbate JL, 2018, PLOS NEGLECT TROP D, V12, DOI 10.1371/journal.pntd.0006483
Adame-Alvarez RM, 2014, FEMS MICROBIOL LETT, V355, P100, DOI 10.1111/1574-6968.12454
Al-Naimi FA, 2005, OECOLOGIA, V143, P449, DOI 10.1007/s00442-004-1814-x
[Anonymous], 2020, Linear and nonlinear mixed effects models R package nlme version 3.1149
Bandilla M, 2006, INT J PARASITOL, V36, P987, DOI 10.1016/j.ijpara.2006.05.001
Bashey F, 2013, EVOLUTION, V67, P900, DOI 10.1111/j.1558-5646.2012.01825.x
Belkaid Y, 2014, CELL, V157, P121, DOI 10.1016/j.cell.2014.03.011
Berry D, 2014, FRONT MICROBIOL, V5, DOI 10.3389/fmicb.2014.00219
Bordes Frederic, 2011, Infect Ecol Epidemiol, V1, DOI 10.3402/iee.v1i0.7346
Borer ET, 2013, BASIC APPL ECOL, V14, P533, DOI 10.1016/j.baae.2013.08.009
Bronstein JL, 2001, ECOL LETT, V4, P277, DOI 10.1046/j.1461-0248.2001.00218.x
Buck JC, 2019, TRENDS PARASITOL, V35, P835, DOI 10.1016/j.pt.2019.07.007
Chaneton EJ, 2000, OIKOS, V88, P380, DOI 10.1034/j.1600-0706.2000.880217.x
Chase JM, 2003, OECOLOGIA, V136, P489, DOI 10.1007/s00442-003-1311-7
CLAY K, 1990, ANNU REV ECOL SYST, V21, P275, DOI 10.1146/annurev.es.21.110190.001423
Clay PA, 2019, EVOLUTION, V73, P2189, DOI 10.1111/evo.13843
Clay PA, 2019, OIKOS, V128, P571, DOI 10.1111/oik.05937
Clay PA, 2019, AM NAT, V193, P187, DOI 10.1086/701126
Dickie IA, 2012, ECOL LETT, V15, P133, DOI 10.1111/j.1461-0248.2011.01722.x

Diuk-Wasser MA, 2016, TRENDS PARASITOL, V32, P30, DOI 10.1016/j.pt.2015.09.008
Dushoff J, 2019, METHODS ECOL EVOL, V10, P756, DOI 10.1111/2041-210X.13159
Ezenwa VO, 2015, SCIENCE, V347, P175, DOI 10.1126/science.1261714
Fisher MC, 2012, NATURE, V484, P186, DOI 10.1038/nature10947
Fukami T, 2015, ANNU REV ECOL EVOL S, V46, P1, DOI 10.1146/annurev-ecolsys-110411-160340
Gerardo NM, 2014, CURR OPIN INSECT SCI, V4, P8, DOI 10.1016/j.cois.2014.08.002
Gibson AK, 2011, PLOS NEGLECT TROP D, V5, DOI 10.1371/journal.pntd.0001142
Glazebrook J, 2005, ANNU REV PHYTOPATHOL, V43, P205, DOI 10.1146/annurev.phyto.43.040204.135923
Hacquard S, 2015, NEW PHYTOL, V205, P1424, DOI 10.1111/nph.13133
Haine ER, 2008, P ROY SOC B-BIOL SCI, V275, P353, DOI 10.1098/rspb.2007.1211
Halliday FW, 2020, NAT ECOL EVOL, V4, P1510, DOI 10.1038/s41559-020-01289-9
Halliday FW, 2018, P ROY SOC B-BIOL SCI, V285, DOI 10.1098/rspb.2018.2075
Halliday FW, 2017, P ROY SOC B-BIOL SCI, V284, DOI 10.1098/rspb.2017.1340
Halliday FW, 2017, ECOL LETT, V20, P1285, DOI 10.1111/ele.12825
Hassani MA, 2018, MICROBIOME, V6, DOI 10.1186/s40168-018-0445-0
Hood ME, 2003, AM NAT, V162, P122, DOI 10.1086/375539
Hopkins SR, 2017, TRENDS PARASITOL, V33, P53, DOI 10.1016/j.pt.2016.10.003
Johnson PTJ, 2015, ECOL LETT, V18, P1119, DOI 10.1111/ele.12479
Johnson PTJ, 2012, P NATL ACAD SCI USA, V109, P9006, DOI 10.1073/pnas.1201790109
Karvonen A, 2019, TRENDS PARASITOL, V35, P109, DOI 10.1016/j.pt.2018.11.007
Larimer AL, 2010, SYMBIOSIS, V51, P139, DOI 10.1007/s13199-010-0083-1
Lenth R., 2020, R package
Leung JM, 2018, PLOS BIOL, V16, DOI 10.1371/journal.pbio.2004108
Li JCA, 2003, SOCIOL METHOD RES, V32, P117, DOI 10.1177/0049124103031004005
Liu Y, 2006, J PHYTOPATHOL, V154, P6, DOI 10.1111/j.1439-0434.2005.01048.x
Lohr JN, 2010, PARASITOLOGY, V137, P1493, DOI 10.1017/S0031182010000296
Marchetto KM, 2018, AM NAT, V191, P173, DOI 10.1086/695316
O'Connell RJ, 2012, NAT GENET, V44, P1060, DOI 10.1038/ng.2372
O'Keeffe KR, 2017, CURR OPIN PLANT BIOL, V38, P78, DOI 10.1016/j.pbi.2017.04.014
O'Keeffe KR., 2021, DRYAD DIGITAL REPOSI, DOI [10.5061/dryad.83bk3j9s0, DOI 10.5061/DRYAD.83BK3J9S0]
Panaccione DG, 2014, FUNCT ECOL, V28, P299, DOI 10.1111/1365-2435.12076
Panka D, 2013, ANN APPL BIOL, V163, P257, DOI 10.1111/aab.12051
Pathirana NUK, 2019, ECOL EVOL, V9, P6220, DOI 10.1002/ece3.5180
POTTER LR, 1982, ANN APPL BIOL, V100, P321, DOI 10.1111/j.1744-7348.1982.tb01945.x

POTTER LR, 1980, ANN APPL BIOL, V94, P11, DOI 10.1111/j.1744-7348.1980.tb03891.x
R Core Team, 2017, R LANG ENV STAT COMP
Rynkiewicz EC, 2015, TRENDS PARASITOL, V31, P212, DOI 10.1016/j.pt.2015.02.005
Saikkonen K, 2016, PLANT MOL BIOL, V90, P665, DOI 10.1007/s11103-015-0399-6
Saikkonen K, 2013, J CHEM ECOL, V39, P962, DOI 10.1007/s10886-013-0310-3
Sait M, 2002, ENVIRON MICROBIOL, V4, P654, DOI 10.1046/j.1462-2920.2002.00352.x
Spoel SH, 2007, P NATL ACAD SCI USA, V104, P18842, DOI 10.1073/pnas.0708139104
Susi H, 2015, NAT COMMUN, V6, DOI 10.1038/ncomms6975
Suzuki SU, 2019, AM NAT, V194, P90, DOI 10.1086/703485
Swanson SJ, 2006, CLIN MICROBIOL REV, V19, P708, DOI 10.1128/CMR.00011-06
Tan JQ, 2012, ECOLOGY, V93, P1164, DOI 10.1890/11-1557.1
Telfer S, 2010, SCIENCE, V330, P243, DOI 10.1126/science.1190333
Tollenaere C, 2016, TRENDS PLANT SCI, V21, P80, DOI 10.1016/j.tplants.2015.10.014
Vaumourin E, 2015, PARASITE VECTOR, V8, DOI 10.1186/s13071-015-1167-9
West SA, 2003, P ROY SOC B-BIOL SCI, V270, P37, DOI 10.1098/rspb.2002.2209
Wintermantel WM, 2008, PHYTOPATHOLOGY, V98, P1340, DOI 10.1094/PHYTO-98-12-1340
WOOTTON JT, 1994, ANNU REV ECOL SYST, V25, P443, DOI 10.1146/annurev.es.25.110194.002303
Zélé F, 2018, NAT COMMUN, V9, DOI 10.1038/s41467-018-06779-w
Zilio G, 2020, J ANIM ECOL, V89, P2367, DOI 10.1111/1365-2656.13302
NR 72
TC 8
Z9 8
U1 0
U2 16
PU ROYAL SOC
PI LONDON
PA 6-9 CARLTON HOUSE TERRACE, LONDON SW1Y 5AG, ENGLAND
SN 0962-8452
EI 1471-2954
J9 P ROY SOC B-BIOL SCI
JI Proc. R. Soc. B-Biol. Sci.
PD AUG 11
PY 2021
VL 288
IS 1956
AR 20211313
DI 10.1098/rspb.2021.1313
PG 10
WC Biology; Ecology; Evolutionary Biology
WE Science Citation Index Expanded (SCI-EXPANDED)
SC Life Sciences & Biomedicine - Other Topics; Environmental Sciences & Ecology; Evolutionary Biology
GA UA5YL

UT WOS:000685237300006
PM 34375557
OA Green Published, Green Submitted
DA 2025-05-07
ER

PT J
AU Sirocko, KT
   Angstmann, H
   Papenmeier, S
   Wagner, C
   Spohn, M
   Indenbirken, D
   Ehrhardt, B
   Kovacevic, D
   Hammer, B
   Svanes, C
   Rabe, KF
   Roeder, T
   Uliczka, K
   Krauss-Etschmann, S
AF Sirocko, Karolina-Theresa
   Angstmann, Hanna
   Papenmeier, Stephanie
   Wagner, Christina
   Spohn, Michael
   Indenbirken, Daniela
   Ehrhardt, Birte
   Kovacevic, Draginja
   Hammer, Barbara
   Svanes, Cecilie
   Rabe, Klaus F.
   Roeder, Thomas
   Uliczka, Karin
   Krauss-Etschmann, Susanne
TI Early-life exposure to tobacco smoke alters airway signaling pathways
   and later mortality in D. melanogaster
SO ENVIRONMENTAL POLLUTION
LA English
DT Article
DE Cigarette smoke; Drosophila melanogaster; Early life; Development;
   Oxidative stress; Adult mortality
ID CIGARETTE-SMOKE; OXIDATIVE STRESS; DROSOPHILA; ASTHMA; EXPRESSION;
LUNG;
   IMPACT; MODEL; 1A1
AB Early life environmental influences such as exposure to cigarette
smoke (CS) can disturb molecular processes of lung development and
thereby increase the risk for later development of chronic respiratory
diseases. Among the latter, asthma and chronic obstructive pulmonary
disease (COPD) are the most common. The airway epithelium plays a key
role in their disease pathophysiology but how CS exposure in early life
influences airway develop-mental pathways and epithelial stress responses
or survival is poorly understood.Using Drosophila melanogaster larvae as
a model for early life, we demonstrate that CS enters the entire larval

airway system, where it activates cyp18a1 which is homologues to human CYP1A1 to metabolize CS-derived polycyclic aromatic hydrocarbons and further induces heat shock protein 70. RNASeq studies of isolated airways showed that CS dysregulates pathways involved in oxidative stress response, innate immune response, xenobiotic and glutathione metabolic processes as well as developmental processes (BMP, FGF signaling) in both sexes, while other pathways were exclusive to females or males. Glutathione S-transferase genes were further validated by qPCR showing upregulation of gstD4, gstD5 and gstD8 in respiratory tracts of females, while gstD8 was downregulated and gstD5 unchanged in males. ROS levels were increased in airways after CS. Exposure to CS further resulted in higher larval mortality, lower larval-pupal transition, and hatching rates in males only as compared to air-exposed controls.Taken together, early life CS induces airway epithelial stress responses and dysregulates pathways involved in the fly's branching morphogenesis as well as in mammalian lung development. CS further affected fitness and development in a highly sex-specific manner.
C1 [Sirocko, Karolina-Theresa; Papenmeier, Stephanie; Wagner, Christina] German Ctr Lung Res DZL, Res Ctr Borstel, Airway Res Ctr North ARCN, Div Invertebrate Models, Borstel, Germany.
[Wagner, Christina; Uliczka, Karin] German Ctr Lung Res DZL, Res Ctr Borstel, Airway Res Ctr North ARCN, Div Innate Immun, Borstel, Germany.
[Spohn, Michael; Indenbirken, Daniela] Leibniz Inst Virol LIV, Technol Platform Next Generat Sequencing, Hamburg, Germany.
[Kovacevic, Draginja; Hammer, Barbara] German Ctr Lung Res DZL, Res Ctr Borstel, Airway Res Ctr North ARCN, DZL Lab Expt Microbiome Res, Borstel, Germany.
[Svanes, Cecilie] Univ Bergen, Ctr Int Hlth, Dept Global Publ Hlth & Primary Care, Bergen, Norway.
[Svanes, Cecilie] Haukeland Hosp, Dept Occupat Med, Bergen, Norway.
[Rabe, Klaus F.] German Ctr Lung Res DZL, Airway Res Ctr North ARCN, LungenClin, Grosshansdorf, Germany.
[Rabe, Klaus F.] Univ Kiel, Airway Res Ctr North ARCN, German Ctr Lung Res DZL, Dept Med, Kiel, Germany.
[Roeder, Thomas] Christian Albrechts Univ Kiel, Inst Zool, Airway Res Ctr North ARCN, German Ctr Lung Res DZL, Kiel, Germany.
[Krauss-Etschmann, Susanne] Christian Albrechts Univ Kiel, Inst Expt Med, Kiel, Germany.
[Angstmann, Hanna; Ehrhardt, Birte; Kovacevic, Draginja; Hammer, Barbara; Uliczka, Karin; Krauss-Etschmann, Susanne] Div Early Origins Chron Lung Dis, Borstel, Germany.
[Krauss-Etschmann, Susanne] Leibniz Lung Ctr, Res Ctr Borstel, Parkallee 1, D-23845 Borstel, Germany.
C3 Leibniz Association; Forschungszentrum Borstel; Leibniz Association; Forschungszentrum Borstel; Leibniz Association; Forschungszentrum Borstel; University of Bergen; University of Bergen; Haukeland University Hospital; Grosshansdorf Hospital; University of Kiel; University of Kiel; University of Kiel; Leibniz Association; Forschungszentrum Borstel
RP Krauss-Etschmann, S (autor correspondente), Leibniz Lung Ctr, Res Ctr Borstel, Parkallee 1, D-23845 Borstel, Germany.
EM skrauss-etschmann@fz-borstel.de
RI Wagner, Christina/M-7796-2018; Rabe, Klaus/AAW-6296-2021; Svanes, Cecilie/N-7556-2017; Roeder, Thomas/B-9016-2011

OI Wagner, Christina/0009-0005-8121-0046; Ehrhardt,
Birte/0000-0002-2332-9752; Angstmann, Hanna/0000-0002-1522-2429;
Roeder,
Thomas/0000-0002-3489-3834
FU Leibniz Science Campus Evolutionary Medicine of the Lung (EvoLUNG);
award of the Balzan Prize; DFG miTarget - The Microbiome as a
Therapeutic Target in Inflammatory Bowel Diseases, Research Unit 5042
FX The work was funded by the Leibniz Science Campus Evolutionary
Medicine
of the Lung (EvoLUNG) and the award of the Balzan Prize to the German
Center for Lung Research (DZL) scientists Erika von Mutius, Klaus F.
Rabe, Werner Seeger and Tobias Welte and the DFG miTarget - The
Microbiome as a Therapeutic Target in Inflammatory Bowel Diseases,
Research Unit 5042.
CR Agabiti N, 1999, EPIDEMIOLOGY, V10, P692, DOI 10.1097/00001648-
199911000-00008
Ahookhosh K, 2020, EUR J PHARM SCI, V145, DOI
10.1016/j.ejps.2020.105233
Ambrocio-Ortiz E, 2020, FRONT GENET, V10, DOI 10.3389/fgene.2019.01307
[Anonymous], 2012, Harmful and potentially harmful constituents in
tobacco products and tobacco smoke; established list.
Baarsma HA, 2017, J EXP MED, V214, P143, DOI 10.1084/jem.20160675
Barnes PJ, 2022, ANTIOXIDANTS-BASEL, V11, DOI 10.3390/antiox11050965
Bonvallot V, 2001, AM J RESP CELL MOL, V25, P515, DOI
10.1165/ajrcmb.25.4.4515
Bradley TJ, 2006, RESP PHYSIOL NEUROBI, V154, P30, DOI
10.1016/j.resp.2006.02.015
Brody JS, 2012, INT J CANCER, V131, P2754, DOI 10.1002/ijc.27829
Camilleri-Carter TL, 2019, J GERONTOL A-BIOL, V74, P1582, DOI
10.1093/gerona/glz154
Cantin Andre M, 2010, Proc Am Thorac Soc, V7, P368, DOI
10.1513/pats.201001-014AW
Cappello F, 2011, PLOS ONE, V6, DOI 10.1371/journal.pone.0028200
Chang WH, 2017, INT J MOL SCI, V18, DOI 10.3390/ijms18112386
Conlon TM, 2020, NATURE, V588, P151, DOI 10.1038/s41586-020-2882-8
Dai X, 2017, J ASTHMA, V54, P662, DOI 10.1080/02770903.2016.1253730
Dong JJ, 2013, INT IMMUNOPHARMACOL, V17, P885, DOI
10.1016/j.intimp.2013.09.003
Edgar R, 2002, NUCLEIC ACIDS RES, V30, P207, DOI 10.1093/nar/30.1.207
El Golli-Bennour E, 2011, TOXICOLOGY, V287, P1, DOI
10.1016/j.tox.2011.06.002
El-Merhie N, 2021, SCI REP-UK, V11, DOI 10.1038/s41598-021-81607-8
Emborski C, 2019, PHILOS T R SOC B, V374, DOI 10.1098/rstb.2018.0181
Erzurum SC, 2016, ANN AM THORAC SOC, V13, pS35, DOI
10.1513/AnnalsATS.201506-385MG
Faramawy MM, 2009, J CRIT CARE, V24, DOI 10.1016/j.jcrc.2009.06.004
Feyereisen R., 2005, P1
Gilliland FD, 2001, AM J RESP CRIT CARE, V163, P429, DOI
10.1164/ajrccm.163.2.2006009
Goh GH, 2021, J EXP BIOL, V224, DOI 10.1242/jeb.233213
Guittard E, 2011, DEV BIOL, V349, P35, DOI 10.1016/j.ydbio.2010.09.023
Herriges M, 2014, DEVELOPMENT, V141, P502, DOI 10.1242/dev.098186
Hukkanen J, 2002, CRIT REV TOXICOL, V32, P391, DOI
10.1080/20024091064273

Islam MS, 2020, INT J ENV RES PUB HE, V17, DOI 10.3390/ijerph17020380
Gray LJ, 2021, FLY, V15, P47, DOI 10.1080/19336934.2021.1896960
Jafari S, 2021, PLOS BIOL, V19, DOI 10.1371/journal.pbio.3001101
Kim JH, 2004, TOXICOL APPL PHARM, V199, P210, DOI 10.1016/j.taap.2003.11.015
Kim JH, 2021, FOOD CHEM TOXICOL, V151, DOI 10.1016/j.fct.2021.112153
Kotini MP, 2019, VASC PHARMACOL, V112, P8, DOI 10.1016/j.vph.2018.11.002
Lemaitre B, 2007, ANNU REV IMMUNOL, V25, P697, DOI 10.1146/annurev.immunol.25.022106.141615
Li HJ, 2022, SCIENCE, V375, P991, DOI 10.1126/science.abk2432
Li YF, 2005, CHEST, V127, P1232, DOI 10.1378/chest.127.4.1232
Linford NJ, 2013, JOVE-J VIS EXP, DOI 10.3791/50068
Liu J, 2019, NAT COMMUN, V10, DOI 10.1038/s41467-019-09843-1
Lodge CJ, 2018, CLIN EXP ALLERGY, V48, P167, DOI 10.1111/cea.13031
Love MI, 2014, GENOME BIOL, V15, DOI 10.1186/s13059-014-0550-8
Magnus MC, 2015, THORAX, V70, P237, DOI 10.1136/thoraxjnl-2014-206438
Manning Gerards, 1993, P609
McDonald JMC, 2021, EVODEVO, V12, DOI 10.1186/s13227-021-00175-0
Mercer Becky A, 2006, Int J Chron Obstruct Pulmon Dis, V1, P137, DOI 10.2147/copd.2006.1.2.137
Milnerowicz H, 2015, EXP TOXICOL PATHOL, V67, P377, DOI 10.1016/j.etp.2015.04.004
Nelson DR, 1996, PHARMACOGENETICS, V6, P1, DOI 10.1097/00008571-199602000-00002
Ochoa-Espinosa A, 2012, CSH PERSPECT BIOL, V4, DOI 10.1101/cshperspect.a008243
Owusu-Ansah E., 2008, A protocol for in vivo detection of reactive oxygen species, P1, DOI [10.1038/nprot.2008.23, DOI 10.1038/NPROT.2008.23]
Parisi Michael, 2011, BMC Research Notes, V4, DOI 10.1186/1756-0500-4-198
Parkash R, 2021, COMP BIOCHEM PHYS A, V258, DOI 10.1016/j.cbpa.2021.110985
Piacentini S, 2013, MOL BIOL REP, V40, P3299, DOI 10.1007/s11033-012-2405-2
Poobalasingam T, 2017, DIS MODEL MECH, V10, P409, DOI 10.1242/dmm.028175
Posgai R, 2009, SCI TOTAL ENVIRON, V408, P439, DOI 10.1016/j.scitotenv.2009.10.008
Prange R, 2018, AGING-US, V10, P2122, DOI 10.18632/aging.101536
PRYOR WA, 1993, ANN NY ACAD SCI, V686, P12, DOI 10.1111/j.1749-6632.1993.tb39148.x
Rayner RE, 2022, CELL BIOCHEM BIOPHYS, V80, P217, DOI 10.1007/s12013-021-01042-4
Roeder T, 2012, ADV EXP MED BIOL, V710, P37, DOI 10.1007/978-1-4419-5638-5_5
Roeder T, 2009, AM J RESP CRIT CARE, V179, P979, DOI 10.1164/rccm.200811-1777PP
Sadiq MFG, 2014, DRUG CHEM TOXICOL, V37, P163, DOI 10.3109/01480545.2013.834355
Schittny JC, 2017, CELL TISSUE RES, V367, P427, DOI 10.1007/s00441-016-2545-0

Somborac-Bacura A, 2018, EXP PHYSIOL, V103, P1666, DOI 10.1113/EP087038
Stading R, 2021, SEMIN CANCER BIOL, V76, P3, DOI 10.1016/j.semcancer.2021.07.001
Svanes C, 2017, INT J EPIDEMIOL, V46, P235, DOI 10.1093/ije/dyw151
Tong W, 2000, Respirology, V5, P227, DOI 10.1046/j.1440-1843.2000.00267.x
Uchiyama T, 2016, MUTAGENESIS, V31, P9, DOI 10.1093/mutage/gev051
Uv A, 2003, TRENDS CELL BIOL, V13, P301, DOI 10.1016/S0962-8924(03)00083-7
Velazquez-Ulloa NA, 2017, PLOS ONE, V12, DOI 10.1371/journal.pone.0177710
Vitenberga Z, 2019, MEDICINA-LITHUANIA, V55, DOI 10.3390/medicina55080496
Wagner C, 2021, CELL REP, V35, DOI 10.1016/j.celrep.2021.108956
Wang YW, 2018, DEV BIOL, V437, P75, DOI 10.1016/j.ydbio.2018.02.019
Wardlaw SA, 1998, CARCINOGENESIS, V19, P655, DOI 10.1093/carcin/19.4.655
Wat LW, 2021, ELIFE, V10, DOI [10.7554/eLife.72350, 10.7554/eLife.72350.sa1, 10.7554/eLife.72350.sa2]
Wingen A, 2017, DEV BIOL, V430, P52, DOI 10.1016/j.ydbio.2017.08.018
Wohak LE, 2016, ARCH TOXICOL, V90, P291, DOI 10.1007/s00204-014-1409-1
Yang JS, 2009, J GERONTOL A-BIOL, V64, P828, DOI 10.1093/gerona/glp054
NR 76
TC 4
Z9 5
U1 1
U2 11
PU ELSEVIER SCI LTD
PI London
PA 125 London Wall, London, ENGLAND
SN 0269-7491
EI 1873-6424
J9 ENVIRON POLLUT
JI Environ. Pollut.
PD SEP 15
PY 2022
VL 309
AR 119696
DI 10.1016/j.envpol.2022.119696
EA JUL 2022
PG 12
WC Environmental Sciences
WE Science Citation Index Expanded (SCI-EXPANDED)
SC Environmental Sciences & Ecology
GA 3F7ZN
UT WOS:000830882600011
PM 35780997
DA 2025-05-07
ER

PT J
AU Coomber, A
Saville, A

Ristaino, JB
AF Coomber, Allison
Saville, Amanda
Ristaino, Jean Beagle
TI Evolution of Phytophthora infestans on its potato host since the Irish potato famine
SO NATURE COMMUNICATIONS
LA English
DT Article
ID LATE BLIGHT-RESISTANCE; SOLANUM-TUBEROSUM; CHLOROPLAST DNA; GENOME SEQUENCE; EFFECTOR AVR3A; RXLR EFFECTORS; R-GENES; EXPRESSION; PATHOGEN; PLANT
AB Phytophthora infestans is a major oomycete plant pathogen, responsible for potato late blight, which led to the Irish Potato Famine from 1845-1852. Since then, potatoes resistant to this disease have been bred and deployed worldwide. Their resistance (R) genes recognize pathogen effectors responsible for virulence and then induce a plant response stopping disease progression. However, most deployed R genes are quickly overcome by the pathogen. We use targeted sequencing of effector and R genes on herbarium specimens to examine the joint evolution in both P. infestans and potato from 1845-1954. Currently relevant effectors are historically present in P. infestans, but with alternative alleles compared to modern reference genomes. The historic FAM-1 lineage has the virulent Avr1 allele and the ability to break the R1 resistance gene before breeders deployed it in potato. The FAM-1 lineage is diploid, but later, triploid US-1 lineages appear. We show that pathogen virulence genes and host resistance genes have undergone significant changes since the Famine, from both natural and artificial selection.
C1 [Coomber, Allison; Saville, Amanda; Ristaino, Jean Beagle] NC State Univ, Dept Entomol & Plant Pathol, Raleigh, NC 27695 USA.
[Coomber, Allison] NC State Univ, Funct Genom Program, Raleigh, NC USA.
[Ristaino, Jean Beagle] NC State Univ, Emerging Plant Dis & Global Food Secur Cluster, Raleigh, NC 27695 USA.
C3 North Carolina State University; North Carolina State University; North Carolina State University
RP Ristaino, JB (autor correspondente), NC State Univ, Dept Entomol & Plant Pathol, Raleigh, NC 27695 USA.; Ristaino, JB (autor correspondente), NC State Univ, Emerging Plant Dis & Global Food Secur Cluster, Raleigh, NC 27695 USA.
EM jean_ristaino@ncsu.edu
RI ; Ristaino, Jean/N-3616-2018
OI Coomber, Allison/0009-0006-3546-9388; Ristaino, Jean/0000-0002-9458-0514
FU North Carolina State University (NC State University) [2018-1966, 5572998]; Seed Grant from the Triangle Center for Evolutionary Medicine [5572998]
FX Funding for salaries for AC provided by NSF AgBioFews Training Grant Number, 2018-1966, JR Co-PI and salaries for AS, sequencing and supplies funded by Grip4PSI Grant Number 5572998, JR Co-PI, respectively.

Additional research funds for AC work from a Seed Grant from the Triangle Center for Evolutionary Medicine and Grip4PSI Grant Number 5572998 JR Co-PI. Appreciation is expressed to Dr. Ross Whetten, Dr. Justin Whitehill, Dr. David Rasmussen, Dr. Jeff Thorne and Dr. Ignazio Carbone from NC State University, Ingo Hein, James Hutton Institute and
Dr. Michael Martin, Norwegian Institute of Science and Technology for advice on the computational methods and workflows used to analyze data in this work.
CR Ames M, 2008, AM J BOT, V95, P252, DOI 10.3732/ajb.95.2.252
Andersen EJ, 2018, GENES-BASEL, V9, DOI 10.3390/genes9070339
Andrews S., 2010, FASTQC QUALITY CONTR
[Anonymous], 2013, Transl. Genomics Crop Breed.: |Biot. Stress
Armstrong MR, 2005, P NATL ACAD SCI USA, V102, P7766, DOI 10.1073/pnas.0500113102
Ballvora A, 2002, PLANT J, V30, P361, DOI 10.1046/j.1365-313X.2001.01292.x
Berkeley J.M., 1846, J HORT SOC LOND, V1, P9
BLACK W., 1953, EUPHYTICA, V2, P173, DOI 10.1007/BF00053724
BLACK W., 1951, PROC ROY SOC EDINBURGH, V64B, P312
Bos JIB, 2009, MOL PLANT MICROBE IN, V22, P269, DOI 10.1094/MPMI-22-3-0269
Bourke A., 1993, The visitation of god? The potato and the great Irish famine
Bradshaw JE, 2006, THEOR APPL GENET, V112, P744, DOI 10.1007/s00122-005-0179-9
Broad Institute, 2019, Picard Toolkit
Camacho C, 2009, BMC BIOINFORMATICS, V10, DOI 10.1186/1471-2105-10-421
Cingolani P, 2012, FLY, V6, P80, DOI 10.4161/fly.19695
Danecek P, 2021, GIGASCIENCE, V10, DOI 10.1093/gigascience/giab008
De Bary A., 1876, J. Bot. Paris, V14, P105
Du Y, 2018, STUD MYCOL, P85, DOI 10.1016/j.simyco.2018.01.003
Edgar RC, 2004, NUCLEIC ACIDS RES, V32, P1792, DOI 10.1093/nar/gkh340
Ellson J, 2004, MATH VIS, P127
Ewels P, 2016, BIOINFORMATICS, V32, P3047, DOI 10.1093/bioinformatics/btw354
Flor HH, 1942, PHYTOPATHOLOGY, V32, P653
Foster SJ, 2009, MOL PLANT MICROBE IN, V22, P589, DOI 10.1094/MPMI-22-5-0589
Fry WE, 2015, PHYTOPATHOLOGY, V105, P966, DOI 10.1094/PHYTO-01-15-0005-FI
Fry W, 2008, MOL PLANT PATHOL, V9, P385, DOI [10.1111/j.1364-3703.2007.00465.x, 10.1111/J.1364-3703.2007.00465.X]
Ghislain M, 2019, PLANT BIOTECHNOL J, V17, P1119, DOI 10.1111/pbi.13042
Gilroy EM, 2011, NEW PHYTOL, V191, P763, DOI 10.1111/j.1469-8137.2011.03736.x
GLENDINNING DR, 1983, NEW PHYTOL, V94, P479, DOI 10.1111/j.1469-8137.1983.tb03460.x
Goluch T, 2020, METHODS ECOL EVOL, V11, P494, DOI 10.1111/2041-210X.13358
Haas BJ, 2009, NATURE, V461, P393, DOI 10.1038/nature08358
Hosaka K, 2002, AM J POTATO RES, V79, P119, DOI 10.1007/BF02881520

Huang SW, 2005, PLANT J, V42, P251, DOI 10.1111/j.1365-313X.2005.02365.x
Ignatieva A, 2021, BIOINFORMATICS, V37, P3277, DOI 10.1093/bioinformatics/btab351
Jónsson H, 2013, BIOINFORMATICS, V29, P1682, DOI 10.1093/bioinformatics/btt193
Jupe F, 2013, PLANT J, V76, P530, DOI 10.1111/tpj.12307
Knaus BJ, 2020, MBIO, V11, DOI 10.1128/mBio.00326-20
Krueger F., 2015, Babraham Bioinformatics6.10 (Version 0.6.10), DOI DOI 10.1093/BIOINFORMATICS/BTU170
Larsson A, 2014, BIOINFORMATICS, V30, P3276, DOI 10.1093/bioinformatics/btu531
Leesutthiphonchai W, 2018, PHYTOPATHOLOGY, V108, P916, DOI 10.1094/PHYTO-04-18-0130-IA
Li GC, 2011, MOL PLANT MICROBE IN, V24, P1132, DOI 10.1094/MPMI-11-10-0276
Li H., 2013, arXiv, DOI [DOI 10.48550/ARXIV.1303.3997, 10.48550/arXiv.1303.3997]
Lin X, 2020, MOL PLANT PATHOL, V21, P1502, DOI 10.1111/mpp.12987
Lindley J., 1848, J. R. Hort. Soc, V3, P65
Lokossou AA, 2009, MOL PLANT MICROBE IN, V22, P630, DOI 10.1094/MPMI-22-6-0630
Maddison W., 2007, MESQUITE 2 MODULAR S
MALCOLMSON JF, 1966, EUPHYTICA, V15, P199, DOI 10.1007/BF00022324
Martin M., 2011, Embnet. J., V17, P10, DOI [DOI 10.14806/EJ.17.1.200, 10.14806/ej.17.1.200]
Martin MD, 2016, MOL BIOL EVOL, V33, P478, DOI 10.1093/molbev/msv241
Martin MD, 2013, NAT COMMUN, V4, DOI 10.1038/ncomms3172
Matson MEH, 2022, PLOS PATHOG, V18, DOI 10.1371/journal.ppat.1010869
May KJ, 2004, MYCOL RES, V108, P471, DOI 10.1017/s0953756204009876
Nowicki M, 2012, PLANT DIS, V96, P4, DOI 10.1094/PDIS-05-11-0458
Oh SK, 2009, PLANT CELL, V21, P2928, DOI 10.1105/tpc.109.068247
Orlando L, 2021, NAT REV METHOD PRIME, V1, DOI 10.1038/s43586-020-00011-0
Paluchowska P, 2022, PLANTA, V255, DOI 10.1007/s00425-022-03910-6
Pel M. A., 2010, Mapping, isolation and characterization of genes responsible for late blight resistance in potato
POWELL W, 1993, ANN APPL BIOL, V123, P403, DOI 10.1111/j.1744-7348.1993.tb04102.x
Prjibelski Andrey, 2020, Curr Protoc Bioinformatics, V70, pe102, DOI 10.1002/cpbi.102
Quinlan Aaron R, 2014, Curr Protoc Bioinformatics, V47, DOI 10.1002/0471250953.bi1112s47
Reddick D., 1940, Chronica botanica, V6, P73
Reddick D., 1938, American Potato Journal, V15, P29, DOI 10.1007/BF02895549
Reddick D, 1930, PHYTOPATHOLOGY, V20, P987
Rietman H, 2012, MOL PLANT MICROBE IN, V25, P910, DOI 10.1094/MPMI-01-12-0010-R
Ristaino JB, 2002, MICROBES INFECT, V4, P1369, DOI 10.1016/S1286-4579(02)00010-2
Ristaino JB, 2001, NATURE, V411, P695, DOI 10.1038/35079606
Ristaino JB, 2020, EMERGING PLANT DIS G, P101, DOI DOI 10.1094/9780890546383.006

   Ristaino JB, 2020, FRONT ECOL EVOL, V7, DOI 10.3389/fevo.2019.00521
   Salaman R., 1949, Potato Varieties, Past Present and Future. The History and Social Influence of the Potato
   Salaman R.N., 1926, Potato Varieties
   Saville AC, 2021, SCI REP-UK, V11, DOI 10.1038/s41598-021-90937-6
   Stamatakis A, 2014, BIOINFORMATICS, V30, P1312, DOI 10.1093/bioinformatics/btu033
   Stefanczyk E, 2017, PHYTOPATHOLOGY, V107, P740, DOI 10.1094/PHYTO-09-16-0328-R
   Stewart HE, 2003, PLANT PATHOL, V52, P193, DOI 10.1046/j.1365-3059.2003.00811.x
   Sukumaran J, 2010, BIOINFORMATICS, V26, P1569, DOI 10.1093/bioinformatics/btq228
   Thilliez GJA, 2019, NEW PHYTOL, V221, P1634, DOI 10.1111/nph.15441
   Van der Auwera GA, 2020, Genomics in the Cloud: Using Docker, GATK, and WDL in Terra, V1st, DOI DOI 10.1093/BIOINFORMATICS/BTS480
   van der Vossen EAG, 2005, PLANT J, V44, P208, DOI 10.1111/j.1365-313X.2005.02527.x
   van Poppel PMJA, 2008, MOL PLANT MICROBE IN, V21, P1460, DOI 10.1094/MPMI-21-11-1460
   van Poppel PMJA, 2009, PHYTOPATHOLOGY, V99, P1150, DOI 10.1094/PHYTO-99-10-1150
   Vleeshouwers VGAA, 2011, ANNU REV PHYTOPATHOL, V49, P507, DOI 10.1146/annurev-phyto-072910-095326
   Vleeshouwers VGAA, 2008, PLOS ONE, V3, DOI 10.1371/journal.pone.0002875
   Vossen JH, 2016, THEOR APPL GENET, V129, P1785, DOI 10.1007/s00122-016-2740-0
   Wawra S, 2017, PLANT CELL, V29, P1184, DOI 10.1105/tpc.16.00552
   Weiss CL, 2018, BMC BIOINFORMATICS, V19, DOI 10.1186/s12859-018-2128-z
   Win J, 2007, PLANT CELL, V19, P2349, DOI 10.1105/tpc.107.051037
   Witek K, 2016, NAT BIOTECHNOL, V34, P656, DOI 10.1038/nbt.3540
   Xu X, 2011, NATURE, V475, P189, DOI 10.1038/nature10158
   Yang LN, 2020, MOL PLANT MICROBE IN, V33, P921, DOI 10.1094/MPMI-07-19-0179-R
   Yoshida K, 2013, ELIFE, V2, DOI 10.7554/eLife.00731
NR 89
TC 1
Z9 1
U1 16
U2 24
PU NATURE PORTFOLIO
PI BERLIN
PA HEIDELBERGER PLATZ 3, BERLIN, 14197, GERMANY
EI 2041-1723
J9 NAT COMMUN
JI Nat. Commun.
PD AUG 5
PY 2024
VL 15
IS 1
AR 6488
DI 10.1038/s41467-024-50749-4
PG 12

WC Multidisciplinary Sciences
WE Science Citation Index Expanded (SCI-EXPANDED)
SC Science & Technology - Other Topics
GA A8E6X
UT WOS:001284820100030
PM 39103347
OA gold, Green Published
DA 2025-05-07
ER

PT J
AU Nikolayevskyy, V
   Niemann, S
   Anthony, R
   van Soolingen, D
   Tagliani, E
   Ködmön, C
   van der Werf, MJ
   Cirillo, DM
AF Nikolayevskyy, V.
   Niemann, S.
   Anthony, R.
   van Soolingen, D.
   Tagliani, E.
   Kodmon, C.
   van der Werf, M. J.
   Cirillo, D. M.
TI Role and value of whole genome sequencing in studying tuberculosis
   transmission
SO CLINICAL MICROBIOLOGY AND INFECTION
LA English
DT Review
DE Outbreak; Standardization; Transmission; Tuberculosis; Whole genome
   sequencing
ID DRUG-RESISTANT TUBERCULOSIS; MYCOBACTERIUM-TUBERCULOSIS; OUTBREAKS;
   SURVEILLANCE; MANAGEMENT; EVOLUTION; SETTINGS; BURDEN; EUROPE; TB
AB Background: Tuberculosis (TB) remains a serious public health threat worldwide. Theoretically ultimate resolution of whole genome sequencing (WGS) for Mycobacterium tuberculosis complex (MTBC) strain classification makes this technology very attractive for epidemiological investigations.
   Objectives: To summarize the evidence available in peer-reviewed publications on the role and place of WGS in detection of TB transmission.
   Sources: A total of 69 peer-reviewed publications identified in Pubmed database.
   Content: Evidence from >30 publications suggests that a cut-off value of fewer than six single nucleotide polymorphisms between strains efficiently excludes cases that are not the result of recent transmission and could be used for the identification of drug-sensitive isolates involved in direct human-to-human TB transmission. Sensitivity of WGS to identify epidemiologically linked isolates is high, reaching 100% in eight studies with specificity (17%-95%) highly dependent on the settings. Drug resistance and specific phylogenetic lineages may be associated with accelerated mutation rates affecting genetic distances.

WGS can be potentially used to distinguish between true relapses and re-infections but in high-incidence low-diversity settings this would require consideration of epidemiological links and minority alleles. Data from four studies looking into within-host diversity highlight a need for developing criteria for acceptance or rejection of WGS relatedness results depending on the proportion of minority alleles. Crown Copyright (C) 2019 Published by Elsevier Ltd on behalf of European Society of Clinical Microbiology and Infectious Diseases. All rights reserved.
C1 [Nikolayevskyy, V.] Publ Hlth England, London, England.
[Nikolayevskyy, V.] Imperial Coll, London, England.
[Niemann, S.] Natl Reference Ctr Mycobacteria, Res Ctr, Mol & Expt Mycobacteriol, Borstel, Germany.
[Niemann, S.] German Ctr Infect Res, Borstel Site, Braunschweig, Germany.
[Anthony, R.; van Soolingen, D.] Natl Inst Publ Hlth & Environm, TB Reference Lab, Infect Dis Res Diagnost & Lab Surveillance, Bilthoven, Netherlands.
[Tagliani, E.; Cirillo, D. M.] IRCCS San Raffaele Sci Inst, Div Immunol Transplantat & Infect Dis, Emerging Bacterial Pathogens Unit, Milan, Italy.
[Kodmon, C.; van der Werf, M. J.] European Ctr Dis Prevent & Control, Stockholm, Sweden.
C3 Public Health England; Imperial College London; Leibniz Association; Forschungszentrum Borstel; German Center for Infection Research; Netherlands National Institute for Public Health & the Environment; Vita-Salute San Raffaele University; IRCCS Ospedale San Raffaele; European Centre for Disease Prevention & Control
RP Nikolayevskyy, V (autor correspondente), Natl Mycobacterium Reference Serv South, 61 Colindale Ave, London NW9 5EQ, England.
EM vlad.nikolayevskyy@phe.gov.uk
RI Ködmön, Csaba/AAP-8247-2021; Cirillo, Daniela/AAB-3646-2019; Tagliani, Elisa/AAN-1955-2020; Niemann, Stefan/C-9327-2011; Nikolayevskyy, Vladyslav/HFZ-7738-2022; Anthony, Richard/ACG-9717-2022; Anthony, Richard/G-5654-2010
OI Anthony, Richard/0000-0002-0550-5273; Kodmon, Csaba/0000-0001-8040-6753;
Niemann, Stefan/0000-0002-6604-0684
FU European Centre for Disease Control within EUSeqMyTB project; German Centre for Infection Research, Deutsche Forschungsgemeinschaft (German Research Foundation) under Germany's Excellence Strategy [EXC 22167-390884018]; Leibniz Science Campus EvoLUNG (Evolutionary medicine of the lung)
FX The authors declare no conflicts of interests related to this study. This study has received funding from European Centre for Disease Control within EUSeqMyTB project. Stefan Niemann received funding from the German Centre for Infection Research, Deutsche Forschungsgemeinschaft (German Research Foundation) under Germany's Excellence Strategy (Grant EXC 22167-390884018) and from Leibniz Science Campus EvoLUNG (Evolutionary medicine of the lung).
CR Allix-Béguec C, 2018, NEW ENGL J MED, V379, P1403, DOI 10.1056/NEJMoa1800474

[Anonymous], 2018, TUB SURV MON EUR 201
Arnold A, 2016, J INFECTION, V73, P210, DOI 10.1016/j.jinf.2016.04.037
Bang D., 2010, DANISH MED B, V57, P1
Barr DA, 2017, QJM-INT J MED, V110, P611, DOI 10.1093/qjmed/hcx115
Bjorn-Mortensen K, 2016, SCI REP-UK, V6, DOI 10.1038/srep33180
Brown AC, 2015, J CLIN MICROBIOL, V53, P2230, DOI 10.1128/JCM.00486-15
Bryant JM, 2013, LANCET RESP MED, V1, P786, DOI 10.1016/S2213-2600(13)70231-5
Bryant JM, 2013, BMC INFECT DIS, V13, DOI 10.1186/1471-2334-13-110
Cabibbe AM, 2018, EUR RESPIR J, V51, DOI 10.1183/13993003.00387-2018
Casali N, 2016, PLOS MED, V13, DOI 10.1371/journal.pmed.1002137
Casali N, 2014, NAT GENET, V46, P279, DOI 10.1038/ng.2878
de Beer JL, 2014, INT J TUBERC LUNG D, V18, P594, DOI 10.5588/ijtld.13.0531
Dheda K, 2017, LANCET RESP MED, V5, P291, DOI [10.1016/S2213-2600(17)30079-6, 10.1016/s2213-2600(17)30079-6]
Eldholm V, 2016, ELIFE, V5, DOI 10.7554/eLife.16644
Ford C, 2012, TUBERCULOSIS, V92, P194, DOI 10.1016/j.tube.2011.11.003
Ford CB, 2011, NAT GENET, V43, P482, DOI 10.1038/ng.811
Fox GJ, 2013, EUR RESPIR J, V41, P140, DOI 10.1183/09031936.00070812
Gardy JL, 2011, NEW ENGL J MED, V364, P730, DOI 10.1056/NEJMoa1003176
Guerra-Assunçao JA, 2015, ELIFE, V4, DOI 10.7554/eLife.05166
Gurjav U, 2016, PLOS ONE, V11, DOI 10.1371/journal.pone.0163612
Hatherell HA, 2016, BMC MED, V14, DOI 10.1186/s12916-016-0566-x
Jajou R., 2018, J CLIN MICROBIOL, V56
Jajou R, 2018, PLOS ONE, V13, DOI 10.1371/journal.pone.0195413
Kato-Maeda M, 2013, PLOS ONE, V8, DOI 10.1371/journal.pone.0058235
Köser CU, 2012, PLOS PATHOG, V8, DOI 10.1371/journal.ppat.1002824
Kohl TA, 2014, J CLIN MICROBIOL, V52, P2479, DOI 10.1128/JCM.00567-14
Kontsevaya I, 2017, INFECT GENET EVOL, V48, P76, DOI 10.1016/j.meegid.2016.12.016
Lange C, 2018, J INTERN MED, V284, P163, DOI 10.1111/joim.12780
Lee RS, 2015, P NATL ACAD SCI USA, V112, P13609, DOI 10.1073/pnas.1507071112
Luo T, 2014, TUBERCULOSIS, V94, P434, DOI 10.1016/j.tube.2014.04.005
Macedo R, 2018, TUBERCULOSIS, V110, P44, DOI 10.1016/j.tube.2018.03.009
Mahomed S, 2017, TUBERCULOSIS, V107, P137, DOI 10.1016/j.tube.2017.09.005
Mears J, 2016, THORAX, V71, P734, DOI 10.1136/thoraxjnl-2014-206480
Mehaffy C, 2014, PLOS ONE, V9, DOI 10.1371/journal.pone.0112928
Merker M, 2013, PLOS ONE, V8
Merker M, 2017, ADV EXP MED BIOL, V1019, P43, DOI 10.1007/978-3-319-64371-7_3
Merker M, 2015, NAT GENET, V47, P242, DOI 10.1038/ng.3195
Mustazzolu A, 2018, EUR RESPIR J, V52, DOI 10.1183/13993003.00070-2018
Niemann S, 2014, CSH PERSPECT MED, V4, DOI 10.1101/cshperspect.a021188
Nikolayevskyy V, 2016, TUBERCULOSIS, V98, P77, DOI 10.1016/j.tube.2016.02.009
Norheim G, 2017, J CLIN MICROBIOL, V55, P1327, DOI 10.1128/JCM.01152-16
Pankhurst LJ, 2016, LANCET RESP MED, V4, P49, DOI 10.1016/S2213-2600(15)00466-X

Pavlenko E, 2018, INT J TUBERC LUNG D, V22, P197, DOI 10.5588/ijtld.17.0254
Phelan JE, 2016, BMC GENOMICS, V17, DOI 10.1186/s12864-016-2467-y
Popovici O, 2018, EPIDEMIOL INFECT, V146, P824, DOI 10.1017/S095026881800047X
Revez J, 2017, FRONT PUBLIC HEALTH, V5, DOI 10.3389/fpubh.2017.00347
Roetzer A, 2013, PLOS MED, V10, DOI 10.1371/journal.pmed.1001387
Schürch AC, 2010, J CLIN MICROBIOL, V48, P3403, DOI 10.1128/JCM.00370-10
Senghore M, 2017, PLOS ONE, V12, DOI 10.1371/journal.pone.0184510
Shah NS, 2017, NEW ENGL J MED, V376, P243, DOI 10.1056/NEJMoa1604544
Stucki D, 2016, J CLIN MICROBIOL, V54, P1862, DOI 10.1128/JCM.00126-16
Supply P, 2018, EBIOMEDICINE, V36, P14, DOI 10.1016/j.ebiom.2018.08.052
Tagliani E, 2018, LANCET INFECT DIS, V18, P377, DOI 10.1016/S1473-3099(18)30132-4
Tagliani E, 2017, SCI REP-UK, V7, DOI 10.1038/s41598-017-17705-3
Török ME, 2013, J CLIN MICROBIOL, V51, P611, DOI 10.1128/JCM.02279-12
van der Werf MJ, 2017, CLIN MICROBIOL INFEC, V23, DOI 10.1016/j.cmi.2017.02.017
Walker TM, 2017, CLIN MICROBIOL INFEC, V23, P161, DOI 10.1016/j.cmi.2016.10.014
Walker TM, 2016, QJM-INT J MED, V109, P819, DOI 10.1093/qjmed/hcw202
Walker TM, 2013, CLIN MICROBIOL INFEC, V19, P796, DOI 10.1111/1469-0691.12183
Walker TM, 2018, LANCET INFECT DIS, V18, P431, DOI 10.1016/S1473-3099(18)30004-5
Walker TM, 2015, LANCET INFECT DIS, V15, P1193, DOI 10.1016/S1473-3099(15)00062-6
Walker TM, 2014, LANCET RESP MED, V2, P285, DOI 10.1016/S2213-2600(14)70027-X
Walker TM, 2013, LANCET INFECT DIS, V13, P137, DOI 10.1016/S1473-3099(12)70277-3
Witney AA, 2016, BMC MED, V14, DOI 10.1186/s12916-016-0598-2
Witney AA, 2015, J CLIN MICROBIOL, V53, P1473, DOI 10.1128/JCM.02993-14
Wollenberg KR, 2017, J CLIN MICROBIOL, V55, P457, DOI 10.1128/JCM.02116-16
Wyllie DH, 2018, EBIOMEDICINE, V34, P122, DOI 10.1016/j.ebiom.2018.07.019
Wyllie DH, 2018, J CLIN MICROBIOL, V56, DOI [10.1128/JCM.00104-18, 10.1128/jcm.00104-18]
NR 69
TC 55
Z9 58
U1 0
U2 14
PU ELSEVIER SCI LTD
PI OXFORD
PA THE BOULEVARD, LANGFORD LANE, KIDLINGTON, OXFORD OX5 1GB, OXON, ENGLAND
SN 1198-743X
EI 1469-0691
J9 CLIN MICROBIOL INFEC

JI Clin. Microbiol. Infect.
PD NOV
PY 2019
VL 25
IS 11
BP 1377
EP 1382
DI 10.1016/j.cmi.2019.03.022
PG 6
WC Infectious Diseases; Microbiology
WE Science Citation Index Expanded (SCI-EXPANDED)
SC Infectious Diseases; Microbiology
GA JH3QX
UT WOS:000492683600013
PM 30980928
OA Bronze
DA 2025-05-07
ER

PT J
AU Andres, S
   Merker, M
   Heyckendorf, J
   Kalsdorf, B
   Rumetshofer, R
   Indra, A
   Hofmann-Thiel, S
   Hoffmann, H
   Lange, C
   Niemann, S
   Maurer, FP
AF Andres, Soenke
   Merker, Matthias
   Heyckendorf, Jan
   Kalsdorf, Barbara
   Rumetshofer, Rudolf
   Indra, Alexander
   Hofmann-Thiel, Sabine
   Hoffmann, Harald
   Lange, Christoph
   Niemann, Stefan
   Maurer, Florian P.
TI Bedaquiline-Resistant Tuberculosis: Dark Clouds on the Horizon
SO AMERICAN JOURNAL OF RESPIRATORY AND CRITICAL CARE MEDICINE
LA English
DT Letter
C1 [Andres, Soenke; Merker, Matthias; Heyckendorf, Jan; Kalsdorf, Barbara; Lange, Christoph; Niemann, Stefan; Maurer, Florian P.] Res Ctr Borstel, Borstel, Germany.
   [Merker, Matthias; Heyckendorf, Jan; Kalsdorf, Barbara; Lange, Christoph; Niemann, Stefan] German Ctr Infect Res, Borstel, Germany.
   [Rumetshofer, Rudolf] Otto Wagner Hosp, Vienna, Austria.
   [Indra, Alexander] Austrian Agcy Hlth & Food Safety AGES, Vienna, Austria.

[Hofmann-Thiel, Sabine; Hoffmann, Harald] World Hlth Org Supranat Reference Lab TB Munich, SYNLAB, Gauting, Germany.
[Hofmann-Thiel, Sabine; Hoffmann, Harald] SYNLAB Human Genet Munich, Gauting, Germany.
[Lange, Christoph] Univ Lubeck, Lubeck, Germany.
[Lange, Christoph] Karolinska Inst, Stockholm, Sweden.
[Maurer, Florian P.] Univ Med Ctr Hamburg Eppendorf, Hamburg, Germany.
C3 Leibniz Association; Forschungszentrum Borstel; German Center for Infection Research; SYNLAB Group; SYNLAB Group; University of Lubeck; Karolinska Institutet; University of Hamburg; University Medical Center Hamburg-Eppendorf
RP Maurer, FP (autor correspondente), Res Ctr Borstel, Borstel, Germany.; Maurer, FP (autor correspondente), Univ Med Ctr Hamburg Eppendorf, Hamburg, Germany.
EM fmaurer@fz-borstel.de
RI Niemann, Stefan/C-9327-2011; Heyckendorf, Jan/AAA-8782-2021; Maurer, Florian/ABA-1918-2020; Merker, Matthias/P-7399-2015; Lange, Christoph/J-6289-2012; Maurer, Florian/J-9687-2014
OI Merker, Matthias/0000-0003-1386-2331; Lange, Christoph/0000-0002-9691-4741; Maurer, Florian/0000-0001-8353-5964; Niemann, Stefan/0000-0002-6604-0684
FU German Center for Infection Research (DZIF); Cluster of Excellence "Precision Medicine in Chronic Inflammation"; Leibniz Science Campus Evolutionary Medicine of the Lung (EvoLUNG); Joachim Herz Foundation
FX Supported by internal funding. In addition, C.L., J.H., S.N., and M.M. are supported by the German Center for Infection Research (DZIF); B.K., S.N., and C.L. are supported by the Cluster of Excellence "Precision Medicine in Chronic Inflammation"; S.N. and M.M. are supported by the Leibniz Science Campus Evolutionary Medicine of the Lung (EvoLUNG); and F.P.M. is supported by a research grant from Joachim Herz Foundation.
CR Andries K, 2005, SCIENCE, V307, P223, DOI 10.1126/science.1106753
Andries K, 2014, PLOS ONE, V9, DOI 10.1371/journal.pone.0102135
de Vos M, 2019, NEW ENGL J MED, V380, P2178, DOI 10.1056/NEJMc1815121
Diacon AH, 2014, NEW ENGL J MED, V371, P723, DOI 10.1056/NEJMoa1313865
European Committee on Antimicrobial Susceptibility Testing (EUCAST), 2019, BED RAT EUC CLIN BRE
Ghodousi A, 2019, ANTIMICROB AGENTS CH, V63
Köser CU, 2019, INT J INFECT DIS, V80, pS32, DOI 10.1016/j.ijid.2019.02.027
Kranzer K, 2019, AM J RESP CRIT CARE, V200, P514, DOI 10.1164/rccm.201902-0260LE
Merker M, 2018, ELIFE, V7, DOI 10.7554/eLife.38200
Nahid P, 2019, AM J RESP CRIT CARE, V200, pE93, DOI 10.1164/rccm.201909-1874ST
Polsfuss S, 2019, CLIN INFECT DIS, V69, P1229, DOI 10.1093/cid/ciz074
Schnippel K, 2018, LANCET RESP MED, V6, P699, DOI 10.1016/S2213-2600(18)30235-2
World Health Organization, 2023, Global tuberculosis report 2019. 283, DOI DOI 10.2471/BLT.21.286807
World Health Organization, 2020, WHO CONS GUID DRUG R, DOI 10.30978/TB2021-2-86

   World Health Organization (WHO), 2018, TECHNICAL REPORT CRI
NR 15
TC 55
Z9 60
U1 1
U2 13
PU AMER THORACIC SOC
PI NEW YORK
PA 25 BROADWAY, 18 FL, NEW YORK, NY 10004 USA
SN 1073-449X
EI 1535-4970
J9 AM J RESP CRIT CARE
JI Am. J. Respir. Crit. Care Med.
PD JUN 15
PY 2020
VL 201
IS 12
BP 1564
EP 1568
DI 10.1164/rccm.201909-1819LE
PG 6
WC Critical Care Medicine; Respiratory System
WE Science Citation Index Expanded (SCI-EXPANDED)
SC General & Internal Medicine; Respiratory System
GA MA3SG
UT WOS:000541836500025
PM 32053752
DA 2025-05-07
ER

PT J
AU Botelho, J
   Cazares, A
   Schulenburg, H
AF Botelho, Joao
   Cazares, Adrian
   Schulenburg, Hinrich
TI The ESKAPE mobilome contributes to the spread of antimicrobial
   resistance and CRISPR-mediated conflict between mobile genetic
elements
SO NUCLEIC ACIDS RESEARCH
LA English
DT Article
ID ANTI-CRISPR; CLASSIFICATION; IDENTIFICATION; RESTRICTION; ANNOTATION;
   DIVERSITY; ALGORITHM; NETWORK; GENOMES; SYSTEMS
AB Mobile genetic elements (MGEs) mediate the shuffling of genes among
organisms. They contribute to the spread of virulence and antibiotic
resistance (AMR) genes in human pathogens, such as the particularly
problematic group of ESKAPE pathogens. Here, we performed the first
systematic analysis of MGEs, including plasmids, prophages, and
integrative and conjugative/mobilizable elements (ICEs/IMEs), across all
ESKAPE pathogens. We found that different MGE types are asymmetrically
distributed across these pathogens, and that most horizontal gene
transfer (HGT) events are restricted by phylum or genus. We show that the

MGEs proteome is involved in diverse functional processes and distinguish widespread proteins within the ESKAPE context. Moreover, anti-CRISPRs and AMR genes are overrepresented in the ESKAPE mobilome. Our results also underscore species-specific trends shaping the number of MGEs, AMR, and virulence genes across pairs of conspecific ESKAPE genomes with and without CRISPR-Cas systems. Finally, we observed that CRISPR spacers found on prophages, ICEs/IMEs, and plasmids have different targeting biases: while plasmid and prophage CRISPRs almost exclusively target other plasmids and prophages, respectively, ICEs/IMEs CRISPRs preferentially target prophages. Overall, our study highlights the general importance of the ESKAPE mobilome in contributing to the spread of AMR and mediating conflict among MGEs.
C1 [Botelho, Joao; Schulenburg, Hinrich] Max Planck Inst Evolutionary Biol, Antibiot Resistance Evolut Grp, Plon, Germany.
[Botelho, Joao; Schulenburg, Hinrich] Univ Kiel, Zool Inst, Dept Evolutionary Ecol & Genet, Kiel, Germany.
[Cazares, Adrian] EMBLs European Bioinformat Inst EMBL EBI, Wellcome Genome Campus, Hinxton, England.
[Cazares, Adrian] Wellcome Sanger Inst, Wellcome Genome Campus, Cambridge, England.
C3 Max Planck Society; University of Kiel; Wellcome Trust Sanger Institute
RP Botelho, J (autor correspondente), Max Planck Inst Evolutionary Biol, Antibiot Resistance Evolut Grp, Plon, Germany.; Botelho, J (autor correspondente), Univ Kiel, Zool Inst, Dept Evolutionary Ecol & Genet, Kiel, Germany.
EM botelho@evolbio.mpg.de
RI Cazares, Adrian/GRS-3471-2022; Botelho, Joao/T-9066-2017
OI Cazares, Adrian/0000-0001-6242-4405; Botelho, Joao/0000-0002-2771-2345
FU Max-Planck Society; Max-Planck Institute for Evolutionary Biology in Ploen; German Science Foundation; Leibniz ScienceCampus Evolutionary Medicine of the Lung (EvoLUNG) [EXC 2167-390884018]; Kiel Life Science Postdoc Award; Max Planck Society
FX Max-Planck Society; Max-Planck Institute for Evolutionary Biology in Ploen (fellowship to H.S.); German Science Foundation (funding under Germany's Excellence Strategy EXC 2167-390884018 as well as the Research Training Group 2501 TransEvo to H.S.); Leibniz ScienceCampus Evolutionary Medicine of the Lung (EvoLUNG, to H.S.); Kiel Life Science Postdoc Award (to J.B.). Funding for open access charge: Max Planck Society.
CR Achtman M, 2008, NAT REV MICROBIOL, V6, P431, DOI 10.1038/nrmicro1872
Acman M, 2020, NAT COMMUN, V11, DOI 10.1038/s41467-020-16282-w
Akhter S, 2012, NUCLEIC ACIDS RES, V40, DOI 10.1093/nar/gks406
Al-Shayeb B, 2020, NATURE, V578, P425, DOI 10.1038/s41586-020-2007-4
Almpanis A, 2018, MICROB GENOMICS, V4, DOI 10.1099/mgen.0.000168
[Anonymous], 2014, NAT COMMUN, DOI DOI 10.1038/NCOMMS6208
Bazin A, 2020, BIOINFORMATICS, V36, pI651, DOI 10.1093/bioinformatics/btaa792
Bondy-Denomy J, 2013, NATURE, V493, P429, DOI 10.1038/nature11723
Botelho J, 2020, MICROB GENOMICS, V6, DOI 10.1099/mgen.0.000424
Botelho J, 2021, TRENDS MICROBIOL, V29, P8, DOI 10.1016/j.tim.2020.05.011

Botelho J, 2019, DRUG RESIST UPDATE, V44, P26, DOI 10.1016/j.drup.2019.07.002
Buchfink B, 2021, NAT METHODS, V18, P366, DOI 10.1038/s41592-021-01101-x
Cantalapiedra CP, 2021, MOL BIOL EVOL, V38, P5825, DOI 10.1093/molbev/msab293
Cassini A, 2019, LANCET INFECT DIS, V19, P56, DOI 10.1016/S1473-3099(18)30605-4
Chen J, 2018, SCIENCE, V362, P207, DOI 10.1126/science.aat5867
Chen LH, 2016, NUCLEIC ACIDS RES, V44, pD694, DOI 10.1093/nar/gkv1239
Copin R, 2019, P NATL ACAD SCI USA, V116, P1745, DOI 10.1073/pnas.1814265116
Croucher NJ, 2014, NAT COMMUN, V5, DOI 10.1038/ncomms6471
Cury J, 2018, MOL BIOL EVOL, V35, P2230, DOI 10.1093/molbev/msy123
Cury J, 2017, NUCLEIC ACIDS RES, V45, P8943, DOI 10.1093/nar/gkx607
Dagan T, 2008, P NATL ACAD SCI USA, V105, P10039, DOI 10.1073/pnas.0800679105
Davin-Regli A, 2019, CLIN MICROBIOL REV, V32, DOI 10.1128/CMR.00002-19
De Oliveira DMP, 2020, CLIN MICROBIOL REV, V33, DOI 10.1128/CMR.00181-19
Doron S, 2018, SCIENCE, V359, DOI 10.1126/science.aar4120
Eddy SR, 2011, PLOS COMPUT BIOL, V7, DOI 10.1371/journal.pcbi.1002195
Ellabaan MMH, 2021, NAT COMMUN, V12, DOI 10.1038/s41467-021-22757-1
Enault F, 2017, ISME J, V11, P237, DOI 10.1038/ismej.2016.90
Fillol-Salom A, 2018, ISME J, V12, P2114, DOI 10.1038/s41396-018-0156-3
Forster SC, 2022, NAT COMMUN, V13, DOI 10.1038/s41467-022-29096-9
Galperin MY, 2021, NUCLEIC ACIDS RES, V49, pD274, DOI 10.1093/nar/gkaa1018
Galperin MY, 2019, BRIEF BIOINFORM, V20, P1063, DOI 10.1093/bib/bbx117
Gao W, 2018, CURR OPIN MICROBIOL, V41, P76, DOI 10.1016/j.mib.2017.11.030
Gautreau G, 2020, PLOS COMPUT BIOL, V16, DOI 10.1371/journal.pcbi.1007732
Ghaly TM, 2018, TRENDS MICROBIOL, V26, P904, DOI 10.1016/j.tim.2018.05.008
Halary S, 2010, P NATL ACAD SCI USA, V107, P127, DOI 10.1073/pnas.0908978107
Harding CM, 2018, NAT REV MICROBIOL, V16, P91, DOI 10.1038/nrmicro.2017.148
Iranzo J, 2016, MBIO, V7, DOI 10.1128/mBio.00978-16
Jain C, 2018, NAT COMMUN, V9, DOI 10.1038/s41467-018-07641-9
John J, 2019, GENOME BIOL EVOL, V11, P2917, DOI 10.1093/gbe/evz213
Kazazian HH, 2004, SCIENCE, V303, P1626, DOI 10.1126/science.1089670
Khedkar S, 2022, NUCLEIC ACIDS RES, V50, P3155, DOI 10.1093/nar/gkac163
Kloesges T, 2011, MOL BIOL EVOL, V28, P1057, DOI 10.1093/molbev/msq297
Koonin EV, 2020, NAT REV GENET, V21, P119, DOI 10.1038/s41576-019-0172-9
Koonin EV, 2016, PHILOS T R SOC B, V371, DOI 10.1098/rstb.2015.0442
Lam MMC, 2018, MICROB GENOMICS, V4, DOI 10.1099/mgen.0.000196
Lee MD, 2019, BIOINFORMATICS, V35, P4162, DOI 10.1093/bioinformatics/btz188
León LM, 2021, NUCLEIC ACIDS RES, V49, P2114, DOI 10.1093/nar/gkab006

Li YP, 2021, CELL HOST MICROBE, V29, P704, DOI 10.1016/j.chom.2020.12.007
Liu M, 2019, NUCLEIC ACIDS RES, V47, pD660, DOI 10.1093/nar/gky1123
Mahendra C, 2020, NAT MICROBIOL, V5, P620, DOI 10.1038/s41564-020-0692-2
Makarova KS, 2020, NAT REV MICROBIOL, V18, P67, DOI 10.1038/s41579-019-0299-x
Mortensen K, 2021, FRONT MICROBIOL, V12, DOI 10.3389/fmicb.2021.758782
Moya-Beltrán A, 2021, CRISPR J, V4, P656, DOI 10.1089/crispr.2021.0051
Nguyen Lam-Tung, 2015, Mol Biol Evol, V32, P268, DOI 10.1093/molbev/msu300
Nishida Hiromi, 2012, Int J Evol Biol, V2012, P342482, DOI 10.1155/2012/342482
Oliveira PH, 2014, NUCLEIC ACIDS RES, V42, P10618, DOI 10.1093/nar/gku734
Paauw A, 2010, PLOS ONE, V5, DOI 10.1371/journal.pone.0008662
Partridge SR, 2018, CLIN MICROBIOL REV, V31, DOI 10.1128/CMR.00088-17
Pinilla-Redondo R, 2020, NUCLEIC ACIDS RES, V48, P2000, DOI 10.1093/nar/gkz1197
Pinilla-Redondo R, 2022, NUCLEIC ACIDS RES, V50, P4315, DOI 10.1093/nar/gkab859
Pinilla-Redondo R, 2020, NAT COMMUN, V11, DOI 10.1038/s41467-020-19415-3
Popa O, 2011, GENOME RES, V21, P599, DOI 10.1101/gr.115592.110
Pursey E, 2022, PHILOS T R SOC B, V377, DOI 10.1098/rstb.2020.0464
Quinlan AR, 2010, BIOINFORMATICS, V26, P841, DOI 10.1093/bioinformatics/btq033
Redondo-Salvo S, 2020, NAT COMMUN, V11, DOI 10.1038/s41467-020-17278-2
Richter M, 2009, P NATL ACAD SCI USA, V106, P19126, DOI 10.1073/pnas.0906412106
Roberts RJ, 2015, NUCLEIC ACIDS RES, V43, pD298, DOI 10.1093/nar/gku1046
Rodriguez-Beltran J, 2018, NAT ECOL EVOL, V2, P873, DOI 10.1038/s41559-018-0529-z
Rollie C, 2020, NATURE, V578, P149, DOI 10.1038/s41586-020-1936-2
Russel J, 2020, CRISPR J, V3, P462, DOI 10.1089/crispr.2020.0059
Millan AS, 2017, MICROBIOL SPECTR, V5, DOI 10.1128/microbiolspec.MTBP-0016-2017
Seemann T, 2014, BIOINFORMATICS, V30, P2068, DOI 10.1093/bioinformatics/btu153
Shapiro BJ, 2012, SCIENCE, V336, P48, DOI 10.1126/science.1218198
Shmakov SA, 2017, MBIO, V8, DOI 10.1128/mBio.01397-17
Smillie C, 2010, MICROBIOL MOL BIOL R, V74, P434, DOI 10.1128/MMBR.00020-10
Smillie CS, 2011, NATURE, V480, P241, DOI 10.1038/nature10571
Steinegger M, 2018, NAT COMMUN, V9, DOI 10.1038/s41467-018-04964-5
van Belkum A, 2015, MBIO, V6, DOI 10.1128/mBio.01796-15
Wein T, 2019, NAT COMMUN, V10, DOI 10.1038/s41467-019-10600-7
Wheatley RM, 2021, ISME J, V15, P1420, DOI 10.1038/s41396-020-00860-3
Wyres KL, 2020, GENOME MED, V12, DOI 10.1186/s13073-019-0706-y
Zankari E, 2012, J ANTIMICROB CHEMOTH, V67, P2640, DOI 10.1093/jac/dks261
Zhang ZY, 2022, NAT COMMUN, V13, DOI 10.1038/s41467-022-29283-8

Zhao XF, 2019, BIOINFORMATICS, V35, P671, DOI
10.1093/bioinformatics/bty651
NR 85
TC 21
Z9 22
U1 3
U2 24
PU OXFORD UNIV PRESS
PI OXFORD
PA GREAT CLARENDON ST, OXFORD OX2 6DP, ENGLAND
SN 0305-1048
EI 1362-4962
J9 NUCLEIC ACIDS RES
JI Nucleic Acids Res.
PD JAN 11
PY 2023
VL 51
IS 1
BP 236
EP 252
DI 10.1093/nar/gkac1220
EA JAN 2023
PG 17
WC Biochemistry & Molecular Biology
WE Science Citation Index Expanded (SCI-EXPANDED)
SC Biochemistry & Molecular Biology
GA 7X5KO
UT WOS:000908326200001
PM 36610752
OA Green Submitted, Green Published, gold
DA 2025-05-07
ER

PT J
AU O'Keeffe, KR
Halliday, FW
Jones, CD
Carbone, I
Mitchell, CE
AF O'Keeffe, Kayleigh R.
Halliday, Fletcher W.
Jones, Corbin D.
Carbone, Ignazio
Mitchell, Charles E.
TI Parasites, niche modification and the host microbiome: A field survey of
multiple parasites
SO MOLECULAR ECOLOGY
LA English
DT Article
DE biotic interactions; disease ecology; metabarcoding; microbiome;
mycobiome; niche modification
ID INTERNAL TRANSCRIBED SPACER; FUNGAL ENDOPHYTES; SPECIES-DIVERSITY;
ECOLOGY; SUSCEPTIBILITY; ANTIBIOTICS; COMMUNITIES; ABUNDANCE; MARKER;

SHAPE
AB Parasites can affect and be affected by the host's microbiome, with consequences for host susceptibility, parasite transmission, and host and parasite fitness. Yet, two aspects of the relationship between parasite infection and host microbiota remain little understood: the nature of the relationship under field conditions, and how the relationship varies among parasites. To overcome these limitations, we performed a field survey of the within-leaf fungal community in a tall fescue population. We investigated how diversity and composition of the fungal microbiome associate with natural infection by fungal parasites with different feeding strategies. A parasite's feeding strategy affects both parasite requirements of the host environment and parasite impacts on the host environment. We hypothesized that parasites that more strongly modify niches available within a host will be associated with greater changes in microbiome diversity and composition. Parasites with a feeding strategy that creates necrotic tissue to extract resources (necrotrophs) may not only have different niche requirements, but also act as particularly strong niche modifiers. Barcoded amplicon sequencing of the fungal ITS region revealed that leaf segments symptomatic of necrotrophs had lower fungal diversity and distinct composition compared to segments that were asymptomatic or symptomatic of other parasites. There were no clear differences in fungal diversity or composition between leaf segments that were asymptomatic and segments symptomatic of other parasite feeding strategies. Our results motivate future experimental work to test how the relationship between the microbiome and parasite infection is impacted by parasite feeding strategy and highlight the potential importance of parasite traits.
C1 [O'Keeffe, Kayleigh R.; Halliday, Fletcher W.; Jones, Corbin D.; Mitchell, Charles E.] Univ N Carolina, Dept Biol, Chapel Hill, NC 27515 USA.
[O'Keeffe, Kayleigh R.] Univ Penn, Dept Biol, Philadelphia, PA 19104 USA.
[Halliday, Fletcher W.] Univ Zurich, Dept Evolutionary Biol & Environm Studies, Zurich, Switzerland.
[Jones, Corbin D.] Univ N Carolina, Carolina Ctr Genome Sci, Chapel Hill, NC 27515 USA.
[Carbone, Ignazio] North Carolina State Univ, Ctr Integrated Fungal Res, Dept Entomol & Plant Pathol, Raleigh, NC USA.
[Mitchell, Charles E.] Univ N Carolina, Environm Ecol & Energy Program, Chapel Hill, NC 27515 USA.
C3 University of North Carolina; University of North Carolina Chapel Hill;
University of Pennsylvania; University of Zurich; University of North Carolina; University of North Carolina Chapel Hill; North Carolina State
University; University of North Carolina; University of North Carolina Chapel Hill
RP O'Keeffe, KR (autor correspondente), Univ Penn, Dept Biol, Philadelphia, PA 19104 USA.
EM kokeeffe@sas.upenn.edu
RI Carbone, Ignazio/JDW-1853-2023; Halliday, Fletcher/ABD-1950-2020;
O'Keeffe, Kayleigh/AAG-8135-2019; Mitchell, Charles/I-3709-2014
OI O'Keeffe, Kayleigh/0000-0002-8259-6439; Mitchell, Charles/0000-0002-1633-1993; Halliday, Fletcher/0000-0003-3953-0861

FU NSF-USDA joint programme in Ecology and Evolution of Infectious Diseases
[2016-67013-25762, DEB-1541418]; University Cancer Research Fund;
Triangle Center for Evolutionary Medicine; National Science Foundation;
University of North Carolina-Chapel Hill (UNC-CH) Graduate School; NIFA
[2016-67013-25762, 914333] Funding Source: Federal RePORTER
FX NSF-USDA joint programme in Ecology and Evolution of Infectious
Diseases, Grant/Award Number: 2016-67013-25762 and DEB-1541418;
University Cancer Research Fund; Triangle Center for Evolutionary
Medicine; The National Science Foundation; University of North
Carolina-Chapel Hill (UNC-CH) Graduate School
CR Adler PB, 2014, P NATL ACAD SCI USA, V111, P740, DOI 10.1073/pnas.1315179111
Aivelo T, 2018, J ANIM ECOL, V87, P438, DOI 10.1111/1365-2656.12708
[Anonymous], 2020, Linear and nonlinear mixed effects models R package nlme version 3.1149
Arnold AE, 2007, MYCOLOGIA, V99, P185, DOI 10.3852/mycologia.99.2.185
Arnold AE, 2003, P NATL ACAD SCI USA, V100, P15649, DOI 10.1073/pnas.2533483100
Bálint M, 2016, FEMS MICROBIOL REV, V40, P686, DOI 10.1093/femsre/fuw017
Bálint M, 2015, MOL ECOL, V24, P235, DOI 10.1111/mec.13018
Barman M, 2008, INFECT IMMUN, V76, P907, DOI 10.1128/IAI.01432-07
Bashey F, 2015, PHILOS T R SOC B, V370, DOI 10.1098/rstb.2014.0301
Bent SJ, 2008, ISME J, V2, P689, DOI 10.1038/ismej.2008.44
Berg M, 2018, CURR BIOL, V28, P2487, DOI 10.1016/j.cub.2018.05.085
Bergelson J, 2019, SCI REP-UK, V9, DOI 10.1038/s41598-018-37208-z
Borer ET, 2011, ECOHEALTH, V8, P261, DOI 10.1007/s10393-011-0718-6
Budischak SA, 2018, FUNCT ECOL, V32, P324, DOI 10.1111/1365-2435.12951
Busby PE, 2016, PLANT MOL BIOL, V90, P645, DOI 10.1007/s11103-015-0412-0
Busby PE, 2016, NEW PHYTOL, V209, P1681, DOI 10.1111/nph.13742
Cadotte MW, 2017, ECOL LETT, V20, P989, DOI 10.1111/ele.12796
Callahan BJ, 2016, NAT METHODS, V13, P581, DOI [10.1038/NMETH.3869, 10.1038/nmeth.3869]
Carbone I, 2019, MICROBIOL RESOUR ANN, V8, DOI 10.1128/MRA.00328-19
Carbone I, 2017, BIOINFORMATICS, V33, P1160, DOI 10.1093/bioinformatics/btw808
Cho I, 2012, NAT REV GENET, V13, P260, DOI 10.1038/nrg3182
Christian N, 2015, FRONT MICROBIOL, V6, DOI 10.3389/fmicb.2015.00869
Coyte KZ, 2015, SCIENCE, V350, P663, DOI 10.1126/science.aad2602
Croswell A, 2009, INFECT IMMUN, V77, P2741, DOI 10.1128/IAI.00006-09
Dobon A, 2016, BMC GENOMICS, V17, DOI 10.1186/s12864-016-2684-4
Dushoff J, 2019, METHODS ECOL EVOL, V10, P756, DOI 10.1111/2041-210X.13159
Elhadyl A, 2017, PLOS ONE, V12, DOI 10.1371/journal.pone.0177145
Fukami T, 2015, ANNU REV ECOL EVOL S, V46, P1, DOI 10.1146/annurev-ecolsys-110411-160340
Fukami T, 2011, ECOL LETT, V14, P973, DOI 10.1111/j.1461-0248.2011.01663.x
Gazis R, 2011, MOL ECOL, V20, P3001, DOI 10.1111/j.1365-294X.2011.05110.x

Glazebrook J, 2005, ANNU REV PHYTOPATHOL, V43, P205, DOI 10.1146/annurev.phyto.43.040204.135923
Gotelli NJ, 2001, ECOL LETT, V4, P379, DOI 10.1046/j.1461-0248.2001.00230.x
Graham AL, 2008, P NATL ACAD SCI USA, V105, P566, DOI 10.1073/pnas.0707221105
Halbritter AH, 2020, METHODS ECOL EVOL, V11, P22, DOI 10.1111/2041-210X.13331
Halliday FW, 2018, P ROY SOC B-BIOL SCI, V285, DOI 10.1098/rspb.2018.2075
Halliday FW, 2019, ECOL LETT, V22, P138, DOI 10.1111/ele.13176
Halliday FW, 2017, P ROY SOC B-BIOL SCI, V284, DOI 10.1098/rspb.2017.1340
Halliday FW, 2017, ECOL LETT, V20, P1285, DOI 10.1111/ele.12825
Hayes KS, 2010, SCIENCE, V328, P1391, DOI 10.1126/science.1187703
Higgins KL, 2007, MOL PHYLOGENET EVOL, V42, P543, DOI 10.1016/j.ympev.2006.07.012
HILL MO, 1973, ECOLOGY, V54, P427, DOI 10.2307/1934352
Jakuschkin B, 2016, MICROB ECOL, V72, P870, DOI 10.1007/s00248-016-0777-x
Jani AJ, 2014, P NATL ACAD SCI USA, V111, pE5049, DOI 10.1073/pnas.1412752111
JONES CG, 1994, OIKOS, V69, P373, DOI 10.2307/3545850
Jost L, 2006, OIKOS, V113, P363, DOI 10.1111/j.2006.0030-1299.14714.x
Keller L, 2003, NATURE, V425, P769, DOI 10.1038/425769a
Khosravi A, 2013, CURR OPIN MICROBIOL, V16, P221, DOI 10.1016/j.mib.2013.03.009
Köljalg U, 2013, MOL ECOL, V22, P5271, DOI 10.1111/mec.12481
Laforest-Lapointe I, 2018, MSYSTEMS, V3, DOI 10.1128/mSystems.00201-17
Lebreton L, 2019, PLOS ONE, V14, DOI 10.1371/journal.pone.0204195
Lee SC, 2014, PLOS NEGLECT TROP D, V8, DOI 10.1371/journal.pntd.0002880
Leggett HC, 2017, PHILOS T R SOC B, V372, DOI 10.1098/rstb.2016.0094
Lenth R., 2022, EMMEANS ESTIMATED MA
Leung JM, 2018, PLOS BIOL, V16, DOI 10.1371/journal.pbio.2004108
Lewontin R.C., 1983, P273
Li RW, 2012, INFECT IMMUN, V80, P2150, DOI 10.1128/IAI.00141-12
Libertucci J, 2019, NAT MICROBIOL, V4, P35, DOI 10.1038/s41564-018-0278-4
Lindner DL, 2011, MYCOLOGIA, V103, P241, DOI 10.3852/10-262
Luna PN, 2020, PLOS COMPUT BIOL, V16, DOI 10.1371/journal.pcbi.1008473
Lundberg DS, 2013, NAT METHODS, V10, P999, DOI 10.1038/nmeth.2634
Martin M., 2011, Embnet. J., V17, P10, DOI [DOI 10.14806/EJ.17.1.200, 10.14806/ej.17.1.200]
McMurdie PJ, 2013, PLOS ONE, V8, DOI 10.1371/journal.pone.0061217
Mitchell CE, 2003, GLOBAL CHANGE BIOL, V9, P438, DOI 10.1046/j.1365-2486.2003.00602.x
Mitchell CE, 2002, ECOLOGY, V83, P1713, DOI 10.1890/0012-9658(2002)083[1713:EOGPSD]2.0.CO;2
Mosca A, 2016, FRONT MICROBIOL, V7, DOI 10.3389/fmicb.2016.00455
Naiman R.J., 2009, BIOSCIENCE, V38, P753
Nayfach S, 2016, CELL, V166, P1103, DOI 10.1016/j.cell.2016.08.007
Nguyen NH, 2016, FUNGAL ECOL, V20, P241, DOI 10.1016/j.funeco.2015.06.006

Oksanen J., 2019, Vegan: community ecology package
Oliva J, 2020, ANNU REV PHYTOPATHOL, V58, P343, DOI 10.1146/annurev-phyto-080417-050028
Porras-Alfaro A, 2014, APPL ENVIRON MICROB, V80, P829, DOI 10.1128/AEM.02894-13
Porras-Alfaro A, 2011, ANNU REV PHYTOPATHOL, V49, P291, DOI 10.1146/annurev-phyto-080508-081831
R Core Team, 2012, R: A language and environment for statistical computing
Raaijmakers JM, 2012, ANNU REV PHYTOPATHOL, V50, P403, DOI 10.1146/annurev-phyto-081211-172908
Schoch CL, 2012, P NATL ACAD SCI USA, V109, P6241, DOI 10.1073/pnas.1117018109
Seybold H, 2020, NAT COMMUN, V11, DOI 10.1038/s41467-020-15633-x
Smith DP, 2014, PLOS ONE, V9, DOI 10.1371/journal.pone.0090234
Suzuki SU, 2019, AM NAT, V194, P90, DOI 10.1086/703485
Vannette RL, 2016, ECOLOGY, V97, P2374, DOI 10.1002/ecy.1472
Weinstein SB, 2016, BIOL LETTERS, V12, DOI 10.1098/rsbl.2016.0324
Weiss S, 2017, MICROBIOME, V5, DOI 10.1186/s40168-017-0237-y
White TJ, 1990, PCR PROTOCOLS GUIDE, P315, DOI [10.1016/B978-0-12-372180-8.50042-1, DOI 10.1016/B978-0-12-372180-8.50042-1]
Williams AR, 2017, PLOS ONE, V12, DOI 10.1371/journal.pone.0186546
Wu SB, 2014, VET MICROBIOL, V169, P188, DOI 10.1016/j.vetmic.2014.01.007
Zanne AE, 2020, BIOL REV, V95, P409, DOI 10.1111/brv.12570
Zhang XY, 2017, BMC BIOINFORMATICS, V18, DOI 10.1186/s12859-016-1441-7
NR 86
TC 10
Z9 12
U1 1
U2 15
PU WILEY
PI HOBOKEN
PA 111 RIVER ST, HOBOKEN 07030-5774, NJ USA
SN 0962-1083
EI 1365-294X
J9 MOL ECOL
JI Mol. Ecol.
PD MAY
PY 2021
VL 30
IS 10
BP 2404
EP 2416
DI 10.1111/mec.15892
EA APR 2021
PG 13
WC Biochemistry & Molecular Biology; Ecology; Evolutionary Biology
WE Science Citation Index Expanded (SCI-EXPANDED)
SC Biochemistry & Molecular Biology; Environmental Sciences & Ecology; Evolutionary Biology
GA RZ1ZI
UT WOS:000639363700001
PM 33740826

OA Bronze, Green Submitted
DA 2025-05-07
ER

PT J
AU Uyenoyama, MK
AF Uyenoyama, Marcy K.
TI Wright's Hierarchical F-Statistics
SO MOLECULAR BIOLOGY AND EVOLUTION
LA English
DT Article
DE identity coefficients; IBD; IBS; effective number; selfing
ID POPULATION-STRUCTURE; GENE DIVERSITY; SAMPLING THEORY; VARIANCE; DISTANCE; SYSTEMS
AB This perspective article offers a meditation on FST and other quantities developed by Sewall Wright to describe the population structure, defined as any departure from reproduction through random union of gametes. Concepts related to the F-statistics draw from studies of the partitioning of variation, identity coefficients, and diversity measures. Relationships between the first two approaches have recently been clarified and unified. This essay addresses the third pillar of the discussion: Nei's GST and related measures. A hierarchy of probabilities of identity-by-state provides a description of the relationships among levels of a structured population with respect to genetic diversity. Explicit expressions for the identity-by-state probabilities are determined for models of structured populations undergoing regular inbreeding and recurrent mutation. Levels of genetic diversity within and between subpopulations reflect mutation as well as migration. Accordingly, indices of the population structure are inherently locus-specific, contrary to the intentions of Wright. Some implications of this locus-specificity are explored.
C1 [Uyenoyama, Marcy K.] Duke Univ, Dept Biol, Box 90338, Durham, NC 27708 USA.
C3 Duke University
RP Uyenoyama, MK (autor correspondente), Duke Univ, Dept Biol, Box 90338, Durham, NC 27708 USA.
EM marcy@duke.edu
OI Uyenoyama, Marcy/0000-0001-8249-1103
FU Public Health Service; Institute of Genomics and Evolutionary Medicine (iGEM) at Temple University [GM 37841]; Public Health Service
FX I thank Molecular Biology and Evolution Editors-in-Chief Claudia Russo and Brandon Gaut for inviting this Perspective piece, and the reviewers for their comments. I am deeply indebted to Sudhir Kumar and all members of the Institute of Genomics and Evolutionary Medicine (iGEM) at Temple University for their support and gracious hospitality during the completion of this essay. I especially appreciate the opportunity to work in Masatoshi Nei's former office. Public Health Service grant GM 37841 provided partial funding for this research.
CR Arbisser IM, 2020, THEOR POPUL BIOL, V133, P117, DOI 10.1016/j.tpb.2019.05.003

Bateson W., 1902, Reports to the Evolution Committee of the Royal Society I, V1, P125
CHESSER RK, 1991, GENETICS, V127, P437
COCKERHAM CC, 1967, GENETICS, V56, P89
COCKERHAM CC, 1969, EVOLUTION, V23, P72, DOI 10.1111/j.1558-5646.1969.tb03496.x
COCKERHAM CC, 1973, GENETICS, V74, P679
Crow J F, 1994, Biogr Mem Natl Acad Sci, V64, P439
CROW JF, 1988, EVOLUTION, V42, P482, DOI [10.2307/2409033, 10.1111/j.1558-5646.1988.tb04154.x]
EWENS WJ, 1972, THEOR POPUL BIOL, V3, P87, DOI 10.1016/0040-5809(72)90035-4
EWENS WJ, 1982, THEOR POPUL BIOL, V21, P373, DOI 10.1016/0040-5809(82)90024-7
Haldane JBS, 1924, P CAMB PHILOS SOC-B, V1, P158, DOI 10.1111/j.1469-185X.1924.tb00546.x
Hudson R.R., 1990, Oxford Surveys in Evolutionary Biology, V7, P1
Jacquard A., 1974, Structures Genetiques des Populations
Jakobsson M, 2013, GENETICS, V193, P515, DOI 10.1534/genetics.112.144758
Jost L, 2008, MOL ECOL, V17, P4015, DOI 10.1111/j.1365-294X.2008.03887.x
Jukes TH., 1969, Mammilian protein metabolism, P240
KARLIN S, 1972, THEOR POPUL BIOL, V3, P113, DOI 10.1016/0040-5809(72)90036-6
Li C.C., 1976, First Course in Population Genetics
Malecot G., 1969, Les Mathematiques de l'Heredite
McPhee HC, 1925, J HERED, V16, P205, DOI 10.1093/oxfordjournals.jhered.a102593
NEI M, 1972, AM NAT, V106, P283, DOI 10.1086/282771
NEI M, 1977, ANN HUM GENET, V41, P225, DOI 10.1111/j.1469-1809.1977.tb01918.x
NEI M, 1965, EVOLUTION, V19, P256, DOI 10.2307/2406379
NEI M, 1973, P NATL ACAD SCI USA, V70, P3321, DOI 10.1073/pnas.70.12.3321
POLLAK E, 1987, GENETICS, V117, P353
Ramachandran S, 2005, P NATL ACAD SCI USA, V102, P15942, DOI 10.1073/pnas.0507611102
Rosenberg NA, 2002, SCIENCE, V298, P2381, DOI 10.1126/science.1078311
Russo CAM, 2024, MOL BIOL EVOL, V41, DOI 10.1093/molbev/msad264
SLATKIN M, 1991, GENET RES, V58, P167, DOI 10.1017/S0016672300029827
SLATKIN M, 1982, GENETICS, V100, P533
SPIETH PT, 1974, GENETICS, V78, P961
STROBECK C, 1987, GENETICS, V117, P149
TAKAHATA N, 1983, GENETICS, V104, P497
Thompson EA, 2008, THEOR POPUL BIOL, V73, P369, DOI 10.1016/j.tpb.2007.11.011
Uyenoyama MK, 2019, THEOR POPUL BIOL, V129, P148, DOI 10.1016/j.tpb.2018.10.004
Wahlund S, 1928, HEREDITAS, V11, P65, DOI 10.1111/j.1601-5223.1928.tb02483.x
WALLACE B, 1975, EVOLUTION, V29, P465, DOI 10.1111/j.1558-5646.1975.tb00836.x
Weir BS, 2017, GENETICS, V206, P2085, DOI 10.1534/genetics.116.198424

Weir BS, 2012, PHILOS SCI, V79, P637, DOI 10.1086/667904
Weir BS, 2005, GENOME RES, V15, P1468, DOI 10.1101/gr.4398405
WRIGHT S, 1965, EVOLUTION, V19, P395, DOI 10.2307/2406450
WRIGHT S, 1951, ANN EUGENIC, V15, P323
Wright S, 1921, GENETICS, V6, P111
WRIGHT S, 1952, GENETICS, V37, P312
Wright S, 1923, J HERED, V14, P405, DOI 10.1093/oxfordjournals.jhered.a102373
Wright S, 1943, GENETICS, V28, P114
WRIGHT S, 1978, J ANIM SCI, V46, P1192, DOI 10.2527/jas1978.4651192x
Wright S., 1969, The Theory of Gene Frequencies, V2, DOI DOI 10.1126/SCIENCE.168.3932.722
Wright S., 1977, Evolution and the Genetics of Populations: Experimental results and evolutionary deducations
NR 49
TC 1
Z9 1
U1 3
U2 6
PU OXFORD UNIV PRESS
PI OXFORD
PA GREAT CLARENDON ST, OXFORD OX2 6DP, ENGLAND
SN 0737-4038
EI 1537-1719
J9 MOL BIOL EVOL
JI Mol. Biol. Evol.
PD MAY 3
PY 2024
VL 41
IS 5
AR msae083
DI 10.1093/molbev/msae083
EA MAY 2024
PG 12
WC Biochemistry & Molecular Biology; Evolutionary Biology; Genetics & Heredity
WE Science Citation Index Expanded (SCI-EXPANDED)
SC Biochemistry & Molecular Biology; Evolutionary Biology; Genetics & Heredity
GA RV1I8
UT WOS:001230340300001
PM 38696269
OA Green Published, gold
DA 2025-05-07
ER

PT J
AU Reser, JE
AF Reser, Jared Edward
TI Alzheimer's disease and natural cognitive aging may represent adaptive metabolism reduction programs
SO BEHAVIORAL AND BRAIN FUNCTIONS
LA English
DT Review

ID LATERAL MOTOR COLUMN; POSITRON-EMISSION-TOMOGRAPHY; EVOLUTIONARY NEUROPATHOLOGY; TAU HYPERPHOSPHORYLATION; AMYLOID DEPOSITION; DIABETES-MELLITUS; BRAIN EVOLUTION; NERVOUS-SYSTEM; HIGH-FREQUENCY; LIFE-HISTORY

AB The present article examines several lines of converging evidence suggesting that the slow and insidious brain changes that accumulate over the lifespan, resulting in both natural cognitive aging and Alzheimer's disease (AD), represent a metabolism reduction program. A number of such adaptive programs are known to accompany aging and are thought to have decreased energy requirements for ancestral hunter-gatherers in their 30s, 40s and 50s. Foraging ability in modern hunter-gatherers declines rapidly, more than a decade before the average terminal age of 55 years. Given this, the human brain would have been a tremendous metabolic liability that must have been advantageously tempered by the early cellular and molecular changes of AD which begin to accumulate in all humans during early adulthood. Before the recent lengthening of life span, individuals in the ancestral environment died well before this metabolism reduction program resulted in clinical AD, thus there was never any selective pressure to keep adaptive changes from progressing to a maladaptive extent.

Aging foragers may not have needed the same cognitive capacities as their younger counterparts because of the benefits of accumulated learning and life experience. It is known that during both childhood and adulthood metabolic rate in the brain decreases linearly with age. This trend is thought to reflect the fact that children have more to learn. AD "pathology" may be a natural continuation of this trend. It is characterized by decreasing cerebral metabolism, selective elimination of synapses and reliance on accumulating knowledge (especially implicit and procedural) over raw brain power (working memory). Over decades of subsistence, the behaviors of aging foragers became routinized, their motor movements automated and their expertise ingrained to a point where they no longer necessitated the first-rate working memory they possessed when younger and learning actively. Alzheimer changes selectively and precisely mediate an adaptation to this major life-history transition.

AD symptomatology shares close similarities with deprivation syndromes in other animals including the starvation response. Both molecular and anatomical features of AD imitate brain changes that have been conceptualized as adaptive responses to low food availability in mammals and birds. Alzheimer's patients are known to express low overall metabolic rates and are genetically inclined to exhibit physiologically thrifty traits widely thought to allow mammals to subsist under conditions of nutritional scarcity. Additionally, AD is examined here in the contexts of anthropology, comparative neuroscience, evolutionary medicine, expertise, gerontology, neural Darwinism, neuroecology and the thrifty genotype.

C1 Univ So Calif, Dept Psychol, Encino, CA 91436 USA.
C3 University of Southern California
RP Reser, JE (autor correspondente), Univ So Calif, Dept Psychol, 16380 Meadow Ridge Rd, Encino, CA 91436 USA.
EM reser@usc.edu
OI Reser, Jared/0000-0003-0580-3403

CR Adams R D., 1980, HDB MENTAL HLTH AGIN, P149
AIELLO LC, 1995, CURR ANTHROPOL, V36, P199, DOI 10.1086/204350
[Anonymous], Dennett

Arendt T, 2003, J NEUROSCI, V23, P6972, DOI 10.1523/JNEUROSCI.23-18-06972.2003
Arnold SE, 1991, CEREB CORTEX, V1, P103, DOI 10.1093/cercor/1.1.103
Aschoff J., 1971, Energiehaushalt und Temperaturregulation
Baars B., 2007, COGNITION BRAIN CONS
Baltes PB, 1999, ANNU REV PSYCHOL, V50, P471, DOI 10.1146/annurev.psych.50.1.471
Baron-Cohen S., 1997, The maladapted mind: Classic readings in evolutionary psychology
Bateson P, 2004, NATURE, V430, P419, DOI 10.1038/nature02725
Bobe R, 2002, J HUM EVOL, V42, P475, DOI 10.1006/jhev.2001.0535
BRAINERD CJ, 1993, PSYCHOL REV, V100, P42, DOI 10.1037/0033-295X.100.2.319
BRUN A, 1981, HISTOPATHOLOGY, V5, P549, DOI 10.1111/j.1365-2559.1981.tb01818.x
Budson AE, 2005, NEW ENGL J MED, V352, P692, DOI 10.1056/NEJMra041071
Bufill E, 2006, REV NEUROLOGIA, V42, P25, DOI 10.33588/rn.4201.2005322
Burton R., 2008, BEING CERTAIN
CARLESIMO GA, 1992, NEUROPSYCHOL REV, V3, P119, DOI 10.1007/BF01108841
Cavanaugh J., 2002, ADULT DEV AGING, V4th
Clark W.R., 1999, MEANS END BIOL BASIS
CLUTTONBROCK TH, 1980, J ZOOL, V190, P309
Corbo RM, 1999, ANN HUM GENET, V63, P301, DOI 10.1046/j.1469-1809.1999.6340301.x
de Leon MJ, 1999, ALZHEIMER'S DISEASE AND RELATED DISORDERS, P105
DOIDGE N, 2007, BRAIN CHANGES ITSELF, P82
Dukas R, 2004, ANNU REV ECOL EVOL S, V35, P347, DOI 10.1146/annurev.ecolsys.35.112202.130152
Edelman G.M., 1987, Neural Darwinism: The Theory of Neuronal Group Selection
Epstein HT, 1999, BRAIN DEV-JPN, V21, P535, DOI 10.1016/S0387-7604(99)00066-2
Erickson CA, 2003, EXP GERONTOL, V38, P61, DOI 10.1016/S0531-5565(02)00160-2
ERNST RL, 1994, AM J PUBLIC HEALTH, V84, P1261, DOI 10.2105/AJPH.84.8.1261
Finch CE, 2004, Q REV BIOL, V79, P3, DOI 10.1086/381662
Finch CE, 1999, NEUROBIOL AGING, V20, P407, DOI 10.1016/S0197-4580(99)00053-6
FINLAY BL, 1983, SCIENCE, V219, P1349, DOI 10.1126/science.6828866
FINLAY BL, 1987, BRAIN BEHAV EVOLUT, V30, P102, DOI 10.1159/000118640
Fish JL, 2003, AM J PHYS ANTHROPOL, V120, P171, DOI 10.1002/ajpa.10136
Flier JS, 1998, J CLIN ENDOCR METAB, V83, P1407, DOI 10.1210/jc.83.5.1407
FOLEY RA, 1991, PHILOS T R SOC B, V334, P223, DOI 10.1098/rstb.1991.0111
FRIEDLAND RP, 1983, J COMPUT ASSIST TOMO, V7, P590, DOI 10.1097/00004728-198308000-00003
Garamszegi LZ, 2004, ECOL LETT, V7, P1216, DOI 10.1111/j.1461-0248.2004.00685.x
GIBSON KR, 1987, PRIMATE ONTOGENY COG
Gould E, 1999, NAT NEUROSCI, V2, P260, DOI 10.1038/6365
GREENOUGH WT, 1987, CHILD DEV, V58, P539, DOI 10.2307/1130197

Gunn-Moore DA, 2006, J FELINE MED SURG, V8, P234, DOI 10.1016/j.jfms.2006.01.003
Gurven M, 2006, J HUM EVOL, V51, P454, DOI 10.1016/j.jhevol.2006.05.003
HAIER RJ, 1992, BRAIN RES, V570, P134, DOI 10.1016/0006-8993(92)90573-R
HALES CN, 1992, DIABETOLOGIA, V35, P595, DOI 10.1093/ije/dyt133
Hales CN, 2001, BRIT MED BULL, V60, P5, DOI 10.1093/bmb/60.1.5
HAMBURGER V, 1975, J COMP NEUROL, V160, P535, DOI 10.1002/cne.901600408
Head E, 2005, NEUROBIOL AGING, V26, P749, DOI 10.1016/j.neurobiolaging.2004.06.015
Hedden T, 2004, NAT REV NEUROSCI, V5, P87, DOI 10.1038/nrn1323
Heininger K, 2000, REV NEUROSCIENCE, V11, P213
Heininger K, 2002, AGEING RES REV, V1, P481, DOI 10.1016/S1568-1637(02)00015-6
Herholz K, 2007, BRIT J RADIOL, V80, pS160, DOI 10.1259/bjr/97295129
Hill K.R., 1996, Ache life history: The ecology and demography of a foraging people
Hood Ernie, 2008, Evolution (N Y), V1, P114, DOI 10.1007/s12052-008-0036-9
HORN JL, 1970, LIFE SPAN DEV PSYCH, P146
Hubacek JA, 2001, NEUROPSYCHOBIOLOGY, V43, P200, DOI 10.1159/000054890
Ikeda Y, 2007, FEBS LETT, V581, P891, DOI 10.1016/j.febslet.2007.01.064
JACOBS DS, 1984, J NEUROSCI, V4, P2425
Jellinger KA, 2006, J NEURAL TRANSM, V113, P1603, DOI 10.1007/s00702-006-0578-3
JONES NGB, 1992, AM J PHYS ANTHROPOL, V89, P159, DOI 10.1002/ajpa.1330890204
Kahn SE, 1999, DIABETES, V48, P241, DOI 10.2337/diabetes.48.2.241
Kaplan H, 2000, EVOL ANTHROPOL, V9, P156, DOI 10.1002/1520-6505(2000)9:4<156::AID-EVAN5>3.0.CO;2-7
KAPLAN H, 2003, POPUL DEV REV, V29, P113
Kaplan HS, 2002, P NATL ACAD SCI USA, V99, P10221, DOI 10.1073/pnas.152502899
KATZ MJ, 1978, P NATL ACAD SCI USA, V75, P1349, DOI 10.1073/pnas.75.3.1349
Kempermann G, 2002, J NEUROSCI, V22, P635, DOI 10.1523/JNEUROSCI.22-03-00635.2002
Killiany RJ, 2000, ANN NEUROL, V47, P430, DOI 10.1002/1531-8249(200004)47:4<430::AID-ANA5>3.3.CO;2-9
Landsberg G, 2005, VET CLIN N AM-SMALL, V35, P675, DOI 10.1016/j.cvsm.2004.12.008
LANSER ME, 1984, J NEUROSCI, V4, P2043
LANSER ME, 1987, J COMP NEUROL, V261, P423, DOI 10.1002/cne.902610307
Leifer BP, 2003, J AM GERIATR SOC, V51, pS281, DOI 10.1046/j.1532-5415.5153.x
Li L, 2007, BRAIN RES REV, V56, P384, DOI 10.1016/j.brainresrev.2007.09.001
LYNCH G, 2008, BIG BRAINS ORIGINS F
MCLOON SC, 1982, SCIENCE, V215, P1418, DOI 10.1126/science.7063855
Meguro K, 2001, J NEUROL NEUROSUR PS, V71, P315, DOI 10.1136/jnnp.71.3.315

MILES DB, ANN REV ECOLOGY SYST, V24, P587
MINK JW, 1981, AM J PHYSIOL, V241, pR203, DOI 10.1152/ajpregu.1981.241.3.R203
Minoshima S, 1997, ANN NEUROL, V42, P85, DOI 10.1002/ana.410420114
Morris JC, 1996, NEUROLOGY, V46, P707, DOI 10.1212/WNL.46.3.707
Moscovitch M., 2007, Cognition, brain, and consciousness: Introduction to cognitive neuroscience, P234
NEEL JV, 1962, AM J HUM GENET, V14, P353
Nesse R.M., 1995, WHY WE GET SICK
Nesse RM, 1998, SCI AM, V279, P86, DOI 10.1038/scientificamerican1198-86
Nesse RM., 1999, EVOLUTIONARY MED
Neumann KF, 2008, CURR ALZHEIMER RES, V5, P438, DOI 10.2174/156720508785908919
Niedermeyer E, 2006, CLIN EEG NEUROSCI, V37, P175, DOI 10.1177/155005940603700303
Nilsson M, 1999, J NEUROBIOL, V39, P569, DOI 10.1002/(SICI)1097-4695(19990615)39:4<569::AID-NEU10>3.0.CO;2-F
Ohnishi T, 2001, AM J NEURORADIOL, V22, P1680
OLEARY DDM, 1986, J NEUROSCI, V6, P3692
OWEN OE, 1983, CLIN ENDOCRINOL META, V12, P359, DOI 10.1016/S0300-595X(83)80046-2
Pawlik M, 1999, NEUROBIOL AGING, V20, P47, DOI 10.1016/S0197-4580(99)00017-2
PEARSON RCA, 1985, P NATL ACAD SCI USA, V82, P4531, DOI 10.1073/pnas.82.13.4531
Pennisi E, 2007, SCIENCE, V317, P1308, DOI 10.1126/science.317.5843.1308
Perry G, 1998, SCIENCE, V282, P1268
Planel E, 2001, J BIOL CHEM, V276, P34298, DOI 10.1074/jbc.M102780200
Plomin R, 2003, ANNU REV PSYCHOL, V54, P205, DOI 10.1146/annurev.psych.54.101601.145108
Poehlman ET, 1997, NEUROLOGY, V48, P997, DOI 10.1212/WNL.48.4.997
Poehlman ET, 2000, AM J CLIN NUTR, V71, p650S
RAPOPORT SI, 1988, REV NEUROL-FRANCE, V144, P79
Ravelo AC, 2004, NATURE, V429, P263, DOI 10.1038/nature02567
Razay G, 2007, ARCH NEUROL-CHICAGO, V64, P93, DOI 10.1001/archneur.64.1.93
Reed KE, 1997, J HUM EVOL, V32, P289, DOI 10.1006/jhev.1996.0106
Reser JE, 2007, MED HYPOTHESES, V69, P383, DOI 10.1016/j.mehy.2006.12.031
Reser JE, 2006, MED HYPOTHESES, V67, P529, DOI 10.1016/j.mehy.2006.03.005
Reser JE, 2006, MED HYPOTHESES, V67, P474, DOI 10.1016/j.mehy.2006.03.019
Ricklefs R., 1995, Aging: A natural history
RIDLEY M, 2000, GENOME AUTOBIOGRAPHY, P96
Rosen RF, 2008, J COMP NEUROL, V509, P259, DOI 10.1002/cne.21744
SANDHOLZER C, 1995, HUM GENET, V95, P46
Scheibel Arnold B., 1996, P105
Schneider W, 2003, COGNITIVE SCI, V27, P525, DOI 10.1016/S0364-0213(03)00011-9
SCHWARTZ MW, 1995, AM J PHYSIOL-REG I, V269, pR949, DOI 10.1152/ajpregu.1995.269.5.R949

Selkoe DJ, 1997, AM J PSYCHIAT, V154, P1198
Semino O, 2002, AM J HUM GENET, V70, P265, DOI 10.1086/338306
SMITH BH, 1995, ANNU REV ANTHROPOL, V24, P257, DOI 10.1146/annurev.an.24.100195.001353
SYLVESTER R, 1993, EDUC LEADERSHIP, V51, P46
Terry RD, 2001, NEUROBIOL AGING, V22, P347, DOI 10.1016/S0197-4580(00)00250-5
Thompson PM, 2003, J NEUROSCI, V23, P994
Thompson RE, 2005, ANNU REV PSYCHOL, V56, P1, DOI 10.1146/annurev.psych.56.091103.070239
Trollor JN, 2005, PSYCHIAT CLIN NEUROS, V59, P280, DOI 10.1111/j.1440-1819.2005.01372.x
Twamley EW, 2006, J INT NEUROPSYCH SOC, V12, P707, DOI 10.1017/S1355617706060863
Wada H, 1998, MOL BIOL EVOL, V15, P1189, DOI 10.1093/oxfordjournals.molbev.a026026
Whitbourne S.K., 1996, The aging individual: Physical and psychological perspectives
Wick G, 2003, EXP GERONTOL, V38, P13, DOI 10.1016/S0531-5565(02)00161-4
WILLIAMS DRR, 1993, EYE, V7, P202, DOI 10.1038/eye.1993.48
WILLIAMS GC, 1991, Q REV BIOL, V66, P1, DOI 10.1086/417048
WILLIAMS RW, 1988, ANNU REV NEUROSCI, V11, P423, DOI 10.1146/annurev.neuro.11.1.423
Wynn JG, 2004, AM J PHYS ANTHROPOL, V123, P106, DOI 10.1002/ajpa.10317
Yanagisawa M, 1999, FEBS LETT, V461, P329, DOI 10.1016/S0014-5793(99)01480-5
Zafon C, 2006, MED HYPOTHESES, V67, P15, DOI 10.1016/j.mehy.2006.01.055
Zekraoui L, 1997, HUM BIOL, V69, P575
NR 130
TC 16
Z9 20
U1 0
U2 23
PU BMC
PI LONDON
PA CAMPUS, 4 CRINAN ST, LONDON N1 9XW, ENGLAND
EI 1744-9081
J9 BEHAV BRAIN FUNCT
JI Behav. Brain Funct.
PD FEB 28
PY 2009
VL 5
AR 13
DI 10.1186/1744-9081-5-13
PG 14
WC Behavioral Sciences; Neurosciences
WE Science Citation Index Expanded (SCI-EXPANDED); Social Science Citation Index (SSCI)
SC Behavioral Sciences; Neurosciences & Neurology
GA 427EQ
UT WOS:000264763000001
PM 19250550

OA Green Published, gold
DA 2025-05-07
ER

PT J
AU Diricks, M
Kohl, TA
Käding, N
Leshchinskiy, V
Hauswaldt, S
Vazquez, OJ
Utpatel, C
Niemann, S
Rupp, J
Merker, M
AF Diricks, Margo
Kohl, Thomas A.
Kading, Nadja
Leshchinskiy, Vladislav
Hauswaldt, Susanne
Jimenez Vazquez, Omar
Utpatel, Christian
Niemann, Stefan
Rupp, Jan
Merker, Matthias
TI Whole genome sequencing-based classification of human-related Haemophilus species and detection of antimicrobial resistance genes
SO GENOME MEDICINE
LA English
DT Article
DE Whole genome sequencing; Haemophilus; H; influenzae; H; haemolyticus; Pangenome-wide association study; Identification; Molecular differentiation; Antibiotic resistance; Precision medicine
ID GENUS HAEMOPHILUS; INFLUENZAE; IDENTIFICATION; HAEMOLYTICUS; RECOMBINATION; COLONIZATION; STRAINS; DISEASE; TOOL
AB Background Bacteria belonging to the genus Haemophilus cause a wide range of diseases in humans. Recently, H. influenzae was classified by the WHO as priority pathogen due to the wide spread of ampicillin resistant strains. However, other Haemophilus spp. are often misclassified as H. influenzae. Therefore, we established an accurate and rapid whole genome sequencing (WGS) based classification and serotyping algorithm and combined it with the detection of resistance genes. Methods A gene presence/absence-based classification algorithm was developed, which employs the open-source gene-detection tool SRST2 and a new classification database comprising 36 genes, including capsule loci for serotyping. These genes were identified using a comparative genome analysis of 215 strains belonging to ten human-related Haemophilus (sub)species (training dataset). The algorithm was evaluated on 1329 public short read datasets (evaluation dataset) and used to reclassify 262 clinical Haemophilus spp. isolates from 250 patients (German cohort). In addition, the presence of antibiotic resistance genes within the German dataset was evaluated with SRST2 and correlated with results of traditional phenotyping assays. Results The newly developed algorithm can

differentiate between clinically relevant Haemophilus species including, but not limited to, H. influenzae, H. haemolyticus, and H. parainfluenzae. It can also identify putative haemin-independent H. haemolyticus strains and determine the serotype of typeable Haemophilus strains. The algorithm performed excellently in the evaluation dataset (99.6% concordance with reported species classification and 99.5% with reported serotype) and revealed several misclassifications. Additionally, 83 out of 262 (31.7%) suspected H. influenzae strains from the German cohort were in fact H. haemolyticus strains, some of which associated with mouth abscesses and lower respiratory tract infections. Resistance genes were detected in 16 out of 262 datasets from the German cohort. Prediction of ampicillin resistance, associated with bla(TEM-1D), and tetracycline resistance, associated with tetB, correlated well with available phenotypic data. Conclusions Our new classification database and algorithm have the potential to improve diagnosis and surveillance of Haemophilus spp. and can easily be coupled with other public genotyping and antimicrobial resistance databases. Our data also point towards a possible pathogenic role of H. haemolyticus strains, which needs to be further investigated.
C1 [Diricks, Margo; Kohl, Thomas A.; Jimenez Vazquez, Omar; Utpatel, Christian; Niemann, Stefan; Merker, Matthias] Res Ctr Borstel, Mol & Expt Mycobacteriol, Borstel, Germany.
[Diricks, Margo; Kohl, Thomas A.; Utpatel, Christian; Merker, Matthias] German Ctr Infect Res DZIF, Partner Site Hamburg Lubeck Borstel Riems, Hamburg, Germany.
[Kading, Nadja; Leshchinskiy, Vladislav; Hauswaldt, Susanne; Rupp, Jan] Univ Hosp Schleswig Holstein, Dept Infect Dis & Microbiol, Lubeck, Germany.
[Kading, Nadja; Rupp, Jan] German Ctr Infect Res DZIF, TTU HAARBI, Lubeck, Germany.
[Merker, Matthias] Res Ctr Borstel, Evolut Resistome, Borstel, Germany.
C3 Leibniz Association; Forschungszentrum Borstel; German Center for Infection Research; University of Kiel; Schleswig Holstein University Hospital; German Center for Infection Research; Leibniz Association; Forschungszentrum Borstel
RP Merker, M (autor correspondente), Res Ctr Borstel, Evolut Resistome, Borstel, Germany.
EM mmerker@fz-borstel.de
RI Kohl, Thomas/AAB-9782-2020; Diricks, Margo/LRU-7418-2024; Rupp, Jan/C-8794-2011; Niemann, Stefan/C-9327-2011; Merker, Matthias/P-7399-2015
OI Diricks, Margo/0000-0002-5428-1456; Merker, Matthias/0000-0003-1386-2331; Kading, Nadja/0009-0002-4208-4911; Niemann, Stefan/0000-0002-6604-0684
FU Deutsche Forschungsgemeinschaft (DFG, German Research Foundation) under Germanys Excellence Strategy [EXC 2167]; Leibniz Science Campus Evolutionary Medicine of the LUNG (EvoLUNG); German Center for Infection Research (DZIF)
FX Parts of this work have been supported by the Deutsche Forschungsgemeinschaft (DFG, German Research Foundation) under Germanys

Excellence Strategy -EXC 2167 Precision Medicine in Inflammation, the Leibniz Science Campus Evolutionary Medicine of the LUNG (EvoLUNG), and the German Center for Infection Research (DZIF). The funders had no role in the study design; in the collection, analysis, and interpretation of data; in the writing of the report; and in the decision to submit the paper for publication.
CR Anderson R, 2012, J CLIN MICROBIOL, V50, P2462, DOI 10.1128/JCM.06575-11
Andrews S., 2019, FastQC
Bakaletz LO, 2018, TRENDS MICROBIOL, V26, P727, DOI 10.1016/j.tim.2018.05.001
Bankevich A, 2012, J COMPUT BIOL, V19, P455, DOI 10.1089/cmb.2012.0021
Bayliss SC, 2019, GIGASCIENCE, V8, DOI 10.1093/gigascience/giz119
Bervoets I, 2019, FEMS MICROBIOL REV, V43, P304, DOI 10.1093/femsre/fuz001
BRENNER DJ, 1988, J CLIN MICROBIOL, V26, P1524, DOI 10.1128/JCM.26.8.1524-1534.1988
Bush K, 2010, ANTIMICROB AGENTS CH, V54, P969, DOI 10.1128/AAC.01009-09
Connor TR, 2012, MICROBIOL-SGM, V158, P2958, DOI 10.1099/mic.0.063073-0
Cooke FJ, 2017, FOURTH
Cope EK, 2011, PLOS ONE, V6, DOI 10.1371/journal.pone.0028523
de Gier C, 2015, J CLIN MICROBIOL, V53, P3773, DOI 10.1128/JCM.01969-15
Diricks M, 2021, PRJEB43356 NCBI SEQ
Diricks M., 2021, HAEMOSEQ
Escalona M, 2016, NAT REV GENET, V17, P459, DOI 10.1038/nrg.2016.57
European Society of Clinical Microbiology and Infectious Diseases, EUCAST CLIN BREAKP
Frickmann H, 2014, EUR J MICROBIOL IMMU, V4, P99, DOI 10.1556/EuJMI.4.2014.2.2
Frickmann H, 2013, PLOS ONE, V8, DOI 10.1371/journal.pone.0063222
González-Díaz A, 2019, SCI REP-UK, V9, DOI 10.1038/s41598-019-40812-2
Gröschel MI, 2018, PLOS PATHOG, V14, DOI 10.1371/journal.ppat.1007297
Gupta SK, 2014, ANTIMICROB AGENTS CH, V58, P212, DOI 10.1128/AAC.01310-13
Harris TM, 2020, MICROB GENOMICS, V6, DOI 10.1099/mgen.0.000303
Hedegaard J, 2001, MICROBIOL-SGM, V147, P2599, DOI 10.1099/00221287-147-9-2599
Hendriksen RS, 2019, FRONT PUBLIC HEALTH, V7, DOI 10.3389/fpubh.2019.00242
Inouye M, 2014, GENOME MED, V6, DOI 10.1186/s13073-014-0090-6
Kitts PA, 2016, NUCLEIC ACIDS RES, V44, pD73, DOI 10.1093/nar/gkv1226
Kus JV, 2019, J CLIN MICROBIOL, V57, DOI 10.1128/JCM.01254-19
Leinonen R, 2011, NUCLEIC ACIDS RES, V39, pD19, DOI 10.1093/nar/gkq1019
Letunic I, 2007, BIOINFORMATICS, V23, P127, DOI 10.1093/bioinformatics/btl529
Letunic I, 2019, NUCLEIC ACIDS RES, V47, pW256, DOI 10.1093/nar/gkz239

Lewis DA, 2016, CURR OPIN INFECT DIS, V29, P52, DOI 10.1097/QCO.0000000000000226
Loens K, 2009, J CLIN MICROBIOL, V47, P21, DOI 10.1128/JCM.02037-08
McCormick DW, 2011, EMERG INFECT DIS, V17, P688, DOI 10.3201/eid1704.101045
Mukundan D, 2007, J CLIN MICROBIOL, V45, P3207, DOI 10.1128/JCM.00492-07
MURPHY TF, 1992, INFECT IMMUN, V60, P2016, DOI 10.1128/IAI.60.5.2016-2022.1992
Murphy TF, 1999, J INFECT DIS, V180, P404, DOI 10.1086/314870
Musher DM., 1996, Medical Microbiology
Norskov-Lauritsen N, 2005, INT J SYST EVOL MICR, V55, P449, DOI 10.1099/ijs.0.63325-0
Norskov-Lauritsen N, 2014, CLIN MICROBIOL REV, V27, P214, DOI 10.1128/CMR.00103-13
Norskov-Lauritsen N, 2012, INT J MED MICROBIOL, V302, P78, DOI 10.1016/j.ijmm.2012.01.001
Nürnberg S, 2020, J CLIN MICROBIOL, V58, DOI 10.1128/JCM.00278-20
Osman KL, 2018, SCI REP-UK, V8, DOI 10.1038/s41598-018-32973-3
Page AJ, 2015, BIOINFORMATICS, V31, P3691, DOI 10.1093/bioinformatics/btv421
Peltola H, 2000, CLIN MICROBIOL REV, V13, P302, DOI 10.1128/CMR.13.2.302-317.2000
Petit RA, SHOVILL SE
Pickering J, 2014, FRONT MICROBIOL, V5, DOI 10.3389/fmicb.2014.00664
Pinto M, 2019, INFECT GENET EVOL, V67, P126, DOI 10.1016/j.meegid.2018.10.025
Potts CC, 2019, BMC GENOMICS, V20, DOI 10.1186/s12864-019-6145-8
Price EP, 2017, FUTURE MICROBIOL, V12, P585, DOI 10.2217/fmb-2016-0215
Price EP, 2015, BMC GENOMICS, V16, DOI 10.1186/s12864-015-1857-x
Price MN, 2010, PLOS ONE, V5, DOI 10.1371/journal.pone.0009490
Rambaut A., FigTree
Satola SW, 2007, J CLIN MICROBIOL, V45, P3230, DOI 10.1128/JCM.00794-07
Seemann T., Shovill
Seemann T, 2014, BIOINFORMATICS, V30, P2068, DOI 10.1093/bioinformatics/btu153
Sierra Y, 2021, J ANTIMICROB CHEMOTH, V76, P1741, DOI 10.1093/jac/dkab109
Simberkoff MS., 2012, Goldman's Cecil Medicine, V2, P1861, DOI [10.1016/B978-1-4377-1604-7.00308-0, DOI 10.1016/B978-1-4377-1604-7.00308-0]
Slouka D., 2020, INT J ENV RES PUBLIC, V17, P1
SmithVaughan HC, 1996, EPIDEMIOL INFECT, V116, P177, DOI 10.1017/S0950268800052419
Souvorov A, 2018, GENOME BIOL, V19, DOI 10.1186/s13059-018-1540-z
Thjötta T, 1921, J EXP MED, V34, P97, DOI 10.1084/jem.34.1.97
Van Eldere J, 2014, LANCET INFECT DIS, V14, P1281, DOI 10.1016/S1473-3099(14)70734-0
Watts SC, 2019, J CLIN MICROBIOL, V57, DOI [10.1128/JCM.00190-19, 10.1128/jcm.00190-19]
Winslow CEA, 1917, J BACTERIOL, V2, P505, DOI 10.1128/JB.2.5.505-566.1917

   Witherden EA, 2014, J ANTIMICROB CHEMOTH, V69, P1501, DOI
10.1093/jac/dku022
   Wood DE, 2019, GENOME BIOL, V20, DOI 10.1186/s13059-019-1891-0
   Zhu BQ, 2013, PLOS ONE, V8, DOI 10.1371/journal.pone.0056139
NR 67
TC 7
Z9 7
U1 3
U2 8
PU BMC
PI LONDON
PA CAMPUS, 4 CRINAN ST, LONDON N1 9XW, ENGLAND
SN 1756-994X
J9 GENOME MED
JI Genome Med.
PD FEB 9
PY 2022
VL 14
IS 1
AR 13
DI 10.1186/s13073-022-01017-x
PG 15
WC Genetics & Heredity
WE Science Citation Index Expanded (SCI-EXPANDED)
SC Genetics & Heredity
GA YW4IF
UT WOS:000753377400001
PM 35139905
OA gold, Green Published
DA 2025-05-07
ER

PT J
AU Mccumber, AW
   Kim, YJ
   Granek, J
   Tighe, RM
   Gunsch, CK
AF Mccumber, Alexander W.
   Kim, Yeon Ji
   Granek, Joshua
   Tighe, Robert M.
   Gunsch, Claudia K.
TI Soil exposure modulates the immune response to an influenza challenge
in
   a mouse model
SO SCIENCE OF THE TOTAL ENVIRONMENT
LA English
DT Article
DE Microbiome; Lung; Gut; Old friends hypothesis; Environment
ID LUNG MICROBIOTA; GUT MICROBIOTA; DATABASE
AB There is increasing evidence that early life microbial exposure aids
in immune system maturation, more recently known as the "old friends"
hypothesis. To test this hypothesis, 4-week-old mice were exposed to

soils of increasing microbial diversity for four weeks followed by an intranasal challenge with either live or heat inactivated influenza A virus and monitored for 7 additional days. Perturbations of the gut and lung microbiomes were explored through 16S rRNA amplicon sequencing. RNA-sequencing was used to examine the host response in the lung tissue through differential gene expression. We determined that compared to the gut microbiome, the lung microbiome is more susceptible to changes in beta diversity following soil exposure with Lachnospiraceae ASVs accounting for most of the differences between groups. While several immune system genes were found to be significantly differentially expressed in lung tissue due to soil exposures, there were no differences in viral load or weight loss. This study shows that exposure to diverse microbial communities through soil exposure alters the gut and lung microbiomes resulting in differential expression of specific immune system related genes within the lung following an influenza challenge.
C1 [Mccumber, Alexander W.] Univ N Carolina, Dept Environm Sci & Engn, Chapel Hill, NC USA.
[Kim, Yeon Ji; Gunsch, Claudia K.] Duke Univ, Civil & Environm Engn Dept, Durham, NC USA.
[Granek, Joshua] Duke Univ, Dept Biostat & Bioinformat, Durham, NC USA.
[Tighe, Robert M.] Duke Univ, Dept Med, Durham, NC USA.
[Gunsch, Claudia K.] 203 Wilkinson Bldg, Durham, NC 27708 USA.
C3 University of North Carolina; University of North Carolina Chapel Hill;
Duke University; Duke University; Duke University
RP Gunsch, CK (autor correspondente), 203 Wilkinson Bldg, Durham, NC 27708 USA.
EM ckgunshc@duke.edu
RI Kim, Yeon Ji/AAE-1906-2022
OI Kim, Yeon Ji/0000-0002-1118-5026; Granek, Joshua/0000-0003-3908-5016
FU Triangle Center for Evolutionary Medicine (TriCEM); Duke University School of Medicine Core Facility Voucher; Engineering Research Centers Program of the National Science Foundation under NSF [EEC-2133504]
FX The authors would like to thank the Duke University Rodent Inhalation Core Facility for assisting with mouse influenza exposures and lung injury phenotyping. We also thank the Duke University School of Medicine for the use of the Sequencing and Genomic Technologies Shared Resource, which provided DNA sequencing and RNA-Sequencing. Funding for this work was provided by the Triangle Center for Evolutionary Medicine (TriCEM), the Duke University School of Medicine Core Facility Voucher and by the Engineering Research Centers Program of the National Science Foundation under NSF Cooperative Agreement No. EEC-2133504.
CR Abt MC, 2012, IMMUNITY, V37, P158, DOI 10.1016/j.immuni.2012.04.011
Anderson MJ., 2017, Wiley StatsRef: Statistics Reference Online Internet, P1, DOI DOI 10.1002/9781118445112.STAT07841
Aronesty E., 2013, TOBioiJ, V7
Ashburner M, 2000, NAT GENET, V25, P25, DOI 10.1038/75556

Bailey CC, 2012, PLOS PATHOG, V8, DOI 10.1371/journal.ppat.1002909
Bartley JM, 2017, FRONT IMMUNOL, V8, DOI 10.3389/fimmu.2017.01164
Bradley KC, 2019, CELL REP, V28, P245, DOI 10.1016/j.celrep.2019.05.105
Bradley-Stewart A, 2013, J CLIN VIROL, V58, P100, DOI 10.1016/j.jcv.2013.05.011
Callahan BJ, 2016, NAT METHODS, V13, P581, DOI [10.1038/NMETH.3869, 10.1038/nmeth.3869]
Carbon S, 2021, NUCLEIC ACIDS RES, V49, pD325, DOI 10.1093/nar/gkaa1113
Caspi R, 2008, NUCLEIC ACIDS RES, V36, pD623, DOI [10.1093/nar/gkm900, 10.1093/nar/gkt1103]
Consortium G.R., 2020, Mouse Build, V38
Corcoran BM, 2005, APPL ENVIRON MICROB, V71, P3060, DOI 10.1128/AEM.71.6.3060-3067.2005
Davis NM, 2018, MICROBIOME, V6, DOI 10.1186/s40168-018-0605-2
Dickson RP, 2020, AM J RESP CRIT CARE, V201, P555, DOI 10.1164/rccm.201907-1487OC
Dickson RP, 2018, AM J RESP CRIT CARE, V198, P497, DOI 10.1164/rccm.201711-2180OC
Dickson RP, 2017, MBIO, V8, DOI 10.1128/mBio.02287-16
Dobin A, 2013, BIOINFORMATICS, V29, P15, DOI 10.1093/bioinformatics/bts635
Douglas GM, 2020, NAT BIOTECHNOL, V38, P685, DOI 10.1038/s41587-020-0548-6
Drengenes C, 2019, BMC MICROBIOL, V19, DOI 10.1186/s12866-019-1560-1
Drury NL, 2023, PART FIBRE TOXICOL, V20, DOI 10.1186/s12989-023-00521-1
Edgar R, 2002, NUCLEIC ACIDS RES, V30, P207, DOI 10.1093/nar/30.1.207
Faith JJ, 2013, SCIENCE, V341, P44, DOI 10.1126/science.1237439
Fillion-Bertrand G, 2019, AM J RESP CELL MOL, V60, P189, DOI 10.1165/rcmb.2017-0228OC
Gonzalez-Perez G, 2017, FRONT IMMUNOL, V8, DOI 10.3389/fimmu.2017.00265
Gonzalez-Perez G, 2016, J IMMUNOL, V196, P3768, DOI 10.4049/jimmunol.1502322
Hagan T, 2019, CELL, V178, P1313, DOI 10.1016/j.cell.2019.08.010
Hildebrand F, 2021, CELL HOST MICROBE, V29, P1167, DOI 10.1016/j.chom.2021.05.008
Huang XQ, 2019, APPL SOIL ECOL, V135, P65, DOI 10.1016/j.apsoil.2018.11.011
Ichinohe T, 2011, P NATL ACAD SCI USA, V108, P5354, DOI 10.1073/pnas.1019378108
Iwai S, 2014, PLOS ONE, V9, DOI 10.1371/journal.pone.0095726
Jari Oksanen F.G.B., 2018, Eduard Szoecs and Helene Wagner. Community Ecology Package, vegan
Kanehisa M, 2000, NUCLEIC ACIDS RES, V28, P27, DOI 10.1093/nar/28.1.27
Kanehisa M, 2021, NUCLEIC ACIDS RES, V49, pD545, DOI 10.1093/nar/gkaa970
Kanehisa M, 2019, PROTEIN SCI, V28, P1947, DOI 10.1002/pro.3715
Kau D, 2020, NAT COMMUN, V11, DOI 10.1038/s41467-020-16429-9
Lahti L., 2017, Tools for microbiome analysis in R. Version 0.99.90
Lei N, 2020, EMERG MICROBES INFEC, V9, P976, DOI 10.1080/22221751.2020.1756696

Li H, 2019, SCI TOTAL ENVIRON, V649, P422, DOI 10.1016/j.scitotenv.2018.08.373
Lin H, 2020, NAT COMMUN, V11, DOI 10.1038/s41467-020-17041-7
Lin JD, 2020, CELL HOST MICROBE, V27, P830, DOI 10.1016/j.chom.2020.03.001
Liu Q, 2021, AM J PHYSIOL-LUNG C, V321, pL65, DOI 10.1152/ajplung.00421.2020
Love MI, 2014, GENOME BIOL, V15, DOI 10.1186/s13059-014-0550-8
Luo WJ, 2009, BMC BIOINFORMATICS, V10, DOI 10.1186/1471-2105-10-161
McCoy KD, 2019, IMMUNOL CELL BIOL, V97, P625, DOI 10.1111/imcb.12273
McMurdie PJ, 2013, PLOS ONE, V8, DOI 10.1371/journal.pone.0061217
Megahed A, 2019, SCI REP-UK, V9, DOI 10.1038/s41598-019-50187-z
Morris DE, 2017, FRONT MICROBIOL, V8, DOI 10.3389/fmicb.2017.01041
Ottman N, 2019, J ALLERGY CLIN IMMUN, V143, P1198, DOI 10.1016/j.jaci.2018.06.024
Quast C, 2013, NUCLEIC ACIDS RES, V41, pD590, DOI 10.1093/nar/gks1219
Rosshart SP, 2017, CELL, V171, P1015, DOI 10.1016/j.cell.2017.09.016
Ruokolainen L, 2017, CLIN EXP ALLERGY, V47, P665, DOI 10.1111/cea.12895
Schloissnig S, 2013, NATURE, V493, P45, DOI 10.1038/nature11711
Sonnenburg ED, 2019, NAT REV MICROBIOL, V17, P383, DOI 10.1038/s41579-019-0191-8
Tian XL, 2019, FRONT MICROBIOL, V10, DOI 10.3389/fmicb.2019.00159
Tighe RM, 2018, AM J RESP CELL MOL, V59, P257, DOI 10.1165/rcmb.2017-0357OC
Vacca M, 2020, MICROORGANISMS, V8, DOI 10.3390/microorganisms8040573
van Elden LJR, 2001, J CLIN MICROBIOL, V39, P196, DOI 10.1128/JCM.39.1.196-200.2001
Vose A, 2021, TOXICOL APPL PHARM, V426, DOI 10.1016/j.taap.2021.115645
Wickham H., 2016, ggplot2: elegant graphics for data analysis, DOI DOI 10.1007/978-3-319-24277-4
Zhang YC, 2020, CLIN TRANSL MED, V10, DOI 10.1002/ctm2.161
Zhou DR, 2016, ENVIRON MICROBIOL, V18, P1326, DOI 10.1111/1462-2920.12895
Zimmermann P, 2018, VACCINE, V36, P4433, DOI 10.1016/j.vaccine.2018.04.066
NR 63
TC 2
Z9 2
U1 1
U2 8
PU ELSEVIER
PI AMSTERDAM
PA RADARWEG 29, 1043 NX AMSTERDAM, NETHERLANDS
SN 0048-9697
EI 1879-1026
J9 SCI TOTAL ENVIRON
JI Sci. Total Environ.
PD APR 20
PY 2024
VL 922
AR 170865
DI 10.1016/j.scitotenv.2024.170865
EA MAR 2024

PG 10
WC Environmental Sciences
WE Science Citation Index Expanded (SCI-EXPANDED)
SC Environmental Sciences & Ecology
GA OB6V1
UT WOS:001204848200001
PM 38340827
DA 2025-05-07
ER

PT J
AU Houck, KM
   Thompson, AL
AF Houck, K. M.
   Thompson, A. L.
TI Fluid intake, hydration status and urinary tract infections in mothers
   and children from Galapagos, Ecuador.
SO AMERICAN JOURNAL OF HUMAN BIOLOGY
LA English
DT Meeting Abstract
C1 [Houck, K. M.; Thompson, A. L.] Univ N Carolina, Dept Anthropol,
Chapel Hill, NC 27515 USA.
   [Thompson, A. L.] Univ N Carolina, Carolina Populat Ctr, Chapel Hill,
NC 27515 USA.
C3 University of North Carolina; University of North Carolina Chapel
Hill;
   University of North Carolina; University of North Carolina Chapel Hill
FU NSF DDRIG [1341156]; Wenner-Gren Dissertation Fieldwork Grant;
Triangle
   Center for Evolutionary Medicine; NICHD, Carolina Population Center
   [5T32HD007168]; University of North Carolina's (UNC) Institute for
   Global Health and Infectious Disease; UNC's Royster Society of
Fellows;
   Division Of Behavioral and Cognitive Sci; Direct For Social, Behav &
   Economic Scie [1341156] Funding Source: National Science Foundation
FX NSF DDRIG (#1341156); Wenner-Gren Dissertation Fieldwork Grant;
Triangle
   Center for Evolutionary Medicine; NICHD 5T32HD007168, Carolina
   Population Center; University of North Carolina's (UNC) Institute for
   Global Health and Infectious Disease; UNC's Royster Society of
Fellows.
NR 0
TC 0
Z9 0
U1 0
U2 0
PU WILEY
PI HOBOKEN
PA 111 RIVER ST, HOBOKEN 07030-5774, NJ USA
SN 1042-0533
EI 1520-6300
J9 AM J HUM BIOL
JI Am. J. Hum. Biol.
PD APR

PY 2020
VL 32
SU 1
MA P/AM 67
PG 2
WC Anthropology; Biology
WE Science Citation Index Expanded (SCI-EXPANDED); Social Science Citation Index (SSCI)
SC Anthropology; Life Sciences & Biomedicine - Other Topics
GA LB2WU
UT WOS:000524499200058
DA 2025-05-07
ER

PT J
AU Dillon, MN
Qurollo, BA
Thomas, R
Warren, ME
Mousseau, TA
Betz, JA
Kleiman, NJ
Breen, M
AF Dillon, Megan N.
Qurollo, Barbara A.
Thomas, Rachael
Warren, Madeline E.
Mousseau, Timothy A.
Betz, Jennifer A.
Kleiman, Norman J.
Breen, Matthew
TI Contrasting pathogen prevalence between tick and dog populations at Chornobyl
SO PARASITES & VECTORS
LA English
DT Article
DE Chernobyl; Chornobyl; Ixodes ricinus; Ticks; Tick-borne pathogens; Zoonotic pathogens; Free-breeding dogs; Quantitative PCR; Droplet digital PCR
ID DERMACENTOR-RETICULATUS TICKS; ANAPLASMA-PHAGOCYTOPHILUM INFECTION; REAL-TIME PCR; BABESIA-CANIS; VERTICAL TRANSMISSION; BORRELIA-AFZELII; EXCLUSION ZONE; RADIATION; IRRADIATION; ABUNDANCE
AB BackgroundThe 1986 disaster at the Chornobyl Nuclear Power Plant released massive amounts of radioactive material into the local environment. In addition to radiation, remediation efforts and abandonment of military-industrial complexes contributed to contamination with heavy metals, organics, pesticides and other toxic chemicals. Numerous studies have evaluated the effects of this contamination on the local ecology. However, few studies have reported the effect of this contamination on vector-borne pathogens and their hosts. In this manuscript, we characterize tick-borne pathogen presence at two sample locations within the Chornobyl Exclusion Zone, one at the Nuclear Power Plant (NPP) and another 16 km away in Chornobyl City (CC).MethodsTicks and whole-blood samples were collected from free-breeding dogs captured

at the NPP and CC. Endpoint PCR and quantitative PCR were used to identify tick species and to assess the presence of specific tick-borne pathogens, including Anaplasma phagocytophilum, Borrelia burgdorferi sensu lato, Babesia spp., Bartonella spp., Francisella tularensis and general Anaplasmataceae. A droplet digital PCR assay was developed for Babesia canis and A. phagocytophilum to evaluate their presence in dogs from the two populations. Pathogen prevalences between the two sample populations were compared by calculating Z-scores.ResultsTicks were identified as Ixodes ricinus (n = 102) and Dermacentor reticulatus (n = 4). Overall, 56.9% of I. ricinus ticks were positive for at least one pathogen. A significantly higher prevalence of A. phagocytophilum and B. burgdorferi was found in ticks at the NPP (44.0% and 42.0%, respectively) compared to CC (23.1% and 19.2%, respectively). Babesia spp. (including B. canis and B. caballi) were detected in 8.8% ticks at similar proportions for both populations. Interestingly, we found a significantly lower level of A. phagocytophilum in dogs at the NPP (1.8%) than in dogs at CC (11.7%). In total, 24.3% of dogs were positive for B. canis, evenly distributed across the two populations.ConclusionsThe results of this study show contrasting pathogen prevalence in both ticks and dogs at the NPP and CC, which may reflect the differential exposures at the two locations. This work adds an important new component to our understanding of the consequences of prolonged exposure to environmental contamination on the wildlife and ecology within the Chornobyl Exclusion Zone.
C1 [Dillon, Megan N.; Thomas, Rachael; Breen, Matthew] North Carolina State Univ, Coll Vet Med, Dept Mol Biomed Sci, Raleigh, NC 27695 USA.
[Qurollo, Barbara A.] North Carolina State Univ, Coll Vet Med, Dept Clin Sci, Raleigh, NC USA.
[Warren, Madeline E.] North Carolina State Univ, Dept Biol Sci, Raleigh, NC USA.
[Mousseau, Timothy A.] Univ South Carolina, Dept Biol Sci, Columbia, SC USA.
[Mousseau, Timothy A.; Betz, Jennifer A.] Clean Futures Fund, Godfrey, IL USA.
[Mousseau, Timothy A.; Betz, Jennifer A.] Oregon Equine, Damascus, OR USA.
[Kleiman, Norman J.] Columbia Univ, Mailman Sch Publ Hlth, Dept Environm Hlth Sci, New York, NY USA.
[Breen, Matthew] North Carolina State Univ, Comparat Med Inst, Raleigh, NC 27695 USA.
[Breen, Matthew] North Carolina State Univ, Ctr Human Hlth & Environm, Raleigh, NC USA.
[Breen, Matthew] Univ North Carolina, UNC Lineberger Comprehens Canc Ctr, Canc Genet, Chapel Hill, NC 27599 USA.
[Breen, Matthew] Duke Univ, Duke Canc Inst, Durham, NC 27708 USA.
C3 North Carolina State University; North Carolina State University; North
Carolina State University; University of South Carolina System; University of South Carolina Columbia; Columbia University; North Carolina State University; North Carolina State University; University of North Carolina; University of North Carolina Chapel Hill; Duke University
RP Breen, M (autor correspondente), North Carolina State Univ, Coll Vet Med, Dept Mol Biomed Sci, Raleigh, NC 27695 USA.; Breen, M (autor correspondente), North Carolina State Univ, Comparat Med Inst, Raleigh,

NC 27695 USA.; Breen, M (autor correspondente), North Carolina State Univ, Ctr Human Hlth & Environm, Raleigh, NC USA.; Breen, M (autor correspondente), Univ North Carolina, UNC Lineberger Comprehens Canc Ctr, Canc Genet, Chapel Hill, NC 27599 USA.; Breen, M (autor correspondente), Duke Univ, Duke Canc Inst, Durham, NC 27708 USA.
EM Matthew_breen@ncsu.edu
RI Kleiman, Norman/B-9745-2008; Mousseau, Timothy/A-1253-2011
OI Dillon, Megan/0000-0001-9364-2528; Breen, Matthew/0000-0002-8901-4155; Qurollo, Barbara/0000-0002-9849-2511
FU Triangle Comparative and Evolutionary Medicine Center, Duke University;
Society for the Prevention of Cruelty to Animals International; Triangle
Center for Evolutionary Medicine (TriCEM); University of South Carolina
Office of Research; University of South Carolina Honors College; Samuel
Freeman Charitable Trust
FX The authors wish to thank I. Livingston, for support with cataloguing the samples, and also M. Reiskind and E. Breitschwerdt, for useful discussion. We thank E. Kambarian from the Clean Futures Fund for organizing the canine wellness campaigns in Chornobyl, Ukraine. We also thank G. Spatola, J. Chertok, K. Russell, N. Hank, C. Rulison, J. Hecla and all volunteers who participated in the Dogs of Chernobyl animal wellness campaigns for assistance with field work and sample collection.
Veterinary clinics in Chornobyl were supported by Clean Futures Fund International and the Society for the Prevention of Cruelty to Animals International. We would like to thank the Triangle Center for Evolutionary Medicine (TriCEM) for their funding contribution towards laboratory supplies (MND). Funding for field work and students was provided by the University of South Carolina Office of Research (to TAM), University of South Carolina Honors College (to TAM) and the Samuel Freeman Charitable Trust (to TAM).
CR Adaszek L, 2016, VET MED-CZECH, V61, P263, DOI 10.17221/8881-VETMED
[Anonymous], 2000, UNSCEAR 2000 REPORT, DOI DOI 10.18356/2C4203DC-EN
Bajer A, 2023, PARASITE VECTOR, V16, DOI 10.1186/s13071-023-06042-2
Baker RJ, 1996, J MAMMAL, V77, P155, DOI 10.2307/1382717
Benová K, 2009, ACTA VET BRNO, V78, P173, DOI 10.2754/avb200978010173
Boratynski Z, 2014, SCI REP-UK, V4, DOI 10.1038/srep07141
Boskabady M, 2018, ENVIRON INT, V120, P404, DOI 10.1016/j.envint.2018.08.013
Courtney JW, 2004, J CLIN MICROBIOL, V42, P3164, DOI 10.1128/JCM.42.7.3164-3168.2004
De Cort M, 1998, EUR Report Nr. 16733
Deryabina TG, 2015, CURR BIOL, V25, pR824, DOI 10.1016/j.cub.2015.08.017
Diaz MH, 2012, J CLIN MICROBIOL, V50, P1645, DOI 10.1128/JCM.06621-11
Didyk YM, 2017, TICKS TICK-BORNE DIS, V8, P219, DOI 10.1016/j.ttbdis.2016.10.002
Dillon Megan N, 2023, Canine Med Genet, V10, P1, DOI 10.1186/s40575-023-00124-1

Dugat T, 2015, FRONT CELL INFECT MI, V5, DOI 10.3389/fcimb.2015.00061
Eberts MD, 2011, J AM ANIM HOSP ASSOC, V47, pE86, DOI 10.5326/JAAHA-MS-5578
ENTREKIN DL, 1987, J PARASITOL, V73, P549, DOI 10.2307/3282135
Erdélyi K, 2014, TICKS TICK-BORNE DIS, V5, P281, DOI 10.1016/j.ttbdis.2013.11.003
Foley JE, 2009, AM J TROP MED HYG, V81, P1132, DOI 10.4269/ajtmh.2009.09-0372
Geras'kin SA, 2008, ENVIRON INT, V34, P880, DOI 10.1016/j.envint.2007.12.012
Gosudarstvennyj Komitet po Ispol'zovaniyu Atomnoj Ehnergii SSSR, 1986, Report no. INIS-mf-10523
Hanincová K, 2003, PARASITOLOGY, V126, P11, DOI 10.1017/S0031182002002548
Higginbotham A, 2019, The liquidators. Midnight in Chernobyl: the untold story of the world's greatest nuclear disaster
Higginbotham A, 2019, The Soviet Prometheus. Midnight in Chernobyl: the untold story of the world's greatest nuclear disaster
Hollingsworth BA, 2021, FRONT PHARMACOL, V12, DOI 10.3389/fphar.2021.643283
Hussain S, 2021, MICROORGANISMS, V9, DOI 10.3390/microorganisms9122412
Jiang J, 2012, TICKS TICK-BORNE DIS, V3, P326, DOI 10.1016/j.ttbdis.2012.10.004
Karbowiak G, 2016, EMERG INFECT DIS, V22, P2214, DOI 10.3201/eid2212.160678
Karbowiak G, 2014, VET PARASITOL, V204, P372, DOI 10.1016/j.vetpar.2014.05.030
Khan Khalid I., 2004, Polish Journal of Microbiology, V53, P159
Kocon A, 2020, SCI REP-UK, V10, DOI 10.1038/s41598-020-72981-w
Kormilitsyna MI, 2013, MOL GENET MICROBIOL+, V28, P110, DOI 10.3103/S0891416813030051
Kovryha N, 2021, VECTOR-BORNE ZOONOT, V21, P242, DOI 10.1089/vbz.2020.2716
Król N, 2016, PARASITE VECTOR, V9, DOI 10.1186/s13071-016-1632-0
Krücken J, 2013, J CLIN MICROBIOL, V51, P1958, DOI 10.1128/JCM.00284-13
Krupka I, 2010, VET CLIN N AM-SMALL, V40, P1103, DOI 10.1016/j.cvsm.2010.07.011
Kuo CY, 2020, TICKS TICK-BORNE DIS, V11, DOI 10.1016/j.ttbdis.2019.101362
Lerebours A, 2023, SCI TOTAL ENVIRON, V902, DOI 10.1016/j.scitotenv.2023.165957
Lerebours A, 2018, ENVIRON SCI TECHNOL, V52, P9442, DOI 10.1021/acs.est.8b02378
Levytska VA, 2021, TICKS TICK-BORNE DIS, V12, DOI 10.1016/j.ttbdis.2020.101586
Liberska J, 2021, TICKS TICK-BORNE DIS, V12, DOI 10.1016/j.ttbdis.2021.101786
Lv JZ, 2014, PARASITE VECTOR, V7, DOI 10.1186/1756-3305-7-93
Maslov VI, 1967, Characteristics of the radioecological groups of mammals and birds of biogeocoenoses with high natural radiation. Radioecological concentration processes
Michalski MM, 2020, PATHOGENS, V9, DOI 10.3390/pathogens9060455

Mierzejewskal EJ, 2014, ANN AGR ENV MED, V21, P500, DOI 10.5604/12321966.1120590
Mochizuki H, 2016, VET PATHOL, V53, P764, DOI 10.1177/0300985815614975
Moller AP, 2013, ECOL INDIC, V26, P112, DOI 10.1016/j.ecolind.2012.10.025
Moller AP, 2005, J ANIM ECOL, V74, P1102, DOI 10.1111/j.1365-2656.2005.01009.x
Morley NJ, 2012, J ENVIRON RADIOACTIV, V106, P81, DOI 10.1016/j.jenvrad.2011.12.019
Mousseau TA, 2013, PLOS ONE, V8, DOI 10.1371/journal.pone.0066939
Movila A, 2012, J PARASITOL, V98, P883, DOI 10.1645/GE-3131.1
Mukherjee D, 2014, J PATHOL, V232, P289, DOI 10.1002/path.4299
National Academies of Sciences Engineering and Medicine, 2022, COMP AN SENT PRED EN, DOI [10.17226/26547, DOI 10.17226/26547]
Onyiche TE, 2021, PATHOGENS, V10, DOI 10.3390/pathogens10020230
PURNELL RE, 1972, PARASITOLOGY, V64, P429, DOI 10.1017/S0031182000045492
Qurollo BA, 2018, TICKS TICK-BORNE DIS, V9, P672, DOI 10.1016/j.ttbdis.2018.02.012
Qurollo BA, 2017, PARASITE VECTOR, V10, DOI 10.1186/s13071-017-2064-1
Robinson MT, 2010, EXP PARASITOL, V126, P506, DOI 10.1016/j.exppara.2010.05.026
Rochlin I, 2020, J MED MICROBIOL, V69, P781, DOI 10.1099/jmm.0.001206
Rogovskyy AS, 2019, VECTOR-BORNE ZOONOT, V19, P466, DOI 10.1089/vbz.2018.2318
Rogovskyy AS, 2017, TICKS TICK-BORNE DIS, V8, P99, DOI 10.1016/j.ttbdis.2016.10.004
Shabalev SI, 2012, Mater Res Soc Symp Proc, V465, P1343, DOI [10.1557/proc-465-1343, DOI 10.1557/PROC-465-1343]
Spatola GJ, 2023, SCI ADV, V9, DOI 10.1126/sciadv.ade2537
Turnbull J, 2020, ANTHROPOL TODAY, V36, P21, DOI 10.1111/1467-8322.12620
Tyrrell JD, 2019, ACTA TROP, V200, DOI 10.1016/j.actatropica.2019.105170
Varloud M, 2018, PARASITE VECTOR, V11, DOI 10.1186/s13071-018-2637-7
Webster SC, 2016, FRONT ECOL ENVIRON, V14, P185, DOI 10.1002/fee.1227
Yablokov AV, 2009, ANN NY ACAD SCI, V1181, P318, DOI [10.1111/j.1749-6632.2009.04841.x, 10.1111/j.1749-6632.2009.04836.x]
Zygner W, 2008, VET PARASITOL, V153, P139, DOI 10.1016/j.vetpar.2008.01.036
NR 68
TC 0
Z9 0
U1 2
U2 2
PU BMC
PI LONDON
PA CAMPUS, 4 CRINAN ST, LONDON N1 9XW, ENGLAND
SN 1756-3305
J9 PARASITE VECTOR
JI Parasites Vectors
PD NOV 17
PY 2024
VL 17

IS 1
AR 470
DI 10.1186/s13071-024-06563-4
PG 15
WC Parasitology; Tropical Medicine
WE Science Citation Index Expanded (SCI-EXPANDED)
SC Parasitology; Tropical Medicine
GA M3G9I
UT WOS:001356470900001
PM 39551770
OA Green Published
DA 2025-05-07
ER

PT J
AU Tueffers, L
Barbosa, C
Bobis, I
Schubert, S
Höppner, M
Rühlemann, M
Franke, A
Rosenstiel, P
Friedrichs, A
Krenz-Weinreich, A
Fickenscher, H
Bewig, B
Schreiber, S
Schulenburg, H
AF Tueffers, Leif
Barbosa, Camilo
Bobis, Ingrid
Schubert, Sabine
Hoeppner, Marc
Ruehlemann, Malte
Franke, Andre
Rosenstiel, Philip
Friedrichs, Anette
Krenz-Weinreich, Annegret
Fickenscher, Helmut
Bewig, Burkhard
Schreiber, Stefan
Schulenburg, Hinrich
TI Pseudomonas aeruginosa populations in the cystic fibrosis lung lose susceptibility to newly applied β-lactams within 3 days
SO JOURNAL OF ANTIMICROBIAL CHEMOTHERAPY
LA English
DT Article
ID ANTIBIOTIC-RESISTANCE; BIOLOGICAL COST; READ ALIGNMENT; EVOLUTION; ADAPTATION; STRAINS; GENOME; HYPERMUTATION; MECHANISMS; FRAMEWORK
AB Background Chronic pulmonary infections by Pseudomonas aeruginosa require frequent intravenous antibiotic treatment in cystic fibrosis (CF) patients. Emergence of antimicrobial resistance is common in these

patients, which to date has been investigated at long-term intervals only.
Objectives To investigate under close to real-time conditions the dynamics of the response by P. aeruginosa to a single course of antibiotic therapy and the potentially associated rapid spread of antimicrobial resistance, as well as the impact on the airway microbiome.
Methods We investigated a cohort of adult CF patients that were treated with a single course of antimicrobial combination therapy. Using daily sampling during treatment, we quantified the expression of resistance by P. aeruginosa (median of six isolates per daily sample, 347 isolates in total), measured bacterial load by P. aeruginosa-specific quantitative PCR and characterized the airway microbiome with a 16S rRNA-based approach. WGS was performed to reconstruct intrapatient strain phylogenies.
Results In two patients, we found rapid and large increases in resistance to meropenem and ceftazidime. Phylogenetic reconstruction of strain relationships revealed that resistance shifts are probably due to de novo evolution and/or the selection of resistant subpopulations. We observed high interindividual variation in the reduction of bacterial load, microbiome composition and antibiotic resistance.
Conclusions We show that CF-associated P. aeruginosa populations can quickly respond to antibiotic therapy and that responses are patient specific. Thus, resistance evolution can be a direct consequence of treatment, and drug efficacy can be lost much faster than usually assumed. The consideration of these patient-specific rapid resistance shifts can help to improve treatment of CF-associated infections, for example by deeper sampling of bacteria for diagnostics, repeated monitoring of pathogen susceptibility and switching between drugs.
C1 [Tueffers, Leif; Barbosa, Camilo; Schulenburg, Hinrich] Christian Albrechts Univ Kiel, Zool Inst, Evolutionary Ecol & Genet, Bot Garten 1-9, D-24118 Kiel, Germany.
[Bobis, Ingrid; Friedrichs, Anette; Bewig, Burkhard; Schreiber, Stefan] Univ Med Ctr Schleswig Holstein, Dept Internal Med 1, Kiel Campus,Arnold Heller Str 3, D-24105 Kiel, Germany.
[Schubert, Sabine; Fickenscher, Helmut] Christian Albrechts Univ Kiel, Inst Infect Med, Brunswiker Str 4, D-24105 Kiel, Germany.
[Schubert, Sabine; Fickenscher, Helmut] Univ Med Ctr Schleswig Holstein, Brunswiker Str 4, D-24105 Kiel, Germany.
[Hoeppner, Marc; Ruehlemann, Malte; Franke, Andre; Rosenstiel, Philip; Schreiber, Stefan] Christian Albrechts Univ Kiel, Inst Clin Mol Biol, Rosalind Franklin Str 12, D-24105 Kiel, Germany.
[Krenz-Weinreich, Annegret] LADR Labs Plon, Krogen 6, D-24306 Plon, Germany.
C3 University of Kiel; University of Kiel; Schleswig Holstein University Hospital; University of Kiel; University of Kiel; Schleswig Holstein University Hospital; University of Kiel
RP Schulenburg, H (autor correspondente), Christian Albrechts Univ Kiel, Zool Inst, Evolutionary Ecol & Genet, Bot Garten 1-9, D-24118 Kiel, Germany.
EM hschulenburg@zoologie.uni-kiel.de
RI Schreiber, Stefan/AFM-9178-2022; Rühlemann, Malte/AAA-6528-2019; Bobis,
Ingrid/MCX-9075-2025; Franke, Andre/B-2151-2010; Barbosa, Camilo/AAC-5935-2019; Fickenscher, Helmut/J-9655-2019; Barbosa,

Camilo/IVV-0365-2023; Rosenstiel, Philip/A-5137-2009
OI Ruhlemann, Malte/0000-0002-0685-0052; Tuffers, Leif/0000-0001-5496-9456;
Barbosa, Camilo/0000-0003-0433-9311; Rosenstiel,
Philip/0000-0002-9692-8828
FU Leibniz Science Campus 'Evolutionary Medicine of the Lung';
International Max Planck Research School for Evolutionary Biology; Max
Planck Society; German Research Foundation (DFG) under Germany's
Excellence Strategy [EXC 2216739088401]
FX This work was funded by the Leibniz Science Campus 'Evolutionary
Medicine of the Lung' (grants to L. T., C. B. and H. S.), the
International Max Planck Research School for Evolutionary Biology (L. T.
and C. B.), the Max Planck Society (Fellowship to H. S.) and the German
Research Foundation (DFG) under Germany's Excellence Strategy -EXC
2216739088401 (Excellence Cluster Precision Medicine in Chronic
Inflammation; A. Franke, P. R., S. Schreiber and H. S.).
CR [Anonymous], 2016, ERJ OPEN RES, DOI DOI 10.1016/S1569-1993(16)30337-X
Barbosa C, 2018, PLOS BIOL, V16, DOI 10.1371/journal.pbio.2004356
Beceiro A, 2014, ANTIMICROB AGENTS CH, V58, P518, DOI 10.1128/AAC.01597-13
Berrazeg M, 2015, ANTIMICROB AGENTS CH, V59, P6248, DOI 10.1128/AAC.00825-15
Bolger AM, 2014, BIOINFORMATICS, V30, P2114, DOI 10.1093/bioinformatics/btu170
Boutin S, 2017, EUR RESPIR J, V50, DOI 10.1183/13993003.01086-2017
Breidenstein EBM, 2011, TRENDS MICROBIOL, V19, P419, DOI 10.1016/j.tim.2011.04.005
Cabot G, 2016, ANTIMICROB AGENTS CH, V60, P7415, DOI 10.1128/AAC.01720-16
Cingolani P, 2012, FLY, V6, P80, DOI 10.4161/fly.19695
Ciofu O, 2015, ADV DRUG DELIVER REV, V85, P7, DOI 10.1016/j.addr.2014.11.017
Clark ST, 2019, INT J ANTIMICROB AG, V53, P620, DOI 10.1016/j.ijantimicag.2019.01.009
Cornforth DM, 2018, P NATL ACAD SCI USA, V115, pE5125, DOI 10.1073/pnas.1717525115
Curran B, 2004, J CLIN MICROBIOL, V42, P5644, DOI 10.1128/JCM.42.12.5644-5649.2004
DePristo MA, 2011, NAT GENET, V43, P491, DOI 10.1038/ng.806
Deschaght P, 2013, PLOS ONE, V8, DOI 10.1371/journal.pone.0079010
Deschaght P, 2009, BMC MICROBIOL, V9, DOI 10.1186/1471-2180-9-244
Edgar RC, 2004, NUCLEIC ACIDS RES, V32, P1792, DOI 10.1093/nar/gkh340
Edgar RC, 2010, BIOINFORMATICS, V26, P2460, DOI 10.1093/bioinformatics/btq461
European Committee on Antimicrobial Susceptibility Testing, 2018, BREAKPOINT TABLES IN
Fernández-Barat L, 2017, J CYST FIBROS, V16, P222, DOI 10.1016/j.jcf.2016.08.005
Ferroni A, 2009, PEDIATR PULM, V44, P820, DOI 10.1002/ppul.21076
Fodor AA, 2012, PLOS ONE, V7, DOI 10.1371/journal.pone.0045001
Folkesson A, 2012, NAT REV MICROBIOL, V10, P841, DOI 10.1038/nrmicro2907

Fothergill JL, 2010, J MED MICROBIOL, V59, P472, DOI 10.1099/jmm.0.015875-0
Gellatly SL, 2013, PATHOG DIS, V67, P159, DOI 10.1111/2049-632X.12033
GIWERCMAN B, 1990, J ANTIMICROB CHEMOTH, V26, P247, DOI 10.1093/jac/26.2.247
Hoffman LR, 2009, J CYST FIBROS, V8, P66, DOI 10.1016/j.jcf.2008.09.006
Huang YJ, 2016, CLIN CHEST MED, V37, P59, DOI 10.1016/j.ccm.2015.10.003
Jansen G, 2016, EVOL MED PUBLIC HLTH, P182, DOI 10.1093/emph/eow016
Jorth P, 2017, MBIO, V8, DOI 10.1128/mBio.00517-17
Kramer R, 2015, PLOS ONE, V10, DOI 10.1371/journal.pone.0117436
Langmead B, 2012, NAT METHODS, V9, P357, DOI [10.1038/NMETH.1923, 10.1038/nmeth.1923]
Larsson A, 2014, BIOINFORMATICS, V30, P3276, DOI 10.1093/bioinformatics/btu531
Letunic I, 2016, NUCLEIC ACIDS RES, V44, pW242, DOI 10.1093/nar/gkw290
Li H, 2009, BIOINFORMATICS, V25, P1754, DOI [10.1093/bioinformatics/btp698, 10.1093/bioinformatics/btp324, 10.1093/bioinformatics/btp352]
López-Causapé C, 2017, SCI REP-UK, V7, DOI 10.1038/s41598-017-05621-5
Marvig RL, 2015, NAT GENET, V47, P57, DOI 10.1038/ng.3148
McKenna A, 2010, GENOME RES, V20, P1297, DOI 10.1101/gr.107524.110
Montanari S, 2007, MICROBIOL-SGM, V153, P1445, DOI 10.1099/mic.0.2006/003400-0
Moya B, 2009, PLOS PATHOG, V5, DOI 10.1371/journal.ppat.1000353
Oliver A, 2010, CLIN MICROBIOL INFEC, V16, P798, DOI 10.1111/j.1469-0691.2010.03250.x
Oliver A, 2010, INT J MED MICROBIOL, V300, P563, DOI 10.1016/j.ijmm.2010.08.009
Parkins MD, 2012, CHEST, V141, P485, DOI 10.1378/chest.11-0917
Riou M, 2010, INT J ANTIMICROB AG, V36, P513, DOI 10.1016/j.ijantimicag.2010.08.005
Roemhild R, 2015, EVOL APPL, V8, P945, DOI 10.1111/eva.12330
Rosay T, 2015, MBIO, V6, DOI 10.1128/mBio.01033-15
Schulenburg JHGV, 2001, APPL ENVIRON MICROB, V67, P270, DOI 10.1128/AEM.67.1.270-277.2001
Solé M, 2015, J ANTIMICROB CHEMOTH, V70, P3004, DOI 10.1093/jac/dkv228
Stamatakis A, 2014, BIOINFORMATICS, V30, P1312, DOI 10.1093/bioinformatics/btu033
Stressmann FA, 2011, J CYST FIBROS, V10, P357, DOI 10.1016/j.jcf.2011.05.002
Winstanley C, 2016, TRENDS MICROBIOL, V24, P327, DOI 10.1016/j.tim.2016.01.008
Woods Robert J., 2015, Evolution Medicine and Public Health, P281, DOI 10.1093/emph/eov025
Yang L, 2011, P NATL ACAD SCI USA, V108, P7481, DOI 10.1073/pnas.1018249108
NR 53
TC 21
Z9 22
U1 0
U2 6
PU OXFORD UNIV PRESS

PI OXFORD
PA GREAT CLARENDON ST, OXFORD OX2 6DP, ENGLAND
SN 0305-7453
EI 1460-2091
J9 J ANTIMICROB CHEMOTH
JI J. Antimicrob. Chemother.
PD OCT
PY 2019
VL 74
IS 10
BP 2916
EP 2925
DI 10.1093/jac/dkz297
PG 10
WC Infectious Diseases; Microbiology; Pharmacology & Pharmacy
WE Science Citation Index Expanded (SCI-EXPANDED)
SC Infectious Diseases; Microbiology; Pharmacology & Pharmacy
GA JF2ZB
UT WOS:000491253800012
PM 31355848
DA 2025-05-07
ER

PT J
AU Krulikas, LJ
McDonald, IM
Lee, B
Okumu, DO
East, MP
Gilbert, TSK
Herring, LE
Golitz, BT
Wells, CI
Axtman, AD
Zuercher, WJ
Willson, TM
Kireev, D
Yeh, JJ
Johnson, GL
Baines, AT
Graves, LM
AF Krulikas, Linas J.
McDonald, Ian M.
Lee, Benjamin
Okumu, Denis O.
East, Michael P.
Gilbert, Thomas S. K.
Herring, Laura E.
Golitz, Brian T.
Wells, Carrow I.
Axtman, Allison D.
Zuercher, William J.
Willson, Timothy M.
Kireev, Dmitri

Yeh, Jen Jen
Johnson, Gary L.
Baines, Antonio T.
Graves, Lee M.
TI Application of Integrated Drug Screening/Kinome Analysis to Identify Inhibitors of Gemcitabine-Resistant Pancreatic Cancer Cell Growth
SO SLAS DISCOVERY
LA English
DT Article
DE kinases; high-content screening; fluorescence methods; drug interactions; multiplex assays and technology
ID KINOME; CHALLENGES; DISCOVERY; CULTURE
AB Continuous exposure of a pancreatic cancer cell line MIA PaCa-2 (Mia(S)) to gemcitabine resulted in the formation of a gemcitabine-resistant subline (Mia(R)). In an effort to discover kinase inhibitors that inhibited Mia(R) growth, Mia(R) cells were exposed to kinase inhibitors (PKIS-1 library) in a 384-well screening format. Three compounds (UNC10112721A, UNC10112652A, and UNC10112793A) were identified that inhibited the growth of Mia(R) cells by more than 50% (at 50 nM). Two compounds (UNC10112721A and UNC10112652A) were classified as cyclin-dependent kinase (CDK) inhibitors, whereas UNC10112793A was reported to be a PLK inhibitor. Dose-response experiments supported the efficacy of these compounds to inhibit growth and increase apoptosis in 2D cultures of these cells. However, only UNC10112721A significantly inhibited the growth of 3D spheroids composed of Mia(R) cells and GFP-tagged cancer-associated fibroblasts. Multiplexed inhibitor bead (MIB)-mass spectrometry (MS) kinome competition experiments identified CDK9, CLK1-4, DYRK1A, and CSNK1 as major kinase targets for UNC10112721A in Mia(R) cells. Another CDK9 inhibitor (CDK-IN-2) replicated the growth inhibitory effects of UNC10112721A, whereas inhibitors against the CLK, DYRK, or CSNK1 kinases had no effect. In summary, these studies describe a coordinated approach to discover novel kinase inhibitors, evaluate their efficacy in 3D models, and define their specificity against the kinome.
C1 [Krulikas, Linas J.; McDonald, Ian M.; Lee, Benjamin; Okumu, Denis O.; East, Michael P.; Gilbert, Thomas S. K.; Herring, Laura E.; Golitz, Brian T.; Johnson, Gary L.; Graves, Lee M.] Univ N Carolina, Dept Pharmacol, Chapel Hill, NC 27515 USA.
[Wells, Carrow I.; Zuercher, William J.; Willson, Timothy M.] Univ N Carolina, Struct Genom Consortium, Chapel Hill, NC 27515 USA.
[Axtman, Allison D.; Kireev, Dmitri] Univ N Carolina, Eshelman Sch Pharm, Chapel Hill, NC 27515 USA.
[Yeh, Jen Jen] Univ N Carolina, Lineberger Canc Ctr, Chapel Hill, NC 27515 USA.
[Baines, Antonio T.] North Carolina Cent Univ, Durham, NC 27707 USA.
C3 University of North Carolina; University of North Carolina Chapel Hill;
University of North Carolina; University of North Carolina Chapel Hill;
University of North Carolina; University of North Carolina Chapel Hill;
University of North Carolina; University of North Carolina Chapel Hill;
University of North Carolina; North Carolina Central University

RP Graves, LM (autor correspondente), Univ N Carolina, Dept Pharmacol, Chapel Hill, NC 27515 USA.
EM lmg@med.unc.edu
RI feinstein, doug/M-9414-2019; , Proteomics Core UNC/AFQ-7201-2022; Graves, Lee/AAG-5470-2021; Gilbert, Thomas/E-5187-2013
OI Axtman, Alison/0000-0003-4779-9932; Lee, Benjamin/0000-0003-1040-9443; Willson, Timothy/0000-0003-4181-8223; Gilbert, Thomas/0000-0002-6833-6788; Krulikas, Linas/0009-0008-6139-4580
FU National Institutes of Health [R01 CA199064, U01-MH104999-01]; Triangle Center for Evolutionary Medicine; UNC University Cancer Research Fund
FX We are grateful to the staff of UNC Michael Hooker Proteomics Core Facility for their help with running samples, and data acquisition and analyses. We are thankful to the Structural Genomics Consortium for supplying their inhibitor library and providing technical support. We would also like to thank the National Institutes of Health (R01 CA199064 and U01-MH104999-01), the Triangle Center for Evolutionary Medicine (L.M.G. and A.T.B.), and the UNC University Cancer Research Fund (L.M.G.) for their support of this research.
CR Anderson GR, 2017, CELL REP, V20, P999, DOI 10.1016/j.celrep.2017.07.006
[Anonymous], J CELL BIOCH
[Anonymous], 2013, PLoS ONE
Arend KC, 2017, MOL CELL PROTEOMICS, V16, pS263, DOI 10.1074/mcp.M116.065375
Aroldi F, 2016, WORLD J GASTRO ONCOL, V8, P682, DOI 10.4251/wjgo.v8.i9.682
Attieh Y, 2017, J CELL BIOL, V216, P3509, DOI 10.1083/jcb.201702033
Attieh Y, 2016, EUR J CELL BIOL, V95, P493, DOI 10.1016/j.ejcb.2016.07.004
Bantscheff M, 2007, Ernst Schering Found Symp Proc, P1
Boone BA, 2013, J SURG ONCOL, V108, P236, DOI 10.1002/jso.23392
Borazanci E, 2017, CLIN CANCER RES, V23, P1629, DOI 10.1158/1078-0432.CCR-16-2070
Breslin S, 2016, ONCOTARGET, V7, P45745, DOI 10.18632/oncotarget.9935
Cancer Genome Atlas Research Network. Electronic address: andrew_aguirre@dfci.harvard.edu, 2017, Cancer Cell, V32, P185, DOI 10.1016/j.ccell.2017.07.007
Cox AD, 2014, NAT REV DRUG DISCOV, V13, P828, DOI 10.1038/nrd4389
Dixon-Clarke SE, 2017, BIOCHEM J, V474, P699, DOI 10.1042/BCJ20160941
Drewry DH, 2017, PLOS ONE, V12, DOI 10.1371/journal.pone.0181585
Drewry DH, 2014, CURR TOP MED CHEM, V14, P340, DOI 10.2174/1568026613666131127160819
Duncan JS, 2012, CELL, V149, P307, DOI 10.1016/j.cell.2012.02.053
Edmondson R, 2016, PLOS ONE, V11, DOI 10.1371/journal.pone.0158116
Edmondson R, 2014, ASSAY DRUG DEV TECHN, V12, P207, DOI 10.1089/adt.2014.573
Elkins JM, 2016, NAT BIOTECHNOL, V34, P95, DOI 10.1038/nbt.3374
Eser S, 2014, BRIT J CANCER, V111, P817, DOI 10.1038/bjc.2014.215
Fang Y, 2017, SLAS DISCOV, V22, P456, DOI 10.1177/1087057117696795
Gil-Bazo I, 2016, CANCER BIOL THER, V17, P719, DOI 10.1080/15384047.2016.1190487

Kalra S, 2017, EUR J MED CHEM, V142, P424, DOI
10.1016/j.ejmech.2017.08.071
Klaeger S, 2017, SCIENCE, V358, DOI 10.1126/science.aan4368
Lemke J, 2014, CELL DEATH DIFFER, V21, P491, DOI 10.1038/cdd.2013.179
Lücking U, 2017, CHEMMEDCHEM, V12, P1776, DOI 10.1002/cmdc.201700447
McMillin DW, 2013, NAT REV DRUG DISCOV, V12, P217, DOI 10.1038/nrd3870
Mehta G, 2012, J CONTROL RELEASE, V164, P192, DOI
10.1016/j.jconrel.2012.04.045
Okumu DO, 2017, PLOS ONE, V12, DOI 10.1371/journal.pone.0177871
Stuhlmiller TJ, 2015, CELL REP, V11, P390, DOI
10.1016/j.celrep.2015.03.037
Sun T, 2006, J BIOTECHNOL, V122, P372, DOI
10.1016/j.jbiotec.2005.12.021
Theodoraki MA, 2015, ONCOTARGET, V6, P21255, DOI
10.18632/oncotarget.4013
Vinci M, 2012, BMC BIOL, V10, DOI 10.1186/1741-7007-10-29
Ware MJ, 2016, BIOMATERIALS, V108, P129, DOI
10.1016/j.biomaterials.2016.08.041
Xiao Q, 2016, CANCER RES, V76, P5395, DOI 10.1158/0008-5472.CAN-15-
3264
Xu MD, 2016, ANTI-CANCER AGENT ME, V16, P1117, DOI
10.2174/1871520616666160404115532
Yin TG, 2014, MOL CANCER THER, V13, P1442, DOI 10.1158/1535-7163.MCT-
13-0849
Zeitouni D, 2016, CANCERS, V8, DOI 10.3390/cancers8040045
NR 39
TC 13
Z9 17
U1 0
U2 9
PU ELSEVIER SCIENCE INC
PI NEW YORK
PA STE 800, 230 PARK AVE, NEW YORK, NY 10169 USA
SN 2472-5552
EI 2472-5560
J9 SLAS DISCOV
JI SLAS Discov.
PD SEP
PY 2018
VL 23
IS 8
BP 850
EP 861
DI 10.1177/2472555218773045
PG 12
WC Biochemical Research Methods; Biotechnology & Applied Microbiology;
Chemistry, Analytical
WE Science Citation Index Expanded (SCI-EXPANDED)
SC Biochemistry & Molecular Biology; Biotechnology & Applied
Microbiology;
Chemistry
GA GR1FE
UT WOS:000442275300009
PM 29742358

OA Green Accepted, hybrid
DA 2025-05-07
ER

PT J
AU Ryser, MD
Mallo, D
Hall, A
Hardman, T
King, LM
Tatishchev, S
Sorribes, IC
Maley, CC
Marks, JR
Hwang, ES
Shibata, D
AF Ryser, Marc D.
Mallo, Diego
Hall, Allison
Hardman, Timothy
King, Lorraine M.
Tatishchev, Sergei
Sorribes, Inmaculada C.
Maley, Carlo C.
Marks, Jeffrey R.
Hwang, E. Shelley
Shibata, Darryl
TI Minimal barriers to invasion during human colorectal tumor growth
SO NATURE COMMUNICATIONS
LA English
DT Article
ID CANCER; HETEROGENEITY; EVOLUTION; MODEL
AB Intra-tumoral heterogeneity (ITH) could represent clonal evolution where subclones with greater fitness confer more malignant phenotypes and invasion constitutes an evolutionary bottleneck. Alternatively, ITH could represent branching evolution with invasion of multiple subclones. The two models respectively predict a hierarchy of subclones arranged by phenotype, or multiple subclones with shared phenotypes. We delineate these modes of invasion by merging ancestral, topographic, and phenotypic information from 12 human colorectal tumors (11 carcinomas, 1 adenoma) obtained through saturation microdissection of 325 small tumor regions. The majority of subclones (29/46, 60%) share superficial and invasive phenotypes. Of 11 carcinomas, 9 show evidence of multiclonal invasion, and invasive and metastatic subclones arise early along the ancestral trees. Early multiclonal invasion in the majority of these tumors indicates the expansion of co-evolving subclones with similar malignant potential in absence of late bottlenecks and suggests that barriers to invasion are minimal during colorectal cancer growth.
C1 [Ryser, Marc D.] Duke Univ, Med Ctr, Dept Populat Hlth Sci, Durham, NC 27708 USA.
[Ryser, Marc D.; Sorribes, Inmaculada C.] Duke Univ, Dept Math, Durham, NC 27706 USA.
[Ryser, Marc D.; Marks, Jeffrey R.; Hwang, E. Shelley] Duke Canc Inst, Durham, NC 27710 USA.

[Mallo, Diego; Maley, Carlo C.] Arizona State Univ, Arizona Canc Evolut Ctr, Tempe, AZ USA.
[Mallo, Diego; Maley, Carlo C.] Arizona State Univ, Sch Life Sci, Tempe, AZ USA.
[Hall, Allison] Duke Univ, Med Ctr, Dept Pathol, Durham, NC 27710 USA.
[Hardman, Timothy; King, Lorraine M.; Marks, Jeffrey R.; Hwang, E. Shelley] Duke Univ, Med Ctr, Dept Surg, Durham, NC 27710 USA.
[Tatishchev, Sergei; Shibata, Darryl] Univ Southern Calif, Keck Sch Med, Dept Pathol, Los Angeles, CA 90007 USA.
C3 Duke University; Duke University; Duke University; Arizona State University; Arizona State University-Tempe; Arizona State University; Arizona State University-Tempe; Duke University; Duke University; University of Southern California
RP Ryser, MD (autor correspondente), Duke Univ, Med Ctr, Dept Populat Hlth Sci, Durham, NC 27708 USA.; Ryser, MD (autor correspondente), Duke Univ, Dept Math, Durham, NC 27706 USA.; Ryser, MD (autor correspondente), Duke Canc Inst, Durham, NC 27710 USA.; Shibata, D (autor correspondente), Univ Southern Calif, Keck Sch Med, Dept Pathol, Los Angeles, CA 90007 USA.
EM marc.ryser@duke.edu; dshibata@usc.edu
RI Mallo, Diego/H-3055-2019; Ryser, Marc/JBS-7108-2023
OI Mallo, Diego/0000-0002-9046-8859; Sorribes, Inmaculada C./0000-0001-8348-3746; Maley, Carlo/0000-0002-0745-7076
FU Triangle Center for Evolutionary Medicine, National Institutes of Health [R00 CA207872, P30 CA014236, U54 CA217376, U2C CA233254, P01 CA91955, R01 CA170595, R01 CA185138, R01 CA140657]; National Science Foundation [DMS 1614838]; CDMRP Breast Cancer Research Program Award [BC132057]; Arizona Biomedical Research Commission [ADHS18-198847]
FX This work was supported in parts by grants from the Triangle Center for Evolutionary Medicine, National Institutes of Health (R00 CA207872, P30 CA014236, U54 CA217376, U2C CA233254, P01 CA91955, R01 CA170595, R01 CA185138, R01 CA140657), the National Science Foundation (DMS 1614838), CDMRP Breast Cancer Research Program Award (BC132057) and Arizona Biomedical Research Commission (ADHS18-198847).
CR Burrell RA, 2013, NATURE, V501, P338, DOI 10.1038/nature12625
Casasent AK, 2018, CELL, V172, P205, DOI 10.1016/j.cell.2017.12.007
Davis A, 2017, BBA-REV CANCER, V1867, P151, DOI 10.1016/j.bbcan.2017.01.003
Easwaran H, 2014, MOL CELL, V54, P716, DOI 10.1016/j.molcel.2014.05.015
FEARON ER, 1990, CELL, V61, P759, DOI 10.1016/0092-8674(90)90186-I
Gallaher JA, 2018, CANCER RES, V78, P2127, DOI 10.1158/0008-5472.CAN-17-2649
Greaves M, 2012, NATURE, V481, P306, DOI 10.1038/nature10762
Hu Z, 2019, NAT GENET, V51, P1113, DOI 10.1038/s41588-019-0423-x
Humphries A, 2008, NAT REV CANCER, V8, P415, DOI 10.1038/nrc2392
Humphries A, 2013, P NATL ACAD SCI USA, V110, pE2490, DOI 10.1073/pnas.1220353110
Kim TH, 2014, NATURE, V506, P511, DOI 10.1038/nature12903

Kim TM, 2015, CLIN CANCER RES, V21, P4461, DOI 10.1158/1078-0432.CCR-14-2413
Ling SP, 2015, P NATL ACAD SCI USA, V112, pE6496, DOI 10.1073/pnas.1519556112
Maley CC, 2017, NAT REV CANCER, V17, P605, DOI 10.1038/nrc.2017.69
Martincorena I, 2017, CELL, V171, P1029, DOI 10.1016/j.cell.2017.09.042
McDonald TO, 2018, NAT GENET, V50, P1620, DOI 10.1038/s41588-018-0217-6
McGranahan N, 2017, CELL, V168, P613, DOI 10.1016/j.cell.2017.01.018
Meacham CE, 2013, NATURE, V501, P328, DOI 10.1038/nature12624
Nixon KC, 1999, CLADISTICS, V15, P407, DOI 10.1111/j.1096-0031.1999.tb00277.x
Reiter JG, 2018, SCIENCE, V361, P1033, DOI 10.1126/science.aat7171
Ryser MD, 2018, SCI REP-UK, V8, DOI 10.1038/s41598-018-35621-y
Ryser MD, 2018, P NATL ACAD SCI USA, V115, P5774, DOI 10.1073/pnas.1716552115
SHIBATA D, 1992, AM J PATHOL, V141, P539
Sottoriva A, 2015, NAT GENET, V47, P209, DOI 10.1038/ng.3214
Stanta G, 2018, FRONT MED-LAUSANNE, V5, DOI 10.3389/fmed.2018.00085
Sun RP, 2017, NAT GENET, V49, P1015, DOI 10.1038/ng.3891
Turajlic S, 2016, SCIENCE, V352, P169, DOI 10.1126/science.aaf2784
Ulintz PJ, 2018, CLIN CANCER RES, V24, P2214, DOI 10.1158/1078-0432.CCR-17-1425
van der Maaten L, 2008, J MACH LEARN RES, V9, P2579
Welch HG, 2010, J NATL CANCER I, V102, P605, DOI 10.1093/jnci/djq099
Williams MJ, 2018, NAT GENET, V50, P895, DOI 10.1038/s41588-018-0128-6
Williams MJ, 2016, NAT GENET, V48, P238, DOI 10.1038/ng.3489
Zhao ZM, 2016, P NATL ACAD SCI USA, V113, P2140, DOI 10.1073/pnas.1525677113
NR 33
TC 24
Z9 25
U1 0
U2 1
PU NATURE PORTFOLIO
PI BERLIN
PA HEIDELBERGER PLATZ 3, BERLIN, 14197, GERMANY
EI 2041-1723
J9 NAT COMMUN
JI Nat. Commun.
PD MAR 9
PY 2020
VL 11
IS 1
AR 1280
DI 10.1038/s41467-020-14908-7
PG 10
WC Multidisciplinary Sciences
WE Science Citation Index Expanded (SCI-EXPANDED)
SC Science & Technology - Other Topics
GA ML0KL
UT WOS:000549165900005
PM 32152322

OA Green Published, Green Submitted, gold
DA 2025-05-07
ER

PT J
AU Brazeau, NF
   Whitesell, AN
   Doctor, SM
   Keeler, C
   Mwandagalirwa, MK
   Tshefu, AK
   Likwela, JL
   Juliano, JJ
   Meshnick, SR
AF Brazeau, Nicholas F.
   Whitesell, Amy N.
   Doctor, Stephanie M.
   Keeler, Corinna
   Mwandagalirwa, Melchior Kashamuka
   Tshefu, Antoinette K.
   Likwela, Joris L.
   Juliano, Jonathan J.
   Meshnick, Steven R.
TI Plasmodium vivax Infections in Duffy-Negative Individuals in the
   Democratic Republic of the Congo
SO AMERICAN JOURNAL OF TROPICAL MEDICINE AND HYGIENE
LA English
DT Article
ID MALARIA PARASITES; BLOOD; RESISTANCE; EVOLUTION; SURVEILLANCE;
   FALCIPARUM; INVASION; PCR
AB Although Plasmodium vivax has been assumed to be absent from sub-Saharan Africa because of the protective mutation conferring the Duffy-negative phenotype, recent evidence has suggested that P. vivax cases are prevalent in these regions. We selected 292 dried blood spots from children who participated in the 2013-2014 Demographic and Health Survey of the Democratic Republic of the Congo (DRC), to assess for P. vivax infection. Four P. vivax infections were identified by polymerase chain reaction, each in a geographically different survey cluster. Using these as index cases, we tested the remaining 73 samples from the four clusters. With this approach, 10 confirmed cases, three probable cases, and one possible case of P. vivax were identified. Among the 14 P. vivax cases, nine were coinfected with Plasmodium falciparum. All 14 individuals were confirmed to be Duffy-negative by sequencing for the single point mutation in the GATA motif that represses the expression of the Duffy antigen. This finding is consistent with a growing body of literature that suggests that P. vivax can infect Duffy-negative individuals in Africa. Future molecular and sequencing work is needed to understand the relationship of these isolates with other P. vivax samples from Asia and South America and discover variants linked to P. vivax virulence and erythrocyte invasion.
C1 [Brazeau, Nicholas F.; Whitesell, Amy N.; Doctor, Stephanie M.; Juliano, Jonathan J.; Meshnick, Steven R.] Univ N Carolina, Gillings Sch Global Publ Hlth, Dept Epidemiol, Chapel Hill, NC 27515 USA.

[Brazeau, Nicholas F.] Univ N Carolina, Sch Med, Med Scientist Training Program, Chapel Hill, NC 27515 USA.
[Keeler, Corinna] Univ N Carolina, Dept Geog, Chapel Hill, NC 27515 USA.
[Mwandagalirwa, Melchior Kashamuka; Tshefu, Antoinette K.; Likwela, Joris L.] Programme Natl Lutte Paludisme, Kinshasa, DEM REP CONGO.
[Likwela, Joris L.] Kinshasa Sch Publ Hlth, Kinshasa, DEM REP CONGO.
[Juliano, Jonathan J.] Univ N Carolina, Sch Med, Div Infect Dis, Chapel Hill, NC 27515 USA.
[Keeler, Corinna] Univ N Carolina, Gillings Sch Global Publ Hlth, Dept Geog, Chapel Hill, NC 27515 USA.
[Mwandagalirwa, Melchior Kashamuka; Tshefu, Antoinette K.] Univ Kinshasa, Sch Publ Hlth, Kinshasa, DEM REP CONGO.
C3 University of North Carolina; University of North Carolina Chapel Hill;
University of North Carolina; University of North Carolina Chapel Hill;
University of North Carolina School of Medicine; University of North Carolina; University of North Carolina Chapel Hill; University of North
Carolina; University of North Carolina Chapel Hill; University of North
Carolina School of Medicine; University of North Carolina; University of
North Carolina Chapel Hill; Universite de Kinshasa
RP Brazeau, NF (autor correspondente), Univ N Carolina, 135 Dauer Dr, Chapel Hill, NC 27599 USA.
EM nbrazeau@med.unc.edu; awhitese@live.unc.edu; stephanie.doctor@gmail.com;
cykeeler@live.unc.edu; mkashamuka@yahoo.com; antotshe@yahoo.com;
jorislikwela@gmail.com; jonathan_juliano@med.unc.edu;
meshnick@email.unc.edu
OI Keeler, Corinna/0000-0002-3445-6589; LOSIMBA LIKWELA,
Joris/0000-0002-2110-4488; Brazeau, Nicholas/0000-0003-3976-7965
FU National Institutes of Health [R21AI111108, R01TW010870, R01AI107949];
National Science Foundation [BSC-1339949]; Triangle Center for
Evolutionary Medicine (TriCEM); Duke University; University of North
Carolina-Chapel Hill; North Carolina Central University
FX This work was supported by the National Institutes of Health (grant
numbers R21AI111108, R01TW010870, and R01AI107949) and National Science
Foundation (BSC-1339949). Nicholas Ford Brazeau was supported by the
Triangle Center for Evolutionary Medicine (TriCEM), which is supported
by Duke University, University of North Carolina-Chapel Hill, North
Carolina Central University, and North Carolina State Universities.
CR Abdelraheem MH, 2016, T ROY SOC TROP MED H, V110, P258, DOI 10.1093/trstmh/trw014
Abkallo HM, 2014, INT J PARASITOL, V44, P467, DOI 10.1016/j.ijpara.2014.03.002
[Anonymous], ENQ DEM SANT REP DEM
[Anonymous], 2022, AM J TROP MED HYG, DOI DOI 10.4269/AJTMH.1073RET
[Anonymous], DEMOCRATIC REPUBLIC
[Anonymous], DEM HLTH SURV PROGR

Battle KE, 2012, ADV PARASIT, V80, P1, DOI 10.1016/B978-0-12-397900-1.00001-3
Doctor SM, 2016, MALARIA J, V15, DOI 10.1186/s12936-016-1409-0
Doctor SM, 2016, DIAGN MICR INFEC DIS, V85, P16, DOI 10.1016/j.diagmicrobio.2016.01.004
Guerra CA, 2010, PLOS NEGLECT TROP D, V4, DOI 10.1371/journal.pntd.0000774
Gunalan K, 2016, P NATL ACAD SCI USA, V113, P6271, DOI 10.1073/pnas.1606113113
HADLEY TJ, 1986, ANNU REV MICROBIOL, V40, P451, DOI 10.1146/annurev.mi.40.100186.002315
Hester J, 2013, PLOS NEGLECT TROP D, V7, DOI 10.1371/journal.pntd.0002569
Howes RE, 2016, AM J TROP MED HYG, V95, P15, DOI 10.4269/ajtmh.16-0141
Hupalo DN, 2016, NAT GENET, V48, P953, DOI 10.1038/ng.3588
Liu WM, 2014, NAT COMMUN, V5, DOI 10.1038/ncomms4346
LIVINGSTONE FB, 1984, HUM BIOL, V56, P413
Loy DE, 2017, INT J PARASITOL, V47, P87, DOI 10.1016/j.ijpara.2016.05.008
Mbenda HGN, 2014, PLOS ONE, V9, DOI 10.1371/journal.pone.0103262
Menard D, 2010, P NATL ACAD SCI USA, V107, P5967, DOI 10.1073/pnas.0912496107
Mendes C, 2011, PLOS NEGLECT TROP D, V5, DOI 10.1371/journal.pntd.0001192
MILLER LH, 1975, SCIENCE, V189, P561, DOI 10.1126/science.1145213
MILLER LH, 1976, NEW ENGL J MED, V295, P302, DOI 10.1056/NEJM197608052950602
Motshoge T, 2016, BMC INFECT DIS, V16, DOI 10.1186/s12879-016-1857-8
Parobek CM, 2016, P NATL ACAD SCI USA, V113, pE8096, DOI 10.1073/pnas.1608828113
Pearson RD, 2016, NAT GENET, V48, P959, DOI 10.1038/ng.3599
PLOWE CV, 1995, AM J TROP MED HYG, V52, P565, DOI 10.4269/ajtmh.1995.52.565
Poirier P, 2016, MALARIA J, V15, DOI 10.1186/s12936-016-1620-z
Prugnolle F, 2013, P NATL ACAD SCI USA, V110, P8123, DOI 10.1073/pnas.1306004110
R Core Team, 2018, R: a language and environment for statistical com-puting
Russo G, 2017, MALARIA J, V16, DOI 10.1186/s12936-017-1722-2
Singh B, 1999, AM J TROP MED HYG, V60, P687, DOI 10.4269/ajtmh.1999.60.687
TOURNAMILLE C, 1995, NAT GENET, V10, P224, DOI 10.1038/ng0695-224
Veron V, 2009, EXP PARASITOL, V121, P346, DOI 10.1016/j.exppara.2008.12.012
WICKHAM H., 2017, Package tidyverse. Easily install and load the 'Tidyverse
Woldearegai TG, 2013, T ROY SOC TROP MED H, V107, P328, DOI 10.1093/trstmh/trt016
Wurtz N, 2011, MALARIA J, V10, DOI 10.1186/1475-2875-10-336
Zimmerman PA, 2017, AM J TROP MED HYG, V97, P636, DOI 10.4269/ajtmh.17-0461
NR 38
TC 21
Z9 23

U1 0
U2 2
PU AMER SOC TROP MED & HYGIENE
PI MCLEAN
PA 8000 WESTPARK DR, STE 130, MCLEAN, VA 22101 USA
SN 0002-9637
EI 1476-1645
J9 AM J TROP MED HYG
JI Am. J. Trop. Med. Hyg.
PY 2018
VL 99
IS 5
BP 1128
EP 1133
DI 10.4269/ajtmh.18-0277
PG 6
WC Public, Environmental & Occupational Health; Tropical Medicine
WE Science Citation Index Expanded (SCI-EXPANDED)
SC Public, Environmental & Occupational Health; Tropical Medicine
GA HD3DZ
UT WOS:000452395000009
PM 30203741
OA Green Published, Bronze
DA 2025-05-07
ER

PT J
AU Ralser, DJ
   Kiver, V
   Solomayer, EF
   Neeb, C
   Blohmer, JU
   Abramian, AV
   Maass, N
   Schütz, F
   Kolberg-Liedtke, C
   Müller, C
   Rambow, AC
AF Ralser, Damian J.
   Kiver, Verena
   Solomayer, Erich-Franz
   Neeb, Caroline
   Blohmer, Jens-Uwe
   Abramian, Alina V.
   Maass, Nicolai
   Schuetz, Florian
   Kolberg-Liedtke, Cornelia
   Mueller, Carolin
   Rambow, Anna-Christina
TI Impact of low HER2 expression on response to CDK4/6 inhibitor
treatment
   in advanced HR + /HER2-breast cancer: a multicenter real-world data
   analysis
SO ARCHIVES OF GYNECOLOGY AND OBSTETRICS

LA English
DT Article
DE HER2 low; CDK4/6 inhibitor; Breast cancer; Trastuzumab deruxtecan
ID BREAST-CANCER; FULVESTRANT; LAPATINIB; WOMEN
AB PurposeCDK4/6 inhibitors (CDK4/6i) represent the first-line therapy approach of choice for patients with hormone receptor-positive, HER2-negative advanced breast cancer (HR + /HER-ABC). Approximately 50% of HR + /HER2-ABC displays low HER2 expression (HER2 low). Recent data emerging from the DESTINY-Breast04 trial demonstrated practice-changing efficacy of the antibody-drug conjugate trastuzumab deruxtecan (T-DXd) in patients with low HER2 expression. Here, we aimed to analyze the impact of low HER2 expression on CDK4/6i therapy response in a well-characterized multicenter HR + /HER-ABC cohort.MethodsPatients diagnosed with HR + /HER2-ABC who were treated with CDK4/6i in clinical routine between November 2016 and December 2020 at four certified German Breast Cancer Centers were retrospectively identified. The cohort was stratified according to graduation of positivity in HER2 immunohistochemistry (IHC; HER2 zero = IHC score 0 and HER2 low = IHC score 1 + , 2 + /fluorescence in situ hybridization negative). Subgroups were analyzed with regard to progression-free survival (PFS) following CDK4/6i initiation.FindingsThe study cohort comprised n = 448 patients. For n = 311 patients, HER2 status from the metastatic site was available. n = 91 (29.3%) cases were HER2 zero and n = 220 cases (70.7%) were HER2 low. There was no significant difference in PFS between the two groups (PFS: 17 months versus 18 months, log-rank p = 0.42). Further, we examined the influence of HER2 expression changes between primary and metastatic tissue (n = 171; HER2 gain/HER2 loss/HER2 stable expression) on CDK4/6i treatment response. Again, there was no significant difference between these three groups, respectively (PFS: 16 months versus 13 months versus 17 months, log-rank p = 0.86).ConclusionsIn our analysis, HER2 status did not have a significant impact on treatment response to CDK4/6i.
C1 [Ralser, Damian J.; Abramian, Alina V.] Univ Med Ctr Bonn, Univ Hosp Bonn, Dept Gynecol & Gynecol Oncol, Venusberg Campus 1, D-53127 Bonn, Germany.
[Kiver, Verena; Neeb, Caroline; Blohmer, Jens-Uwe] Charite Univ Med Berlin, Dept Gynecol Breast Ctr, Charite pl 1, D-10117 Berlin, Germany.
[Kiver, Verena; Neeb, Caroline; Blohmer, Jens-Uwe] Free Univ Berlin, Charite pl 1, D-10117 Berlin, Germany.
[Kiver, Verena; Neeb, Caroline; Blohmer, Jens-Uwe] Humboldt Univ, Charite pl 1, D-10117 Berlin, Germany.
[Solomayer, Erich-Franz; Mueller, Carolin] Saarland Univ, Med Ctr, Dept Gynecol Obstet & Reprod Med, Homburg, Saar, Germany.
[Maass, Nicolai; Rambow, Anna-Christina] Univ Med Ctr Schleswig Holstein UKSH, Dept Gynecol & Obstet, Campus Kiel, Kiel, Germany.
[Schuetz, Florian] Diakonissen Stiftungs Krankenhaus Speyer, Dept Gynecol & Obstet, Speyer, Germany.
[Kolberg-Liedtke, Cornelia] Univ Med Ctr, Dept Gynecol & Obstet, Essen, Germany.
[Mueller, Carolin] Cleveland Clin, Dept Anesthesiol Outcomes Res Consortium, Cleveland, OH 44195 USA.
C3 University of Bonn; Berlin Institute of Health; Free University of Berlin; Humboldt University of Berlin; Charite Universitatsmedizin Berlin; Free University of Berlin; Humboldt University of Berlin; Universitatsklinikum des Saarlandes; University of Kiel; Schleswig

Holstein University Hospital; University of Duisburg Essen; Cleveland
Clinic Foundation
RP Ralser, DJ (autor correspondente), Univ Med Ctr Bonn, Univ Hosp Bonn,
Dept Gynecol & Gynecol Oncol, Venusberg Campus 1, D-53127 Bonn, Germany.
EM damian.ralser@ukbonn.de
FU Projekt DEAL; BONFOR program of the Medical Faculty of the University of
Bonn [2021-1A-14]; Clinician Scientist Program in Evolutionary Medicine
of the Deutsche Forschungsgemeinschaft (DFG, German Research Foundation)
[413490537]; Deutsche Forschungsgemeinschaft (DFG, German Research
Foundation) [MU 4812/2-1:2]
FX Open Access funding enabled and organized by Projekt DEAL. DJR is
supported by the BONFOR program of the Medical Faculty of the University
of Bonn (grant ID 2021-1A-14). ACR is supported by the Clinician
Scientist Program in Evolutionary Medicine of the Deutsche
Forschungsgemeinschaft (DFG, German Research Foundation, grant ID
413490537). CM is supported by the Deutsche Forschungsgemeinschaft (DFG,
German Research Foundation, grant ID MU 4812/2-1:2).
CR BARDIA A, 2021, CANCER RES, V81
Bergeron A, 2023, BRIT J CANCER, V129, P122, DOI 10.1038/s41416-023-02287-x
Corti C, 2023, CURR TREAT OPTION ON, V24, P468, DOI 10.1007/s11864-023-01068-1
Cristofanilli M, 2016, LANCET ONCOL, V17, P425, DOI 10.1016/S1470-2045(15)00613-0
CURIGLIANO G, 2024, J CLIN ONCOL, V42, DOI DOI 10.1200/JCO.2024.42.17_SUPPL.LBA1000
Denkert C, 2021, LANCET ONCOL, V22, P1151, DOI 10.1016/S1470-2045(21)00301-6
Fehrenbacher L, 2020, J CLIN ONCOL, V38, DOI 10.1200/JCO.19.01455
Finn RS, 2009, BREAST CANCER RES, V11, DOI 10.1186/bcr2419
Johnston SRD, 2005, ENDOCR-RELAT CANCER, V12, pS145, DOI 10.1677/erc.1.00992
Johnston S, 2009, J CLIN ONCOL, V27, P5538, DOI 10.1200/JCO.2009.23.3734
Johnston SRD, 2009, CLIN BREAST CANCER, V9, pS28, DOI 10.3816/CBC.2009.s.003
Klümper N, 2023, CLIN CANCER RES, V29, P1496, DOI 10.1158/1078-0432.CCR-22-1764
Lal P, 2004, AM J CLIN PATHOL, V121, P631, DOI 10.1309/VE7862V2646BR6EX
Leary A, 2015, CLIN CANCER RES, V21, P2932, DOI 10.1158/1078-0432.CCR-14-1428
Leary AF, 2010, CLIN CANCER RES, V16, P1486, DOI 10.1158/1078-0432.CCR-09-1764
Miglietta F, 2021, NPJ BREAST CANCER, V7, DOI 10.1038/s41523-021-00343-4
Modi S, 2022, NEW ENGL J MED, V387, P9, DOI 10.1056/NEJMoa2203690
Müller C, 2023, BREAST CARE, V18, P31, DOI 10.1159/000527917
Sakach E, 2022, CANCERS, V14, DOI 10.3390/cancers14235936

   Slamon DJ, 2021, ANN ONCOL, V32, P1015, DOI
10.1016/j.annonc.2021.05.353
   Sledge GW, 2017, J CLIN ONCOL, V35, P2875, DOI
10.1200/JCO.2017.73.7585
   Spring LM, 2020, LANCET, V395, P817, DOI 10.1016/S0140-6736(20)30165-3
   Stueber TN, 2019, ARCH GYNECOL OBSTET, V299, P533, DOI 10.1007/s00404-018-4954-7
   Zattarin E, 2023, NPJ BREAST CANCER, V9, DOI 10.1038/s41523-023-00534-1
NR 24
TC 3
Z9 3
U1 3
U2 4
PU SPRINGER HEIDELBERG
PI HEIDELBERG
PA TIERGARTENSTRASSE 17, D-69121 HEIDELBERG, GERMANY
SN 0932-0067
EI 1432-0711
J9 ARCH GYNECOL OBSTET
JI Arch. Gynecol. Obstet.
PD FEB
PY 2025
VL 311
IS 2
BP 423
EP 427
DI 10.1007/s00404-024-07761-2
EA OCT 2024
PG 5
WC Obstetrics & Gynecology
WE Science Citation Index Expanded (SCI-EXPANDED)
SC Obstetrics & Gynecology
GA Z6H5K
UT WOS:001327597400001
PM 39373732
OA hybrid
DA 2025-05-07
ER

PT J
AU Merker, M
   Egbe, NF
   Ngangue, YR
   Vuchas, C
   Kohl, TA
   Dreyer, V
   Kuaban, C
   Noeske, J
   Niemann, S
   Sander, MS
AF Merker, Matthias
   Egbe, Nkongho F.
   Ngangue, Yannick R.

Vuchas, Comfort
Kohl, Thomas A.
Dreyer, Viola
Kuaban, Christopher
Noeske, Jurgen
Niemann, Stefan
Sander, Melissa S.
TI Transmission patterns of rifampicin resistant Mycobacterium tuberculosis complex strains in Cameroon: a genomic epidemiological study
SO BMC INFECTIOUS DISEASES
LA English
DT Article
DE MDR-TB; Mycobacterium tuberculosis; Cameroon; Transmission
ID TREATMENT OUTCOMES; DRUG-RESISTANCE; REGIMEN; INFECTIOUSNESS
AB Background Determining factors affecting the transmission of rifampicin (RR) and multidrug-resistant (MDR) Mycobacterium tuberculosis complex strains under standardized tuberculosis (TB) treatment is key to control TB and prevent the evolution of drug resistance. Methods We combined bacterial whole genome sequencing (WGS) and epidemiological investigations for 37% (n = 195) of all RR/MDR-TB patients in Cameroon (2012-2015) to identify factors associated with recent transmission. Results Patients infected with a strain resistant to high-dose isoniazid, and ethambutol had 7.4 (95% CI 2.6-21.4), and 2.4 (95% CI 1.2-4.8) times increased odds of being in a WGS-cluster, a surrogate for recent transmission. Furthermore, age between 30 and 50 was positively correlated with recent transmission (adjusted OR 3.8, 95% CI 1.3-11.4). We found high drug-resistance proportions against three drugs used in the short standardized MDR-TB regimen in Cameroon, i.e. high-dose isoniazid (77.4%), ethambutol (56.9%), and pyrazinamide (43.1%). Virtually all strains were susceptible to fluoroquinolones, kanamycin, and clofazimine, and treatment outcomes were mostly favourable (87.5%). Conclusion Pre-existing resistance to high-dose isoniazid, and ethambutol is associated with recent transmission of RR/MDR strains in our study. A possible contributing factor for this observation is the absence of universal drug susceptibility testing in Cameroon, likely resulting in prolonged exposure of new RR/MDR-TB patients to sub-optimal or failing first-line drug regimens.
C1 [Merker, Matthias; Kohl, Thomas A.; Dreyer, Viola; Niemann, Stefan] Res Ctr Borstel, Mol & Expt Mycobacteriol, Borstel, Germany.
[Merker, Matthias] Res Ctr Borstel, Evolut Resistome, Borstel, Germany.
[Merker, Matthias; Niemann, Stefan] German Ctr Infect Res, Partner Site Hamburg Lubeck Borstel Riems, Borstel, Germany.
[Egbe, Nkongho F.; Ngangue, Yannick R.; Vuchas, Comfort; Sander, Melissa S.] Ctr Hlth Promot & Res, TB Reference Lab Bamenda, Bamenda, Cameroon.
[Egbe, Nkongho F.] Univ Lincoln, Coll Sci, Sch Life Sci, Lincoln, England.
[Kuaban, Christopher] Univ Bamenda, Fac Hlth Sci, Bamenda, Cameroon.
C3 Leibniz Association; Forschungszentrum Borstel; Leibniz Association; Forschungszentrum Borstel; German Center for Infection Research; University of Lincoln

RP Merker, M (autor correspondente), Res Ctr Borstel, Mol & Expt
Mycobacteriol, Borstel, Germany.; Merker, M (autor correspondente), Res
Ctr Borstel, Evolut Resistome, Borstel, Germany.; Merker, M (autor
correspondente), German Ctr Infect Res, Partner Site Hamburg Lubeck
Borstel Riems, Borstel, Germany.
EM mmerker@fz-borstel.de
RI Niemann, Stefan/C-9327-2011; Noeske, Jűrgen/ABI-7430-2020; Kohl,
Thomas/AAB-9782-2020; Merker, Matthias/P-7399-2015; Egbe Franklyn,
Nkongho/L-1262-2018
OI Niemann, Stefan/0000-0002-6604-0684; Merker,
Matthias/0000-0003-1386-2331; Russel Yannick,
Ngangue/0000-0001-8017-9538; Egbe Franklyn, Nkongho/0000-0002-9547-4609
FU German Center for Infection Research (DZIF); Deutsche
Forschungsgemeinschaft (DFG, German Research Foundation) under Germanys
Excellence Strategy [EXC 2167]; Leibniz Science Campus Evolutionary
Medicine of the LUNG (EvoLUNG); Projekt DEAL
FX Open Access funding enabled and organized by Projekt DEAL. The work
performed in Germany was funded by the German Center for Infection
Research (DZIF), the Deutsche Forschungsgemeinschaft (DFG, German
Research Foundation) under Germanys Excellence Strategy-EXC 2167
Precision Medicine in Chronic Inflammation, and the Leibniz Science
Campus Evolutionary Medicine of the LUNG (EvoLUNG). The funders had no
role in study design; in the collection, analysis, and interpretation of
data; in the writing of the report; and in the decision to submit the
paper for publication.
CR Ahmad N, 2018, LANCET, V392, P821, DOI 10.1016/S0140-6736(18)31644-1
Aung KJM, 2014, INT J TUBERC LUNG D, V18, P1180, DOI 10.5588/ijtld.14.0100
Coll F, 2014, NAT COMMUN, V5, DOI 10.1038/ncomms5812
Comas I, 2012, NAT GENET, V44, P106, DOI 10.1038/ng.1038
Cox HS, 2008, NEW ENGL J MED, V359, P2398, DOI 10.1056/NEJMc0805644
Dheda K, 2017, LANCET RESP MED, V5, P291, DOI [10.1016/S2213-2600(17)30079-6, 10.1016/s2213-2600(17)30079-6]
Diel R, 2019, EUR RESPIR J, V54, DOI 10.1183/13993003.01154-2019
Escombe AR, 2008, PLOS MED, V5, P1387, DOI 10.1371/journal.pmed.0050188
Espinal MA, 2000, JAMA-J AM MED ASSOC, V283, P2537, DOI 10.1001/jama.283.19.2537
Fitzwater SP, 2010, CLIN INFECT DIS, V51, P371, DOI 10.1086/655127
Heep M, 2001, J CLIN MICROBIOL, V39, P107, DOI 10.1128/JCM.39.1.107-110.2001
Hoek KGP, 2009, SAMJ S AFR MED J, V99, P785
Jajou R, 2019, EUROSURVEILLANCE, V24, P8, DOI 10.2807/1560-7917.ES.2019.24.50.1900130
Kendall EA, 2015, LANCET RESP MED, V3, P963, DOI 10.1016/S2213-2600(15)00458-0
Khan FA, 2017, EUR RESPIR J, V50, DOI 10.1183/13993003.00061-2017
Kodama C, 2017, EUR RESPIR J, V50, DOI 10.1183/13993003.01044-2017
Kohl TA, 2018, EBIOMEDICINE, V34, P131, DOI 10.1016/j.ebiom.2018.07.030

Kuaban C, 2015, INT J TUBERC LUNG D, V19, P517, DOI 10.5588/ijtld.14.0535
Lange C, 2016, AM J RESP CRIT CARE, V194, P1029, DOI 10.1164/rccm.201606-1097LE
Lew W, 2008, ANN INTERN MED, V149, P123, DOI 10.7326/0003-4819-149-2-200807150-00008
Merker M, 2018, ELIFE, V7, DOI 10.7554/eLife.38200
Moodley R, 2016, EUR RESPIR REV, V25, DOI 10.1183/16000617.0080-2015
Ngabonziza JCS, 2017, PLOS ONE, V12, DOI 10.1371/journal.pone.0187211
Nunn AJ, 2019, NEW ENGL J MED, V380, P1201, DOI 10.1056/NEJMoa1811867
Piubello A, 2014, INT J TUBERC LUNG D, V18, P1188, DOI 10.5588/ijtld.13.0075
Schwaebel V, 2020, ECLINICALMEDICINE, V20, DOI 10.1016/j.eclinm.2020.100268
Shah NS, 2017, NEW ENGL J MED, V376, P243, DOI 10.1056/NEJMoa1604544
Trébucq A, 2018, INT J TUBERC LUNG D, V22, P17, DOI 10.5588/ijtld.17.0498
Trébucq A, 2020, J CLIN MED, V9, DOI 10.3390/jcm9010055
Van Deun A, 2010, AM J RESP CRIT CARE, V182, P684, DOI 10.1164/rccm.201001-0077OC
Walker TM, 2013, LANCET INFECT DIS, V13, P137, DOI 10.1016/S1473-3099(12)70277-3
World Health Organization, Global tuberculosis report 2020
World Health Organization, WHO CONS GUID DRUG R
Yang CG, 2017, LANCET INFECT DIS, V17, P275, DOI [10.1016/s1473-3099(16)30418-2, 10.1016/S1473-3099(16)30418-2]
NR 34
TC 5
Z9 5
U1 0
U2 4
PU BMC
PI LONDON
PA CAMPUS, 4 CRINAN ST, LONDON N1 9XW, ENGLAND
EI 1471-2334
J9 BMC INFECT DIS
JI BMC Infect. Dis.
PD AUG 31
PY 2021
VL 21
IS 1
AR 891
DI 10.1186/s12879-021-06593-8
PG 10
WC Infectious Diseases
WE Science Citation Index Expanded (SCI-EXPANDED)
SC Infectious Diseases
GA UL1HB
UT WOS:000692409100002
PM 34465301
OA gold, Green Published
DA 2025-05-07
ER

PT B
AU Reed, DA
Scapino, RP
Ross, CF
Chen, D
Diekwisch, TGH
AF Reed, David A.
Scapino, Robert P.
Ross, Callum F.
Chen, Di
Diekwisch, Thomas G. H.
BE Greene, CS
Laskin, DM
TI Developmental and Evolutionary Perspectives on TMJ Tissue Engineering
SO TREATMENT OF TMDS: BRIDGING THE GAP BETWEEN ADVANCES IN RESEARCH AND CLINICAL PATIENT MANAGEMENT
LA English
DT Article; Book Chapter
ID LATERAL PTERYGOID MUSCLE; MANDIBULAR CONDYLAR CARTILAGE; HUMAN TEMPOROMANDIBULAR-JOINT; FIBROBLAST-GROWTH-FACTOR; MECKELS CARTILAGE; ARTICULAR-CARTILAGE; STEM-CELLS; EMBRYOLOGIC DEVELOPMENT; ALKALINE-PHOSPHATASE; SECONDARY CARTILAGES
AB Temporomandibular disorders (TMDs) are reported to occur in 5% to 15% of the general population, depending on the study and the type of symptoms assessed.(1) Of these symptomatic patients, 11% are associated with osteoarthritis (OA), which involves degradation of the articular cartilage.(2)

The articular surfaces of the temporomandibular joint (TMJ) are covered by fibrocartilage, a unique cartilage with limited regenerative capacity that is found in only a few other parts of the body: the meniscus of the knee, the intervertebral discs, and the pubic symphysis. This limited regenerative capacity may contribute to articular cartilage failure in OA of the TMJ and also presents challenges for regenerative procedures such as tissue engineering.

TMJ regeneration and tissue engineering have long been major goals of future therapeutic strategies to remedy the severe pain that some patients experience as a result of TMJ-OA. Tissue engineering approaches are commonly divided along three different aspects together called the tissue engineering triad: (1) the biomaterials used for the implanted scaffold, (2) the cells with which the scaffold is seeded, and (3) the intrinsic and extrinsic signaling factors associated with the implant.

Recent advances in scaffold biomaterials for the TMJ include the use of alginate hydrogels,(3,4) agrose hydrogels,(5) poly(ethylene glycol) diacrylate hydrogels,(6) isotropic fibrous materials such as the nonwoven synthetic polymer, polyglycolic acid,(7-9) and microsphere-based scaffold solutions.(10,11) Nanofibrous scaffolds have been implemented in other dental and craniofacial applications with great success, and they also show promise for the TMJ.(12)

A variety of approaches in cell seeding have also been explored in TMJ tissue engineering, including the use of cells harvested from the joint itself(13) and from other articular surfaces such as the ankle,(14) as well as bone marrow mesenchymal stem cells,(15) adipose stem cells,16 and umbilical stem cells.(9)

Common signaling factors for TMJ tissue engineering include transforming growth factor beta-1(11,17)and bone morphogenetic protein 2.(11,18) However, several other candidates have been explored, including fibroblast growth factor-2,(17,19,20) insulinlike growth factor 1,(17),(21-24) and glucosamine,(25) as well as bioactive signaling gradients that incorporate several signaling factors.(11)

While current tissue engineering approaches show promise for tissue regeneration and integration, a high-fidelity solution that restores full physiologic function to the TMJ has yet to be achieved. This chapter takes an evolutionary medicine approach(26) and proposes that the ancestral evolutionary history of the TMJ provides valuable insights into the design of high-fidelity tissue engineering approaches for treatment of TMDs.

C1 [Reed, David A.; Scapino, Robert P.; Diekwisch, Thomas G. H.] Univ Illinois, Coll Dent, Dept Oral Biol, Chicago, IL 60607 USA.
[Ross, Callum F.] Univ Chicago, Dept Organismal Biol, Chicago, IL 60637 USA.
[Chen, Di] Rush Univ, Dept Biochem, Chicago, IL 60612 USA.
C3 University of Illinois System; University of Illinois Chicago; University of Illinois Chicago Hospital; University of Chicago; Rush University
RP Reed, DA (autor correspondente), Univ Illinois, Coll Dent, Dept Oral Biol, Chicago, IL 60607 USA.
RI Chen, Di/AAJ-3665-2021
CR Alhadlaq A, 2003, J DENT RES, V82, P951, DOI 10.1177/154405910308201203
ALLIN EF, 1975, J MORPHOL, V147, P403, DOI 10.1002/jmor.1051470404
Almarza AJ, 2006, ARCH ORAL BIOL, V51, P215, DOI 10.1016/j.archoralbio.2005.07.002
Almarza AJ, 2011, ANN BIOMED ENG, V39, P2479, DOI 10.1007/s10439-011-0364-8
[Anonymous], CHONDROID BONE SECON
[Anonymous], MORPHOLOGY MAXILLO M
[Anonymous], J DENT RES
[Anonymous], ANAT ANZEIGER
[Anonymous], J DENT RES
[Anonymous], FORMATION TEMPOROMAN
APPLETON J, 1975, ARCH ORAL BIOL, V20, P823, DOI 10.1016/0003-9969(75)90060-6
Bailey MM, 2007, TISSUE ENG, V13, P2003, DOI 10.1089/ten.2006.0150
BARGHUSEN HERBERT R., 1968, POSTILLA [YALE PEABODY MUS], V116, P1
BAUME LJ, 1962, J DENT RES, V41, P1327, DOI 10.1177/00220345620410060901
BAUME LJ, 1970, J DENT RES, V49, P864, DOI 10.1177/00220345700490042501
Benjamin M, 2000, INT REV CYTOL, V196, P85
Carda C, 2005, TISSUE CELL, V37, P247, DOI 10.1016/j.tice.2005.03.002
Carlson DS., 1980, Current Advances in Oral Surgery, VIII., P28
CROMPTON AW, 1963, EVOLUTION, V17, P431, DOI 10.1111/j.1558-5646.1963.tb03299.x
De Beer G., 1937, University Proc. Zool. Soc. Lond, V289, P61
DEBONT LGM, 1984, J ORAL MAXIL SURG, V42, P306, DOI 10.1016/0278-2391(84)90110-1

Delatte M, 2004, ARCH ORAL BIOL, V49, P165, DOI 10.1016/j.archoralbio.2003.09.007
Delatte M, 2004, EUR J ORAL SCI, V112, P156, DOI 10.1111/j.0909-8836.2004.00108.x
Delatte ML, 2005, EUR J ORTHODONT, V27, P17, DOI 10.1093/ejo/cjh068
DEMAR R, 1972, EVOLUTION, V26, P622, DOI 10.1111/j.1558-5646.1972.tb01969.x
Domm C, 2002, OSTEOARTHR CARTILAGE, V10, P13, DOI 10.1053/joca.2001.0477
Dormer NH, 2011, J ORAL MAXIL SURG, V69, pE50, DOI 10.1016/j.joms.2010.12.049
DUBRUL EL, 1964, TEMPOROMANDIBULAR JO
Freed LE, 1998, EXP CELL RES, V240, P58, DOI 10.1006/excr.1998.4010
FROMMER J, 1971, J DENT RES, V50, P1260, DOI 10.1177/00220345710500052801
Fuentes MA, 2002, ARCH ORAL BIOL, V47, P643, DOI 10.1016/S0003-9969(02)00052-3
Fujita S, 2001, J ORAL REHABIL, V28, P560, DOI 10.1046/j.1365-2842.2001.00691.x
FURSTMAN L, 1963, AM J ORTHOD DENTOFAC, V49, P672, DOI 10.1016/0002-9416(63)90038-1
GARTNER LP, 1976, ACTA ANAT, V96, P404
Gross JB, 2008, DEV BIOL, V317, P389, DOI 10.1016/j.ydbio.2008.02.046
Gupte MJ, 2012, J DENT RES, V91, P227, DOI 10.1177/0022034511417441
Hall B.K., 1984, Symposia of the Zoological Society of London, P155
HALL BK, 1986, J EMBRYOL EXP MORPH, V93, P133
Hall Brian, 2005, P1
Harpman JA, 1938, J ANAT, V73, P112
Herring S. W., 2003, Journal of Musculoskeletal & Neuronal Interactions, V3, P391
Heylings David J. A., 1995, Journal of Orofacial Pain, V9, P9
Hinton RJ, 2009, ORTHOD CRANIOFAC RES, V12, P168, DOI 10.1111/j.1601-6343.2009.01450.x
Hinton RJ., 2005, SEM ORTHOD, V11, P209, DOI DOI 10.1053/J.S0D0.2005.07.005
HOPSON JA, 1966, AM ZOOL, V6, P437
Ishizeki K, 2001, CELL TISSUE RES, V304, P67, DOI 10.1007/s004410100354
Ji Q, 2009, SCIENCE, V326, P278, DOI 10.1126/science.1178501
Johansson A, 2003, J OROFAC PAIN, V17, P29
KEITH DA, 1982, BRIT J ORAL SURG, V20, P217, DOI 10.1016/S0007-117X(82)80042-5
Kemp T.S., 1982, MAMMAL LIKE REPTILES
Kjelberg K., 1904, Morphologisches Jahrbuch, Vxxxii, P159
Klinge RF, 1996, MICRON, V27, P381, DOI 10.1016/S0968-4328(96)00038-8
Lee SK, 2001, ANAT REC, V263, P314, DOI 10.1002/ar.1110
LEVY BM, 1964, TEMPOROMANDIBULAR JO
LOMBARD RE, 1979, BIOL J LINN SOC, V11, P19, DOI 10.1111/j.1095-8312.1979.tb00027.x
Luo ZX, 2007, NATURE, V450, P1011, DOI 10.1038/nature06277
Mäenpää K, 2010, J R SOC INTERFACE, V7, P177, DOI 10.1098/rsif.2009.0117
MANLEY GA, 1972, EVOLUTION, V26, P608, DOI 10.1111/j.1558-5646.1972.tb01968.x

MAOR G, 1993, ACTA ENDOCRINOL-COP, V128, P56, DOI 10.1530/acta.0.1280056
MAOR G, 1993, J ENDOCRINOL, V137, P21, DOI 10.1677/joe.0.1370021
Martin I, 2000, BIORHEOLOGY, V37, P141
Mauck RL, 2000, J BIOMECH ENG-T ASME, V122, P252, DOI 10.1115/1.429656
MEJERSJO C, 1984, ACTA RADIOL DIAGN, V25, P169, DOI 10.1177/028418518402500303
Melnick M, 2005, CELLS TISSUES ORGANS, V179, P146, DOI 10.1159/000085950
Mérida-Velasco JR, 1999, ANAT RECORD, V255, P20, DOI 10.1002/(SICI)1097-0185(19990501)255:1<20::AID-AR4>3.0.CO;2-N
Miyake T, 1997, J ANAT, V190, P239, DOI 10.1046/j.1469-7580.1997.19020239.x
Mizuno I, 1990, Shika Kiso Igakkai Zasshi, V32, P69
MURRAY P. D. F., 1963, AUSTRALIAN JOUR ZOOL, V11, P368, DOI 10.1071/ZO9630368
Naidoo LCD, 1996, ORAL SURG ORAL MED O, V82, P4
Ogawa T, 2003, J BONE MINER METAB, V21, P145, DOI 10.1007/s007740300023
Ögütcen-Toller M, 2000, J ORAL MAXIL SURG, V58, P1381, DOI 10.1053/joms.2000.18270
OGUTCENTOLLER M, 1994, EARLY HUM DEV, V39, P57, DOI 10.1016/0378-3782(94)90070-1
OGUTCENTOLLER M, 1993, J ORAL MAXIL SURG, V51, P772
PARRINGTON F. R., 1955, PROC ZOOL SOC LONDON, V125, P1
Payne SL, 2011, ANAT REC, V294, P399, DOI 10.1002/ar.21329
Petrovic AG., 1975, Determinants of Mandibular Growth and Form, P101
Ragan PM, 2000, ARCH BIOCHEM BIOPHYS, V383, P256, DOI 10.1006/abbi.2000.2060
Ramaesh T, 2003, J ANAT, V203, P213, DOI 10.1046/j.1469-7580.2003.00210.x
RAYNE J, 1972, J ANAT, V113, P391
SAVOSTIN.I, 1973, ANAT RECORD, V176, P345, DOI 10.1002/ar.1091760310
Scapino RP, 1996, ARCH ORAL BIOL, V41, P1039, DOI 10.1016/S0003-9969(96)00079-9
Serrano MJ, 2011, ARCH ORAL BIOL, V56, P607, DOI 10.1016/j.archoralbio.2010.11.014
Shibata S, 2006, J ANAT, V208, P169, DOI 10.1111/j.1469-7580.2006.00525.x
Shibata S, 1996, ARCH ORAL BIOL, V41, P47, DOI 10.1016/0003-9969(95)00105-0
Shibata S, 1997, J ANAT, V191, P561, DOI 10.1046/j.1469-7580.1997.19140561.x
Shin M, 2004, TISSUE ENG, V10, P33, DOI 10.1089/107632704322791673
Sidor CA, 2003, PALEOBIOLOGY, V29, P605, DOI 10.1666/0094-8373(2003)029<0605:ETATOO>2.0.CO;2
Singh M, 2010, ACTA BIOMATER, V6, P137, DOI 10.1016/j.actbio.2009.07.042
SPRINZ R., 1965, PROC ZOOL SOC LONDON, V144, P327
Stearns SC, 2010, P NATL ACAD SCI USA, V107, P1691, DOI 10.1073/pnas.0914475107
Suzuki T, 2002, BRIT J ORAL MAX SURG, V40, P201, DOI 10.1054/bjom.2001.0720
SYMONS NBB, 1952, J ANAT, V86, P326

   Tajima Y, 1998, ARCH ORAL BIOL, V43, P873, DOI 10.1016/S0003-9969(98)00070-3
   TENGAN T, 1990, Journal of the Stomatological Society Japan, V57, P32
   TREVISAN RA, 1976, ACTA ANAT, V96, P335
   TREVISAN RA, 1976, ACTA ANAT, V94, P40
   VANDERLINDEN EJ, 1987, AM J ORTHOD DENTOFAC, V91, P22, DOI 10.1016/0889-5406(87)90205-8
   Wang L, 2009, OSTEOARTHR CARTILAGE, V17, P346, DOI 10.1016/j.joca.2008.07.004
   Wang LM, 2009, ARCH ORAL BIOL, V54, P1, DOI 10.1016/j.archoralbio.2008.06.002
   WILKINSON TM, 1988, J PROSTHET DENT, V60, P715, DOI 10.1016/0022-3913(88)90406-4
   YUODELIS RA, 1966, J DENT RES, V45, P182, DOI 10.1177/00220345660450011301
NR 101
TC 0
Z9 0
U1 0
U2 13
PU QUINTESSENCE PUBLISHING CO INC
PI HANOVER PARK
PA 4350 CHANDLER DRIVE, HANOVER PARK, IL 60133 USA
BN 978-0-86715-586-0
PY 2013
BP 167
EP 176
PG 10
WC Dentistry, Oral Surgery & Medicine
WE Book Citation Index– Science (BKCI-S)
SC Dentistry, Oral Surgery & Medicine
GA BEX83
UT WOS:000318576700016
DA 2025-05-07
ER

PT J
AU Mahrt, N
   Tietze, A
   Kunzel, S
   Franzenburg, S
   Barbosa, C
   Jansen, G
   Schulenburg, H
AF Mahrt, Niels
   Tietze, Alexandra
   Kunzel, Sven
   Franzenburg, Soren
   Barbosa, Camilo
   Jansen, Gunther
   Schulenburg, Hinrich
TI Bottleneck size and selection level reproducibly impact evolution of
   antibiotic resistance
SO NATURE ECOLOGY & EVOLUTION

LA English
DT Article
ID ESCHERICHIA-COLI; POPULATION BOTTLENECKS; PSEUDOMONAS-AERUGINOSA; CLONAL
INTERFERENCE; RAPID ADAPTATION; MUTATIONS; GENOME; ENZYME; TRANSMISSION;
GENETICS
AB Strong population bottlenecks in combination with weak antibiotic selection consistently favours the evolution of resistance across independently performed Pseudomonas aeruginosa evolution experiments.
During antibiotic treatment, the evolution of bacterial pathogens is fundamentally affected by bottlenecks and varying selection levels imposed by the drugs. Bottlenecks-that is, reductions in bacterial population size-lead to an increased influence of random effects (genetic drift) during bacterial evolution, and varying antibiotic concentrations during treatment may favour distinct resistance variants. Both aspects influence the process of bacterial evolution during antibiotic therapy and thereby treatment outcome. Surprisingly, the joint influence of these interconnected factors on the evolution of antibiotic resistance remains largely unexplored. Here we combine evolution experiments with genomic and genetic analyses to demonstrate that bottleneck size and antibiotic-induced selection reproducibly impact the evolutionary path to resistance in pathogenic Pseudomonas aeruginosa, one of the most problematic opportunistic human pathogens. Resistance is favoured-expectedly-under high antibiotic selection and weak bottlenecks, but-unexpectedly-also under low antibiotic selection and severe bottlenecks. The latter is likely to result from a reduced probability of losing favourable variants through drift under weak selection. Moreover, the absence of high resistance under low selection and weak bottlenecks is caused by the spread of low-resistance variants with high competitive fitness under these conditions. We conclude that bottlenecks, in combination with drug-induced selection, are currently neglected key determinants of pathogen evolution and outcome of antibiotic treatment.
C1 [Mahrt, Niels; Tietze, Alexandra; Barbosa, Camilo; Jansen, Gunther; Schulenburg, Hinrich] Christian Albrechts Univ Kiel, Dept Zool, Evolutionary Ecol & Genet, Kiel, Germany.
[Kunzel, Sven] Max Planck Inst Evolutionary Biol, Dept Evolutionary Genet, Plon, Germany.
[Franzenburg, Soren] Christian Albrechts Univ Kiel, Dept Clin Mol Biol, Genet & Bioinformat, Kiel, Germany.
[Barbosa, Camilo] Univ Michigan, Dept Internal Med, Div Infect Dis, Ann Arbor, MI 48109 USA.
[Schulenburg, Hinrich] Max Planck Inst Evolutionary Biol, Antibiot Resistance Grp, Plon, Germany.
C3 University of Kiel; Max Planck Society; University of Kiel; University of Michigan System; University of Michigan; Max Planck Society
RP Schulenburg, H (autor correspondente), Christian Albrechts Univ Kiel, Dept Zool, Evolutionary Ecol & Genet, Kiel, Germany.; Schulenburg, H (autor correspondente), Max Planck Inst Evolutionary Biol, Antibiot Resistance Grp, Plon, Germany.
EM hschulenburg@zoologie.uni-kiel.de
RI Barbosa, Camilo/AAC-5935-2019; Jansen, Gert/HCI-8852-2022; Barbosa, Camilo/IVV-0365-2023
OI Barbosa, Camilo/0000-0003-0433-9311; Franzenburg,

Soren/0000-0001-6374-4910
FU Schulenburg group; German Science Foundation [JA 2342/2-1, SCHU 1415/12-2]; German Science Foundation (Germany's Excellence Strategy) [EXC 2167-390884018]; Max Planck Society (Max Planck fellowship); Max Planck Society (IMPRS membership); Leibniz Science Campus Evolutionary Medicine of the Lung (EvoLUNG)
FX We thank the Schulenburg group for advice and support. We acknowledge financial support from the German Science Foundation (grants JA 2342/2-1 and SCHU 1415/12-2, and funding under Germany's Excellence Strategy EXC 2167-390884018), the Max Planck Society (Max Planck fellowship to H.S.; IMPRS membership to N.M.), and the Leibniz Science Campus Evolutionary Medicine of the Lung (EvoLUNG). We thank R. Roemhild for feedback on the manuscript.
CR Abel S, 2015, PLOS PATHOG, V11, DOI 10.1371/journal.ppat.1004823
Aminov RI, 2010, FRONT MICROBIOL, V1, DOI 10.3389/fmicb.2010.00134
Andrews S., 2010, FastQC, DOI DOI 10.1186/1472-6963-10-122
Aslam B, 2018, INFECT DRUG RESIST, V11, P1645, DOI 10.2147/IDR.S173867
Barbosa C, 2021, MOL BIOL EVOL, V38, P449, DOI 10.1093/molbev/msaa233
Barrow K, 2009, ANTIMICROB AGENTS CH, V53, P5150, DOI 10.1128/AAC.00893-09
Bentley DR, 2008, NATURE, V456, P53, DOI 10.1038/nature07517
Bolger AM, 2014, BIOINFORMATICS, V30, P2114, DOI 10.1093/bioinformatics/btu170
Bosch F, 2008, PHARMACOLOGY, V82, P171, DOI 10.1159/000149583
Cabot G, 2016, ANTIMICROB AGENTS CH, V60, P1767, DOI 10.1128/AAC.02676-15
Campos PRA, 2010, EVOLUTION, V64, P1973, DOI 10.1111/j.1558-5646.2010.00981.x
Cingolani P, 2012, FLY, V6, P80, DOI 10.4161/fly.19695
Cock PJA, 2010, NUCLEIC ACIDS RES, V38, P1767, DOI 10.1093/nar/gkp1137
Davies J, 2010, MICROBIOL MOL BIOL R, V74, P417, DOI 10.1128/MMBR.00016-10
Desai MM, 2007, CURR BIOL, V17, P385, DOI 10.1016/j.cub.2007.01.072
Elena SF, 2003, NAT REV GENET, V4, P457, DOI 10.1038/nrg1088
Fernández L, 2010, ANTIMICROB AGENTS CH, V54, P3372, DOI 10.1128/AAC.00242-10
Fogle CA, 2008, GENETICS, V180, P2163, DOI 10.1534/genetics.108.090019
Garoff L, 2020, MOL BIOL EVOL, V37, P1637, DOI 10.1093/molbev/msaa032
Garrison E., 2012, arXiv, P1, DOI DOI 10.48550/ARXIV.1207.3907
Geoghegan JL, 2016, P ROY SOC B-BIOL SCI, V283, DOI 10.1098/rspb.2016.0727
Gerrish PJ, 1998, GENETICA, V102-3, P127, DOI 10.1023/A:1017067816551
Groisman EA, 2001, J BACTERIOL, V183, P1835, DOI 10.1128/JB.183.6.1835-1842.2001
Gullberg E, 2011, PLOS PATHOG, V7, DOI 10.1371/journal.ppat.1002158
Hall BG, 2014, MOL BIOL EVOL, V31, P232, DOI 10.1093/molbev/mst187
Hall MD, 2019, ELIFE, V8, DOI 10.7554/eLife.46402
Handel A, 2008, GENETICS, V180, P2193, DOI 10.1534/genetics.108.093013
Hannan TJ, 2012, FEMS MICROBIOL REV, V36, P616, DOI 10.1111/j.1574-6976.2012.00339.x

HILL WG, 1966, GENET RES, V8, P269, DOI 10.1017/S0016672300010156
Koboldt DC, 2012, GENOME RES, V22, P568, DOI 10.1101/gr.129684.111
Laehnemann D, 2014, GENOME BIOL EVOL, V6, P1287, DOI 10.1093/gbe/evu106
Lee CR, 2005, MOL MICROBIOL, V58, P334, DOI 10.1111/j.1365-2958.2005.04834.x
Lee DG, 2006, GENOME BIOL, V7, DOI 10.1186/gb-2006-7-10-r90
Levin BR, 2000, GENETICS, V154, P985
Levin BR, 1999, SCIENCE, V283, P806, DOI 10.1126/science.283.5403.806
Li H, 2009, BIOINFORMATICS, V25, P2078, DOI 10.1093/bioinformatics/btp352
Li H, 2009, BIOINFORMATICS, V25, P1754, DOI [10.1093/bioinformatics/btp698, 10.1093/bioinformatics/btp324, 10.1093/bioinformatics/btp352]
López-Causapé C, 2018, FRONT MICROBIOL, V9, DOI 10.3389/fmicb.2018.00685
López-Causapé C, 2018, ANTIMICROB AGENTS CH, V62, DOI 10.1128/AAC.02583-17
MacLean RC, 2010, NAT REV GENET, V11, P405, DOI 10.1038/nrg2778
Martínez JL, 2012, FRONT MICROBIOL, V3, DOI 10.3389/fmicb.2011.00265
McCallum H, 2001, TRENDS ECOL EVOL, V16, P295, DOI 10.1016/S0169-5347(01)02144-9
Merker M, 2020, FRONT IMMUNOL, V11, DOI 10.3389/fimmu.2020.01938
Moxon R, 2017, EVOLUTION, V71, P2803, DOI 10.1111/evo.13370
O'Neill J., 2016, REPORT COMMISSIONED
Opatowski L, 2010, ANTIMICROB AGENTS CH, V54, P2330, DOI 10.1128/AAC.00331-09
Papkou A, 2019, P NATL ACAD SCI USA, V116, P923, DOI 10.1073/pnas.1810402116
Papkou A, 2016, ZOOLOGY, V119, P330, DOI 10.1016/j.zool.2016.02.001
Rabus R, 1999, J BIOL CHEM, V274, P26185, DOI 10.1074/jbc.274.37.26185
Reizer J, 1996, GENE, V181, P103, DOI 10.1016/S0378-1119(96)00481-7
Robinson JT, 2011, NAT BIOTECHNOL, V29, P24, DOI 10.1038/nbt.1754
Sanz-García F, 2018, FRONT GENET, V9, DOI 10.3389/fgene.2018.00451
Schulenburg JHGV, 2001, MOL BIOL EVOL, V18, P648, DOI 10.1093/oxfordjournals.molbev.a003845
Szendro IG, 2013, P NATL ACAD SCI USA, V110, P571, DOI 10.1073/pnas.1213613110
Vogwill T, 2016, P ROY SOC B-BIOL SCI, V283, DOI 10.1098/rspb.2016.0749
Wahl LM, 2002, GENETICS, V162, P961
Wein T, 2019, GENOME BIOL EVOL, V11, P3283, DOI 10.1093/gbe/evz243
Willi Y, 2006, ANNU REV ECOL EVOL S, V37, P433, DOI 10.1146/annurev.ecolsys.37.091305.110145
World Health Organization, 2017, Global priority list of antibiotic resistant bacteria to guide research, discovery, and development of new antibiotics, DOI DOI 10.1016/S1473-3099(09)70222-1
Xu HJ, 2005, FEMS MICROBIOL LETT, V253, P103, DOI 10.1016/j.femsle.2005.09.027
Ye K, 2009, BIOINFORMATICS, V25, P2865, DOI 10.1093/bioinformatics/btp394
Zaffiri L, 2012, J INVEST SURG, V25, P67, DOI 10.3109/08941939.2012.664099
NR 62

TC 37
Z9 39
U1 3
U2 44
PU NATURE PORTFOLIO
PI BERLIN
PA HEIDELBERGER PLATZ 3, BERLIN, 14197, GERMANY
SN 2397-334X
J9 NAT ECOL EVOL
JI Nat. Ecol. Evol.
PD SEP
PY 2021
VL 5
IS 9
DI 10.1038/s41559-021-01511-2
EA JUL 2021
PG 22
WC Ecology; Evolutionary Biology
WE Science Citation Index Expanded (SCI-EXPANDED)
SC Environmental Sciences & Ecology; Evolutionary Biology
GA UI9NY
UT WOS:000679047500003
PM 34312522
OA Green Published, hybrid
DA 2025-05-07
ER

PT J
AU Frazee, LJ
   Rifkin, J
   Maheepala, DC
   Grant, AG
   Wright, S
   Kalisz, S
   Litt, A
   Spigler, R
AF Frazee, Lauren J.
   Rifkin, Joanna
   Maheepala, Dinusha C.
   Grant, Alannie-Grace
   Wright, Stephen
   Kalisz, Susan
   Litt, Amy
   Spigler, Rachel
TI New genomic resources and comparative analyses reveal differences in
   floral gene expression in selfing and outcrossing Collinsia
   sister species
SO G3-GENES GENOMES GENETICS
LA English
DT Article
DE Collinsia; RNA-seq; selfing syndrome; pollen; floral development;
   differential gene expression; DESeq2; dichogamy; evolutionary
genomics;
   Hi-C scaffolding; parallel evolution

ID MATING-SYSTEM EVOLUTION; CHALCONE SYNTHASE; REPRODUCTIVE ASSURANCE;
POLLEN COMPETITION; RANGE SIZE; SELECTION; MIMULUS; ANTHER;
FERTILIZATION; POLLINATION
AB The evolutionary transition from outcross- to self-fertilization is one of the most common in angiosperms and is often associated with a parallel shift in floral morphological and developmental traits, such as reduced flower size and pollen to ovule ratios, known as the "selfing syndrome." How these convergent phenotypes arise, the extent to which they are shaped by selection, and the nature of their underlying genetic basis are unsettled questions in evolutionary biology. The genus Collinsia (Plantaginaceae) includes seven independent transitions from outcrossing or mixed mating to high selfing rates accompanied by selfing syndrome traits. Accordingly, Collinsia represents an ideal system for investigating this parallelism, but requires genomic resource development. We present a high quality de novo genome assembly for the highly selfing species Collinsia rattanii. To begin addressing the basis of selfing syndrome developmental shifts, we evaluate and contrast patterns of gene expression from floral transcriptomes across three stages of bud development for C. rattanii and its outcrossing sister species Collinsia linearis. Relative to C. Iinearis, total gene expression is less variable among individuals and bud stages in C. rattanii. In addition, there is a common pattern among differentially expressed genes: lower expression levels that are more constant across bud development in C. rattanii relative to C. Iinearis. Transcriptional regulation of enzymes involved in pollen formation specifically in early bud development may influence floral traits that distinguish selfing and outcrossing Collinsia species through pleiotropic functions. Future work will include additional Collinsia outcrossing-selfing species pairs to identify genomic signatures of parallel evolution.
C1 [Frazee, Lauren J.; Spigler, Rachel] Temple Univ, Dept Biol, Philadelphia, PA 19122 USA.
[Rifkin, Joanna; Wright, Stephen] Univ Toronto, Dept Ecol & Evolutionary Biol, Toronto, ON M5S, Canada.
[Maheepala, Dinusha C.; Litt, Amy] Univ Calif Riverside, Dept Bot & Plant Sci, Riverside, CA 92521 USA.
[Grant, Alannie-Grace; Kalisz, Susan] Univ Tennessee, Dept Ecol & Evolutionary Biol, Knoxville, TN 37996 USA.
[Maheepala, Dinusha C.; Spigler, Rachel] Univ Arizona Hlth Sci, Ctr Innovat Brain Sci, Tucson, AZ 85721 USA.
C3 Pennsylvania Commonwealth System of Higher Education (PCSHE); Temple University; University of Toronto; University of California System; University of California Riverside; University of Tennessee System; University of Tennessee Knoxville; University of Arizona Health Sciences; University of Arizona
RP Spigler, R (autor correspondente), Temple Univ, Dept Biol, Philadelphia, PA 19122 USA.; Spigler, R (autor correspondente), 1900 N 12th St,Biolife 443A, Philadelphia, PA 19122 USA.
EM rachel.spigler@temple.edu
RI Spigler, Rachel/HLH-3924-2023; Wright, Stephen/C-3113-2008
OI Frazee, Lauren J./0000-0001-5248-8763; Rifkin, Joanna/0000-0003-1980-5557; Wright, Stephen/0000-0001-9973-9697; Maheepala, Dinusha/0000-0002-4287-8677; Litt, Amy/0000-0001-6709-8696
FU Institute for Genomics and Evolutionary Medicine (iGEM) at Temple University; NSERC Discovery grant; Canada Research Chair award;

University of Tennessee, Knoxville; Temple University; University of California, Riverside
FX This research was supported by an the Institute for Genomics and Evolutionary Medicine (iGEM) at Temple University (to R.B.S.); NSERC Discovery grant and a Canada Research Chair award (to S.I.W.); University of Tennessee, Knoxville (to S.K.); Temple University (to R.B.S.);; and University of California, Riverside (to A.L.).
CR Alexa A, 2006, BIOINFORMATICS, V22, P1600, DOI 10.1093/bioinformatics/btl140
Armbruster WS, 2002, AM J BOT, V89, P37, DOI 10.3732/ajb.89.1.37
Baldwin BG, 2011, AM J BOT, V98, P731, DOI 10.3732/ajb.1000346
Barrett SCH, 2002, NAT REV GENET, V3, P274, DOI 10.1038/nrg776
BENJAMINI Y, 1995, J R STAT SOC B, V57, P289, DOI 10.1111/j.2517-6161.1995.tb02031.x
Bickhart DM, 2017, NAT GENET, V49, P643, DOI 10.1038/ng.3802
Blount ZD, 2018, SCIENCE, V362, DOI 10.1126/science.aam5979
Carroll SB, 2008, CELL, V134, P25, DOI 10.1016/j.cell.2008.06.030
Cook RD, 1982, Residuals and influence in regression
CRUDEN RW, 1977, EVOLUTION, V31, P32, DOI [10.2307/2407542, 10.1111/j.1558-5646.1977.tb00979.x]
Dare AP, 2013, PLANT J, V74, P398, DOI 10.1111/tpj.12140
Dobin Alexander, 2015, Curr Protoc Bioinformatics, V51, DOI 10.1002/0471250953.bi1114s51
Dobritsa AA, 2010, PLANT PHYSIOL, V153, P937, DOI 10.1104/pp.110.157446
Durand E, 2020, EVOL APPL, V13, P1279, DOI 10.1111/eva.12933
Durand NC, 2016, CELL SYST, V3, P99, DOI 10.1016/j.cels.2015.07.012
DURBIN ML, 1995, P NATL ACAD SCI USA, V92, P3338, DOI 10.1073/pnas.92.8.3338
Eckert CG, 2009, ECOLOGY, V90, P1540, DOI 10.1890/08-1063.1
Elle E, 2003, AM J BOT, V90, P888, DOI 10.3732/ajb.90.6.888
Fishman L, 2008, NEW PHYTOL, V177, P802, DOI 10.1111/j.1469-8137.2007.02265.x
Fujikura U, 2018, DEV CELL, V44, P192, DOI 10.1016/j.devcel.2017.11.022
GARBER E. D., 1958, BOT GAZ, V120, P55, DOI 10.1086/336000
GARBER E. D., 1956, BOT GAZ, V118, P71, DOI 10.1086/335928
Goodwillie C, 2010, NEW PHYTOL, V185, P311, DOI 10.1111/j.1469-8137.2009.03043.x
Grant AG, 2020, EVOLUTION, V74, P73, DOI 10.1111/evo.13870
Grossenbacher D, 2015, ECOL LETT, V18, P706, DOI 10.1111/ele.12449
Hamrick JL, 1996, PHILOS T ROY SOC B, V351, P1291, DOI 10.1098/rstb.1996.0112
Harada T, 2011, J EXP BOT, V62, P815, DOI 10.1093/jxb/erq319
Hazzouri KM, 2013, EVOLUTION, V67, P1263, DOI 10.1111/evo.12027
Hoff KJ, 2019, METHODS MOL BIOL, V1962, P65, DOI 10.1007/978-1-4939-9173-0_5
Jorgensen R, 2013, ANN BOT-LONDON, V112, P821, DOI 10.1093/aob/mct146
Kalisz S, 2004, NATURE, V430, P884, DOI 10.1038/nature02776
Kalisz S, 2003, ECOLOGY, V84, P2928, DOI 10.1890/02-0519
Kalisz S, 1999, AM J BOT, V86, P1239, DOI 10.2307/2656771
Kalisz S, 2012, ANN BOT-LONDON, V109, P571, DOI 10.1093/aob/mcr237
Kassambara A., 2020, FACTOEXTRA EXTRACT V

Kurasawa K, 2009, PLANT CELL PHYSIOL, V50, P413, DOI 10.1093/pcp/pcp003
Lankinen Å, 2019, INT J PLANT SCI, V180, P996, DOI 10.1086/705583
Lankinen A, 2016, AM J BOT, V103, P541, DOI 10.3732/ajb.1500148
Lankinen Å, 2011, AM J BOT, V98, P1191, DOI 10.3732/ajb.1000510
Le S, 2008, J STAT SOFTW, V25, P1, DOI 10.18637/jss.v025.i01
Liao Y, 2014, BIOINFORMATICS, V30, P923, DOI 10.1093/bioinformatics/btt656
Lieberman-Aiden E, 2009, SCIENCE, V326, P289, DOI 10.1126/science.1181369
LLOYD DG, 1979, AM NAT, V113, P67, DOI 10.1086/283365
Love MI, 2014, GENOME BIOL, V15, DOI 10.1186/s13059-014-0550-8
Madjidian JA, 2020, J EVOLUTION BIOL, V33, P1452, DOI 10.1111/jeb.13684
Malagon DA, 2019, AM J BOT, V106, P1271, DOI 10.1002/ajb2.1346
Martin NH, 2007, EVOLUTION, V61, P68, DOI 10.1111/j.1558-5646.2007.00006.x
Mazer SJ, 2020, J ECOL, V108, P1523, DOI 10.1111/1365-2745.13338
MO YY, 1992, P NATL ACAD SCI USA, V89, P7213, DOI 10.1073/pnas.89.15.7213
Moyle LC, 2012, EVOL BIOL, V39, P207, DOI 10.1007/s11692-012-9180-9
Napoli CA, 1999, PLANT PHYSIOL, V120, P615, DOI 10.1104/pp.120.2.615
Pettengill JB, 2012, EVOLUTION, V66, P1210, DOI 10.1111/j.1558-5646.2011.01521.x
R Core Team, 2020, R: a language and environment for statistical computing. R Foundation for Statistical Computing v.4.3.0
Rahnenfuhrer J., 2019, Gene set enrichment analysis with topGO
Randle AM, 2018, EVOLUTION, V72, P1587, DOI 10.1111/evo.13522
Randle AM, 2009, NEW PHYTOL, V183, P618, DOI 10.1111/j.1469-8137.2009.02946.x
Rifkin JL, 2019, ECOL EVOL, V9, P7712, DOI 10.1002/ece3.5329
Salcedo A, 2014, J EVOLUTION BIOL, V27, P1400, DOI 10.1111/jeb.12384
Shimizu KK, 2015, ANNU REV ECOL EVOL S, V46, P593, DOI 10.1146/annurev-ecolsys-112414-054249
Sicard A, 2011, ANN BOT-LONDON, V107, P1433, DOI 10.1093/aob/mcr023
Simao FA, 2015, BIOINFORMATICS, V31, P3210, DOI 10.1093/bioinformatics/btv351
Spigler RB, 2013, BOTANY, V91, P597, DOI 10.1139/cjb-2012-0227
Stebbins G. L., 1974, Flowering plants. Evolution above the species level.
Stern DL, 2008, EVOLUTION, V62, P2155, DOI 10.1111/j.1558-5646.2008.00450.x
Stirling C, 2010, BMC HEALTH SERV RES, V10, DOI 10.1186/1472-6963-10-122
Sun XX, 2016, BMC BIOINFORMATICS, V17, DOI 10.1186/s12859-016-1180-9
Supek F, 2011, PLOS ONE, V6, DOI 10.1371/journal.pone.0021800
Toräng P, 2017, EVOLUTION, V71, P2206, DOI 10.1111/evo.13308
Tsuchimatsu T, 2020, NAT COMMUN, V11, DOI 10.1038/s41467-020-16679-7
Vekemans X, 2014, J EVOLUTION BIOL, V27, P1372, DOI 10.1111/jeb.12372
Wozniak NJ, 2020, PLANT CELL, V32, P935, DOI 10.1105/tpc.19.00551
Wright JW, 2006, EVOL ECOL RES, V8, P1
Wu CA, 2008, HEREDITY, V100, P220, DOI 10.1038/sj.hdy.6801018
Yeh JI, 2008, P NATL ACAD SCI USA, V105, P3280, DOI 10.1073/pnas.0712331105

Zhang DB, 2016, SUBCELL BIOCHEM, V86, P315, DOI 10.1007/978-3-319-25979-6_13
NR 75
TC 7
Z9 7
U1 0
U2 1
PU OXFORD UNIV PRESS INC
PI CARY
PA JOURNALS DEPT, 2001 EVANS RD, CARY, NC 27513 USA
SN 2160-1836
J9 G3-GENES GENOM GENET
JI G3-Genes Genomes Genet.
PD AUG
PY 2021
VL 11
IS 8
AR jkab177
DI 10.1093/g3journal/jkab177
EA MAY 2021
PG 15
WC Genetics & Heredity
WE Science Citation Index Expanded (SCI-EXPANDED)
SC Genetics & Heredity
GA XW8KT
UT WOS:000735861700025
PM 34014319
OA gold, Green Published
DA 2025-05-07
ER

PT J
AU Meredith, HR
Kularatna, S
Nagaro, K
Nagahawatte, A
Bodinayake, C
Kurukulasooriya, R
Wijesingha, N
Harden, LB
Piyasiri, B
Hammouda, A
Wiegmann, BM
Nicholson, BP
Joyce, M
Woods, CW
Van Vliet, AHM
Thakur, S
Tillekeratne, LG
AF Meredith, Hannah R.
Kularatna, Sarath
Nagaro, Kristin
Nagahawatte, Ajith
Bodinayake, Champica

Kurukulasooriya, Ruvini
Wijesingha, Nishadhi
Harden, Lyndy B.
Piyasiri, Bhagya
Hammouda, Amr
Wiegmann, Brian M.
Nicholson, Bradly P.
Joyce, Maria
Woods, Christopher W.
Van Vliet, Arnoud H. M.
Thakur, Siddhartha
Tillekeratne, L. Gayani
TI Colonization with multidrug-resistant Enterobacteriaceae among infants: an observational study in southern Sri Lanka
SO ANTIMICROBIAL RESISTANCE AND INFECTION CONTROL
LA English
DT Article
DE ESBL; CRE; Multidrug-resistant Enterobacteriaceae; Sri Lanka; Intestinal colonization
ID LACTAMASE-PRODUCING ENTEROBACTERIACEAE; SPECTRUM BETA-LACTAMASES; ESCHERICHIA-COLI; FECAL CARRIAGE; PREVALENCE; COMMUNITY; BACTERIA
AB Background The timing of and risk factors for intestinal colonization with multidrug-resistant Enterobacteriaceae (MDRE) are still poorly understood in areas with high MDRE carriage. We determined the prevalence, timing, and risk factors associated with MDRE intestinal colonization among infants in southern Sri Lanka. Methods Women and their newborn children were enrolled within 48 h after delivery in southern Sri Lanka. Rectal swabs were collected from women and infants at enrollment and 4-6 weeks later. Enterobacteriaceae were isolated and identified as MDRE (positive for extended-spectrum beta-lactamases or carbapenem resistant) using standard microbiologic procedures. We used exact methods (Fisher's exact and Kruskal-Wallis tests) and multivariable logistic regression to identify sociodemographic and clinical features associated with MDRE intestinal colonization. Whole-genome sequencing was performed on selected MDRE isolates to identify phylogroups and antibiotic resistance-encoding genes were identified with NCBI's AMRfinder tool. Results Overall, 199 post-partum women and 199 infants were enrolled; 148/199 (74.4%) women and 151/199 (75.9%) infants were reassessed later in the community. Twenty-four/199 (12.1%) women and 3/199 (1.5%) infants displayed intestinal colonization with MDRE at enrollment, while 26/148 (17.6%) women and 24/151 (15.9%) infants displayed intestinal colonization with MDRE at the reassessment. While there were no risk factors associated with infant colonization at enrollment, multivariable analysis indicated that risk factors for infant colonization at reassessment included mother colonized at enrollment (aOR = 3.62) or reassessment (aOR = 4.44), delivery by Cesarean section (aOR = 2.91), and low birth weight (aOR = 5.39). Of the 20 MDRE isolates from infants that were sequenced, multilocus sequence typing revealed that 6/20 (30%) were clustered on the same branch as MDRE isolates found in the respective mothers. All sequenced isolates for mothers (47) and infants (20) had at least one ESBL-producing gene. Genes encoding fosfomycin resistance were found in 33/47 (70%) of mothers' isolates and 16/20 (80%) of infants' isolates and genes encoding resistance to colistin were found in one (2%)

mother's isolate. Conclusions Our results suggest that a substantial proportion of infants undergo MDRE intestinal colonization within 6 weeks of birth, potentially due to postnatal rather than intranatal transmission.
C1 [Meredith, Hannah R.] Duke Univ, Dept Biomed Engn, Durham, NC 27706 USA.
[Meredith, Hannah R.] Johns Hopkins Bloomberg Sch Publ Hlth, Dept Epidemiol, Baltimore, MD 21205 USA.
[Kularatna, Sarath] Univ Ruhuna, Fac Med, Dept Obstet & Gynecol, Galle, Sri Lanka.
[Nagaro, Kristin; Joyce, Maria; Woods, Christopher W.; Tillekeratne, L. Gayani] Duke Univ, Sch Med, Dept Med, Div Infect Dis, Durham, NC 27706 USA.
[Nagahawatte, Ajith; Kurukulasooriya, Ruvini; Wijesingha, Nishadhi] Univ Ruhuna, Fac Med, Dept Microbiol, Galle, Sri Lanka.
[Nagahawatte, Ajith; Bodinayake, Champica; Woods, Christopher W.; Tillekeratne, L. Gayani] Duke Univ, Duke Global Hlth Inst, Durham, NC 27708 USA.
[Bodinayake, Champica; Tillekeratne, L. Gayani] Univ Ruhuna, Fac Med, Dept Med, Galle, Sri Lanka.
[Piyasiri, Bhagya] Teaching Hosp Karapitiya, Galle, Sri Lanka.
[Wiegmann, Brian M.] NC State Univ, Dept Entomol & Plant Pathol, Raleigh, NC USA.
[Nicholson, Bradly P.] Inst Med Res, Durham, NC USA.
[Hammouda, Amr; Joyce, Maria; Woods, Christopher W.; Tillekeratne, L. Gayani] Durham Vet Affairs Hlth Syst, Durham, NC 27705 USA.
[Van Vliet, Arnoud H. M.] Univ Surrey, Sch Vet Med, Dept Pathol & Infect Dis, Guildford, Surrey, England.
[Harden, Lyndy B.; Thakur, Siddhartha] NC State Univ, Dept Populat Hlth & Pathobiol, Raleigh, NC USA.
[Thakur, Siddhartha] NC State Univ, Comparat Med Inst, Raleigh, NC USA.
C3 Duke University; Johns Hopkins University; Johns Hopkins Bloomberg School of Public Health; University Ruhuna; Duke University; University Ruhuna; Duke University; University Ruhuna; North Carolina State University; University of Surrey; North Carolina State University; North Carolina State University
RP Meredith, HR (autor correspondente), Duke Univ, Dept Biomed Engn, Durham, NC 27706 USA.; Meredith, HR (autor correspondente), Johns Hopkins Bloomberg Sch Publ Hlth, Dept Epidemiol, Baltimore, MD 21205 USA.; Tillekeratne, LG (autor correspondente), Duke Univ, Sch Med, Dept Med, Div Infect Dis, Durham, NC 27706 USA.; Tillekeratne, LG (autor correspondente), Duke Univ, Duke Global Hlth Inst, Durham, NC 27708 USA.; Tillekeratne, LG (autor correspondente), Univ Ruhuna, Fac Med, Dept Med, Galle, Sri Lanka.; Tillekeratne, LG (autor correspondente), Durham Vet Affairs Hlth Syst, Durham, NC 27705 USA.
EM hmeredi4@jhu.edu; gayani.tillekeratne@duke.edu
RI Vliet, Arnoud/B-5155-2013
OI Wiegmann, Brian/0000-0002-1869-9759; van Vliet, Arnoud/0000-0003-0203-1305; Meredith, Hannah/0000-0002-5315-7568; Thakur, Siddhartha/0000-0003-3787-9939
FU Triangle Center for Evolutionary Medicine (TriCEM); Duke Global Health

Institute; National Institute of Allergy and Infectious Diseases
[K23AI125677]; FDA GenomeTrakr program [1U18FD00678801]; National
Institute of Allergy and Infectious Diseases [K23AI125677] Funding
Source: NIH RePORTER
FX This study was supported by funds from the Triangle Center for
Evolutionary Medicine (TriCEM). HRM received a travel grant from the
Duke Global Health Institute. LGT is supported by the National
Institute
of Allergy and Infectious Diseases (K23AI125677). Site support is
provided by the Duke Global Health Institute and the Duke Hubert-
Yeargan
Center for Global Health. The whole genome sequencing was completed by
the FDA GenomeTrakr program funded grant 1U18FD00678801.
CR [Anonymous], 2013, Antibiotic Resistance Threats in the United States
[Anonymous], 2023, Performance Standards for Antimicrobial
Susceptivility Testing
Babu R, 2016, J GLOB ANTIMICROB RE, V6, P150, DOI
10.1016/j.jgar.2016.05.007
Bankevich A, 2012, J COMPUT BIOL, V19, P455, DOI 10.1089/cmb.2012.0021
Beghain J, 2018, MICROB GENOMICS, V4, DOI 10.1099/mgen.0.000192
Chereau F, 2015, ANTIMICROB AGENTS CH, V59, P3652, DOI
10.1128/AAC.00029-15
Denkel LA, 2014, J ANTIMICROB CHEMOTH, V69, P2230, DOI
10.1093/jac/dku097
Dominguez-Bello MG, 2010, P NATL ACAD SCI USA, V107, P11971, DOI
10.1073/pnas.1002601107
Falagas ME, 2011, FUTURE MICROBIOL, V6, P653, DOI [10.2217/fmb.11.49,
10.2217/FMB.11.49]
Feldgarden M, 2019, ANTIMICROB AGENTS CH, V63, DOI 10.1128/AAC.00483-
19
Gurevich A, 2013, BIOINFORMATICS, V29, P1072, DOI
10.1093/bioinformatics/btt086
Harris AD, 2004, INFECT CONT HOSP EP, V25, P105, DOI 10.1086/502358
Hawkey PM, 2008, CLIN MICROBIOL INFEC, V14, P159, DOI 10.1111/j.1469-
0691.2007.01855.x
Houghteling PD, 2015, J PEDIATR GASTR NUTR, V60, P294, DOI
10.1097/MPG.0000000000000597
Hsu LY, 2017, CLIN MICROBIOL REV, V30, P1, DOI [10.1128/CMR.00042-16,
10.1128/cmr.00042-16]
Rodríguez-Revuelta MJ, 2018, INT J ANTIMICROB AG, V52, P835, DOI
10.1016/j.ijantimicag.2018.09.007
Joensen KG, 2015, J CLIN MICROBIOL, V53, P2410, DOI 10.1128/JCM.00008-
15
Kothari C, 2013, BMC MICROBIOL, V13, DOI 10.1186/1471-2180-13-136
Lautenbach E, 2001, CLIN INFECT DIS, V33, P1288, DOI 10.1086/322667
Laxminarayan R, 2013, LANCET INFECT DIS, V13, P1057, DOI
10.1016/S1473-3099(13)70318-9
McConville TH, 2017, PLOS ONE, V12, DOI 10.1371/journal.pone.0186195
Meyer E, 2012, INFECTION, V40, P685, DOI 10.1007/s15010-012-0324-8
Murk JLAN, 2009, J CLIN MICROBIOL, V47, P1885, DOI 10.1128/JCM.01406-
08
Nelson Edwin, 2014, BMC Res Notes, V7, P279, DOI 10.1186/1756-0500-7-
279

Nicolas-Chanoine MH, 2008, J ANTIMICROB CHEMOTH, V61, P273, DOI 10.1093/jac/dkm464
Nicolas-Chanoine MH, 2014, CLIN MICROBIOL REV, V27, P543, DOI 10.1128/CMR.00125-13
Pathak A, 2013, BMC INFECT DIS, V13, DOI 10.1186/1471-2334-13-199
Perez PF, 2007, PEDIATRICS, V119, pE724, DOI 10.1542/peds.2006-1649
Pornsukarom S, 2018, BMC GENOMICS, V19, DOI 10.1186/s12864-018-5137-4
Sakeena MHF, 2018, BMC INFECT DIS, V18, DOI 10.1186/s12879-018-3107-8
Tillekeratne LG, 2017, BMC FAM PRACT, V18, DOI 10.1186/s12875-017-0619-z
Tillekeratne LG, 2015, AM J TROP MED HYG, V93, P1031, DOI 10.4269/ajtmh.15-0269
Treangen TJ, 2014, GENOME BIOL, V15, DOI 10.1186/s13059-014-0524-x
Valenza G, 2014, ANTIMICROB AGENTS CH, V58, P1228, DOI 10.1128/AAC.01993-13
Valverde A, 2004, J CLIN MICROBIOL, V42, P4769, DOI 10.1128/JCM.42.10.4769-4775.2004
Wang SP, 2020, TRENDS MICROBIOL, V28, P28, DOI 10.1016/j.tim.2019.07.010
Wirth T, 2006, MOL MICROBIOL, V60, P1136, DOI 10.1111/j.1365-2958.2006.05172.x
Woerther PL, 2013, CLIN MICROBIOL REV, V26, P744, DOI 10.1128/CMR.00023-13
Worby CJ, 2014, PLOS COMPUT BIOL, V10, DOI 10.1371/journal.pcbi.1003549
Zankari E, 2012, J ANTIMICROB CHEMOTH, V67, P2640, DOI 10.1093/jac/dks261
NR 40
TC 8
Z9 8
U1 1
U2 1
PU BMC
PI LONDON
PA CAMPUS, 4 CRINAN ST, LONDON N1 9XW, ENGLAND
SN 2047-2994
J9 ANTIMICROB RESIST IN
JI Antimicrob. Resist. Infect. Control
PD APR 30
PY 2021
VL 10
IS 1
AR 72
DI 10.1186/s13756-021-00938-3
PG 12
WC Public, Environmental & Occupational Health; Infectious Diseases; Microbiology; Pharmacology & Pharmacy
WE Science Citation Index Expanded (SCI-EXPANDED)
SC Public, Environmental & Occupational Health; Infectious Diseases; Microbiology; Pharmacology & Pharmacy
GA RV5XK
UT WOS:000645905200001
PM 33931120
OA Green Published, gold

DA 2025-05-07
ER

PT J
AU Dreyer, V
   Mandal, A
   Dev, P
   Merker, M
   Barilar, I
   Utpatel, C
   Nilgiriwala, K
   Rodrigues, C
   Crook, DW
   Rasigade, JP
   Wirth, T
   Mistry, N
   Niemann, S
AF Dreyer, Viola
   Mandal, Ayan
   Dev, Prachi
   Merker, Matthias
   Barilar, Ivan
   Utpatel, Christian
   Nilgiriwala, Kayzad
   Rodrigues, Camilla
   Crook, Derrick W.
   Rasigade, Jean-Philippe
   Wirth, Thierry
   Mistry, Nerges
   Niemann, Stefan
CA CRyPTIC Consortium
TI High fluoroquinolone resistance proportions among multidrug-resistant
   tuberculosis driven by dominant L2 Mycobacterium tuberculosis
   clones in the Mumbai Metropolitan Region
SO GENOME MEDICINE
LA English
DT Article
DE Tuberculosis; Resistant TB; Multidrug-resistant TB; Fluoroquinolone
   resistance; India; Pre-XDR/XDR-TB; Pre-XDR/XDR-TB transmission;
   Transmission success
ID BEIJING GENOTYPE; DRUG-RESISTANCE; CROSS-RESISTANCE; UP-REGULATION;
   BEDAQUILINE; SPREAD; INDIA; EPIDEMIOLOGY; TRANSMISSION; EMERGENCE
AB Background: Multidrug-resistant (MDR) Mycobacterium tuberculosis complex (MTBC) strains are a serious health problem in India, also contributing to one-fourth of the global MDR tuberculosis (TB) burden. About 36% of the MDR MTBC strains are reported fluoroquinolone (FQ) resistant leading to high pre-extensively drug-resistant (pre-XDR) and XDR-TB (further resistance against bedaquiline and/or linezolid) rates. Still, factors driving the MDR/pre-XDR epidemic in India are not well defined.

   Methods: In a retrospective study, we analyzed 1852 consecutive MTBC strains obtained from patients from a tertiary care hospital laboratory in Mumbai by whole genome sequencing (WGS). Univariate and multivariate statistics was used to investigate factors associated with pre-XDR. Core

genome multi locus sequence typing, time scaled haplotypic density (THD) method and homoplasy analysis were used to analyze epidemiological success, and positive selection in different strain groups, respectively.
Results: In total, 1016 MTBC strains were MDR, out of which 703 (69.2%) were pre-XDR and 45 (4.4%) were XDR. Cluster rates were high among MDR (57.8%) and pre-XDR/XDR (79%) strains with three dominant L2 (Beijing) strain clusters (CI 1-3) representing half of the pre-XDR and 40% of the XDR-TB cases. L2 strains were associated with pre-XDR/ XDR-TB (P < 0.001) and, particularly CI 1-3 strains, had high first-line and FQ resistance rates (81.6-90.6%). Epidemic success analysis using THD showed that L2 strains outperformed L1, L3, and L4 strains in short- and long-term time scales. More importantly, L2 MDR and MDR+ strains had higher THD success indices than their not-MDR counterparts. Overall, compensatory mutation rates were highest in L2 strains and positive selection was detected in genes of L2 strains associated with drug tolerance (prpB and ppsA) and virulence (Rv2828c). Compensatory mutations in L2 strains were associated with a threefold increase of THD indices, suggesting improved transmissibility.
Conclusions: Our data indicate a drastic increase of FQ resistance, as well as emerging bedaquiline resistance which endangers the success of newly endorsed MDR-TB treatment regimens. Rapid changes in treatment and control strategies are required to contain transmission of highly successful pre-XDR L2 strains in the Mumbai Metropolitan region but presumably also India-wide.
C1 [Dreyer, Viola; Merker, Matthias; Barilar, Ivan; Utpatel, Christian; Niemann, Stefan] Res Ctr Borstel, Mol & Expt Mycobacteriol, Borstel, Germany.
[Dreyer, Viola; Niemann, Stefan] German Ctr Infect Res DZIF, Partner Site Hamburg Lubeck Borstel Riems, Borstel, Germany.
[Mandal, Ayan; Dev, Prachi; Nilgiriwala, Kayzad; Mistry, Nerges] Fdn Med Res, Mumbai, Maharashtra, India.
[Merker, Matthias] Res Ctr Borstel, Evolut Resistome, Borstel, Germany.
[Merker, Matthias; Crook, Derrick W.; Niemann, Stefan; CRyPTIC Consortium] CRyPTIC Consortium, Oxford, England.
[Rodrigues, Camilla] PD Hinduja Natl Hosp & Med Res Ctr, Dept Microbiol, Mumbai, Maharashtra, India.
[Crook, Derrick W.] Univ Oxford, Nuffield Dept Med, Oxford, England.
[Rasigade, Jean-Philippe] Univ Lyon, ENS Lyon, Ctr Int Rech Infectiol, INSERM U1111,CNRS,UMR5308, Lyon, France.
[Rasigade, Jean-Philippe] Hosp Civils Lyon, Inst Agents Infect, Lyon, France.
[Wirth, Thierry] Sorbonne Univ, Ecole Prat Hautes Etud, Univ Antilles,Museum Natl Hist Nat,Univ Pierre &, Inst Systemat Evolut Biodiversite,UMR CNRS 7205, Paris, France.
[Wirth, Thierry] PSL Univ, EPHE, Paris, France.
C3 Leibniz Association; Forschungszentrum Borstel; German Center for Infection Research; Leibniz Association; Forschungszentrum Borstel; University of Oxford; Institut National de la Sante et de la Recherche Medicale (Inserm); Ecole Normale Superieure de Lyon (ENS de LYON); Centre National de la Recherche Scientifique (CNRS); CNRS - National Institute for Biology (INSB); Universite Claude Bernard Lyon 1; CHU Lyon; Museum National d'Histoire Naturelle (MNHN); Sorbonne Universite;

Universite PSL; Ecole Pratique des Hautes Etudes (EPHE); Centre National
de la Recherche Scientifique (CNRS); CNRS - Institute of Ecology &
Environment (INEE); Universite PSL; Ecole Pratique des Hautes Etudes
(EPHE)
RP Niemann, S (autor correspondente), Res Ctr Borstel, Mol & Expt
Mycobacteriol, Borstel, Germany.; Niemann, S (autor correspondente),
German Ctr Infect Res DZIF, Partner Site Hamburg Lubeck Borstel Riems,
Borstel, Germany.; Niemann, S (autor correspondente), CRyPTIC Consortium,
Oxford, England.
EM sniemann@fz-borstel.de
RI Rasigade, Jean-Philippe/AAE-1667-2021; Niemann, Stefan/C-9327-2011;
Merker, Matthias/P-7399-2015; Ismail, Nazir/KCL-2903-2024; Wirth,
Thierry/B-4915-2008; Fowler, Philip/C-5116-2009
OI Omar, Shaheed Vally/0000-0001-6983-1636; Merker,
Matthias/0000-0003-1386-2331; Lalvani, Ajit/0000-0002-2396-246X; Ismail,
Nazir/0000-0001-7696-3776; Wilkinson, Robert/0000-0002-2753-1800; Wirth,
Thierry/0000-0002-7371-0172; Spitaleri, Andrea/0000-0003-3012-3557;
Cohen, Ted/0000-0002-8091-7198; Nilgiriwala, Kayzad/0000-0002-5458-4916;
Niemann, Stefan/0000-0002-6604-0684; Fowler, Philip/0000-0003-0912-4483;
Moore, David/0000-0002-3748-0154; Knaggs, Jeff/0000-0002-1206-0583
FU CRYPTIC consortium by the Bill & Melinda Gates Foundation [OPP1133541];
Wellcome Trust [200205/Z/15/Z]; Deutsche Forschungsgemeinschaft (DFG,
German Research Foundation) under Germanys Excellence Strategy [EXC
2167]; Leibniz Science Campus Evolutionary Medicine of the LUNG
(EvoLUNG)
FX Parts of this work have been supported within the CRYPTIC consortium by
the Bill & Melinda Gates Foundation [OPP1133541] and the Wellcome Trust
[200205/Z/15/Z], the Deutsche Forschungsgemeinschaft (DFG, German
Research Foundation) under Germanys Excellence Strategy - EXC 2167
Precision Medicine in Inflammation, and the Leibniz Science Campus
Evolutionary Medicine of the LUNG (EvoLUNG). The funders had no role in
study design; in the collection, analysis, and interpretation of data;
in the writing of the report; and in the decision to submit the paper
for publication.
CR Advani J, 2019, FRONT MICROBIOL, V10, DOI 10.3389/fmicb.2019.00309
Ahmad N, 2018, LANCET, V392, P821, DOI 10.1016/S0140-6736(18)31644-1
Almeida D, 2005, CLIN INFECT DIS, V40, P881, DOI 10.1086/427940
Andres S, 2020, AM J RESP CRIT CARE, V201, P1564, DOI 10.1164/rccm.201909-1819LE
[Anonymous], 2019, Global Tuberculosis Report
[Anonymous], Times of India
ANSELIN L, 1995, GEOGR ANAL, V27, P93, DOI 10.1111/j.1538-4632.1995.tb00338.x
Barbier M, 2018, SCI REP-UK, V8, DOI 10.1038/s41598-018-24034-6
Bhatter P, 2012, PLOS ONE, V7, DOI 10.1371/journal.pone.0033507

Bisson GP, 2012, J BACTERIOL, V194, P6441, DOI 10.1128/JB.01013-12
Burgos M, 2003, J INFECT DIS, V188, P1878, DOI 10.1086/379895
Chatterjee A, 2017, TUBERCULOSIS, V107, P63, DOI 10.1016/j.tube.2017.08.002
Chatterjee S, 2018, BMJ GLOB HEALTH, V3, DOI 10.1136/bmjgh-2018-000971
Coll F, 2014, NAT COMMUN, V5, DOI 10.1038/ncomms5812
Comas I, 2012, NAT GENET, V44, P106, DOI 10.1038/ng.1038
Cox HS, 2008, NEW ENGL J MED, V359, P2398, DOI 10.1056/NEJMc0805644
Crispell J, 2019, MICROB GENOMICS, V5, DOI 10.1099/mgen.0.000245
D'souza DTB, 2009, BMC PUBLIC HEALTH, V9, DOI 10.1186/1471-2458-9-211
Dalal A, 2015, PLOS ONE, V10, DOI 10.1371/journal.pone.0116798
Dheda K, 2019, LANCET RESP MED, V7, P820, DOI 10.1016/S2213-2600(19)30263-2
Dheda K, 2017, LANCET RESP MED, V5, P291, DOI [10.1016/S2213-2600(17)30079-6, 10.1016/s2213-2600(17)30079-6]
Ford CB, 2013, NAT GENET, V45, P784, DOI 10.1038/ng.2656
Gagneux S, 2006, SCIENCE, V312, P1944, DOI 10.1126/science.1124410
Grandjean L, 2020, THORAX, V75, P584, DOI 10.1136/thoraxjnl-2019-213281
Hanekom M, 2007, J CLIN MICROBIOL, V45, P1483, DOI 10.1128/JCM.02191-06
Hanekom M, 2011, TUBERCULOSIS, V91, P510, DOI 10.1016/j.tube.2011.07.005
Hartkoorn RC, 2014, ANTIMICROB AGENTS CH, V58, P2979, DOI 10.1128/AAC.00037-14
Hicks ND, 2018, NAT MICROBIOL, V3, P1032, DOI 10.1038/s41564-018-0218-3
Hoffmann H, 2016, AM J RESP CRIT CARE, V193, P337, DOI 10.1164/rccm.201502-0372LE
Ismail NA, 2018, EBIOMEDICINE, V28, P136, DOI 10.1016/j.ebiom.2018.01.005
Kambli P, 2021, TUBERCULOSIS, V127, DOI 10.1016/j.tube.2021.102051
Kohl TA, 2018, EBIOMEDICINE, V34, P131, DOI 10.1016/j.ebiom.2018.07.030
Kohl TA, 2018, PEERJ, V6, DOI 10.7717/peerj.5895
Kranzer K, 2019, AM J RESP CRIT CARE, V200, P514, DOI 10.1164/rccm.201902-0260LE
Kremer K, 2009, EMERG INFECT DIS, V15, P335, DOI 10.3201/eid1502.080795
Ladipo Manyika Sarah, 2021, Meeting Report of the wHO Expert Consultation on the Definition of Extensively Drug-Resistant tuberculosis, 2729 October 2020
Nguyen LT, 2015, MOL BIOL EVOL, V32, P268, DOI 10.1093/molbev/msu300
Li H, 2009, BIOINFORMATICS, V25, P2078, DOI 10.1093/bioinformatics/btp352
Li H, 2009, BIOINFORMATICS, V25, P1094, DOI [10.1093/bioinformatics/btp100, 10.1093/bioinformatics/btp324]
Mamdani H, 2021, FRONT ONCOL, V11, DOI 10.3389/fonc.2021.736620
Manson AL, 2017, CLIN INFECT DIS, V64, P1494, DOI 10.1093/cid/cix169
Mathema B, 2006, CLIN MICROBIOL REV, V19, P658, DOI 10.1128/CMR.00061-05
Meehan CJ, 2018, EBIOMEDICINE, V37, P410, DOI 10.1016/j.ebiom.2018.10.013
Merker M, 2020, GENOME MED, V12, DOI 10.1186/s13073-020-00726-5

   Merker M, 2018, ELIFE, V7, DOI 10.7554/eLife.38200
   Merker M, 2015, NAT GENET, V47, P242, DOI 10.1038/ng.3195
   Polsfuss S, 2019, CLIN INFECT DIS, V69, P1229, DOI 10.1093/cid/ciz074
   Rasigade JP, 2017, SCI REP-UK, V7, DOI 10.1038/srep45326
   Sanchez-Padilla E, 2015, NEW ENGL J MED, V372, P1181, DOI 10.1056/NEJMc1413930
   Sethi S, 2020, J GLOB ANTIMICROB RE, V20, P11, DOI 10.1016/j.jgar.2019.05.019
   Sharma P, 2019, J FAM MED PRIM CARE, V8, P3388, DOI 10.4103/jfmpc.jfmpc_409_19
   Sherman DR, 1996, SCIENCE, V272, P1641, DOI 10.1126/science.272.5268.1641
   Trébucq A, 2020, J CLIN MED, V9, DOI 10.3390/jcm9010055
   Wirth T, 2020, EVOL APPL, V13, P1513, DOI 10.1111/eva.12991
   Wirth T, 2020, NAT MICROBIOL, V5, P735, DOI 10.1038/s41564-020-0676-2
   World Health Organization, Global tuberculosis report 2020
   World Health Organization, 2021, Catalogue of Mutations in Mycobacterium tuberculosis Complex and Their Association with Drug Resistance
   Yates TA, 2016, LANCET INFECT DIS, V16, P227, DOI 10.1016/S1473-3099(15)00499-5
   Zhang HK, 2012, NUCLEIC ACIDS RES, V40, pW569, DOI 10.1093/nar/gks576
NR 59
TC 21
Z9 23
U1 0
U2 6
PU BMC
PI LONDON
PA CAMPUS, 4 CRINAN ST, LONDON N1 9XW, ENGLAND
SN 1756-994X
J9 GENOME MED
JI Genome Med.
PD AUG 22
PY 2022
VL 14
IS 1
AR 95
DI 10.1186/s13073-022-01076-0
PG 16
WC Genetics & Heredity
WE Science Citation Index Expanded (SCI-EXPANDED)
SC Genetics & Heredity
GA 3X9KO
UT WOS:000843351700001
PM 35989319
OA gold, Green Published
DA 2025-05-07
ER

PT J
AU Barilar, I
   Fernando, T
   Utpatel, C

Abujate, C
Madeira, CM
José, B
Mutaquiha, C
Kranzer, K
Niemann, T
Ismael, N
de Araujo, L
Wirth, T
Niemann, S
Viegas, S
AF Barilar, Ivan
Fernando, Tatiana
Utpatel, Christian
Abujate, Claudio
Madeira, Carla Maria
Jose, Benedita
Mutaquiha, Claudia
Kranzer, Katharina
Niemann, Tanja
Ismael, Nalia
de Araujo, Leonardo
Wirth, Thierry
Niemann, Stefan
Viegas, Sofia
TI Emergence of bedaquiline-resistant tuberculosis and of multidrug-resistant and extensively drug-resistant Mycobacterium tuberculosis strains with rpoB Ile491Phe mutation not detected by Xpert MTB/RIF in Mozambique: a retrospective observational study
SO LANCET INFECTIOUS DISEASES
LA English
DT Article
AB Background In 2021, an estimated 4800 people developed rifampicin-resistant tuberculosis in Mozambique, 75% of which went undiagnosed. Detailed molecular data on rifampicin-resistant and multidrug-resistant (MDR) tuberculosis are not available. Here, we aimed at gaining precise data on the determinants of rifampicin-resistant and MDR tuberculosis in Mozambique. Methods In this retrospective observational study, we performed whole-genome sequencing of 704 rifampicinresistant Mycobacterium tuberculosis complex (Mtbc) strains submitted to the National Tuberculosis Reference Laboratory (NTRL) in Maputo, Mozambique, between 2015 and 2021. Phylogenetic strain classification, genomic resistance prediction, and cluster analysis were performed. Findings Between Jan 1, 2015, and July 31, 2021, 2606 Mtbc isolates with an isoniazid or rifampicin resistance were identified in the NTRL biobank, of which, 1483 (56 center dot 9%) were from men, 1114 (42 center dot 7%) from women, and nine (0 center dot 4%) were unknown. Genome-based drug-resistant prediction classified 704 Mtbc strains as rifampicin resistant. 628 (89%) of the 704 Mtbc strains were classified MDR; of those, 146 (23%) were pre-extensively drug resistant (preXDR; additional fluoroquinolone resistance), and 24 (4%) extensively drug resistant (XDR; combined fluoroquinolone and bedaquiline resistance). Overall, 61 (9%) of 704 strains revealed resistance to bedaquiline: five (7%) of 76

rifampicin resistant plus bedaquiline resistant, 32 (7%) of 458 MDR plus bedaquiline resistant, and 24 (100%) of 24 XDR. Prevalence of bedaquiline resistance increased from 3% in 2016 to 14% in 2021. The cluster rate (12 singlenucleotide polymorphism threshold) was 42% for rifampicin-resistant strains, 78% for MDR strains, 94% for preXDR strains, and 96% for XDR Mtbc strains. 31 (4%) of 704 Mtbc strains, belonging to a diagnostic escape outbreak strain previously described in Eswatini (group_56), had an rpoB Ile491Phe mutation which is not detected by Xpert MTB/RIF (no other rpoB mutation). Of these, 23 (74%) showed additional resistance to bedaquiline, 13 (42%) had bedaquiline and fluoroquinolone resistance, and two (6%) were bedaquiline, fluoroquinolone, and delamanid resistant. Interpretation Pre-XDR resistance is highly prevalent among MDR Mtbc strains in Mozambique and so is bedaquiline resistance; and the frequency of bedaquiline resistance quadrupled over time and was found even in Mtbc strains without fluoroquinolone resistance. Importantly, strains with Ile491Phe mutation were frequent, accounting for 31% (n=10) of MDR plus bedaquiline-resistant strains and 54% (n=13) of XDR Mtbc strains. Given the current diagnostic algorithms and treatment regimens, both the emergence of rifampicin resistance due to Ile491Phe and bedaquiline resistance might jeopardise MDR tuberculosis prevention and care unless sequencing-based technology is rolled out. The potential cross border spread of diagnostic escape strains needs further investigation.
Copyright (c) 2023 Elsevier Ltd. All rights reserved.
C1 [Barilar, Ivan; Utpatel, Christian; Niemann, Tanja; de Araujo, Leonardo; Niemann, Stefan] Res Ctr Borstel, Mol & Expt Mycobacteriol, D-23845 Borstel, Germany.

[Barilar, Ivan; Utpatel, Christian; Niemann, Tanja; de Araujo, Leonardo; Niemann, Stefan] German Ctr Infect Res, Partner Site Hamburg Lubeck Borstel Riems, Borstel, Germany.

[Fernando, Tatiana; Madeira, Carla Maria; Ismael, Nalia] Inst Nacl Saude, Marracuene, Mozambique.

[Jose, Benedita; Mutaquiha, Claudia] Natl TB Control Program, Directorate Publ Hlth, Minist Hlth, Maputo, Mozambique.

[Kranzer, Katharina] Biomed Res & Training Inst, Harare, Zimbabwe.

[Kranzer, Katharina] London Sch Hyg & Trop Med, Dept Clin Res, Fac Infect & Trop Dis, London, England.

[Kranzer, Katharina] Ludwig Maximilians Univ Munchen, Univ Hosp, Div Infect Dis & Trop Med, Munich, Germany.

[Wirth, Thierry] Paris Sci & Lettres Univ, Ecole Pratique Hautes Etud, Paris, France.

[Wirth, Thierry] Sorbonne Univ, CNRS, Museum Natl Hist Nat, Inst Systemat Evolut Biodiversite, Paris, France.

[Wirth, Thierry] Univ Antilles, Ecole Prat Hautes Etud, Paris, France.

[Niemann, Stefan] Univ Namibia, Sch Med, Dept Human Biol & Translat Med Sci, Windhoek, Namibia.

C3 Leibniz Association; Forschungszentrum Borstel; German Center for Infection Research; Biomedical Research Training Institute; University of London; London School of Hygiene & Tropical Medicine; University of Munich; Universite PSL; Ecole Pratique des Hautes Etudes (EPHE); Sorbonne Universite; Centre National de la Recherche Scientifique (CNRS); Museum National d'Histoire Naturelle (MNHN); Universite PSL; Ecole Pratique des Hautes Etudes (EPHE); University of Namibia

RP Niemann, S (autor correspondente), Res Ctr Borstel, Mol & Expt Mycobacteriol, D-23845 Borstel, Germany.; Niemann, S (autor

correspondente), Univ Namibia, Sch Med, Dept Human Biol & Translat Med Sci, Windhoek, Namibia.
EM sniemann@fz-borstel.de
RI Niemann, Stefan/C-9327-2011; Kranzer, Katharina/O-8845-2014; Wirth, Thierry/B-4915-2008
OI Wirth, Thierry/0000-0002-7371-0172; Mutaquiha, Claudia Cardoso/0000-0003-3030-3769; Niemann, Stefan/0000-0002-6604-0684
FU German Ministry of Health through the Seq_MDRTB-Net project; Deutsche Forschungsgemeinschaft under Germany's Excellence Strategy Precision Medicine in Inflammation; Research Training Group 2501 TransEvo; Leibniz Science Campus Evolutionary Medicine of the Lung; German Ministry of Education and Research via the German Center for Infection Research
FX The German Ministry of Health through the Seq_MDRTB-Net project, the Deutsche Forschungsgemeinschaft under Germany's Excellence Strategy Precision Medicine in Inflammation and the Research Training Group 2501 TransEvo, the Leibniz Science Campus Evolutionary Medicine of the Lung, and the German Ministry of Education and Research via the German Center for Infection Research.
CR Bates PA, 2001, PROTEINS, P39
Baym M, 2015, PLOS ONE, V10, DOI 10.1371/journal.pone.0128036
Beckert P, 2020, GENOME MED, V12, DOI 10.1186/s13073-020-00793-8
Chesov E, 2022, EUR RESPIR J, V59, DOI 10.1183/13993003.00621-2021
Coll F, 2014, NAT COMMUN, V5, DOI 10.1038/ncomms5812
Dheda K, 2017, LANCET RESP MED, V5, P291, DOI [10.1016/S2213-2600(17)30079-6, 10.1016/s2213-2600(17)30079-6]
Dreyer V, 2022, GENOME MED, V14, DOI 10.1186/s13073-022-01076-0
Feuerriegel S, 2021, EUR RESPIR J, V57, DOI 10.1183/13993003.01796-2020
He WC, 2021, J GLOB ANTIMICROB RE, V26, P241, DOI 10.1016/j.jgar.2021.06.007
INSIDA, 2021, Mozambique population-based HIV impact assessment
Ismail NA, 2022, LANCET INFECT DIS, V22, P496, DOI 10.1016/S1473-3099(21)00470-9
Katale BZ, 2020, BMC GENOMICS, V21, DOI 10.1186/s12864-020-6577-1
Kohl TA, 2018, PEERJ, V6, DOI 10.7717/peerj.5895
Makhado NA, 2018, LANCET INFECT DIS, V18, P1350, DOI 10.1016/S1473-3099(18)30496-1
Merker M, 2022, NAT COMMUN, V13, DOI 10.1038/s41467-022-32455-1
Merker M, 2015, NAT GENET, V47, P242, DOI 10.1038/ng.3195
Mvelase NR, 2023, AFR J LAB MED, V12, DOI 10.4102/ajlm.v12i1.1975
Omar SV, 2022, NEW ENGL J MED, V386, P93, DOI 10.1056/NEJMc2103049
Oostvogels S, 2022, MICROB GENOMICS, V8, DOI 10.1099/mgen.0.000815
Republica de Mocambique. Ministerio Da Saude Direccao Nacional De Saude Publica, 2021, Relatorio anual 2021
Roetzer A, 2011, J CLIN MICROBIOL, V49, P4173, DOI 10.1128/JCM.05293-11
Sibandze DB, 2022, GENOME MED, V14, DOI 10.1186/s13073-022-01054-6
Sonnenkalb L, 2023, LANCET MICROBE, V4, pE358, DOI 10.1016/S2666-5247(23)00002-2

   Van Rie A, 2022, LANCET INFECT DIS, V22, P1411, DOI 10.1016/S1473-3099(22)00543-6
   VANEMBDEN JDA, 1993, J CLIN MICROBIOL, V31, P406, DOI 10.1128/JCM.31.2.406-409.1993
   Walker TM, 2013, LANCET INFECT DIS, V13, P137, DOI 10.1016/S1473-3099(12)70277-3
   WHO, 2010, TREATMENT OF TUBERCULOSIS: GUIDELINES, FOURTH EDITION, P1
   WHO, 2022, Rapid communication: key changes to the treatment of drug-resistant tuberculosis
   World Health Organization, Global tuberculosis report 2020
NR 29
TC 16
Z9 18
U1 3
U2 8
PU ELSEVIER SCI LTD
PI London
PA 125 London Wall, London, ENGLAND
SN 1473-3099
EI 1474-4457
J9 LANCET INFECT DIS
JI Lancet Infect. Dis.
PD MAR
PY 2024
VL 24
IS 3
BP 297
EP 307
DI 10.1016/S1473-3099(23)00498-X
EA FEB 2024
PG 11
WC Infectious Diseases
WE Science Citation Index Expanded (SCI-EXPANDED)
SC Infectious Diseases
GA NQ7G4
UT WOS:001201979000001
PM 37956677
DA 2025-05-07
ER

PT J
AU Beckert, P
   Sanchez-Padilla, E
   Merker, M
   Dreyer, V
   Kohl, TA
   Utpatel, C
   Köser, CU
   Barilar, I
   Ismail, N
   Omar, SV
   Klopper, M
   Warren, RM
   Hoffmann, H

Maphalala, G
   Ardizzoni, E
   de Jong, BC
   Kerschberger, B
   Schramm, B
   Andres, S
   Kranzer, K
   Maurer, FP
   Bonnet, M
   Niemann, S
AF Beckert, Patrick
   Sanchez-Padilla, Elisabeth
   Merker, Matthias
   Dreyer, Viola
   Kohl, Thomas A.
   Utpatel, Christian
   Koeser, Claudio U.
   Barilar, Ivan
   Ismail, Nazir
   Omar, Shaheed Vally
   Klopper, Marisa
   Warren, Robin M.
   Hoffmann, Harald
   Maphalala, Gugu
   Ardizzoni, Elisa
   de Jong, Bouke C.
   Kerschberger, Bernhard
   Schramm, Birgit
   Andres, Soenke
   Kranzer, Katharina
   Maurer, Florian P.
   Bonnet, Maryline
   Niemann, Stefan
TI MDR M. tuberculosis outbreak clone in Eswatini missed by Xpert
   has elevated bedaquiline resistance dated to the pre-treatment era
SO GENOME MEDICINE
LA English
DT Article
DE Tuberculosis; Multidrug resistance; Resistance evolution; MDR outbreak
   strains; Diagnostice escape; Treatment escape; Treatment failure
ID HIGH PREVALENCE; SWAZILAND; SUSCEPTIBILITY; MTB/RIF; COMPLEX
AB Background Multidrug-resistant (MDR) Mycobacterium tuberculosis complex strains not detected by commercial molecular drug susceptibility testing (mDST) assays due to the RpoB I491F resistance mutation are threatening the control of MDR tuberculosis (MDR-TB) in Eswatini. Methods We investigate the evolution and spread of MDR strains in Eswatini with a focus on bedaquiline (BDQ) and clofazimine (CFZ) resistance using whole-genome sequencing in two collections ((1) national drug resistance survey, 2009-2010; (2) MDR strains from the Nhlangano region, 2014-2017). Results MDR strains in collection 1 had a high cluster rate (95%, 117/123 MDR strains) with 55% grouped into the two largest clusters (gCL3, n = 28; gCL10, n = 40). All gCL10 isolates, which likely emerged around 1993 (95% highest posterior density 1987-1998), carried the mutation RpoB I491F that is missed by commercial mDST assays. In addition, 21 (53%)

gCL10 isolates shared a Rv0678 M146T mutation that correlated with elevated minimum inhibitory concentrations (MICs) to BDQ and CFZ compared to wild type isolates. gCL10 isolates with the Rv0678 M146T mutation were also detected in collection 2. Conclusion The high clustering rate suggests that transmission has been driving the MDR-TB epidemic in Eswatini for three decades. The presence of MDR strains in Eswatini that are not detected by commercial mDST assays and have elevated MICs to BDQ and CFZ potentially jeopardizes the successful implementation of new MDR-TB treatment guidelines. Measures to limit the spread of these outbreak isolates need to be implemented urgently.
C1 [Beckert, Patrick; Merker, Matthias; Dreyer, Viola; Kohl, Thomas A.; Utpatel, Christian; Barilar, Ivan; Niemann, Stefan] Res Ctr Borstel, Mol & Expt Mycobacteriol, Parkallee 1, D-23845 Borstel, Germany.
[Beckert, Patrick; Niemann, Stefan] German Ctr Infect Res DZIF, Partner Site Hamburg Lubeck Borstel Riems, Borstel, Germany.
[Sanchez-Padilla, Elisabeth; Schramm, Birgit; Bonnet, Maryline] Epicentre, Paris, France.
[Koeser, Claudio U.] Univ Cambridge, Dept Genet, Cambridge, England.
[Ismail, Nazir; Omar, Shaheed Vally] Natl Inst Communicable Dis, Ctr TB, Natl TB Reference Lab, WHO TB Supranatl Lab Network,Natl Hlth Lab Serv, Johannesburg, South Africa.
[Ismail, Nazir] Univ Pretoria, Dept Med Microbiol, Pretoria, South Africa.
[Ismail, Nazir] Univ Witwatersrand, Dept Internal Med, Johannesburg, South Africa.
[Klopper, Marisa; Warren, Robin M.] Stellenbosch Univ, Fac Med & Hlth Sci, DST NRF Ctr Excellence Biomed TB Res, Cape Town, South Africa.
[Klopper, Marisa; Warren, Robin M.] Stellenbosch Univ, Fac Med & Hlth Sci, South African Med Res Council Ctr TB Res, Div Mol Biol & Human Genet, Cape Town, South Africa.
[Hoffmann, Harald] SYNLAB Gauting, Gauting, Germany.
[Hoffmann, Harald] IML red GmbH, WHO Supranatl Reference Lab TB, Inst Microbiol & Lab Med, Gauting, Germany.
[Maphalala, Gugu] Minist Hlth, Natl TB Reference Lab NTRL, Mbabane, Switzerland.
[Ardizzoni, Elisa; de Jong, Bouke C.] Inst Trop Med, Dept Biomed Sci, Mycobacteriol Unit, Antwerp, Belgium.
[Kerschberger, Bernhard] Med Sans Frontieres MSF Eswatini, Mbabane, Eswatini.
[Andres, Soenke; Kranzer, Katharina; Maurer, Florian P.; Niemann, Stefan] Res Ctr Borstel, Natl & WHO Supranatl Reference Ctr Mycobacteria, Borstel, Germany.
[Kranzer, Katharina] London Sch Hyg & Trop Med, London, England.
[Maurer, Florian P.] Univ Med Ctr Hamburg, Inst Med Microbiol Virol & Hyg, Eppendorf, Hamburg, Germany.
[Bonnet, Maryline] Univ Montpellier, INSERM U1175, IRD UMI233, Montpellier, France.
[Niemann, Stefan] Univ Namibia, Sch Med, Biochem & Microbiol, Windhoek, Namibia.
C3 Leibniz Association; Forschungszentrum Borstel; German Center for Infection Research; University of Cambridge; National Institute for Communicable Diseases (NICD); National Health Laboratory Service; University of Pretoria; University of Witwatersrand; Stellenbosch University; National Research Foundation - South Africa; Stellenbosch

University; SYNLAB Group; Institute of Tropical Medicine (ITM); Leibniz
Association; Forschungszentrum Borstel; University of London; London
School of Hygiene & Tropical Medicine; University of Hamburg; University
Medical Center Hamburg-Eppendorf; Universite de Montpellier; Institut
National de la Sante et de la Recherche Medicale (Inserm); University of
Namibia
RP Niemann, S (autor correspondente), Res Ctr Borstel, Mol & Expt Mycobacteriol, Parkallee 1, D-23845 Borstel, Germany.; Niemann, S (autor correspondente), German Ctr Infect Res DZIF, Partner Site Hamburg Lubeck Borstel Riems, Borstel, Germany.; Niemann, S (autor correspondente), Res Ctr Borstel, Natl & WHO Supranatl Reference Ctr Mycobacteria, Borstel, Germany.; Niemann, S (autor correspondente), Univ Namibia, Sch Med, Biochem & Microbiol, Windhoek, Namibia.
EM sniemann@fz-borstel.de
RI Klopper, Marisa/U-9887-2018; Omar, Shaheed/AAB-5071-2020; Kerschberger,
Bernhard/JXX-4260-2024; Niemann, Stefan/C-9327-2011; Maurer,
Florian/ABA-1918-2020; Kohl, Thomas/AAB-9782-2020; Ismail,
Nazir/KCL-2903-2024; Maurer, Florian/J-9687-2014; Kranzer,
Katharina/O-8845-2014; de Jong, Bouke/A-3636-2013; Koser,
Claudio/I-3740-2013; Merker, Matthias/P-7399-2015
OI Ismail, Nazir/0000-0001-7696-3776; Maurer, Florian/0000-0001-8353-5964;
Omar, Shaheed Vally/0000-0001-6983-1636; Niemann,
Stefan/0000-0002-6604-0684; Klopper, Marisa/0000-0002-9318-8289;
Kranzer, Katharina/0000-0001-5691-7270; de Jong,
Bouke/0000-0002-1017-4675; Koser, Claudio/0000-0002-0232-846X; Warren,
Robin/0000-0001-5741-7358; Kerschberger, Bernhard/0000-0003-0525-7262;
Merker, Matthias/0000-0003-1386-2331
FU European Union TB-PAN-NET project [FP7-223681]; Medecins Sans
Frontieres-Switzerland; German Center for Infection Research, Deutsche
Forschungsgemeinschaft (DFG, German Research Foundation) under Germanys
Excellence Strategy [EXC 2167]; Leibniz Science Campus Evolutionary
Medicine of the LUNG (EvoLUNG)
FX Parts of this work have been supported by the European Union TB-PAN-NET
(FP7-223681) project, by Medecins Sans Frontieres-Switzerland, and by
German Center for Infection Research, Deutsche Forschungsgemeinschaft
(DFG, German Research Foundation) under Germanys Excellence Strategy -
EXC 2167, and Leibniz Science Campus Evolutionary Medicine of the LUNG
(EvoLUNG). The funders had no role in the study design; in the
collection, analysis, and interpretation of the data; in the writing of
the report; and in the decision to submit the paper for publication.
CR Andres S, 2020, AM J RESP CRIT CARE, V201, P1564, DOI 10.1164/rccm.201909-1819LE
[Anonymous], 2021, ACC BUDG
de Vos M, 2019, NEW ENGL J MED, V380, P2178, DOI 10.1056/NEJMc1815121
El Achkar S, 2019, EMERG INFECT DIS, V25, P564, DOI 10.3201/eid2503.181375

Feuerriegel S, 2021, EUR RESPIR J, V57, DOI 10.1183/13993003.01796-2020
Grandjean L, 2017, PLOS ONE, V12, DOI 10.1371/journal.pone.0189838
Gröschel MI, 2018, PLOS PATHOG, V14, DOI 10.1371/journal.ppat.1007297
Hariguchi N, 2020, ANTIMICROB AGENTS CH, V64, DOI 10.1128/AAC.02020-19
Ismail NA, 2018, EBIOMEDICINE, V28, P136, DOI 10.1016/j.ebiom.2018.01.005
Jouet A, 2021, EUR RESPIR J, V57, DOI 10.1183/13993003.02338-2020
Kadura S, 2020, J ANTIMICROB CHEMOTH, V75, P2031, DOI 10.1093/jac/dkaa136
Kahlmeter G, 2019, J CLIN MICROBIOL, V57, DOI 10.1128/JCM.01129-19
Kahlmeter G, 2015, J ANTIMICROB CHEMOTH, V70, P2427, DOI 10.1093/jac/dkv145
Köser CU, 2012, ANTIMICROB AGENTS CH, V56, P6080, DOI 10.1128/AAC.01641-12
Köser CU, 2019, INT J INFECT DIS, V80, pS32, DOI 10.1016/j.ijid.2019.02.027
Kranzer K, 2019, AM J RESP CRIT CARE, V200, P514, DOI 10.1164/rccm.201902-0260LE
Leinonen R, 2011, NUCLEIC ACIDS RES, V39, pD28, DOI 10.1093/nar/gkq967
Makhado NA, 2018, LANCET INFECT DIS, V18, P1350, DOI 10.1016/S1473-3099(18)30496-1
Mauch-Mücke K, 2020, FRONT CELL INFECT MI, V10, DOI 10.3389/fcimb.2020.577428
Merker M, 2020, GENOME MED, V12, DOI 10.1186/s13073-020-00726-5
Merker M, 2018, ELIFE, V7, DOI 10.7554/eLife.38200
Ministry of Health and Social Welfare Eswatini, DRUG RES TUB MAN GUI
Nimmo C, 2020, LANCET MICROBE, V1, pE165, DOI 10.1016/S2666-5247(20)30031-8
Nimmo C, 2020, EUR RESPIR J, V55, DOI 10.1183/13993003.02383-2019
O'Donnell MR, 2014, LANCET, V383, P1193, DOI 10.1016/S0140-6736(14)60043-X
Polsfuss S, 2019, CLIN INFECT DIS, V69, P1229, DOI 10.1093/cid/ciz074
Roetzer A, 2013, PLOS MED, V10, DOI 10.1371/journal.pmed.1001387
Sanchez-Padilla E, 2015, NEW ENGL J MED, V372, P1181, DOI 10.1056/NEJMc1413930
Sanchez-Padilla E, 2012, EMERG INFECT DIS, V18, P29, DOI 10.3201/eid1801.110850
Sikhondze W, 2015, PUBLIC HEALTH ACTION, V5, P140, DOI 10.5588/pha.15.0001
Sikhondze Welile, 2019, INT J TUBERC LUNG D, V23
Nguyen TVA, 2018, CLIN INFECT DIS, V66, P1625, DOI 10.1093/cid/cix992
Valsesia G, 2015, J CLIN MICROBIOL, V53, P1806, DOI 10.1128/JCM.03505-14
Vambe D, 2017, PUBLIC HEALTH ACTION, V7, P240, DOI 10.5588/pha.17.0054
Villellas C, 2017, J ANTIMICROB CHEMOTH, V72, P684, DOI 10.1093/jac/dkw502
Weniger T, 2012, INFECT GENET EVOL, V12, P748, DOI 10.1016/j.meegid.2012.01.021
WHO, 2020, Life Expectancy and Healthy Life Expectancy
World Health Organisation, 2018, TECHN REP CRIT CONCR
World Health Organization, 2019, GLOB TUB REP 20119
World Health Organization, 2019, RAPID COMMUNICATION

World Health Organization, 2020, WHO CONS GUID DRUG R, DOI 10.30978/TB2021-2-86
Xu J, 2017, ANTIMICROB AGENTS CH, V61, DOI [10.1128/AAC.00239-17, 10.1128/aac.00239-17]
Zignol M, 2018, LANCET INFECT DIS, V18, P675, DOI 10.1016/S1473-3099(18)30073-2
NR 43
TC 53
Z9 55
U1 0
U2 2
PU BMC
PI LONDON
PA CAMPUS, 4 CRINAN ST, LONDON N1 9XW, ENGLAND
SN 1756-994X
J9 GENOME MED
JI Genome Med.
PD DEC 25
PY 2020
VL 12
IS 1
AR 104
DI 10.1186/s13073-020-00793-8
PG 11
WC Genetics & Heredity
WE Science Citation Index Expanded (SCI-EXPANDED)
SC Genetics & Heredity
GA PA6XJ
UT WOS:000595775500001
PM 33239092
OA gold, Green Published
DA 2025-05-07
ER

PT J
AU Kelly, H
Thompson, AL
Sorensen, MV
AF Kelly, H.
Thompson, A. L.
Sorensen, M. V.
TI Immunoregulatory effects of childhood endotoxemia on total-IgE response:
Implications for the hygiene hypothesis from Galapagos, Ecuador
SO AMERICAN JOURNAL OF HUMAN BIOLOGY
LA English
DT Meeting Abstract
C1 [Kelly, H.; Thompson, A. L.; Sorensen, M. V.] Univ North Carolina Chapel Hill, Dept Anthropol, Chapel Hill, NC USA.
[Thompson, A. L.] Univ N Carolina, Carolina Populat Ctr, Chapel Hill, NC USA.
C3 University of North Carolina; University of North Carolina Chapel Hill;
University of North Carolina School of Medicine; University of North

Carolina; University of North Carolina Chapel Hill
FU NSF DDRIG [1341156]; Wenner-Gren Dissertation Fieldwork Grant;
Triangle
Center for Evolutionary Medicine; NICHD [5T32HD007168]; University of
North Carolina's (UNC) Institute for Global Health and Infectious
Disease; UNC's Royster Society; Direct For Social, Behav & Economic
Scie; Division Of Behavioral and Cognitive Sci [1341156] Funding
Source:
National Science Foundation
FX NSF DDRIG (# 1341156); Wenner-Gren Dissertation Fieldwork Grant;
Triangle Center for Evolutionary Medicine; NICHD 5T32HD007168,
Carolina
Population Center; University of North Carolina's (UNC) Institute for
Global Health and Infectious Disease; UNC's Royster Society of
Fellows.
NR 0
TC 0
Z9 0
U1 0
U2 0
PU WILEY
PI HOBOKEN
PA 111 RIVER ST, HOBOKEN 07030-5774, NJ USA
SN 1042-0533
EI 1520-6300
J9 AM J HUM BIOL
JI Am. J. Hum. Biol.
PD MAR-APR
PY 2018
VL 30
IS 2
MA P56
PG 2
WC Anthropology; Biology
WE Science Citation Index Expanded (SCI-EXPANDED); Social Science
Citation Index (SSCI)
SC Anthropology; Life Sciences & Biomedicine - Other Topics
GA FZ0CB
UT WOS:000427233300068
DA 2025-05-07
ER

PT J
AU Kranke, N
AF Kranke, Nina
TI Do concepts of individuality account for individuation practices in
studies of host-parasite systems? A modeling account of biological
individuality
SO THEORY IN BIOSCIENCES
LA English
DT Article; Early Access
DE Biological individuality; Individuation practices; Host-parasite
interaction; Host-parasite coevolution; Models; Pluralism
ID DARWINIAN MEDICINE; HYGIENE; PHILOSOPHY; ORGANISMS; SELECTION;

EVOLUTION; ADEQUACY; HISTORY
AB In recent discussions, the widespread conviction that scientific individuation practices are governed by theories and concepts of biological individuality has been challenged, particularly by advocates of practice-based approaches. This discussion raises questions about the relationship between individuation practices and concepts of individuality. In this paper, I discuss four studies of host-parasite systems and analyze the respective individuation practices to see whether they correspond to established concepts of biological individuality. My analysis suggests that scientists individuate biological systems on different levels of organization and that the researchers' respective emphasis on one of the levels depends on the explanandum and research context as well as epistemic aims and purposes. It thus makes sense to use different concepts of individuality to account for different individuation practices. However, not all individuation practices are represented equally well by concepts of biological individuality. To account for this observation, I propose that concepts of individuality should be understood as abstracted, idealized, or simplified models that represent only certain aspects of scientific practice. A modeling account suggests a pluralistic view of concepts of biological individuality that not only allows the coexistence of different kinds of individuality (e.g., evolutionary individuality, immunological individuality, ecological individuality) but also of normative and descriptive concepts.
C1 [Kranke, Nina] Univ Freiburg, Chair Nat Conservat & Landscape Ecol, Stefan Meier Str 76, D-79104 Freiburg, Germany.
C3 University of Freiburg
RP Kranke, N (autor correspondente), Univ Freiburg, Chair Nat Conservat & Landscape Ecol, Stefan Meier Str 76, D-79104 Freiburg, Germany.
EM nina.kranke@nature.uni-freiburg.de
RI Kranke, Nina/LFV-5481-2024
FU Projekt DEAL; German Research Foundation (DFG) [281125614/GRK2220]
FX Open Access funding enabled and organized by Projekt DEAL. My research leading to these results was funded by the German Research Foundation (DFG)-281125614/GRK2220.
CR [Anonymous], 2001, Journal of Economic Methodology, DOI [DOI 10.1080/13501780110078972, 10.1080/13501780110078972]
Baedke J., 2019, OLD QUESTIONS YOUNG, P269, DOI [10.1007/978-3-030-18202-1_13, DOI 10.1007/978-3-030-18202-1_13]
Bailer-Jones DanielaM., 2002, The Blackwell Guide to the Philosophy of Science, P108, DOI DOI 10.1002/9780470756614.CH6
Blackwell A, 2000, DIAGRAMMATIC REPRESENTATION AND REASONING, P47
Booth A, 2014, BIOL PHILOS, V29, P657, DOI 10.1007/s10539-014-9449-8
Bourrat P, 2018, HIST PHIL LIFE SCI, V40, DOI 10.1007/s40656-018-0194-1
Bueno Otavio, 2018, Individuation, Process, and Scientific Practices., P1
Catania F, 2017, THEOR BIOSCI, V136, P89, DOI 10.1007/s12064-016-0240-z
Clarke E, 2013, J PHILOS, V110, P413, DOI 10.5840/jphil2013110817
Clarke Ellen, 2011, Biology Theory, V5, P312
Currie A, 2015, PALGRAVE HANDBOOK OF PHILOSOPHICAL METHODS, P553
Dawkins R., 2016, The selfish gene, V40th
Dawkins R., 1999, EXTENDED PHENOTYPE L

DiFrisco J, 2019, BRIT J PHILOS SCI, V70, P845, DOI 10.1093/bjps/axy006
Dupre J., 2009, PHILOS THEORY BIOL, V1, pe003, DOI [10.3998/ptb.6959004.0001.003, DOI 10.3998/PTB.6959004.0001.003]
Ereshefsky M, 2015, P NATL ACAD SCI USA, V112, P10126, DOI 10.1073/pnas.1421377112
Ereshefsky M, 2013, BIOL PHILOS, V28, P331, DOI 10.1007/s10539-012-9340-4
Filyk HA, 2016, EBIOMEDICINE, V13, P46, DOI 10.1016/j.ebiom.2016.10.007
Frigg Roman, 2020, The Stanford Encyclopedia of Philosophy
GHISELIN MT, 1974, SYST ZOOL, V23, P536, DOI 10.2307/2412471
Giere RN, 2004, PHILOS SCI, V71, P742, DOI 10.1086/425063
Godfrey-Smith P, 2013, VIENNA SER THEOR BIO, P17
Godfrey-Smith Peter., 2009, Darwinian Populations and Natural Selection, DOI DOI 10.1093/ACPROF:OSOBL/9780199552047.001.0001
Gould SJ, 1999, P NATL ACAD SCI USA, V96, P11904, DOI 10.1073/pnas.96.21.11904
Hawdon JM, 1996, CURR OPIN GENET DEV, V6, P618, DOI 10.1016/S0959-437X(96)80092-X
Hotez PJ, 2004, NEW ENGL J MED, V351, P799, DOI 10.1056/NEJMra032492
HULL DL, 1976, SYST ZOOL, V25, P174, DOI 10.2307/2412744
HULL DL, 1978, PHILOS SCI, V45, P335, DOI 10.1086/288811
Huneman Philippe, 2014, Biological Theory, V9, P361, DOI 10.1007/s13752-014-0192-9
Huneman Philippe, 2014, Biology Theory, V9, P374, DOI 10.1007/s13752-014-0193-8
Kaiser MI, 2021, BIOL PHILOS, V36, DOI 10.1007/s10539-021-09791-5
Kendig K., 2016, Natural kinds and classification in scientific practice
Kinzel K, 2015, STUD HIST PHILOS SCI, V49, P48, DOI 10.1016/j.shpsa.2014.12.001
Knuuttila T, 2011, STUD HIST PHILOS SCI, V42, P262, DOI 10.1016/j.shpsa.2010.11.034
Kovaka K, 2015, PHILOS SCI, V82, P1092, DOI 10.1086/683443
Lewontin R.C., 1970, Annual review of ecology and systematics, V1, P1
Love A. C., 2018, INDIVIDUATION PROCES, P165
Love AC., 2017, Biological individuality: Integrating scientific, philosophical and historical perspectives, P318
McConwell AK., 2023, Biological individuality, DOI [10.1017/9781108942775, DOI 10.1017/9781108942775]
Méthot PO, 2011, THEOR MED BIOETH, V32, P75, DOI 10.1007/s11017-010-9167-4
Millstein RobertaL., 2018, Individuation, Process, and Scientific Practices, P279, DOI [10.1093/oso/9780190636814.003.0013, DOI 10.1093/OSO/9780190636814.003.0013]
Moran NA, 2015, PLOS BIOL, V13, DOI 10.1371/journal.pbio.1002311
Morgan M. S., 1999, Models as mediators: Perspectives on natural and social science, DOI 10.1017/CBO9780511660108
Nyhart LK., 2017, Biological individuality: integrating scientific, philosophical, and historical perspectives
[truncated: 378,501 more chars]
